# Supplementary material for: Functional lability of RNA-dependent RNA polymerases in animals
Source: PLoS Genet. 2019 Feb 19;15(2):e1007915. doi: 10.1371/journal.pgen.1007915 (PMC6396948; doi:10.1371/journal.pgen.1007915)
Supplement: S1 Fig — Among the 538 analyzed proteomes, 442 contain at least 1,000 proteins of at least 1,000 amino acids (left panel) and 383 contain at least 5,000 proteins of at least 500 amino acids (right panel). Selective analysis of these species does not fundamentally change the results shown in Fig 1A. Same conventions than in Fig 1A. Some clades analyzed in Fig 1A could not be analyzed here after proteome exclusion: they are shown in grey. (PDF) [file pgen.1007915.s001.pdf]

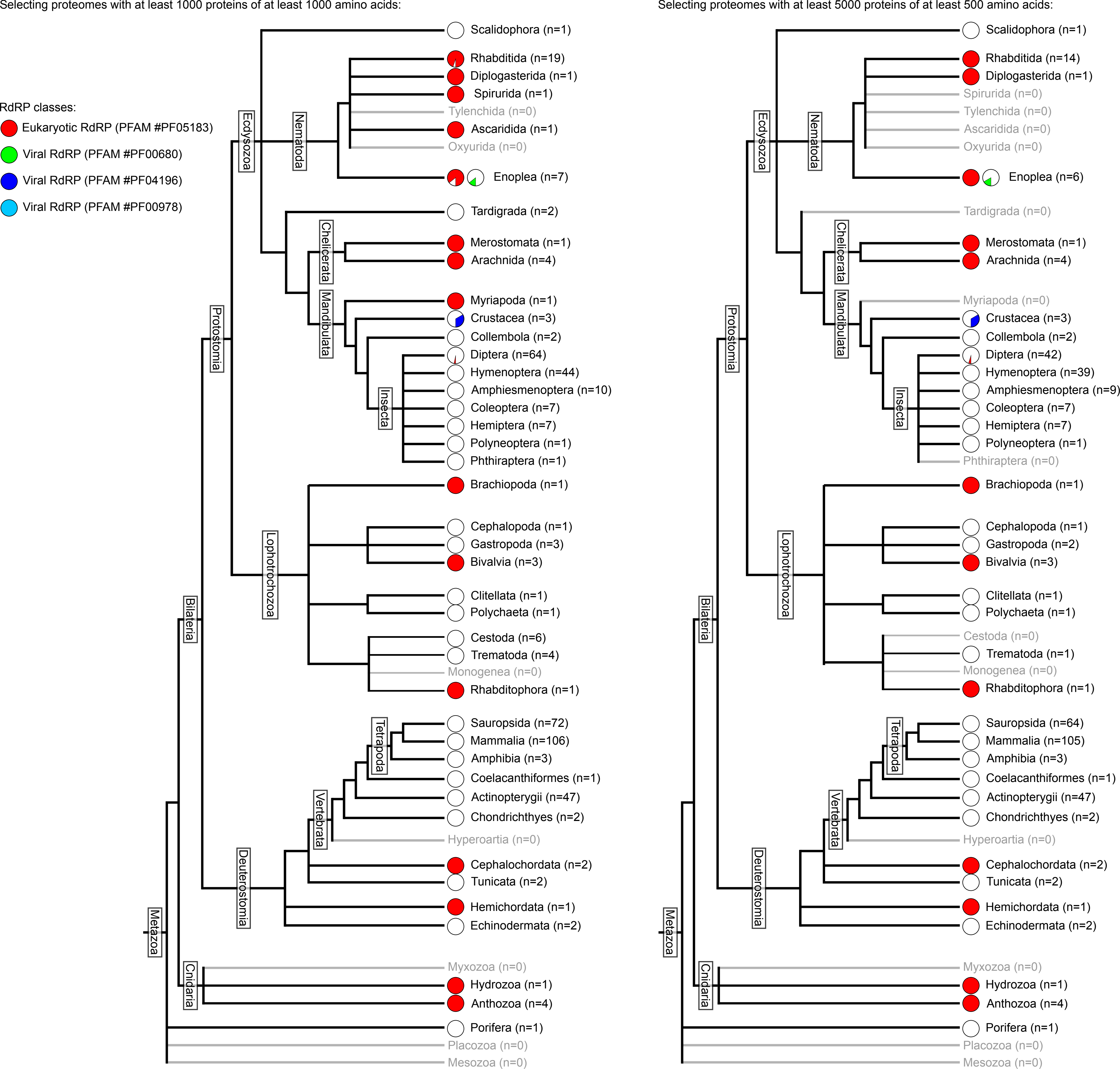
Selecting proteomes with at least 1000 proteins of at least 1000 amino acids:
RdRP classes:
Eukaryotic RdRP (PFAM #PF05183)
Viral RdRP (PFAM #PF00680)
Viral RdRP (PFAM #PF04196)
Viral RdRP (PFAM #PF00978)
Metazoa
Bilateria
Protostomia
Ecdysozoa
Nematoda
Chelicerata
Mandibulata
Insecta
Lophotrochozoa
Deuterostomia
Vertebrata
Tetrapoda
Cnidaria
Scalidophora (n=1)
Rhabditida (n=19)
Diplogasterida (n=1)
Spirurida (n=1)
Tylenchida (n=0)
Ascaridida (n=1)
Oxyurida (n=0)
Enoplea (n=7)
Tardigrada (n=2)
Merostomata (n=1)
Arachnida (n=4)
Myriapoda (n=1)
Crustacea (n=3)
Collembola (n=2)
Diptera (n=64)
Hymenoptera (n=44)
Amphiesmenoptera (n=10)
Coleoptera (n=7)
Hemiptera (n=7)
Polyneoptera (n=1)
Phthiraptera (n=1)
Brachiopoda (n=1)
Cephalopoda (n=1)
Gastropoda (n=3)
Bivalvia (n=3)
Clitellata (n=1)
Polychaeta (n=1)
Cestoda (n=6)
Trematoda (n=4)
Monogenea (n=0)
Rhabditophora (n=1)
Sauropsida (n=72)
Mammalia (n=106)
Amphibia (n=3)
Coelacanthiformes (n=1)
Actinopterygii (n=47)
Chondrichthyes (n=2)
Hyperoartia (n=0)
Cephalochordata (n=2)
Tunicata (n=2)
Hemichordata (n=1)
Echinodermata (n=2)
Myxozoa (n=0)
Hydrozoa (n=1)
Anthozoa (n=4)
Porifera (n=1)
Placozoa (n=0)
Mesozoa (n=0)
Selecting proteomes with at least 5000 proteins of at least 500 amino acids:
Metazoa
Bilateria
Protostomia
Ecdysozoa
Nematoda
Chelicerata
Mandibulata
Insecta
Lophotrochozoa
Deuterostomia
Vertebrata
Tetrapoda
Cnidaria
Scalidophora (n=1)
Rhabditida (n=14)
Diplogasterida (n=1)
Spirurida (n=0)
Tylenchida (n=0)
Ascaridida (n=0)
Oxyurida (n=0)
Enoplea (n=6)
Tardigrada (n=0)
Merostomata (n=1)
Arachnida (n=4)
Myriapoda (n=0)
Crustacea (n=3)
Collembola (n=2)
Diptera (n=42)
Hymenoptera (n=39)
Amphiesmenoptera (n=9)
Coleoptera (n=7)
Hemiptera (n=7)
Polyneoptera (n=1)
Phthiraptera (n=0)
Brachiopoda (n=1)
Cephalopoda (n=1)
Gastropoda (n=2)
Bivalvia (n=3)
Clitellata (n=1)
Polychaeta (n=1)
Cestoda (n=0)
Trematoda (n=1)
Monogenea (n=0)
Rhabditophora (n=1)
Sauropsida (n=64)
Mammalia (n=105)
Amphibia (n=3)
Coelacanthiformes (n=1)
Actinopterygii (n=47)
Chondrichthyes (n=2)
Hyperoartia (n=0)
Cephalochordata (n=2)
Tunicata (n=2)
Hemichordata (n=1)
Echinodermata (n=2)
Myxozoa (n=0)
Hydrozoa (n=1)
Anthozoa (n=4)
Porifera (n=1)
Placozoa (n=0)
Mesozoa (n=0)
