# Supplementary material for: Functional lability of RNA-dependent RNA polymerases in animals
Source: PLoS Genet. 2019 Feb 19;15(2):e1007915. doi: 10.1371/journal.pgen.1007915 (PMC6396948; doi:10.1371/journal.pgen.1007915)
Supplement: S2 Fig — “No adapter” indicates that the 3′ adapter was not detected in the read. “Extragenomic” means that the adapter-trimmed read does not match the B. lanceolatum genome assembly. “Abundant ncRNA” means that it maps on the genome assembly, on one of the genes for known abundant non-coding RNAs (rRNAs, tRNAs, snRNAs, snoRNAs, scaRNAs). “Genome mapper, not matching abundant ncRNAs” means that it maps elsewhere in the genome assembly. (PDF) [file pgen.1007915.s002.pdf]

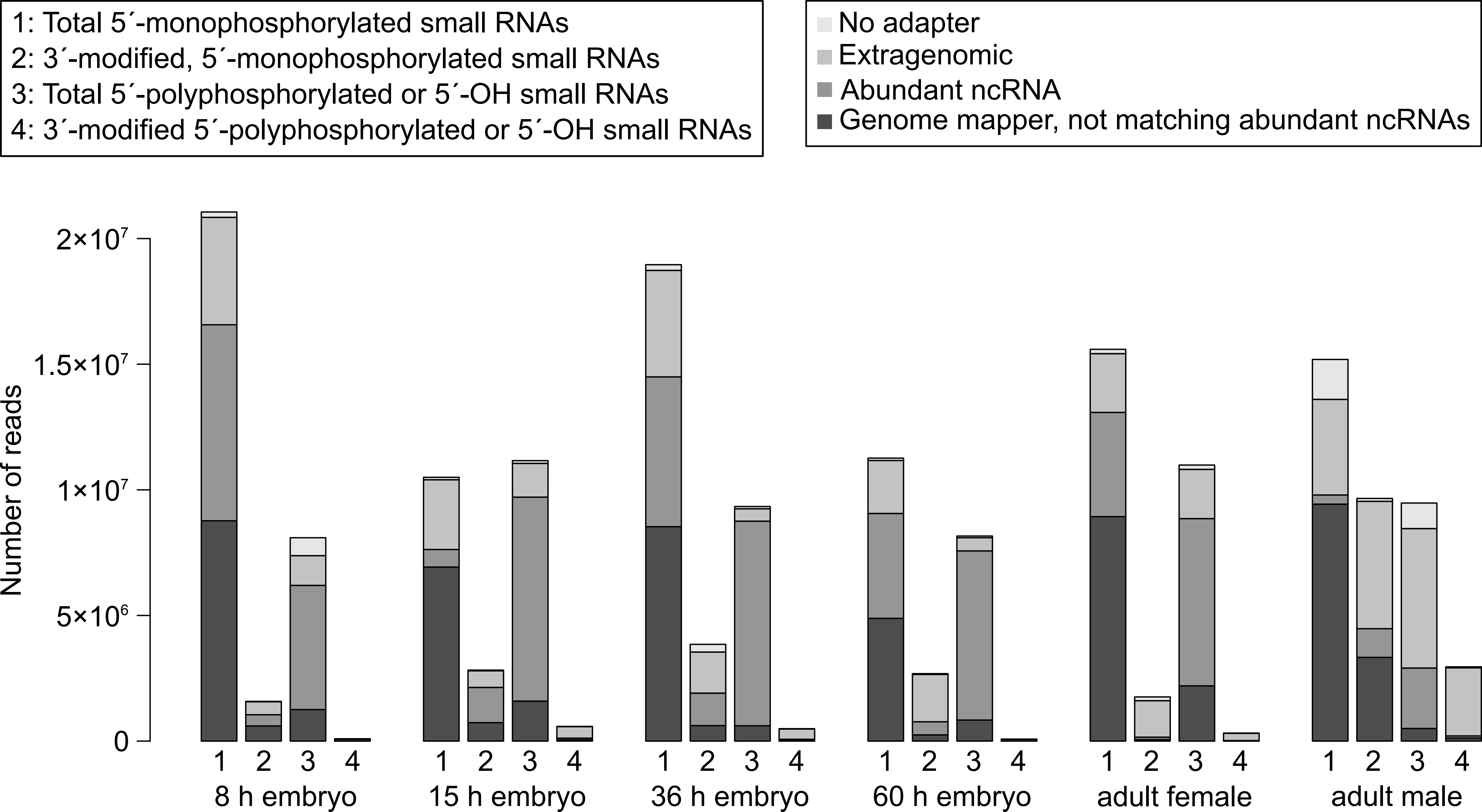

1: Total 5´-monophosphorylated small RNAs
2: 3´-modified, 5´-monophosphorylated small RNAs
3: Total 5´-polyphosphorylated or 5´-OH small RNAs
4: 3´-modified 5´-polyphosphorylated or 5´-OH small RNAs
No adapter
Extragenomic
Abundant ncRNA
Genome mapper, not matching abundant ncRNAs
Number of reads
2×10^7
1.5×10^7
1×10^7
5×10^6
0
1 2 3 4
8 h embryo
15 h embryo
36 h embryo
60 h embryo
adult female
adult male
