# Supplementary figures and images for: Functional lability of RNA-dependent RNA polymerases in animals
Source: PLoS Genet. 2019 Feb 19;15(2):e1007915. doi: 10.1371/journal.pgen.1007915 (PMC6396948; doi:10.1371/journal.pgen.1007915)

In 15 hpf embryos:

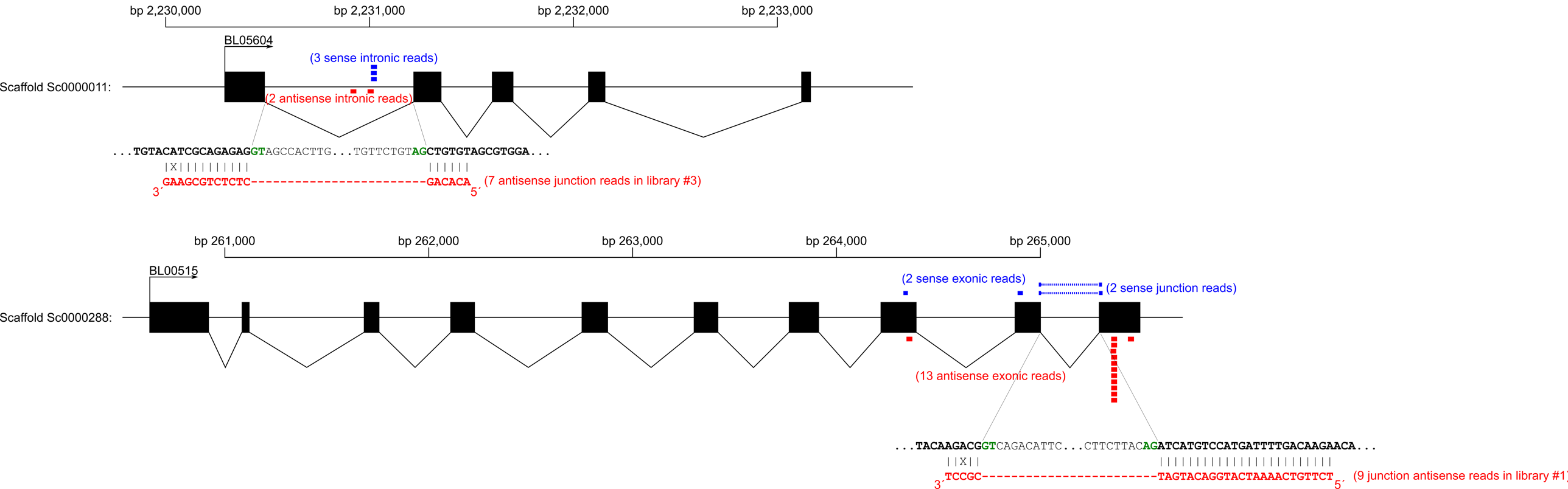

Supplement: S3 Fig — Exons are represented by black rectangles. Detected small RNAs mapping on these genes in the sense orientation are shown in blue, those mapping in antisense orientation are in red. For antisense reads mapping on exon-exon junctions, their precise sequence (in red) is aligned with the gene sequence (in black; splicing donor and acceptor sites are in green). (PDF) [file pgen.1007915.s003.pdf]

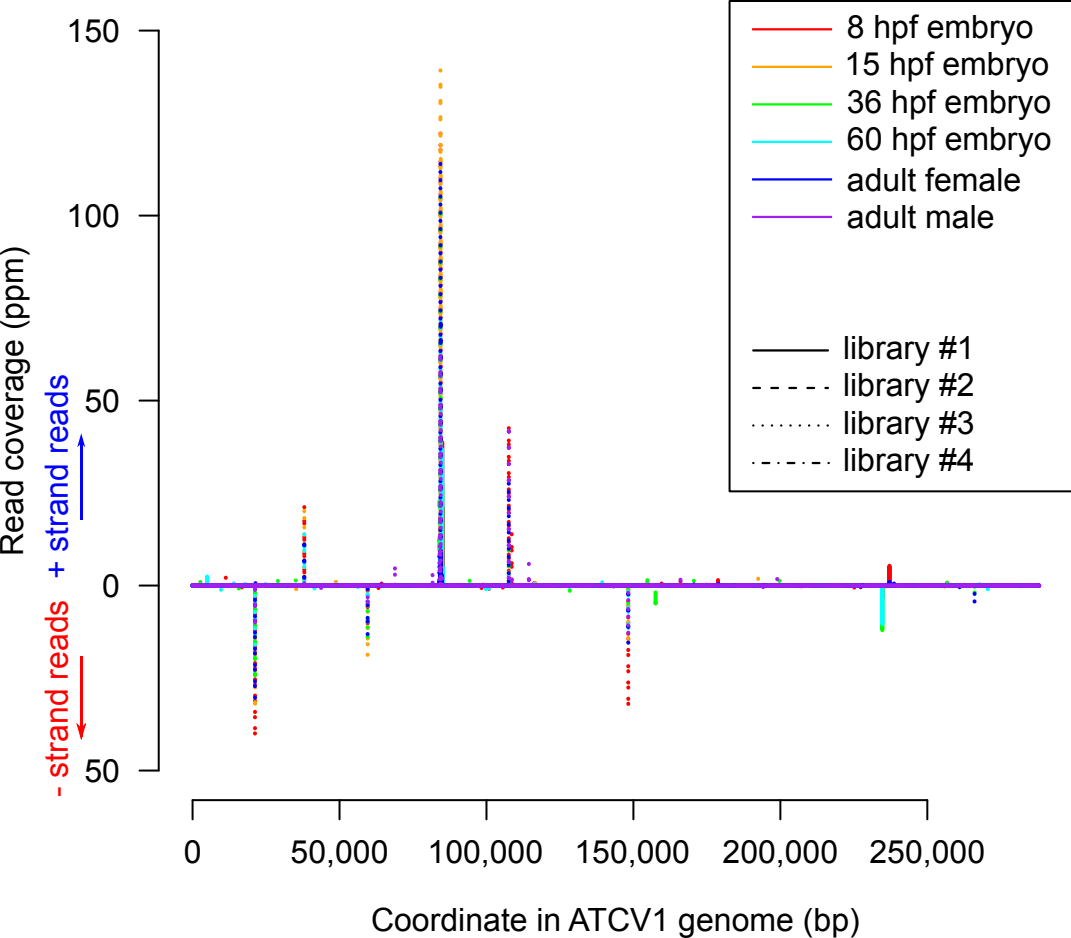

Supplement: S5 Fig — x axis: genomic coordinate along the ATCV1 genome. y axis: number of reads covering each bp in the viral genome. Numbers of reads are expressed as parts per million (ppm) after normalization to the total number of Branchiostoma genome-matching reads that do not match abundant non-coding RNAs. (PDF) [file pgen.1007915.s005.pdf]
