# Supplementary material for: Functional lability of RNA-dependent RNA polymerases in animals
Source: PLoS Genet. 2019 Feb 19;15(2):e1007915. doi: 10.1371/journal.pgen.1007915 (PMC6396948; doi:10.1371/journal.pgen.1007915)
Supplement: S4 Fig — For each of the six RdRP genes, mRNA abundance in various developmental stages was measured by RNA-Seq, and reported as cRPKM (corrected-for-mappability reads per kb and per million mapped reads; [105]). RdRP genes where an intact active site is predicted (see Fig 1B) are annotated “with active site”. Adult RNA-Seq data is from NCBI’s BioSample accession #SAMN09381006 and SAMN09381007, other stages are from [43]. Adult male and female data were averaged. Temporal regulation of RdRP expression in embryos and juveniles was assessed by the Kruskal-Wallis test (p-values are indicated in the legend for each RdRP). (PDF) [file pgen.1007915.s004.pdf]

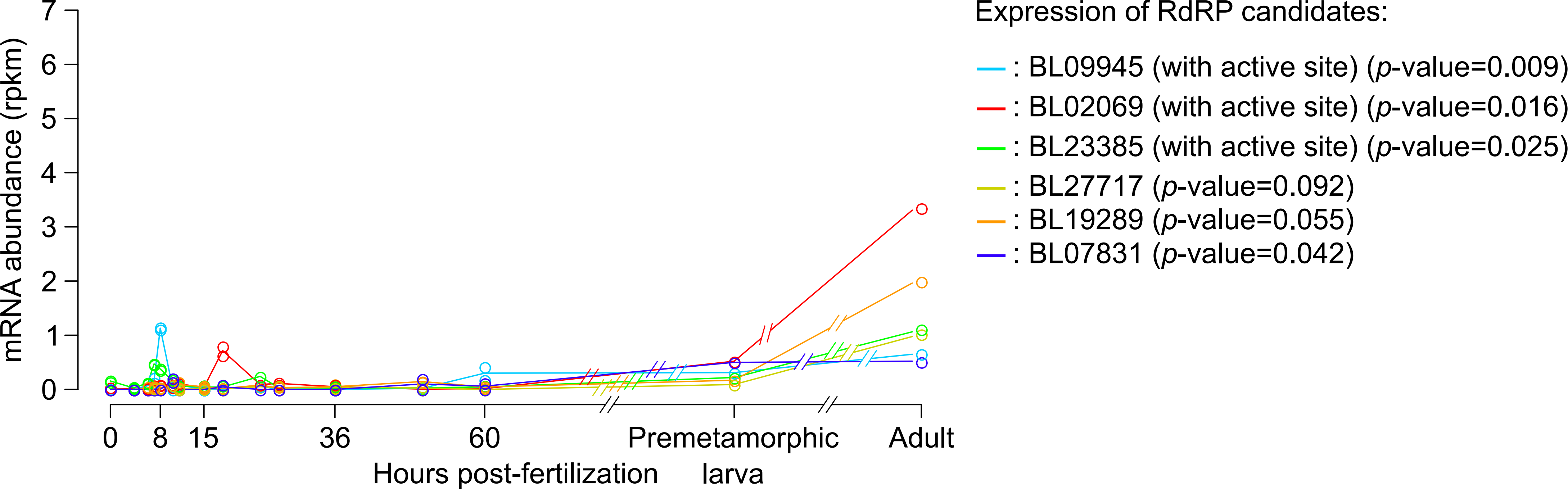

Expression of RdRP candidates:
: BL09945 (with active site) (*p*-value=0.009)
: BL02069 (with active site) (*p*-value=0.016)
: BL23385 (with active site) (*p*-value=0.025)
: BL27717 (*p*-value=0.092)
: BL19289 (*p*-value=0.055)
: BL07831 (*p*-value=0.042)
mRNA abundance (rpkm)
0
1
2
3
4
5
6
7
0
8
15
36
60
Premetamorphic
Adult
Hours post-fertilization
larva
