# Supplementary material for: Functional lability of RNA-dependent RNA polymerases in animals
Source: PLoS Genet. 2019 Feb 19;15(2):e1007915. doi: 10.1371/journal.pgen.1007915 (PMC6396948; doi:10.1371/journal.pgen.1007915)
Supplement: S6 Fig — Sequences of 5 known Hen1 proteins (from Nematostella vectensis, Danio rerio, Mus musculus, Arabidopsis thaliana and Drosophila melanogaster) were aligned with the identified Branchiostoma lanceolatum Hen1 candidate (only the part of the alignment spanning amino acids 661–939 of the Arabidopsis protein is shown). Alignment was performed with t-coffee (version 11.00.8cbe486); other alignment programs (Clustal Omega v.1.2.4, t-coffee v.8.93, Kalign v.2.03, MAFFT v.7.215, but not muscle v.3.8.31) give the same main result: amino acids and amino acid combinations required for Hen1 catalytic activity [103] are conserved in the Branchiostoma candidate. Amino acids boxed in red were shown to be essential for Arabodipsis Hen1 activity; in orange: amino acids whose absence affects Hen1 activity without abolishing it entirely. Amino acid numbering is based on the Arabidopsis sequence. (PDF) [file pgen.1007915.s006.pdf]

```
                                                                   R701
B. lanceolatum BL03504     D-----------------------EEEFGGPVFSPPLYRQRYQTVADLVK----KYRPKR
N. vectensis AGW15602      -------------------------REQLGPKFDPPVYRQRYHRVIEVVK----EHKAKR
D. rerio NP_001017842      -----------------------------ATPFSPPLYMQRYQFVIDYVK----TYRPRK
M. musculus NP_001072114   E-----------------------VSPEKVIRFKPPLYKQRYQFVRDLVD----RHEPKK
A. thaliana NP_567616      IRSLLSERPCLNYNILLLGVKGPSEERMEAAFFKPPLSKQRVEYALKHIR----ESSAST
D. melanogaster NP_610732  ------------------------KMTETGITFDPPVYEQRYCATIQILEDARWKDQIKK

B. lanceolatum BL03504     LVDFGCAEGKLIRFLK-PEESLEQLTGIDLEGEVLESIRGIIKPLLSDYVQPRPRPFTVS
N. vectensis AGW15602      VLDFGCAEAKMLRSLINSTTNIEELVGVDIDRDLLEDSIFRIRPLTTDYLTPRPHPLAVS
D. rerio NP_001017842      VIDFGCAECCLLKKLKFHRNGIQLLVGVDINSVVLLKRMHSLAPLVSDYLQPSDGPLTIE
M. musculus NP_001072114   VADLGCGDAKLLKLLKI-YPCIQLLVGVDINEEKLHSNGHRLSPYLGEFVKPRDLDLTVT
A. thaliana NP_567616      LVDFGCGSGSLLDSLLDYPTSLQTIIGVDISPKGLARAAKMLHVKLN---KEACNVKSAT
D. melanogaster NP_610732  VVEFGCAEMRFFQLMR-RIETIEHIGLVDIDKSLLMRNLTSVNPLVSDYIRSRASPLKVQ

                                                 E796   E799, H800
B. lanceolatum BL03504     LYQGSIAECDDRFKSYDMVTCVEVIEHLDPPVLDAMPSNVFGHMRPSVVVVTTPNSEFNV
N. vectensis AGW15602      LYQGSISKADDRFCDFDVVACIEIVEHLVPEHLEAMPAVLLGQLSPLVAIVTTPNADFNV
D. rerio NP_001017842      LYQGSVMEREPCTKGFDLVTCVELIEHLELEEVERFSEVVFGYMAPGAVIVTTPNAEFNP
M. musculus NP_001072114   LYHGSVVERDSRLLGFDLITCIELIEHLDSDDLARFPDVVFGYLSPAMVVISTPNAEFNP
A. thaliana NP_567616      LYDGSILEFDSRLHDVDIGTCLEVIEHMEEDQACEFGEKVLSLFHPKLLIVSTPNYEFNT
D. melanogaster NP_610732  ILQGNVADSSEELRDTDAVIAIELIEHVYDDVLAKIPVNIFGFMQPKLVVFSTPNSDFNV

                                                          H860
B. lanceolatum BL03504     LFPN--------------F-----SGFRNADHRFEWTRQEFQTWAEGVAQRF-SYDVTFH
N. vectensis AGW15602      LFPD--------------L-----VGFRHWDHKFEWTRAEFKDWATSQADKF-GYSVTFE
D. rerio NP_001017842      LLPG--------------L-----RGFRNYGHKFEWTRAEFQTWAHRVCREH-GYSVQFT
M. musculus NP_001072114   LFPT--------------V------TLRDADHKFEWSRMEFQTWALHVANCY-NYRVEFT
A. thaliana NP_567616      ILQRSTPETQEENNSEPQL-----PKFRNHDHKFEWTREQFNQWASKLGKRH-NYSVEFS
D. melanogaster NP_610732  IFTR--------------FNPLLPNGFRHEDHKFEWSRDEFKNWCLGIVEKYPNYMFSLT

B. lanceolatum BL03504     GIGTGPEGTEHLGCCTQMAIFERKQTPYDE--------N-STVLWGTPYELIAEAVFPYR
N. vectensis AGW15602      GIGSGPSGTEHLGCCSQMALFIKQNTAPA---------G-RQTGFGEPYNLIARVEHPYR
D. rerio NP_001017842      GVGEAAGHWRDVGFCTQIAVFQRNFDGVNRSMS-----N-AEHLEPSVYRLLYRVVYPSL
M. musculus NP_001072114   GVGTPPAGSEHVGYCTQIGVFTKNGGKLSK-PS-----V-SQQCDQHVYKPVYTTSYPSL
A. thaliana NP_567616      GVGGS--GEVEPGFASQIAIFRREASSVE---------N-VAESSMQPYKVIWEWKKEDV
D. melanogaster NP_610732  GVGNPPKEYESVGPVSQIAIFVRKDMLEMQ-LVNPLVSKPNIDKESIPYKLIHTVEYPFY

B. lanceolatum BL03504     ENTLSKEQQILQEVQYYIRQIM--QRRVHGEDEEKD---NADDDDSAPE-----------
N. vectensis AGW15602      KCTLTEEEKILIELDRTLWFLS--QPSA-YEDDEISD--SEDLGD--DK-----------
D. rerio NP_001017842      CDNNIYQKTLINEVLYEAQHLR--QQWL-IRENMNNN----AHFYS---P-PLMEALHHG
M. musculus NP_001072114   QQEKVLKFVLVGELLIQVDRLRLRYQRM-LRDREKDRGPKPGDMDSCPAPHLLLGAVFTE
A. thaliana NP_567616      ---------------------------------EKKK-----------------------
D. melanogaster NP_610732  VDTRTEKEKLWTEVQIELQRFK--RQF---ESSEIEE---GTYQDT--------------
```
