# Supplementary material for: Functional lability of RNA-dependent RNA polymerases in animals
Source: PLoS Genet. 2019 Feb 19;15(2):e1007915. doi: 10.1371/journal.pgen.1007915 (PMC6396948; doi:10.1371/journal.pgen.1007915)
Supplement: S1 File — For each of the following classes, small RNA populations were analyzed as in Figs 3B, 3C, 4 and 5: reads matching the B. lanceolatum genome without matching abundant non-coding RNAs (section 1); reads matching B. lanceolatum pre-miRNA hairpins (section 2); reads matching the B. lanceolatum transcriptome without matching pre-miRNAs or abundant non-coding RNAs (section 3); reads matching B. lanceolatum mRNAs with long ORFs (section 4); reads not matching the B. lanceolatum genome or transcriptome (section 5); C. elegans small RNAs cloned with a procedure detecting 5′ mono- and polyphosphorylated RNAs [22] (section 6); reads not matching the B. lanceolatum genome or transcriptome, and matching the Staphylococcus aureus genome (section 7); reads not matching the B. lanceolatum genome or transcriptome, and matching the Vibrio alginolyticus genome (section 8); reads not matching the B. lanceolatum genome or transcriptome, and matching the Vibrio anguillarum genome (section 9); reads not matching the B. lanceolatum genome or transcriptome, and matching the Acanthocystis turfacea Chlorella virus 1 (ATCV1) genome (section 10); reads not matching the B. lanceolatum genome or transcriptome, and matching non-Branchiostoma contig #18690 (covered with 1,982.33 ppm small RNA reads across all 24 libraries) (section 11); reads not matching the B. lanceolatum genome or transcriptome, and matching non-Branchiostoma contig #7601 (covered with 1,534.35 ppm small RNA reads across all 24 libraries) (section 12); reads not matching the B. lanceolatum genome or transcriptome, and matching non-Branchiostoma contig #38312 (covered with 236.037 ppm small RNA reads across all 24 libraries) (section 13); reads not matching the B. lanceolatum genome or transcriptome, and matching non-Branchiostoma contig #3365 (covered with 223.535 ppm small RNA reads across all 24 libraries) (section 14); reads not matching the B. lanceolatum genome or transcriptome, and matching non-Branchiostoma contig #10883 [file pgen.1007915.s007.pdf]

Functional lability of RNA-dependent RNA polymerases in animals: Supplementary Data

Natalia Pinzón, Stéphanie Bertrand, Lucie Subirana, Isabelle Busseau, Hector Escrivá and Hervé Seitz

Contents

1 Genomic reads not matching abundant ncRNAs 3

1.1 Libraries #1 (total 5´ monophosphorylated small RNAs) . . . 3
1.2 Libraries #2 (3´ modified, 5´ monophosphorylated small RNAs) . . . 9
1.3 Libraries #3 (total 5´ hydroxyl or polyphosphorylated small RNAs) . . . 15
1.4 Libraries #4 (3´ modified, 5´ hydroxyl or polyphosphorylated small RNAs) . . . 21

2 pre-miRNA hairpin-matching reads 27

2.1 Libraries #1 (total 5´ monophosphorylated small RNAs) . . . 27
2.2 Libraries #2 (3´ modified, 5´ monophosphorylated small RNAs) . . . 39
2.3 Libraries #3 (total 5´ hydroxyl or polyphosphorylated small RNAs) . . . 51
2.4 Libraries #4 (3´ modified, 5´ hydroxyl or polyphosphorylated small RNAs) . . . 63

3 Transcriptome-matching reads (excuding pre-miRNA and abundant ncRNA-matching reads) 75

3.1 Libraries #1 (total 5´ monophosphorylated small RNAs) . . . 75
3.2 Libraries #2 (3´ modified, 5´ monophosphorylated small RNAs) . . . 87
3.3 Libraries #3 (total 5´ hydroxyl or polyphosphorylated small RNAs) . . . 99
3.4 Libraries #4 (3´ modified, 5´ hydroxyl or polyphosphorylated small RNAs) . . . 111

4 Reads matching RNAs with long ORF's 123

4.1 Libraries #1 (total 5´ monophosphorylated small RNAs) . . . 123
4.2 Libraries #2 (3´ modified, 5´ monophosphorylated small RNAs) . . . 135
4.3 Libraries #3 (total 5´ hydroxyl or polyphosphorylated small RNAs) . . . 147
4.4 Libraries #4 (3´ modified, 5´ hydroxyl or polyphosphorylated small RNAs) . . . 159

5 Extragenomic and extratranscriptomic reads 171

5.1 Libraries #1 (total 5´ monophosphorylated small RNAs) . . . 171
5.2 Libraries #2 (3´ modified, 5´ monophosphorylated small RNAs) . . . 177
5.3 Libraries #3 (total 5´ hydroxyl or polyphosphorylated small RNAs) . . . 183
5.4 Libraries #4 (3´ modified, 5´ hydroxyl or polyphosphorylated small RNAs) . . . 189

6 Analysis of *Cænorhabditis elegans* data [Gu et al., 2009] with the same method 195

6.1 Genomic reads not matching abundant ncRNAs . . . 195
6.2 pre-miRNA hairpin-matching reads . . . 198
6.3 Transcriptome-matching reads (excuding pre-miRNA and abundant ncRNA-matching reads) . . . 204
6.4 Reads matching RNAs with long ORF's . . . 210
6.5 Extragenomic and extratranscriptomic reads . . . 216
6.6 Extragenomic reads matching the transcriptome . . . 219

7 Extragenomic and extratranscriptomic reads matching the *Staphylococcus aureus* genome 225

7.1 Libraries #1 (total 5´ monophosphorylated small RNAs) . . . 225
7.2 Libraries #2 (3´ modified, 5´ monophosphorylated small RNAs) . . . 231
7.3 Libraries #3 (total 5´ hydroxyl or polyphosphorylated small RNAs) . . . 237
7.4 Libraries #4 (3´ modified, 5´ hydroxyl or polyphosphorylated small RNAs) . . . 243

8 Extragenomic and extratranscriptomic reads matching the *Vibrio alginolyticus* genome 249
8.1 Libraries #1 (total 5´ monophosphorylated small RNAs) . . . 249
8.2 Libraries #2 (3´ modified, 5´ monophosphorylated small RNAs) . . . 255
8.3 Libraries #3 (total 5´ hydroxyl or polyphosphorylated small RNAs) . . . 261
8.4 Libraries #4 (3´ modified, 5´ hydroxyl or polyphosphorylated small RNAs) . . . 267

9 Extragenomic and extratranscriptomic reads matching the *Vibrio anguillarum* genome 273
9.1 Libraries #1 (total 5´ monophosphorylated small RNAs) . . . 273
9.2 Libraries #2 (3´ modified, 5´ monophosphorylated small RNAs) . . . 279
9.3 Libraries #3 (total 5´ hydroxyl or polyphosphorylated small RNAs) . . . 285
9.4 Libraries #4 (3´ modified, 5´ hydroxyl or polyphosphorylated small RNAs) . . . 291

10 Extragenomic and extratranscriptomic reads matching the ATCV1 genome 297
10.1 Libraries #1 (total 5´ monophosphorylated small RNAs) . . . 297
10.2 Libraries #2 (3´ modified, 5´ monophosphorylated small RNAs) . . . 303
10.3 Libraries #3 (total 5´ hydroxyl or polyphosphorylated small RNAs) . . . 309
10.4 Libraries #4 (3´ modified, 5´ hydroxyl or polyphosphorylated small RNAs) . . . 315

11 Extragenomic and extratranscriptomic reads matching extragenomic contig #18690 321
11.1 Libraries #1 (total 5´ monophosphorylated small RNAs) . . . 321
11.2 Libraries #2 (3´ modified, 5´ monophosphorylated small RNAs) . . . 327
11.3 Libraries #3 (total 5´ hydroxyl or polyphosphorylated small RNAs) . . . 333
11.4 Libraries #4 (3´ modified, 5´ hydroxyl or polyphosphorylated small RNAs) . . . 339

12 Extragenomic and extratranscriptomic reads matching extragenomic contig #7601 345
12.1 Libraries #1 (total 5´ monophosphorylated small RNAs) . . . 345
12.2 Libraries #2 (3´ modified, 5´ monophosphorylated small RNAs) . . . 351
12.3 Libraries #3 (total 5´ hydroxyl or polyphosphorylated small RNAs) . . . 357
12.4 Libraries #4 (3´ modified, 5´ hydroxyl or polyphosphorylated small RNAs) . . . 363

13 Extragenomic and extratranscriptomic reads matching extragenomic contig #38312 369
13.1 Libraries #1 (total 5´ monophosphorylated small RNAs) . . . 369
13.2 Libraries #2 (3´ modified, 5´ monophosphorylated small RNAs) . . . 375
13.3 Libraries #3 (total 5´ hydroxyl or polyphosphorylated small RNAs) . . . 381
13.4 Libraries #4 (3´ modified, 5´ hydroxyl or polyphosphorylated small RNAs) . . . 387

14 Extragenomic and extratranscriptomic reads matching extragenomic contig #3365 393
14.1 Libraries #1 (total 5´ monophosphorylated small RNAs) . . . 393
14.2 Libraries #2 (3´ modified, 5´ monophosphorylated small RNAs) . . . 399
14.3 Libraries #3 (total 5´ hydroxyl or polyphosphorylated small RNAs) . . . 405
14.4 Libraries #4 (3´ modified, 5´ hydroxyl or polyphosphorylated small RNAs) . . . 411

15 Extragenomic and extratranscriptomic reads matching extragenomic contig #10883 417
15.1 Libraries #1 (total 5´ monophosphorylated small RNAs) . . . 417
15.2 Libraries #2 (3´ modified, 5´ monophosphorylated small RNAs) . . . 423
15.3 Libraries #3 (total 5´ hydroxyl or polyphosphorylated small RNAs) . . . 429
15.4 Libraries #4 (3´ modified, 5´ hydroxyl or polyphosphorylated small RNAs) . . . 435

1 Genomic reads not matching abundant ncRNAs

1.1 Libraries #1 (total 5´ monophosphorylated small RNAs)

Embryo 8h, library 1:

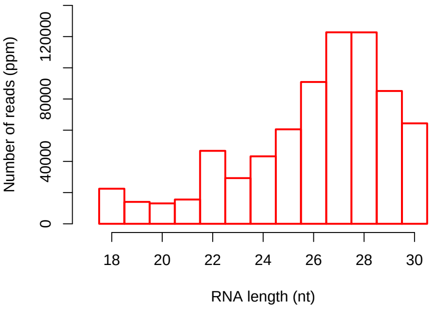

18-mers:

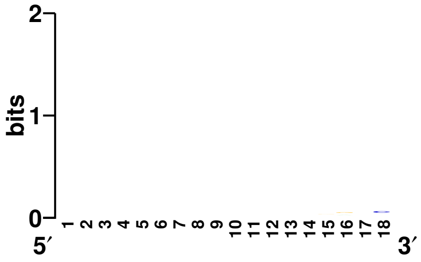

19-mers:

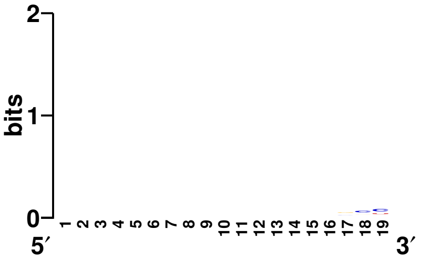

20-mers:

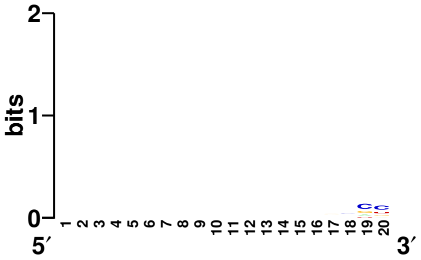

21-mers:

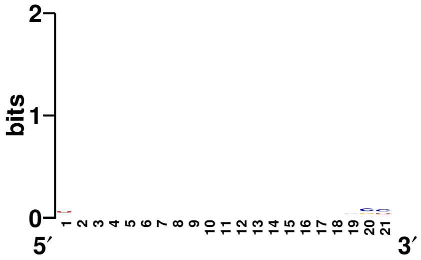

22-mers:

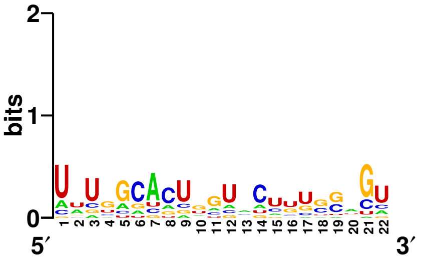

23-mers:

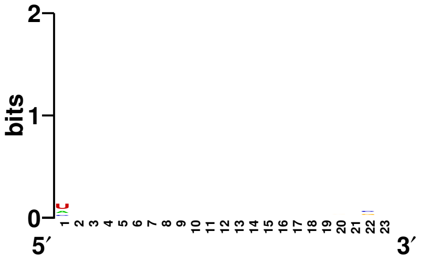

24-mers:

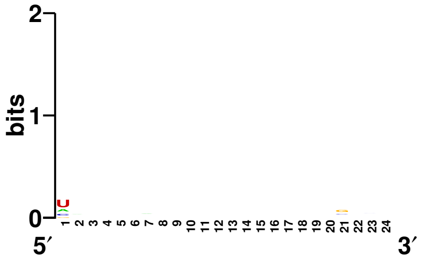

25-mers:

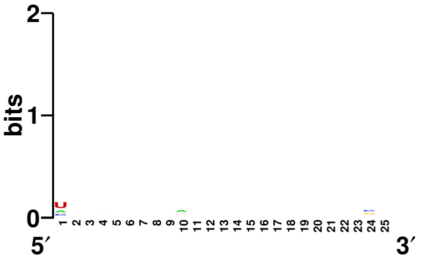

26-mers:

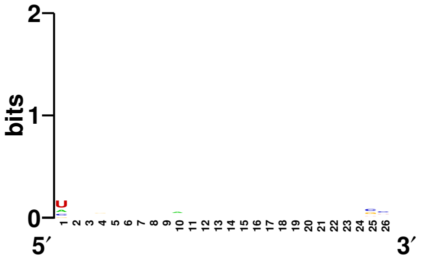

27-mers:

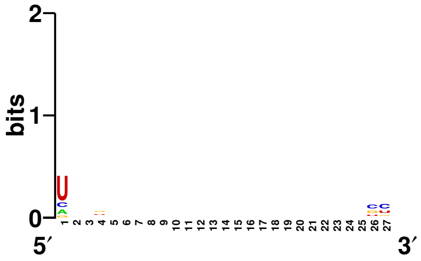

28-mers:

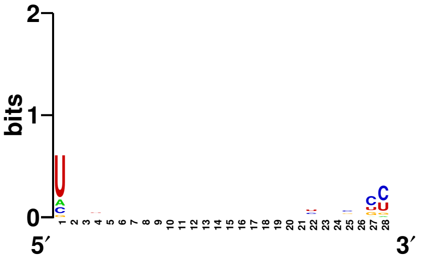

29-mers:

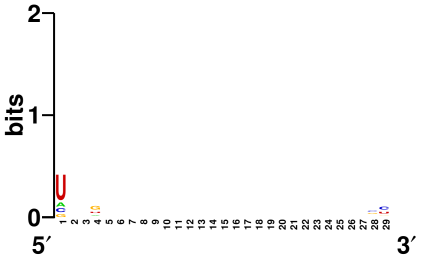

30-mers:

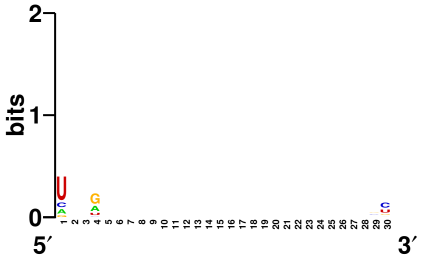

Embryo 15h, library 1:

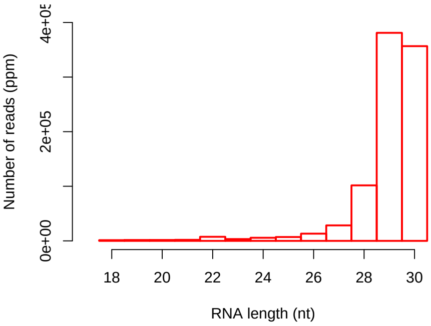

18-mers:

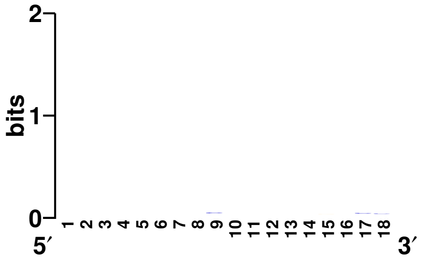

19-mers:

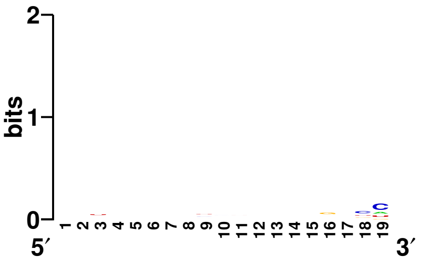

20-mers:

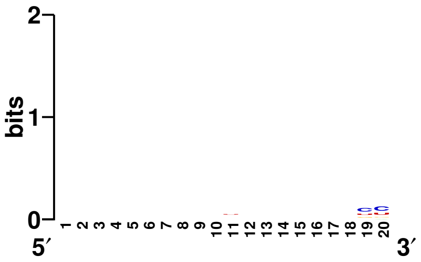

21-mers:

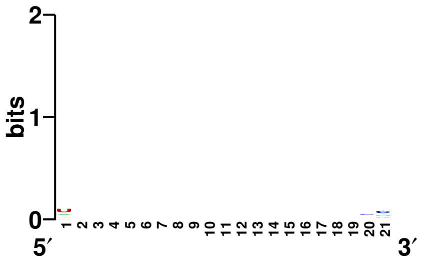

22-mers:

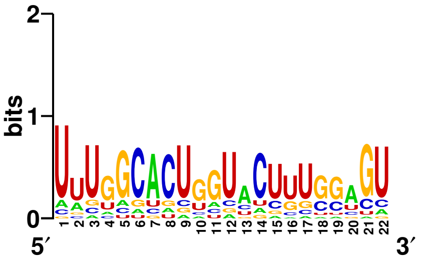

23-mers:

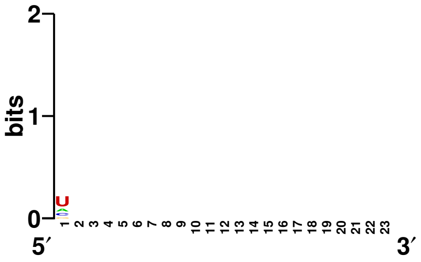

24-mers:

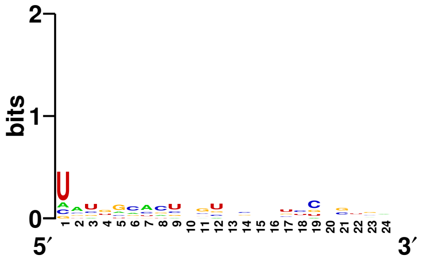

25-mers:

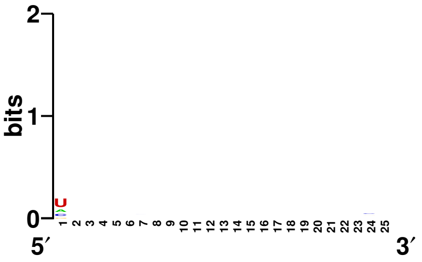

26-mers:

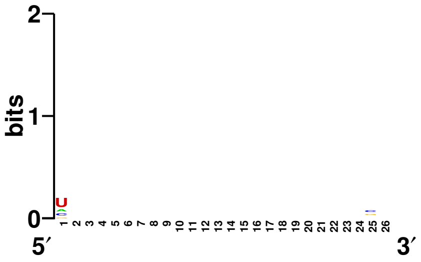

27-mers:

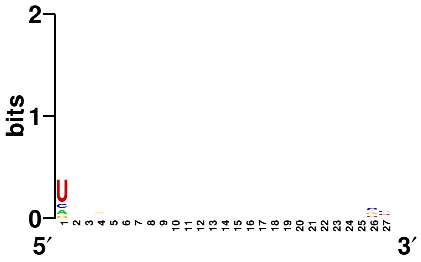

28-mers:

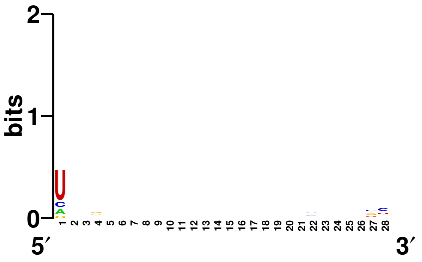

29-mers:

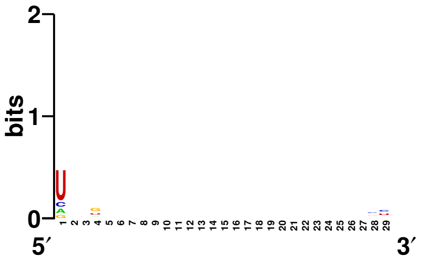

30-mers:

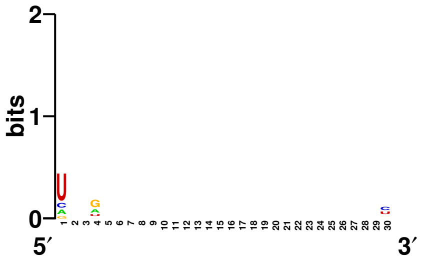

Embryo 36h, library 1:

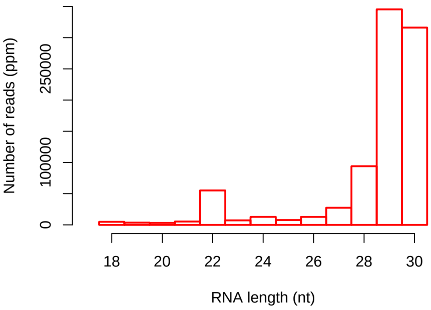

18-mers:

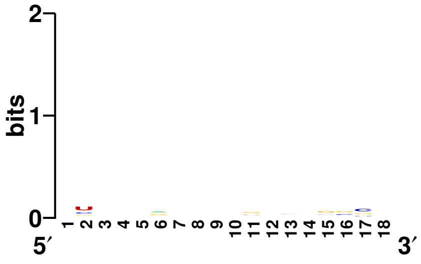

19-mers:

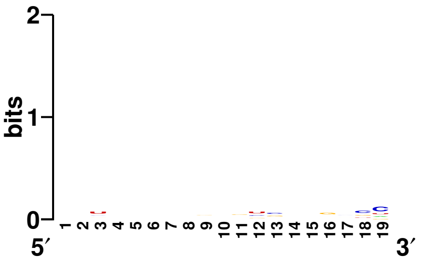

20-mers:

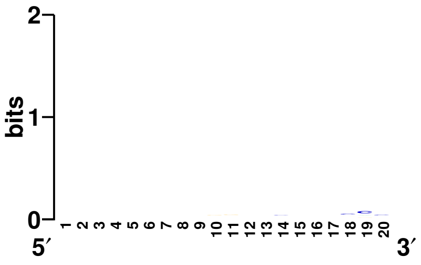

21-mers:

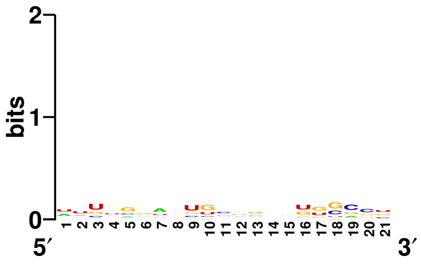

22-mers:

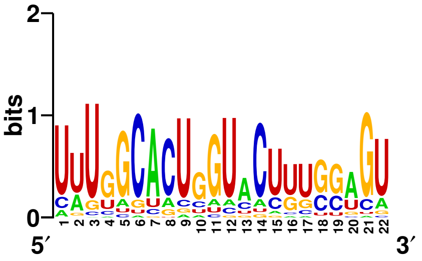

23-mers:

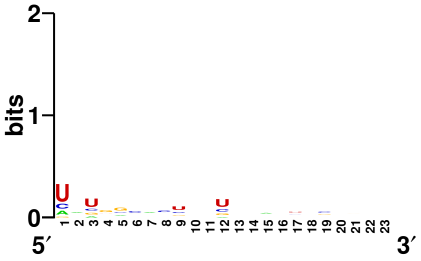

24-mers:

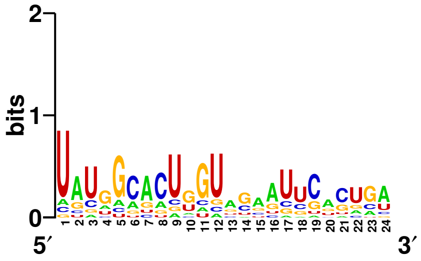

25-mers:

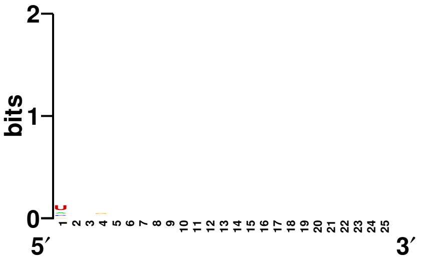

26-mers:

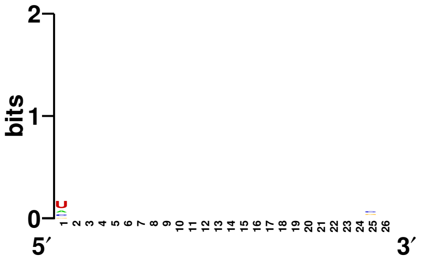

27-mers:

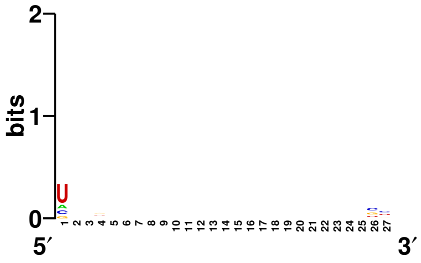

28-mers:

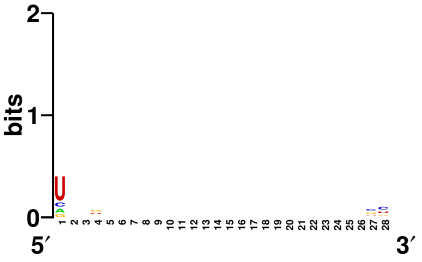

29-mers:

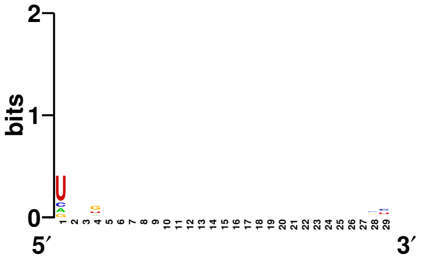

30-mers:

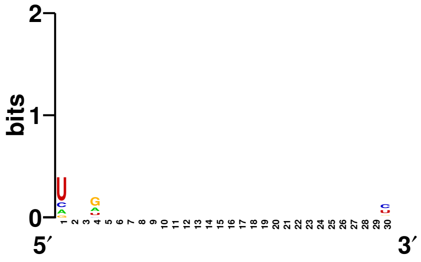

Embryo 60h, library 1:

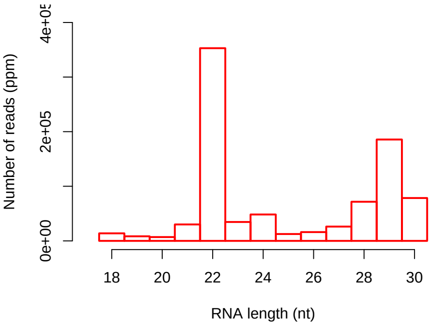

18-mers:

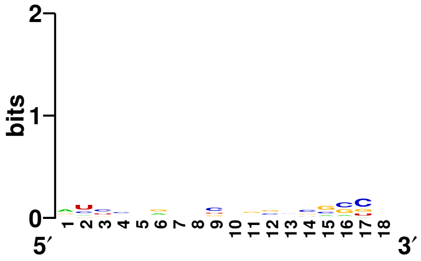

19-mers:

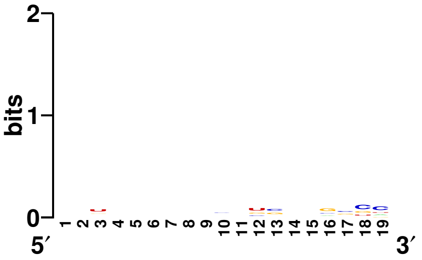

20-mers:

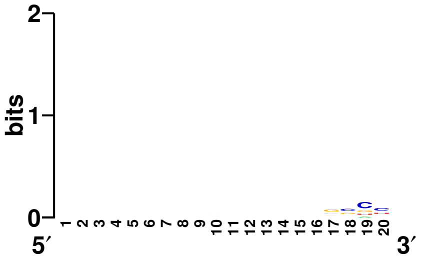

21-mers:

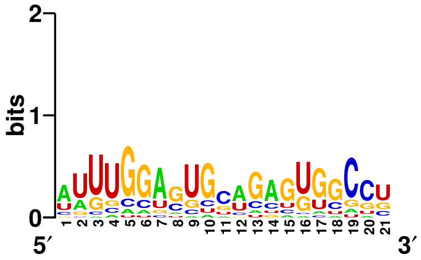

22-mers:

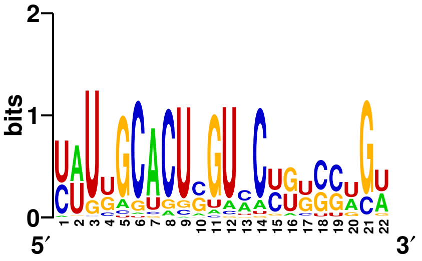

23-mers:

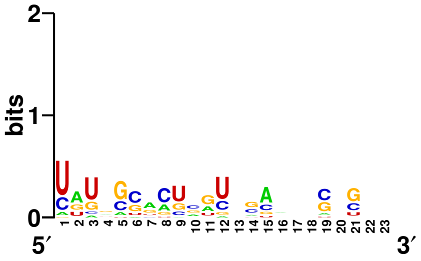

24-mers:

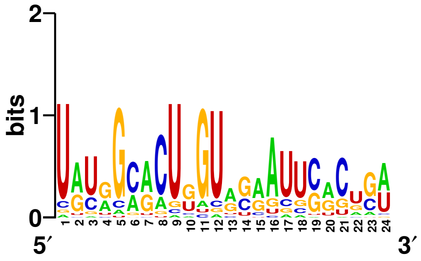

25-mers:

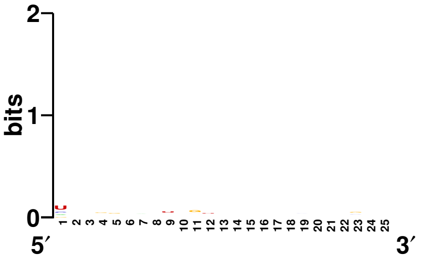

26-mers:

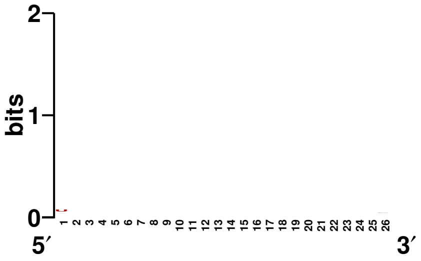

27-mers:

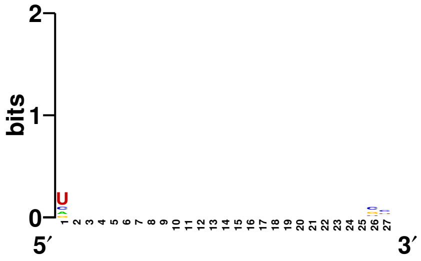

28-mers:

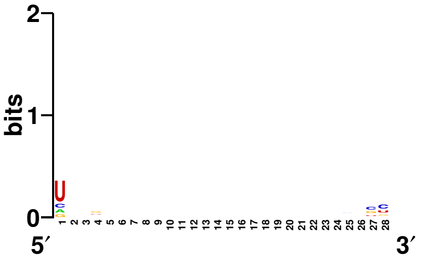

29-mers:

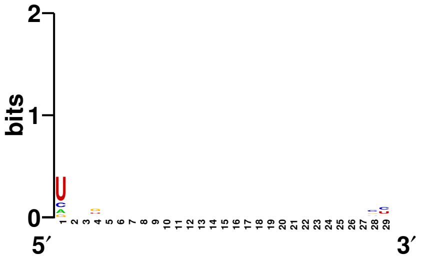

30-mers:

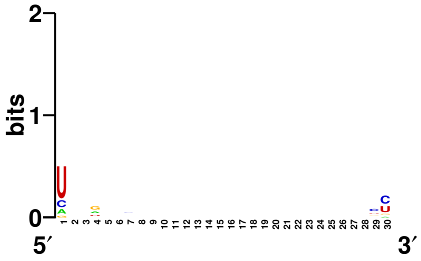

Adult female, library 1:

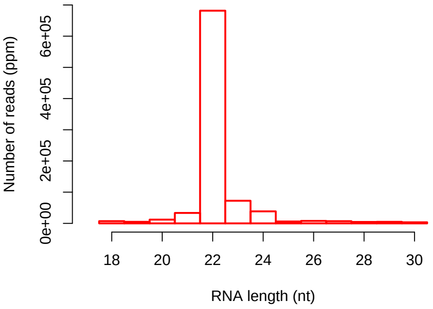

18-mers:

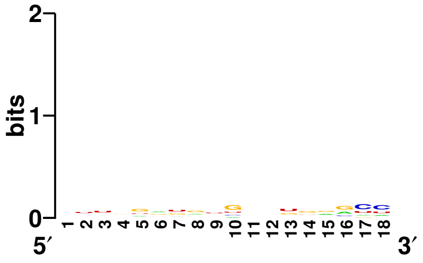

19-mers:

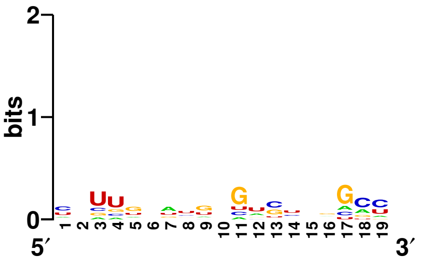

20-mers:

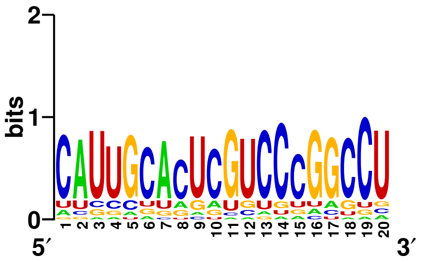

21-mers:

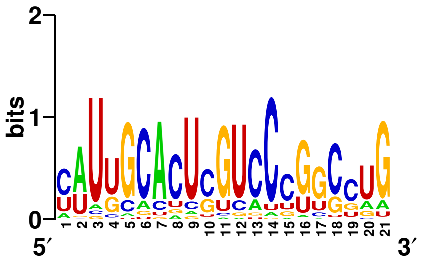

22-mers:

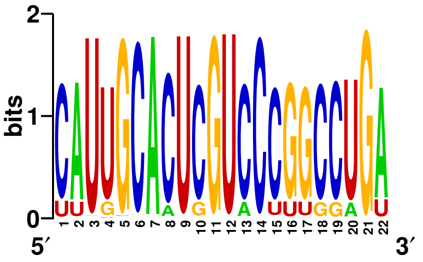

23-mers:

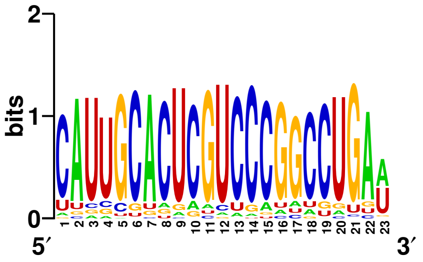

24-mers:

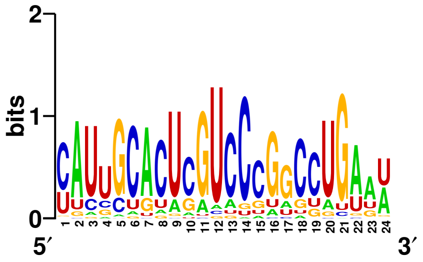

25-mers:

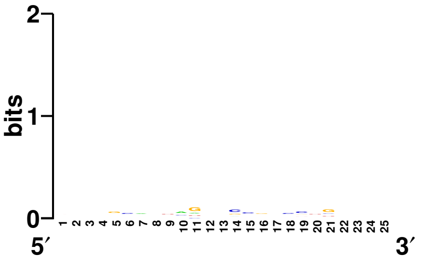

26-mers:

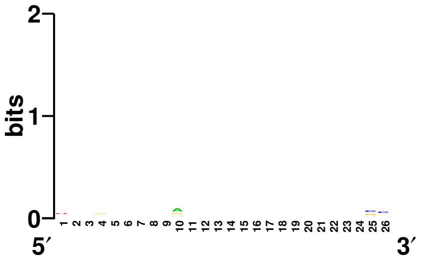

27-mers:

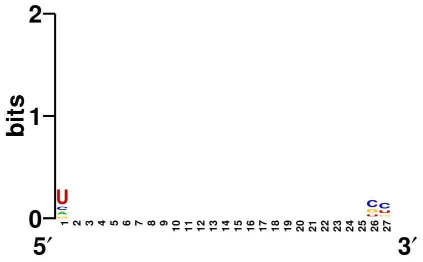

28-mers:

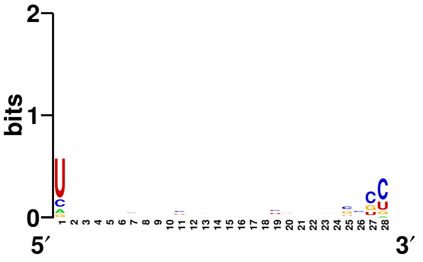

29-mers:

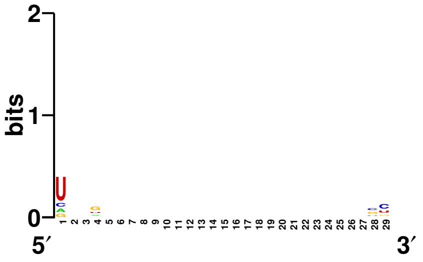

30-mers:

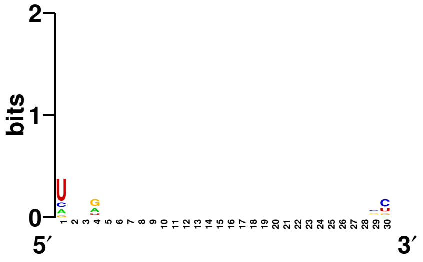

Adult male, library 1:

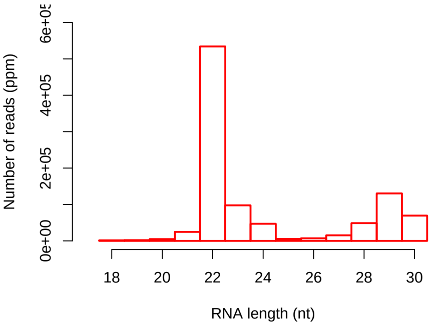

18-mers:

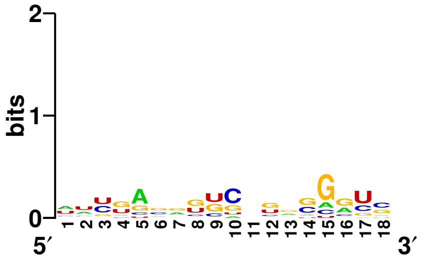

19-mers:

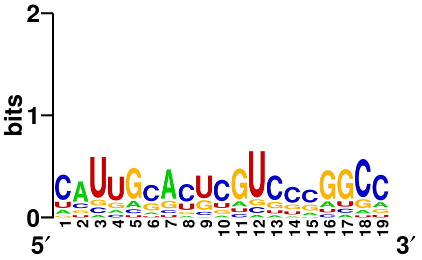

20-mers:

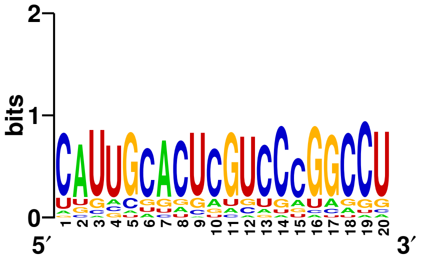

21-mers:

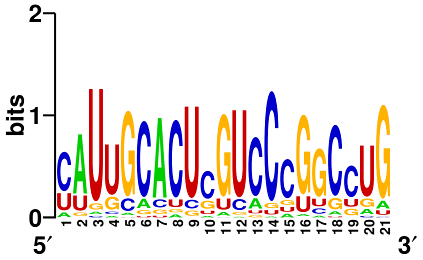

22-mers:

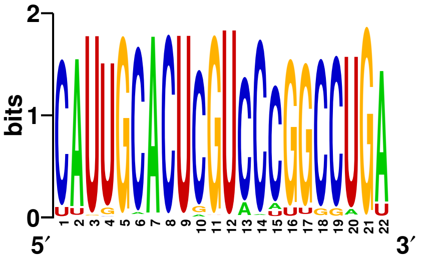

23-mers:

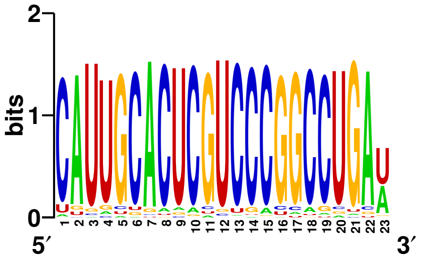

24-mers:

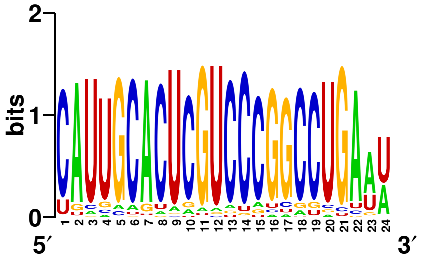

25-mers:

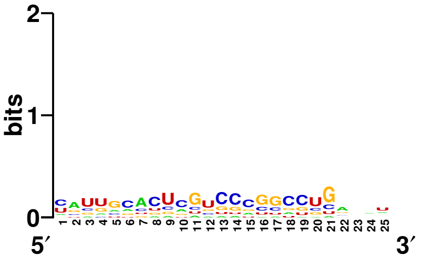

26-mers:

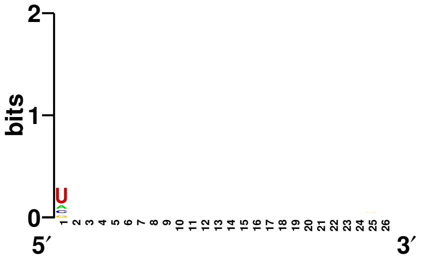

27-mers:

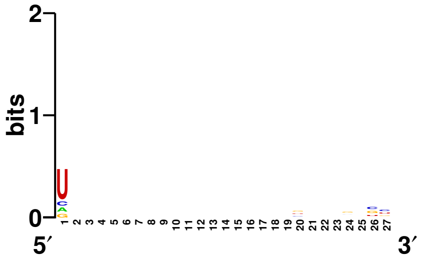

28-mers:

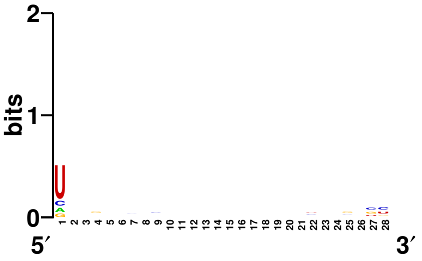

29-mers:

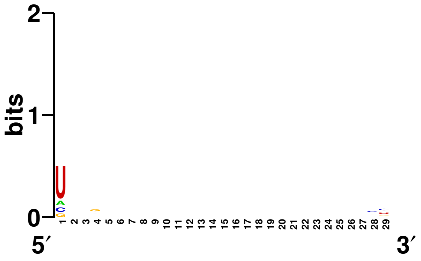

30-mers:

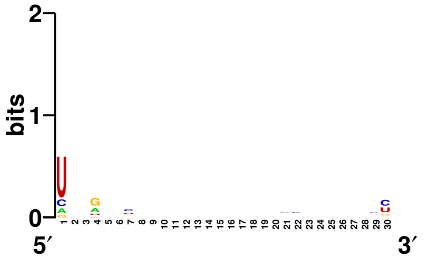

1.2 Libraries #2 (3´ modified, 5´ monophosphorylated small RNAs)

Embryo 8h, library 2:

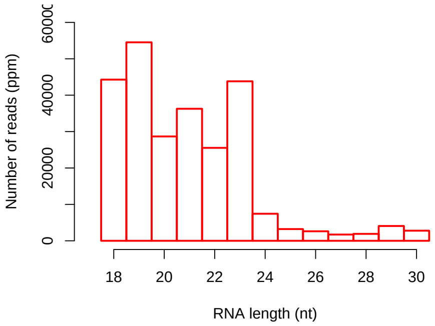

18-mers:

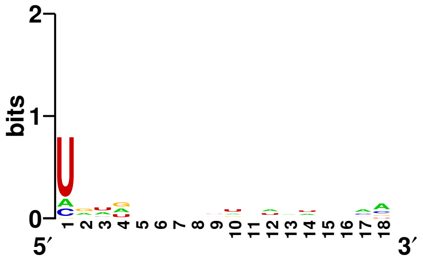

19-mers:

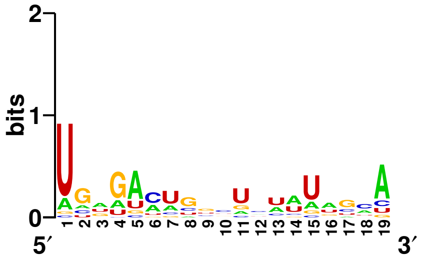

20-mers:

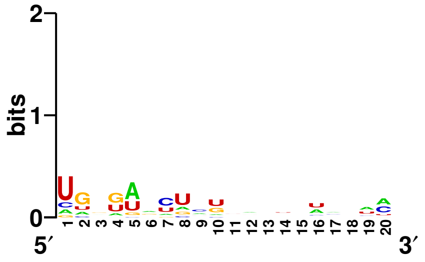

21-mers:

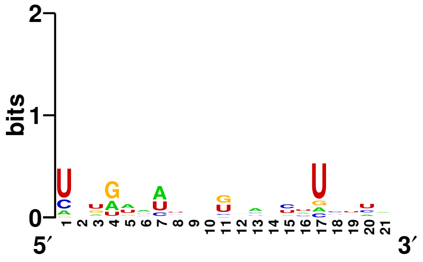

22-mers:

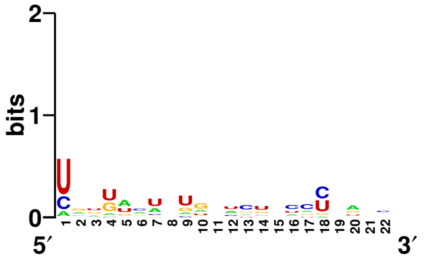

23-mers:

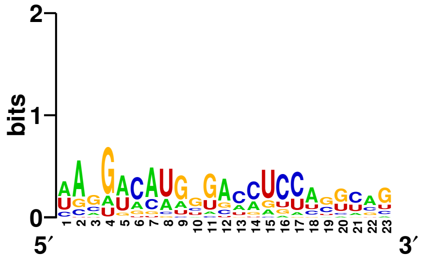

24-mers:

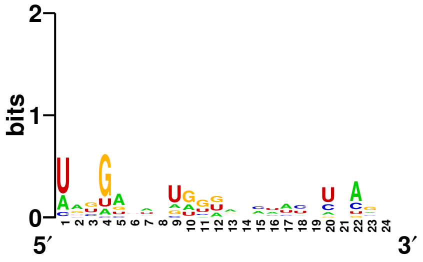

25-mers:

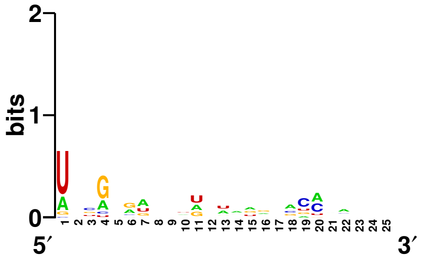

26-mers:

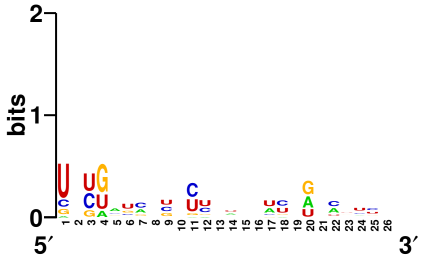

27-mers:

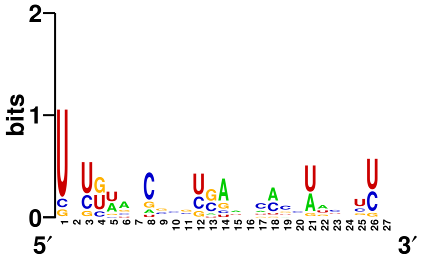

28-mers:

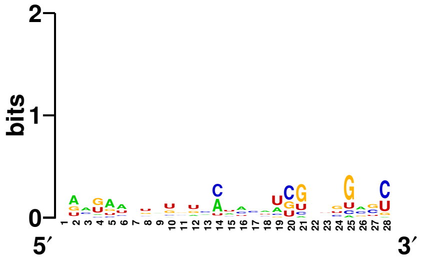

29-mers:

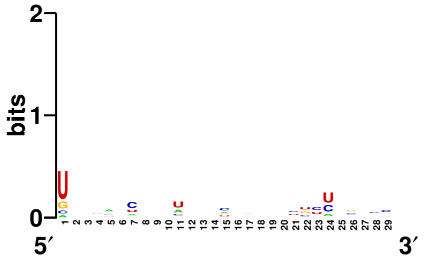

30-mers:

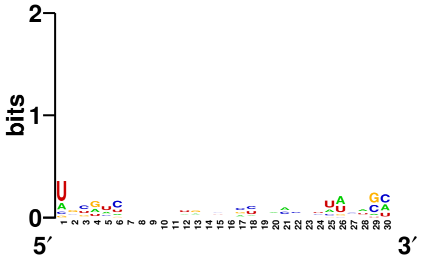

Embryo 15h, library 2:

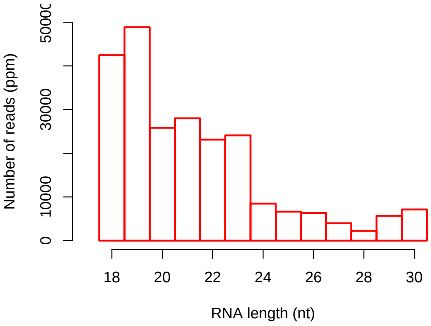

18-mers:

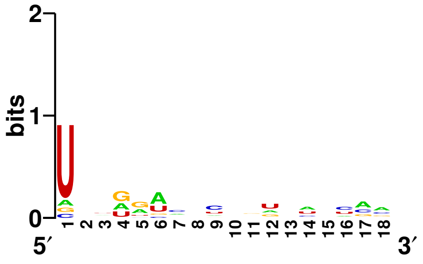

19-mers:

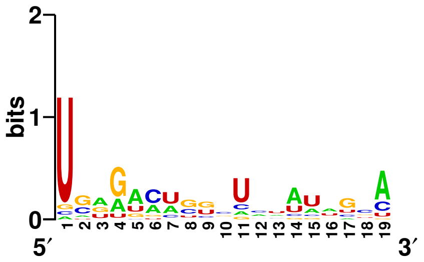

20-mers:

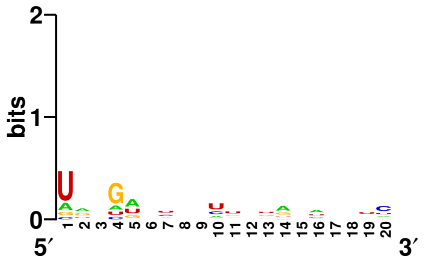

21-mers:

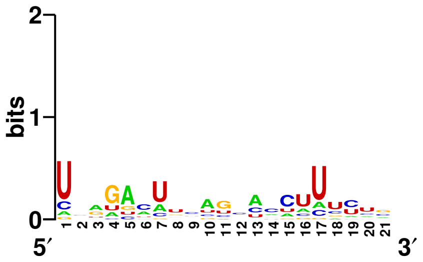

22-mers:

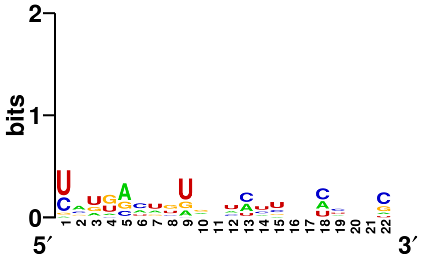

23-mers:

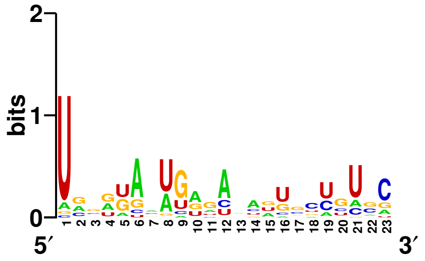

24-mers:

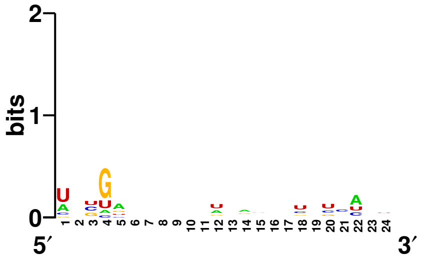

25-mers:

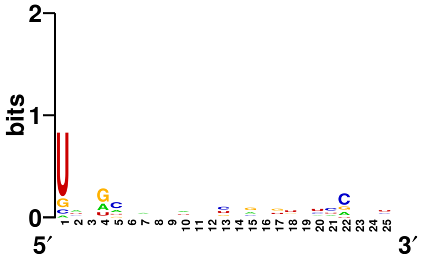

26-mers:

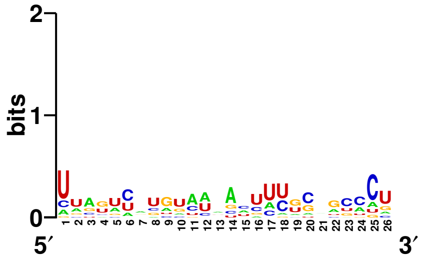

27-mers:

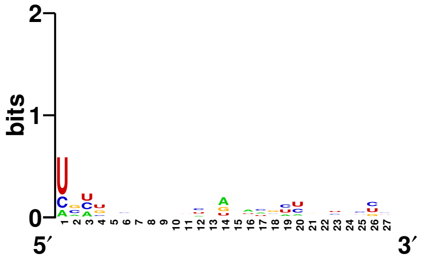

28-mers:

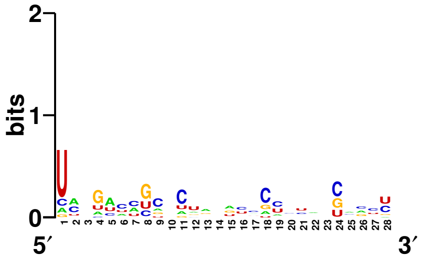

29-mers:

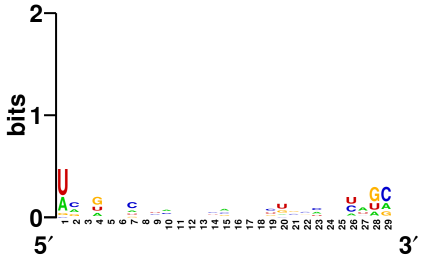

30-mers:

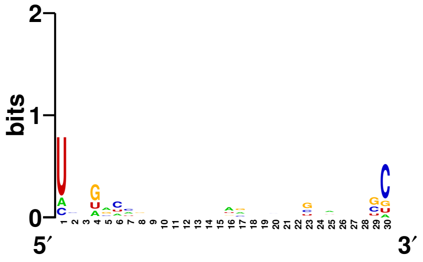

Embryo 36h, library 2:

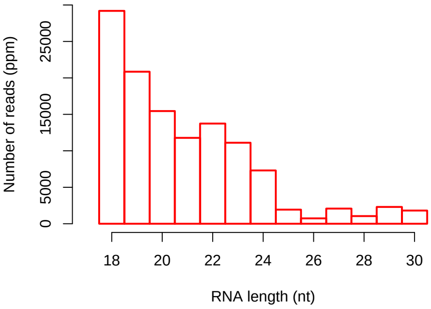

18-mers:

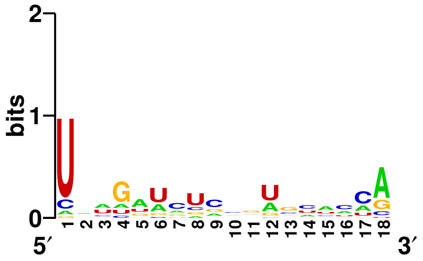

19-mers:

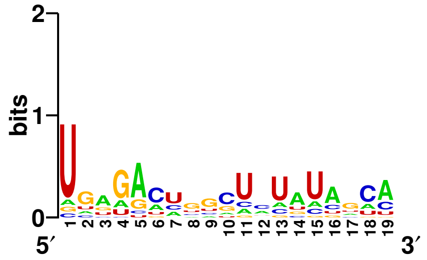

20-mers:

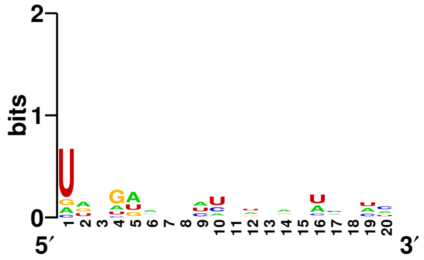

21-mers:

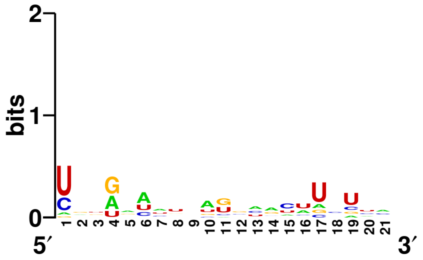

22-mers:

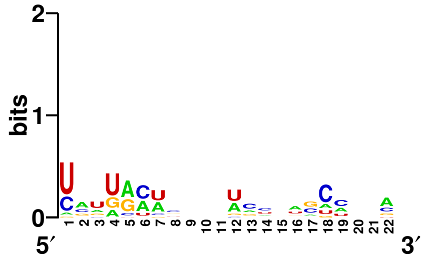

23-mers:

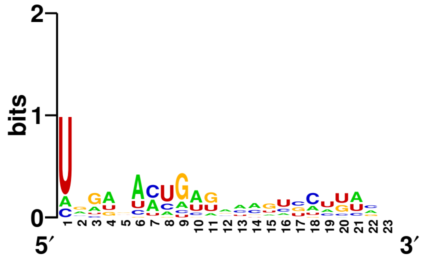

24-mers:

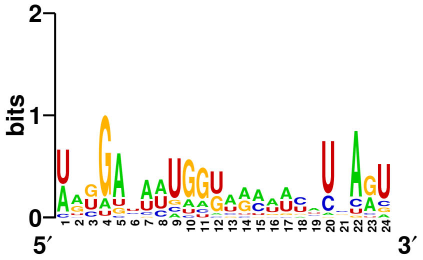

25-mers:

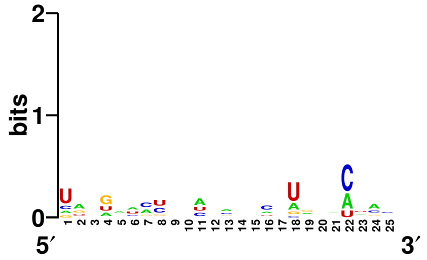

26-mers:

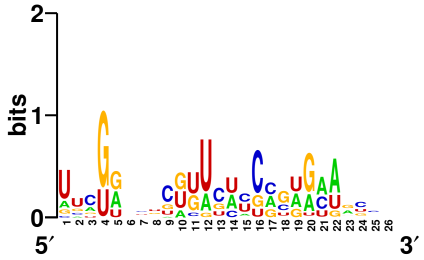

27-mers:

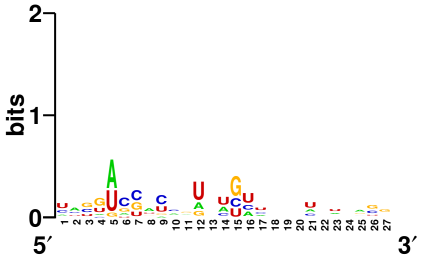

28-mers:

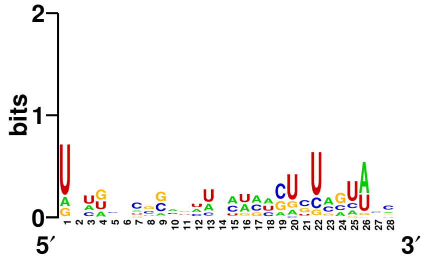

29-mers:

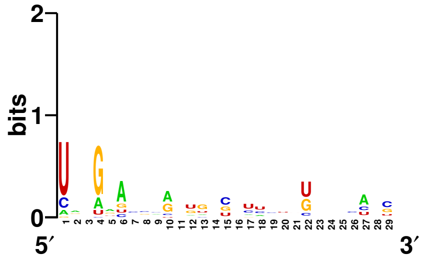

30-mers:

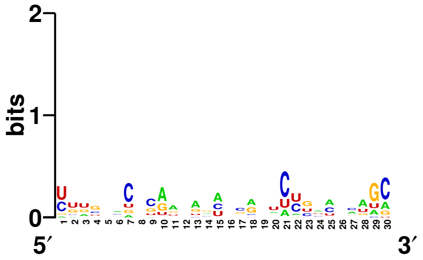

Embryo 60h, library 2:

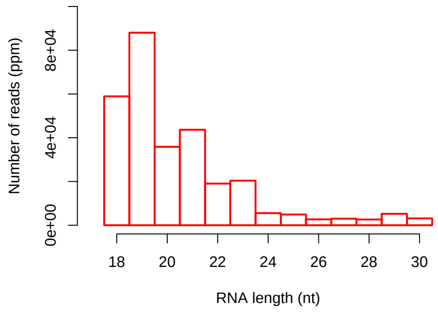

18-mers:

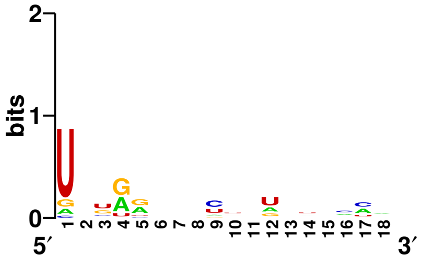

19-mers:

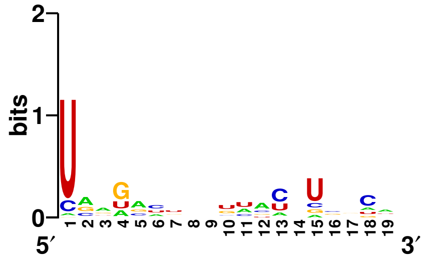

20-mers:

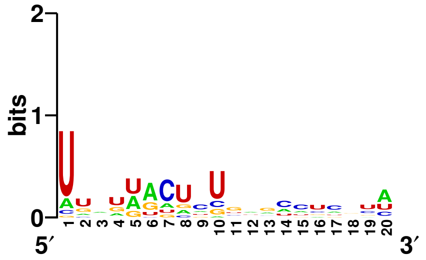

21-mers:

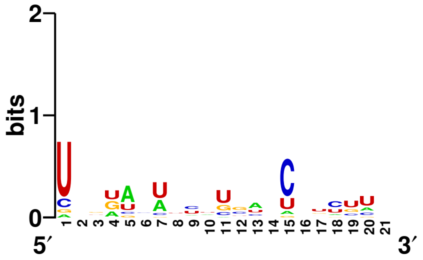

22-mers:

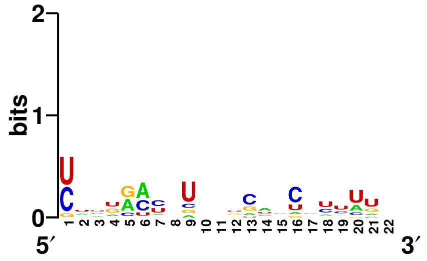

23-mers:

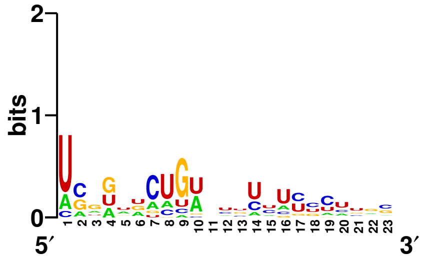

24-mers:

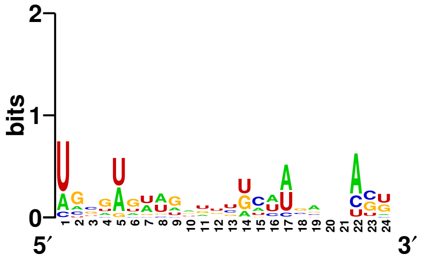

25-mers:

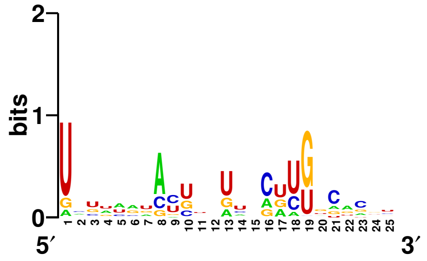

26-mers:

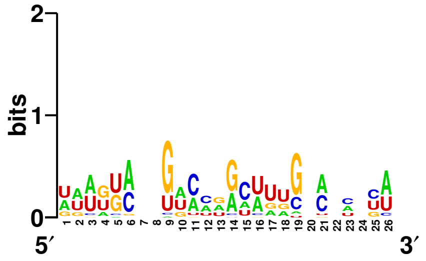

27-mers:

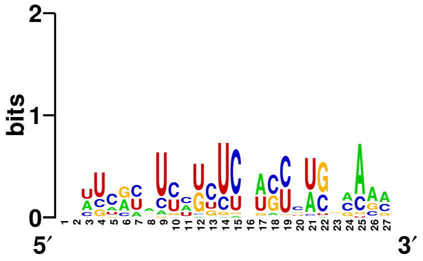

28-mers:

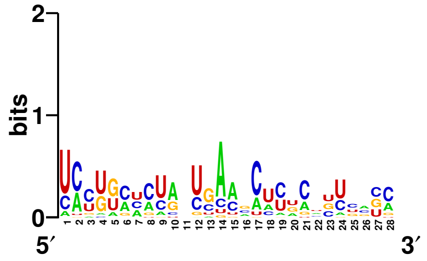

29-mers:

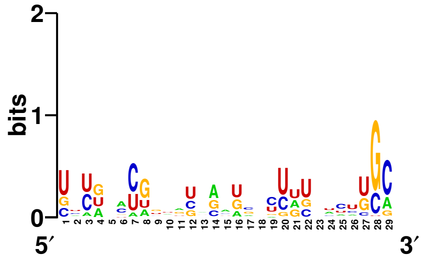

30-mers:

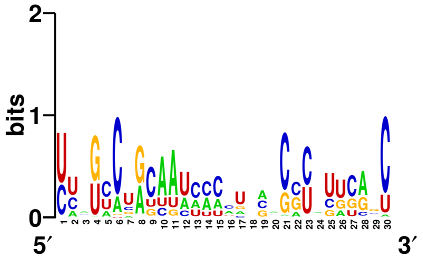

Adult female, library 2:

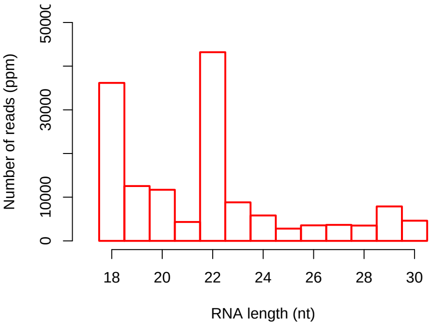

18-mers:

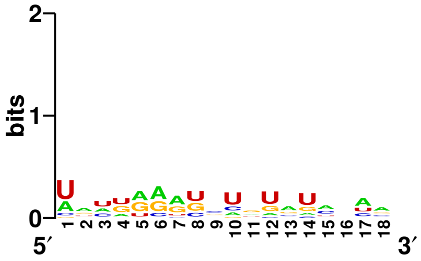

19-mers:

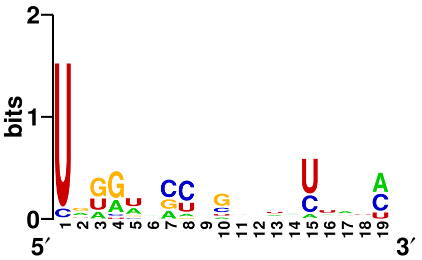

20-mers:

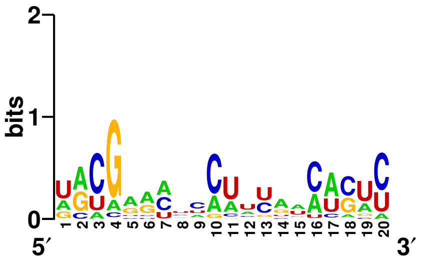

21-mers:

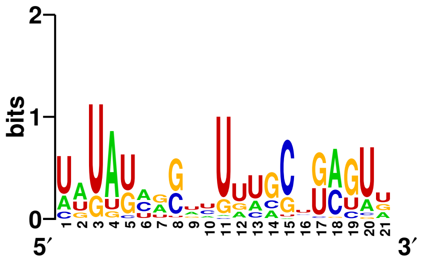

22-mers:

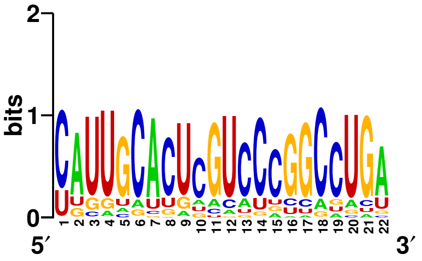

23-mers:

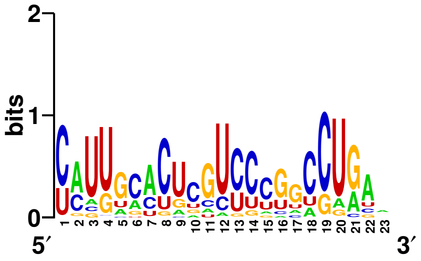

24-mers:

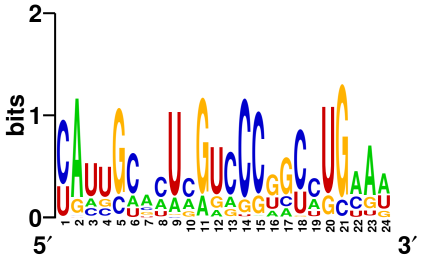

25-mers:

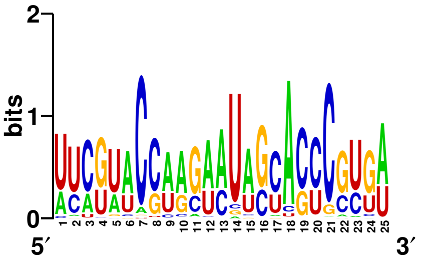

26-mers:

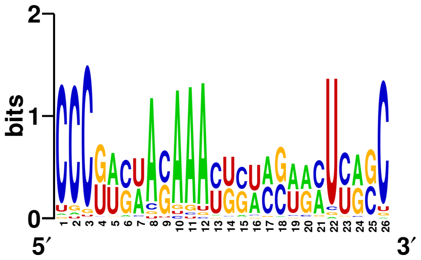

27-mers:

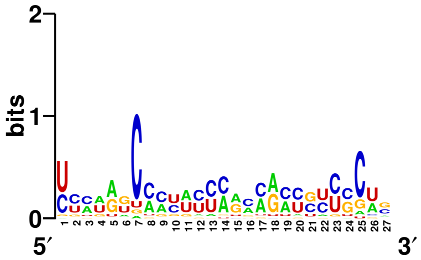

28-mers:

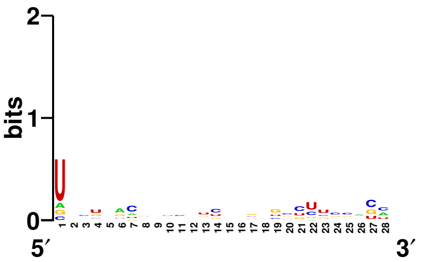

29-mers:

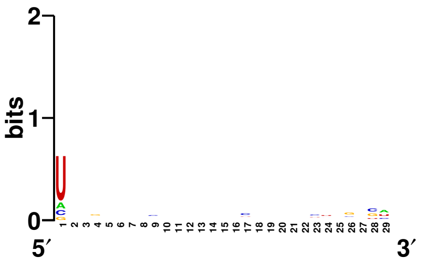

30-mers:

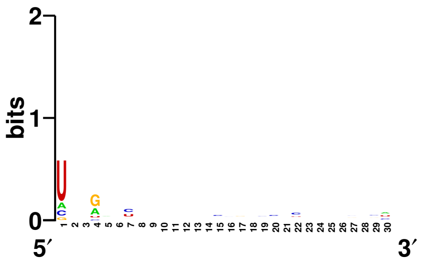

Adult male, library 2:

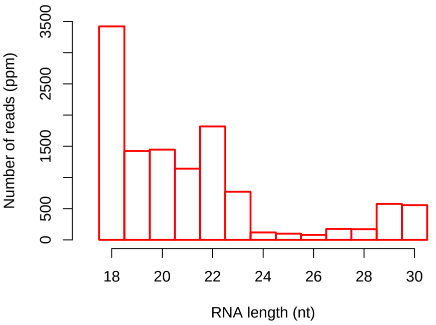

18-mers:

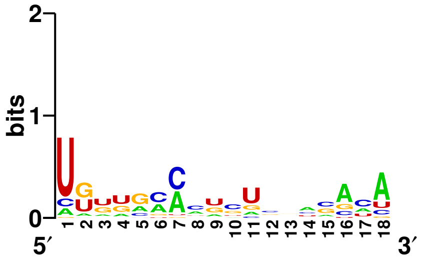

19-mers:

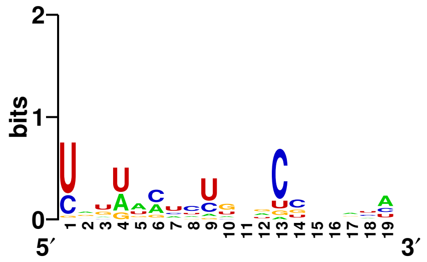

20-mers:

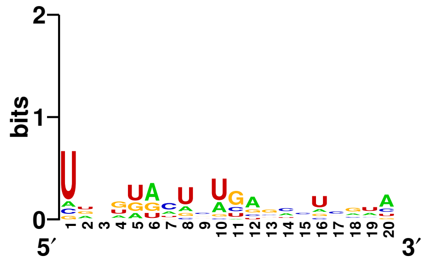

21-mers:

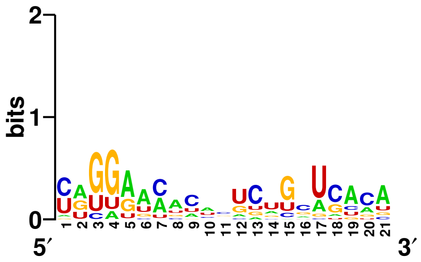

22-mers:

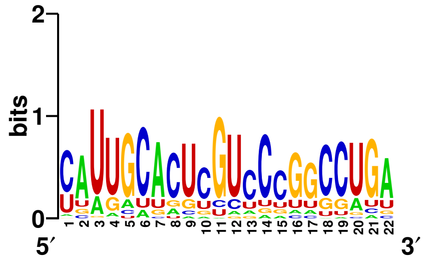

23-mers:

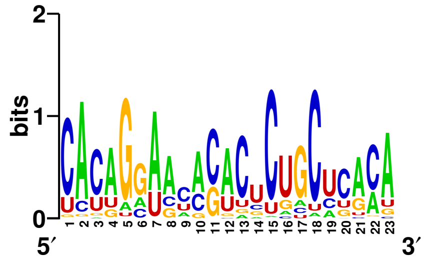

24-mers:

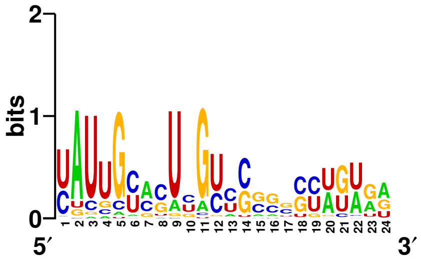

25-mers:

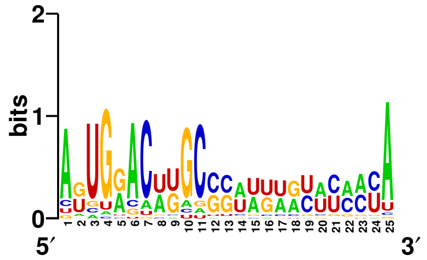

26-mers:

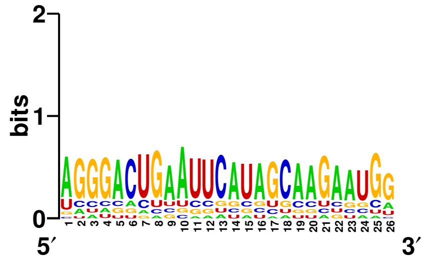

27-mers:

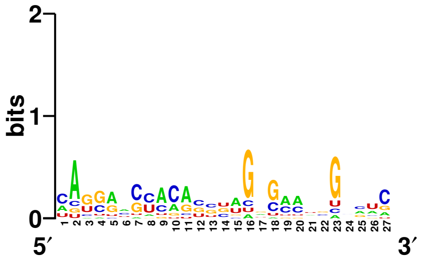

28-mers:

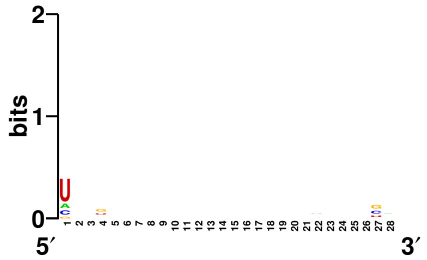

29-mers:

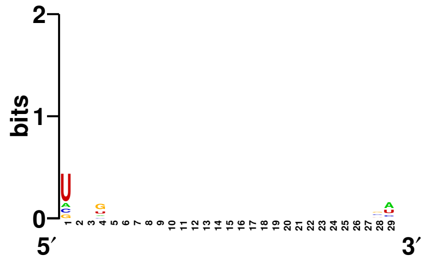

30-mers:

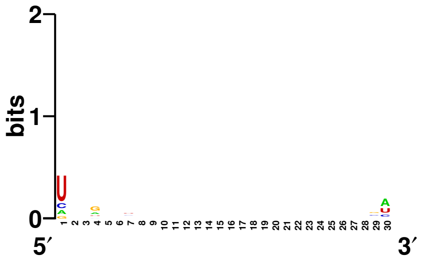

1.3 Libraries #3 (total 5´ hydroxyl or polyphosphorylated small RNAs)

Embryo 8h, library 3:

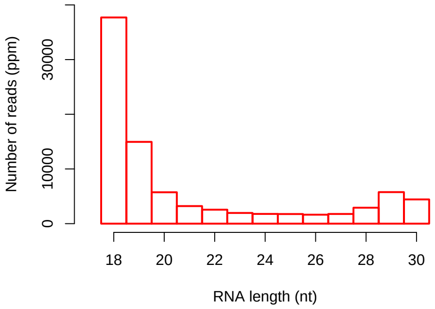

18-mers:

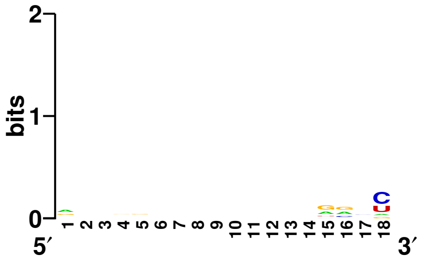

19-mers:

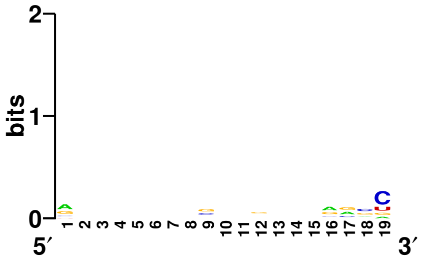

20-mers:

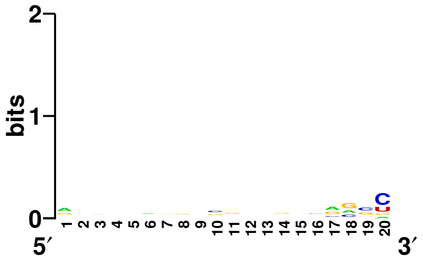

21-mers:

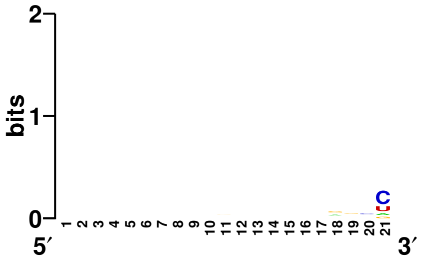

22-mers:

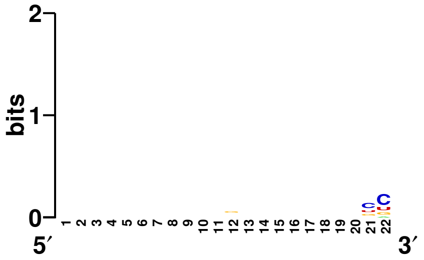

23-mers:

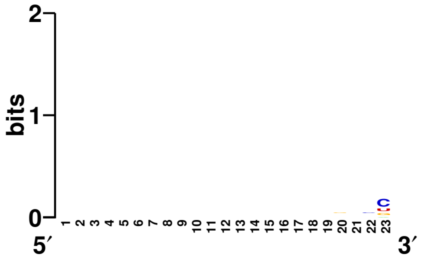

24-mers:

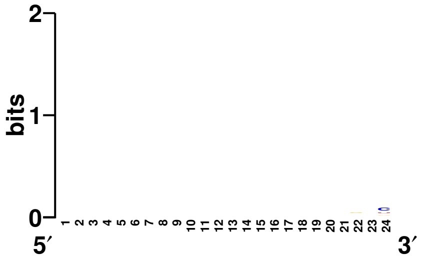

25-mers:

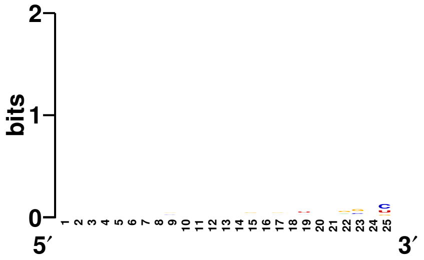

26-mers:

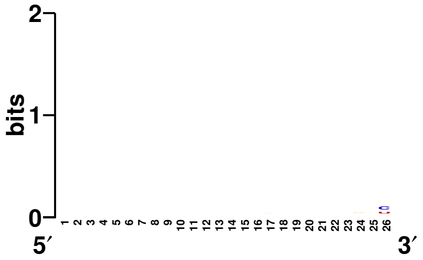

27-mers:

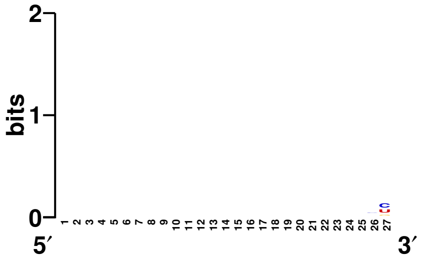

28-mers:

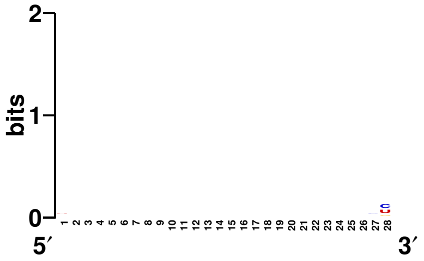

29-mers:

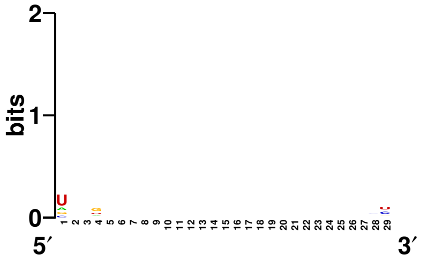

30-mers:

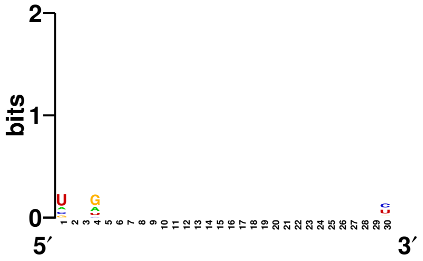

Embryo 15h, library 3:

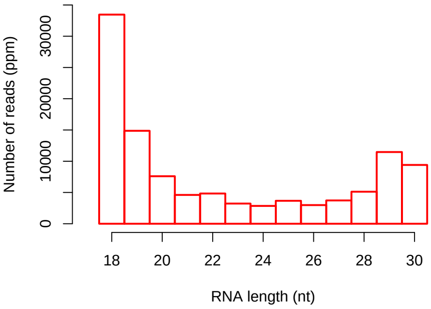

18-mers:

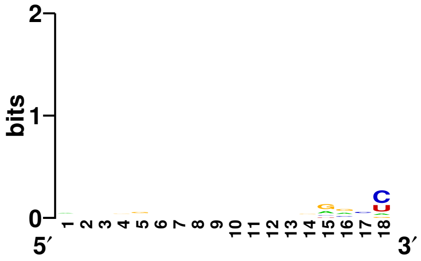

19-mers:

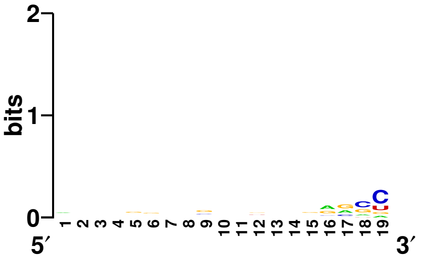

20-mers:

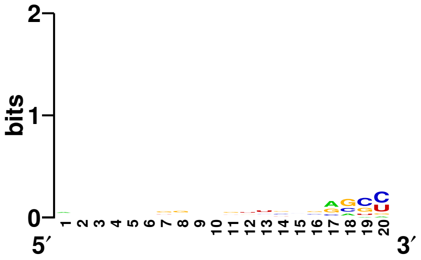

21-mers:

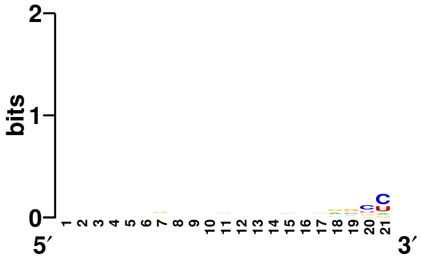

22-mers:

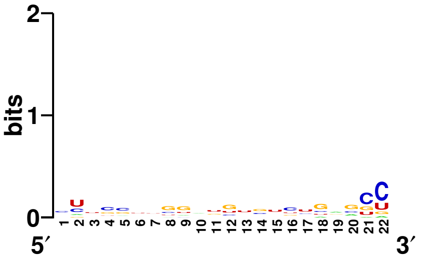

23-mers:

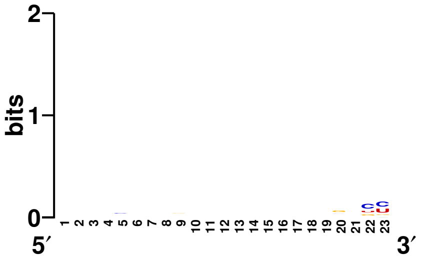

24-mers:

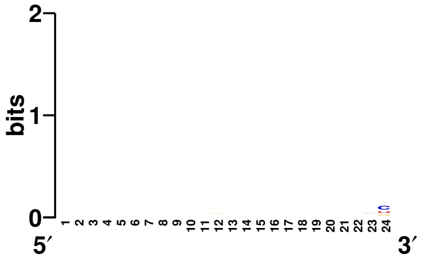

25-mers:

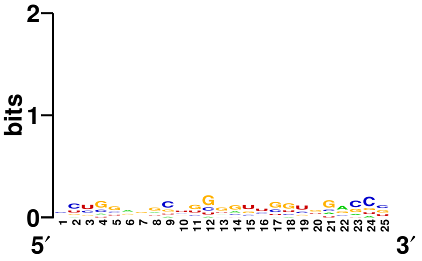

26-mers:

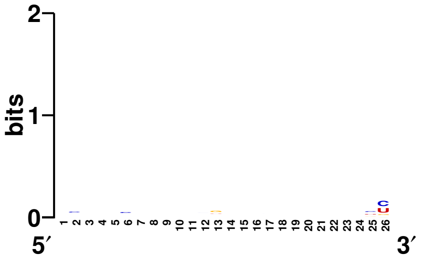

27-mers:

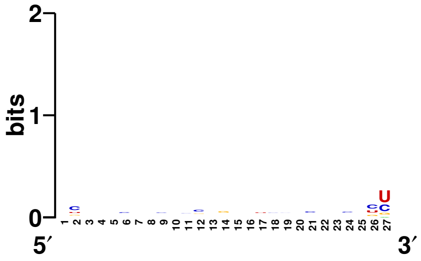

28-mers:

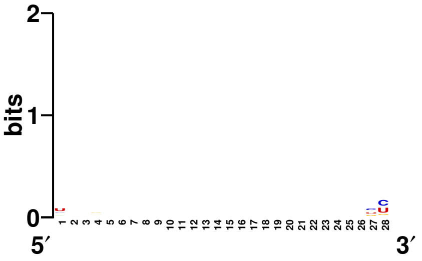

29-mers:

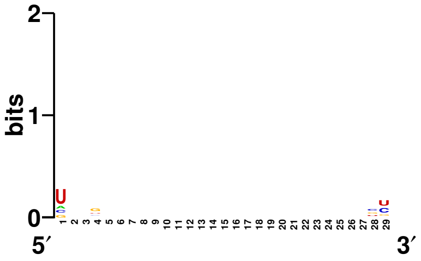

30-mers:

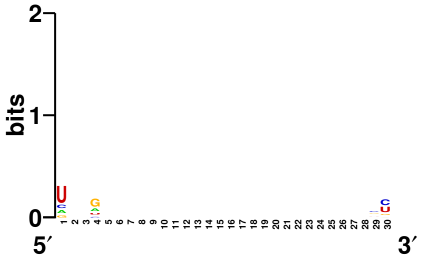

Embryo 36h, library 3:

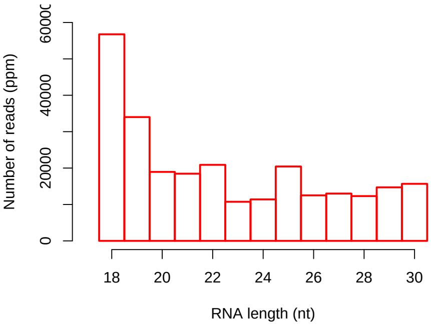

18-mers:

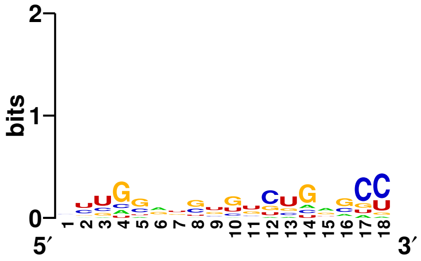

19-mers:

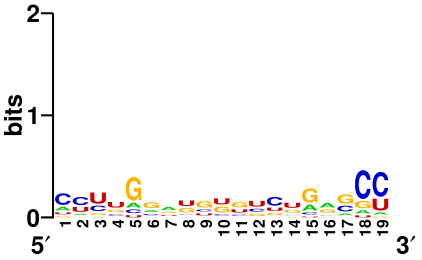

20-mers:

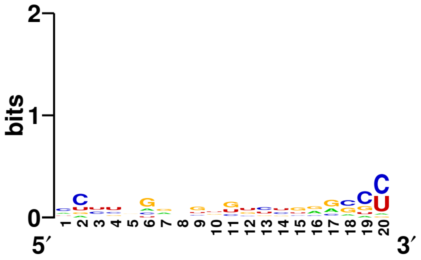

21-mers:

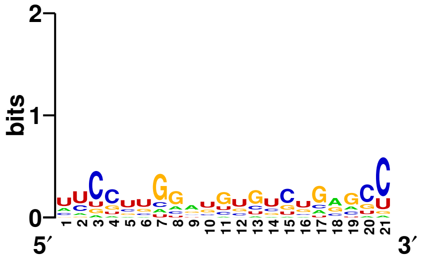

22-mers:

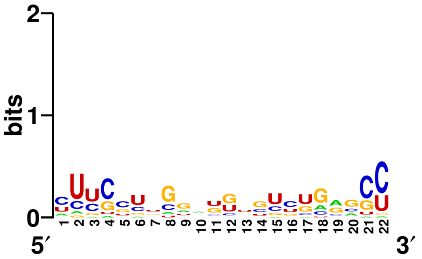

23-mers:

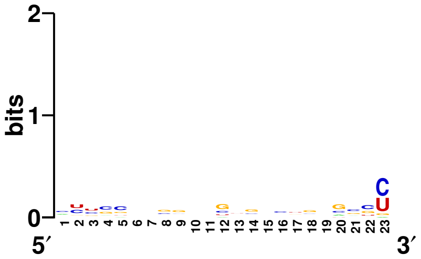

24-mers:

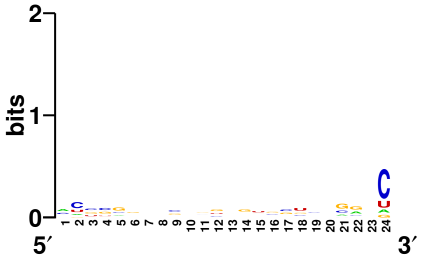

25-mers:

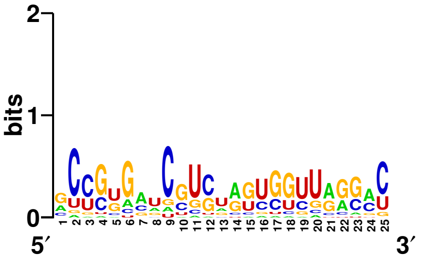

26-mers:

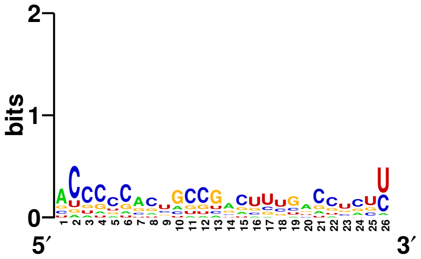

27-mers:

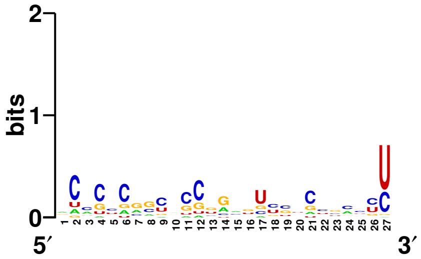

28-mers:

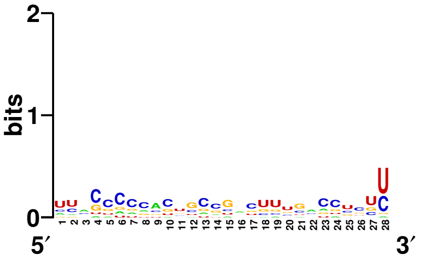

29-mers:

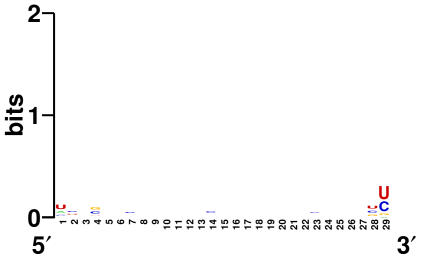

30-mers:

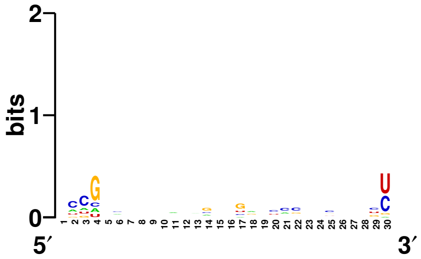

Embryo 60h, library 3:

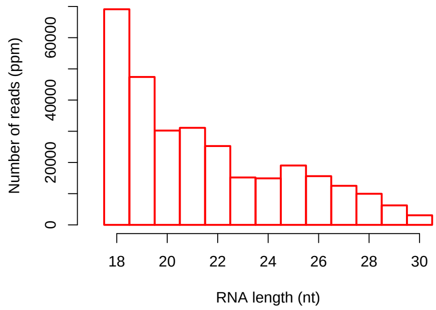

18-mers:

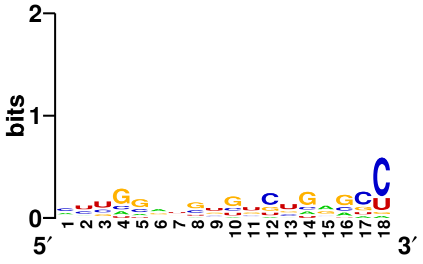

19-mers:

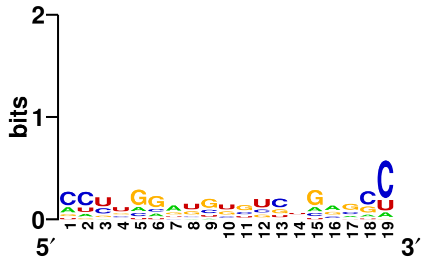

20-mers:

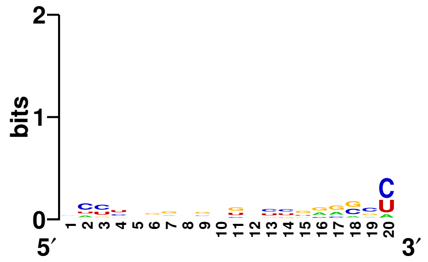

21-mers:

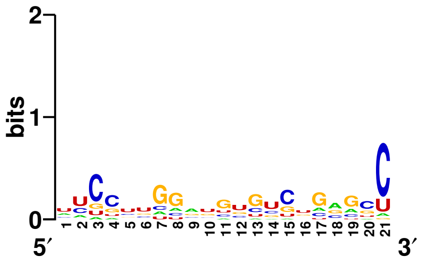

22-mers:

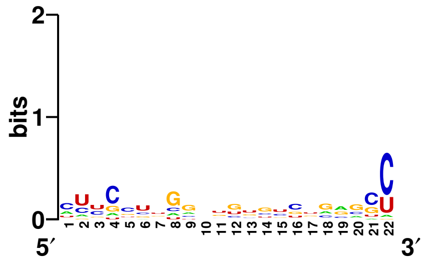

23-mers:

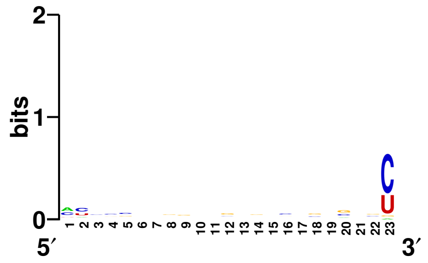

24-mers:

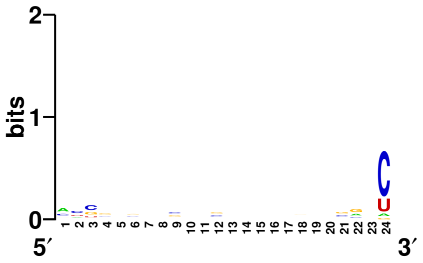

25-mers:

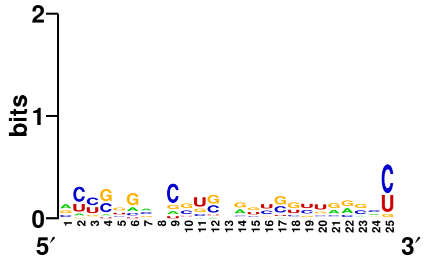

26-mers:

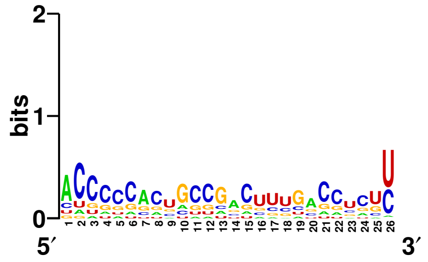

27-mers:

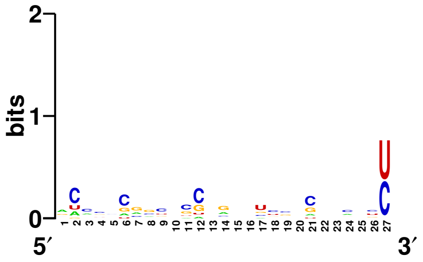

28-mers:

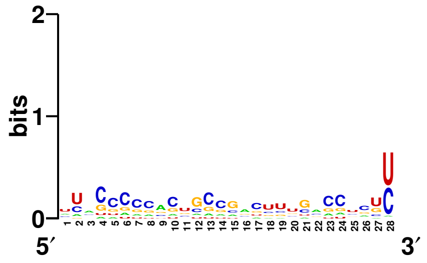

29-mers:

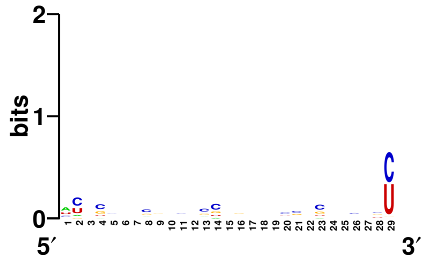

30-mers:

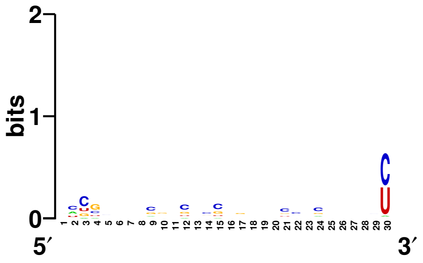

Adult female, library 3:

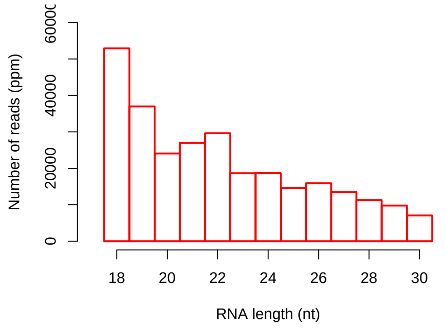

18-mers:

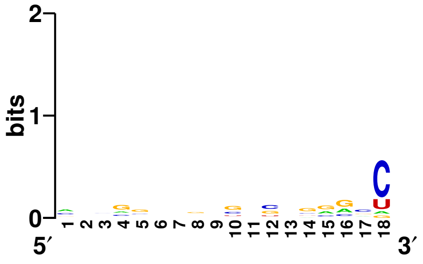

19-mers:

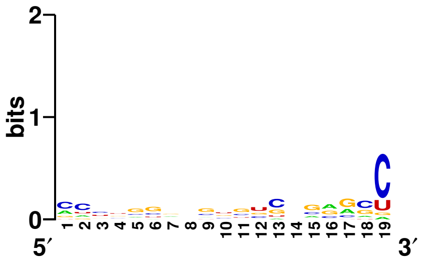

20-mers:

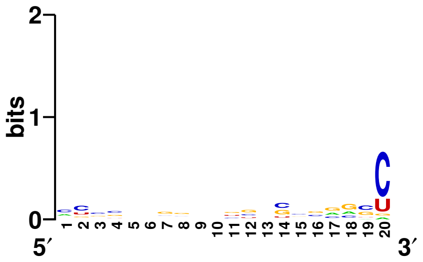

21-mers:

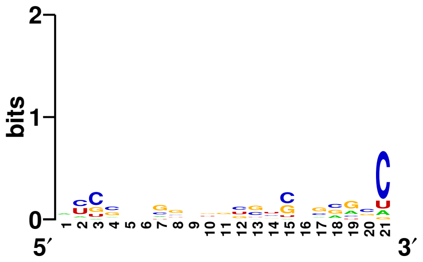

22-mers:

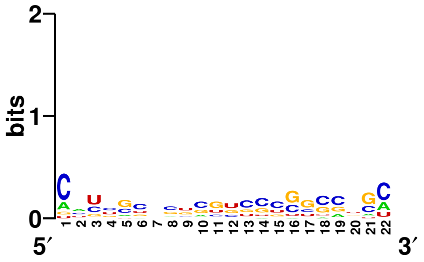

23-mers:

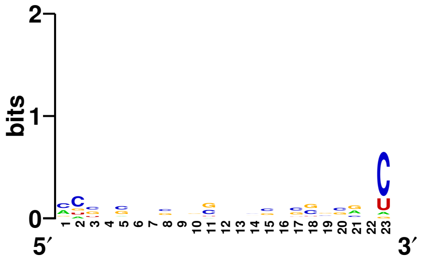

24-mers:

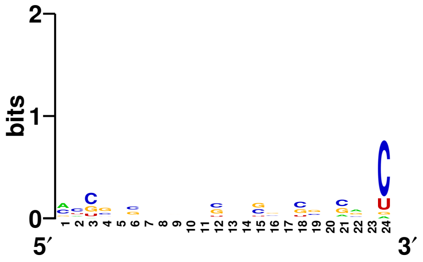

25-mers:

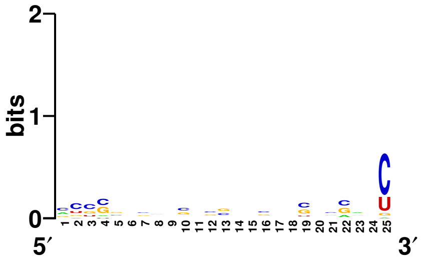

26-mers:

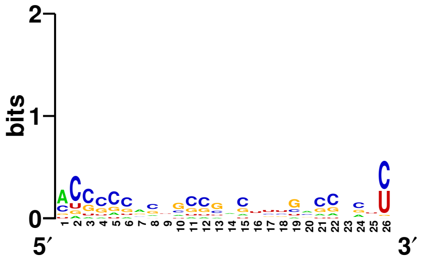

27-mers:

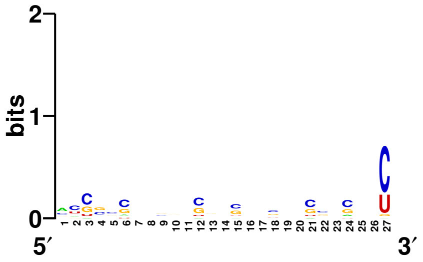

28-mers:

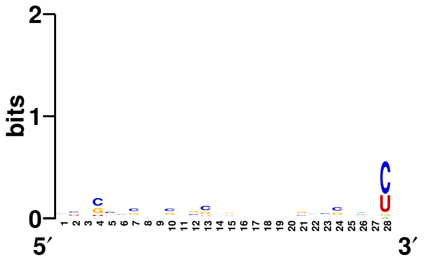

29-mers:

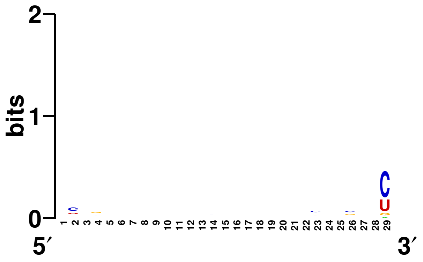

30-mers:

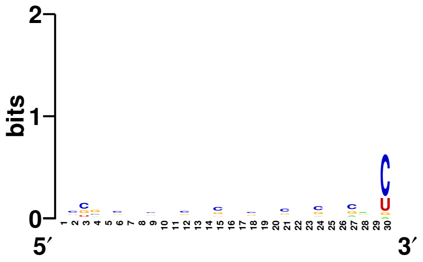

Adult male, library 3:

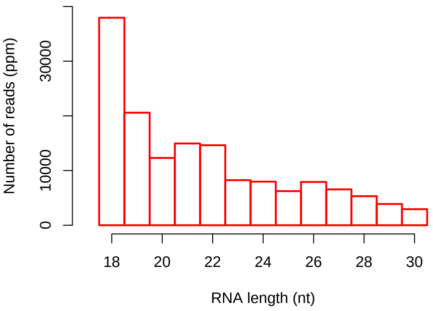

18-mers:

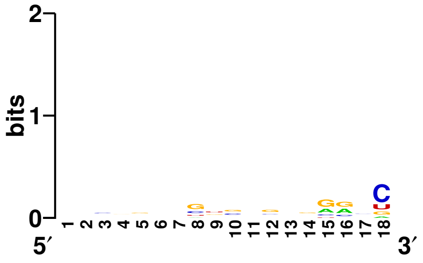

19-mers:

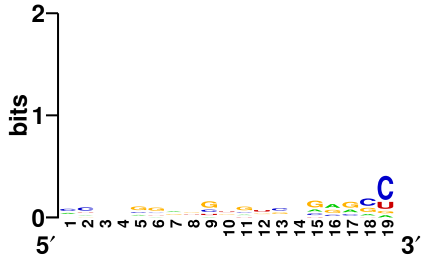

20-mers:

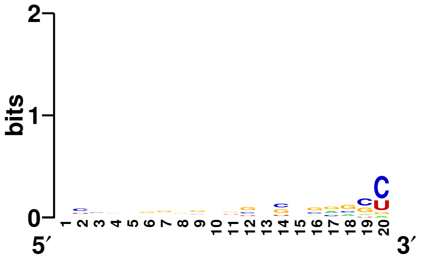

21-mers:

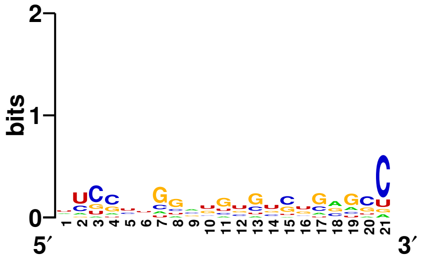

22-mers:

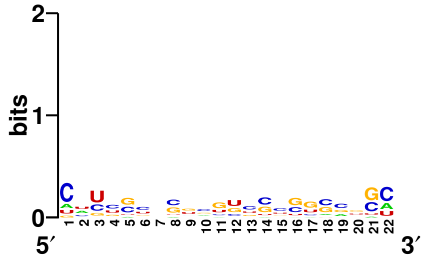

23-mers:

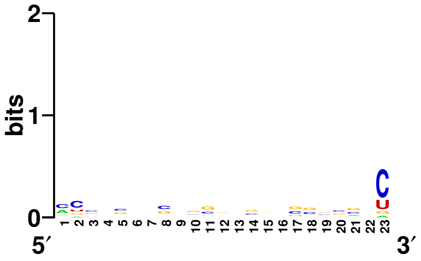

24-mers:

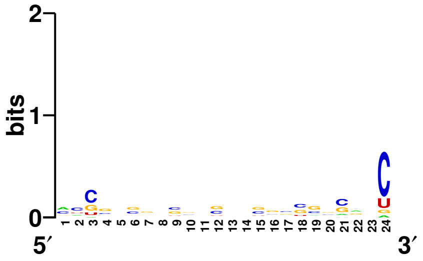

25-mers:

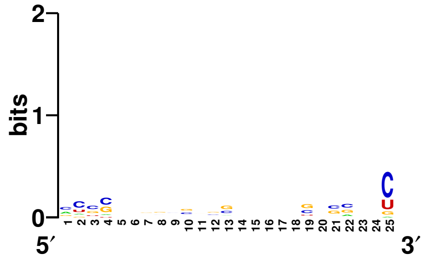

26-mers:

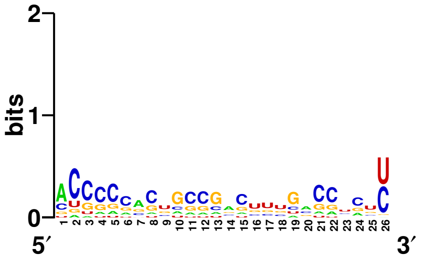

27-mers:

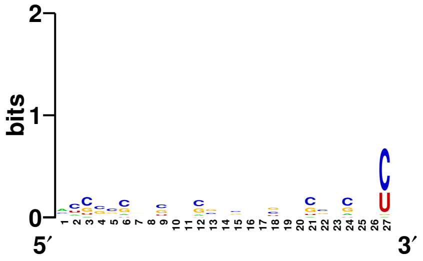

28-mers:

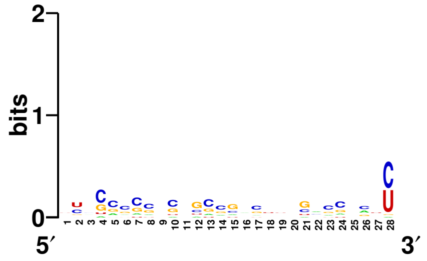

29-mers:

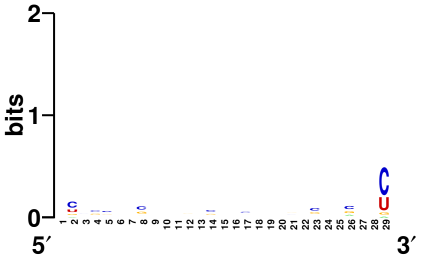

30-mers:

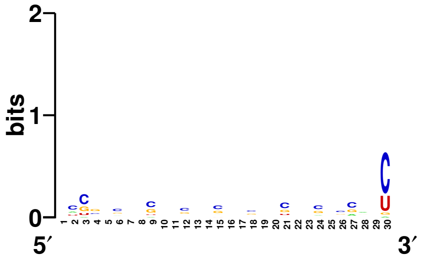

1.4 Libraries #4 (3´ modified, 5´ hydroxyl or polyphosphorylated small RNAs)

Embryo 8h, library 4:

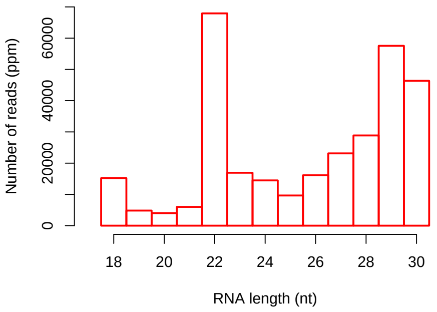

18-mers:

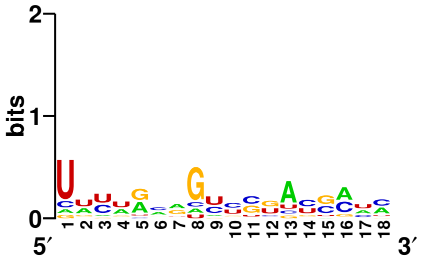

19-mers:

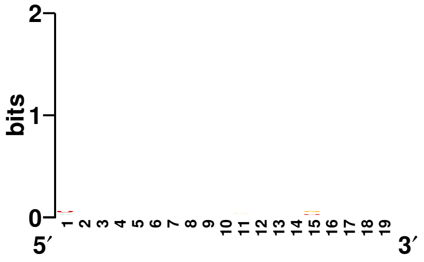

20-mers:

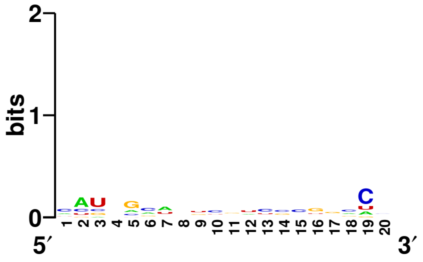

21-mers:

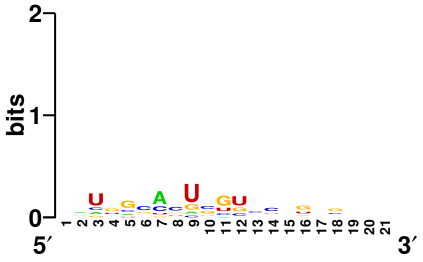

22-mers:

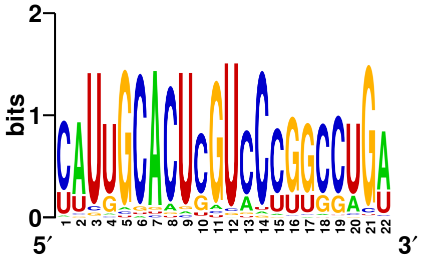

23-mers:

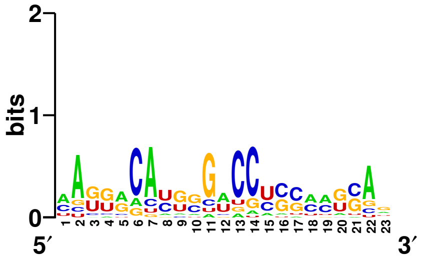

24-mers:

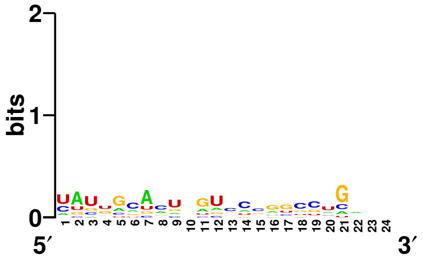

25-mers:

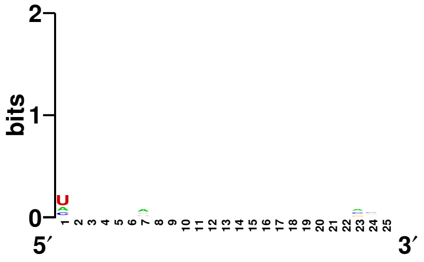

26-mers:

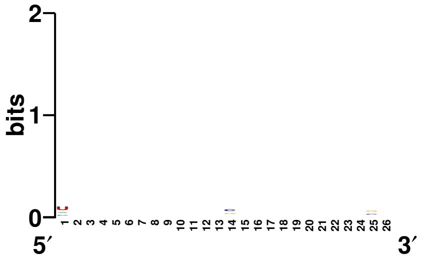

27-mers:

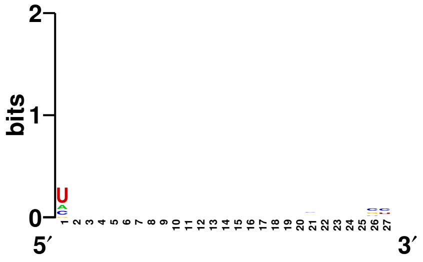

28-mers:

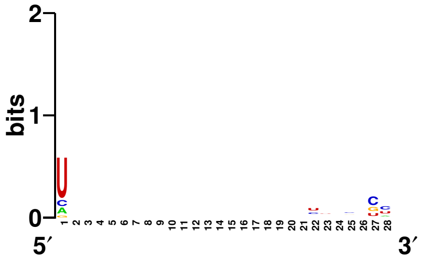

29-mers:

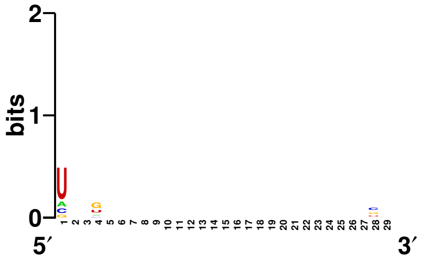

30-mers:

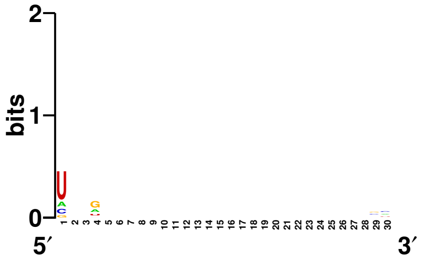

Embryo 15h, library 4:

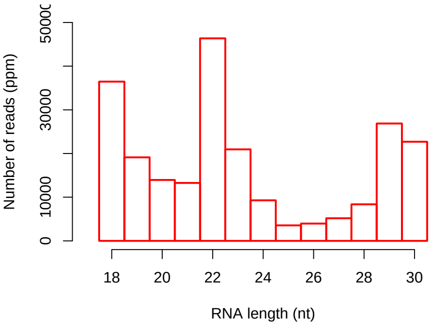

18-mers:

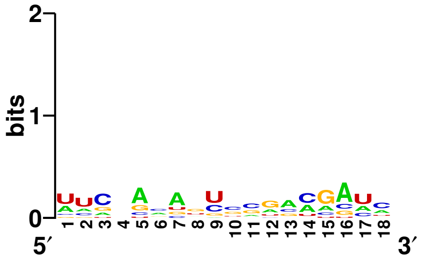

19-mers:

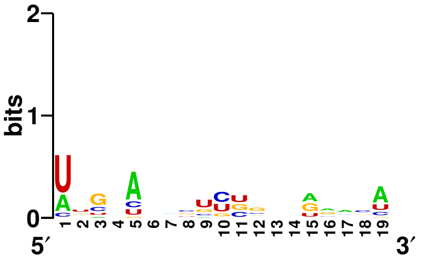

20-mers:

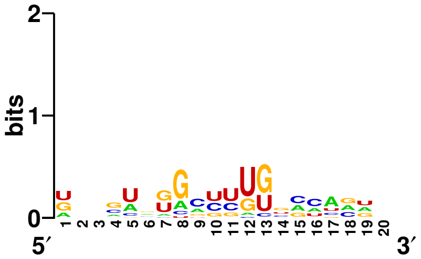

21-mers:

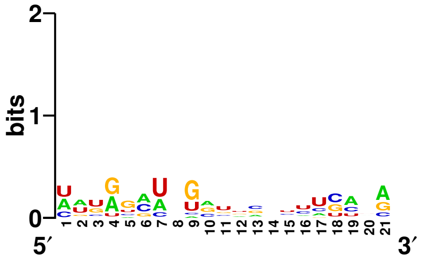

22-mers:

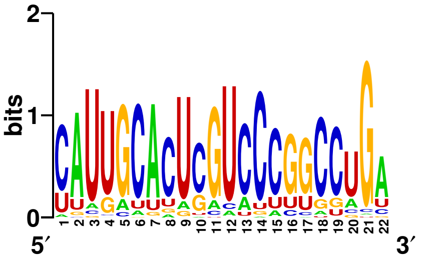

23-mers:

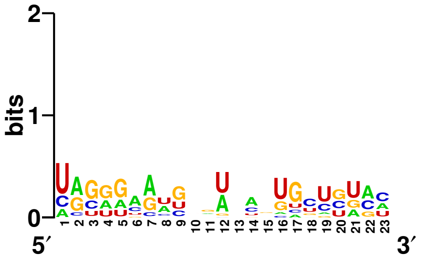

24-mers:

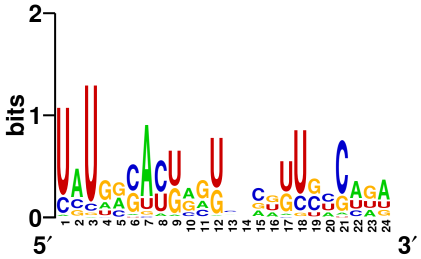

25-mers:

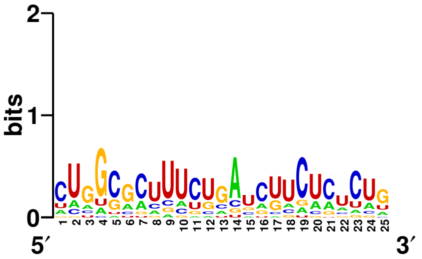

26-mers:

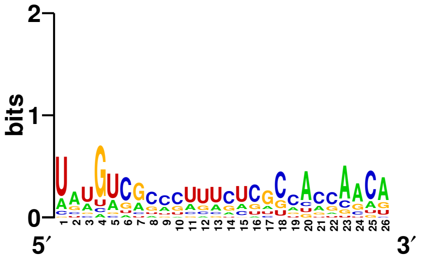

27-mers:

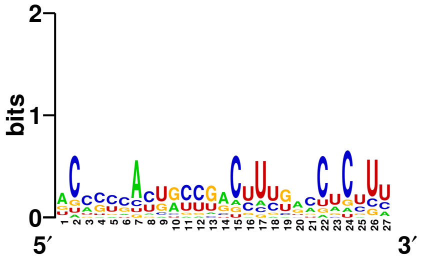

28-mers:

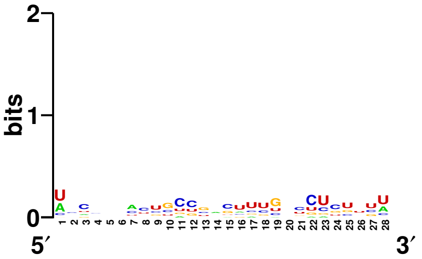

29-mers:

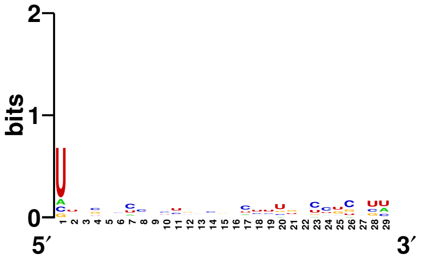

30-mers:

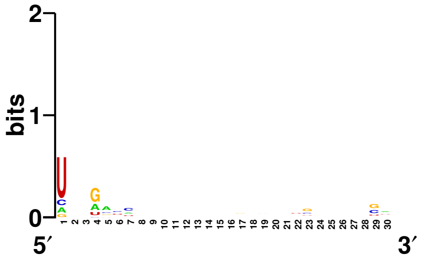

Embryo 36h, library 4:

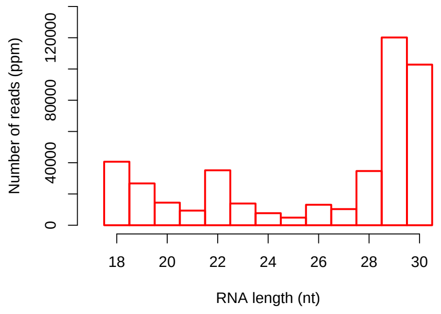

18-mers:

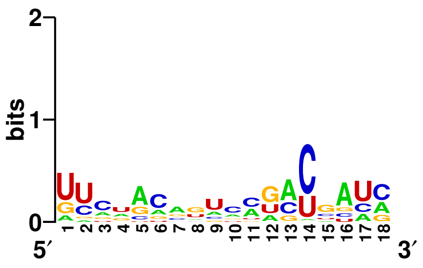

19-mers:

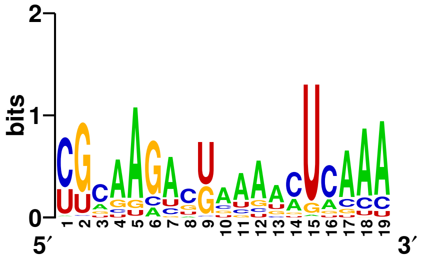

20-mers:

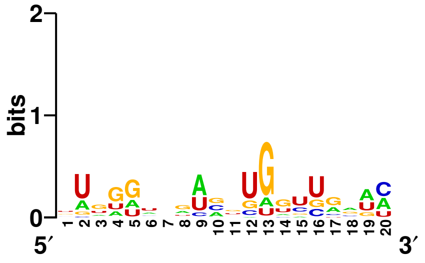

21-mers:

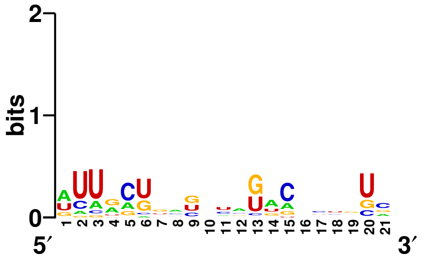

22-mers:

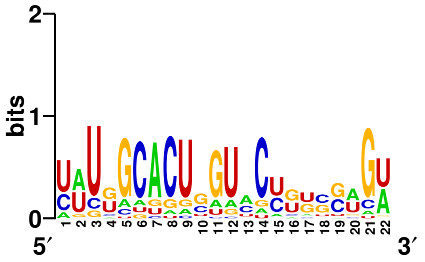

23-mers:

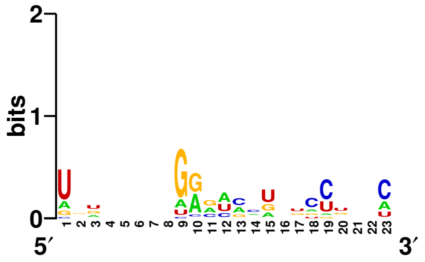

24-mers:

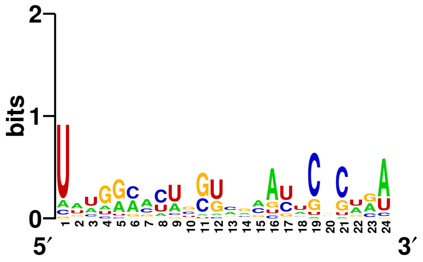

25-mers:

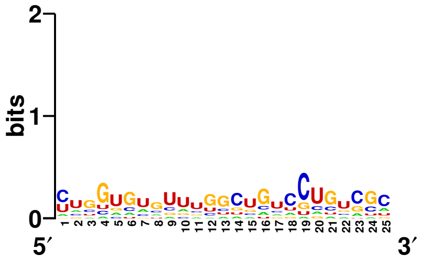

26-mers:

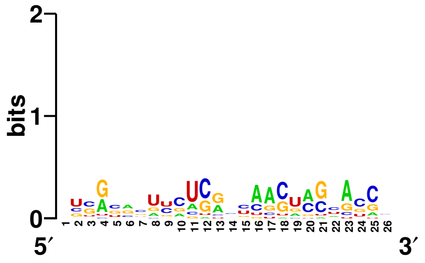

27-mers:

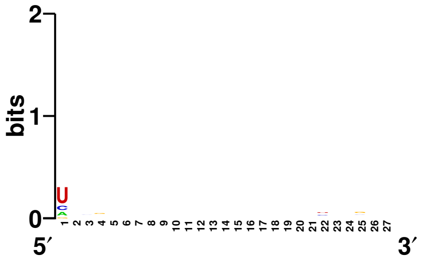

28-mers:

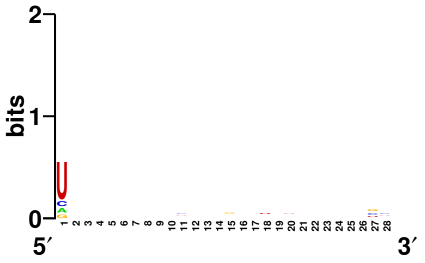

29-mers:

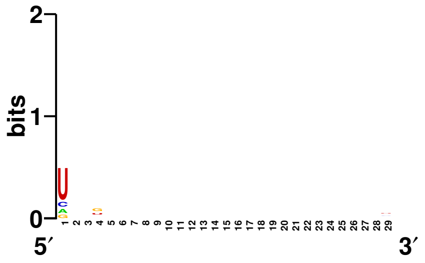

30-mers:

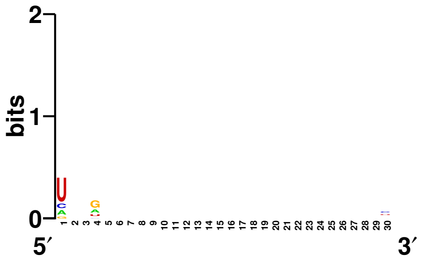

Embryo 60h, library 4:

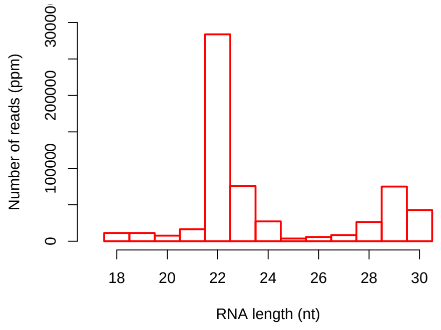

18-mers:

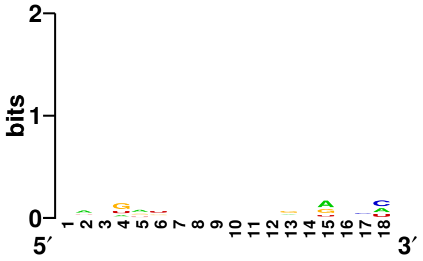

19-mers:

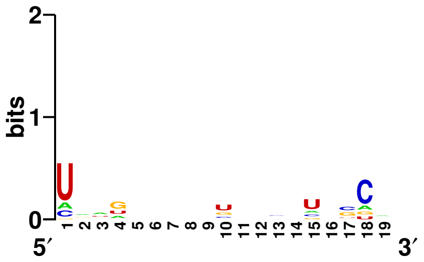

20-mers:

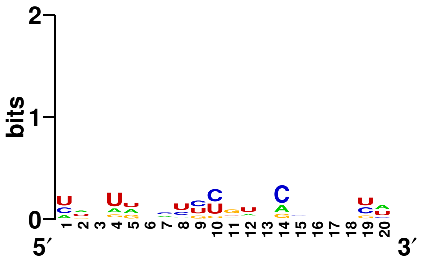

21-mers:

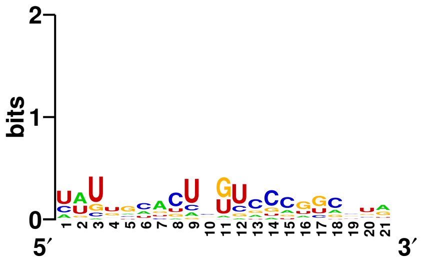

22-mers:

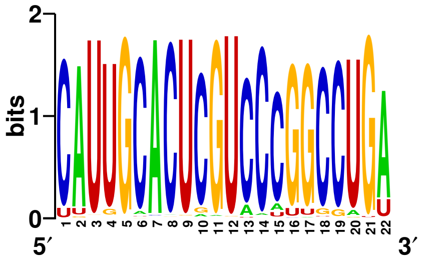

23-mers:

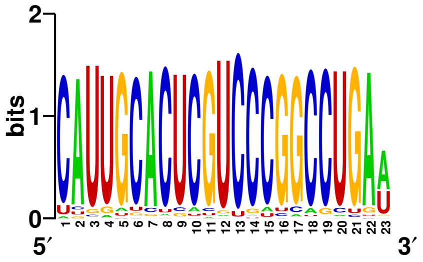

24-mers:

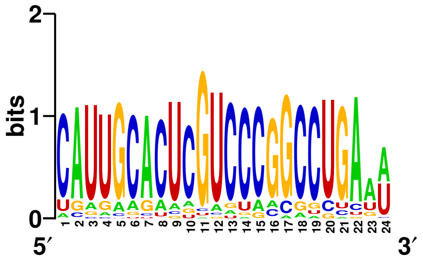

25-mers:

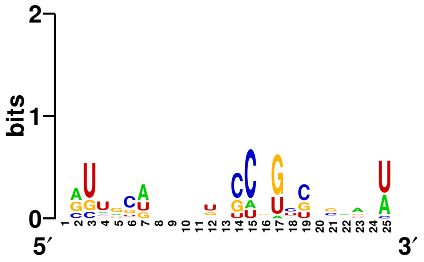

26-mers:

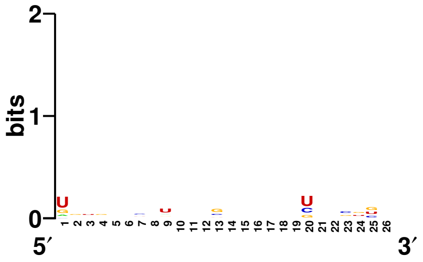

27-mers:

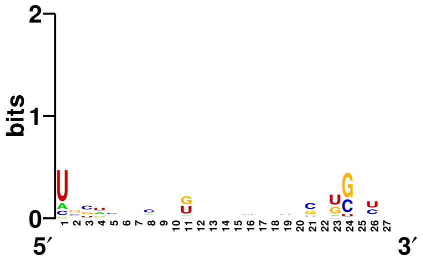

28-mers:

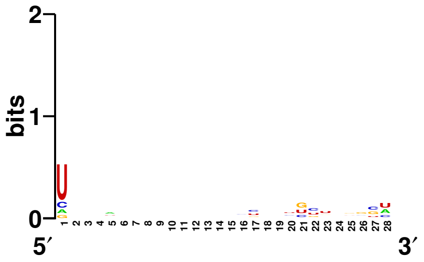

29-mers:

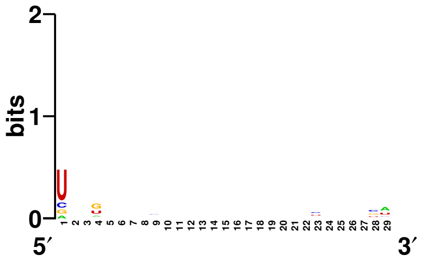

30-mers:

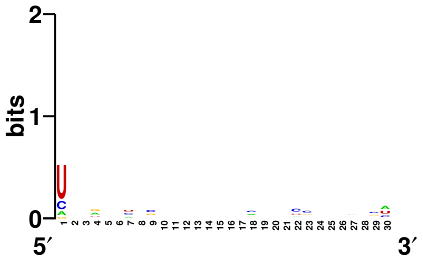

Adult female, library 4:

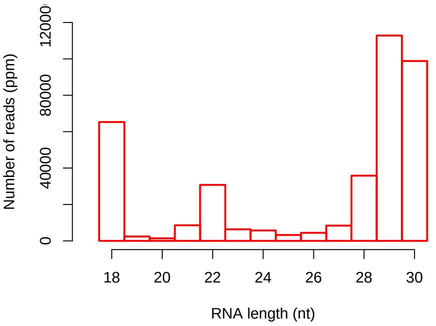

18-mers:

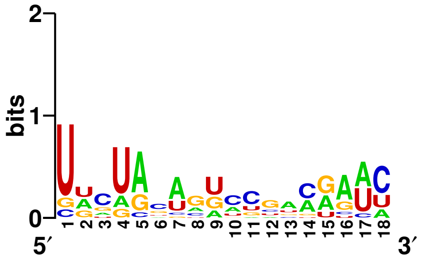

19-mers:

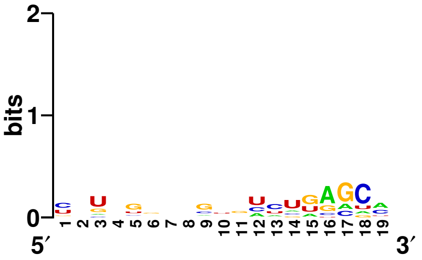

20-mers:

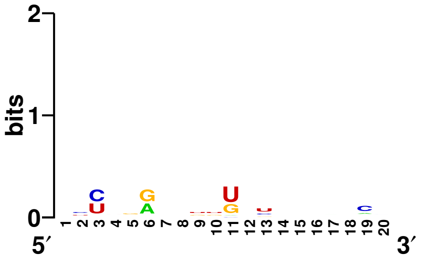

21-mers:

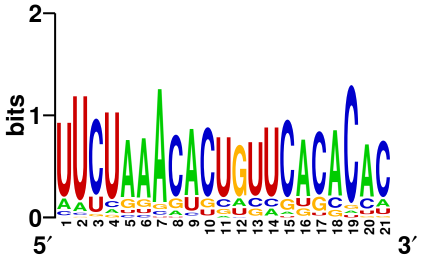

22-mers:

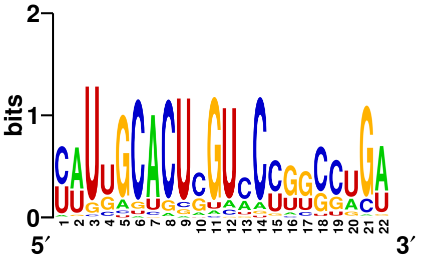

23-mers:

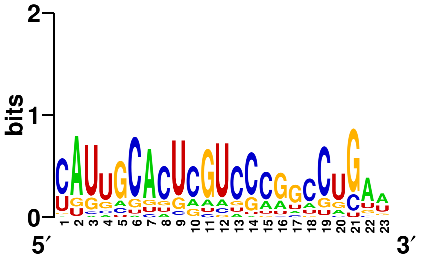

24-mers:

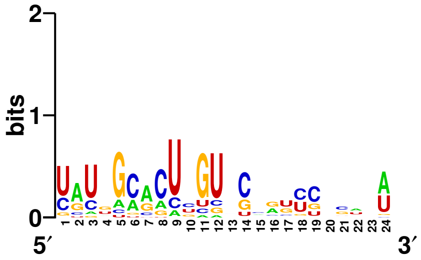

25-mers:

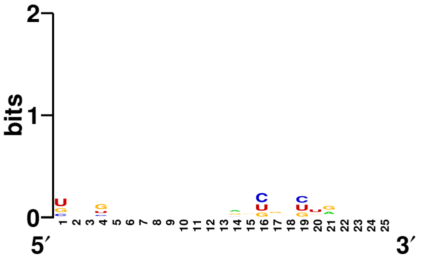

26-mers:

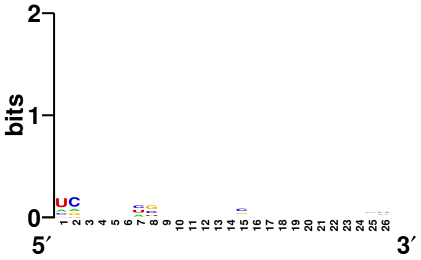

27-mers:

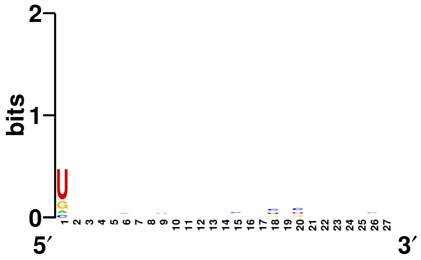

28-mers:

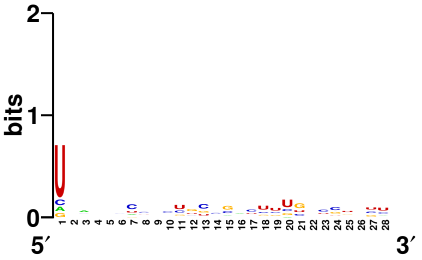

29-mers:

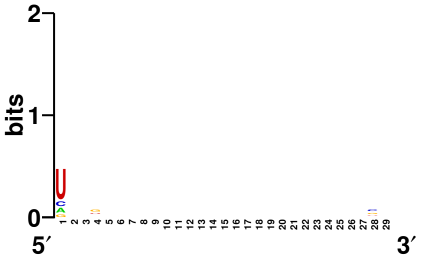

30-mers:

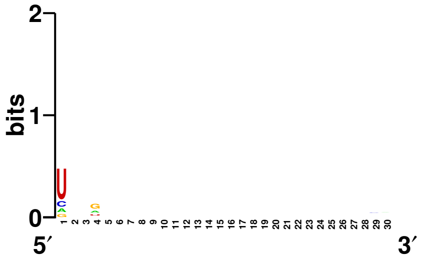

Adult male, library 4:

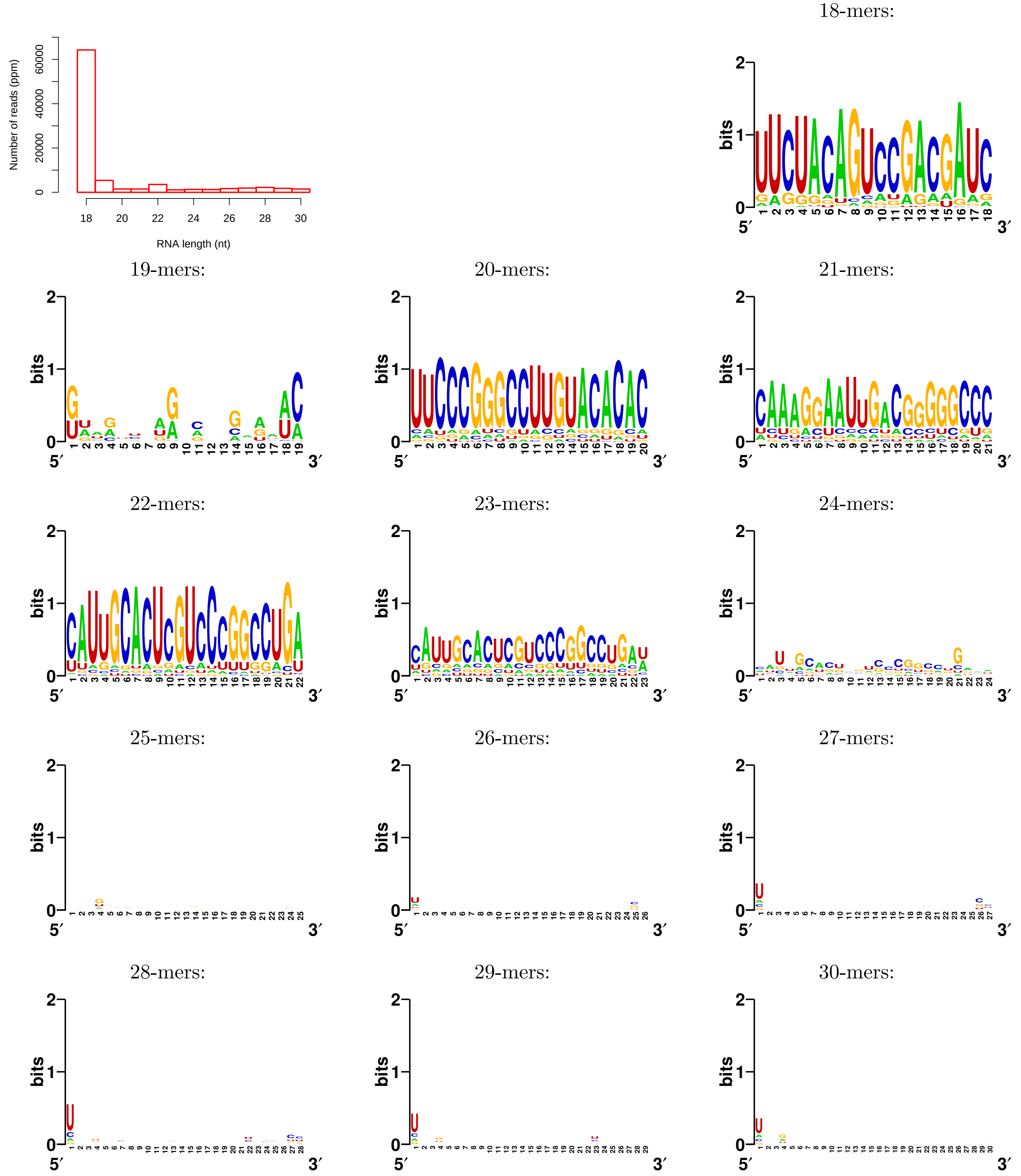

2 pre-miRNA hairpin-matching reads

2.1 Libraries #1 (total 5´ monophosphorylated small RNAs)

Embryo 8h, library 1:

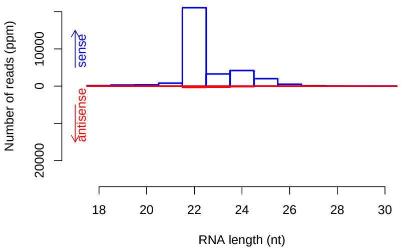

Sense reads:

18-mers:

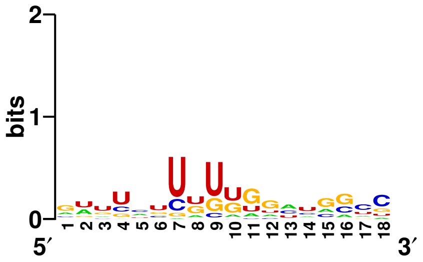

19-mers:

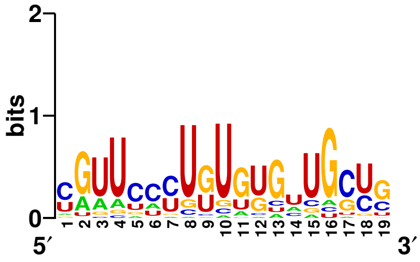

20-mers:

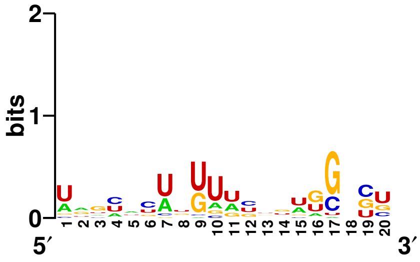

21-mers:

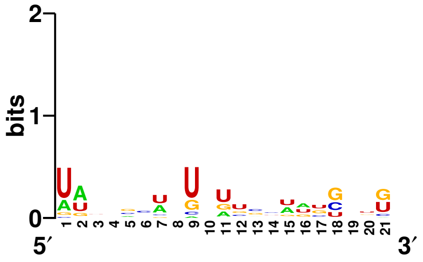

22-mers:

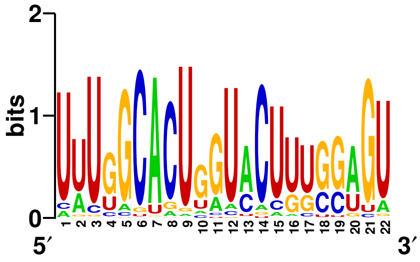

23-mers:

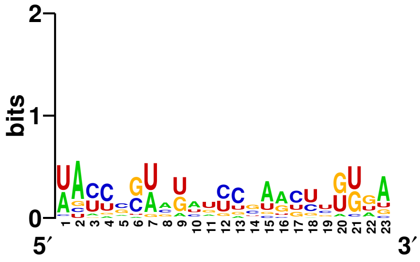

24-mers:

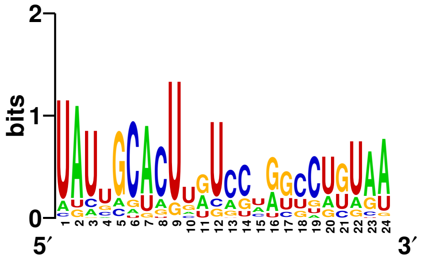

25-mers:

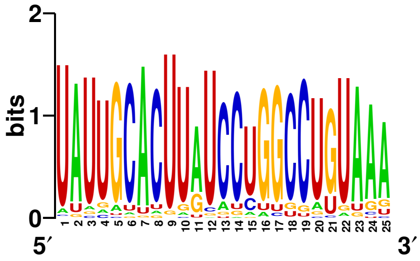

26-mers:

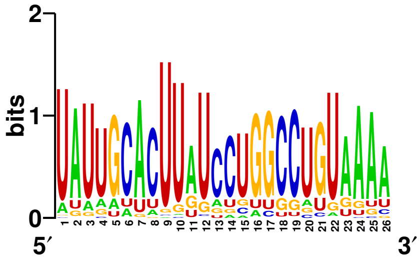

27-mers:

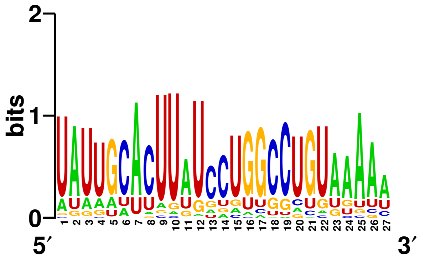

28-mers:

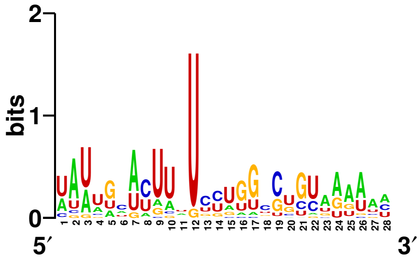

29-mers:

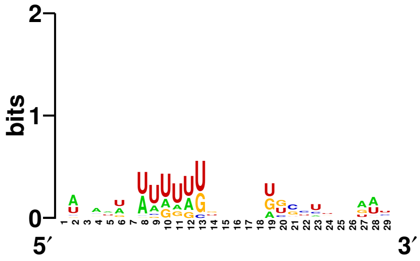

30-mers:

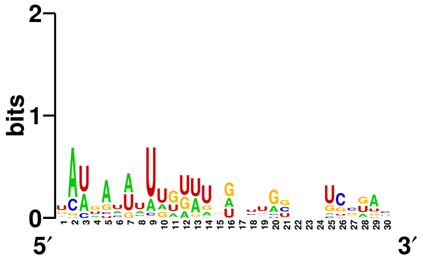

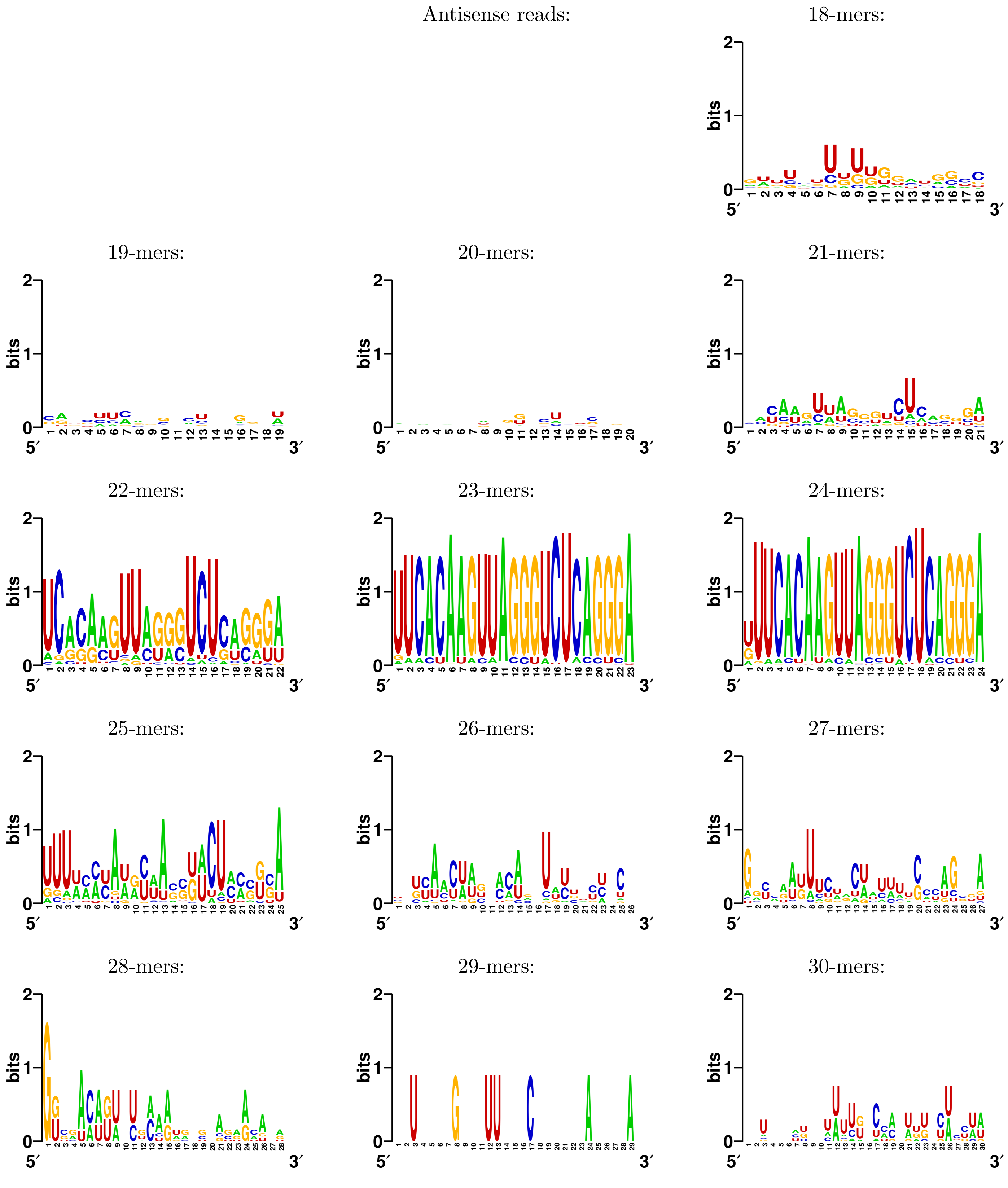
Antisense reads:
18-mers:
19-mers:
20-mers:
21-mers:
22-mers:
23-mers:
24-mers:
25-mers:
26-mers:
27-mers:
28-mers:
29-mers:
30-mers:
bits
5′
3′

Embryo 15h, library 1:

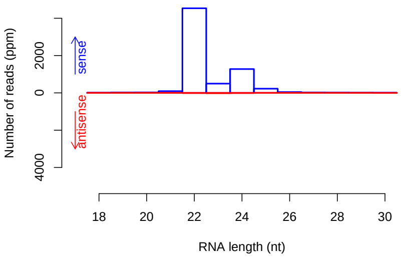

Sense reads:

18-mers:

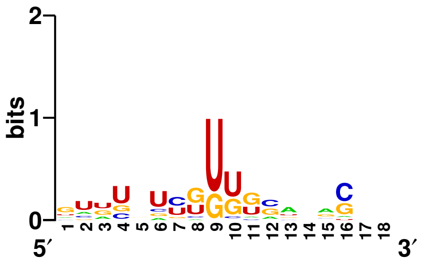

19-mers:

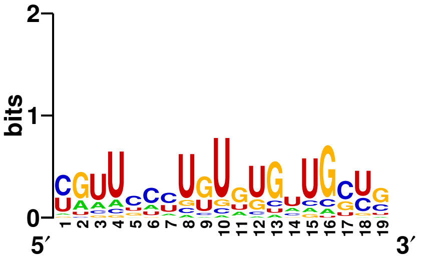

20-mers:

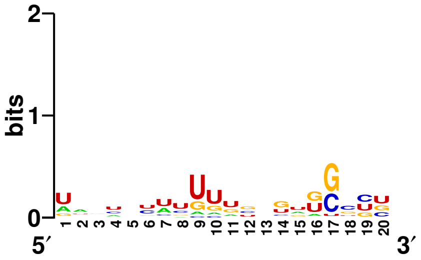

21-mers:

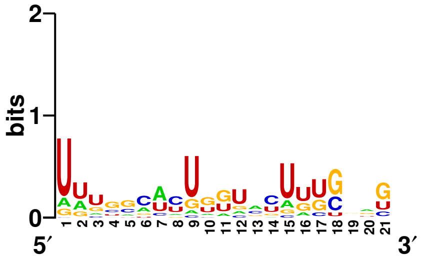

22-mers:

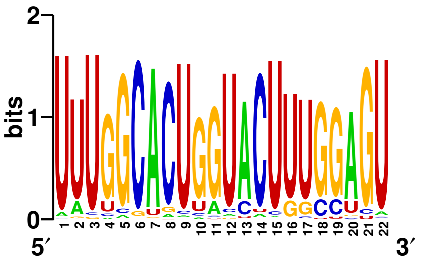

23-mers:

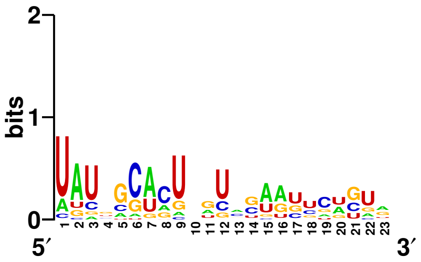

24-mers:

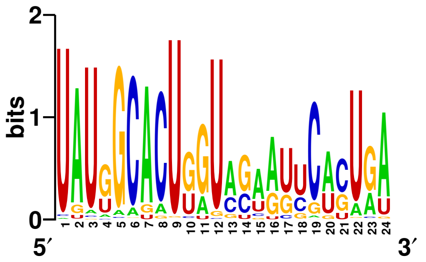

25-mers:

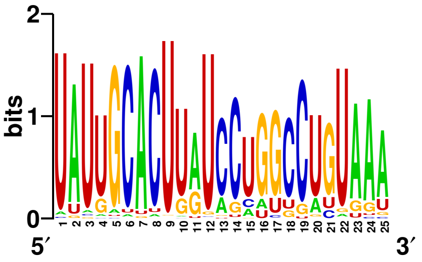

26-mers:

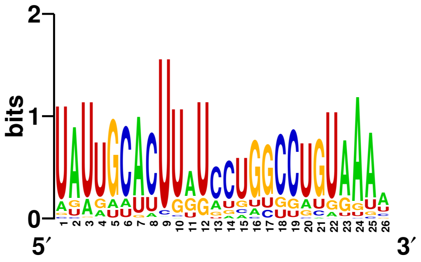

27-mers:

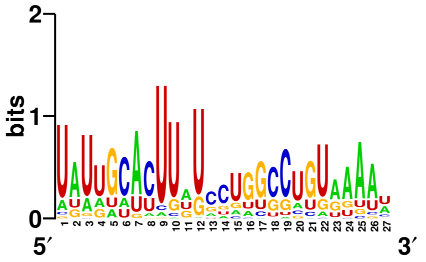

28-mers:

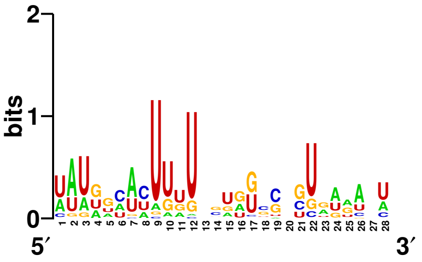

29-mers:

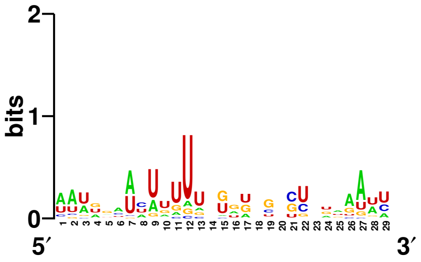

30-mers:

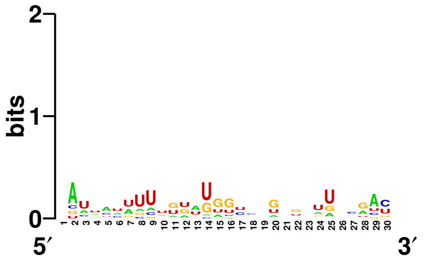

Antisense reads:

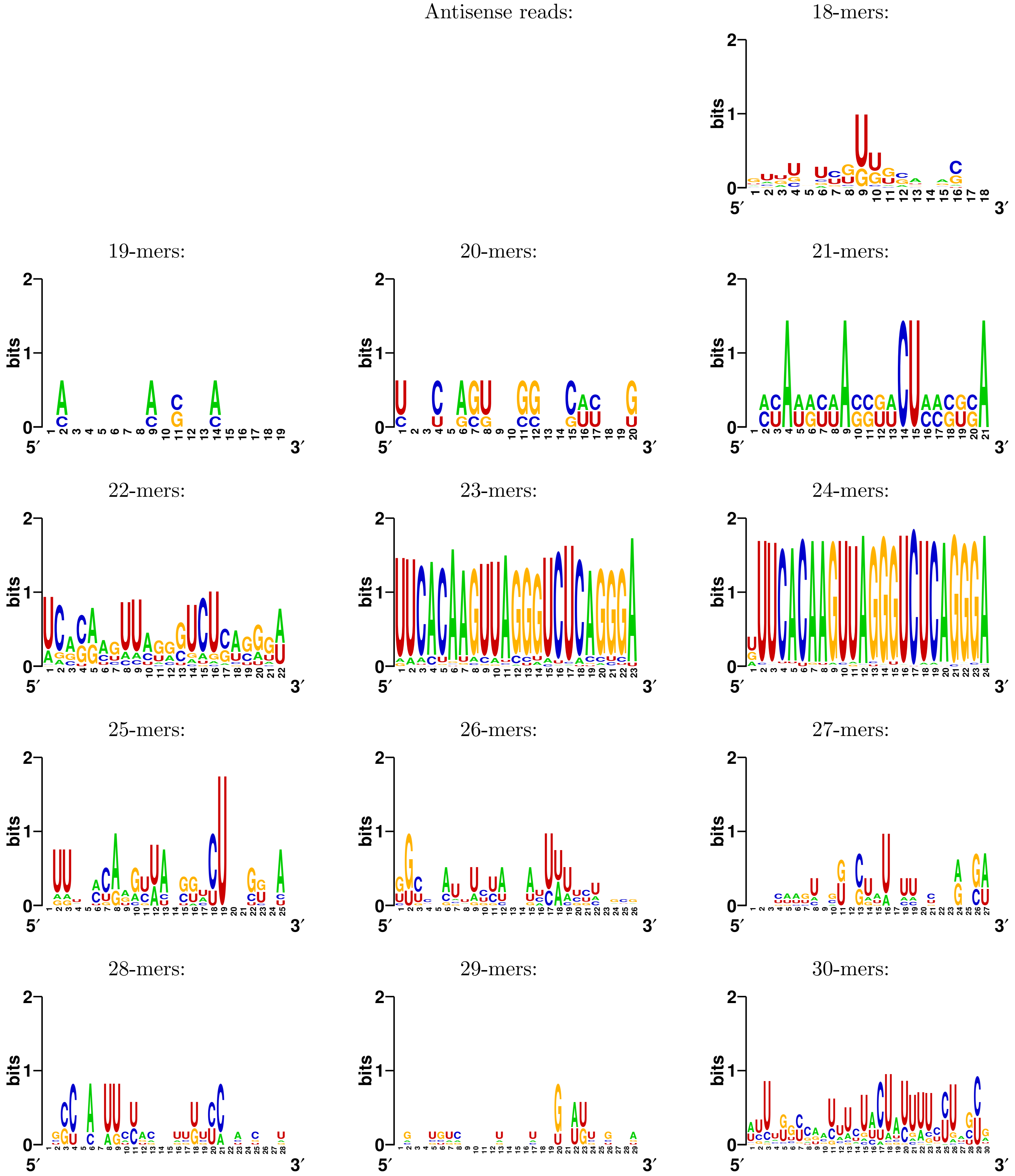

Embryo 36h, library 1:

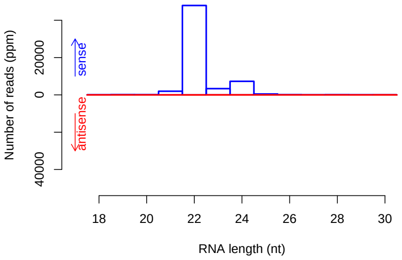

Sense reads:

18-mers:

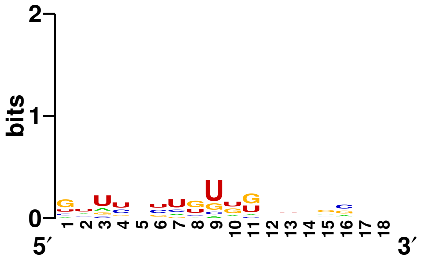

19-mers:

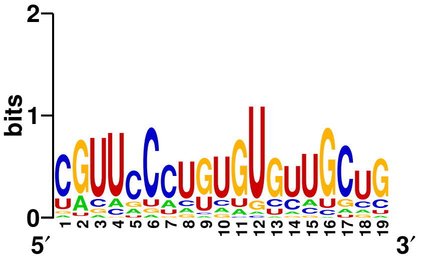

20-mers:

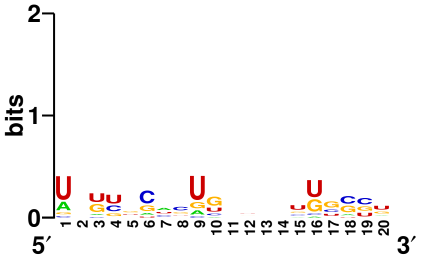

21-mers:

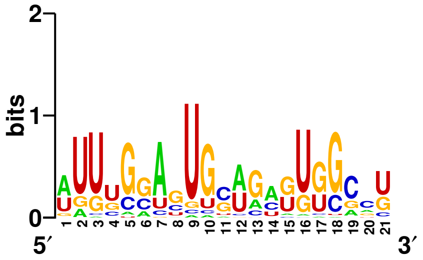

22-mers:

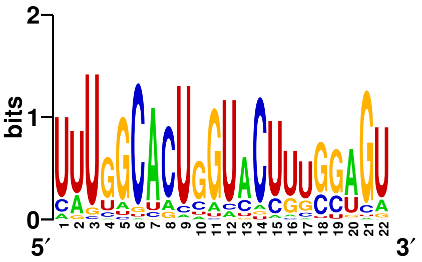

23-mers:

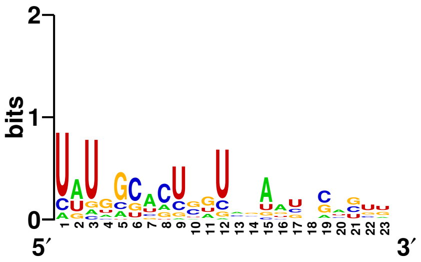

24-mers:

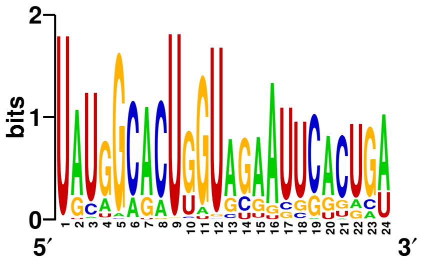

25-mers:

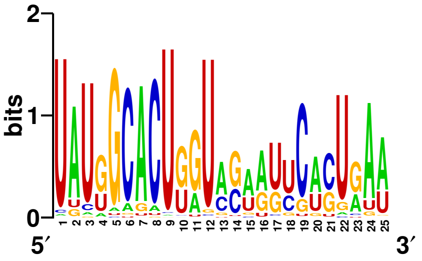

26-mers:

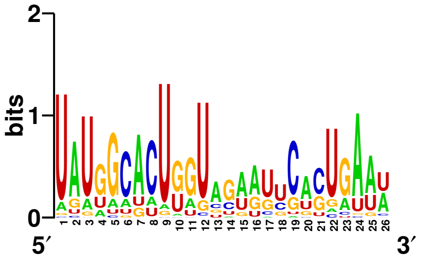

27-mers:

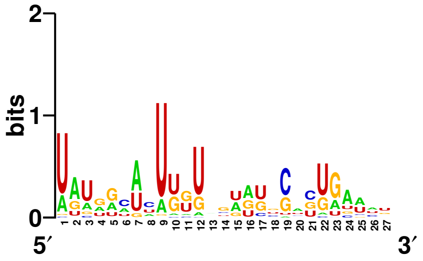

28-mers:

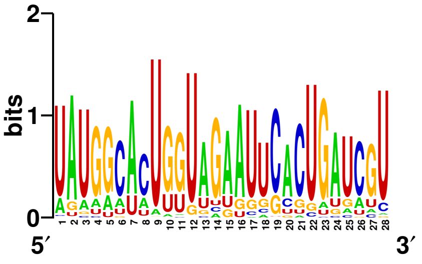

29-mers:

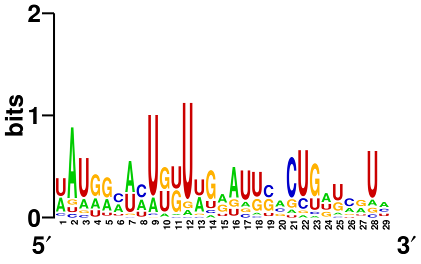

30-mers:

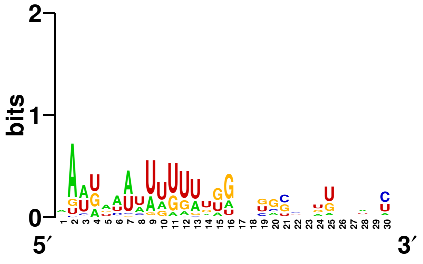

Antisense reads:

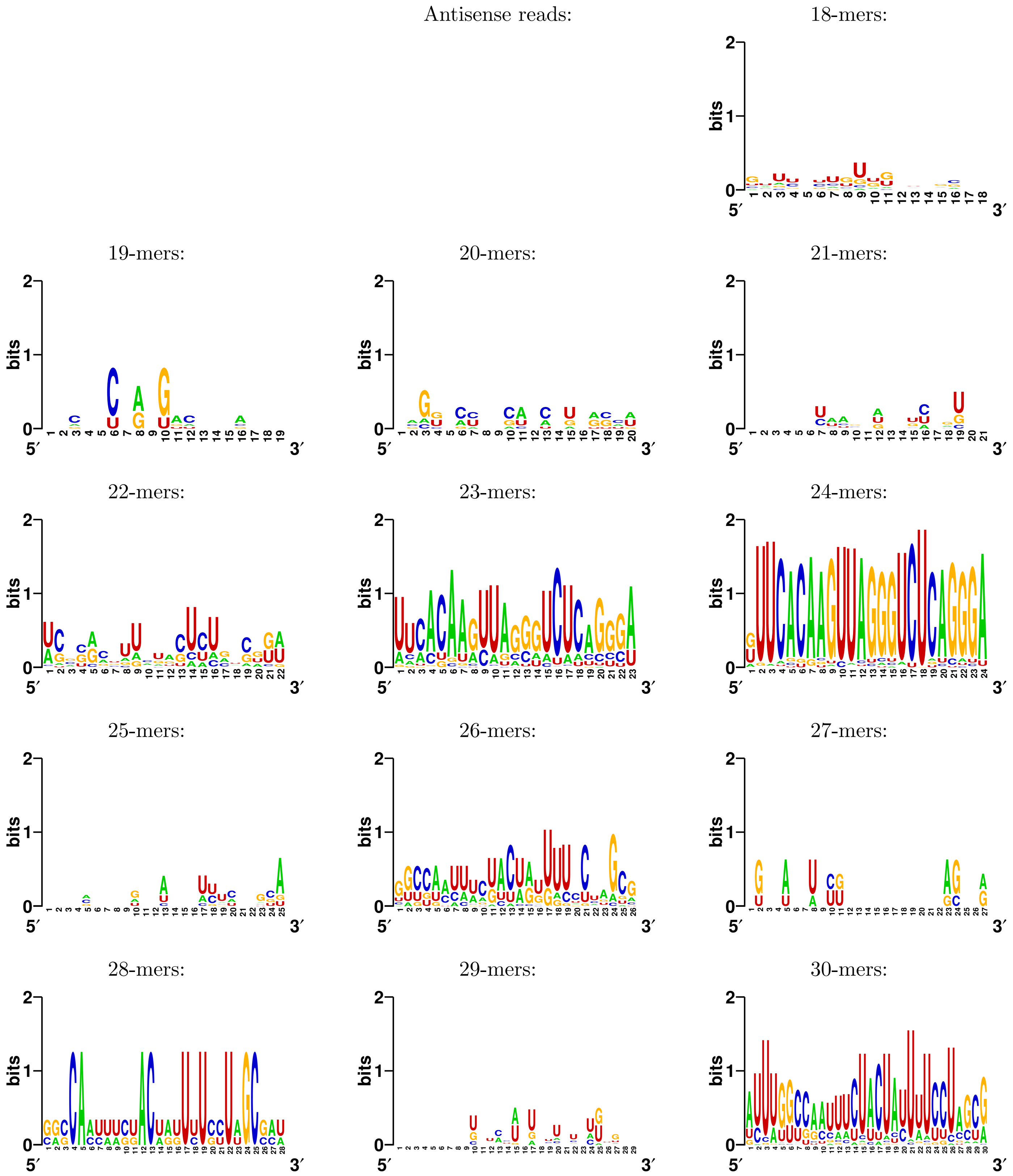

Embryo 60h, library 1:

Sense reads:

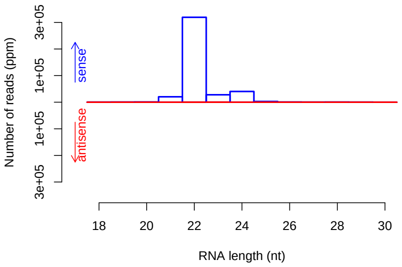

18-mers:

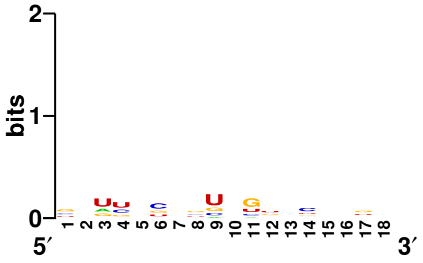

19-mers:

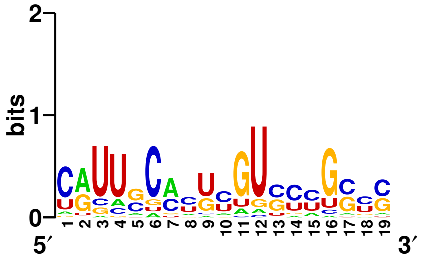

20-mers:

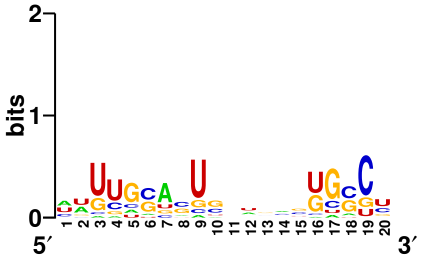

21-mers:

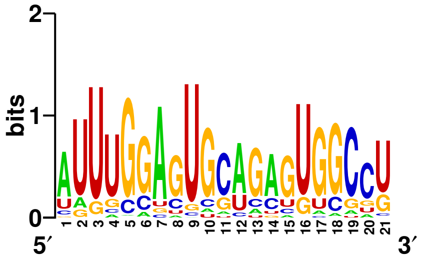

22-mers:

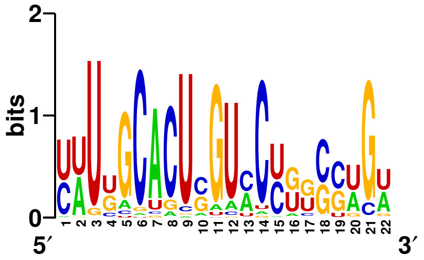

23-mers:

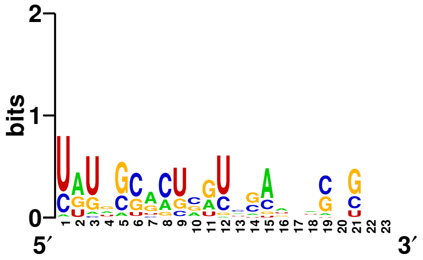

24-mers:

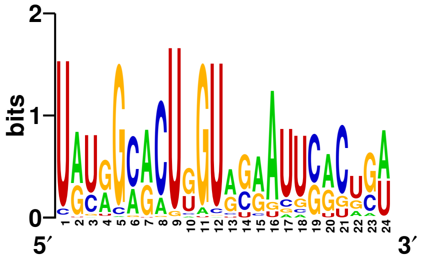

25-mers:

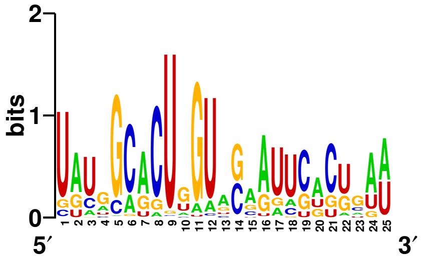

26-mers:

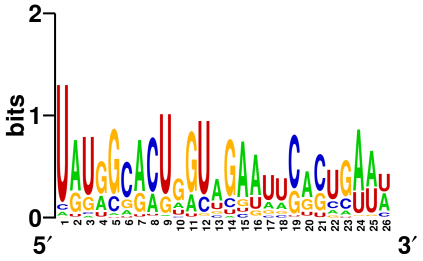

27-mers:

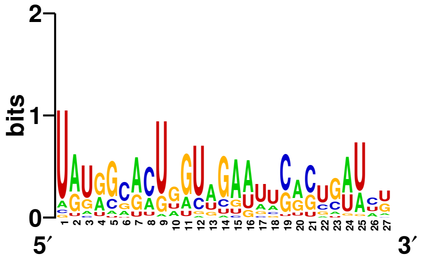

28-mers:

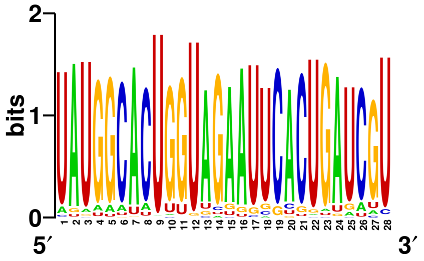

29-mers:

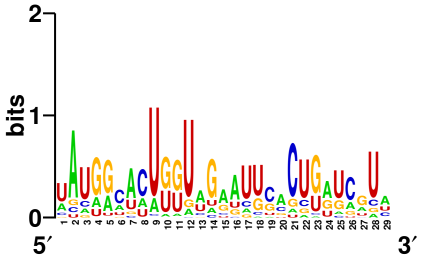

30-mers:

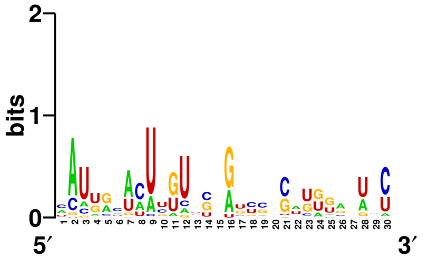

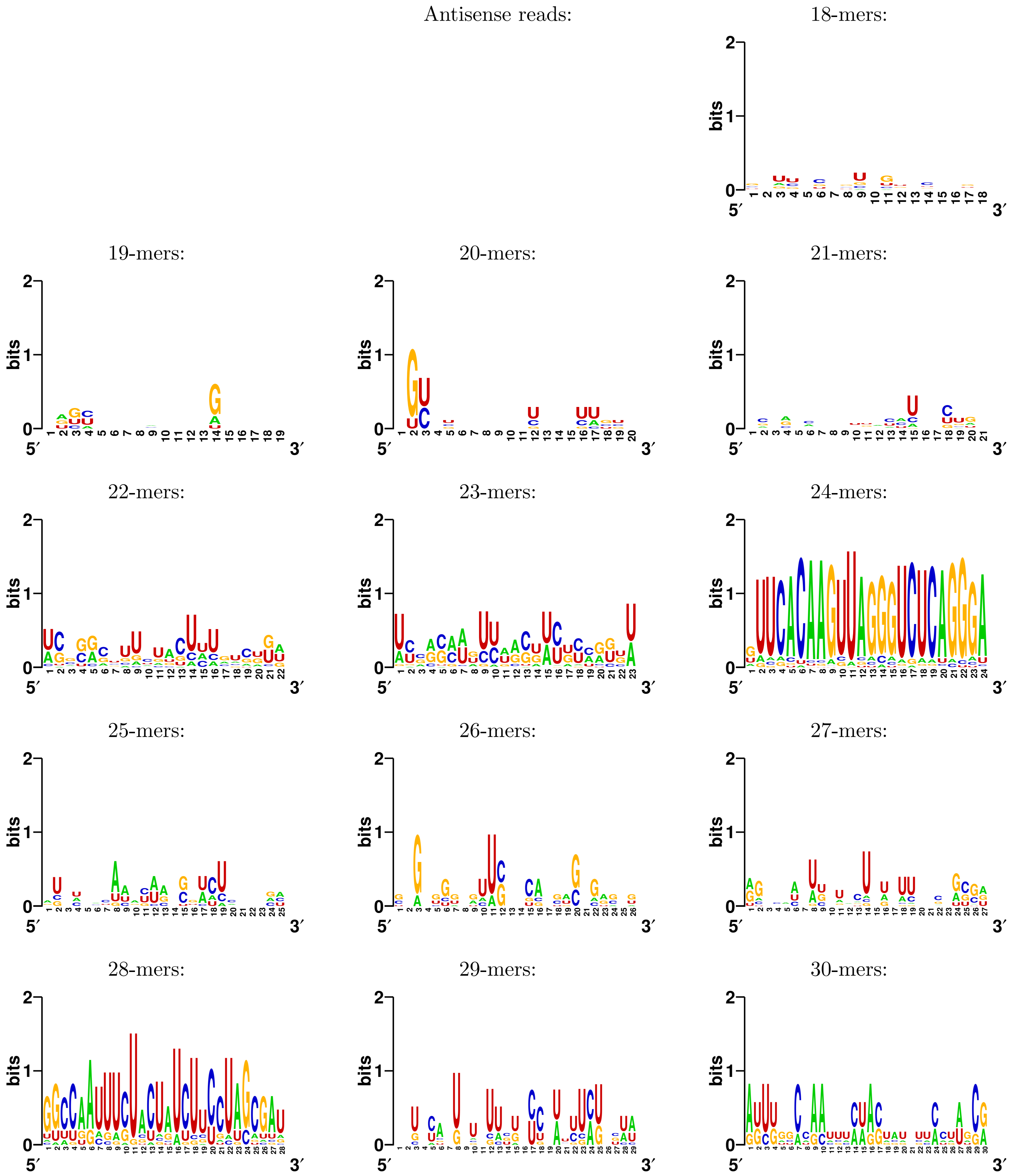
Antisense reads:
18-mers:
19-mers:
20-mers:
21-mers:
22-mers:
23-mers:
24-mers:
25-mers:
26-mers:
27-mers:
28-mers:
29-mers:
30-mers:
bits
5′
3′

Adult female, library 1:

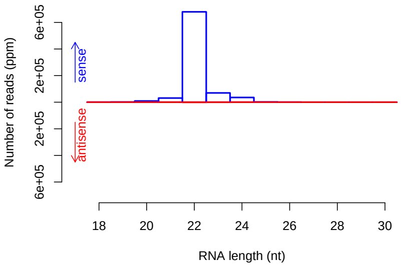

Sense reads:

18-mers:

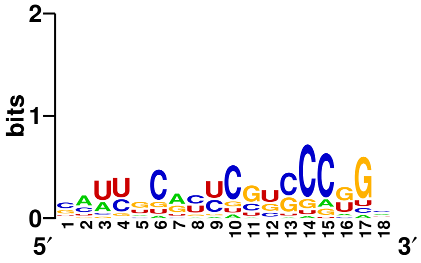

19-mers:

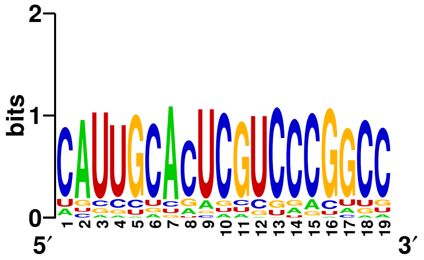

20-mers:

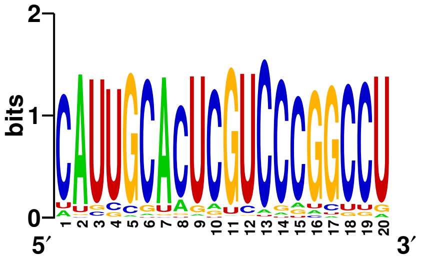

21-mers:

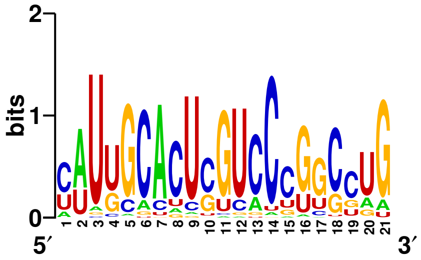

22-mers:

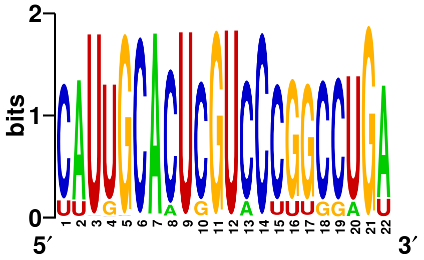

23-mers:

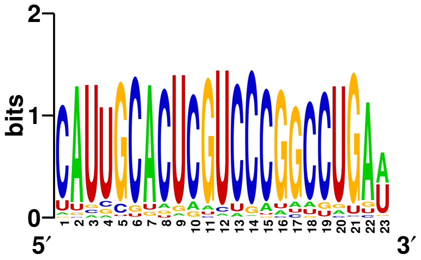

24-mers:

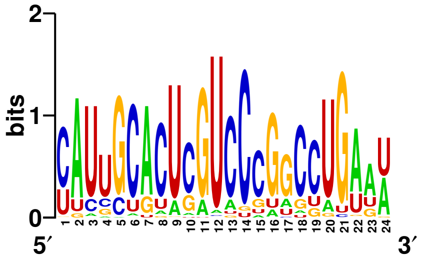

25-mers:

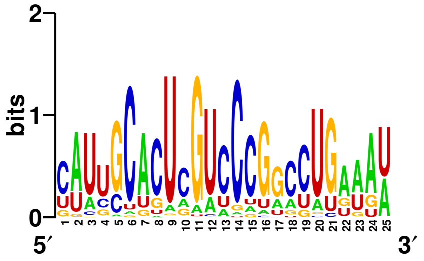

26-mers:

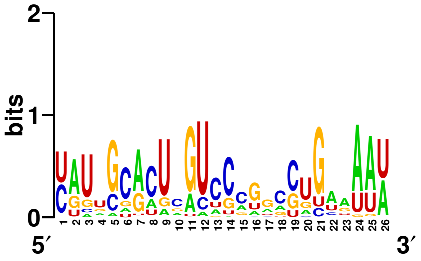

27-mers:

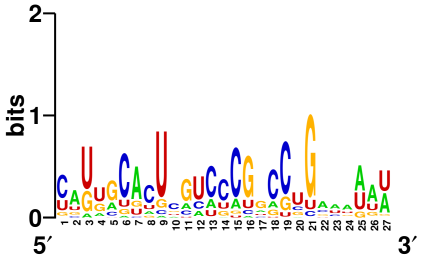

28-mers:

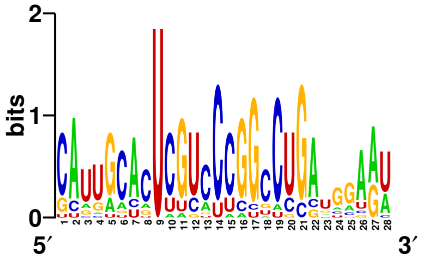

29-mers:

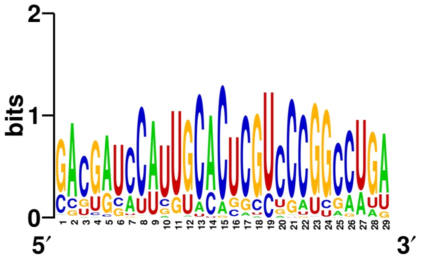

30-mers:

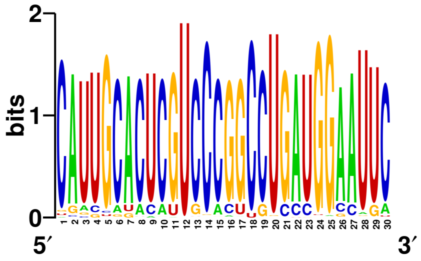

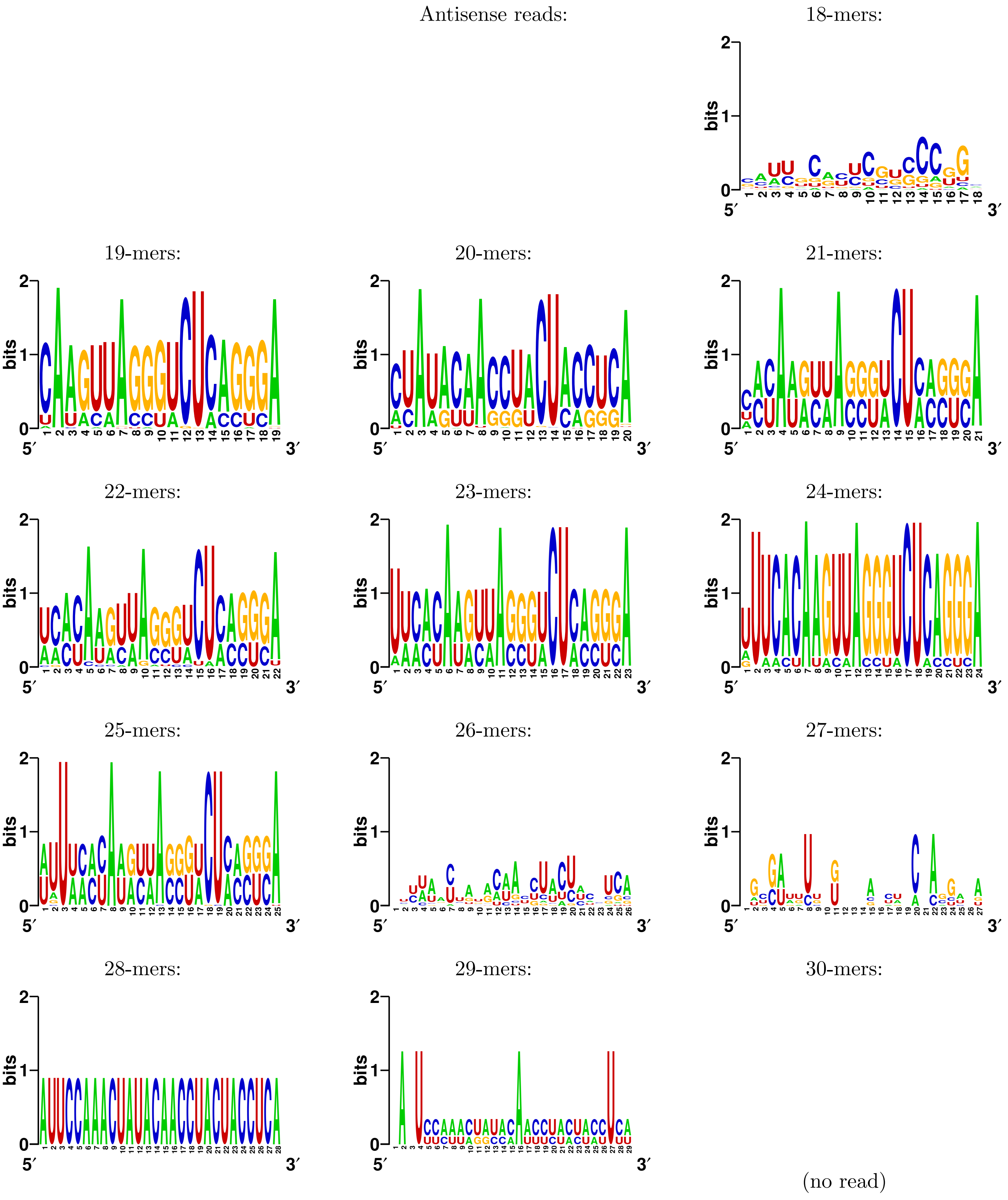
Antisense reads:
18-mers:
19-mers:
20-mers:
21-mers:
22-mers:
23-mers:
24-mers:
25-mers:
26-mers:
27-mers:
28-mers:
29-mers:
30-mers:
(no read)
bits
5′
3′

Adult male, library 1:

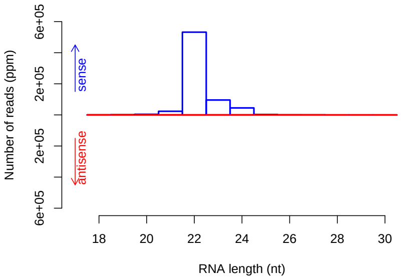

Sense reads:

18-mers:

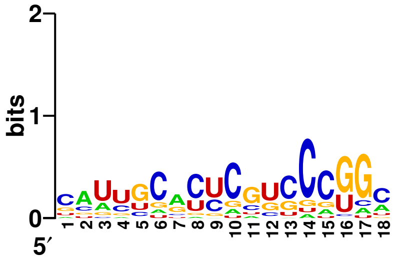

19-mers:

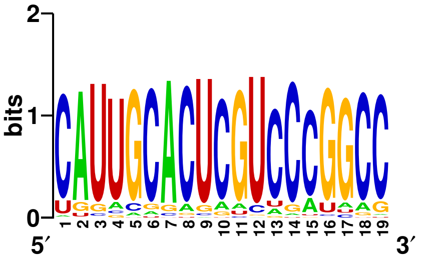

20-mers:

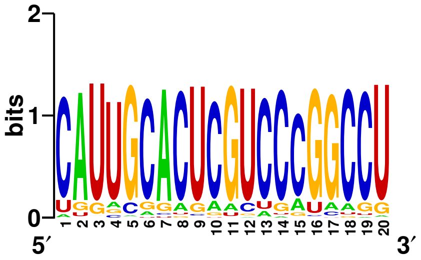

21-mers:

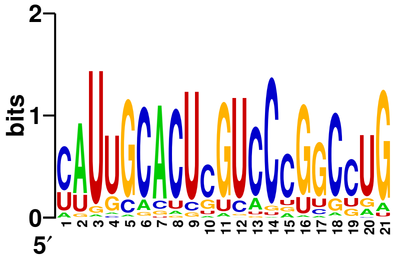

22-mers:

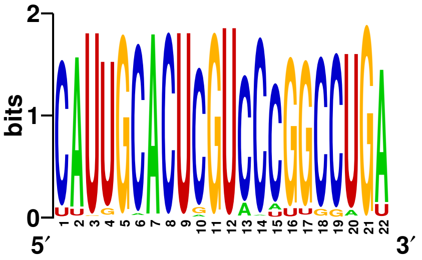

23-mers:

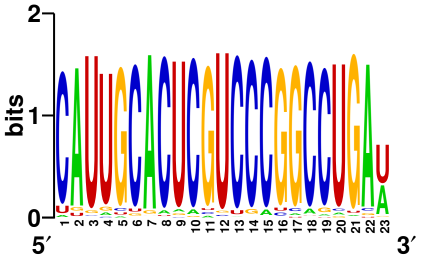

24-mers:

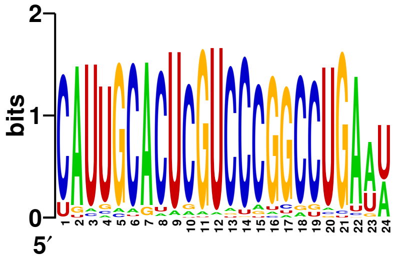

25-mers:

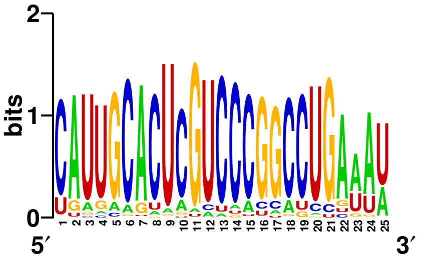

26-mers:

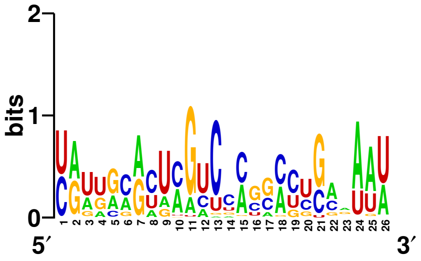

27-mers:

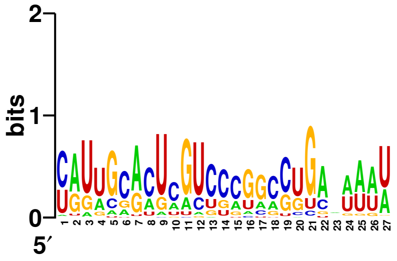

28-mers:

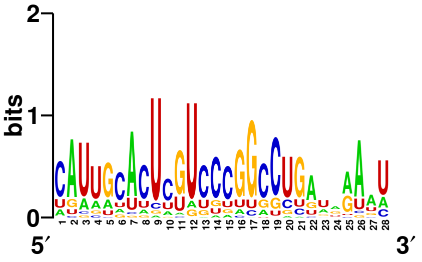

29-mers:

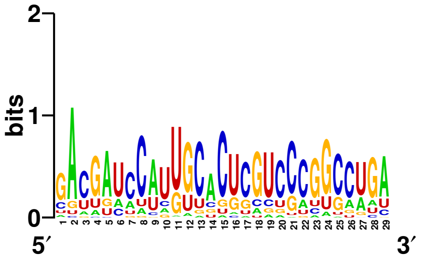

30-mers:

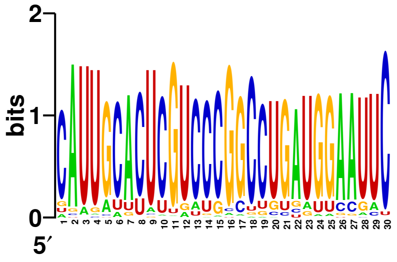

Antisense reads:

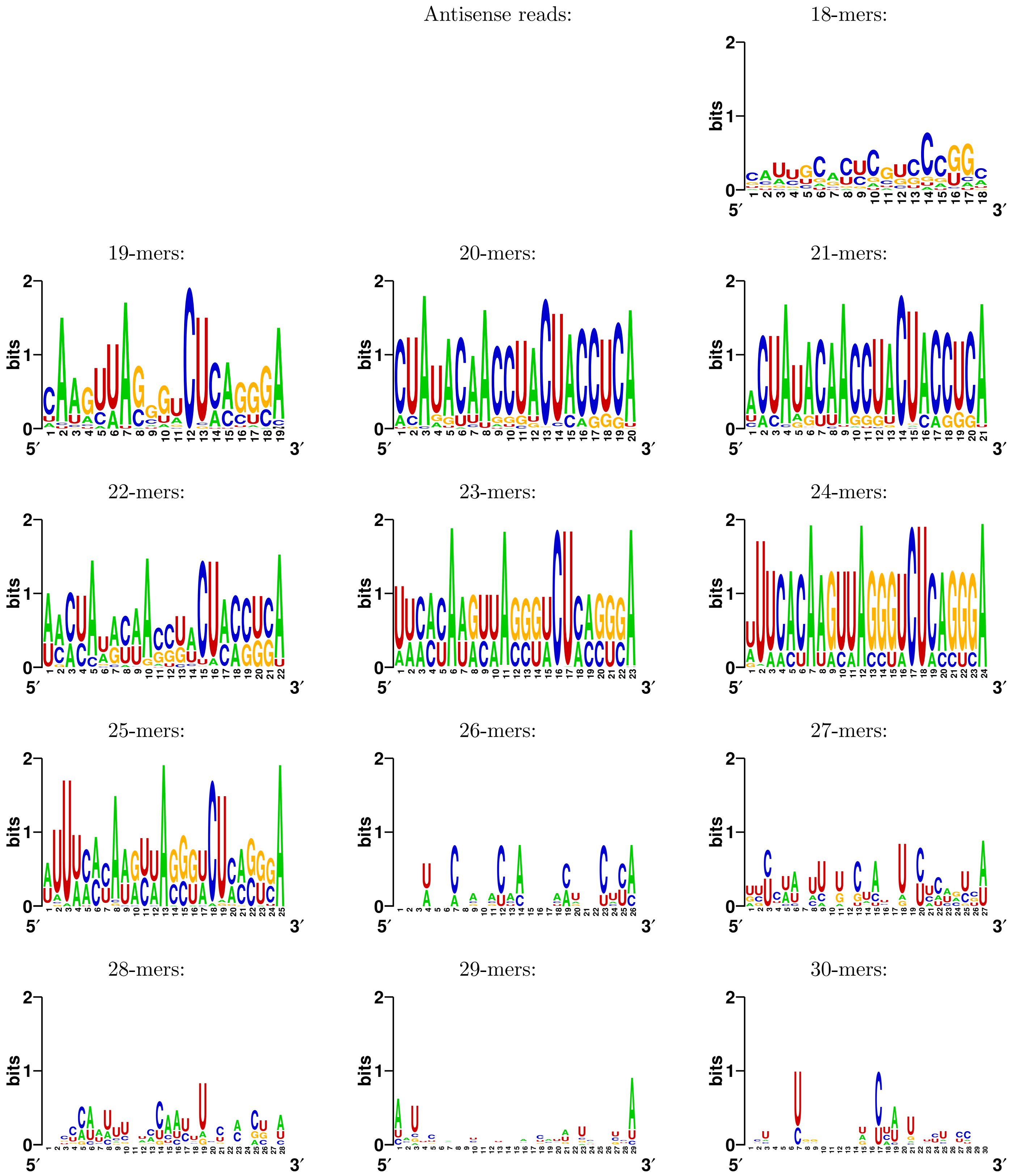

2.2 Libraries #2 (3´ modified, 5´ monophosphorylated small RNAs)

Embryo 8h, library 2:

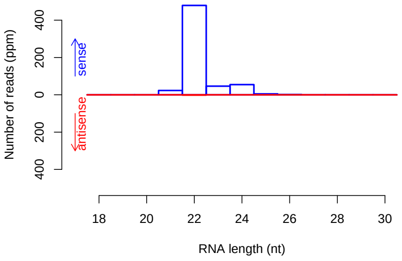

Sense reads:

18-mers:

(no read)

19-mers:

(no read)

20-mers:

(no read)

21-mers:

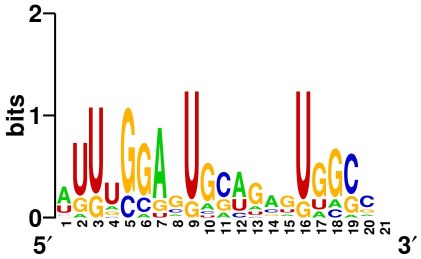

22-mers:

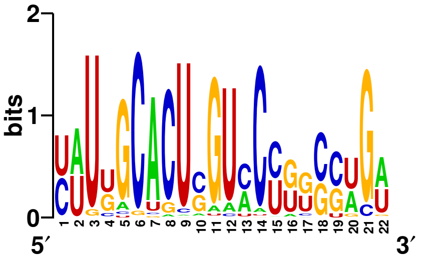

23-mers:

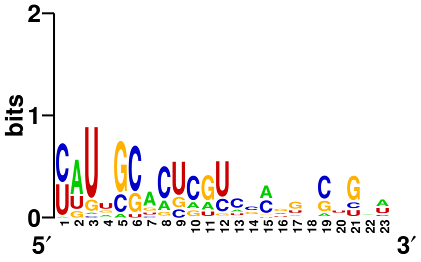

24-mers:

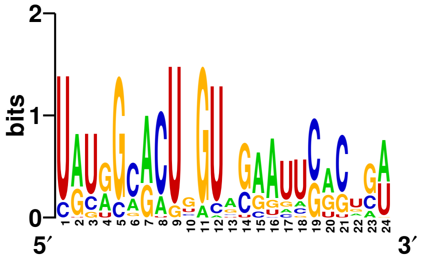

25-mers:

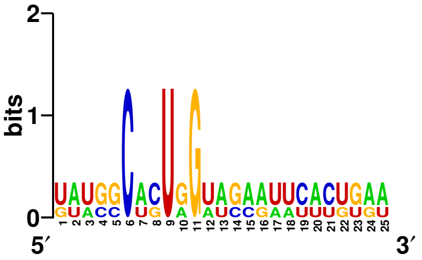

26-mers:

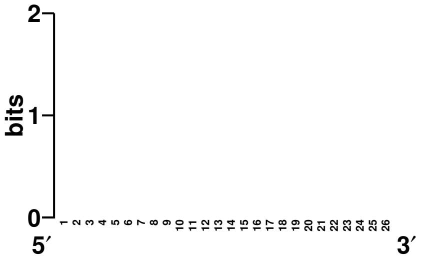

27-mers:

(no read)

28-mers:

(no read)

29-mers:

(no read)

30-mers:

(no read)

	Antisense reads:	18-mers: (no read)
19-mers: (no read)	20-mers: (no read)	21-mers: (no read)
22-mers: 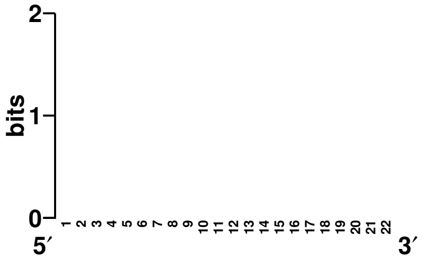	23-mers: (no read)	24-mers: (no read)
25-mers: (no read)	26-mers: (no read)	27-mers: (no read)
28-mers: (no read)	29-mers: (no read)	30-mers: (no read)

Embryo 15h, library 2:

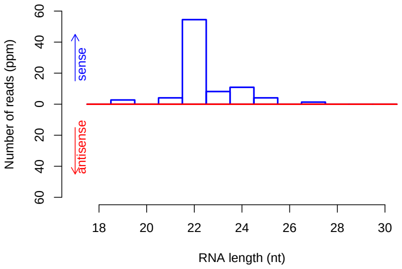

Sense reads:

18-mers:

(no read)

19-mers:

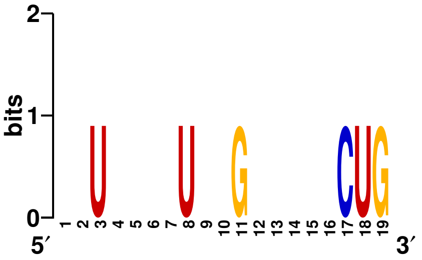

20-mers:

(no read)

21-mers:

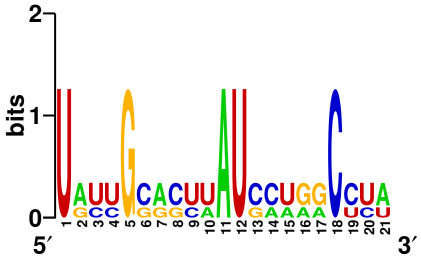

22-mers:

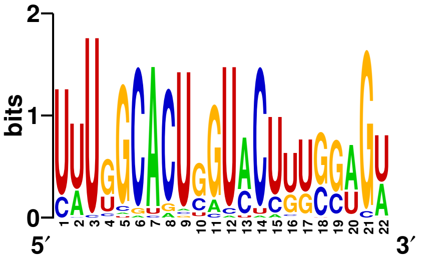

23-mers:

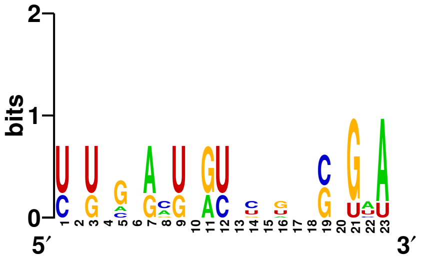

24-mers:

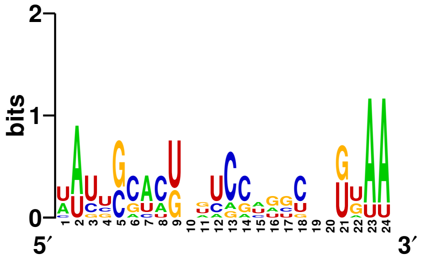

25-mers:

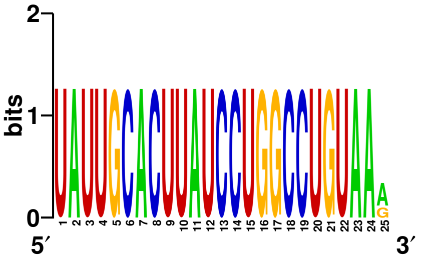

26-mers:

(no read)

27-mers:

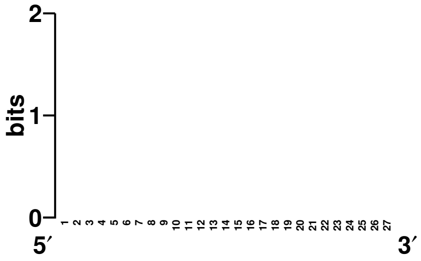

28-mers:

(no read)

29-mers:

(no read)

30-mers:

(no read)

	Antisense reads:	18-mers:<br>(no read)
19-mers:<br>(no read)	20-mers:<br>(no read)	21-mers:<br>(no read)
22-mers:<br>(no read)	23-mers:<br>(no read)	24-mers:<br>(no read)
25-mers:<br>(no read)	26-mers:<br>(no read)	27-mers:<br>(no read)
28-mers:<br>(no read)	29-mers:<br>(no read)	30-mers:<br>(no read)

Embryo 36h, library 2:

Sense reads:

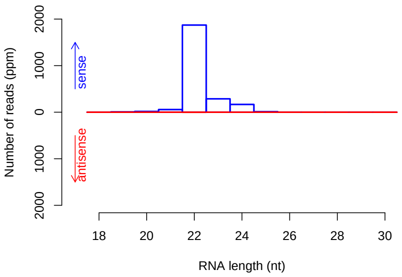

18-mers:

(no read)

19-mers:

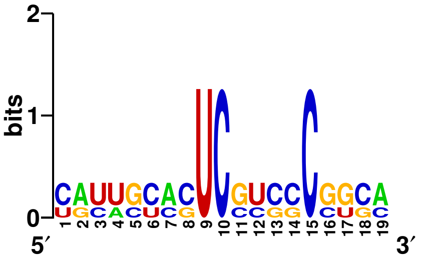

20-mers:

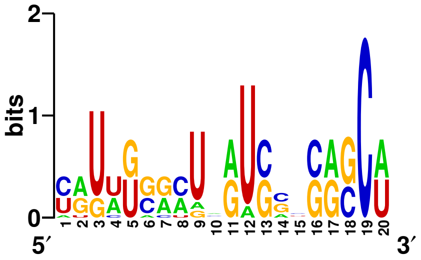

21-mers:

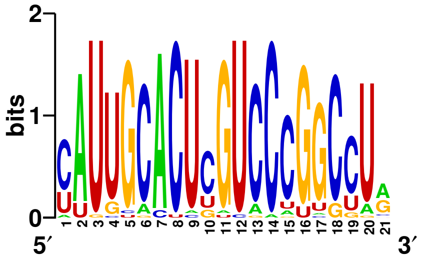

22-mers:

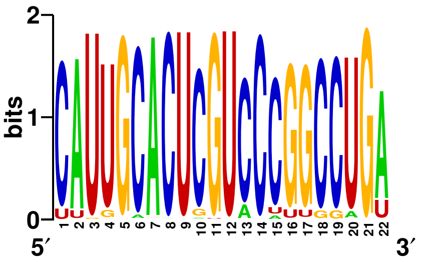

23-mers:

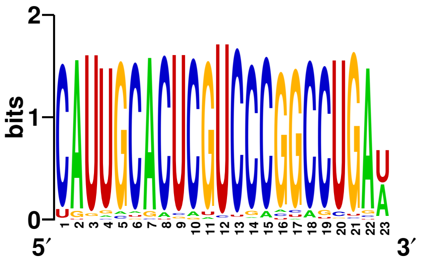

24-mers:

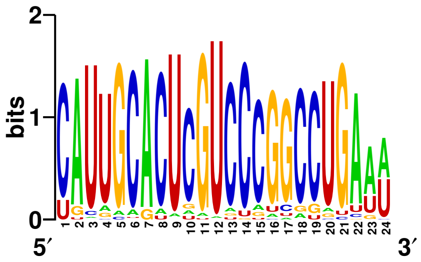

25-mers:

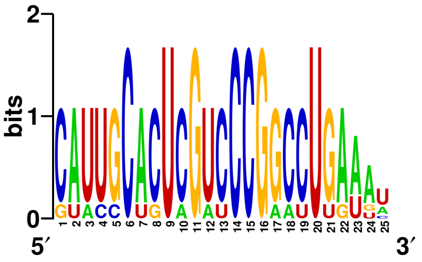

26-mers:

(no read)

27-mers:

(no read)

28-mers:

(no read)

29-mers:

(no read)

30-mers:

(no read)

Antisense reads:

18-mers:
(no read)

19-mers:
(no read)

20-mers:
(no read)

21-mers:
(no read)

22-mers:

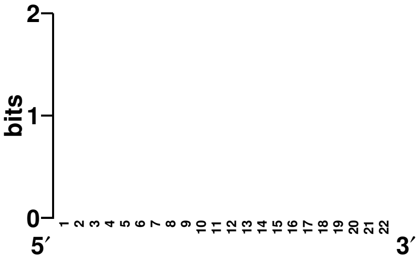

23-mers:
(no read)

24-mers:
(no read)

25-mers:
(no read)

26-mers:
(no read)

27-mers:
(no read)

28-mers:
(no read)

29-mers:
(no read)

30-mers:
(no read)

Embryo 60h, library 2:

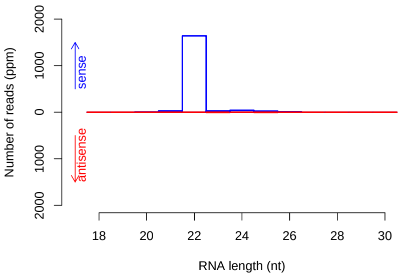

Sense reads:

18-mers:

(no read)

19-mers:

(no read)

20-mers:

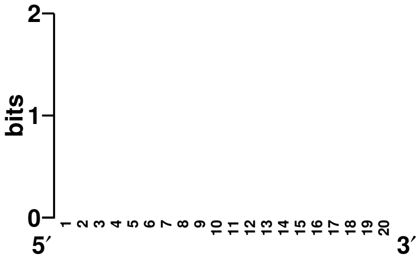

21-mers:

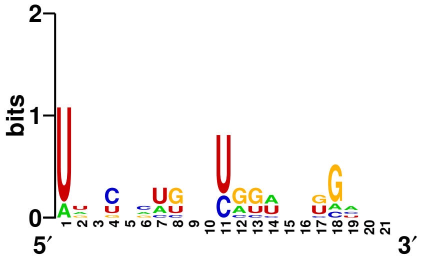

22-mers:

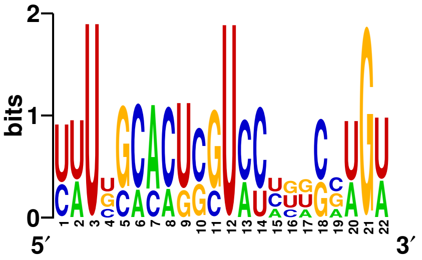

23-mers:

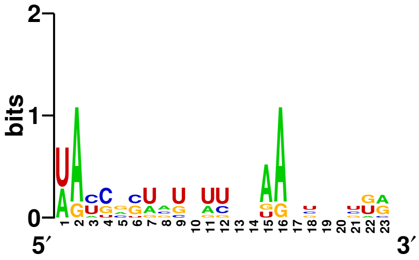

24-mers:

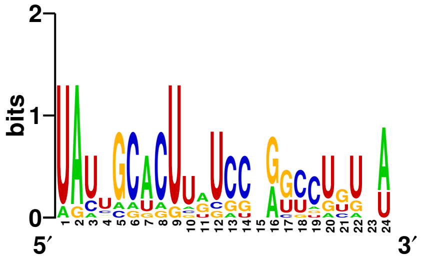

25-mers:

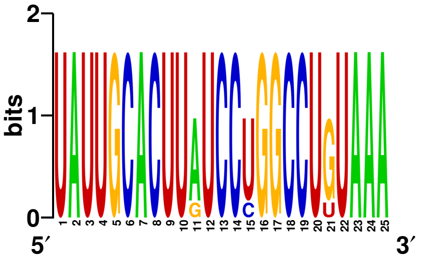

26-mers:

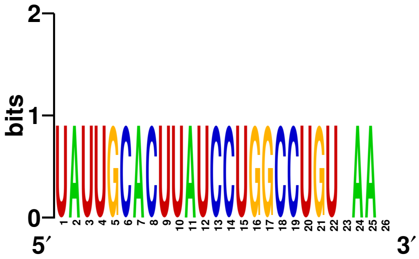

27-mers:

(no read)

28-mers:

(no read)

29-mers:

(no read)

30-mers:

(no read)

Antisense reads:

18-mers:
(no read)

19-mers:
(no read)

20-mers:
(no read)

21-mers:
(no read)

22-mers:
(no read)

23-mers:
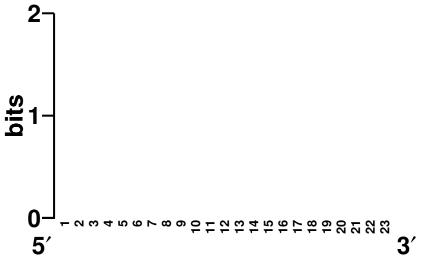

24-mers:
(no read)

25-mers:
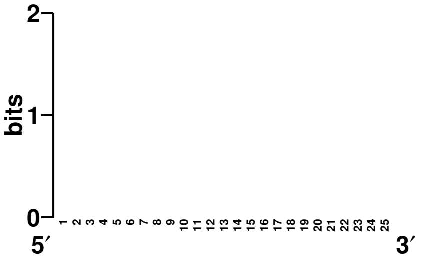

26-mers:
(no read)

27-mers:
(no read)

28-mers:
(no read)

29-mers:
(no read)

30-mers:
(no read)

Adult female, library 2:

Sense reads:

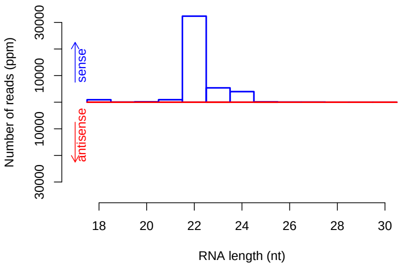

18-mers:

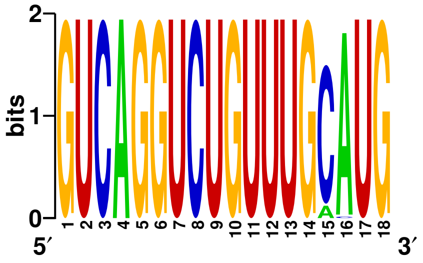

19-mers:

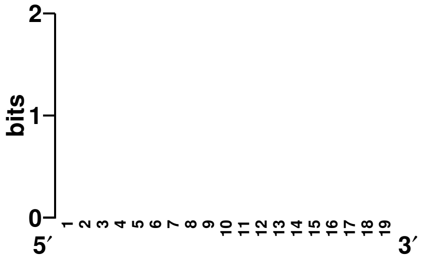

20-mers:

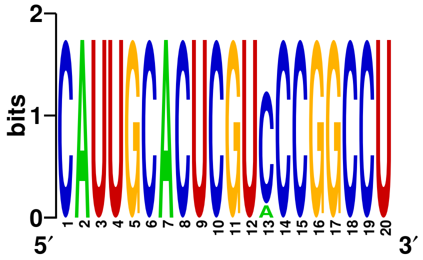

21-mers:

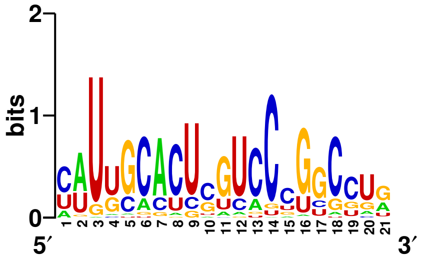

22-mers:

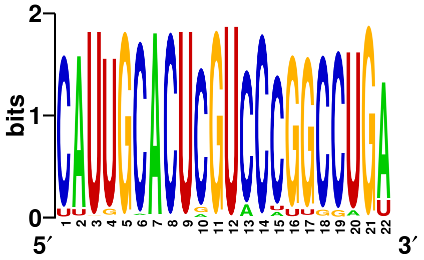

23-mers:

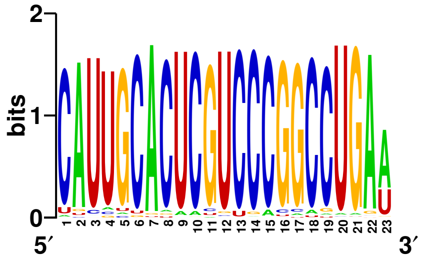

24-mers:

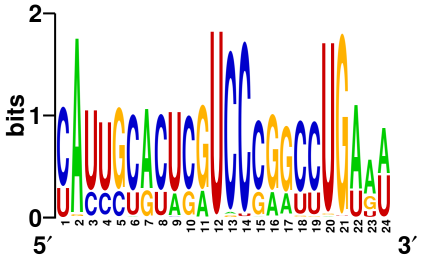

25-mers:

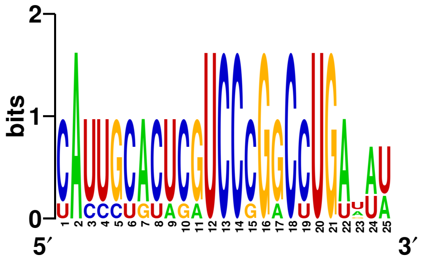

26-mers:

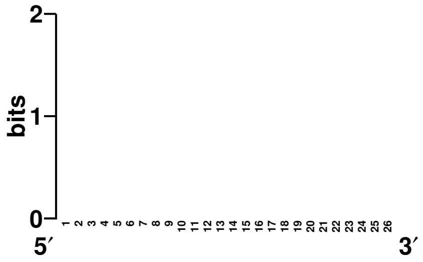

27-mers:

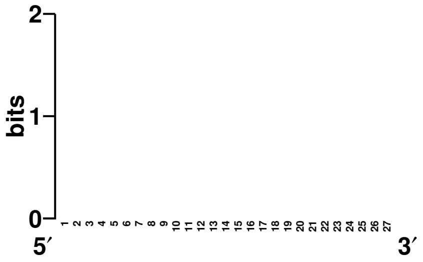

28-mers:
(no read)

29-mers:
(no read)

30-mers:
(no read)

Antisense reads:

18-mers:

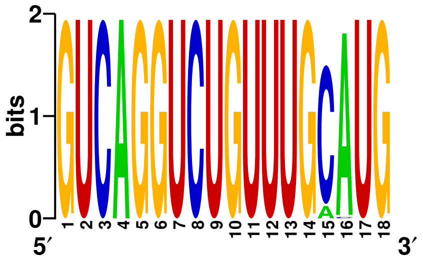

19-mers:
(no read)

20-mers:
(no read)

21-mers:
(no read)

22-mers:
(no read)

23-mers:

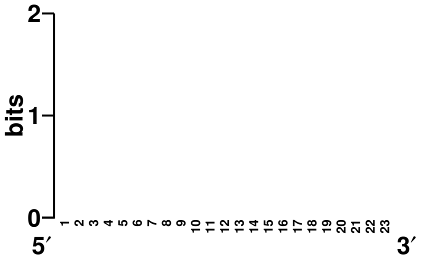

24-mers:
(no read)

25-mers:

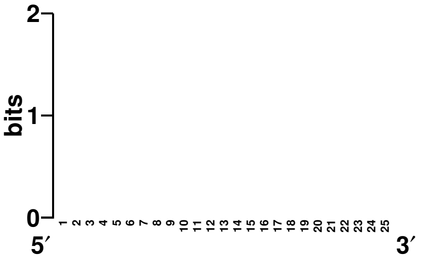

26-mers:
(no read)

27-mers:
(no read)

28-mers:
(no read)

29-mers:
(no read)

30-mers:
(no read)

Adult male, library 2:

Sense reads:

18-mers:

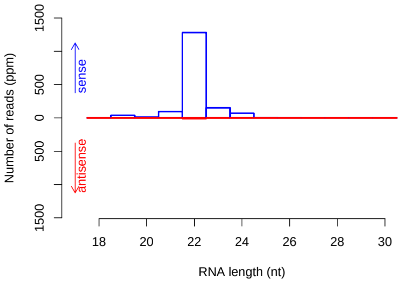

(no read)

19-mers:

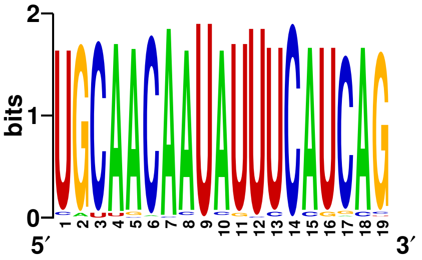

20-mers:

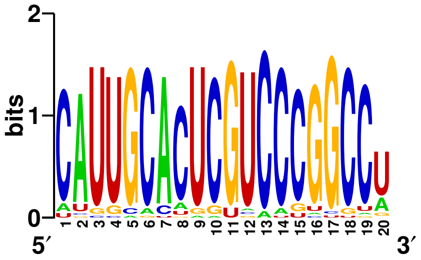

21-mers:

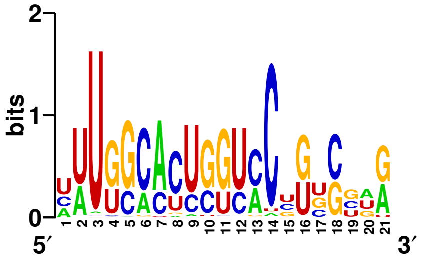

22-mers:

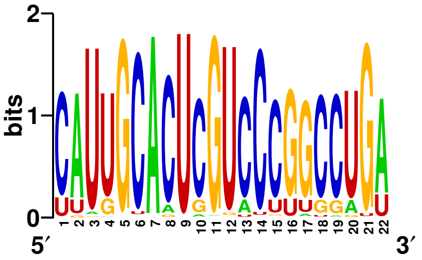

23-mers:

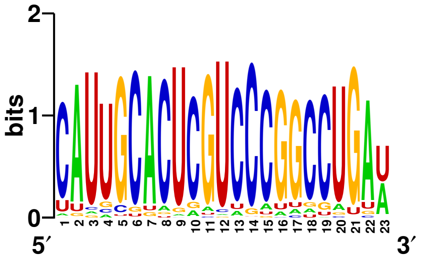

24-mers:

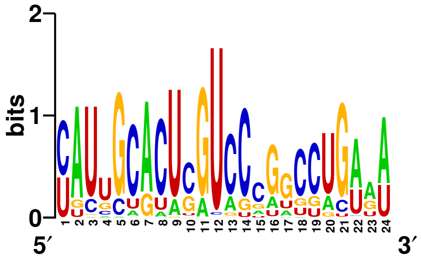

25-mers:

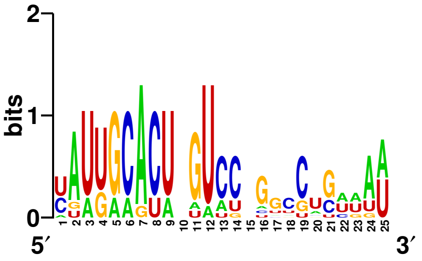

26-mers:

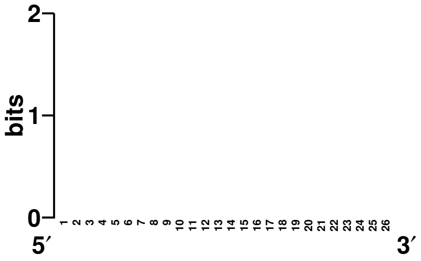

27-mers:

(no read)

28-mers:
(no read)

29-mers:
(no read)

30-mers:
(no read)

Antisense reads:

18-mers:
(no read)

19-mers:
(no read)

20-mers:

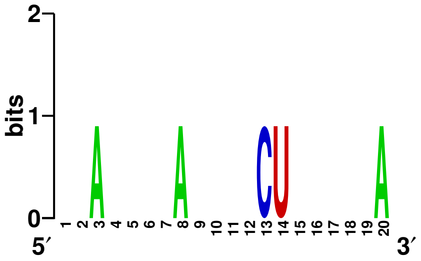

21-mers:

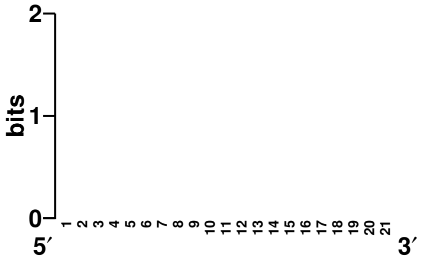

22-mers:

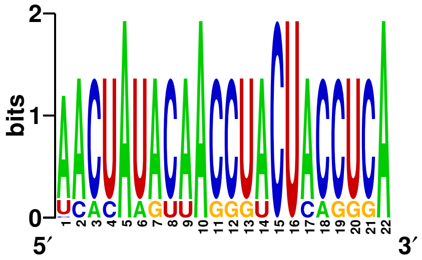

23-mers:

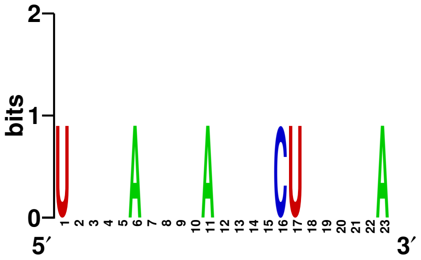

24-mers:

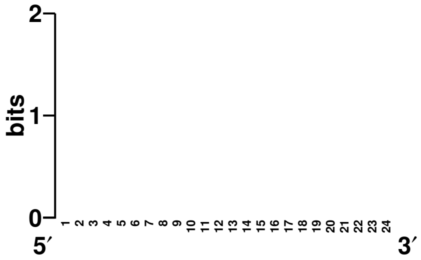

25-mers:

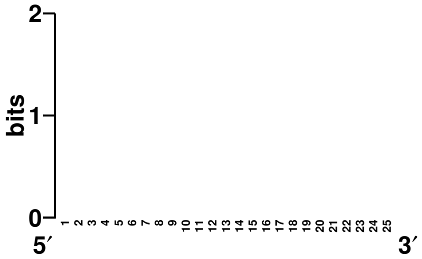

26-mers:
(no read)

27-mers:
(no read)

28-mers:
(no read)

29-mers:
(no read)

30-mers:
(no read)

2.3 Libraries #3 (total 5´ hydroxyl or polyphosphorylated small RNAs)

Embryo 8h, library 3:

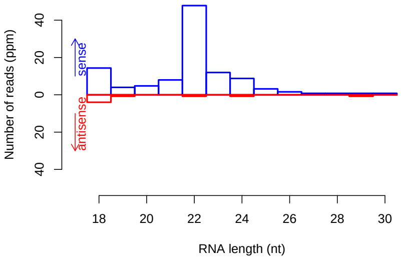

Sense reads:

18-mers:

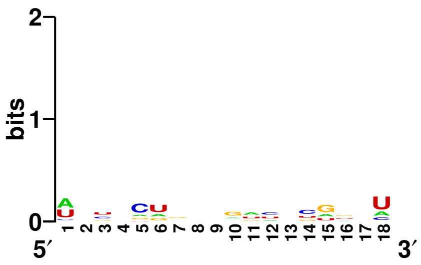

19-mers:

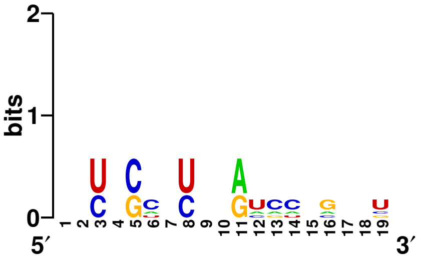

20-mers:

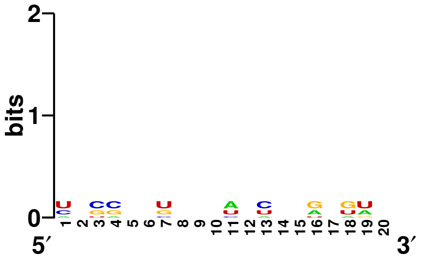

21-mers:

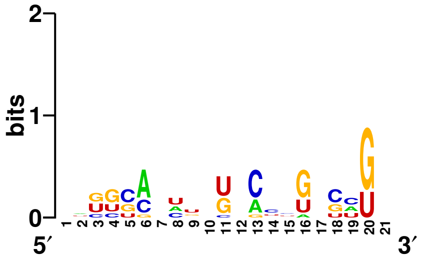

22-mers:

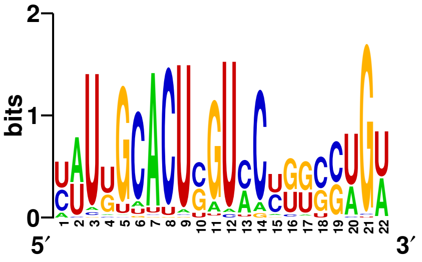

23-mers:

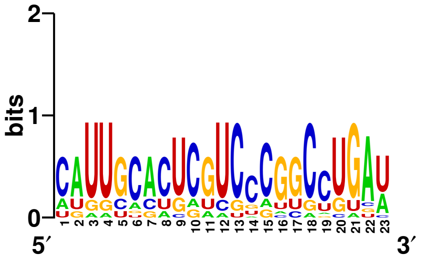

24-mers:

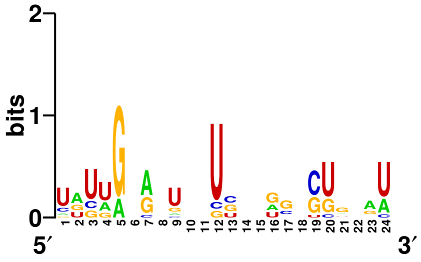

25-mers:

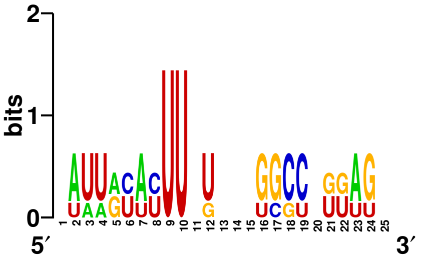

26-mers:

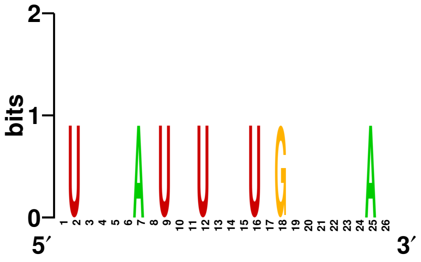

27-mers:

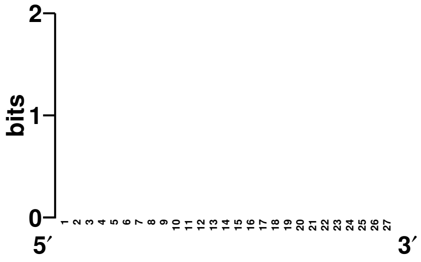

28-mers:

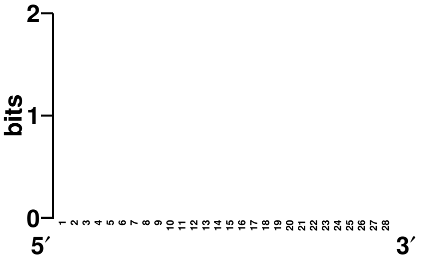

29-mers:

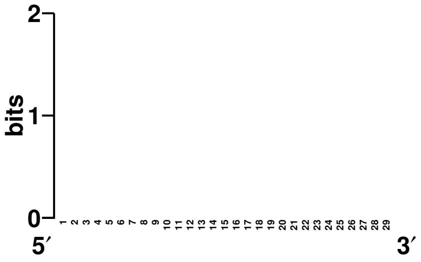

30-mers:

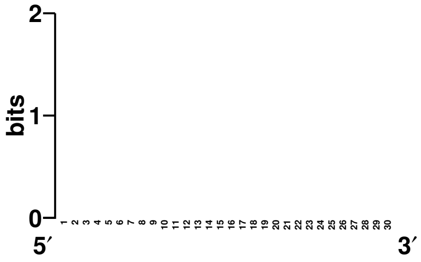

Antisense reads:

18-mers:

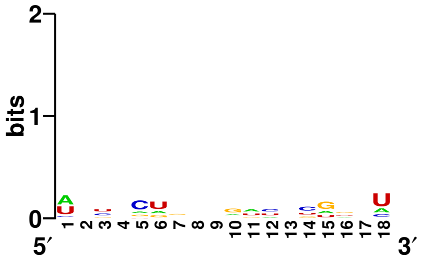

19-mers:

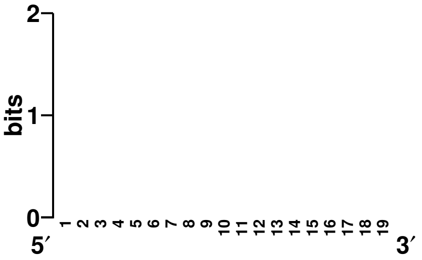

20-mers:

(no read)

21-mers:

(no read)

22-mers:

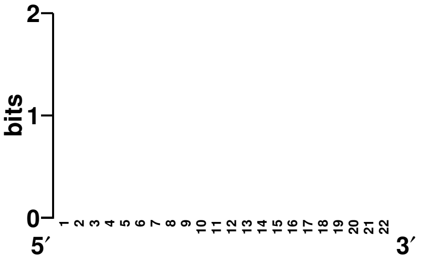

23-mers:

(no read)

24-mers:

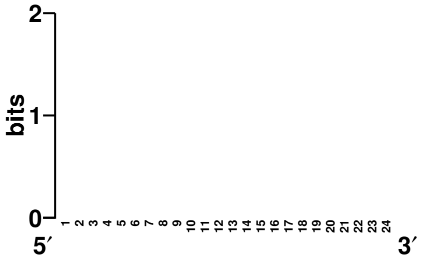

25-mers:

(no read)

26-mers:

(no read)

27-mers:

(no read)

28-mers:

(no read)

29-mers:

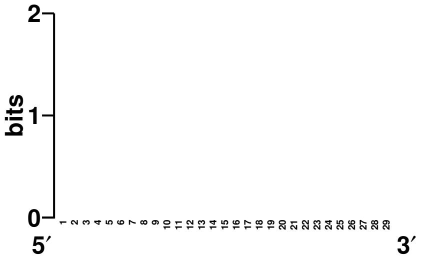

30-mers:

(no read)

Embryo 15h, library 3:

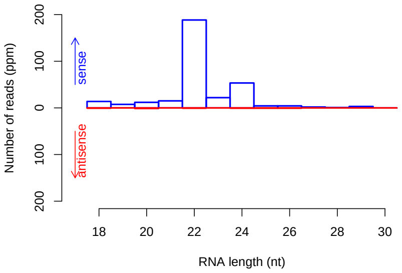

Sense reads:

18-mers:

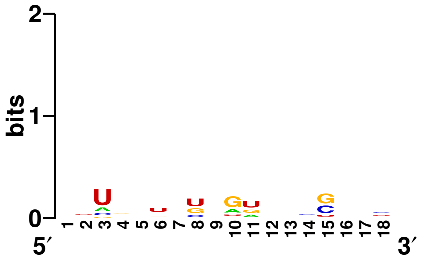

19-mers:

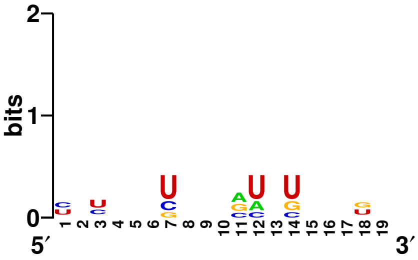

20-mers:

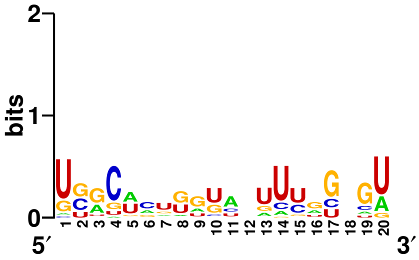

21-mers:

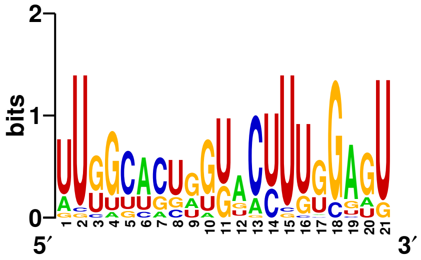

22-mers:

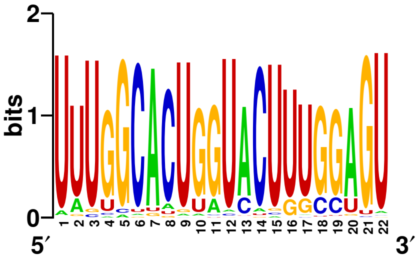

23-mers:

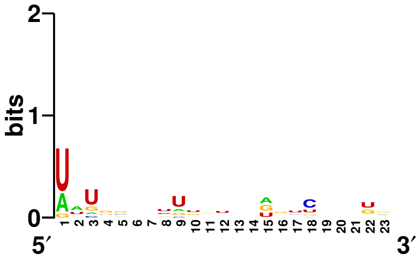

24-mers:

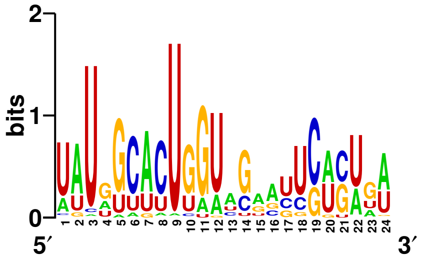

25-mers:

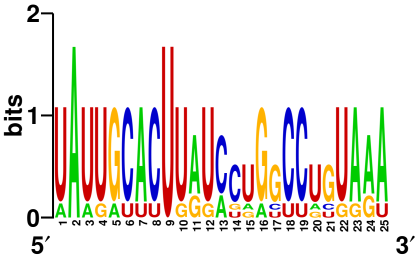

26-mers:

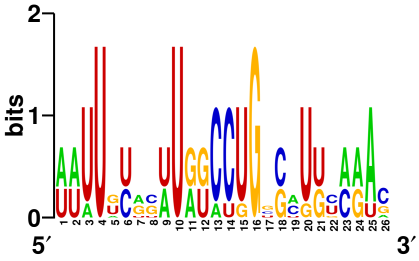

27-mers:

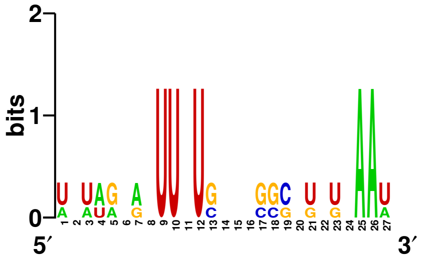

28-mers:

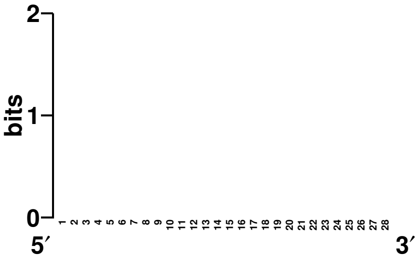

29-mers:

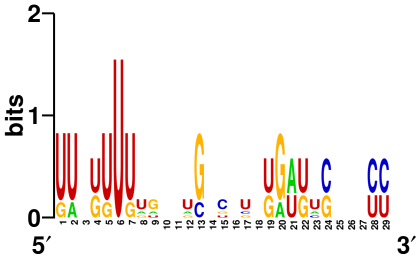

30-mers:

(no read)

Antisense reads:

18-mers:

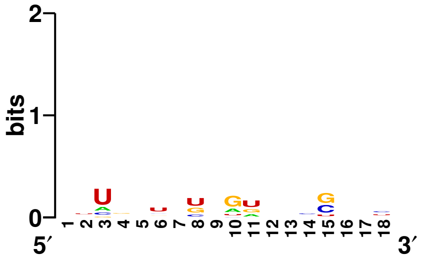

19-mers:

(no read)

20-mers:

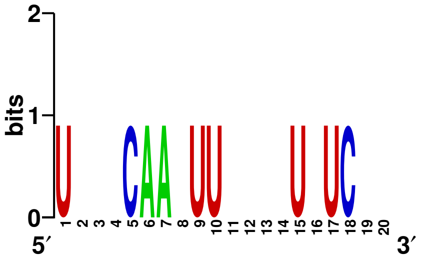

21-mers:

(no read)

22-mers:

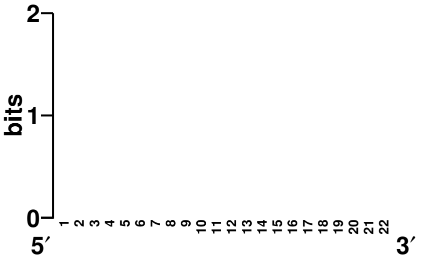

23-mers:

(no read)

24-mers:

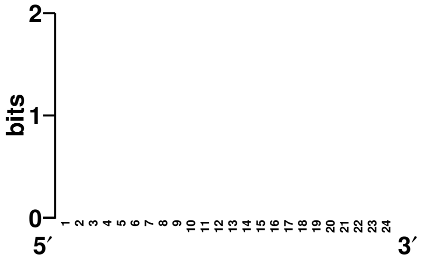

25-mers:

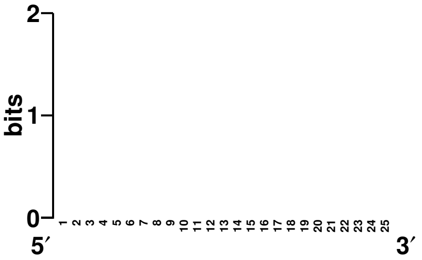

26-mers:

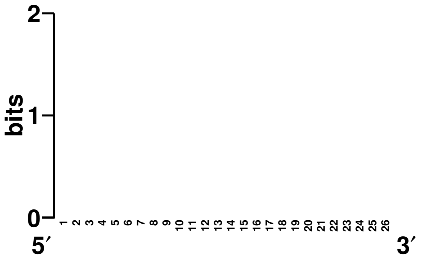

27-mers:

(no read)

28-mers:

(no read)

29-mers:

(no read)

30-mers:

(no read)

Embryo 36h, library 3:

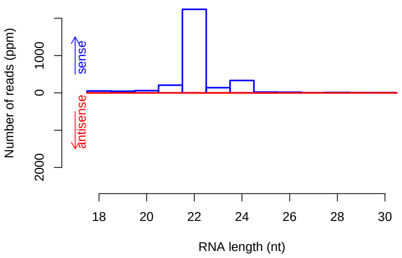

Sense reads:

18-mers:

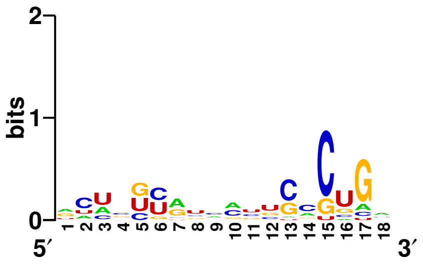

19-mers:

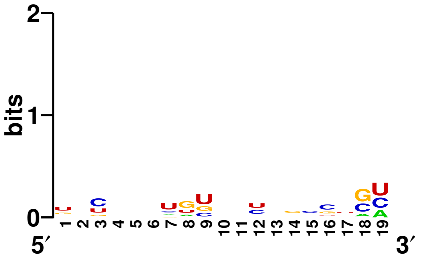

20-mers:

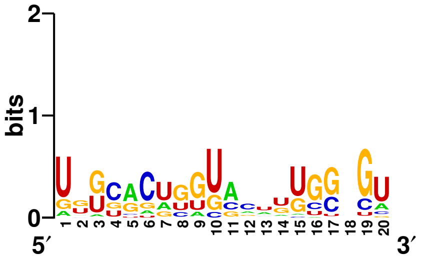

21-mers:

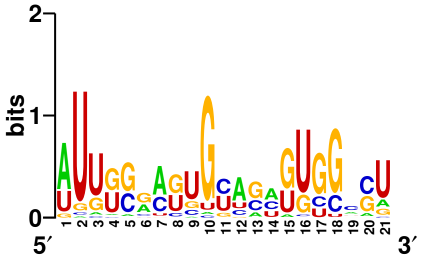

22-mers:

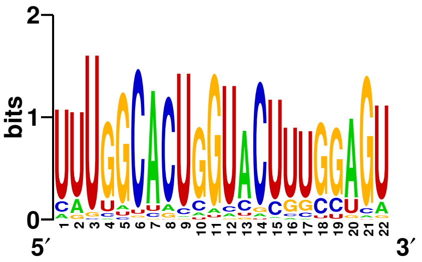

23-mers:

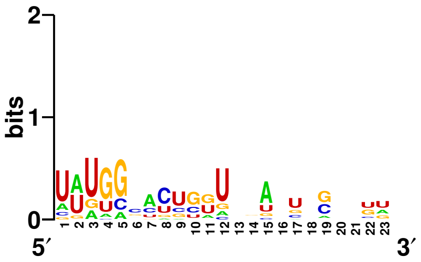

24-mers:

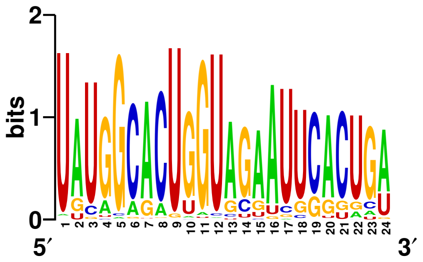

25-mers:

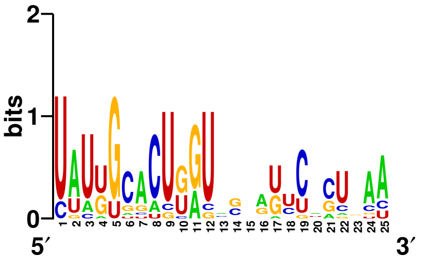

26-mers:

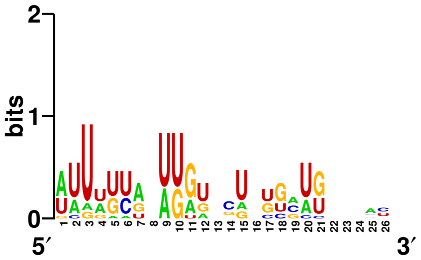

27-mers:

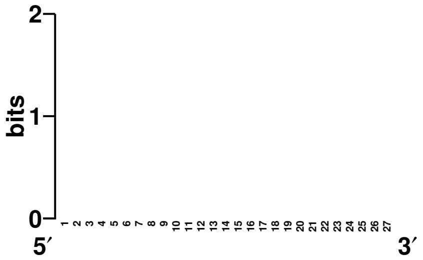

28-mers:

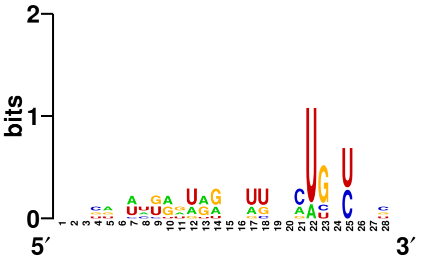

29-mers:

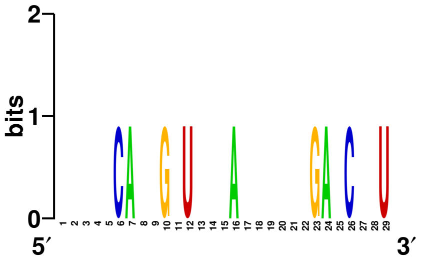

30-mers:

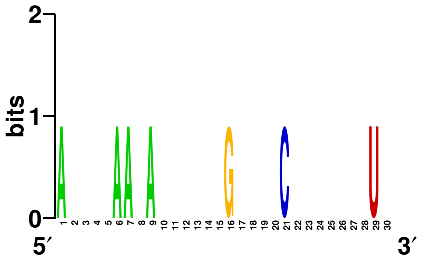

Antisense reads:

18-mers:

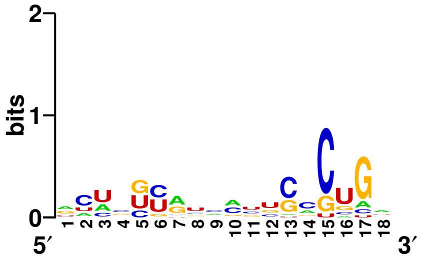

19-mers:
(no read)

20-mers:
(no read)

21-mers:
(no read)

22-mers:

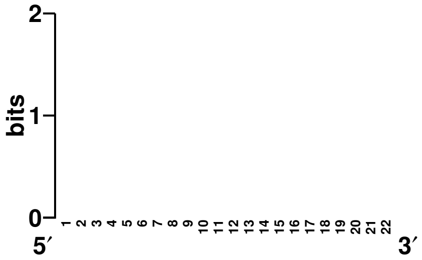

23-mers:

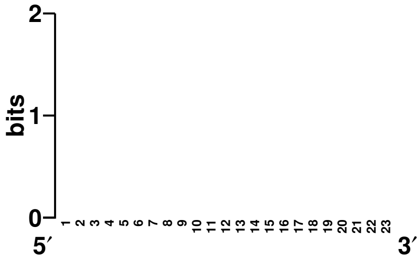

24-mers:

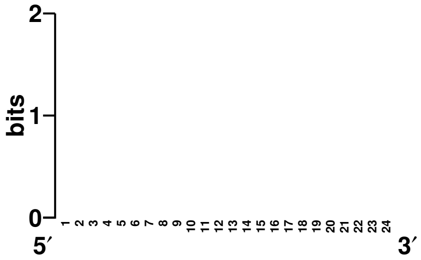

25-mers:

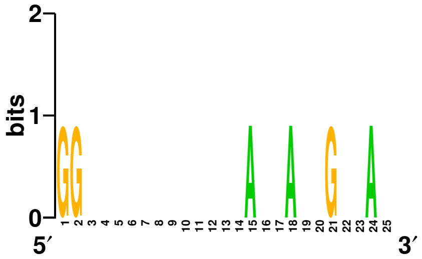

26-mers:
(no read)

27-mers:
(no read)

28-mers:
(no read)

29-mers:
(no read)

30-mers:
(no read)

Embryo 60h, library 3:

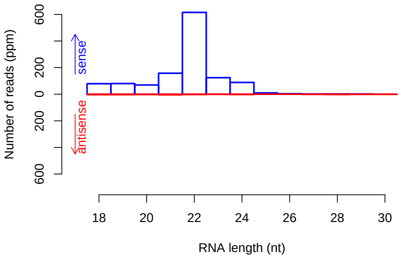

Sense reads:

18-mers:

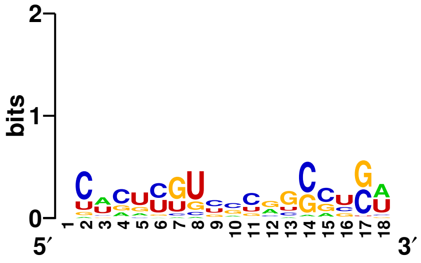

19-mers:

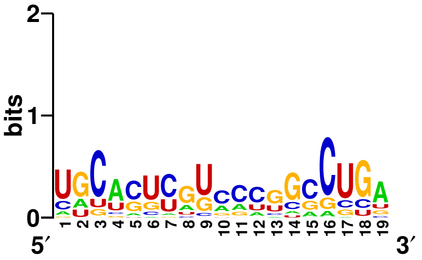

20-mers:

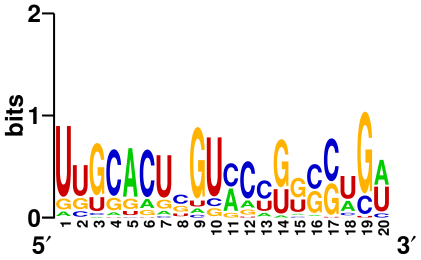

21-mers:

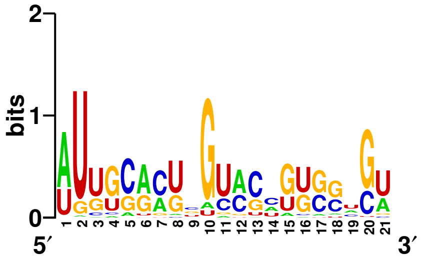

22-mers:

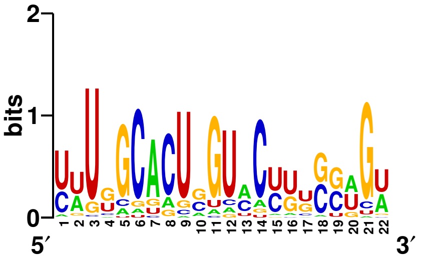

23-mers:

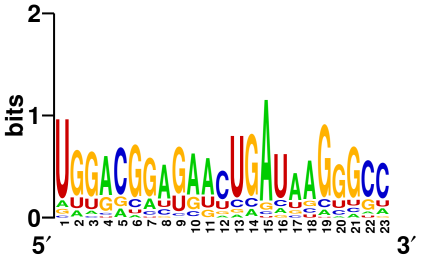

24-mers:

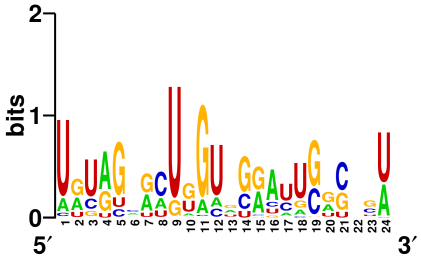

25-mers:

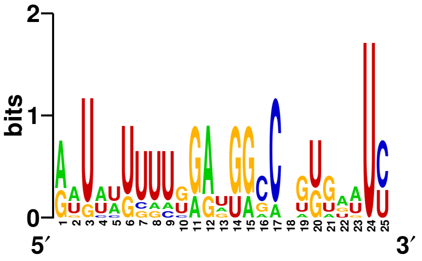

26-mers:

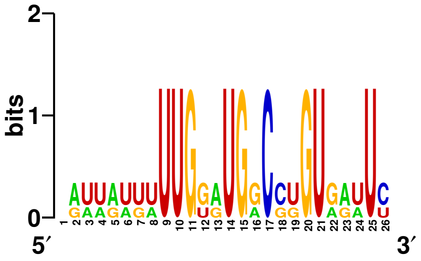

27-mers:

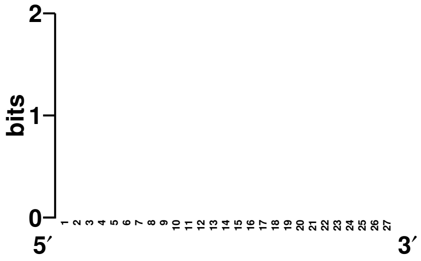

28-mers:

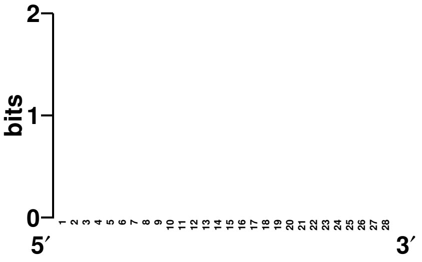

29-mers:

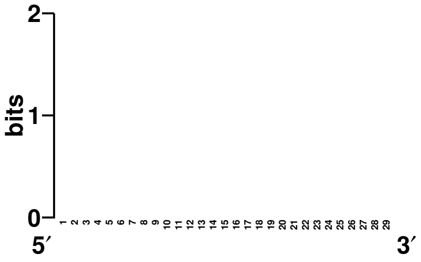

30-mers:

(no read)

Antisense reads:

18-mers:

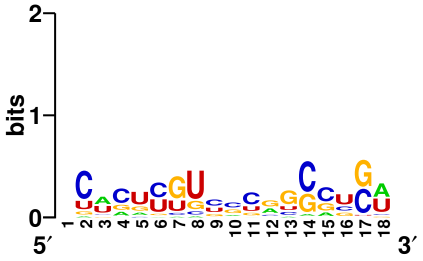

19-mers:

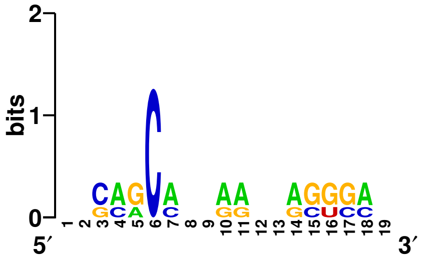

20-mers:

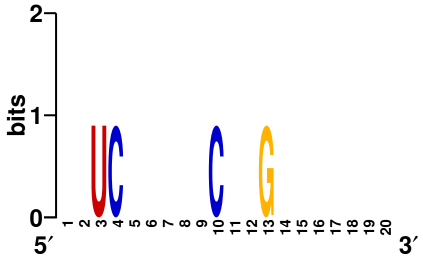

21-mers:

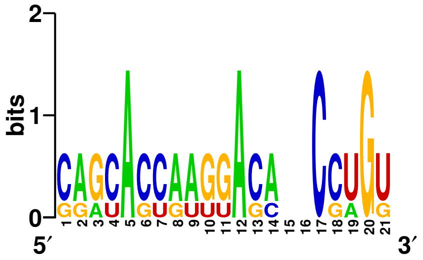

22-mers:

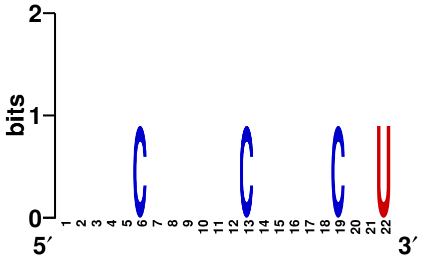

23-mers:

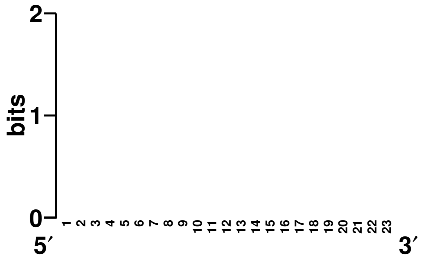

24-mers:

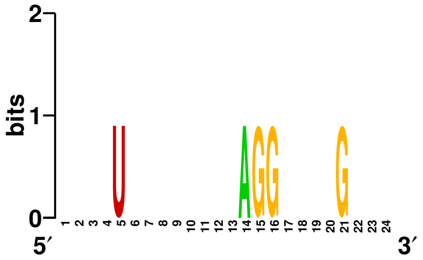

25-mers:

(no read)

26-mers:

(no read)

27-mers:

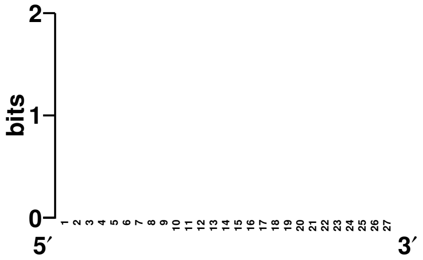

28-mers:

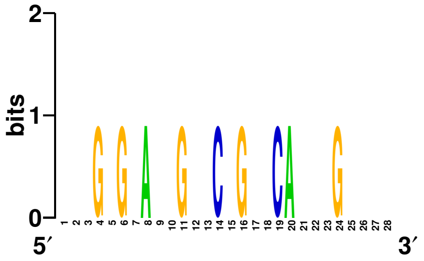

29-mers:

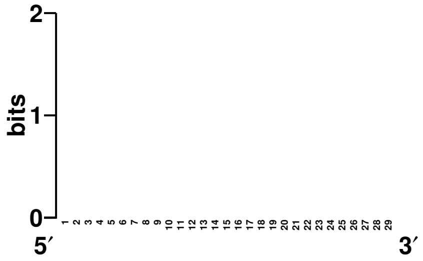

30-mers:

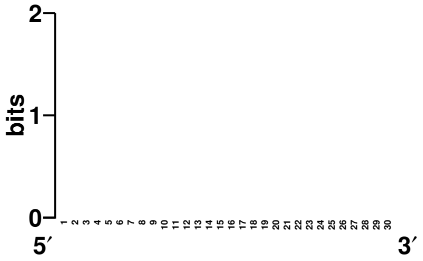

Adult female, library 3:

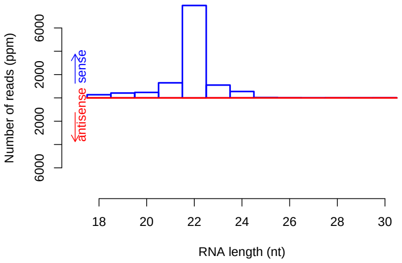

Sense reads:

18-mers:

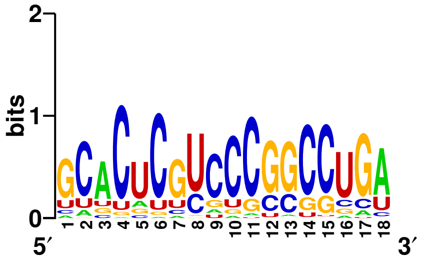

19-mers:

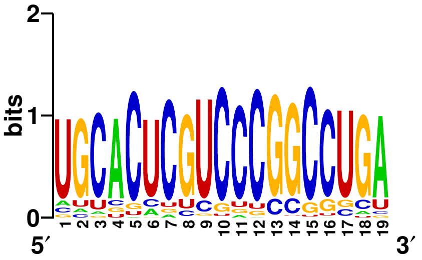

20-mers:

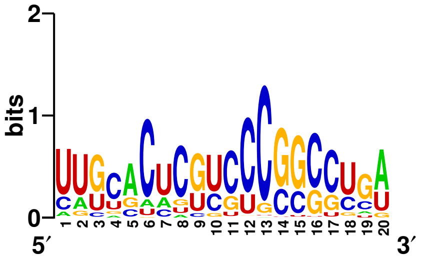

21-mers:

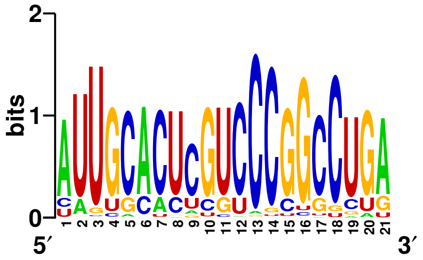

22-mers:

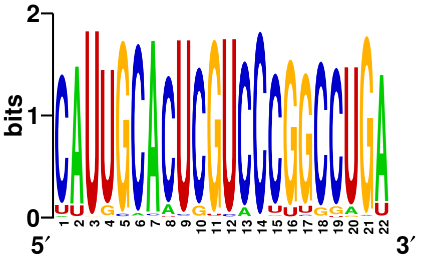

23-mers:

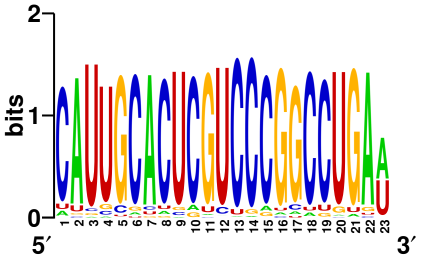

24-mers:

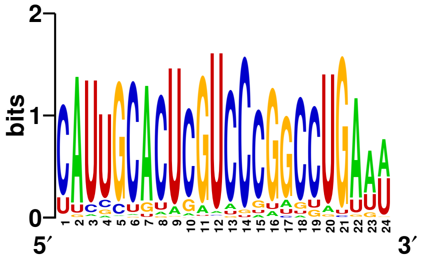

25-mers:

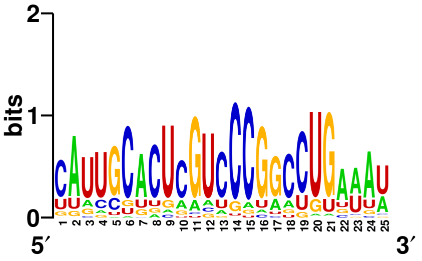

26-mers:

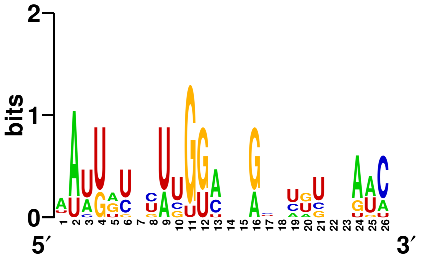

27-mers:

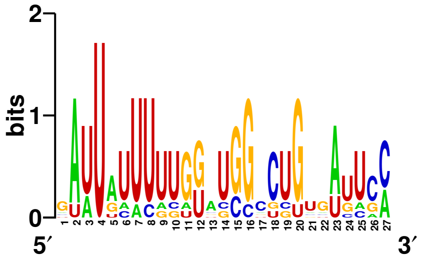

28-mers:

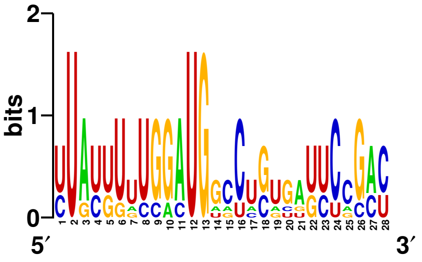

29-mers:

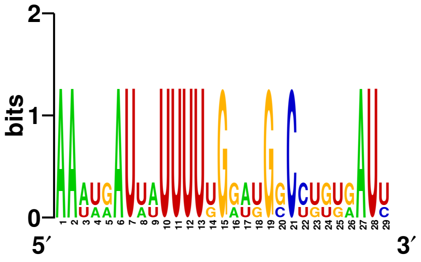

30-mers:

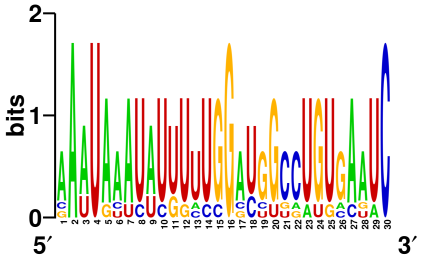

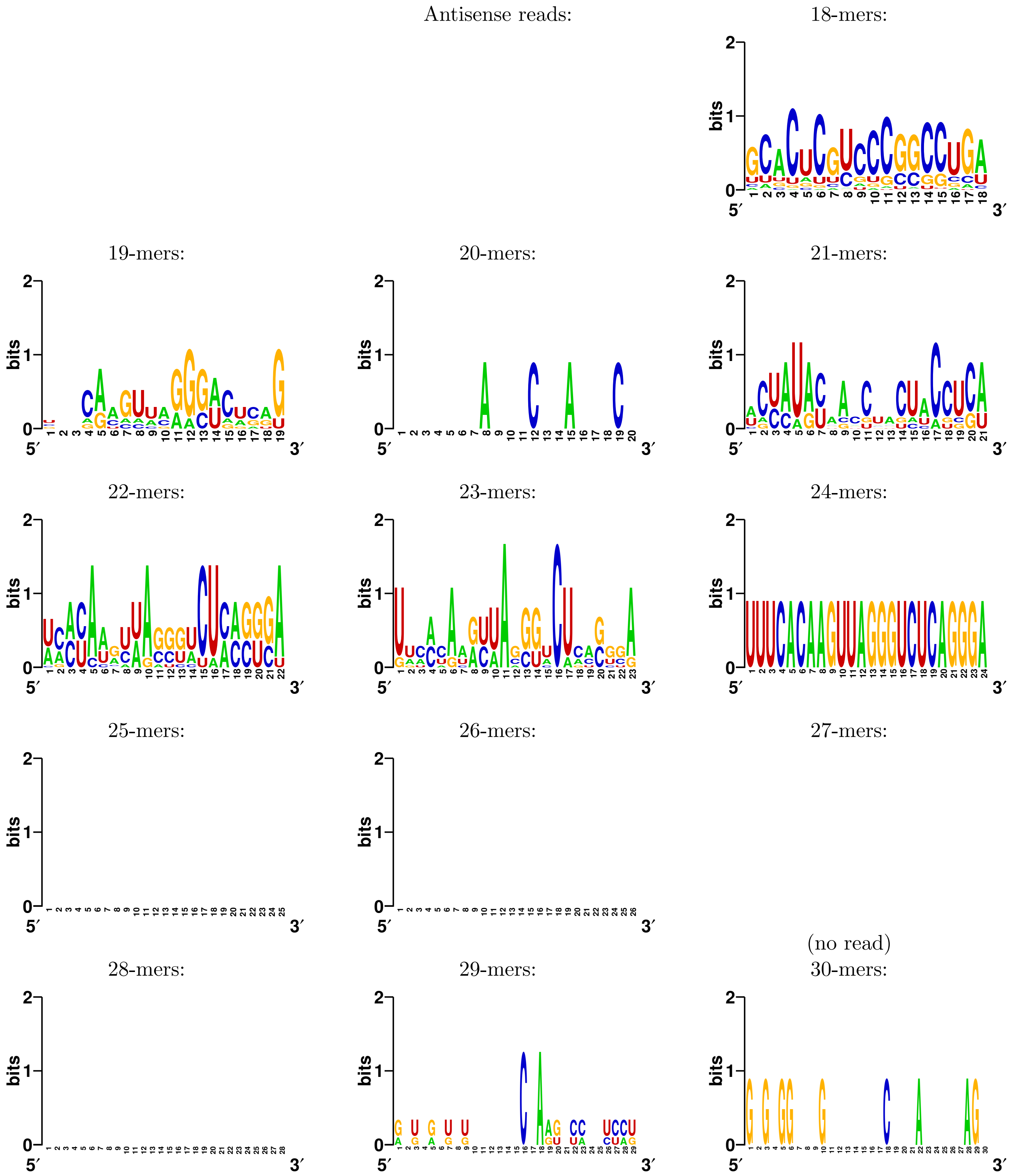
Antisense reads:
18-mers:
19-mers:
20-mers:
21-mers:
22-mers:
23-mers:
24-mers:
25-mers:
26-mers:
27-mers:
(no read)
28-mers:
29-mers:
30-mers:

Adult male, library 3:

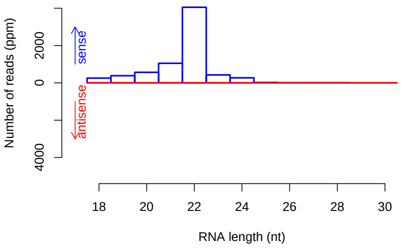

Sense reads:

18-mers:

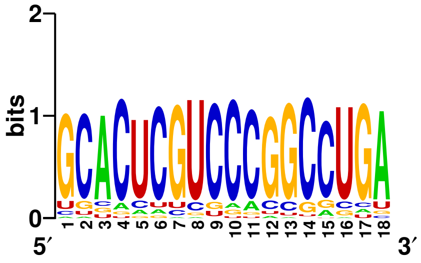

19-mers:

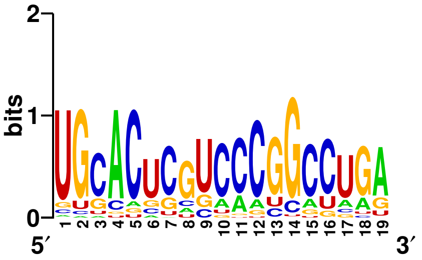

20-mers:

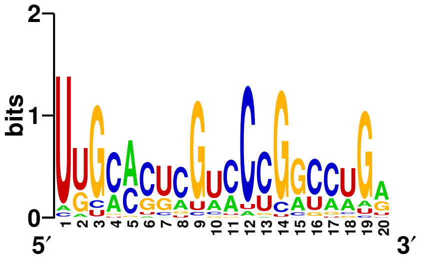

21-mers:

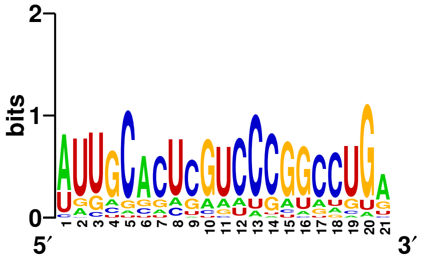

22-mers:

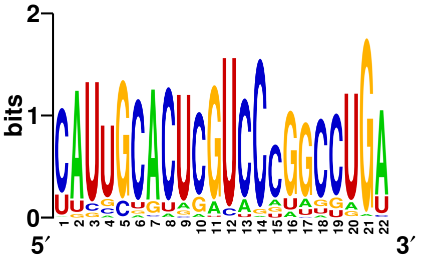

23-mers:

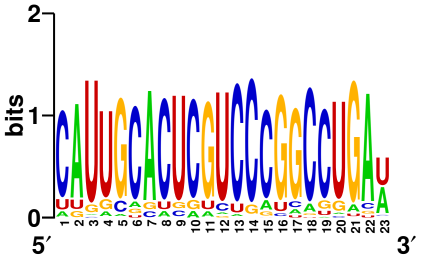

24-mers:

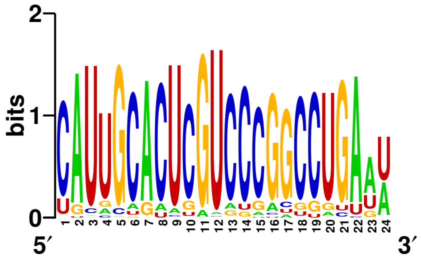

25-mers:

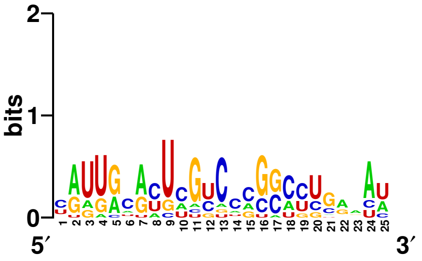

26-mers:

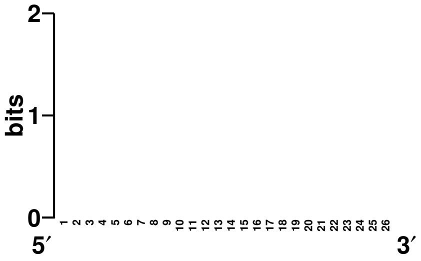

27-mers:

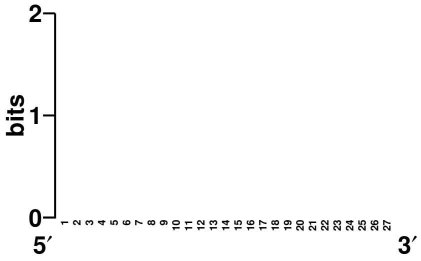

28-mers:

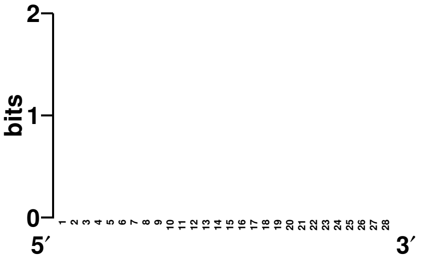

29-mers:

(no read)

30-mers:

(no read)

Antisense reads:

18-mers:

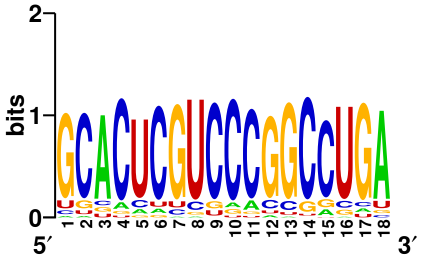

19-mers:

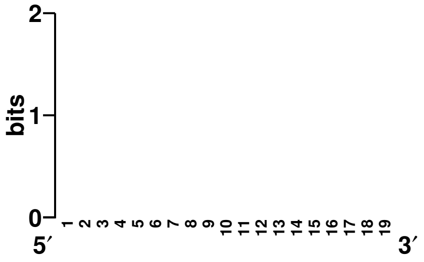

20-mers:

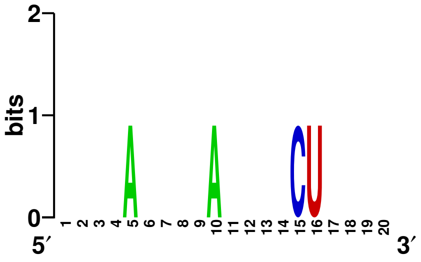

21-mers:

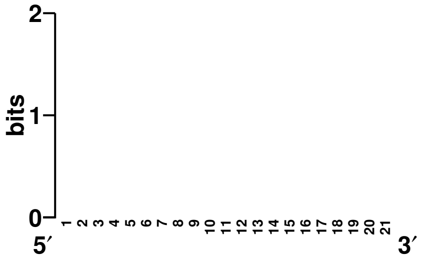

22-mers:

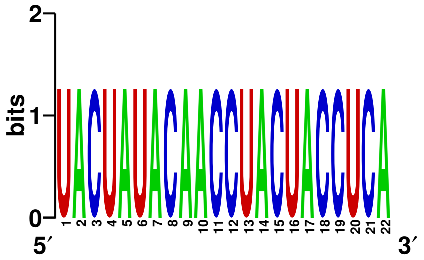

23-mers:

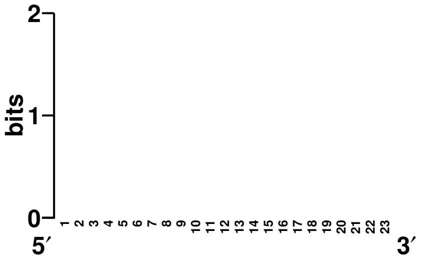

24-mers:

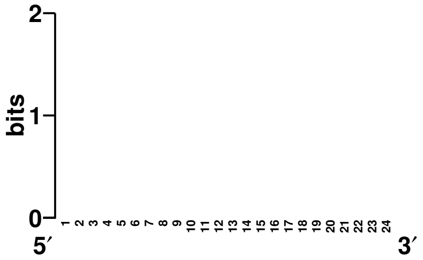

25-mers:
(no read)

26-mers:
(no read)

27-mers:
(no read)

28-mers:
(no read)

29-mers:
(no read)

30-mers:
(no read)

2.4 Libraries #4 (3´ modified, 5´ hydroxyl or polyphosphorylated small RNAs)

Embryo 8h, library 4:

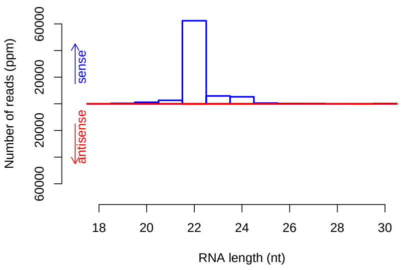

Sense reads:

18-mers:

(no read)

19-mers:

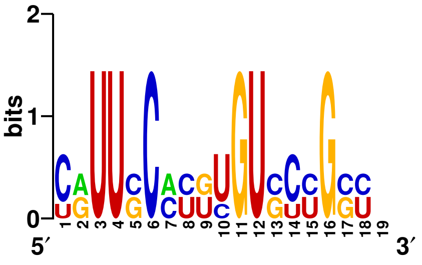

20-mers:

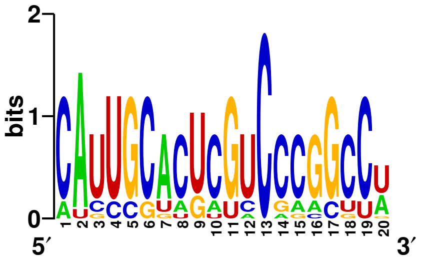

21-mers:

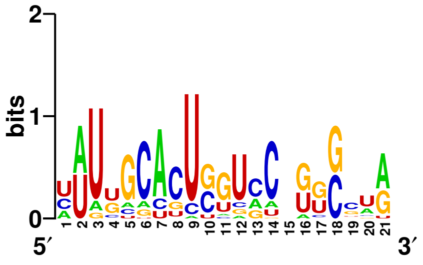

22-mers:

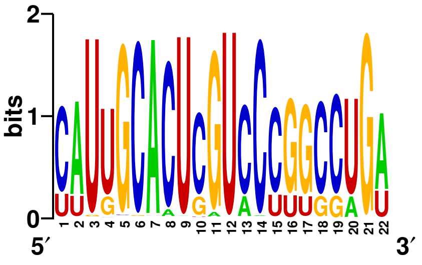

23-mers:

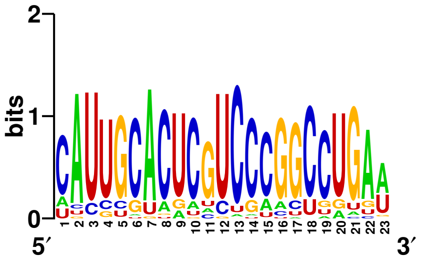

24-mers:

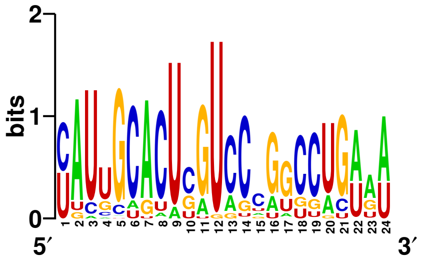

25-mers:

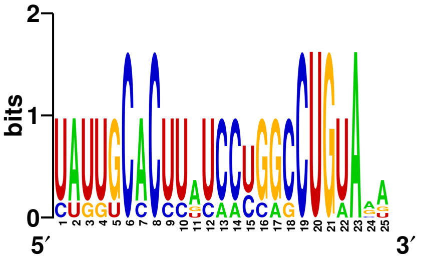

26-mers:

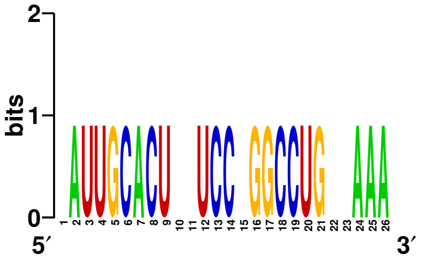

27-mers:

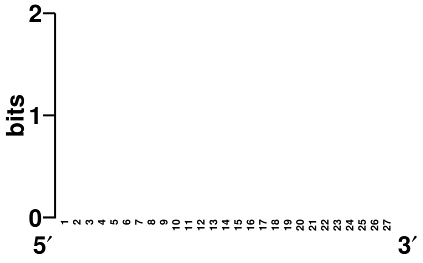

28-mers:

(no read)

29-mers:

(no read)

30-mers:

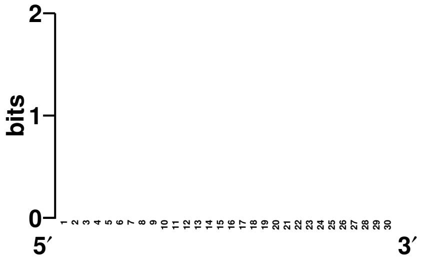

Antisense reads:

18-mers:
(no read)

19-mers:
(no read)

20-mers:
(no read)

21-mers:
(no read)

22-mers:

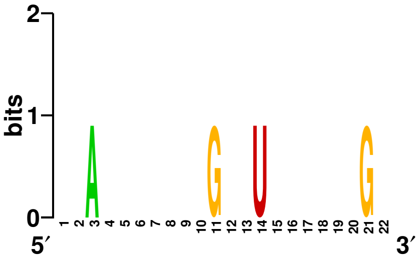

23-mers:

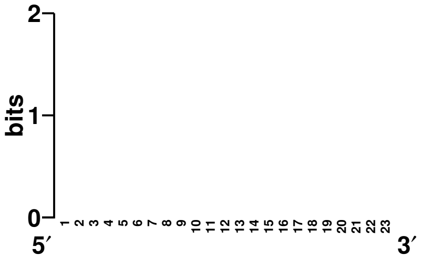

24-mers:

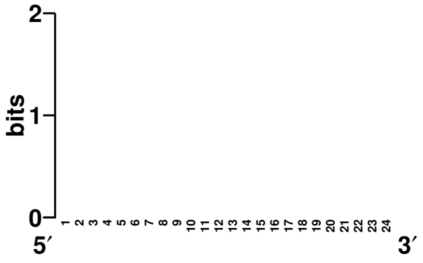

25-mers:
(no read)

26-mers:
(no read)

27-mers:
(no read)

28-mers:
(no read)

29-mers:

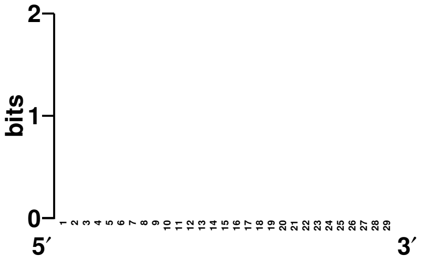

30-mers:
(no read)

Embryo 15h, library 4:

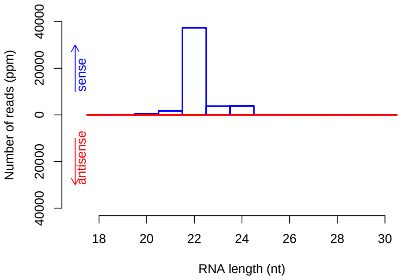

Sense reads:

18-mers:

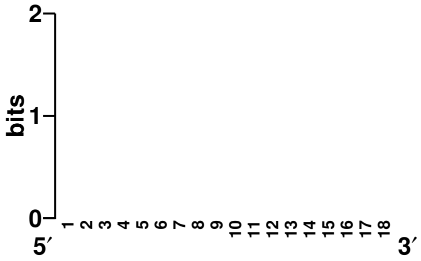

19-mers:

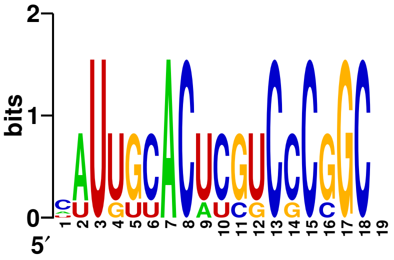

20-mers:

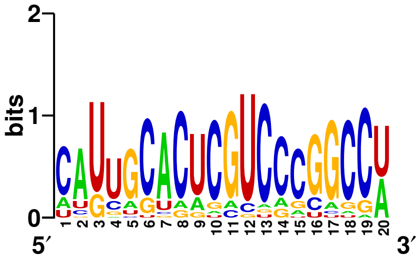

21-mers:

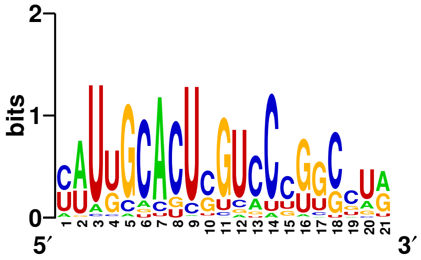

22-mers:

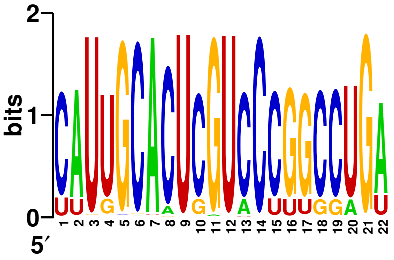

23-mers:

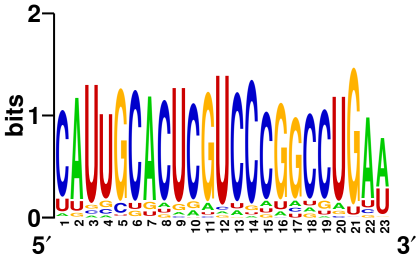

24-mers:

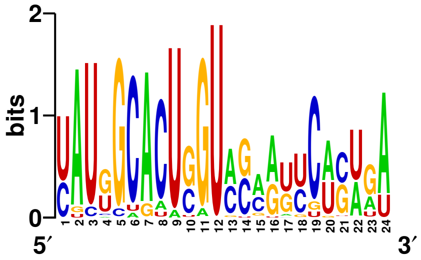

25-mers:

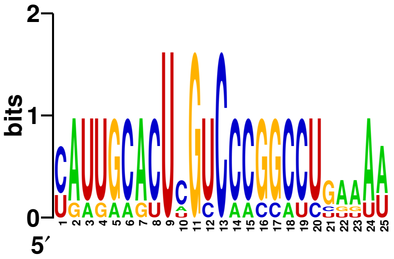

26-mers:

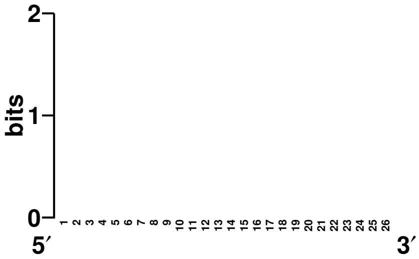

27-mers:

(no read)

28-mers:

(no read)

29-mers:

(no read)

30-mers:

(no read)

Antisense reads:

18-mers:

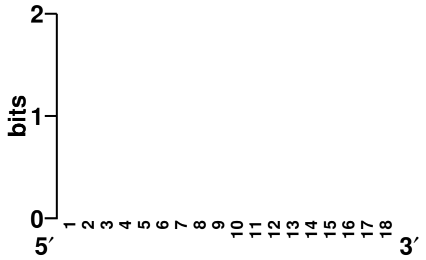

19-mers:

(no read)

20-mers:

(no read)

21-mers:

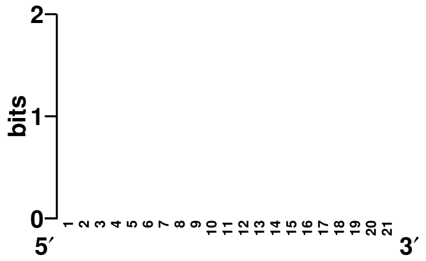

22-mers:

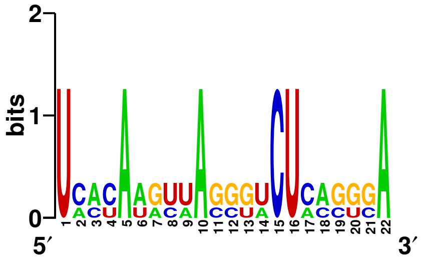

23-mers:

(no read)

24-mers:

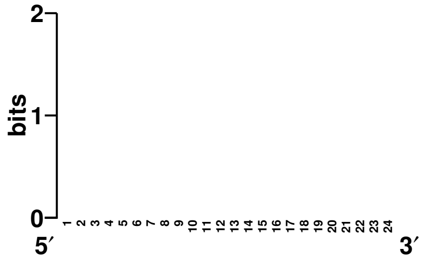

25-mers:

(no read)

26-mers:

(no read)

27-mers:

(no read)

28-mers:

(no read)

29-mers:

(no read)

30-mers:

(no read)

Embryo 36h, library 4:

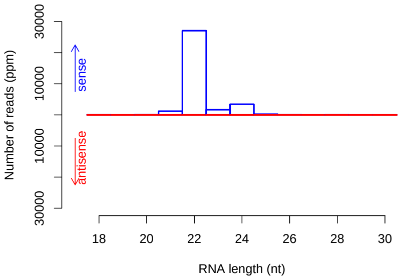

Sense reads:

18-mers:

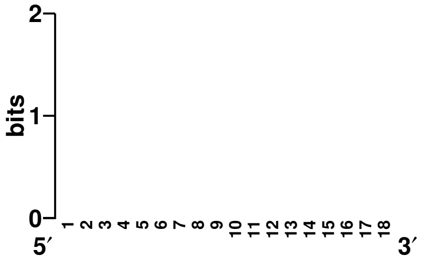

19-mers:

(no read)

20-mers:

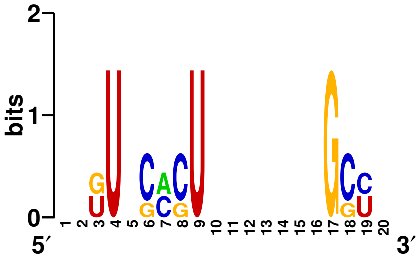

21-mers:

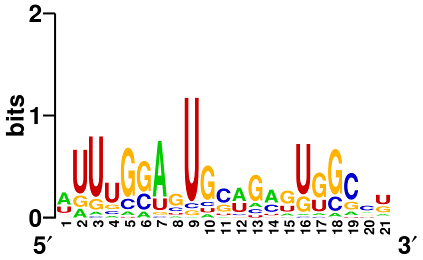

22-mers:

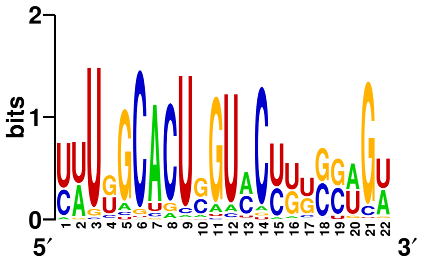

23-mers:

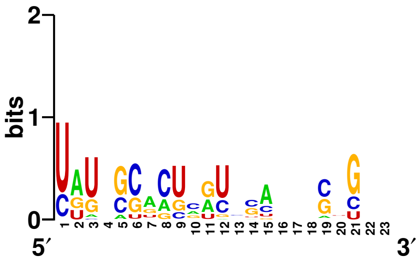

24-mers:

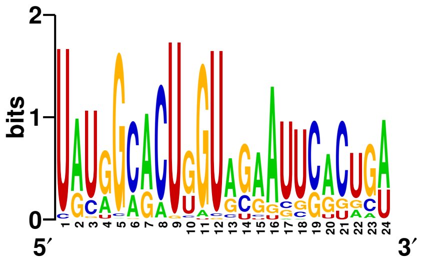

25-mers:

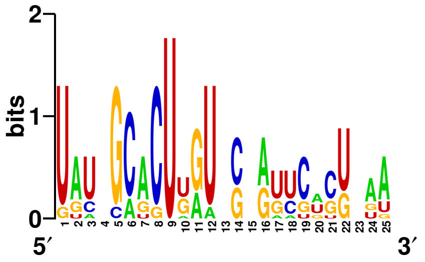

26-mers:

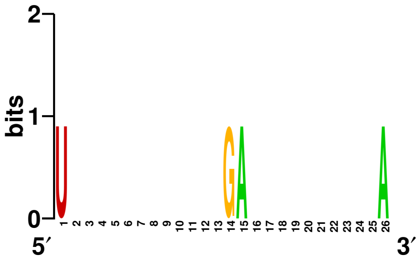

27-mers:

(no read)

28-mers:

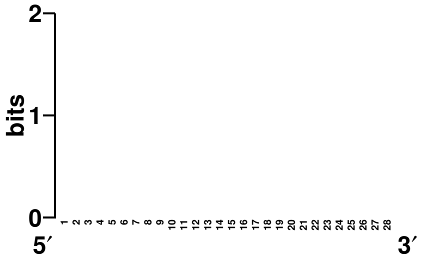

29-mers:

(no read)

30-mers:

(no read)

Antisense reads: 18-mers:

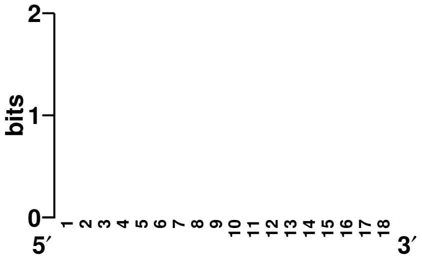

19-mers: 20-mers: 21-mers:

(no read) (no read) (no read)

22-mers: 23-mers: 24-mers:

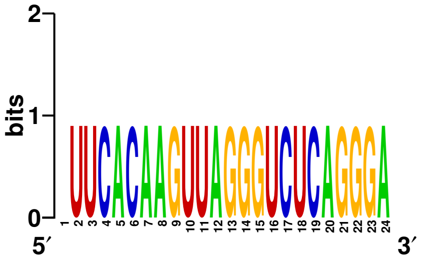

(no read) (no read)

25-mers: 26-mers: 27-mers:

(no read) (no read) (no read)

28-mers: 29-mers: 30-mers:

(no read) (no read) (no read)

Embryo 60h, library 4:

Sense reads:

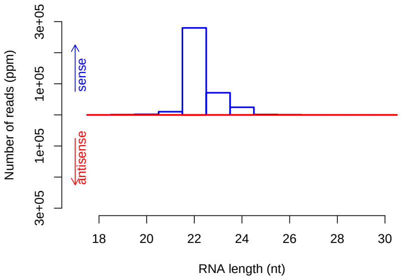

18-mers:

(no read)

19-mers:

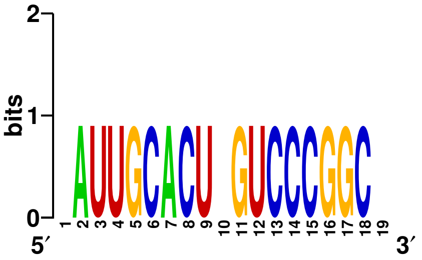

20-mers:

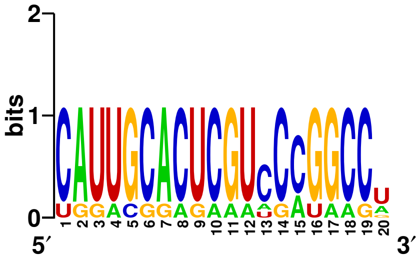

21-mers:

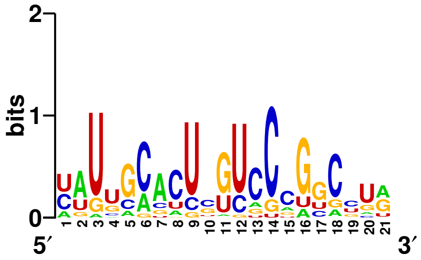

22-mers:

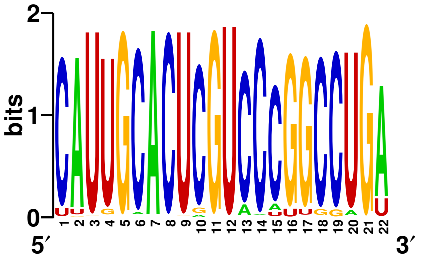

23-mers:

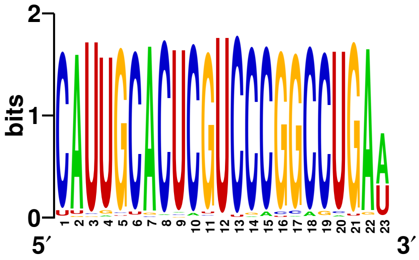

24-mers:

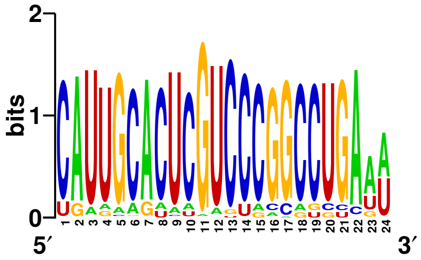

25-mers:

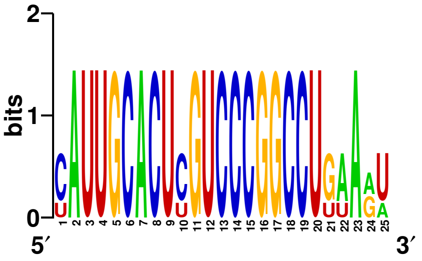

26-mers:

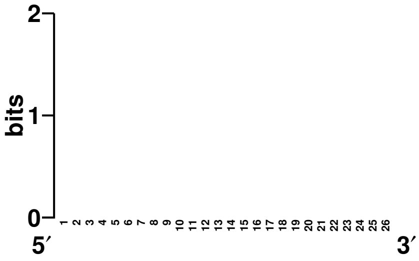

27-mers:

(no read)

28-mers:

(no read)

29-mers:

(no read)

30-mers:

(no read)

	Antisense reads:	18-mers:
		(no read)
19-mers:	20-mers:	21-mers:
(no read)	(no read)	(no read)
22-mers:	23-mers:	24-mers:
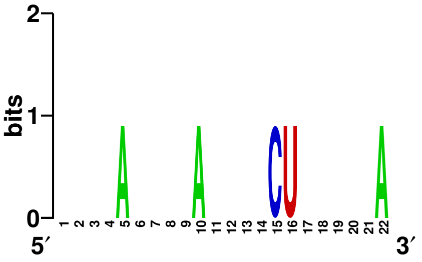	(no read)	(no read)
25-mers:	26-mers:	27-mers:
(no read)	(no read)	(no read)
28-mers:	29-mers:	30-mers:
(no read)	(no read)	(no read)

Adult female, library 4:

Sense reads:

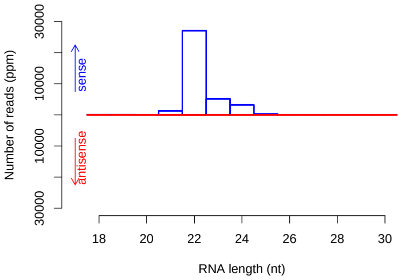

18-mers:

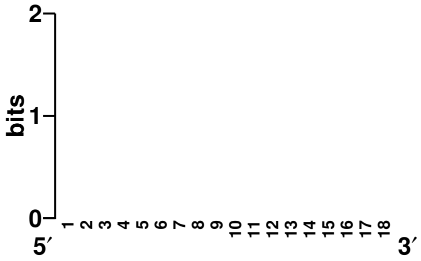

19-mers:

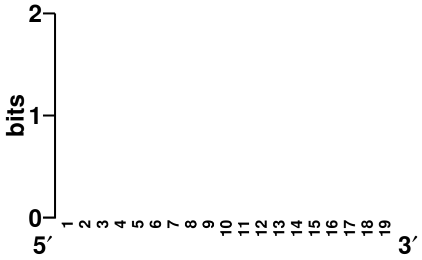

20-mers:

(no read)

21-mers:

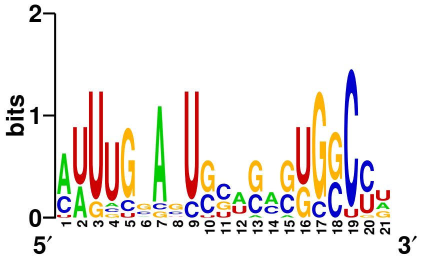

22-mers:

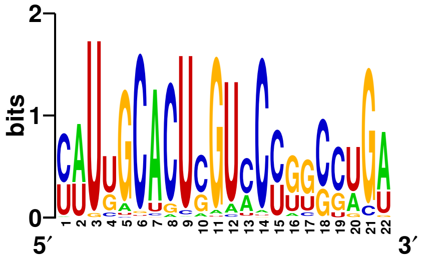

23-mers:

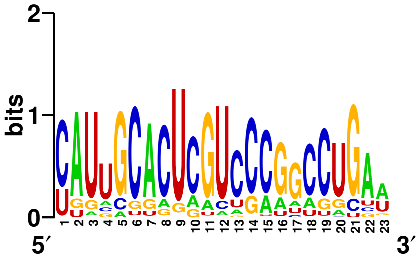

24-mers:

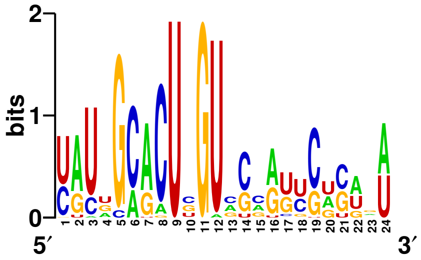

25-mers:

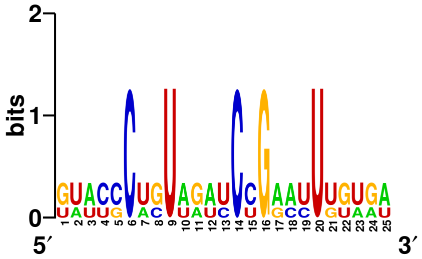

26-mers:

(no read)

27-mers:

(no read)

28-mers:

(no read)

29-mers:

(no read)

30-mers:

(no read)

Antisense reads:

18-mers:

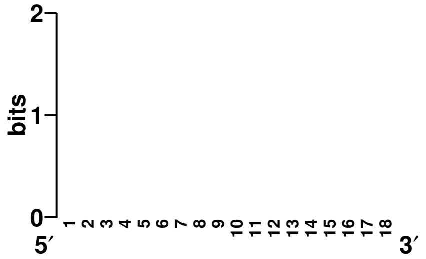

19-mers:
(no read)

20-mers:
(no read)

21-mers:
(no read)

22-mers:

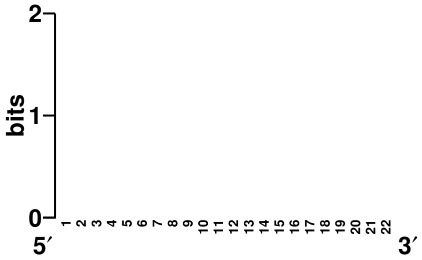

23-mers:
(no read)

24-mers:
(no read)

25-mers:
(no read)

26-mers:
(no read)

27-mers:
(no read)

28-mers:
(no read)

29-mers:
(no read)

30-mers:
(no read)

Adult male, library 4:

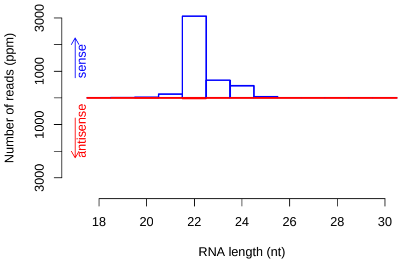

Sense reads:

18-mers:

(no read)

19-mers:

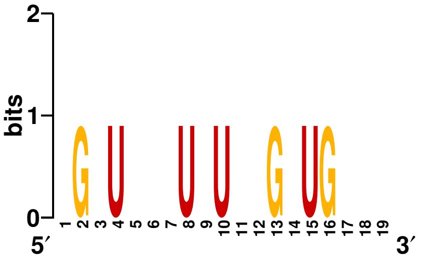

20-mers:

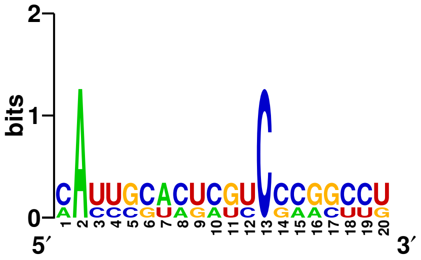

21-mers:

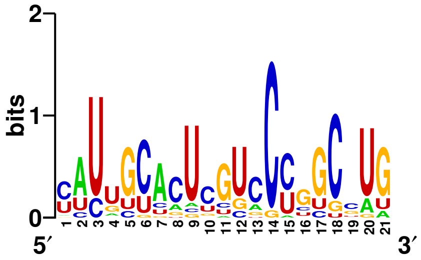

22-mers:

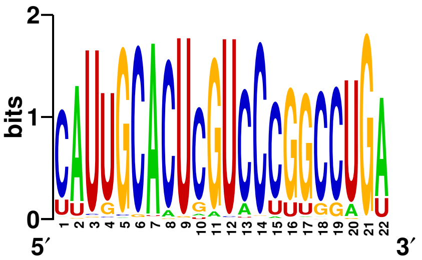

23-mers:

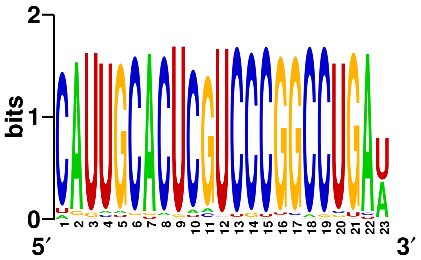

24-mers:

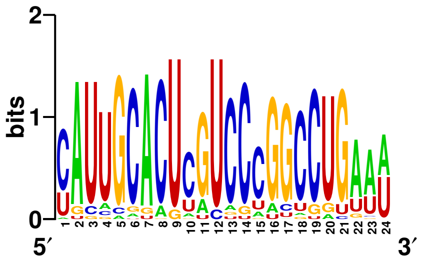

25-mers:

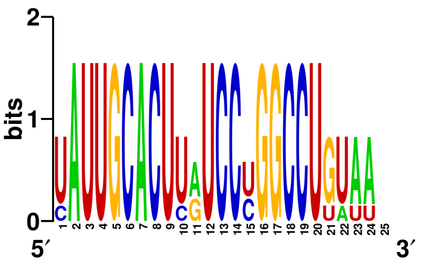

26-mers:

(no read)

27-mers:

(no read)

28-mers:

(no read)

29-mers:

(no read)

30-mers:

(no read)

Antisense reads:

18-mers:
(no read)

19-mers:
(no read)

20-mers:

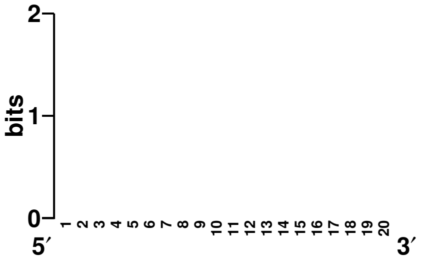

21-mers:
(no read)

22-mers:

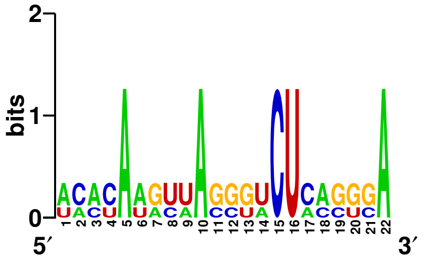

23-mers:
(no read)

24-mers:
(no read)

25-mers:
(no read)

26-mers:
(no read)

27-mers:
(no read)

28-mers:
(no read)

29-mers:
(no read)

30-mers:
(no read)

3 Transcriptome-matching reads (excuding pre-miRNA and abundant ncRNA-matching reads)

3.1 Libraries #1 (total 5´ monophosphorylated small RNAs)

Embryo 8h, library 1:

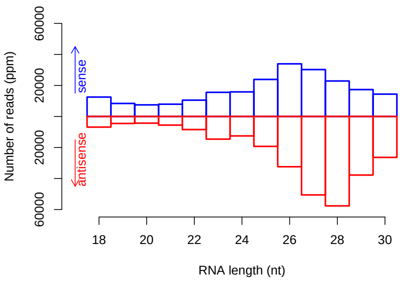

Sense reads:

18-mers:

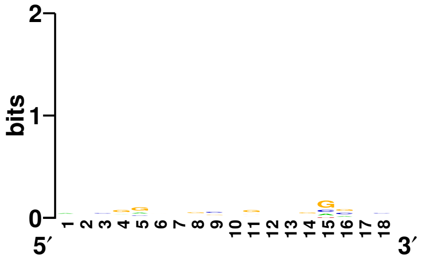

19-mers:

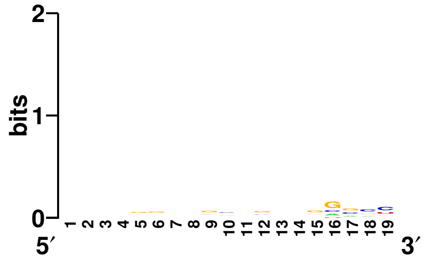

20-mers:

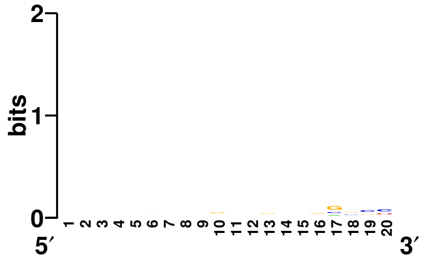

21-mers:

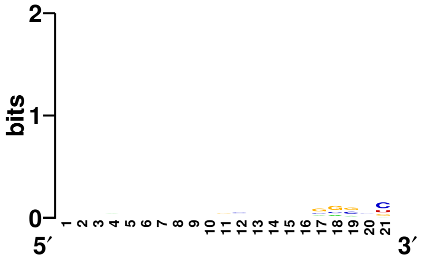

22-mers:

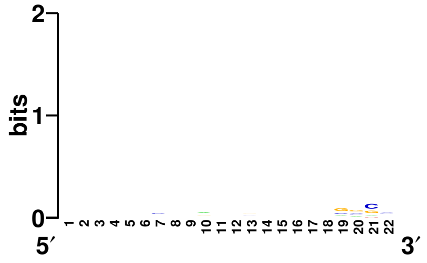

23-mers:

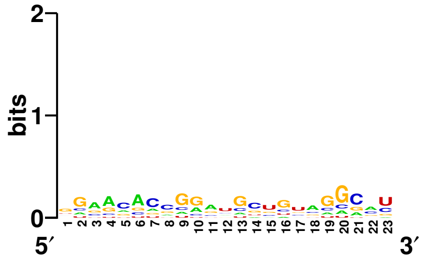

24-mers:

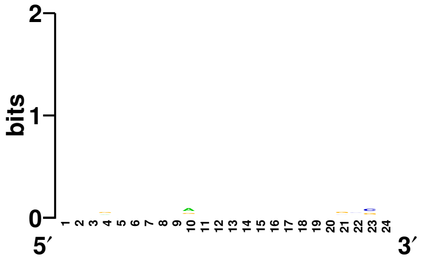

25-mers:

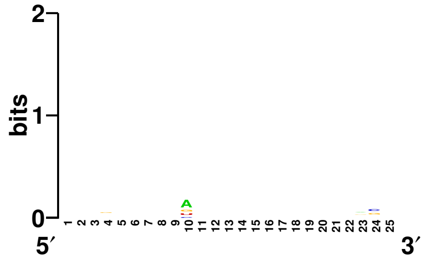

26-mers:

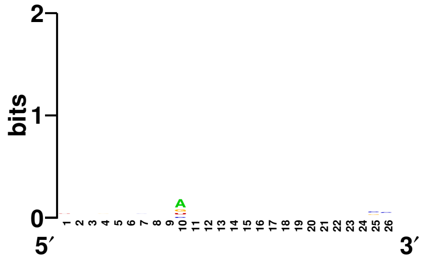

27-mers:

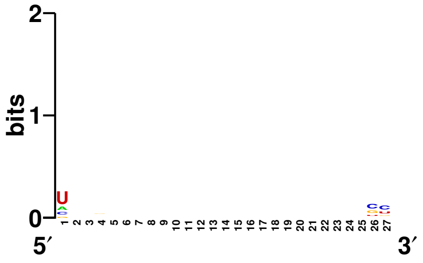

28-mers:

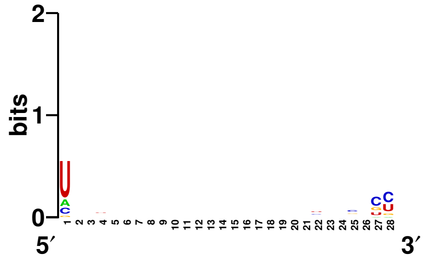

29-mers:

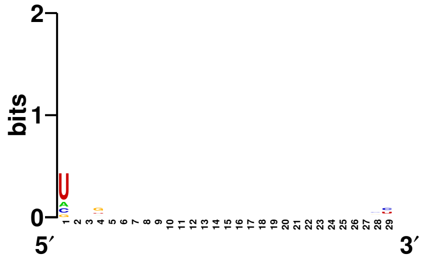

30-mers:

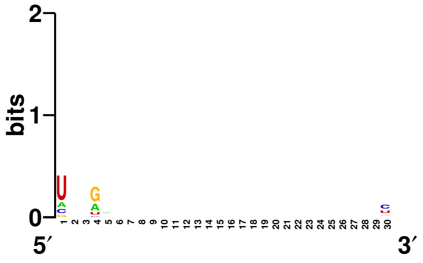

Antisense reads:

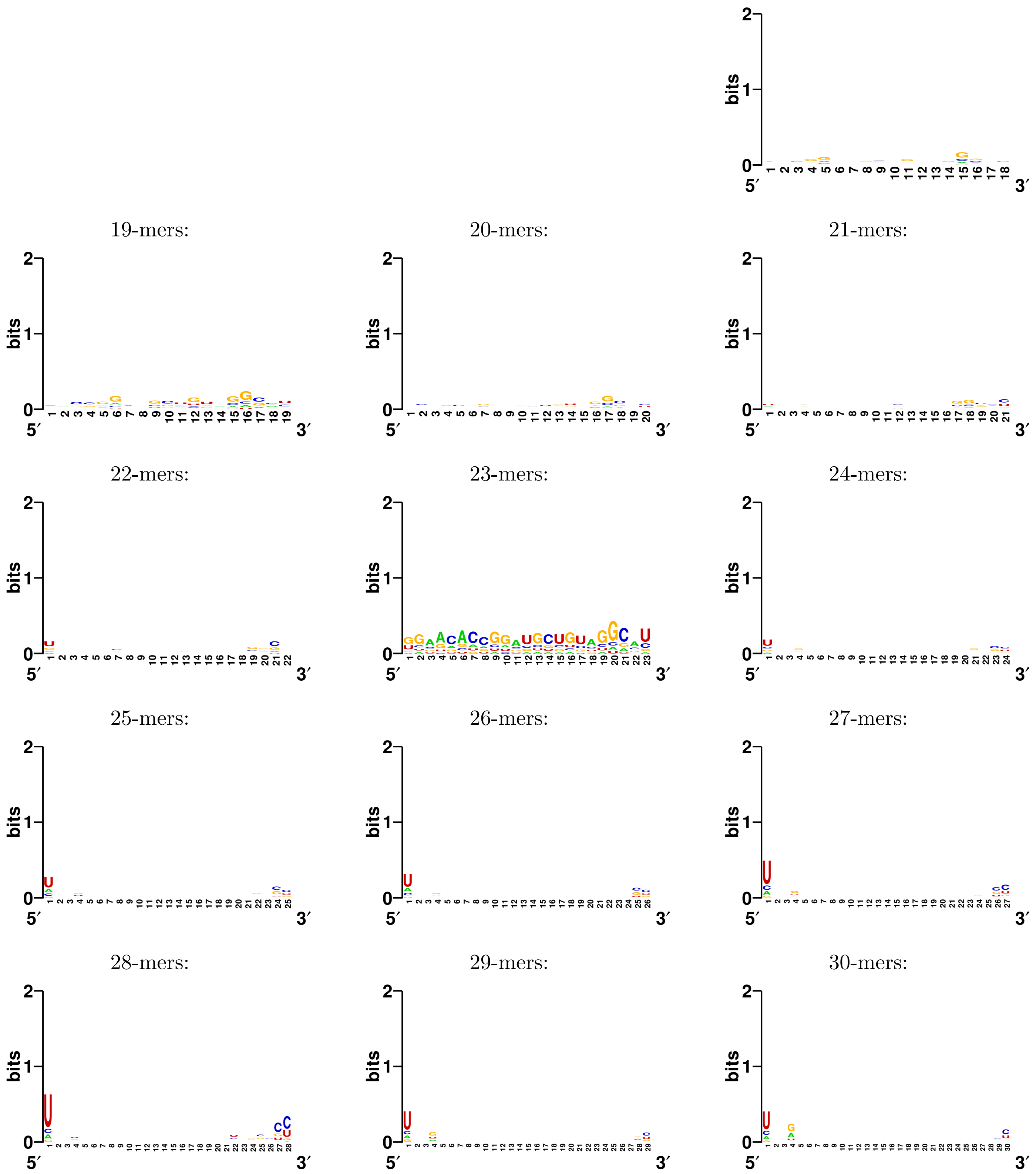

Embryo 15h, library 1:

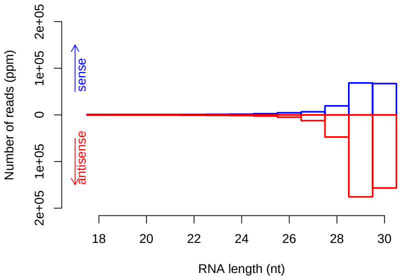

Sense reads:

18-mers:

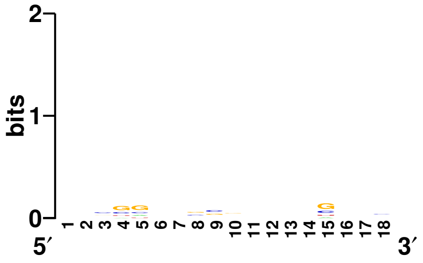

19-mers:

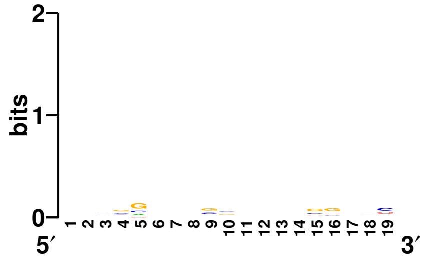

20-mers:

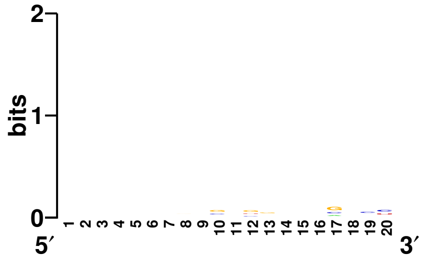

21-mers:

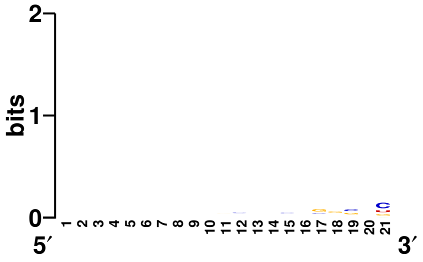

22-mers:

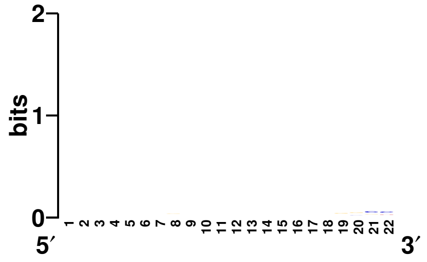

23-mers:

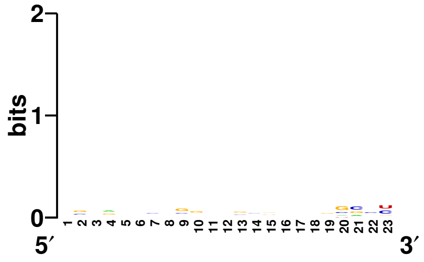

24-mers:

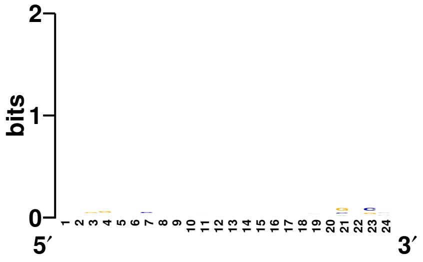

25-mers:

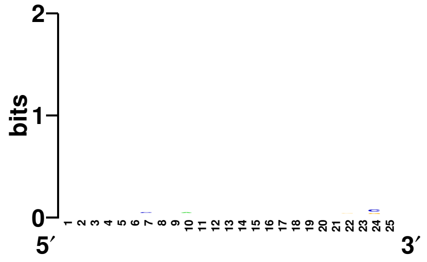

26-mers:

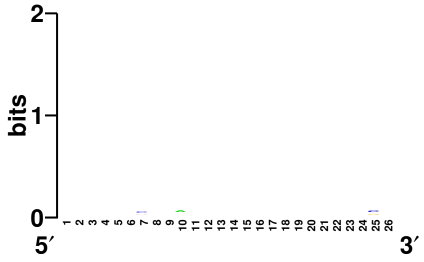

27-mers:

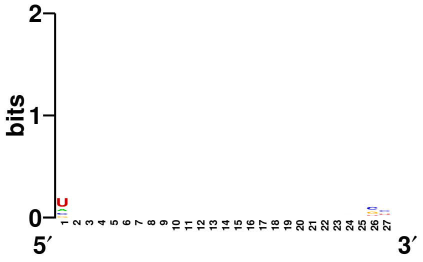

28-mers:

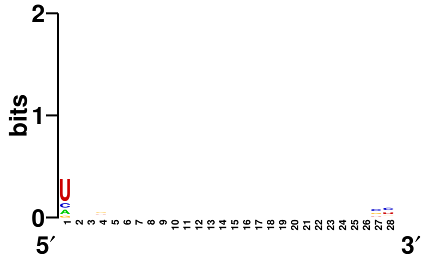

29-mers:

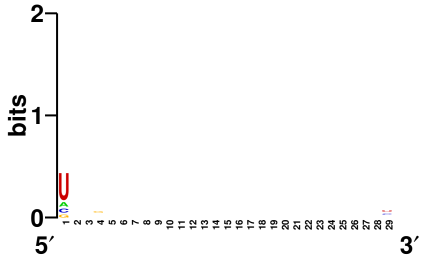

30-mers:

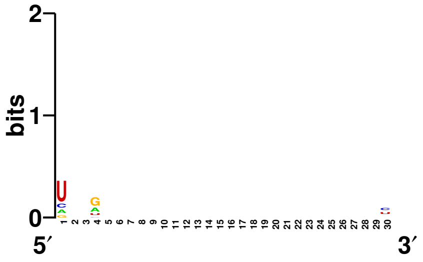

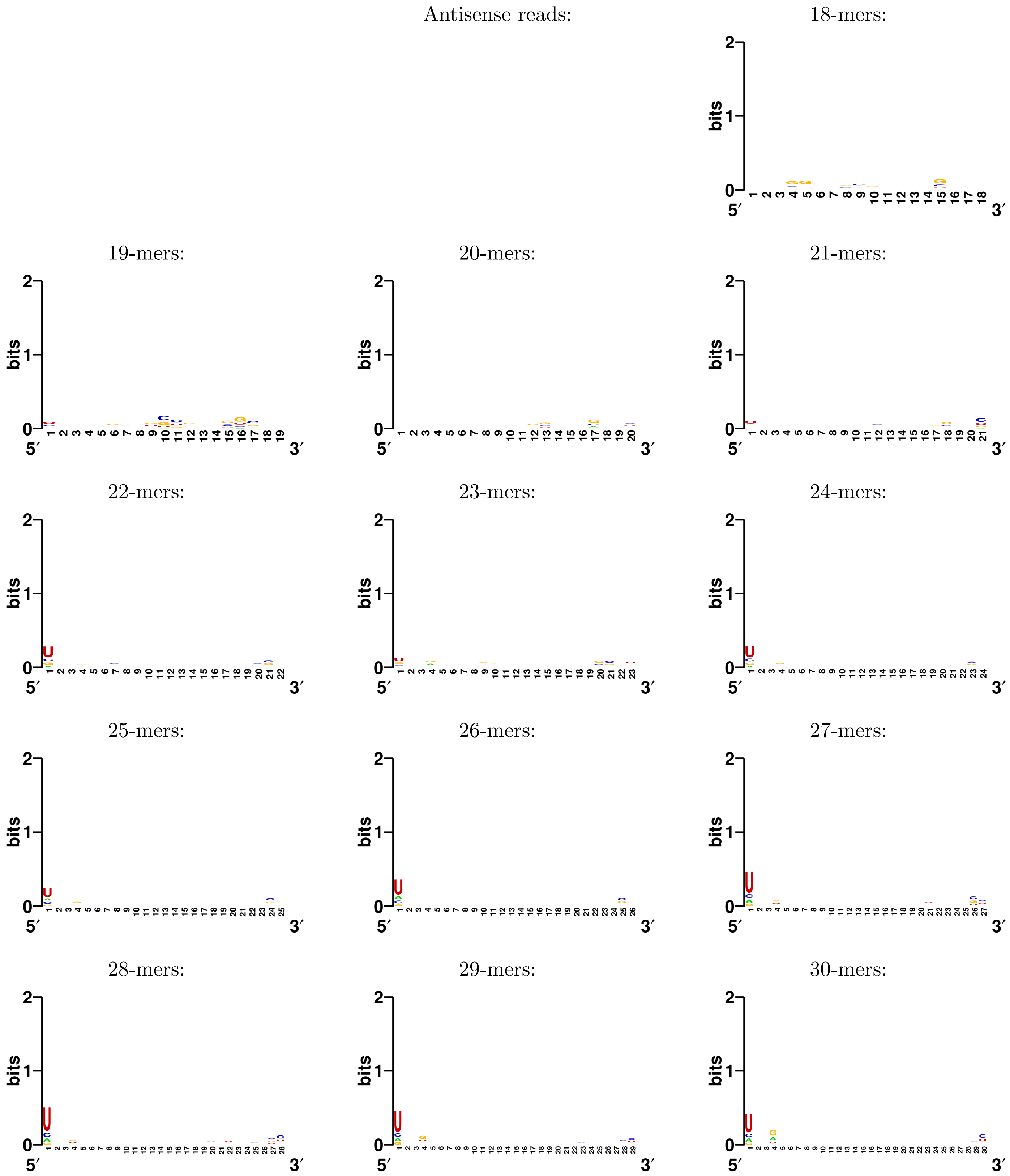
Antisense reads:
18-mers:
19-mers:
20-mers:
21-mers:
22-mers:
23-mers:
24-mers:
25-mers:
26-mers:
27-mers:
28-mers:
29-mers:
30-mers:
bits
5′
3′

Embryo 36h, library 1:

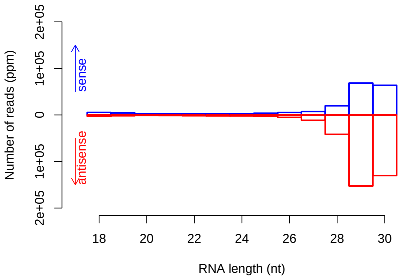

Sense reads:

18-mers:

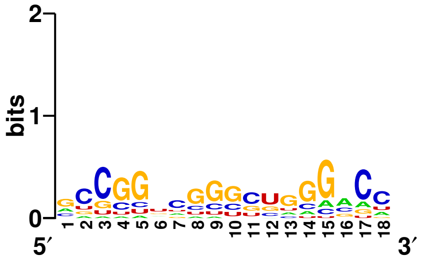

19-mers:

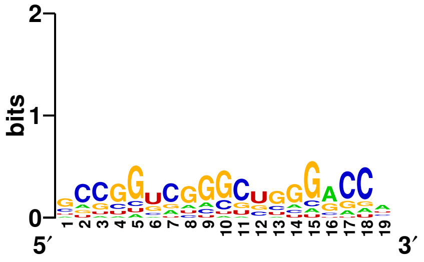

20-mers:

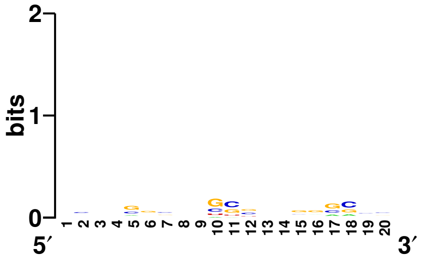

21-mers:

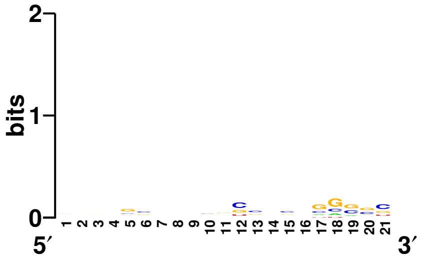

22-mers:

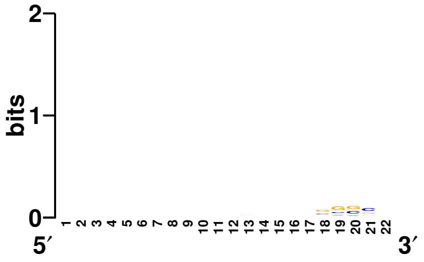

23-mers:

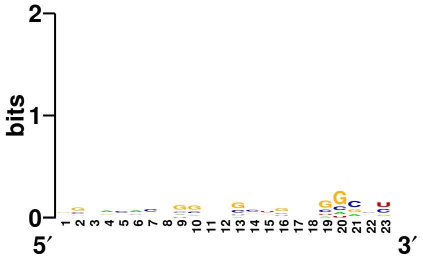

24-mers:

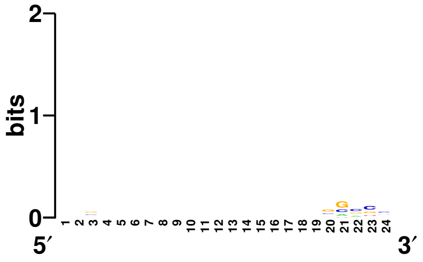

25-mers:

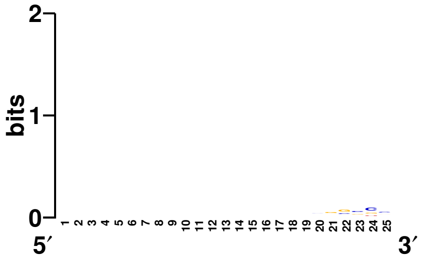

26-mers:

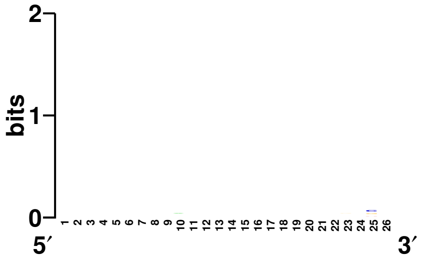

27-mers:

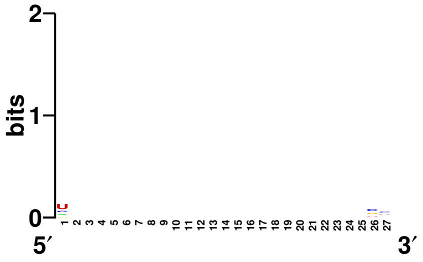

28-mers:

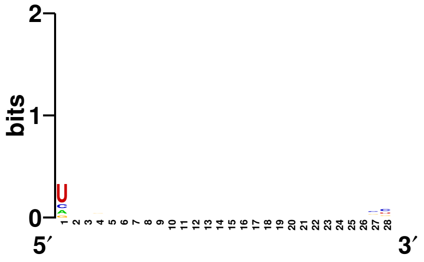

29-mers:

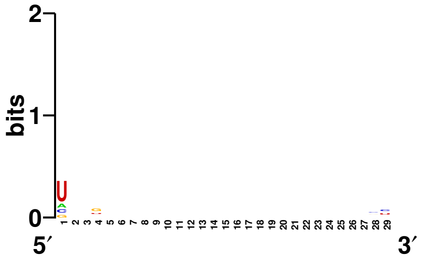

30-mers:

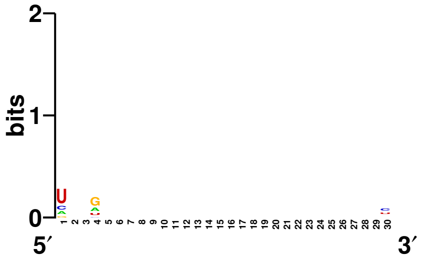

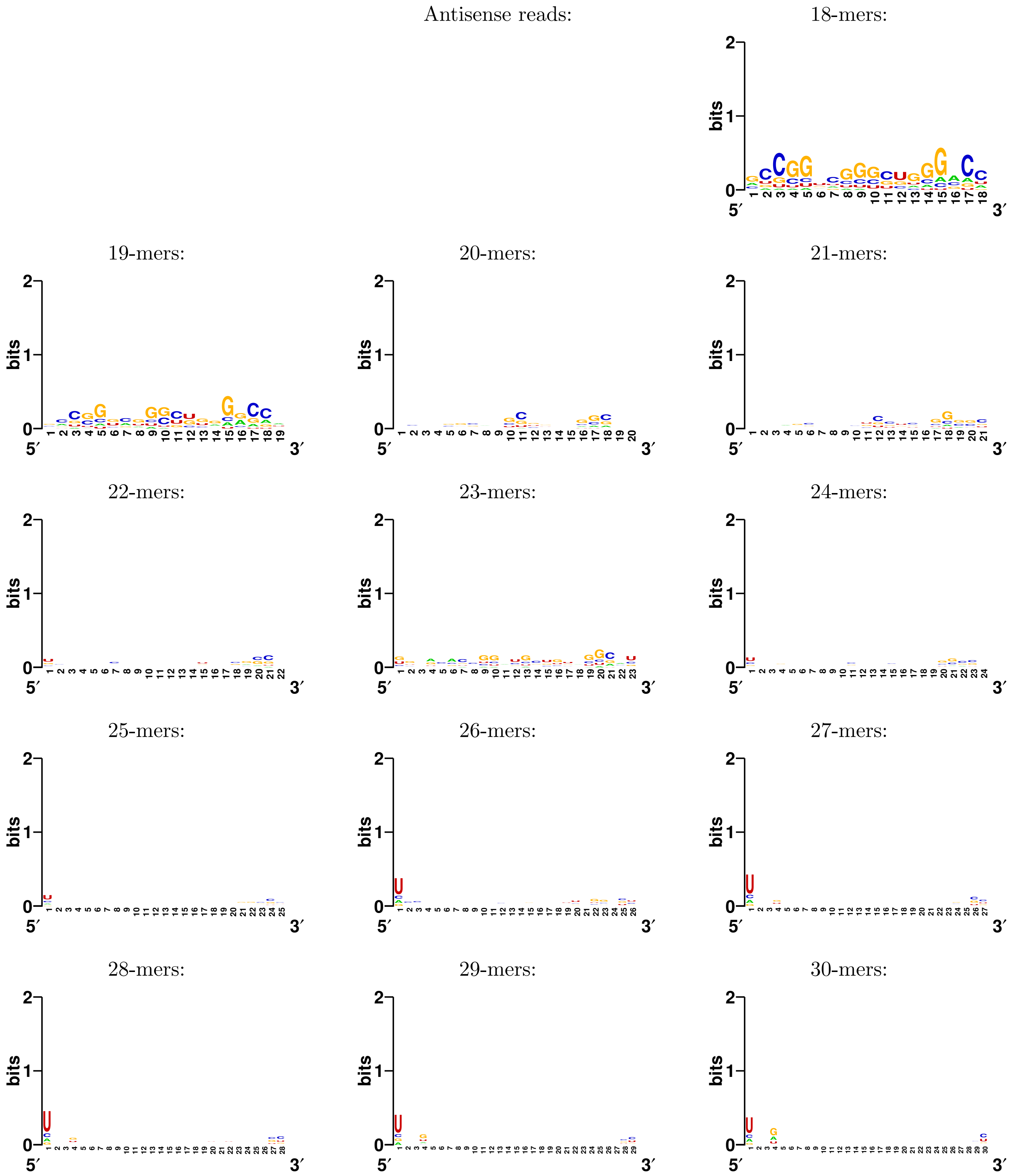
Antisense reads:
18-mers:
19-mers:
20-mers:
21-mers:
22-mers:
23-mers:
24-mers:
25-mers:
26-mers:
27-mers:
28-mers:
29-mers:
30-mers:
bits
5′
3′

Embryo 60h, library 1:

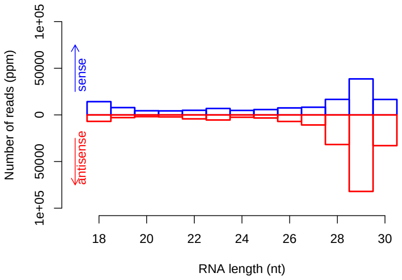

Sense reads:

18-mers:

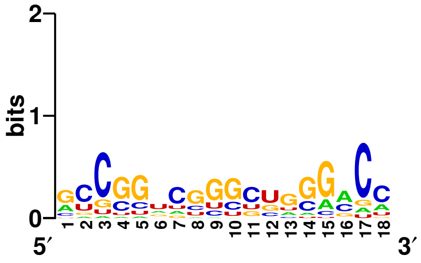

19-mers:

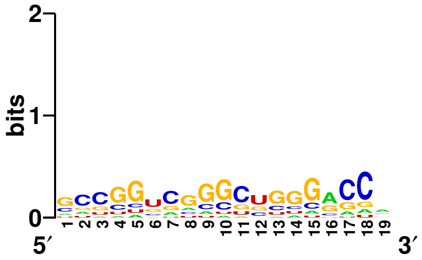

20-mers:

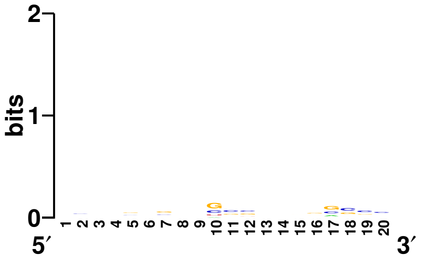

21-mers:

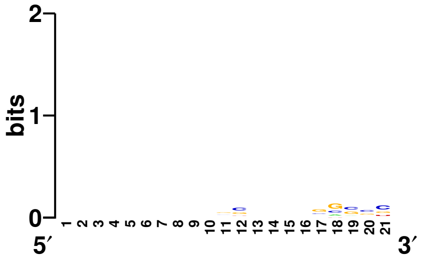

22-mers:

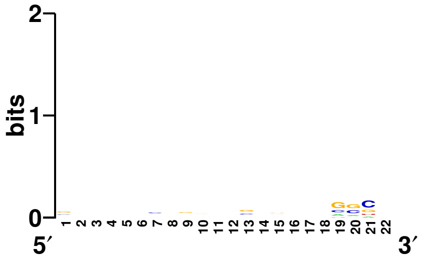

23-mers:

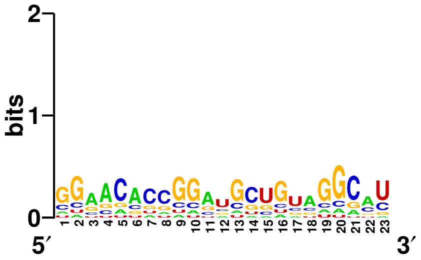

24-mers:

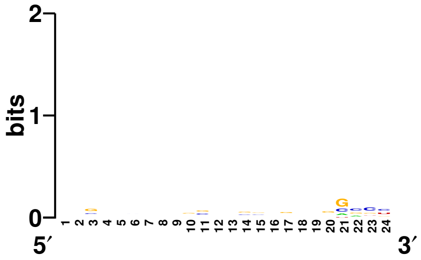

25-mers:

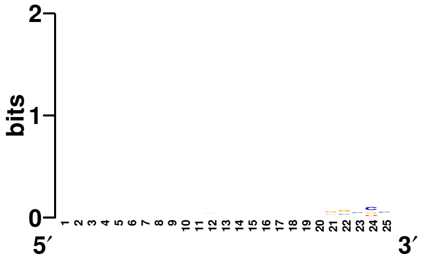

26-mers:

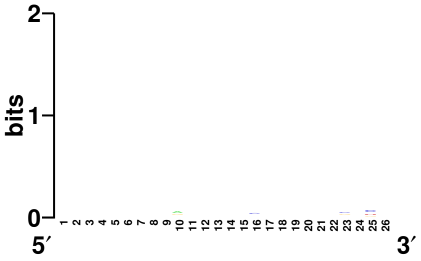

27-mers:

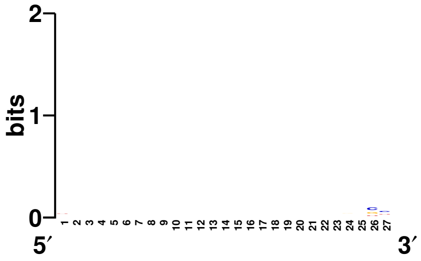

28-mers:

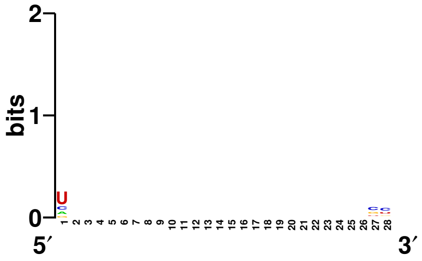

29-mers:

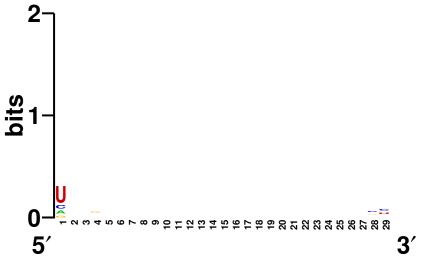

30-mers:

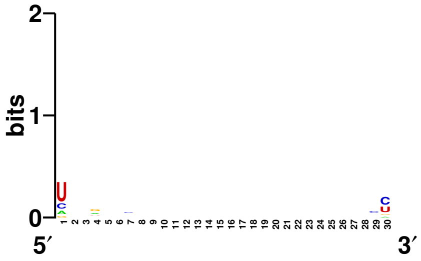

Antisense reads:

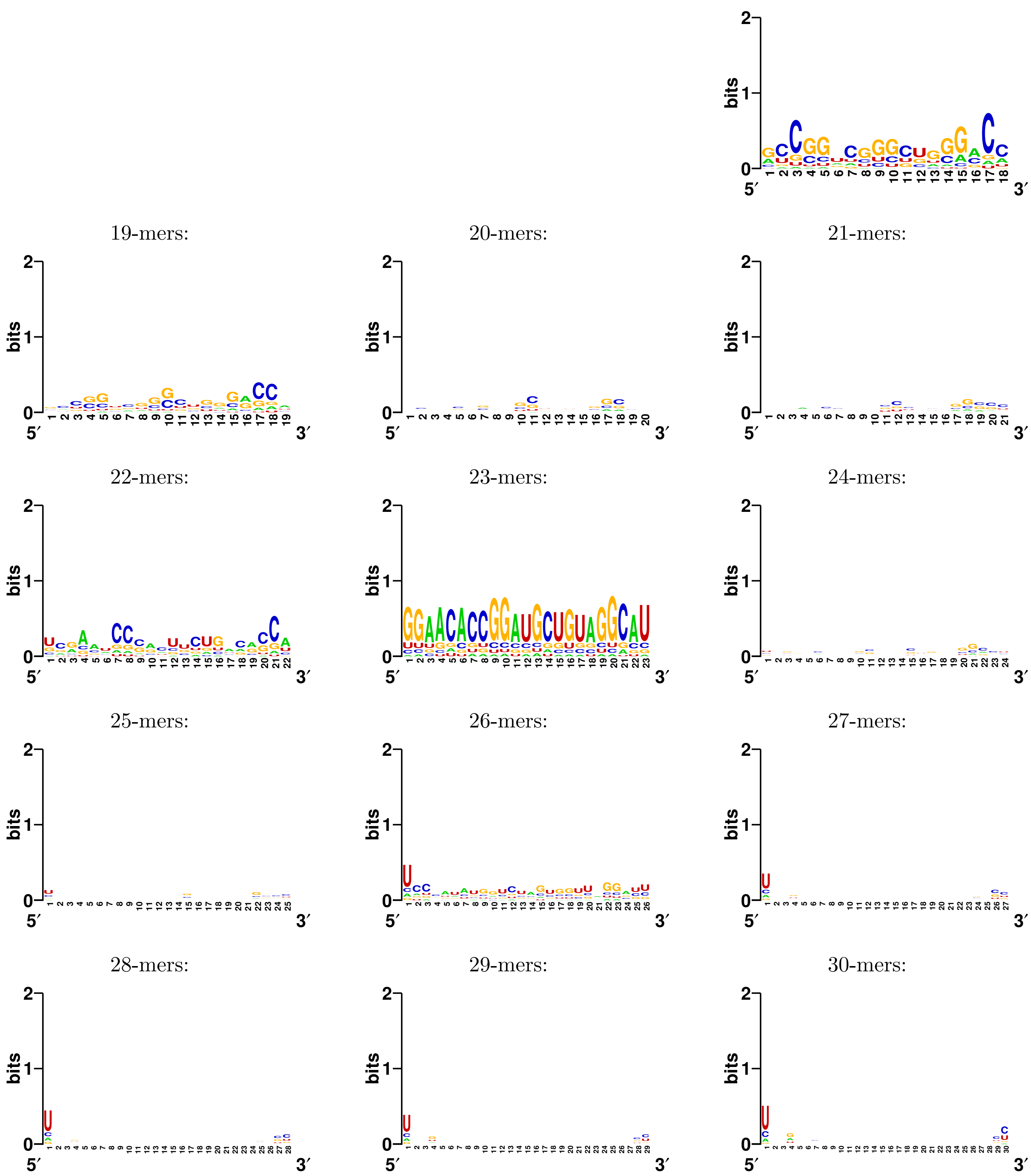

Adult female, library 1:

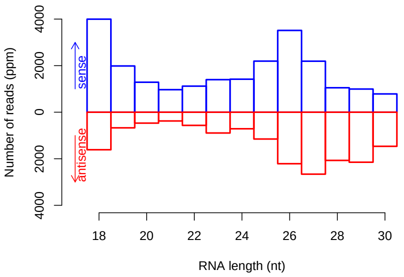

Sense reads:

18-mers:

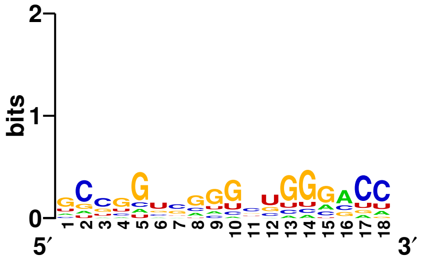

19-mers:

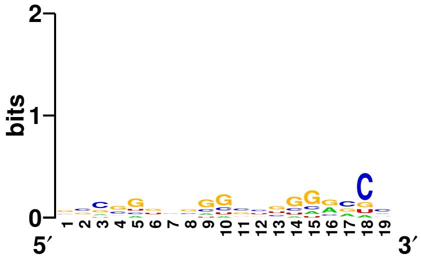

20-mers:

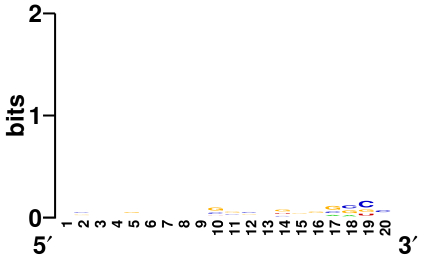

21-mers:

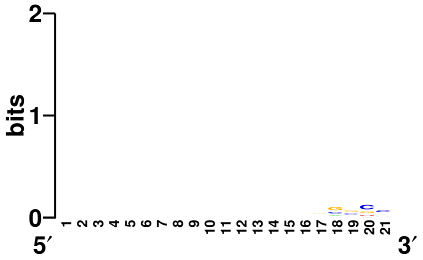

22-mers:

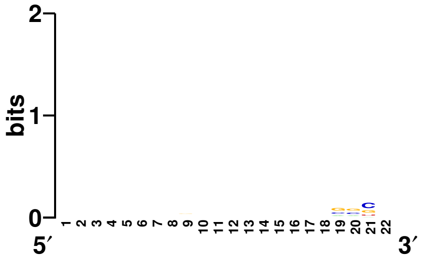

23-mers:

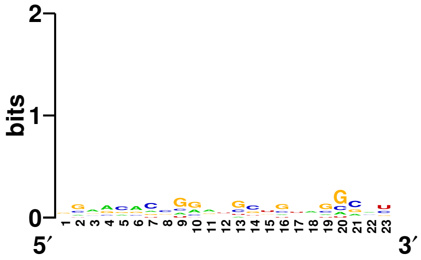

24-mers:

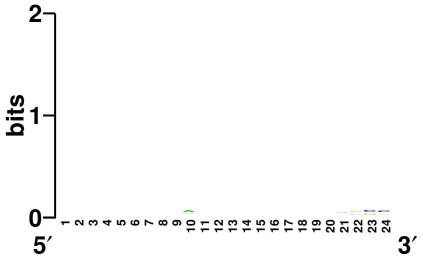

25-mers:

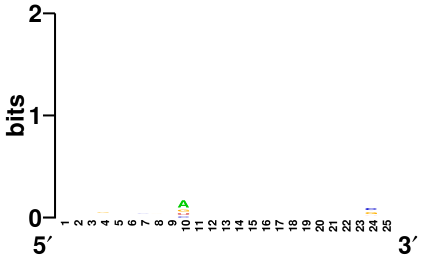

26-mers:

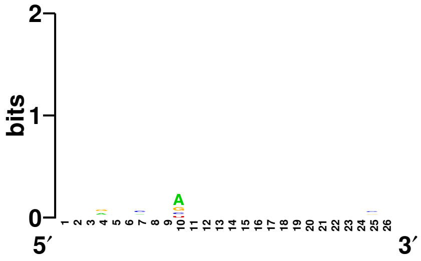

27-mers:

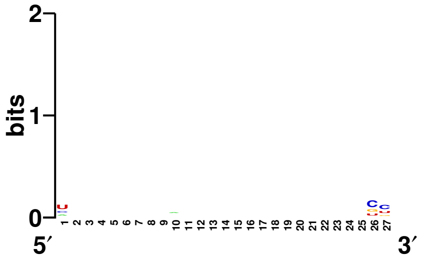

28-mers:

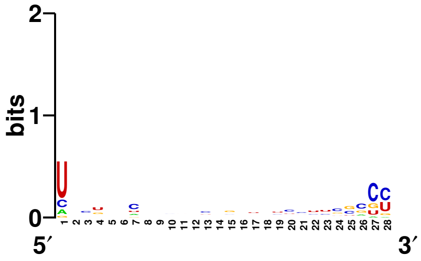

29-mers:

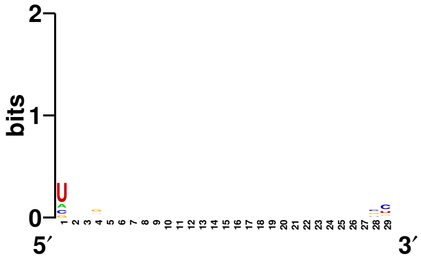

30-mers:

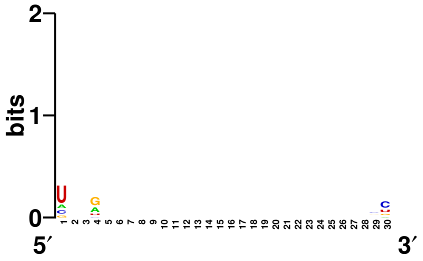

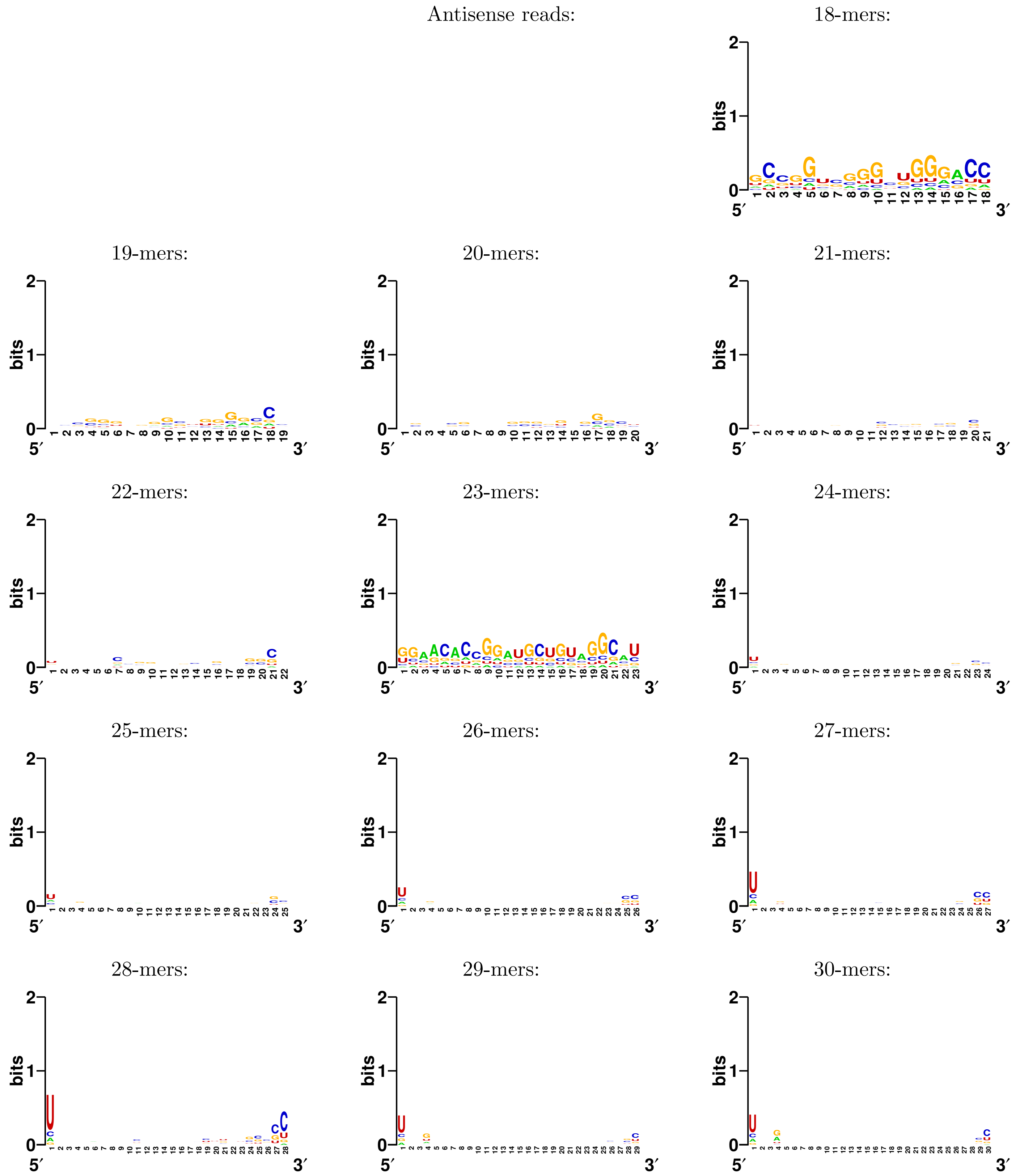
Antisense reads:
18-mers:
19-mers:
20-mers:
21-mers:
22-mers:
23-mers:
24-mers:
25-mers:
26-mers:
27-mers:
28-mers:
29-mers:
30-mers:
bits
5′
3′

Adult male, library 1:

Sense reads:

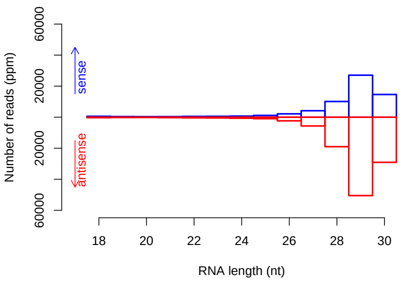

18-mers:

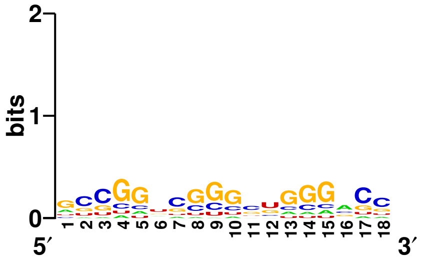

19-mers:

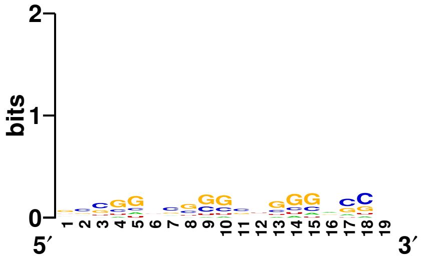

20-mers:

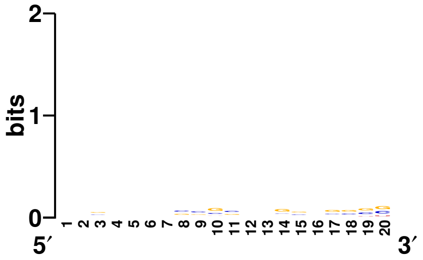

21-mers:

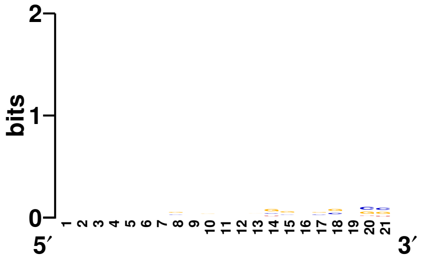

22-mers:

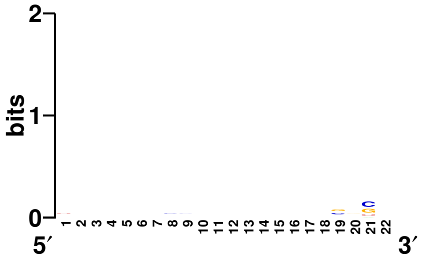

23-mers:

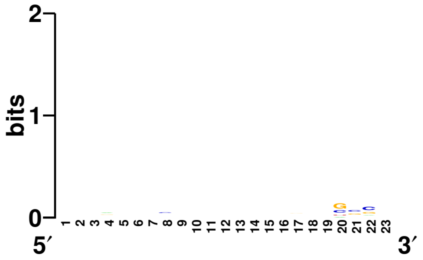

24-mers:

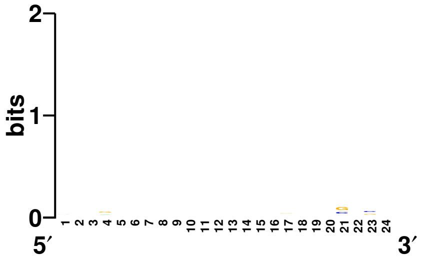

25-mers:

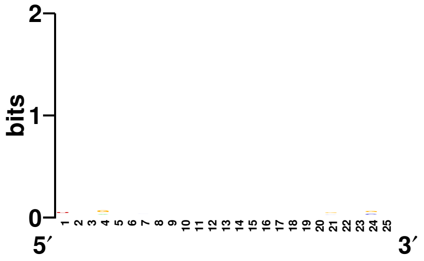

26-mers:

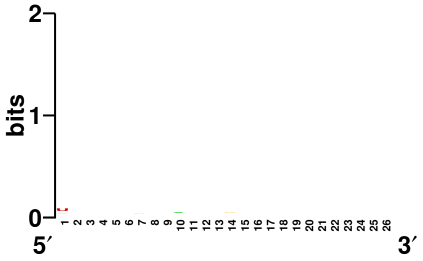

27-mers:

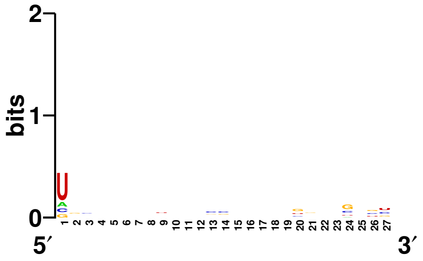

28-mers:

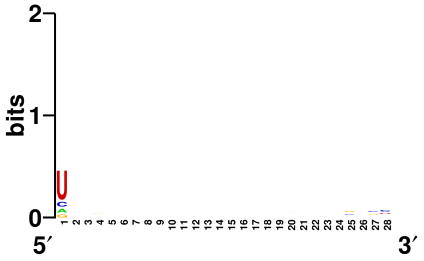

29-mers:

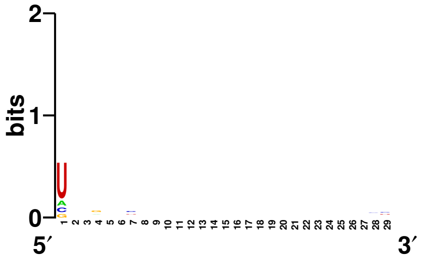

30-mers:

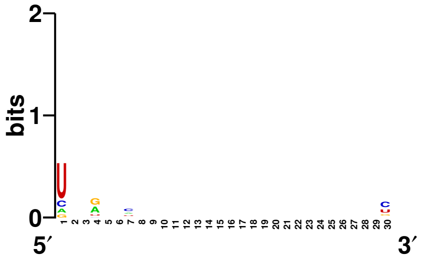

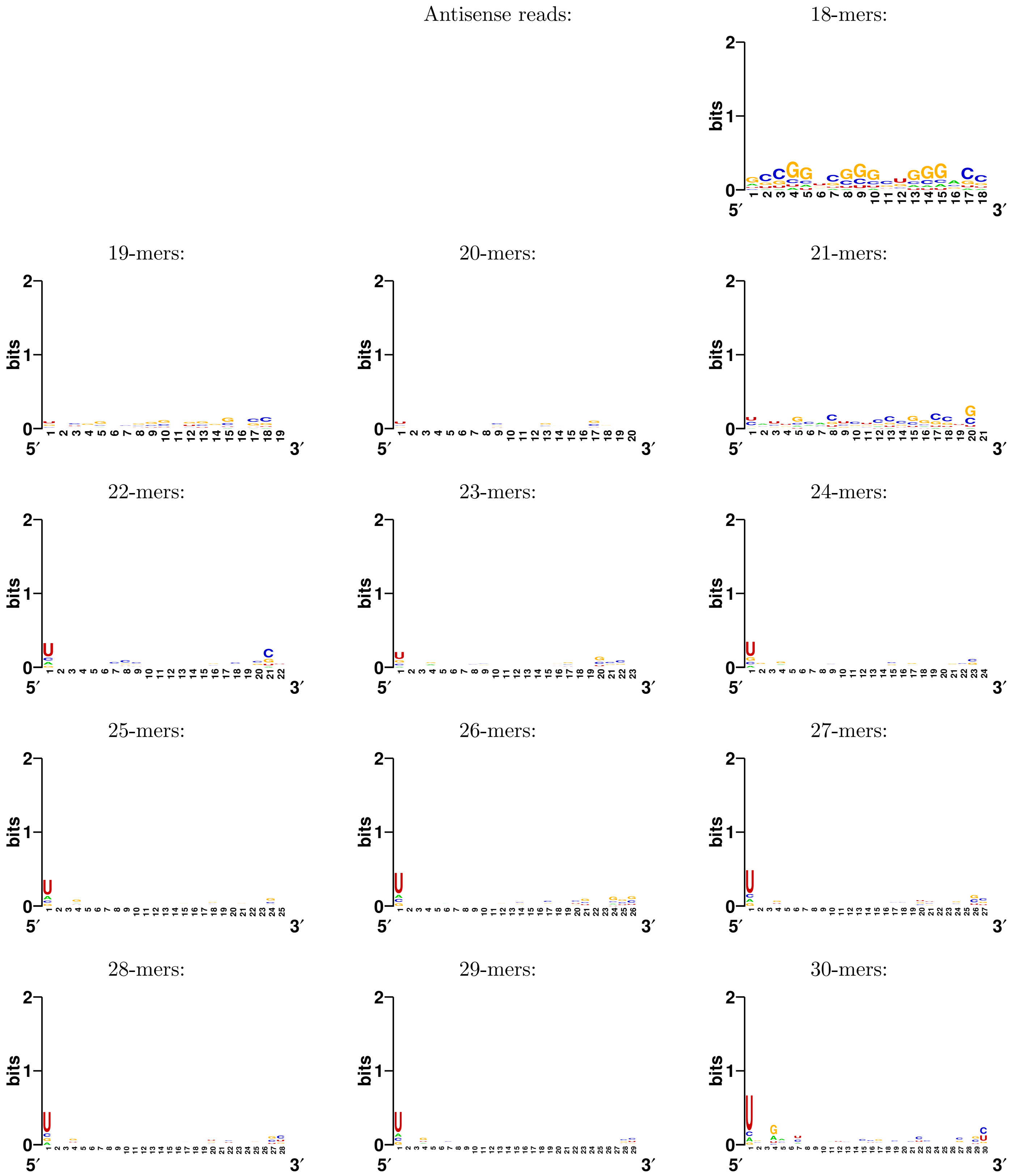
Antisense reads:
18-mers:
19-mers:
20-mers:
21-mers:
22-mers:
23-mers:
24-mers:
25-mers:
26-mers:
27-mers:
28-mers:
29-mers:
30-mers:
bits
5′
3′

3.2 Libraries #2 (3´ modified, 5´ monophosphorylated small RNAs)

Embryo 8h, library 2:

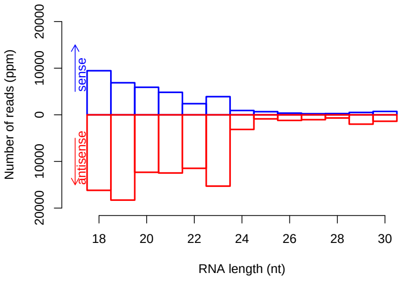

Sense reads:

18-mers:

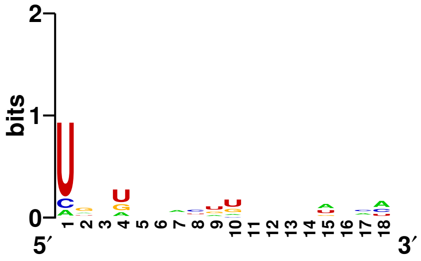

19-mers:

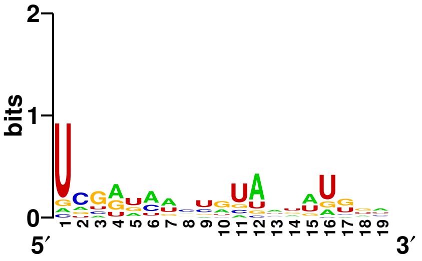

20-mers:

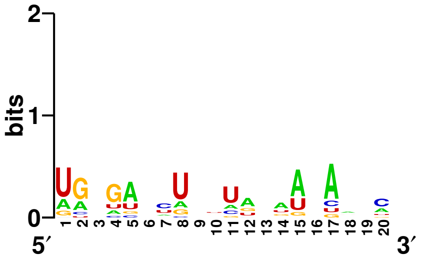

21-mers:

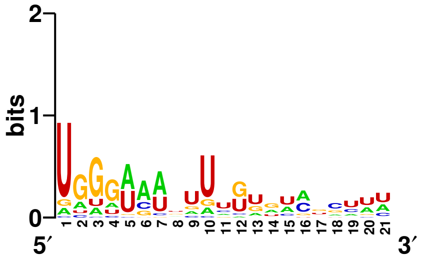

22-mers:

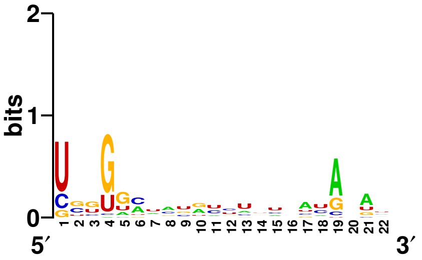

23-mers:

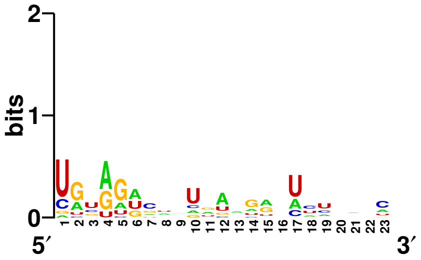

24-mers:

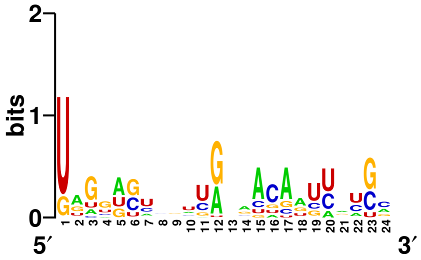

25-mers:

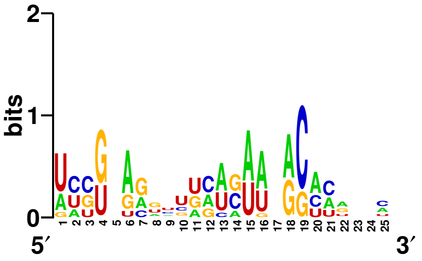

26-mers:

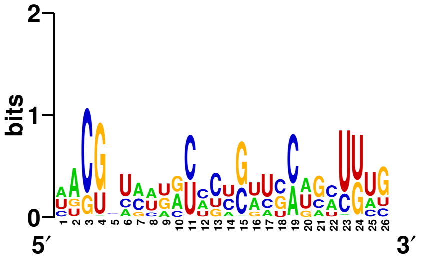

27-mers:

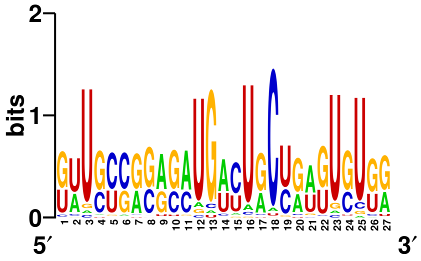

28-mers:

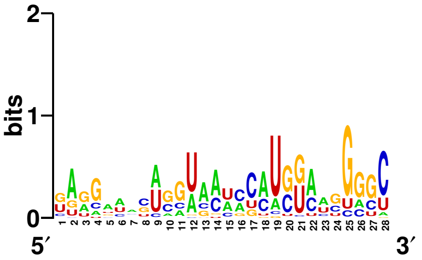

29-mers:

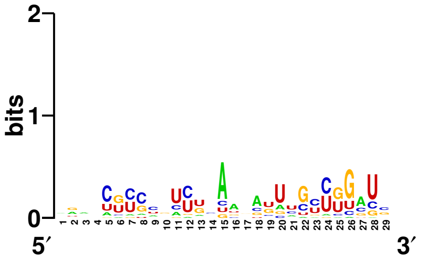

30-mers:

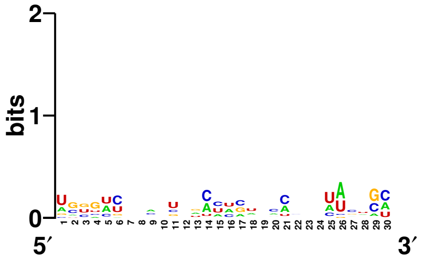

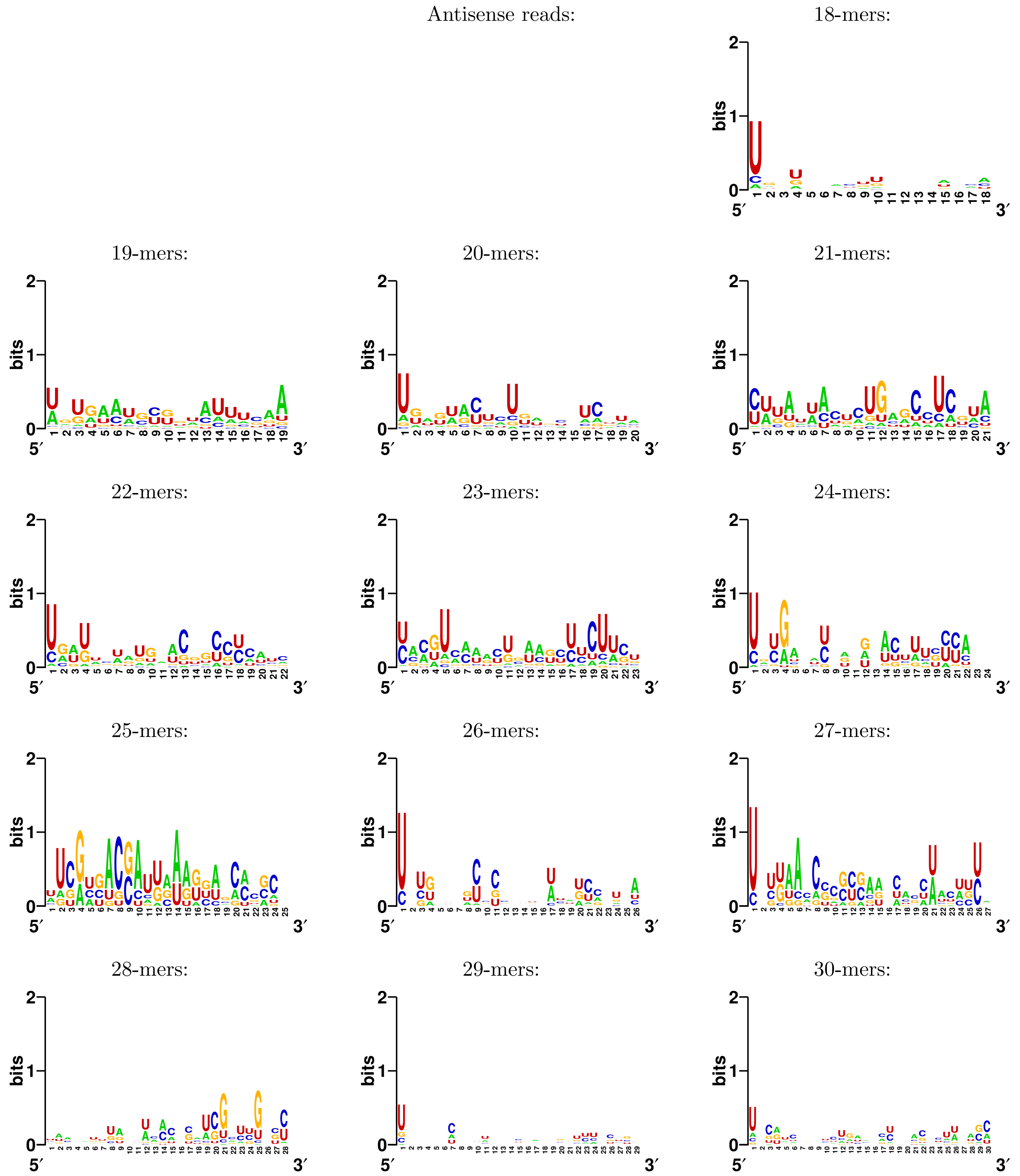
Antisense reads:
18-mers:
19-mers:
20-mers:
21-mers:
22-mers:
23-mers:
24-mers:
25-mers:
26-mers:
27-mers:
28-mers:
29-mers:
30-mers:
bits
5′
3′

Embryo 15h, library 2:

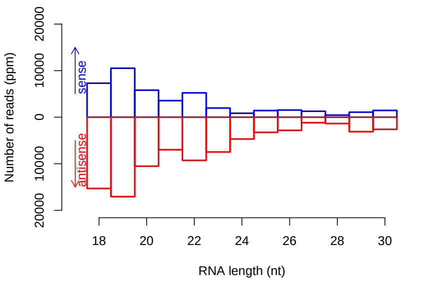

Sense reads:

18-mers:

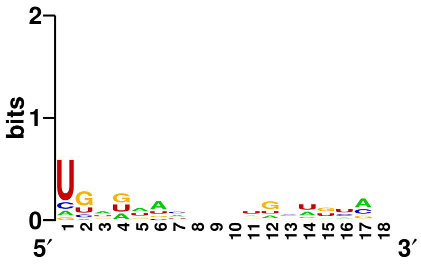

19-mers:

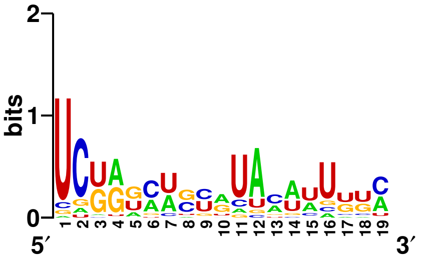

20-mers:

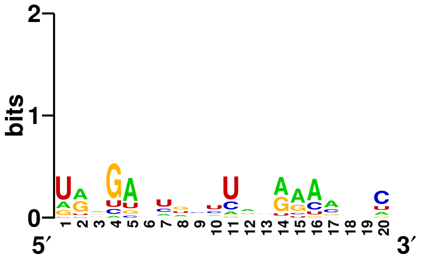

21-mers:

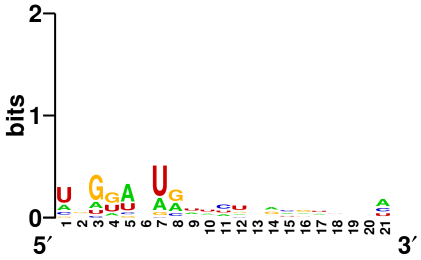

22-mers:

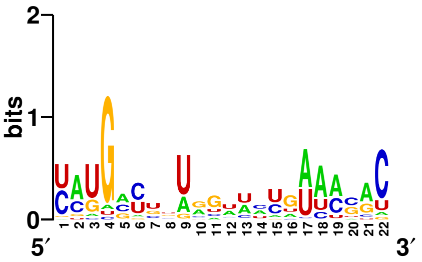

23-mers:

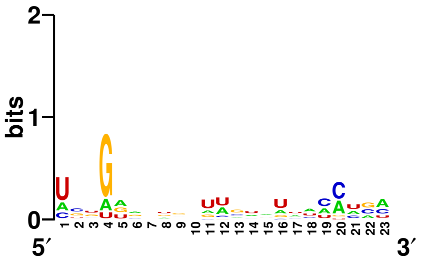

24-mers:

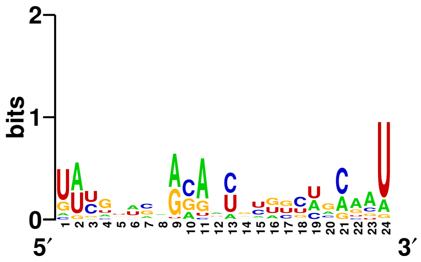

25-mers:

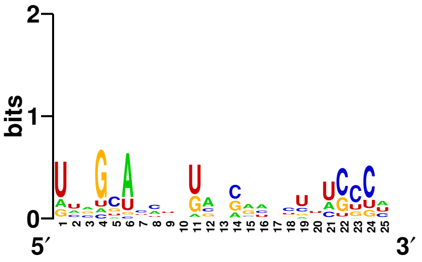

26-mers:

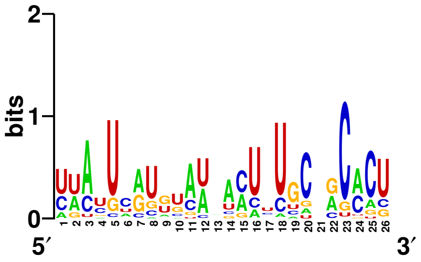

27-mers:

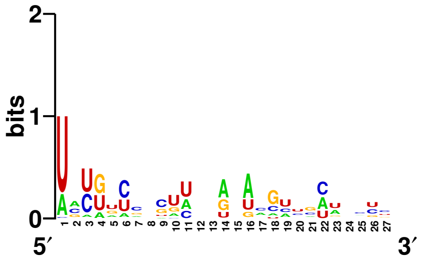

28-mers:

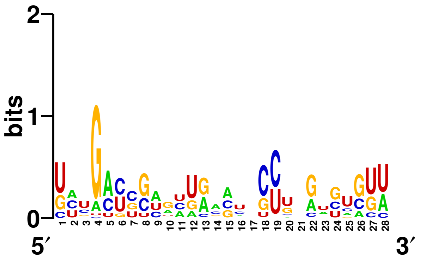

29-mers:

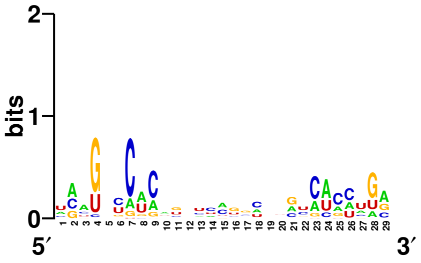

30-mers:

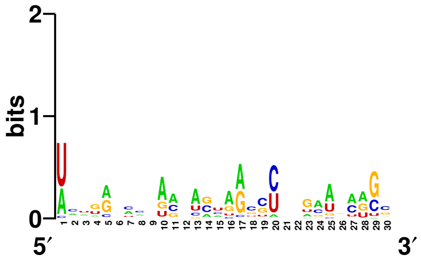

Antisense reads:

18-mers:

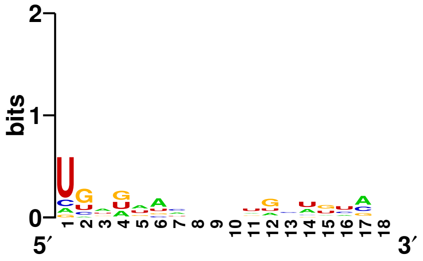

19-mers:

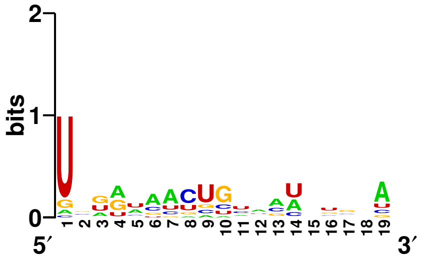

20-mers:

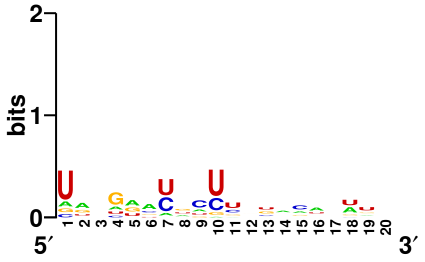

21-mers:

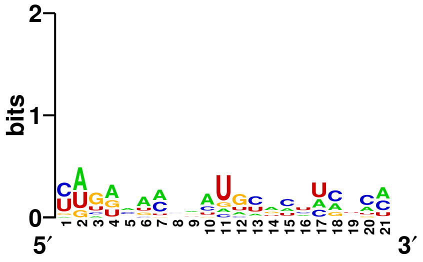

22-mers:

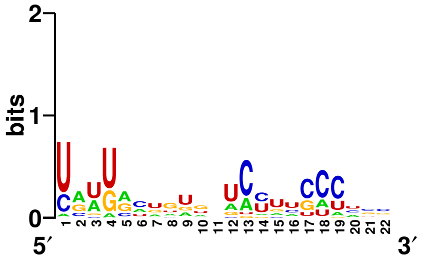

23-mers:

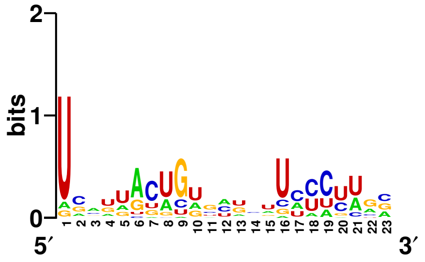

24-mers:

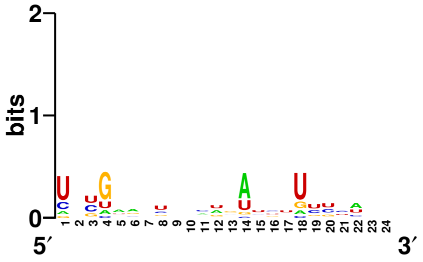

25-mers:

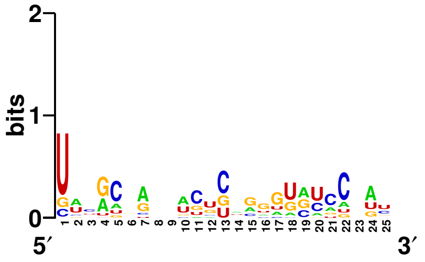

26-mers:

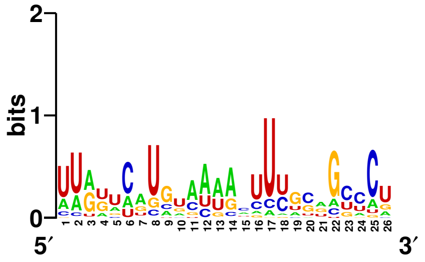

27-mers:

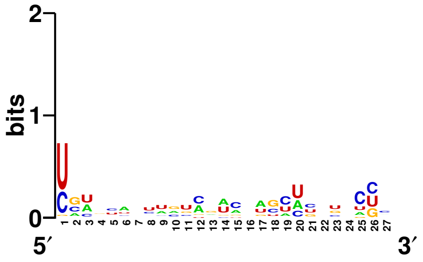

28-mers:

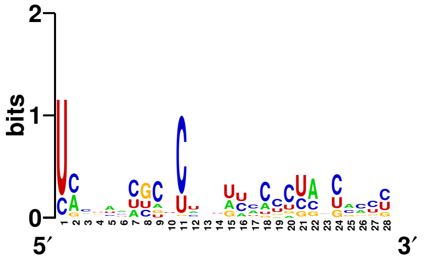

29-mers:

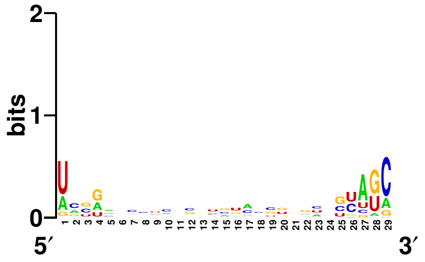

30-mers:

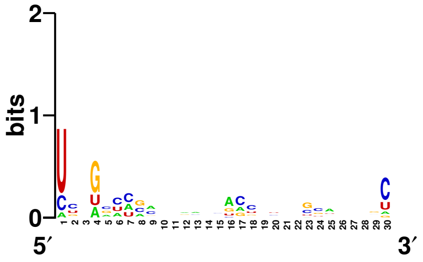

Embryo 36h, library 2:

Sense reads:

18-mers:

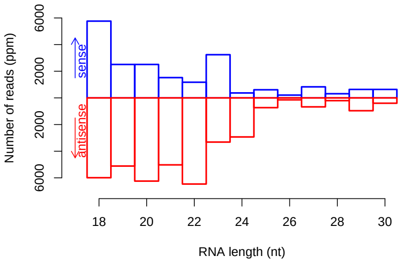

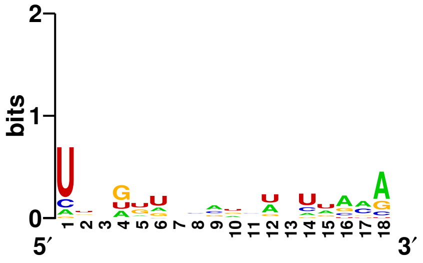

19-mers:

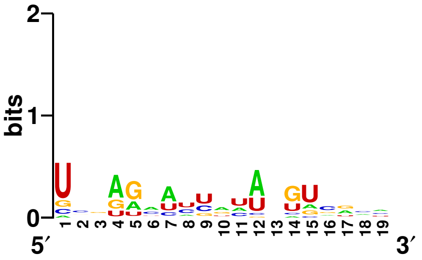

20-mers:

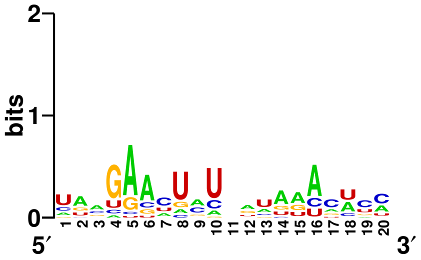

21-mers:

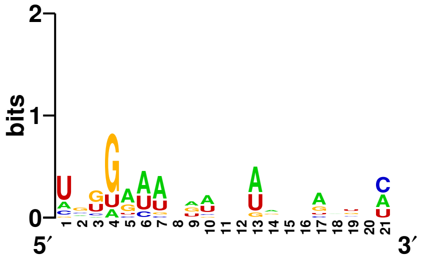

22-mers:

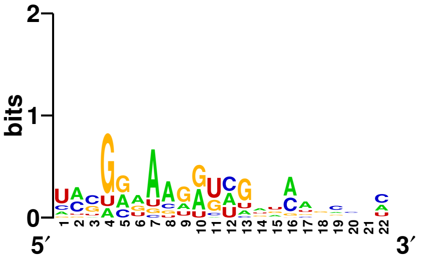

23-mers:

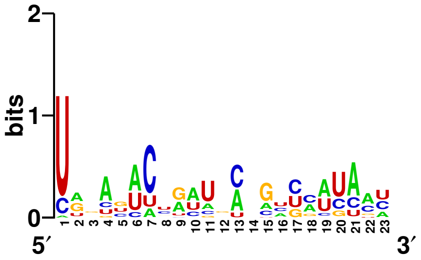

24-mers:

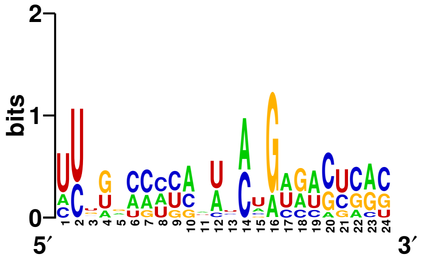

25-mers:

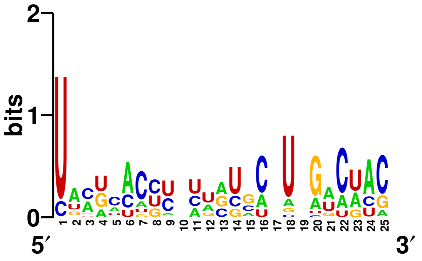

26-mers:

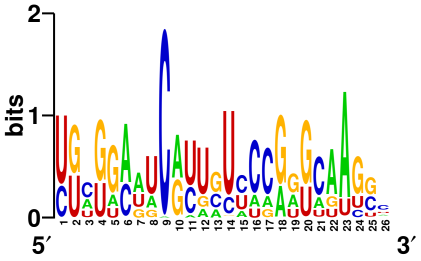

27-mers:

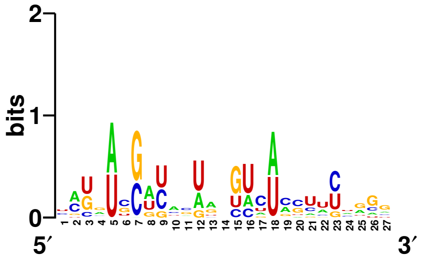

28-mers:

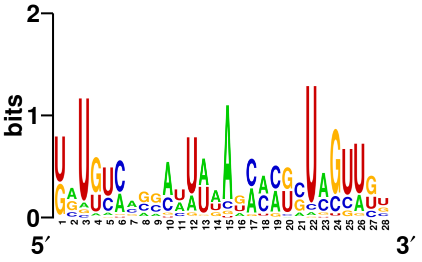

29-mers:

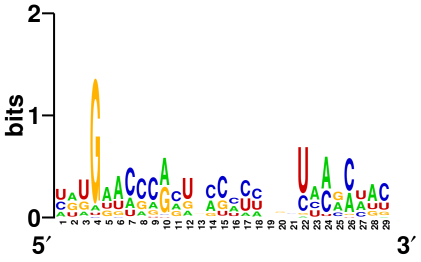

30-mers:

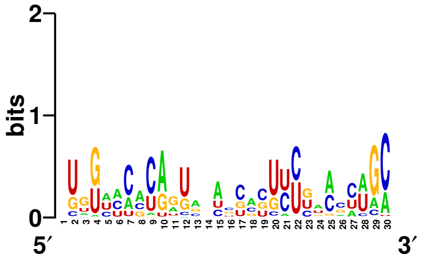

Antisense reads:

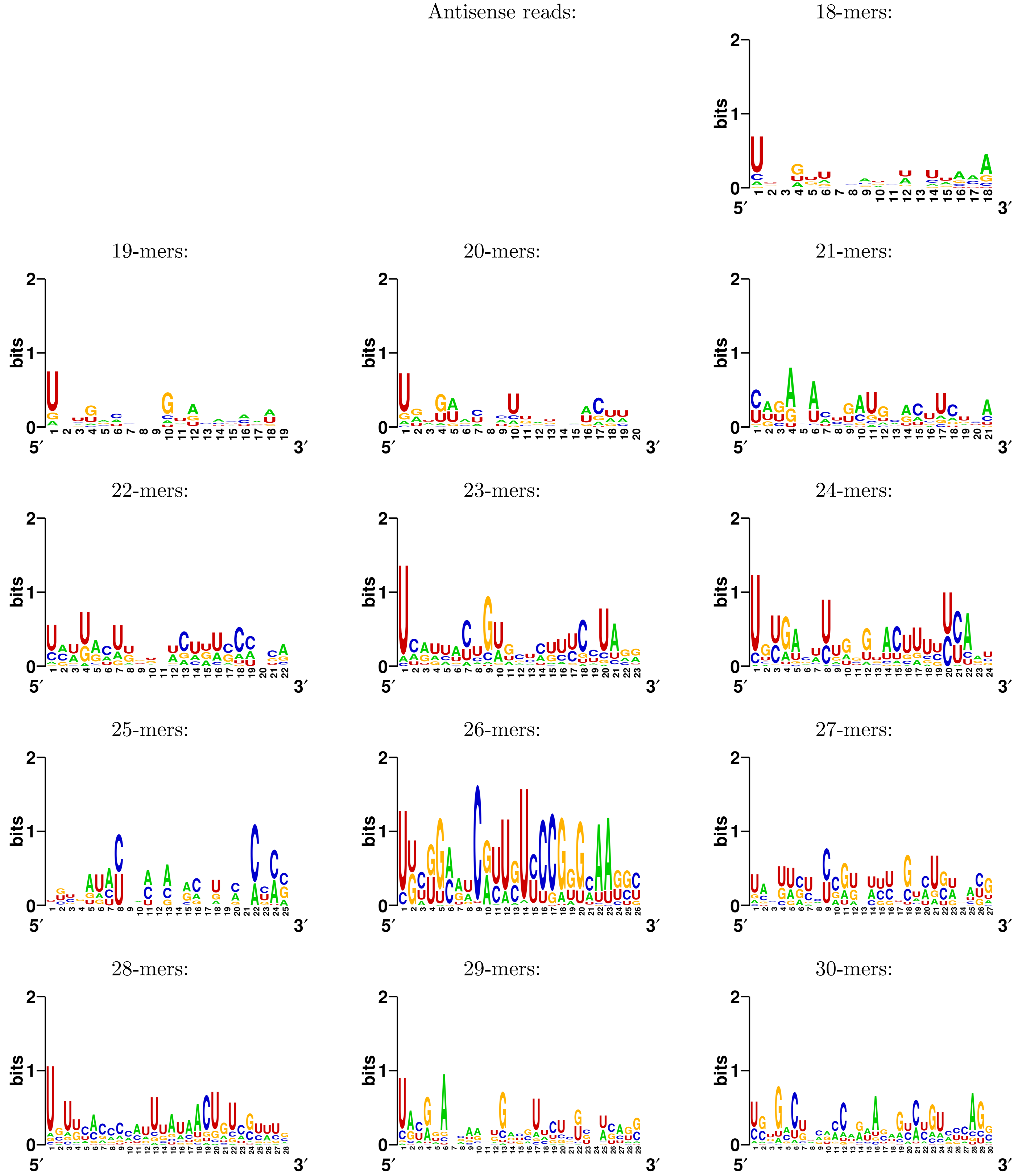

Embryo 60h, library 2:

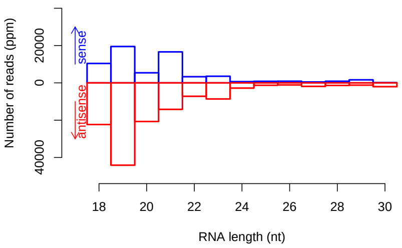

Sense reads:

18-mers:

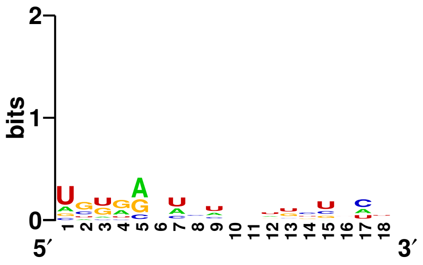

19-mers:

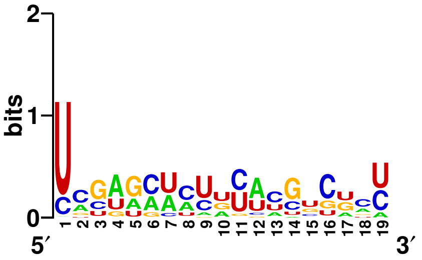

20-mers:

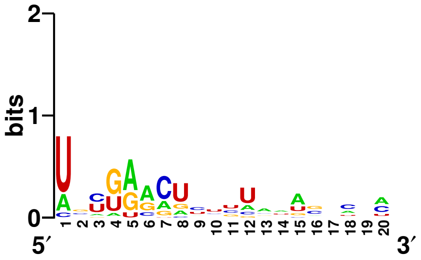

21-mers:

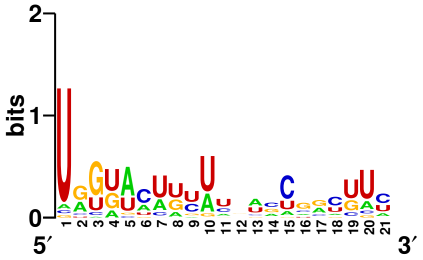

22-mers:

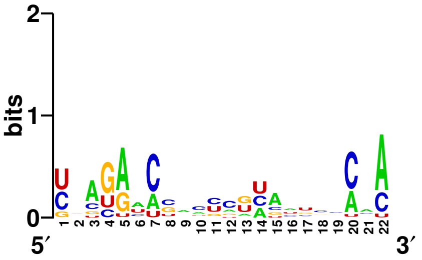

23-mers:

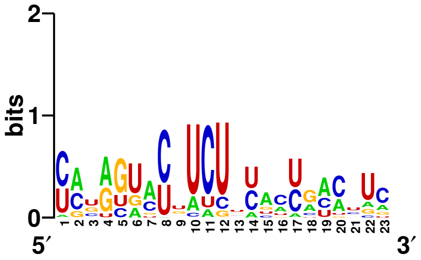

24-mers:

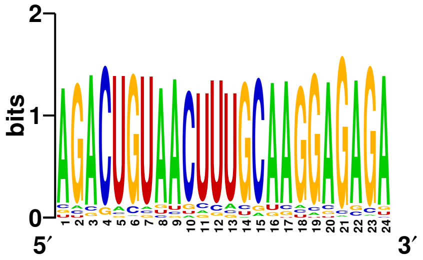

25-mers:

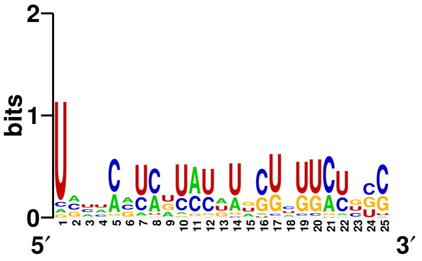

26-mers:

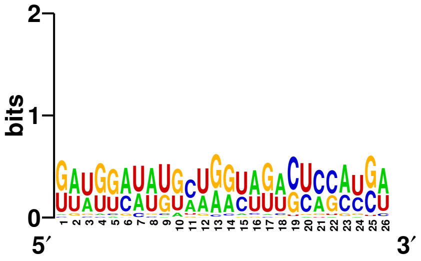

27-mers:

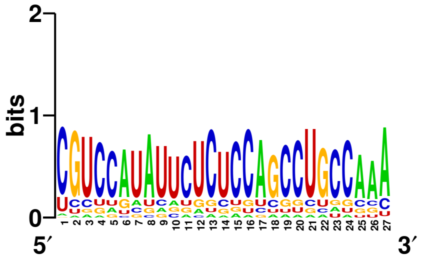

28-mers:

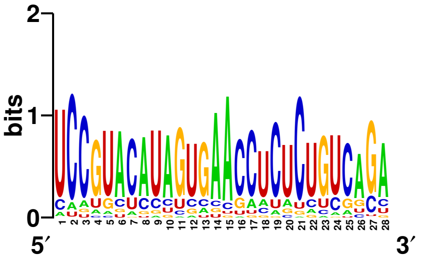

29-mers:

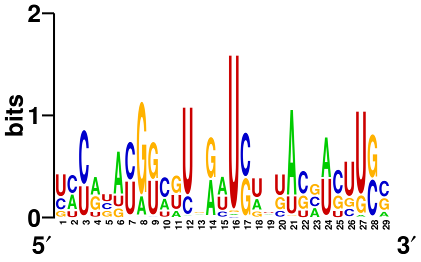

30-mers:

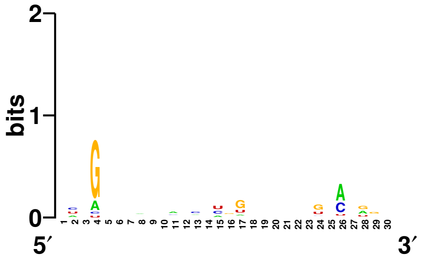

Antisense reads:

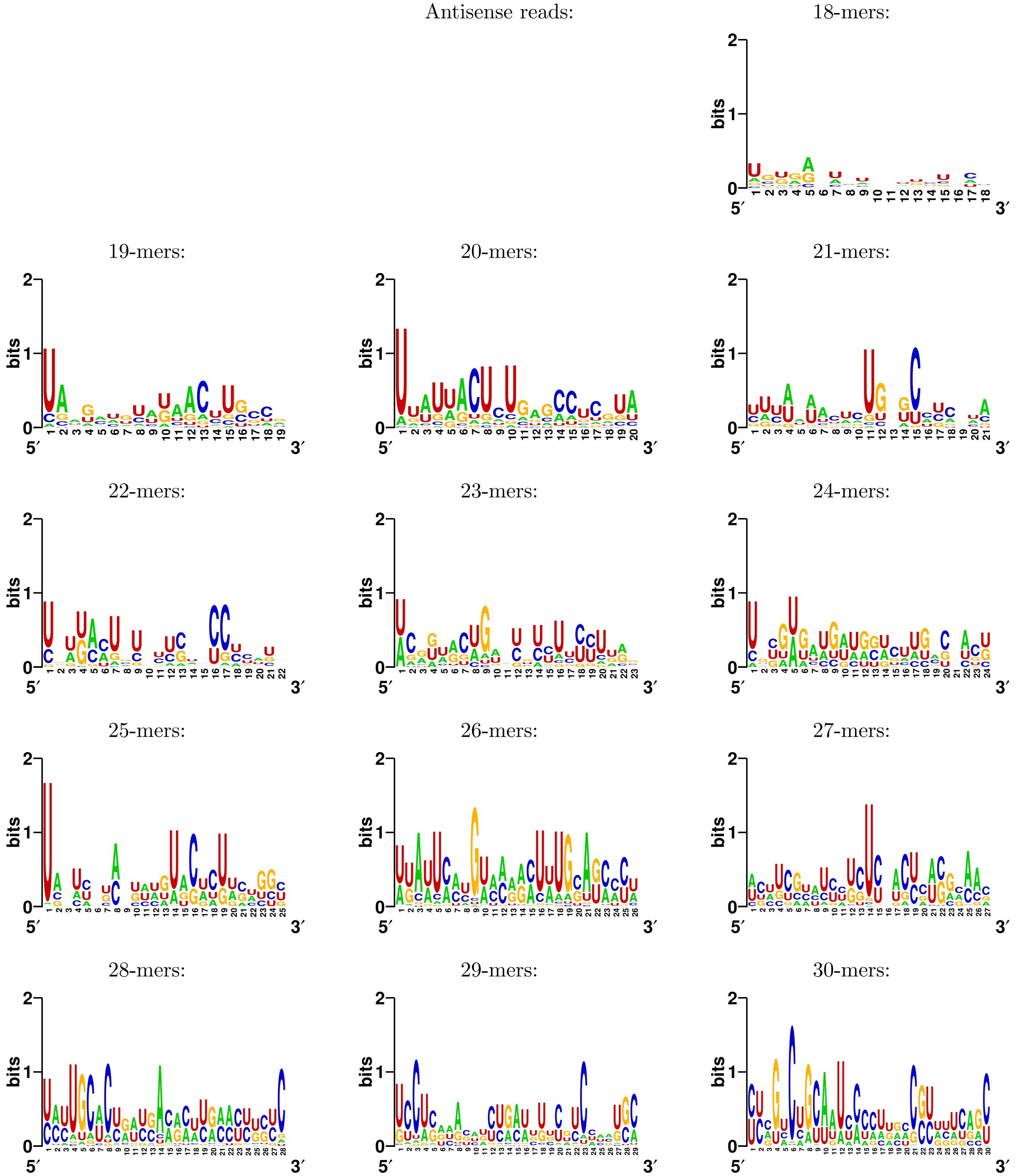

Adult female, library 2:

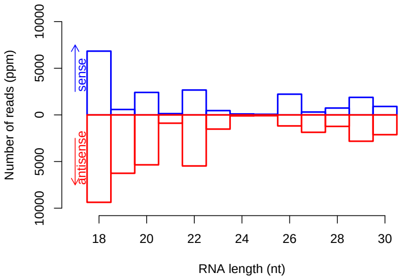

Sense reads:

18-mers:

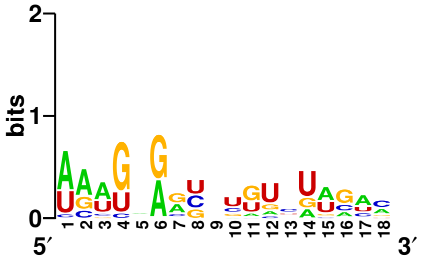

19-mers:

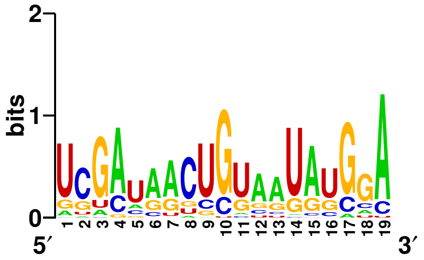

20-mers:

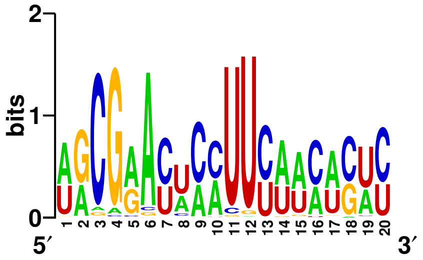

21-mers:

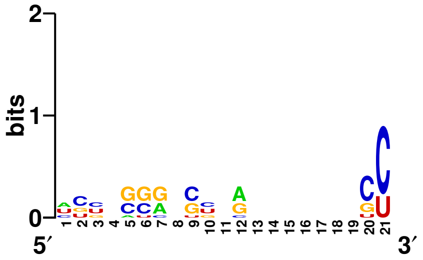

22-mers:

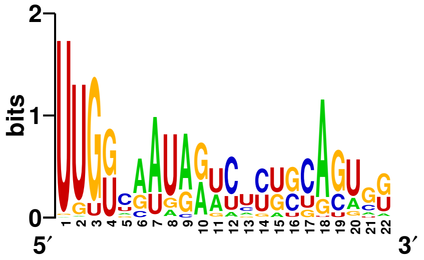

23-mers:

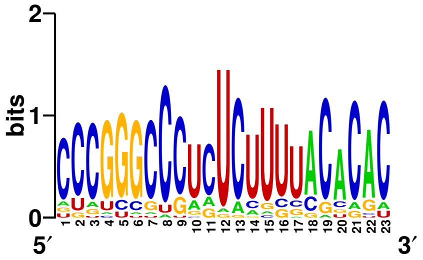

24-mers:

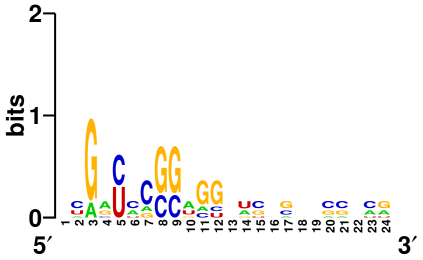

25-mers:

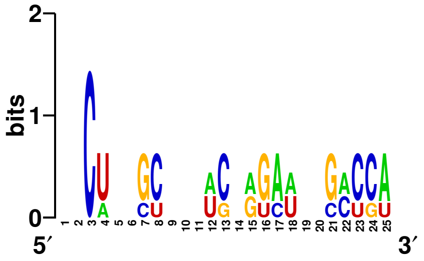

26-mers:

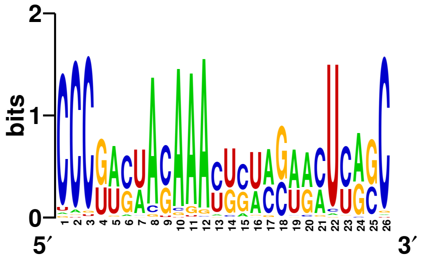

27-mers:

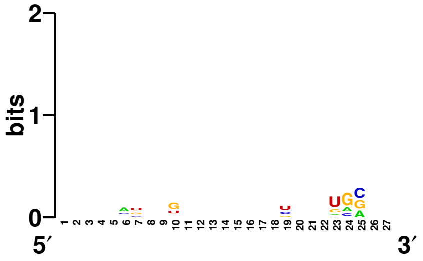

28-mers:

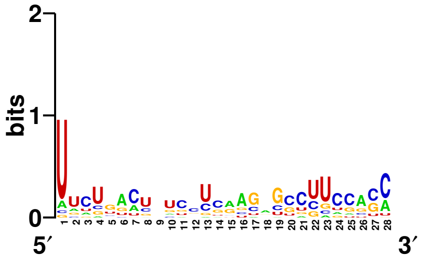

29-mers:

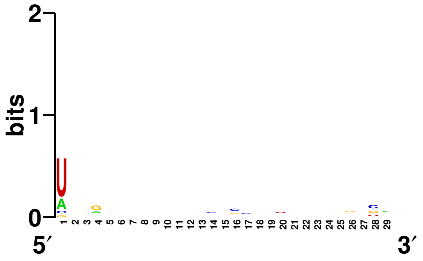

30-mers:

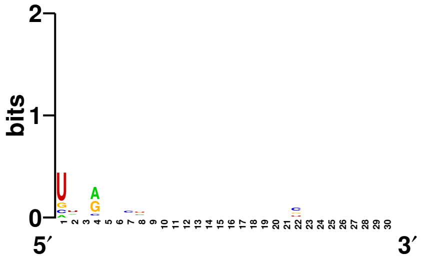

Antisense reads:

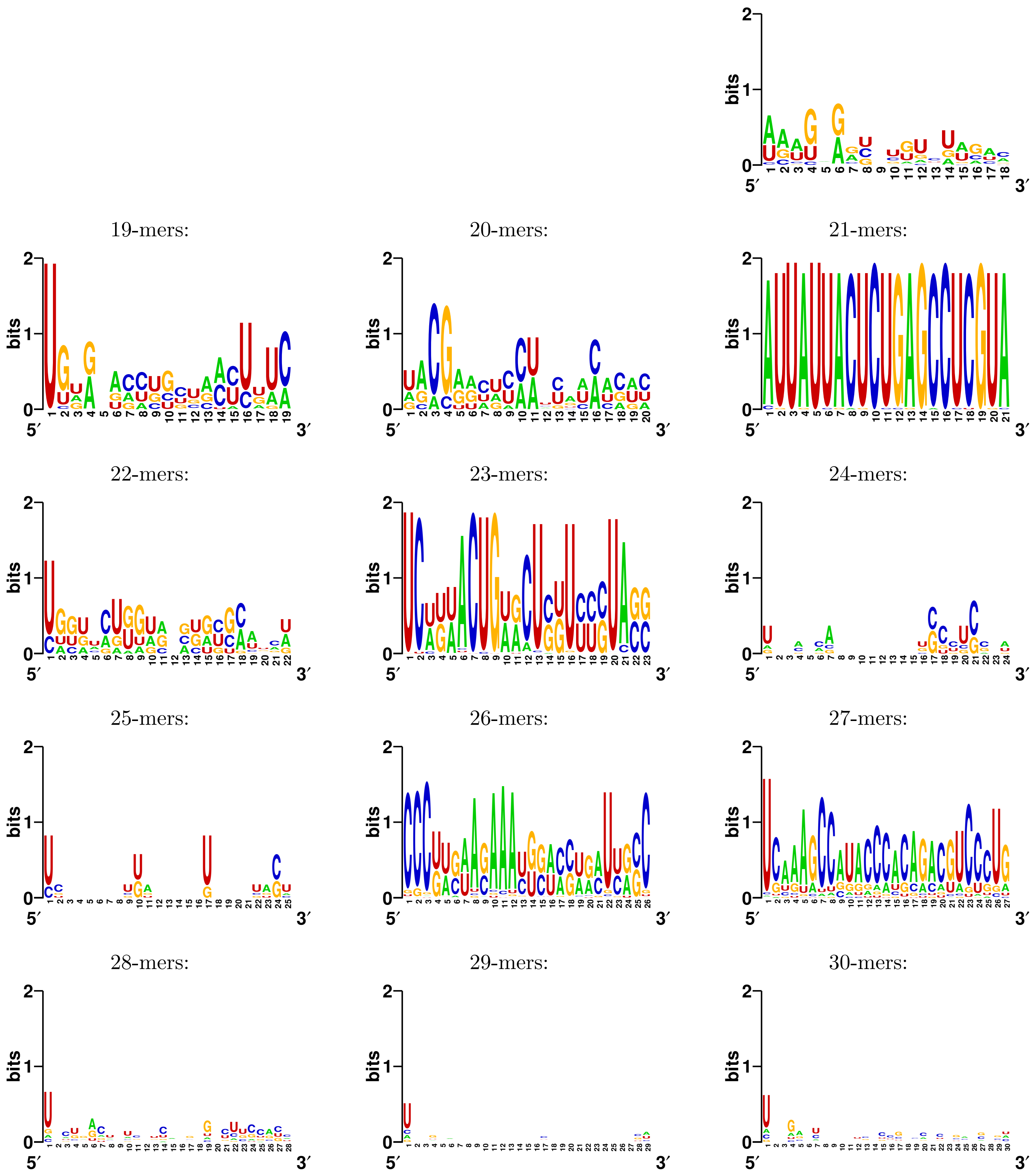

Adult male, library 2:

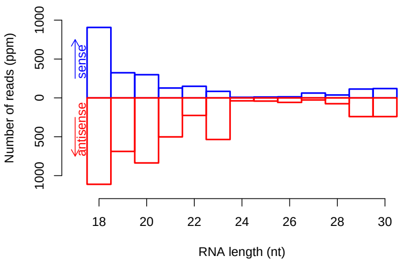

Sense reads:

18-mers:

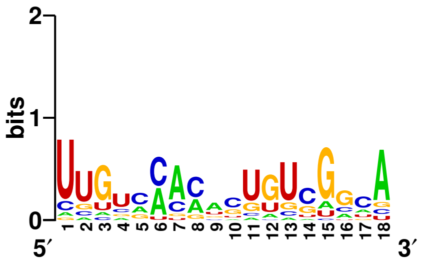

19-mers:

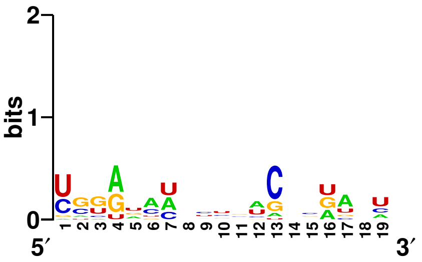

20-mers:

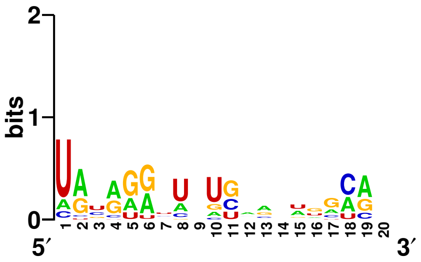

21-mers:

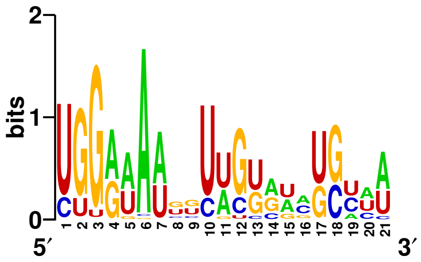

22-mers:

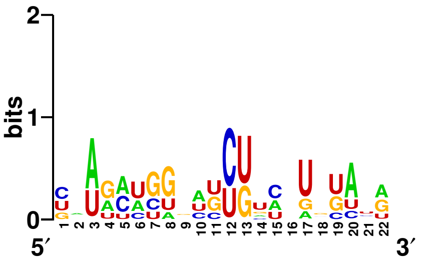

23-mers:

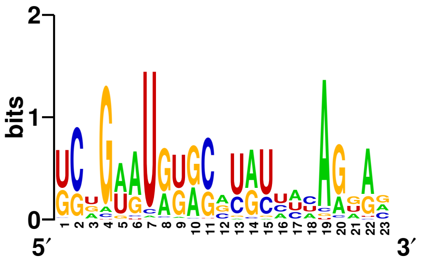

24-mers:

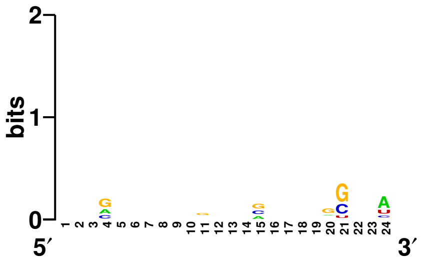

25-mers:

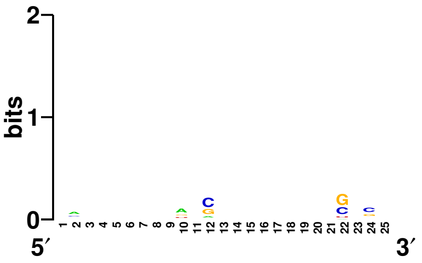

26-mers:

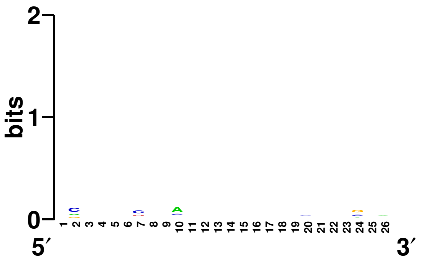

27-mers:

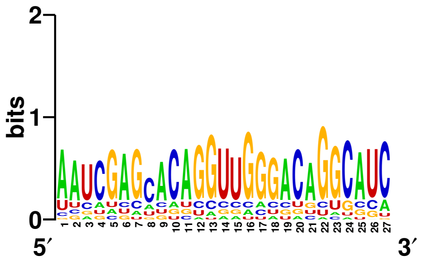

28-mers:

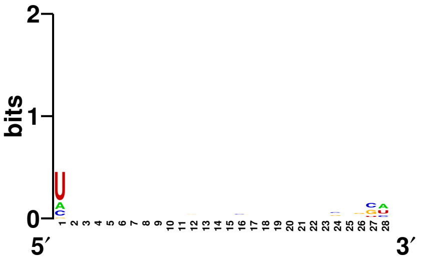

29-mers:

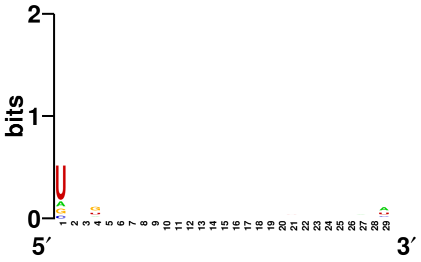

30-mers:

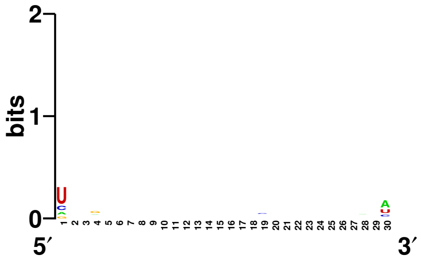

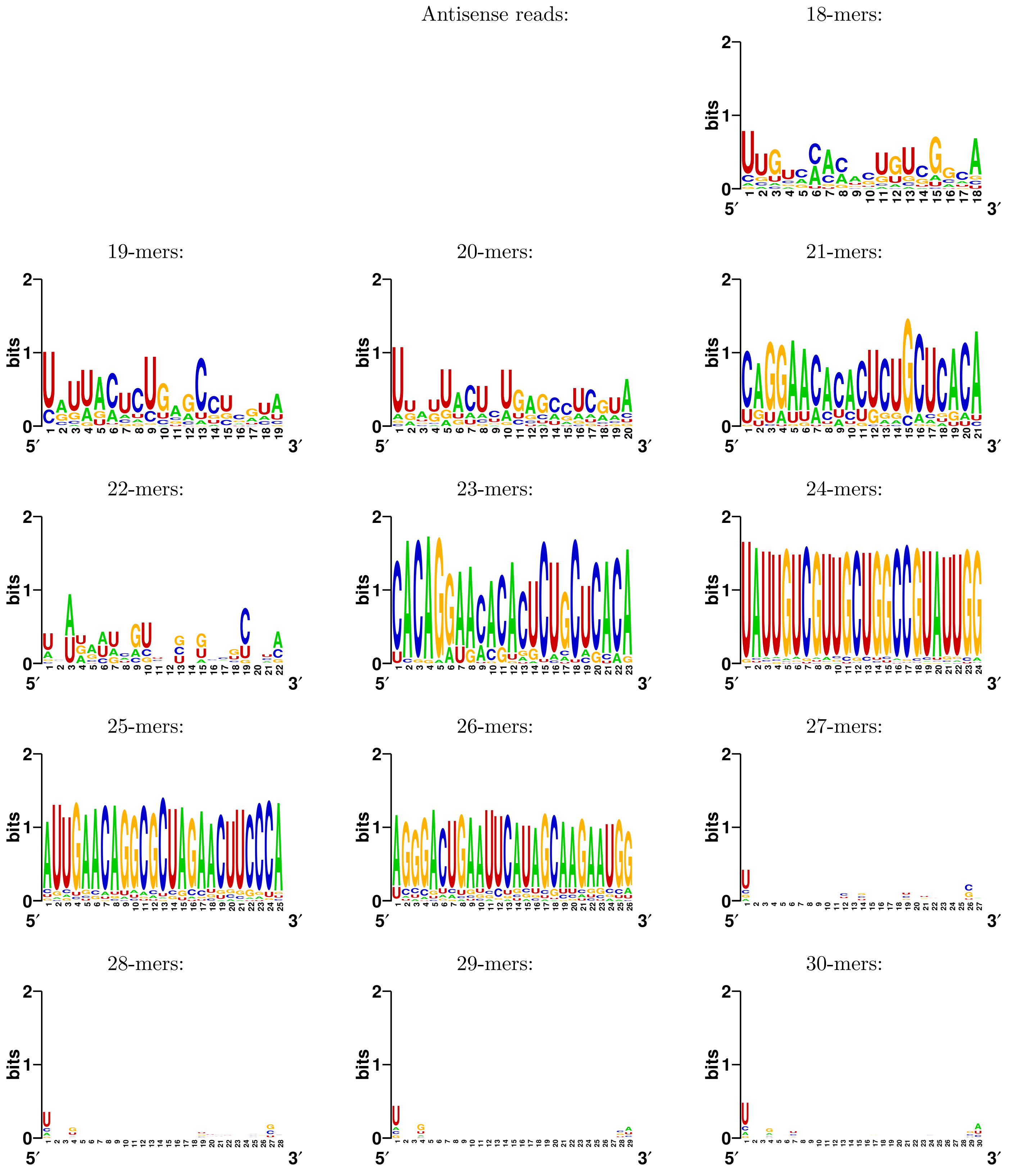
Antisense reads:
18-mers:
19-mers:
20-mers:
21-mers:
22-mers:
23-mers:
24-mers:
25-mers:
26-mers:
27-mers:
28-mers:
29-mers:
30-mers:
bits
5′
3′

3.3 Libraries #3 (total 5´ hydroxyl or polyphosphorylated small RNAs)

Embryo 8h, library 3:

Sense reads:

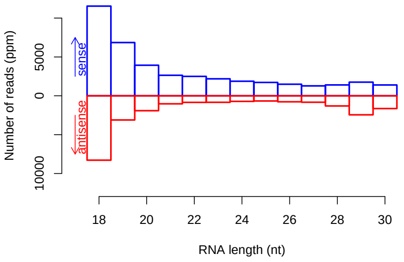

18-mers:

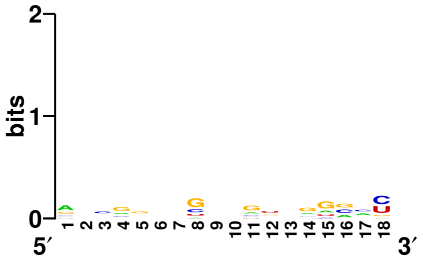

19-mers:

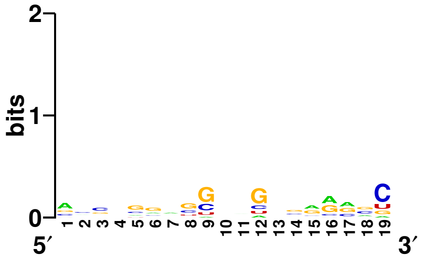

20-mers:

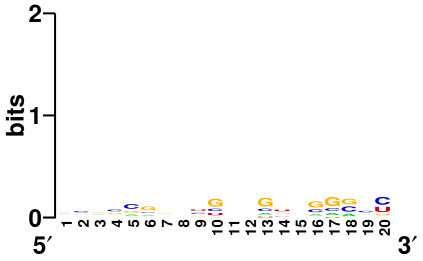

21-mers:

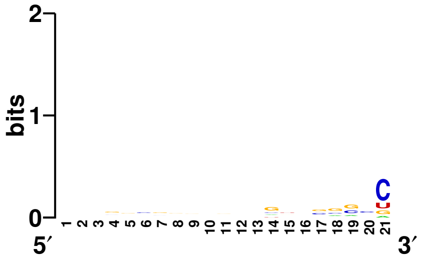

22-mers:

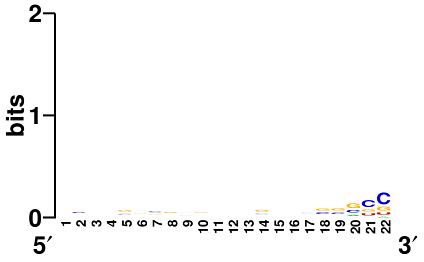

23-mers:

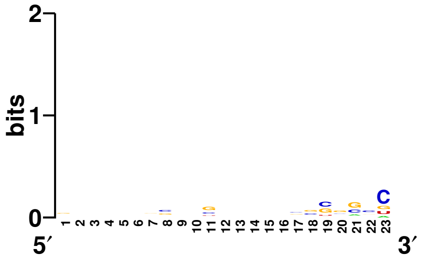

24-mers:

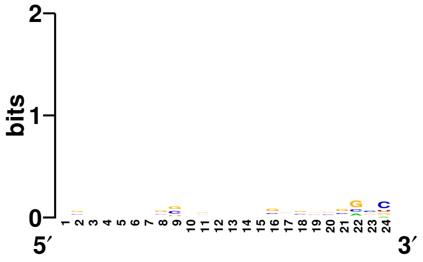

25-mers:

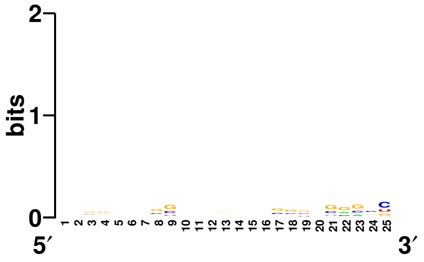

26-mers:

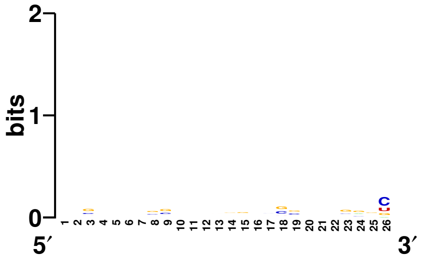

27-mers:

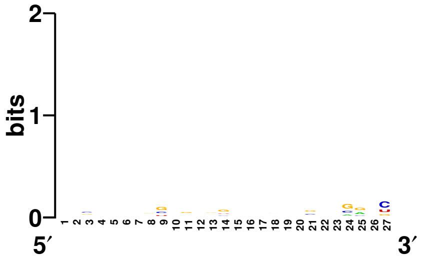

28-mers:

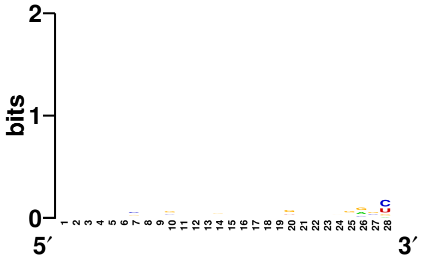

29-mers:

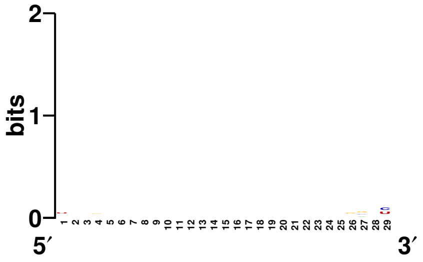

30-mers:

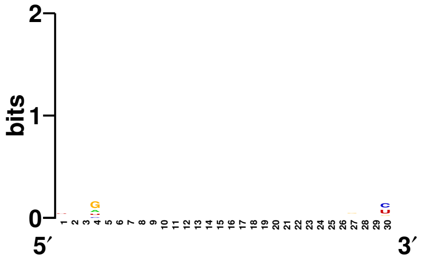

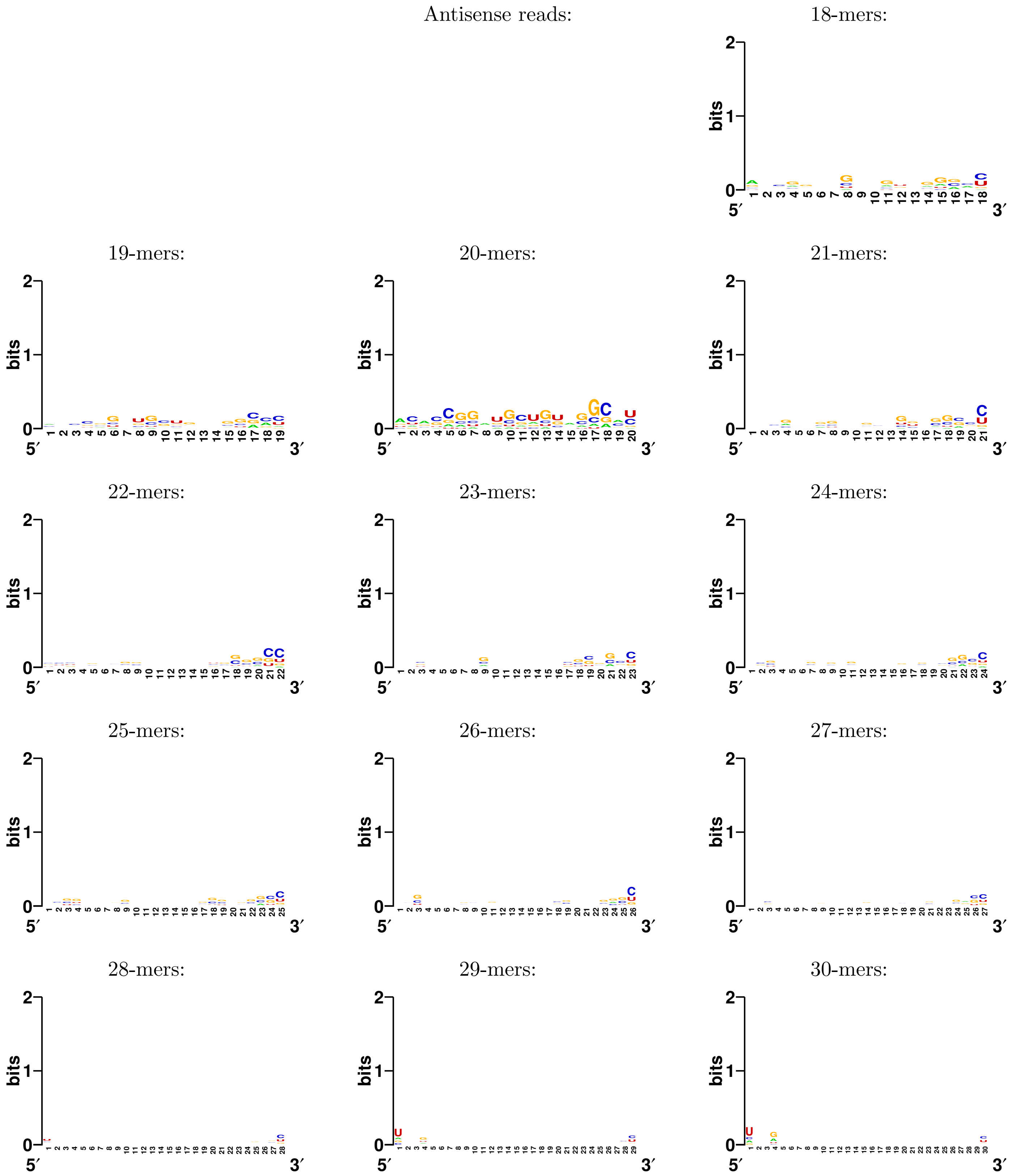
Antisense reads:
18-mers:
19-mers:
20-mers:
21-mers:
22-mers:
23-mers:
24-mers:
25-mers:
26-mers:
27-mers:
28-mers:
29-mers:
30-mers:
bits
5′
3′

Embryo 15h, library 3:

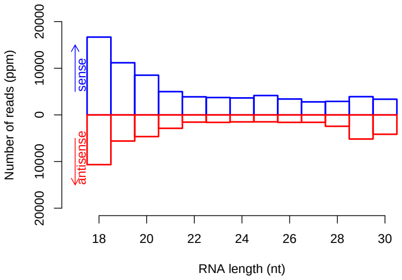

Sense reads:

18-mers:

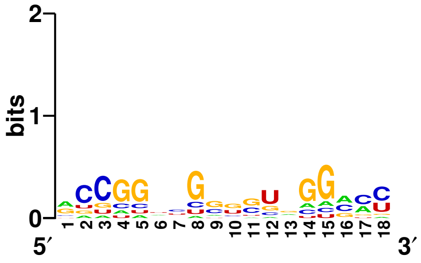

19-mers:

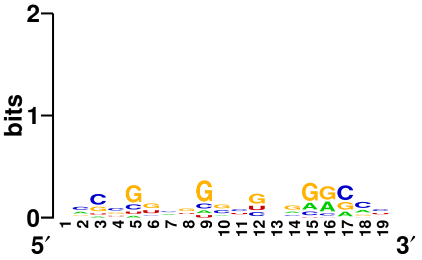

20-mers:

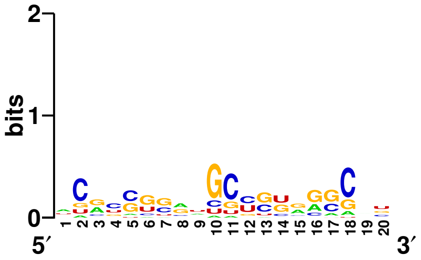

21-mers:

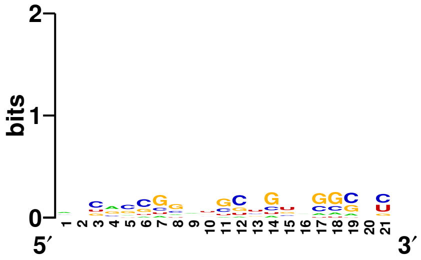

22-mers:

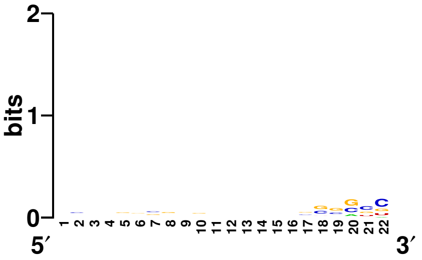

23-mers:

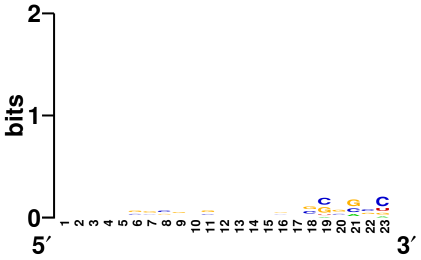

24-mers:

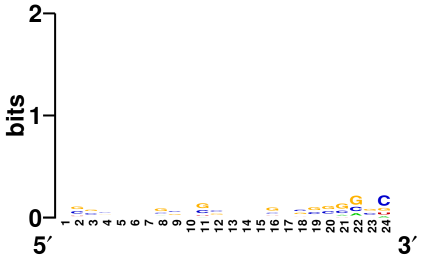

25-mers:

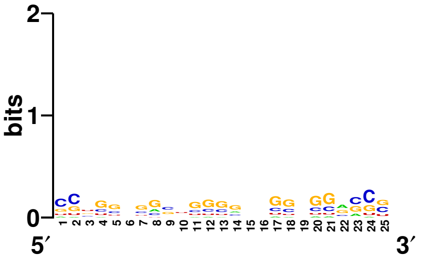

26-mers:

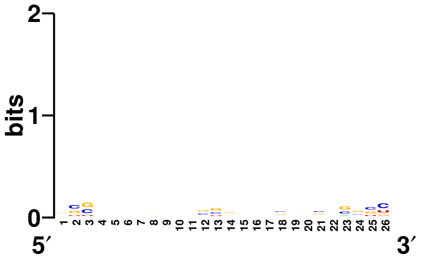

27-mers:

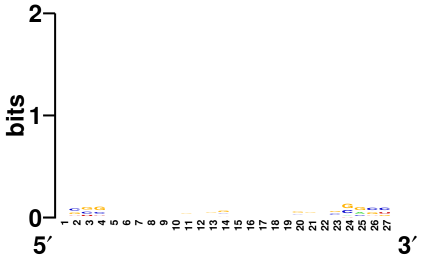

28-mers:

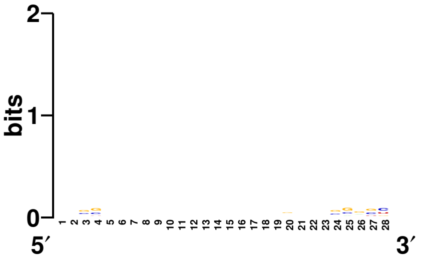

29-mers:

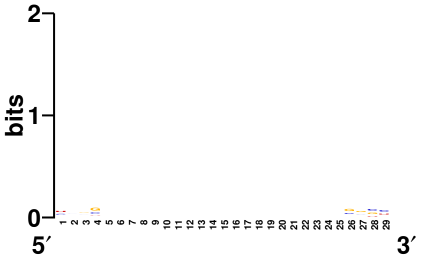

30-mers:

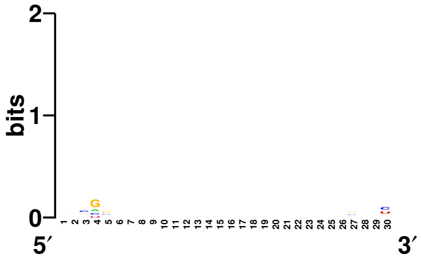

Antisense reads:

18-mers:

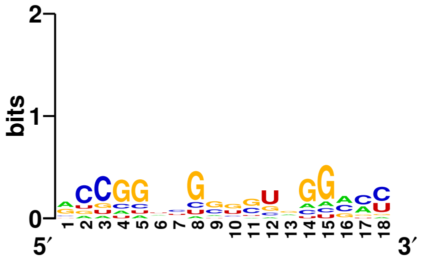

19-mers:

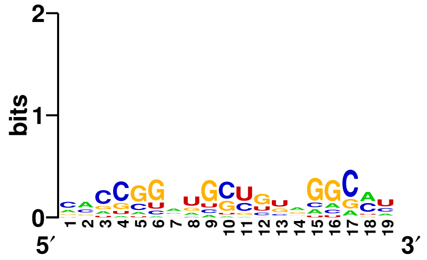

20-mers:

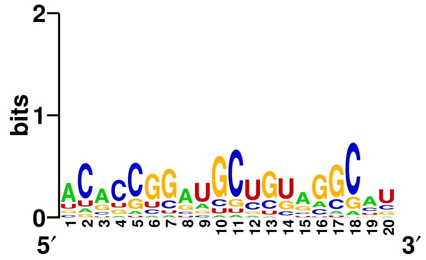

21-mers:

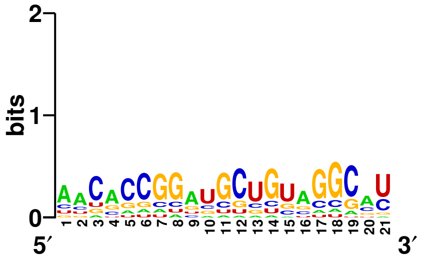

22-mers:

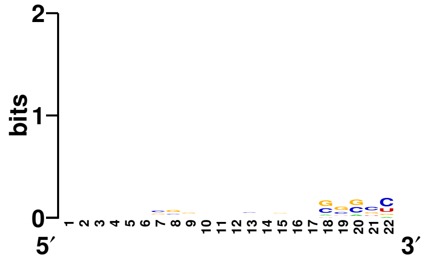

23-mers:

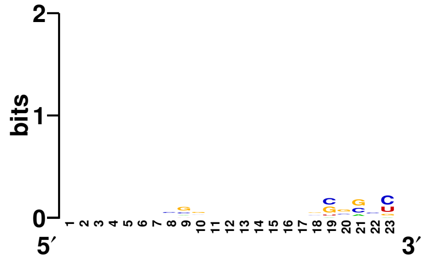

24-mers:

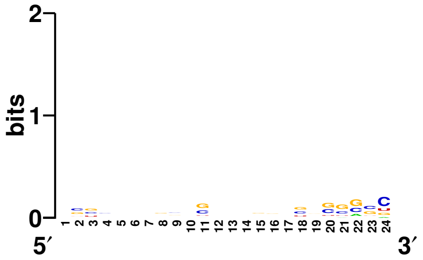

25-mers:

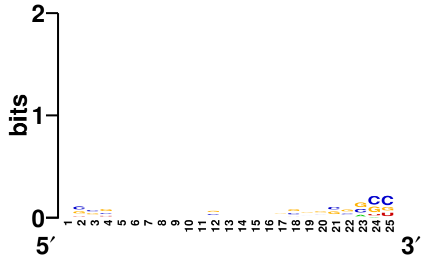

26-mers:

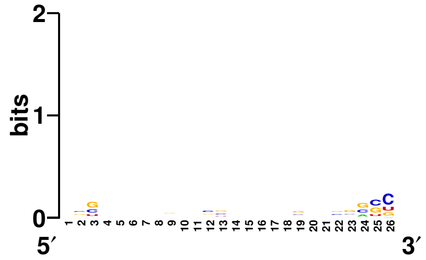

27-mers:

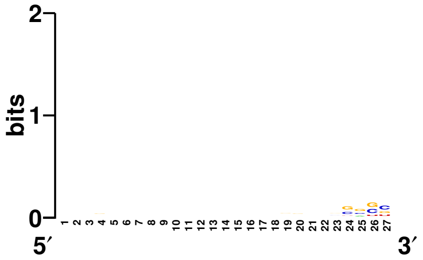

28-mers:

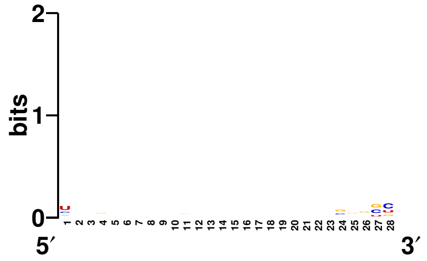

29-mers:

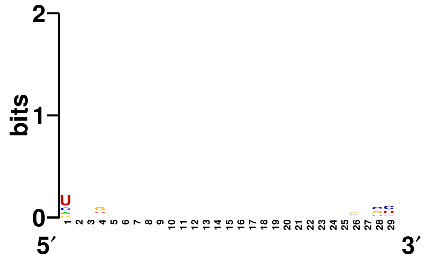

30-mers:

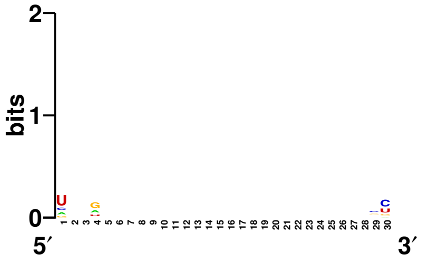

Embryo 36h, library 3:

Sense reads:

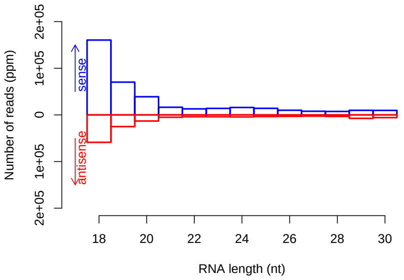

18-mers:

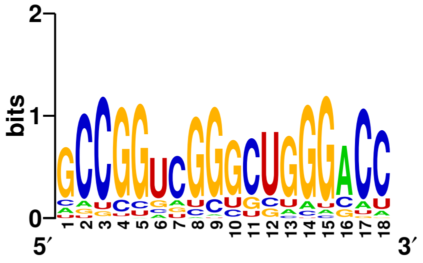

19-mers:

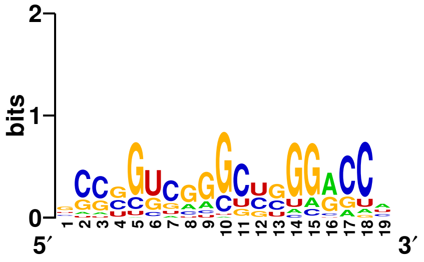

20-mers:

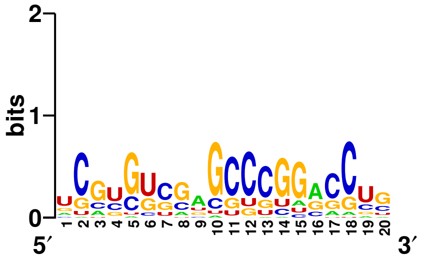

21-mers:

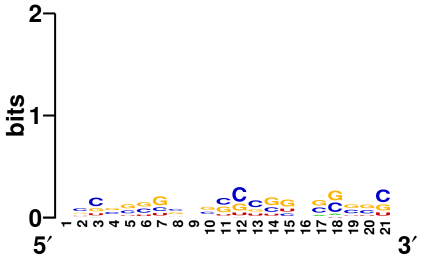

22-mers:

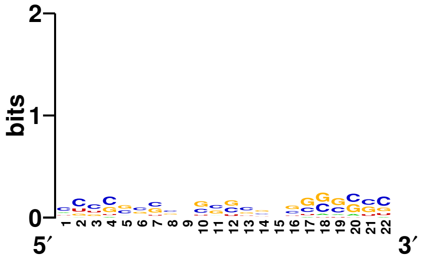

23-mers:

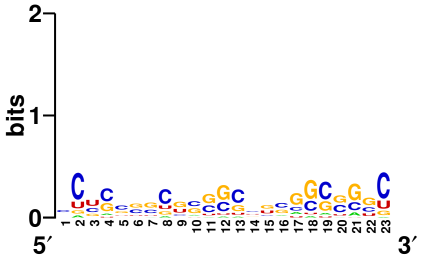

24-mers:

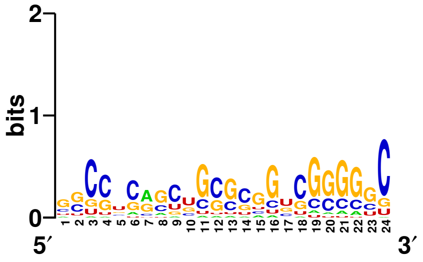

25-mers:

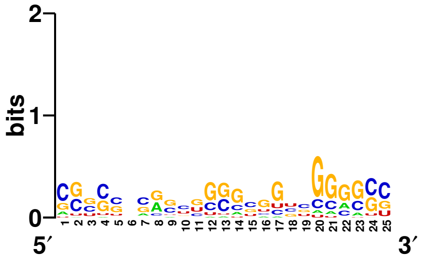

26-mers:

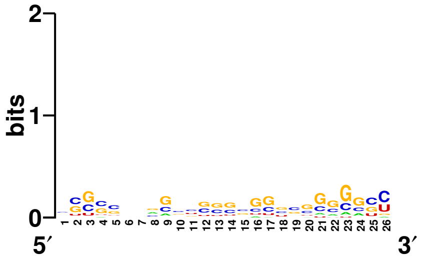

27-mers:

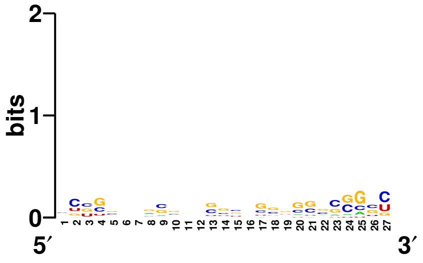

28-mers:

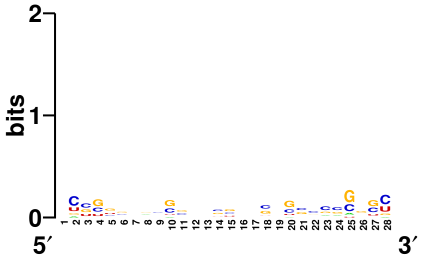

29-mers:

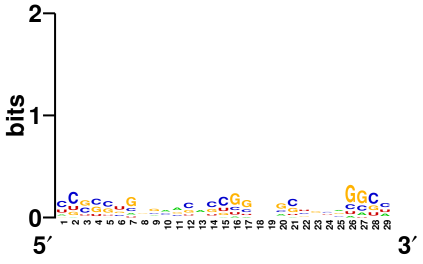

30-mers:

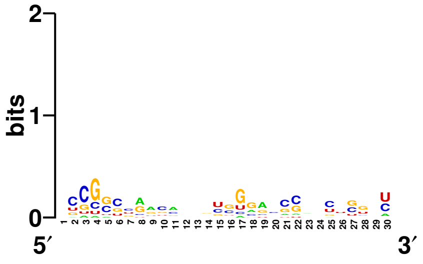

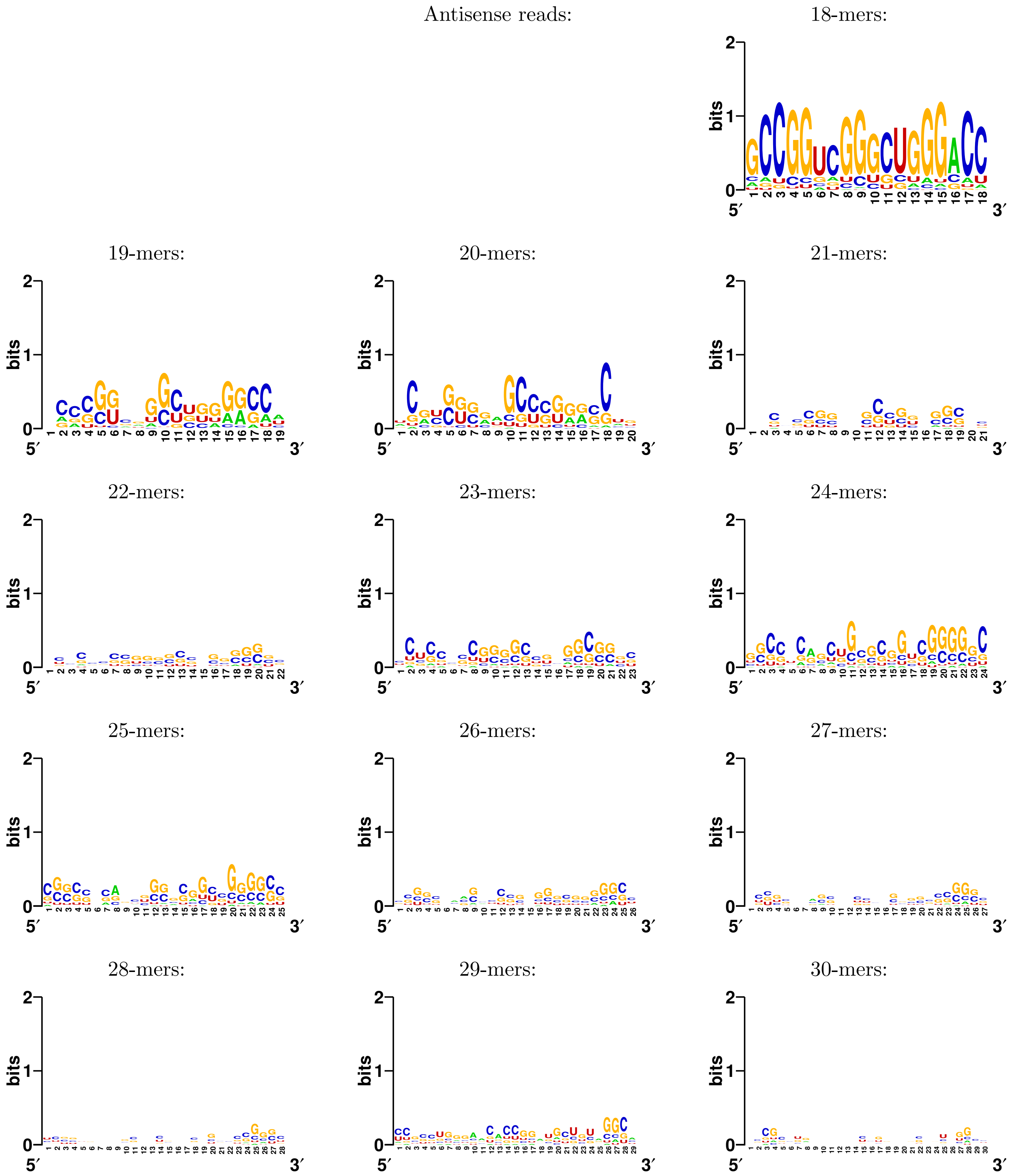
Antisense reads:
18-mers:
19-mers:
20-mers:
21-mers:
22-mers:
23-mers:
24-mers:
25-mers:
26-mers:
27-mers:
28-mers:
29-mers:
30-mers:

Embryo 60h, library 3:

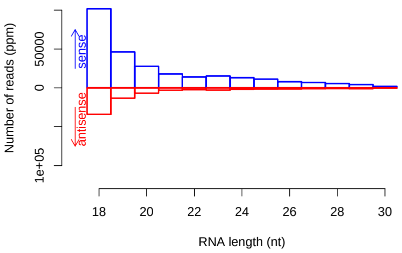

Sense reads:

18-mers:

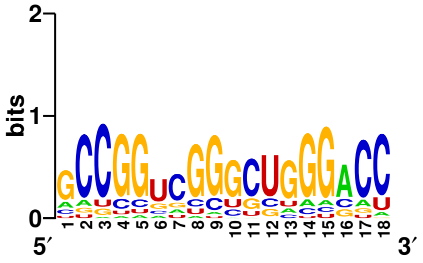

19-mers:

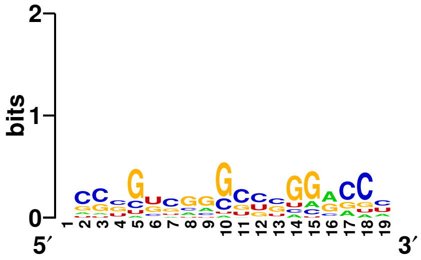

20-mers:

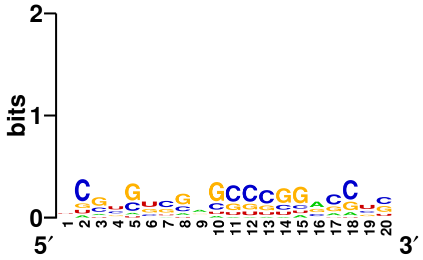

21-mers:

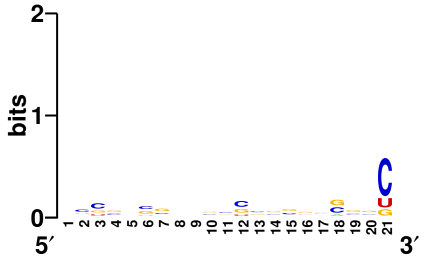

22-mers:

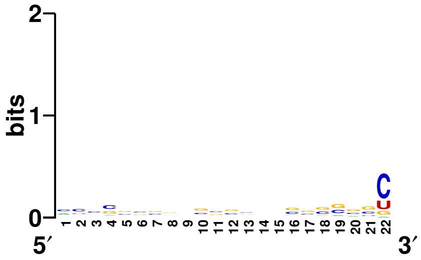

23-mers:

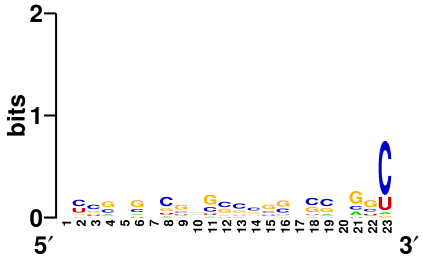

24-mers:

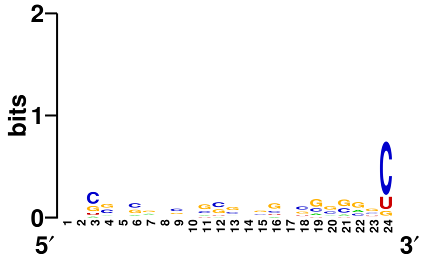

25-mers:

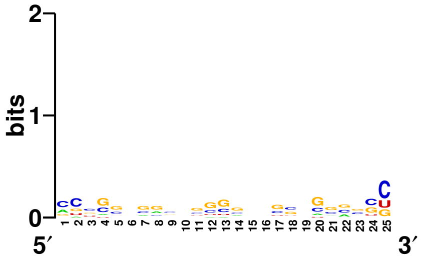

26-mers:

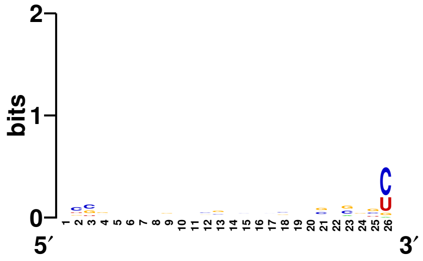

27-mers:

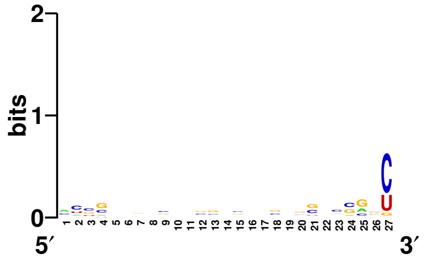

28-mers:

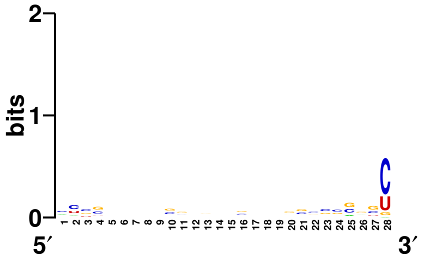

29-mers:

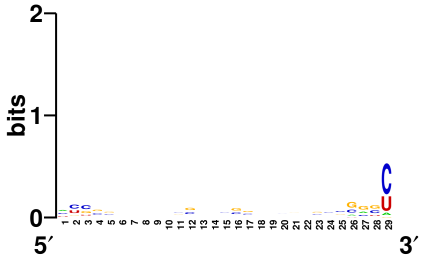

30-mers:

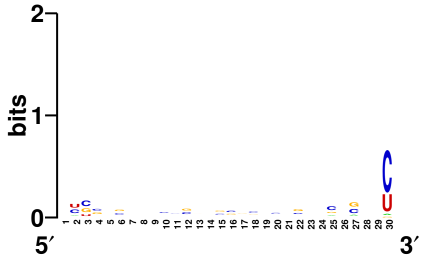

Antisense reads:

18-mers:

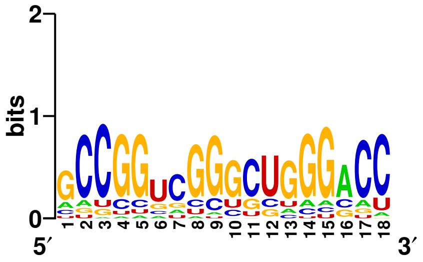

19-mers:

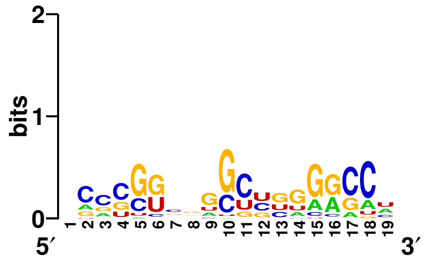

20-mers:

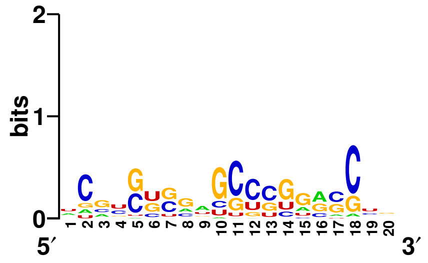

21-mers:

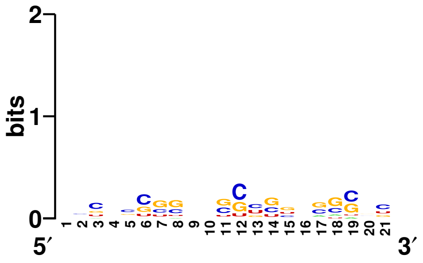

22-mers:

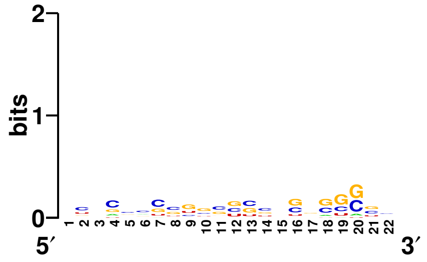

23-mers:

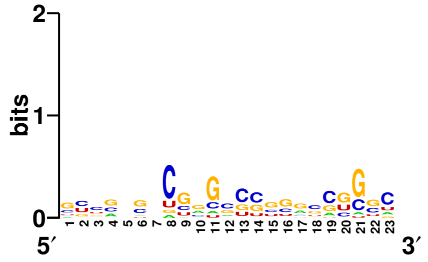

24-mers:

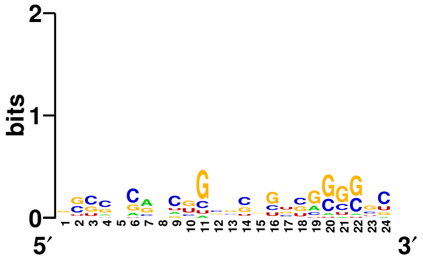

25-mers:

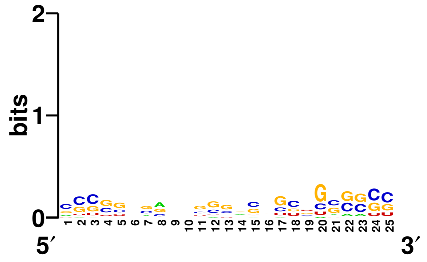

26-mers:

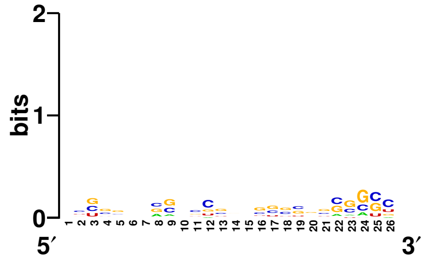

27-mers:

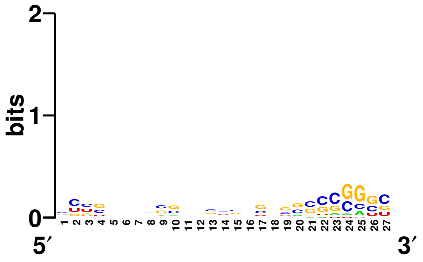

28-mers:

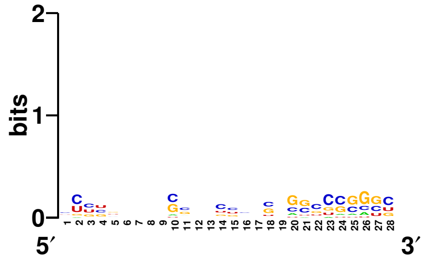

29-mers:

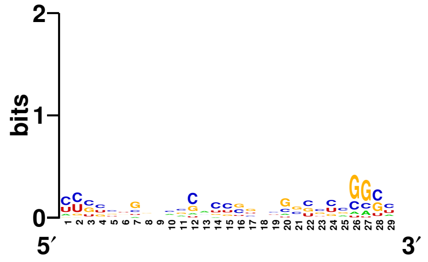

30-mers:

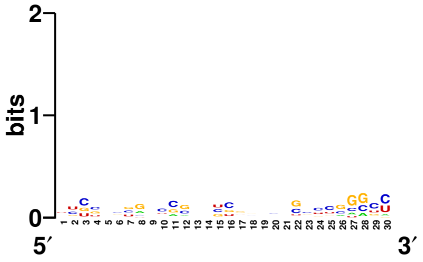

Adult female, library 3:

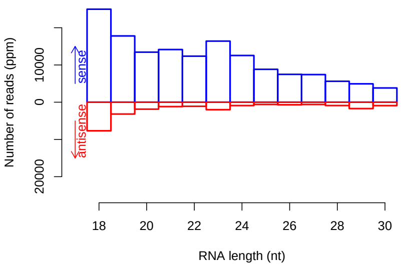

Sense reads:

18-mers:

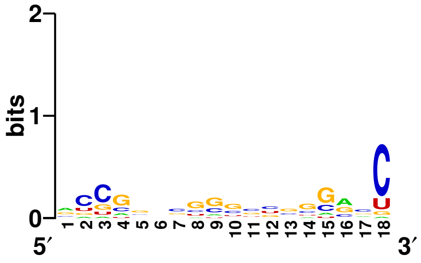

19-mers:

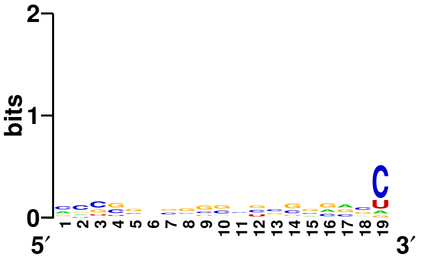

20-mers:

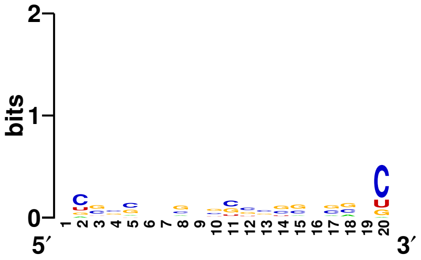

21-mers:

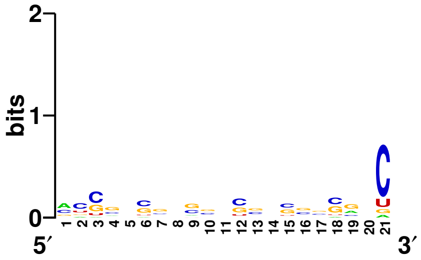

22-mers:

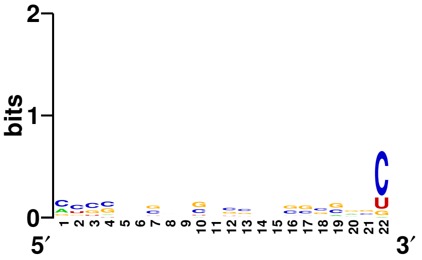

23-mers:

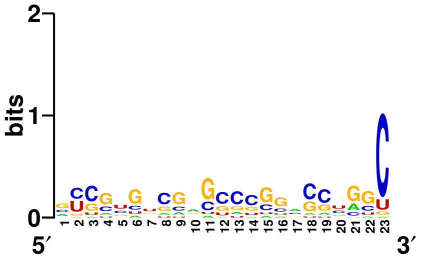

24-mers:

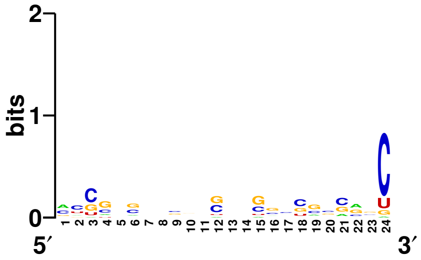

25-mers:

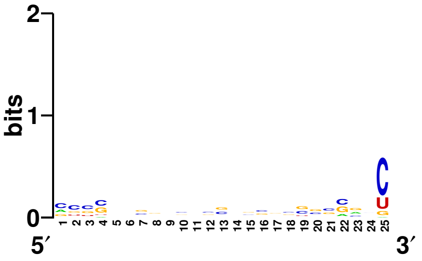

26-mers:

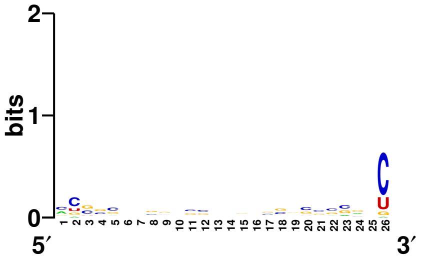

27-mers:

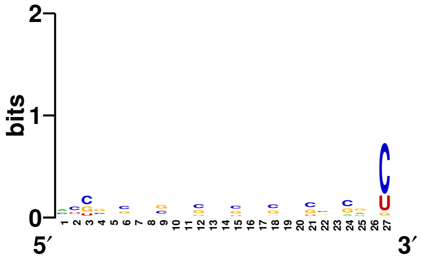

28-mers:

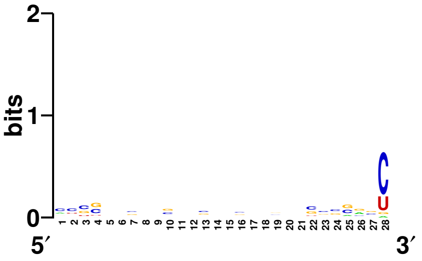

29-mers:

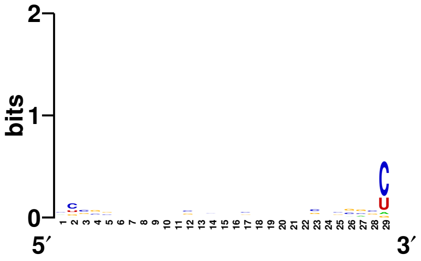

30-mers:

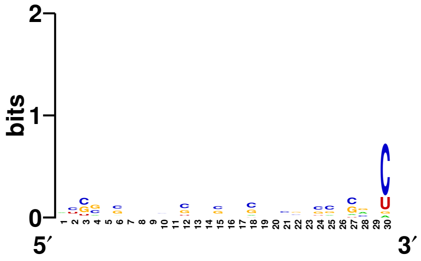

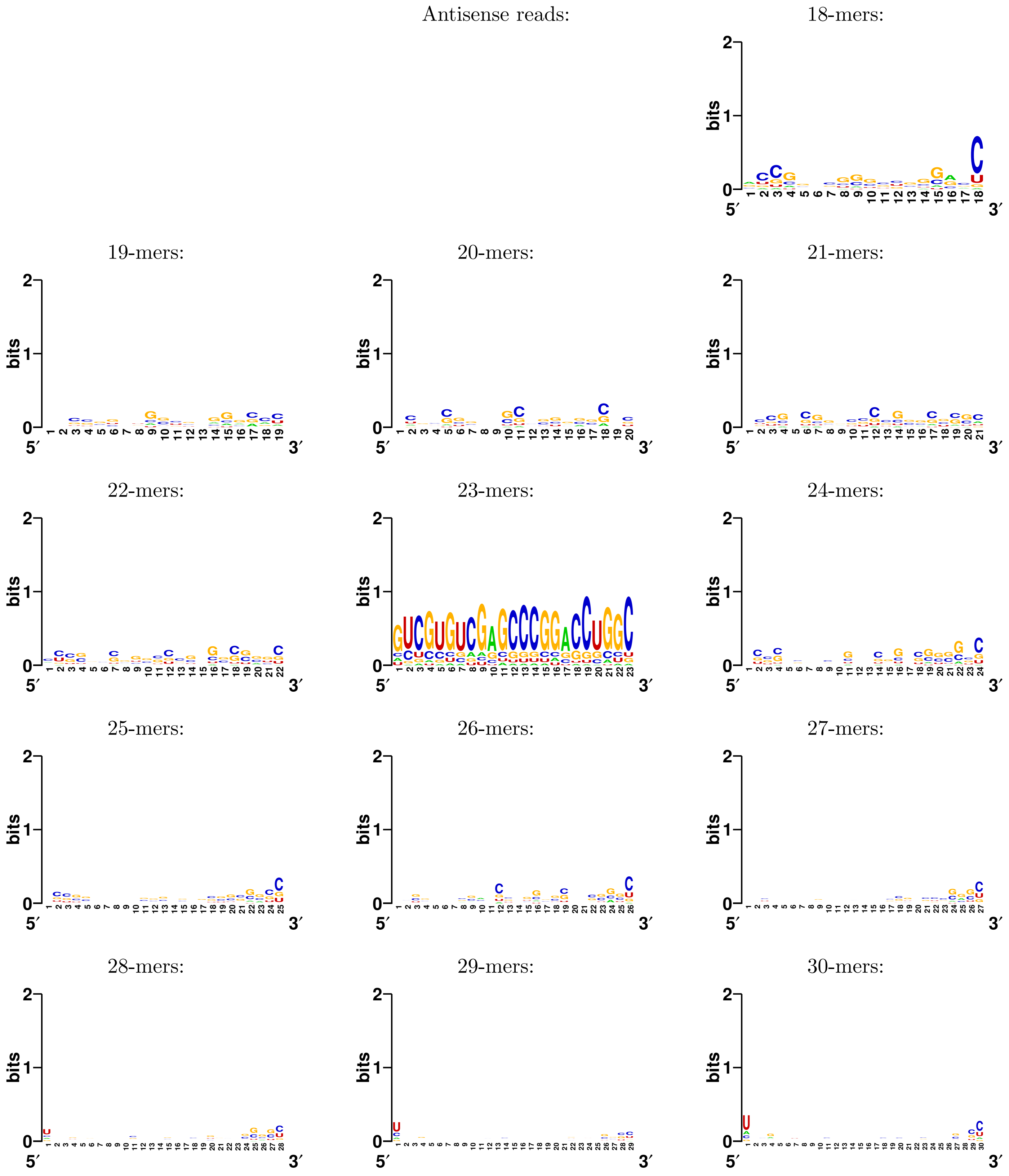
Antisense reads:
18-mers:
19-mers:
20-mers:
21-mers:
22-mers:
23-mers:
24-mers:
25-mers:
26-mers:
27-mers:
28-mers:
29-mers:
30-mers:
bits
5′
3′

Adult male, library 3:

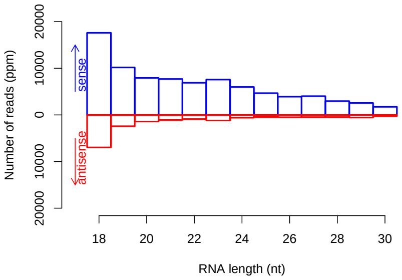

Sense reads:

18-mers:

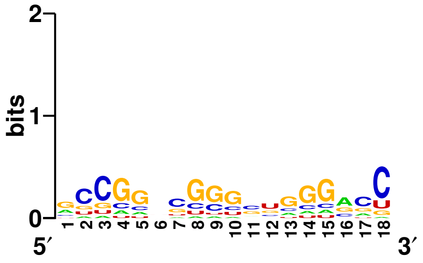

19-mers:

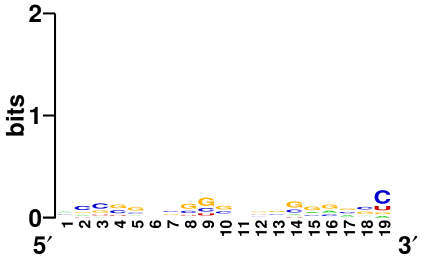

20-mers:

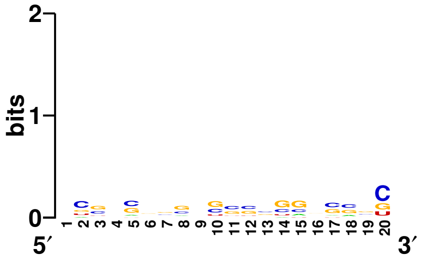

21-mers:

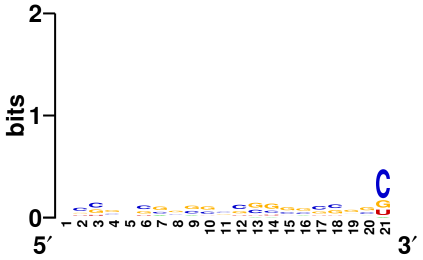

22-mers:

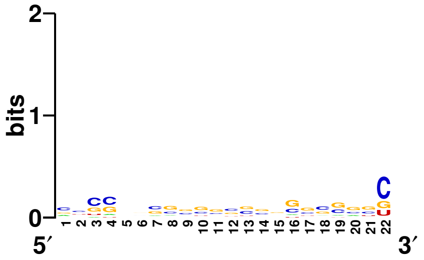

23-mers:

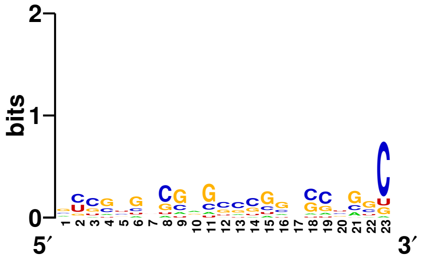

24-mers:

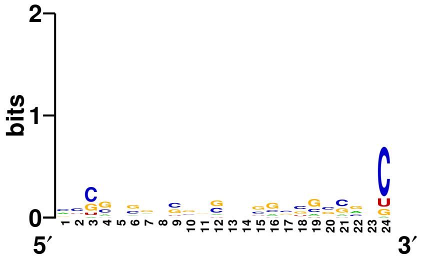

25-mers:

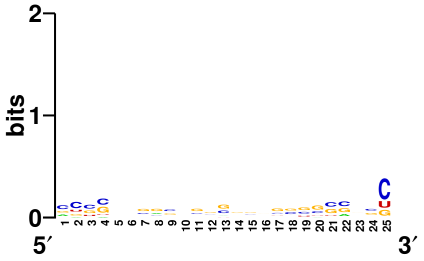

26-mers:

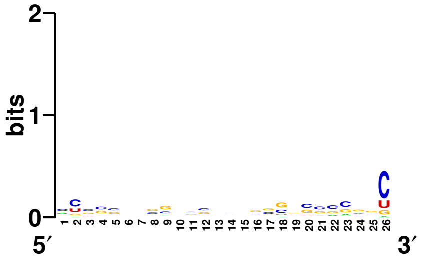

27-mers:

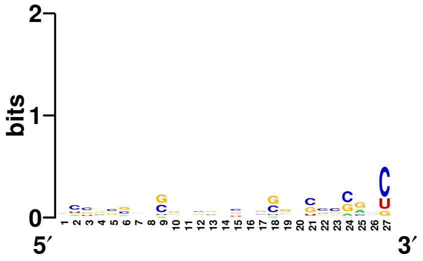

28-mers:

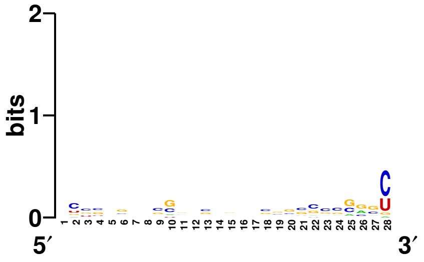

29-mers:

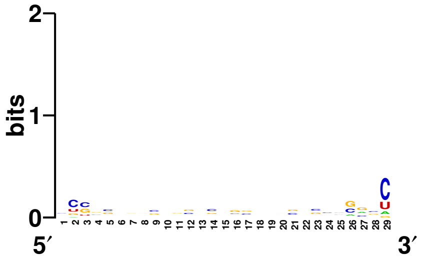

30-mers:

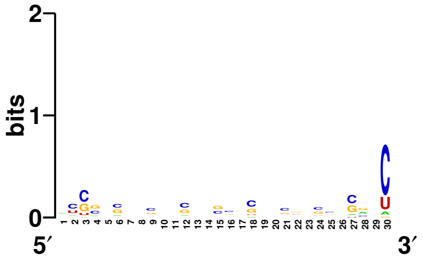

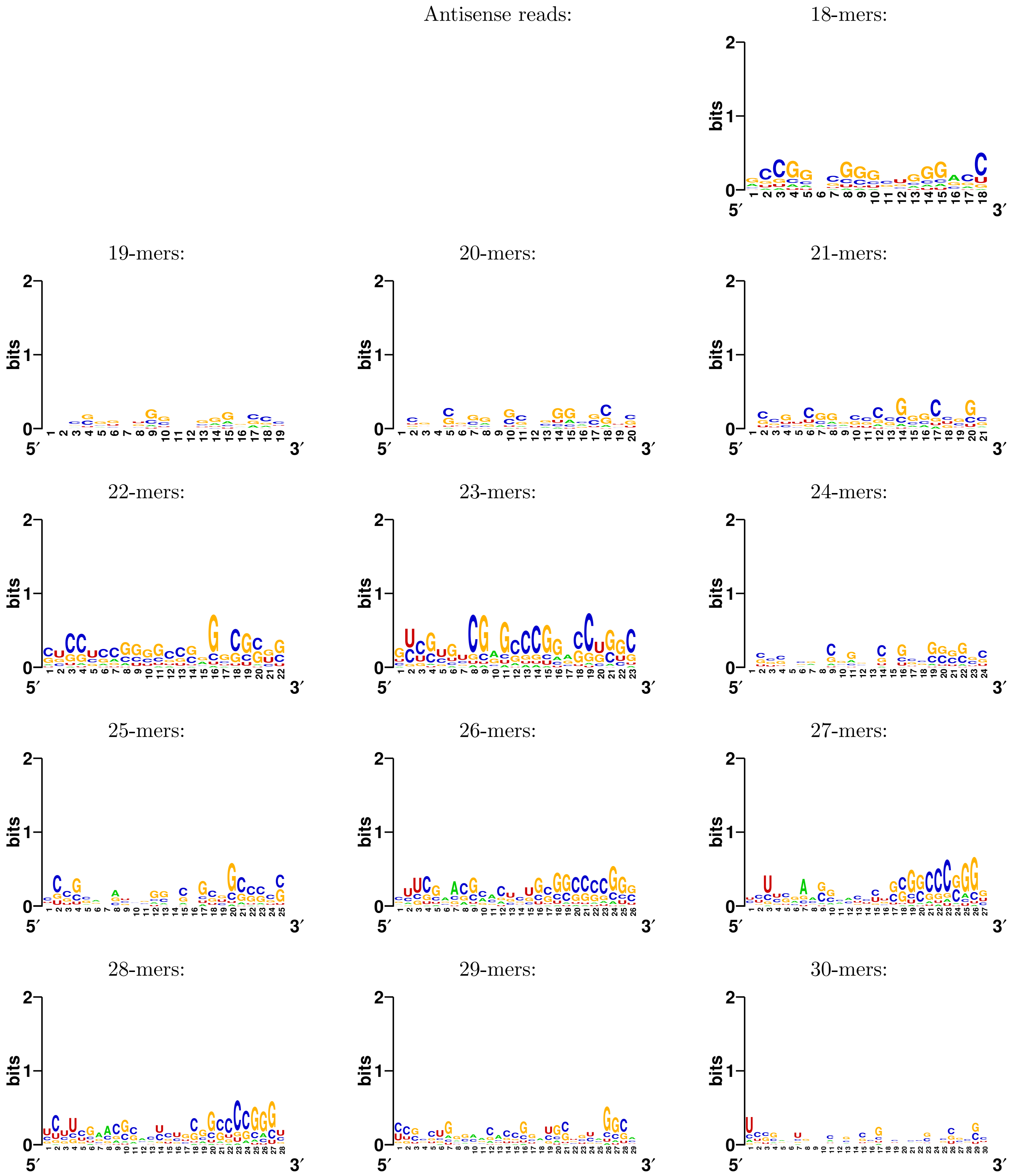
Antisense reads:
18-mers:
19-mers:
20-mers:
21-mers:
22-mers:
23-mers:
24-mers:
25-mers:
26-mers:
27-mers:
28-mers:
29-mers:
30-mers:
bits
5′
3′

3.4 Libraries #4 (3´ modified, 5´ hydroxyl or polyphosphorylated small RNAs)

Embryo 8h, library 4:

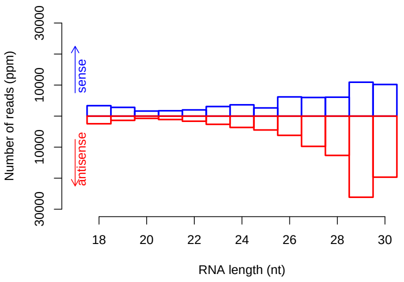

Sense reads:

18-mers:

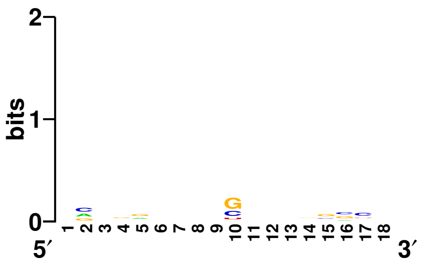

19-mers:

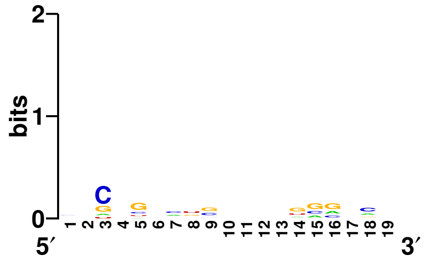

20-mers:

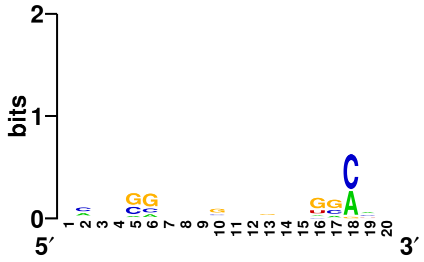

21-mers:

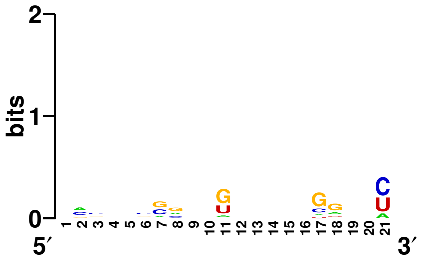

22-mers:

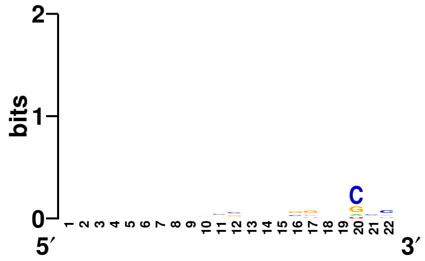

23-mers:

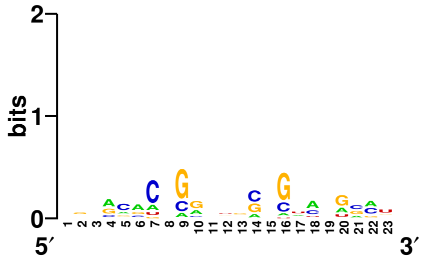

24-mers:

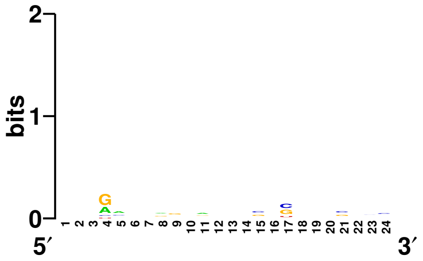

25-mers:

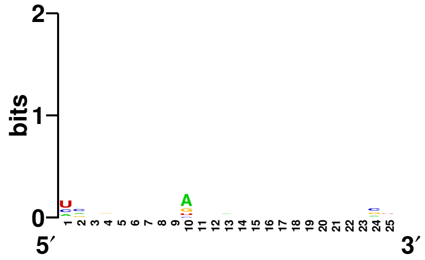

26-mers:

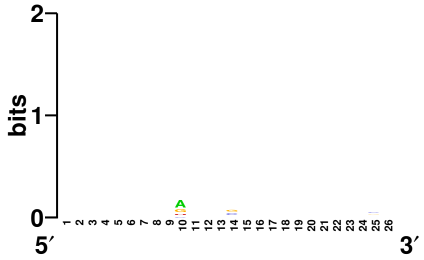

27-mers:

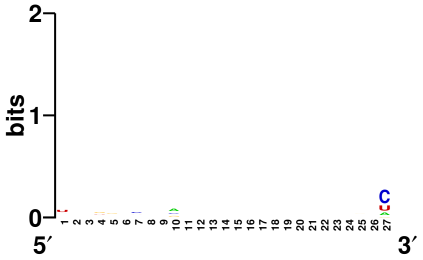

28-mers:

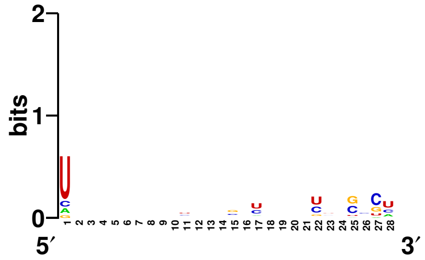

29-mers:

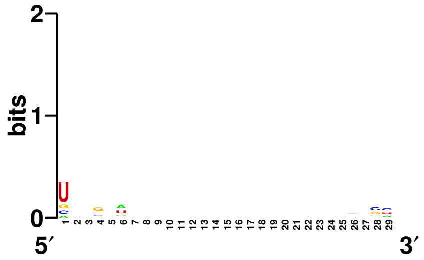

30-mers:

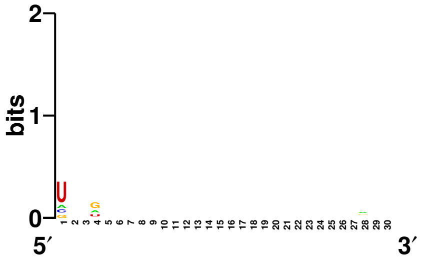

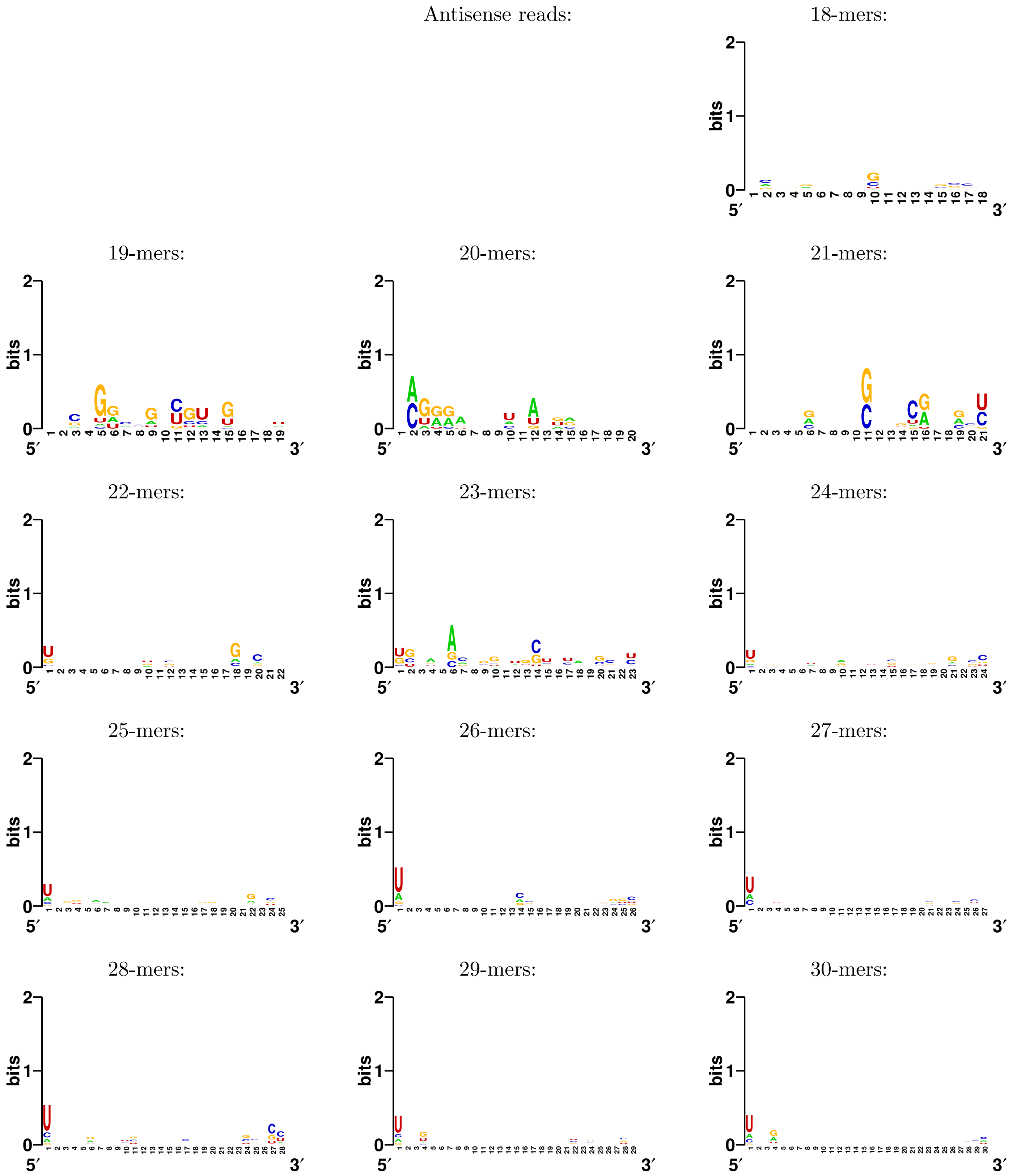
Antisense reads:
18-mers:
19-mers:
20-mers:
21-mers:
22-mers:
23-mers:
24-mers:
25-mers:
26-mers:
27-mers:
28-mers:
29-mers:
30-mers:
bits
5′
3′

Embryo 15h, library 4:

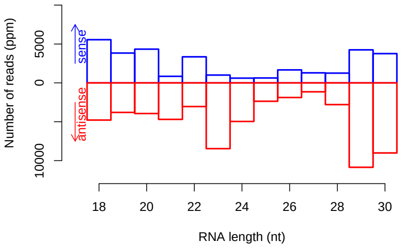

Sense reads:

18-mers:

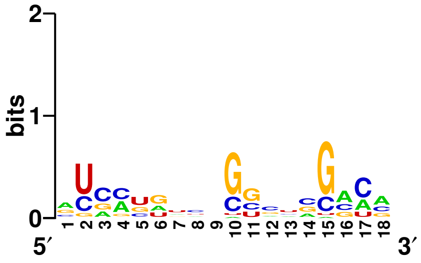

19-mers:

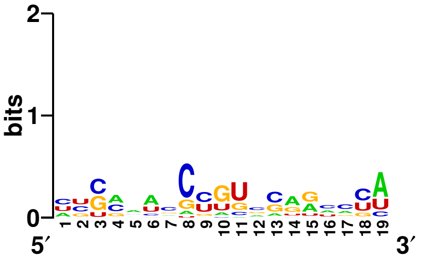

20-mers:

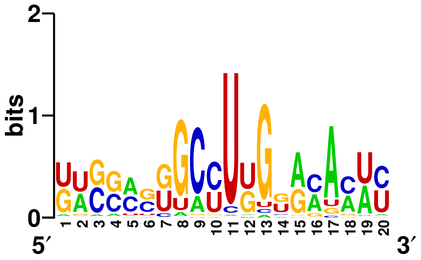

21-mers:

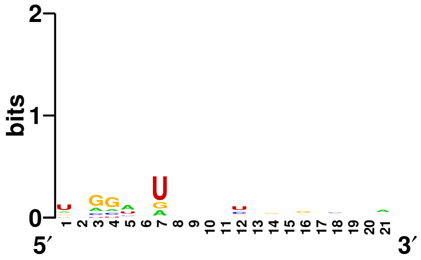

22-mers:

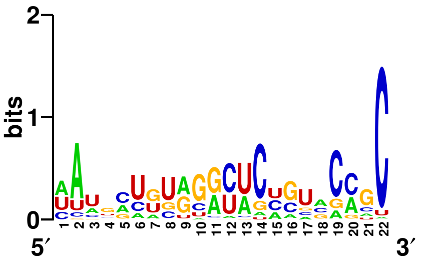

23-mers:

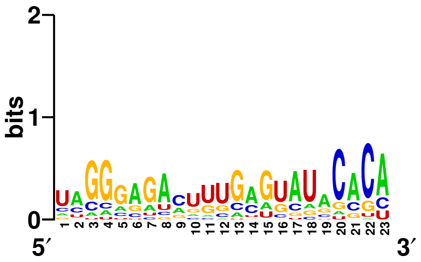

24-mers:

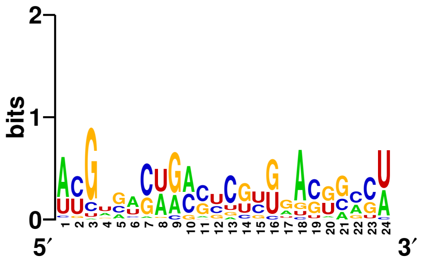

25-mers:

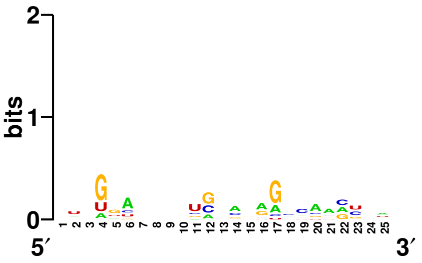

26-mers:

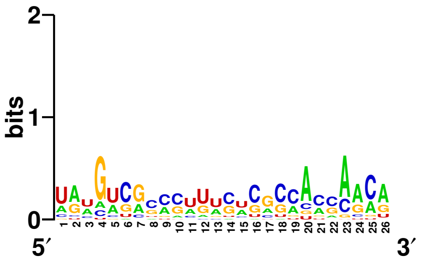

27-mers:

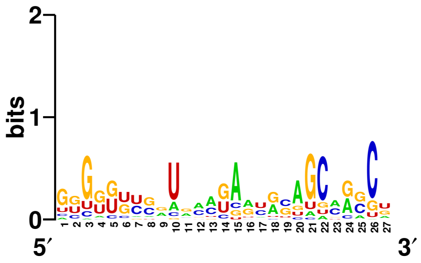

28-mers:

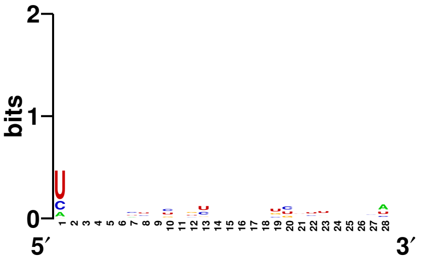

29-mers:

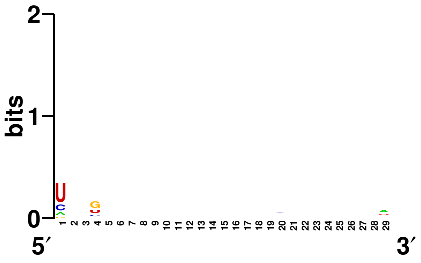

30-mers:

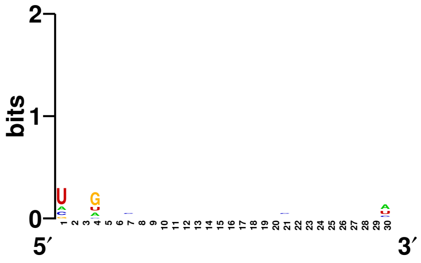

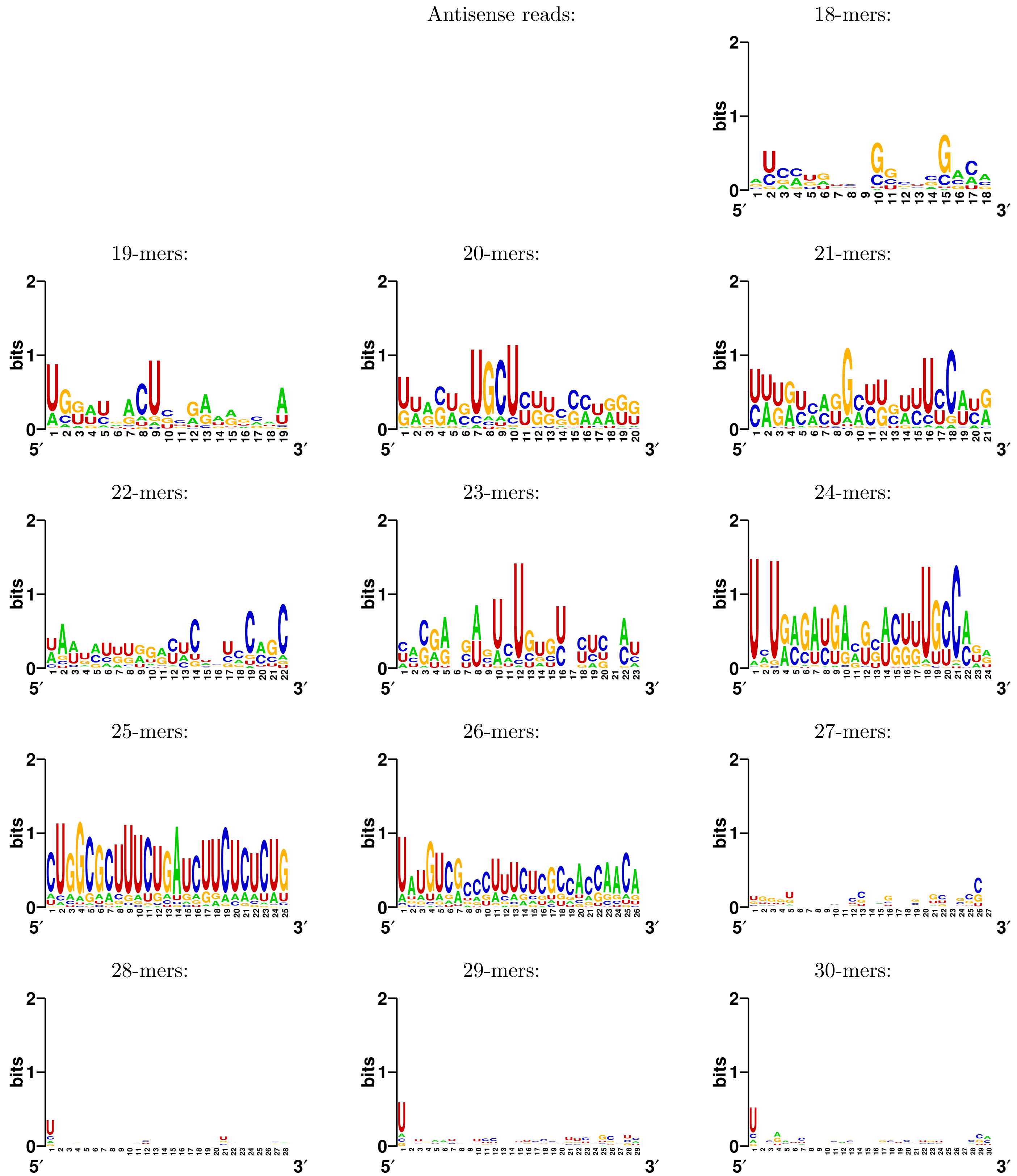
Antisense reads:
18-mers:
19-mers:
20-mers:
21-mers:
22-mers:
23-mers:
24-mers:
25-mers:
26-mers:
27-mers:
28-mers:
29-mers:
30-mers:
bits
5′
3′

Embryo 36h, library 4:

Sense reads:

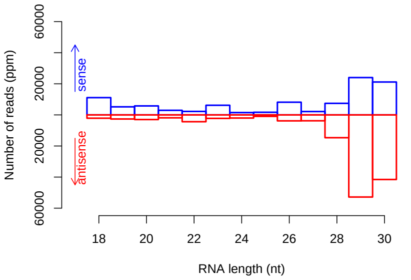

18-mers:

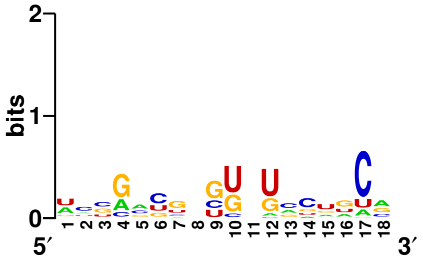

19-mers:

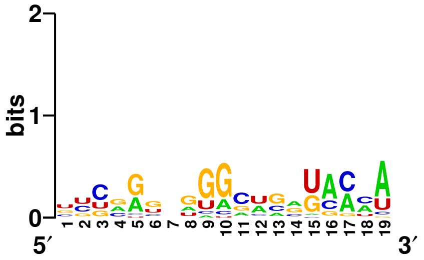

20-mers:

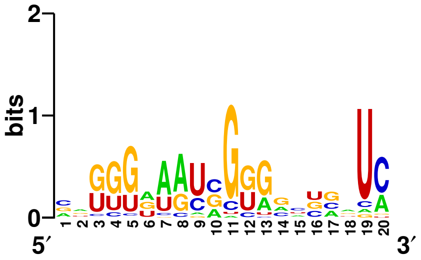

21-mers:

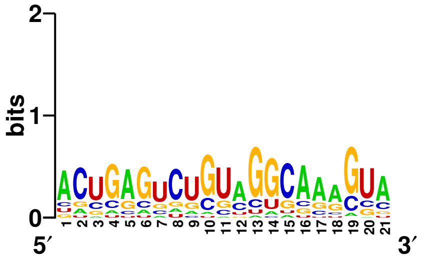

22-mers:

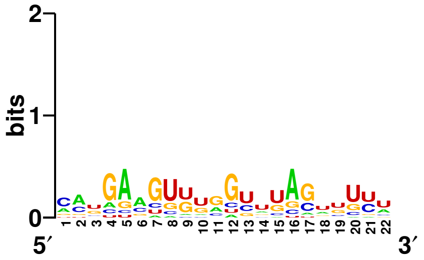

23-mers:

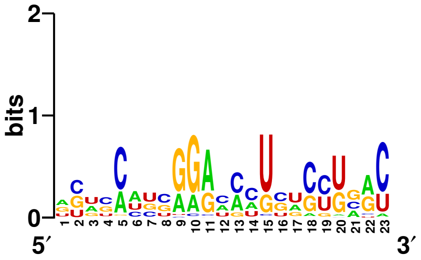

24-mers:

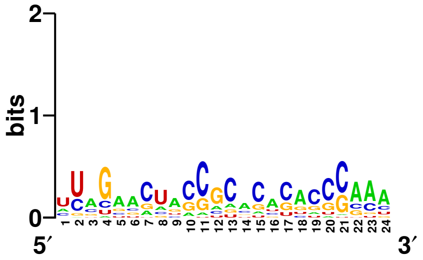

25-mers:

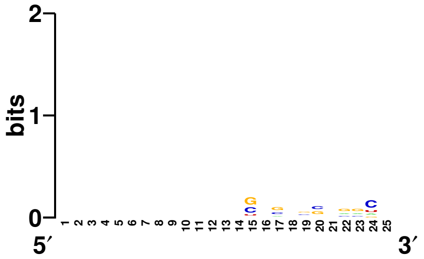

26-mers:

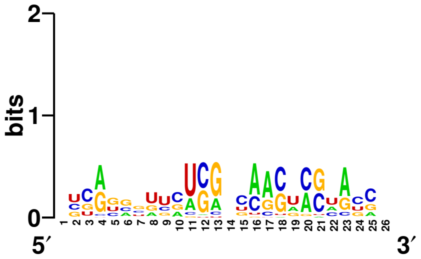

27-mers:

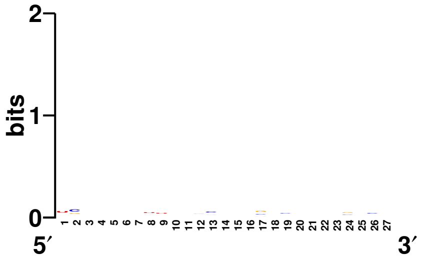

28-mers:

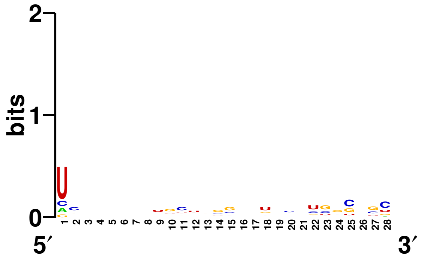

29-mers:

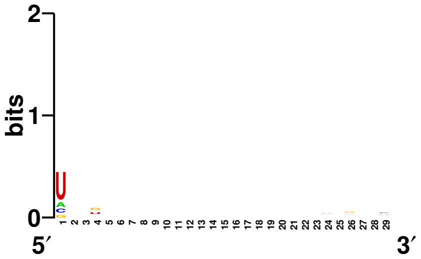

30-mers:

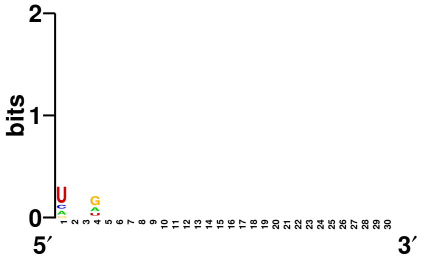

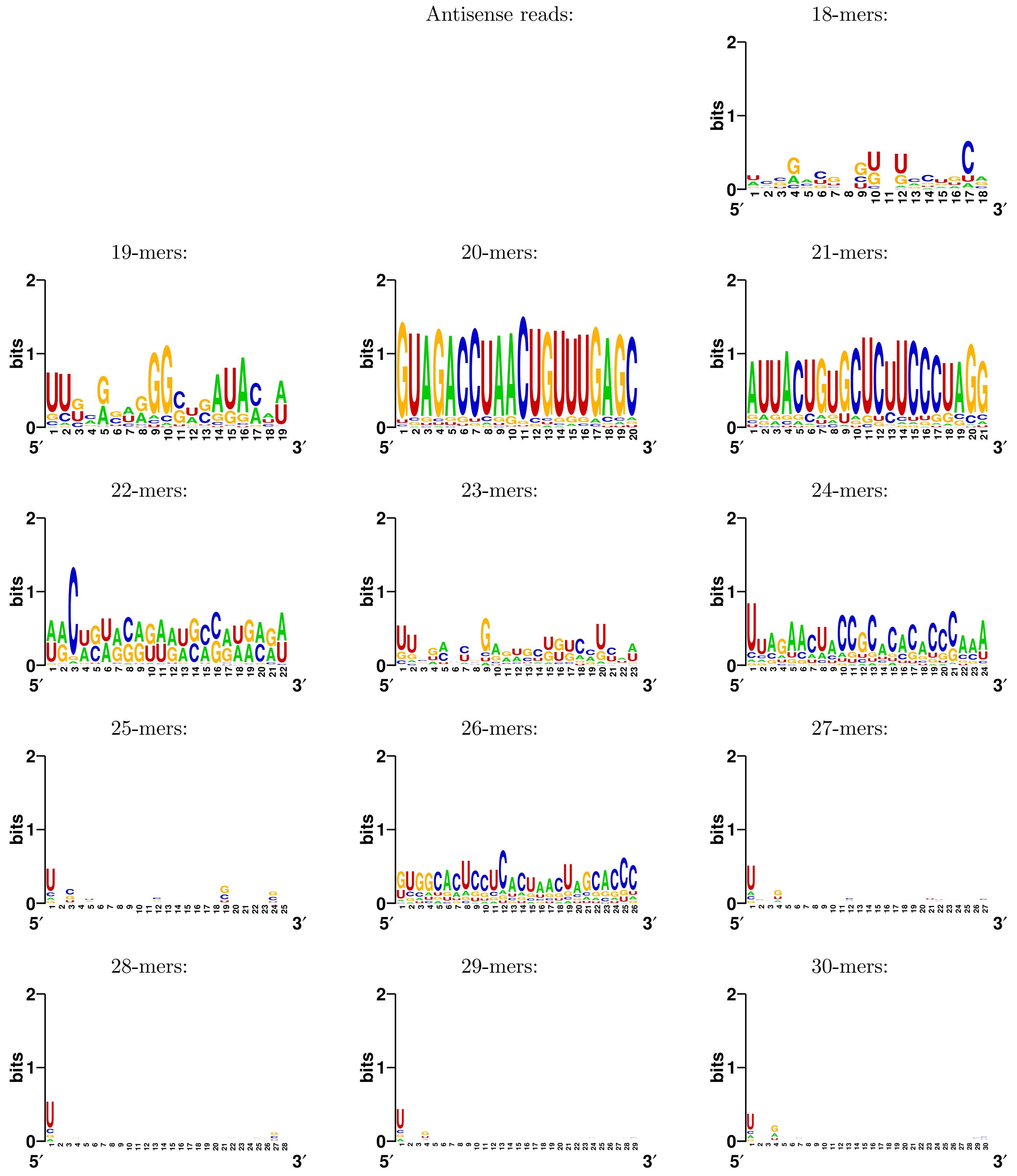
Antisense reads:
18-mers:
19-mers:
20-mers:
21-mers:
22-mers:
23-mers:
24-mers:
25-mers:
26-mers:
27-mers:
28-mers:
29-mers:
30-mers:
bits
5′
3′

Embryo 60h, library 4:

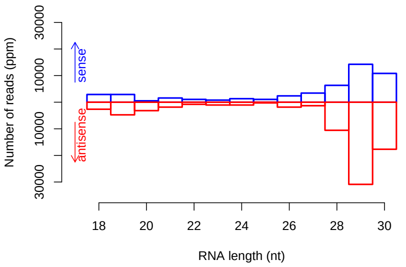

Sense reads:

18-mers:

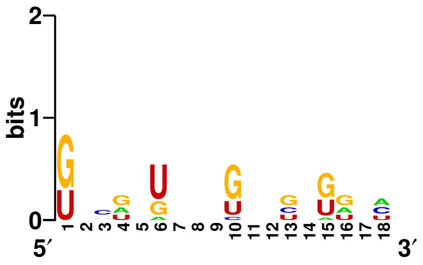

19-mers:

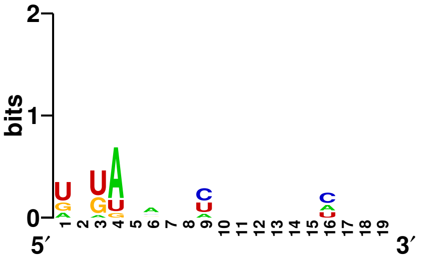

20-mers:

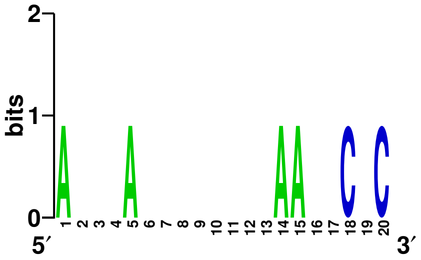

21-mers:

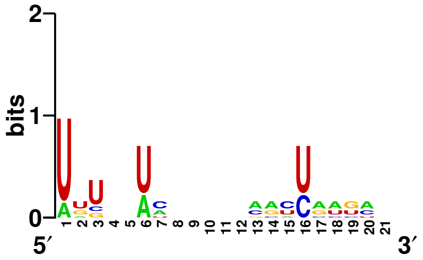

22-mers:

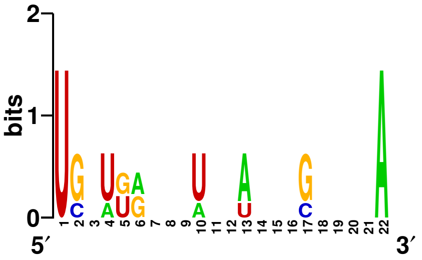

23-mers:

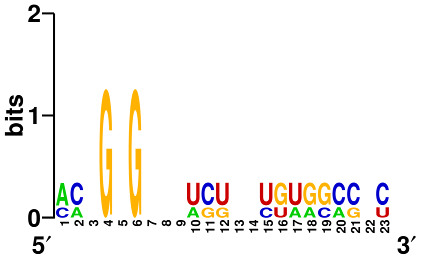

24-mers:

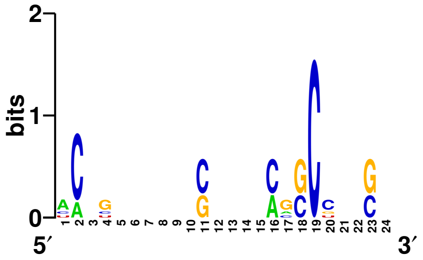

25-mers:

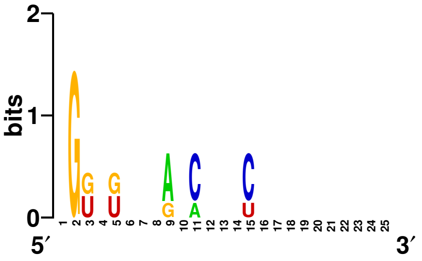

26-mers:

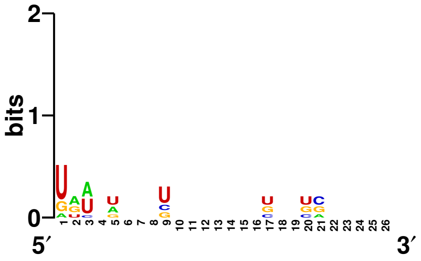

27-mers:

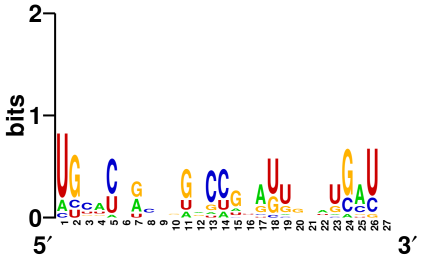

28-mers:

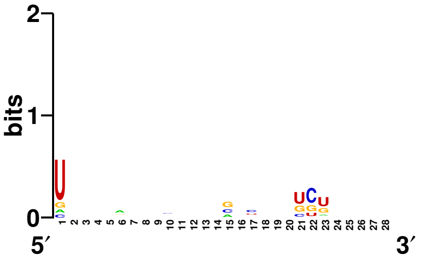

29-mers:

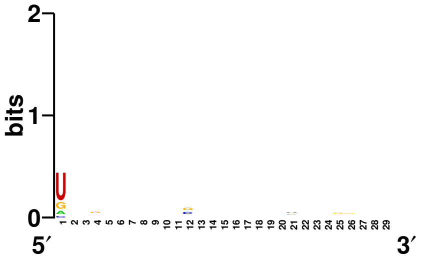

30-mers:

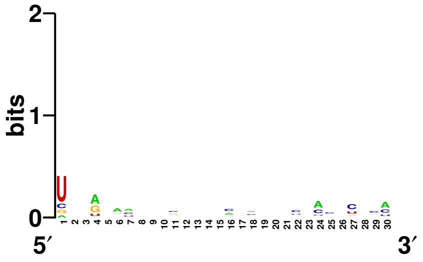

Antisense reads:

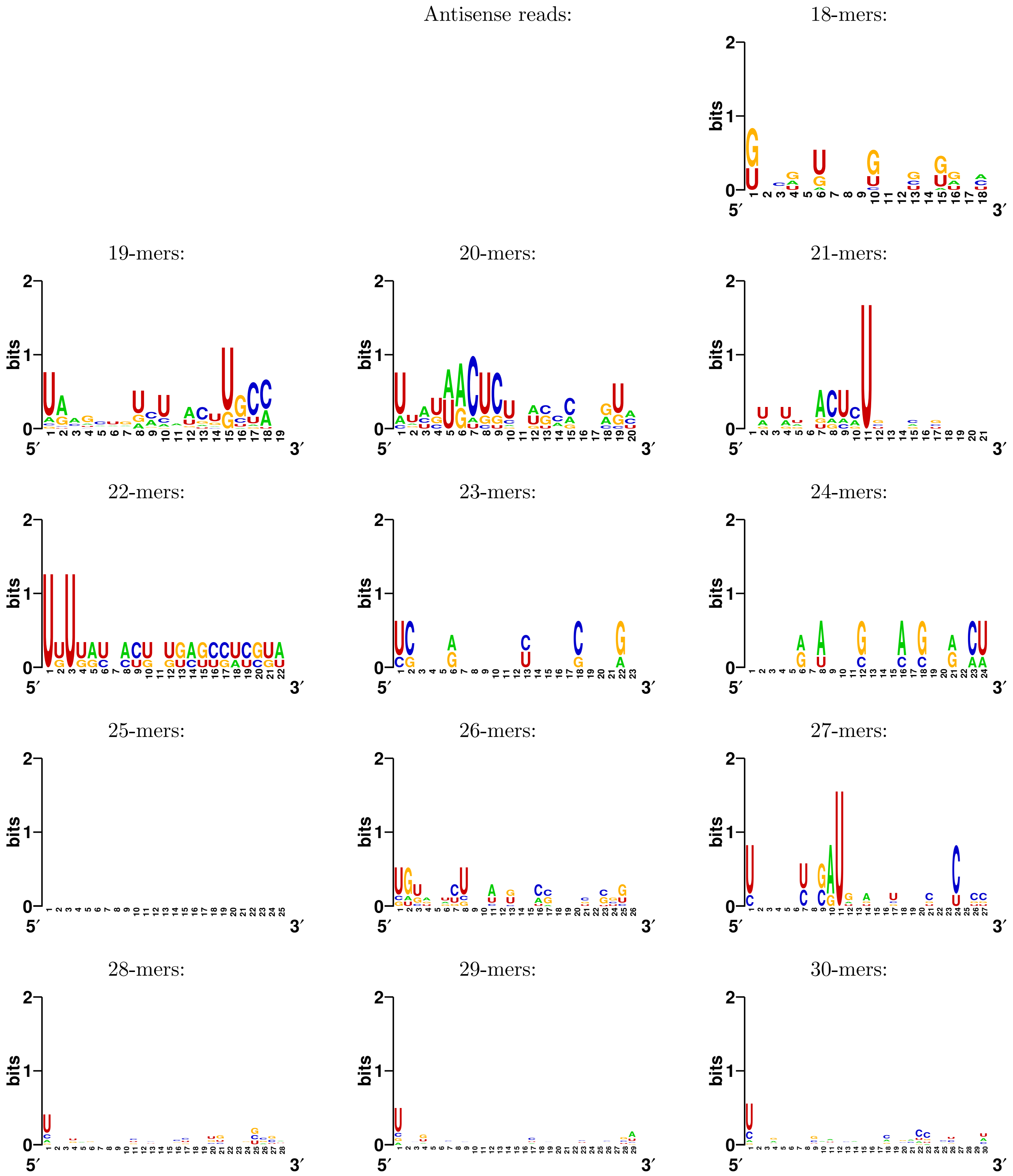

Adult female, library 4:

Sense reads:

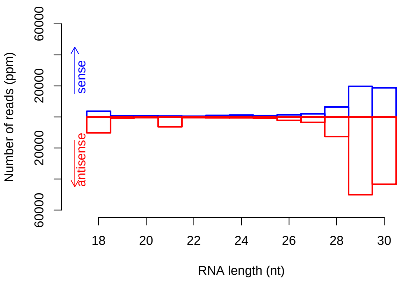

18-mers:

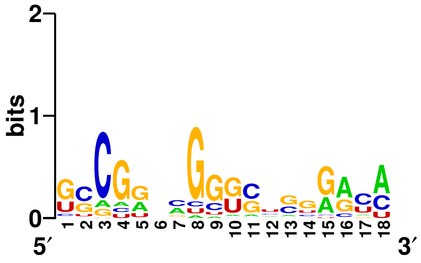

19-mers:

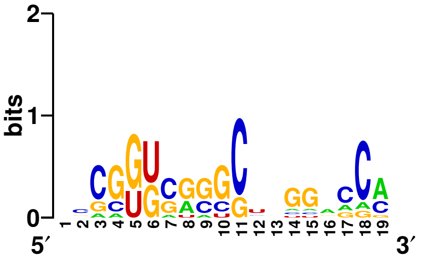

20-mers:

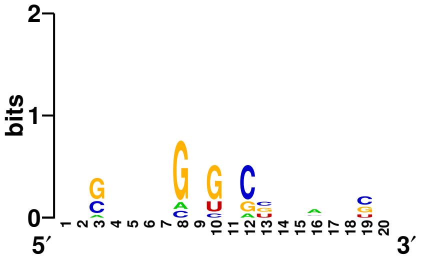

21-mers:

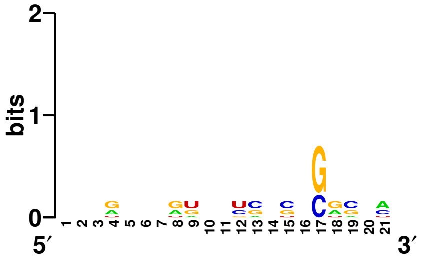

22-mers:

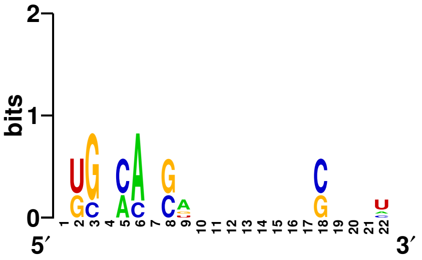

23-mers:

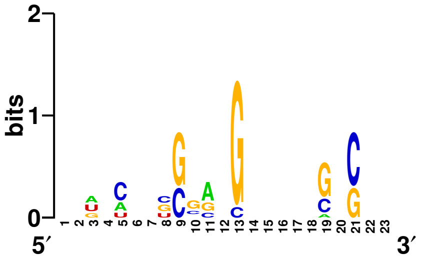

24-mers:

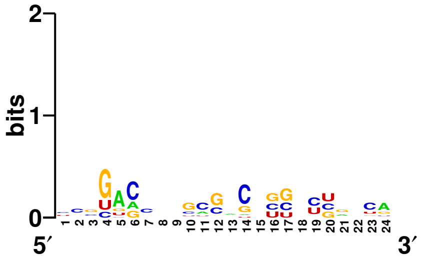

25-mers:

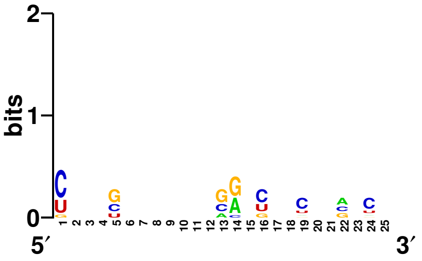

26-mers:

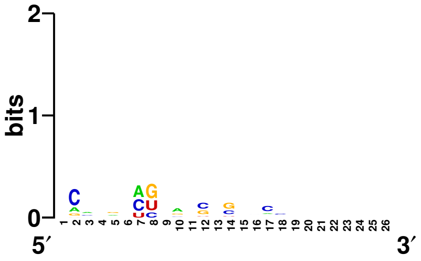

27-mers:

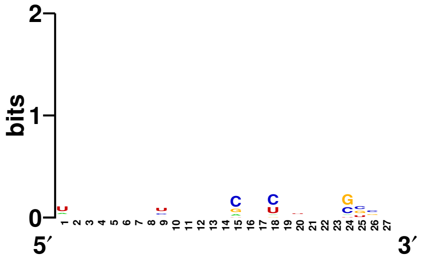

28-mers:

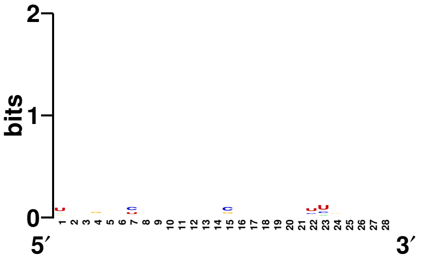

29-mers:

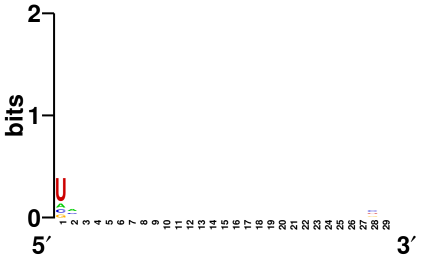

30-mers:

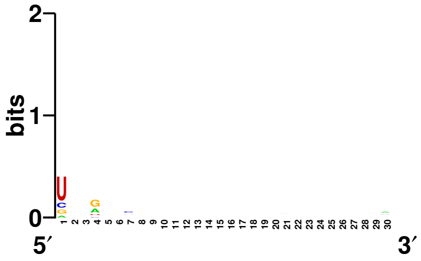

Antisense reads:

18-mers:

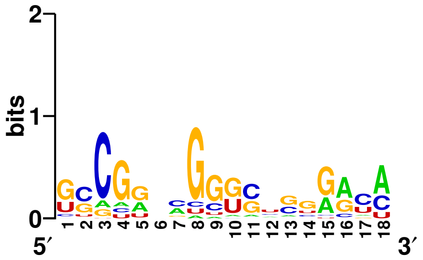

19-mers:

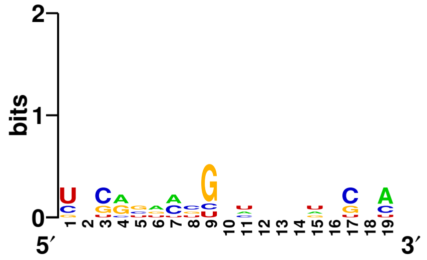

20-mers:

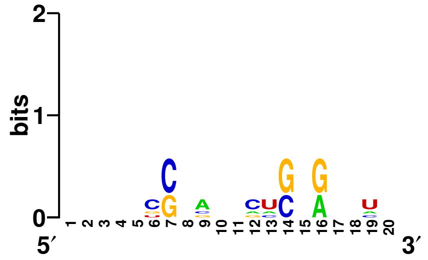

21-mers:

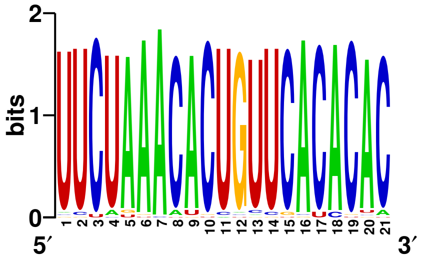

22-mers:

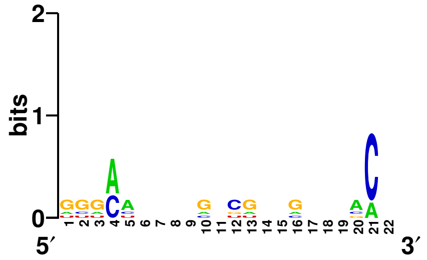

23-mers:

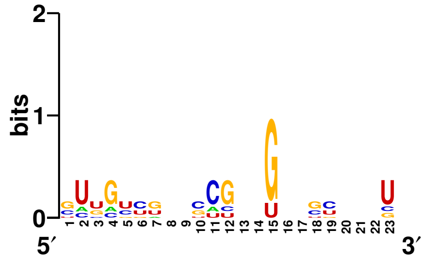

24-mers:

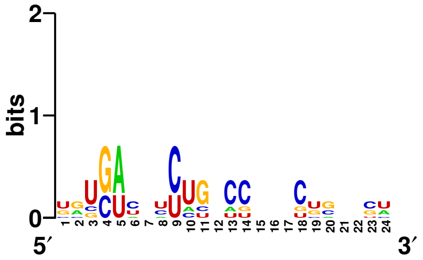

25-mers:

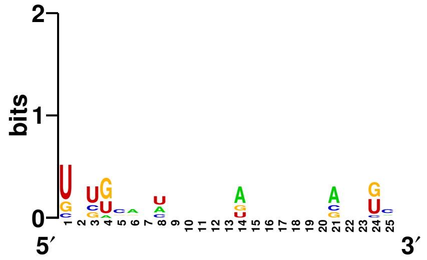

26-mers:

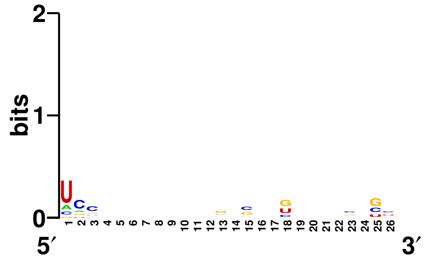

27-mers:

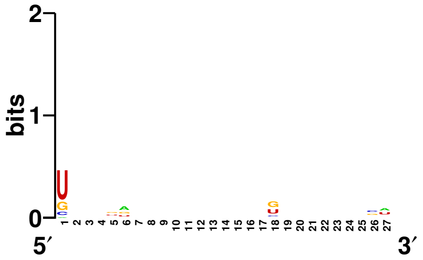

28-mers:

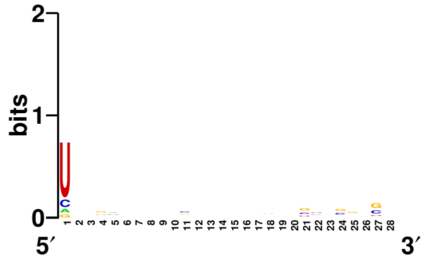

29-mers:

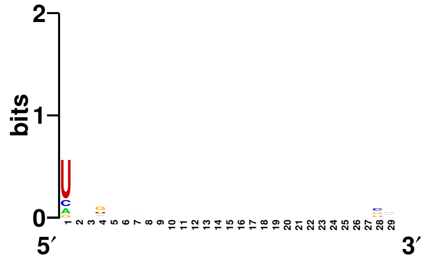

30-mers:

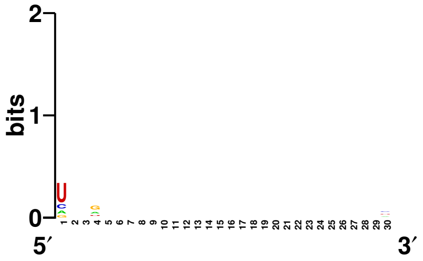

Adult male, library 4:

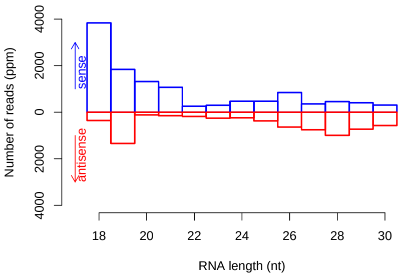

Sense reads:

18-mers:

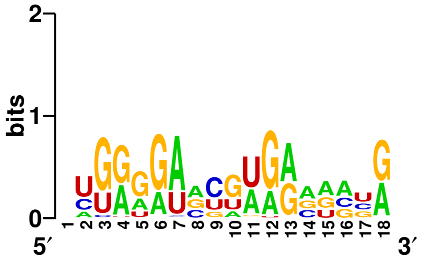

19-mers:

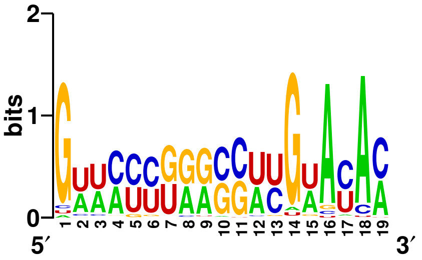

20-mers:

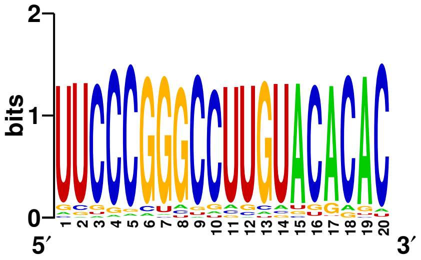

21-mers:

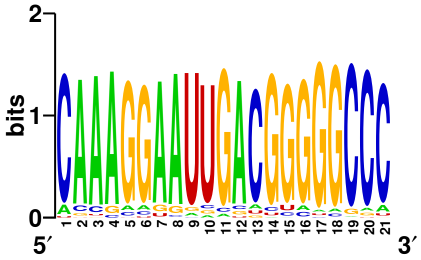

22-mers:

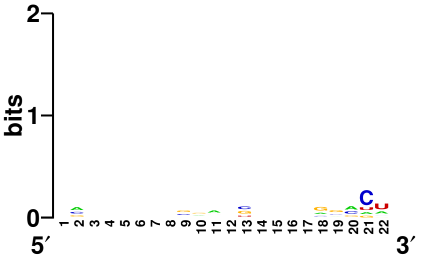

23-mers:

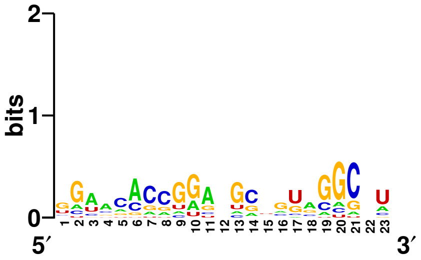

24-mers:

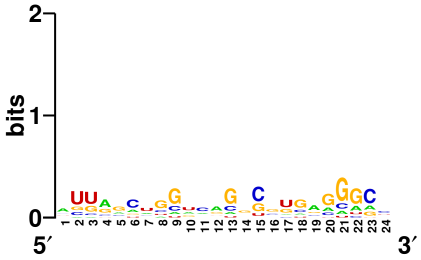

25-mers:

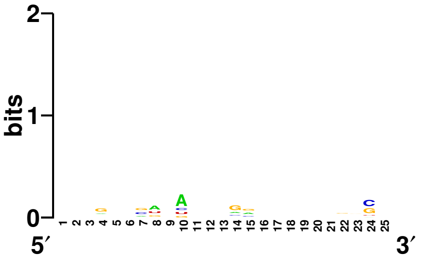

26-mers:

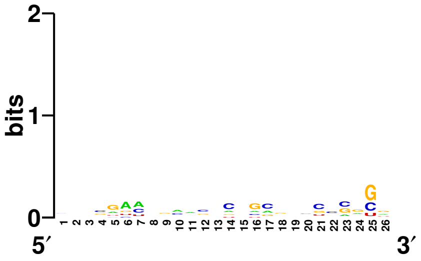

27-mers:

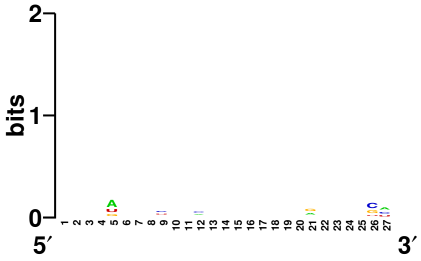

28-mers:

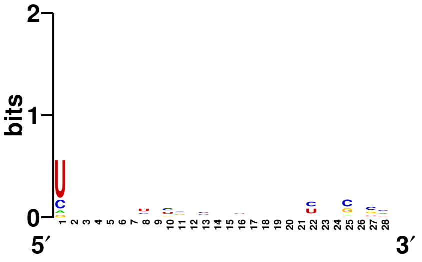

29-mers:

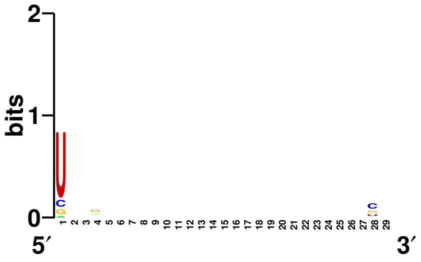

30-mers:

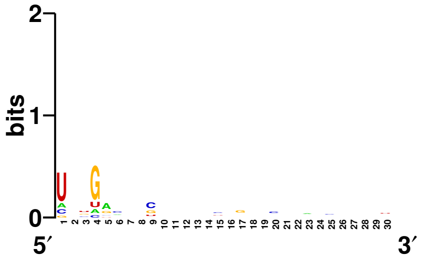

Antisense reads:

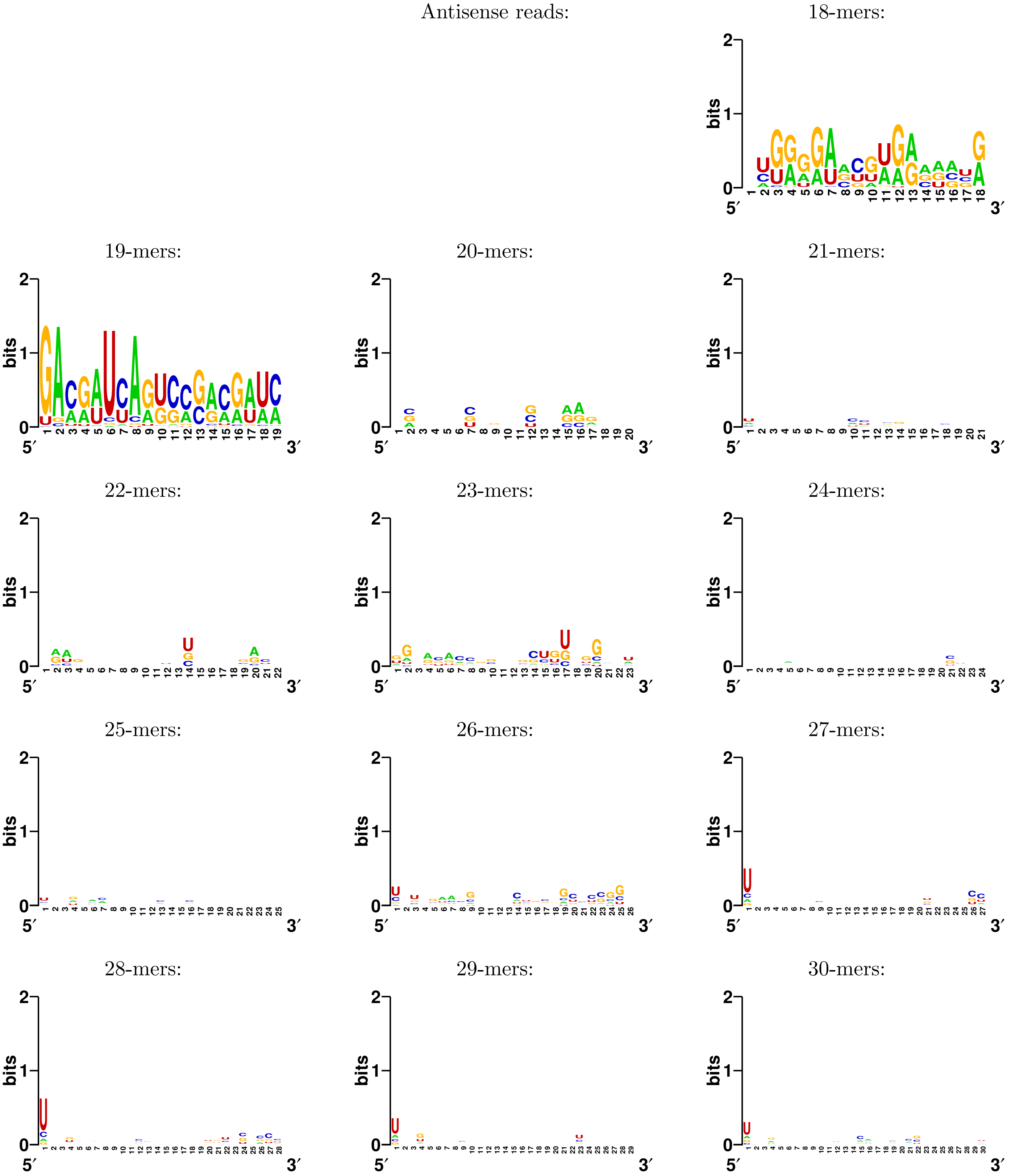

4 Reads matching RNAs with long ORF’s

4.1 Libraries #1 (total 5´ monophosphorylated small RNAs)

Embryo 8h, library 1:

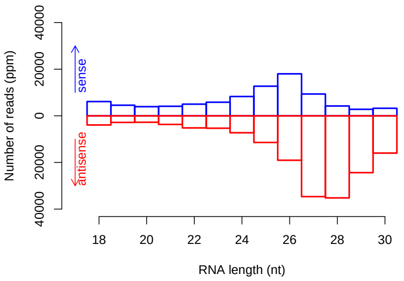

Sense reads:

18-mers:

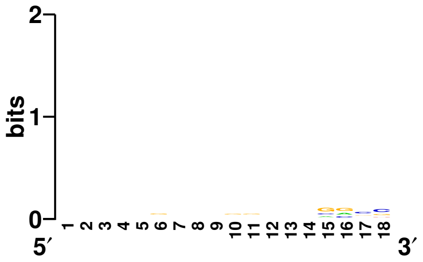

19-mers:

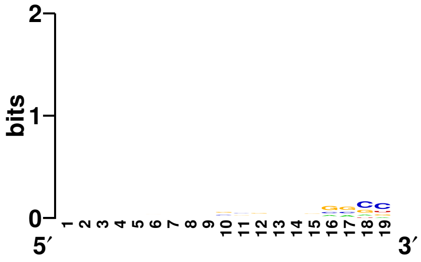

20-mers:

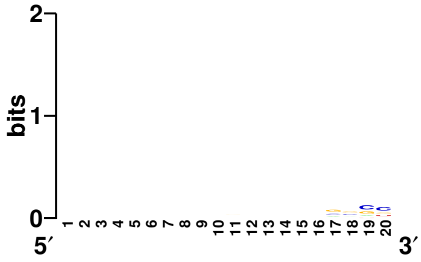

21-mers:

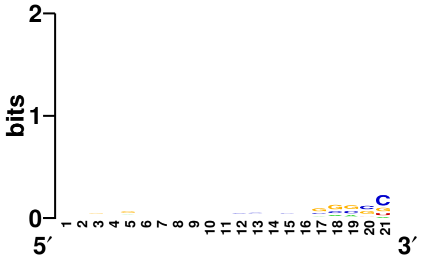

22-mers:

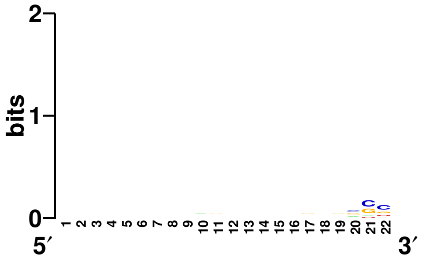

23-mers:

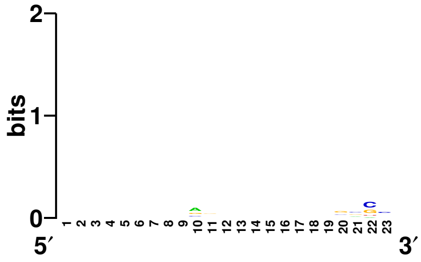

24-mers:

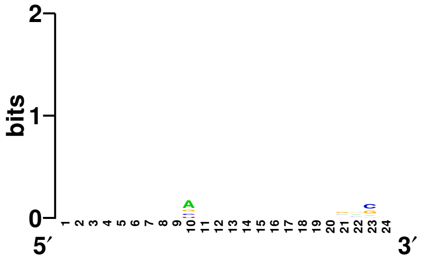

25-mers:

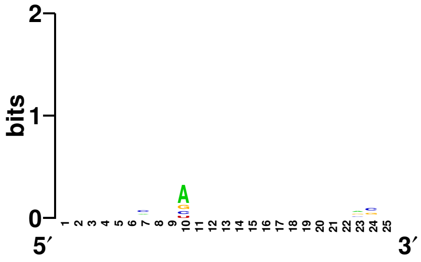

26-mers:

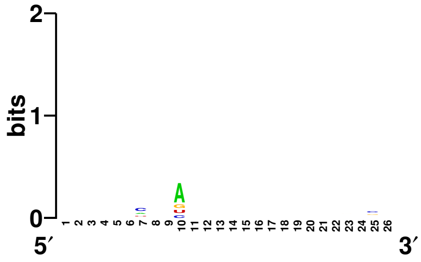

27-mers:

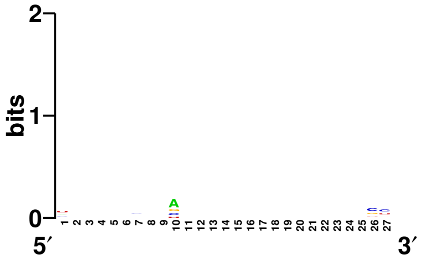

28-mers:

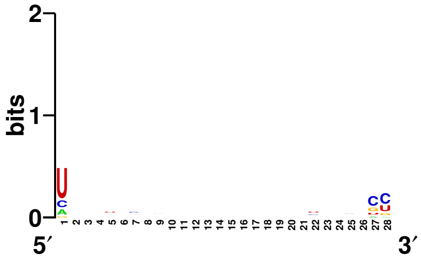

29-mers:

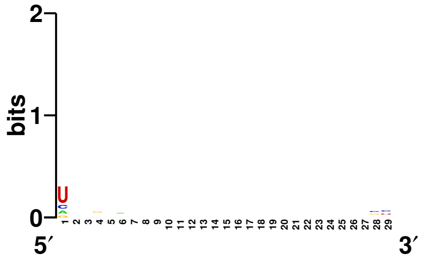

30-mers:

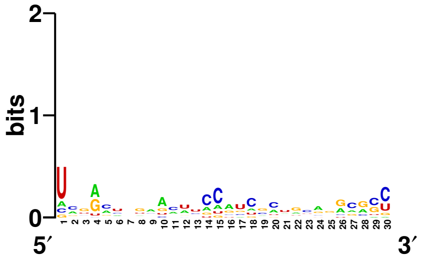

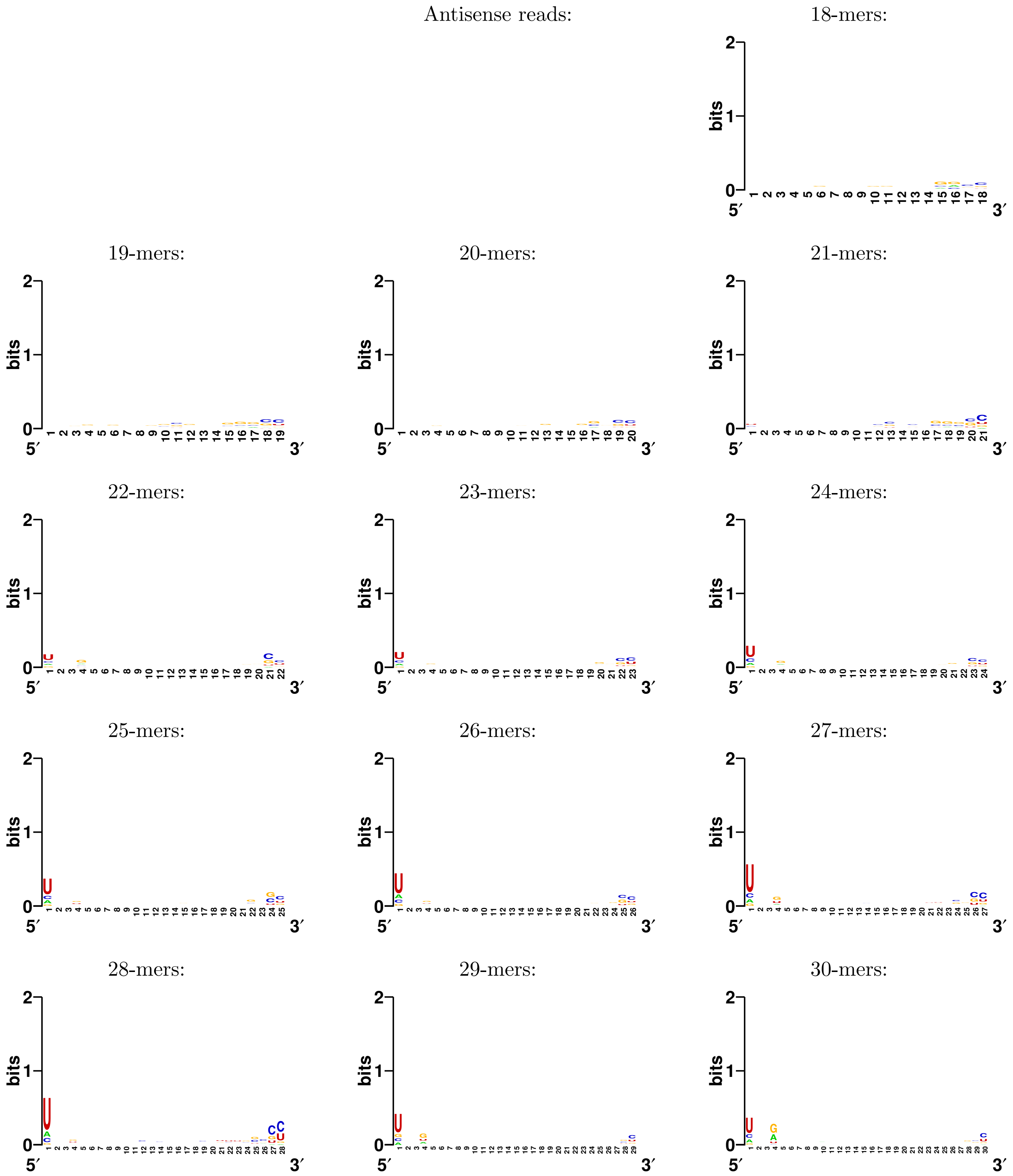
Antisense reads:
18-mers:
19-mers:
20-mers:
21-mers:
22-mers:
23-mers:
24-mers:
25-mers:
26-mers:
27-mers:
28-mers:
29-mers:
30-mers:
bits
5′
3′

Embryo 15h, library 1:

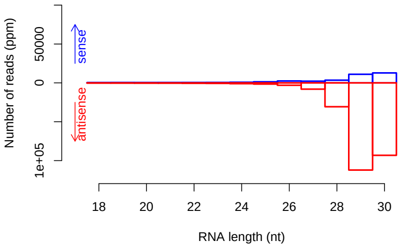

Sense reads:

18-mers:

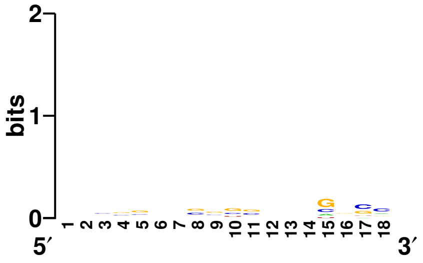

19-mers:

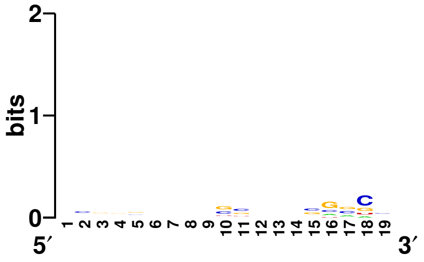

20-mers:

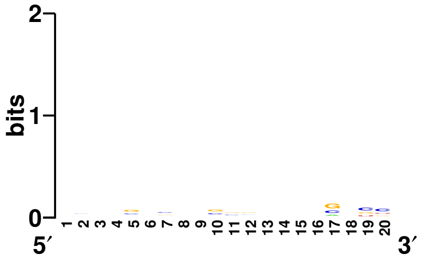

21-mers:

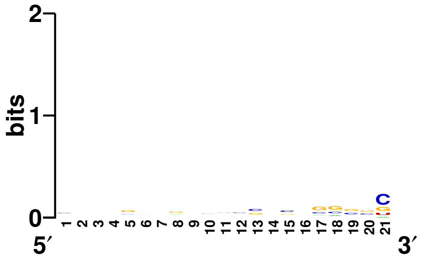

22-mers:

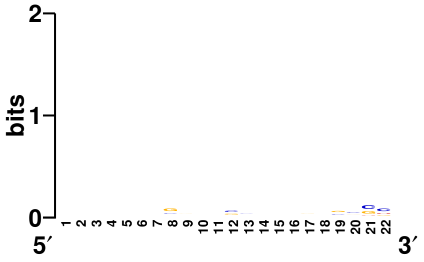

23-mers:

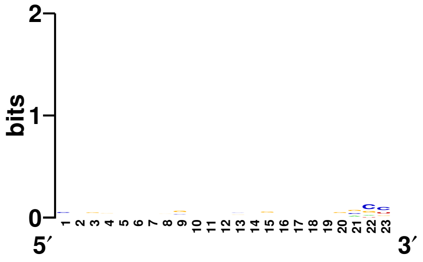

24-mers:

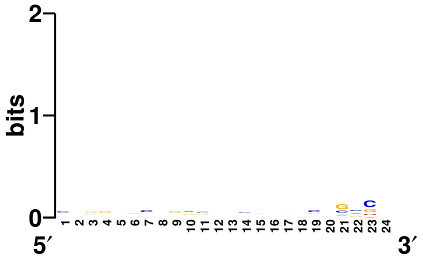

25-mers:

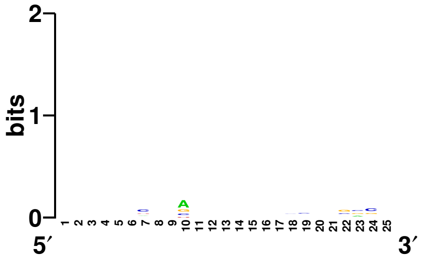

26-mers:

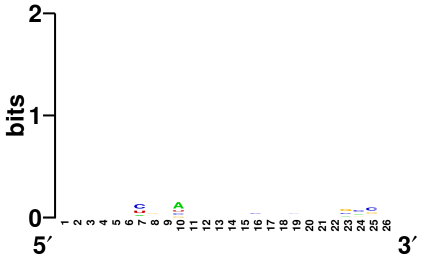

27-mers:

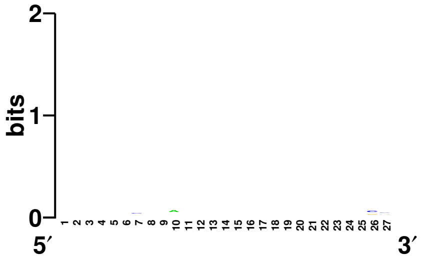

28-mers:

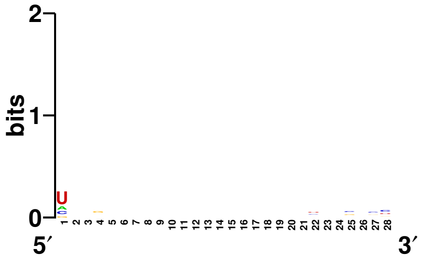

29-mers:

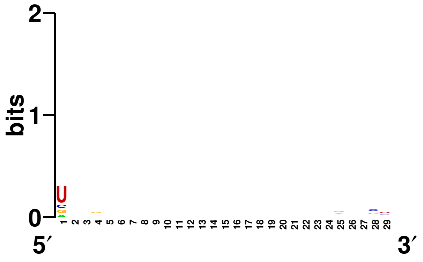

30-mers:

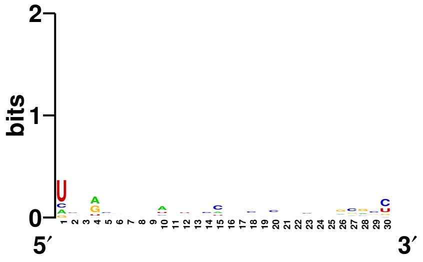

Antisense reads:

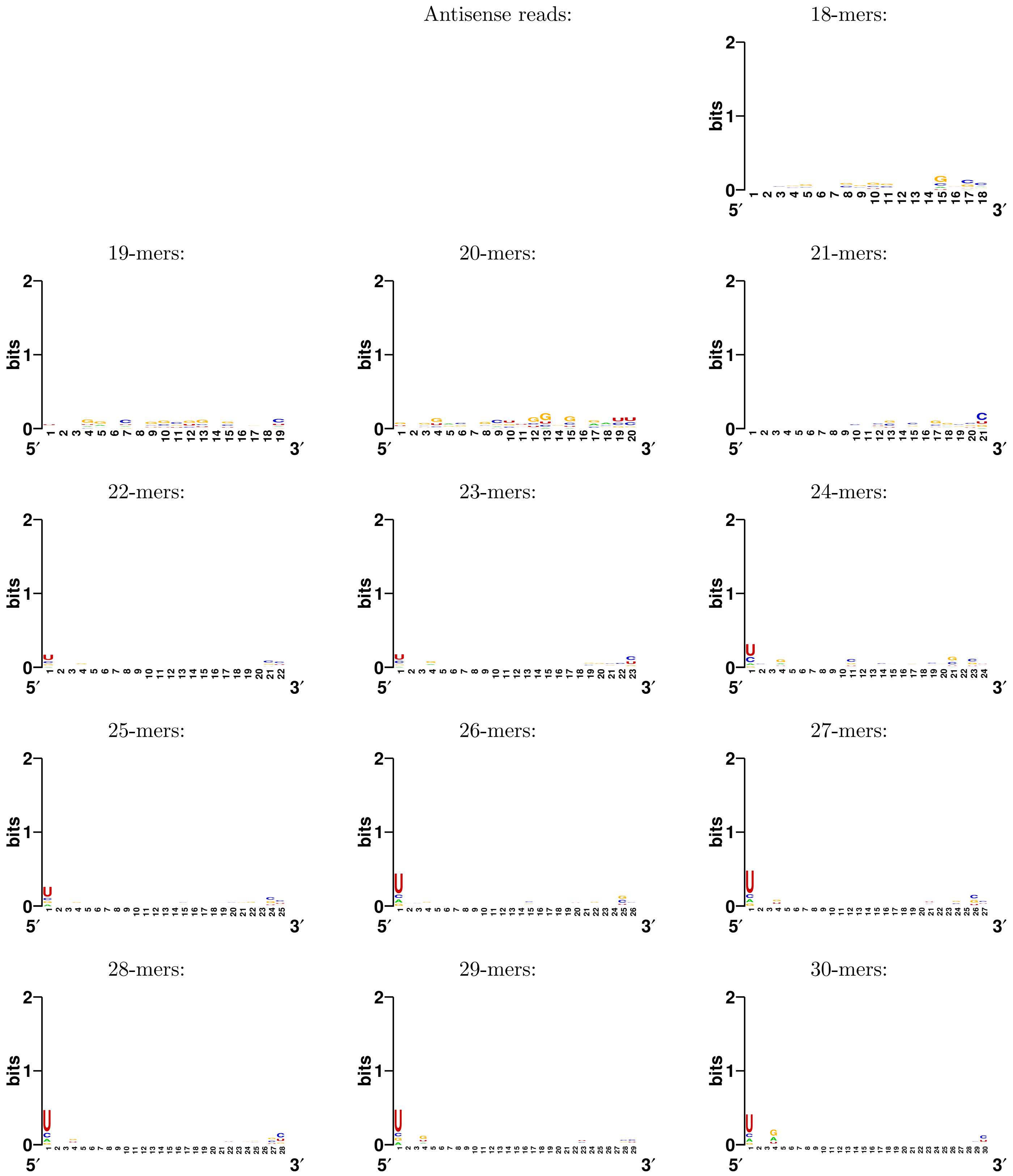

Embryo 36h, library 1:

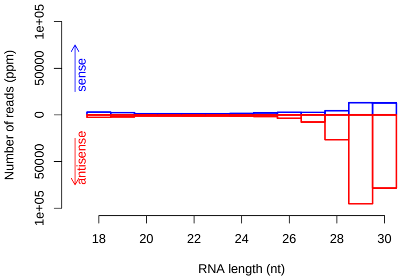

Sense reads:

18-mers:

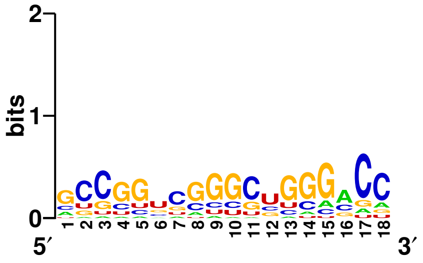

19-mers:

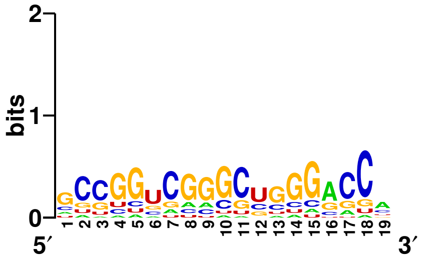

20-mers:

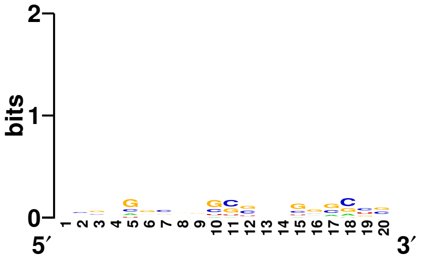

21-mers:

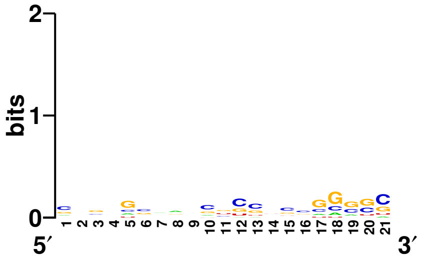

22-mers:

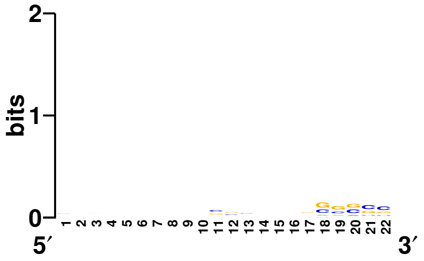

23-mers:

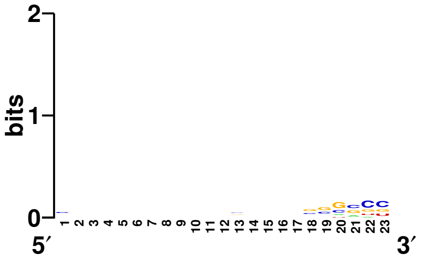

24-mers:

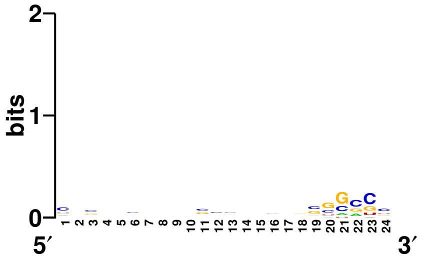

25-mers:

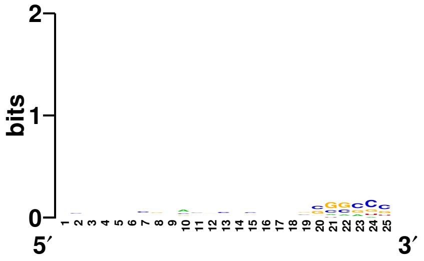

26-mers:

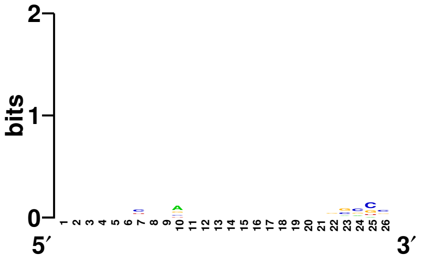

27-mers:

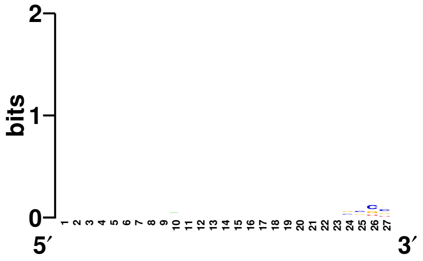

28-mers:

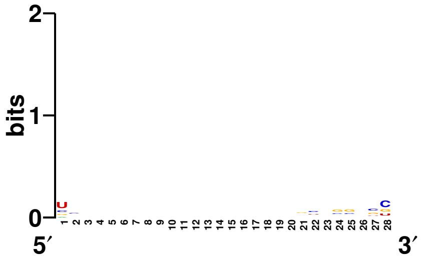

29-mers:

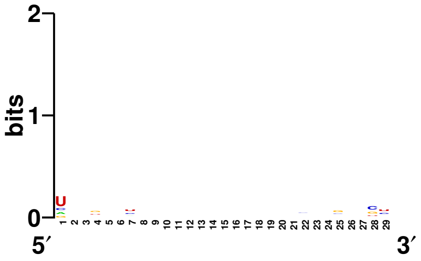

30-mers:

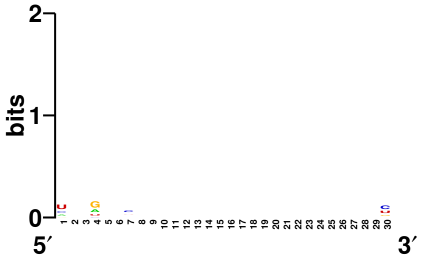

Antisense reads:

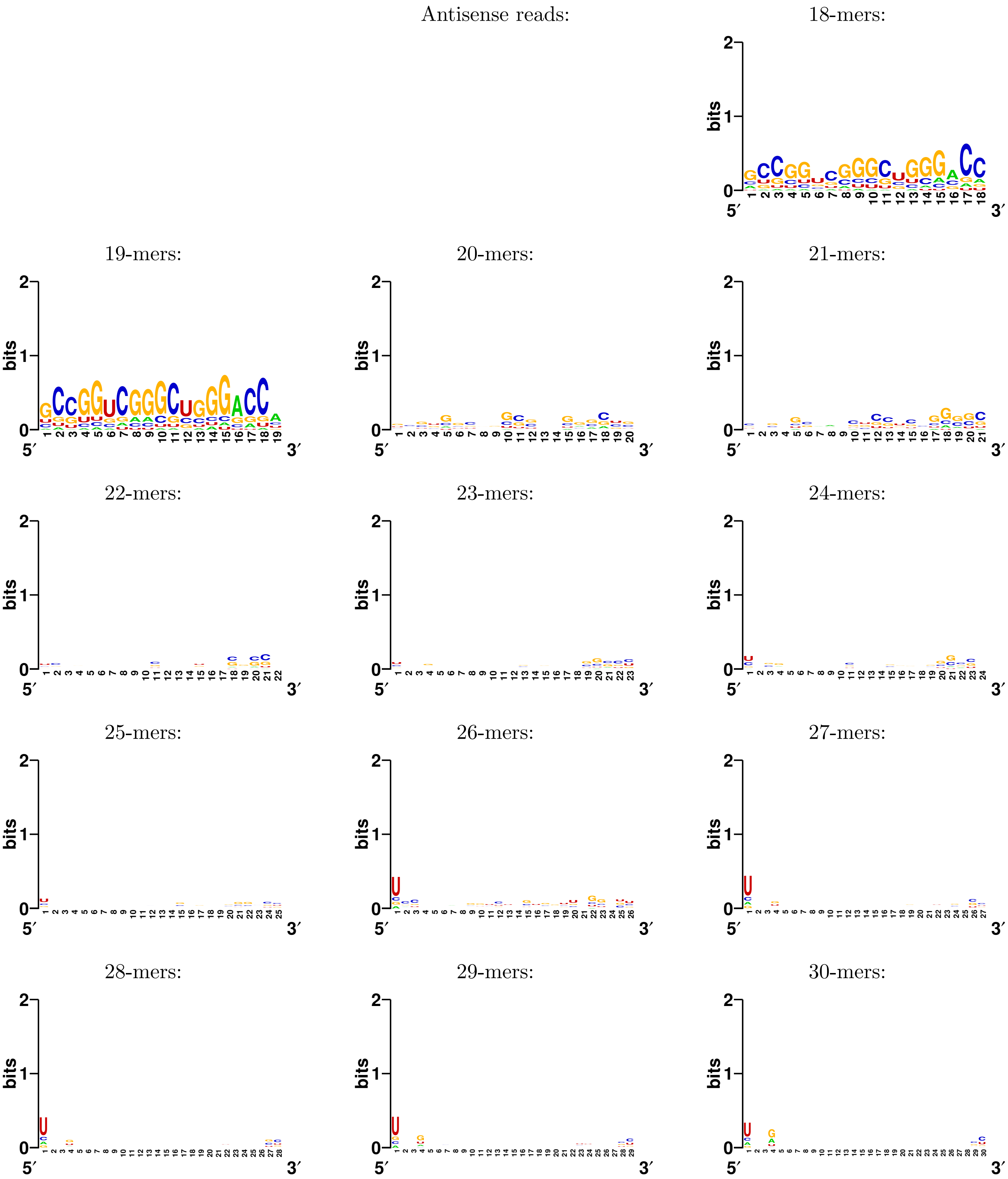

Embryo 60h, library 1:

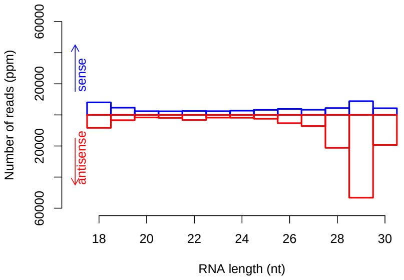

Sense reads:

18-mers:

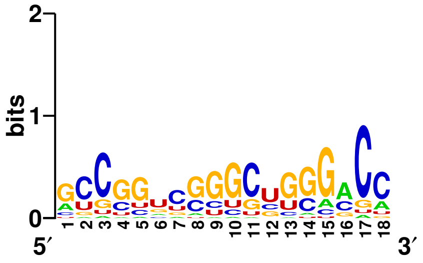

19-mers:

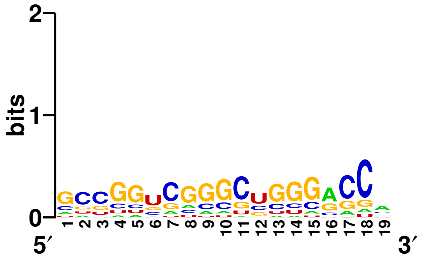

20-mers:

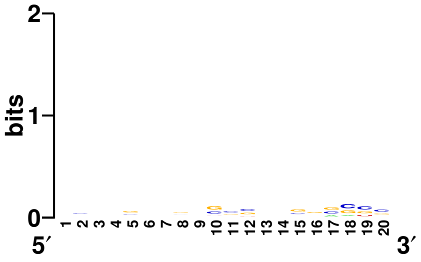

21-mers:

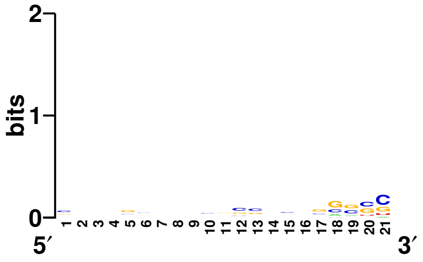

22-mers:

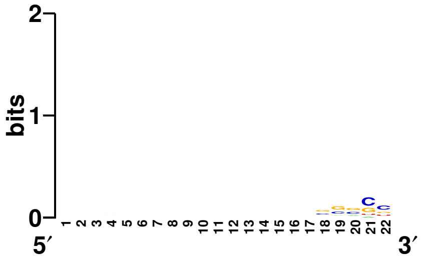

23-mers:

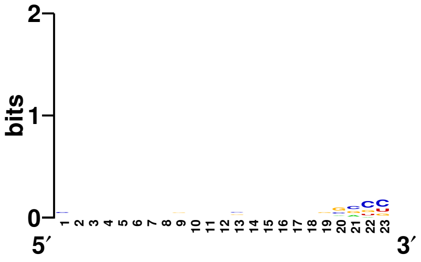

24-mers:

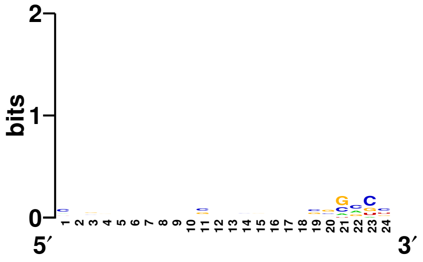

25-mers:

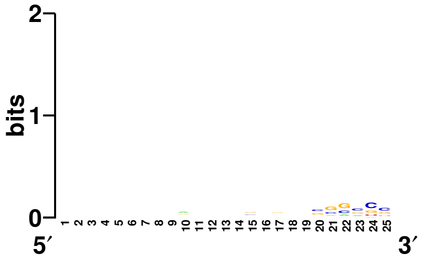

26-mers:

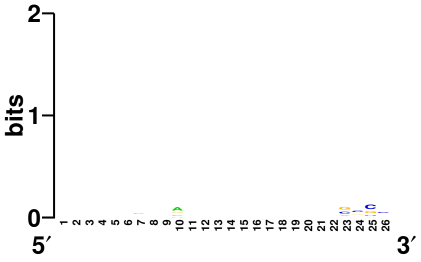

27-mers:

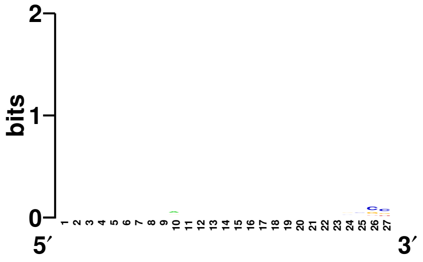

28-mers:

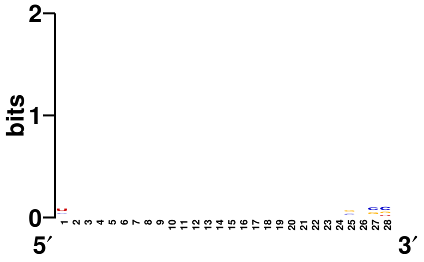

29-mers:

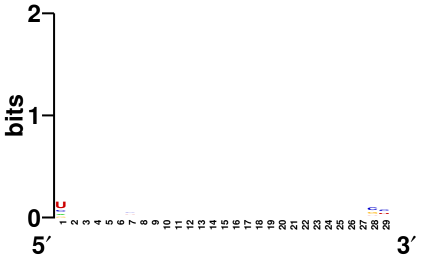

30-mers:

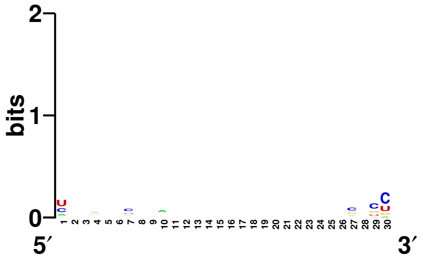

Antisense reads:

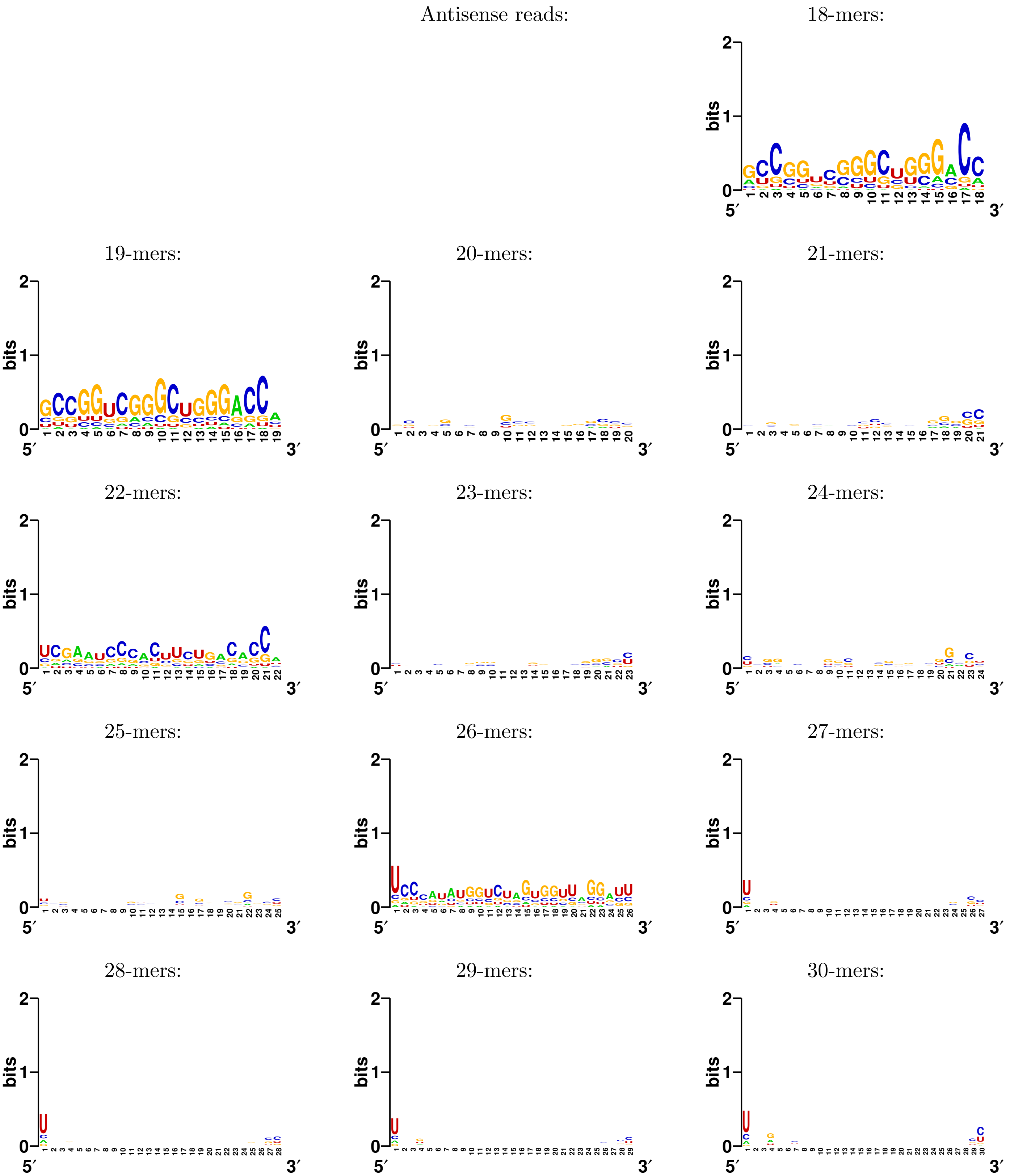

Adult female, library 1:

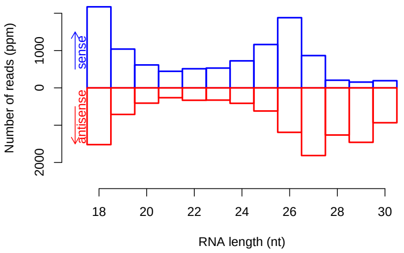

Sense reads:

18-mers:

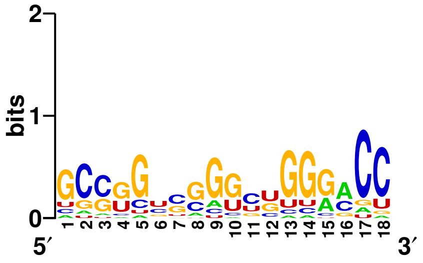

19-mers:

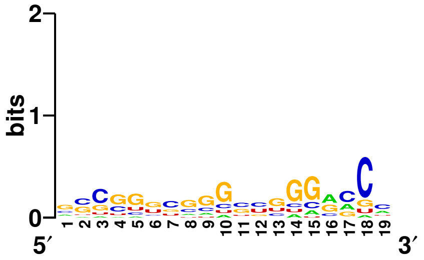

20-mers:

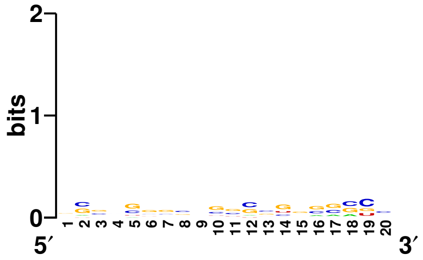

21-mers:

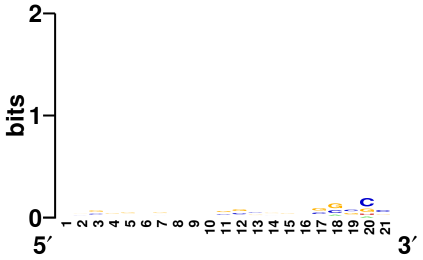

22-mers:

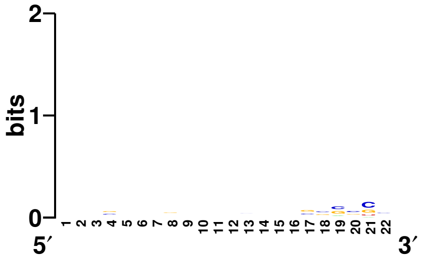

23-mers:

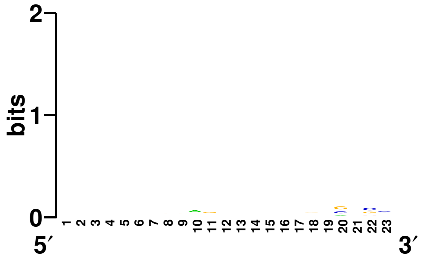

24-mers:

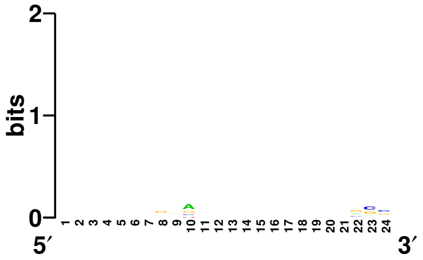

25-mers:

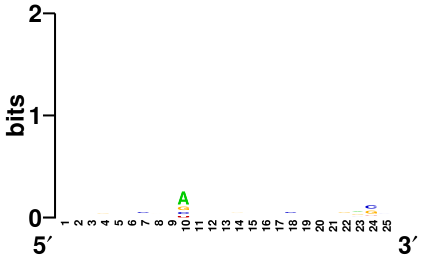

26-mers:

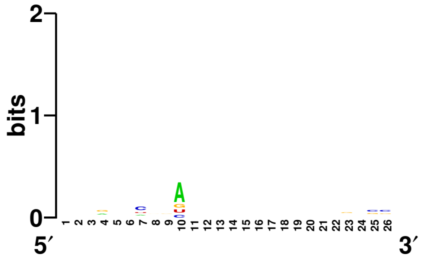

27-mers:

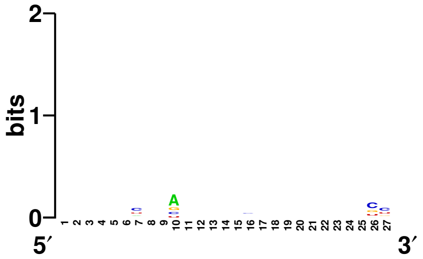

28-mers:

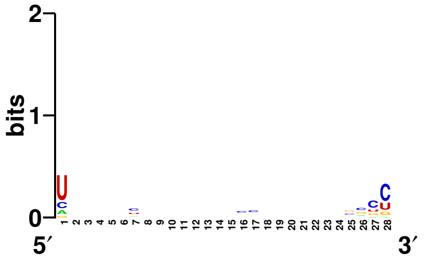

29-mers:

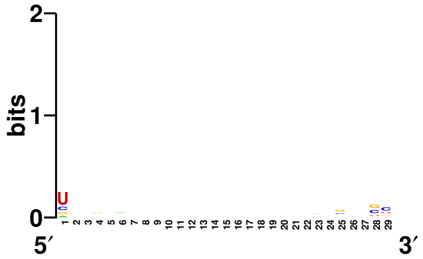

30-mers:

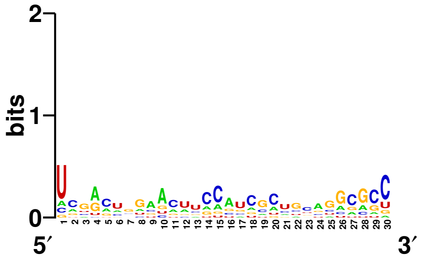

Antisense reads:

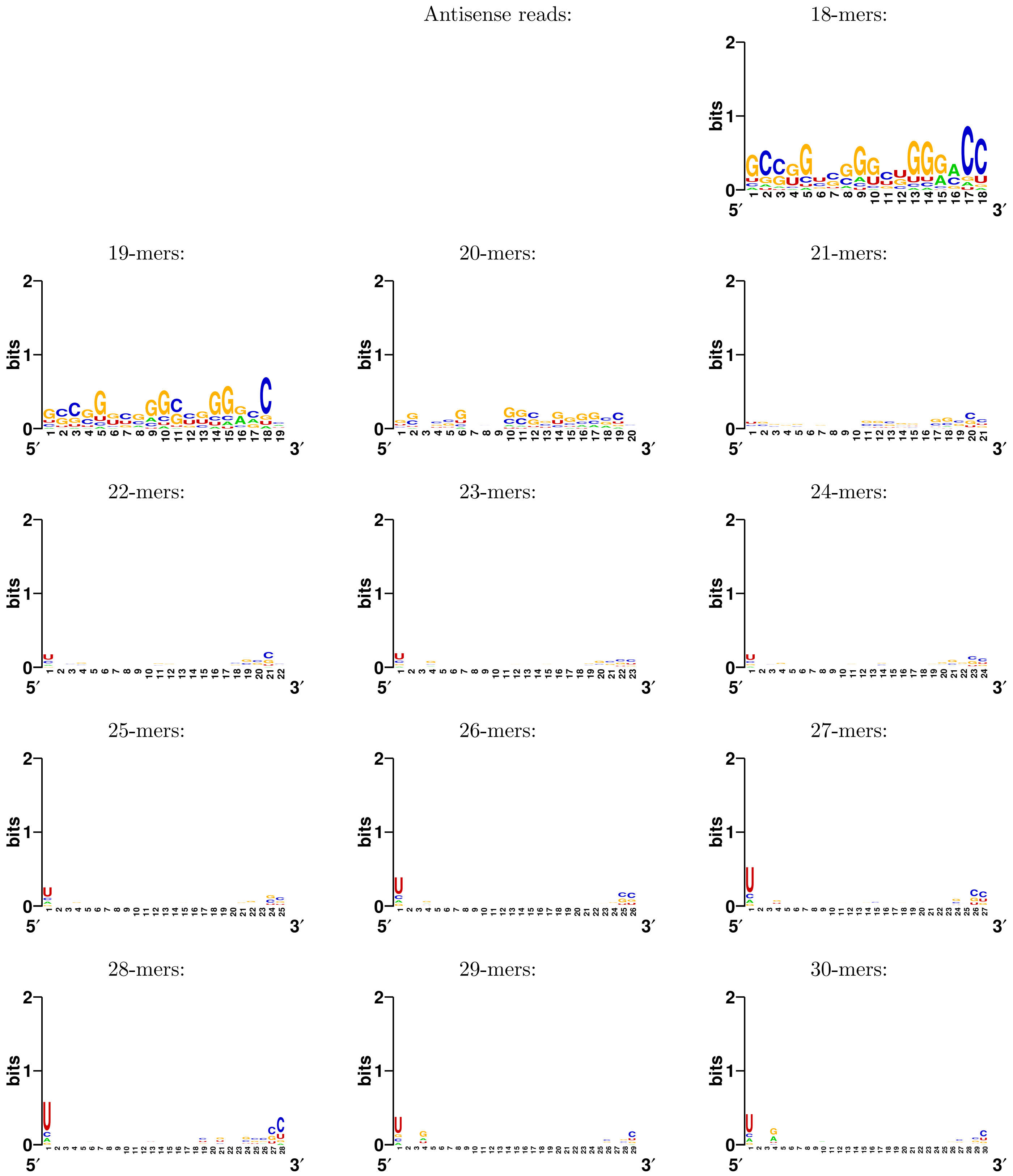

Adult male, library 1:

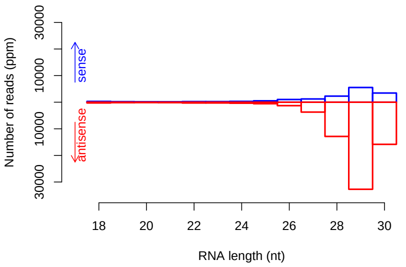

Sense reads:

18-mers:

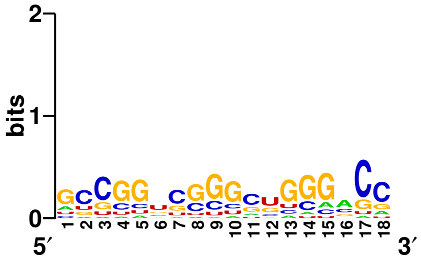

19-mers:

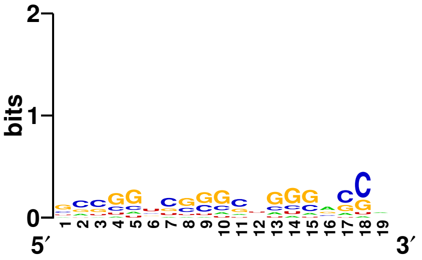

20-mers:

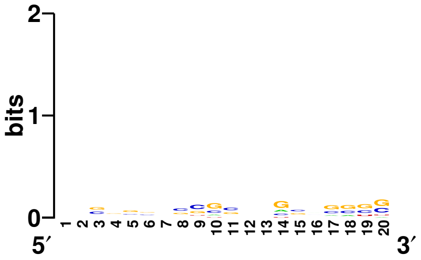

21-mers:

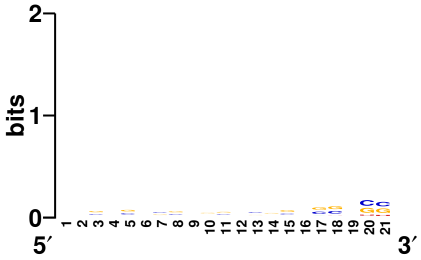

22-mers:

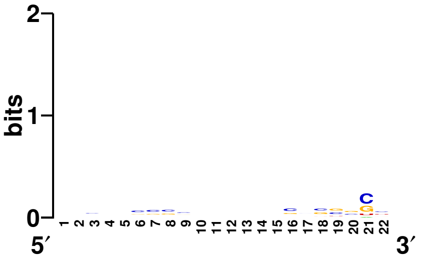

23-mers:

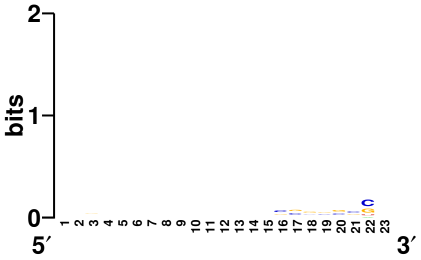

24-mers:

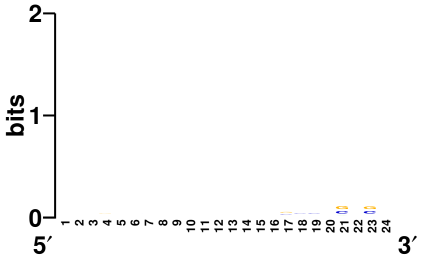

25-mers:

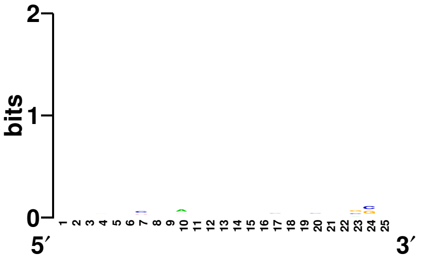

26-mers:

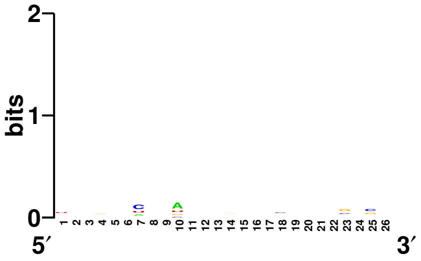

27-mers:

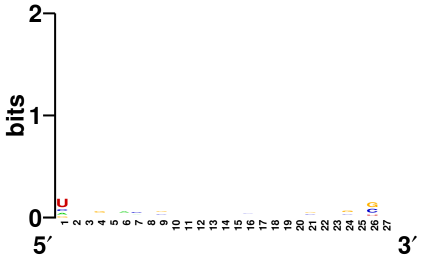

28-mers:

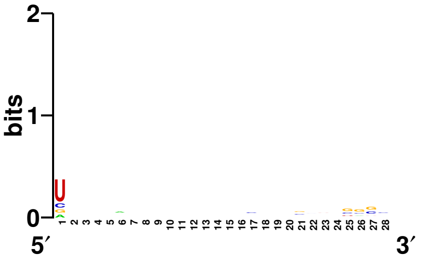

29-mers:

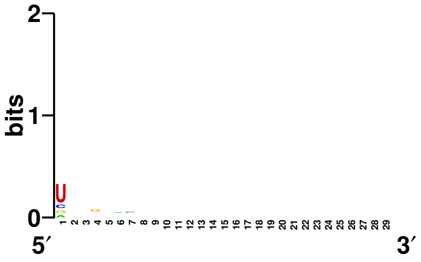

30-mers:

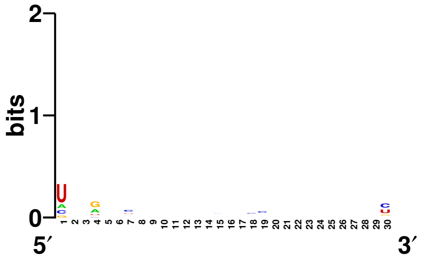

Antisense reads:

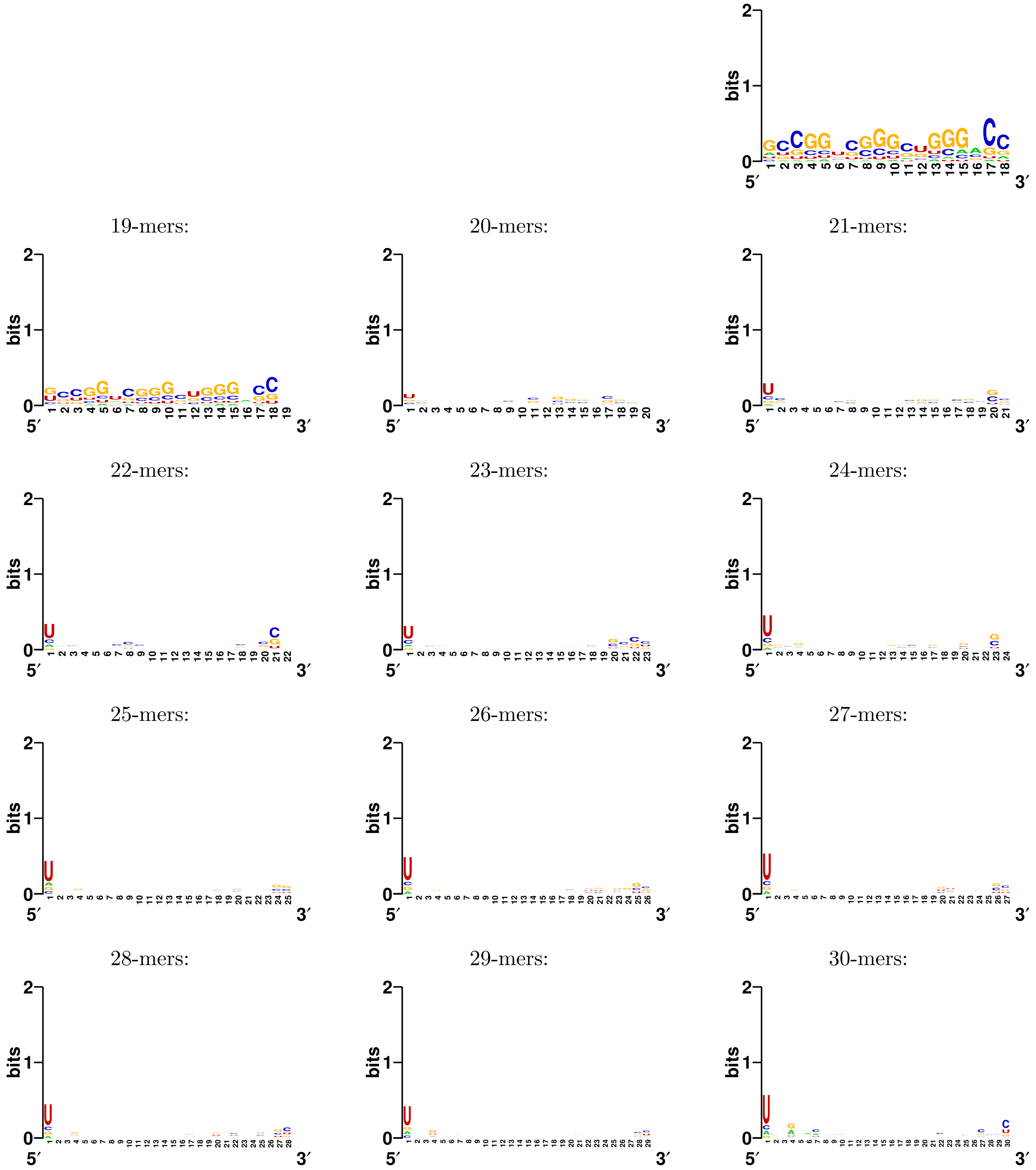

4.2 Libraries #2 (3´ modified, 5´ monophosphorylated small RNAs)

Embryo 8h, library 2:

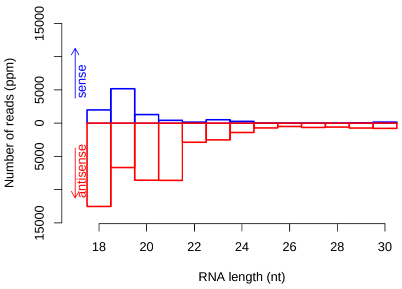

Sense reads:

18-mers:

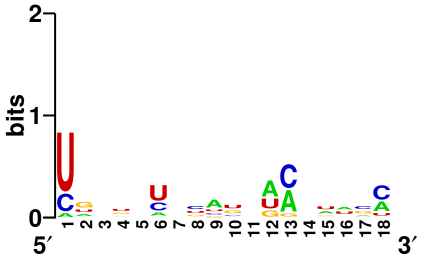

19-mers:

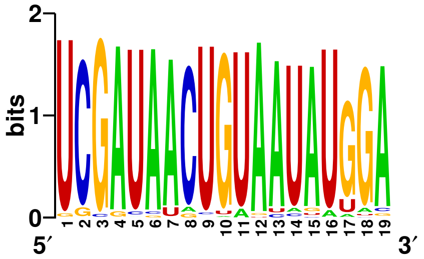

20-mers:

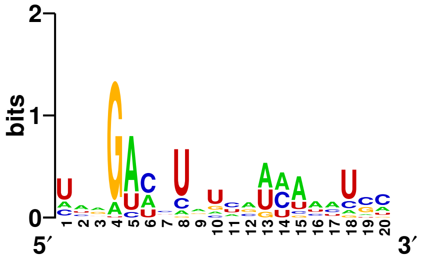

21-mers:

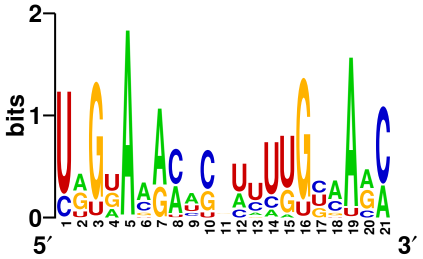

22-mers:

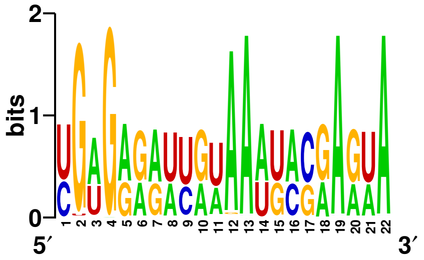

23-mers:

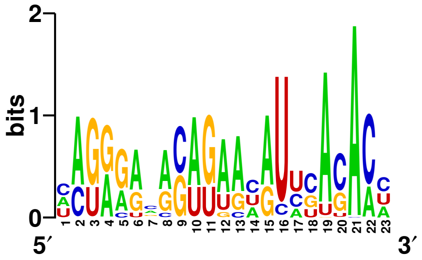

24-mers:

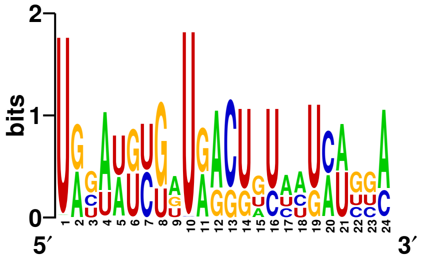

25-mers:

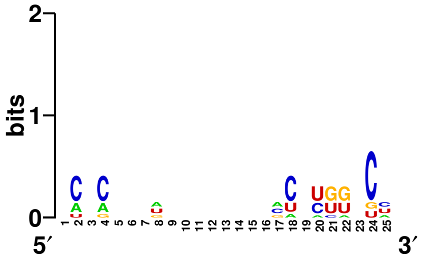

26-mers:

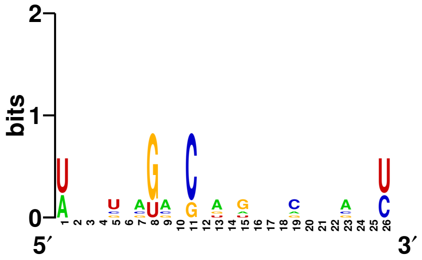

27-mers:

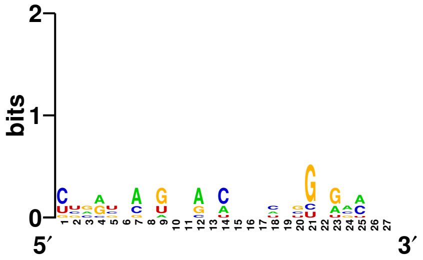

28-mers:

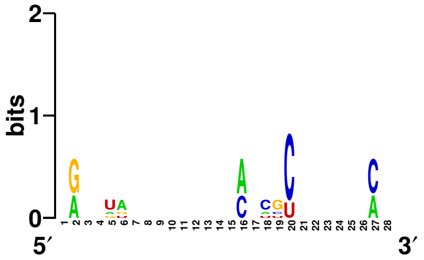

29-mers:

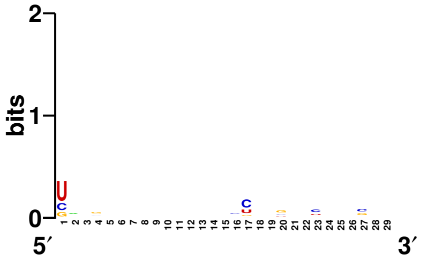

30-mers:

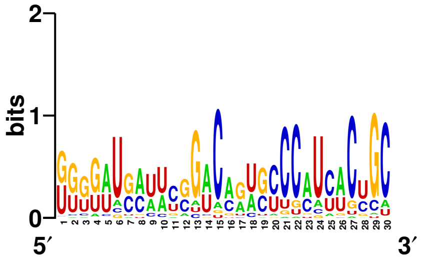

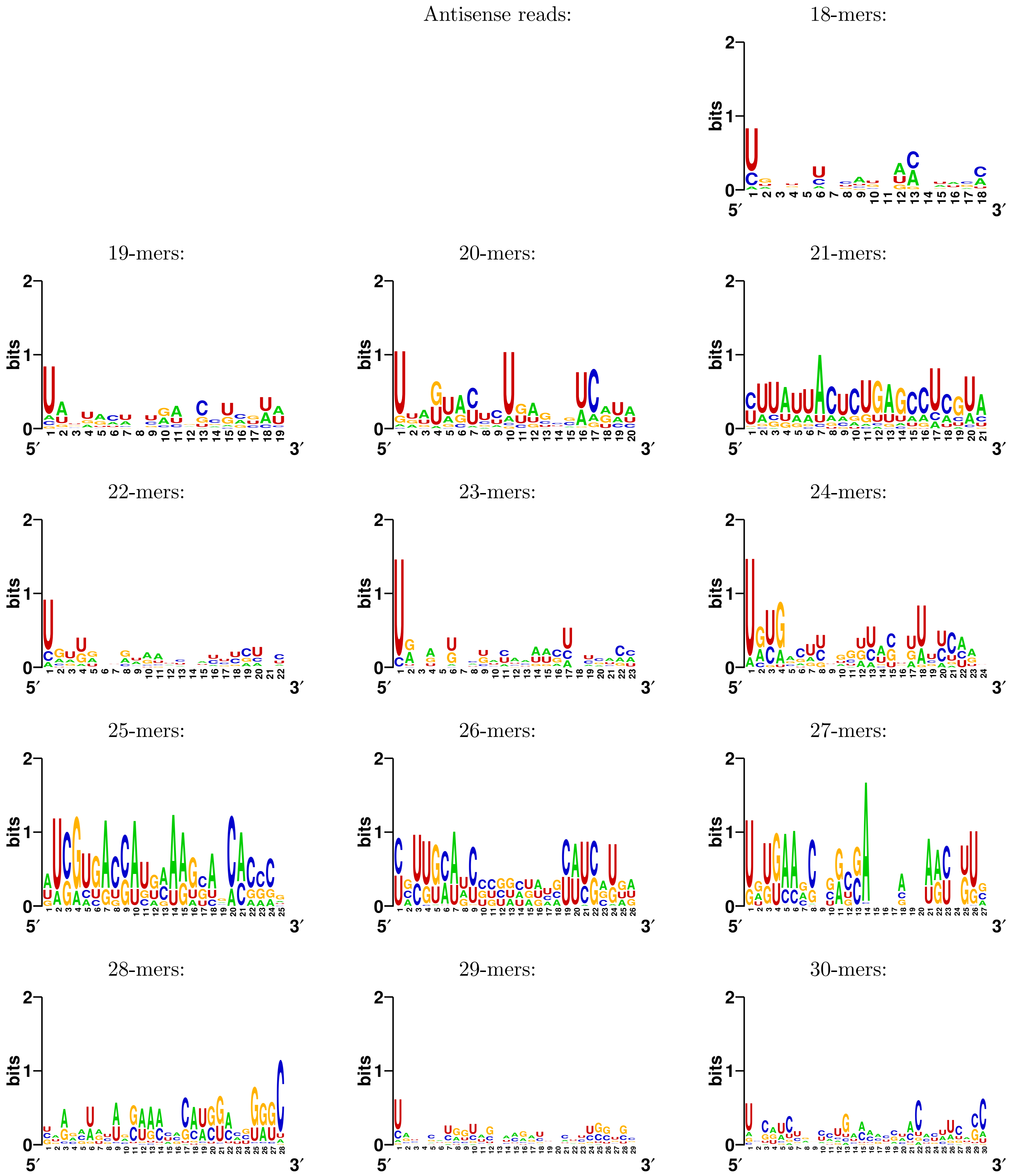
Antisense reads:
18-mers:
19-mers:
20-mers:
21-mers:
22-mers:
23-mers:
24-mers:
25-mers:
26-mers:
27-mers:
28-mers:
29-mers:
30-mers:
bits
5′
3′

Embryo 15h, library 2:

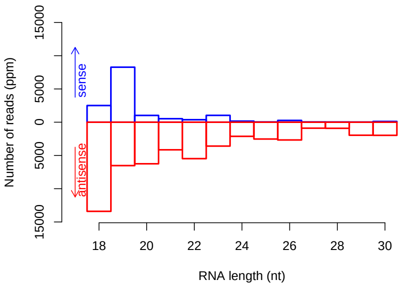

Sense reads:

18-mers:

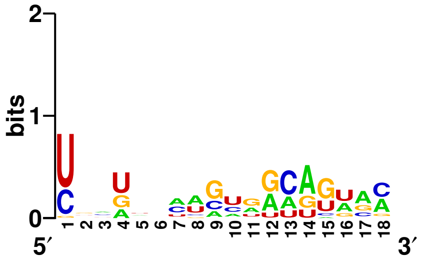

19-mers:

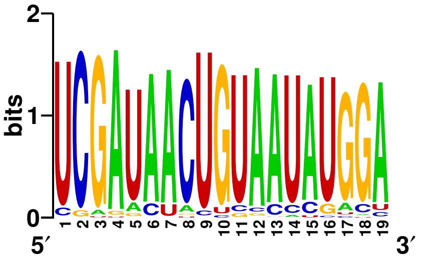

20-mers:

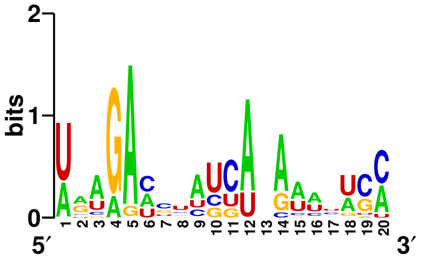

21-mers:

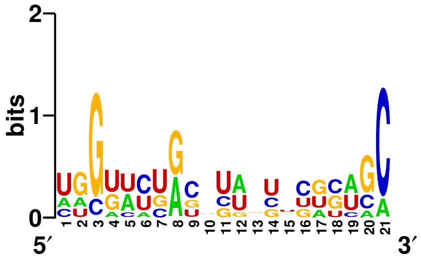

22-mers:

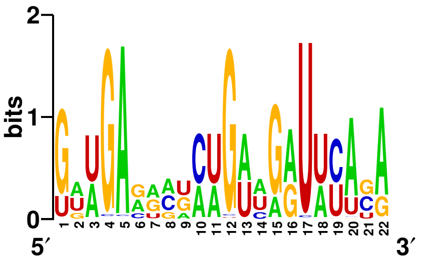

23-mers:

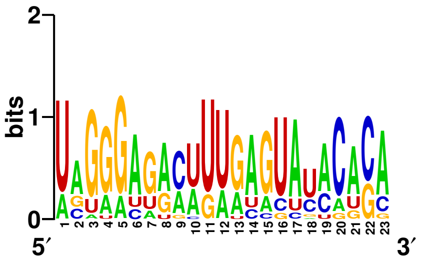

24-mers:

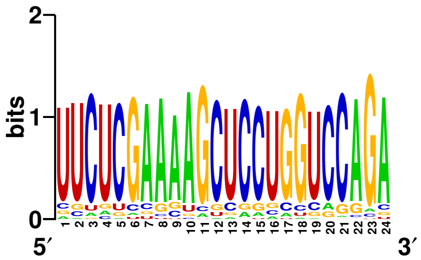

25-mers:

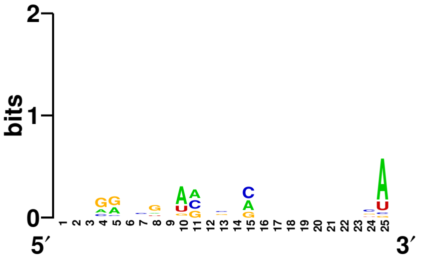

26-mers:

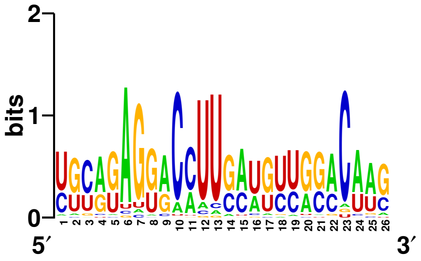

27-mers:

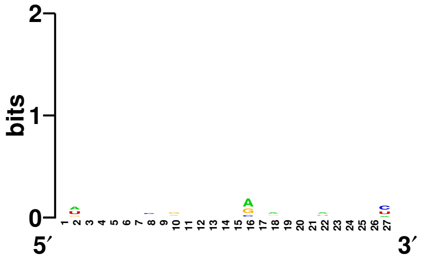

28-mers:

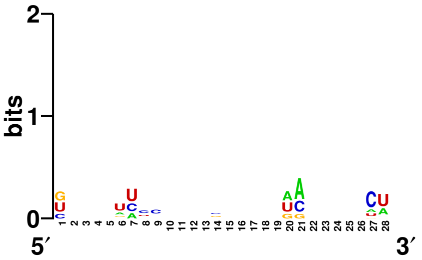

29-mers:

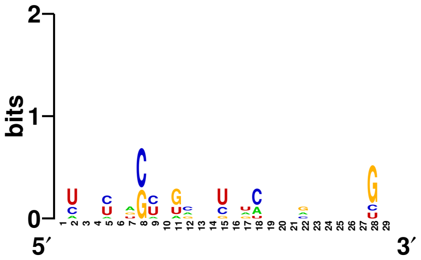

30-mers:

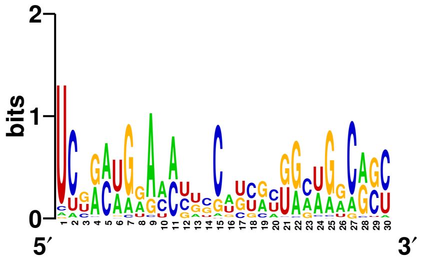

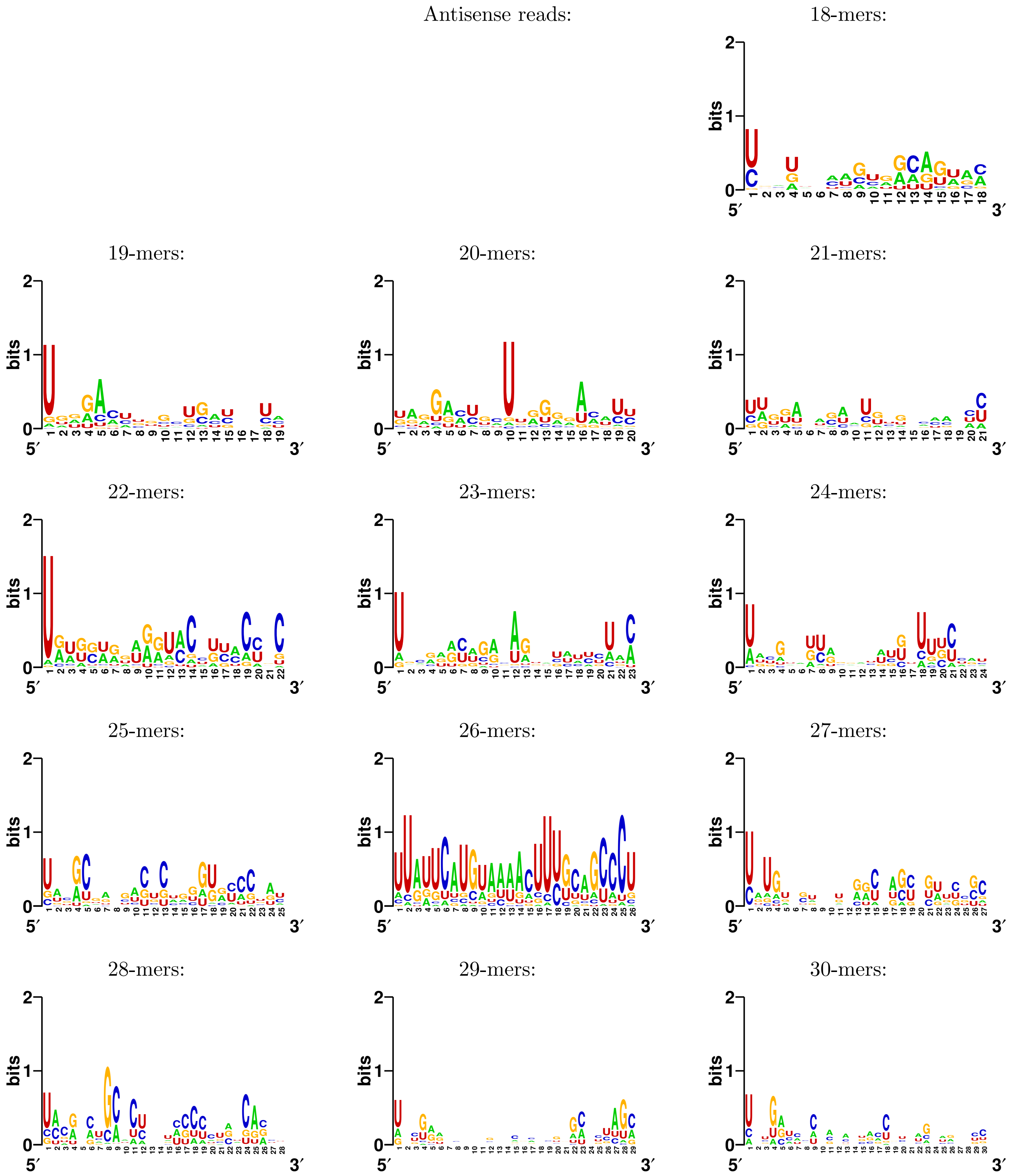
Antisense reads:
18-mers:
19-mers:
20-mers:
21-mers:
22-mers:
23-mers:
24-mers:
25-mers:
26-mers:
27-mers:
28-mers:
29-mers:
30-mers:
bits
5′
3′

Embryo 36h, library 2:

Sense reads:

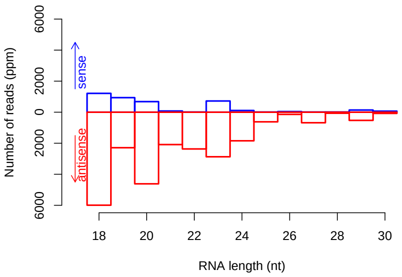

18-mers:

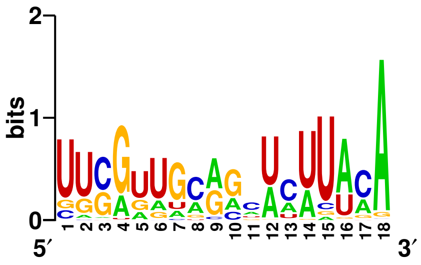

19-mers:

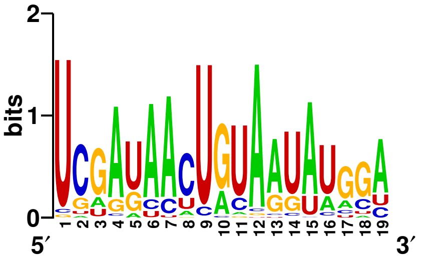

20-mers:

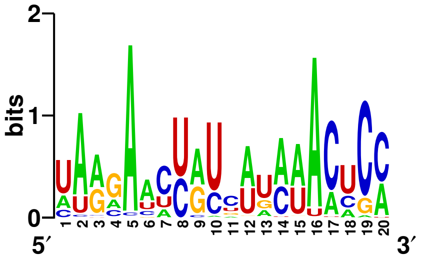

21-mers:

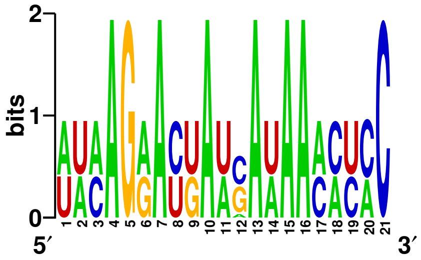

22-mers:

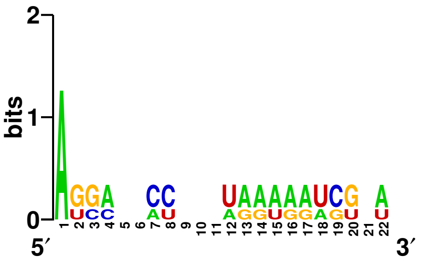

23-mers:

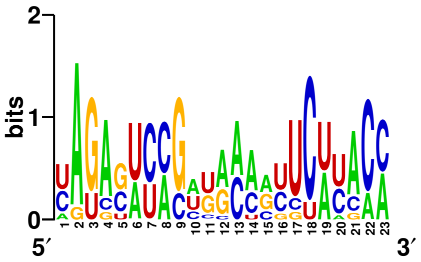

24-mers:

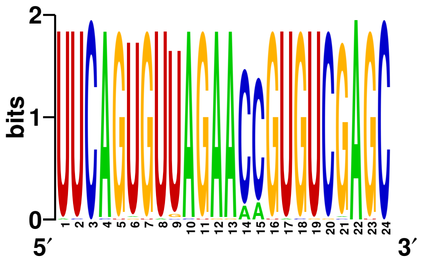

25-mers:

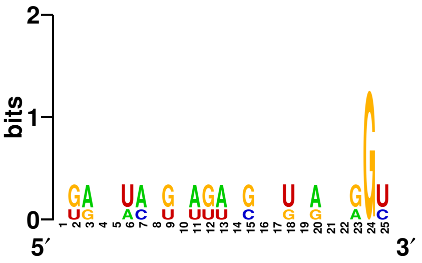

26-mers:

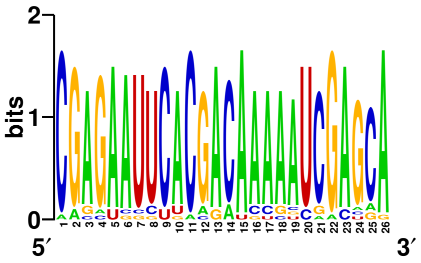

27-mers:

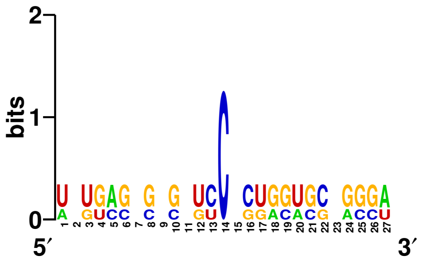

28-mers:

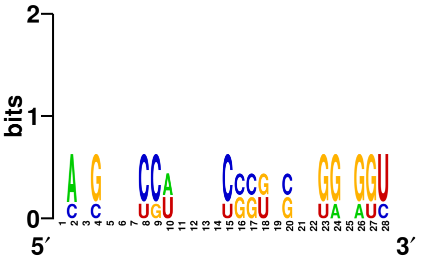

29-mers:

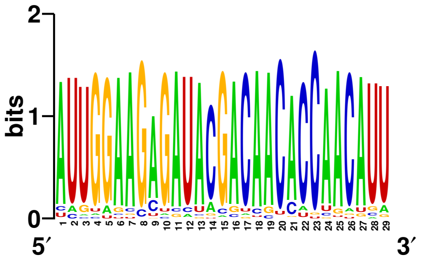

30-mers:

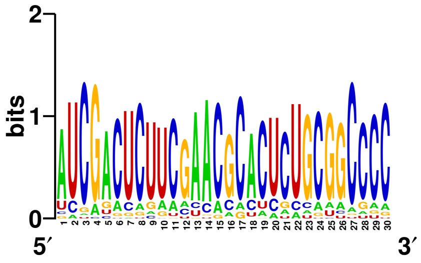

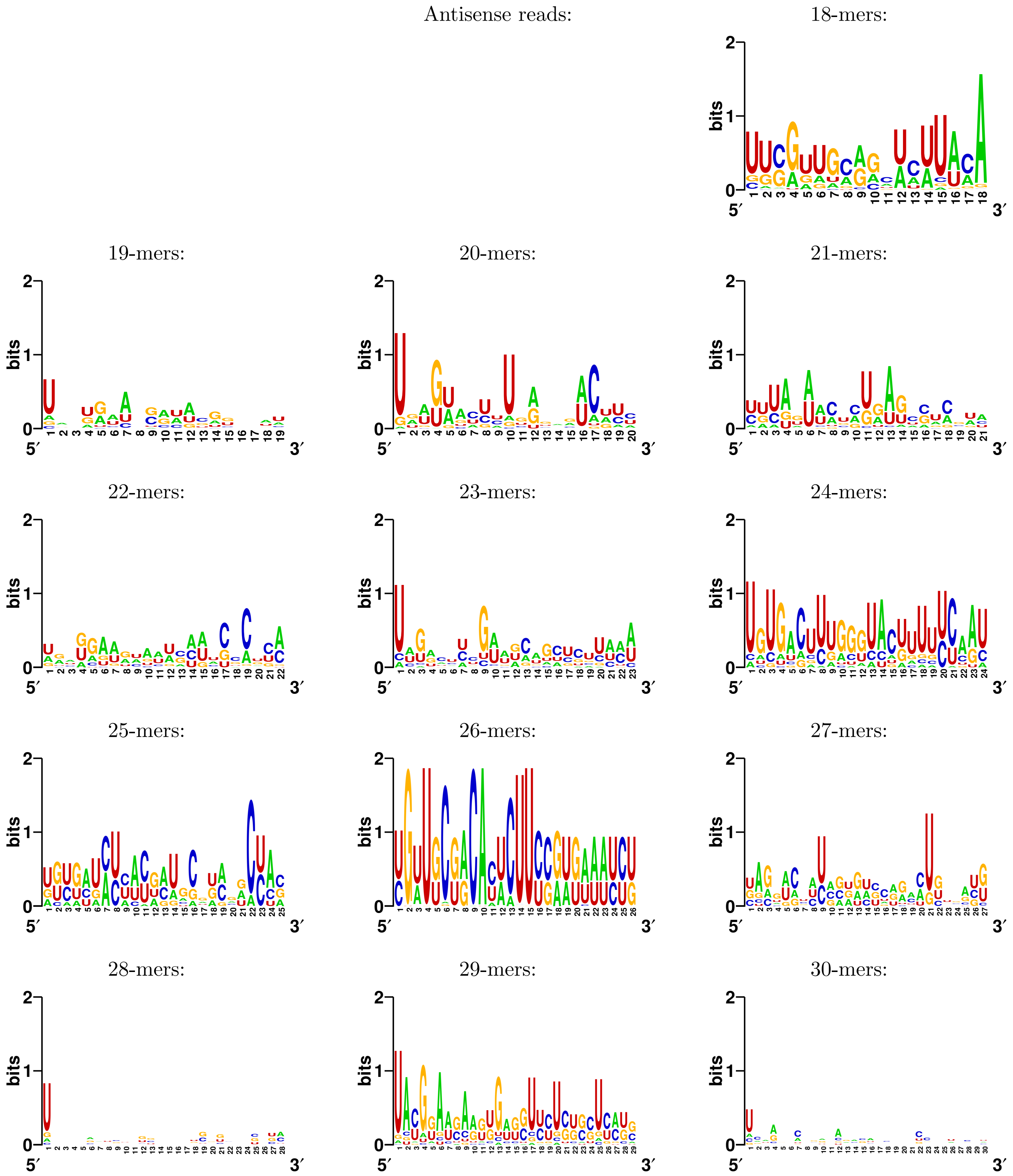
Antisense reads:
18-mers:
19-mers:
20-mers:
21-mers:
22-mers:
23-mers:
24-mers:
25-mers:
26-mers:
27-mers:
28-mers:
29-mers:
30-mers:
bits
5′
3′

Embryo 60h, library 2:

Sense reads:

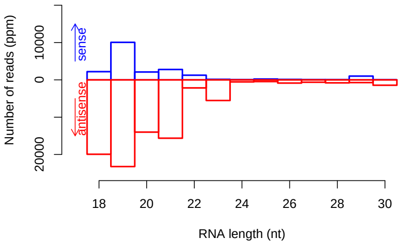

18-mers:

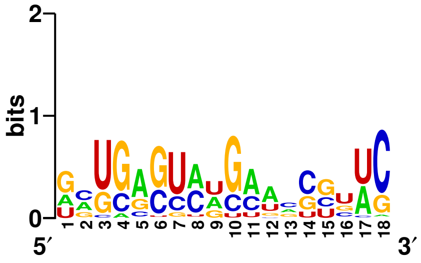

19-mers:

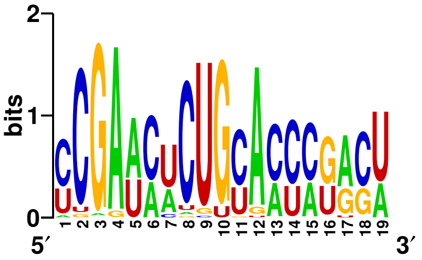

20-mers:

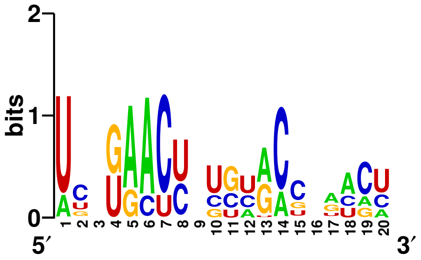

21-mers:

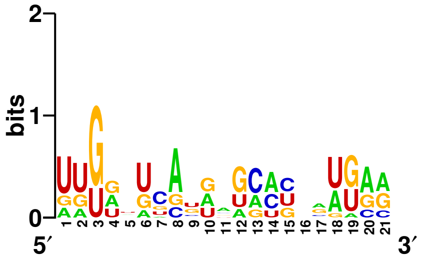

22-mers:

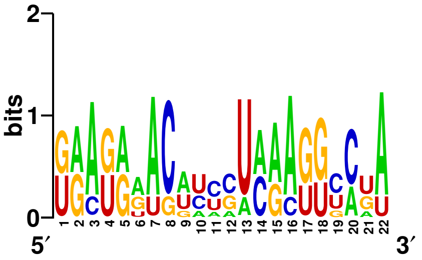

23-mers:

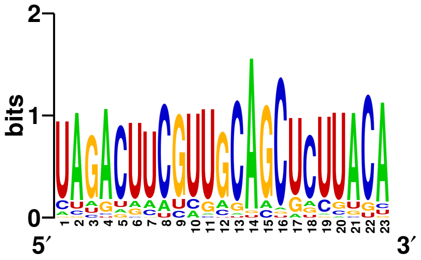

24-mers:

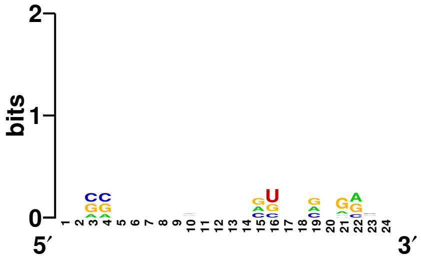

25-mers:

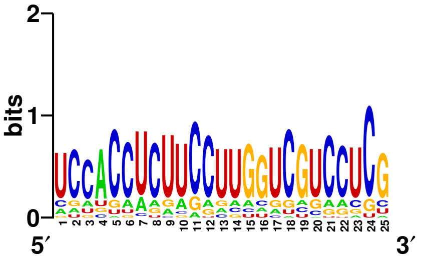

26-mers:

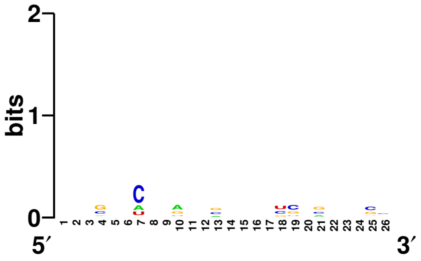

27-mers:

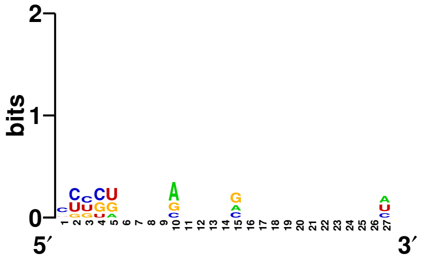

28-mers:

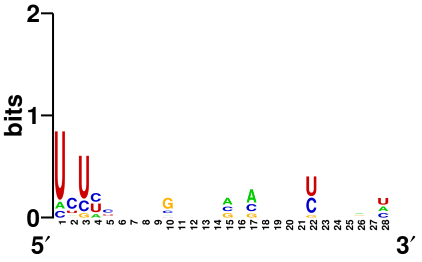

29-mers:

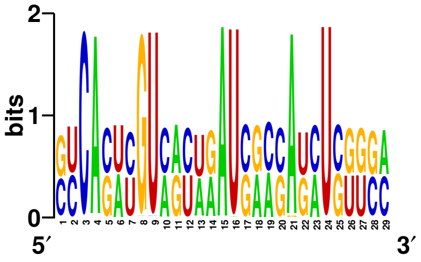

30-mers:

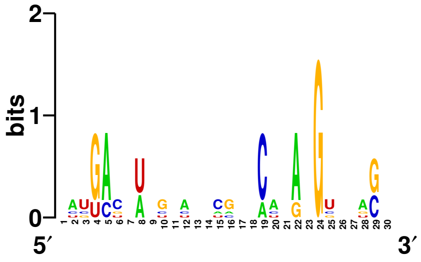

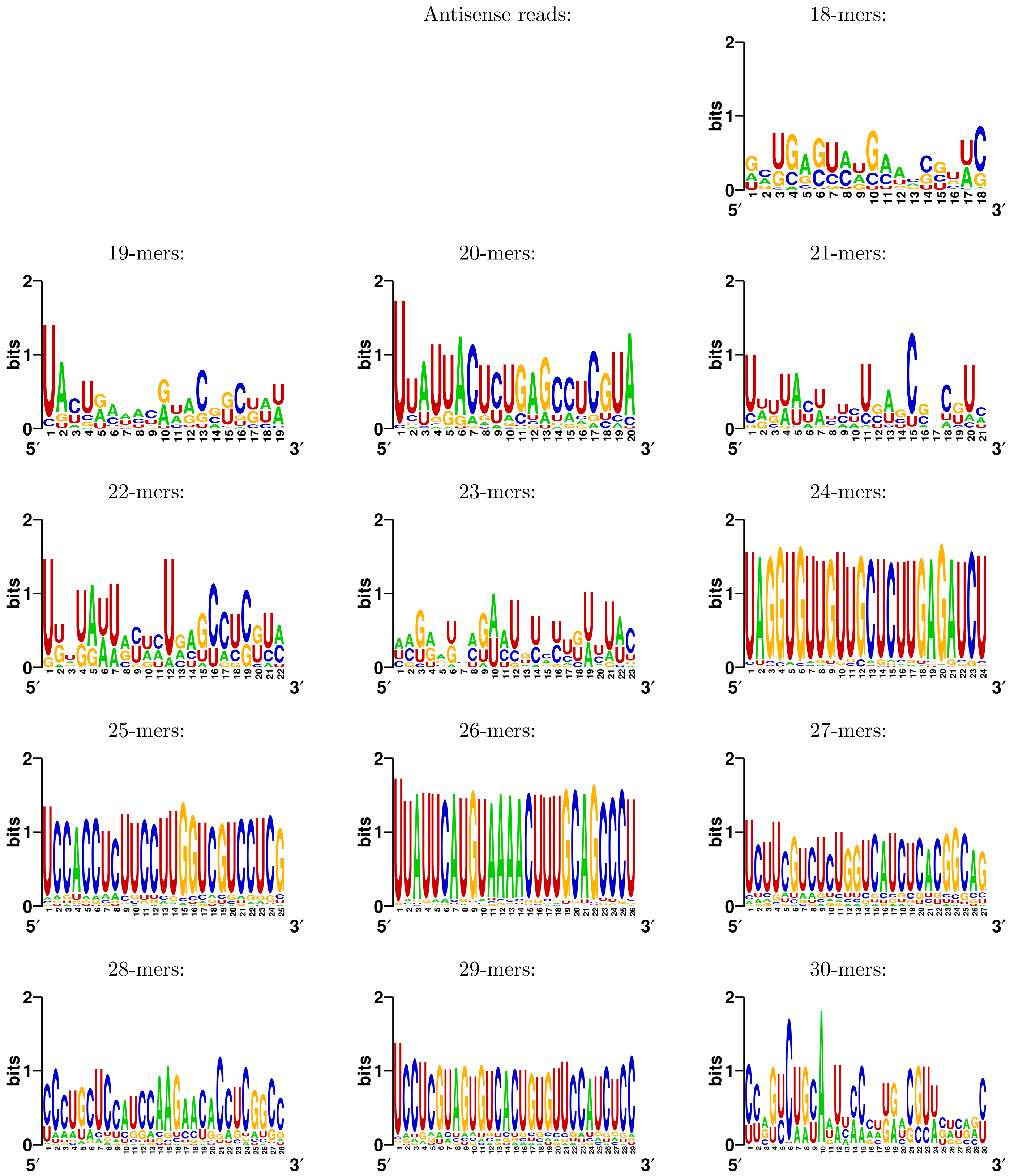
Antisense reads:
18-mers:
19-mers:
20-mers:
21-mers:
22-mers:
23-mers:
24-mers:
25-mers:
26-mers:
27-mers:
28-mers:
29-mers:
30-mers:
bits
5′
3′

Adult female, library 2:

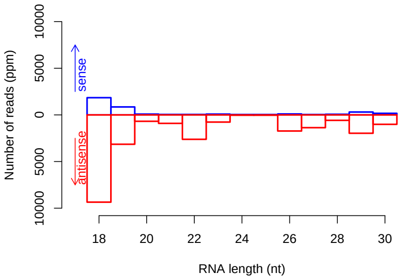

Sense reads:

18-mers:

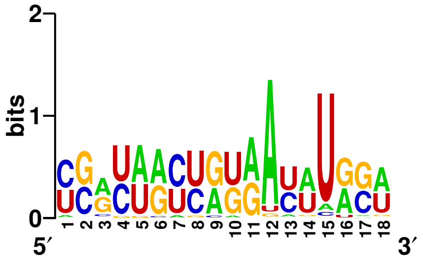

19-mers:

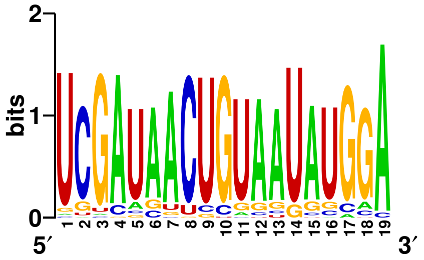

20-mers:

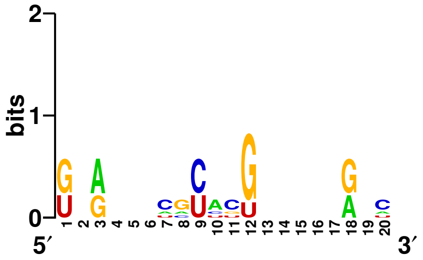

21-mers:

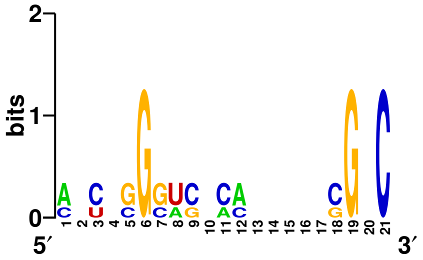

22-mers:

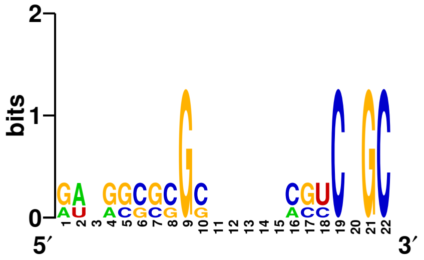

23-mers:

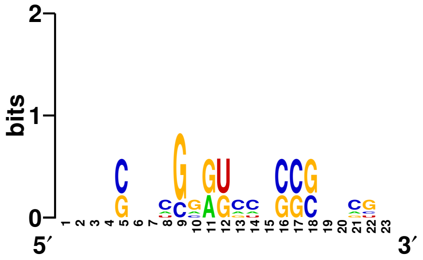

24-mers:

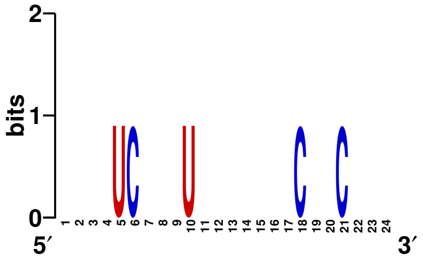

25-mers:

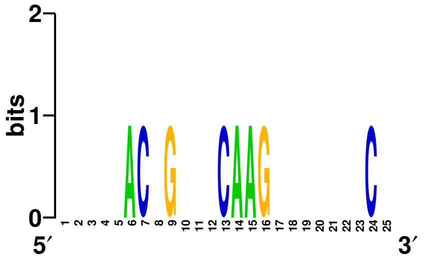

26-mers:

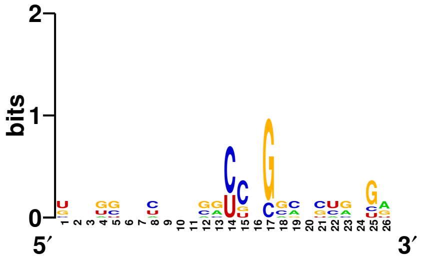

27-mers:

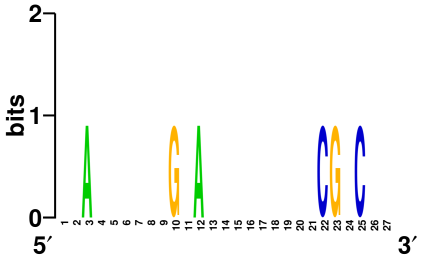

28-mers:

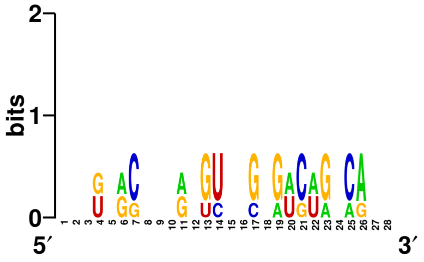

29-mers:

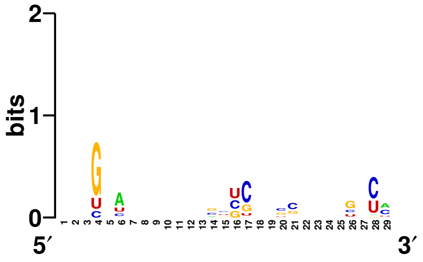

30-mers:

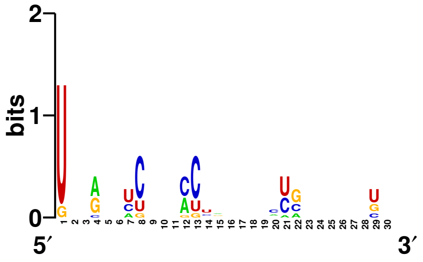

Antisense reads:

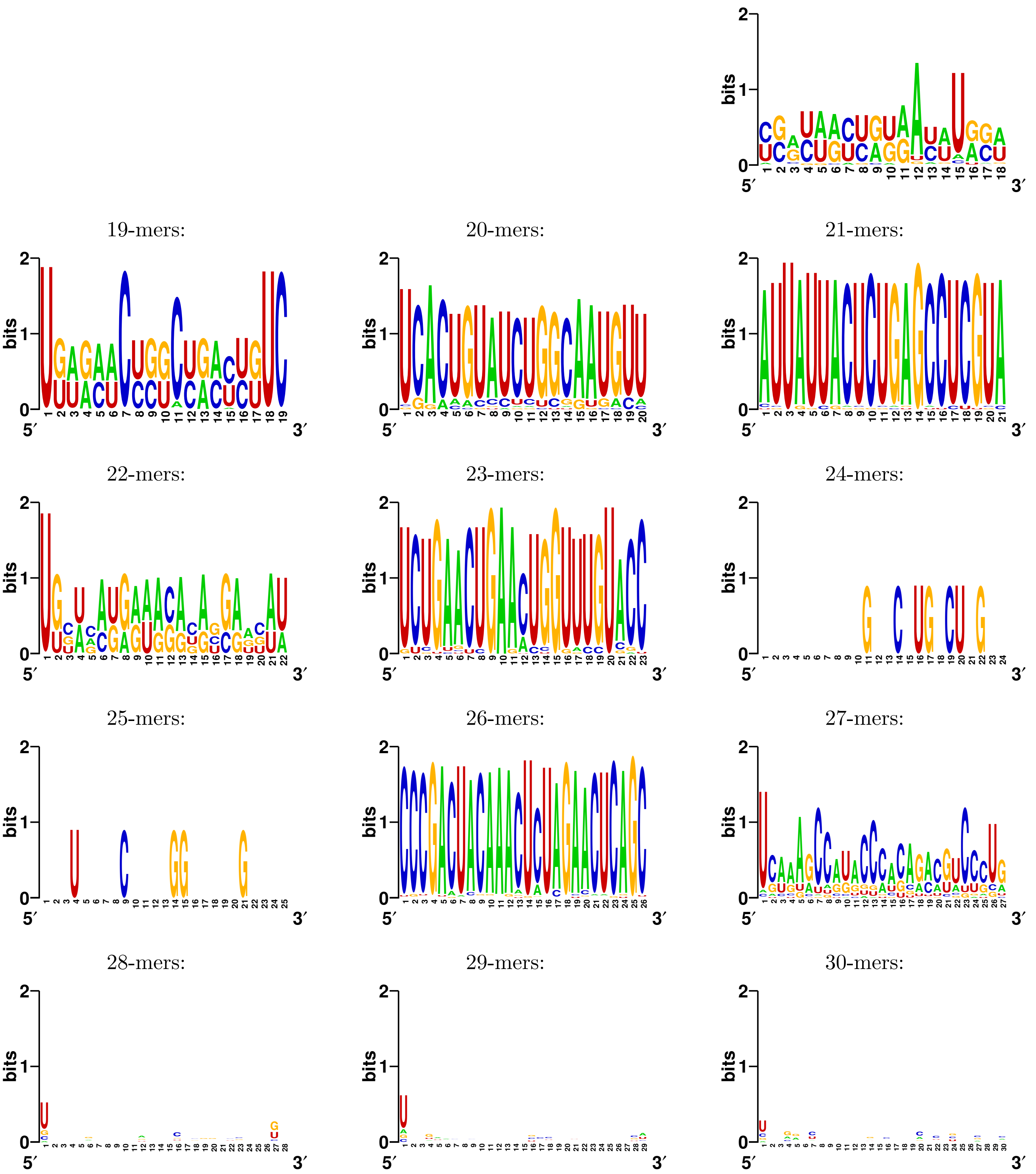

Adult male, library 2:

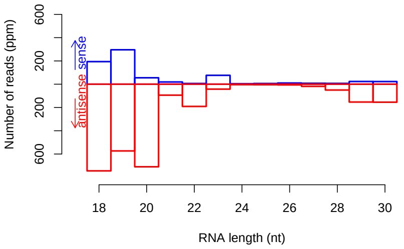

Sense reads:

18-mers:

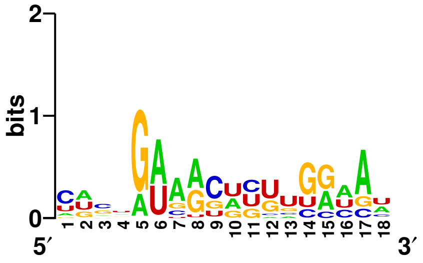

19-mers:

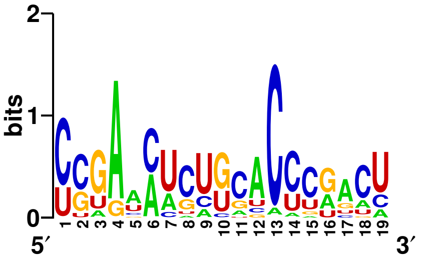

20-mers:

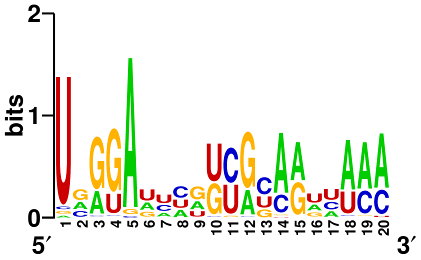

21-mers:

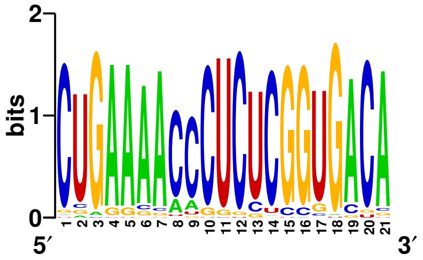

22-mers:

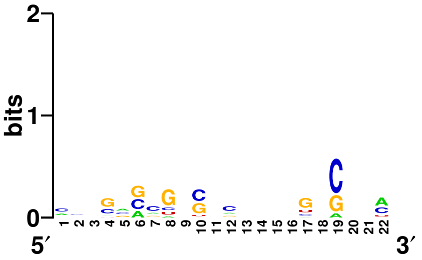

23-mers:

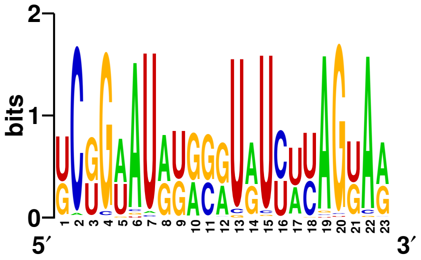

24-mers:

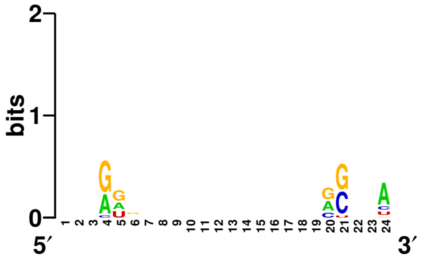

25-mers:

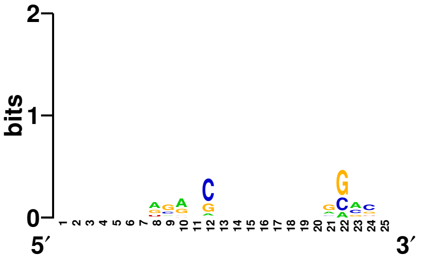

26-mers:

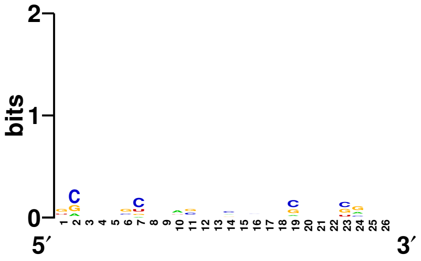

27-mers:

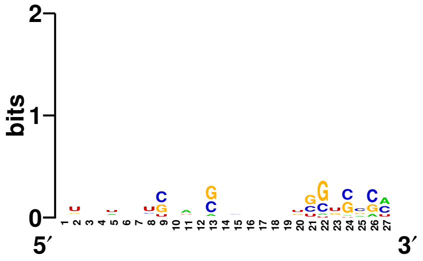

28-mers:

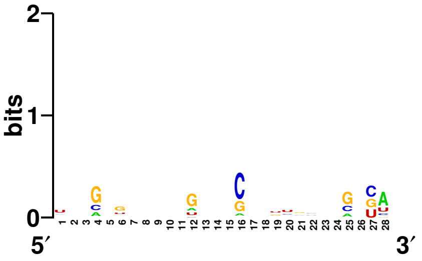

29-mers:

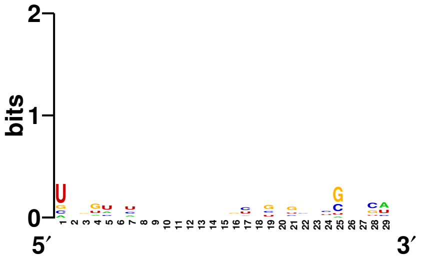

30-mers:

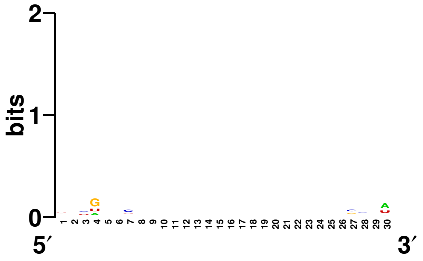

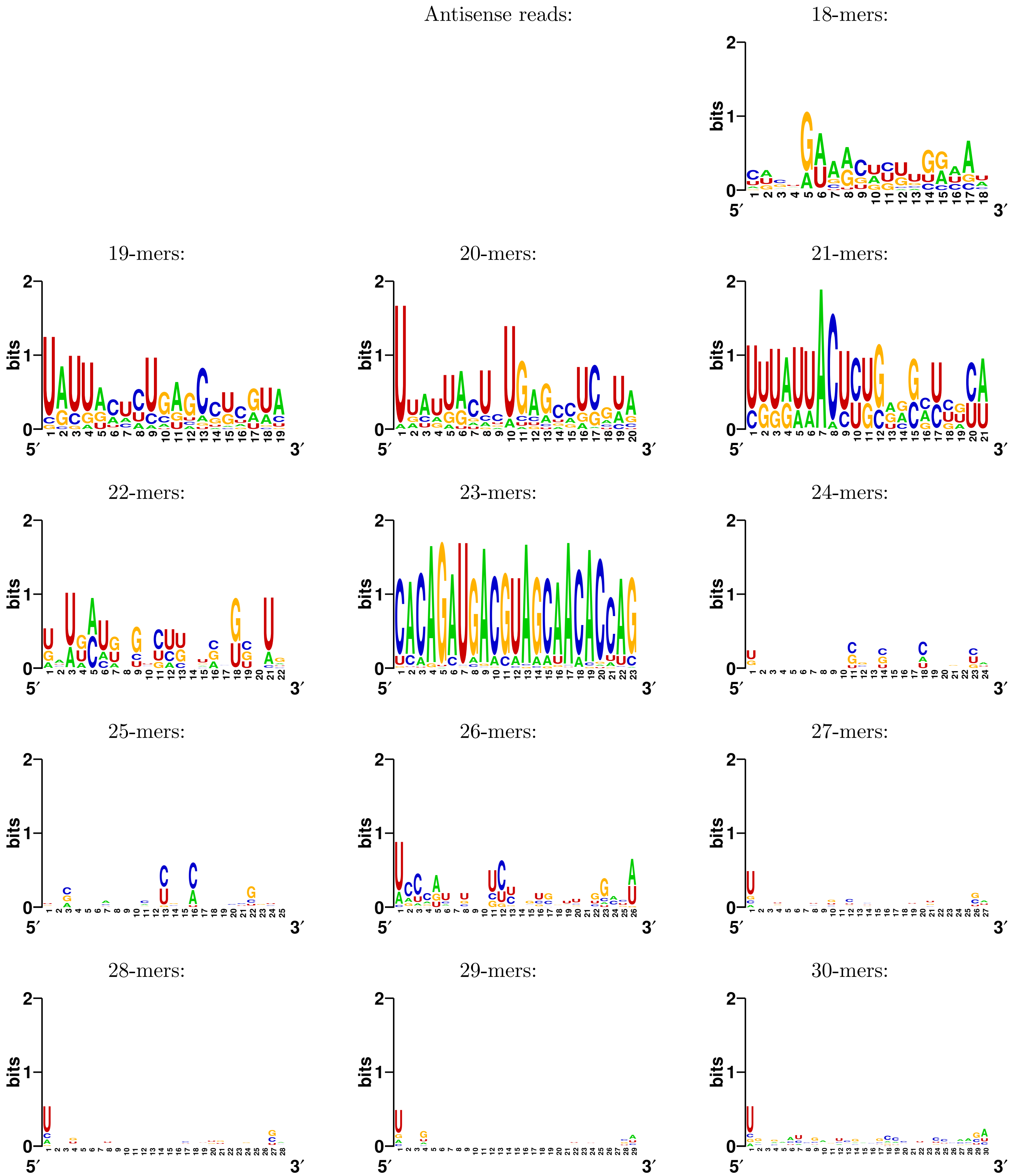
Antisense reads:
18-mers:
19-mers:
20-mers:
21-mers:
22-mers:
23-mers:
24-mers:
25-mers:
26-mers:
27-mers:
28-mers:
29-mers:
30-mers:
bits
5′
3′

4.3 Libraries #3 (total 5´ hydroxyl or polyphosphorylated small RNAs)

Embryo 8h, library 3:

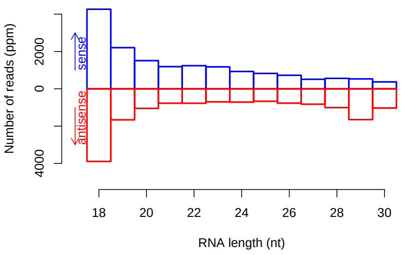

Sense reads:

18-mers:

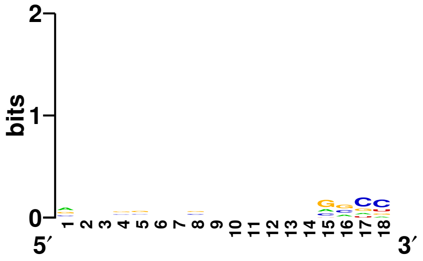

19-mers:

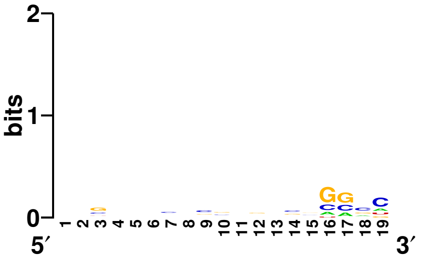

20-mers:

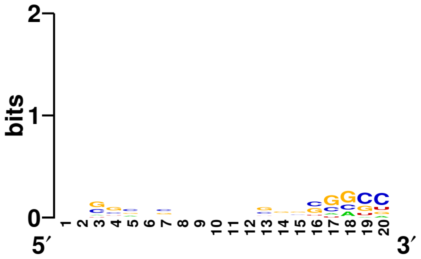

21-mers:

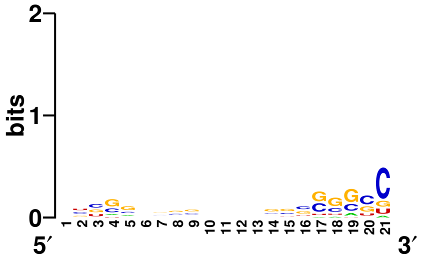

22-mers:

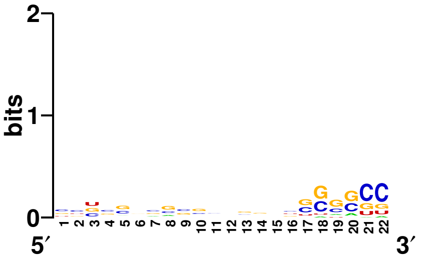

23-mers:

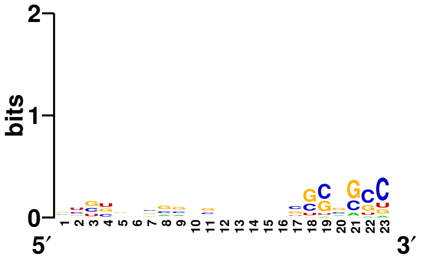

24-mers:

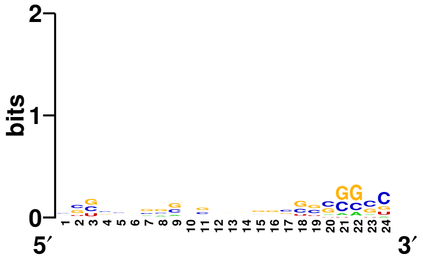

25-mers:

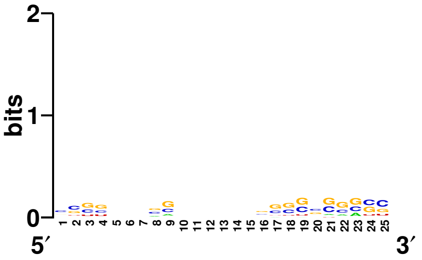

26-mers:

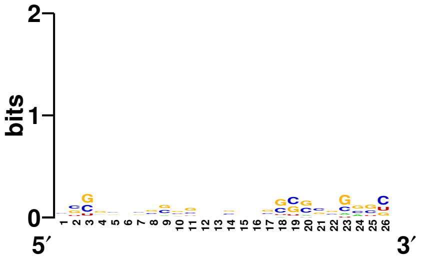

27-mers:

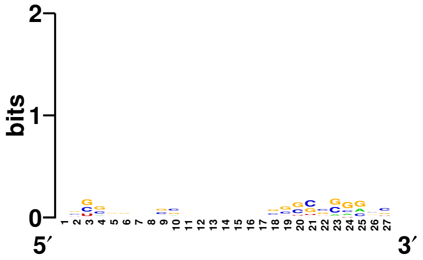

28-mers:

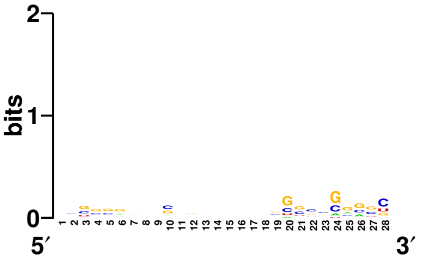

29-mers:

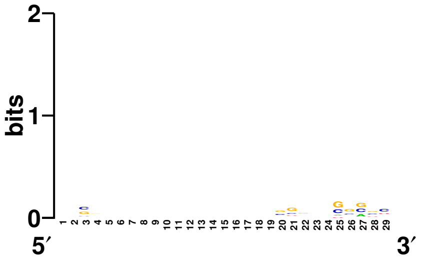

30-mers:

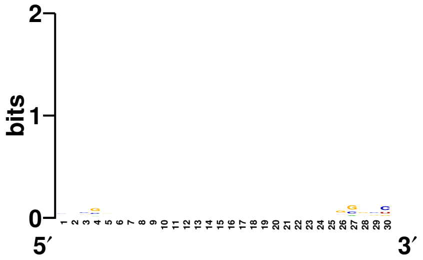

Antisense reads:

18-mers:

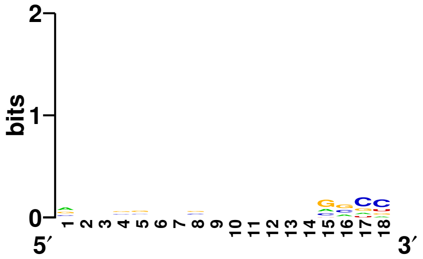

19-mers:

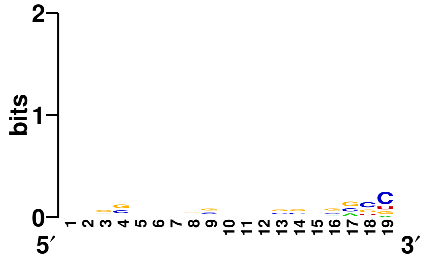

20-mers:

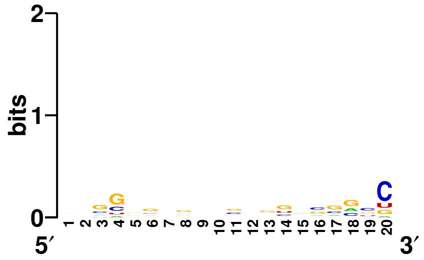

21-mers:

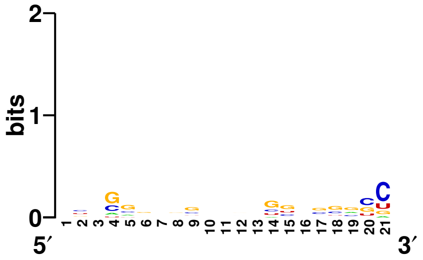

22-mers:

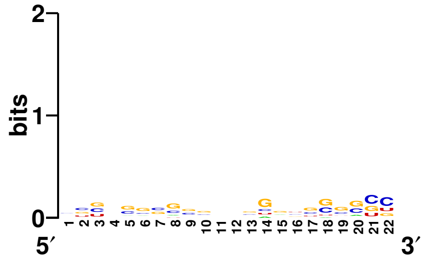

23-mers:

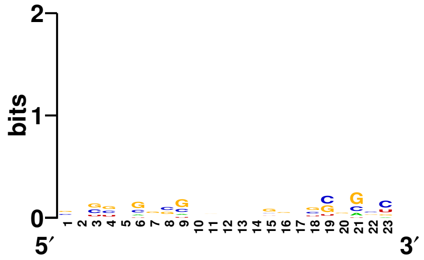

24-mers:

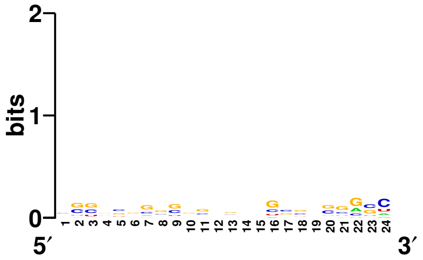

25-mers:

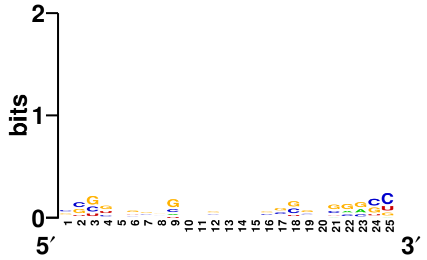

26-mers:

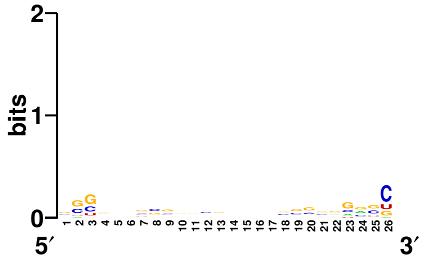

27-mers:

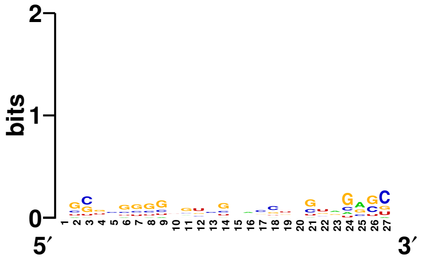

28-mers:

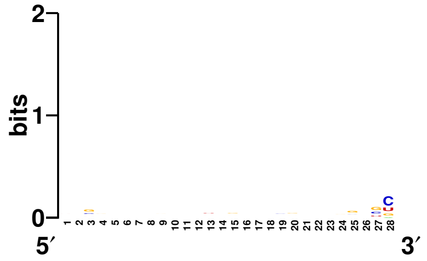

29-mers:

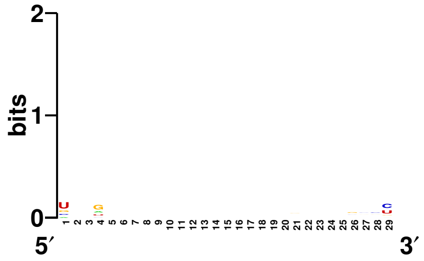

30-mers:

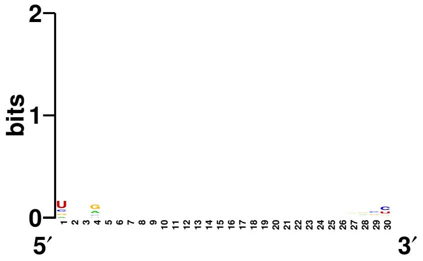

Embryo 15h, library 3:

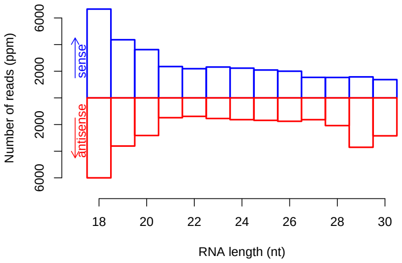

Sense reads:

18-mers:

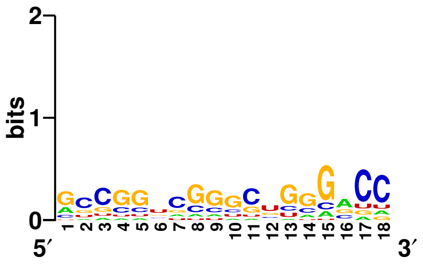

19-mers:

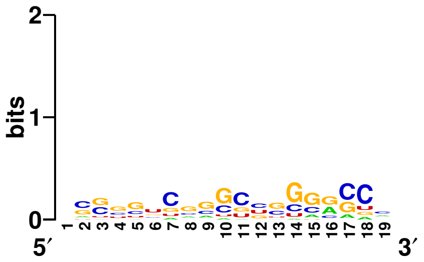

20-mers:

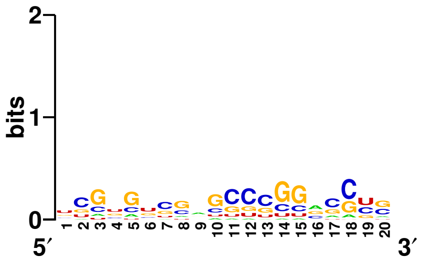

21-mers:

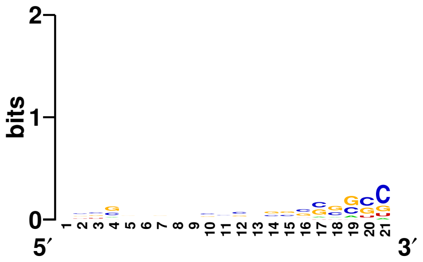

22-mers:

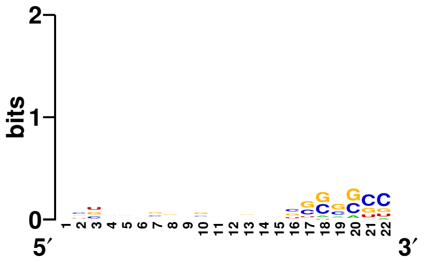

23-mers:

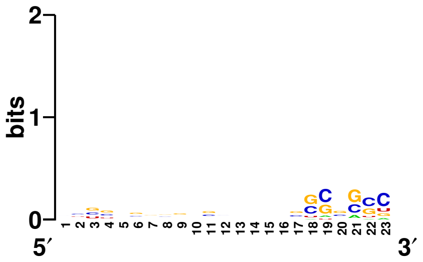

24-mers:

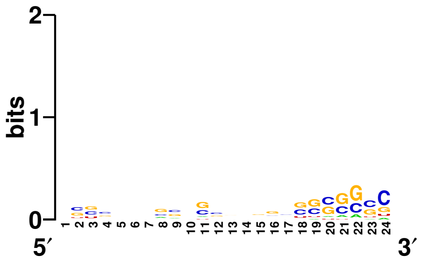

25-mers:

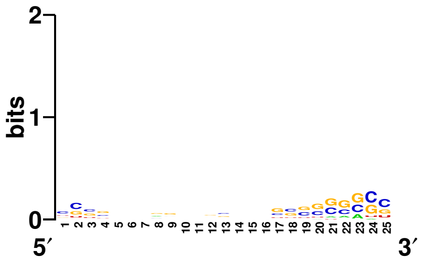

26-mers:

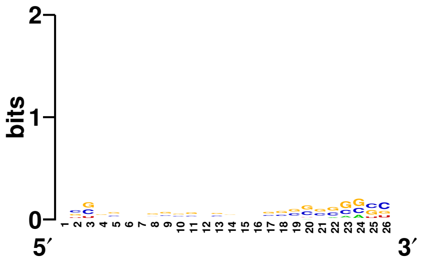

27-mers:

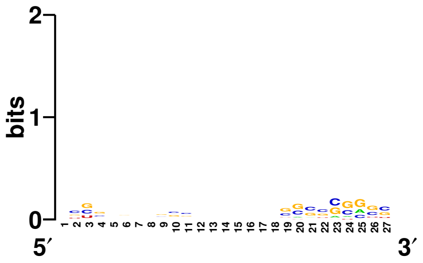

28-mers:

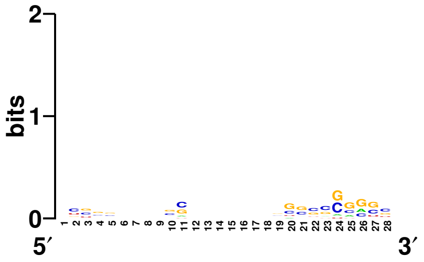

29-mers:

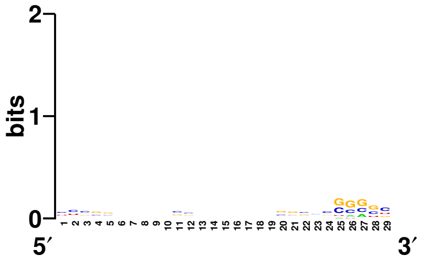

30-mers:

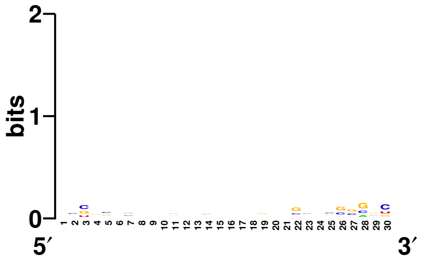

Antisense reads:

18-mers:

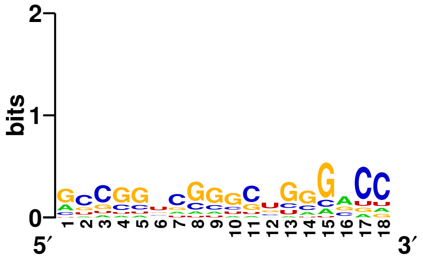

19-mers:

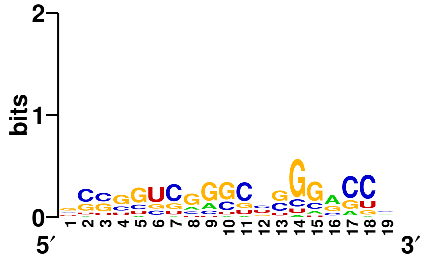

20-mers:

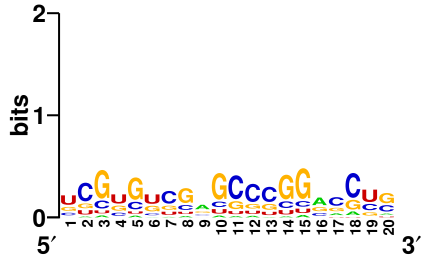

21-mers:

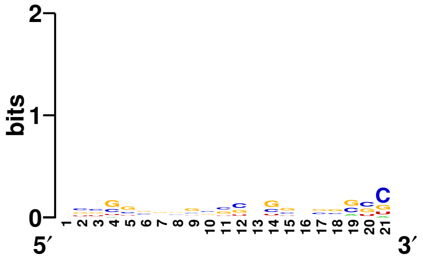

22-mers:

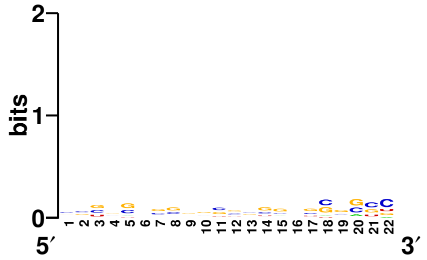

23-mers:

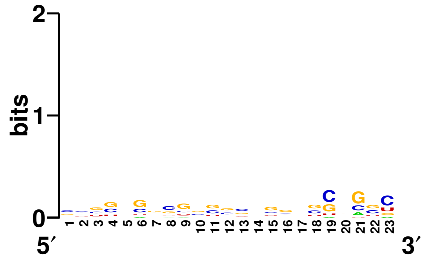

24-mers:

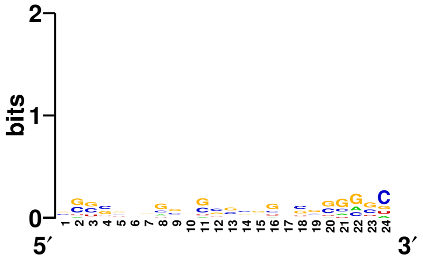

25-mers:

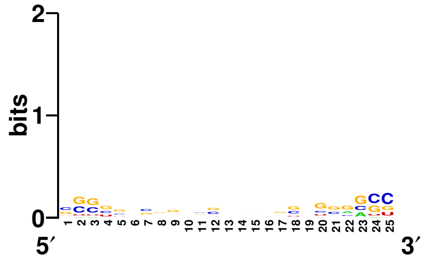

26-mers:

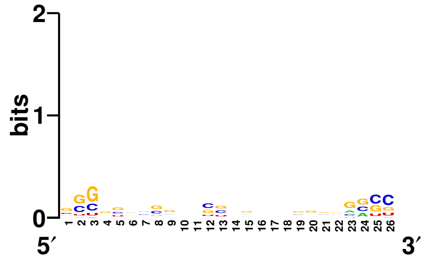

27-mers:

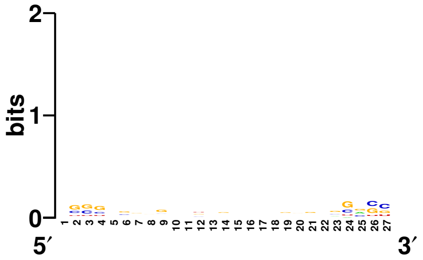

28-mers:

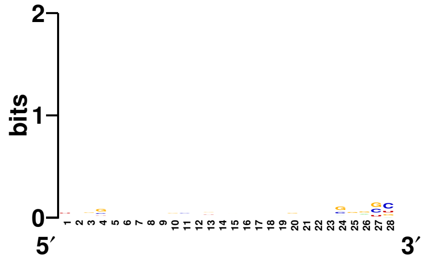

29-mers:

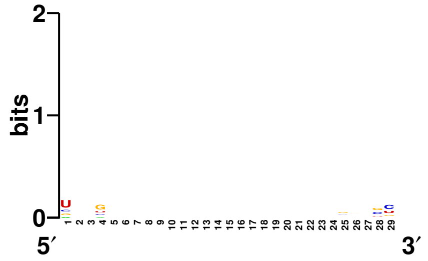

30-mers:

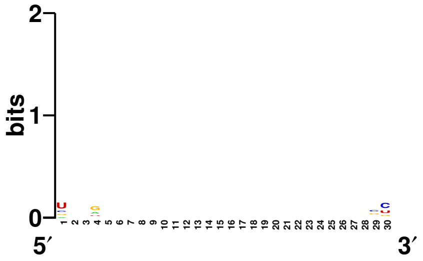

Embryo 36h, library 3:

Sense reads:

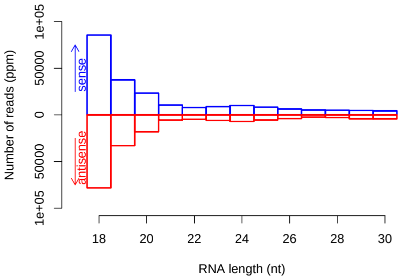

18-mers:

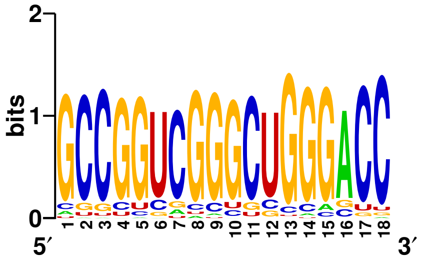

19-mers:

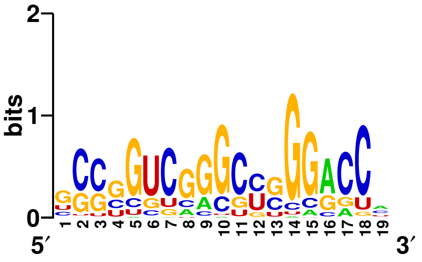

20-mers:

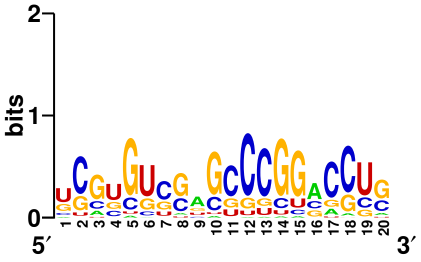

21-mers:

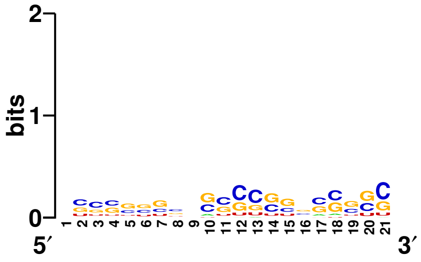

22-mers:

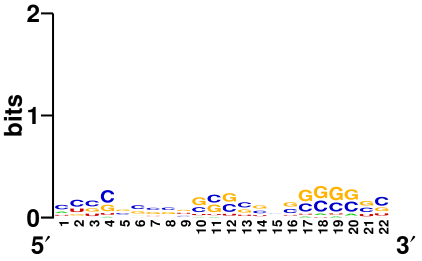

23-mers:

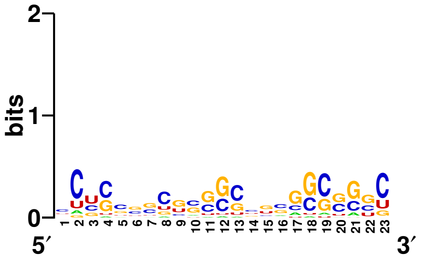

24-mers:

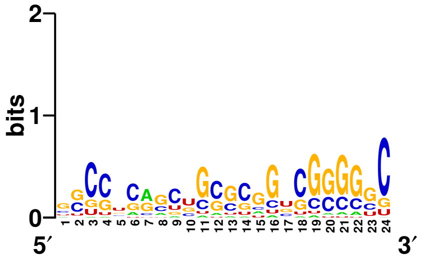

25-mers:

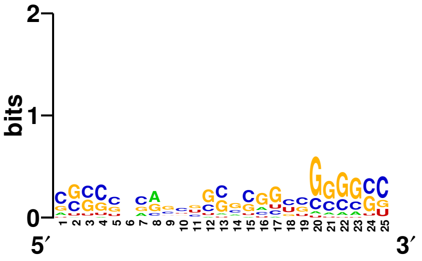

26-mers:

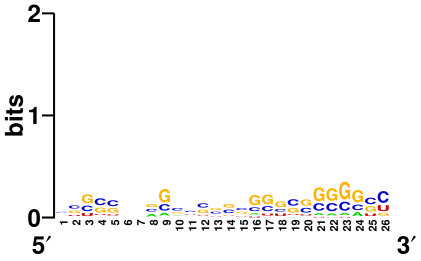

27-mers:

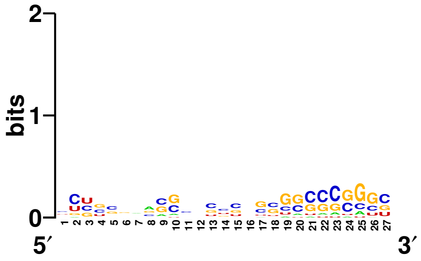

28-mers:

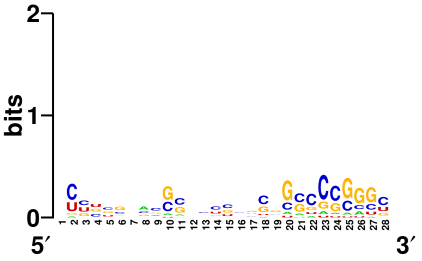

29-mers:

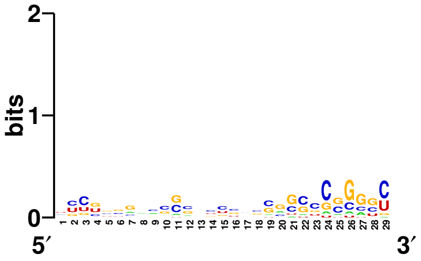

30-mers:

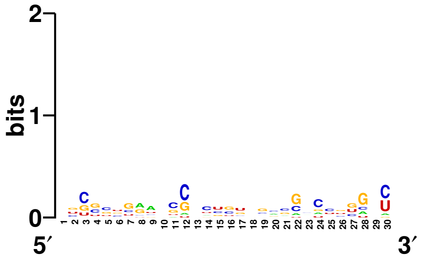

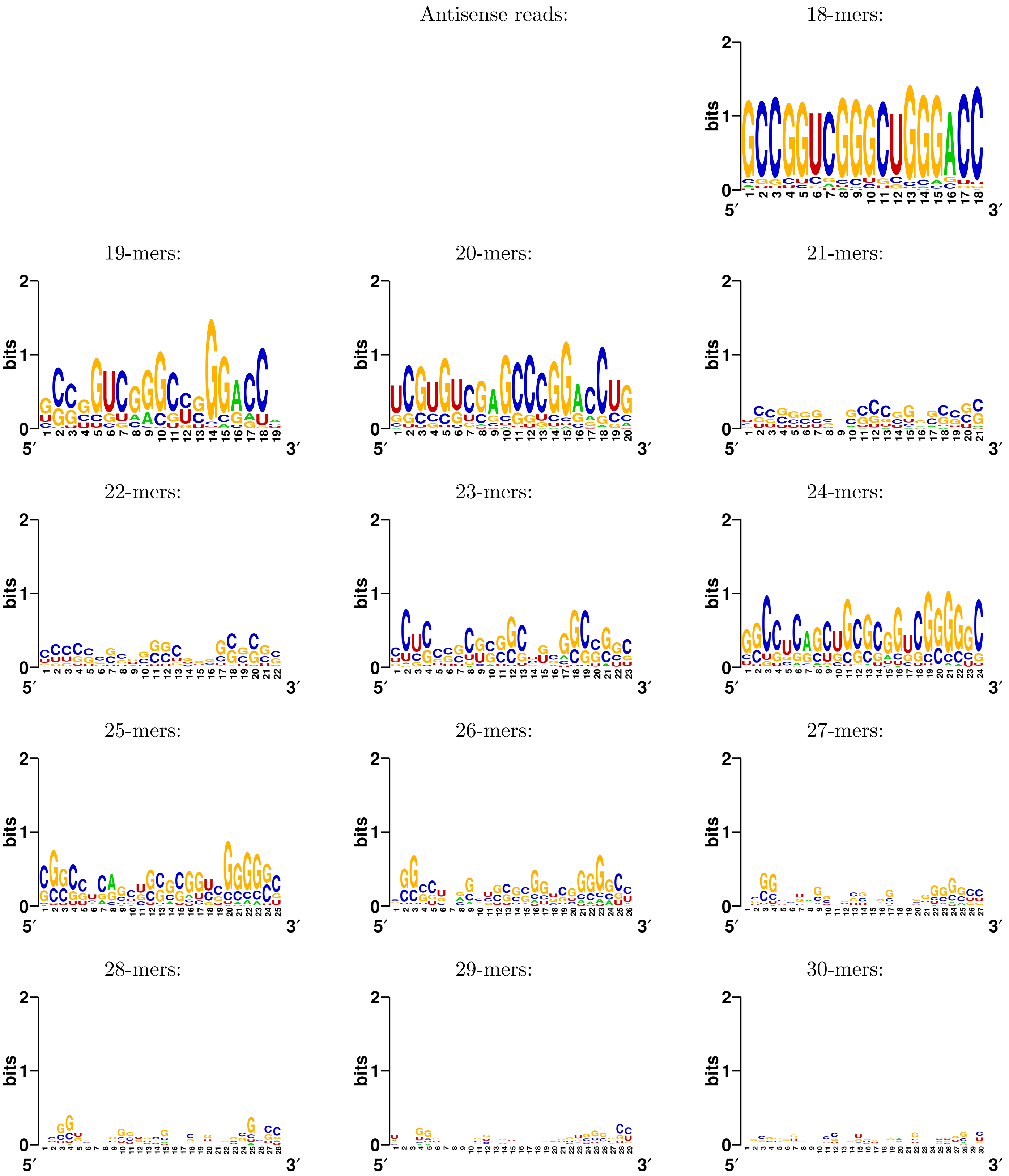

Antisense reads:
18-mers:
19-mers:
20-mers:
21-mers:
22-mers:
23-mers:
24-mers:
25-mers:
26-mers:
27-mers:
28-mers:
29-mers:
30-mers:
bits
5′
3′

Embryo 60h, library 3:

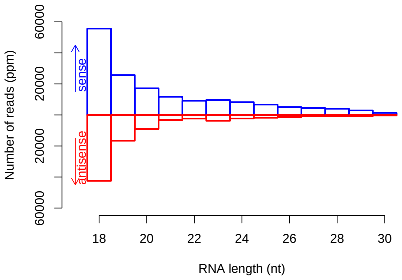

Sense reads:

18-mers:

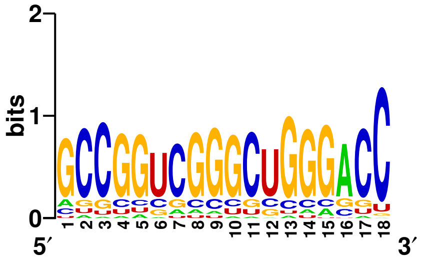

19-mers:

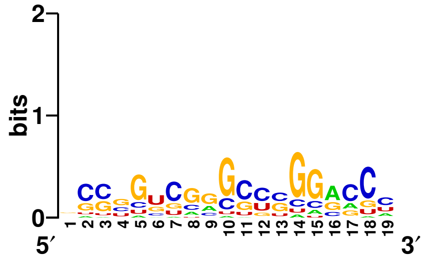

20-mers:

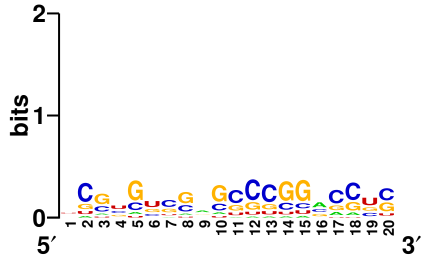

21-mers:

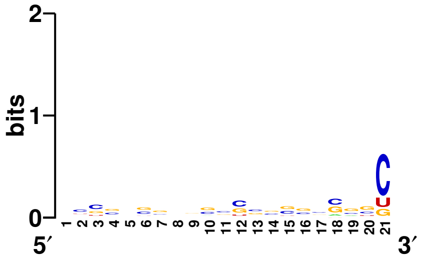

22-mers:

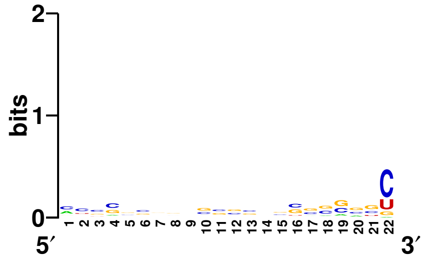

23-mers:

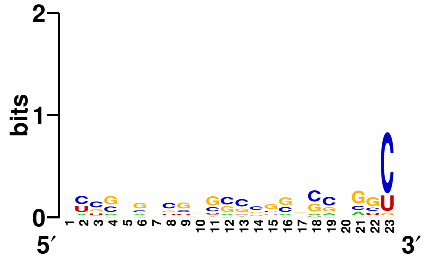

24-mers:

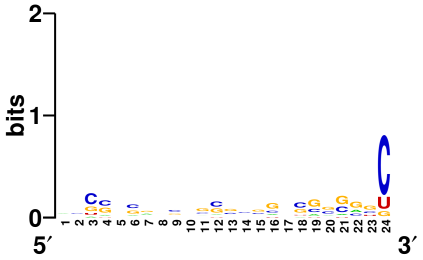

25-mers:

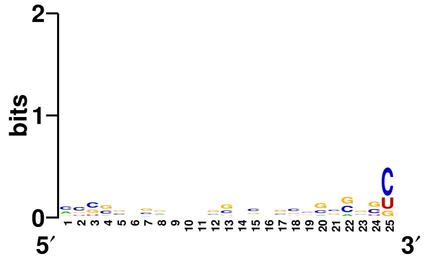

26-mers:

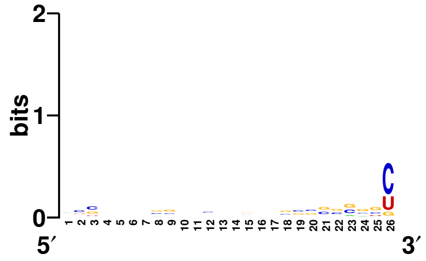

27-mers:

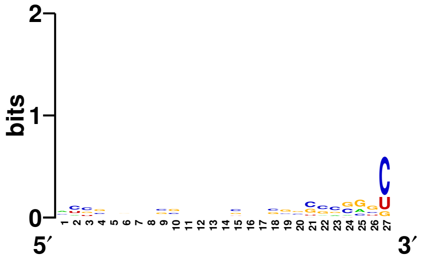

28-mers:

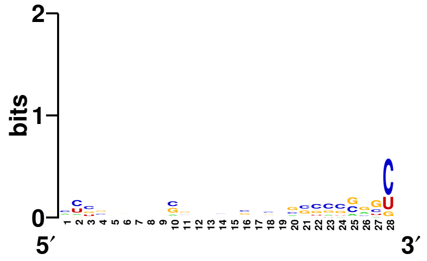

29-mers:

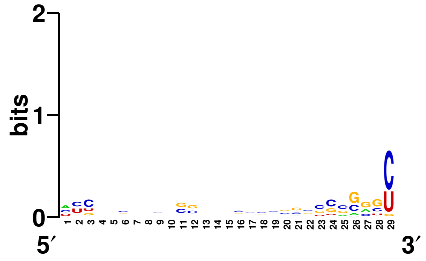

30-mers:

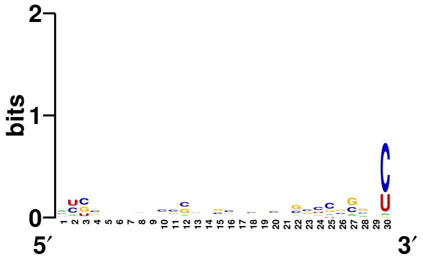

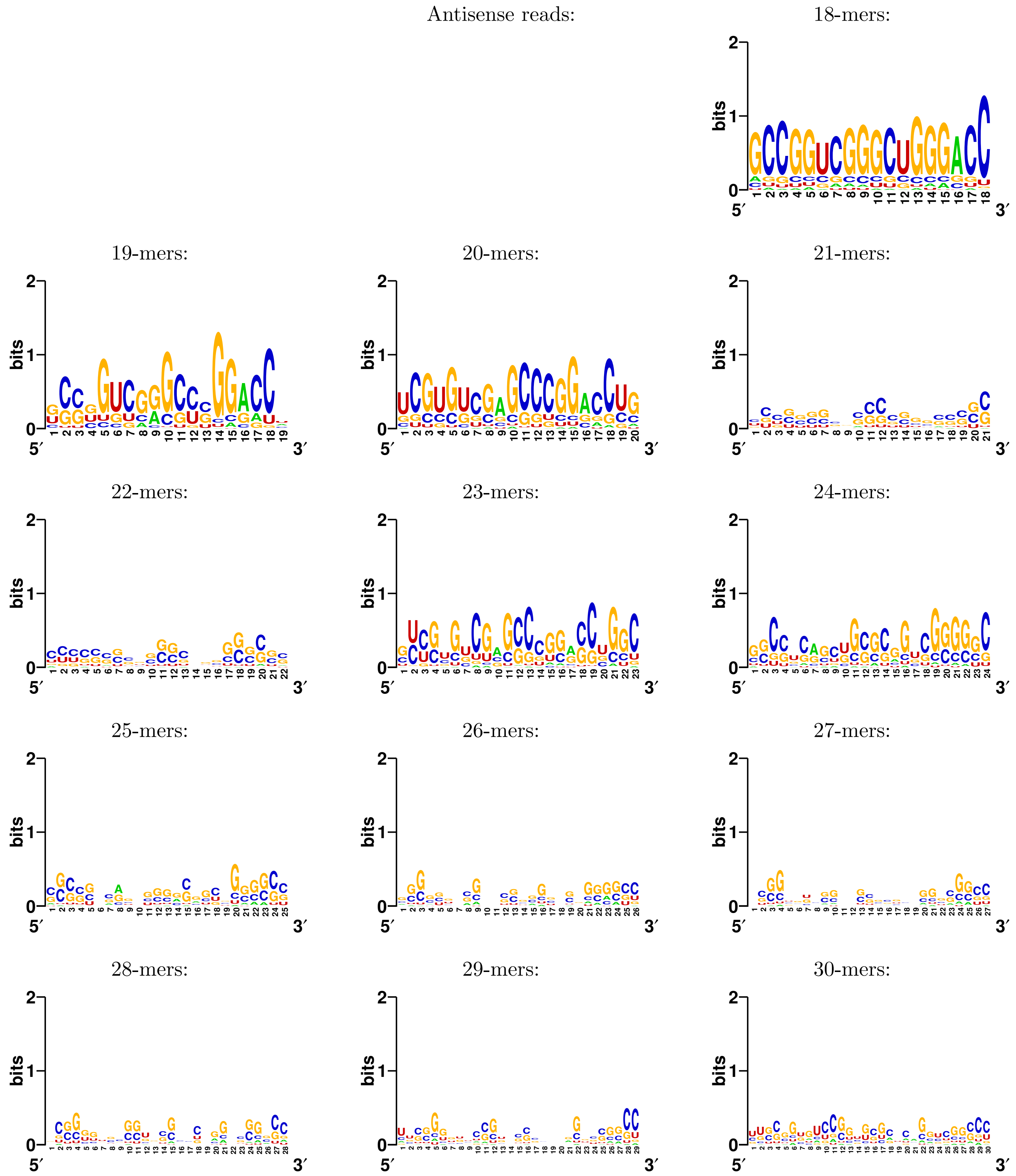
Antisense reads:
18-mers:
19-mers:
20-mers:
21-mers:
22-mers:
23-mers:
24-mers:
25-mers:
26-mers:
27-mers:
28-mers:
29-mers:
30-mers:
bits
5′
3′

Adult female, library 3:

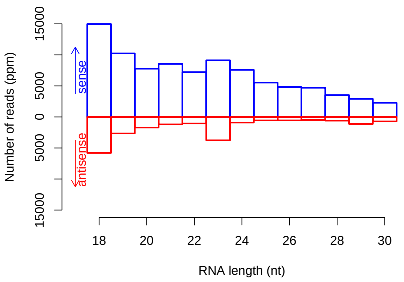

Sense reads:

18-mers:

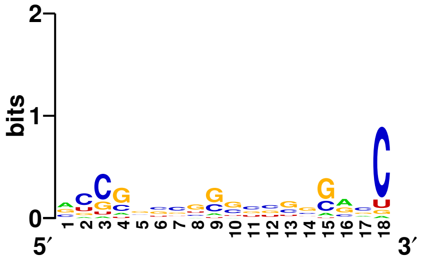

19-mers:

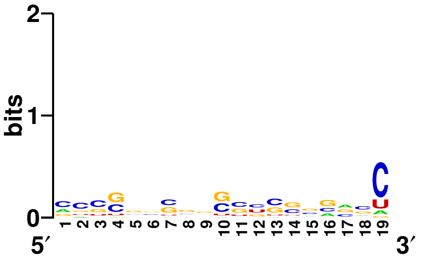

20-mers:

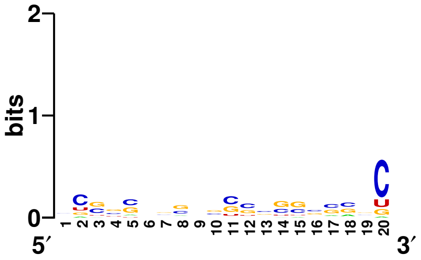

21-mers:

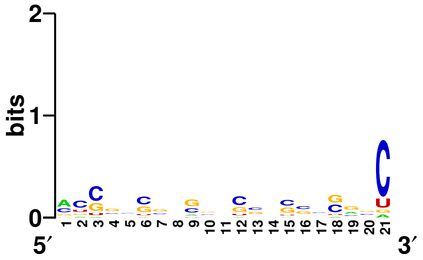

22-mers:

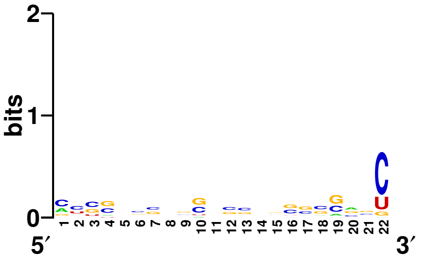

23-mers:

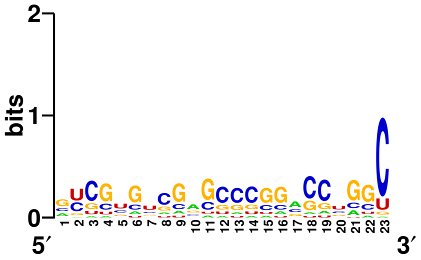

24-mers:

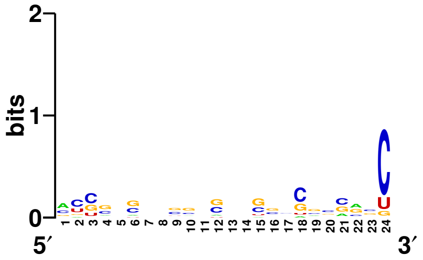

25-mers:

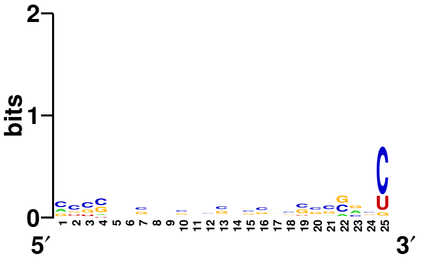

26-mers:

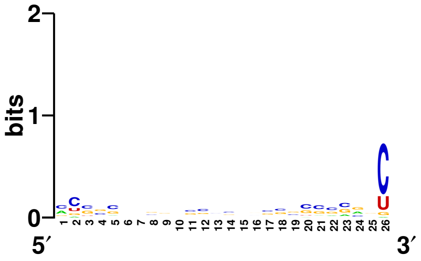

27-mers:

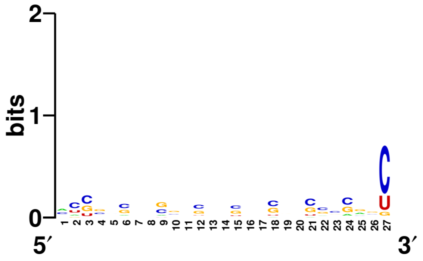

28-mers:

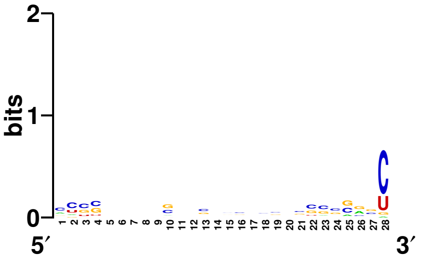

29-mers:

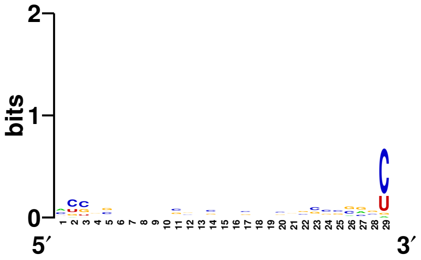

30-mers:

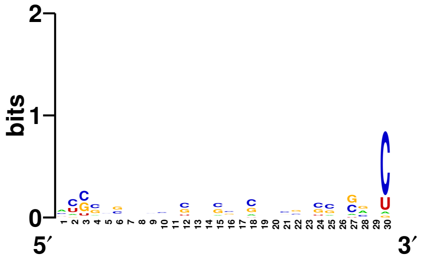

Antisense reads:

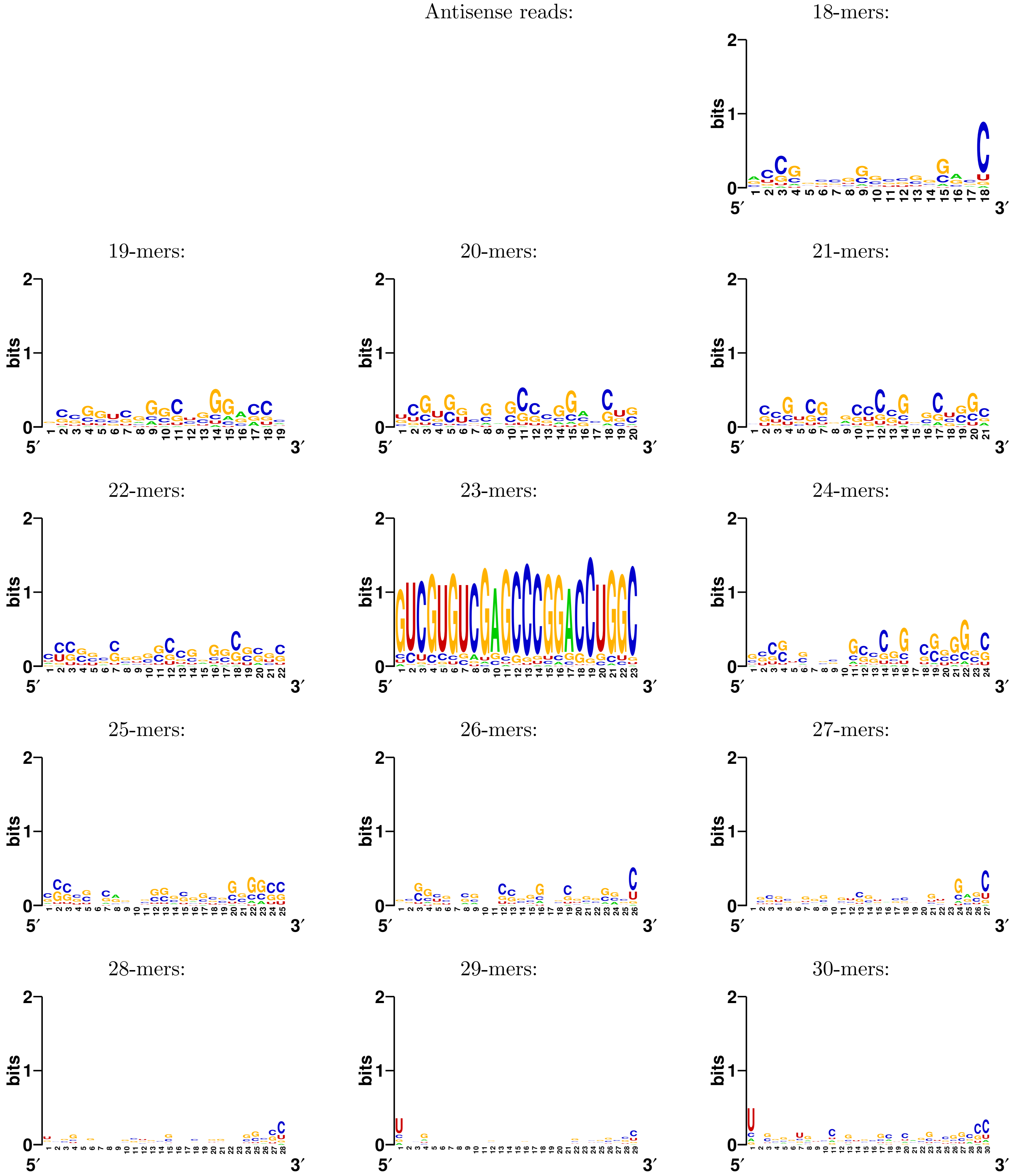

Adult male, library 3:

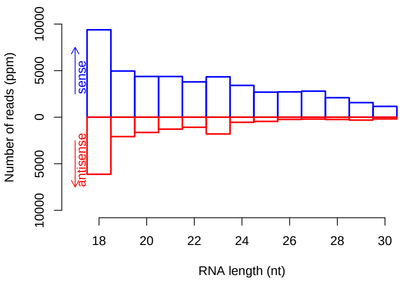

Sense reads:

18-mers:

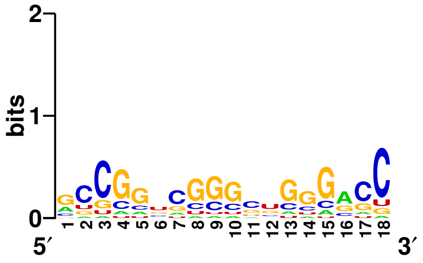

19-mers:

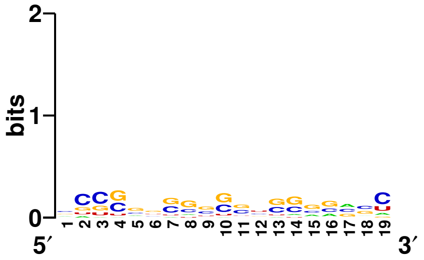

20-mers:

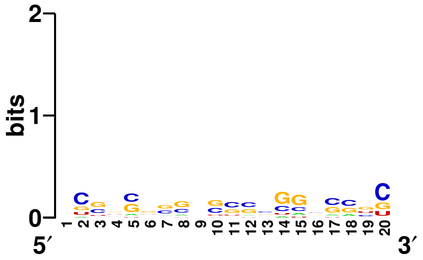

21-mers:

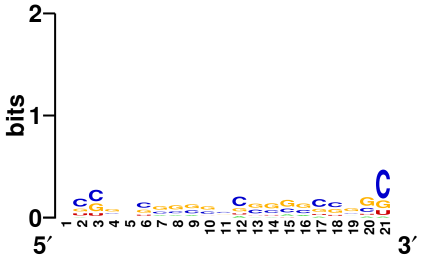

22-mers:

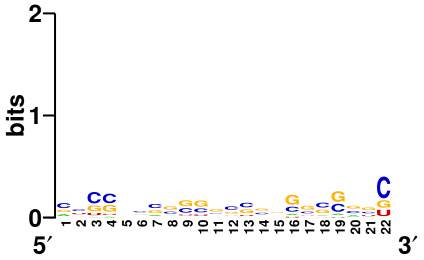

23-mers:

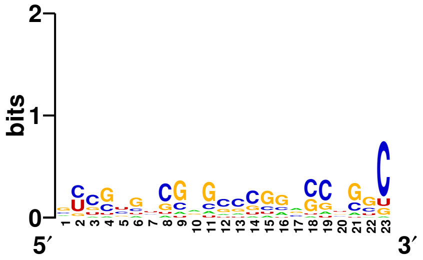

24-mers:

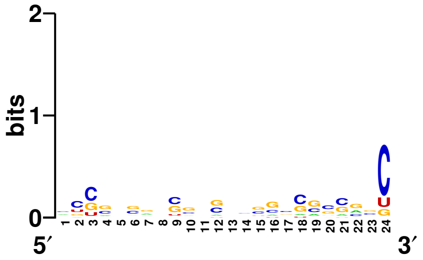

25-mers:

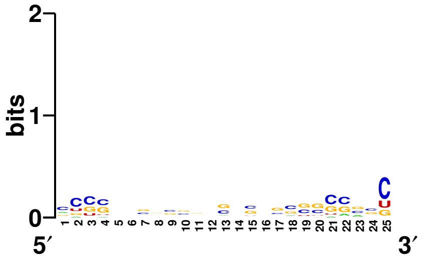

26-mers:

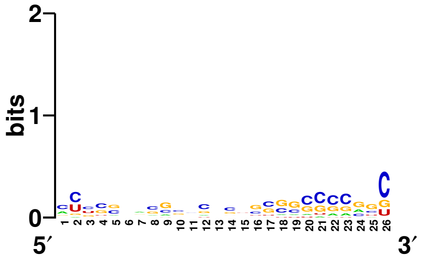

27-mers:

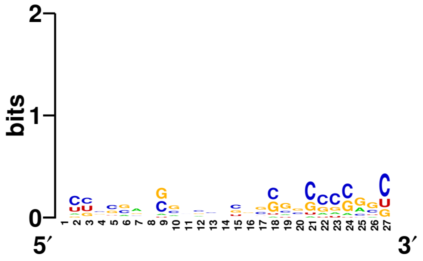

28-mers:

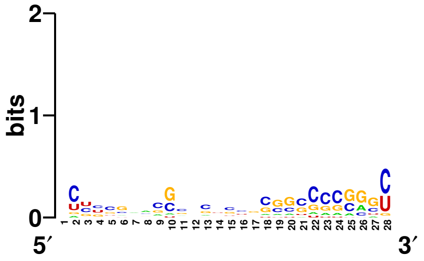

29-mers:

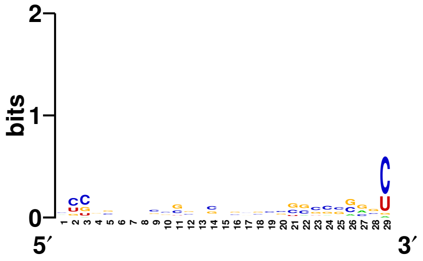

30-mers:

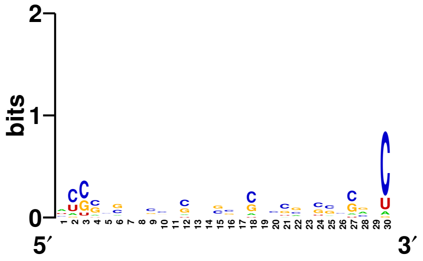

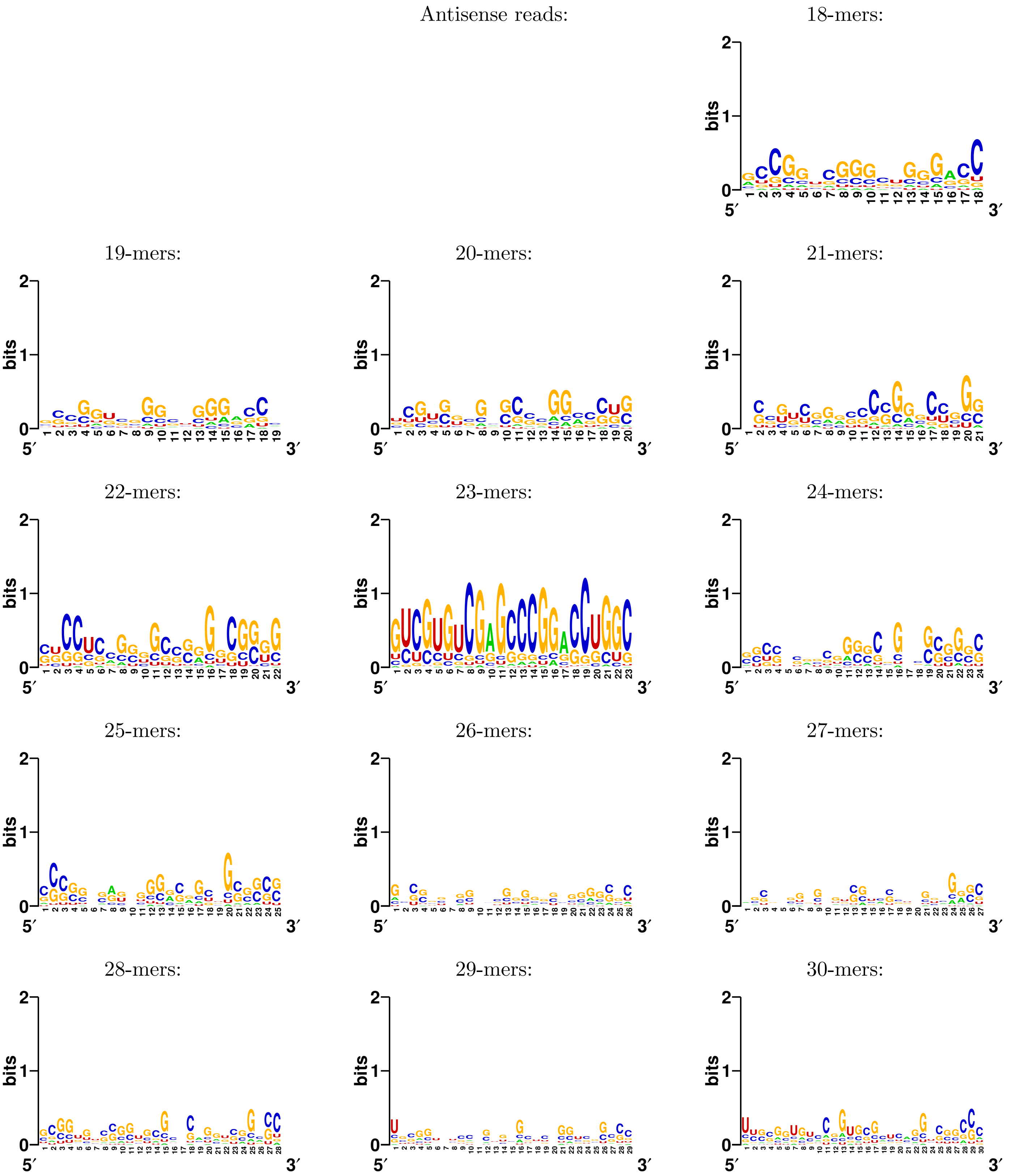
Antisense reads:
18-mers:
19-mers:
20-mers:
21-mers:
22-mers:
23-mers:
24-mers:
25-mers:
26-mers:
27-mers:
28-mers:
29-mers:
30-mers:
bits
5′
3′

4.4 Libraries #4 (3´ modified, 5´ hydroxyl or polyphosphorylated small RNAs)

Embryo 8h, library 4:

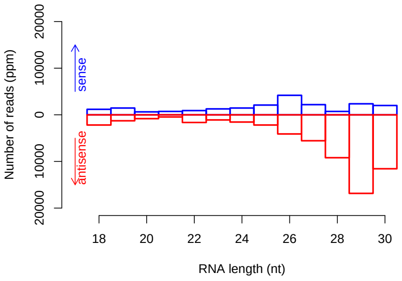

Sense reads:

18-mers:

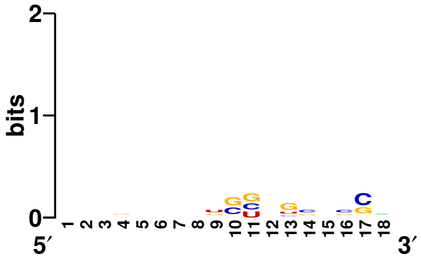

19-mers:

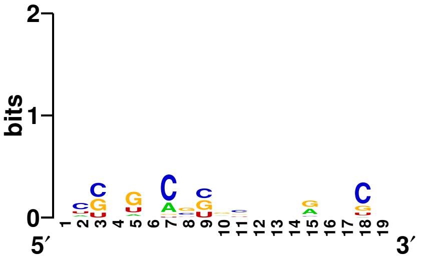

20-mers:

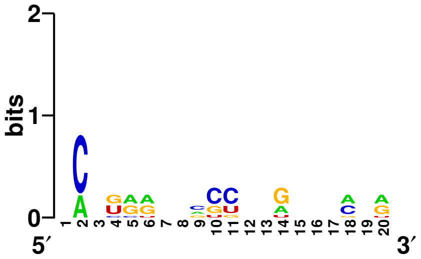

21-mers:

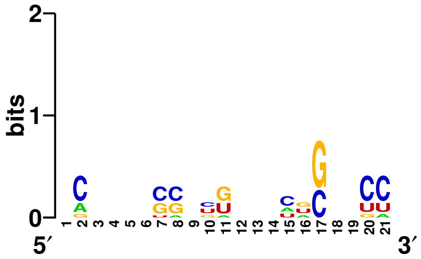

22-mers:

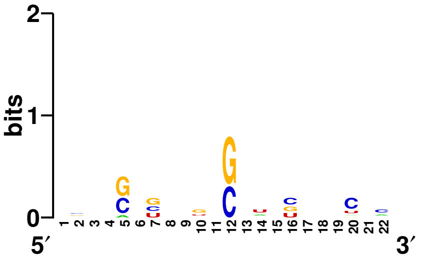

23-mers:

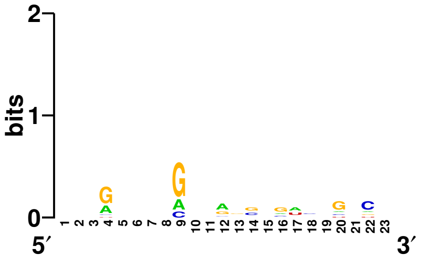

24-mers:

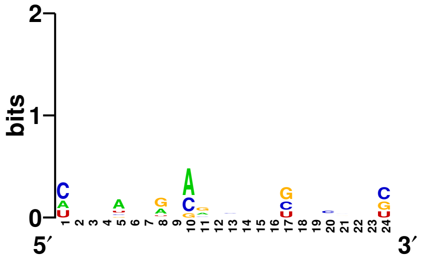

25-mers:

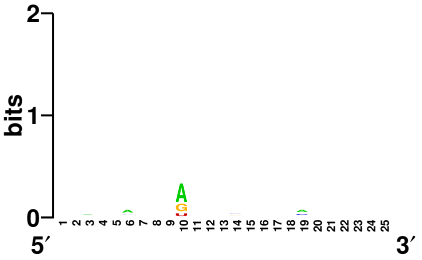

26-mers:

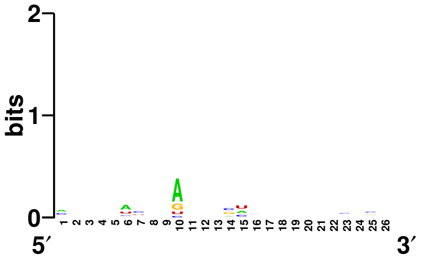

27-mers:

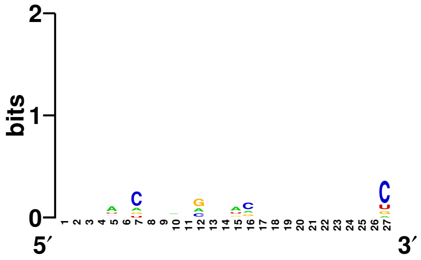

28-mers:

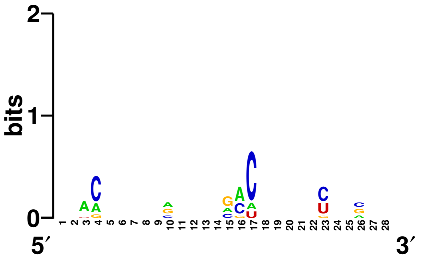

29-mers:

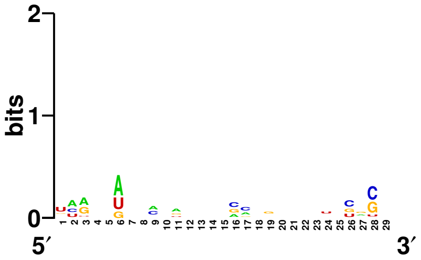

30-mers:

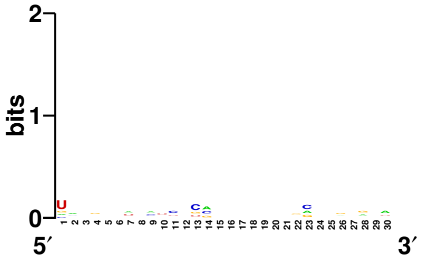

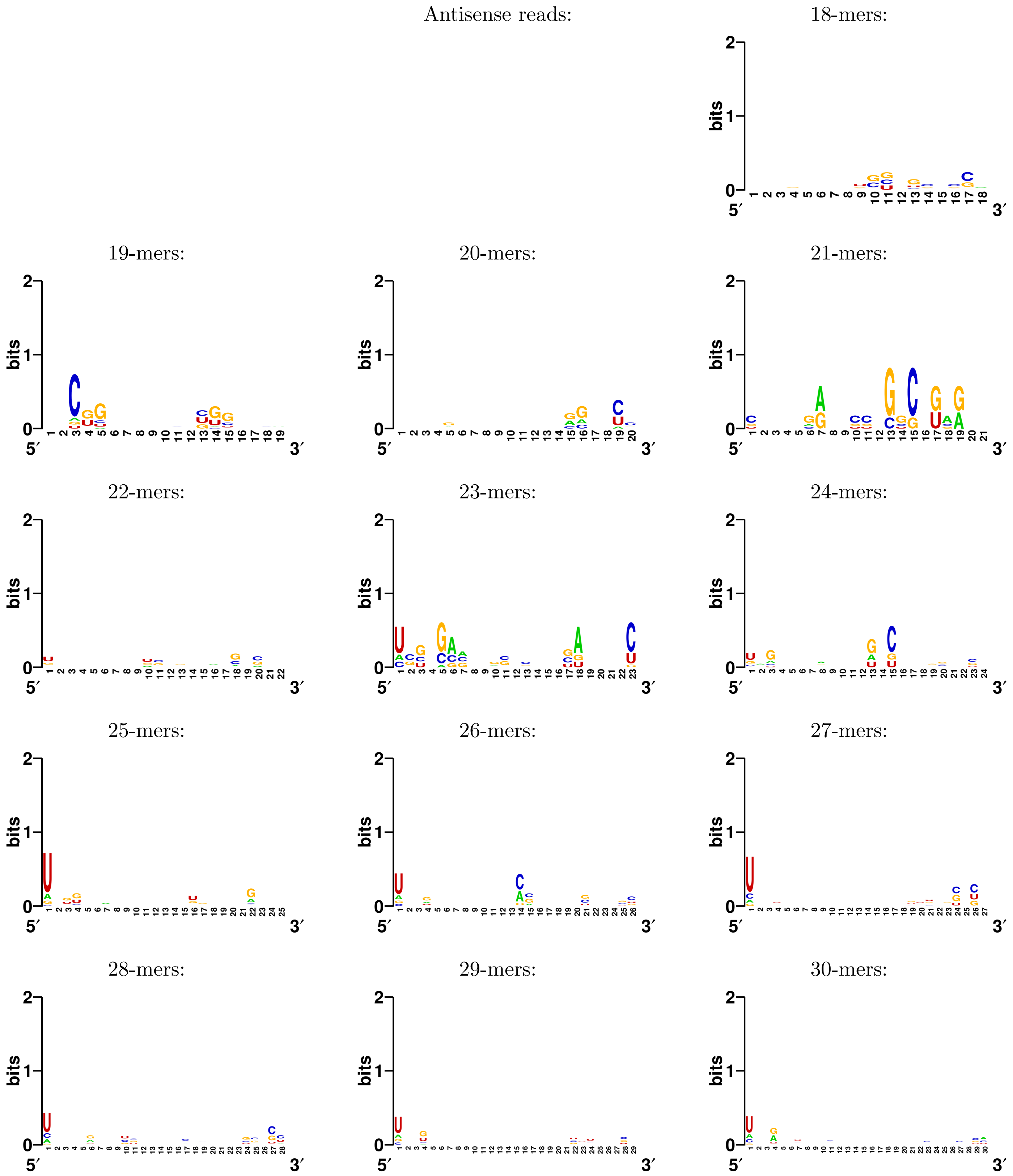
Antisense reads:
18-mers:
19-mers:
20-mers:
21-mers:
22-mers:
23-mers:
24-mers:
25-mers:
26-mers:
27-mers:
28-mers:
29-mers:
30-mers:
bits
5′
3′

Embryo 15h, library 4:

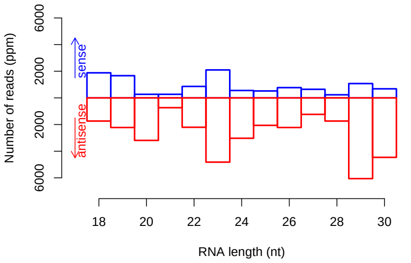

Sense reads:

18-mers:

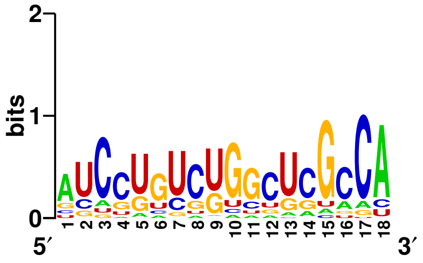

19-mers:

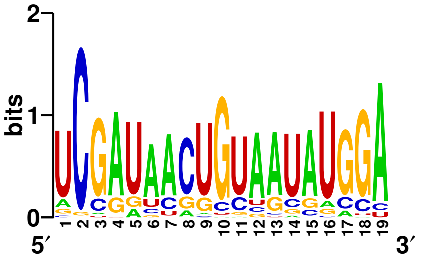

20-mers:

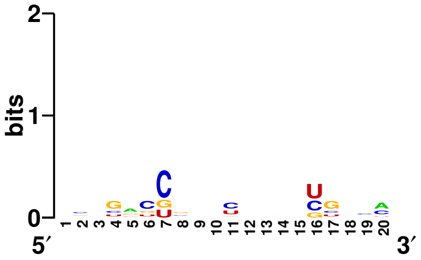

21-mers:

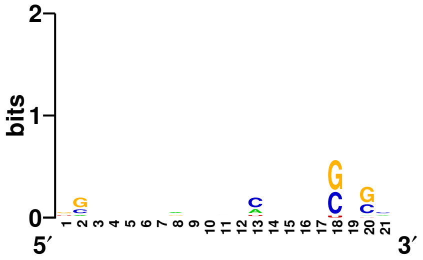

22-mers:

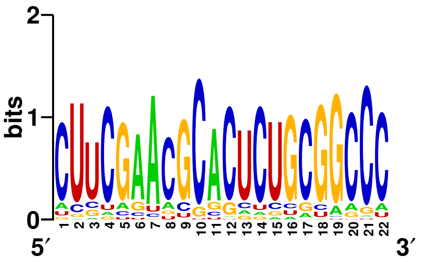

23-mers:

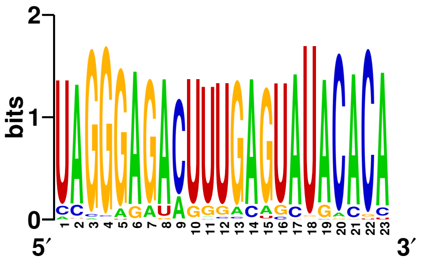

24-mers:

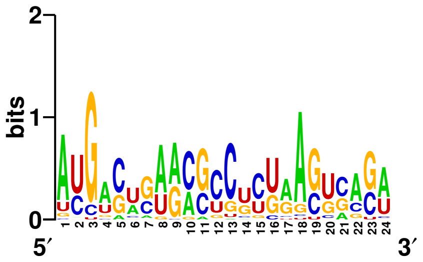

25-mers:

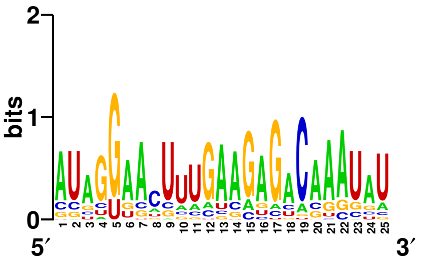

26-mers:

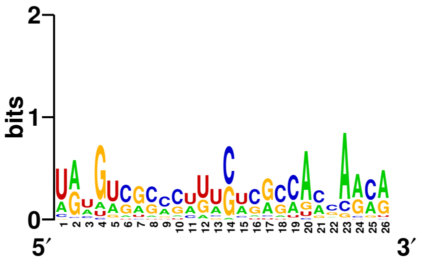

27-mers:

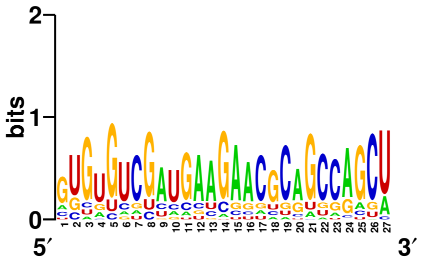

28-mers:

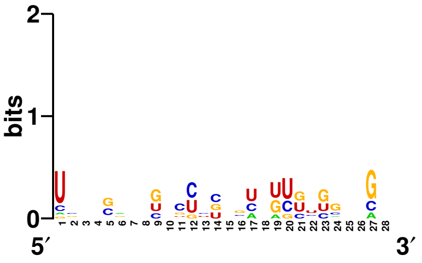

29-mers:

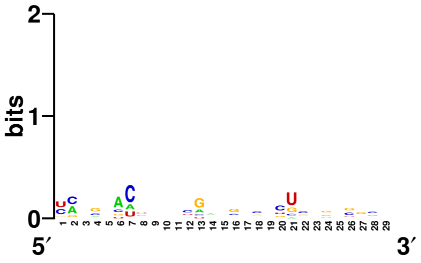

30-mers:

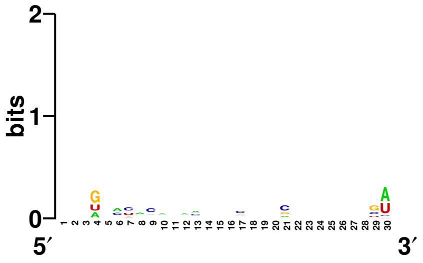

Antisense reads:

18-mers:

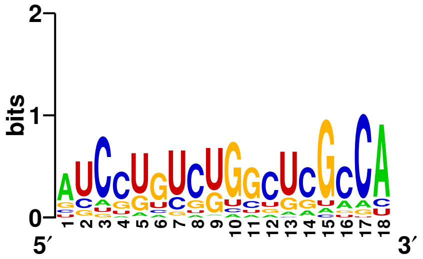

19-mers:

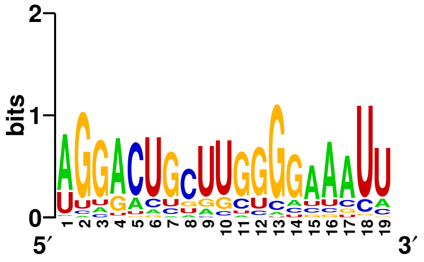

20-mers:

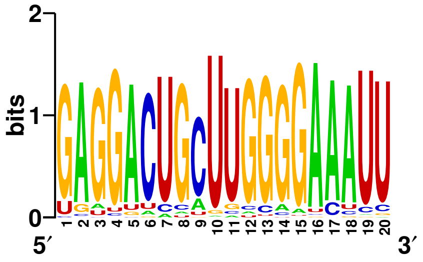

21-mers:

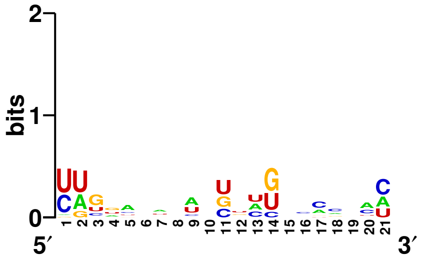

22-mers:

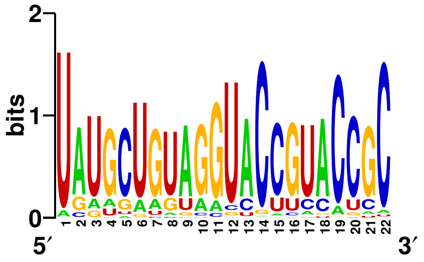

23-mers:

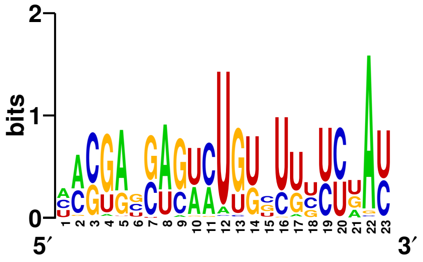

24-mers:

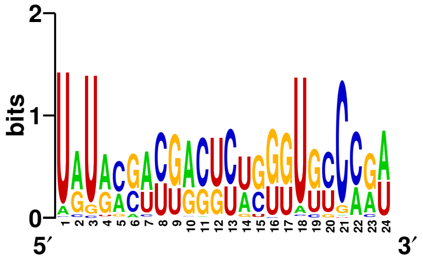

25-mers:

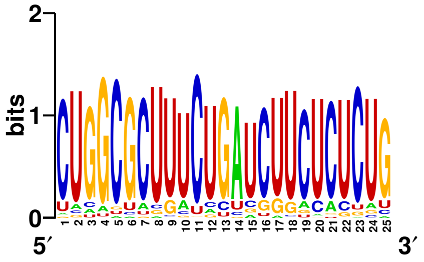

26-mers:

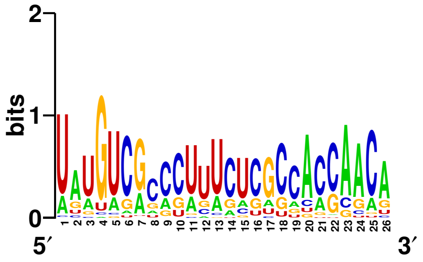

27-mers:

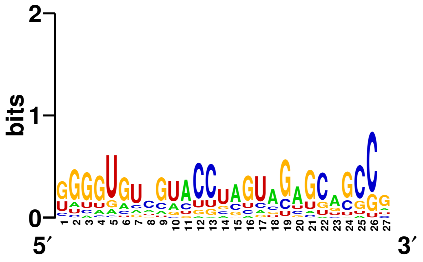

28-mers:

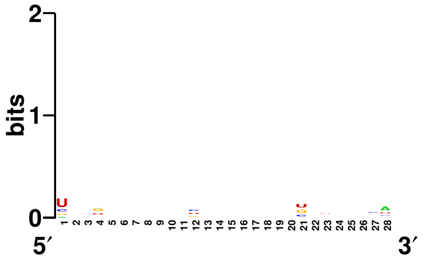

29-mers:

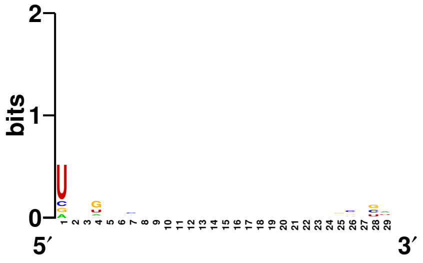

30-mers:

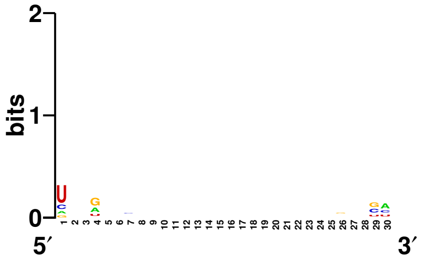

Embryo 36h, library 4:

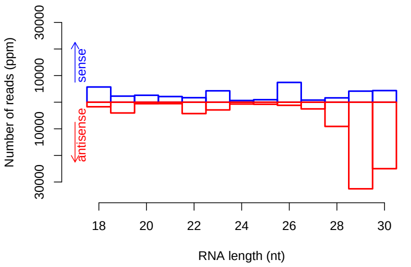

Sense reads:

18-mers:

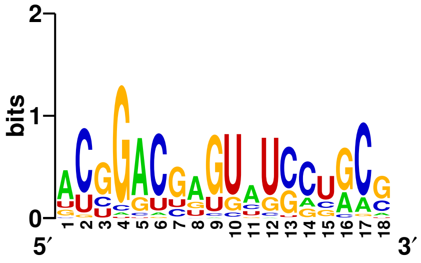

19-mers:

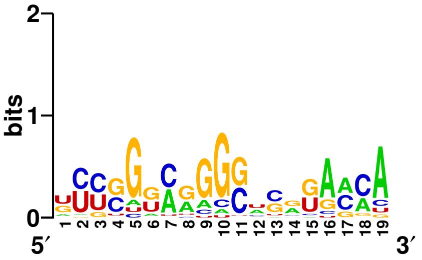

20-mers:

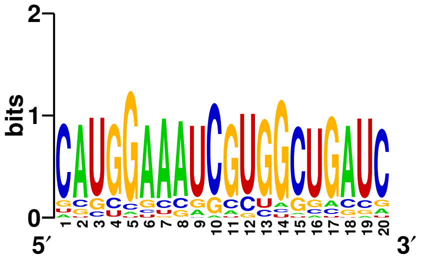

21-mers:

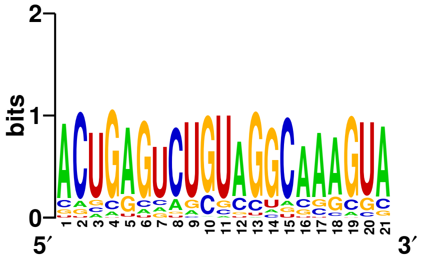

22-mers:

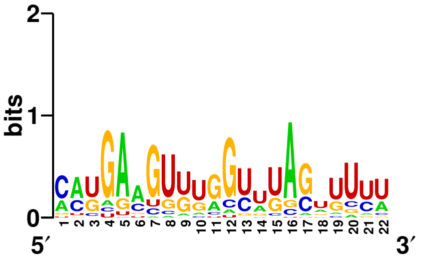

23-mers:

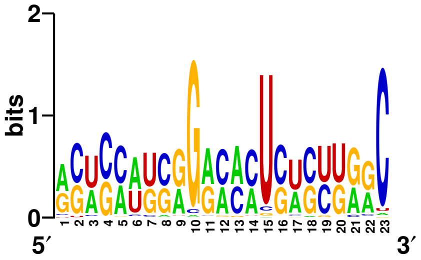

24-mers:

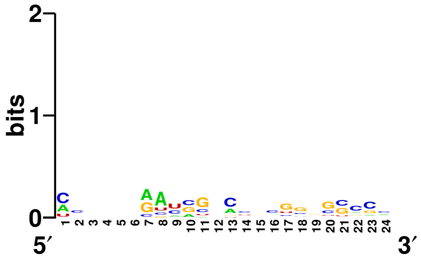

25-mers:

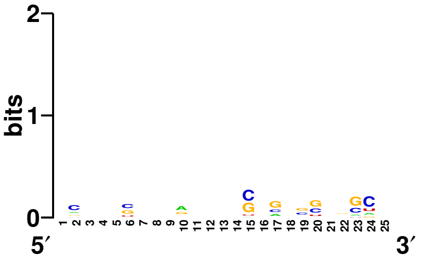

26-mers:

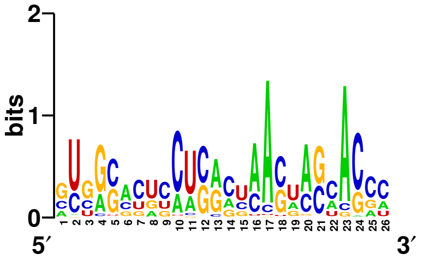

27-mers:

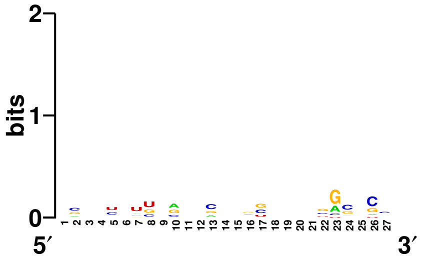

28-mers:

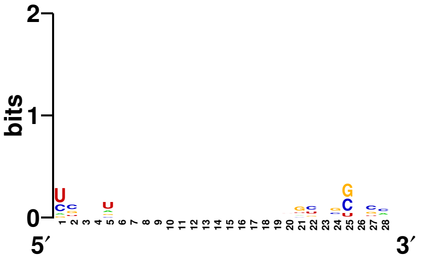

29-mers:

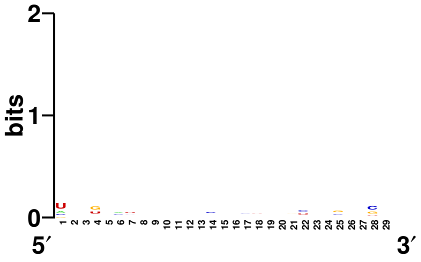

30-mers:

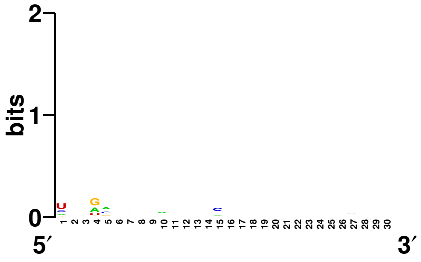

Antisense reads:

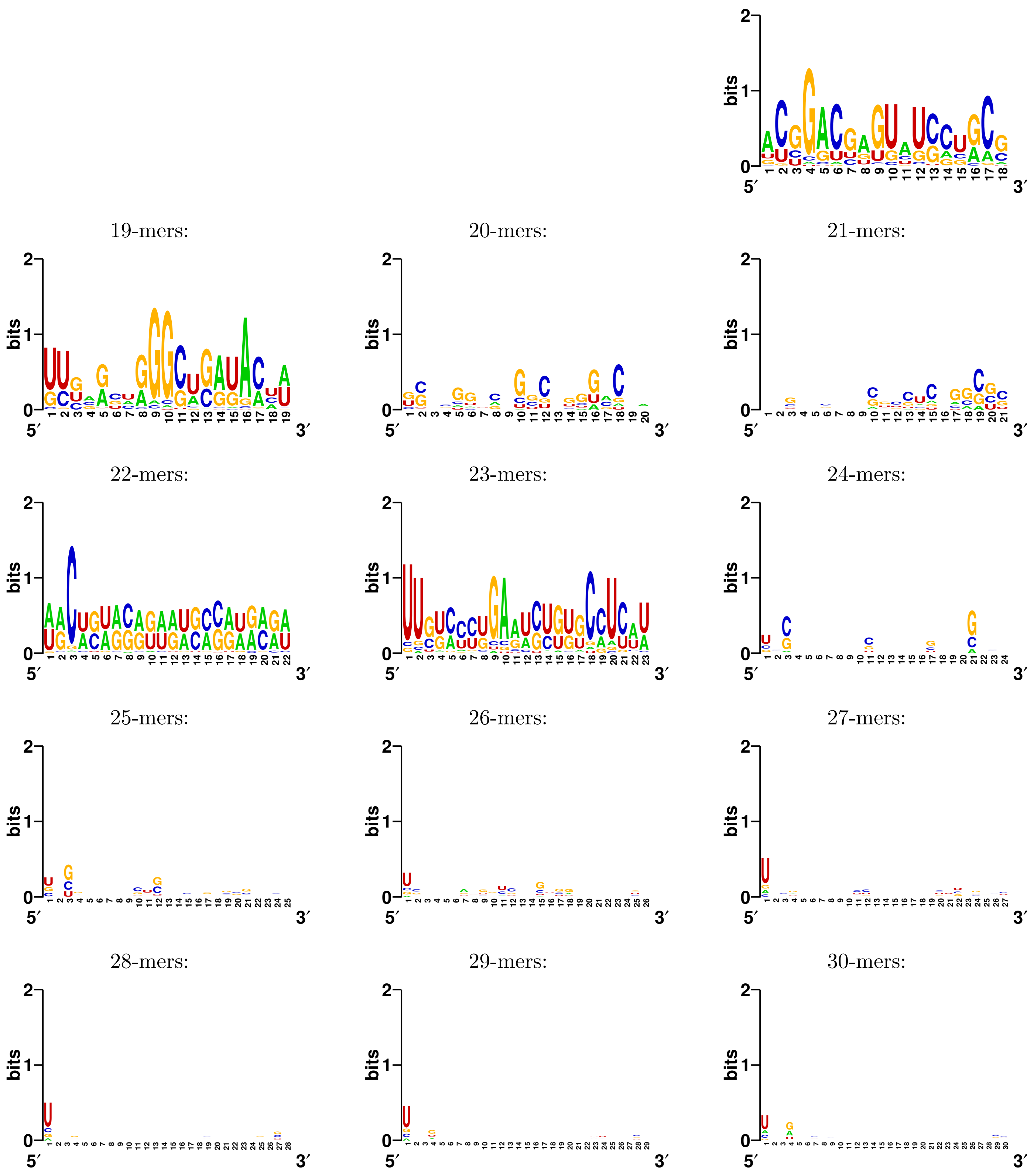

Embryo 60h, library 4:

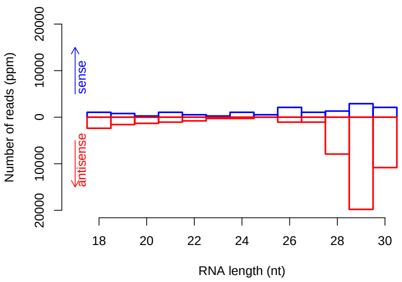

Sense reads:

18-mers:

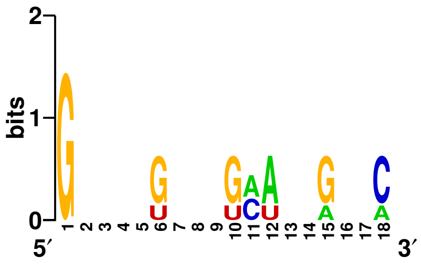

19-mers:

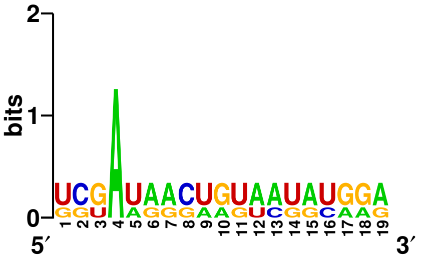

20-mers:

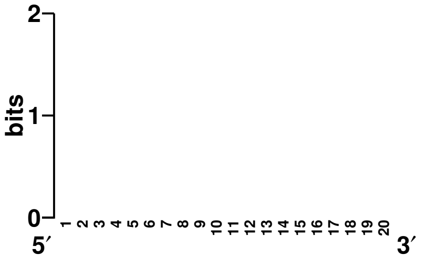

21-mers:

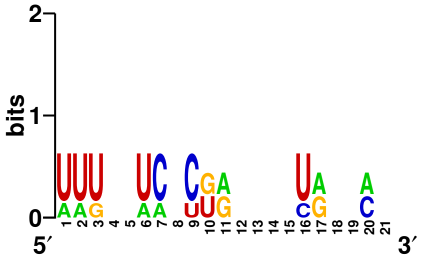

22-mers:

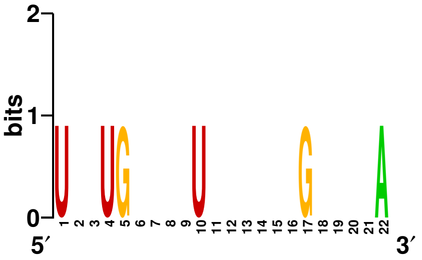

23-mers:

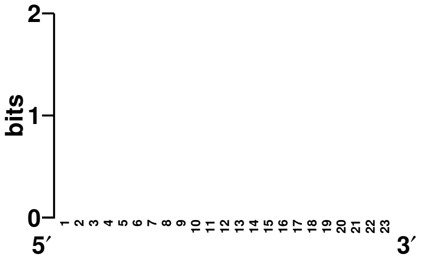

24-mers:

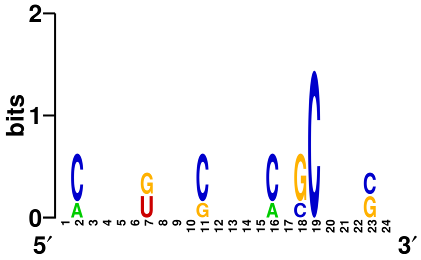

25-mers:

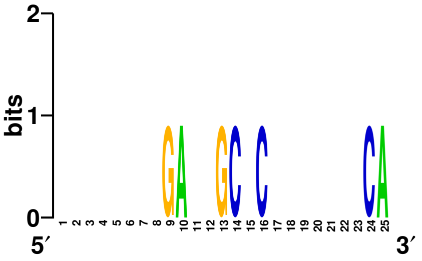

26-mers:

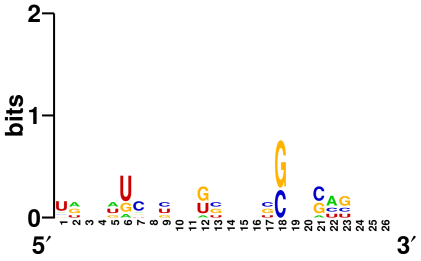

27-mers:

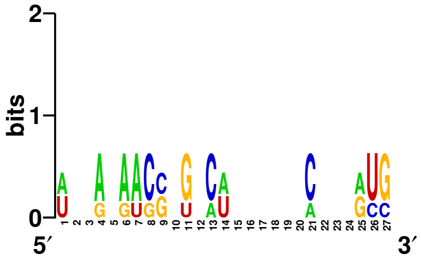

28-mers:

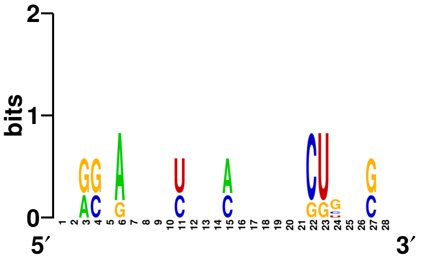

29-mers:

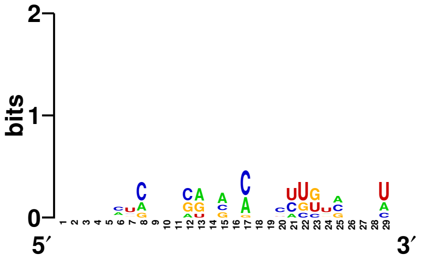

30-mers:

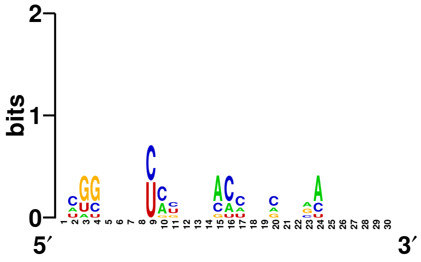

Antisense reads:

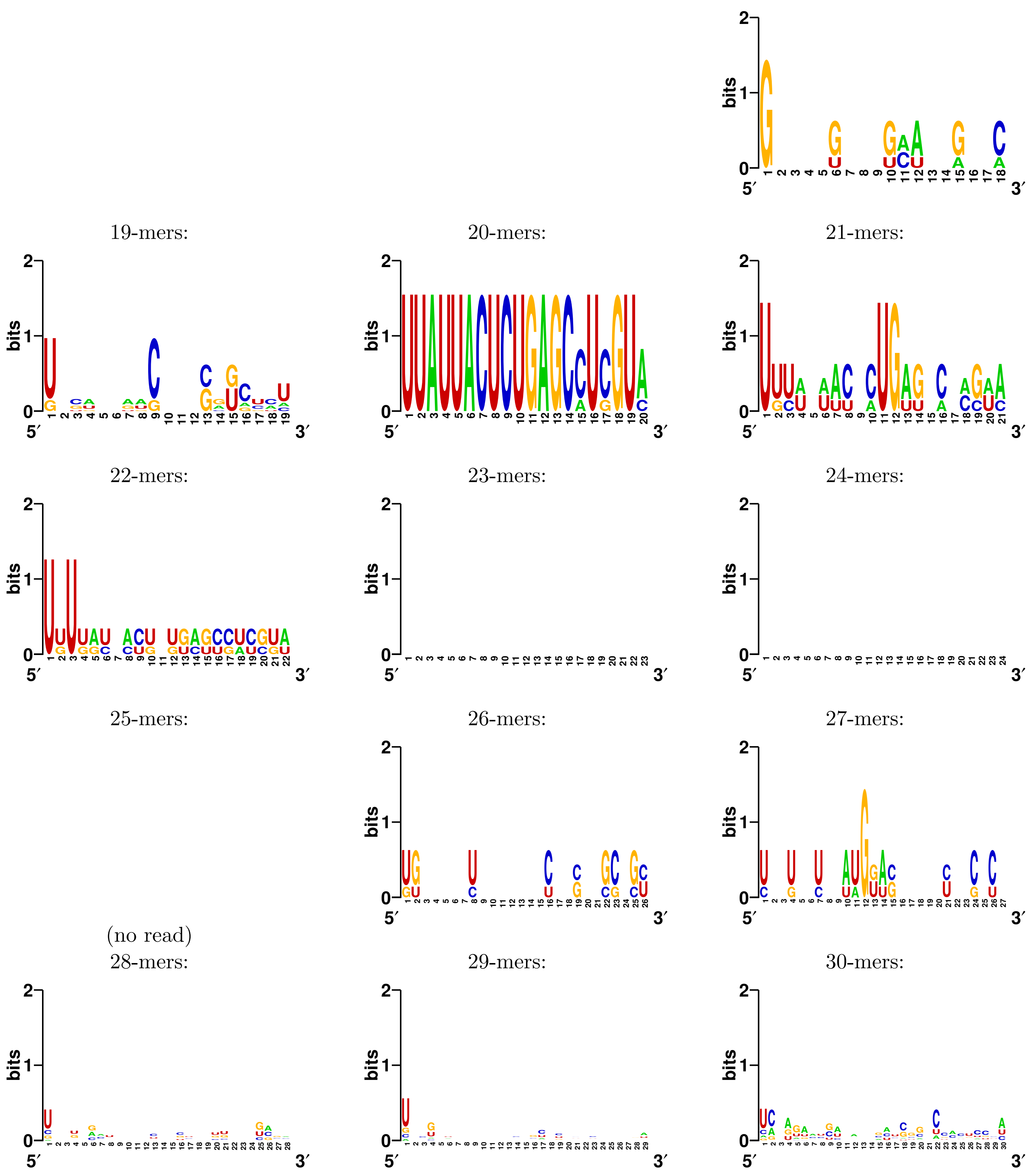

Adult female, library 4:

Sense reads:

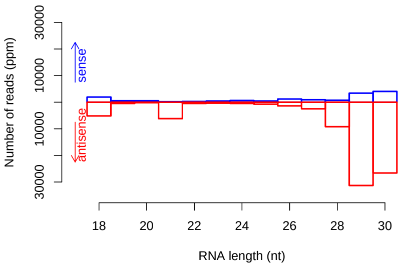

18-mers:

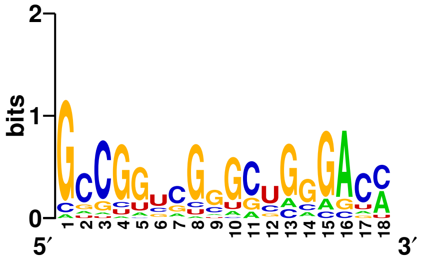

19-mers:

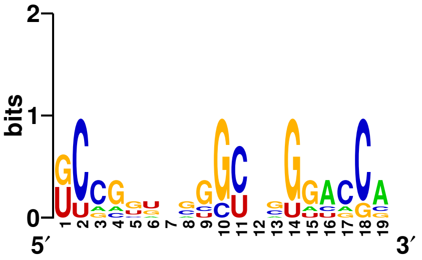

20-mers:

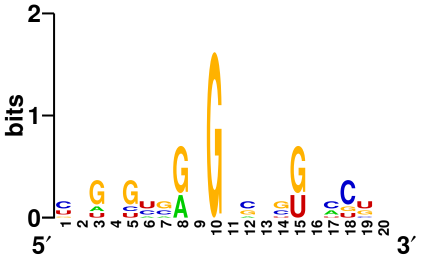

21-mers:

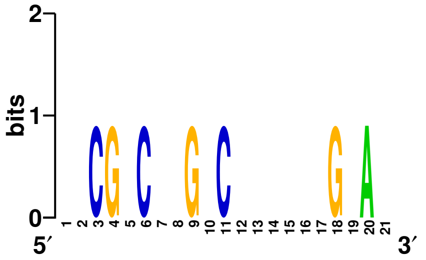

22-mers:

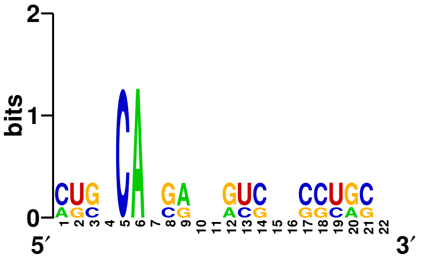

23-mers:

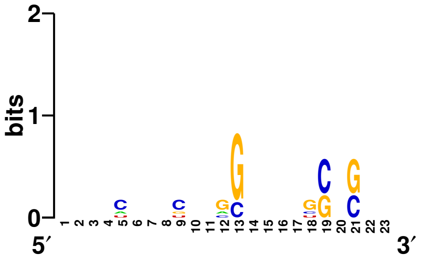

24-mers:

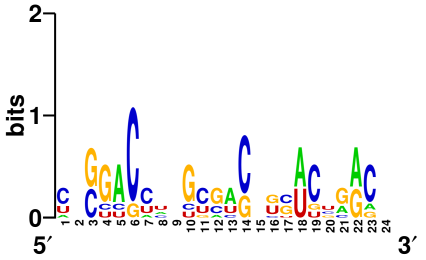

25-mers:

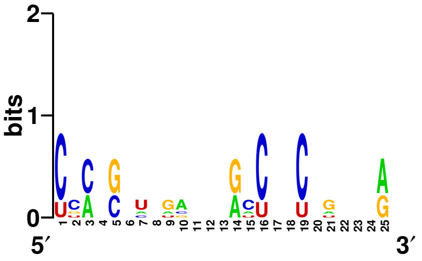

26-mers:

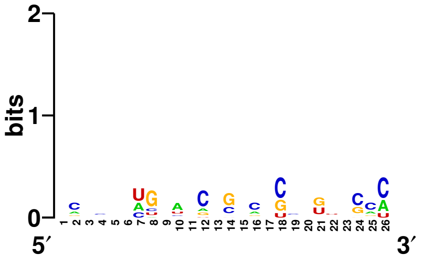

27-mers:

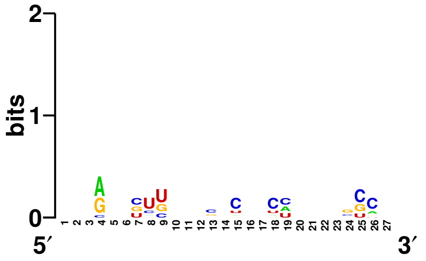

28-mers:

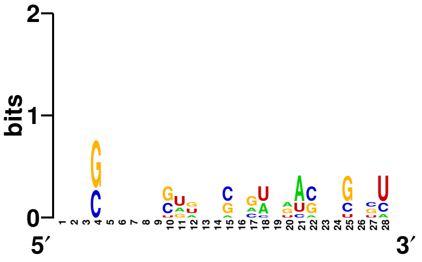

29-mers:

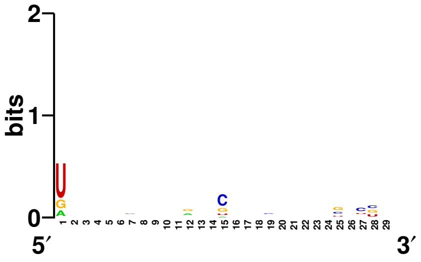

30-mers:

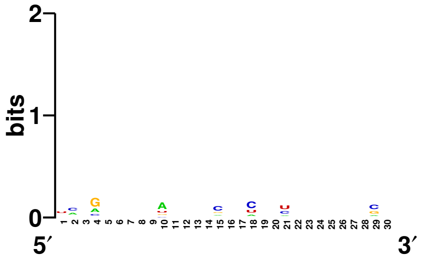

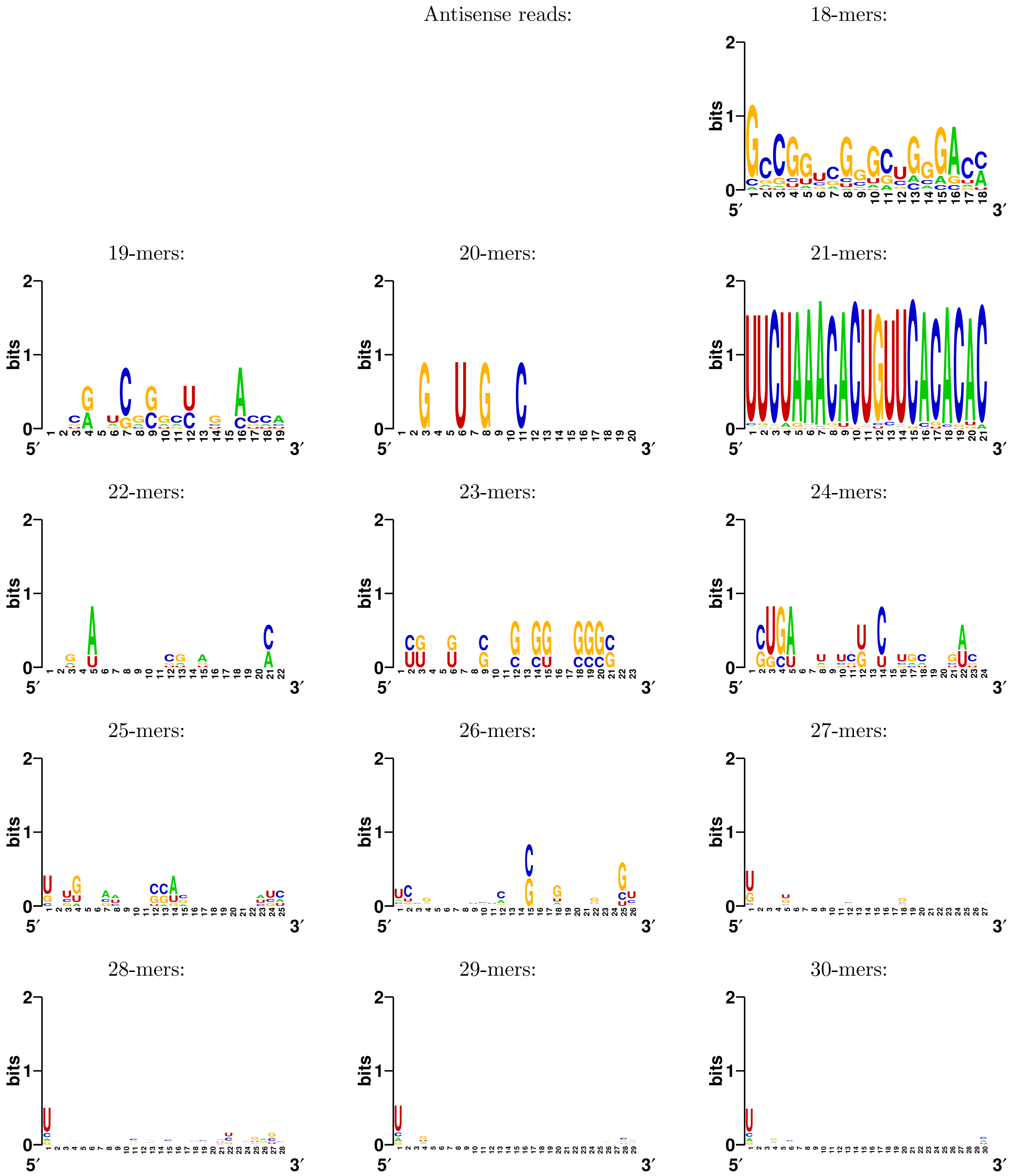
Antisense reads:
18-mers:
19-mers:
20-mers:
21-mers:
22-mers:
23-mers:
24-mers:
25-mers:
26-mers:
27-mers:
28-mers:
29-mers:
30-mers:
bits
5′
3′

Adult male, library 4:

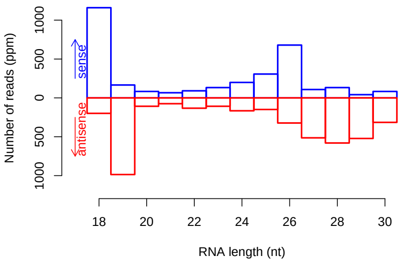

Sense reads:

18-mers:

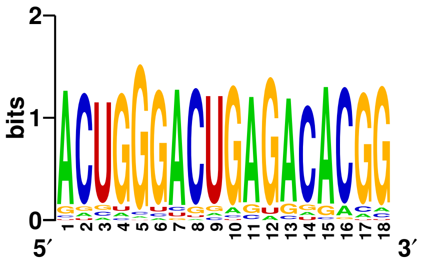

19-mers:

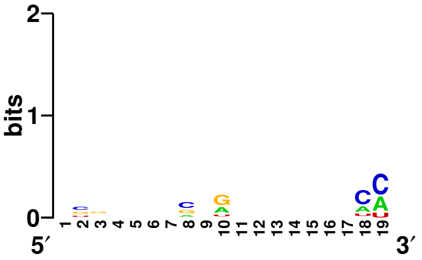

20-mers:

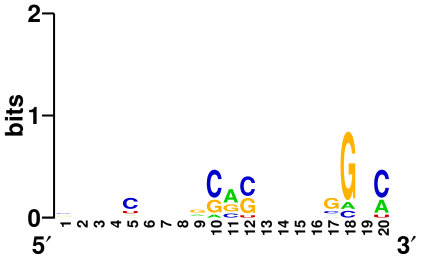

21-mers:

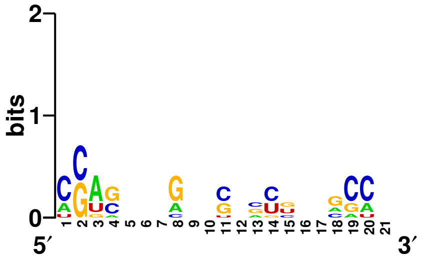

22-mers:

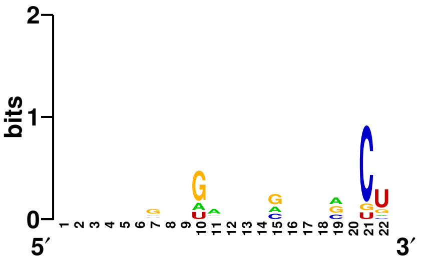

23-mers:

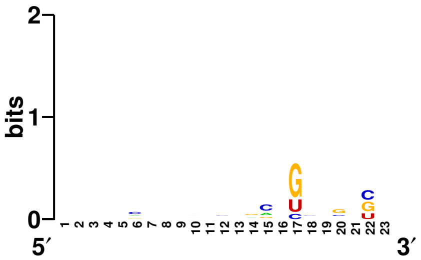

24-mers:

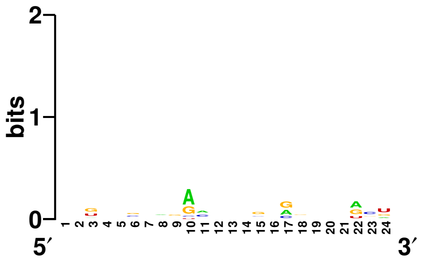

25-mers:

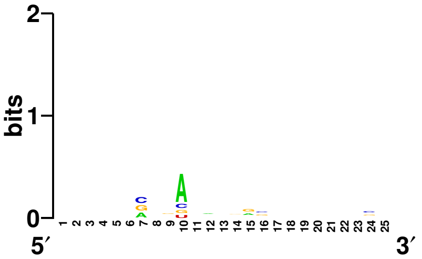

26-mers:

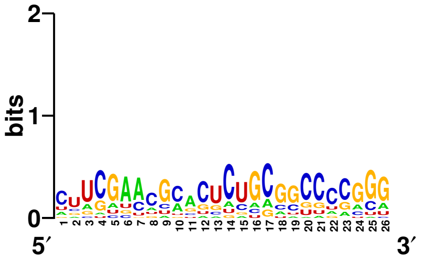

27-mers:

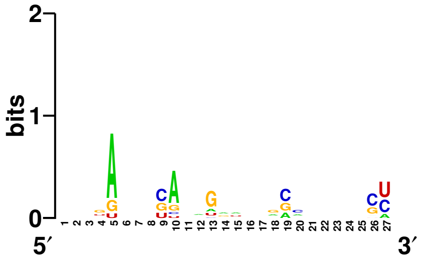

28-mers:

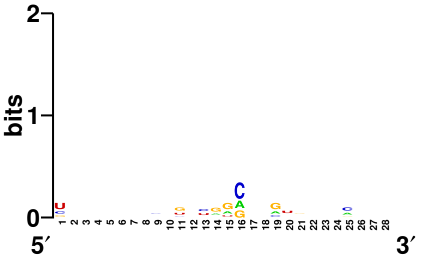

29-mers:

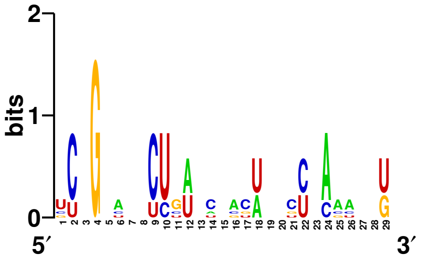

30-mers:

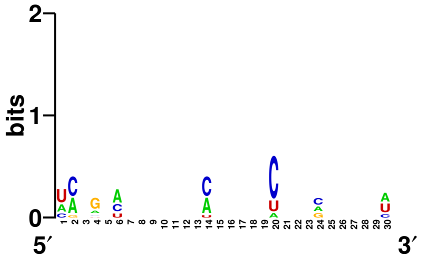

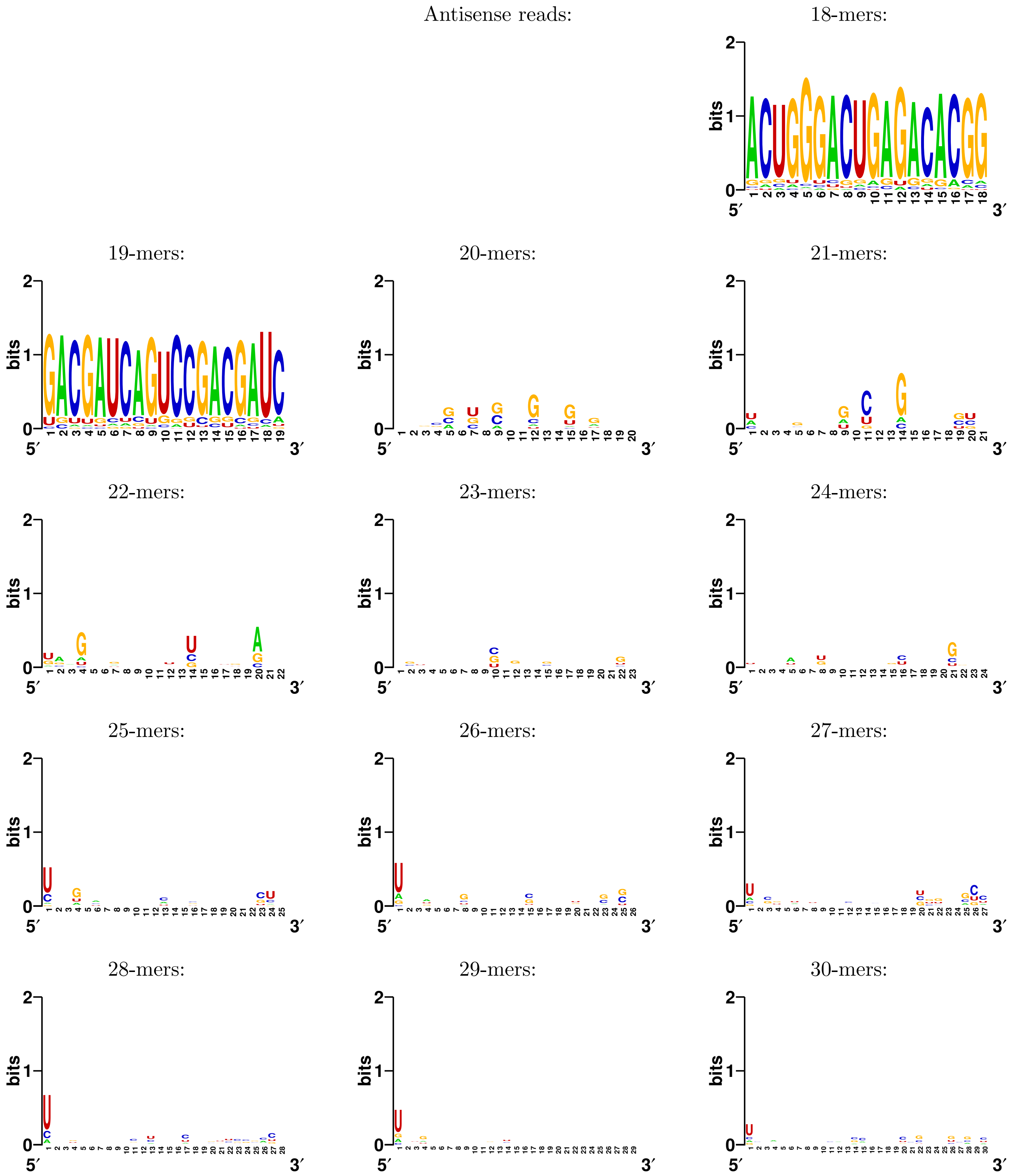
Antisense reads:
18-mers:
19-mers:
20-mers:
21-mers:
22-mers:
23-mers:
24-mers:
25-mers:
26-mers:
27-mers:
28-mers:
29-mers:
30-mers:
bits
5′
3′

5 Extragenomic and extratranscriptomic reads

5.1 Libraries #1 (total 5´ monophosphorylated small RNAs)

Embryo 8h, library 1:

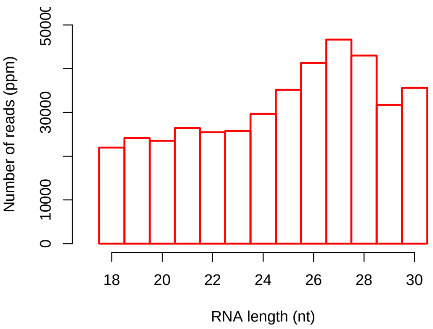

18-mers:

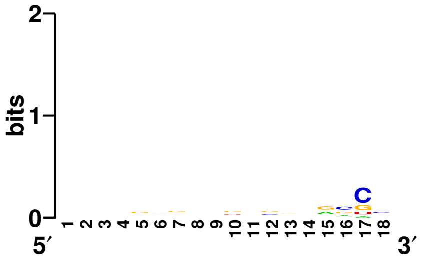

19-mers:

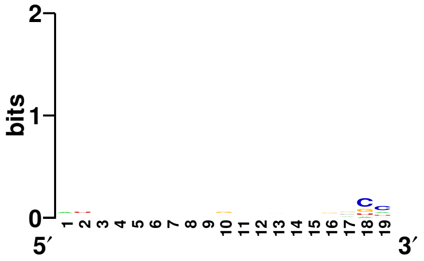

20-mers:

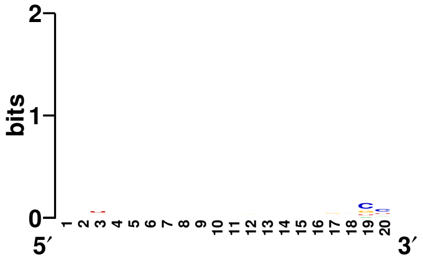

21-mers:

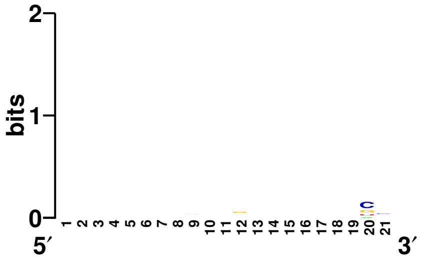

22-mers:

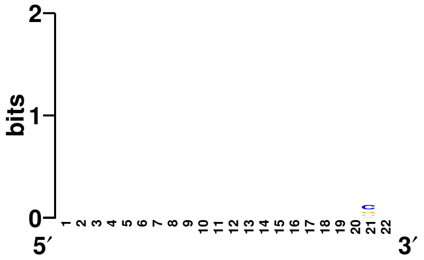

23-mers:

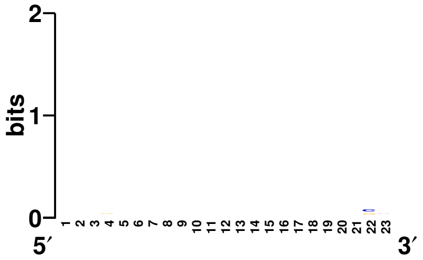

24-mers:

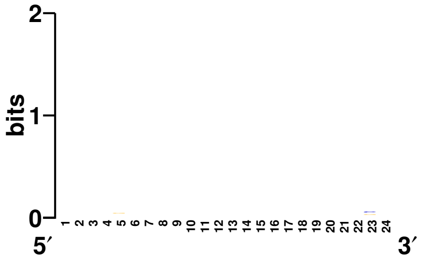

25-mers:

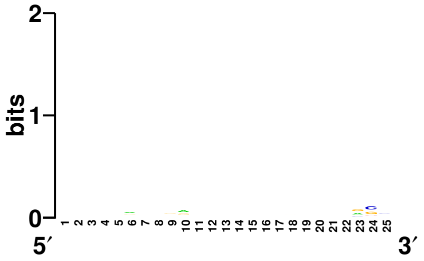

26-mers:

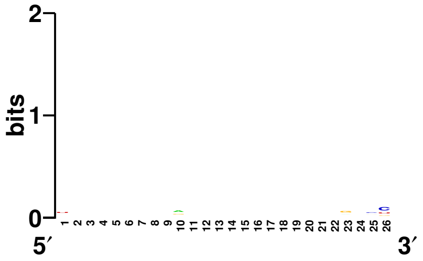

27-mers:

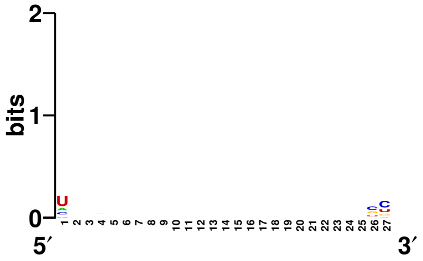

28-mers:

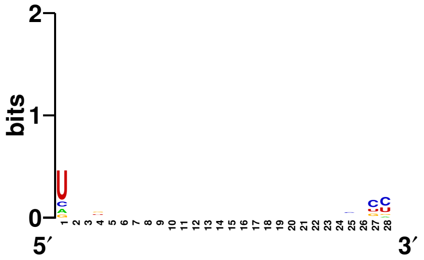

29-mers:

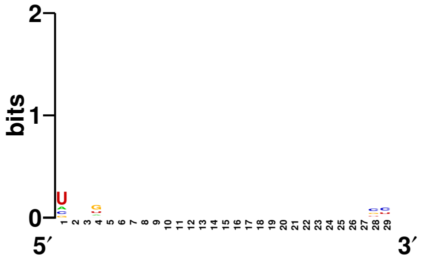

30-mers:

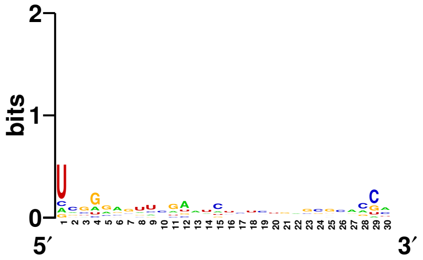

Embryo 15h, library 1:

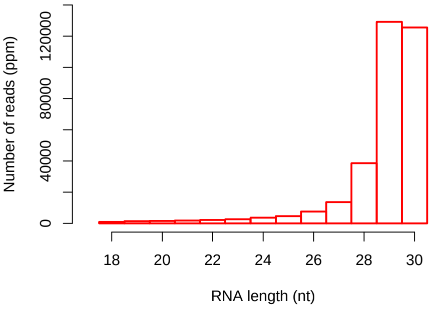

18-mers:

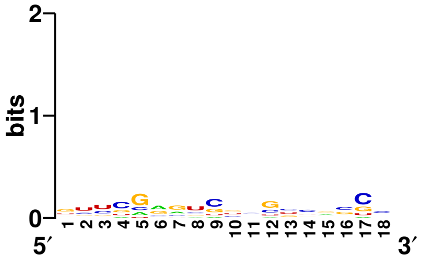

19-mers:

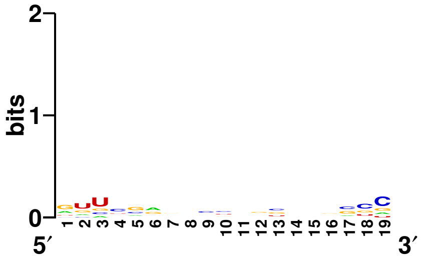

20-mers:

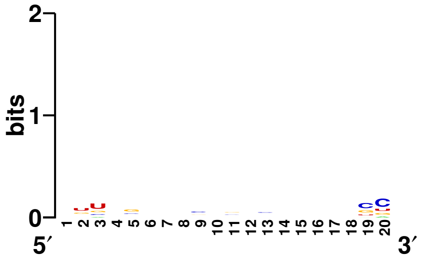

21-mers:

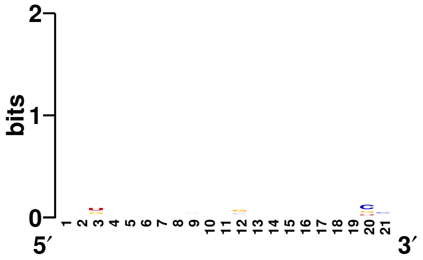

22-mers:

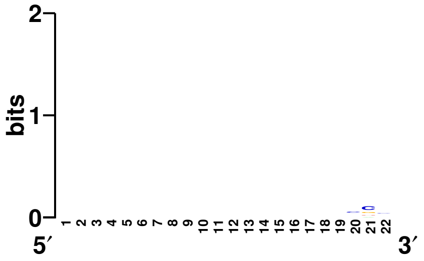

23-mers:

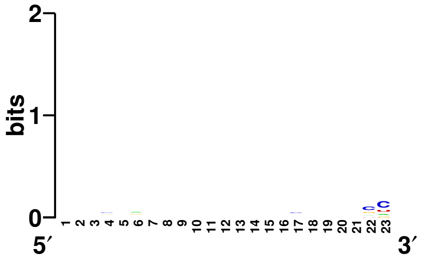

24-mers:

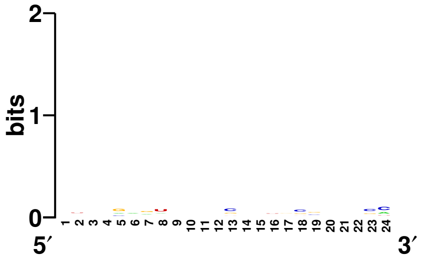

25-mers:

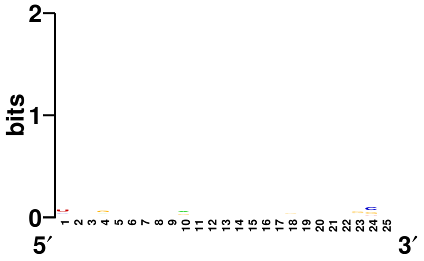

26-mers:

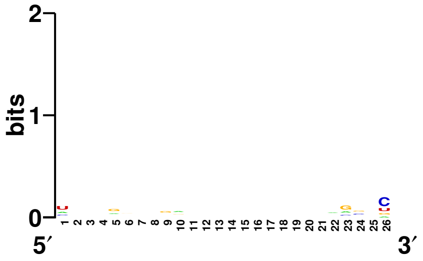

27-mers:

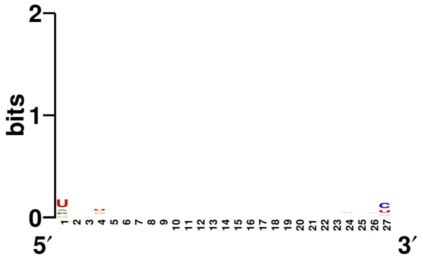

28-mers:

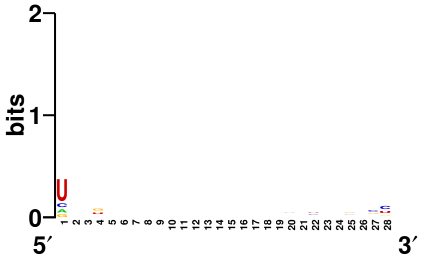

29-mers:

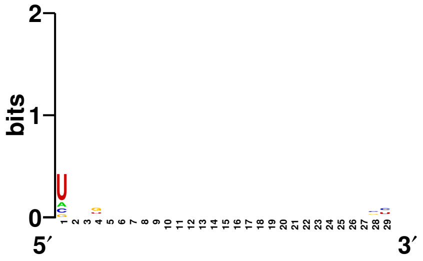

30-mers:

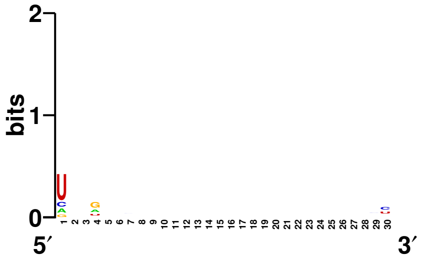

Embryo 36h, library 1:

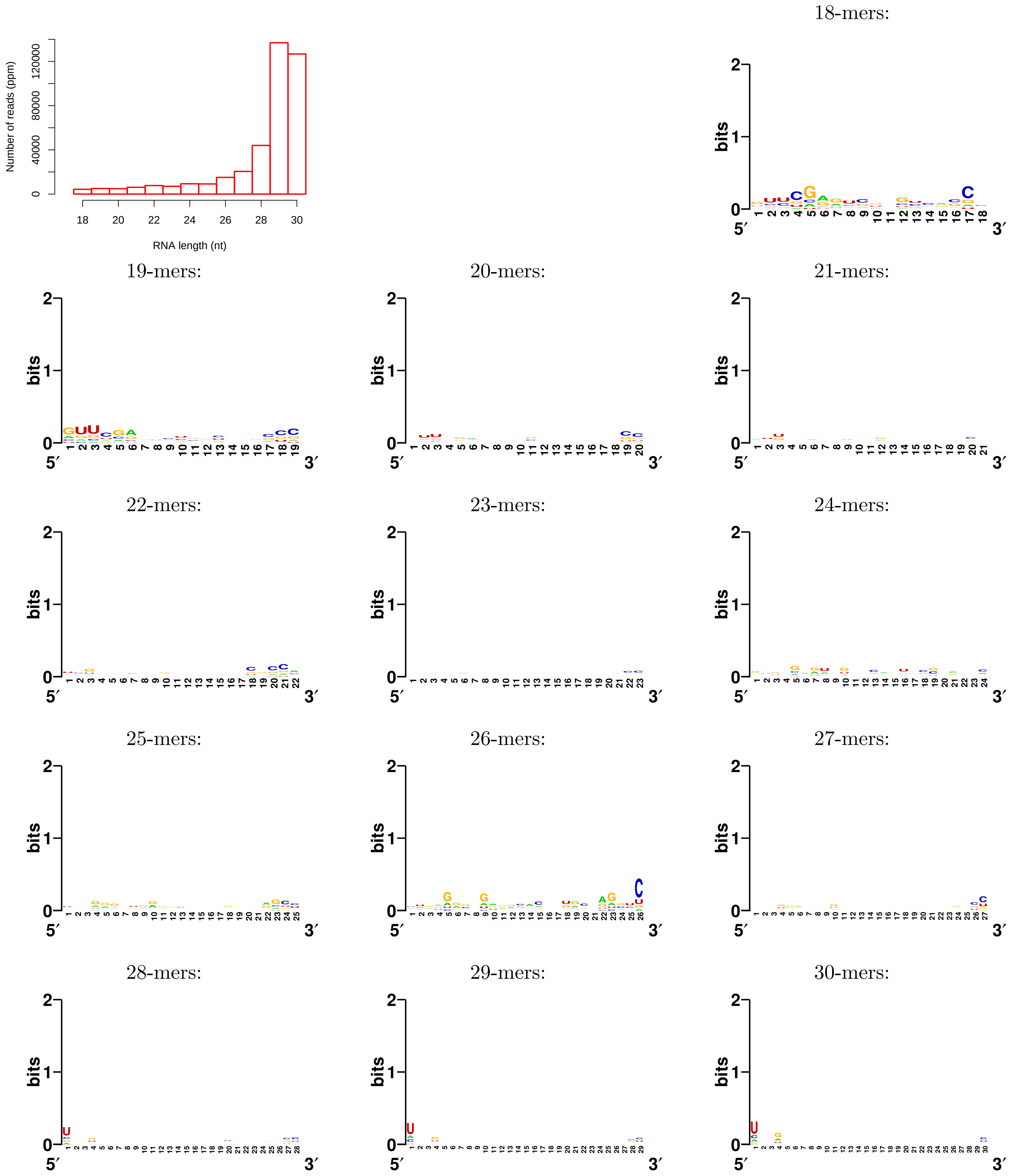

Embryo 60h, library 1:

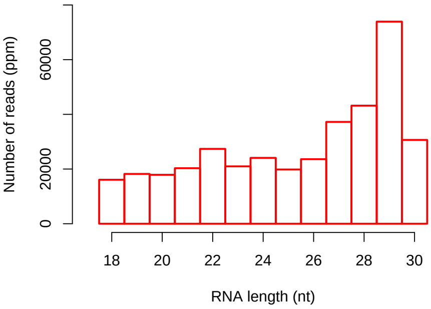

18-mers:

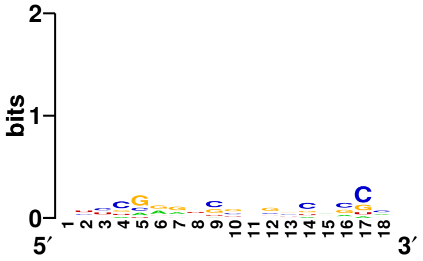

19-mers:

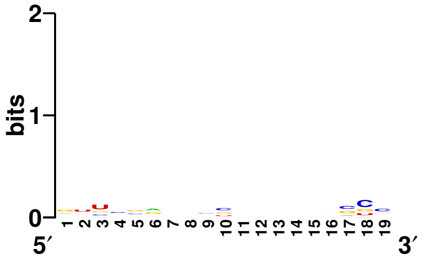

20-mers:

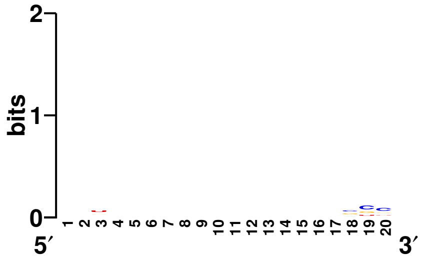

21-mers:

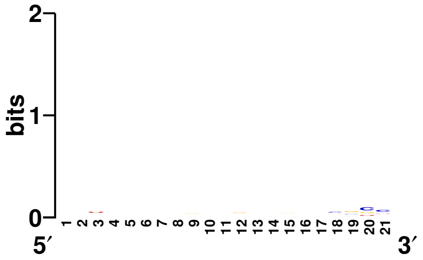

22-mers:

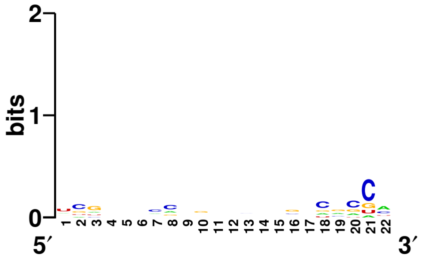

23-mers:

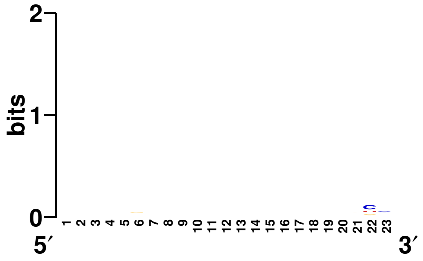

24-mers:

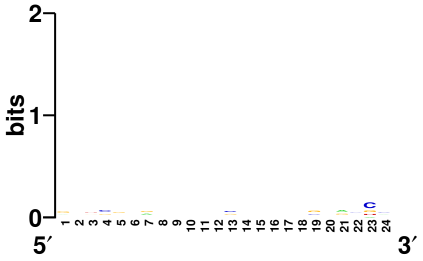

25-mers:

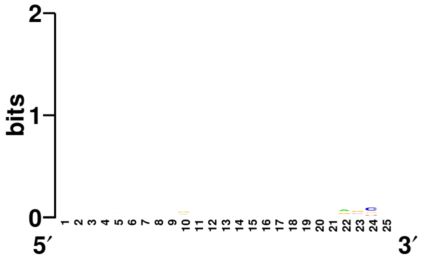

26-mers:

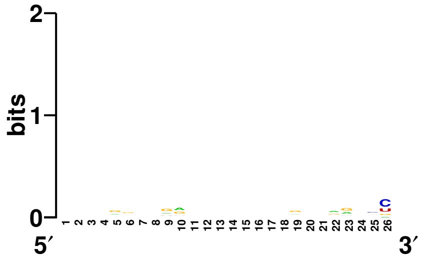

27-mers:

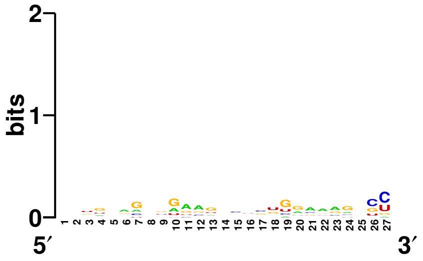

28-mers:

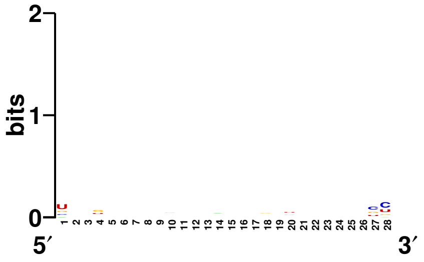

29-mers:

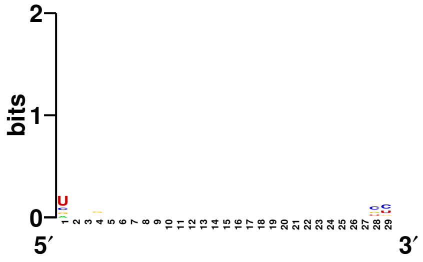

30-mers:

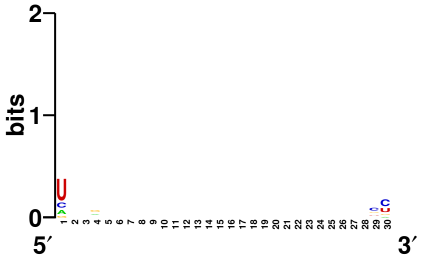

Adult female, library 1:

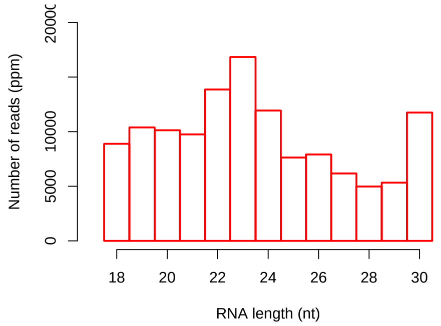

18-mers:

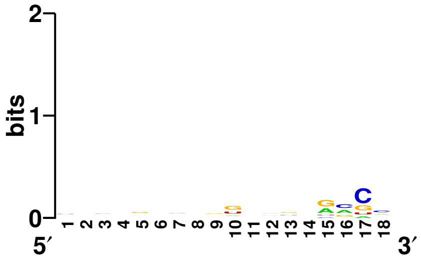

19-mers:

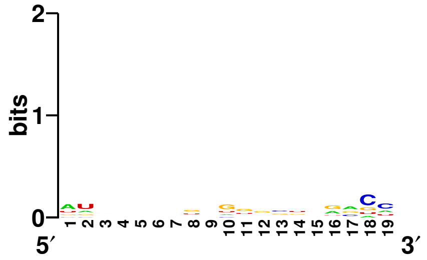

20-mers:

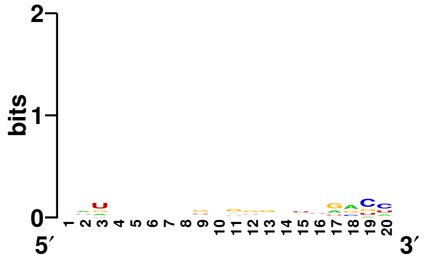

21-mers:

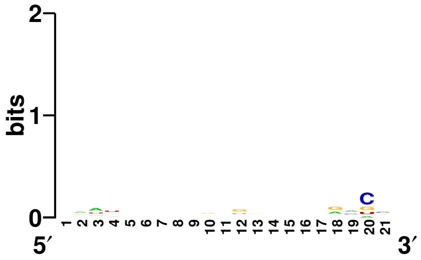

22-mers:

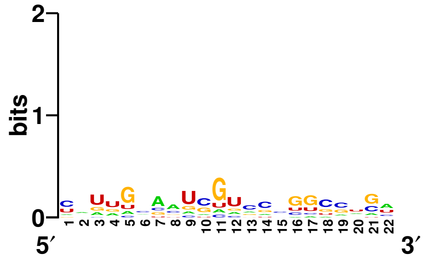

23-mers:

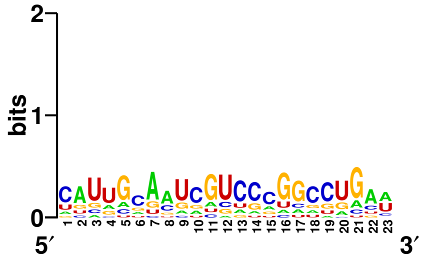

24-mers:

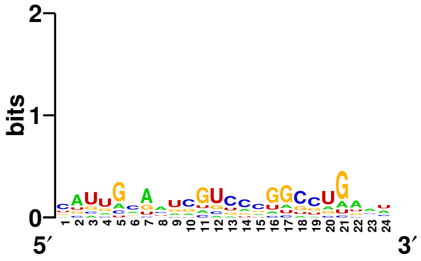

25-mers:

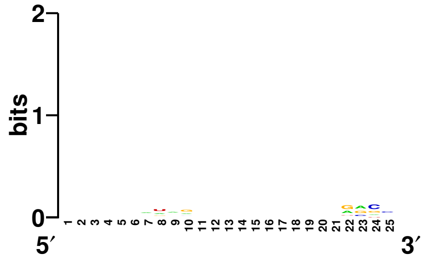

26-mers:

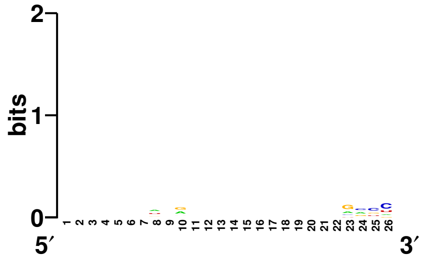

27-mers:

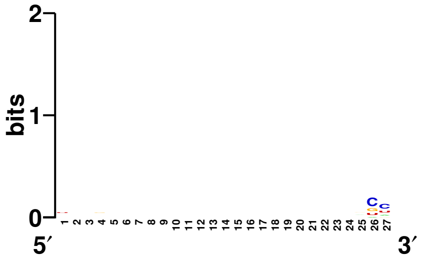

28-mers:

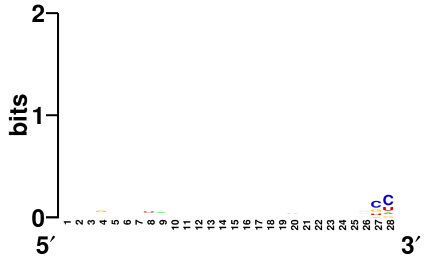

29-mers:

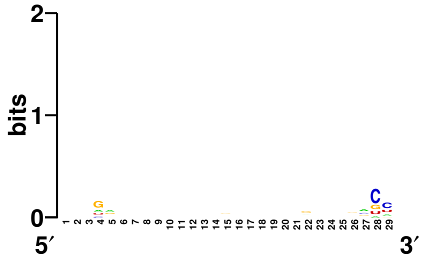

30-mers:

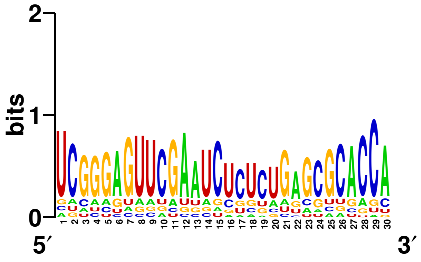

Adult male, library 1:

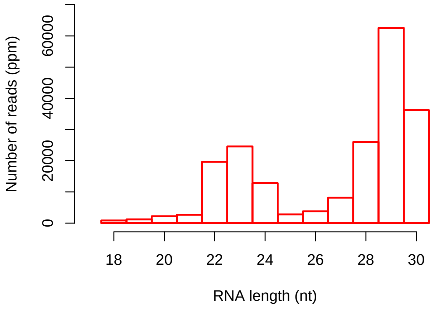

18-mers:

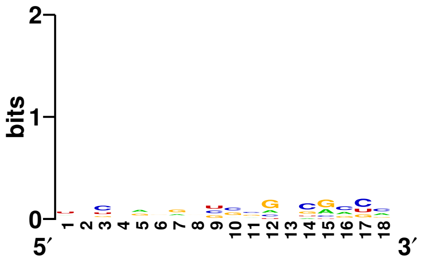

19-mers:

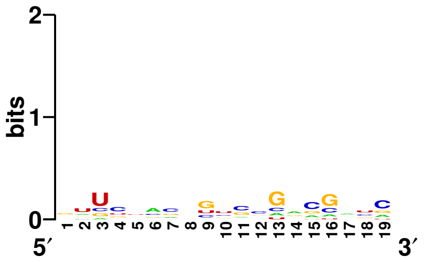

20-mers:

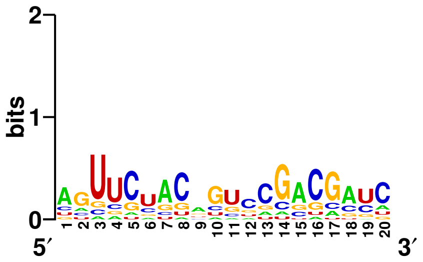

21-mers:

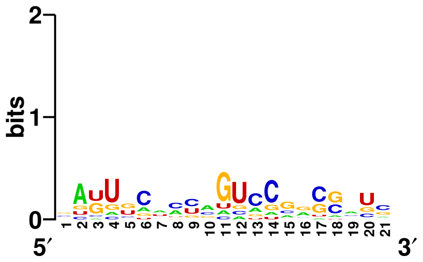

22-mers:

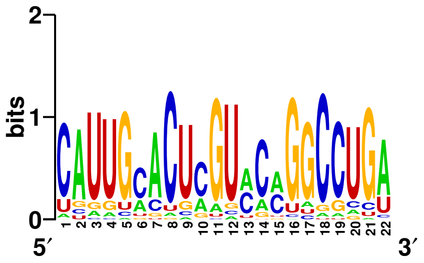

23-mers:

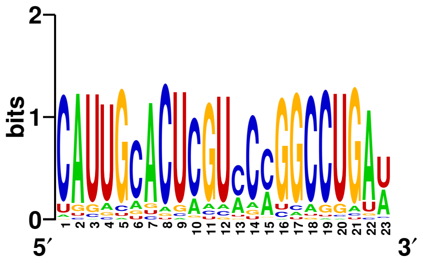

24-mers:

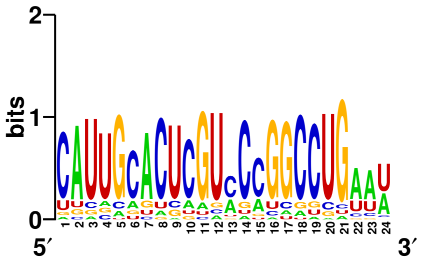

25-mers:

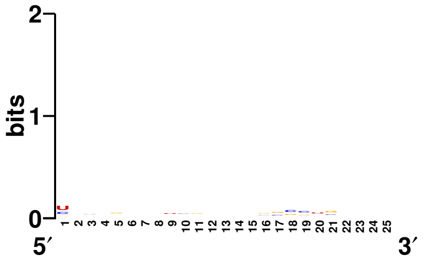

26-mers:

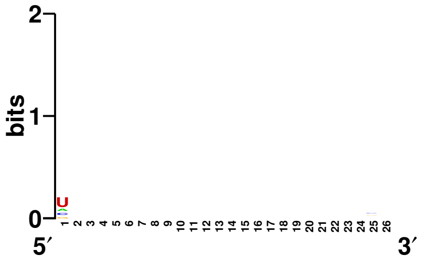

27-mers:

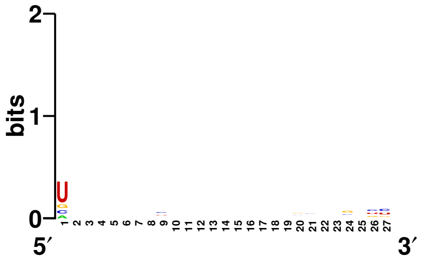

28-mers:

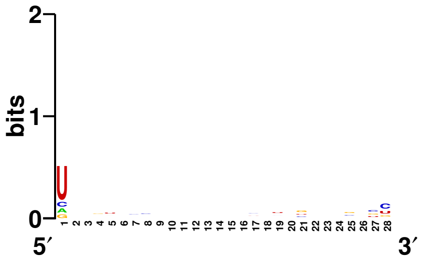

29-mers:

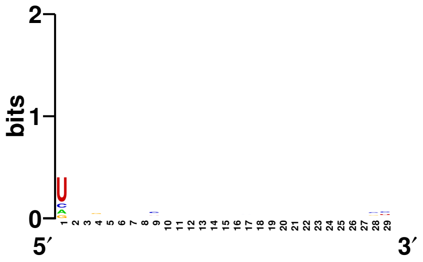

30-mers:

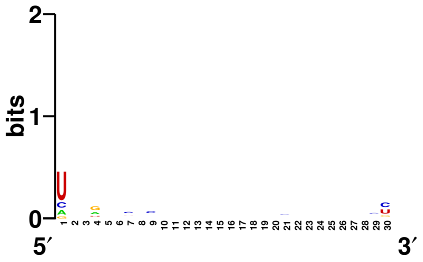

5.2 Libraries #2 (3´ modified, 5´ monophosphorylated small RNAs)

Embryo 8h, library 2:

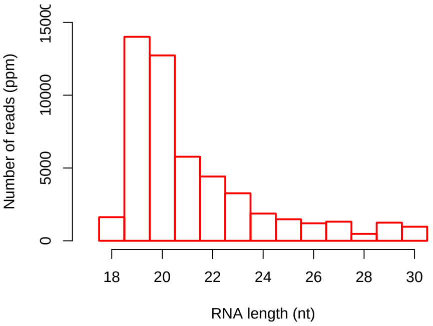

18-mers:

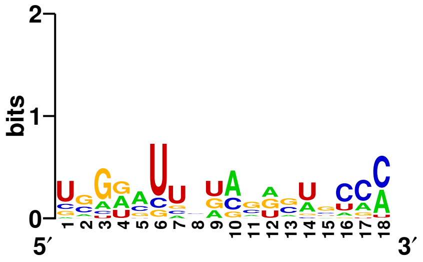

19-mers:

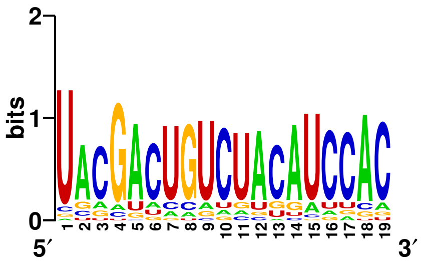

20-mers:

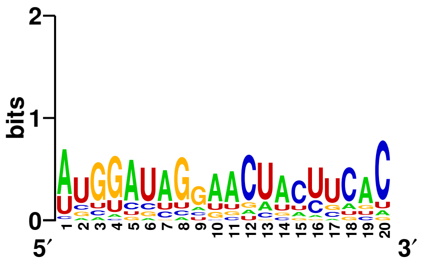

21-mers:

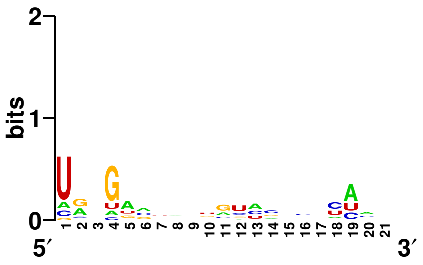

22-mers:

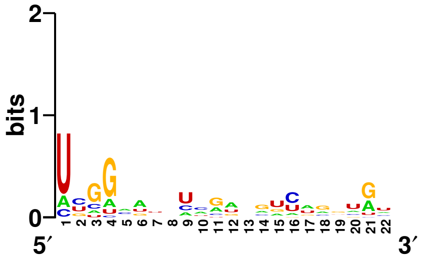

23-mers:

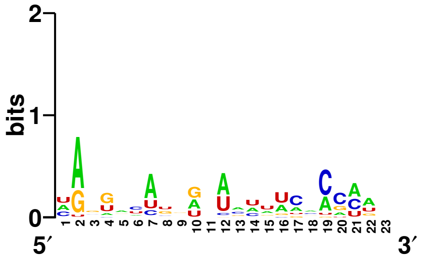

24-mers:

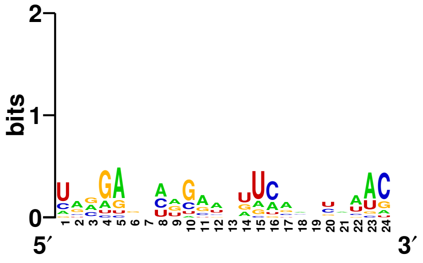

25-mers:

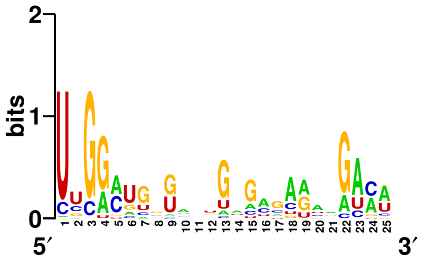

26-mers:

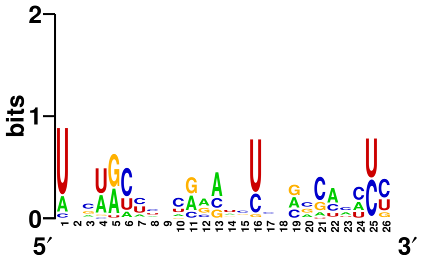

27-mers:

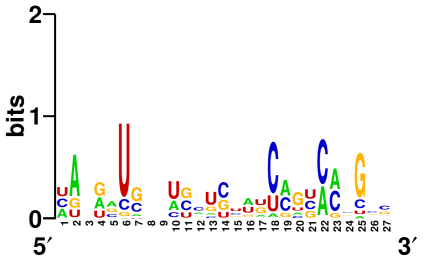

28-mers:

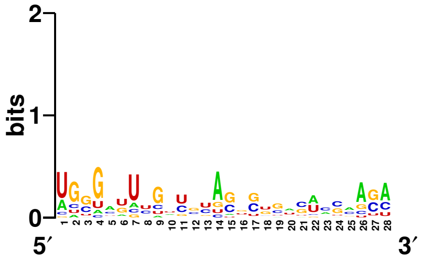

29-mers:

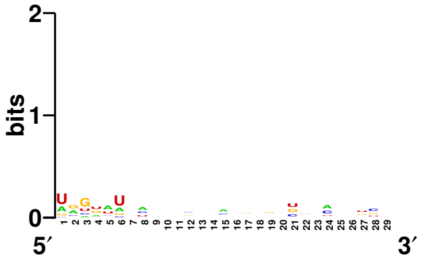

30-mers:

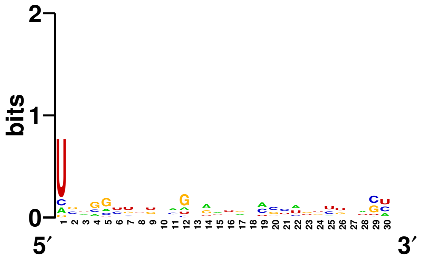

Embryo 15h, library 2:

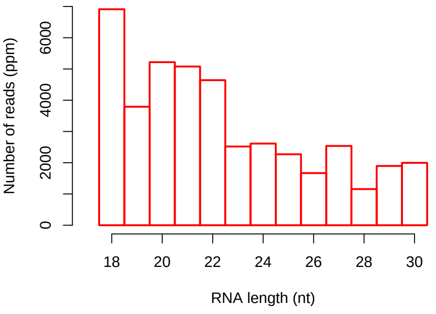

18-mers:

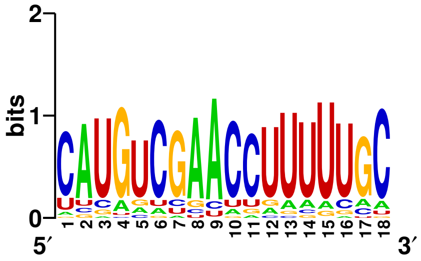

19-mers:

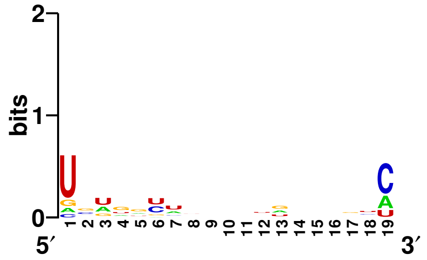

20-mers:

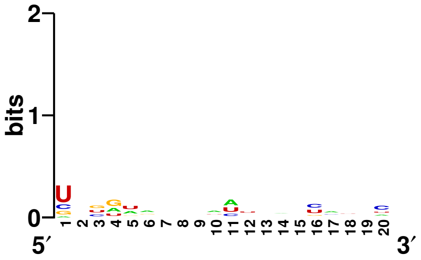

21-mers:

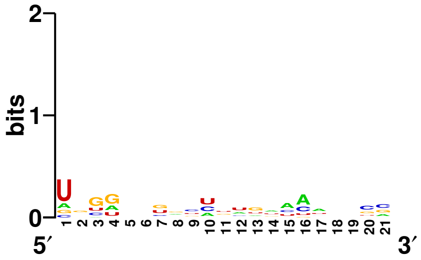

22-mers:

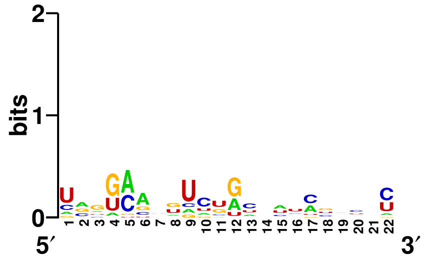

23-mers:

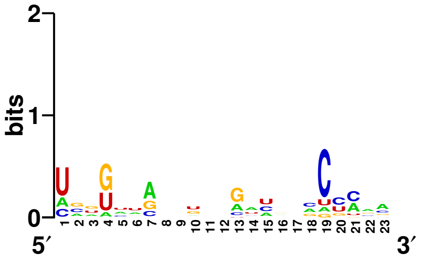

24-mers:

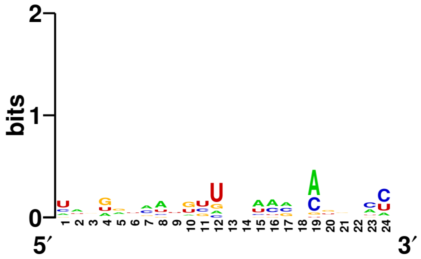

25-mers:

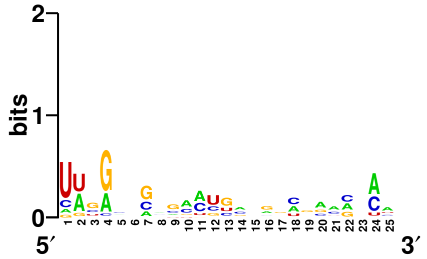

26-mers:

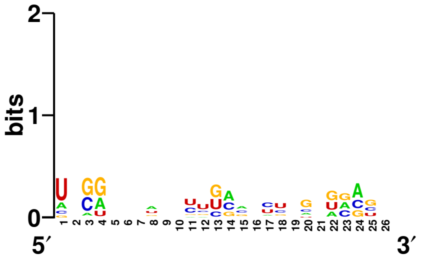

27-mers:

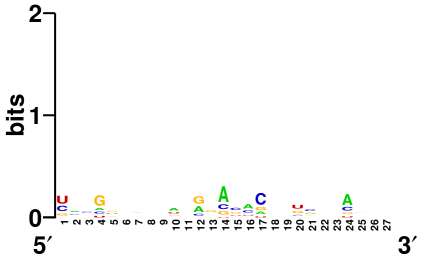

28-mers:

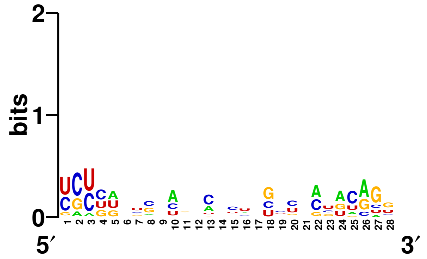

29-mers:

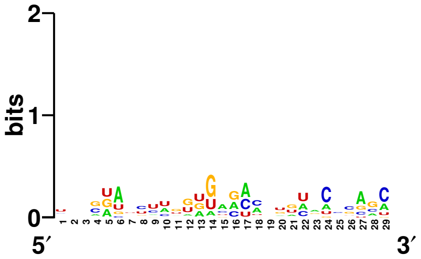

30-mers:

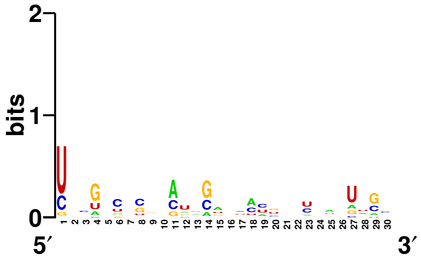

Embryo 36h, library 2:

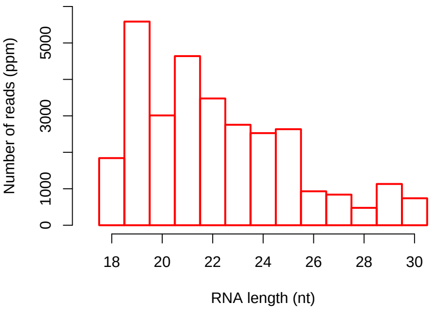

18-mers:

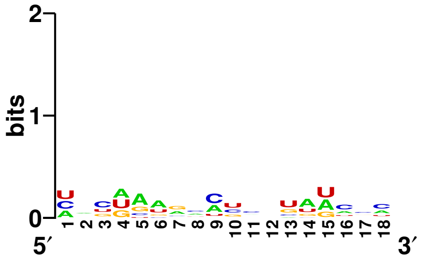

19-mers:

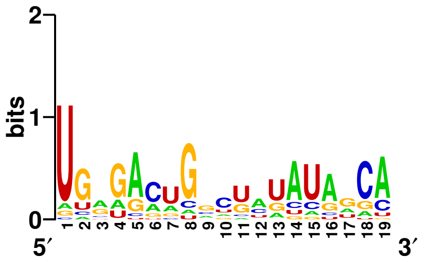

20-mers:

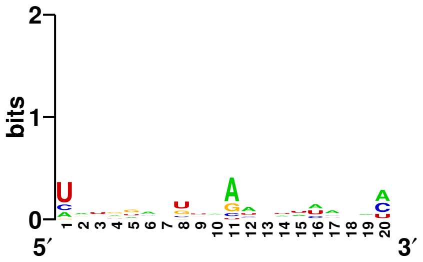

21-mers:

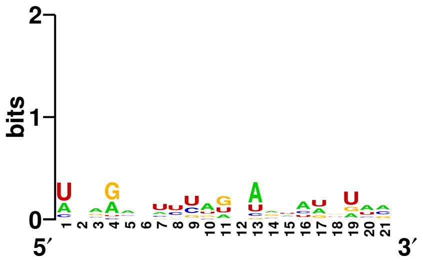

22-mers:

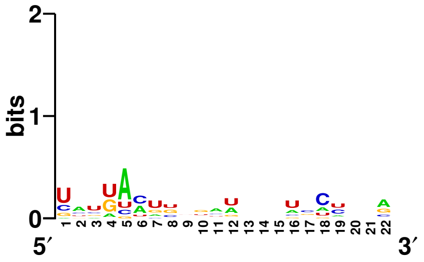

23-mers:

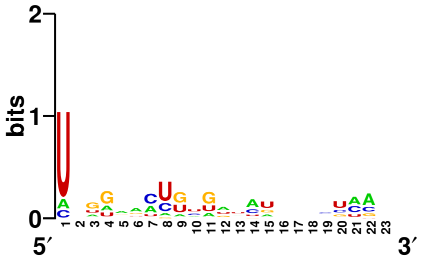

24-mers:

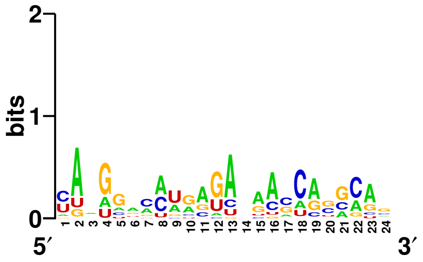

25-mers:

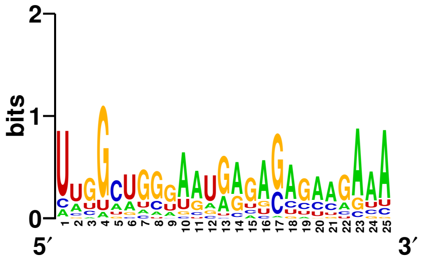

26-mers:

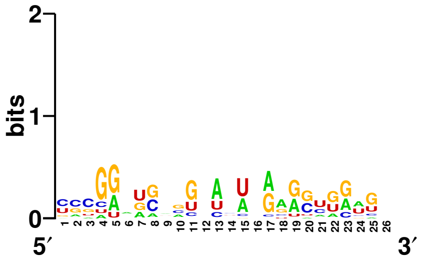

27-mers:

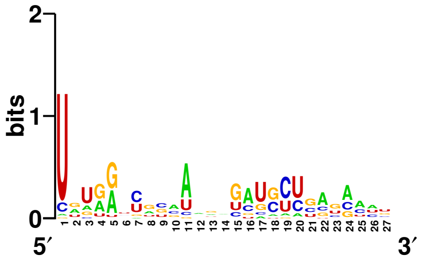

28-mers:

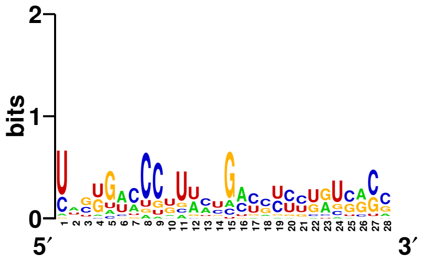

29-mers:

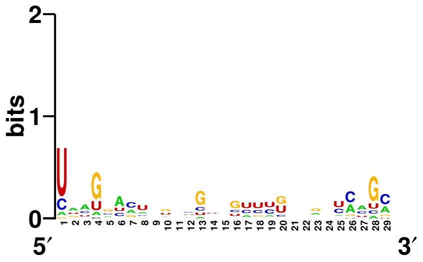

30-mers:

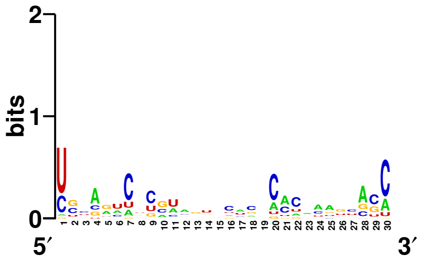

Embryo 60h, library 2:

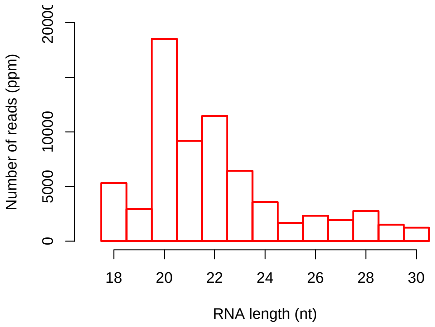

18-mers:

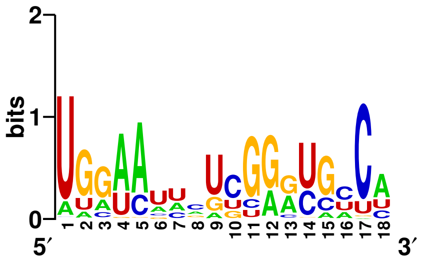

19-mers:

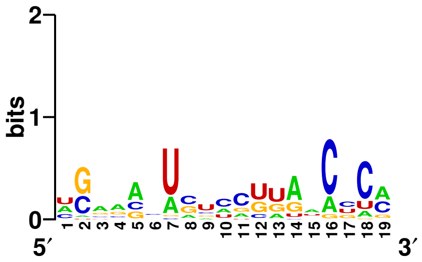

20-mers:

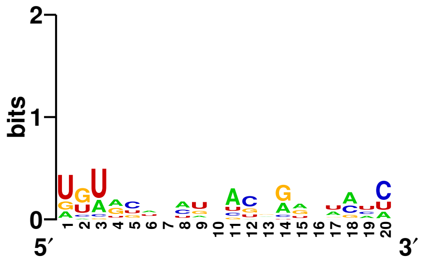

21-mers:

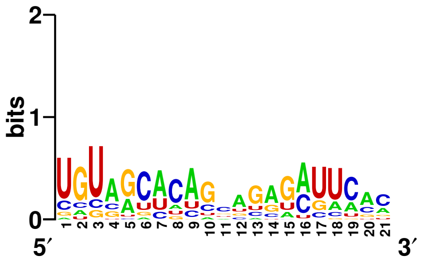

22-mers:

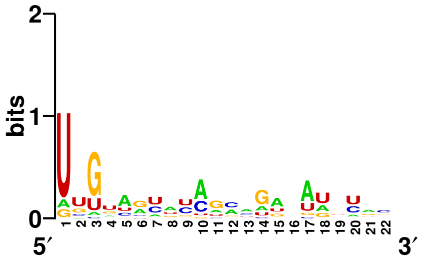

23-mers:

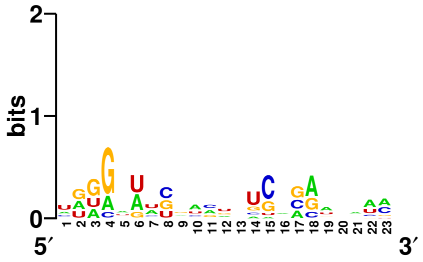

24-mers:

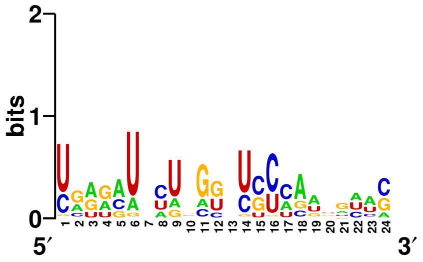

25-mers:

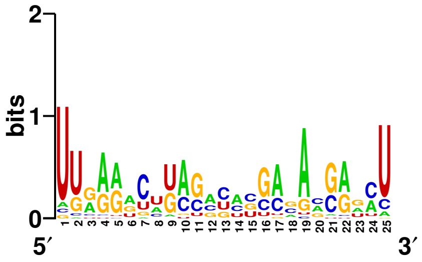

26-mers:

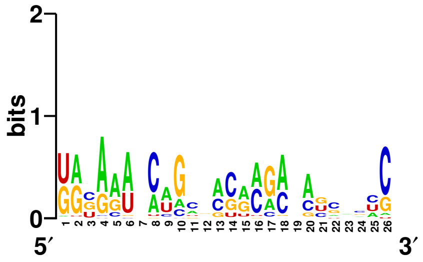

27-mers:

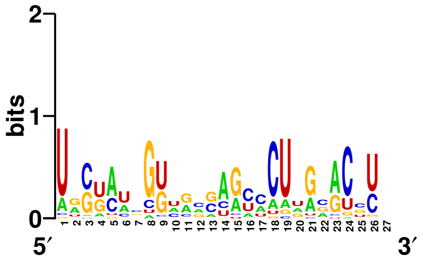

28-mers:

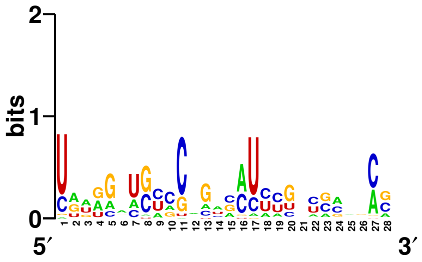

29-mers:

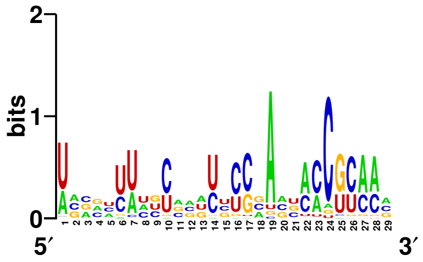

30-mers:

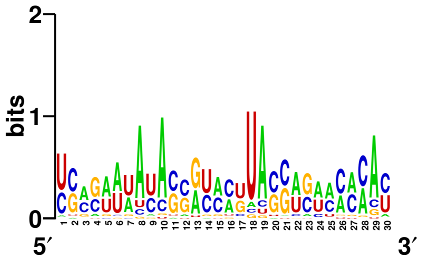

Adult female, library 2:

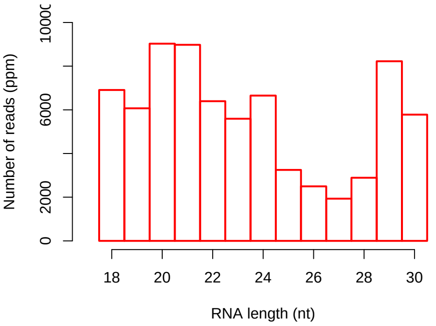

18-mers:

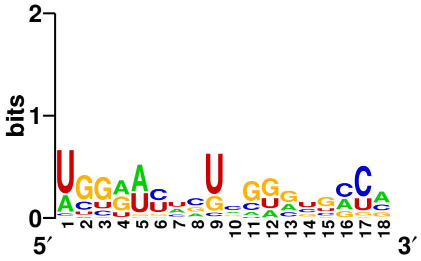

19-mers:

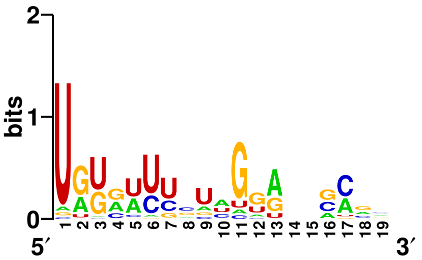

20-mers:

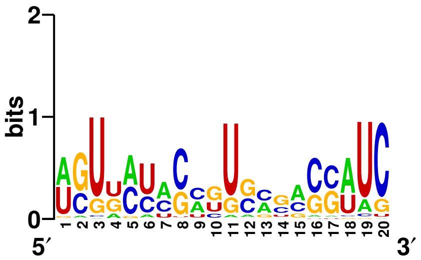

21-mers:

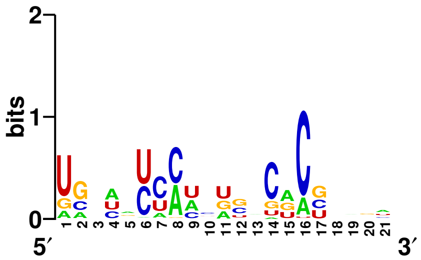

22-mers:

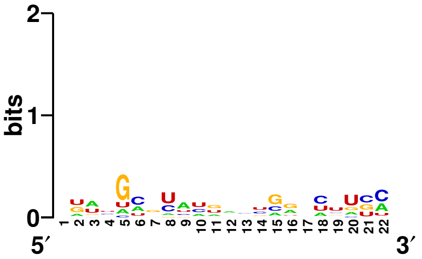

23-mers:

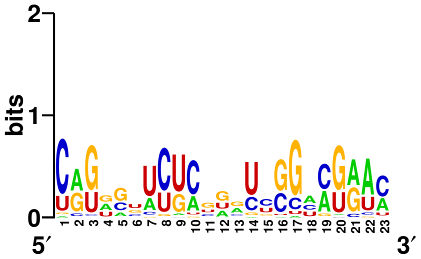

24-mers:

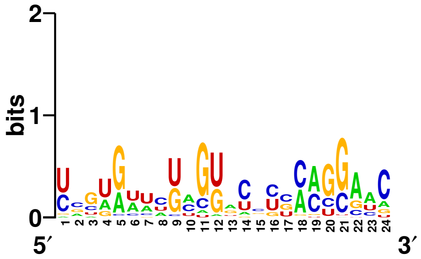

25-mers:

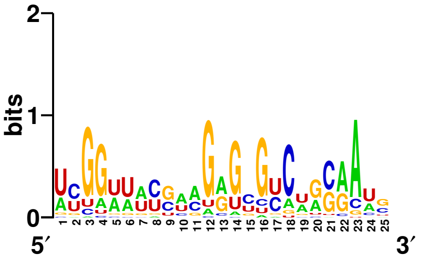

26-mers:

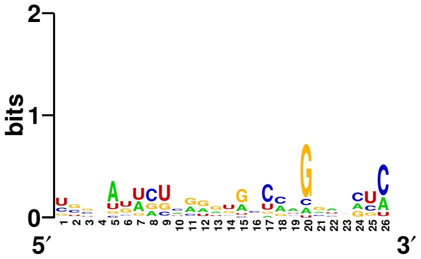

27-mers:

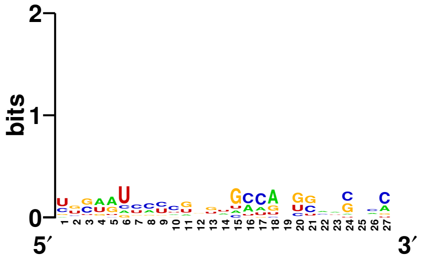

28-mers:

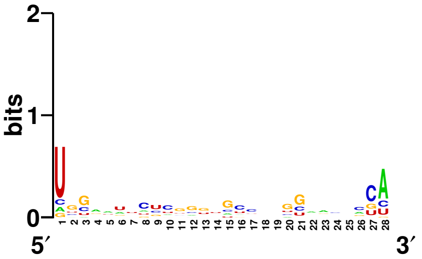

29-mers:

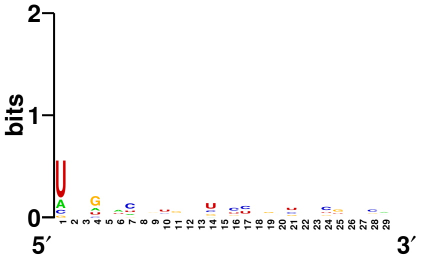

30-mers:

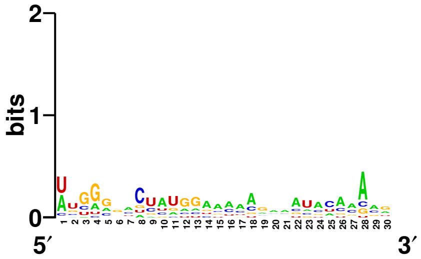

Adult male, library 2:

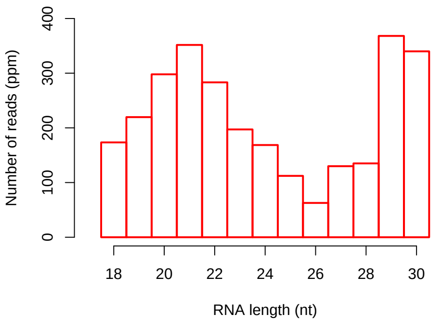

18-mers:

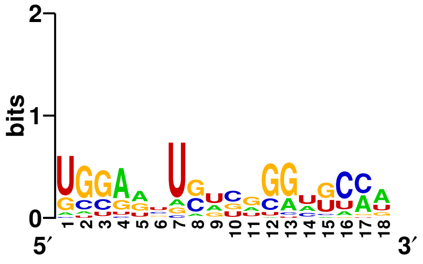

19-mers:

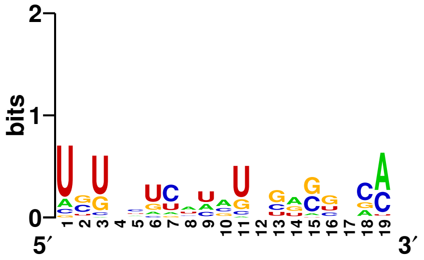

20-mers:

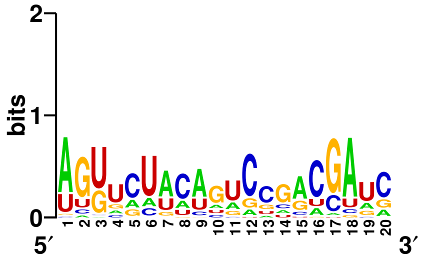

21-mers:

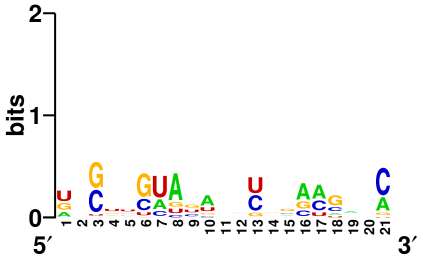

22-mers:

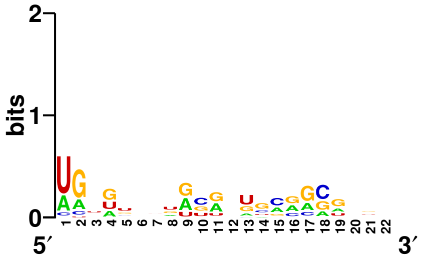

23-mers:

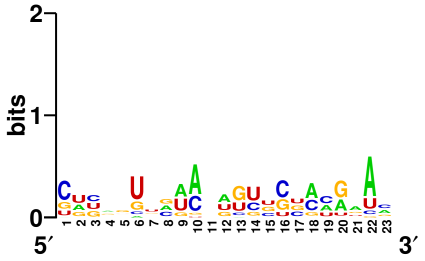

24-mers:

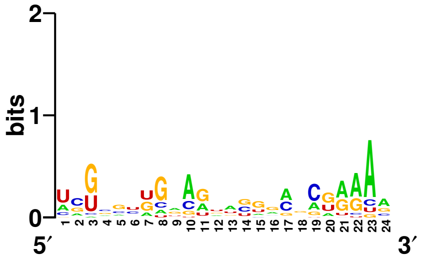

25-mers:

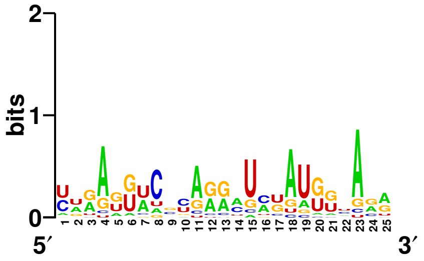

26-mers:

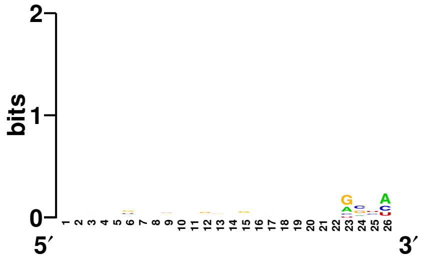

27-mers:

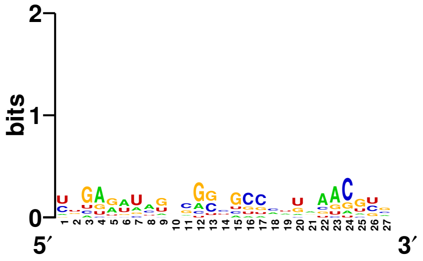

28-mers:

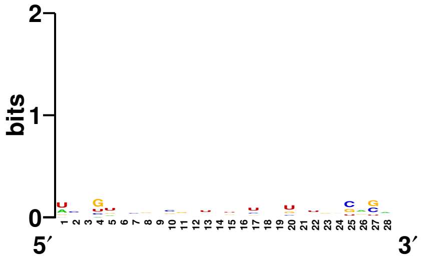

29-mers:

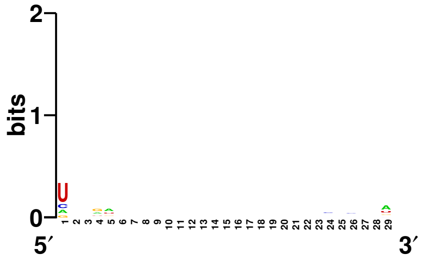

30-mers:

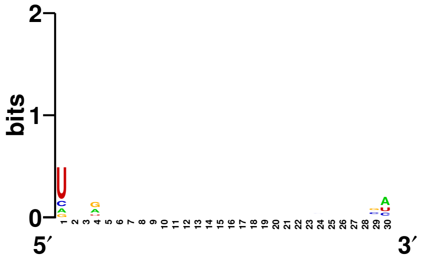

5.3 Libraries #3 (total 5´ hydroxyl or polyphosphorylated small RNAs)

Embryo 8h, library 3:

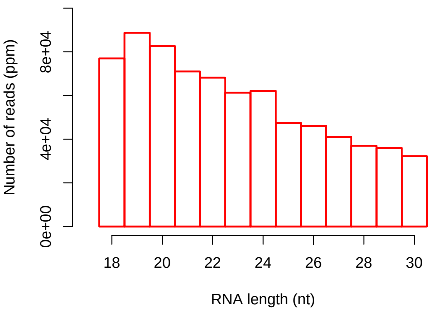

18-mers:

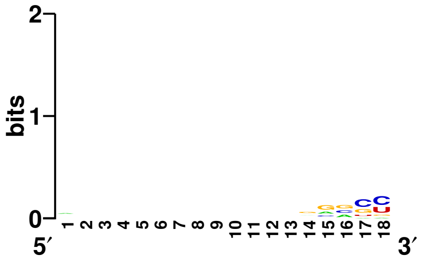

19-mers:

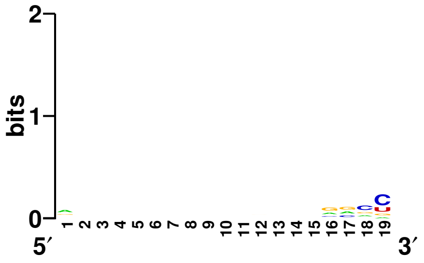

20-mers:

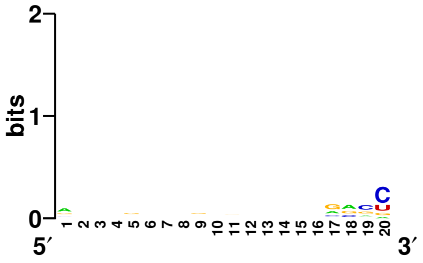

21-mers:

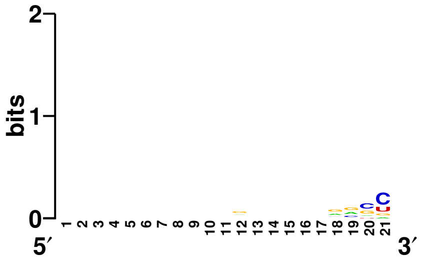

22-mers:

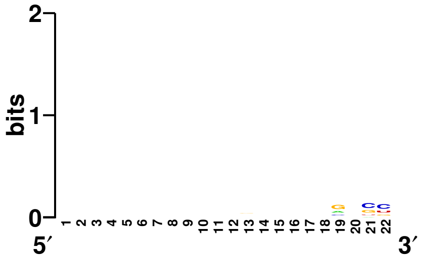

23-mers:

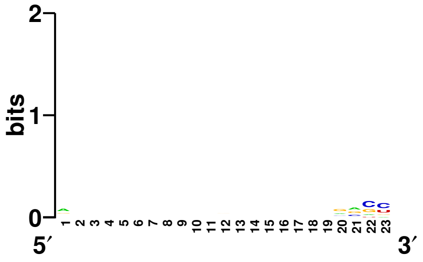

24-mers:

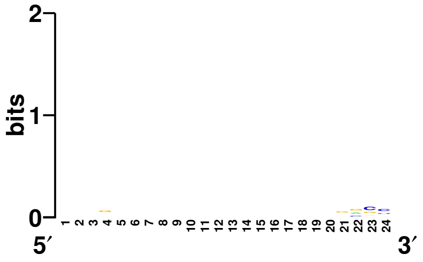

25-mers:

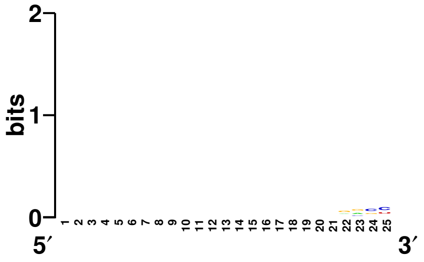

26-mers:

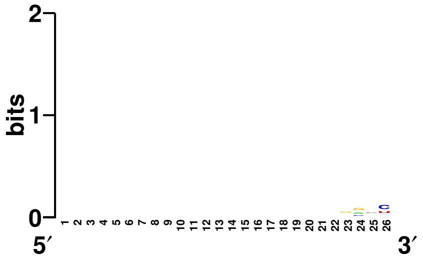

27-mers:

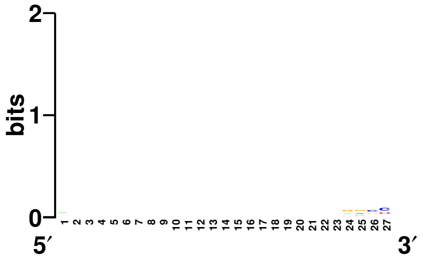

28-mers:

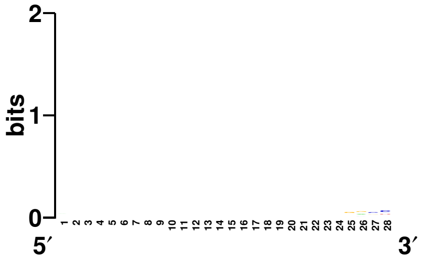

29-mers:

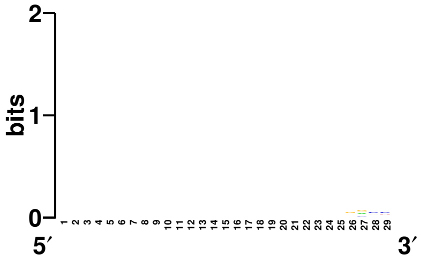

30-mers:

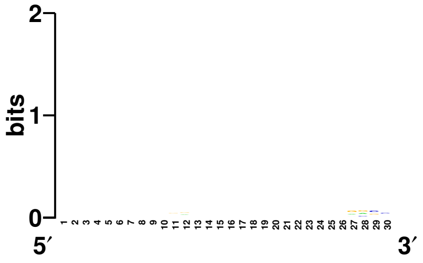

Embryo 15h, library 3:

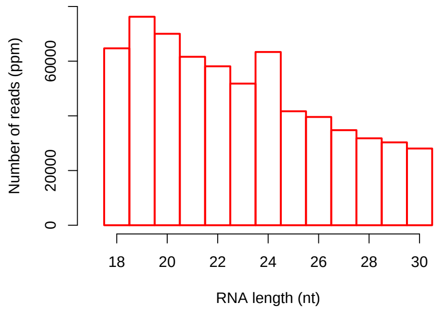

18-mers:

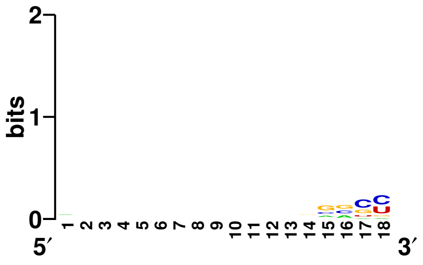

19-mers:

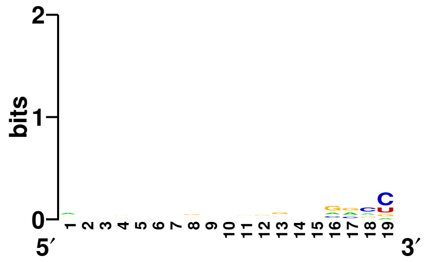

20-mers:

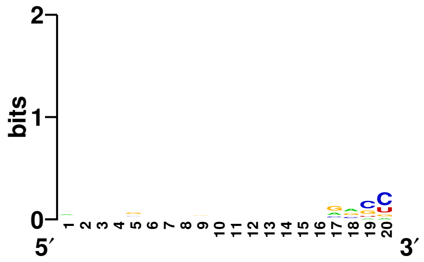

21-mers:

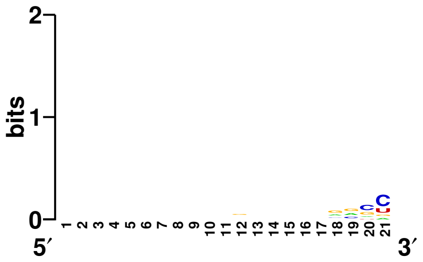

22-mers:

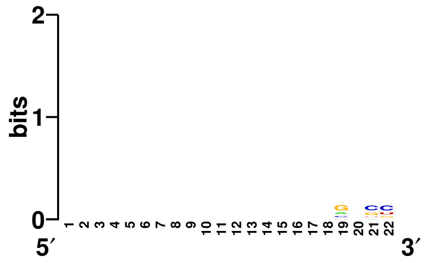

23-mers:

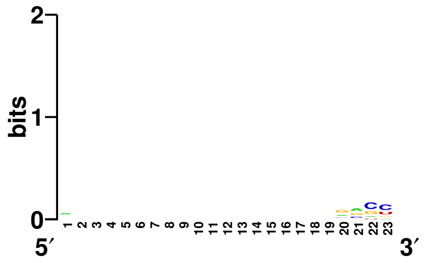

24-mers:

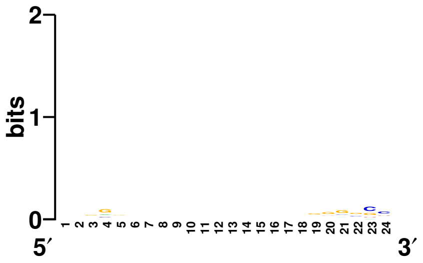

25-mers:

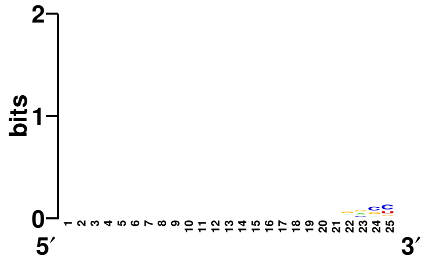

26-mers:

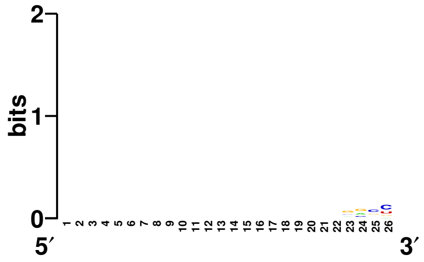

27-mers:

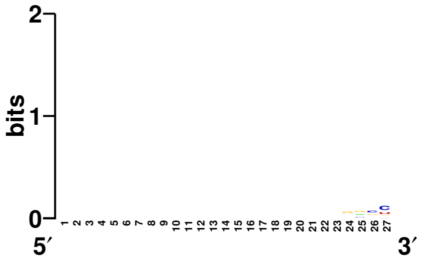

28-mers:

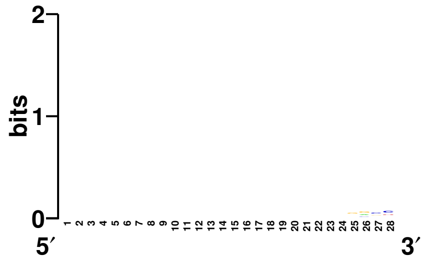

29-mers:

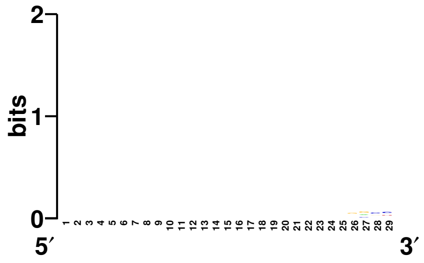

30-mers:

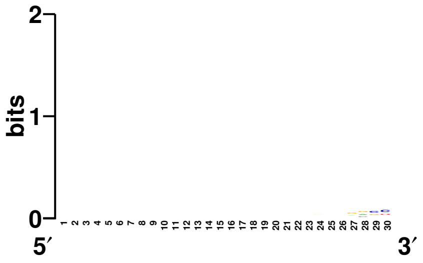

Embryo 36h, library 3:

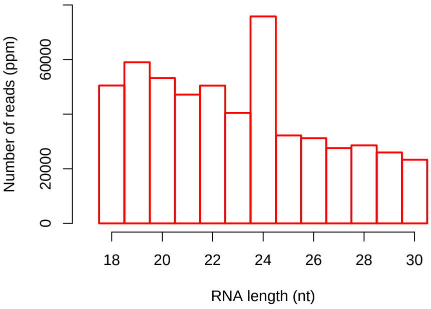

18-mers:

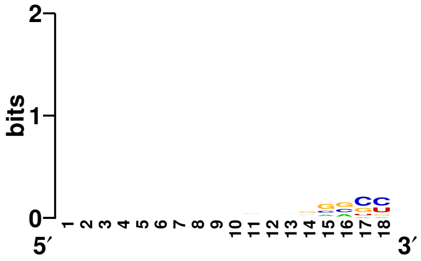

19-mers:

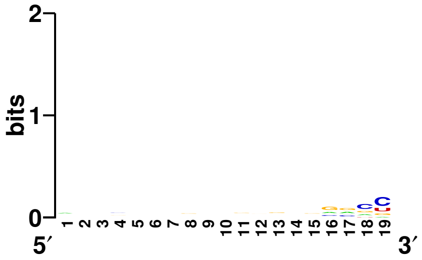

20-mers:

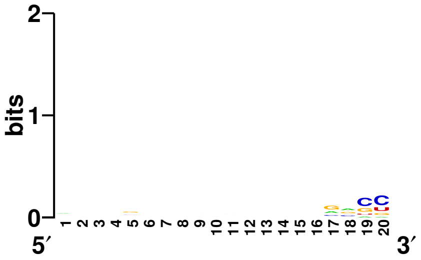

21-mers:

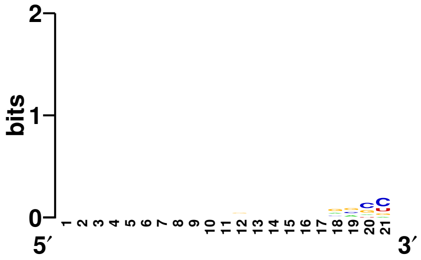

22-mers:

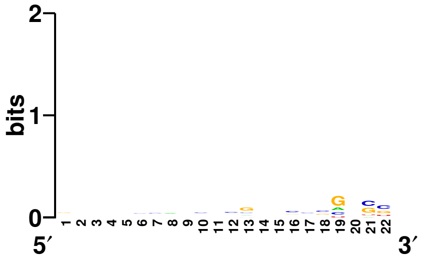

23-mers:

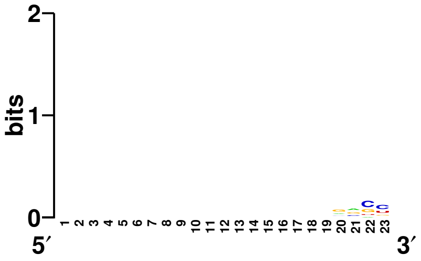

24-mers:

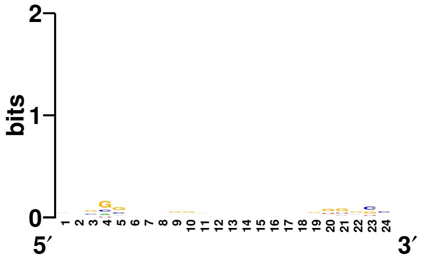

25-mers:

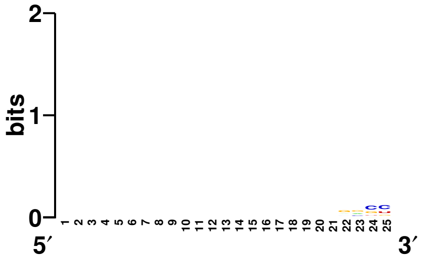

26-mers:

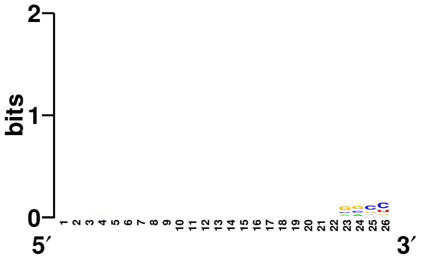

27-mers:

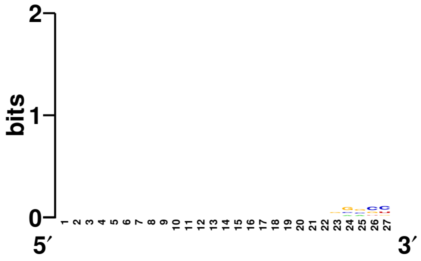

28-mers:

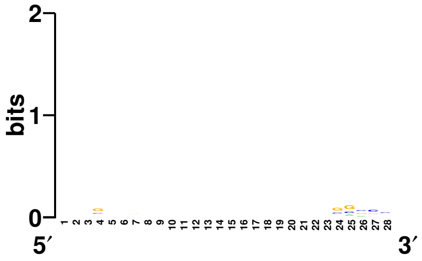

29-mers:

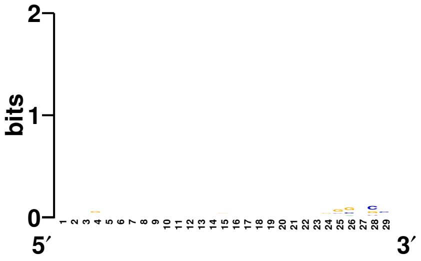

30-mers:

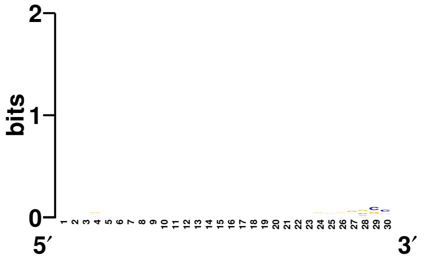

Embryo 60h, library 3:

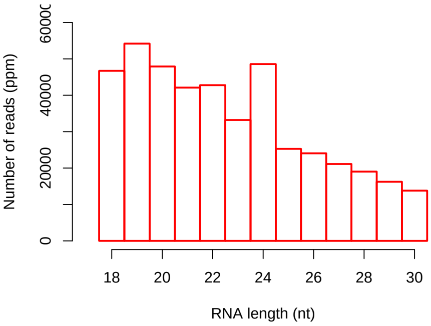

18-mers:

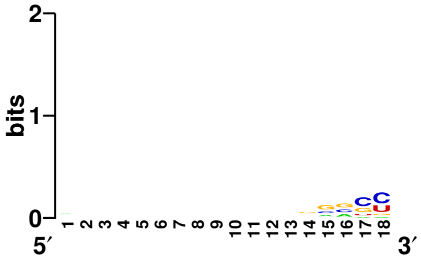

19-mers:

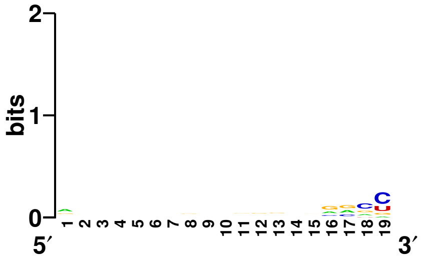

20-mers:

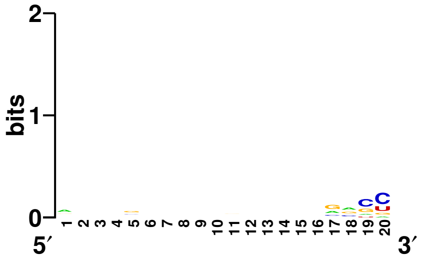

21-mers:

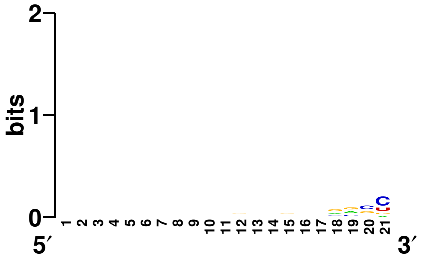

22-mers:

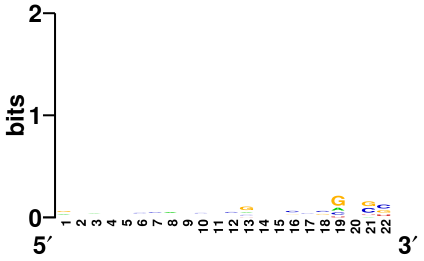

23-mers:

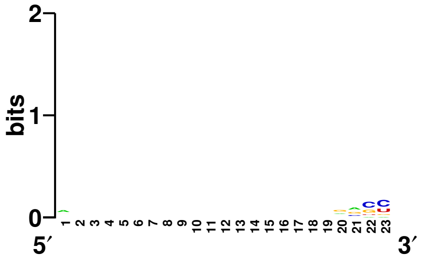

24-mers:

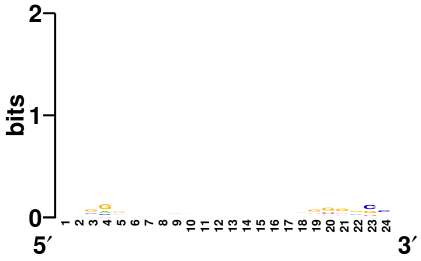

25-mers:

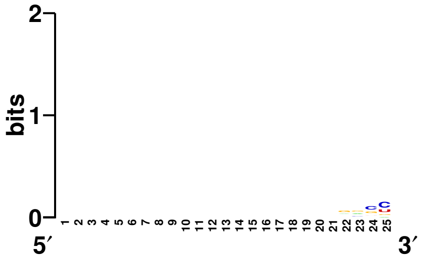

26-mers:

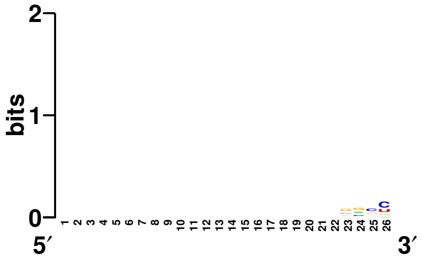

27-mers:

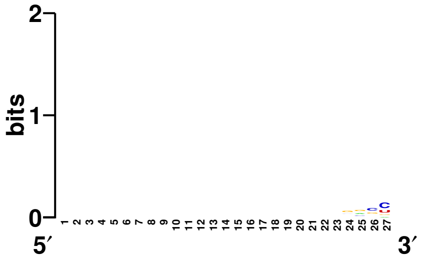

28-mers:

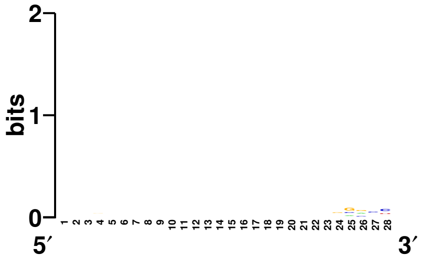

29-mers:

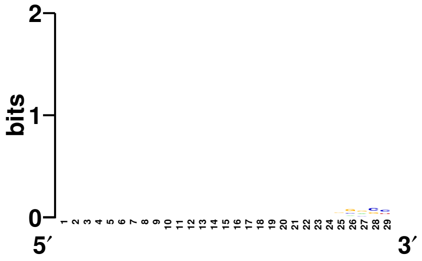

30-mers:

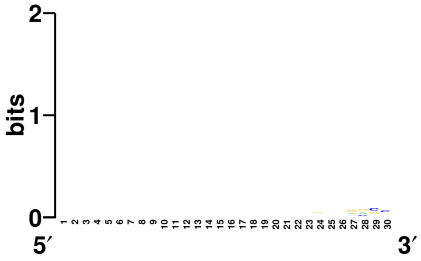

Adult female, library 3:

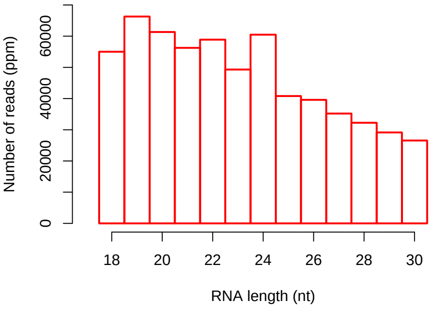

18-mers:

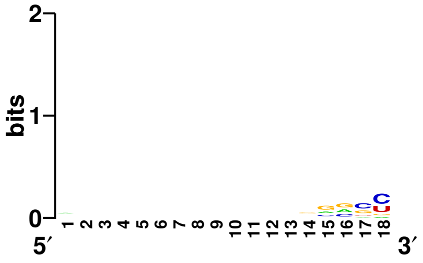

19-mers:

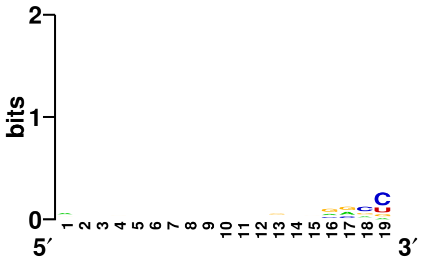

20-mers:

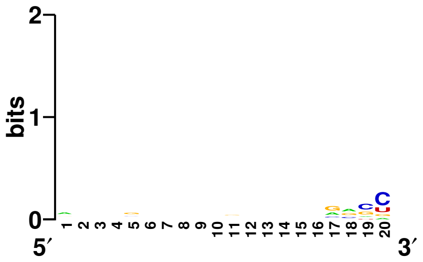

21-mers:

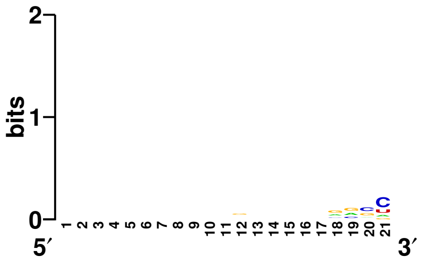

22-mers:

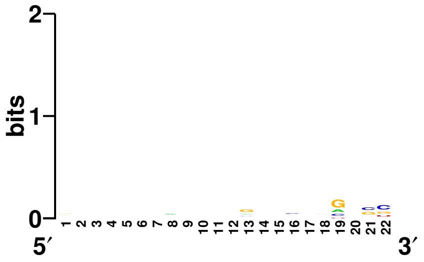

23-mers:

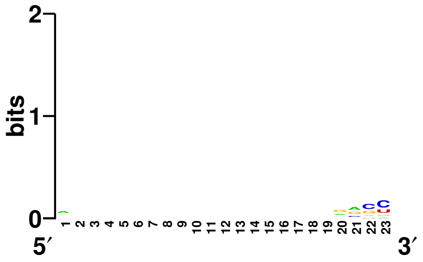

24-mers:

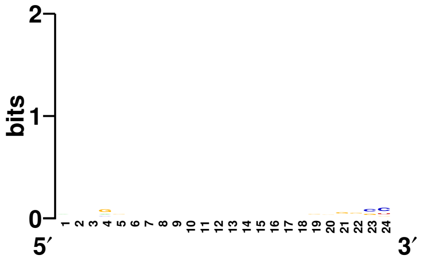

25-mers:

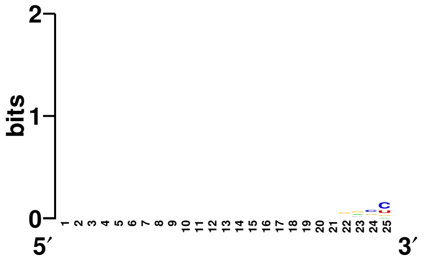

26-mers:

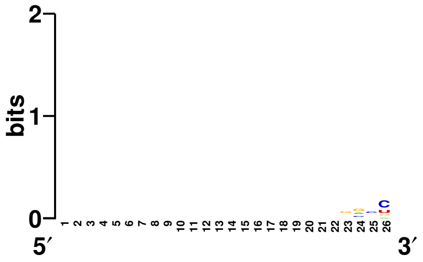

27-mers:

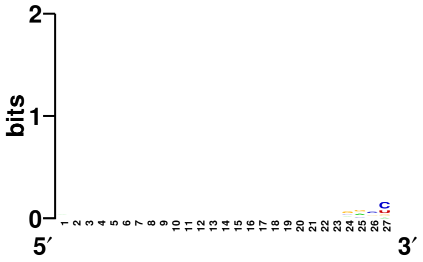

28-mers:

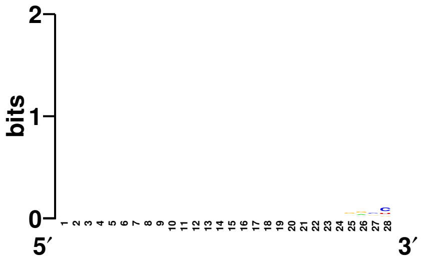

29-mers:

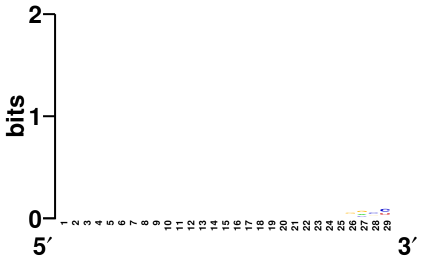

30-mers:

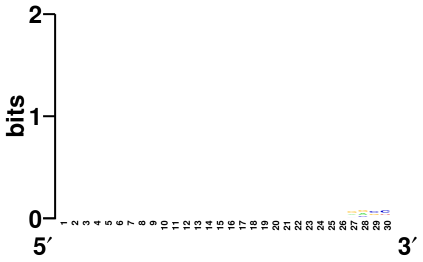

Adult male, library 3:

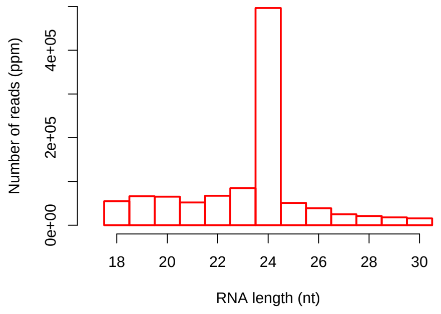

18-mers:

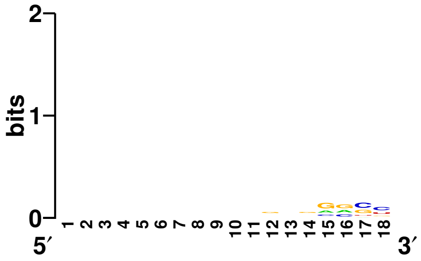

19-mers:

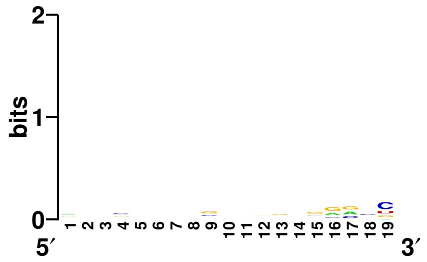

20-mers:

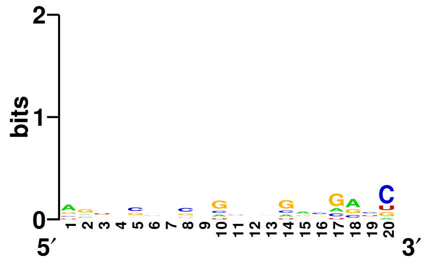

21-mers:

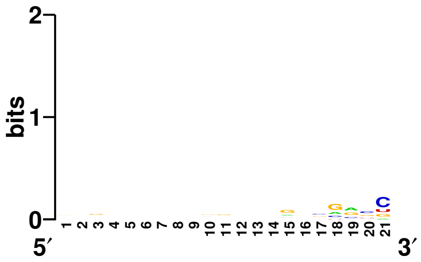

22-mers:

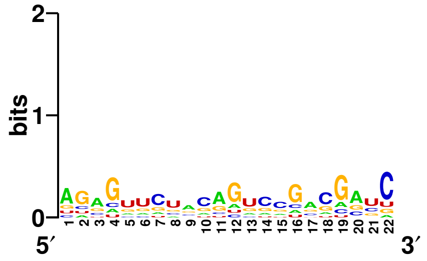

23-mers:

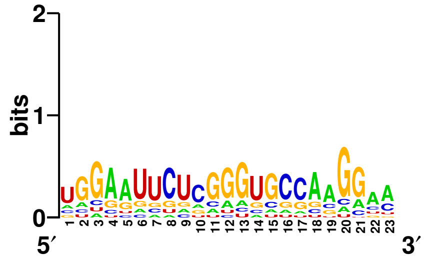

24-mers:

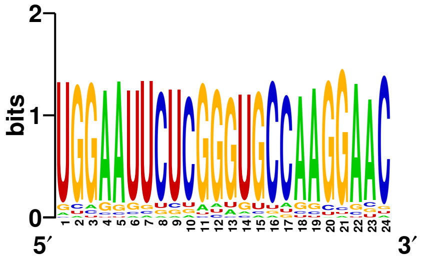

25-mers:

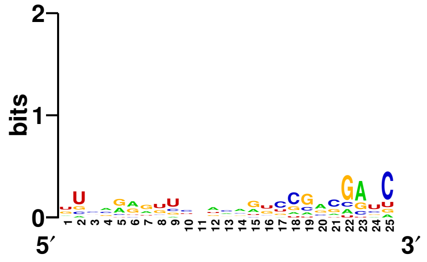

26-mers:

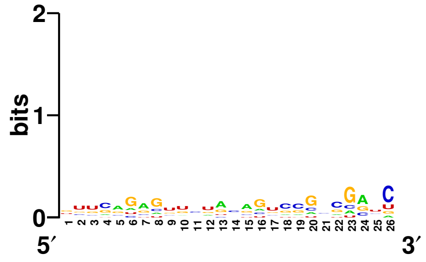

27-mers:

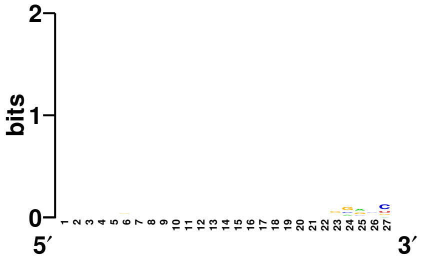

28-mers:

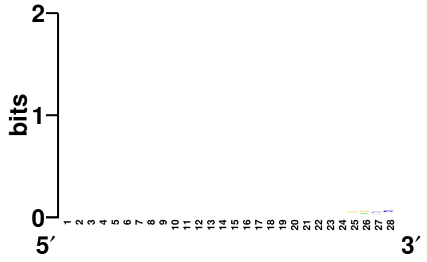

29-mers:

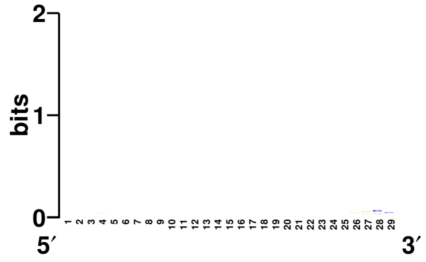

30-mers:

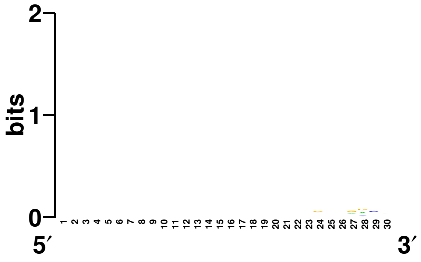

5.4 Libraries #4 (3´ modified, 5´ hydroxyl or polyphosphorylated small RNAs)

Embryo 8h, library 4:

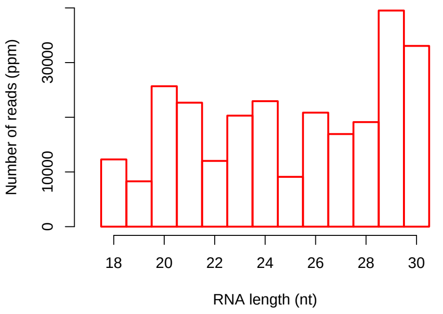

18-mers:

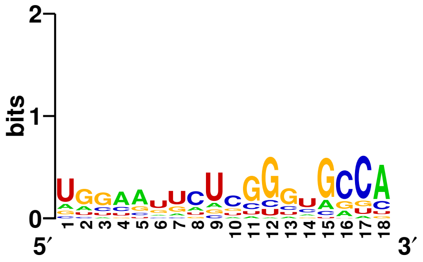

19-mers:

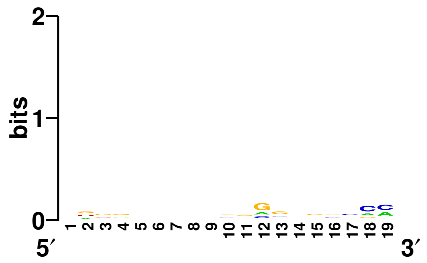

20-mers:

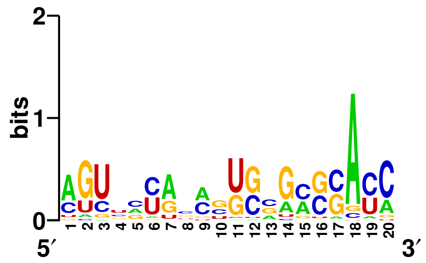

21-mers:

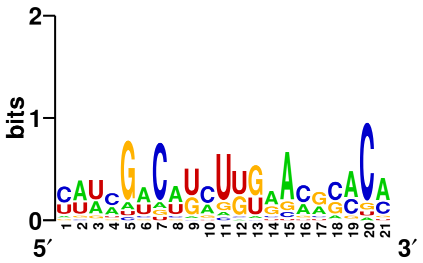

22-mers:

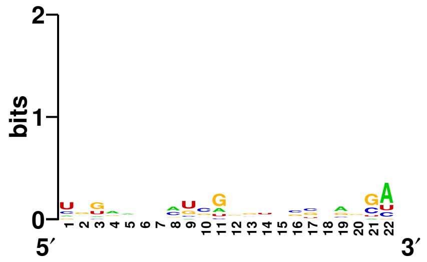

23-mers:

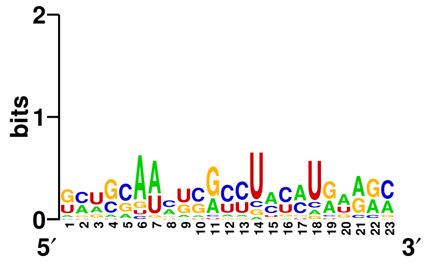

24-mers:

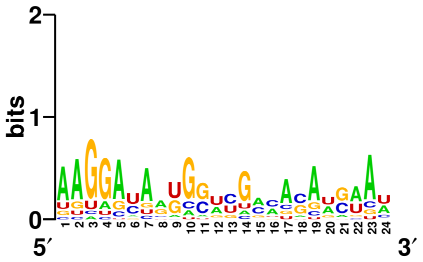

25-mers:

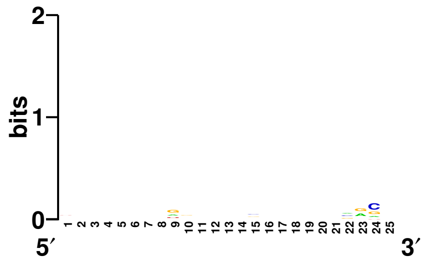

26-mers:

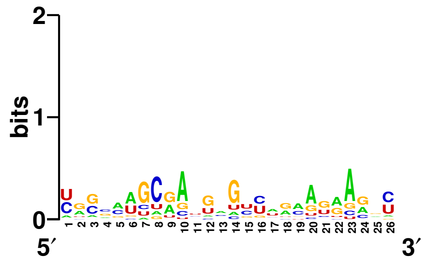

27-mers:

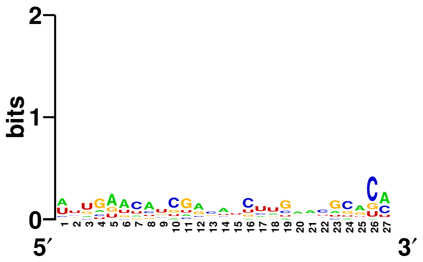

28-mers:

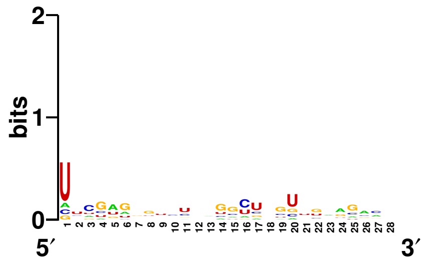

29-mers:

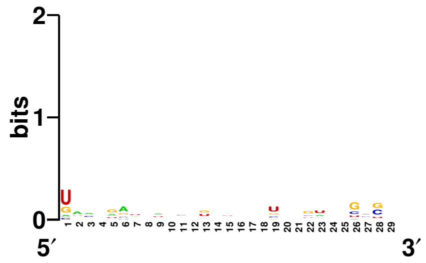

30-mers:

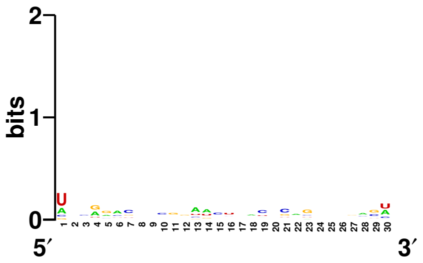

Embryo 15h, library 4:

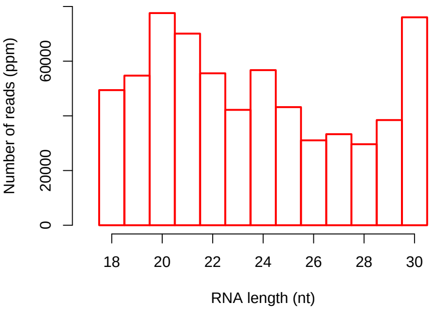

18-mers:

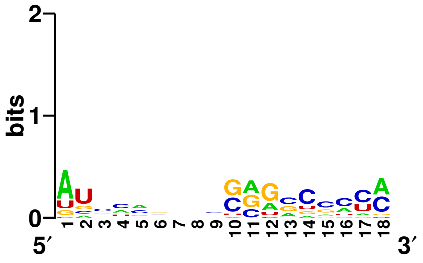

19-mers:

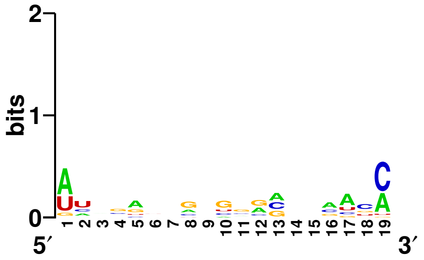

20-mers:

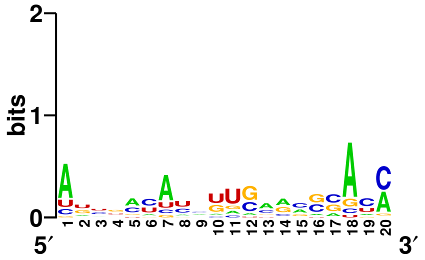

21-mers:

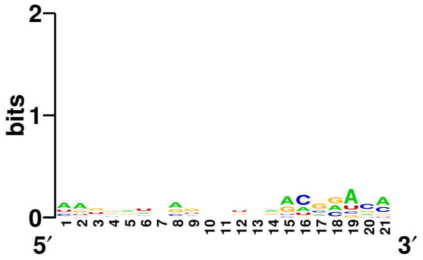

22-mers:

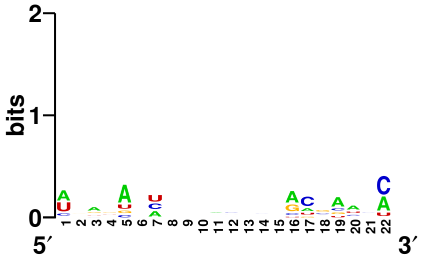

23-mers:

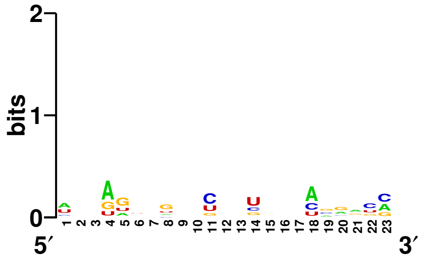

24-mers:

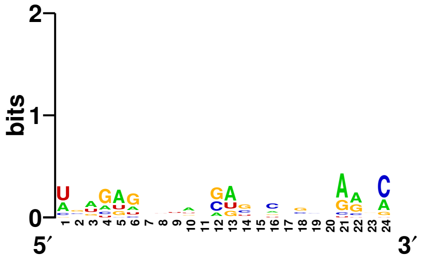

25-mers:

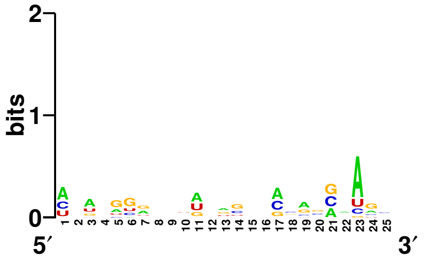

26-mers:

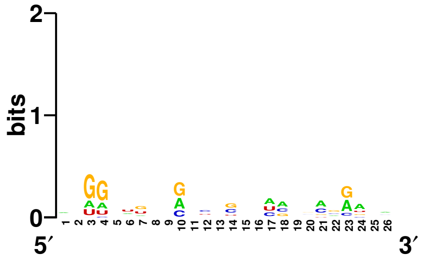

27-mers:

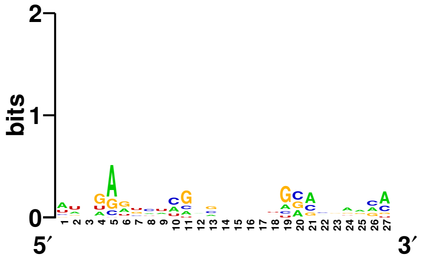

28-mers:

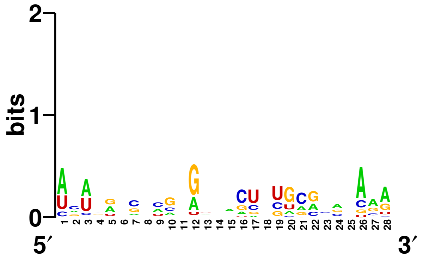

29-mers:

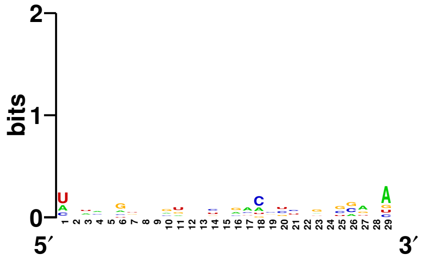

30-mers:

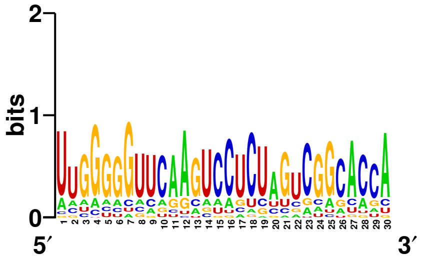

Embryo 36h, library 4:

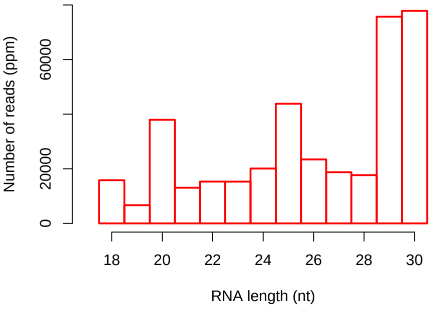

18-mers:

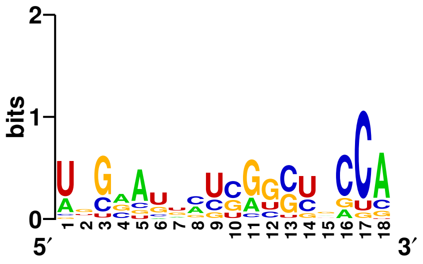

19-mers:

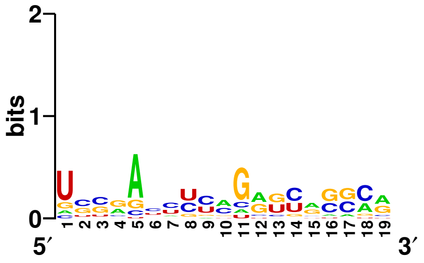

20-mers:

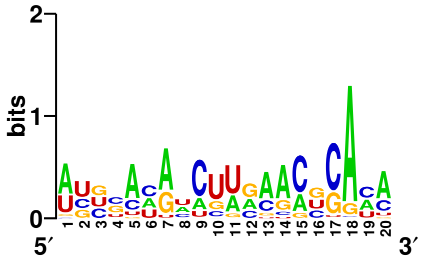

21-mers:

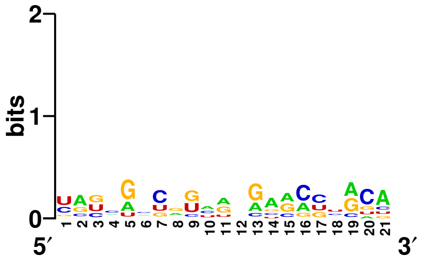

22-mers:

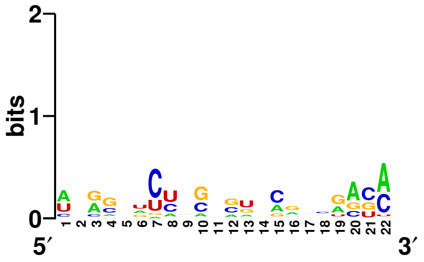

23-mers:

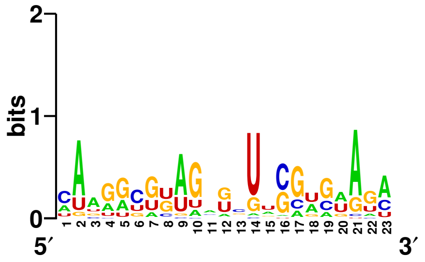

24-mers:

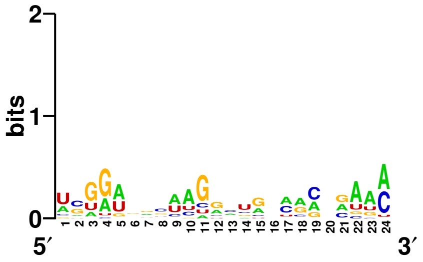

25-mers:

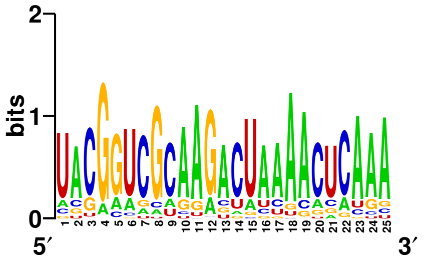

26-mers:

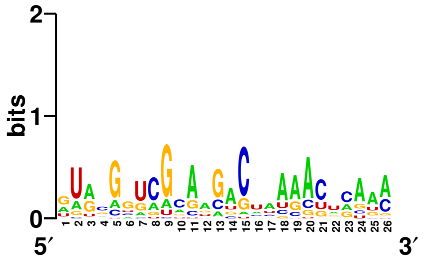

27-mers:

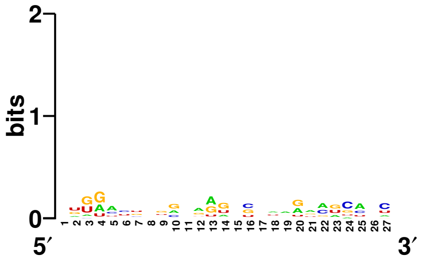

28-mers:

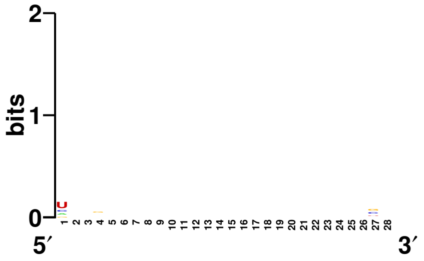

29-mers:

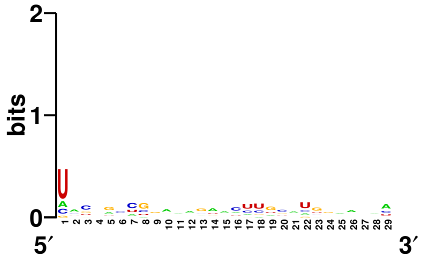

30-mers:

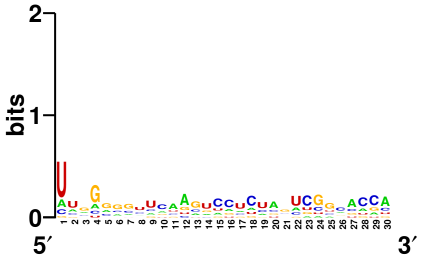

Embryo 60h, library 4:

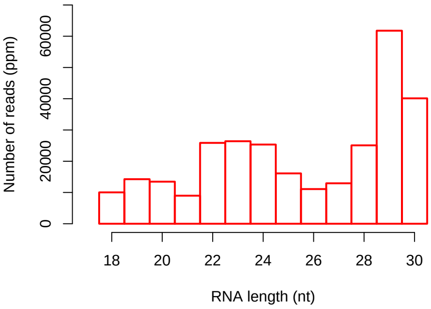

18-mers:

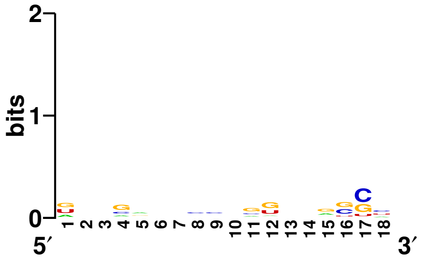

19-mers:

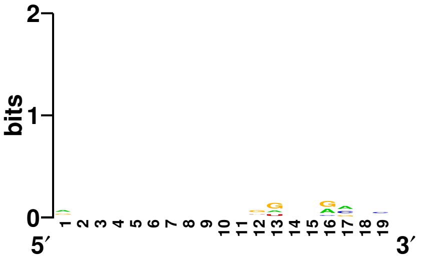

20-mers:

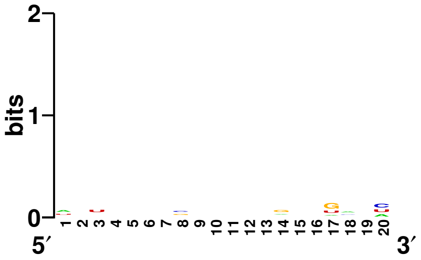

21-mers:

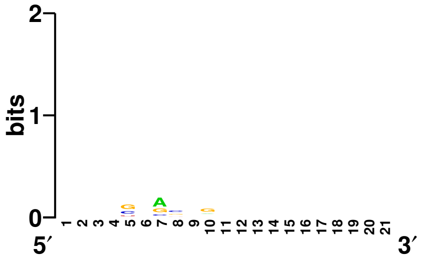

22-mers:

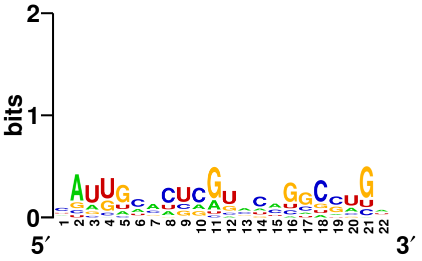

23-mers:

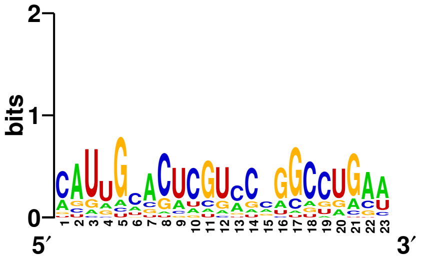

24-mers:

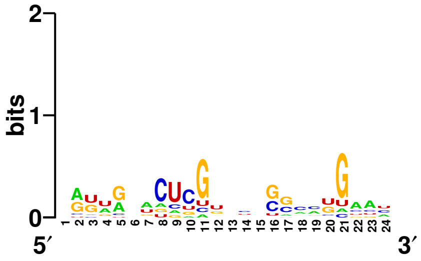

25-mers:

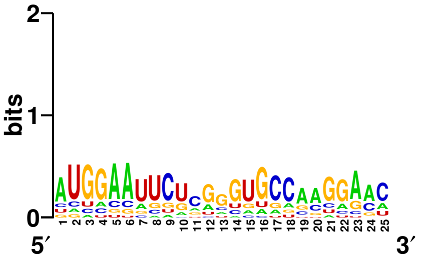

26-mers:

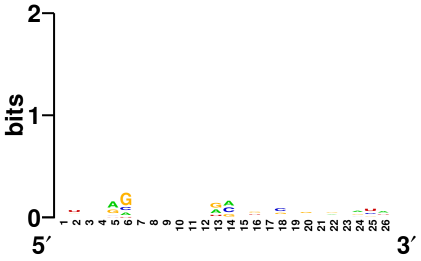

27-mers:

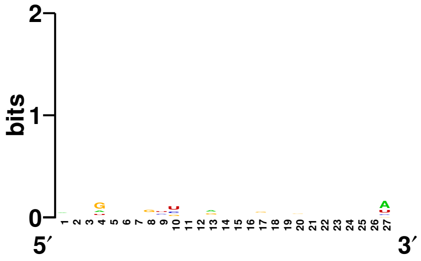

28-mers:

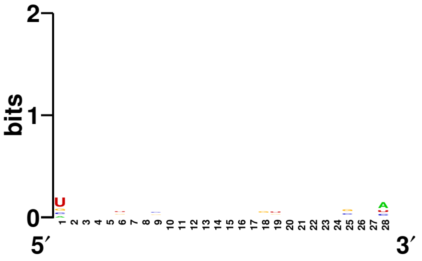

29-mers:

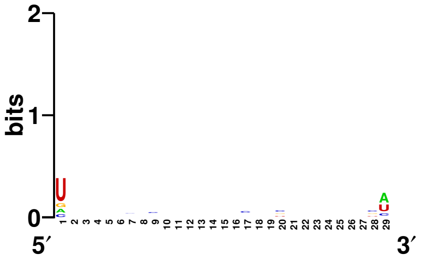

30-mers:

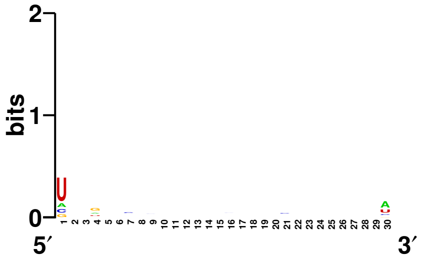

Adult female, library 4:

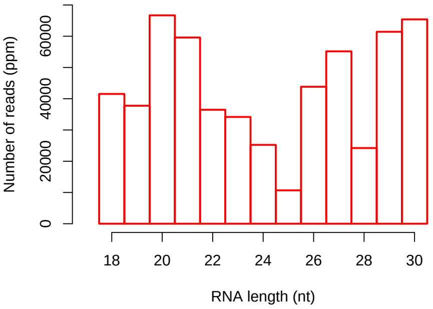

18-mers:

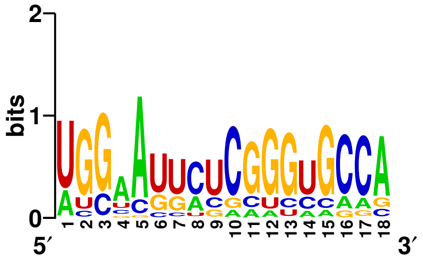

19-mers:

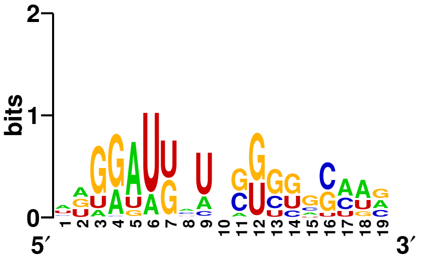

20-mers:

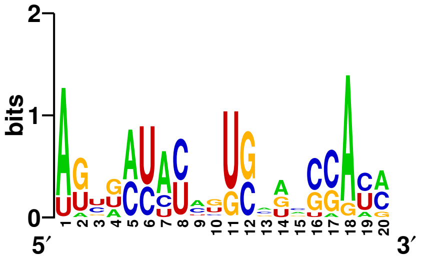

21-mers:

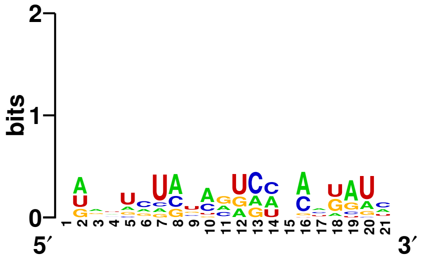

22-mers:

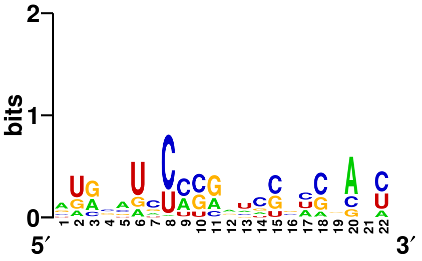

23-mers:

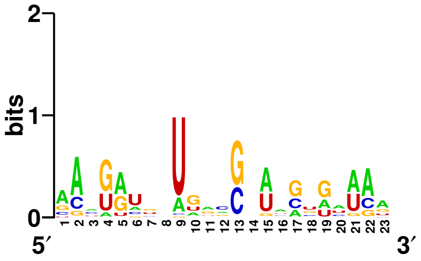

24-mers:

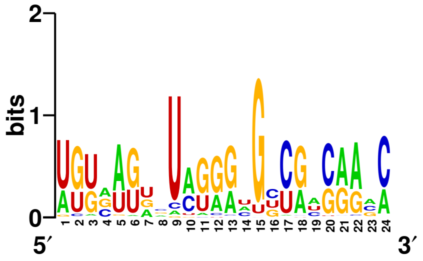

25-mers:

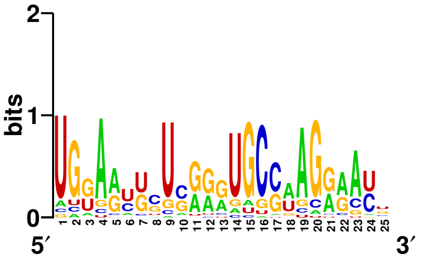

26-mers:

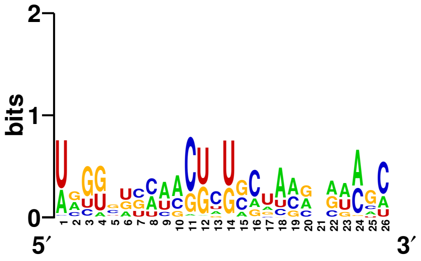

27-mers:

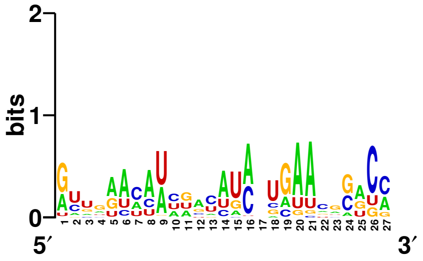

28-mers:

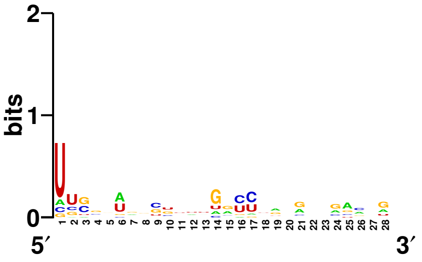

29-mers:

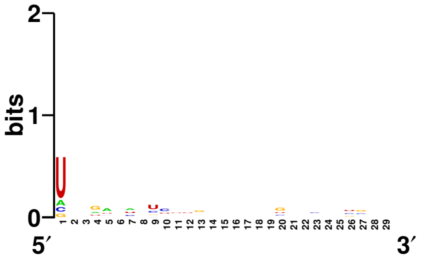

30-mers:

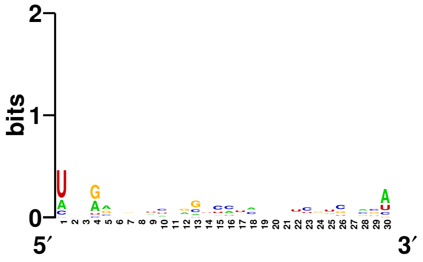

Adult male, library 4:

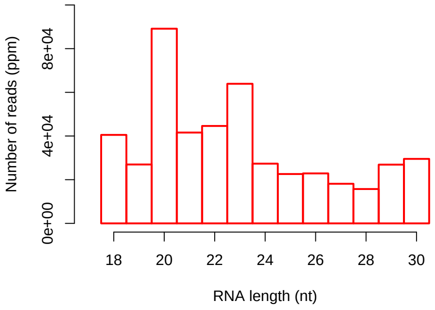

18-mers:

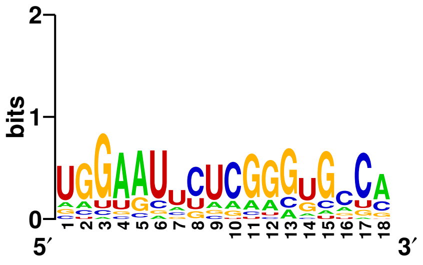

19-mers:

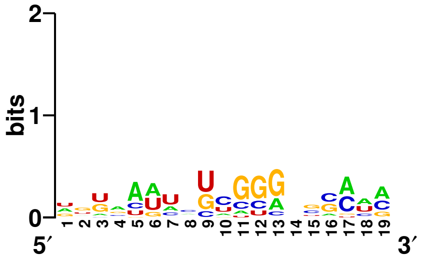

20-mers:

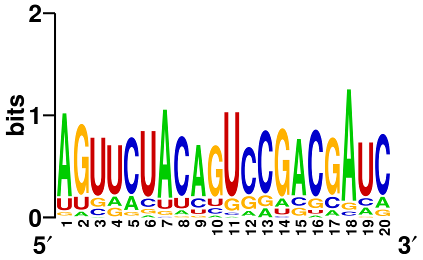

21-mers:

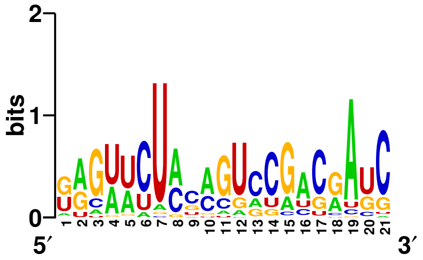

22-mers:

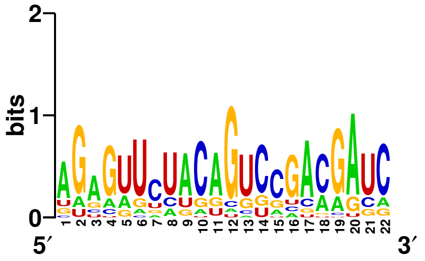

23-mers:

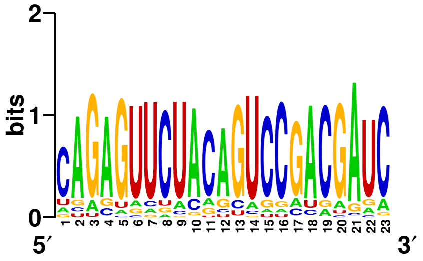

24-mers:

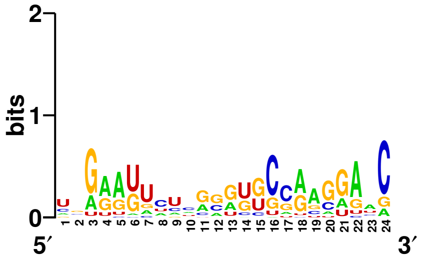

25-mers:

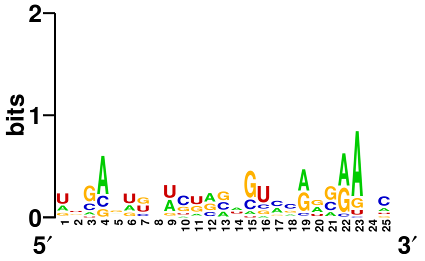

26-mers:

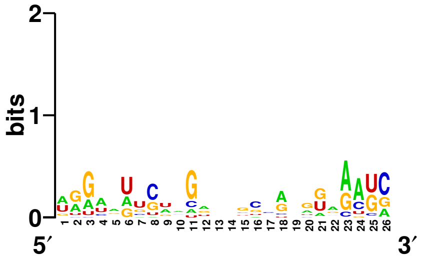

27-mers:

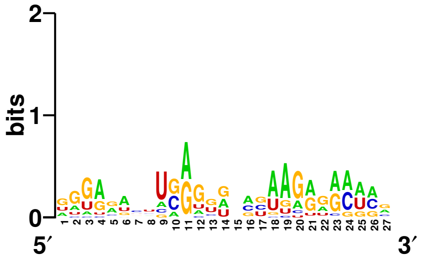

28-mers:

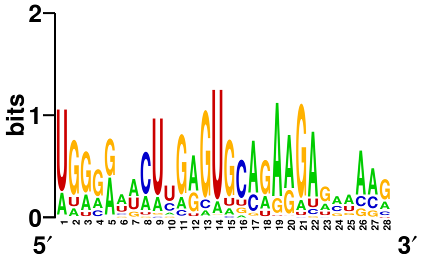

29-mers:

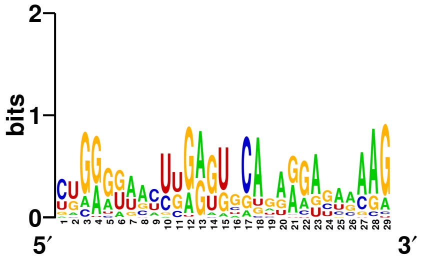

30-mers:

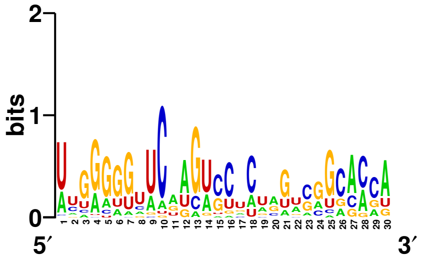

6 Analysis of *Cænorhabditis elegans* data [Gu et al., 2009] with the same method

6.1 Genomic reads not matching abundant ncRNAs

Library GSM455391 (18-26-mers, any number of 5′ phosphates, replicate 1):

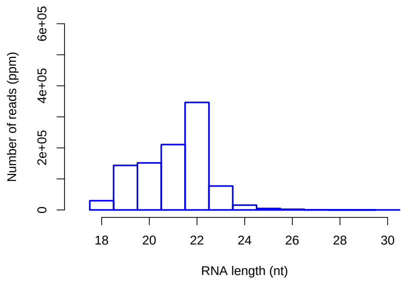

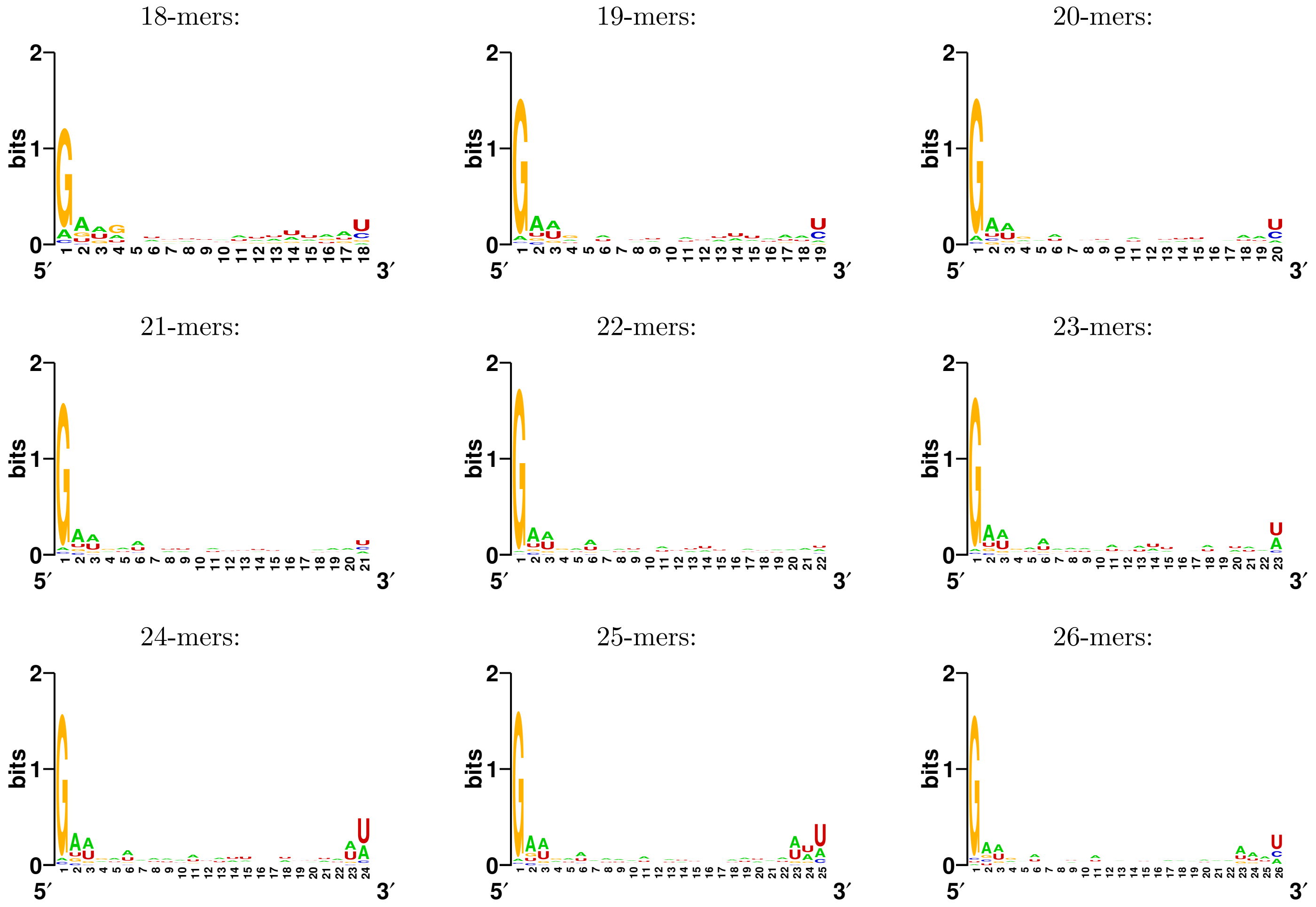

Library GSM455392 (18-26-mers, any number of 5´ phosphates, replicate 2):

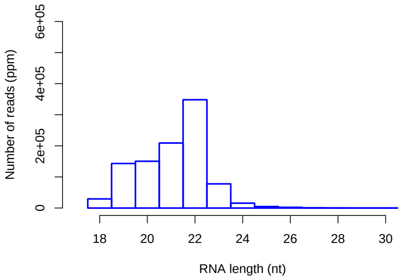

18-mers:

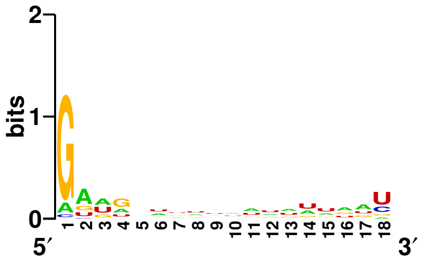

19-mers:

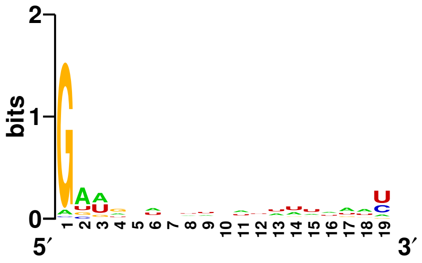

20-mers:

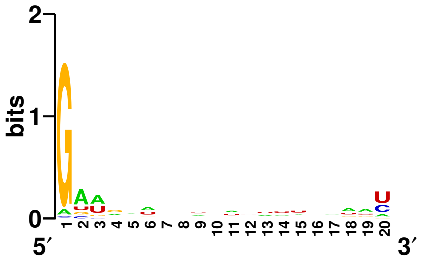

21-mers:

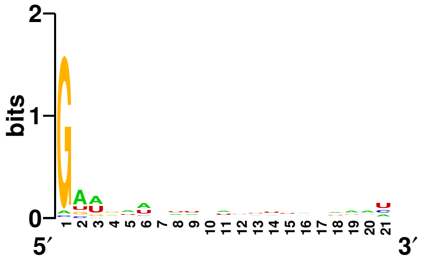

22-mers:

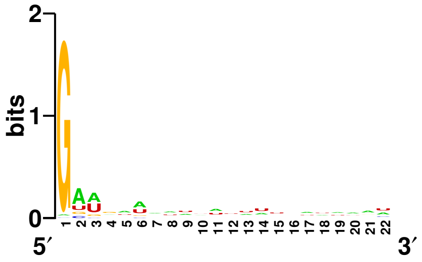

23-mers:

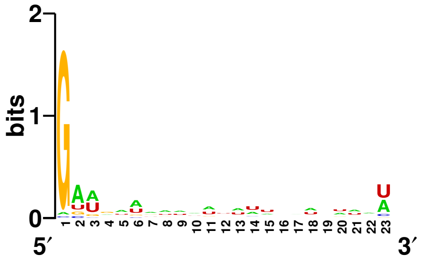

24-mers:

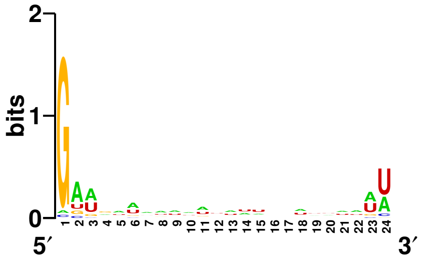

25-mers:

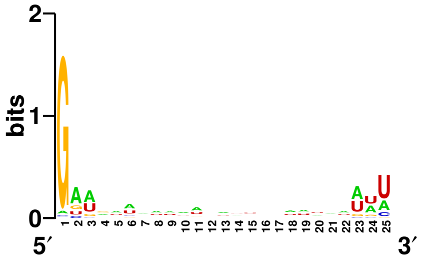

26-mers:

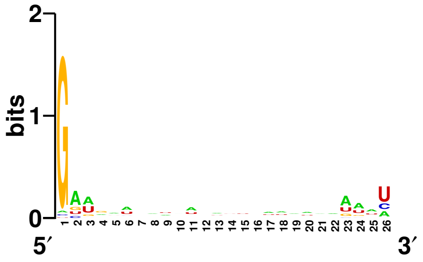

Library GSM455393 (18-26-mers, any number of 5´ phosphates, replicate 3):

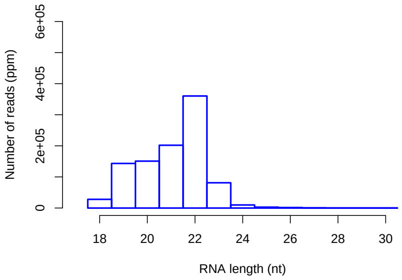

18-mers:

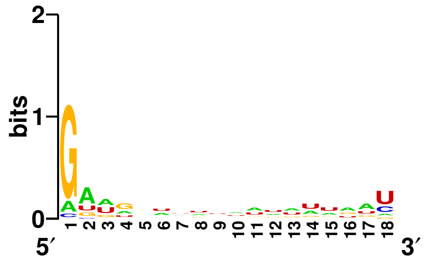

19-mers:

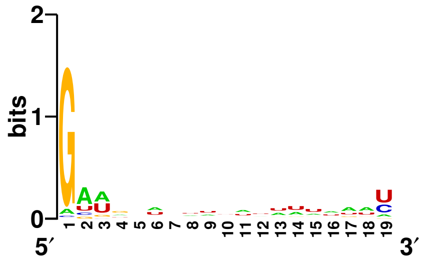

20-mers:

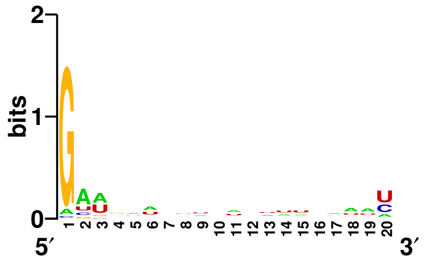

21-mers:

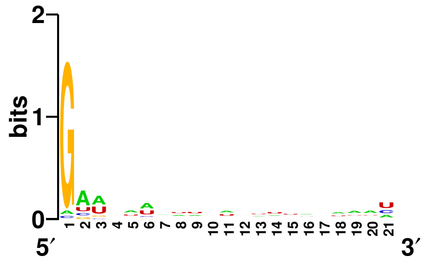

22-mers:

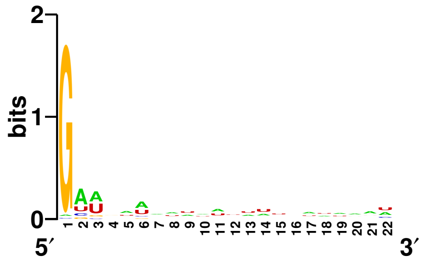

23-mers:

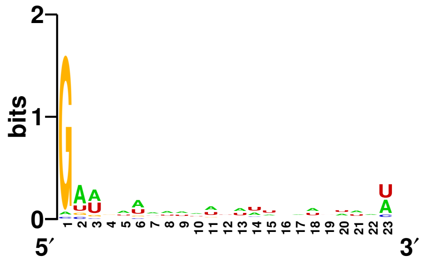

24-mers:

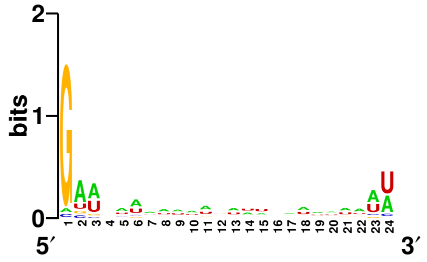

25-mers:

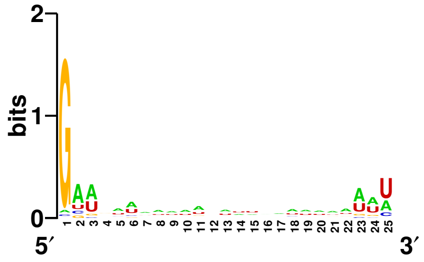

26-mers:

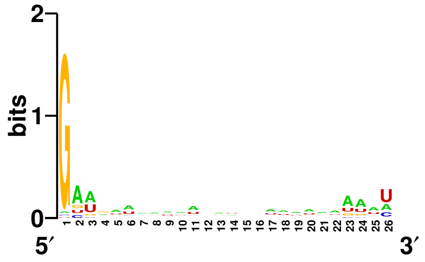

6.2 pre-miRNA hairpin-matching reads

Library GSM455391 (18-26-mers, any number of 5´ phosphates, replicate 1):

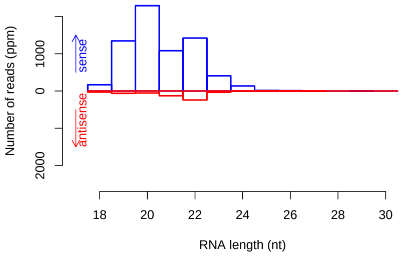

Sense reads:

18-mers:

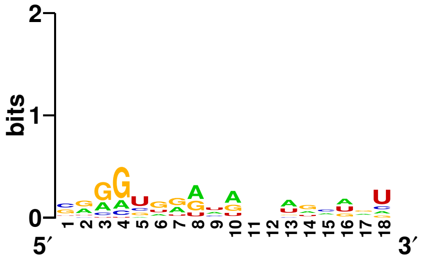

19-mers:

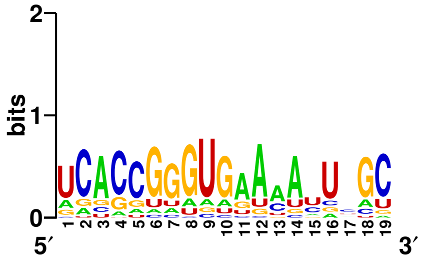

20-mers:

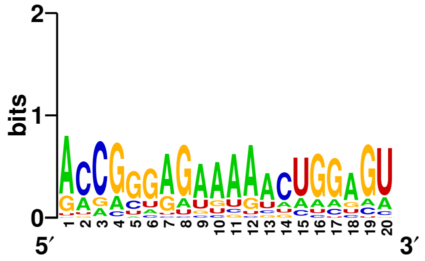

21-mers:

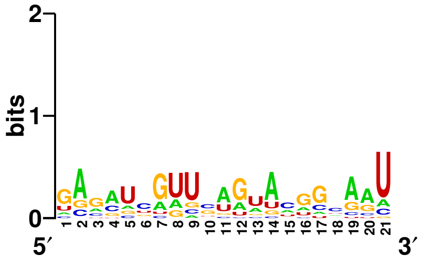

22-mers:

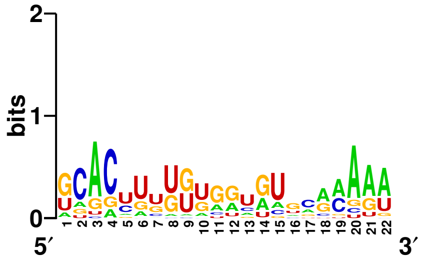

23-mers:

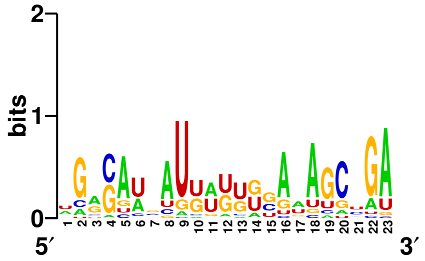

24-mers:

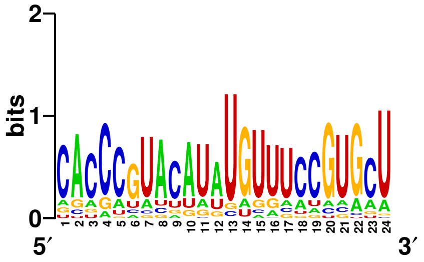

25-mers:

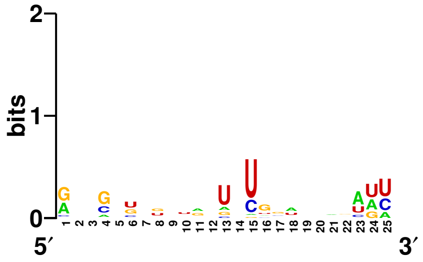

26-mers:

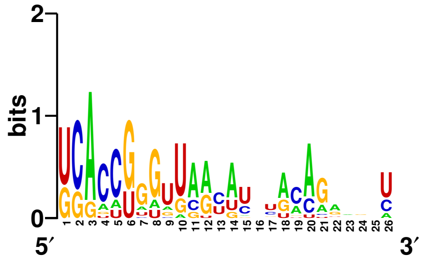

Antisense reads:

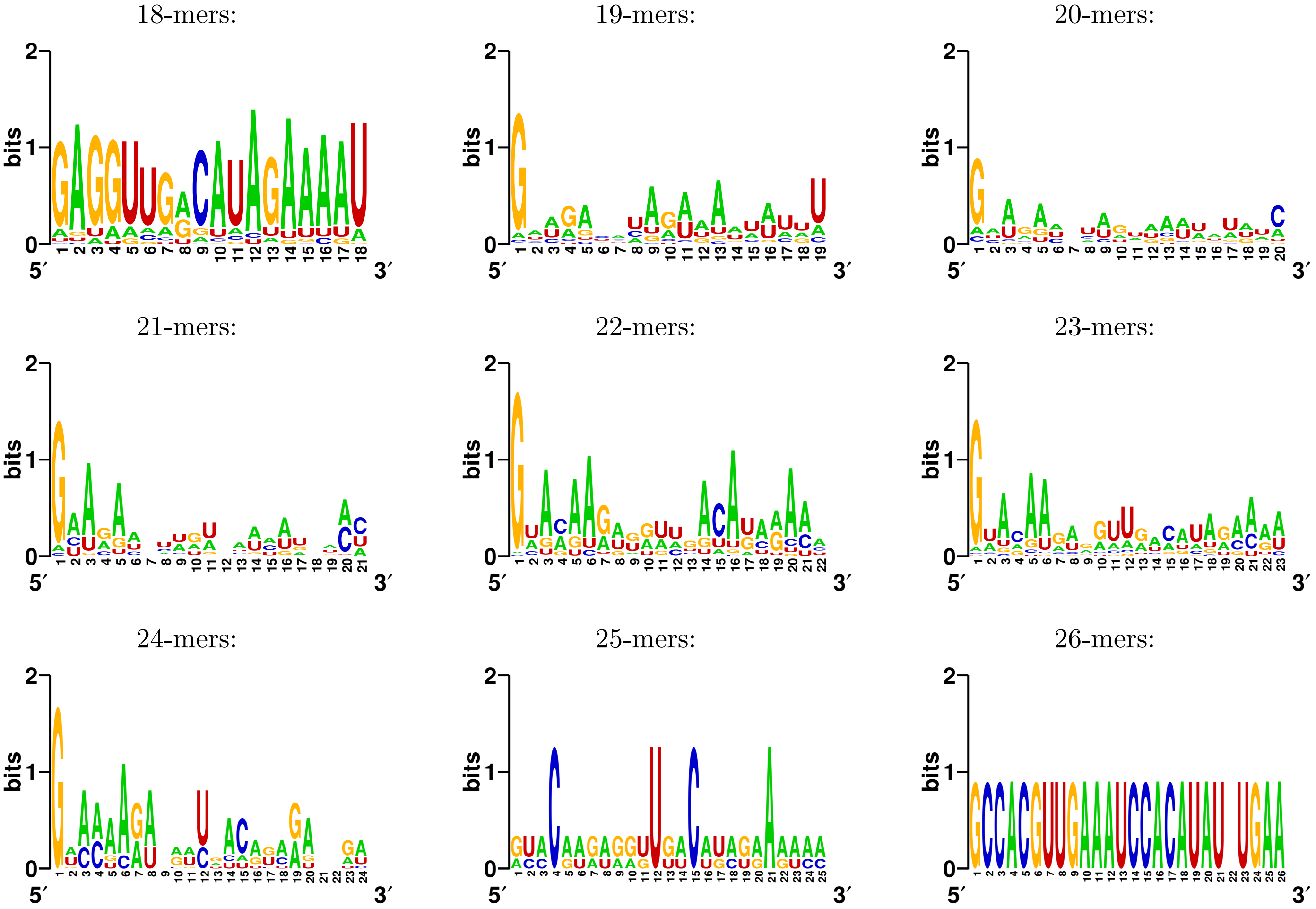

Library GSM455392 (18-26-mers, any number of 5´ phosphates, replicate 2):

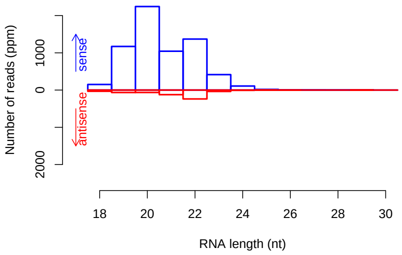

Sense reads:

18-mers:

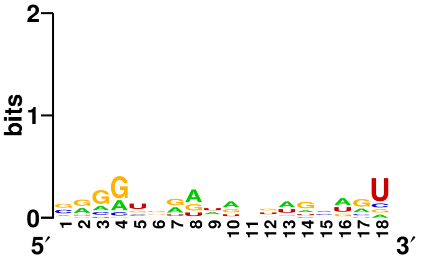

19-mers:

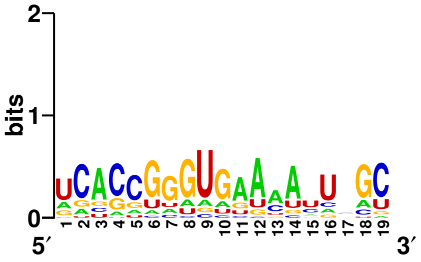

20-mers:

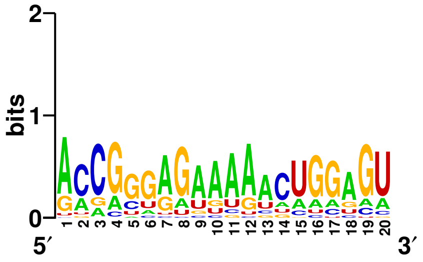

21-mers:

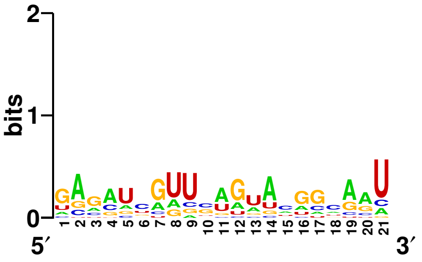

22-mers:

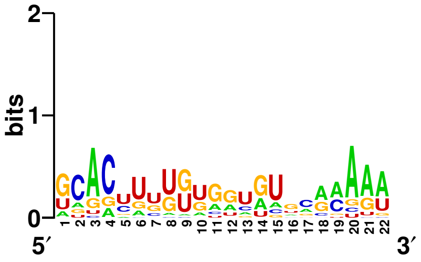

23-mers:

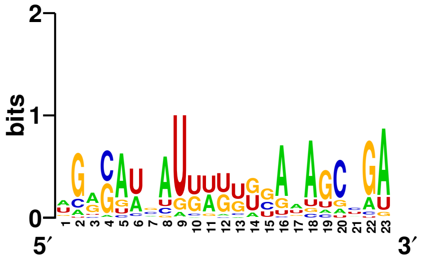

24-mers:

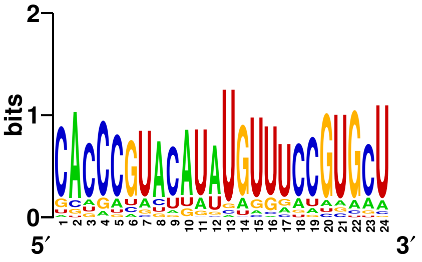

25-mers:

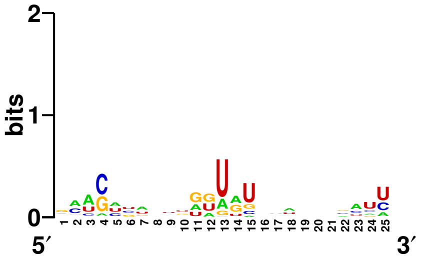

26-mers:

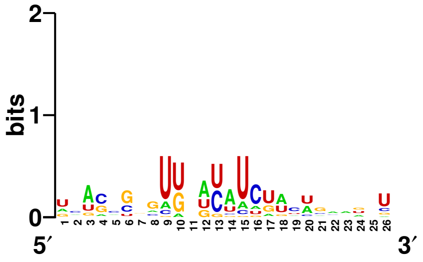

Antisense reads:

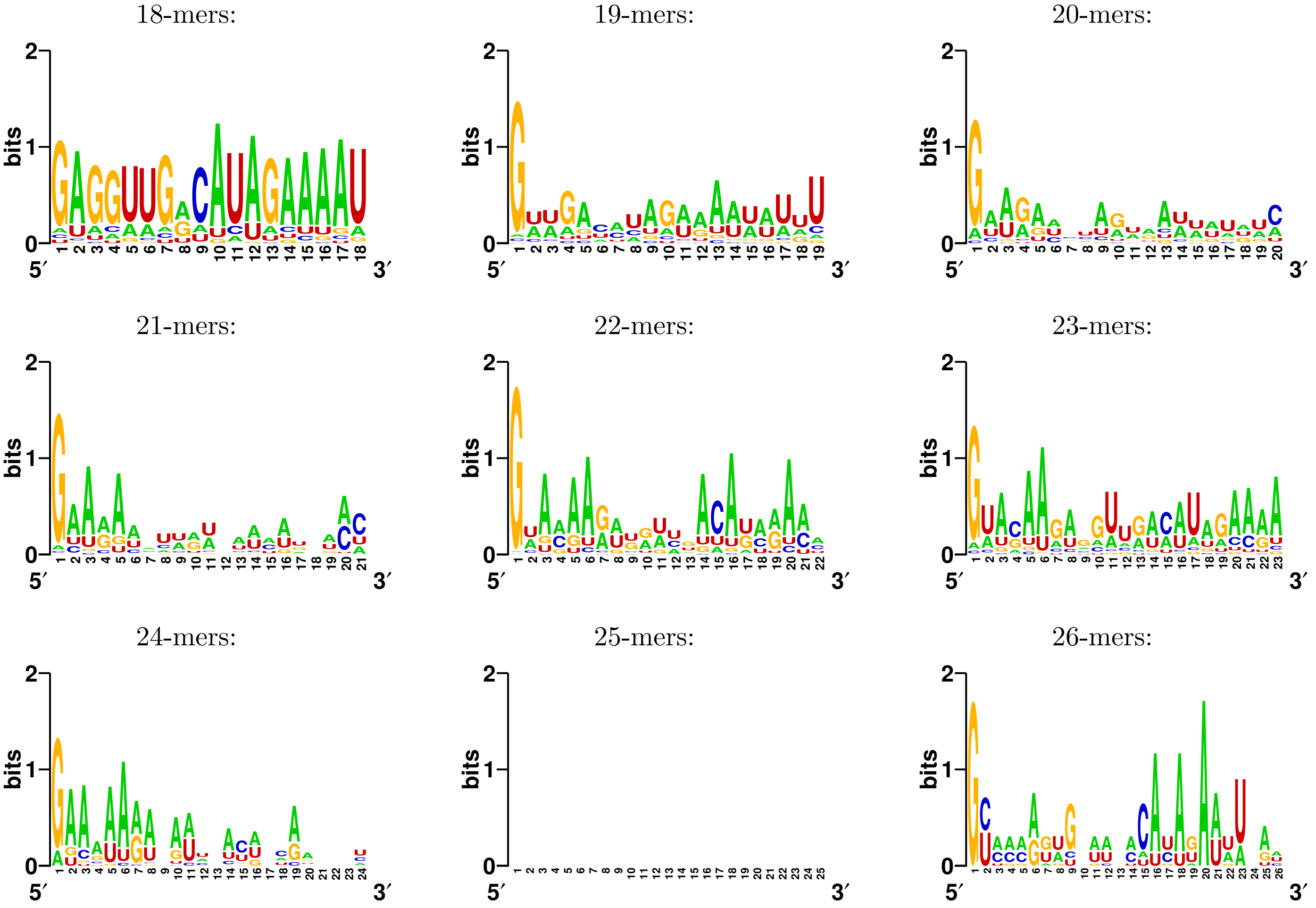

Library GSM455393 (18-26-mers, any number of 5´ phosphates, replicate 3):

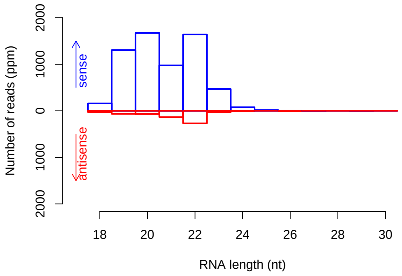

Sense reads:

18-mers:

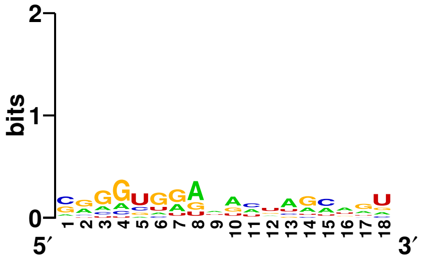

19-mers:

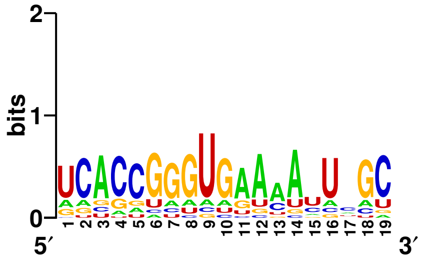

20-mers:

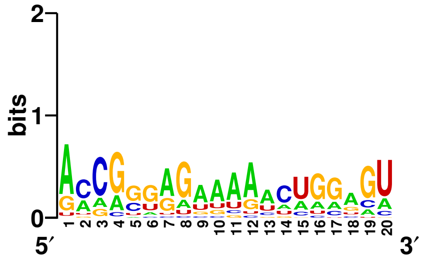

21-mers:

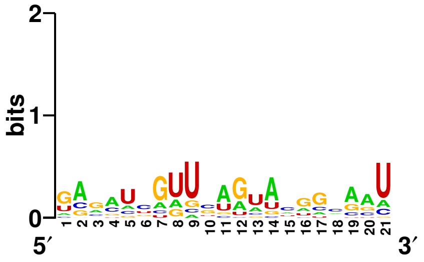

22-mers:

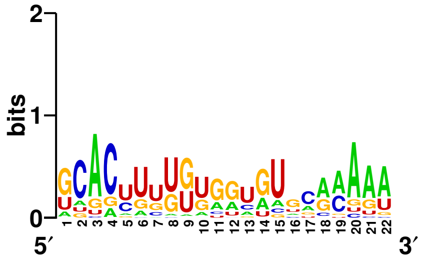

23-mers:

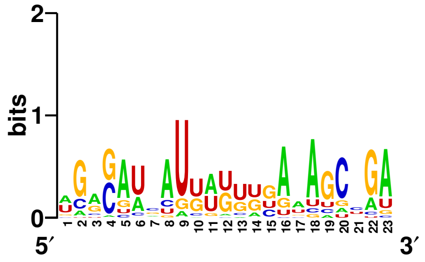

24-mers:

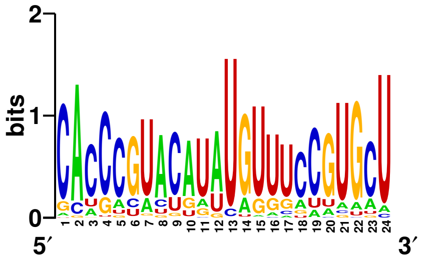

25-mers:

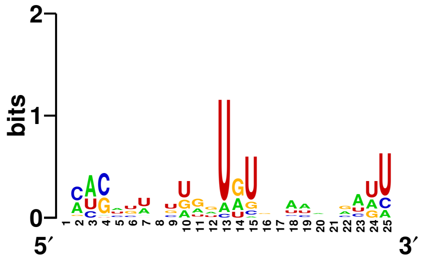

26-mers:

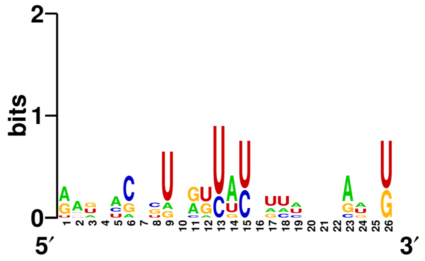

Antisense reads:

18-mers:

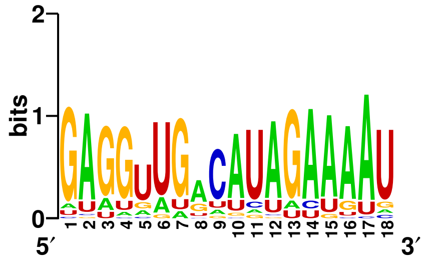

19-mers:

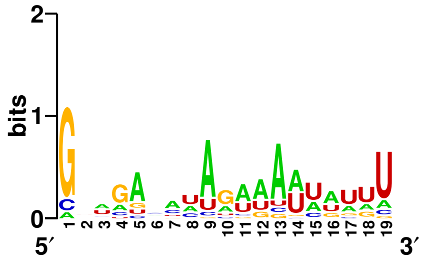

20-mers:

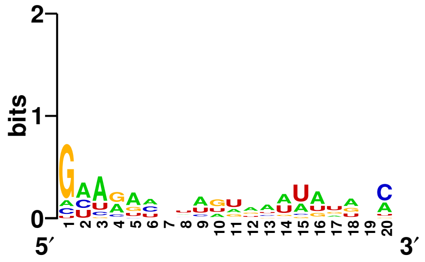

21-mers:

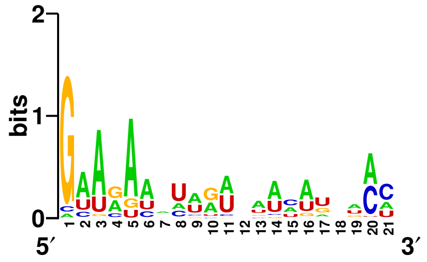

22-mers:

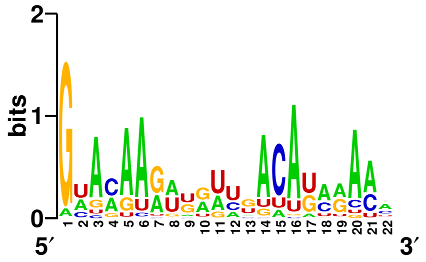

23-mers:

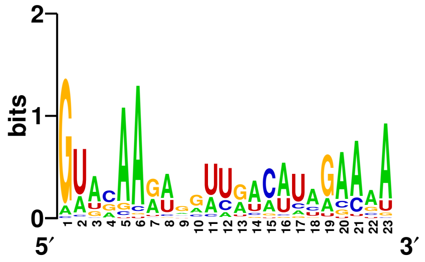

24-mers:

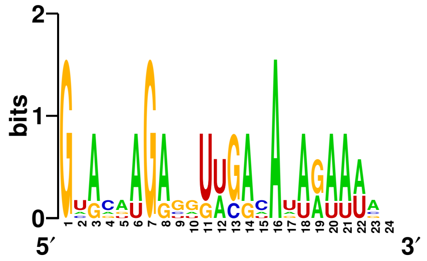

25-mers:

(no read)

26-mers:

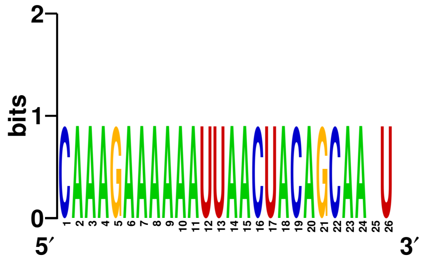

6.3 Transcriptome-matching reads (excuding pre-miRNA and abundant ncRNA-matching reads)

Library GSM455391 (18-26-mers, any number of 5´ phosphates, replicate 1):

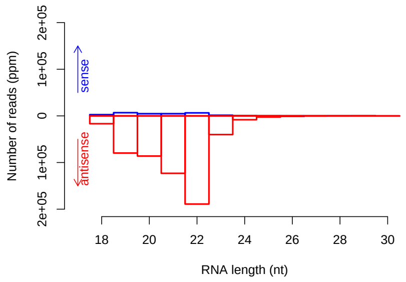

Sense reads:

18-mers:

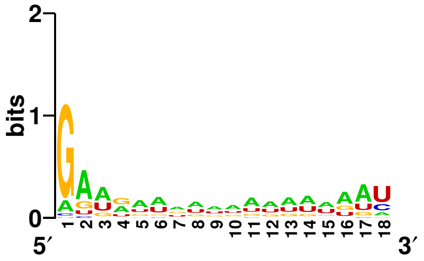

19-mers:

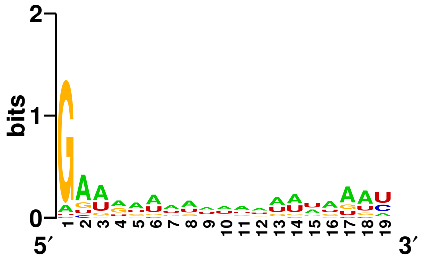

20-mers:

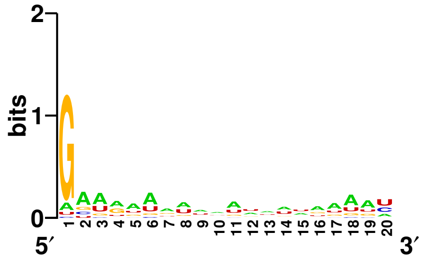

21-mers:

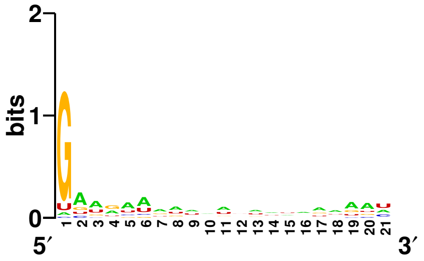

22-mers:

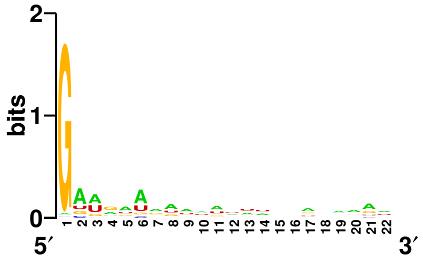

23-mers:

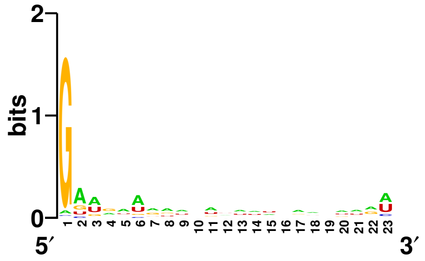

24-mers:

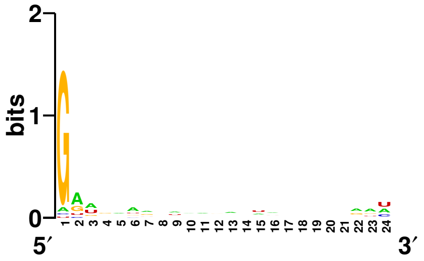

25-mers:

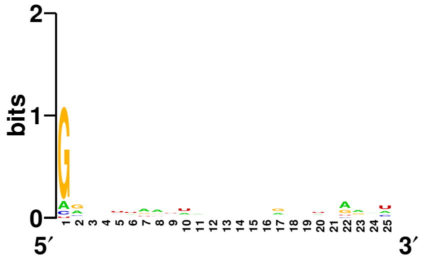

26-mers:

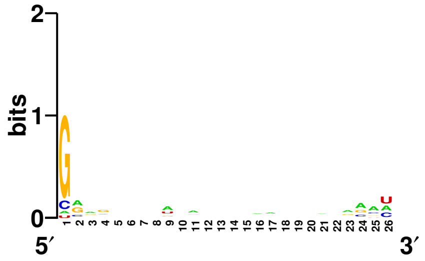

Antisense reads:

18-mers:

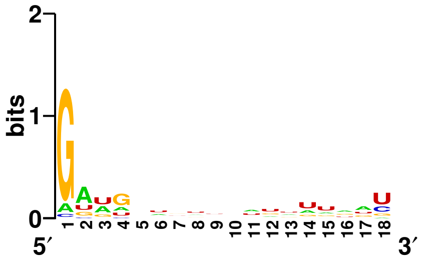

19-mers:

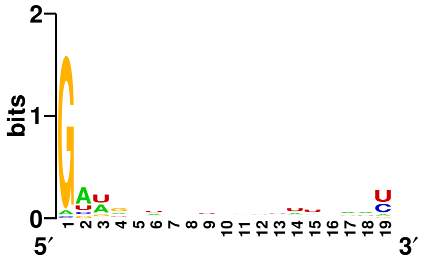

20-mers:

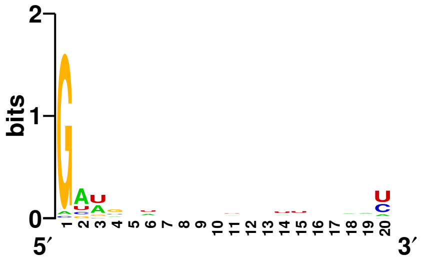

21-mers:

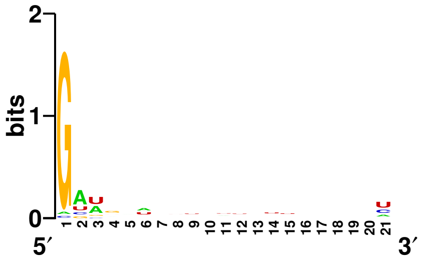

22-mers:

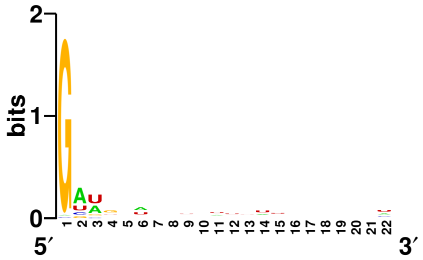

23-mers:

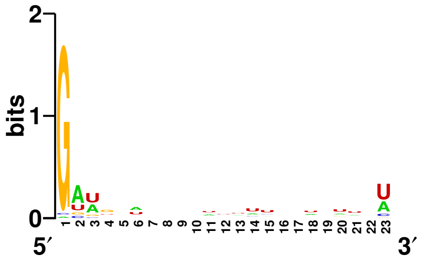

24-mers:

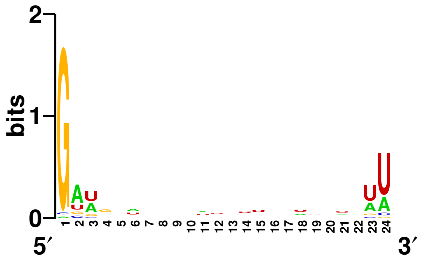

25-mers:

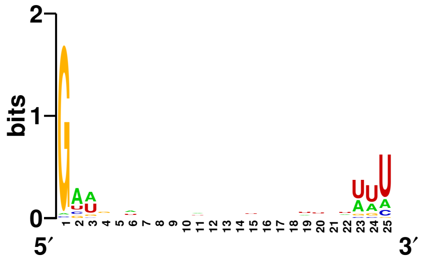

26-mers:

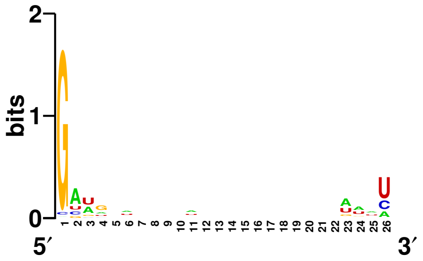

Library GSM455392 (18-26-mers, any number of 5´ phosphates, replicate 2):

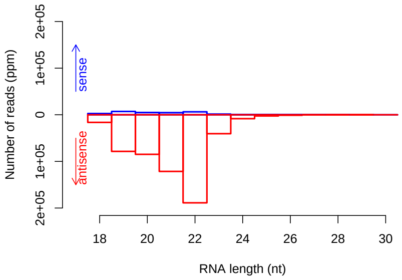

Sense reads:

18-mers:

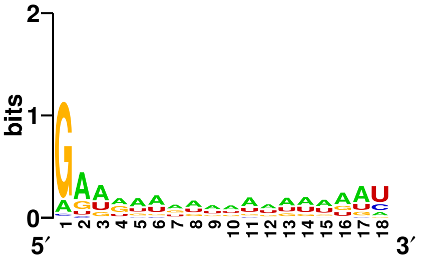

19-mers:

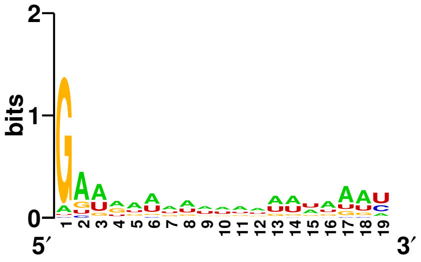

20-mers:

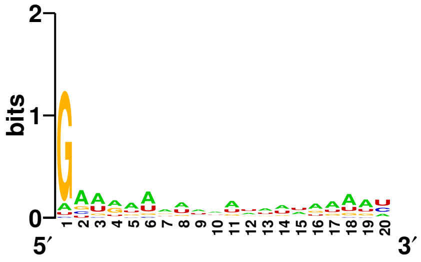

21-mers:

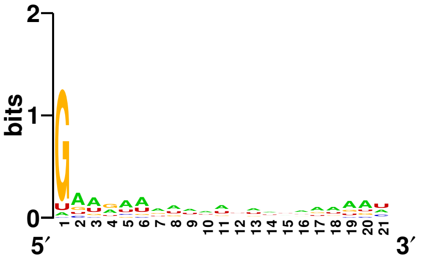

22-mers:

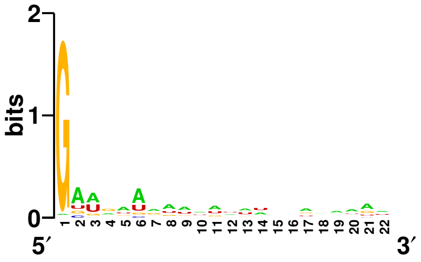

23-mers:

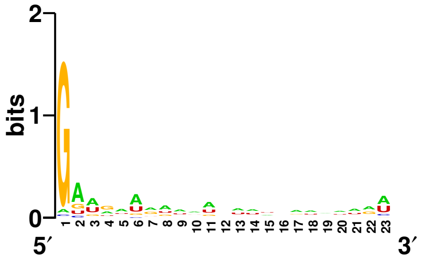

24-mers:

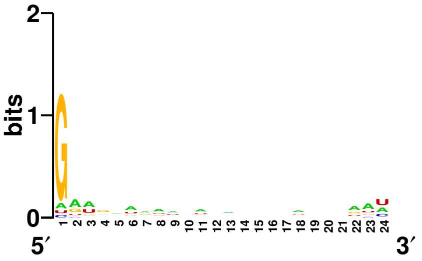

25-mers:

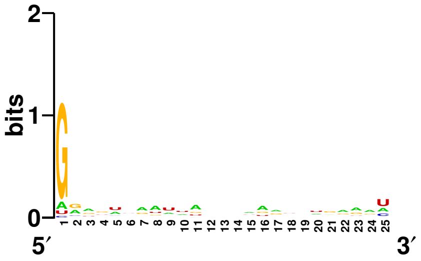

26-mers:

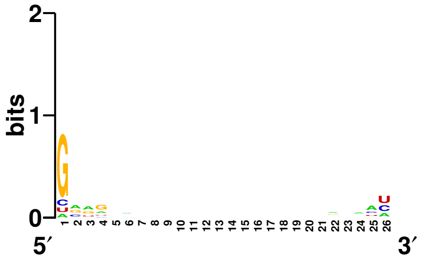

Antisense reads:

18-mers:

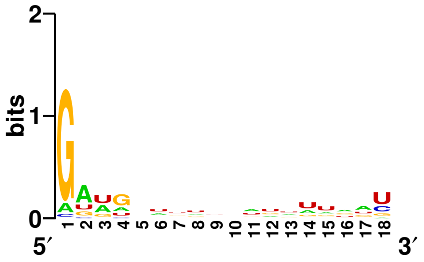

19-mers:

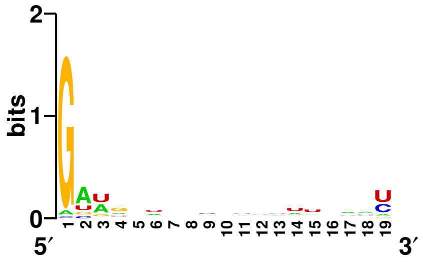

20-mers:

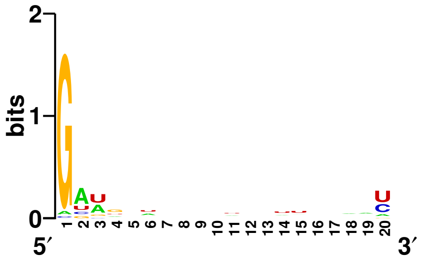

21-mers:

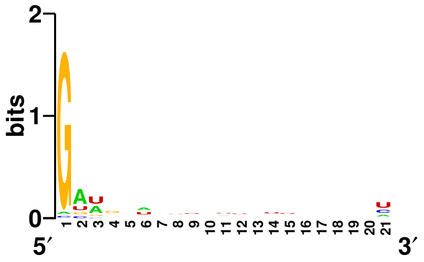

22-mers:

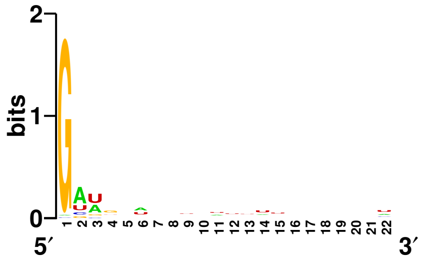

23-mers:

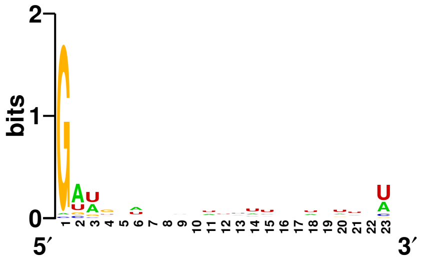

24-mers:

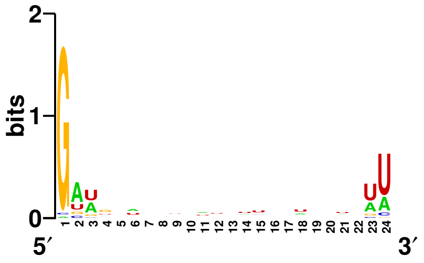

25-mers:

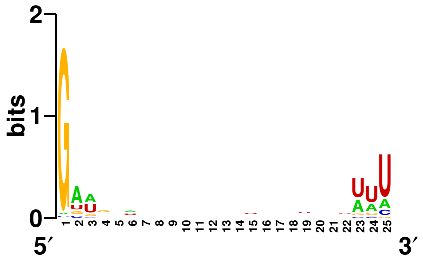

26-mers:

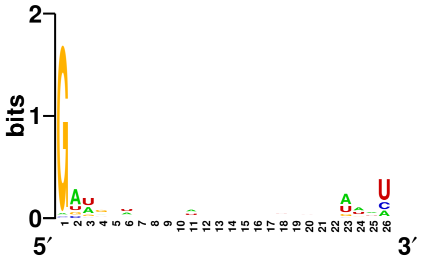

Library GSM455393 (18-26-mers, any number of 5´ phosphates, replicate 3):

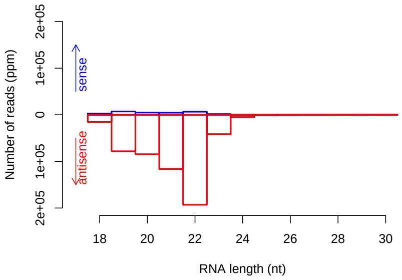

Sense reads:

18-mers:

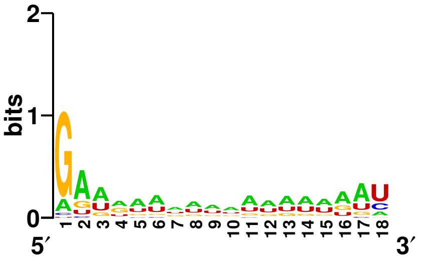

19-mers:

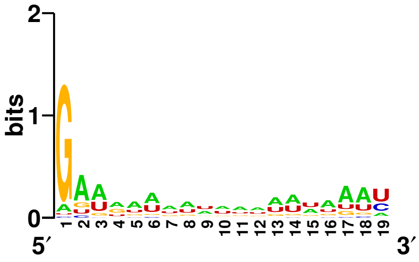

20-mers:

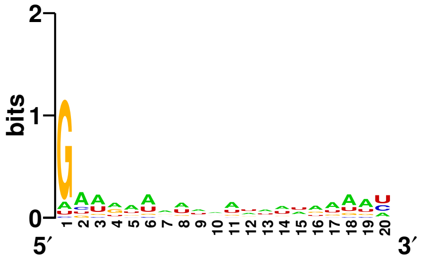

21-mers:

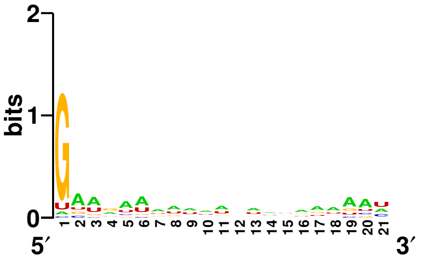

22-mers:

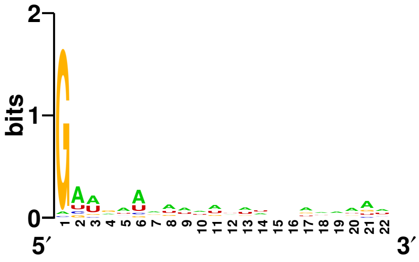

23-mers:

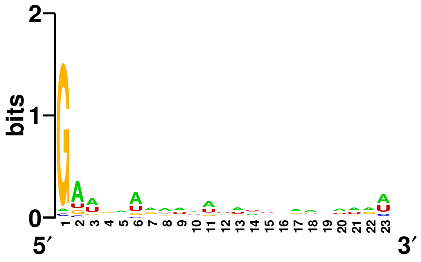

24-mers:

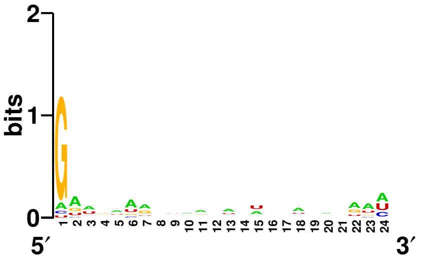

25-mers:

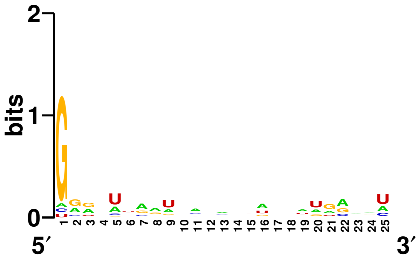

26-mers:

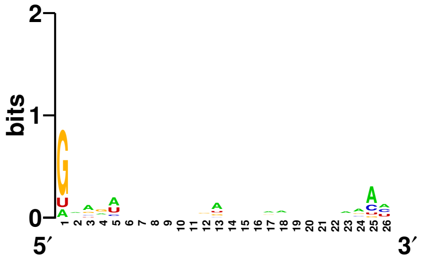

Antisense reads:

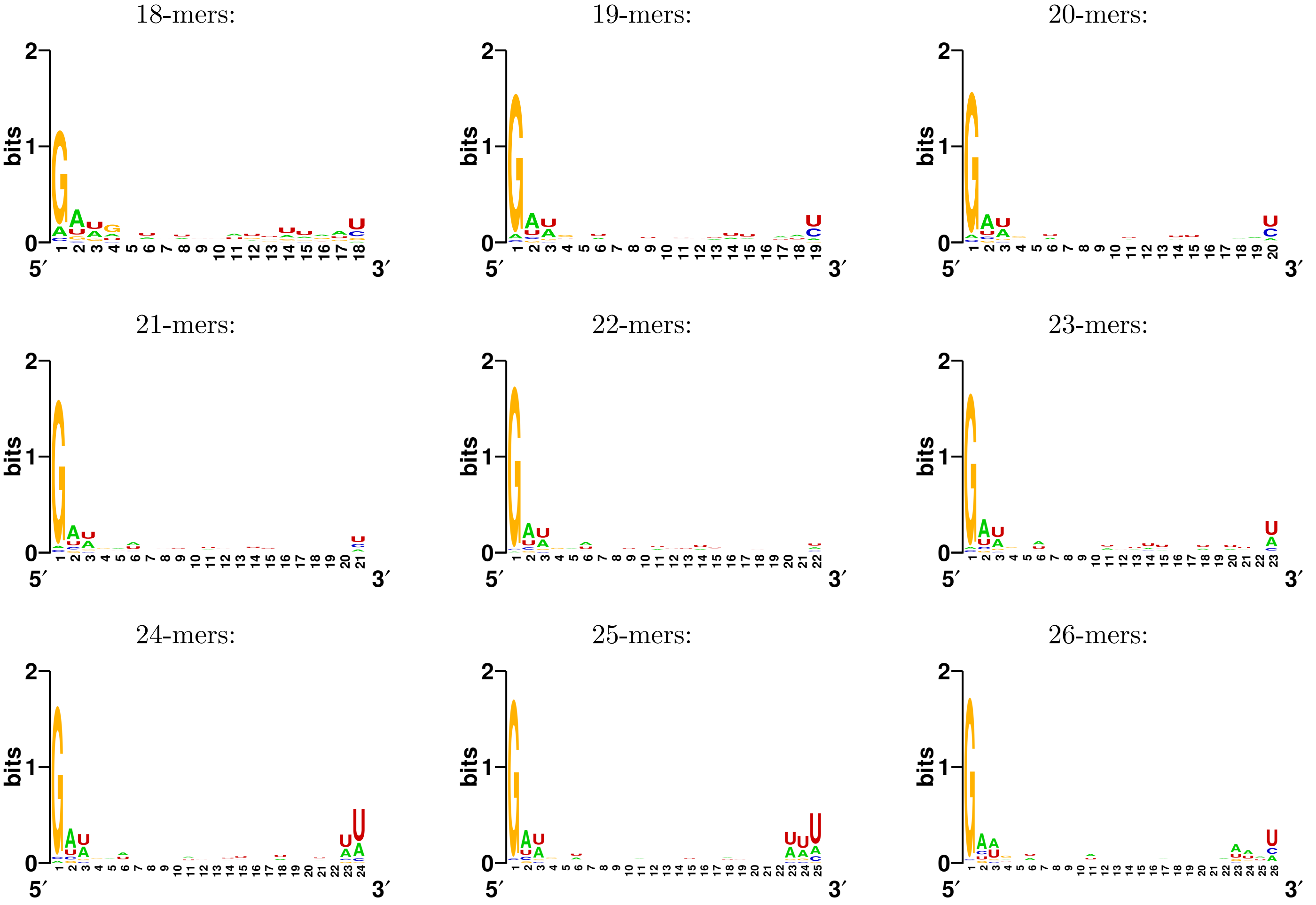

6.4 Reads matching RNAs with long ORF's

Library GSM455391 (18-26-mers, any number of 5´ phosphates, replicate 1):

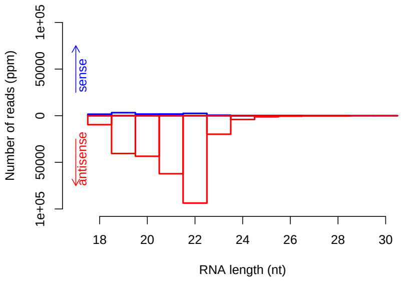

Sense reads:

18-mers:

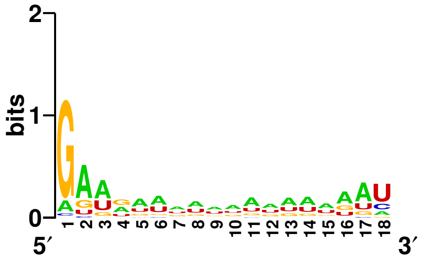

19-mers:

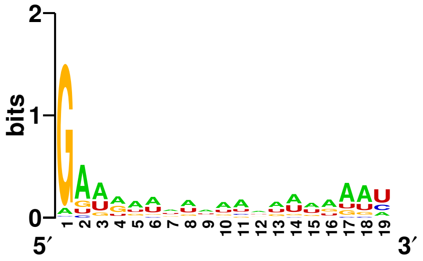

20-mers:

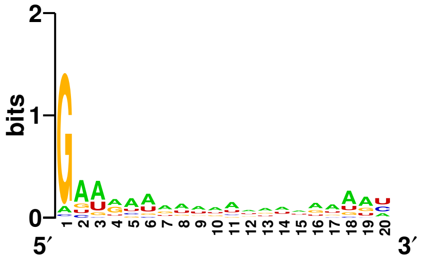

21-mers:

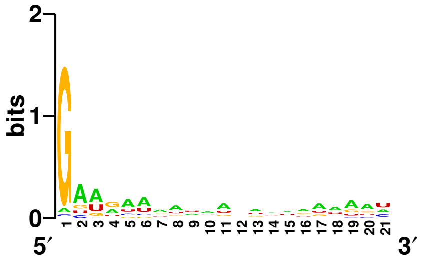

22-mers:

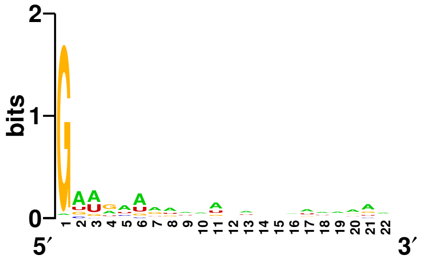

23-mers:

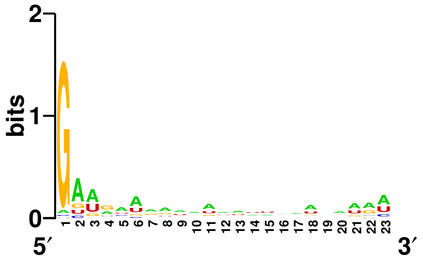

24-mers:

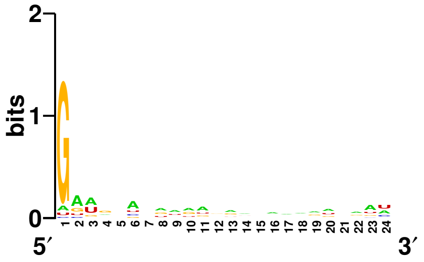

25-mers:

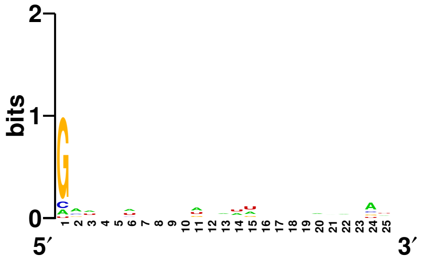

26-mers:

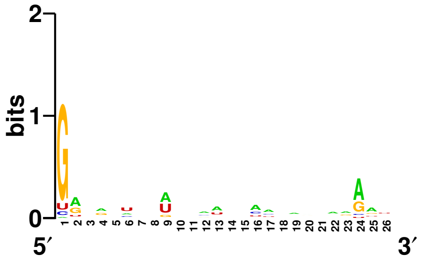

Antisense reads:

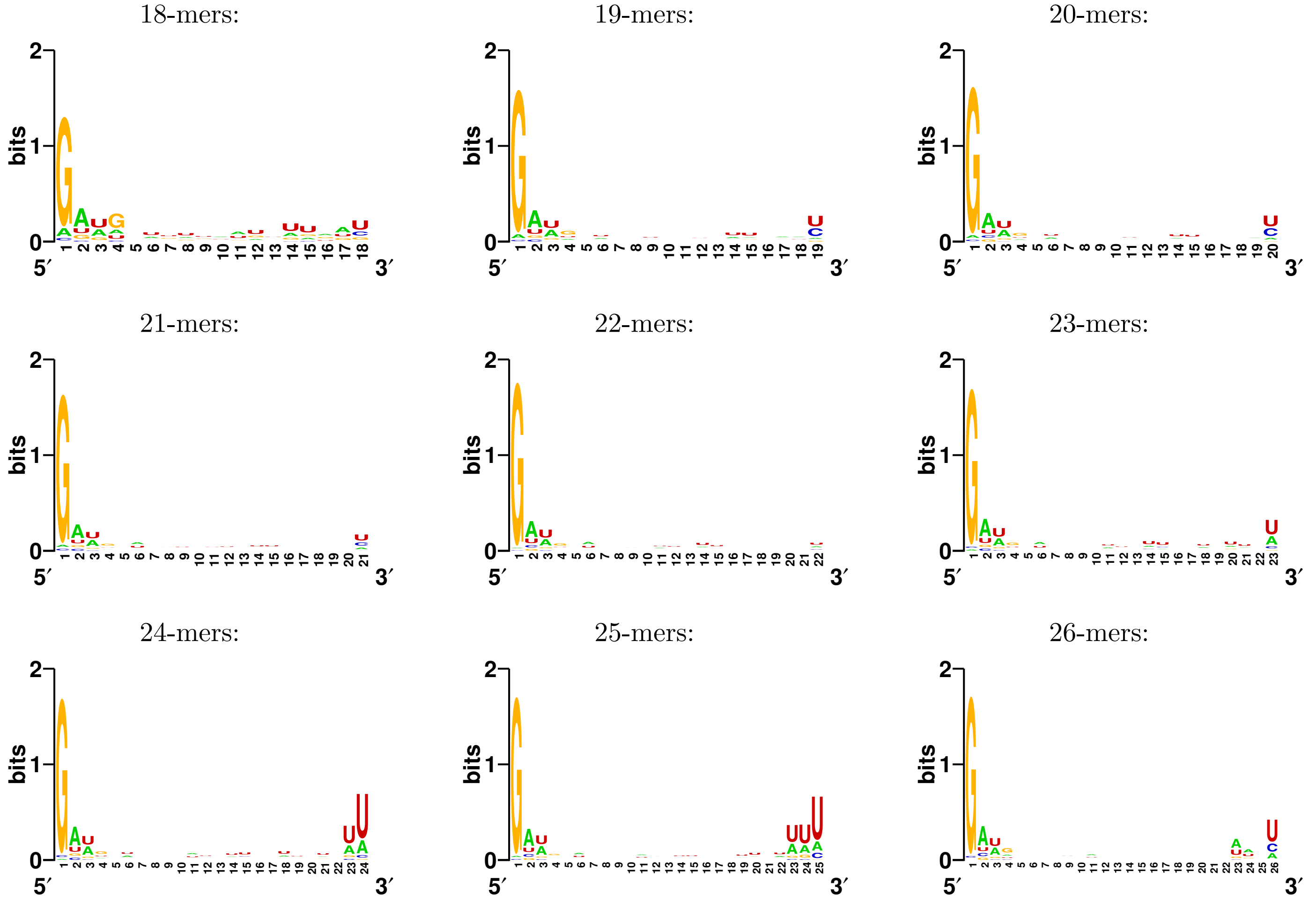

Library GSM455392 (18-26-mers, any number of 5´ phosphates, replicate 2):

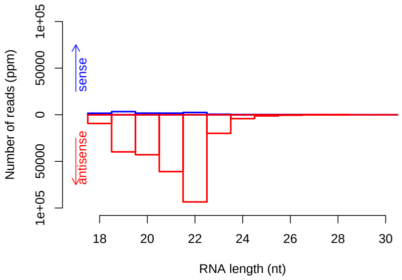

Sense reads:

18-mers:

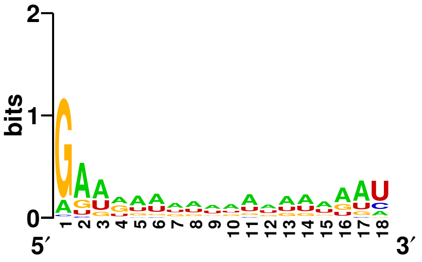

19-mers:

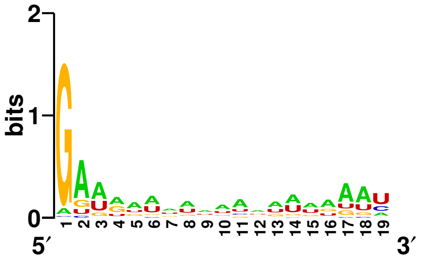

20-mers:

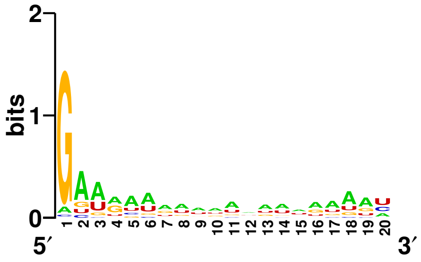

21-mers:

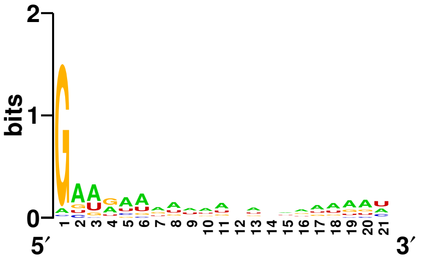

22-mers:

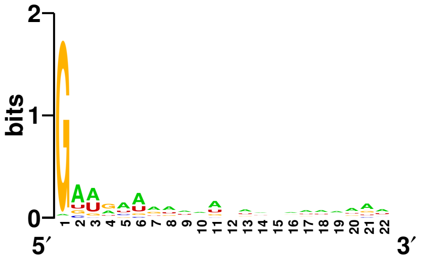

23-mers:

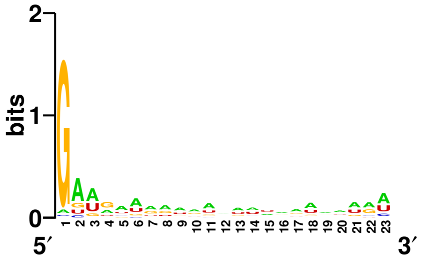

24-mers:

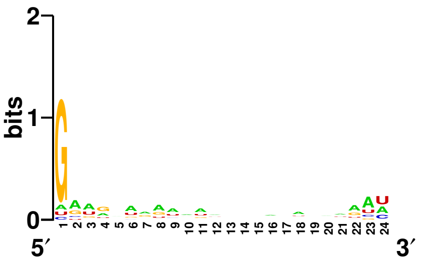

25-mers:

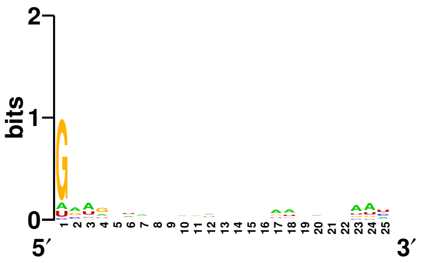

26-mers:

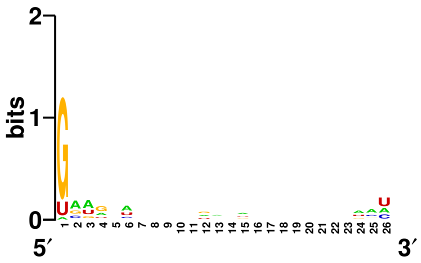

Antisense reads:

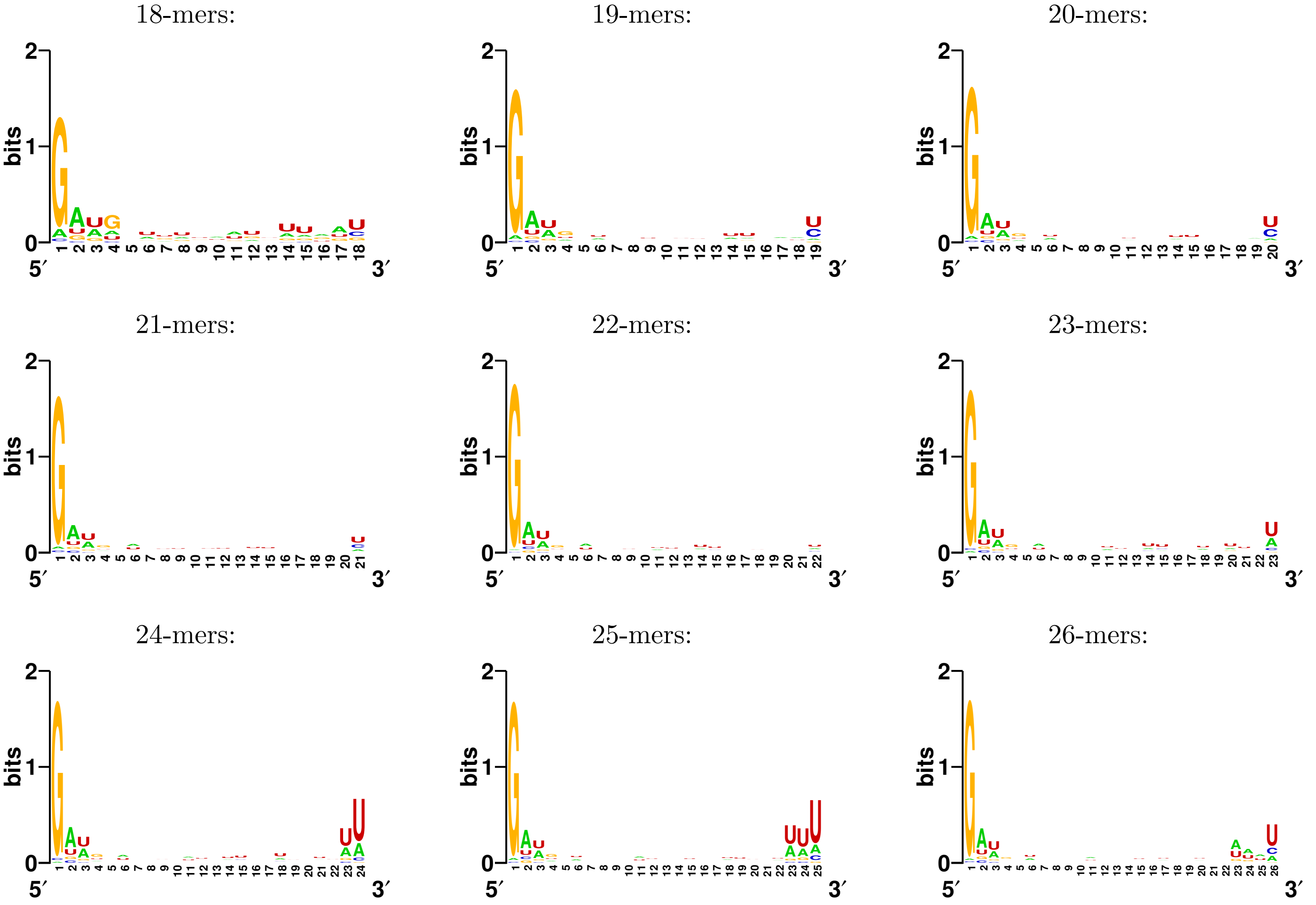

Library GSM455393 (18-26-mers, any number of 5´ phosphates, replicate 3):

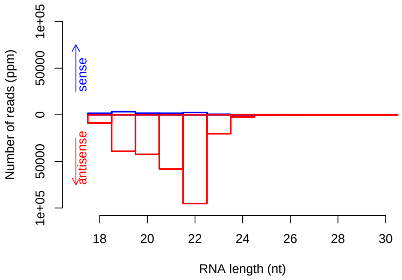

Sense reads:

18-mers:

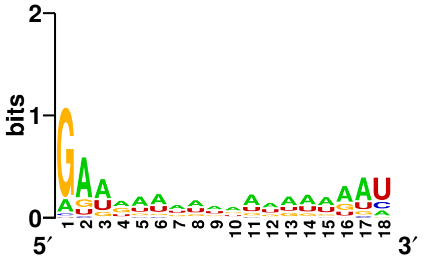

19-mers:

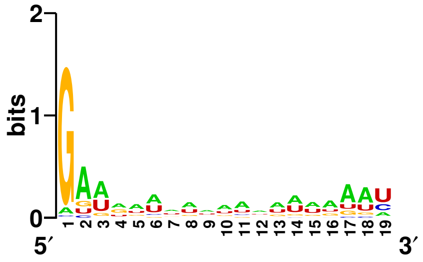

20-mers:

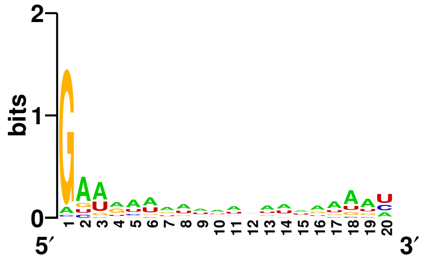

21-mers:

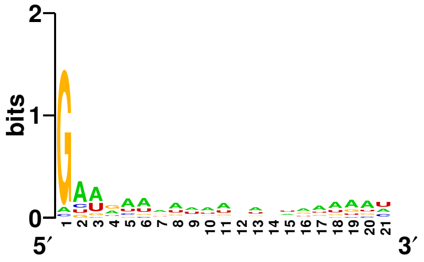

22-mers:

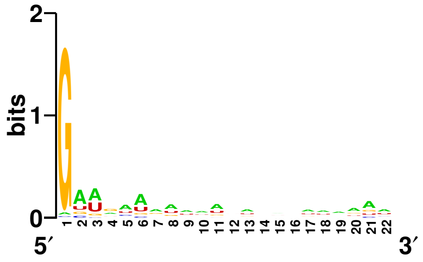

23-mers:

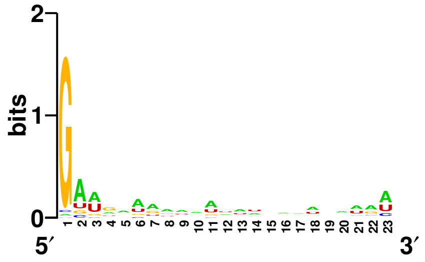

24-mers:

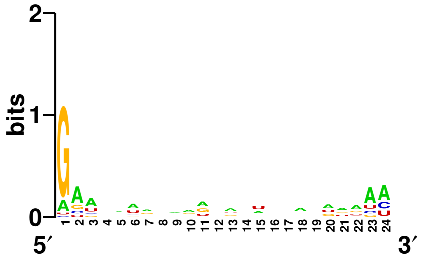

25-mers:

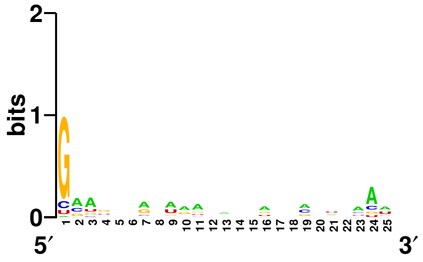

26-mers:

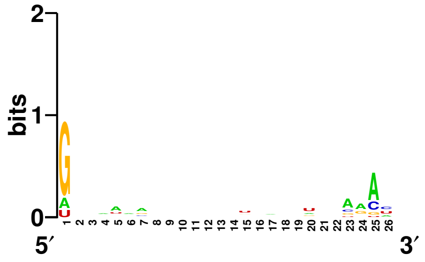

Antisense reads:

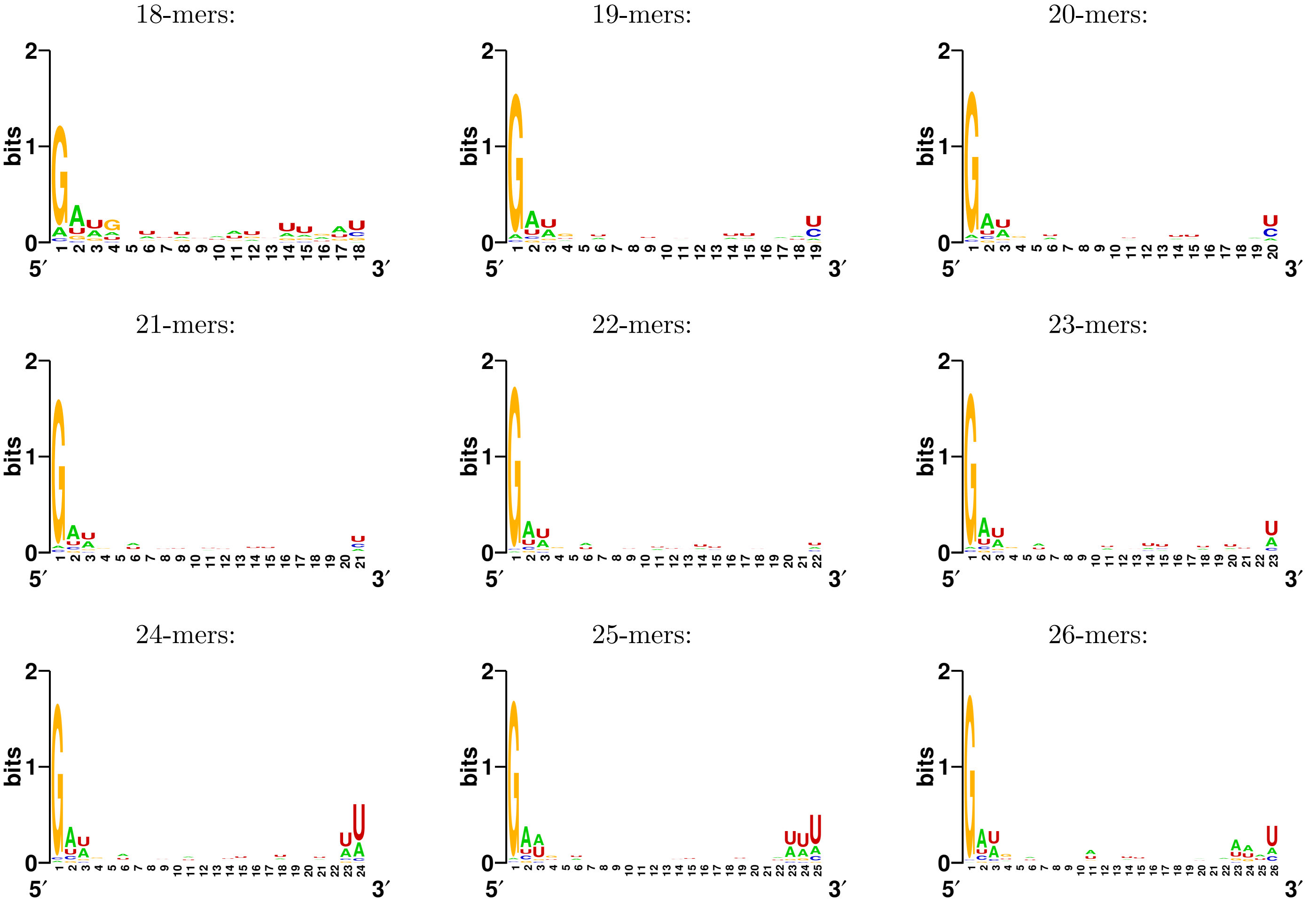

6.5 Extragenomic and extratranscriptomic reads

Library GSM455391 (18-26-mers, any number of 5´ phosphates, replicate 1):

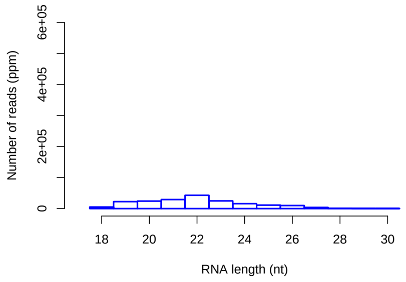

18-mers:

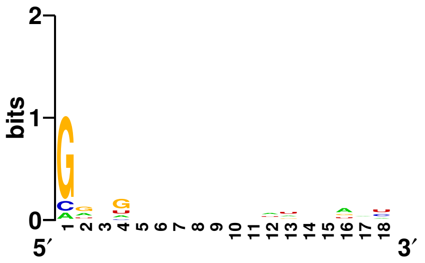

19-mers:

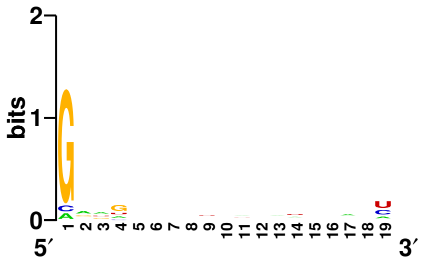

20-mers:

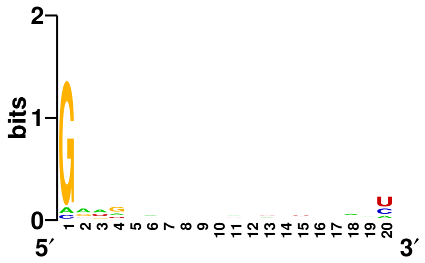

21-mers:

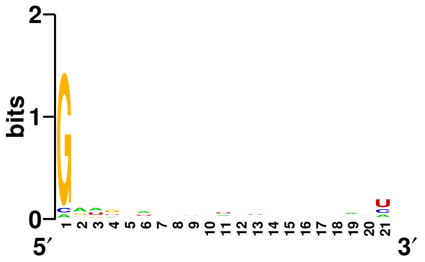

22-mers:

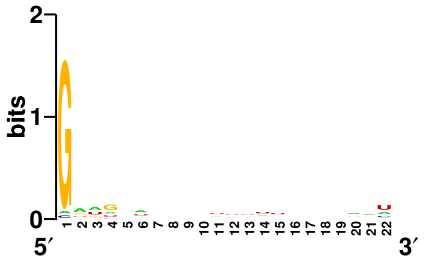

23-mers:

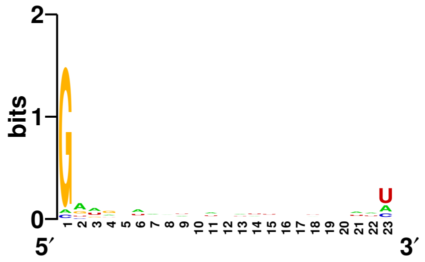

24-mers:

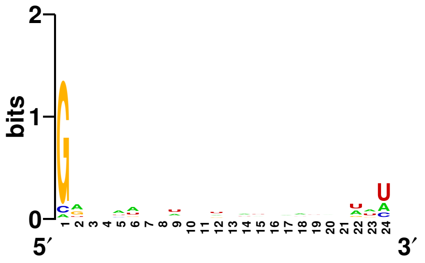

25-mers:

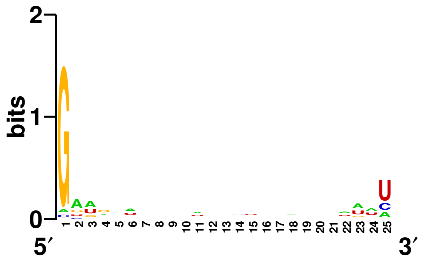

26-mers:

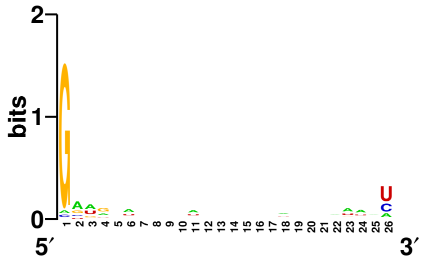

Library GSM455392 (18-26-mers, any number of 5′ phosphates, replicate 2):

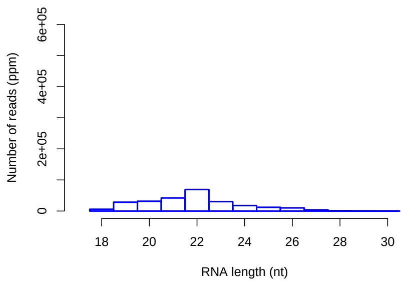

18-mers:

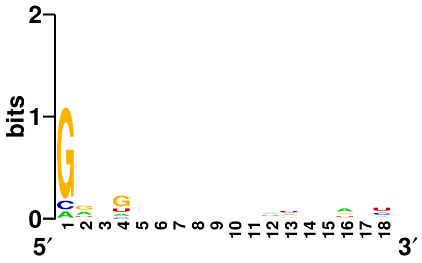

19-mers:

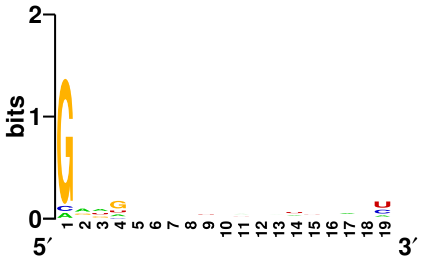

20-mers:

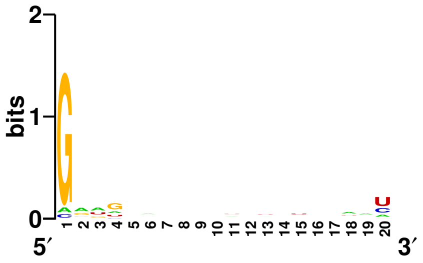

21-mers:

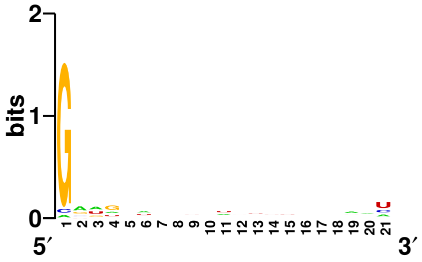

22-mers:

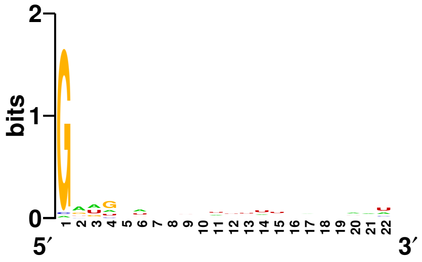

23-mers:

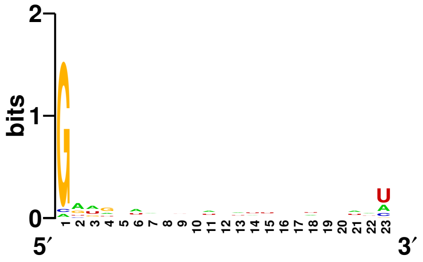

24-mers:

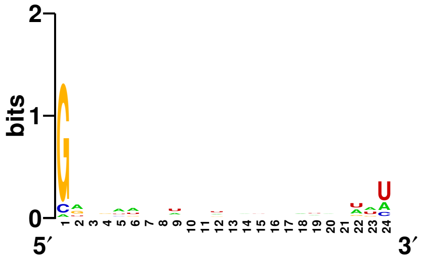

25-mers:

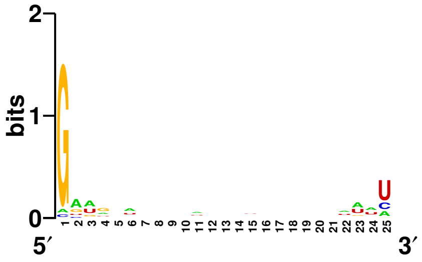

26-mers:

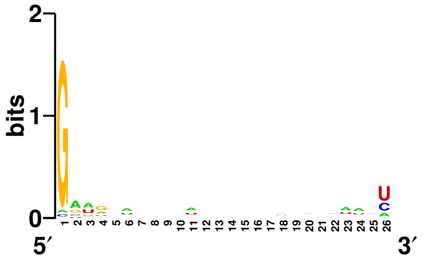

Library GSM455393 (18-26-mers, any number of 5´ phosphates, replicate 3):

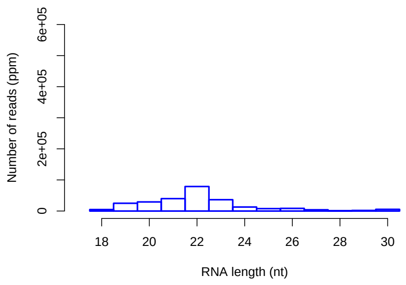

18-mers:

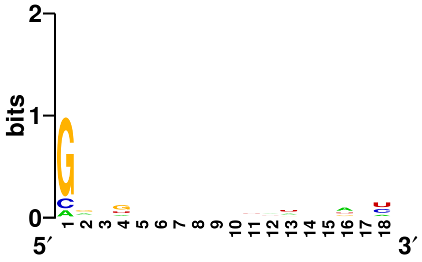

19-mers:

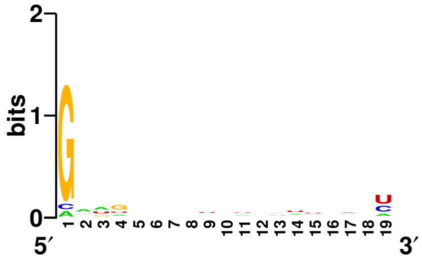

20-mers:

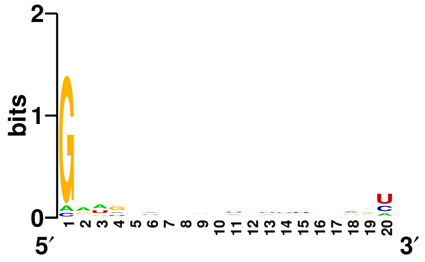

21-mers:

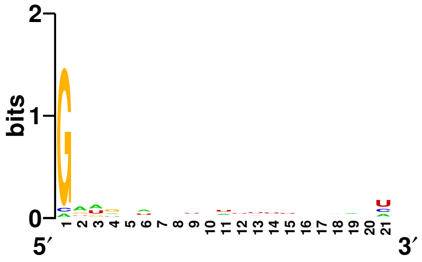

22-mers:

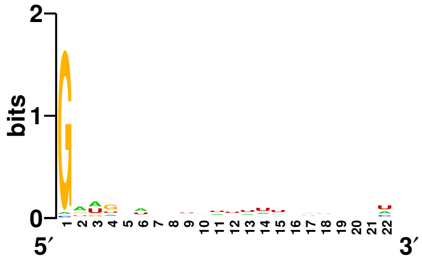

23-mers:

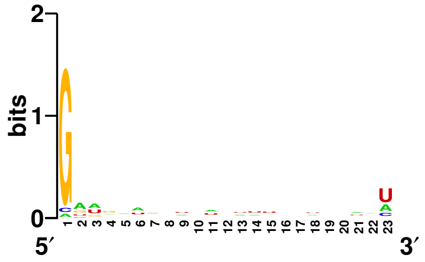

24-mers:

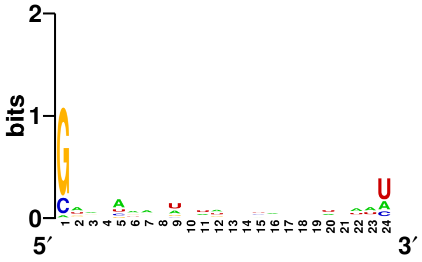

25-mers:

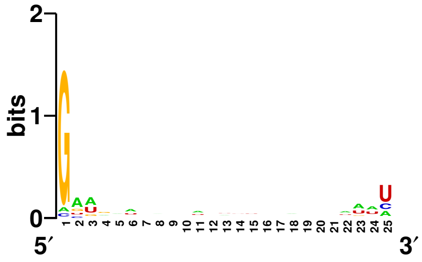

26-mers:

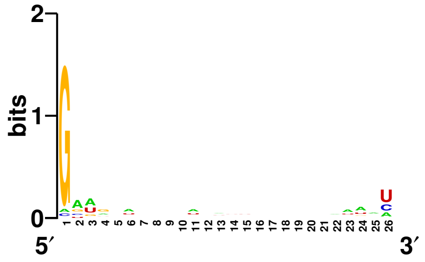

6.6 Extragenomic reads matching the transcriptome

Library GSM455391 (18-26-mers, any number of 5´ phosphates, replicate 1):

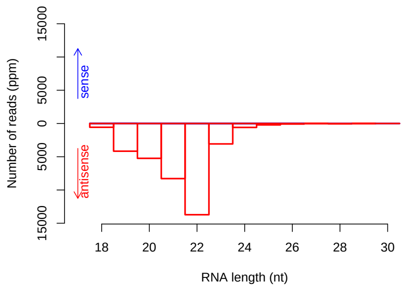

Sense reads:

18-mers:

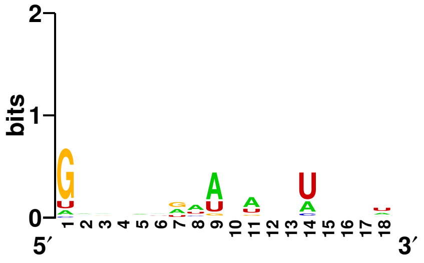

19-mers:

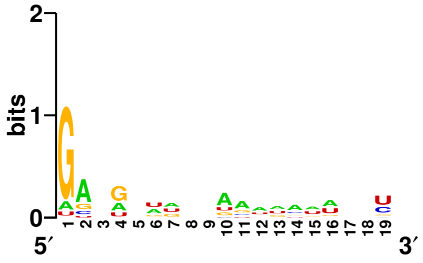

20-mers:

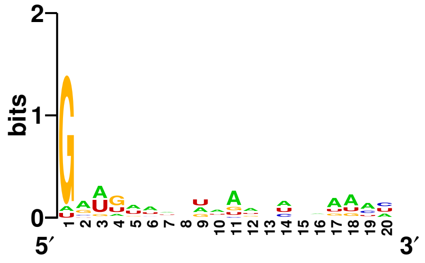

21-mers:

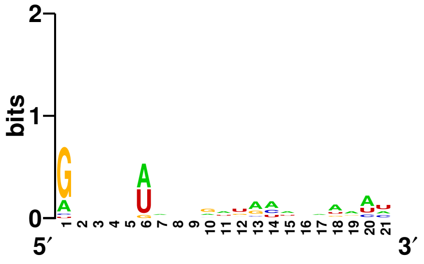

22-mers:

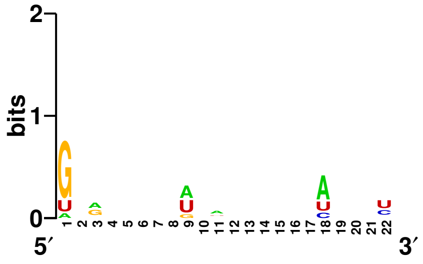

23-mers:

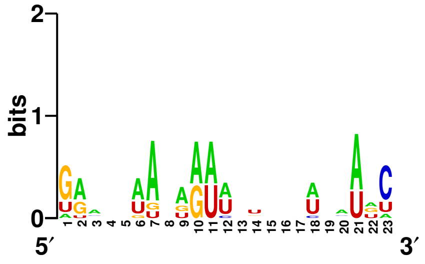

24-mers:

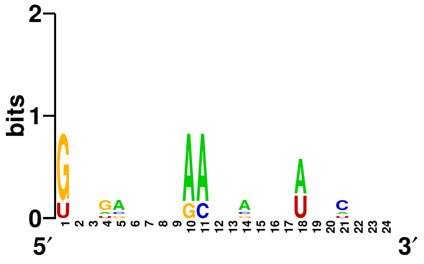

25-mers:

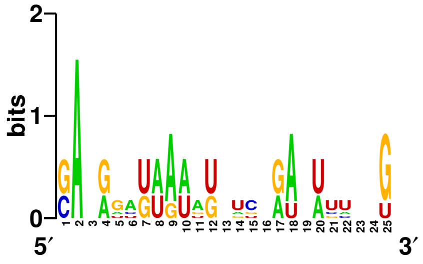

26-mers:

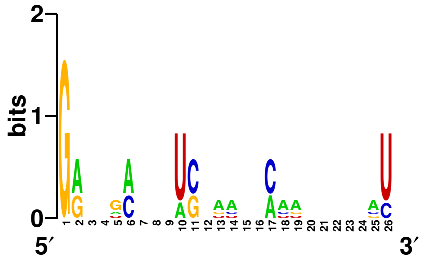

Antisense reads:

18-mers:

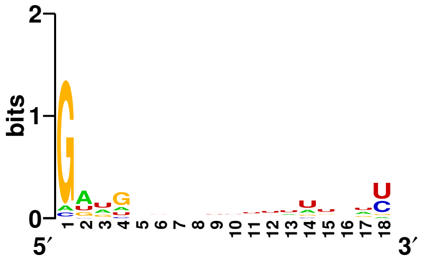

19-mers:

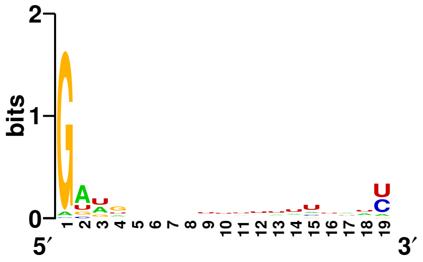

20-mers:

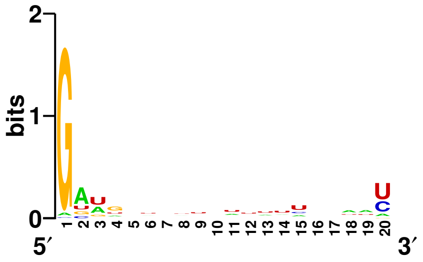

21-mers:

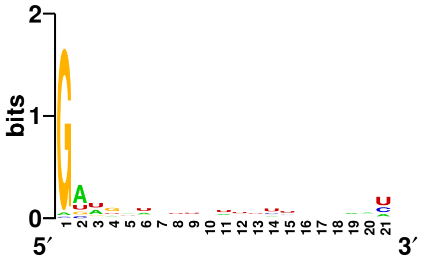

22-mers:

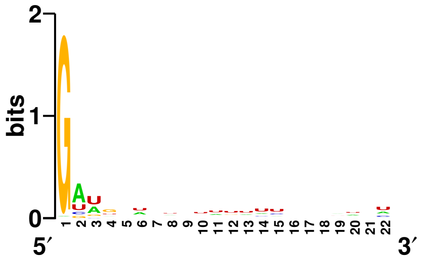

23-mers:

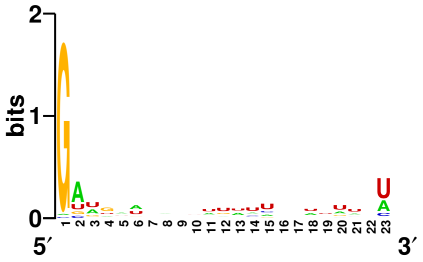

24-mers:

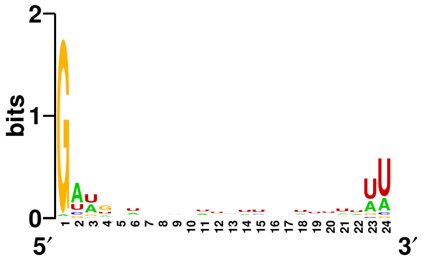

25-mers:

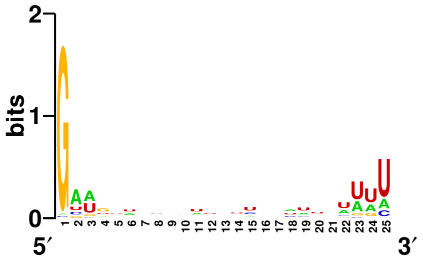

26-mers:

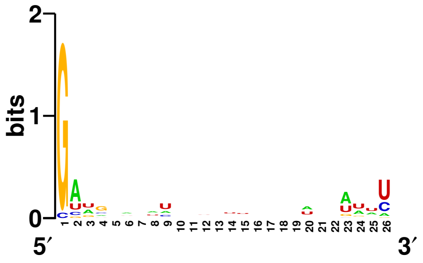

Library GSM455392 (18-26-mers, any number of 5´ phosphates, replicate 2):

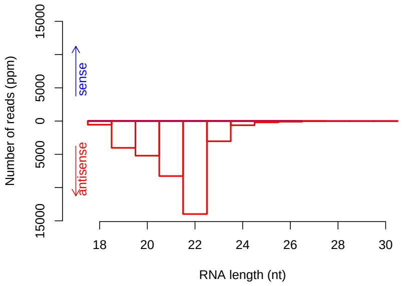

Sense reads:

18-mers:

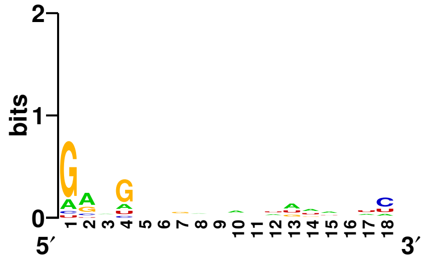

19-mers:

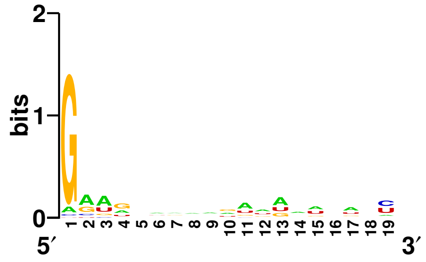

20-mers:

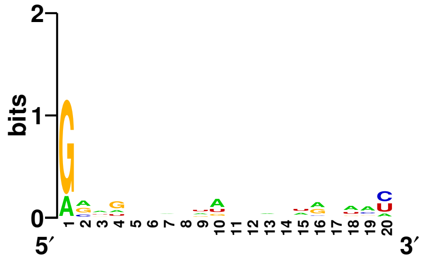

21-mers:

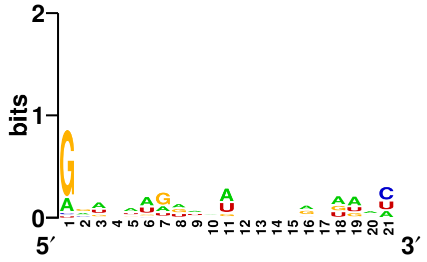

22-mers:

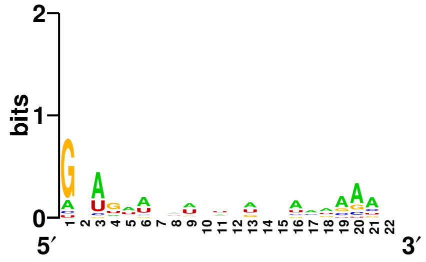

23-mers:

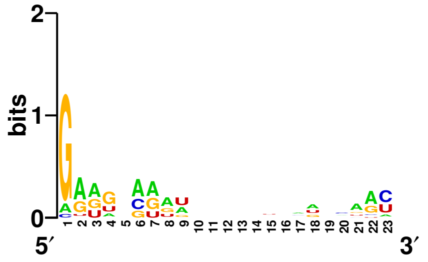

24-mers:

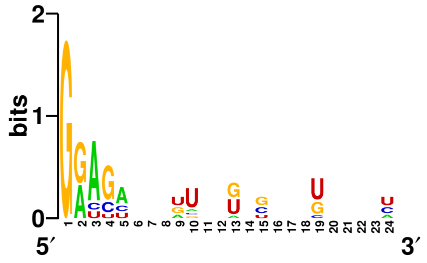

25-mers:

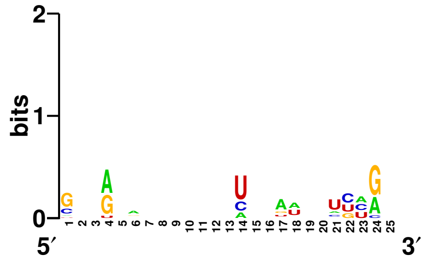

26-mers:

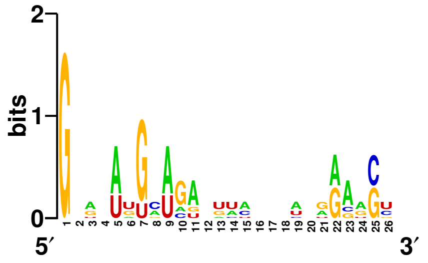

Antisense reads:

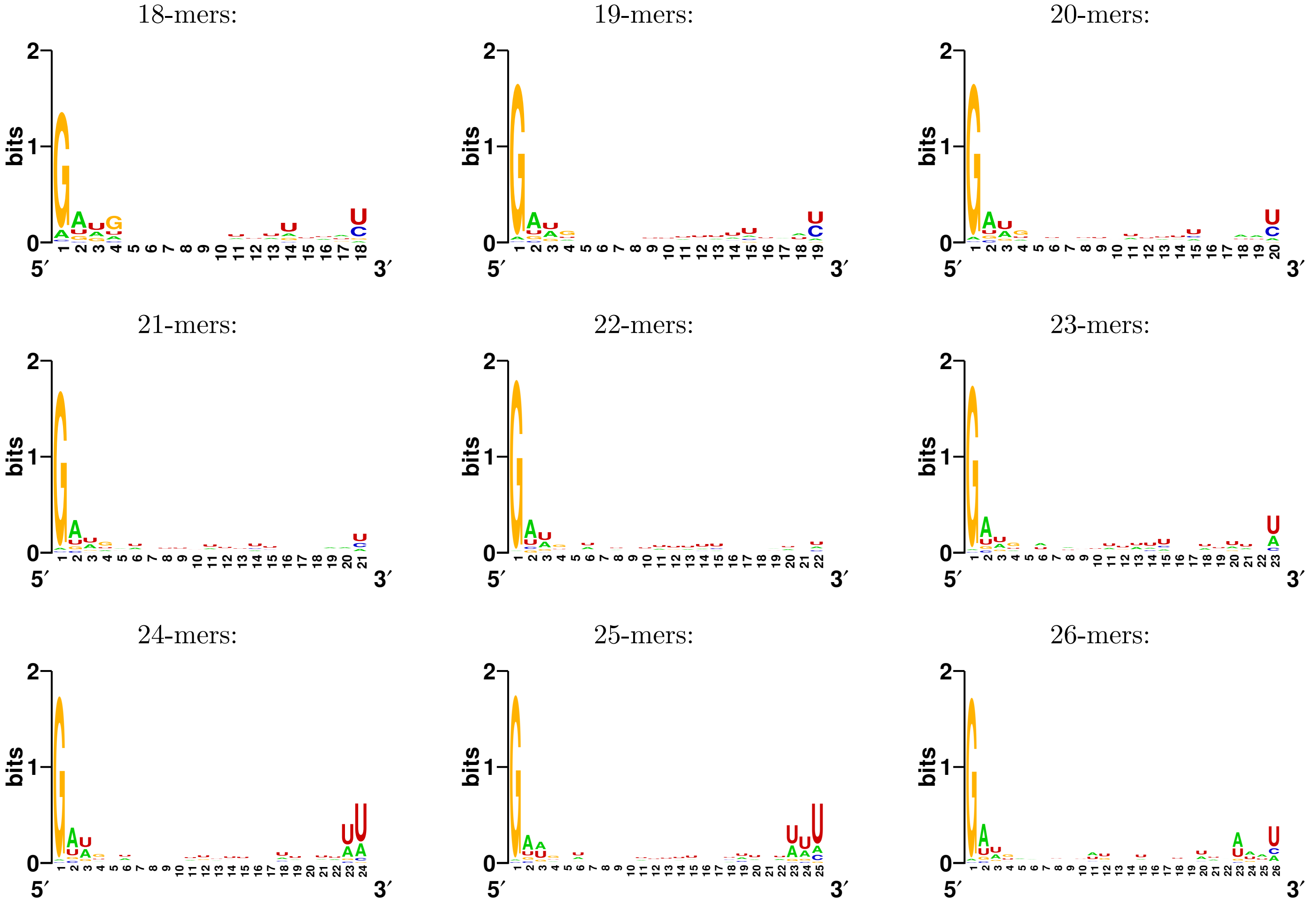

Library GSM455393 (18-26-mers, any number of 5´ phosphates, replicate 3):

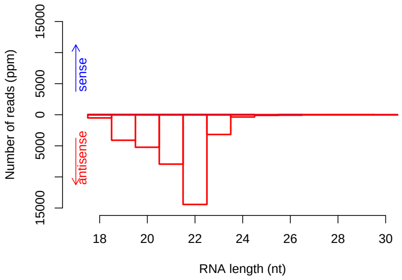

Sense reads:

18-mers:

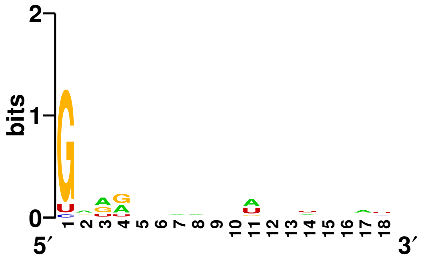

19-mers:

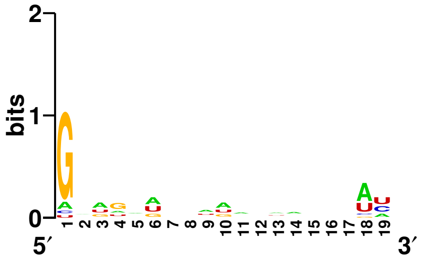

20-mers:

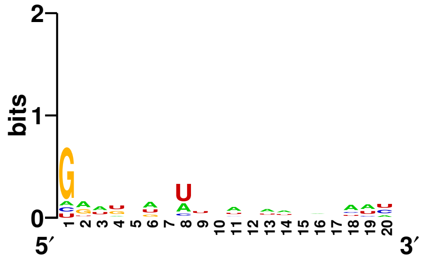

21-mers:

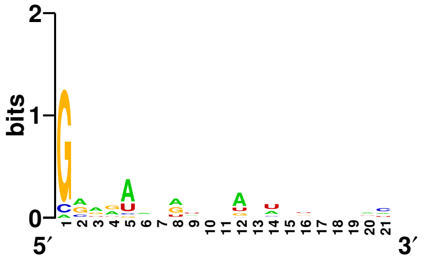

22-mers:

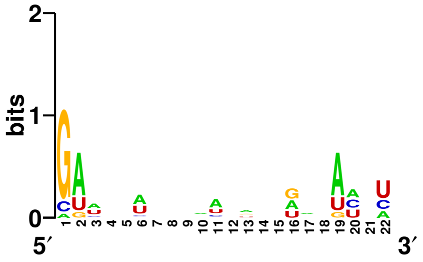

23-mers:

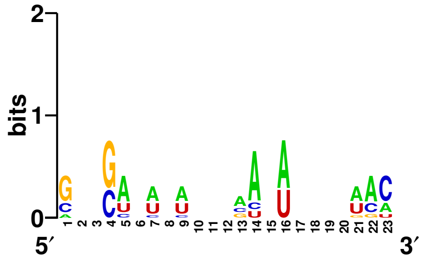

24-mers:

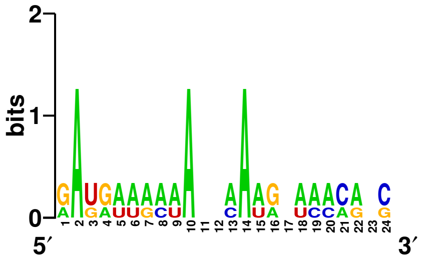

25-mers:

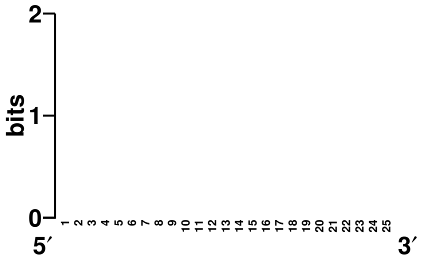

26-mers:

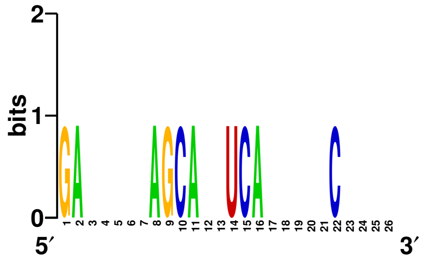

Antisense reads:

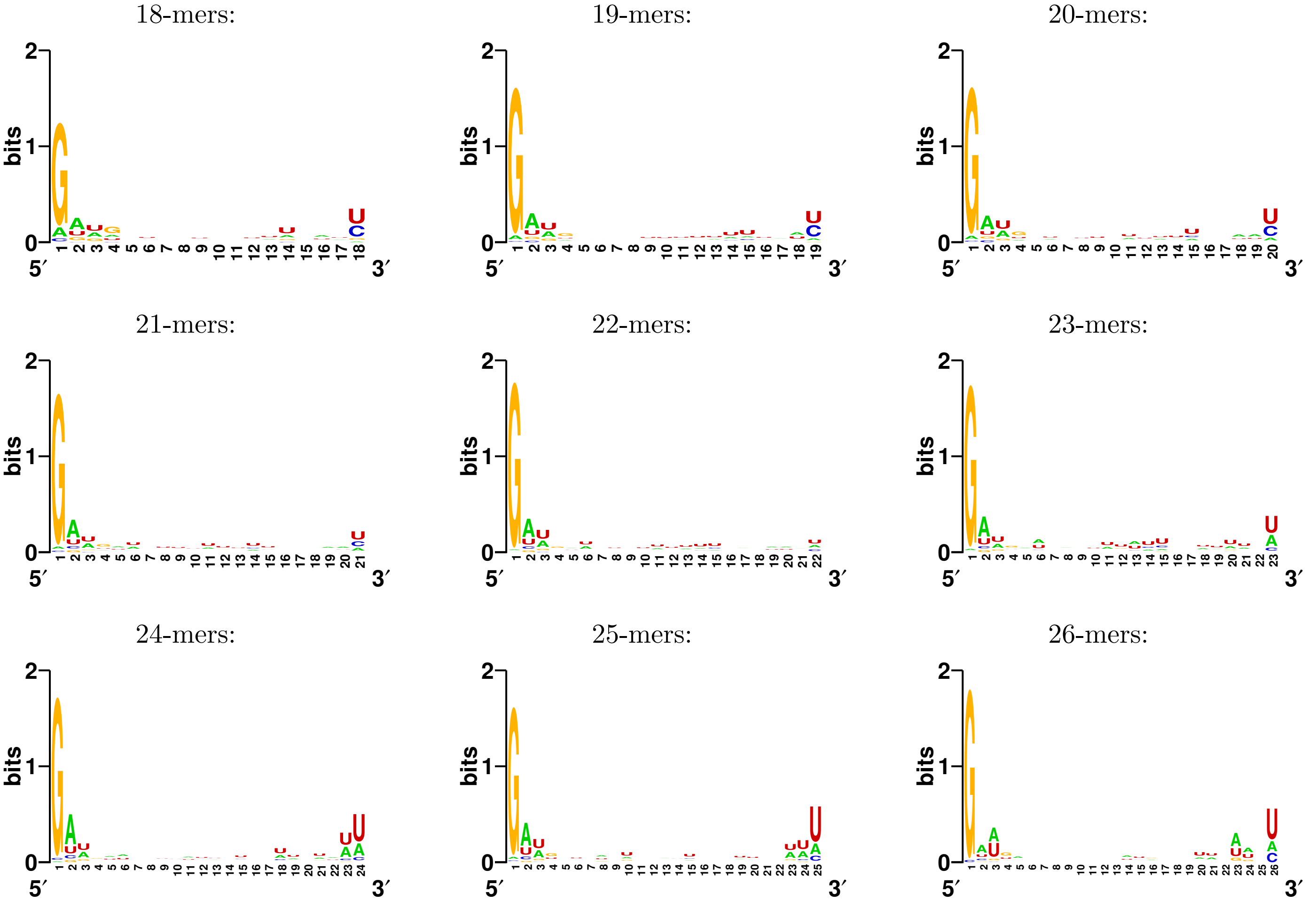

7 Extragenomic and extratranscriptomic reads matching the *Staphylococcus aureus* genome

7.1 Libraries #1 (total 5´ monophosphorylated small RNAs)

Embryo 8h, library 1:

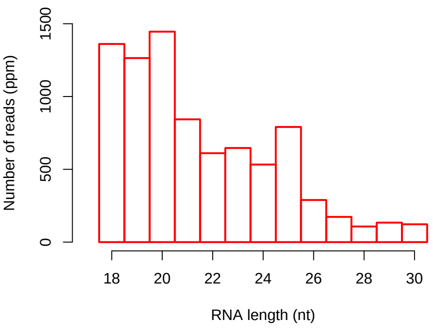

18-mers:

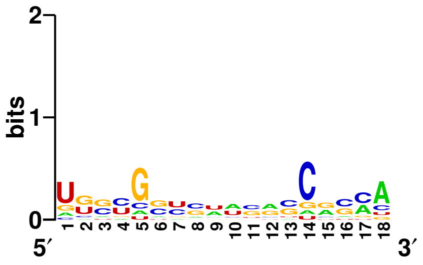

19-mers:

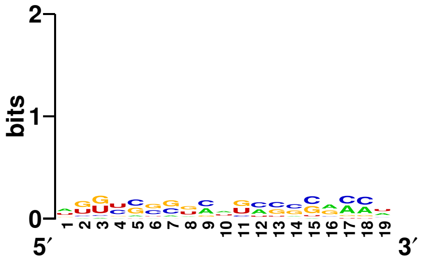

20-mers:

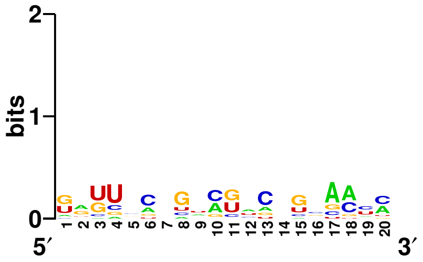

21-mers:

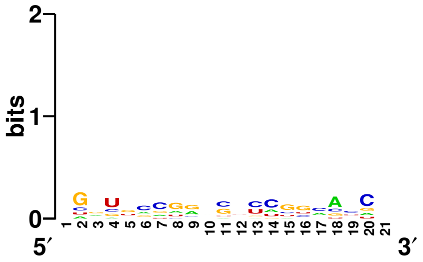

22-mers:

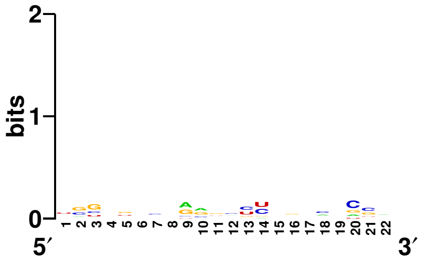

23-mers:

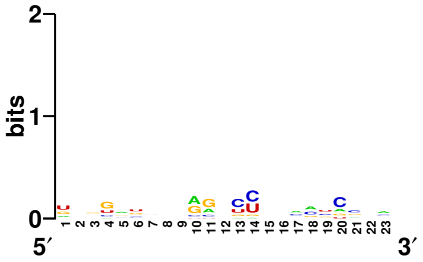

24-mers:

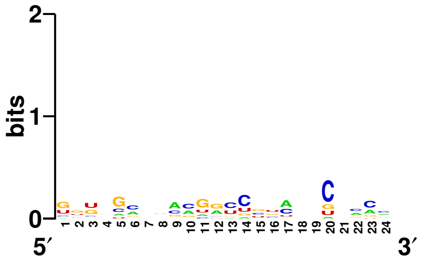

25-mers:

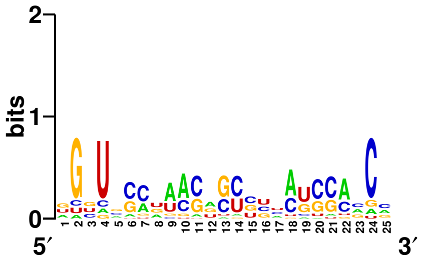

26-mers:

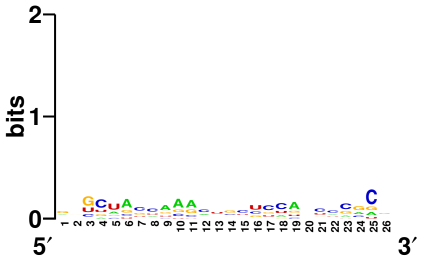

27-mers:

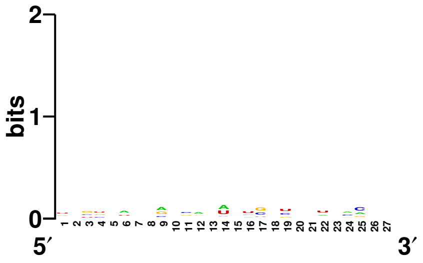

28-mers:

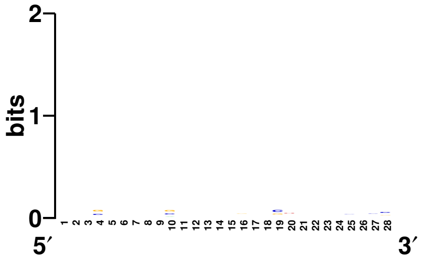

29-mers:

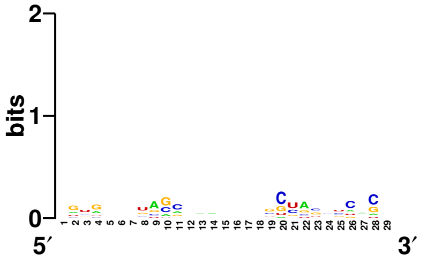

30-mers:

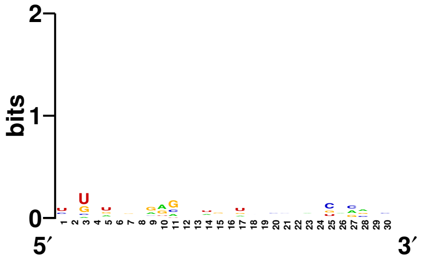

Embryo 15h, library 1:

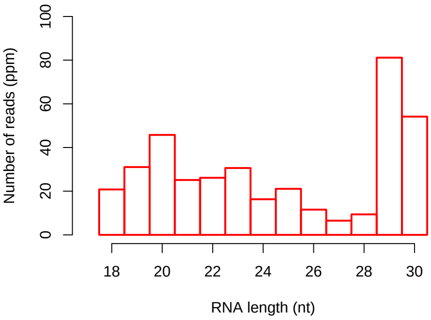

18-mers:

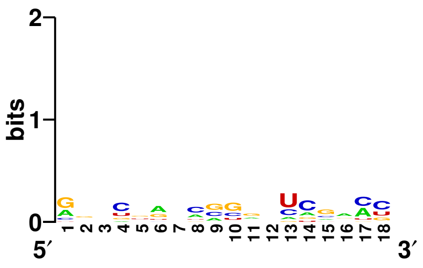

19-mers:

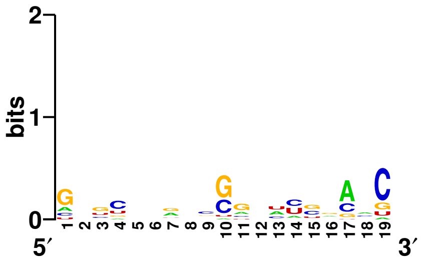

20-mers:

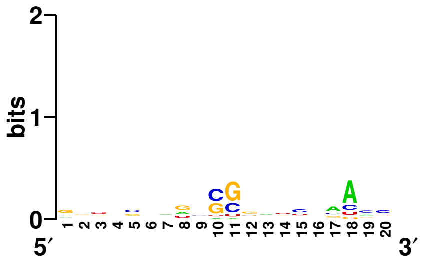

21-mers:

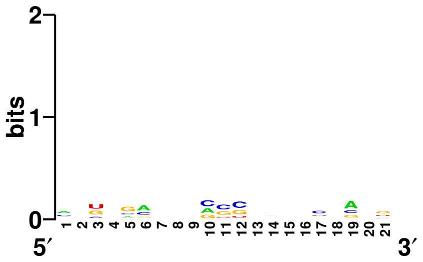

22-mers:

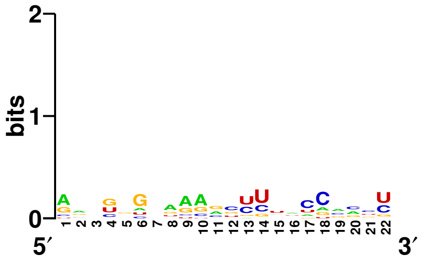

23-mers:

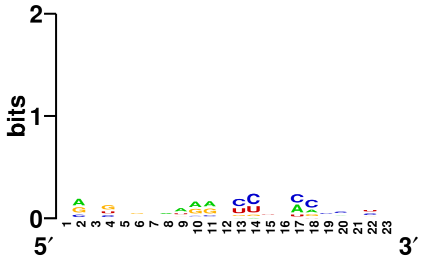

24-mers:

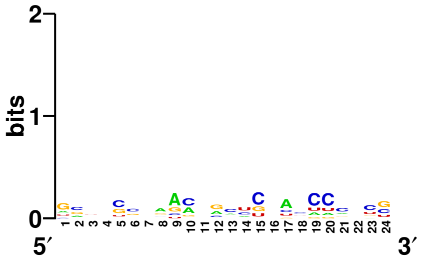

25-mers:

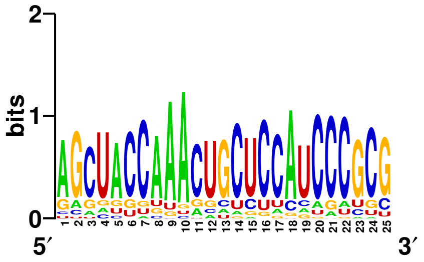

26-mers:

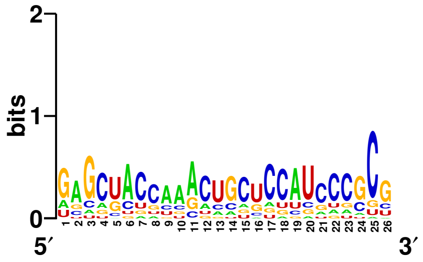

27-mers:

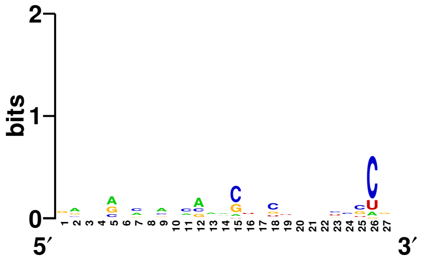

28-mers:

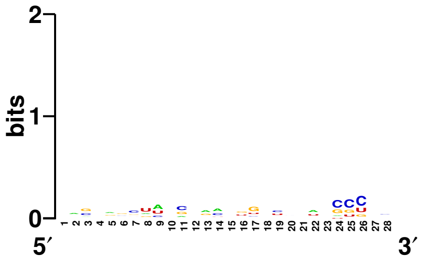

29-mers:

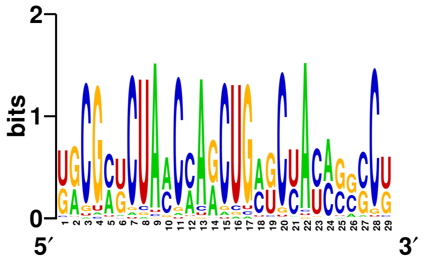

30-mers:

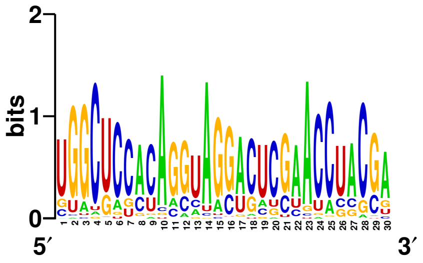

Embryo 36h, library 1:

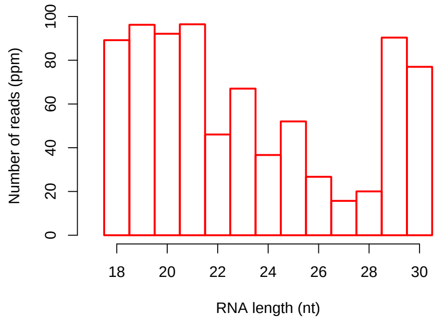

18-mers:

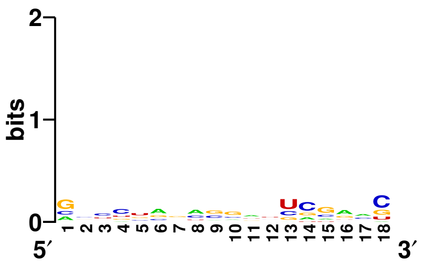

19-mers:

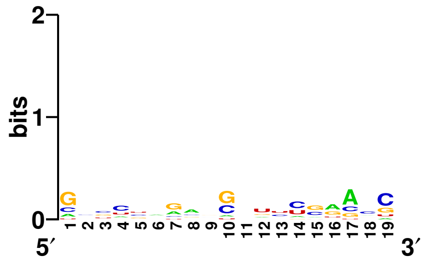

20-mers:

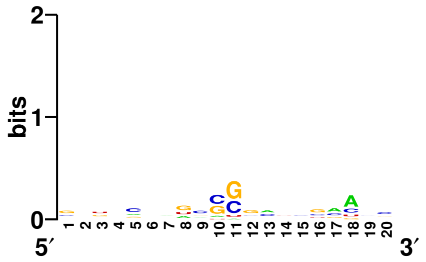

21-mers:

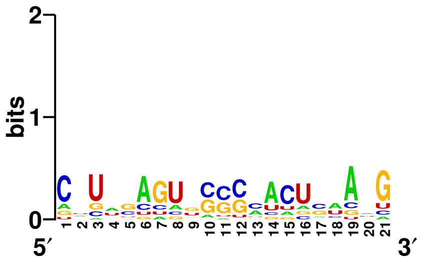

22-mers:

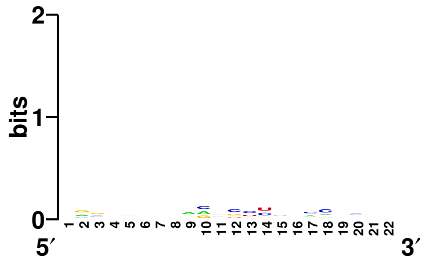

23-mers:

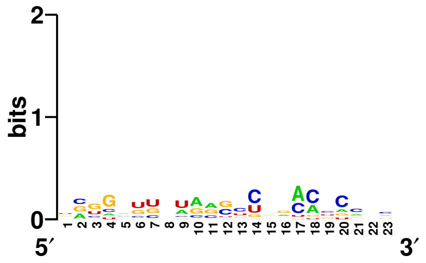

24-mers:

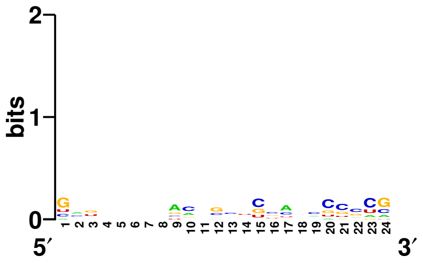

25-mers:

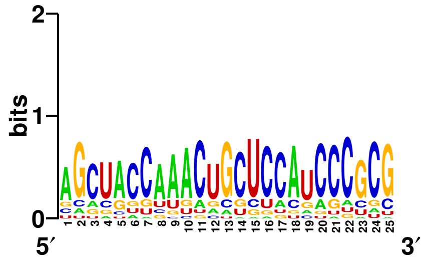

26-mers:

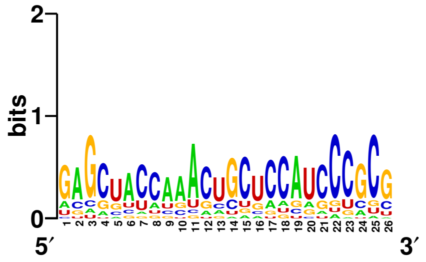

27-mers:

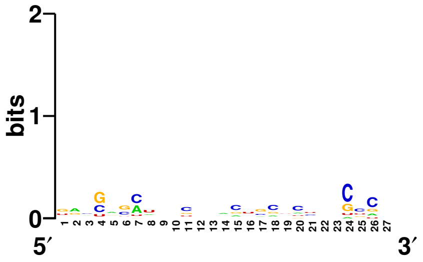

28-mers:

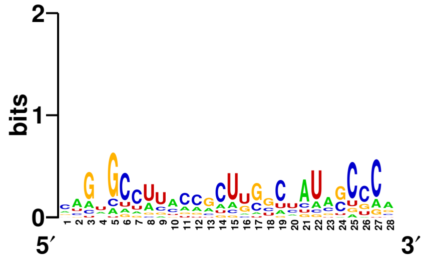

29-mers:

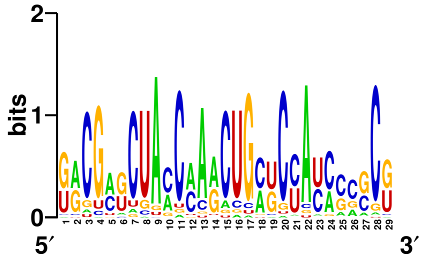

30-mers:

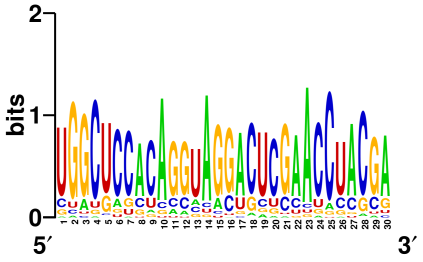

Embryo 60h, library 1:

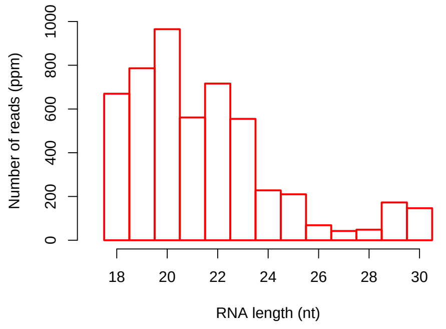

18-mers:

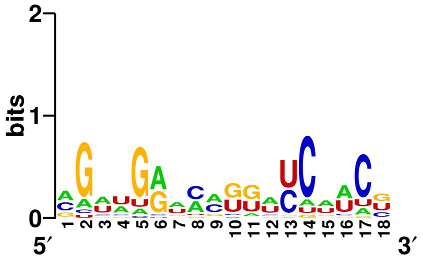

19-mers:

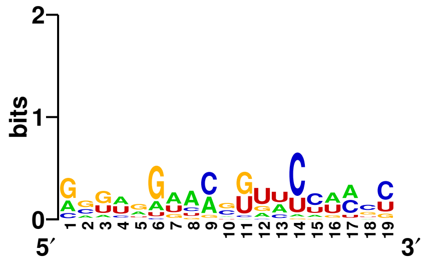

20-mers:

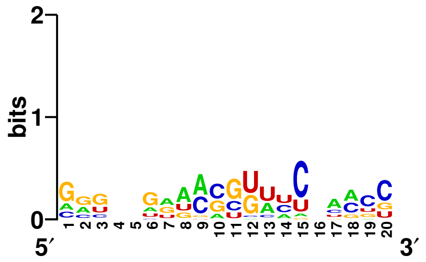

21-mers:

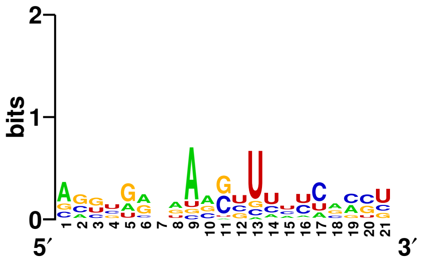

22-mers:

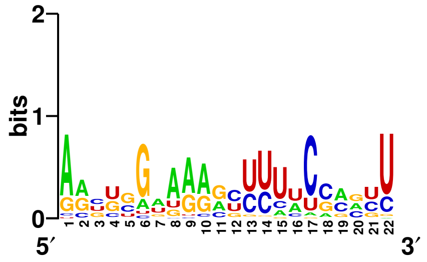

23-mers:

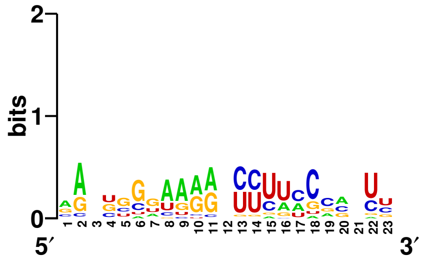

24-mers:

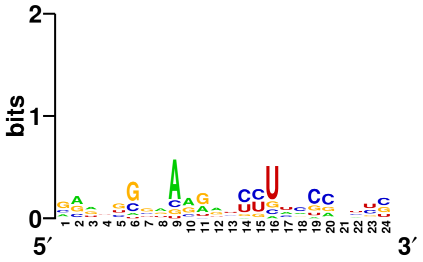

25-mers:

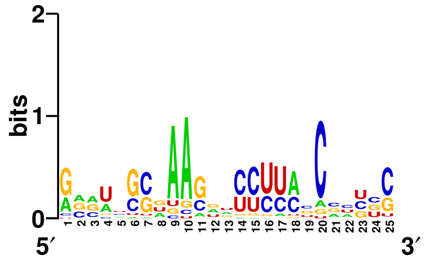

26-mers:

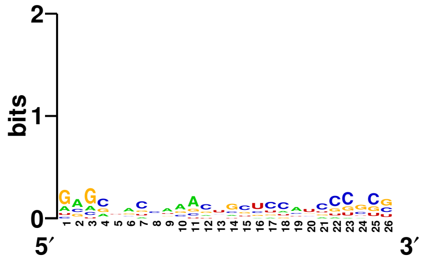

27-mers:

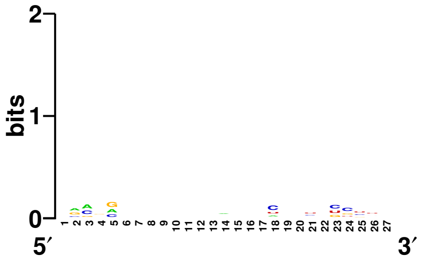

28-mers:

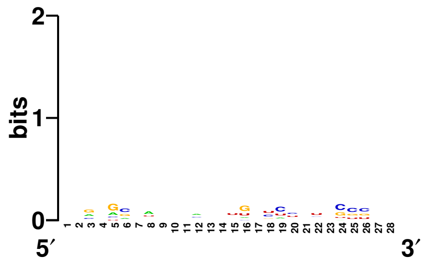

29-mers:

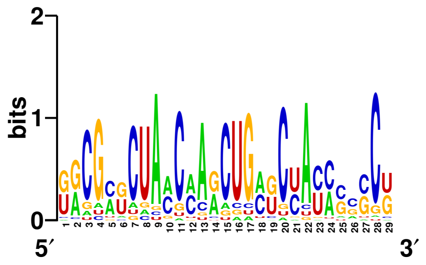

30-mers:

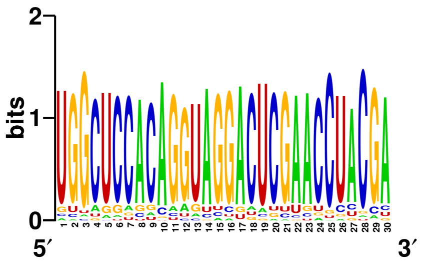

Adult female, library 1:

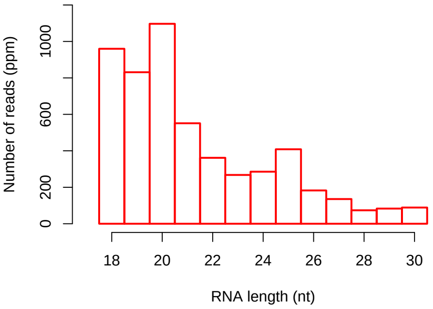

18-mers:

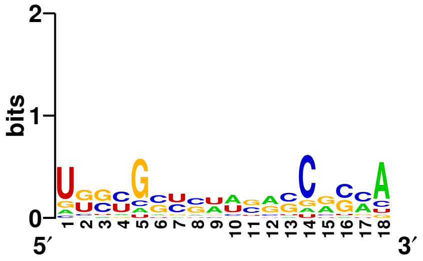

19-mers:

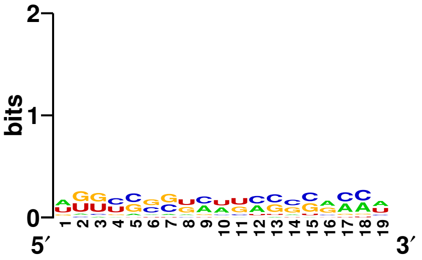

20-mers:

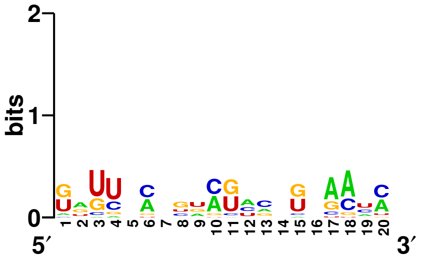

21-mers:

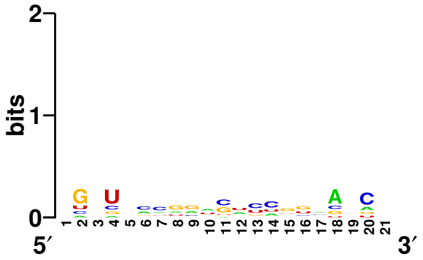

22-mers:

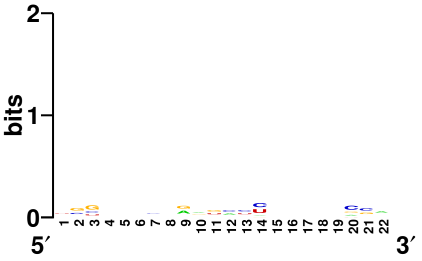

23-mers:

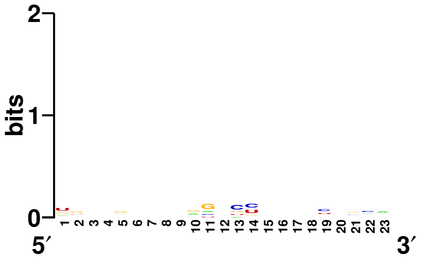

24-mers:

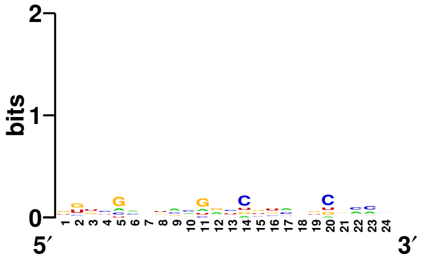

25-mers:

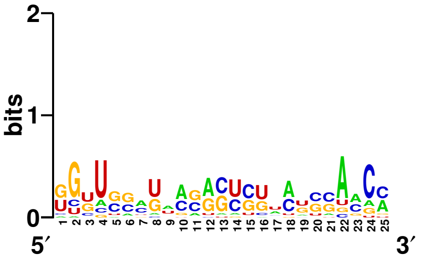

26-mers:

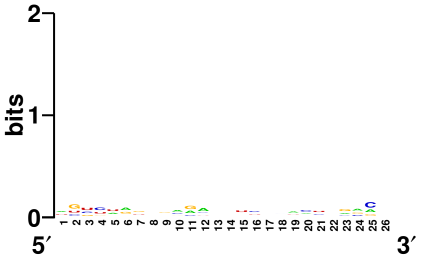

27-mers:

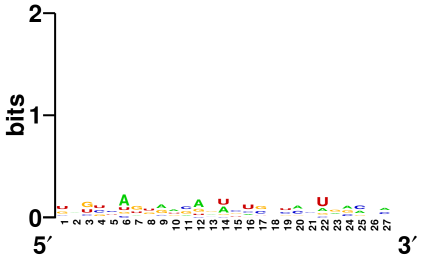

28-mers:

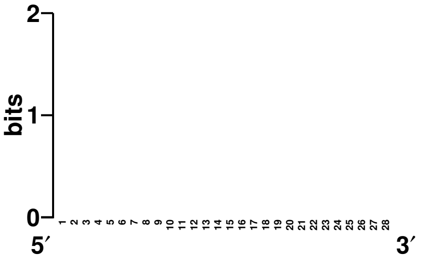

29-mers:

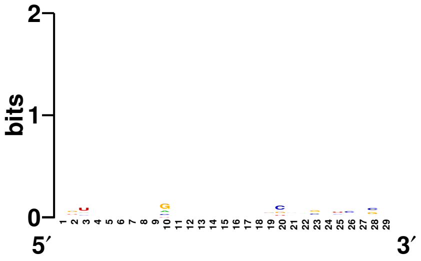

30-mers:

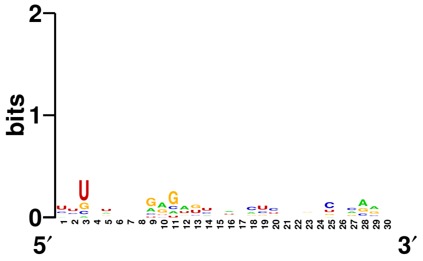

Adult male, library 1:

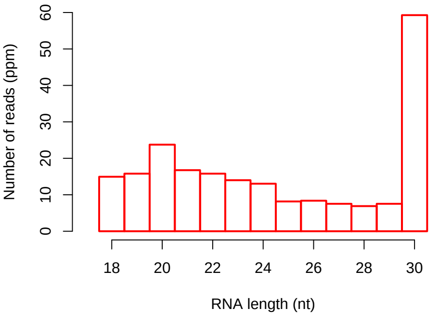

18-mers:

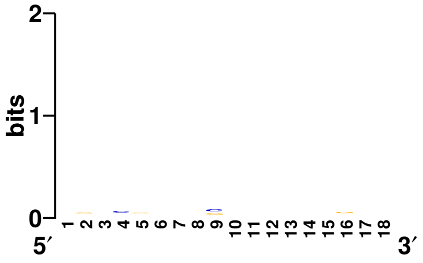

19-mers:

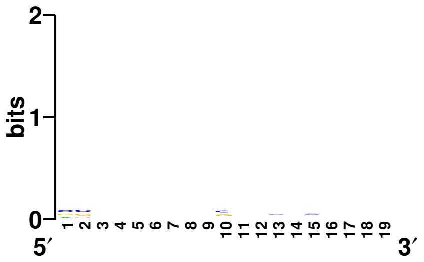

20-mers:

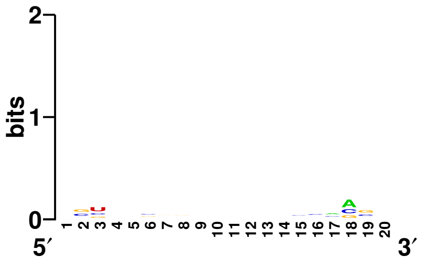

21-mers:

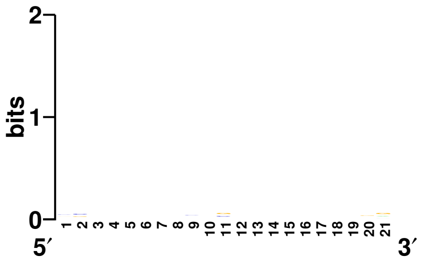

22-mers:

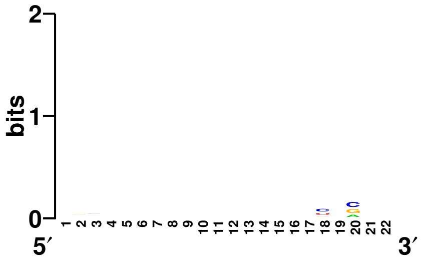

23-mers:

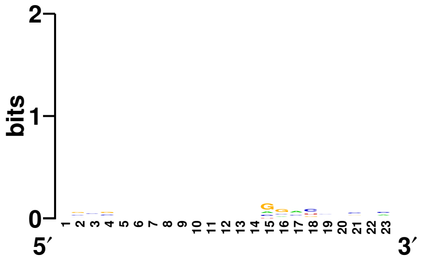

24-mers:

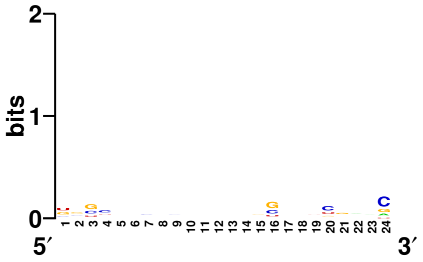

25-mers:

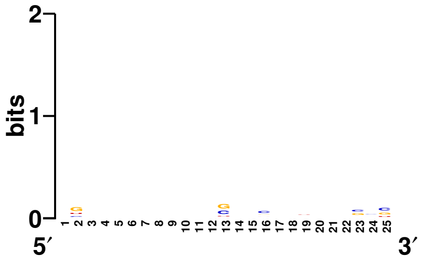

26-mers:

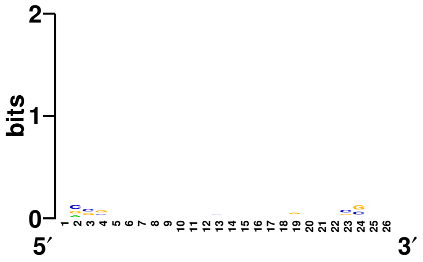

27-mers:

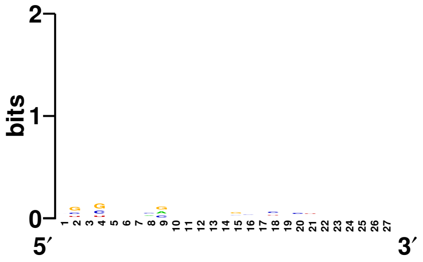

28-mers:

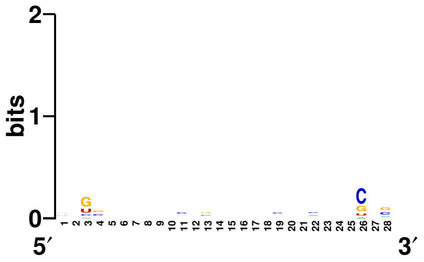

29-mers:

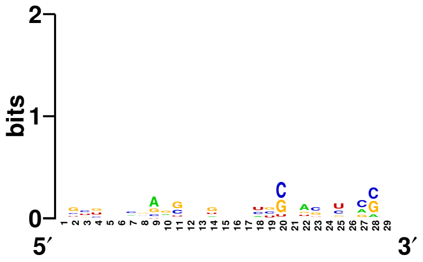

30-mers:

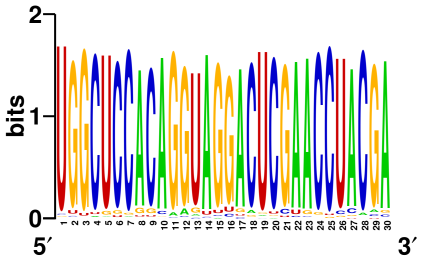

7.2 Libraries #2 (3´ modified, 5´ monophosphorylated small RNAs)

Embryo 8h, library 2:

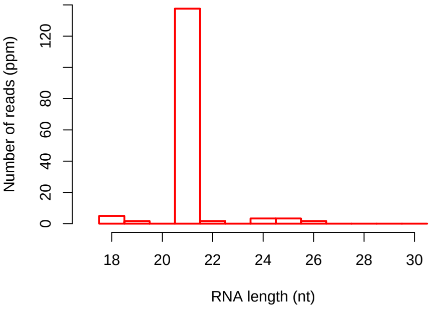

18-mers:

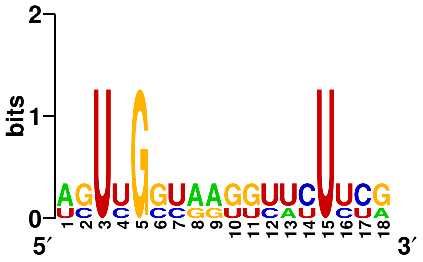

19-mers:

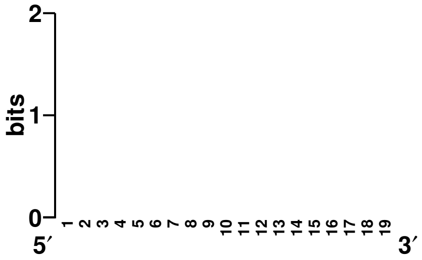

20-mers:

(no read)

21-mers:

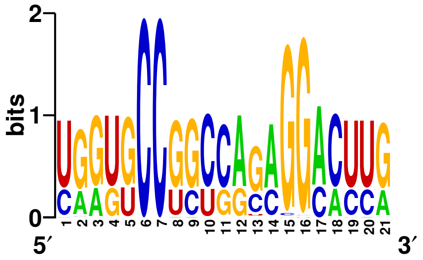

22-mers:

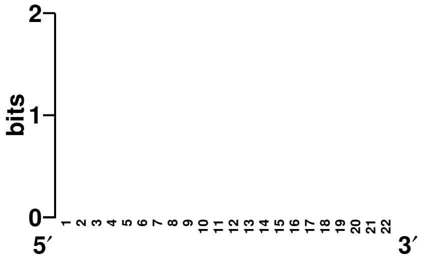

23-mers:

(no read)

24-mers:

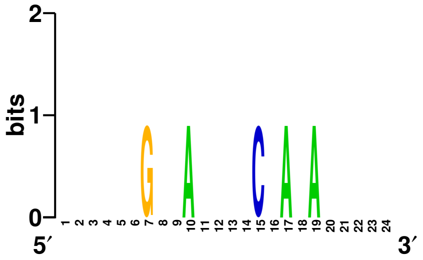

25-mers:

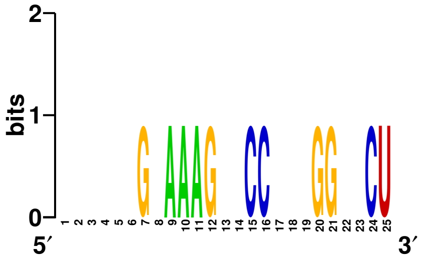

26-mers:

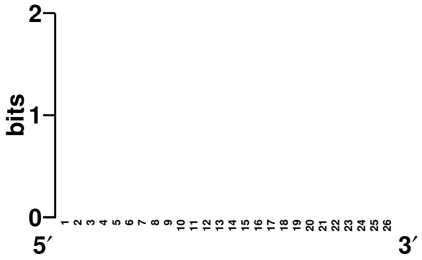

27-mers:

(no read)

28-mers:

(no read)

29-mers:

(no read)

30-mers:

(no read)

Embryo 15h, library 2:

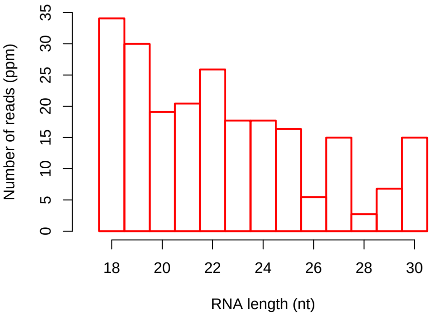

18-mers:

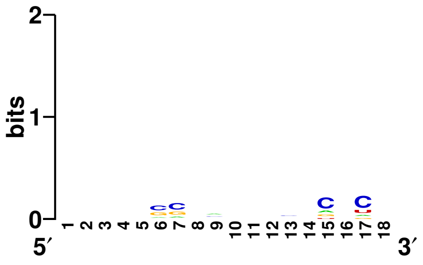

19-mers:

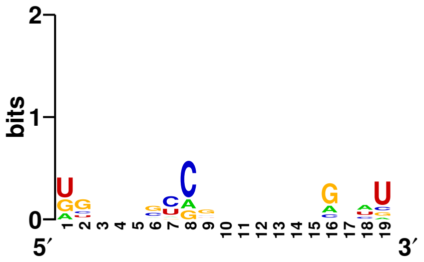

20-mers:

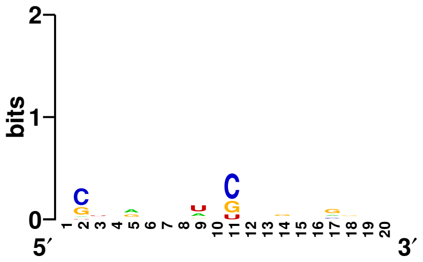

21-mers:

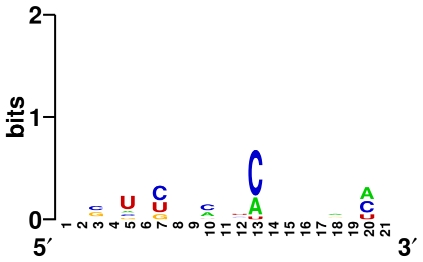

22-mers:

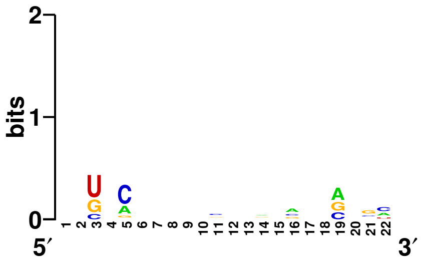

23-mers:

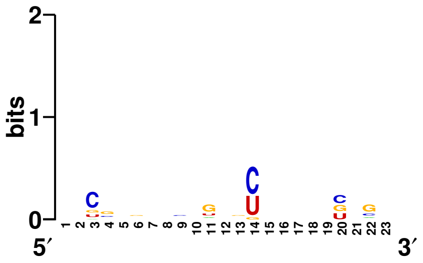

24-mers:

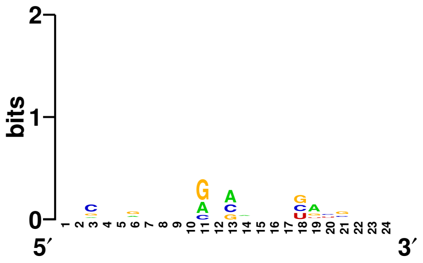

25-mers:

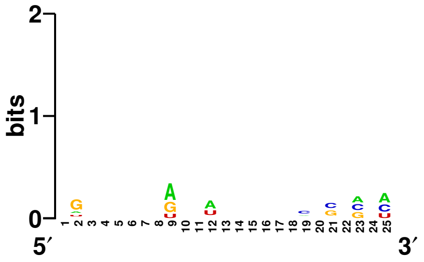

26-mers:

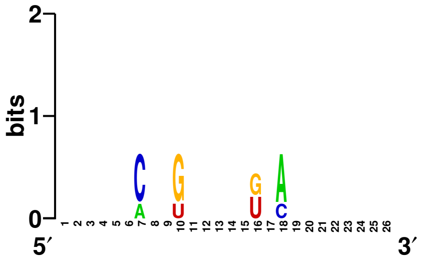

27-mers:

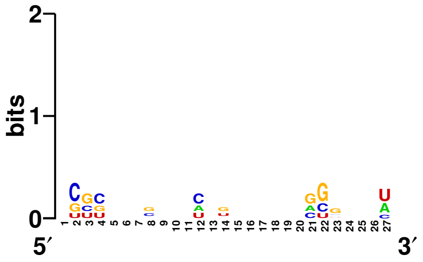

28-mers:

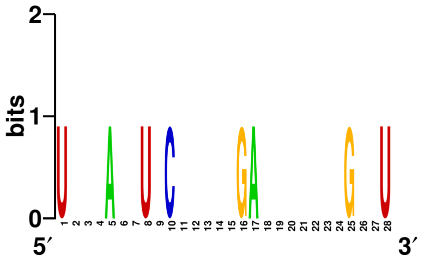

29-mers:

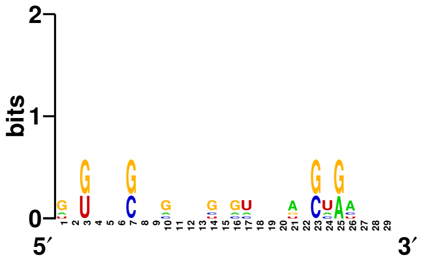

30-mers:

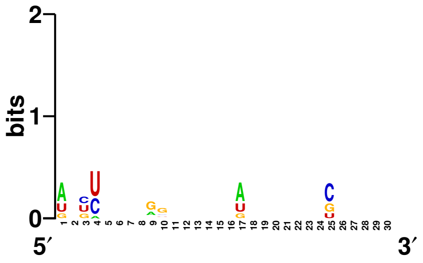

Embryo 36h, library 2:

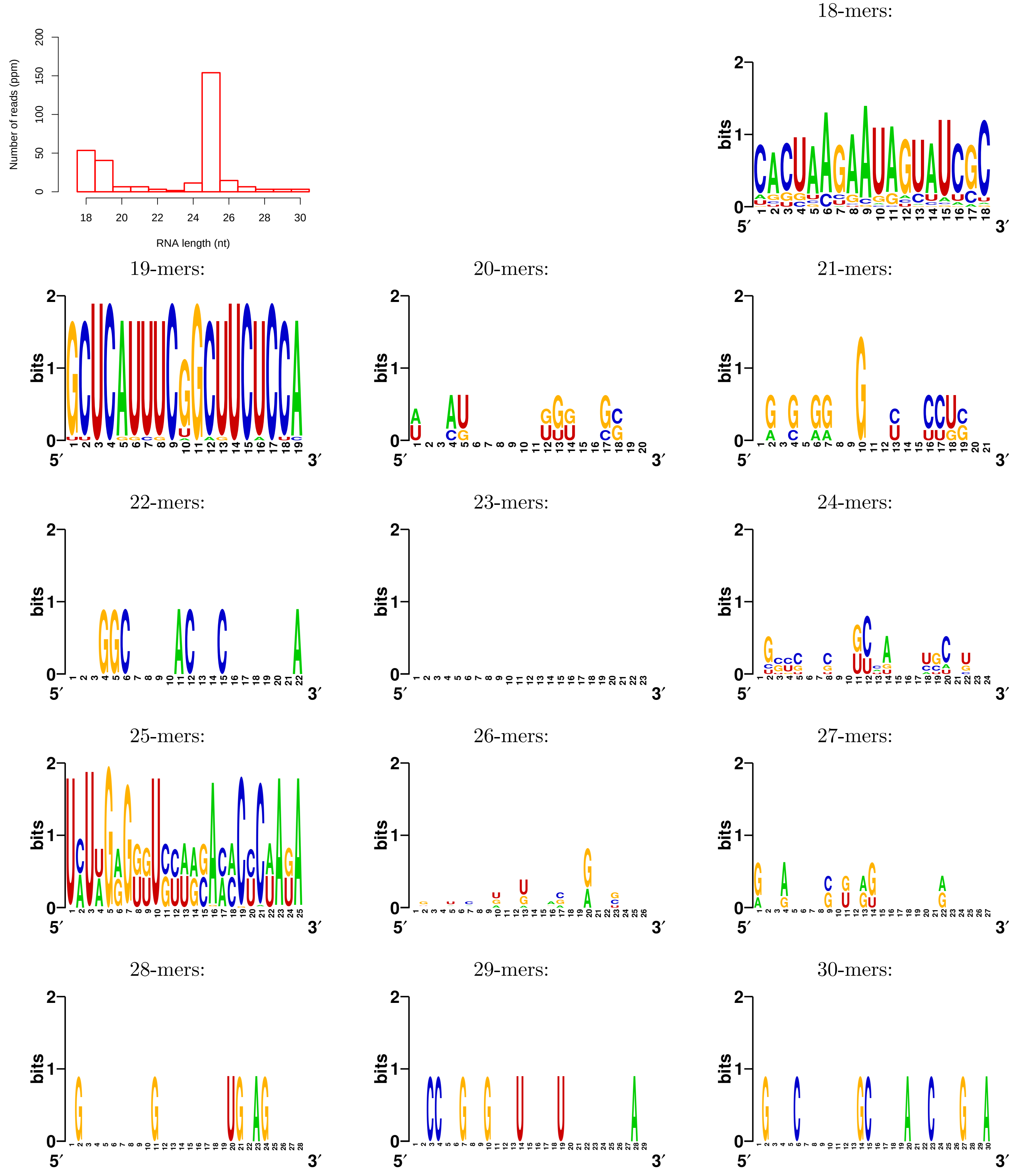

Embryo 60h, library 2:

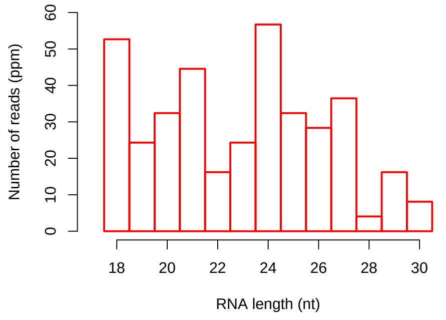

18-mers:

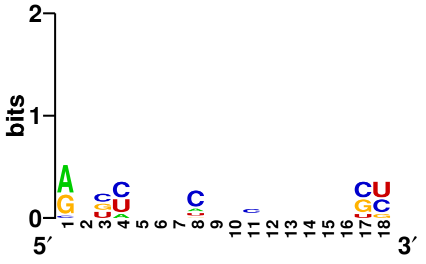

19-mers:

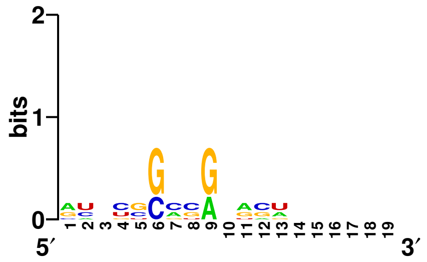

20-mers:

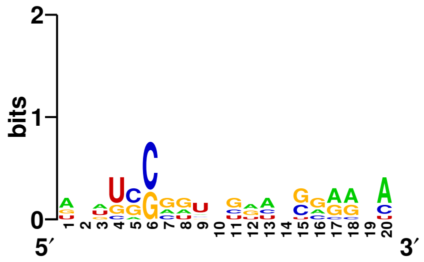

21-mers:

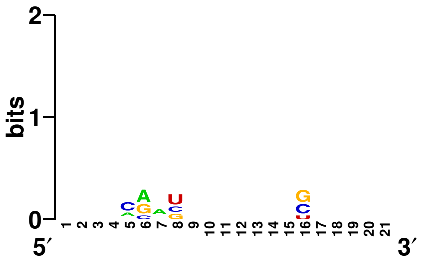

22-mers:

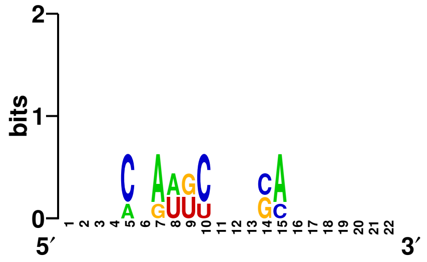

23-mers:

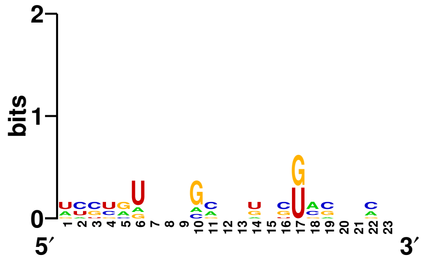

24-mers:

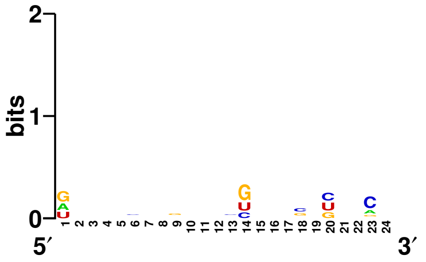

25-mers:

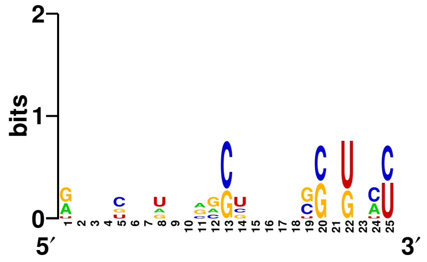

26-mers:

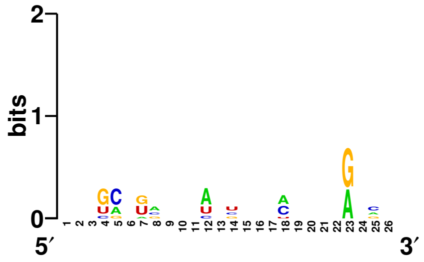

27-mers:

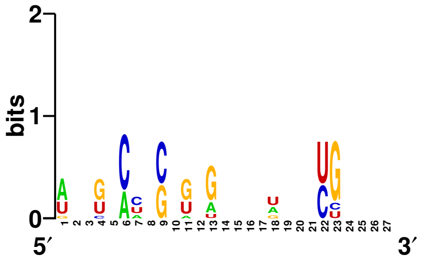

28-mers:

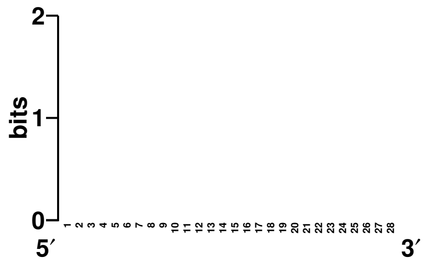

29-mers:

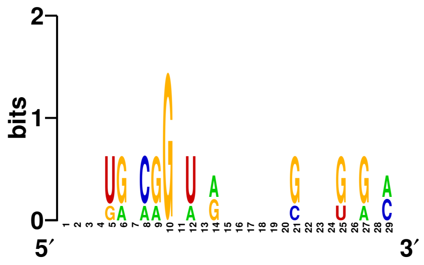

30-mers:

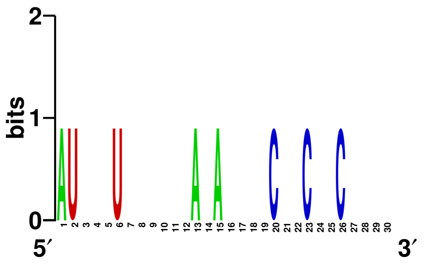

Adult female, library 2:

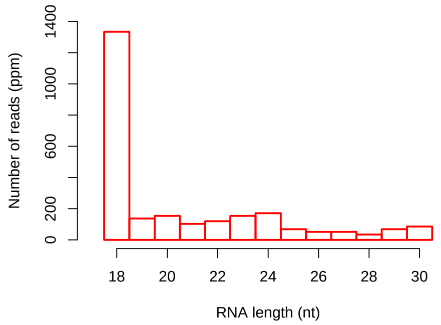

18-mers:

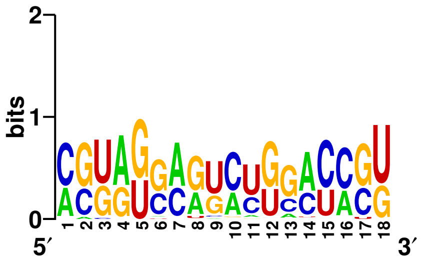

19-mers:

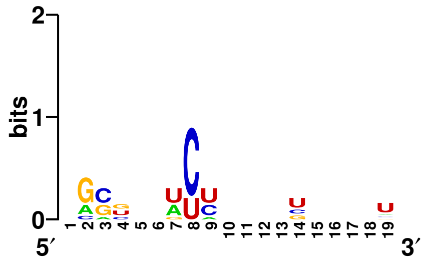

20-mers:

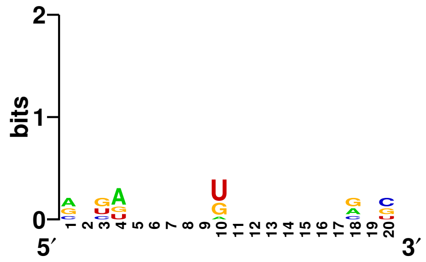

21-mers:

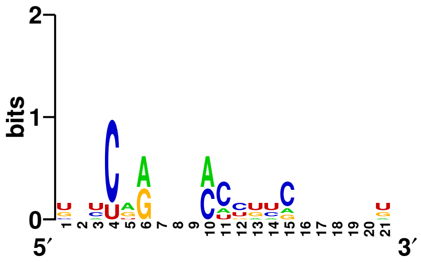

22-mers:

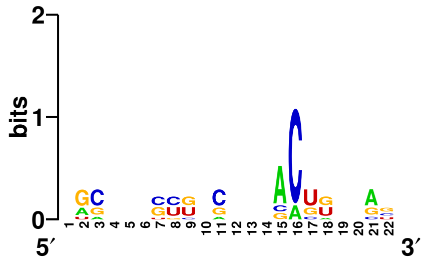

23-mers:

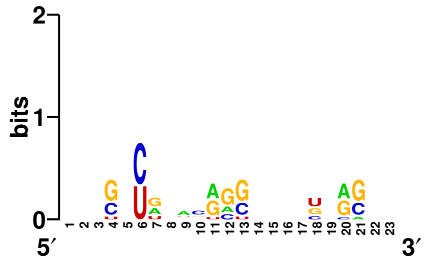

24-mers:

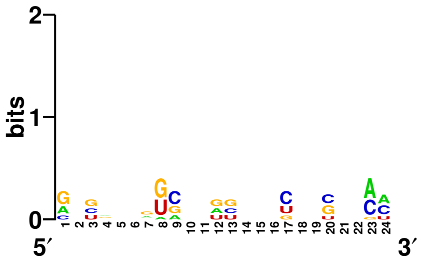

25-mers:

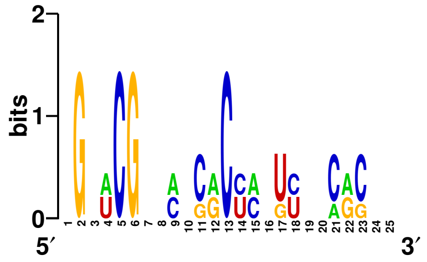

26-mers:

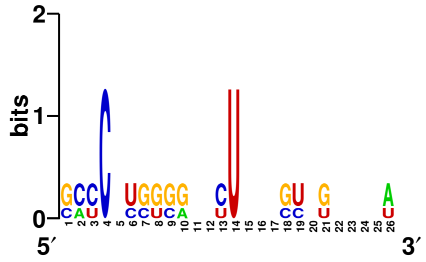

27-mers:

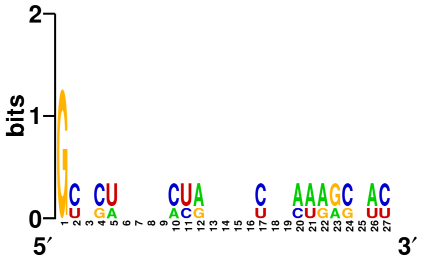

28-mers:

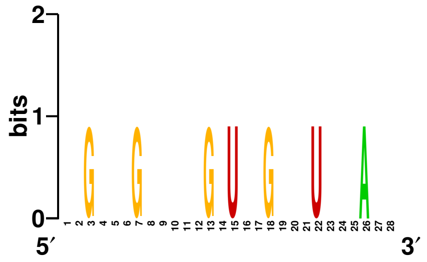

29-mers:

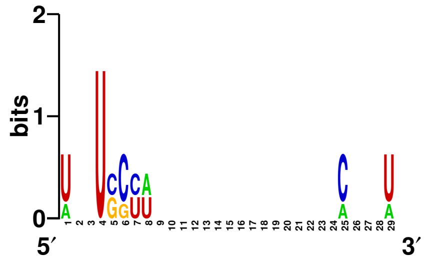

30-mers:

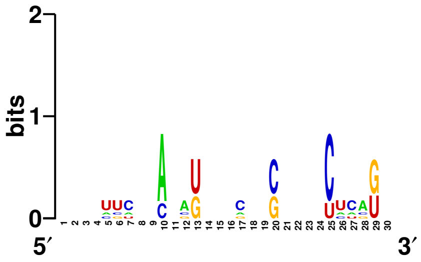

Adult male, library 2:

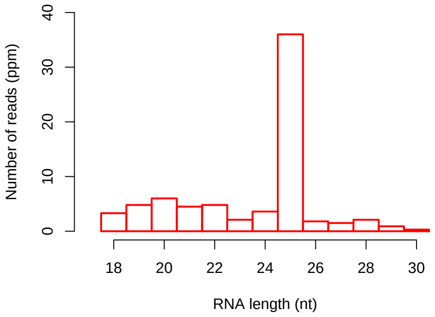

18-mers:

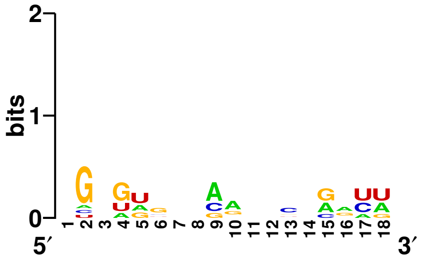

19-mers:

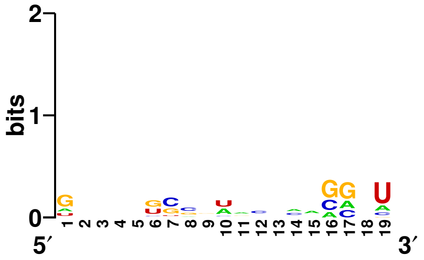

20-mers:

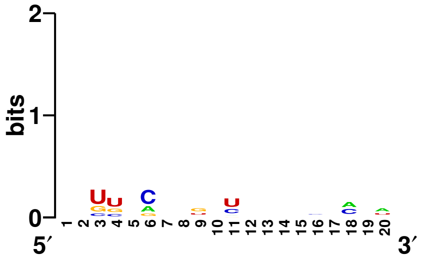

21-mers:

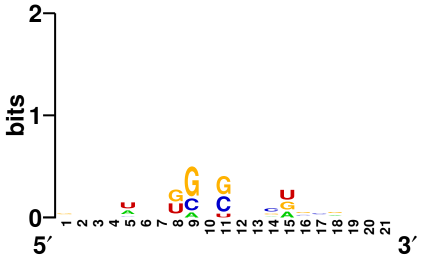

22-mers:

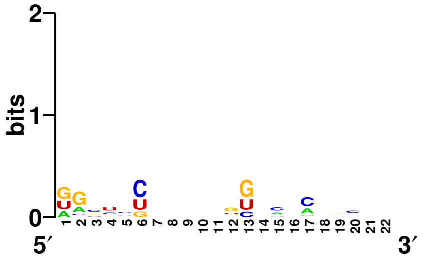

23-mers:

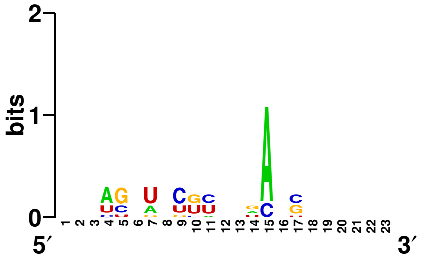

24-mers:

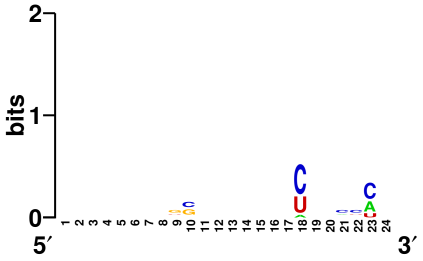

25-mers:

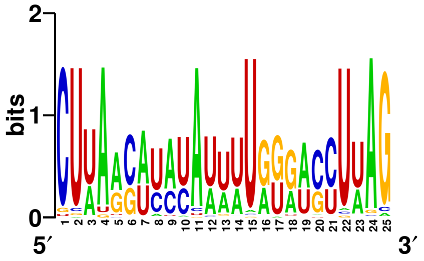

26-mers:

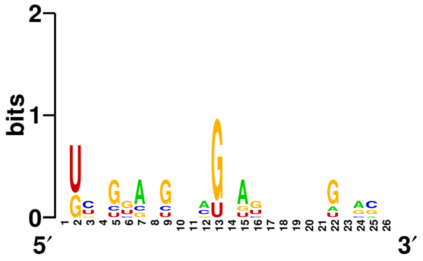

27-mers:

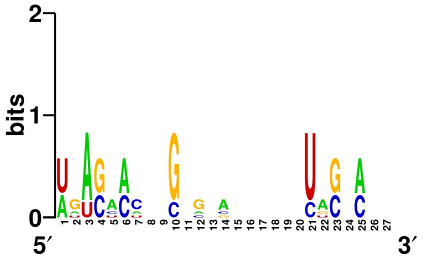

28-mers:

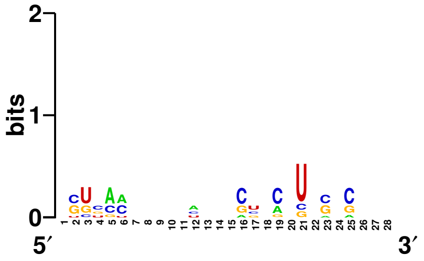

29-mers:

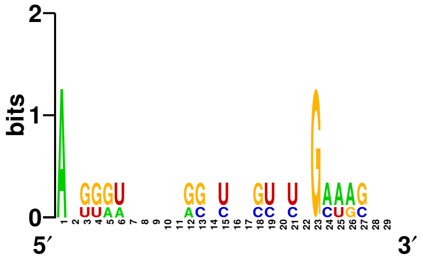

30-mers:

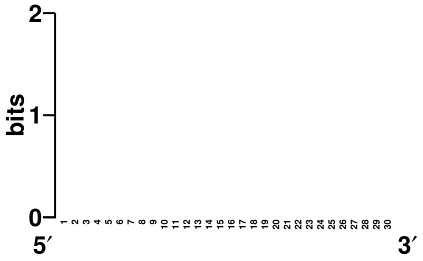

7.3 Libraries #3 (total 5´ hydroxyl or polyphosphorylated small RNAs)

Embryo 8h, library 3:

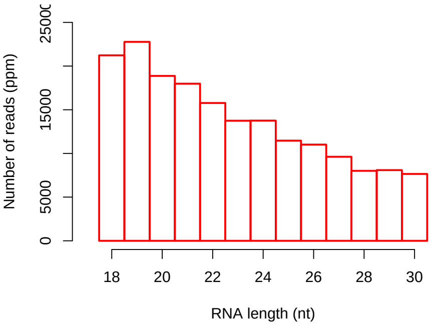

18-mers:

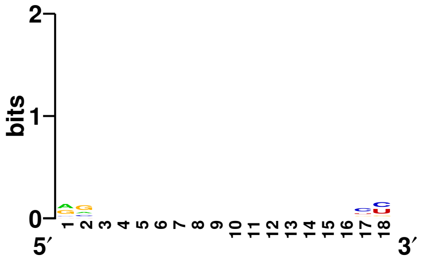

19-mers:

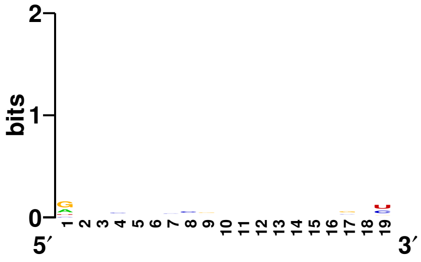

20-mers:

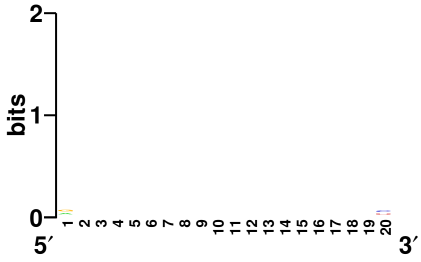

21-mers:

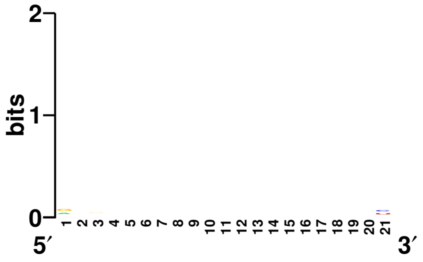

22-mers:

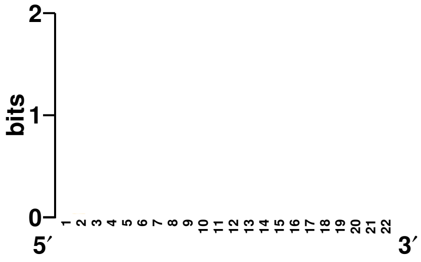

23-mers:

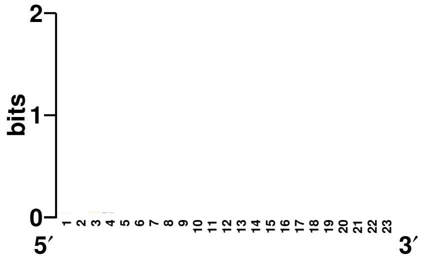

24-mers:

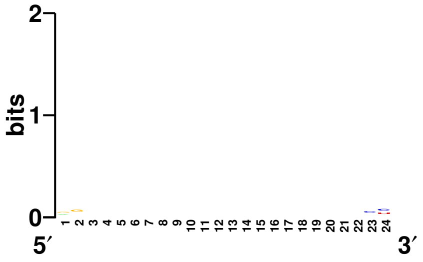

25-mers:

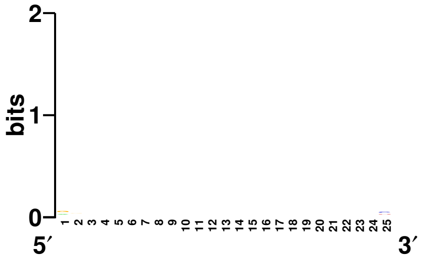

26-mers:

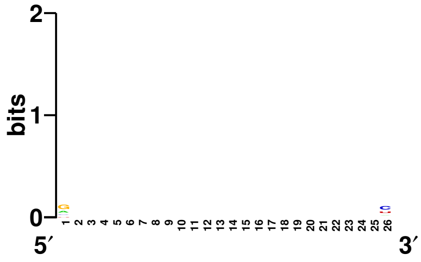

27-mers:

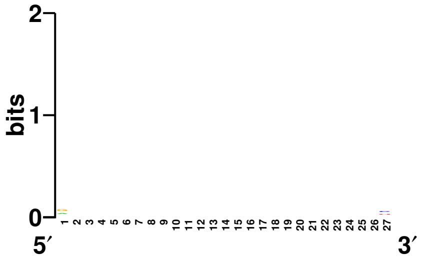

28-mers:

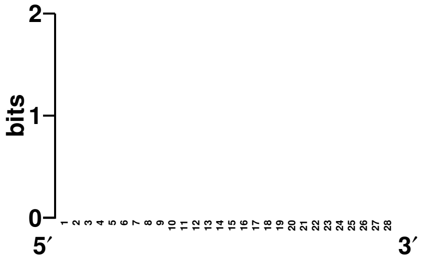

29-mers:

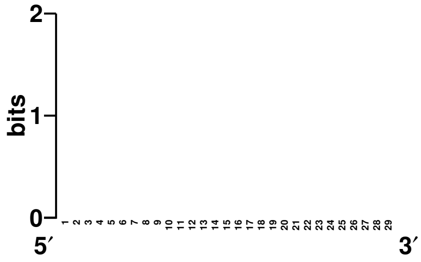

30-mers:

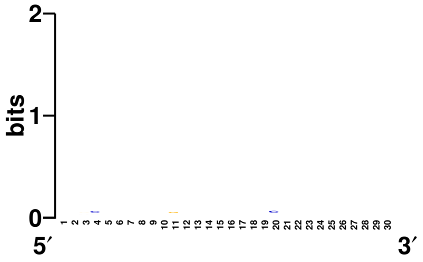

Embryo 15h, library 3:

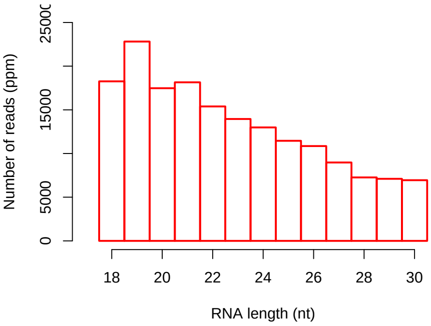

18-mers:

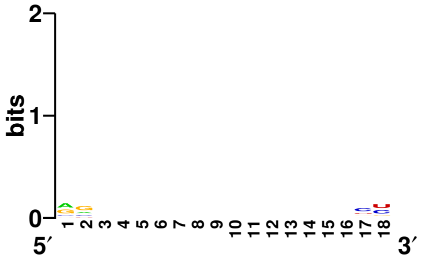

19-mers:

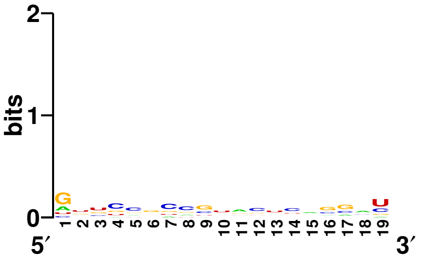

20-mers:

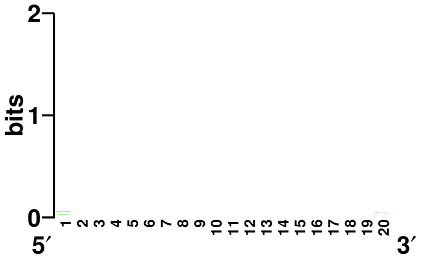

21-mers:

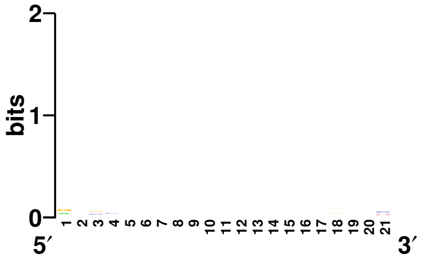

22-mers:

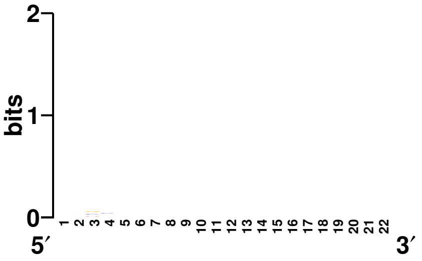

23-mers:

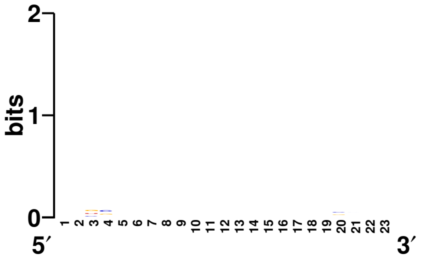

24-mers:

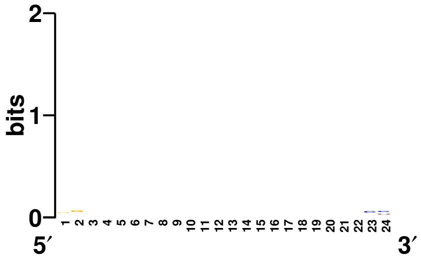

25-mers:

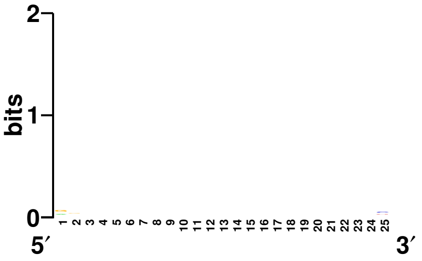

26-mers:

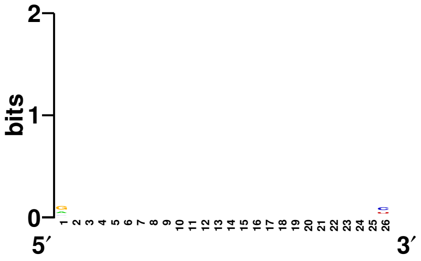

27-mers:

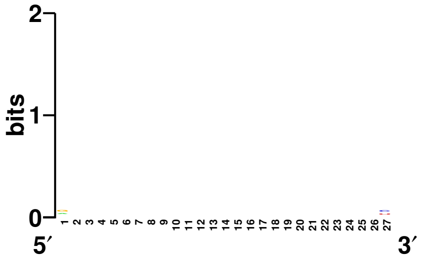

28-mers:

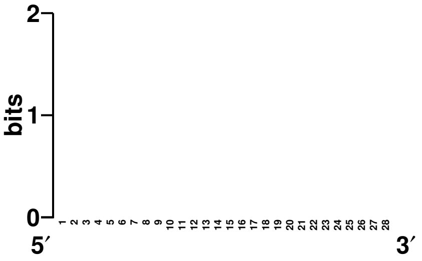

29-mers:

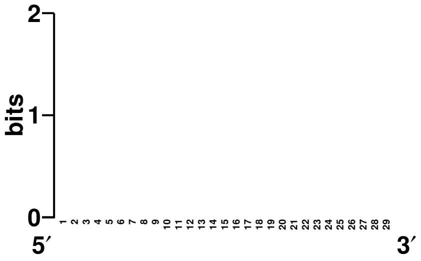

30-mers:

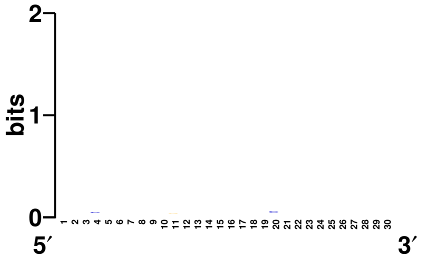

Embryo 36h, library 3:

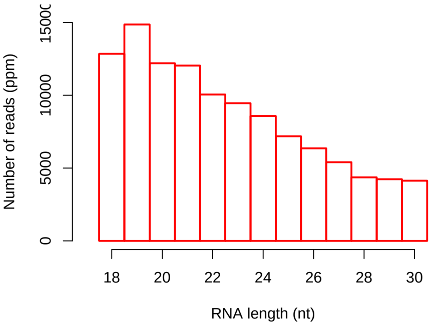

18-mers:

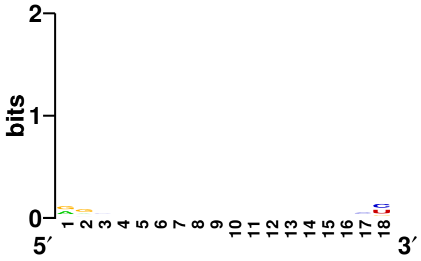

19-mers:

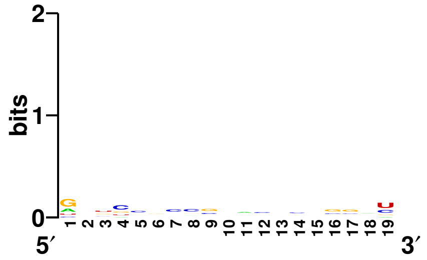

20-mers:

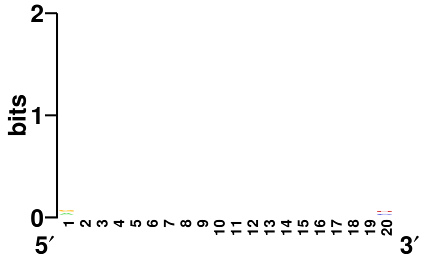

21-mers:

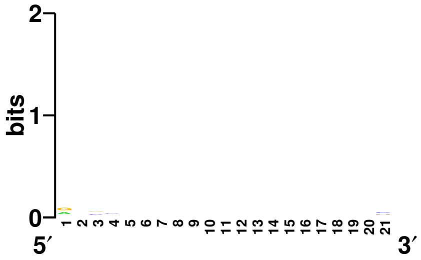

22-mers:

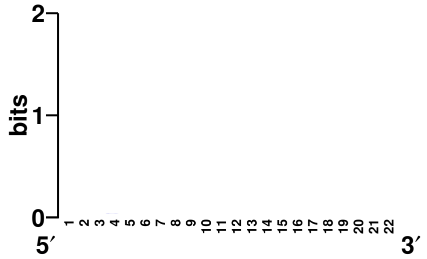

23-mers:

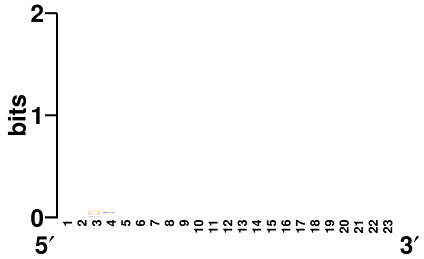

24-mers:

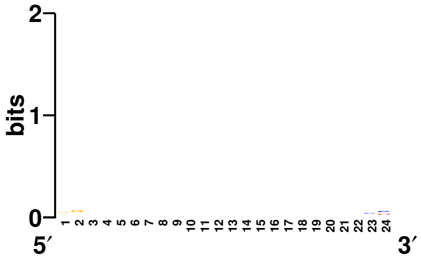

25-mers:

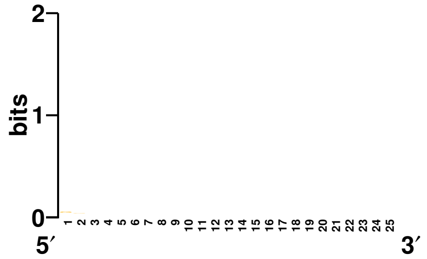

26-mers:

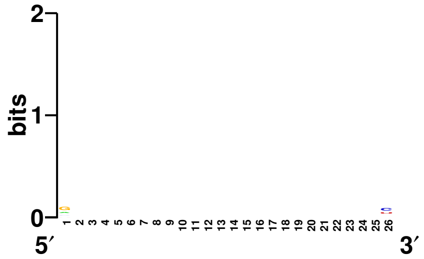

27-mers:

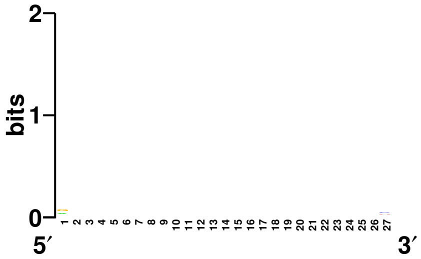

28-mers:

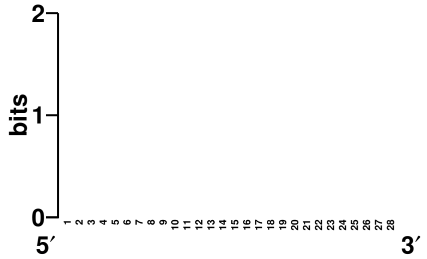

29-mers:

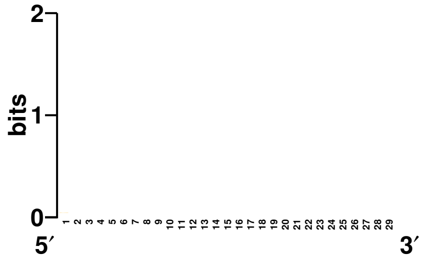

30-mers:

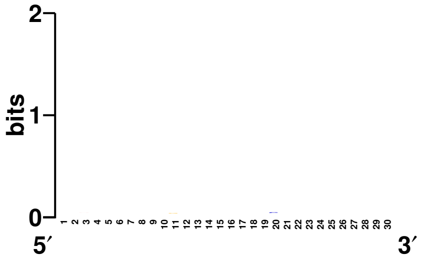

Embryo 60h, library 3:

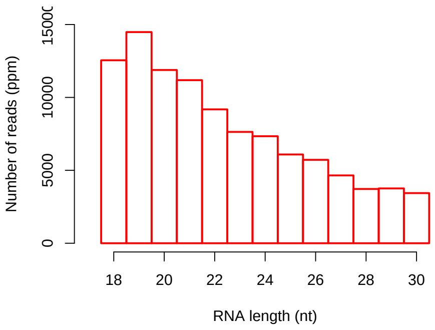

18-mers:

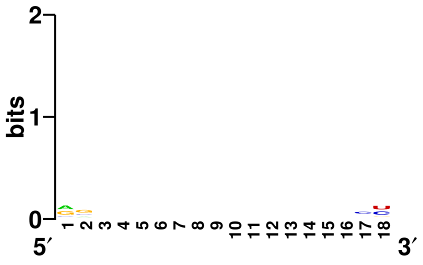

19-mers:

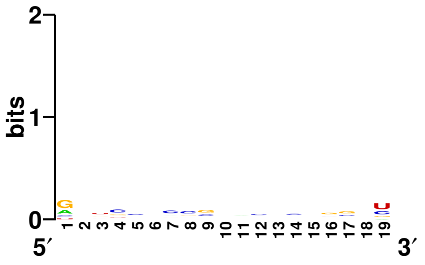

20-mers:

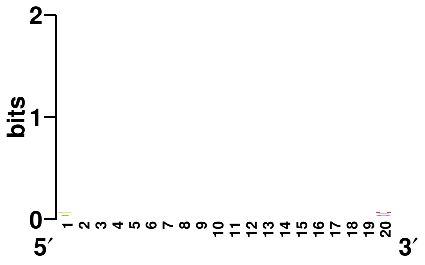

21-mers:

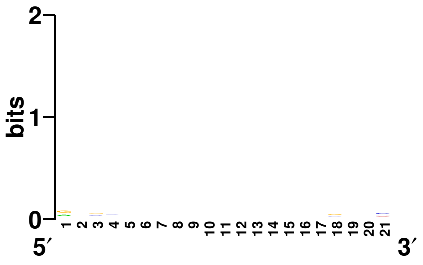

22-mers:

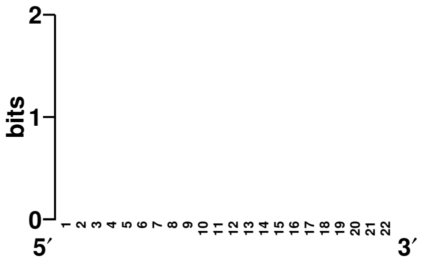

23-mers:

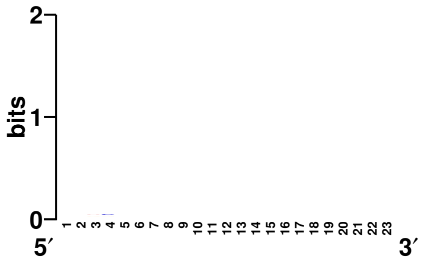

24-mers:

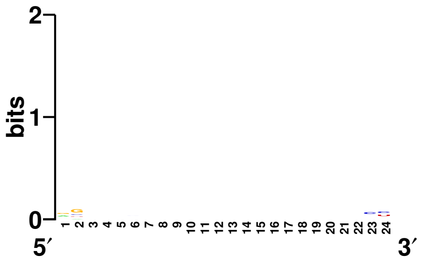

25-mers:

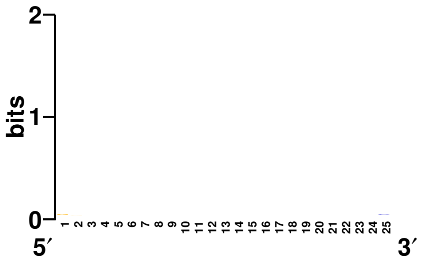

26-mers:

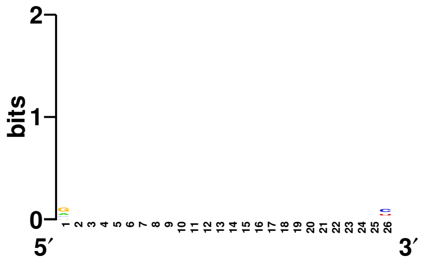

27-mers:

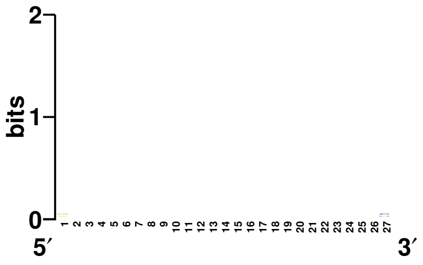

28-mers:

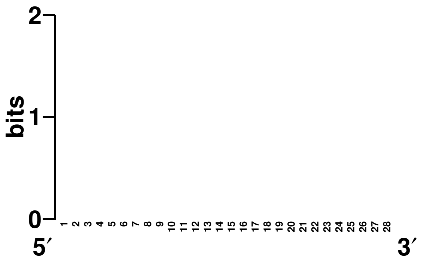

29-mers:

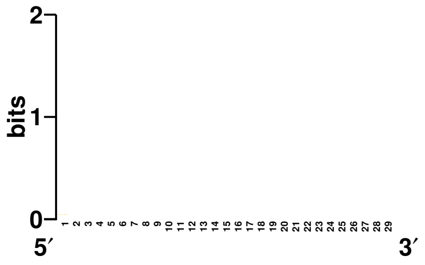

30-mers:

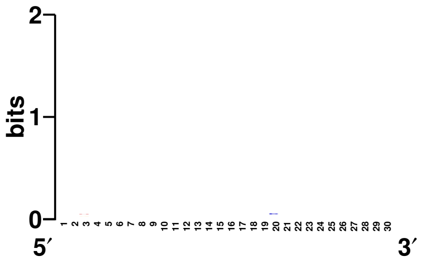

Adult female, library 3:

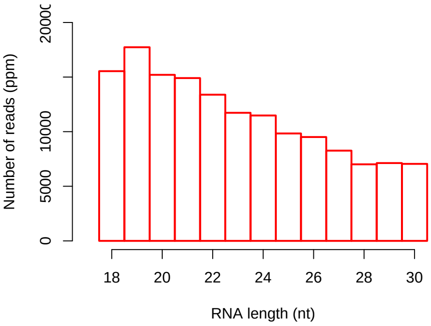

18-mers:

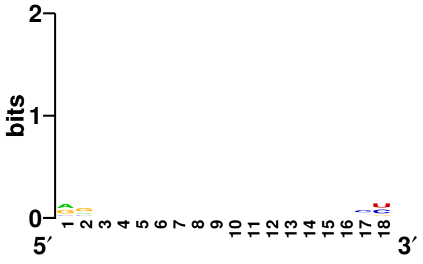

19-mers:

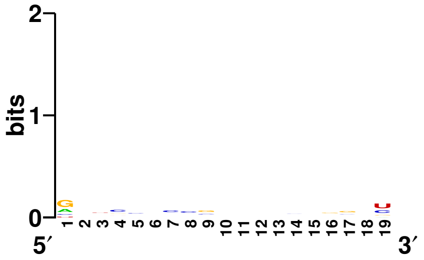

20-mers:

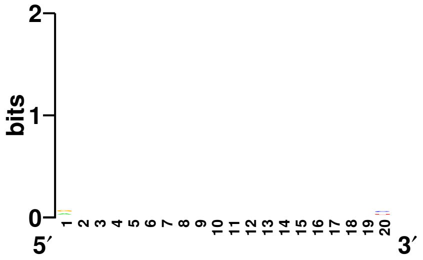

21-mers:

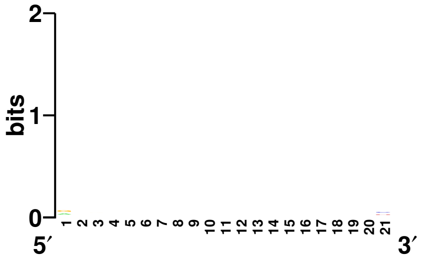

22-mers:

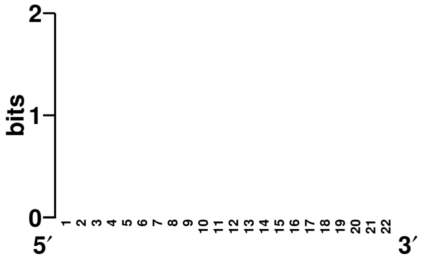

23-mers:

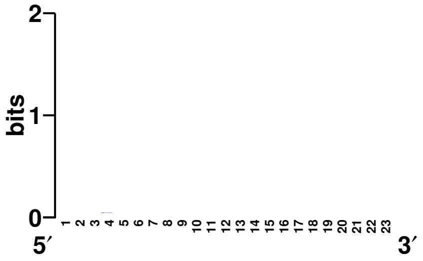

24-mers:

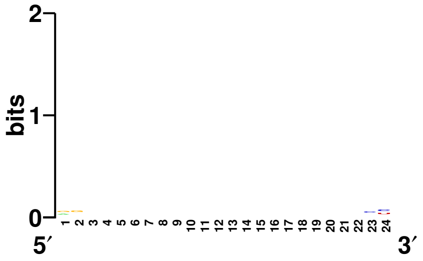

25-mers:

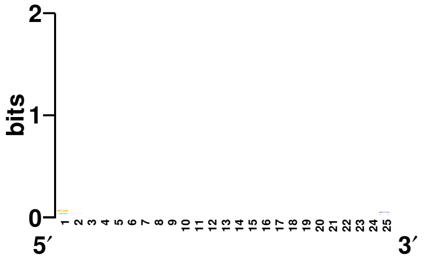

26-mers:

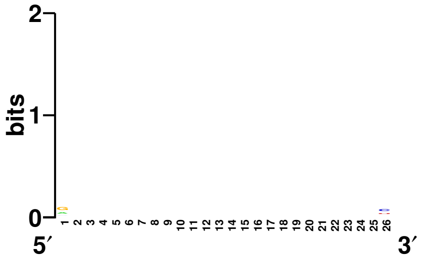

27-mers:

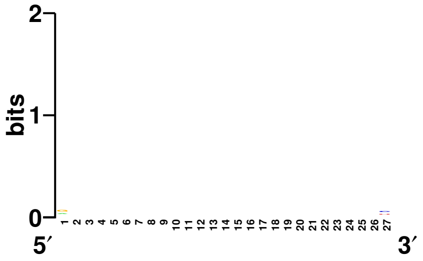

28-mers:

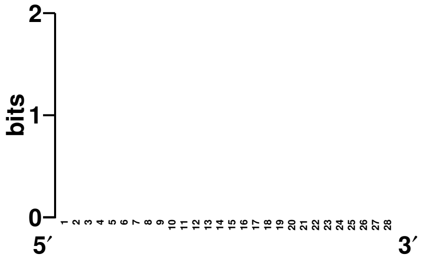

29-mers:

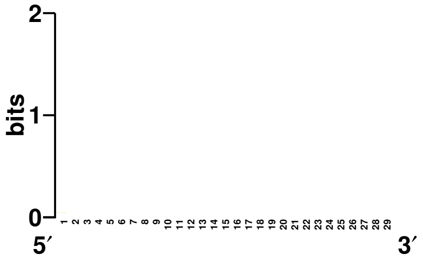

30-mers:

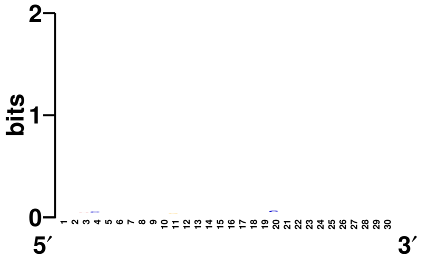

Adult male, library 3:

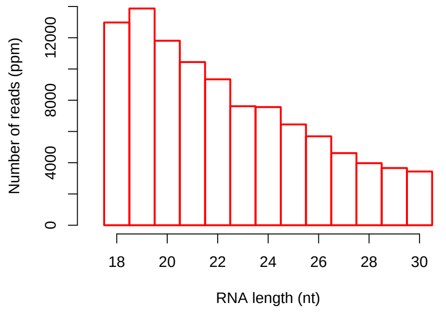

18-mers:

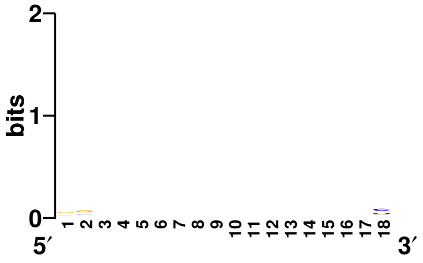

19-mers:

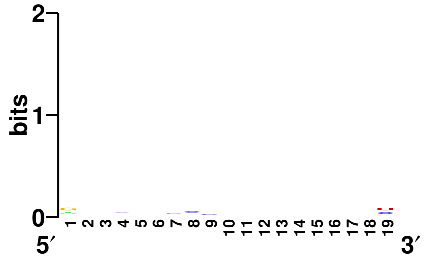

20-mers:

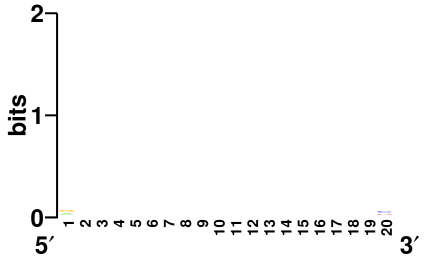

21-mers:

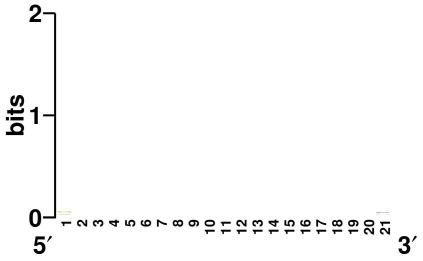

22-mers:

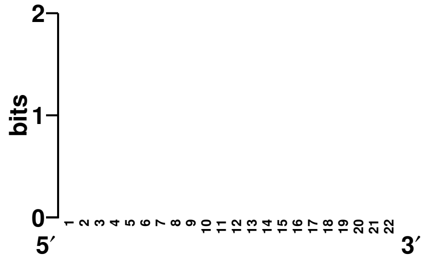

23-mers:

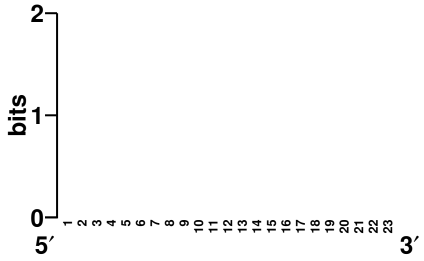

24-mers:

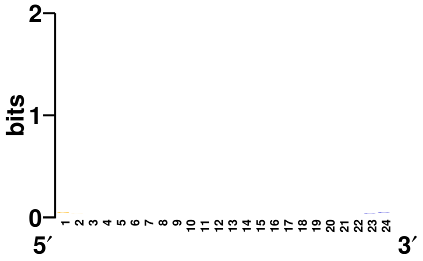

25-mers:

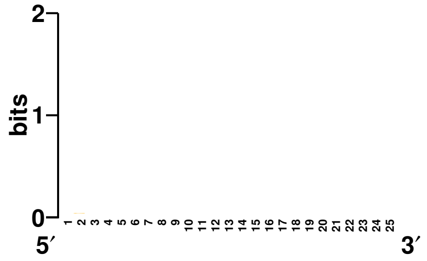

26-mers:

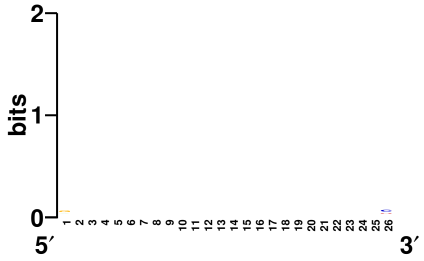

27-mers:

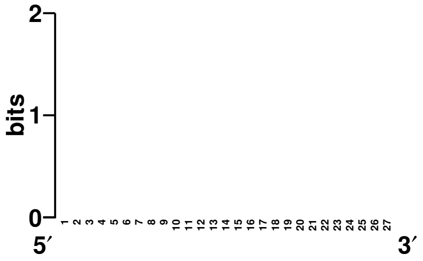

28-mers:

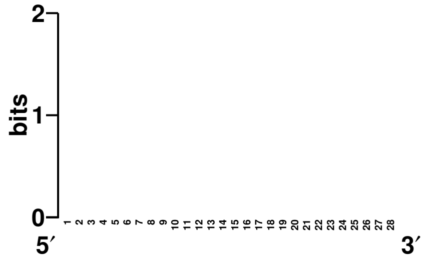

29-mers:

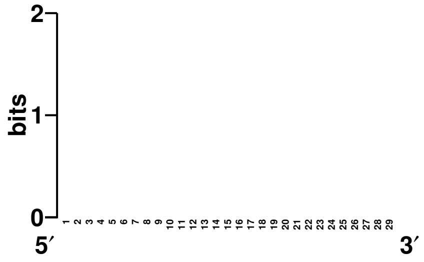

30-mers:

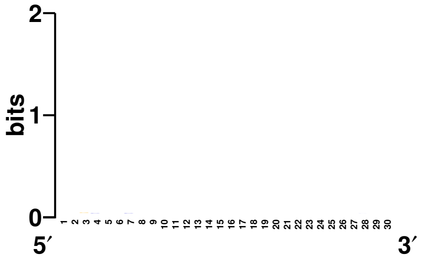

7.4 Libraries #4 (3´ modified, 5´ hydroxyl or polyphosphorylated small RNAs)

Embryo 8h, library 4:

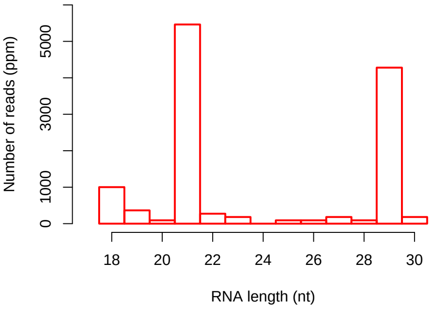

18-mers:

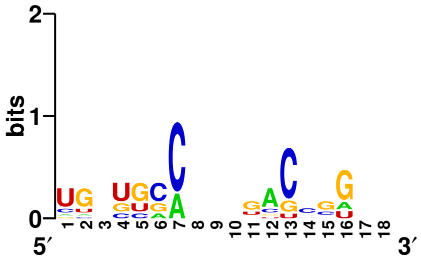

19-mers:

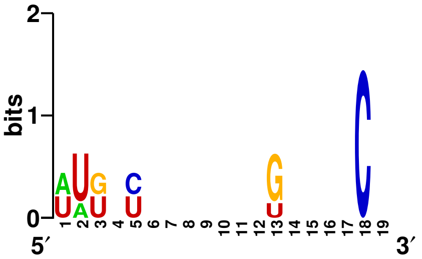

20-mers:

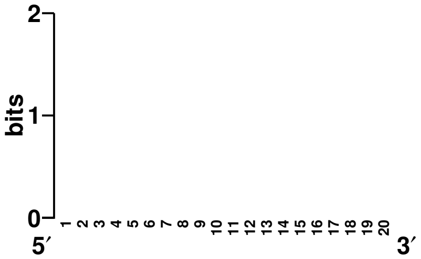

21-mers:

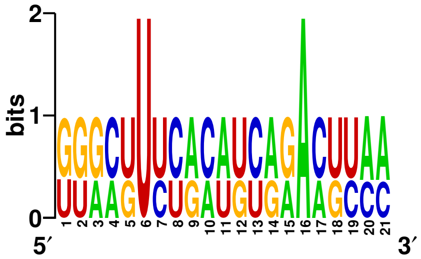

22-mers:

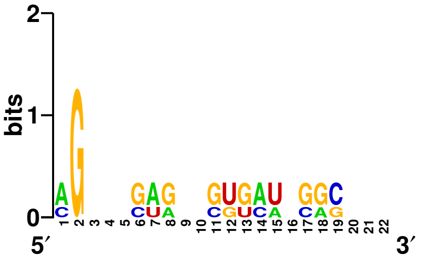

23-mers:

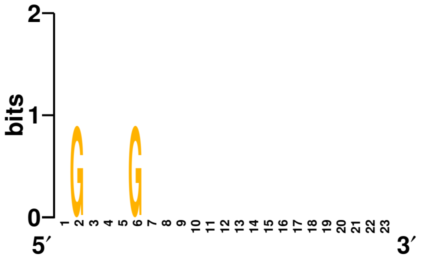

24-mers:

(no read)

25-mers:

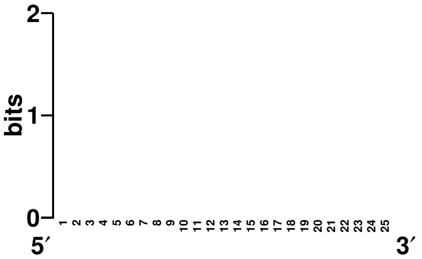

26-mers:

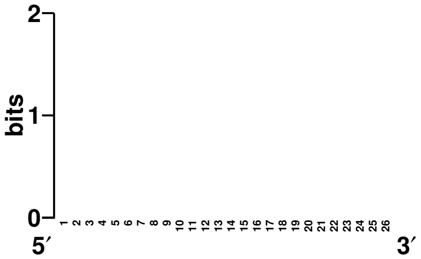

27-mers:

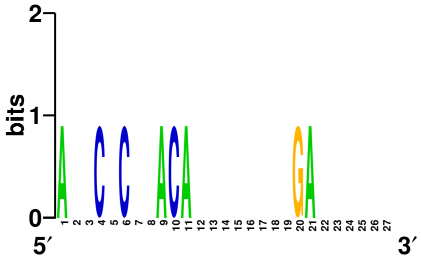

28-mers:

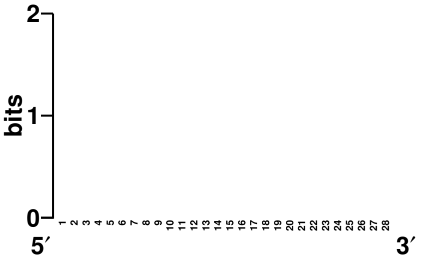

29-mers:

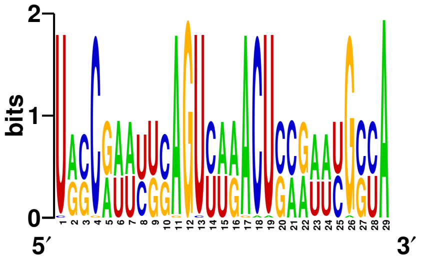

30-mers:

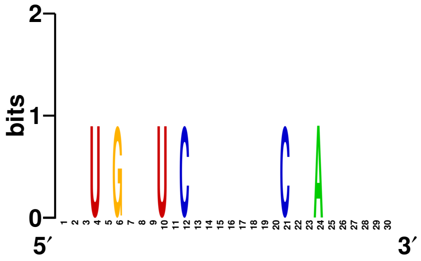

Embryo 15h, library 4:

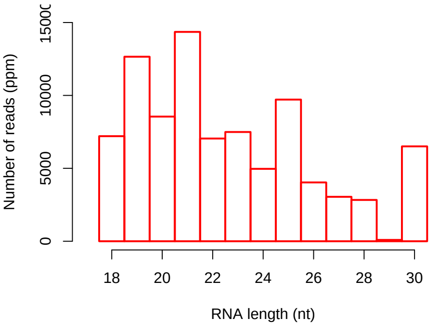

18-mers:

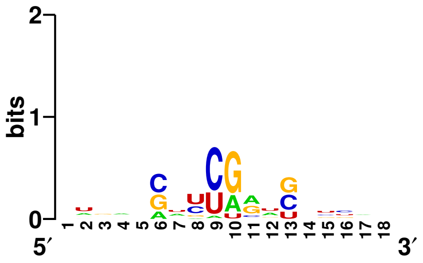

19-mers:

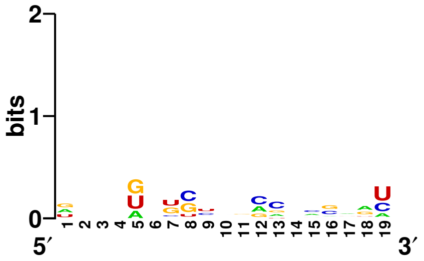

20-mers:

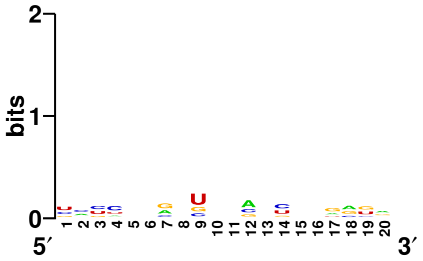

21-mers:

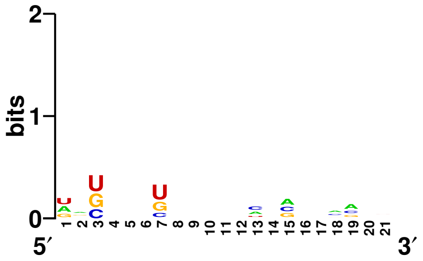

22-mers:

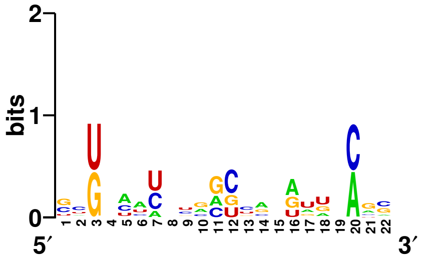

23-mers:

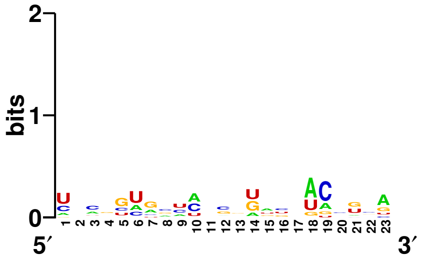

24-mers:

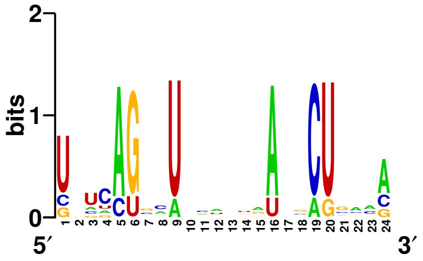

25-mers:

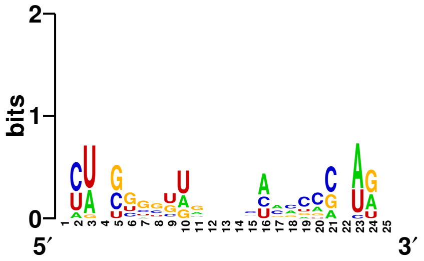

26-mers:

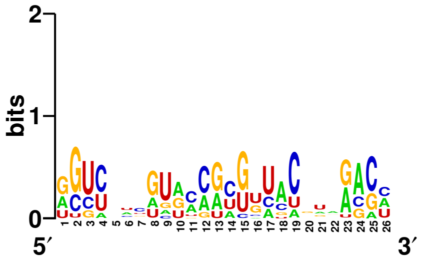

27-mers:

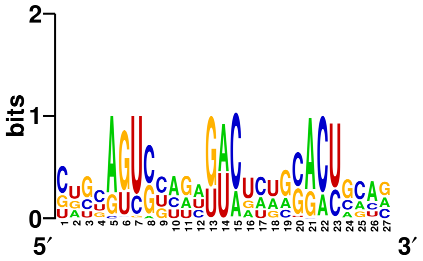

28-mers:

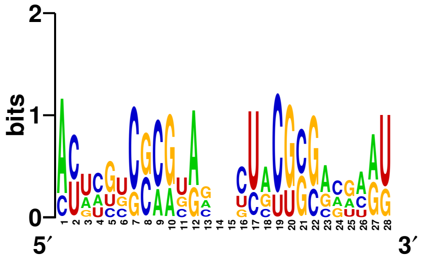

29-mers:

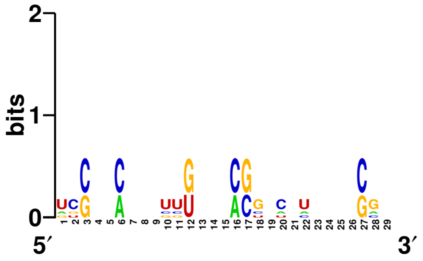

30-mers:

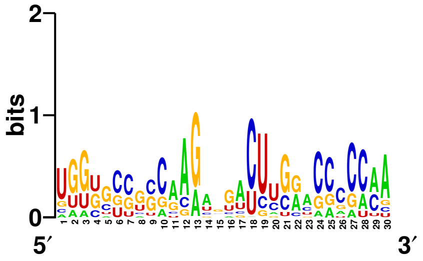

Embryo 36h, library 4:

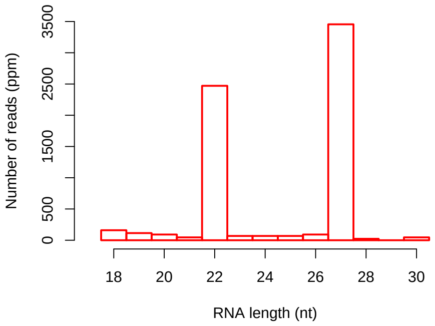

18-mers:

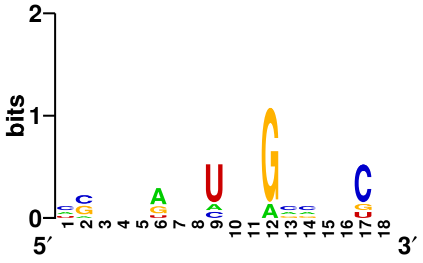

19-mers:

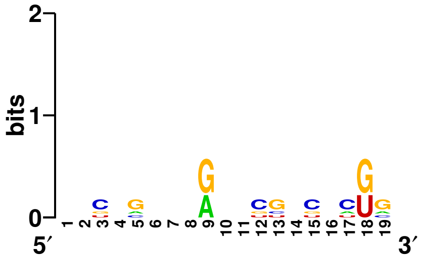

20-mers:

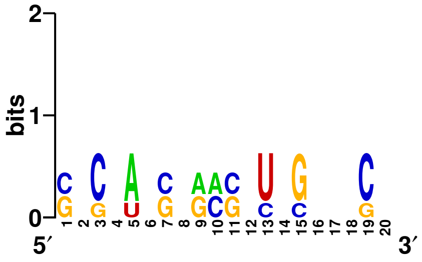

21-mers:

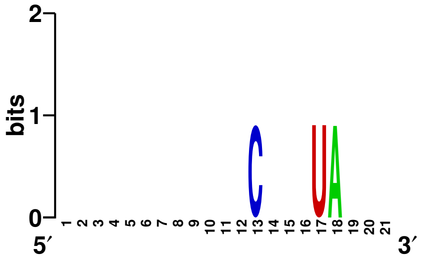

22-mers:

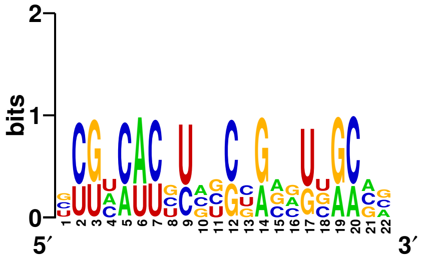

23-mers:

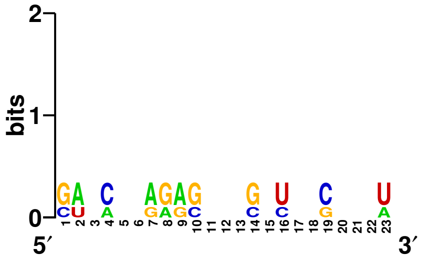

24-mers:

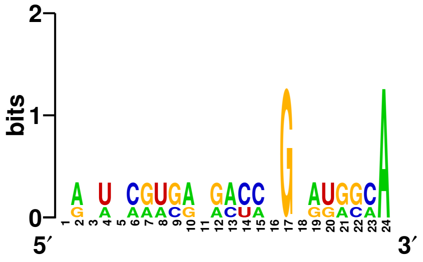

25-mers:

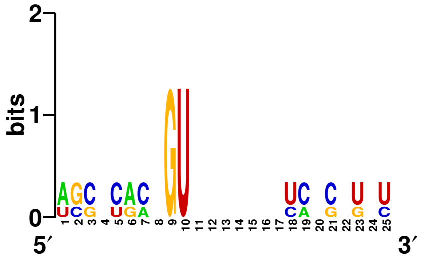

26-mers:

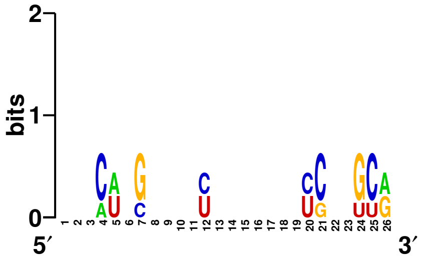

27-mers:

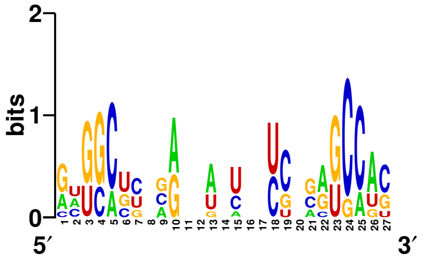

28-mers:

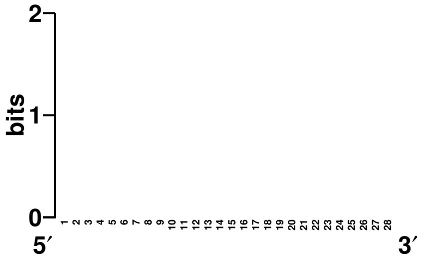

29-mers:

(no read)

30-mers:

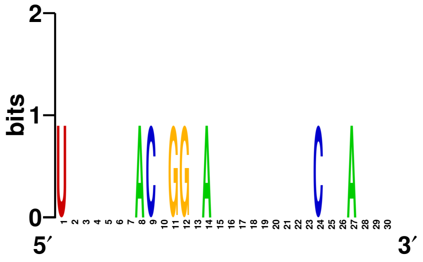

Embryo 60h, library 4:

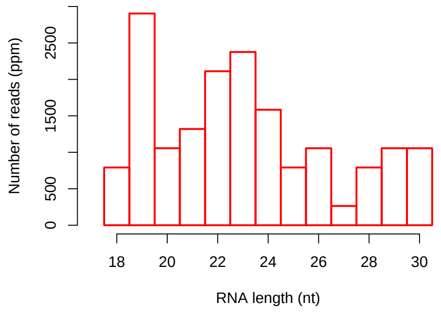

18-mers:

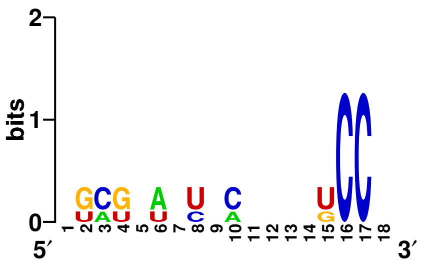

19-mers:

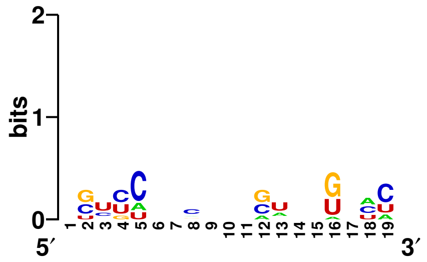

20-mers:

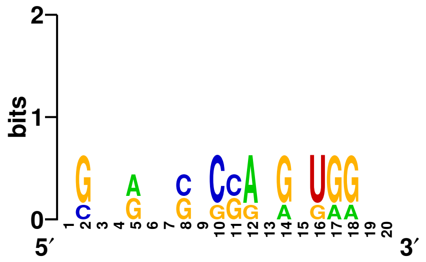

21-mers:

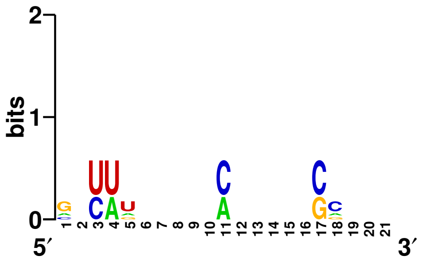

22-mers:

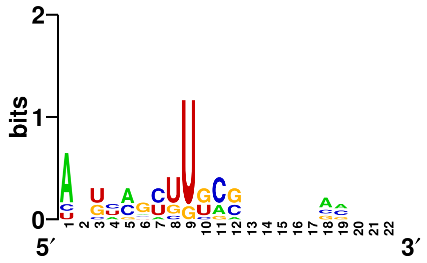

23-mers:

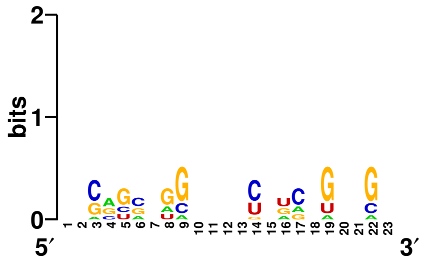

24-mers:

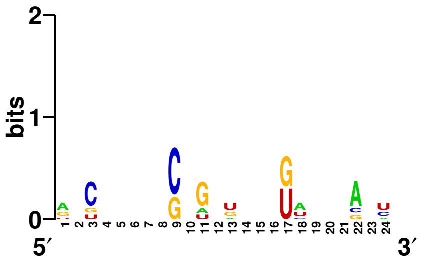

25-mers:

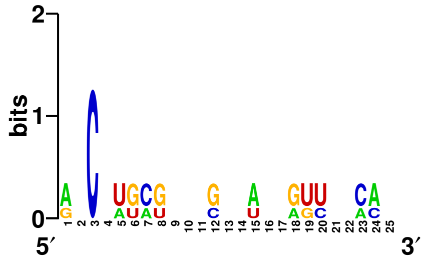

26-mers:

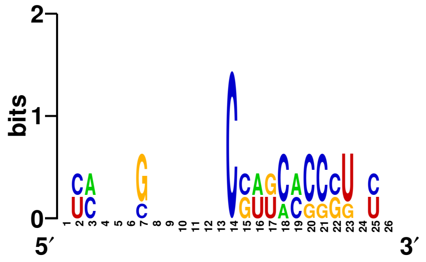

27-mers:

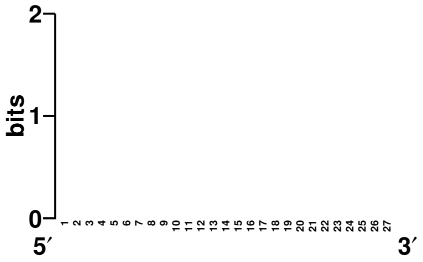

28-mers:

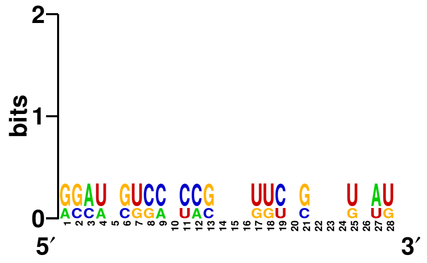

29-mers:

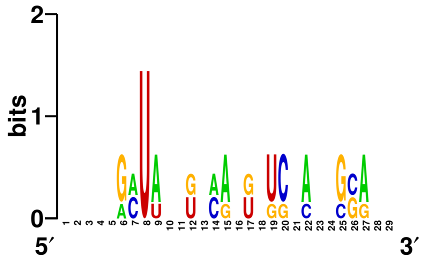

30-mers:

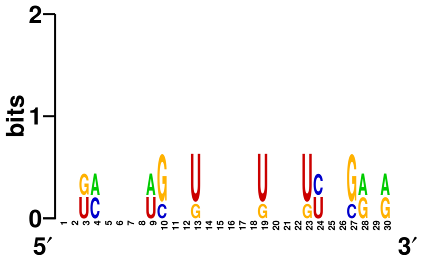

Adult female, library 4:

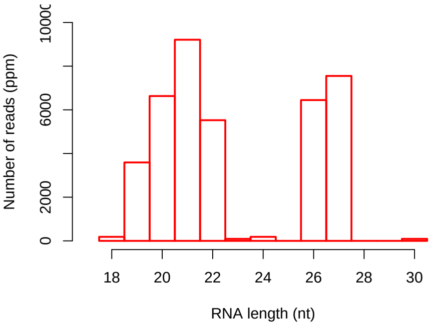

18-mers:

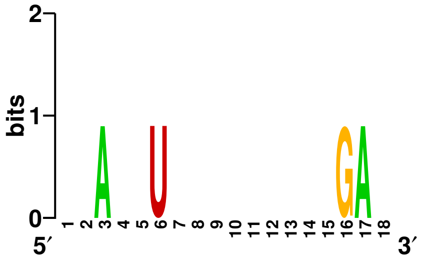

19-mers:

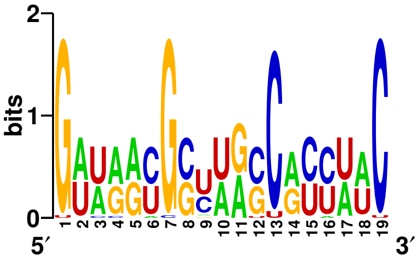

20-mers:

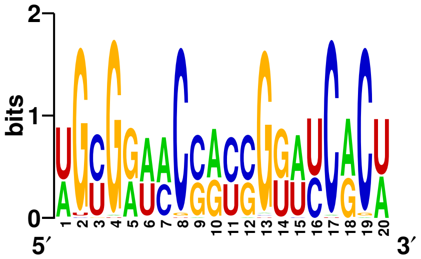

21-mers:

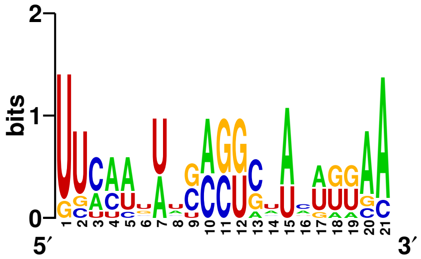

22-mers:

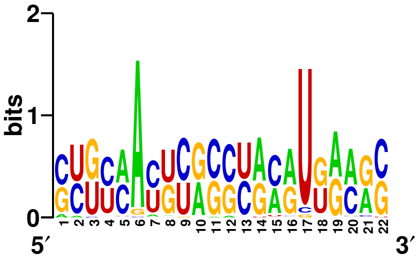

23-mers:

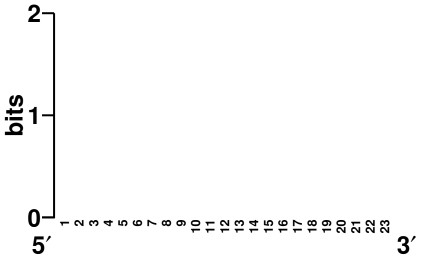

24-mers:

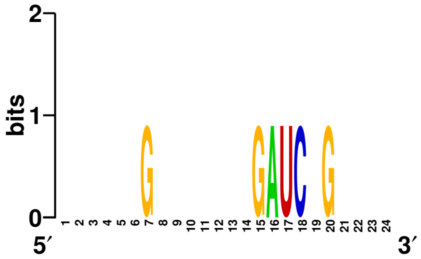

25-mers:

(no read)

26-mers:

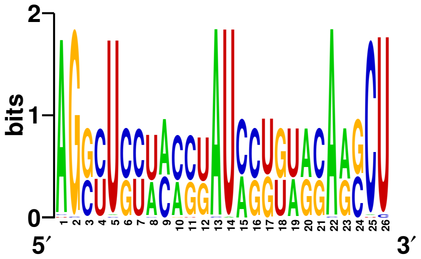

27-mers:

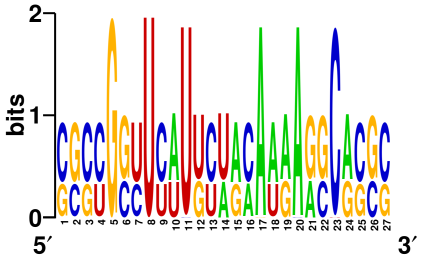

28-mers:

(no read)

29-mers:

(no read)

30-mers:

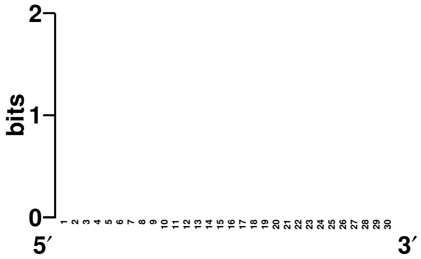

Adult male, library 4:

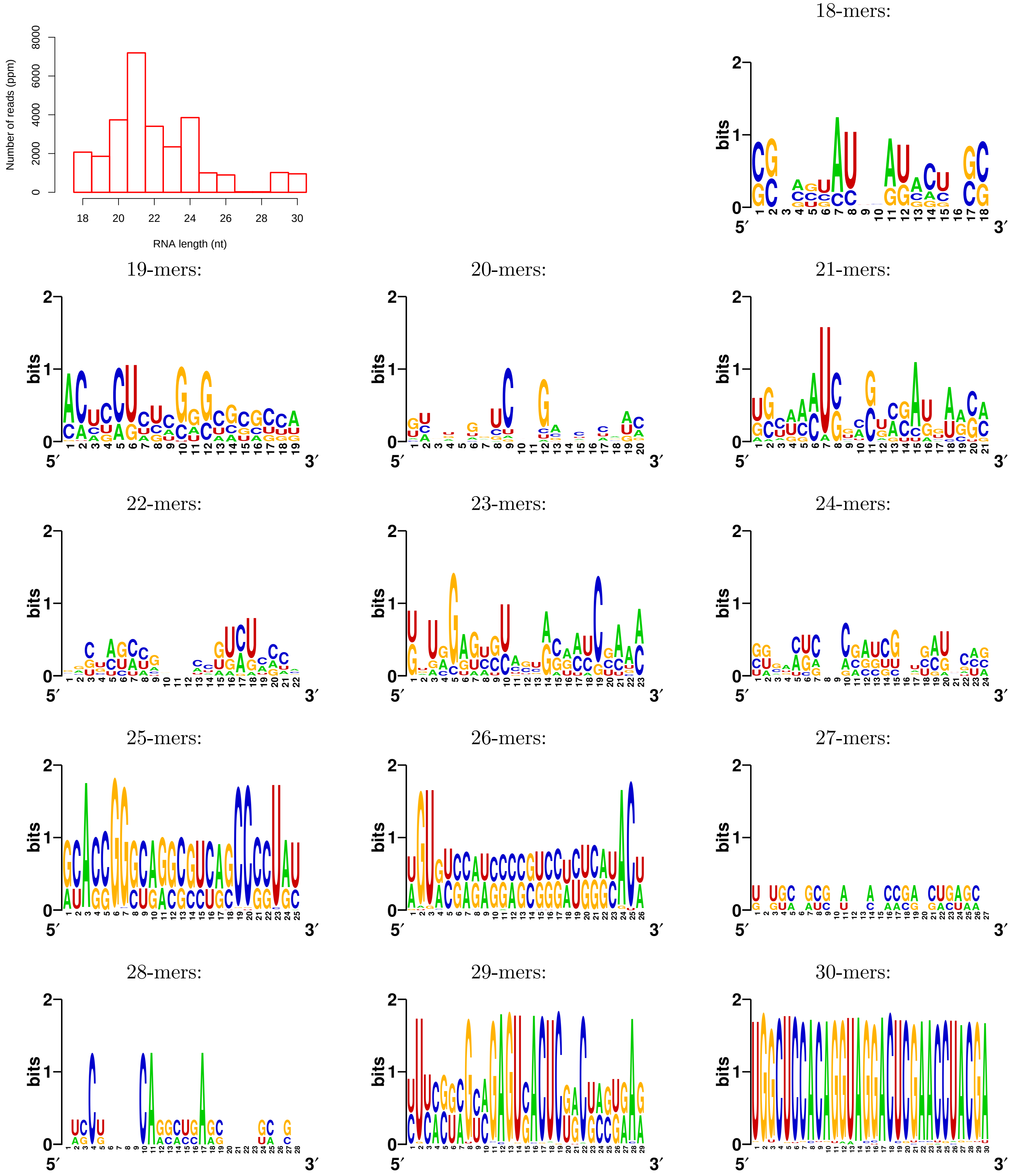

8 Extragenomic and extratranscriptomic reads matching the *Vibrio alginolyticus* genome

8.1 Libraries #1 (total 5´ monophosphorylated small RNAs)

Embryo 8h, library 1:

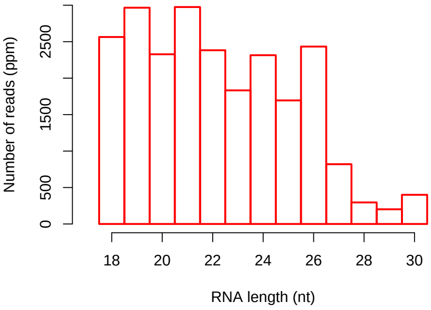

18-mers:

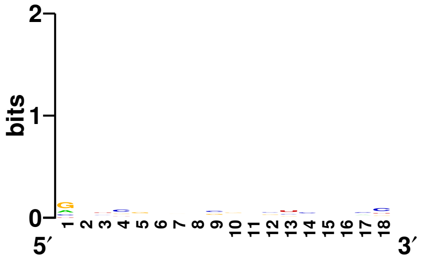

19-mers:

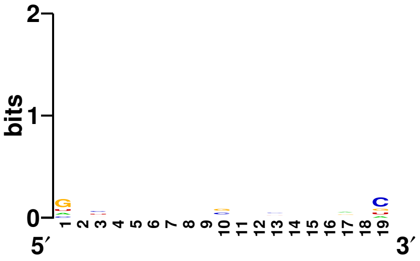

20-mers:

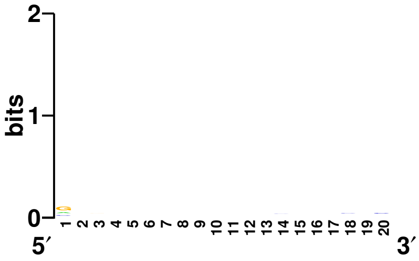

21-mers:

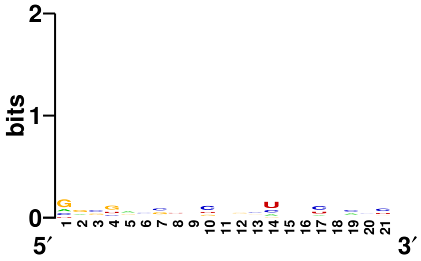

22-mers:

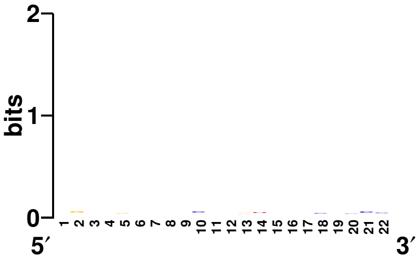

23-mers:

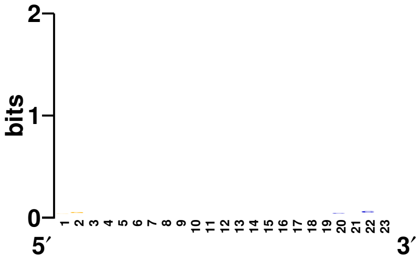

24-mers:

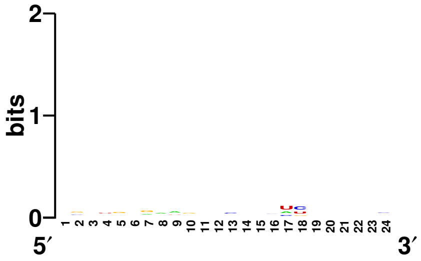

25-mers:

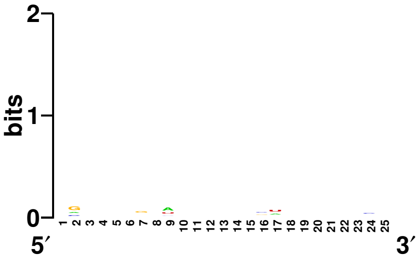

26-mers:

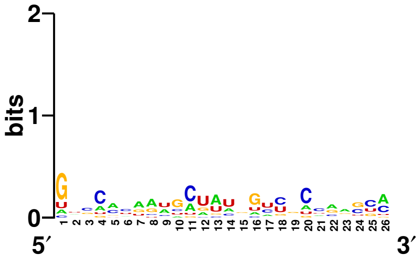

27-mers:

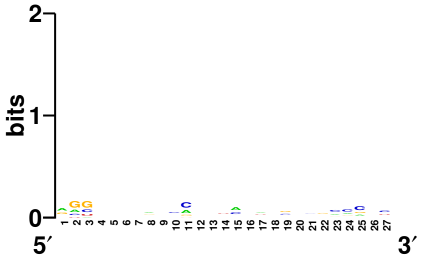

28-mers:

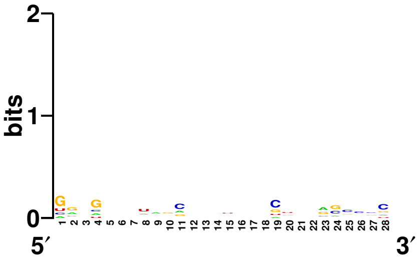

29-mers:

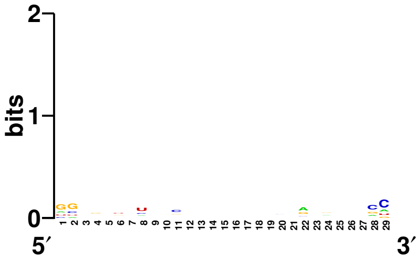

30-mers:

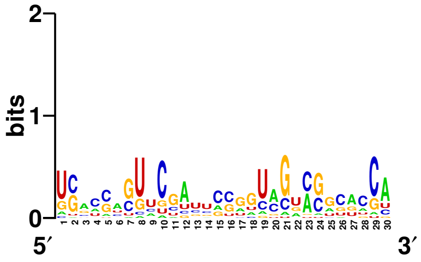

Embryo 15h, library 1:

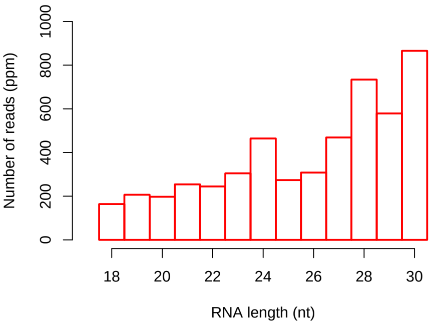

18-mers:

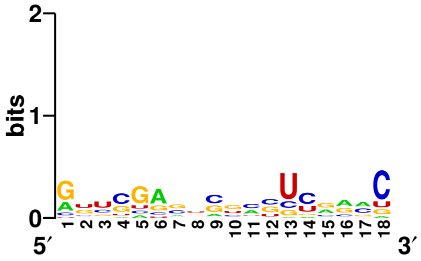

19-mers:

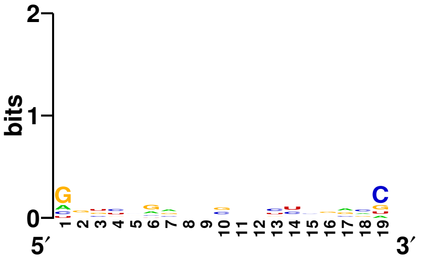

20-mers:

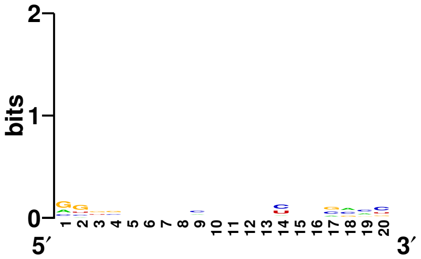

21-mers:

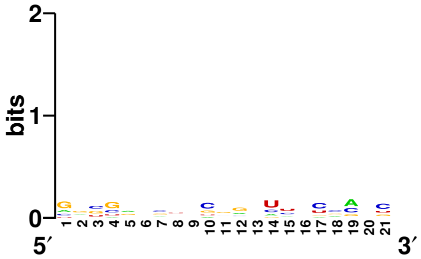

22-mers:

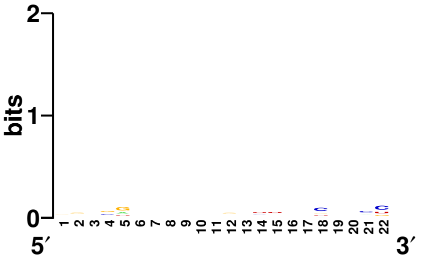

23-mers:

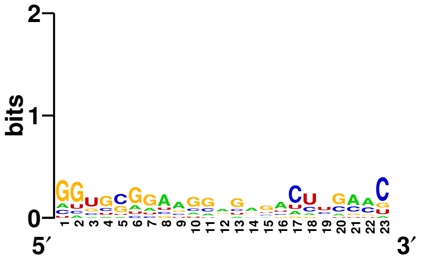

24-mers:

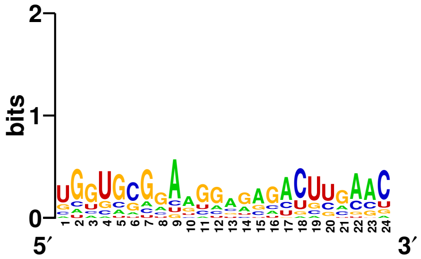

25-mers:

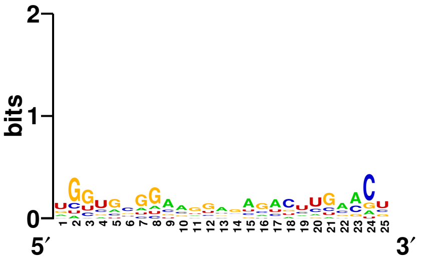

26-mers:

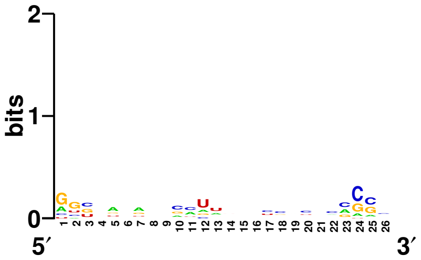

27-mers:

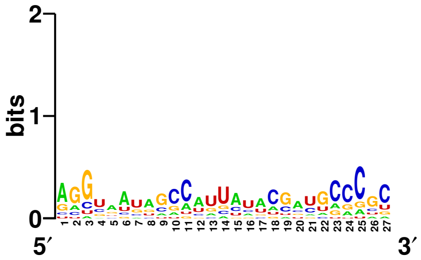

28-mers:

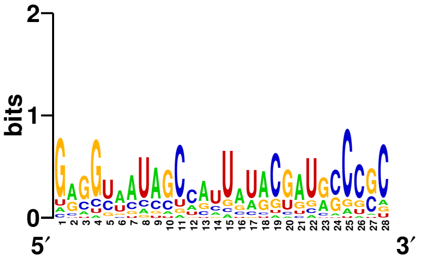

29-mers:

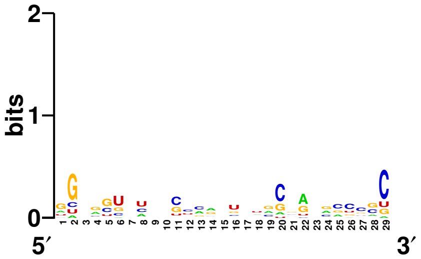

30-mers:

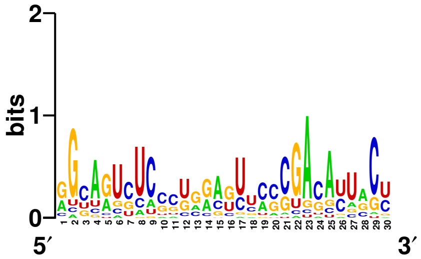

Embryo 36h, library 1:

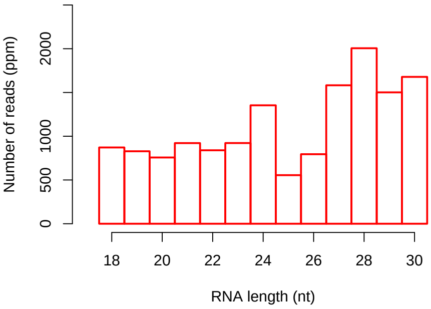

18-mers:

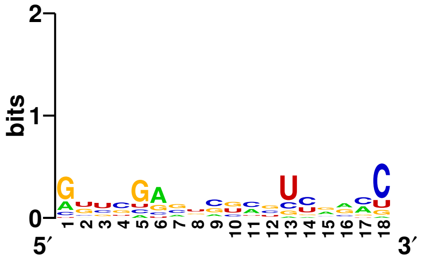

19-mers:

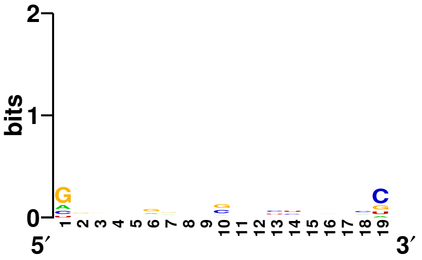

20-mers:

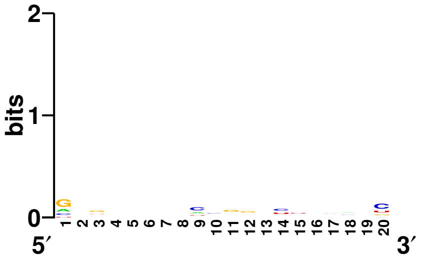

21-mers:

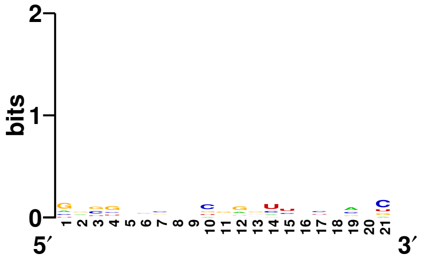

22-mers:

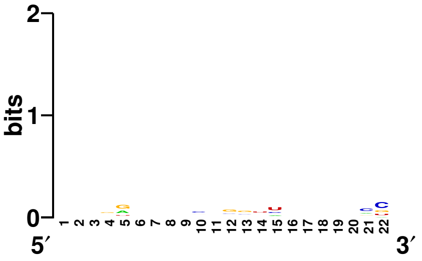

23-mers:

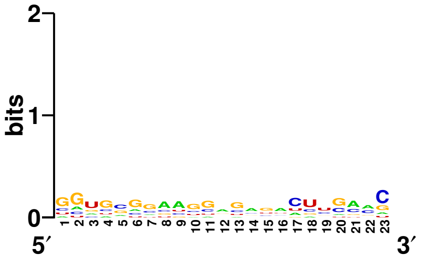

24-mers:

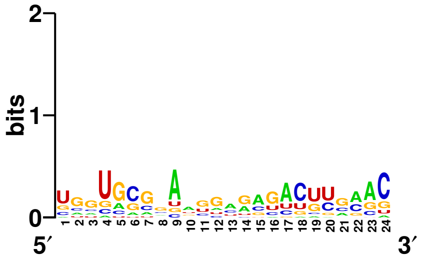

25-mers:

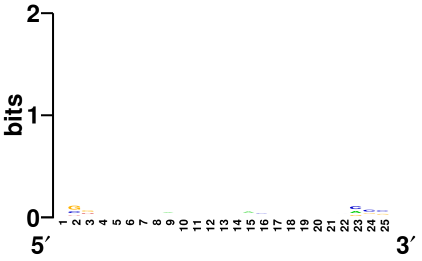

26-mers:

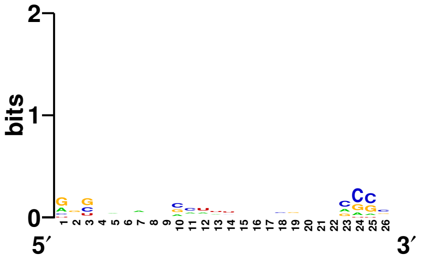

27-mers:

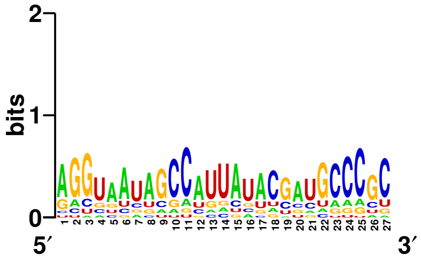

28-mers:

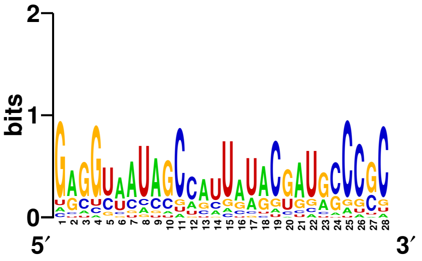

29-mers:

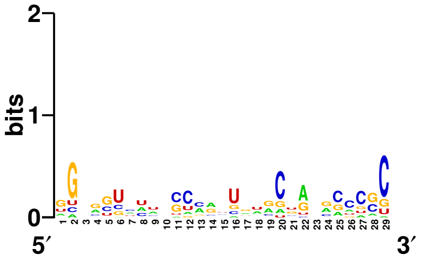

30-mers:

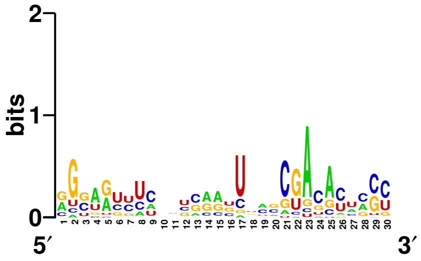

Embryo 60h, library 1:

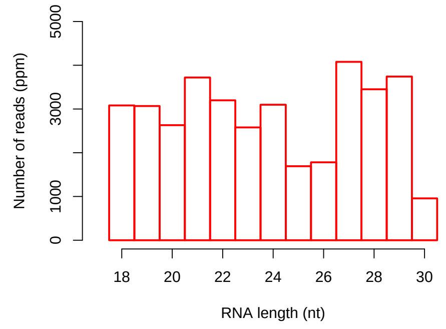

18-mers:

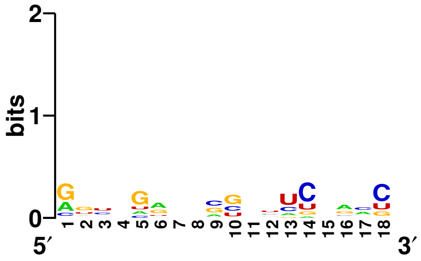

19-mers:

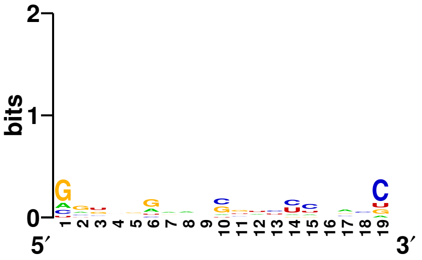

20-mers:

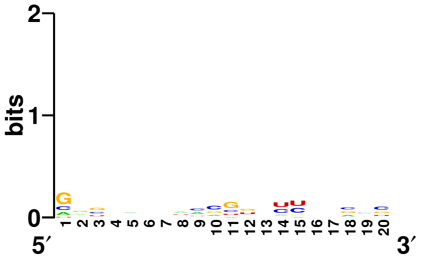

21-mers:

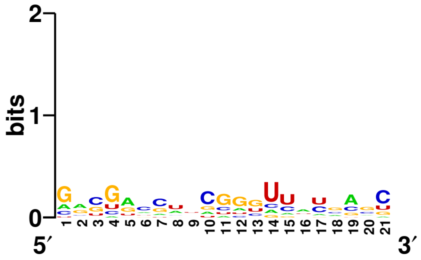

22-mers:

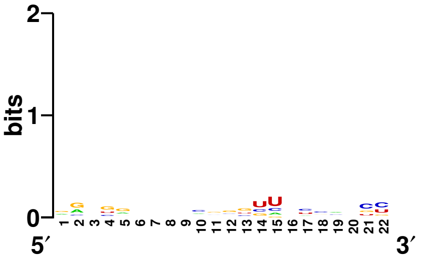

23-mers:

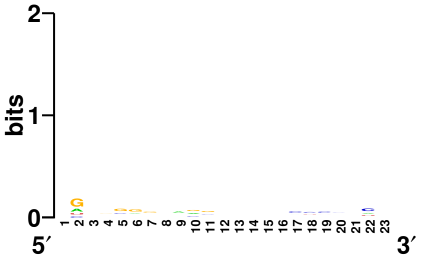

24-mers:

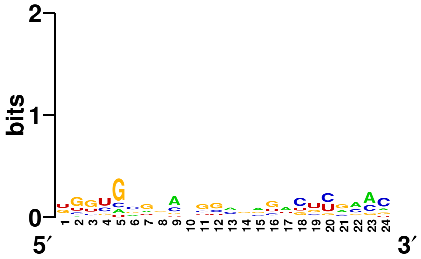

25-mers:

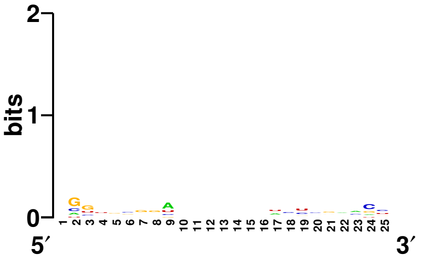

26-mers:

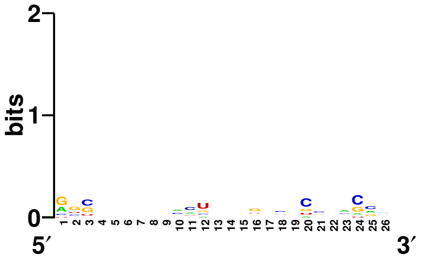

27-mers:

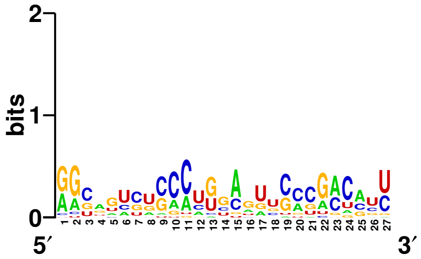

28-mers:

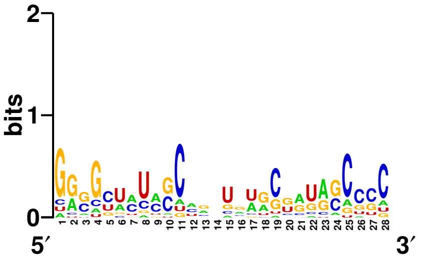

29-mers:

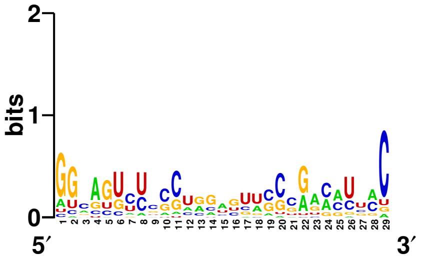

30-mers:

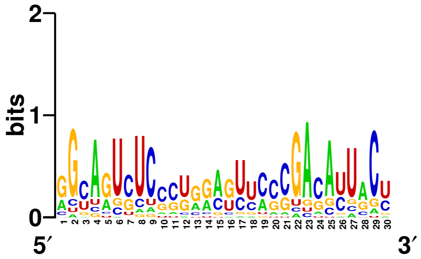

Adult female, library 1:

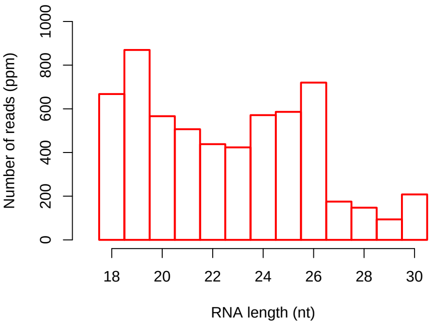

18-mers:

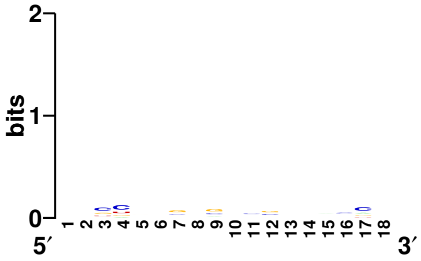

19-mers:

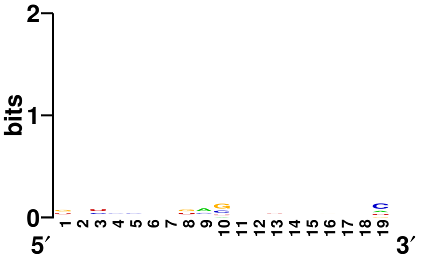

20-mers:

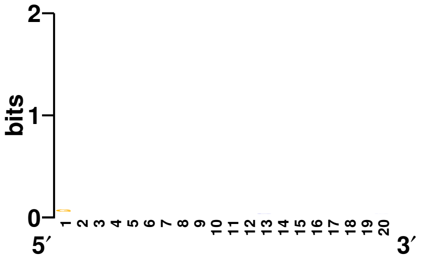

21-mers:

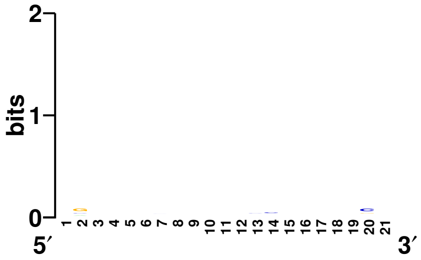

22-mers:

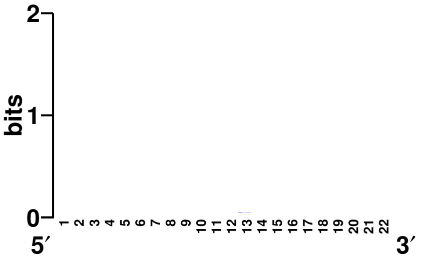

23-mers:

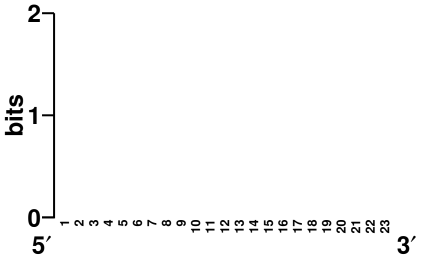

24-mers:

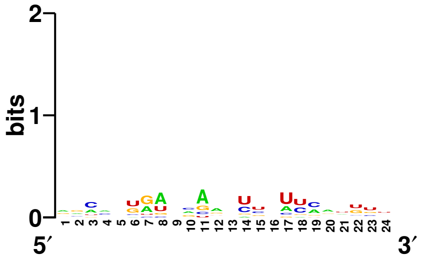

25-mers:

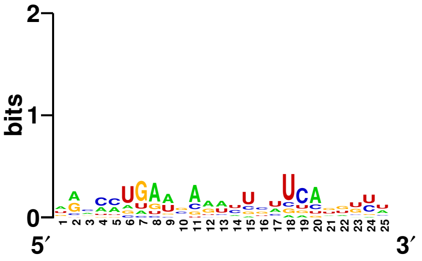

26-mers:

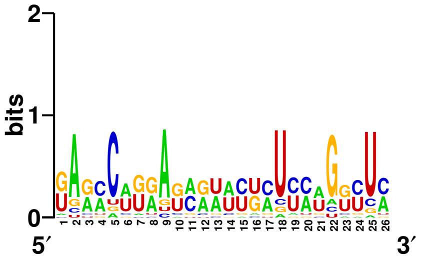

27-mers:

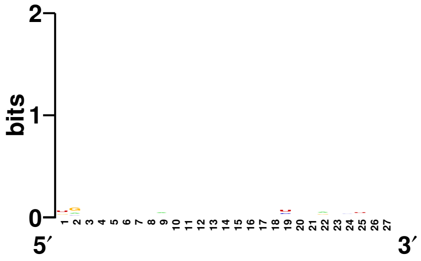

28-mers:

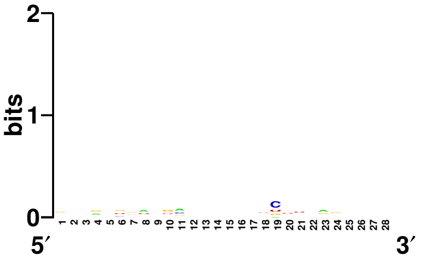

29-mers:

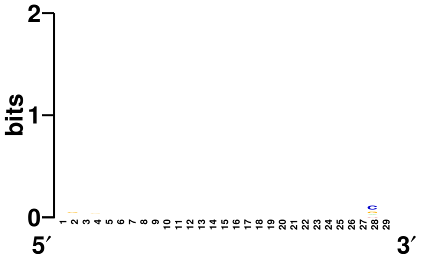

30-mers:

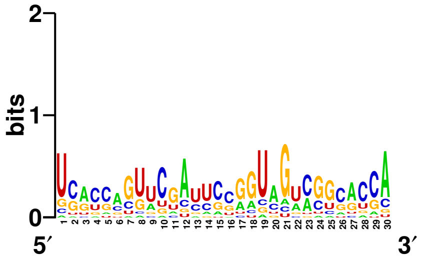

Adult male, library 1:

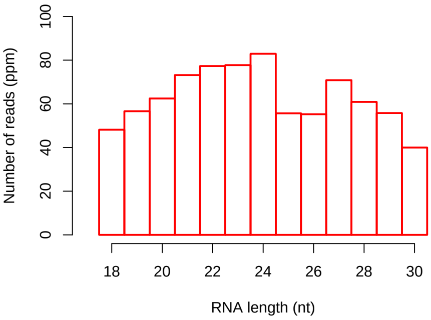

18-mers:

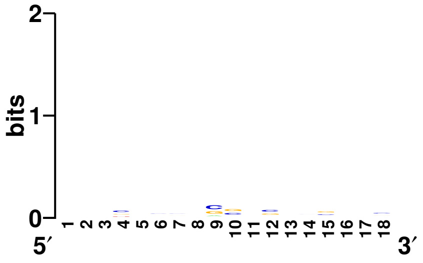

19-mers:

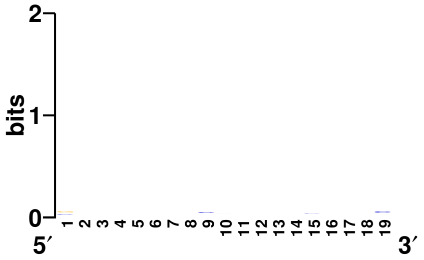

20-mers:

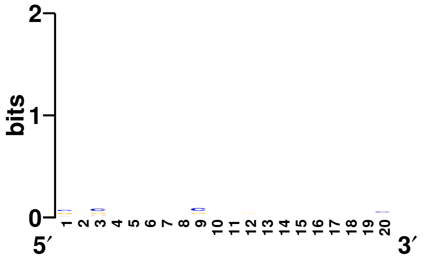

21-mers:

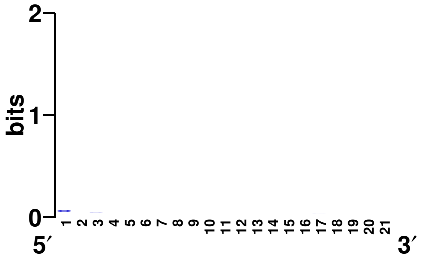

22-mers:

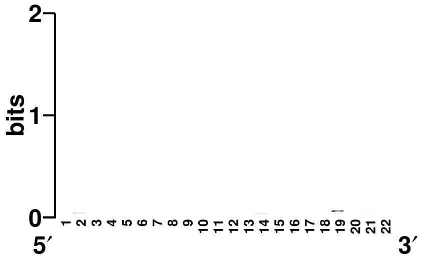

23-mers:

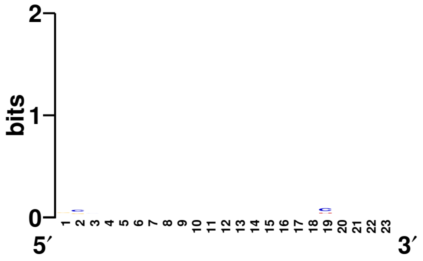

24-mers:

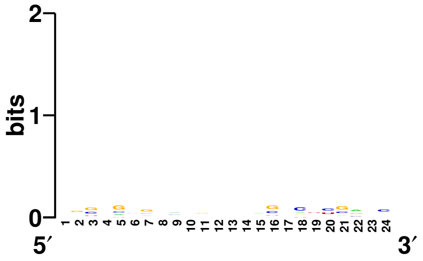

25-mers:

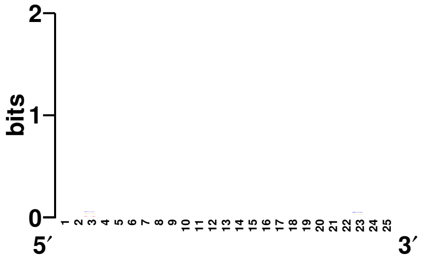

26-mers:

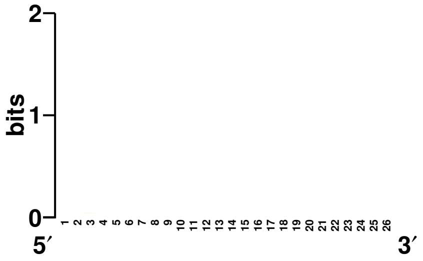

27-mers:

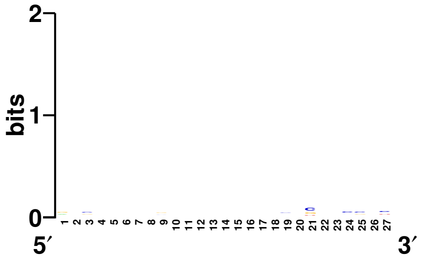

28-mers:

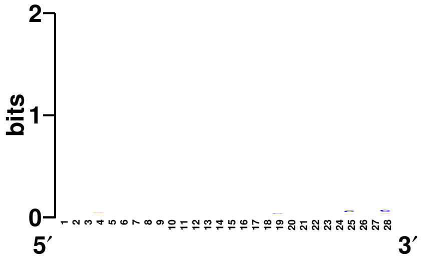

29-mers:

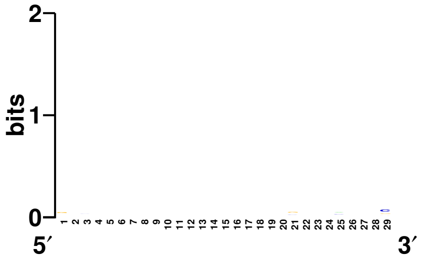

30-mers:

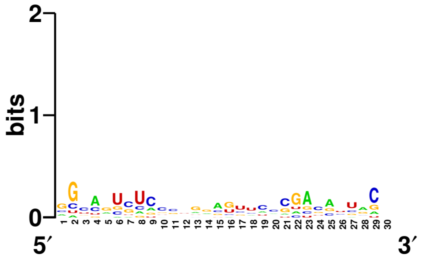

8.2 Libraries #2 (3´ modified, 5´ monophosphorylated small RNAs)

Embryo 8h, library 2:

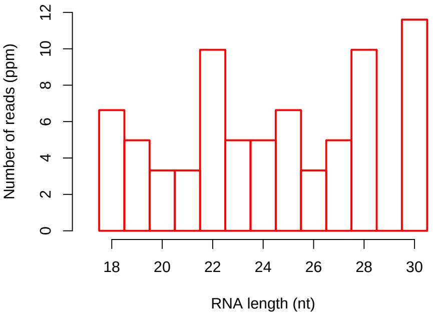

18-mers:

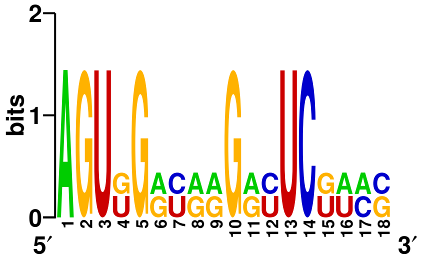

19-mers:

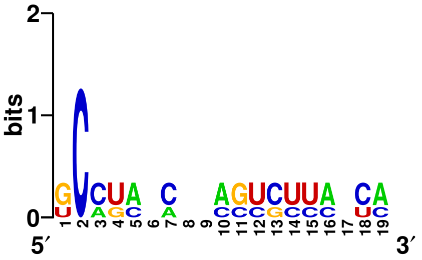

20-mers:

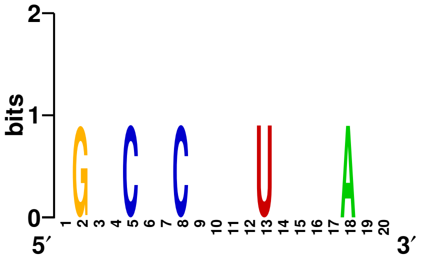

21-mers:

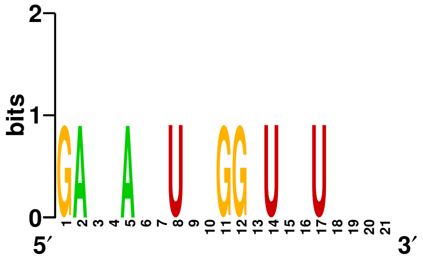

22-mers:

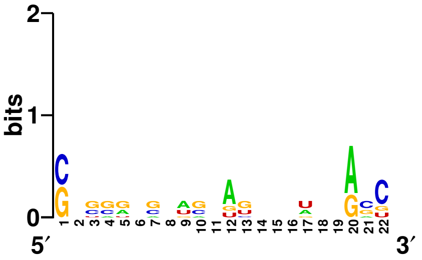

23-mers:

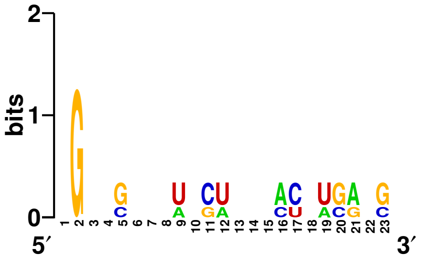

24-mers:

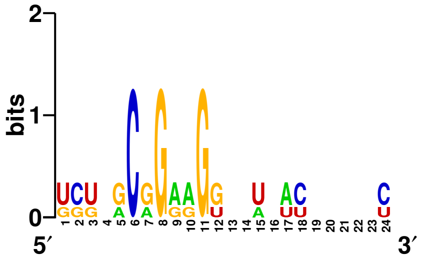

25-mers:

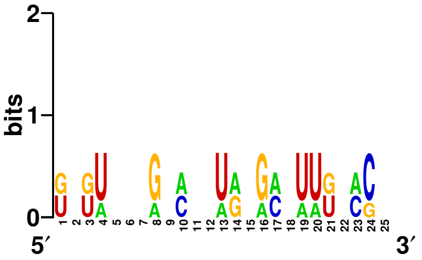

26-mers:

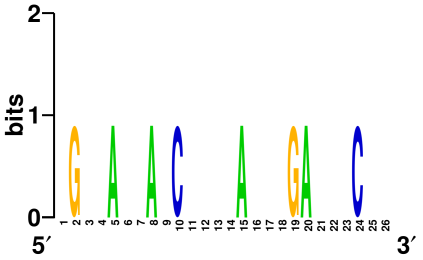

27-mers:

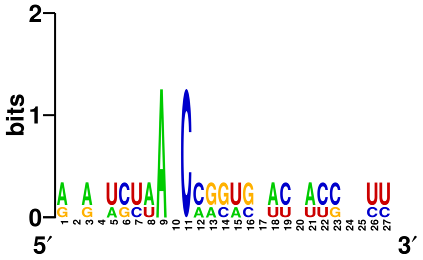

28-mers:

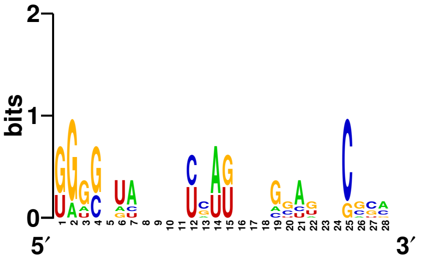

29-mers:

(no read)

30-mers:

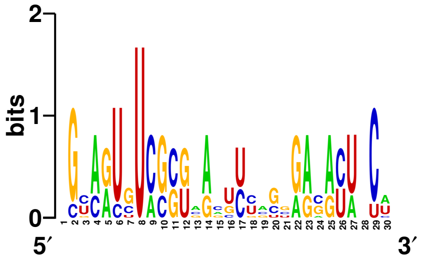

Embryo 15h, library 2:

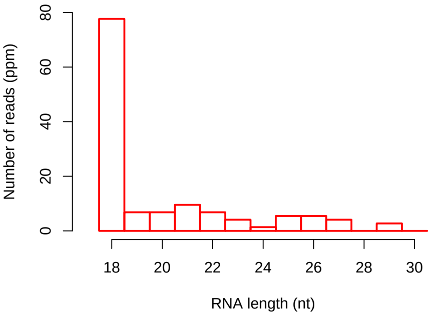

18-mers:

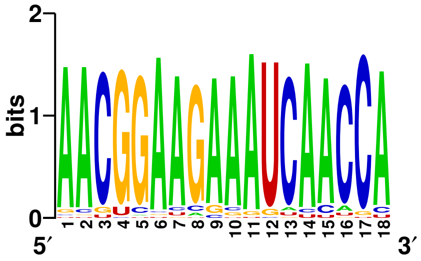

19-mers:

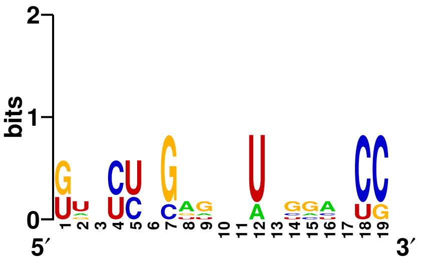

20-mers:

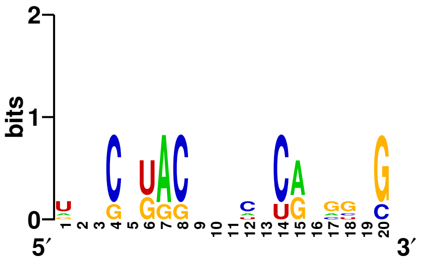

21-mers:

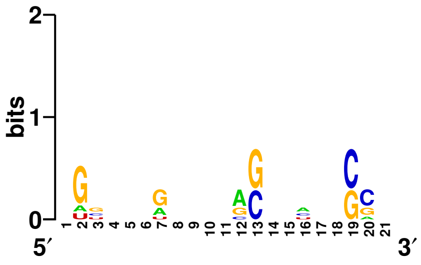

22-mers:

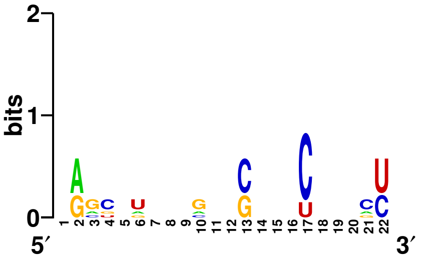

23-mers:

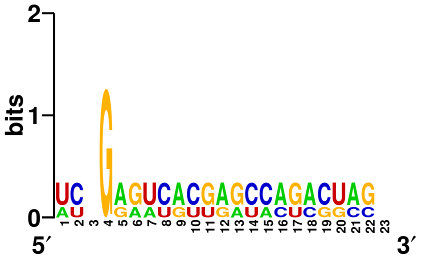

24-mers:

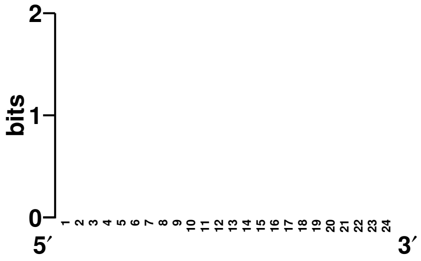

25-mers:

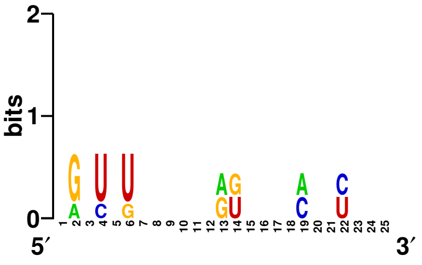

26-mers:

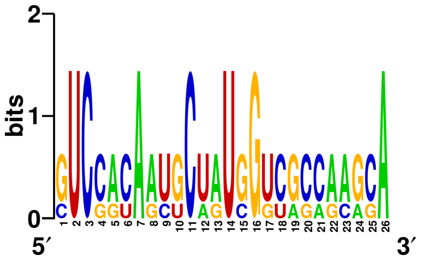

27-mers:

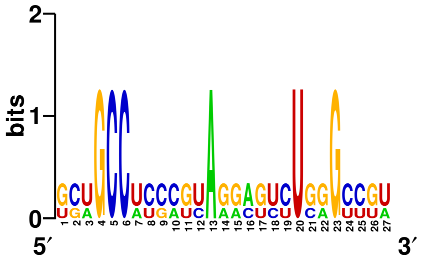

28-mers:

(no read)

29-mers:

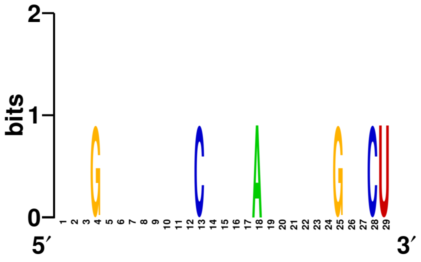

30-mers:

(no read)

Embryo 36h, library 2:

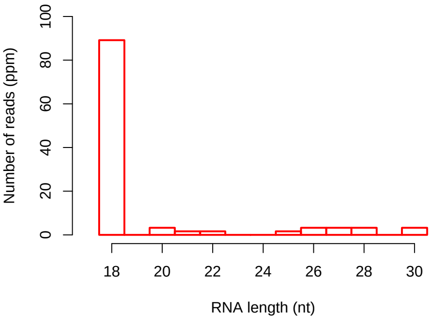

18-mers:

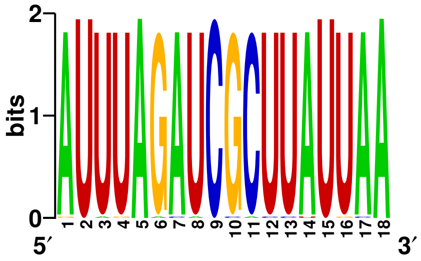

19-mers:

(no read)

20-mers:

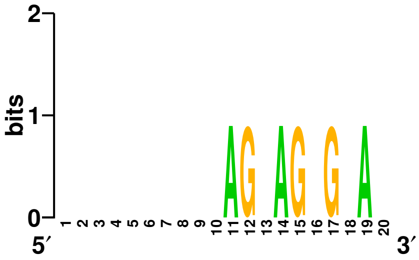

21-mers:

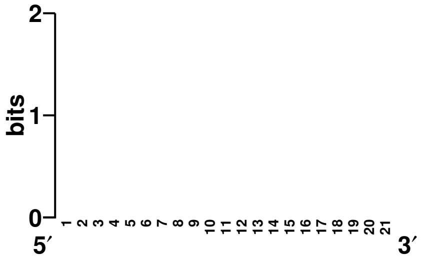

22-mers:

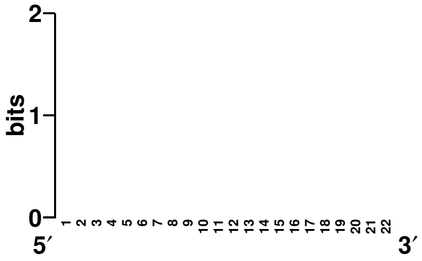

23-mers:

(no read)

24-mers:

(no read)

25-mers:

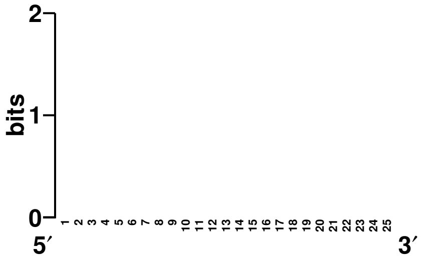

26-mers:

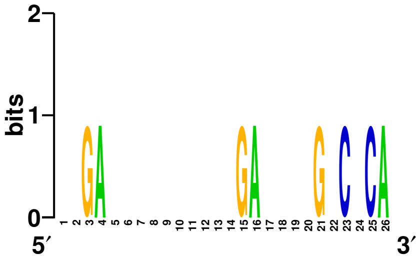

27-mers:

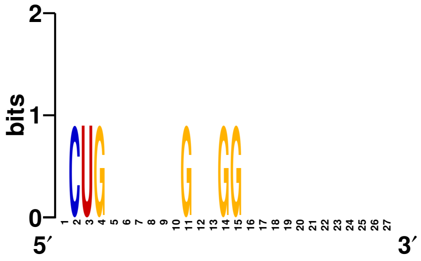

28-mers:

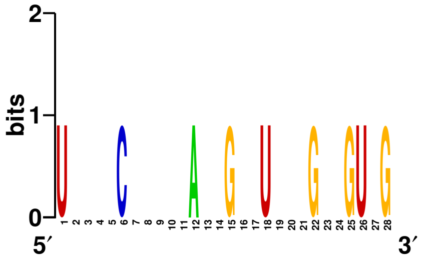

29-mers:

(no read)

30-mers:

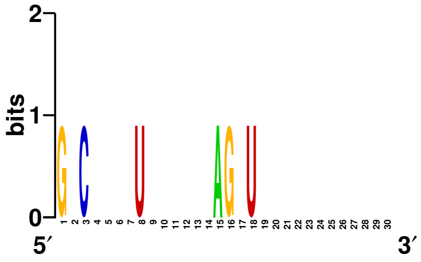

Embryo 60h, library 2:

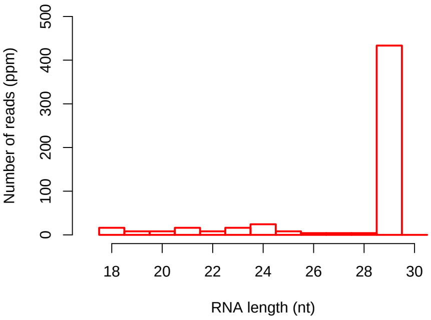

18-mers:

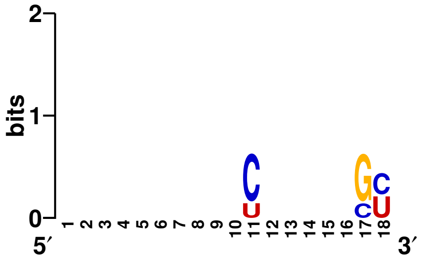

19-mers:

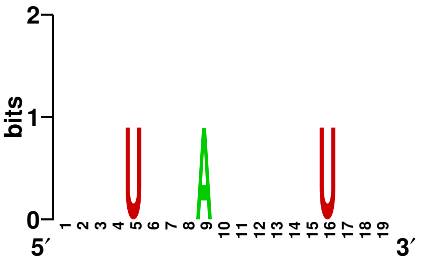

20-mers:

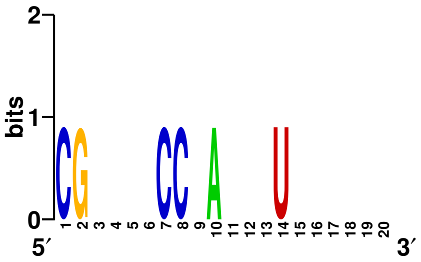

21-mers:

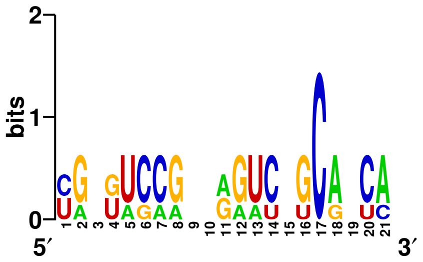

22-mers:

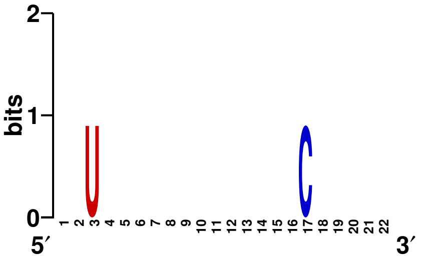

23-mers:

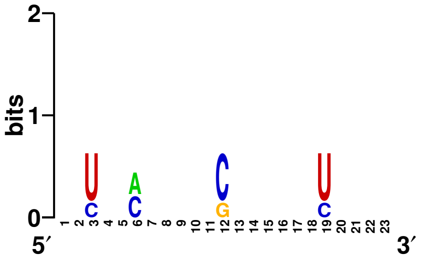

24-mers:

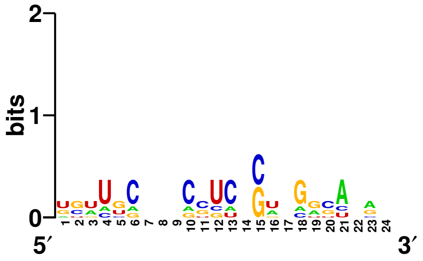

25-mers:

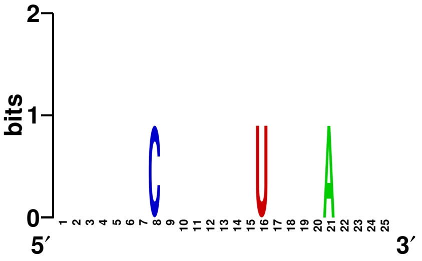

26-mers:

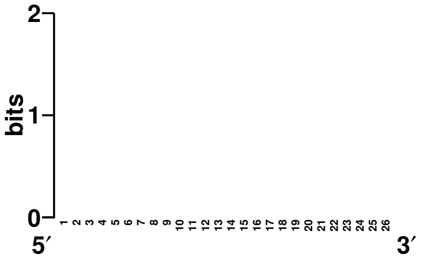

27-mers:

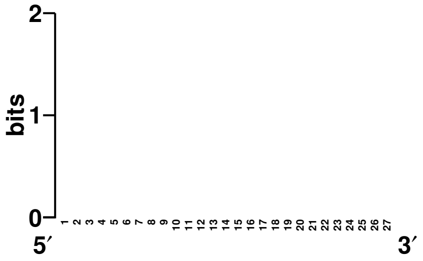

28-mers:

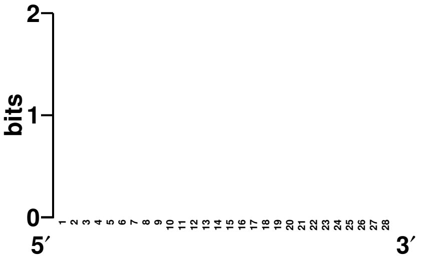

29-mers:

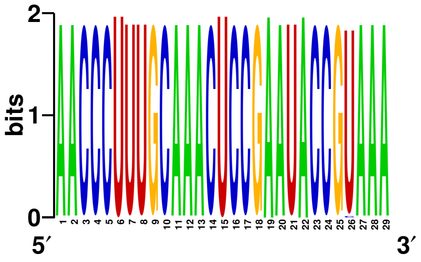

30-mers:

(no read)

Adult female, library 2:

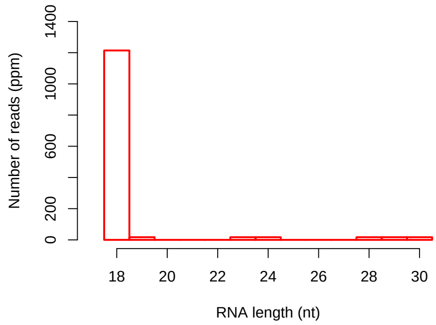

18-mers:

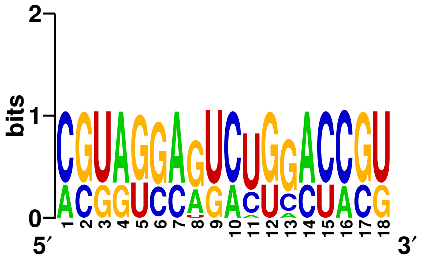

19-mers:

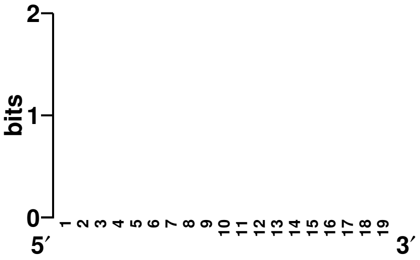

20-mers:

(no read)

21-mers:

(no read)

22-mers:

(no read)

23-mers:

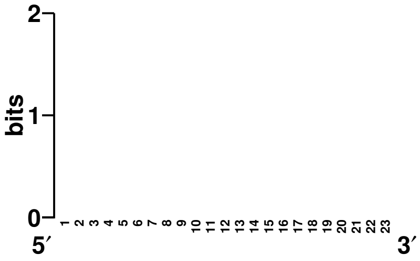

24-mers:

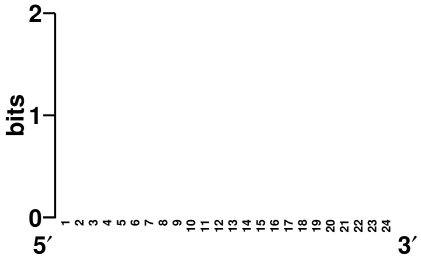

25-mers:

(no read)

26-mers:

(no read)

27-mers:

(no read)

28-mers:

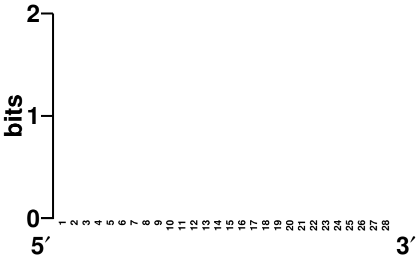

29-mers:

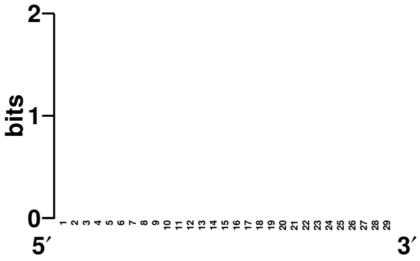

30-mers:

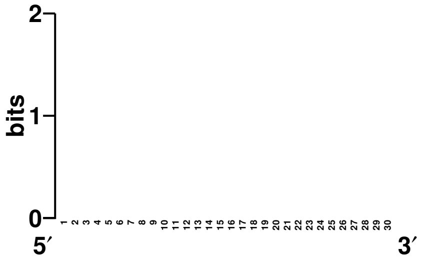

Adult male, library 2:

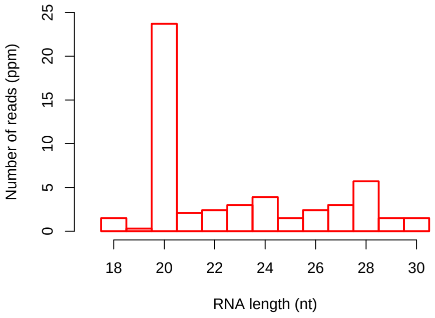

18-mers:

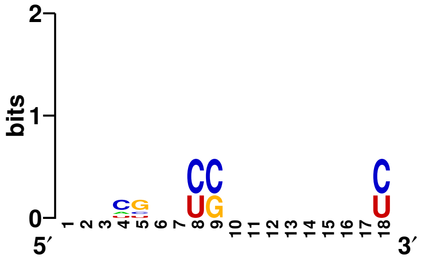

19-mers:

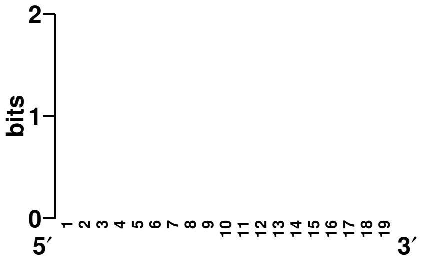

20-mers:

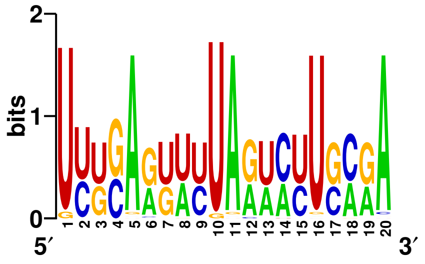

21-mers:

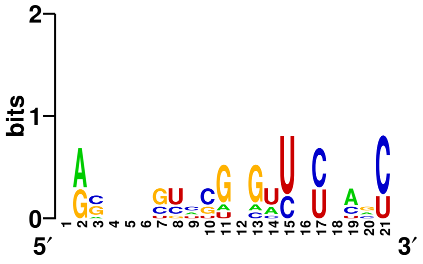

22-mers:

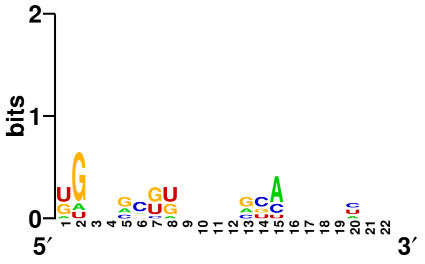

23-mers:

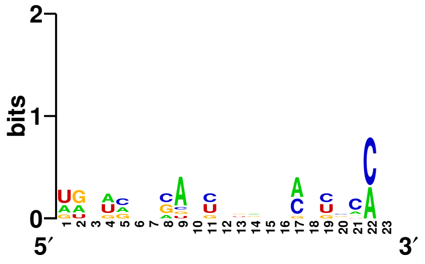

24-mers:

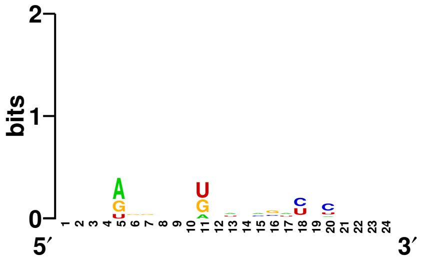

25-mers:

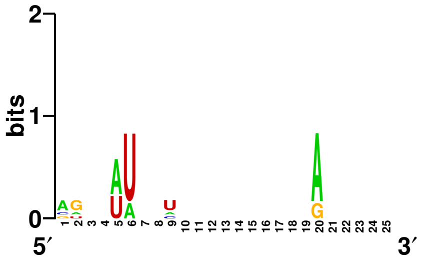

26-mers:

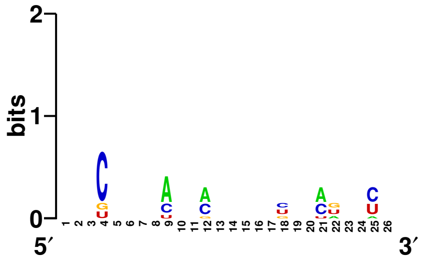

27-mers:

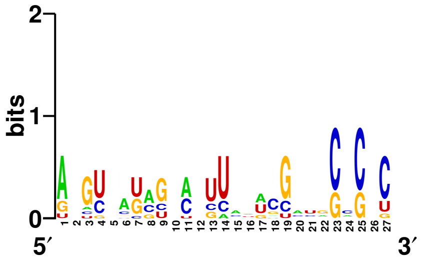

28-mers:

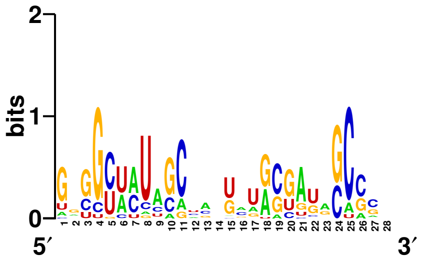

29-mers:

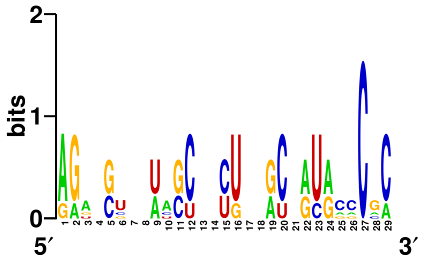

30-mers:

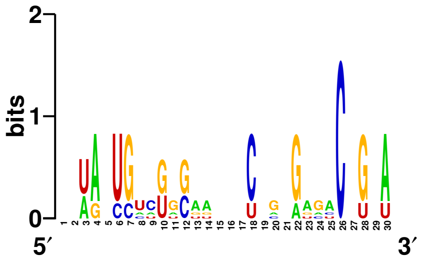

8.3 Libraries #3 (total 5´ hydroxyl or polyphosphorylated small RNAs)

Embryo 8h, library 3:

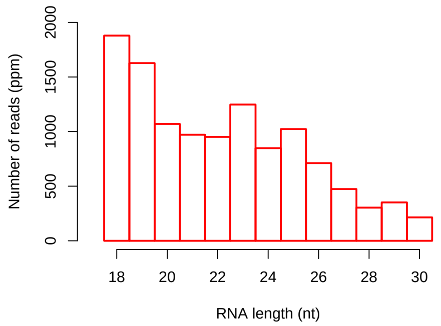

18-mers:

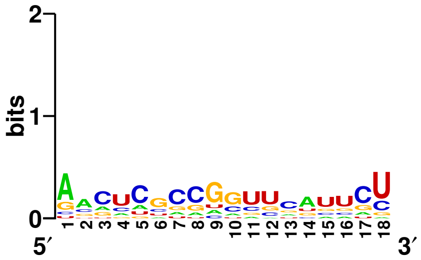

19-mers:

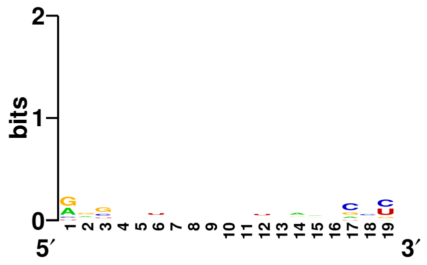

20-mers:

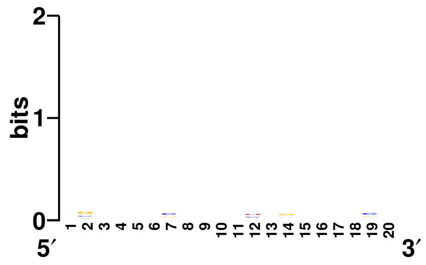

21-mers:

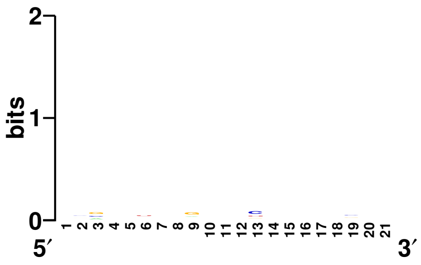

22-mers:

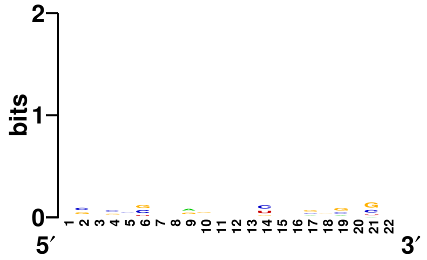

23-mers:

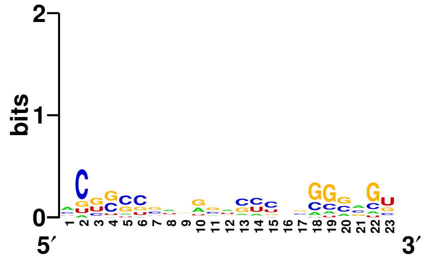

24-mers:

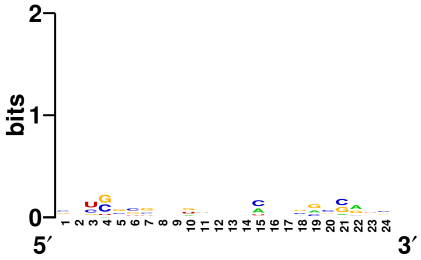

25-mers:

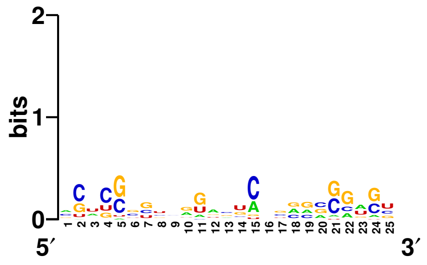

26-mers:

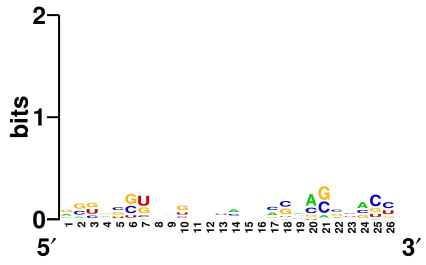

27-mers:

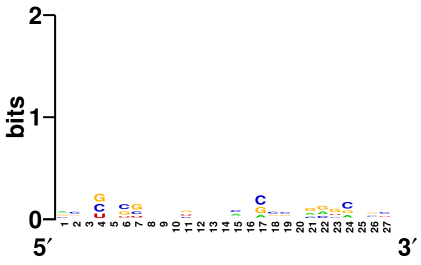

28-mers:

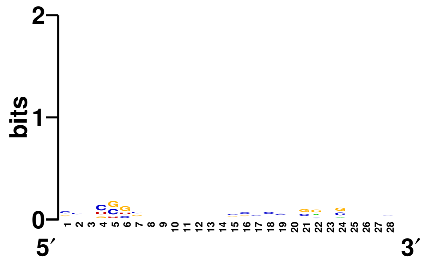

29-mers:

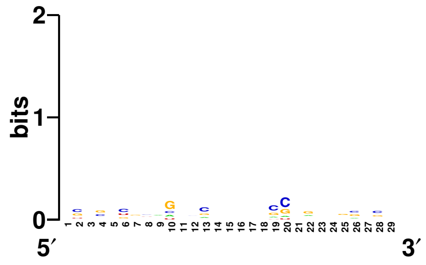

30-mers:

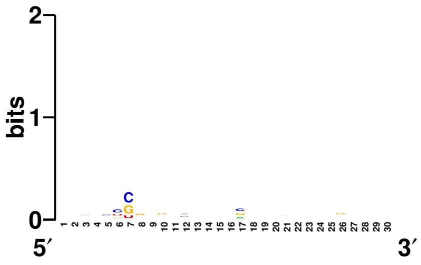

Embryo 15h, library 3:

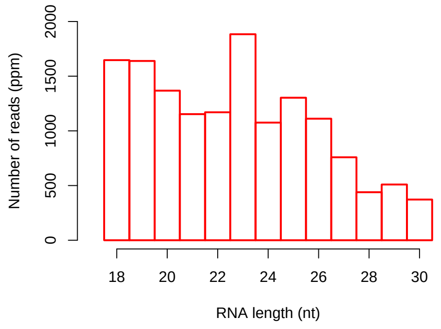

18-mers:

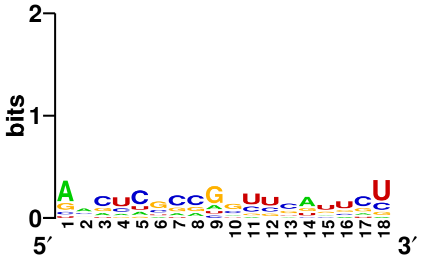

19-mers:

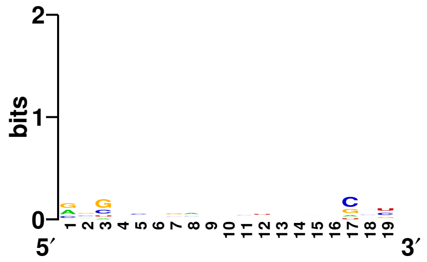

20-mers:

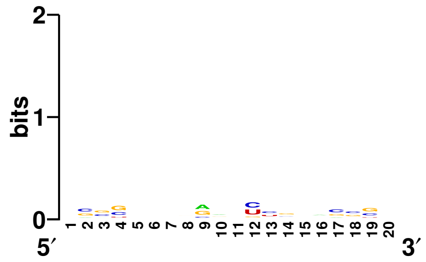

21-mers:

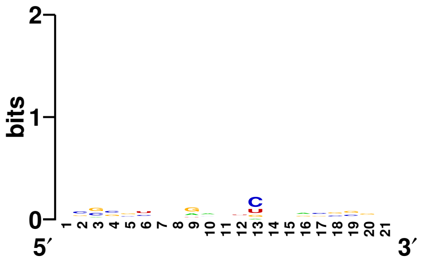

22-mers:

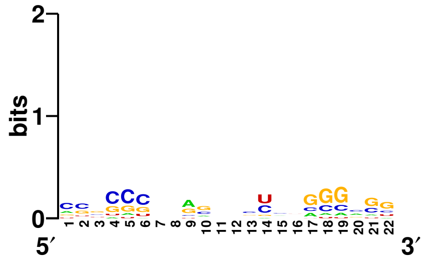

23-mers:

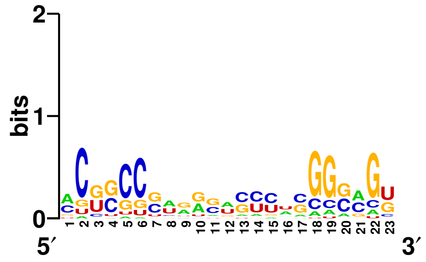

24-mers:

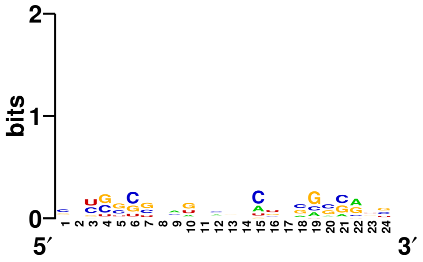

25-mers:

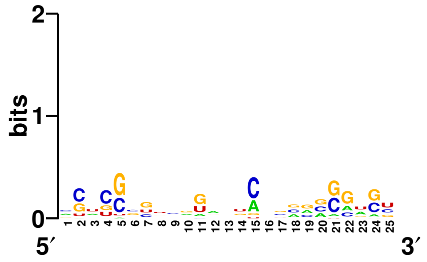

26-mers:

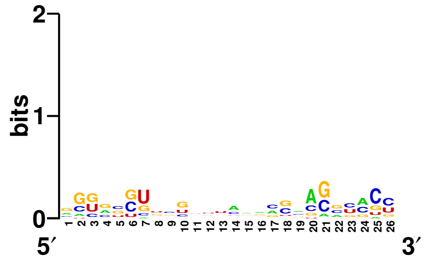

27-mers:

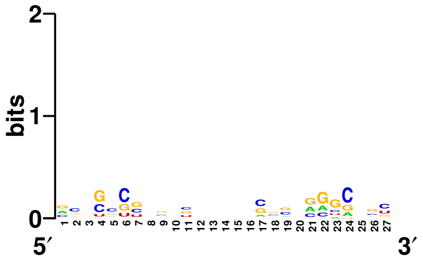

28-mers:

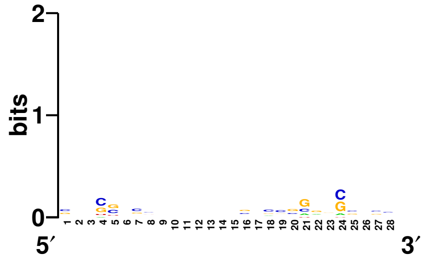

29-mers:

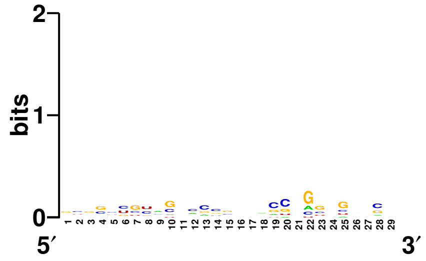

30-mers:

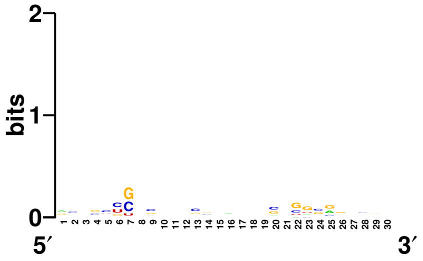

Embryo 36h, library 3:

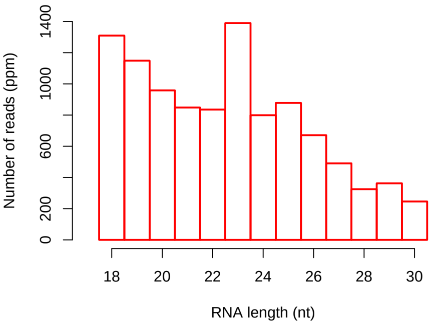

18-mers:

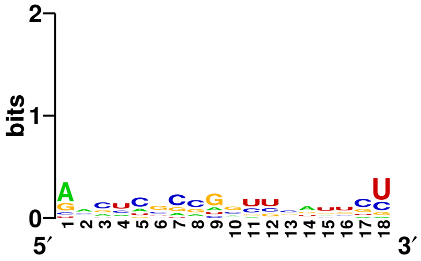

19-mers:

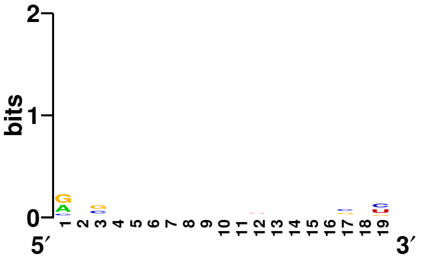

20-mers:

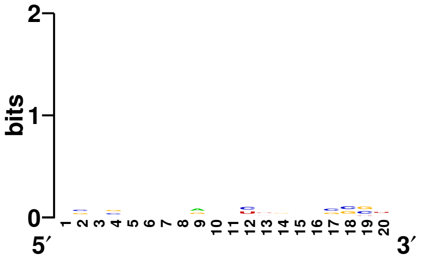

21-mers:

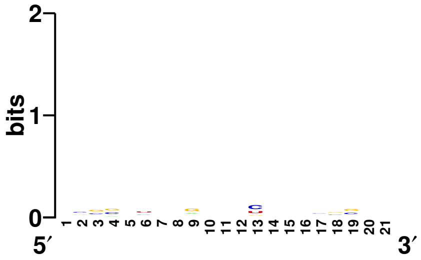

22-mers:

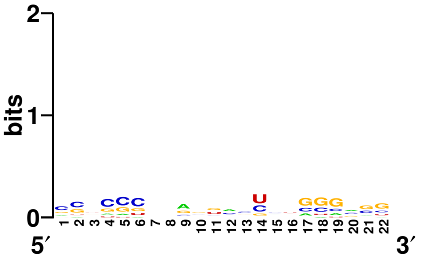

23-mers:

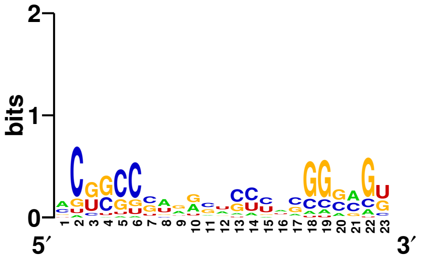

24-mers:

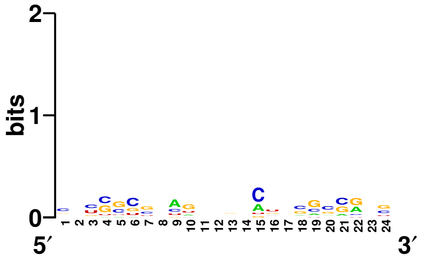

25-mers:

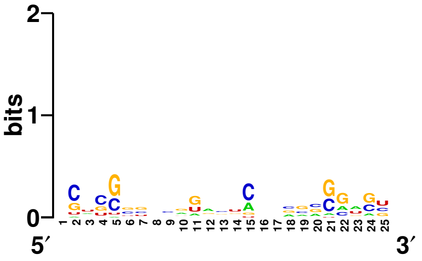

26-mers:

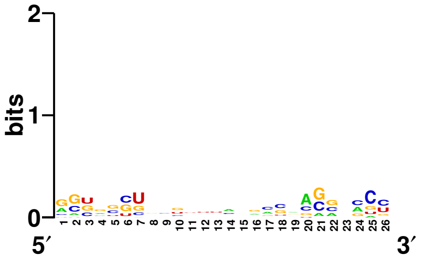

27-mers:

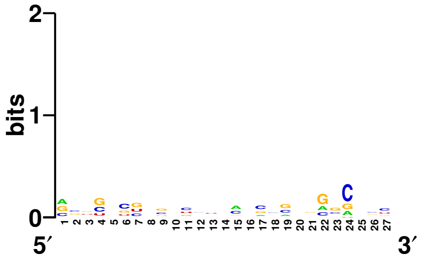

28-mers:

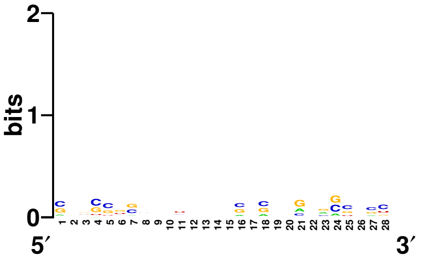

29-mers:

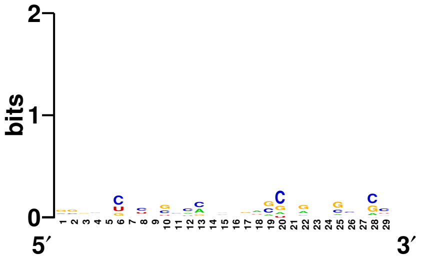

30-mers:

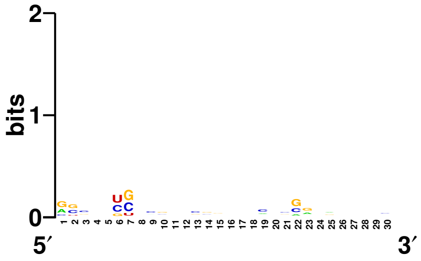

Embryo 60h, library 3:

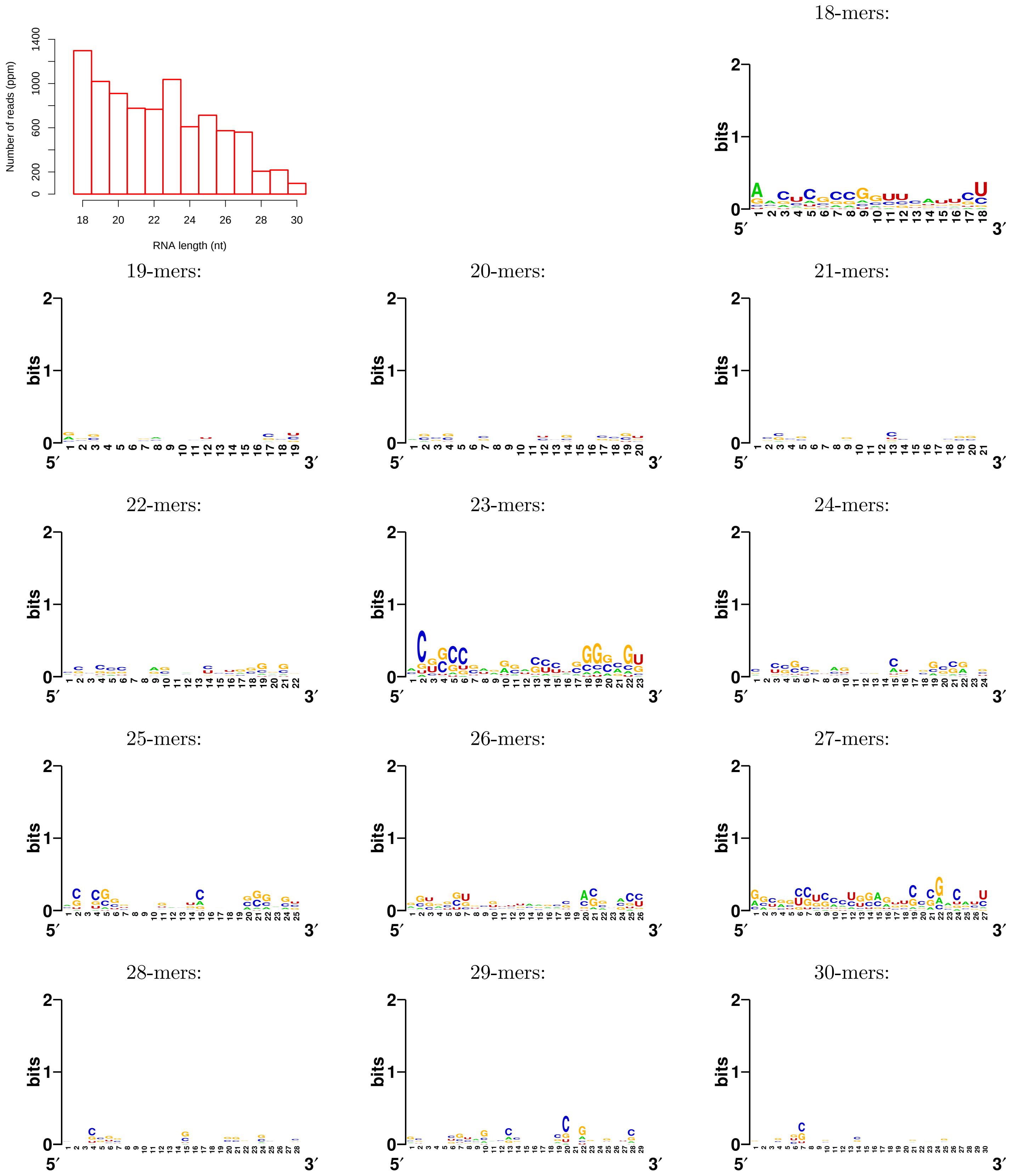

Adult female, library 3:

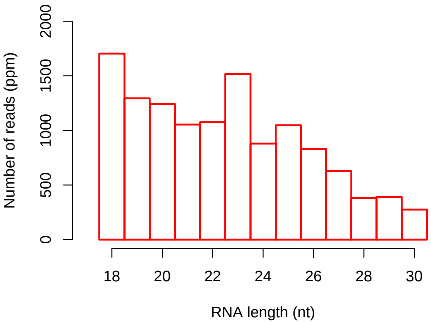

18-mers:

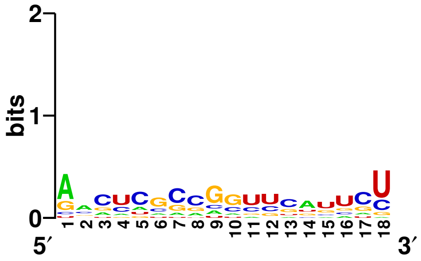

19-mers:

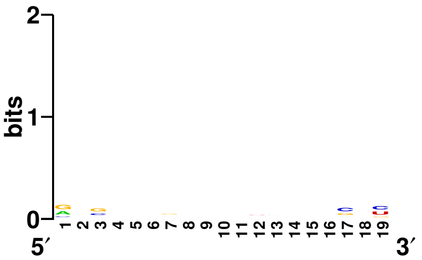

20-mers:

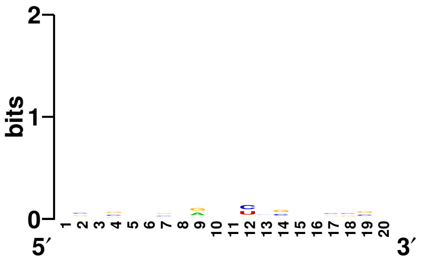

21-mers:

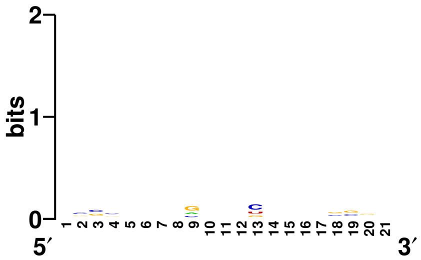

22-mers:

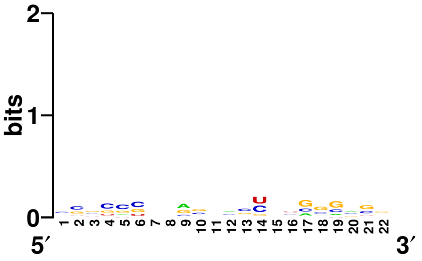

23-mers:

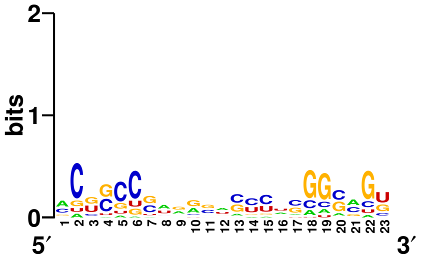

24-mers:

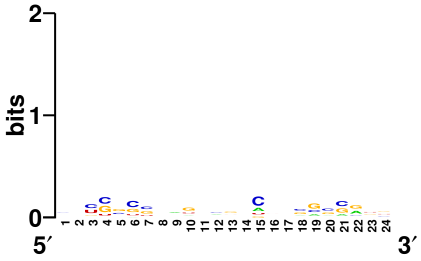

25-mers:

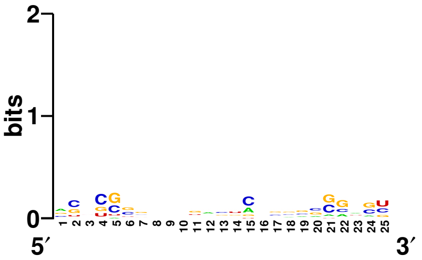

26-mers:

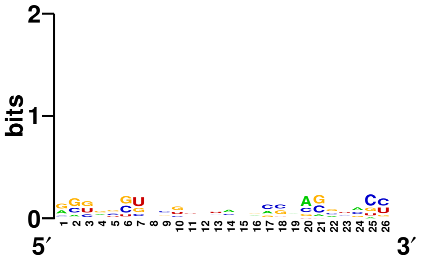

27-mers:

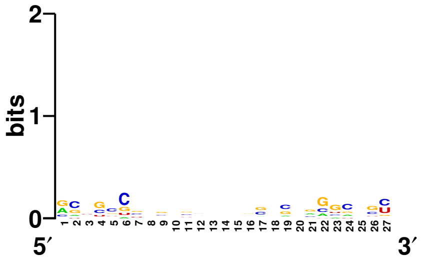

28-mers:

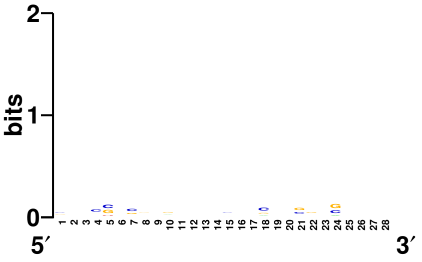

29-mers:

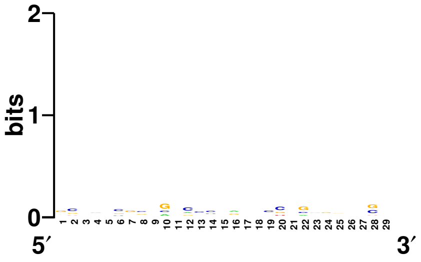

30-mers:

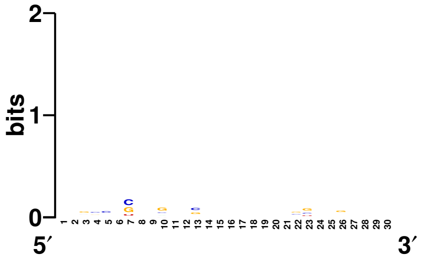

Adult male, library 3:

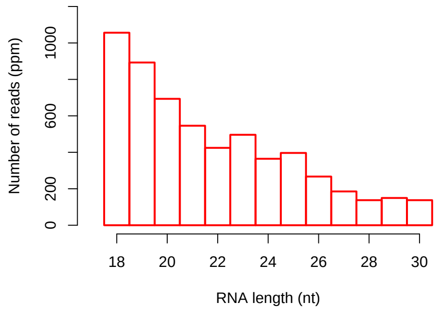

18-mers:

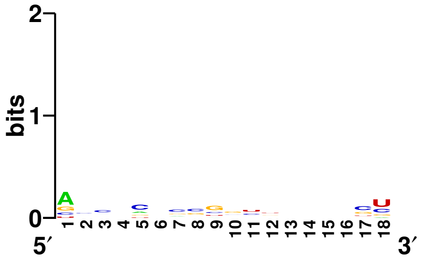

19-mers:

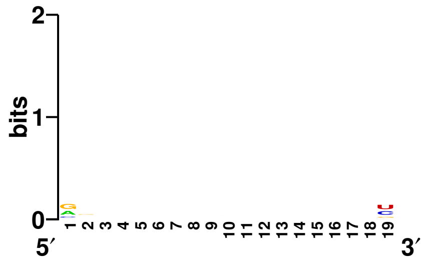

20-mers:

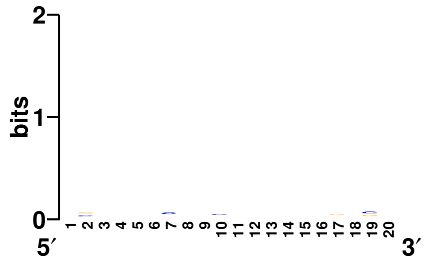

21-mers:

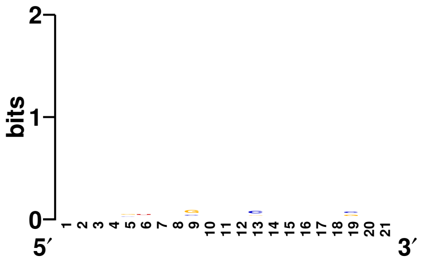

22-mers:

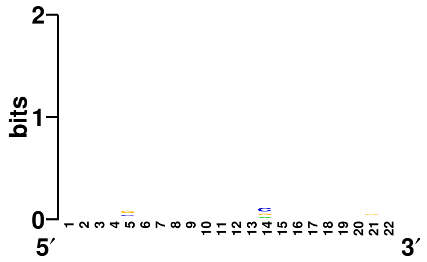

23-mers:

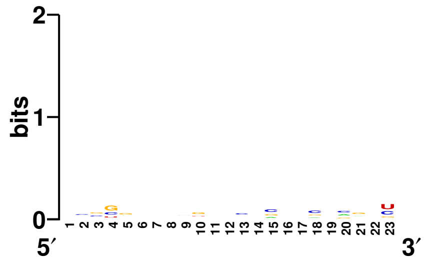

24-mers:

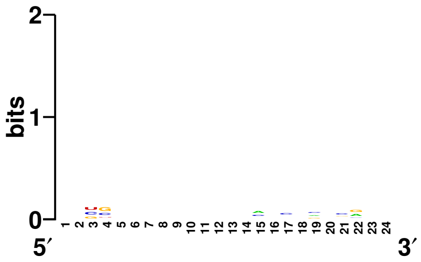

25-mers:

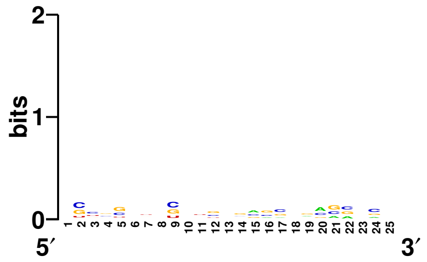

26-mers:

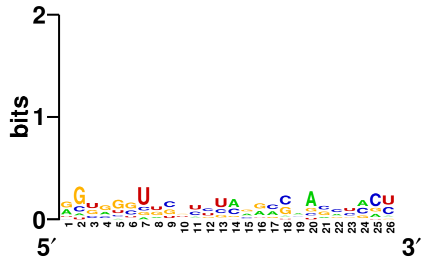

27-mers:

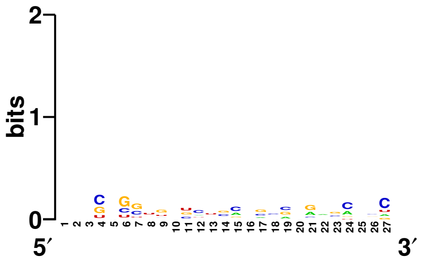

28-mers:

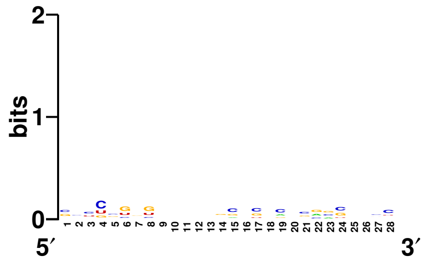

29-mers:

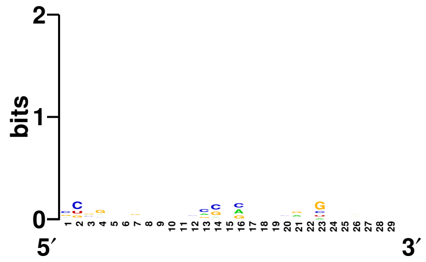

30-mers:

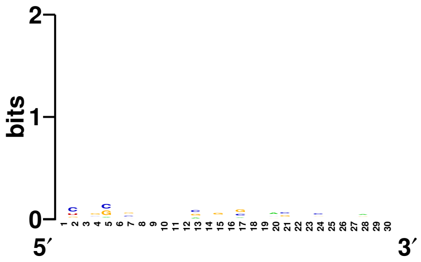

8.4 Libraries #4 (3´ modified, 5´ hydroxyl or polyphosphorylated small RNAs)

Embryo 8h, library 4:

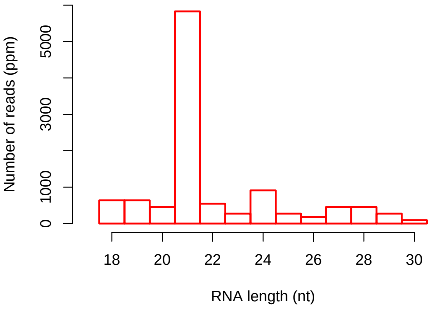

18-mers:

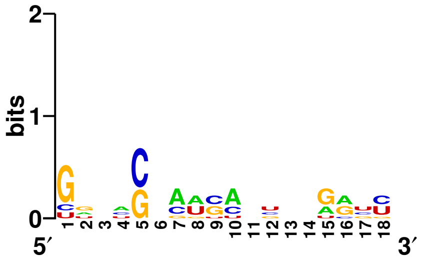

19-mers:

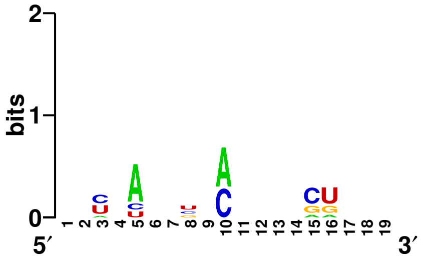

20-mers:

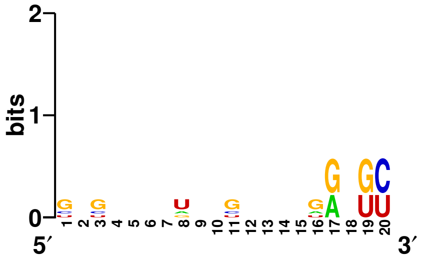

21-mers:

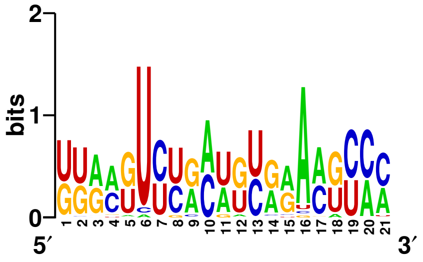

22-mers:

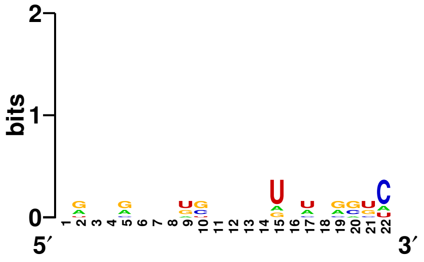

23-mers:

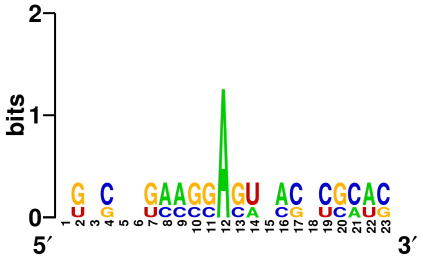

24-mers:

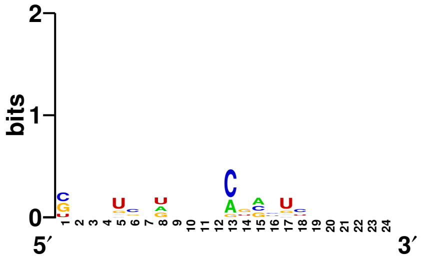

25-mers:

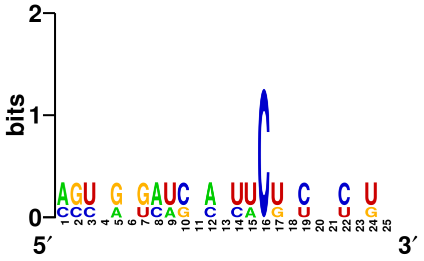

26-mers:

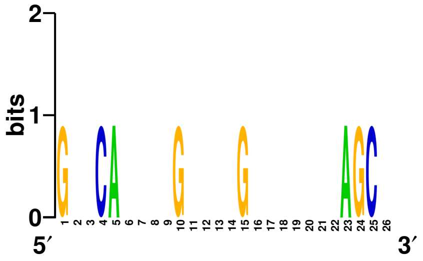

27-mers:

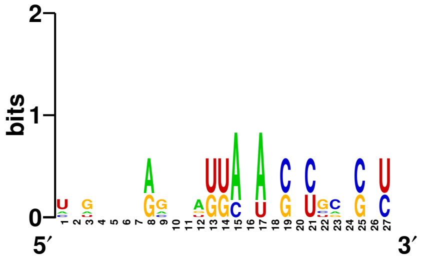

28-mers:

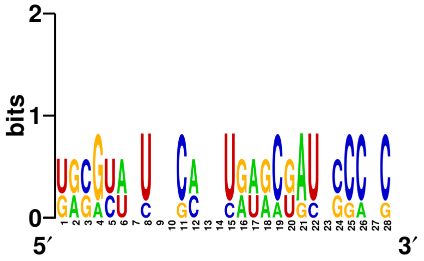

29-mers:

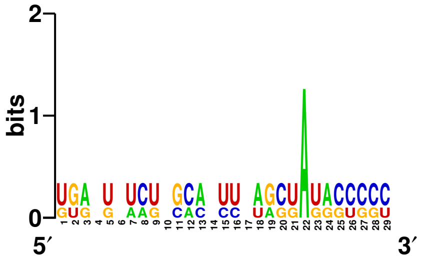

30-mers:

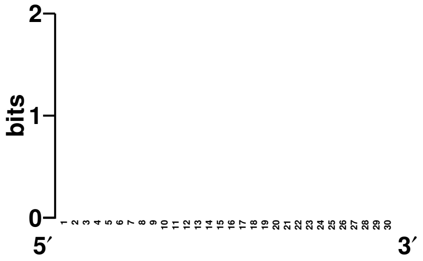

Embryo 15h, library 4:

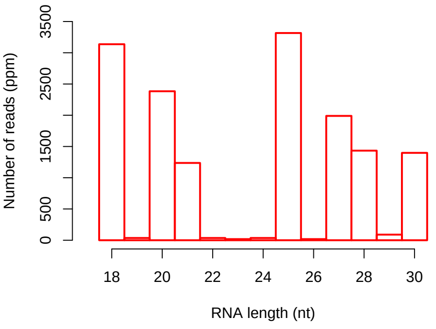

18-mers:

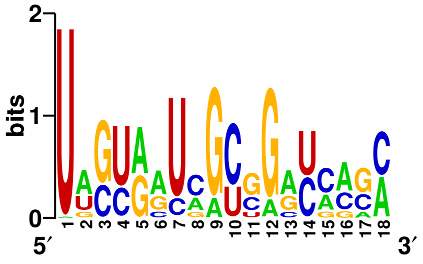

19-mers:

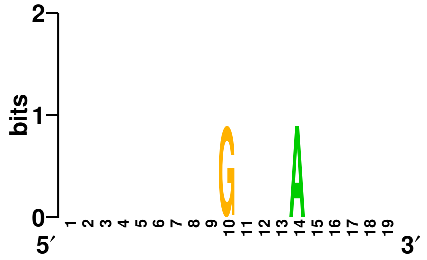

20-mers:

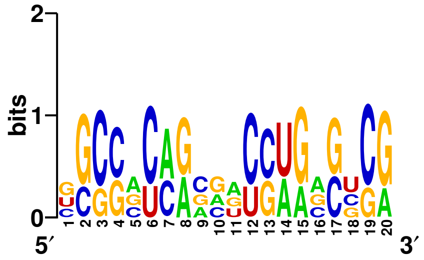

21-mers:

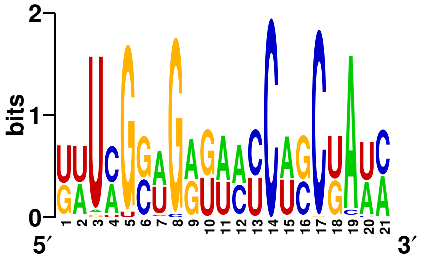

22-mers:

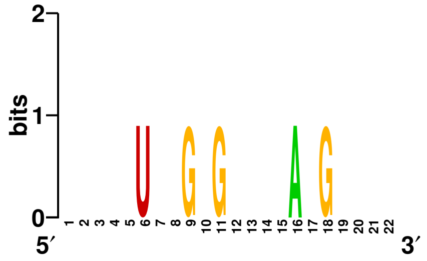

23-mers:

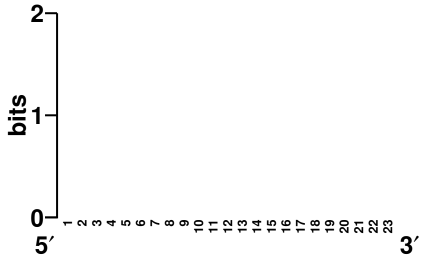

24-mers:

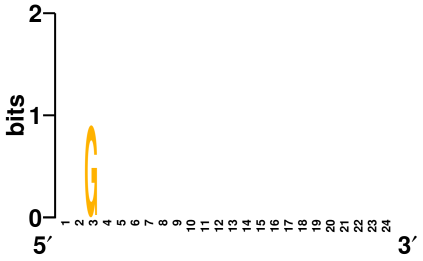

25-mers:

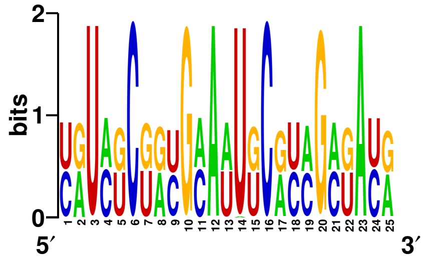

26-mers:

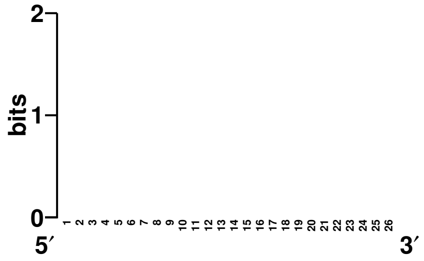

27-mers:

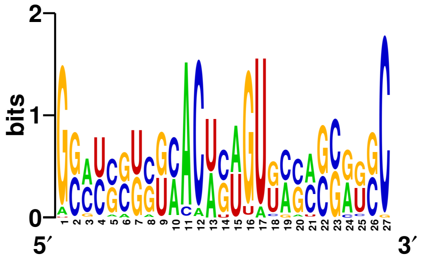

28-mers:

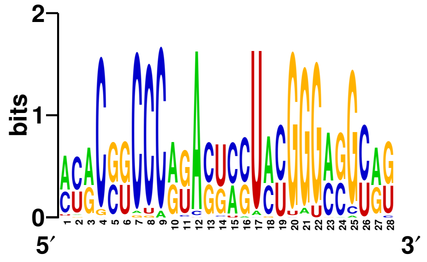

29-mers:

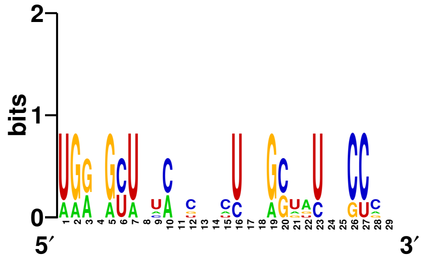

30-mers:

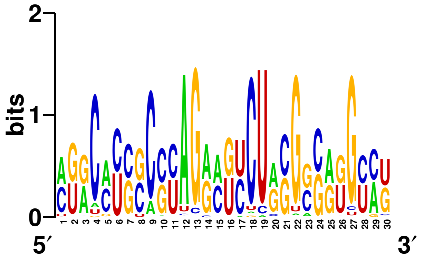

Embryo 36h, library 4:

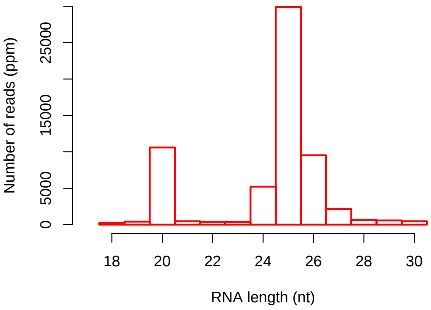

18-mers:

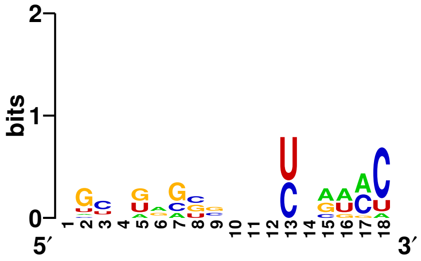

19-mers:

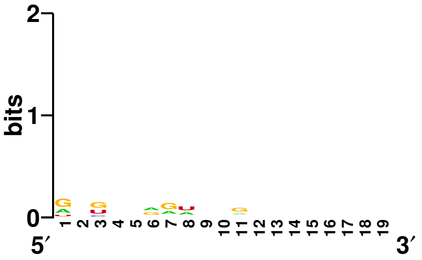

20-mers:

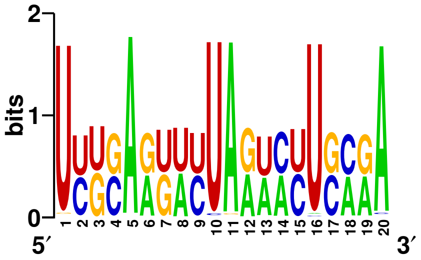

21-mers:

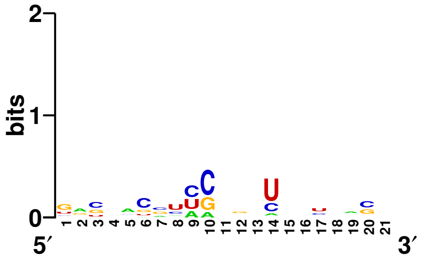

22-mers:

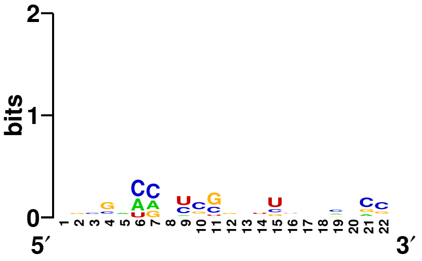

23-mers:

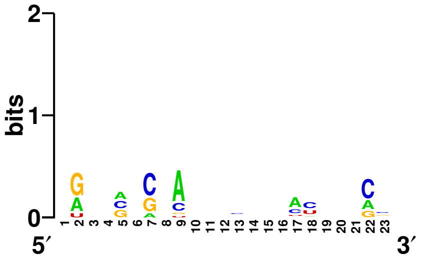

24-mers:

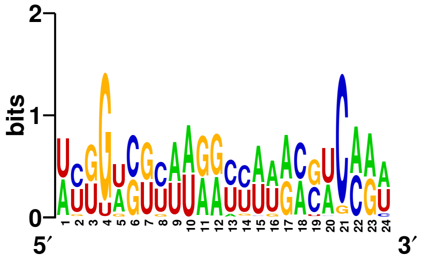

25-mers:

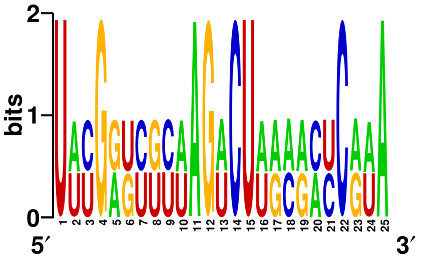

26-mers:

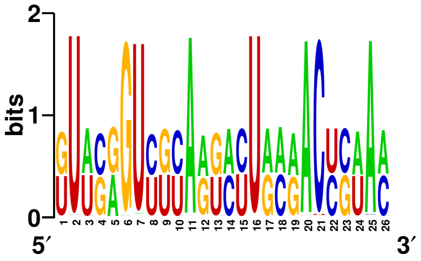

27-mers:

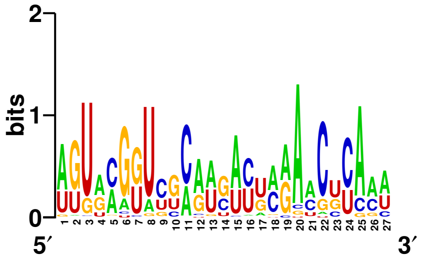

28-mers:

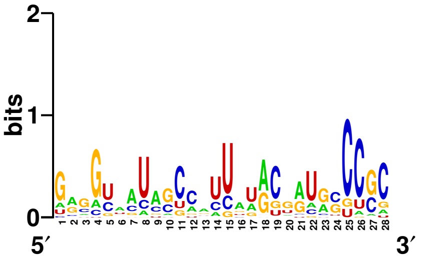

29-mers:

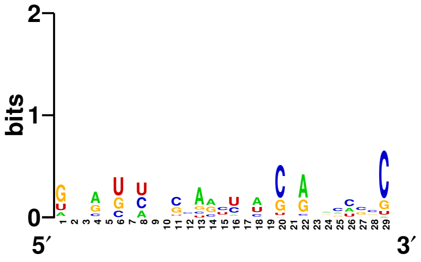

30-mers:

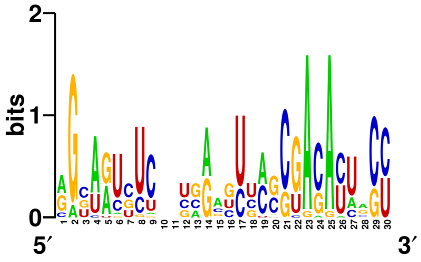

Embryo 60h, library 4:

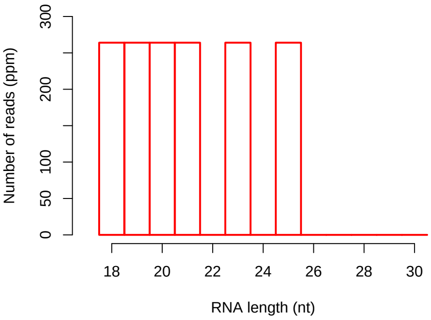

18-mers:

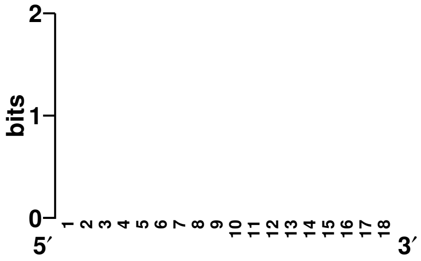

19-mers:

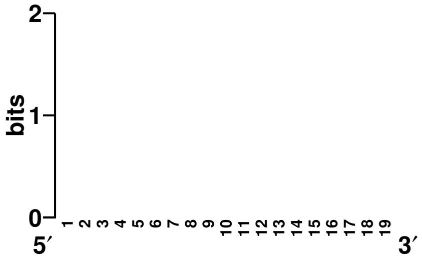

20-mers:

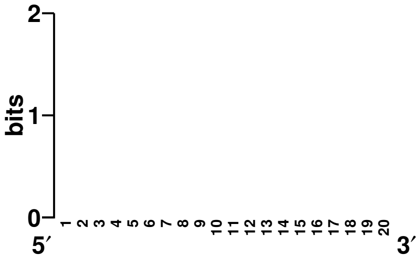

21-mers:

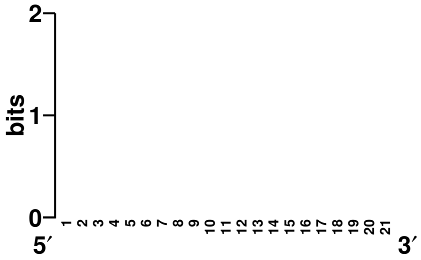

22-mers:

(no read)

23-mers:

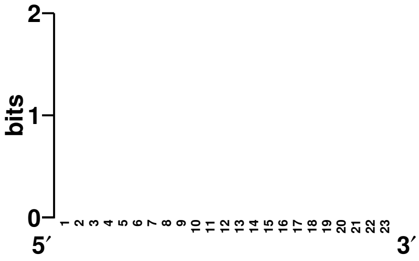

24-mers:

(no read)

25-mers:

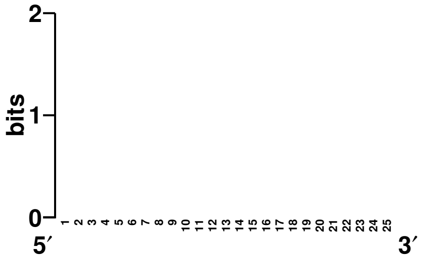

26-mers:

(no read)

27-mers:

(no read)

28-mers:

(no read)

29-mers:

(no read)

30-mers:

(no read)

Adult female, library 4:

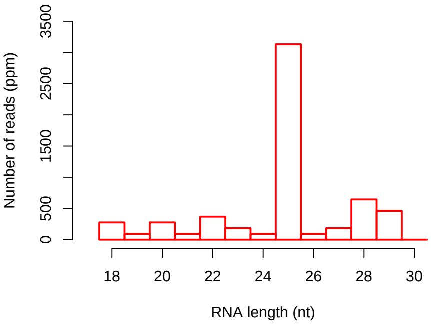

18-mers:

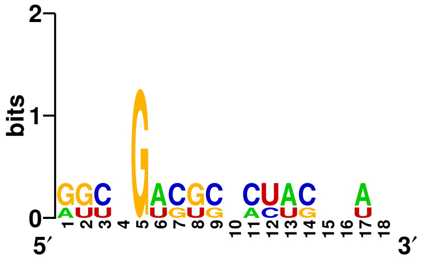

19-mers:

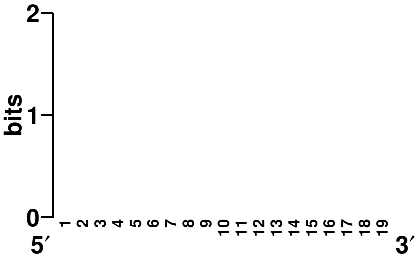

20-mers:

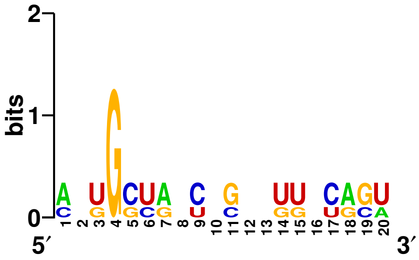

21-mers:

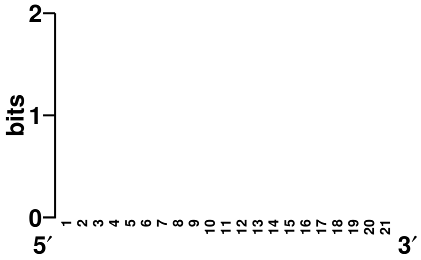

22-mers:

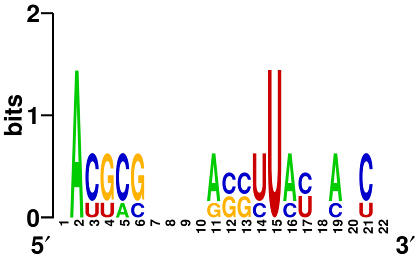

23-mers:

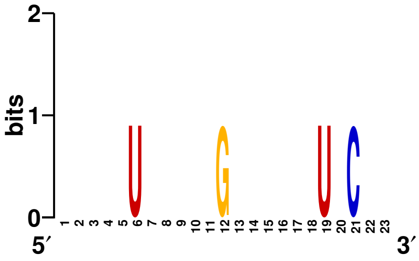

24-mers:

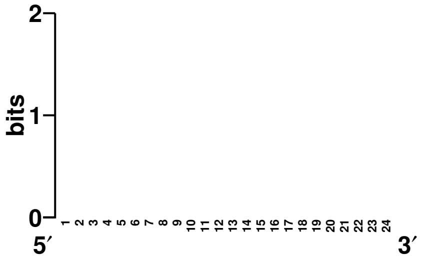

25-mers:

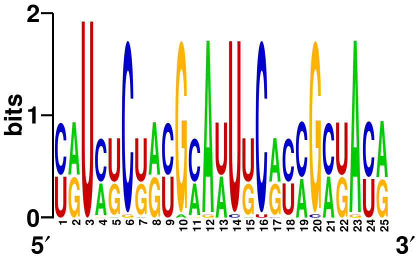

26-mers:

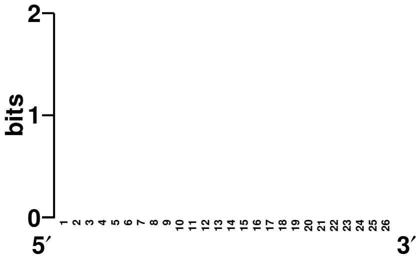

27-mers:

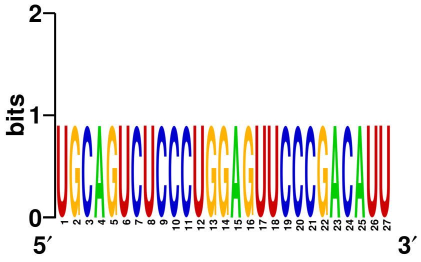

28-mers:

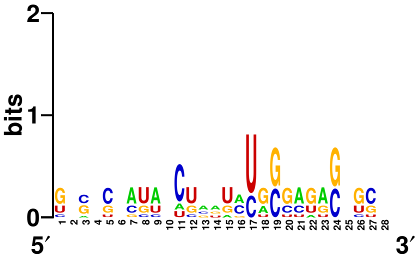

29-mers:

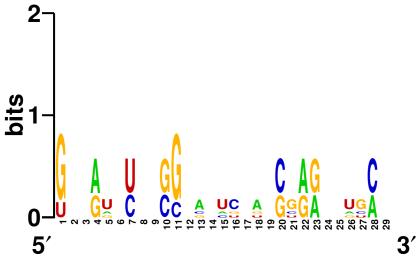

30-mers:

(no read)

Adult male, library 4:

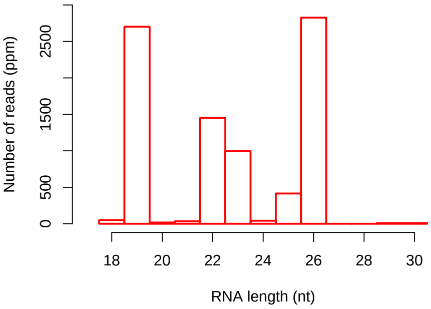

18-mers:

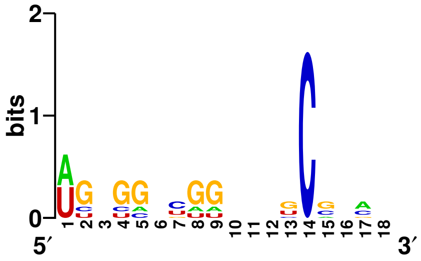

19-mers:

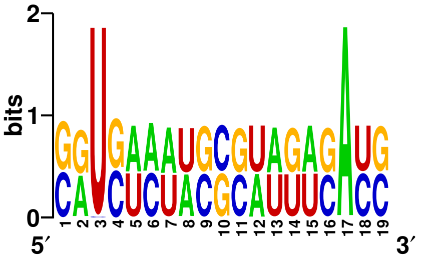

20-mers:

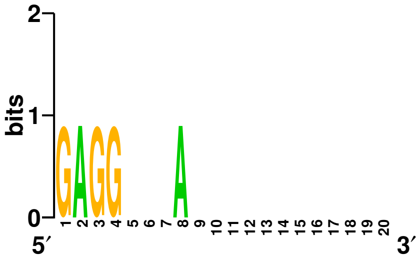

21-mers:

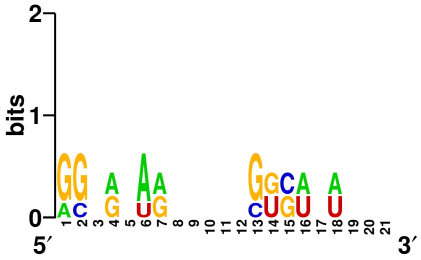

22-mers:

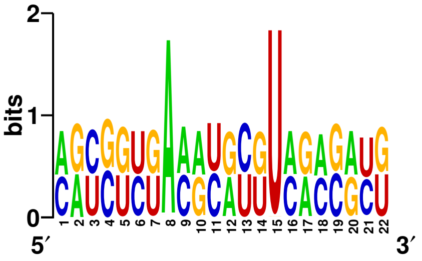

23-mers:

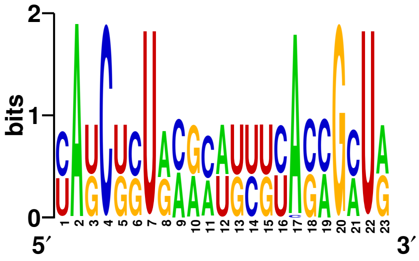

24-mers:

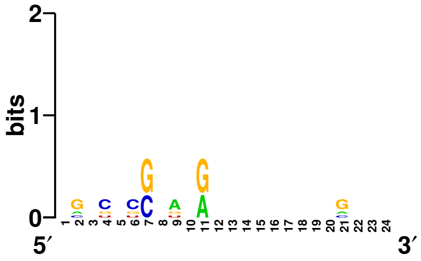

25-mers:

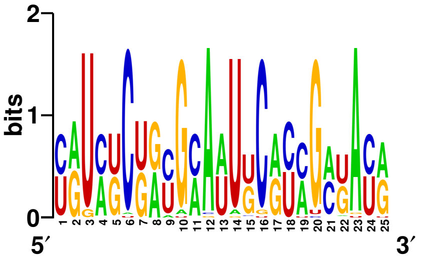

26-mers:

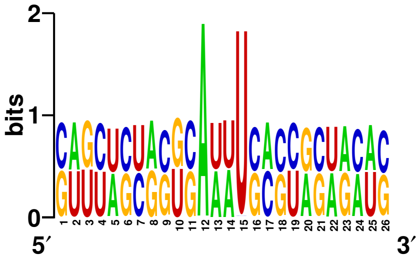

27-mers:

(no read)

28-mers:

(no read)

29-mers:

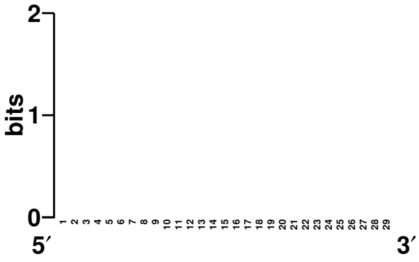

30-mers:

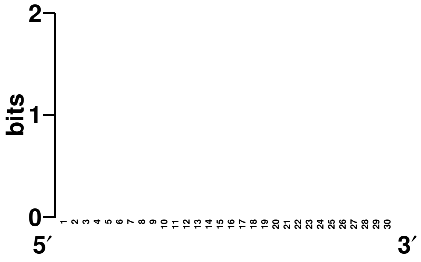

9 Extragenomic and extratranscriptomic reads matching the *Vibrio anguillarum* genome

9.1 Libraries #1 (total 5´ monophosphorylated small RNAs)

Embryo 8h, library 1:

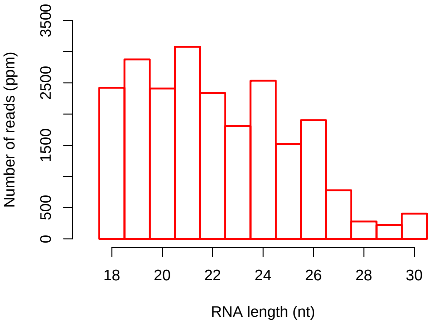

18-mers:

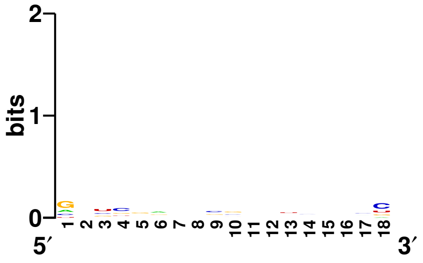

19-mers:

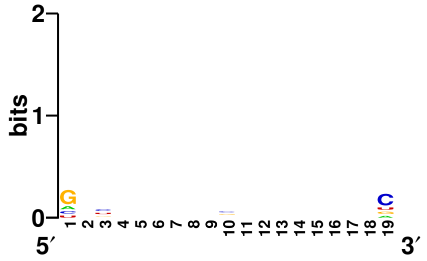

20-mers:

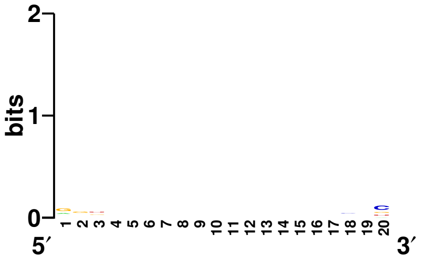

21-mers:

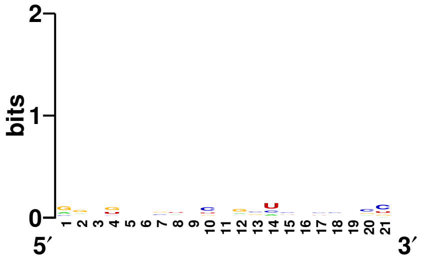

22-mers:

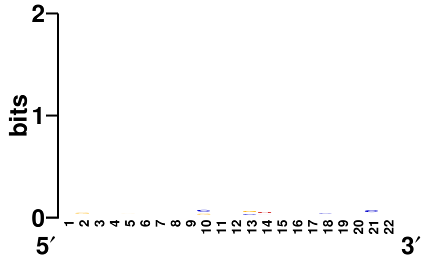

23-mers:

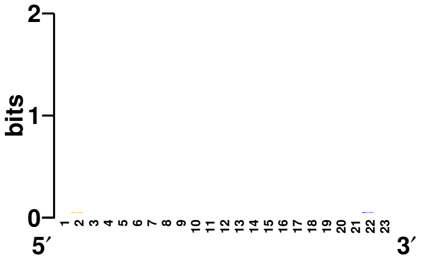

24-mers:

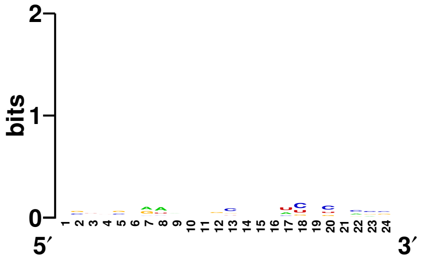

25-mers:

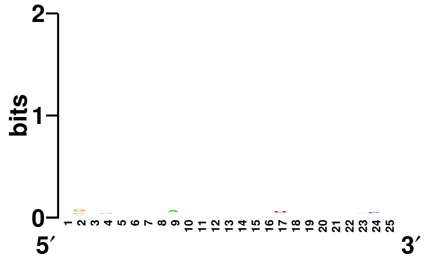

26-mers:

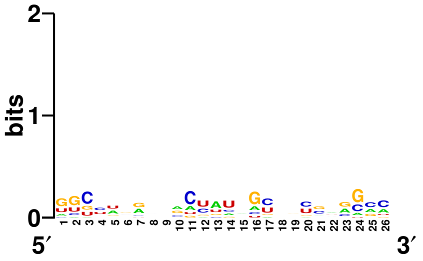

27-mers:

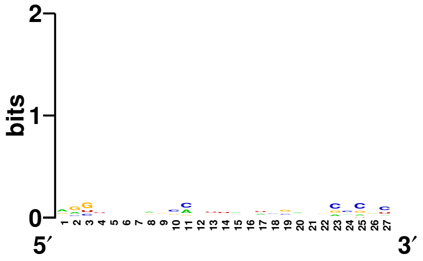

28-mers:

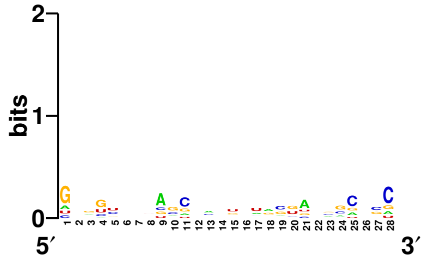

29-mers:

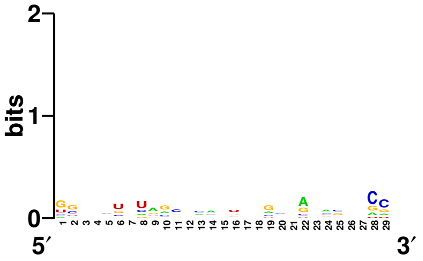

30-mers:

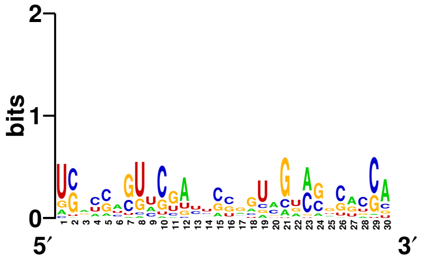

Embryo 15h, library 1:

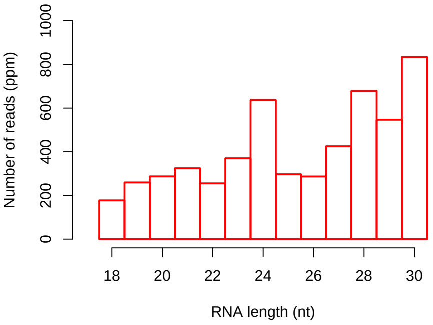

18-mers:

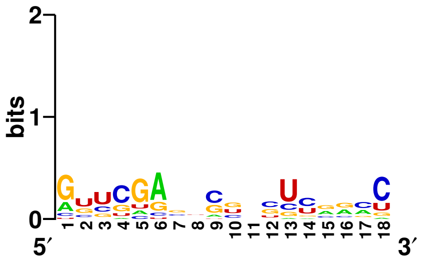

19-mers:

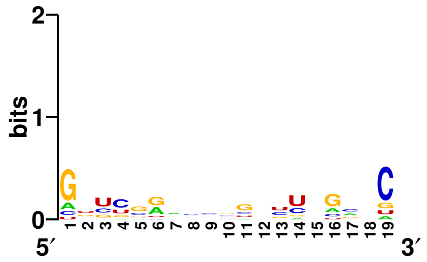

20-mers:

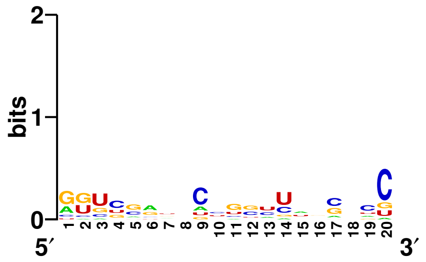

21-mers:

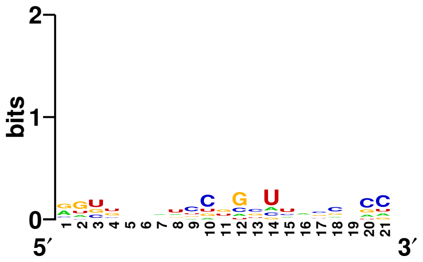

22-mers:

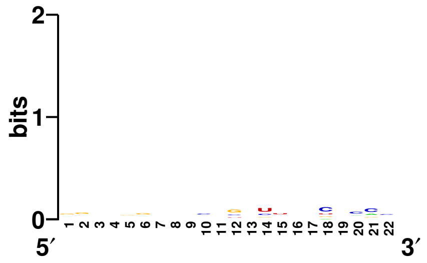

23-mers:

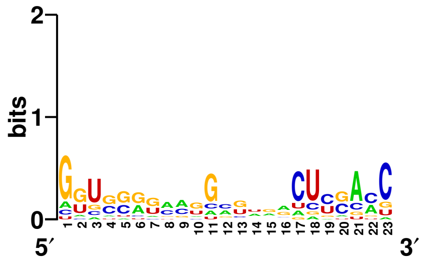

24-mers:

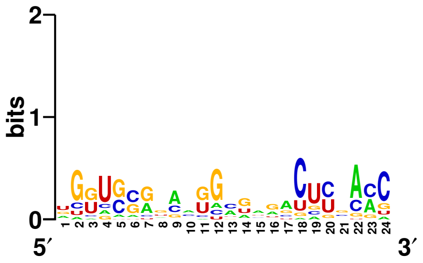

25-mers:

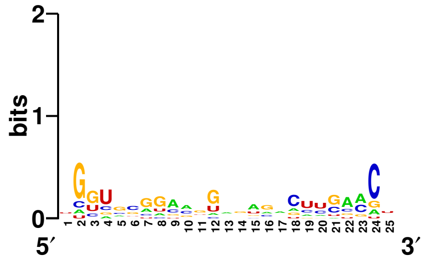

26-mers:

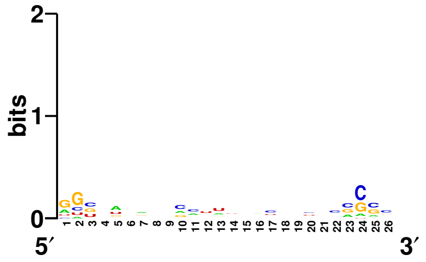

27-mers:

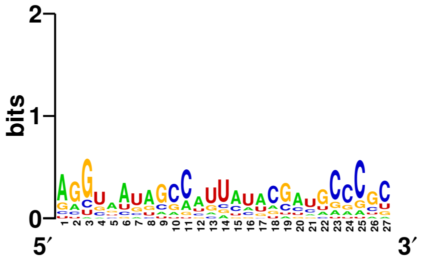

28-mers:

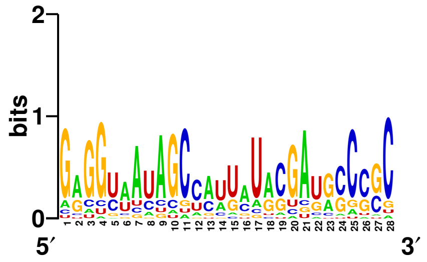

29-mers:

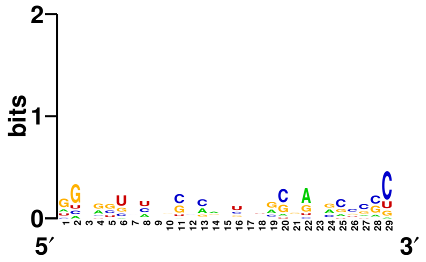

30-mers:

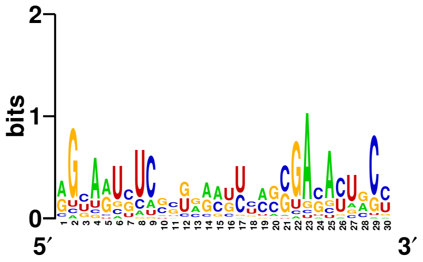

Embryo 36h, library 1:

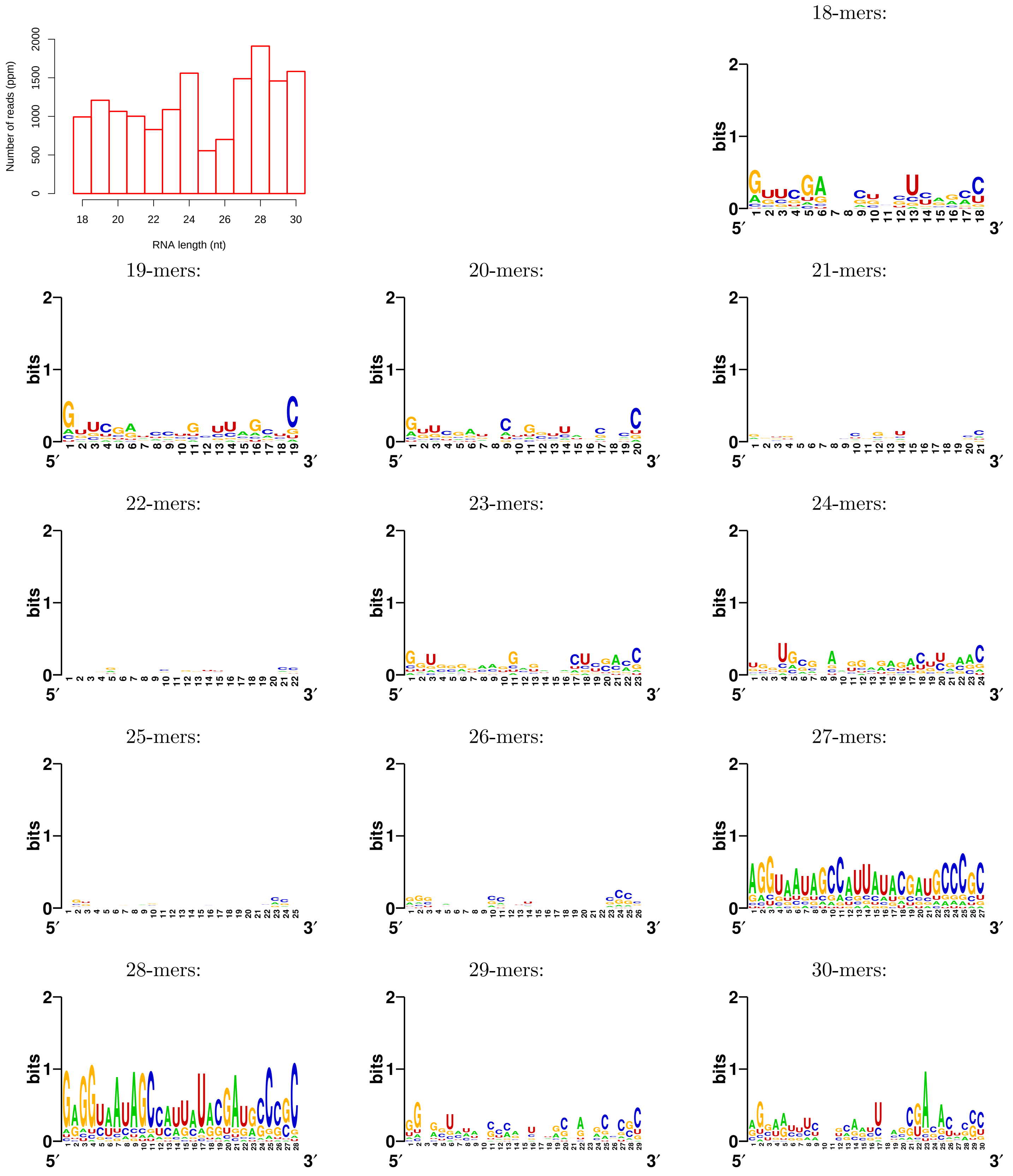

Embryo 60h, library 1:

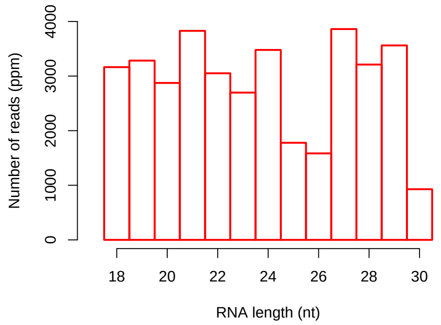

18-mers:

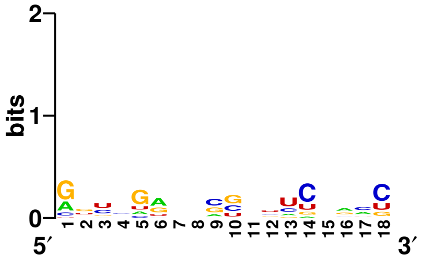

19-mers:

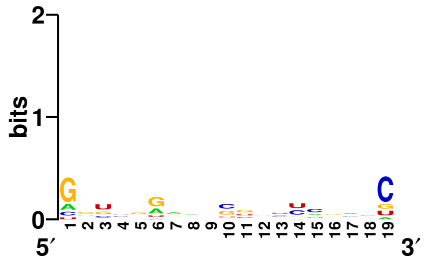

20-mers:

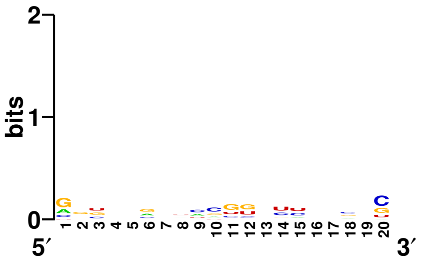

21-mers:

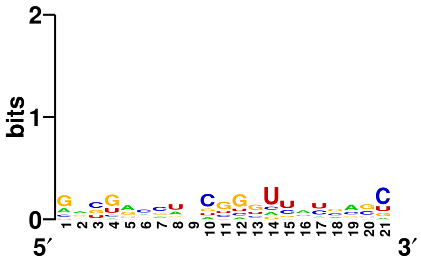

22-mers:

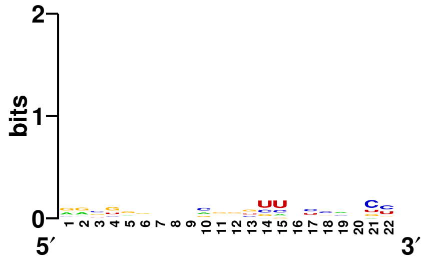

23-mers:

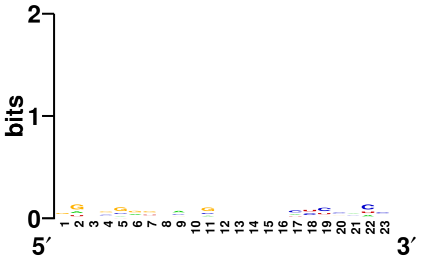

24-mers:

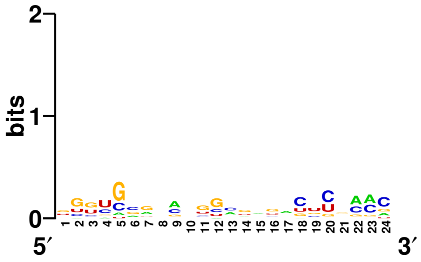

25-mers:

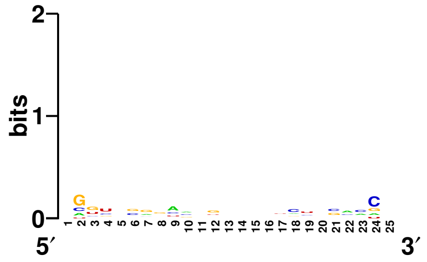

26-mers:

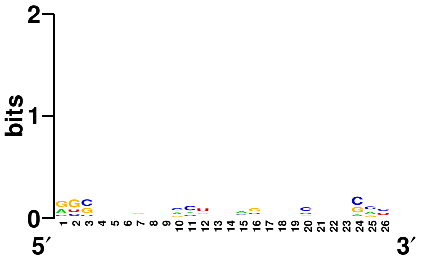

27-mers:

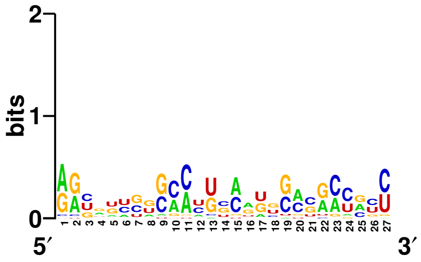

28-mers:

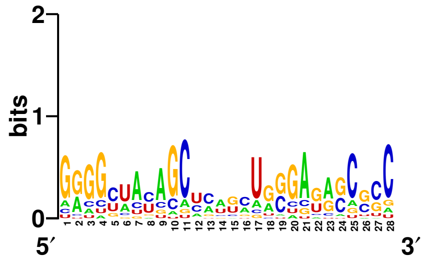

29-mers:

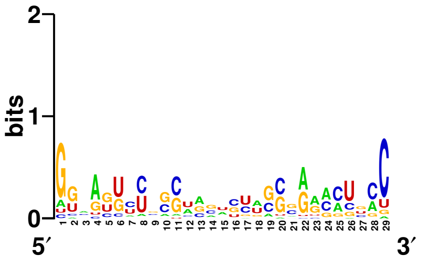

30-mers:

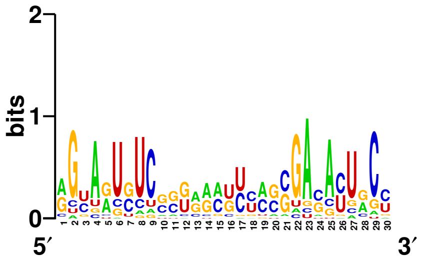

Adult female, library 1:

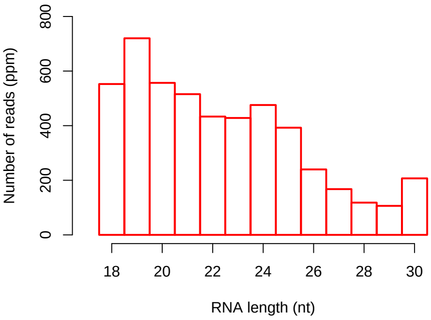

18-mers:

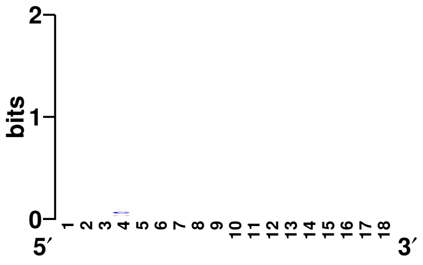

19-mers:

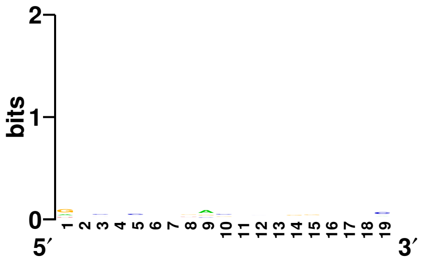

20-mers:

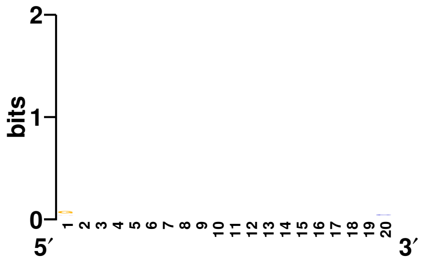

21-mers:

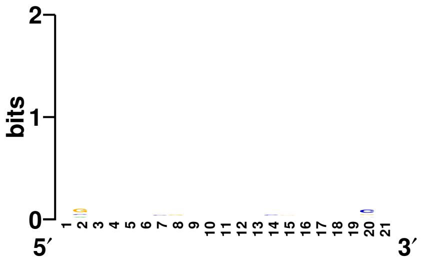

22-mers:

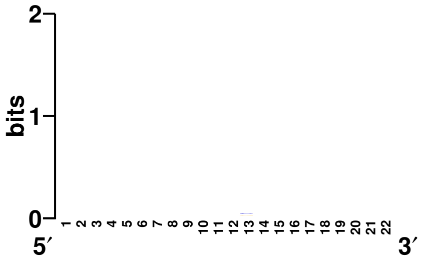

23-mers:

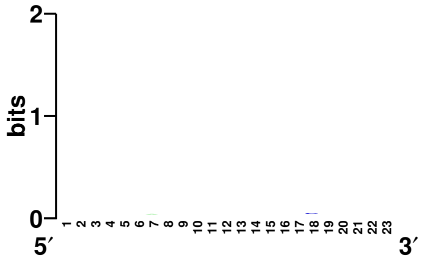

24-mers:

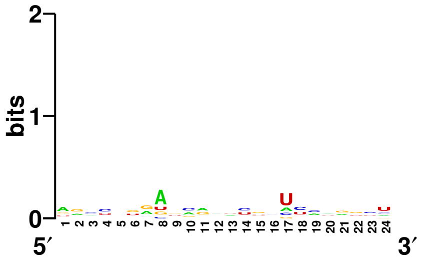

25-mers:

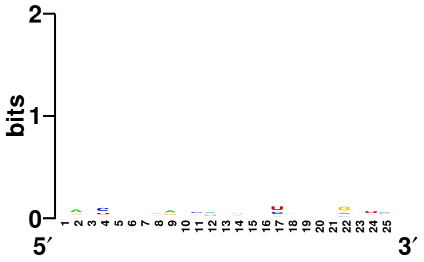

26-mers:

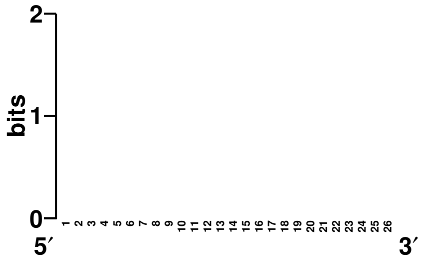

27-mers:

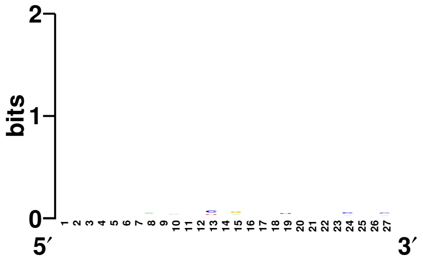

28-mers:

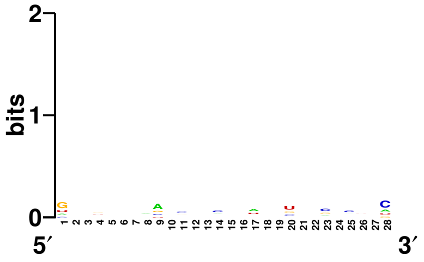

29-mers:

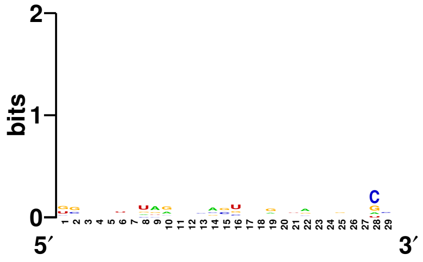

30-mers:

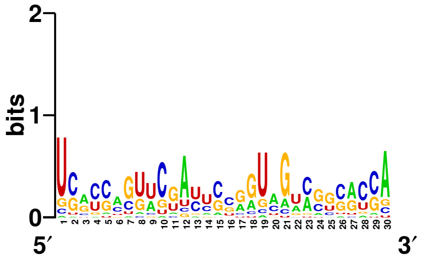

Adult male, library 1:

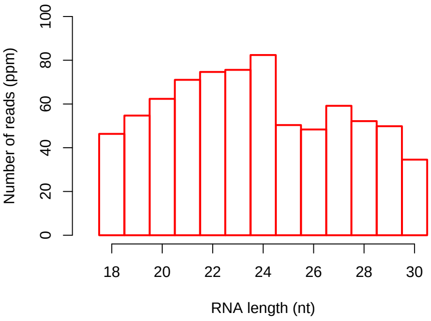

18-mers:

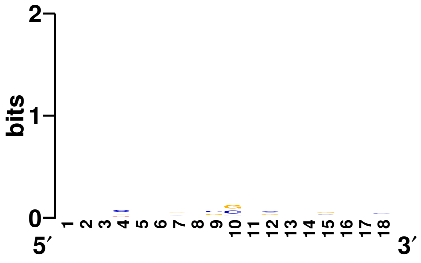

19-mers:

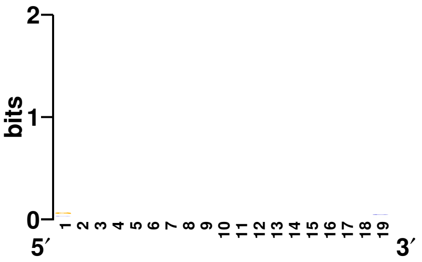

20-mers:

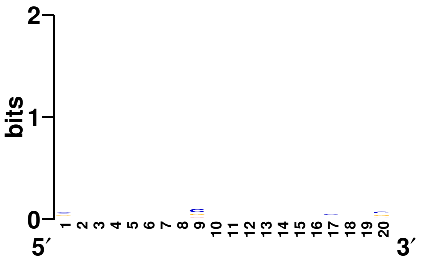

21-mers:

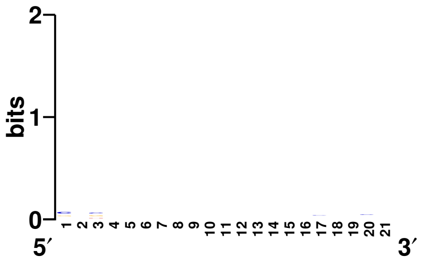

22-mers:

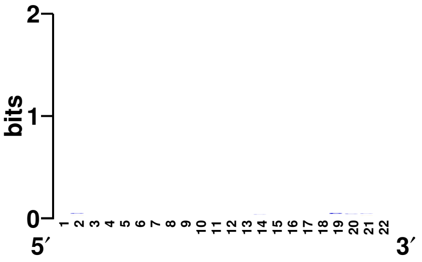

23-mers:

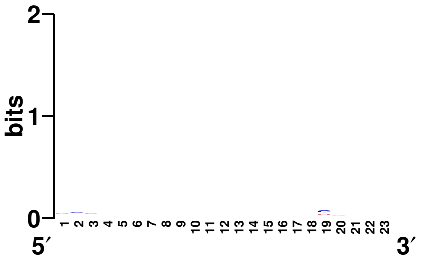

24-mers:

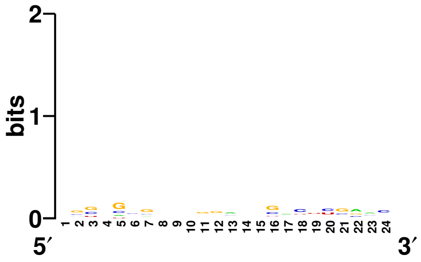

25-mers:

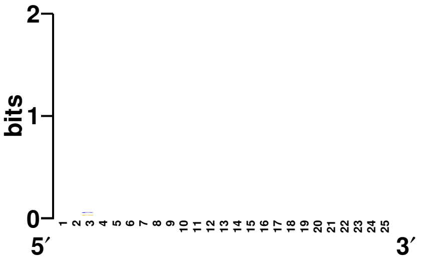

26-mers:

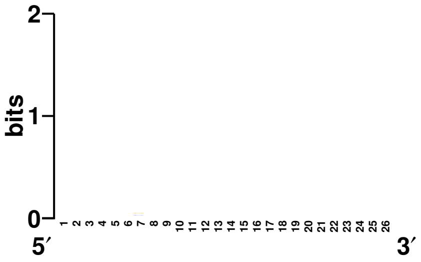

27-mers:

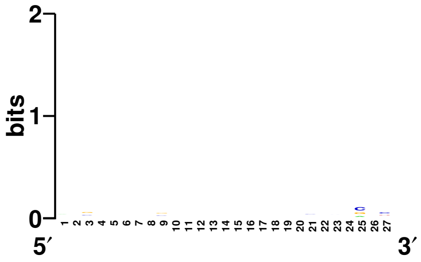

28-mers:

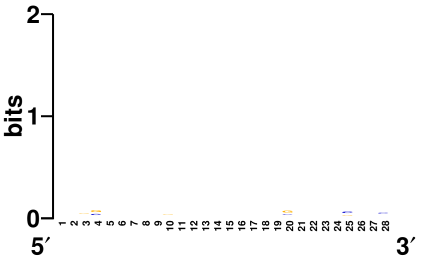

29-mers:

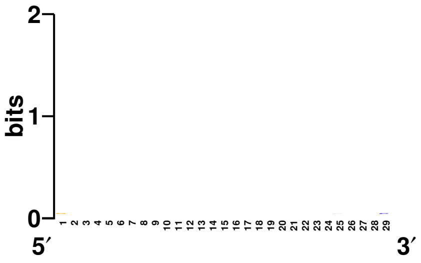

30-mers:

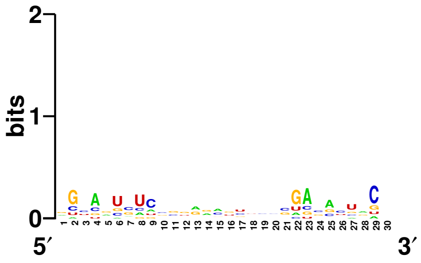

9.2 Libraries #2 (3´ modified, 5´ monophosphorylated small RNAs)

Embryo 8h, library 2:

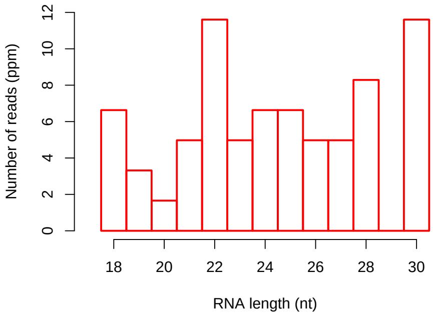

18-mers:

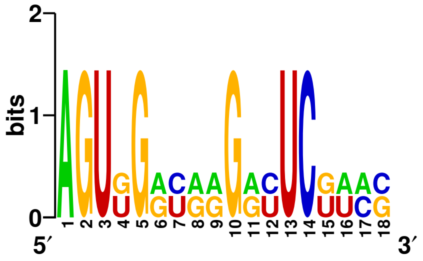

19-mers:

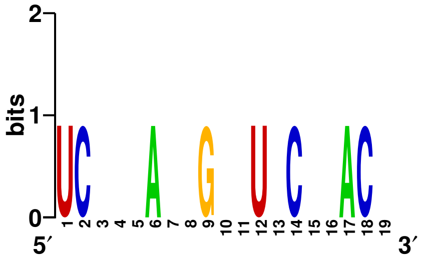

20-mers:

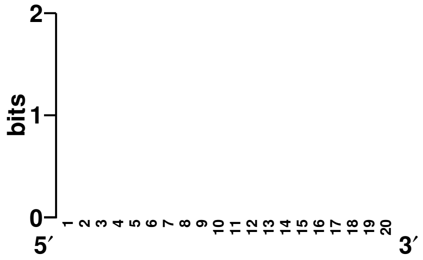

21-mers:

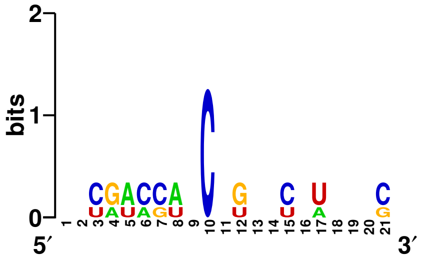

22-mers:

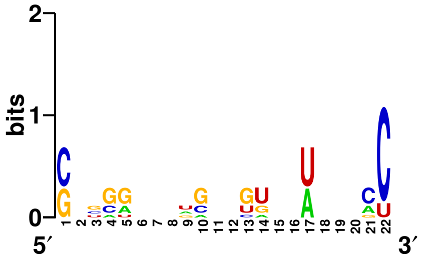

23-mers:

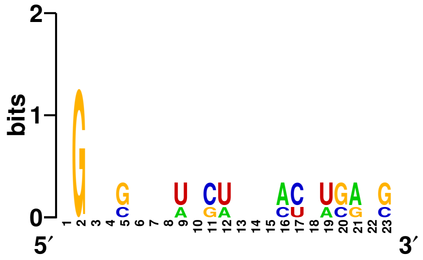

24-mers:

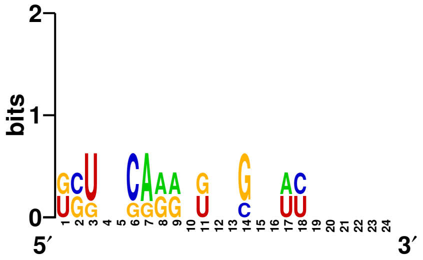

25-mers:

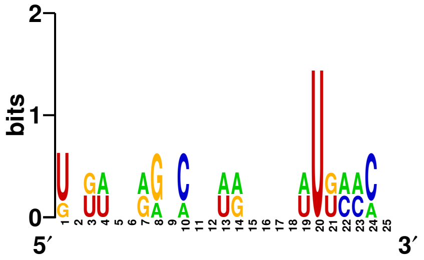

26-mers:

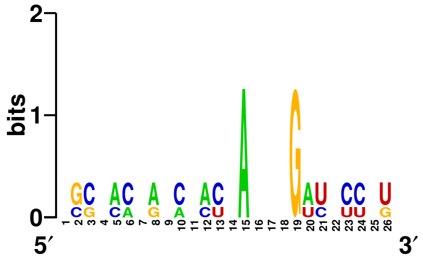

27-mers:

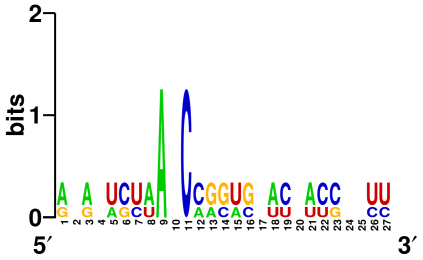

28-mers:

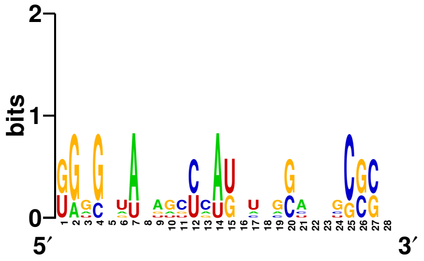

29-mers:

(no read)

30-mers:

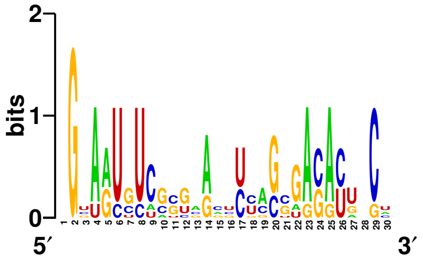

Embryo 15h, library 2:

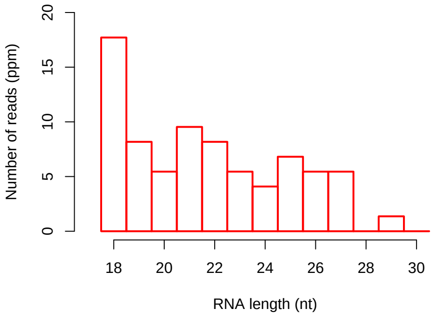

18-mers:

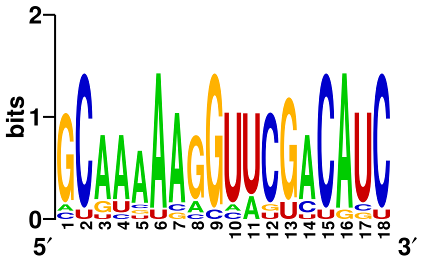

19-mers:

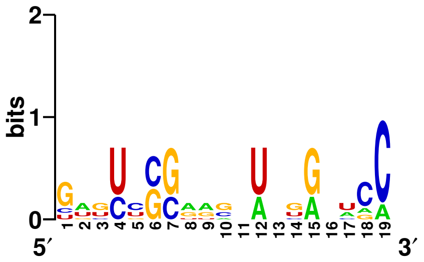

20-mers:

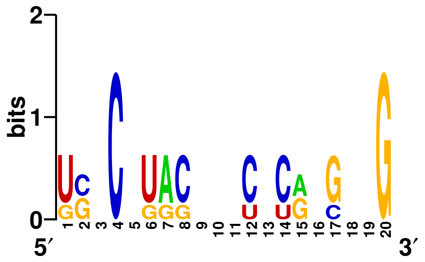

21-mers:

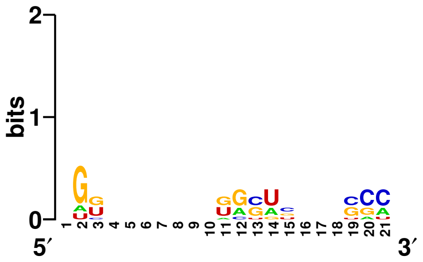

22-mers:

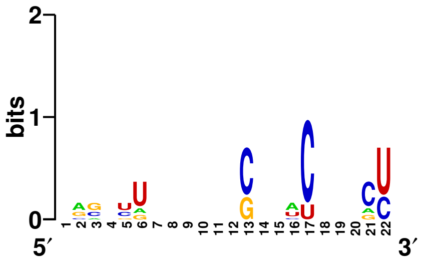

23-mers:

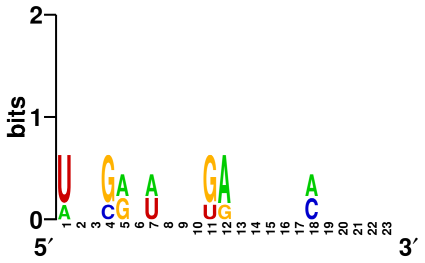

24-mers:

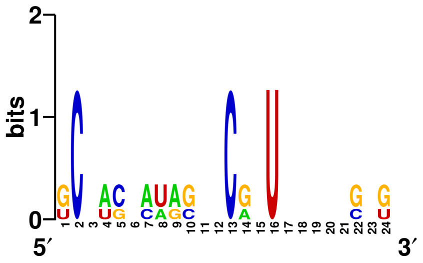

25-mers:

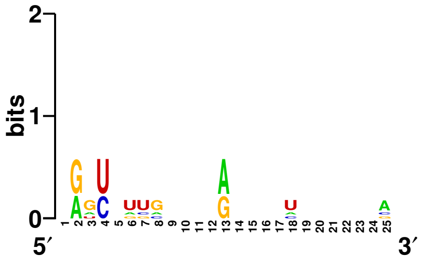

26-mers:

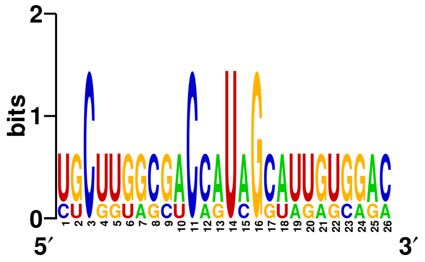

27-mers:

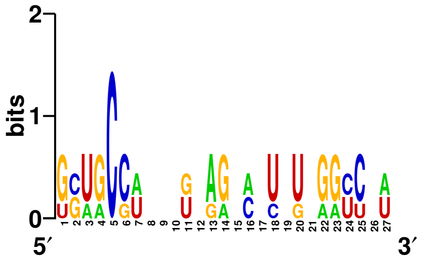

28-mers:

(no read)

29-mers:

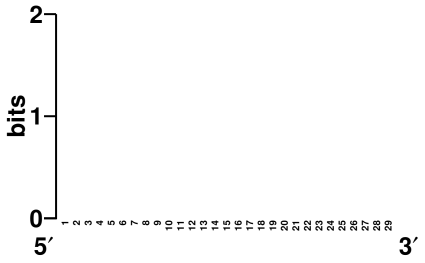

30-mers:

(no read)

Embryo 36h, library 2:

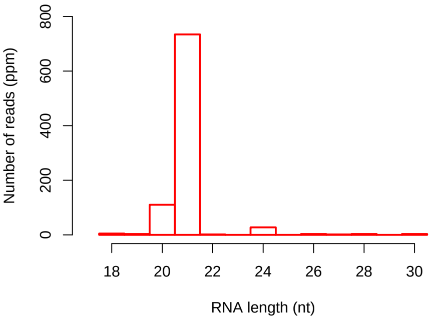

18-mers:

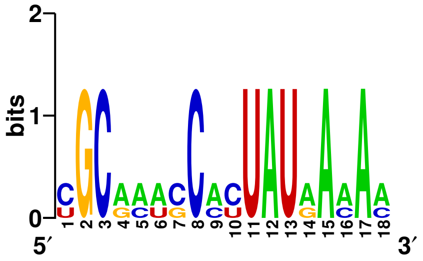

19-mers:

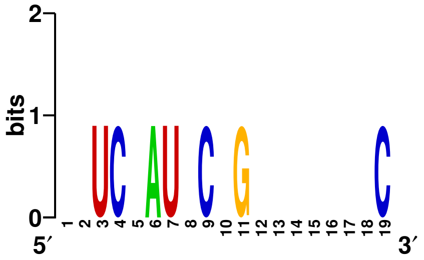

20-mers:

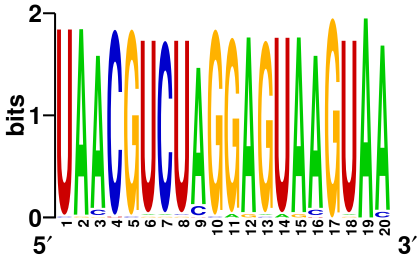

21-mers:

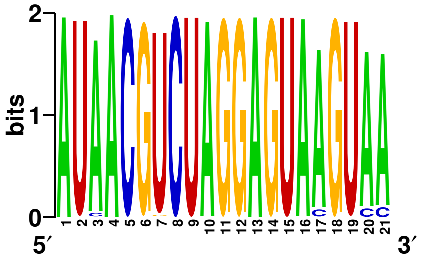

22-mers:

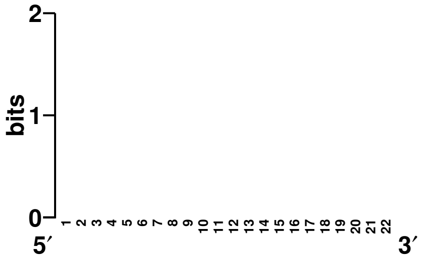

23-mers:

(no read)

24-mers:

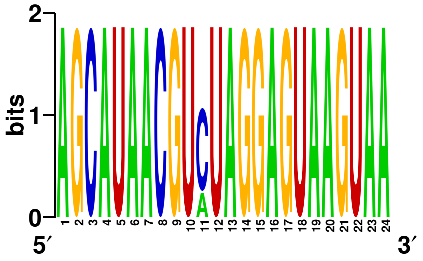

25-mers:

(no read)

26-mers:

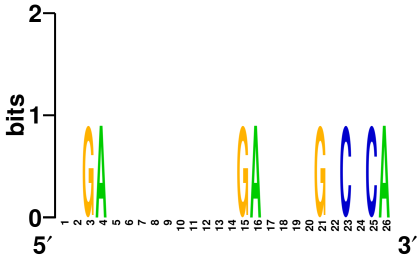

27-mers:

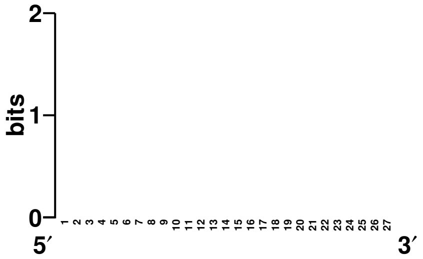

28-mers:

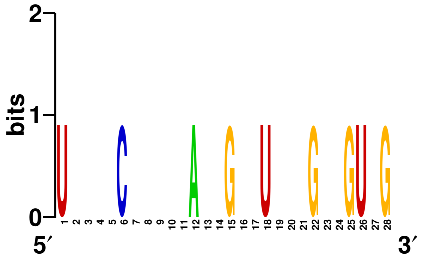

29-mers:

(no read)

30-mers:

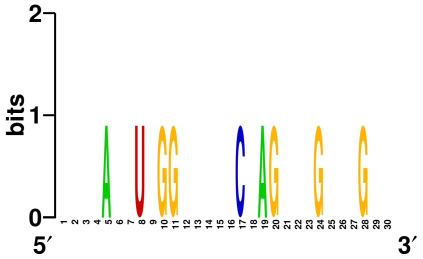

Embryo 60h, library 2:

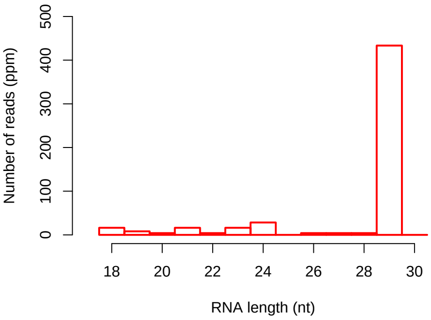

18-mers:

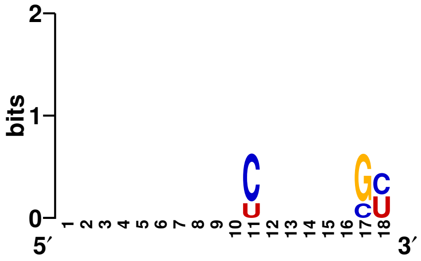

19-mers:

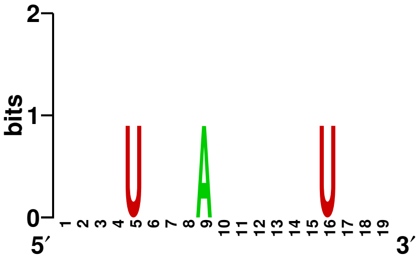

20-mers:

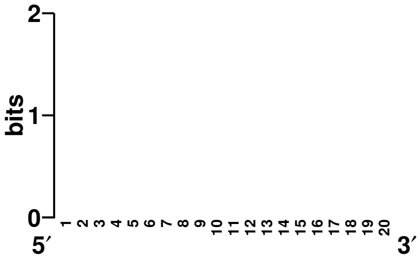

21-mers:

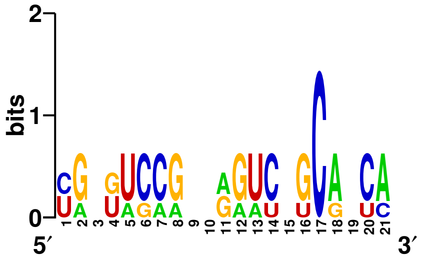

22-mers:

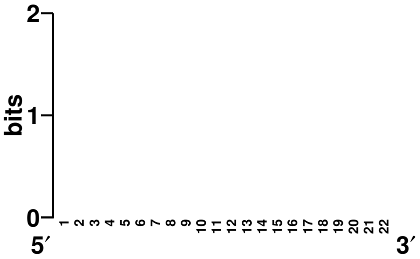

23-mers:

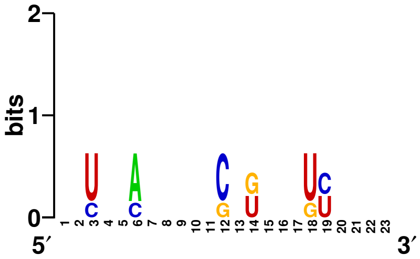

24-mers:

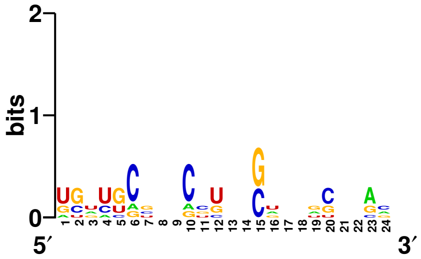

25-mers:

(no read)

26-mers:

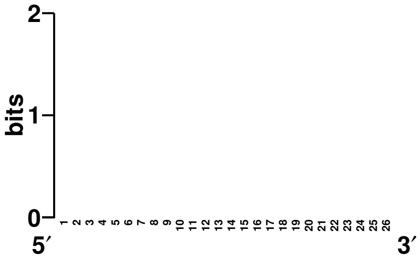

27-mers:

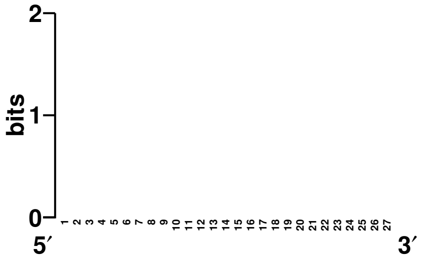

28-mers:

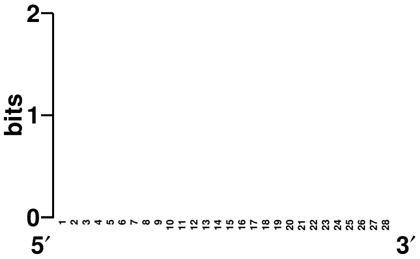

29-mers:

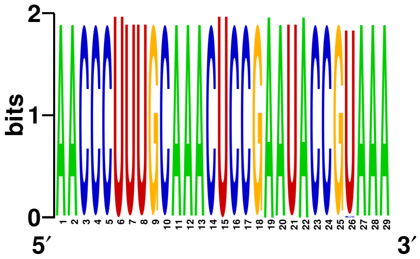

30-mers:

(no read)

Adult female, library 2:

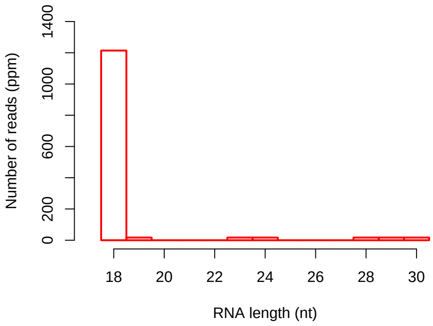

18-mers:

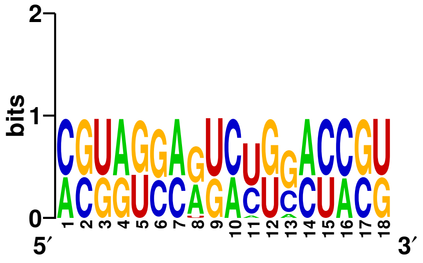

19-mers:

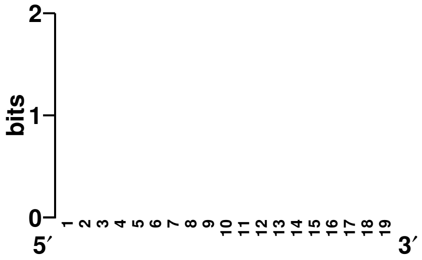

20-mers:

(no read)

21-mers:

(no read)

22-mers:

(no read)

23-mers:

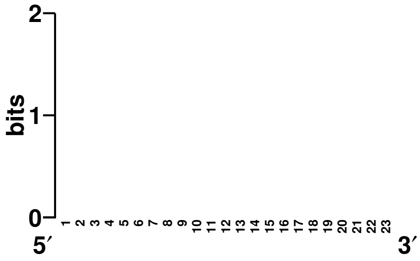

24-mers:

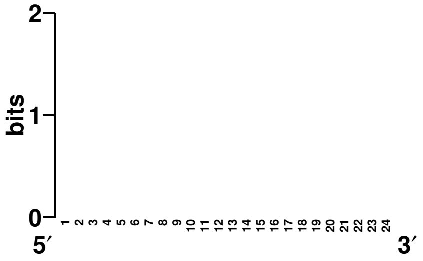

25-mers:

(no read)

26-mers:

(no read)

27-mers:

(no read)

28-mers:

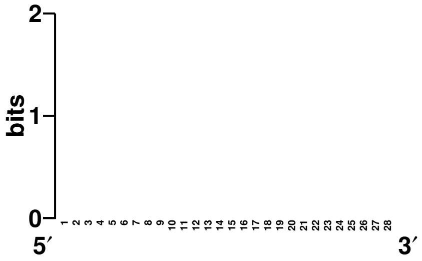

29-mers:

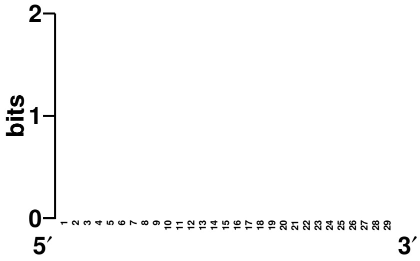

30-mers:

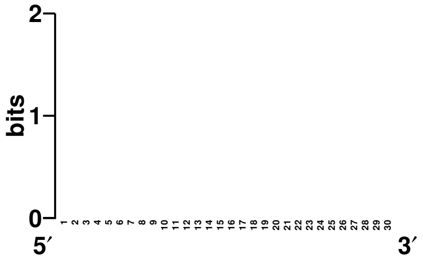

Adult male, library 2:

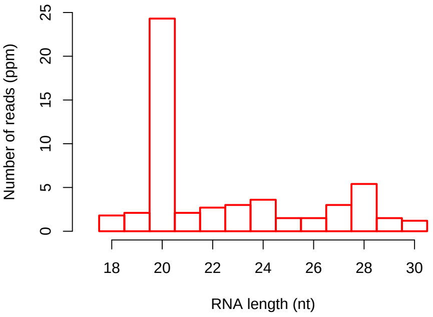

18-mers:

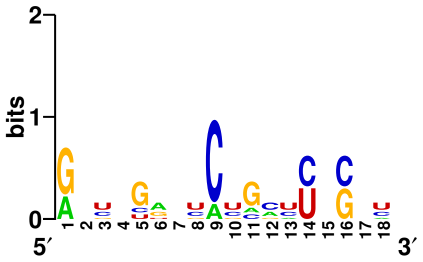

19-mers:

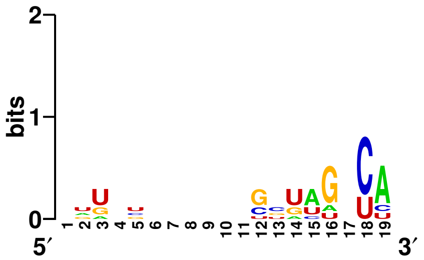

20-mers:

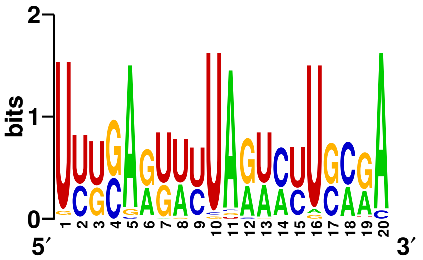

21-mers:

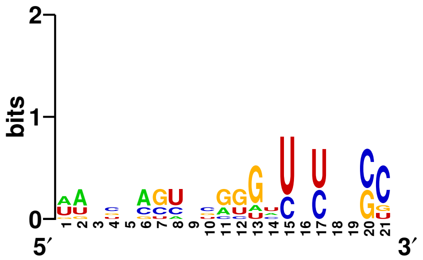

22-mers:

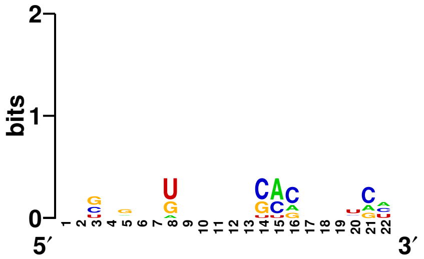

23-mers:

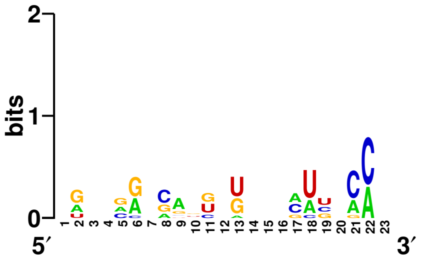

24-mers:

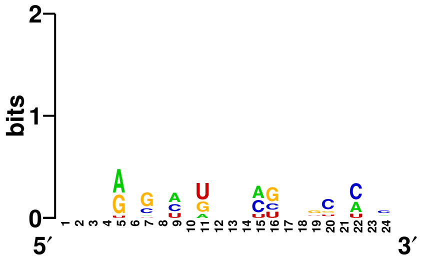

25-mers:

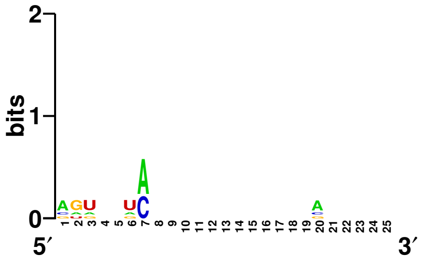

26-mers:

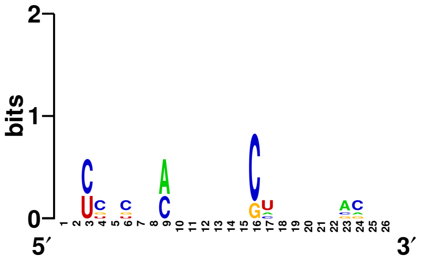

27-mers:

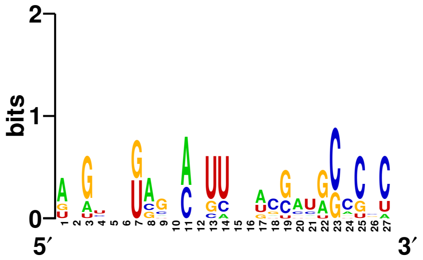

28-mers:

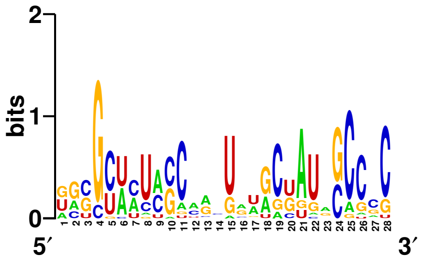

29-mers:

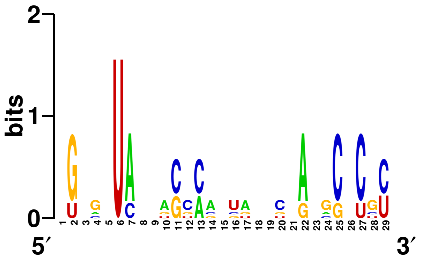

30-mers:

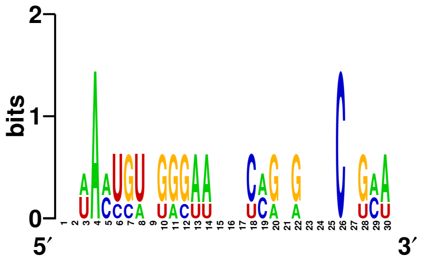

9.3 Libraries #3 (total 5´ hydroxyl or polyphosphorylated small RNAs)

Embryo 8h, library 3:

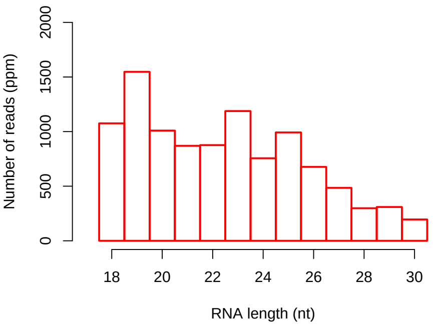

18-mers:

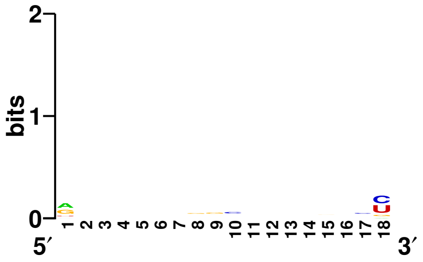

19-mers:

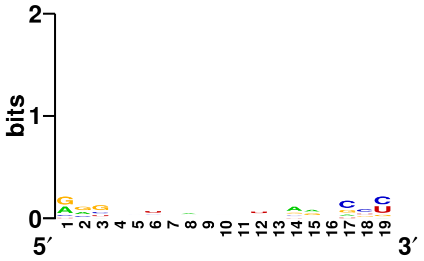

20-mers:

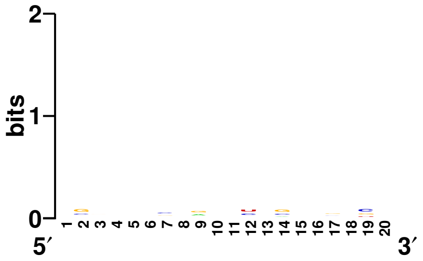

21-mers:

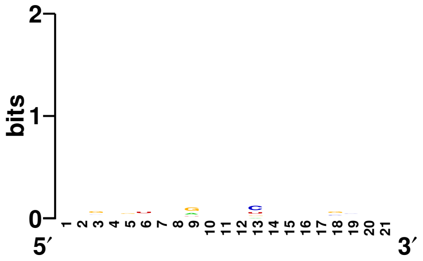

22-mers:

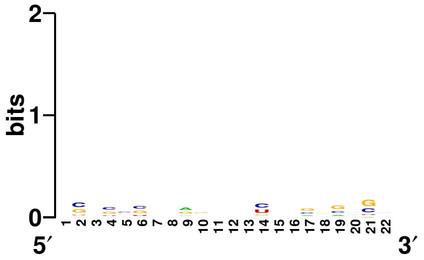

23-mers:

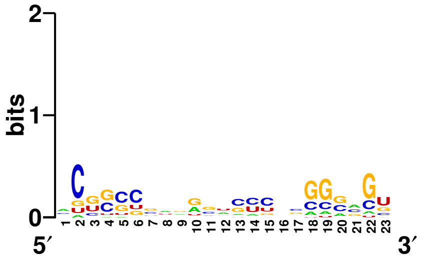

24-mers:

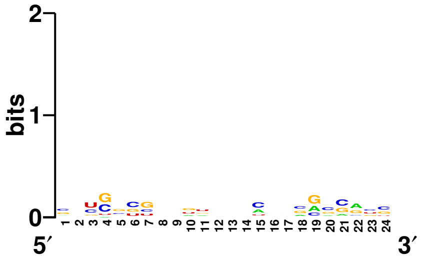

25-mers:

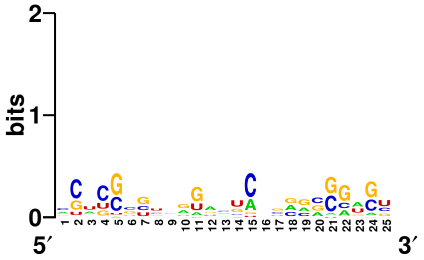

26-mers:

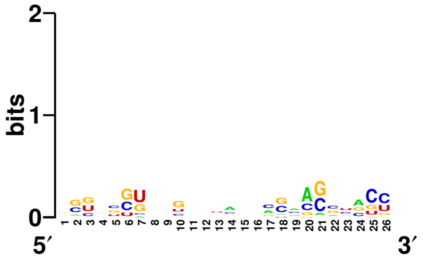

27-mers:

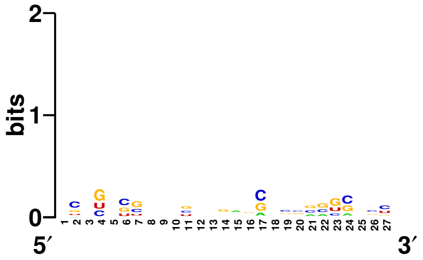

28-mers:

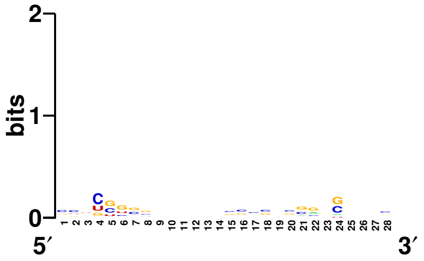

29-mers:

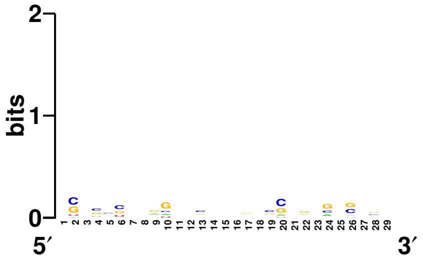

30-mers:

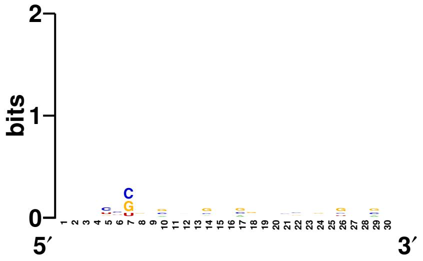

Embryo 15h, library 3:

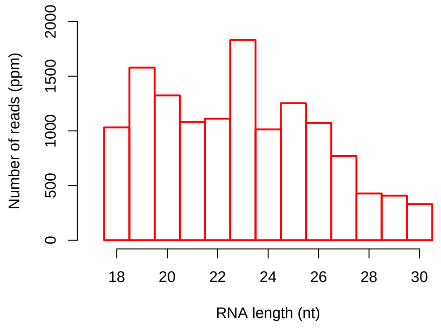

18-mers:

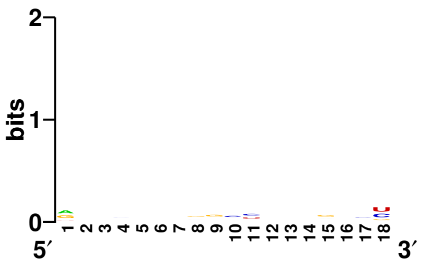

19-mers:

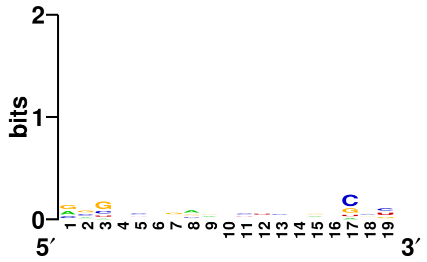

20-mers:

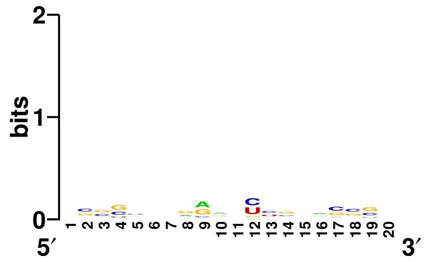

21-mers:

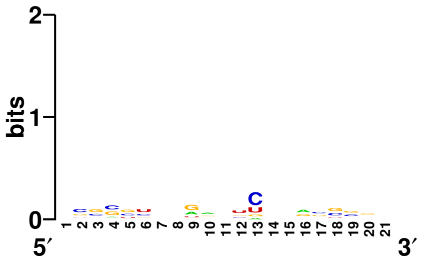

22-mers:

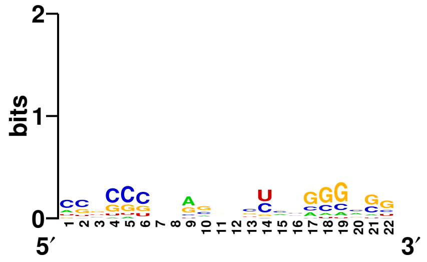

23-mers:

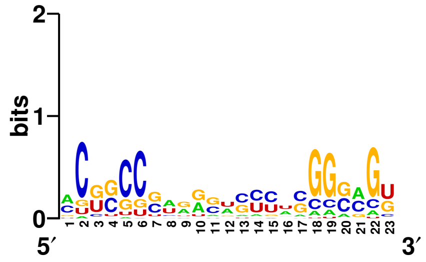

24-mers:

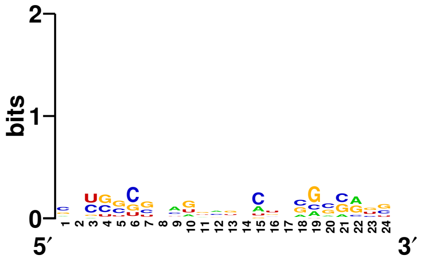

25-mers:

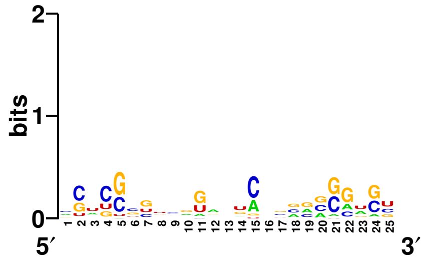

26-mers:

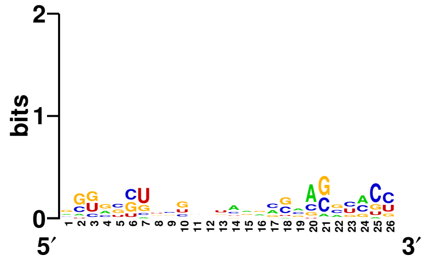

27-mers:

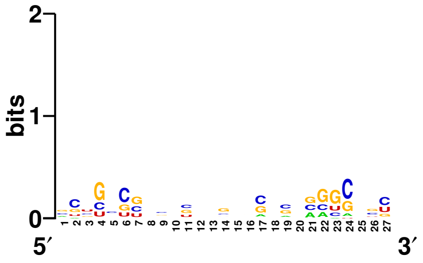

28-mers:

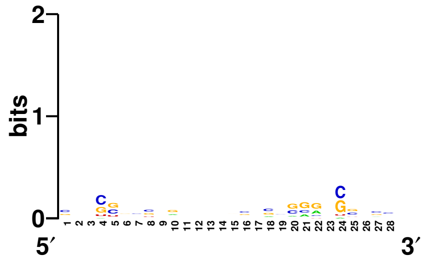

29-mers:

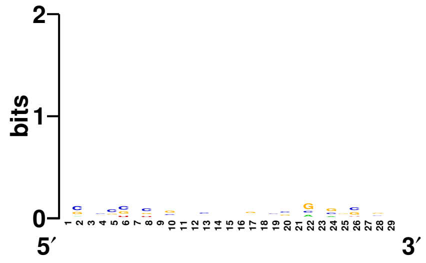

30-mers:

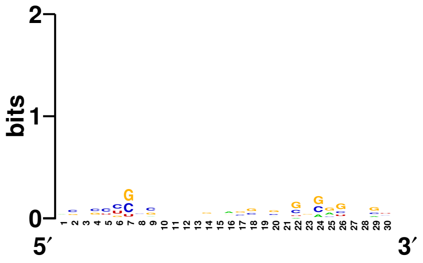

Embryo 36h, library 3:

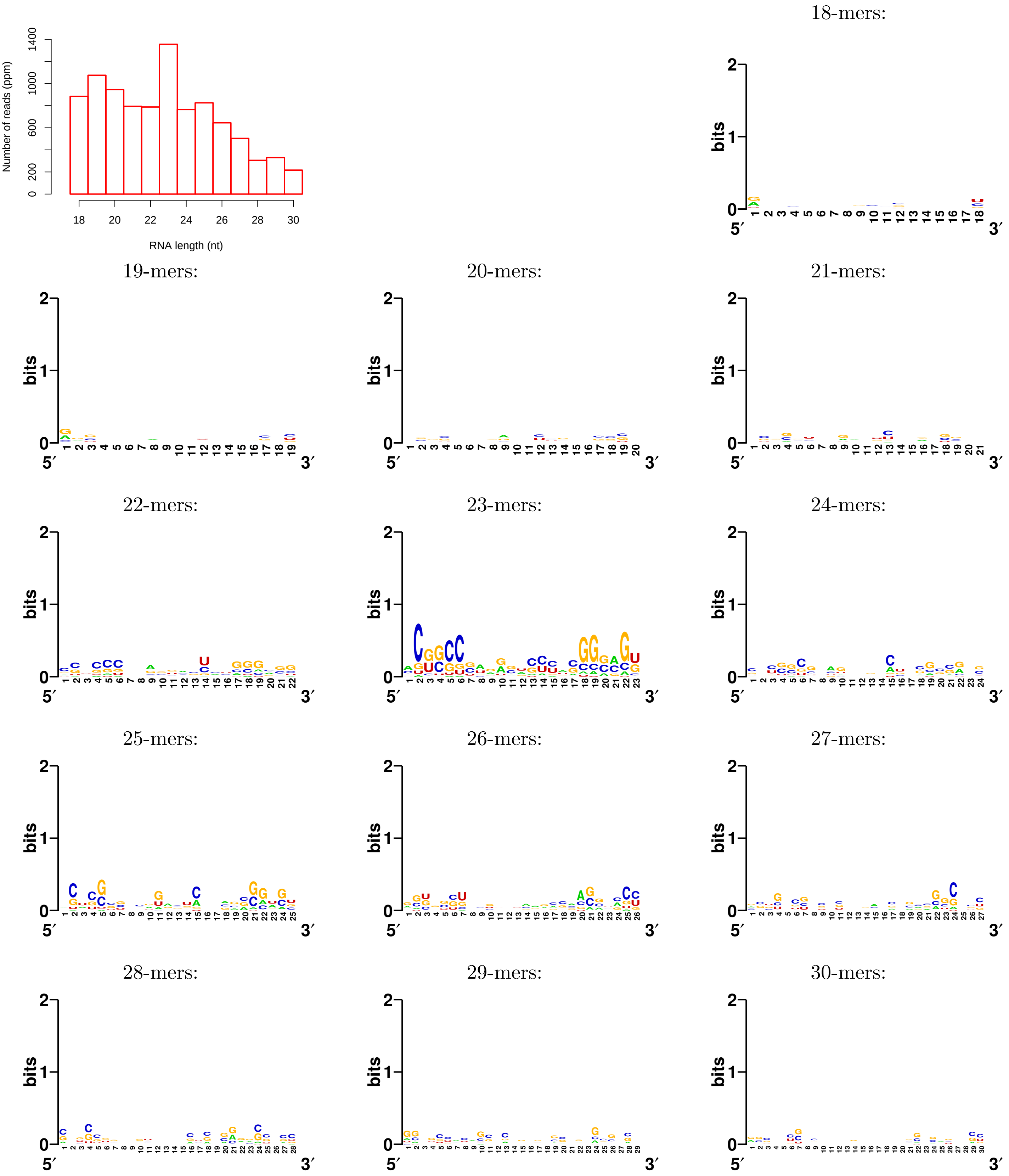

Embryo 60h, library 3:

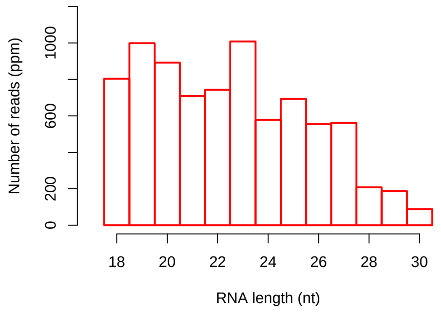

18-mers:

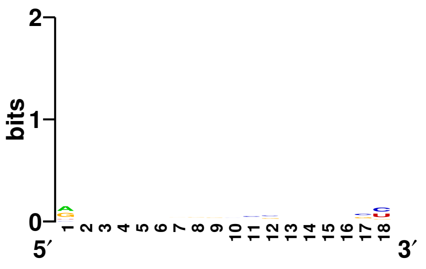

19-mers:

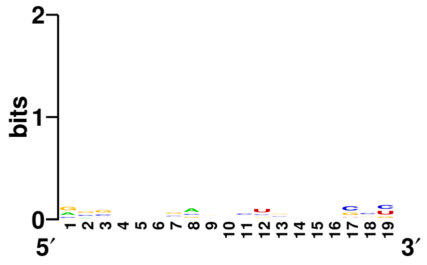

20-mers:

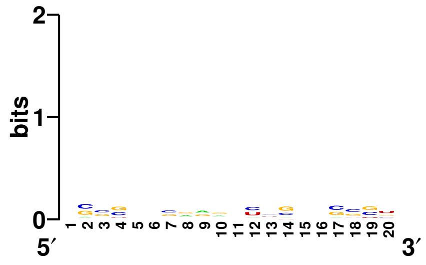

21-mers:

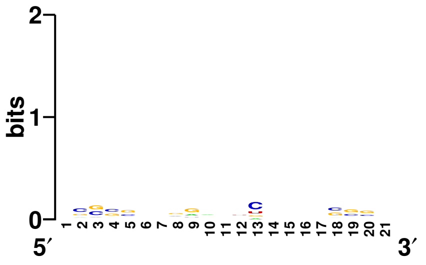

22-mers:

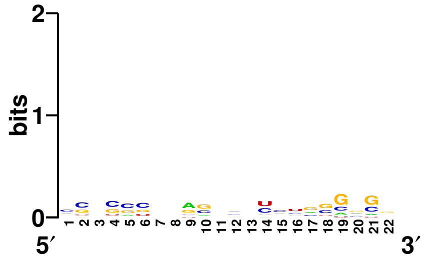

23-mers:

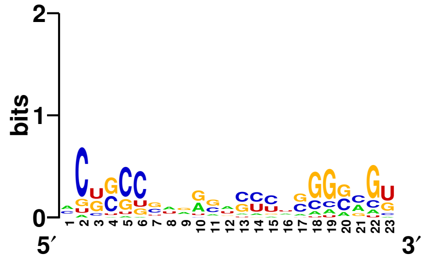

24-mers:

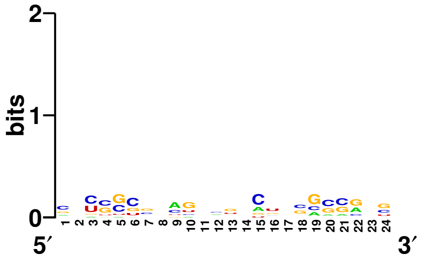

25-mers:

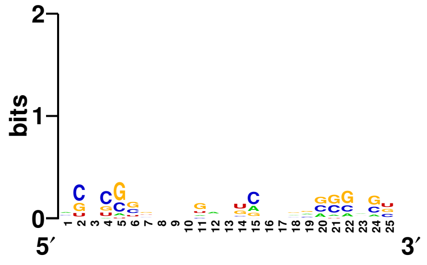

26-mers:

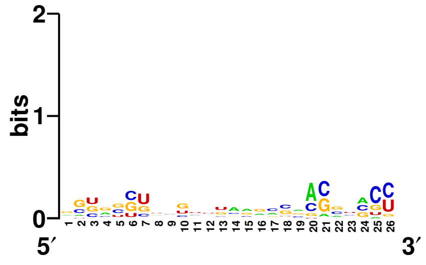

27-mers:

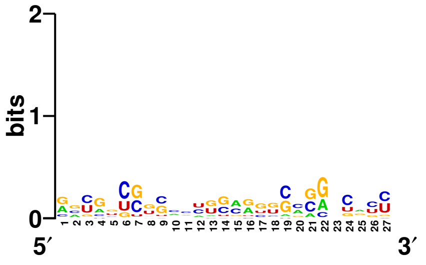

28-mers:

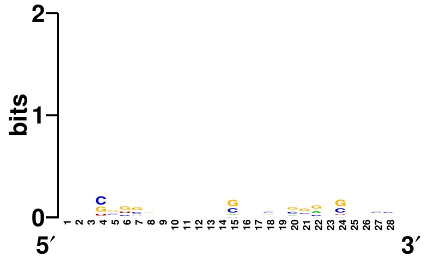

29-mers:

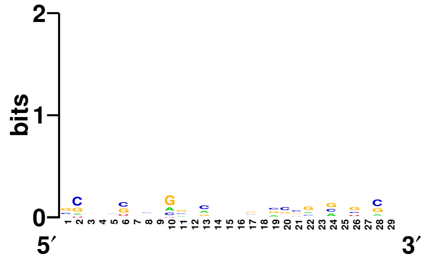

30-mers:

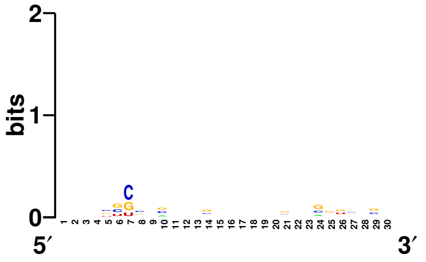

Adult female, library 3:

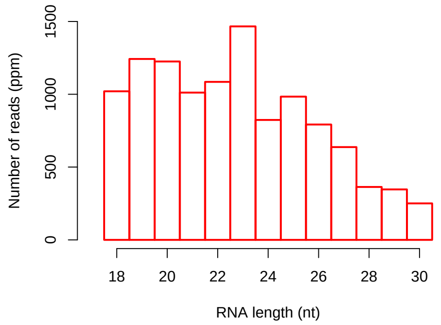

18-mers:

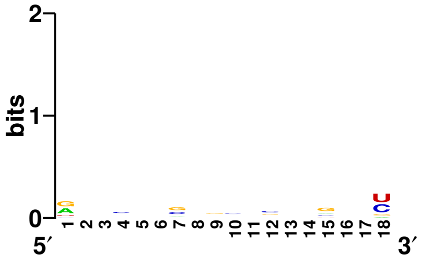

19-mers:

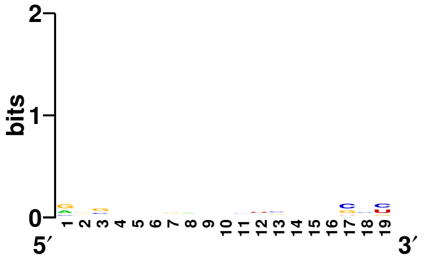

20-mers:

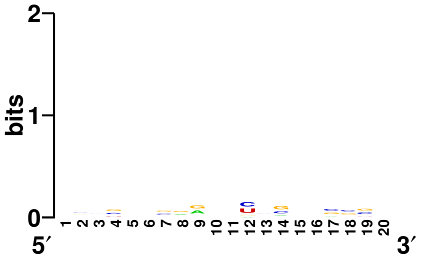

21-mers:

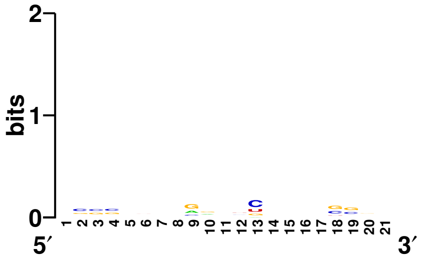

22-mers:

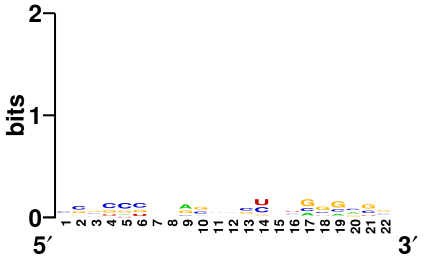

23-mers:

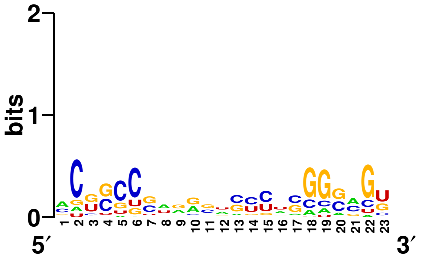

24-mers:

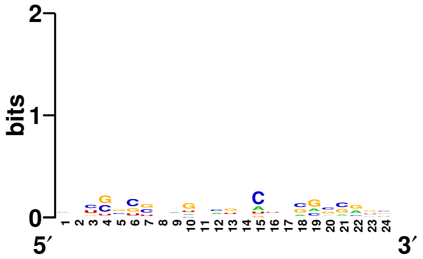

25-mers:

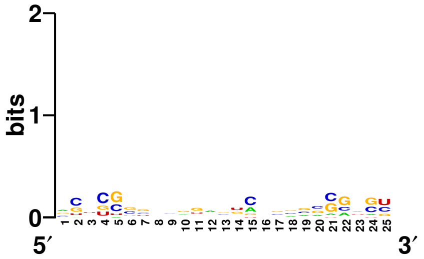

26-mers:

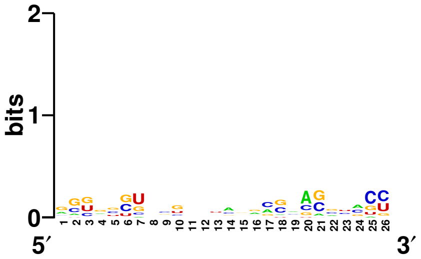

27-mers:

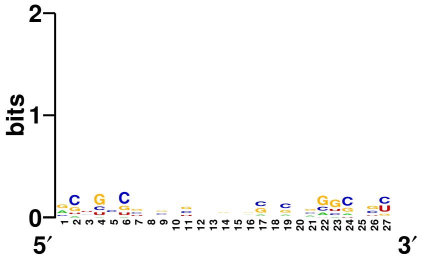

28-mers:

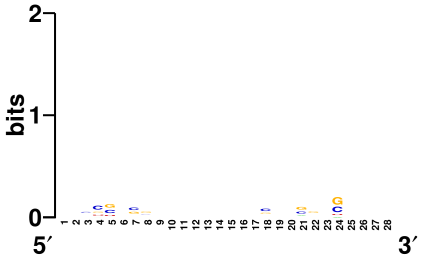

29-mers:

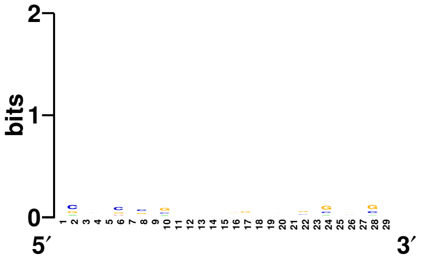

30-mers:

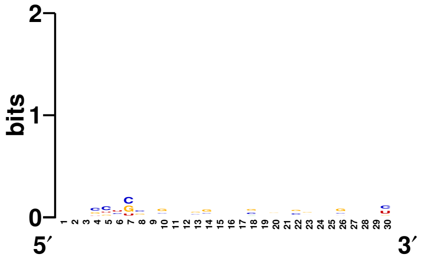

Adult male, library 3:

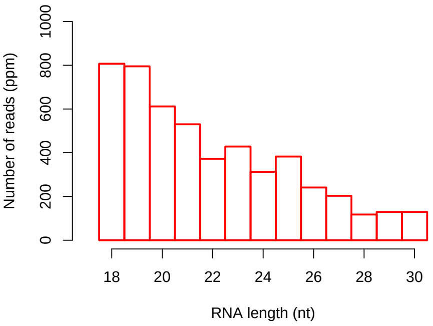

18-mers:

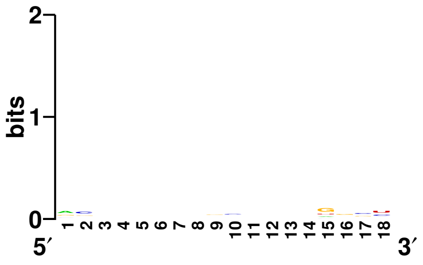

19-mers:

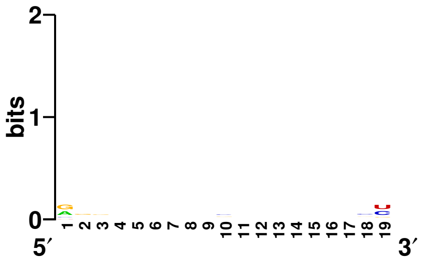

20-mers:

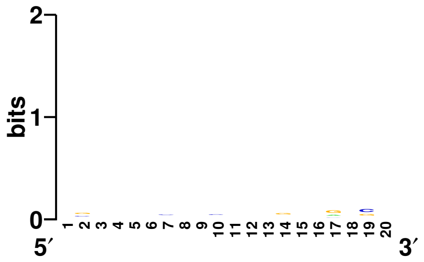

21-mers:

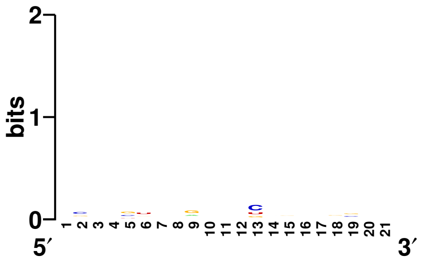

22-mers:

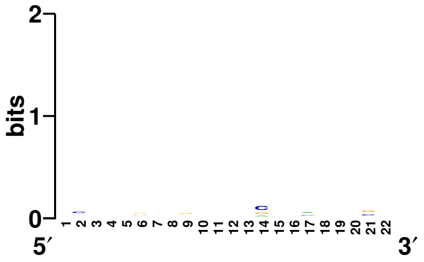

23-mers:

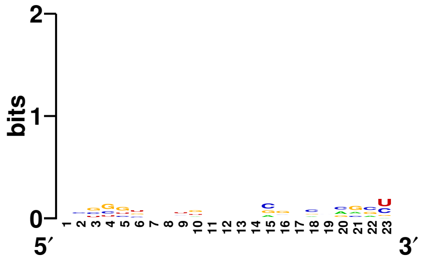

24-mers:

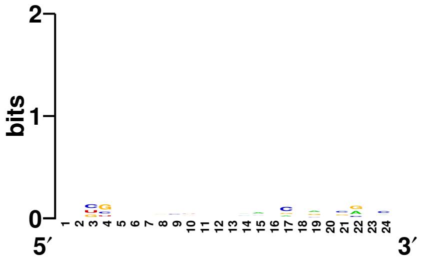

25-mers:

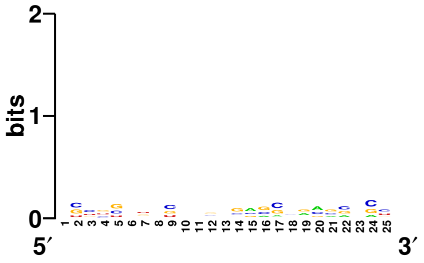

26-mers:

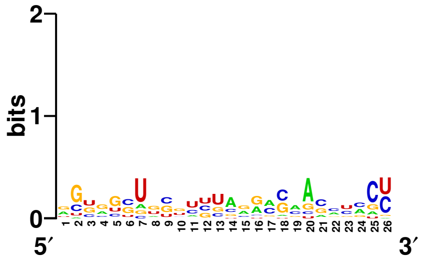

27-mers:

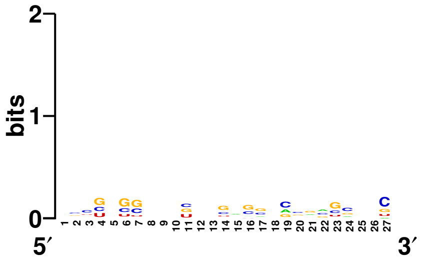

28-mers:

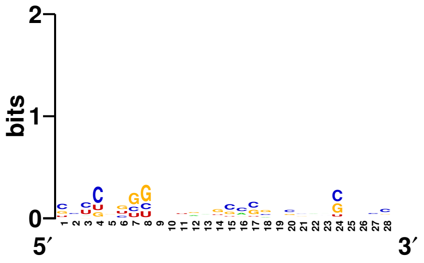

29-mers:

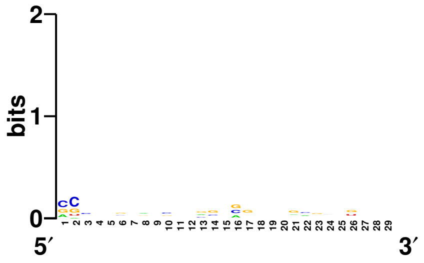

30-mers:

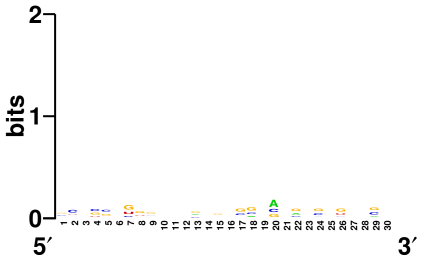

9.4 Libraries #4 (3´ modified, 5´ hydroxyl or polyphosphorylated small RNAs)

Embryo 8h, library 4:

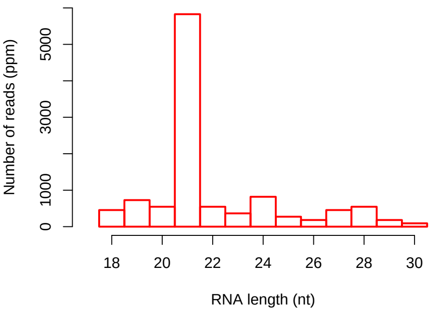

18-mers:

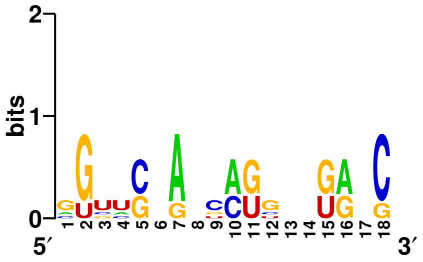

19-mers:

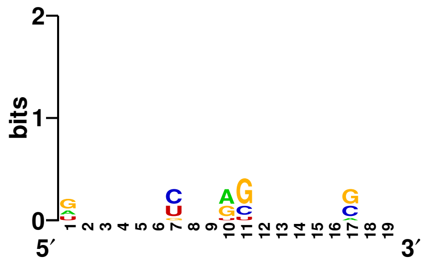

20-mers:

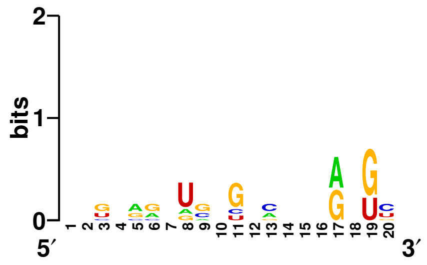

21-mers:

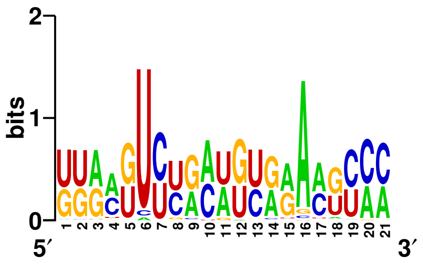

22-mers:

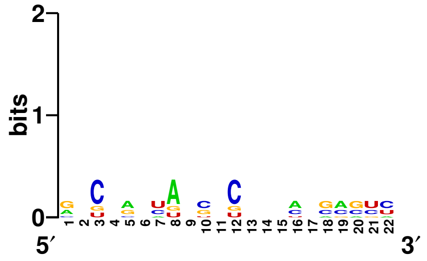

23-mers:

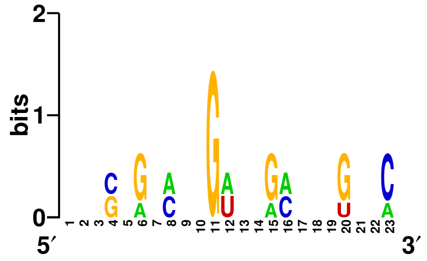

24-mers:

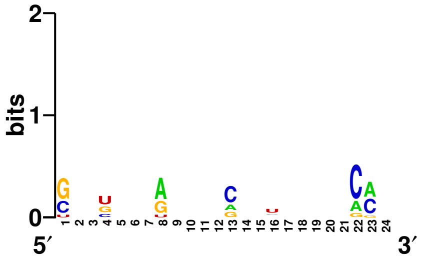

25-mers:

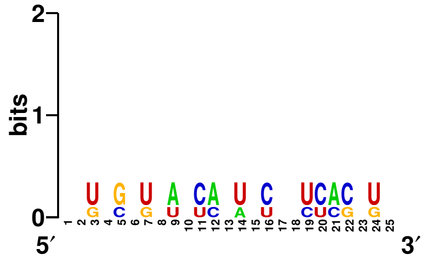

26-mers:

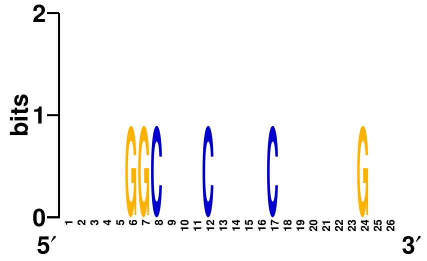

27-mers:

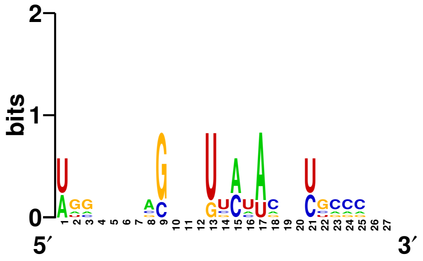

28-mers:

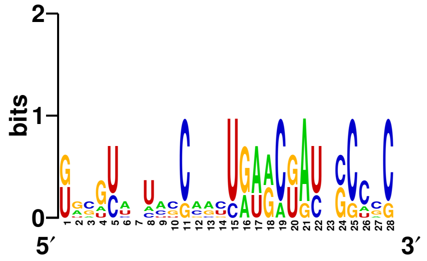

29-mers:

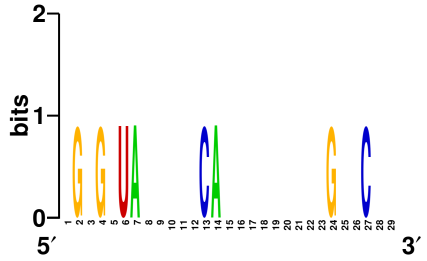

30-mers:

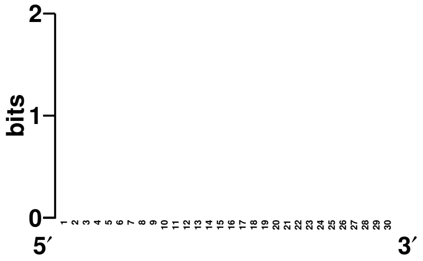

Embryo 15h, library 4:

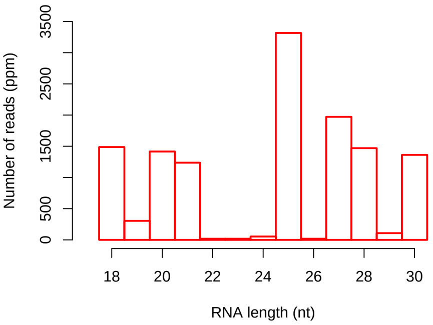

18-mers:

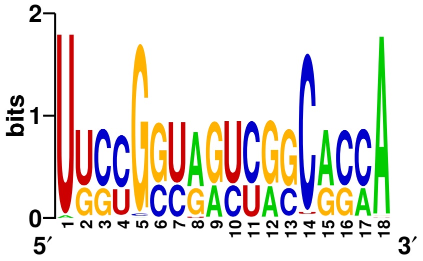

19-mers:

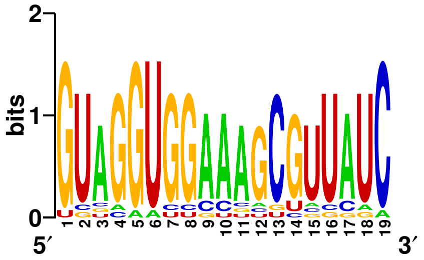

20-mers:

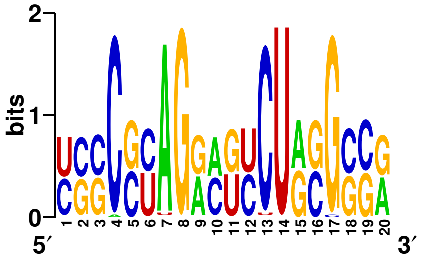

21-mers:

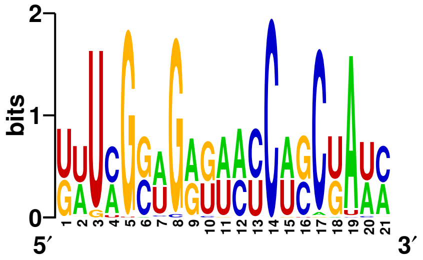

22-mers:

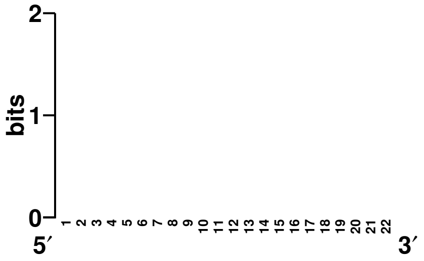

23-mers:

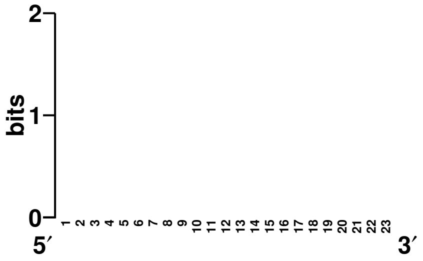

24-mers:

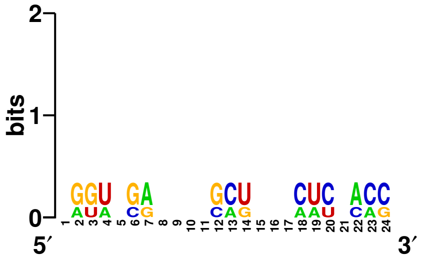

25-mers:

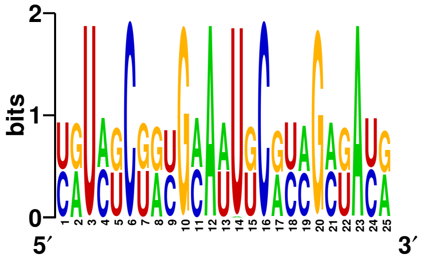

26-mers:

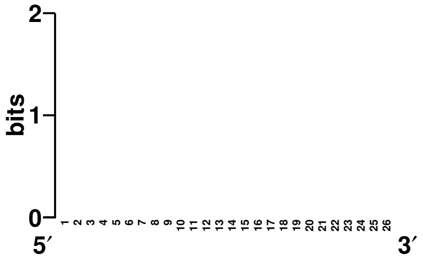

27-mers:

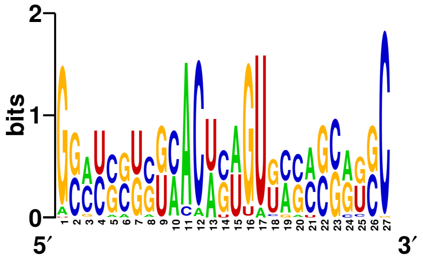

28-mers:

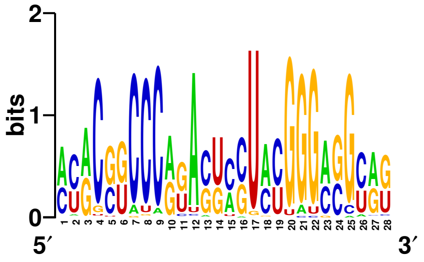

29-mers:

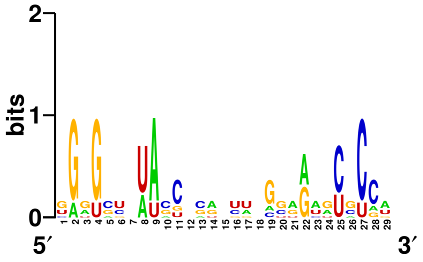

30-mers:

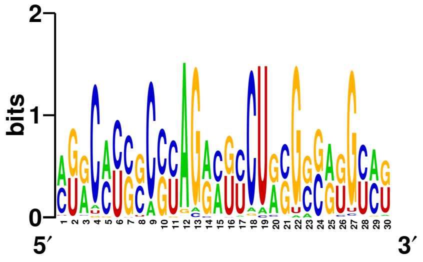

Embryo 36h, library 4:

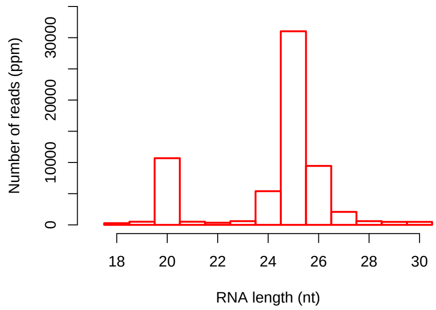

18-mers:

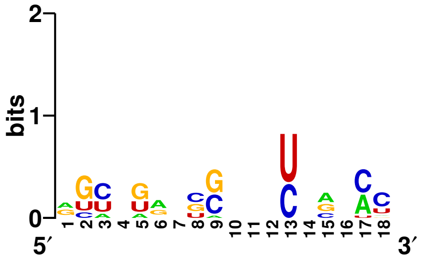

19-mers:

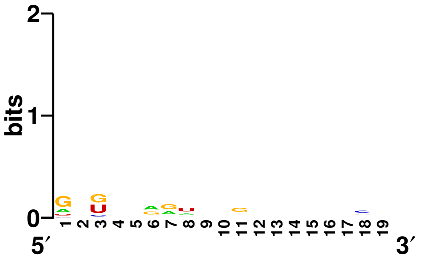

20-mers:

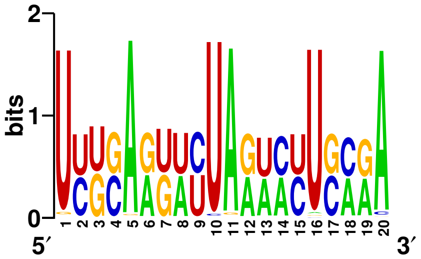

21-mers:

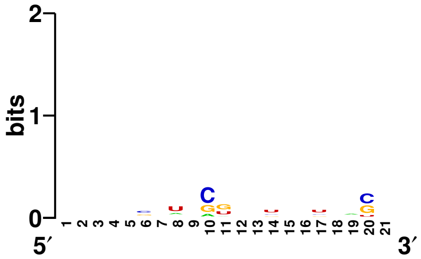

22-mers:

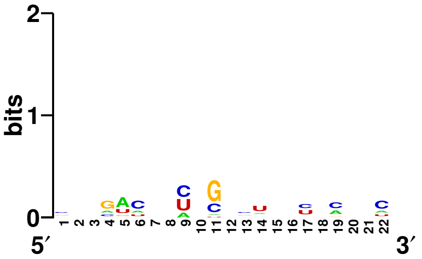

23-mers:

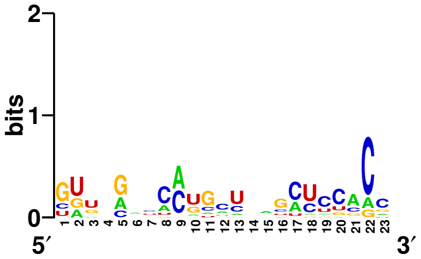

24-mers:

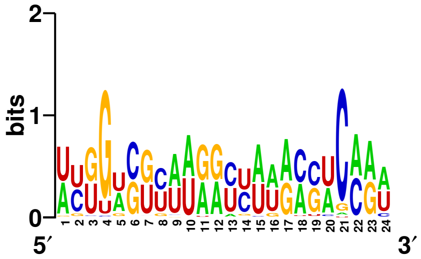

25-mers:

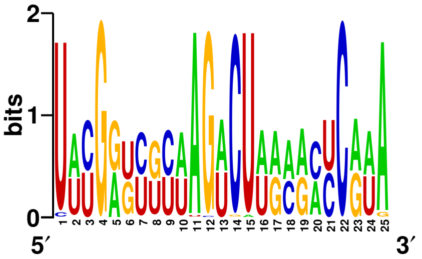

26-mers:

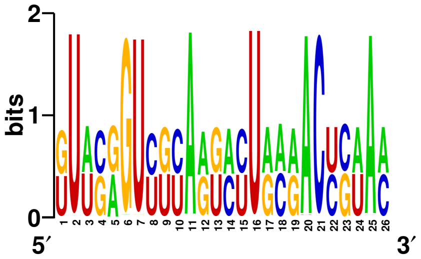

27-mers:

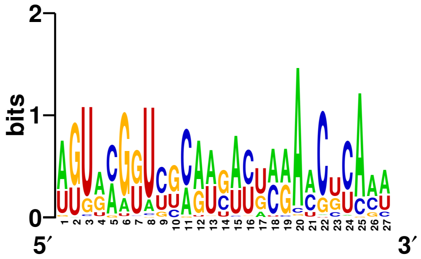

28-mers:

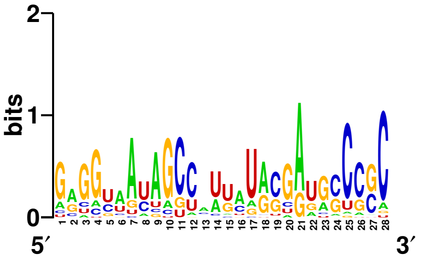

29-mers:

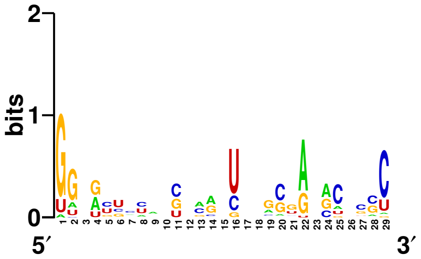

30-mers:

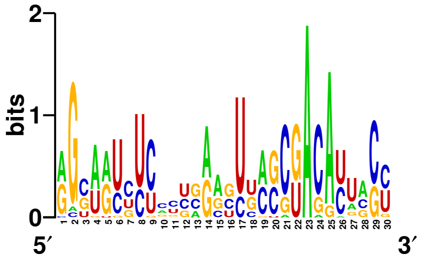

Embryo 60h, library 4:

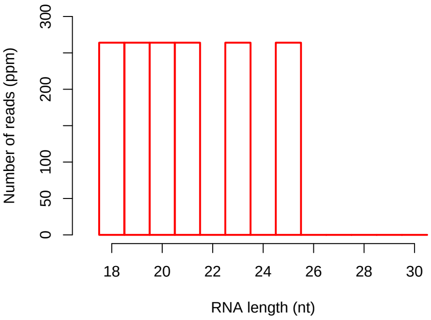

18-mers:

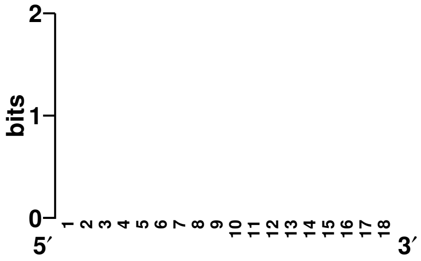

19-mers:

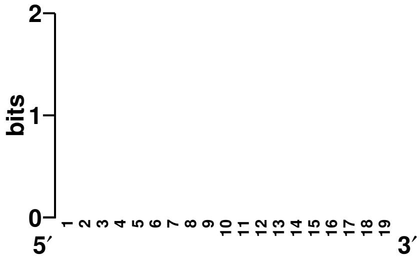

20-mers:

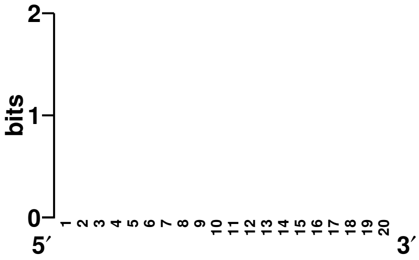

21-mers:

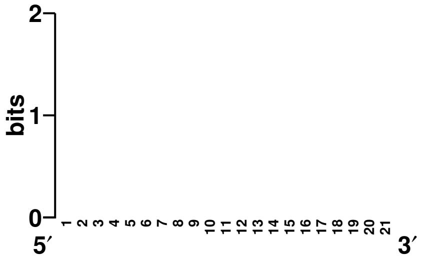

22-mers:

(no read)

23-mers:

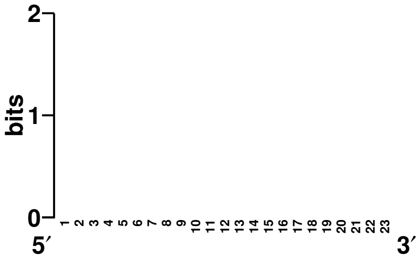

24-mers:

(no read)

25-mers:

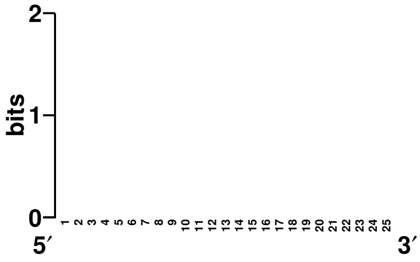

26-mers:

(no read)

27-mers:

(no read)

28-mers:

(no read)

29-mers:

(no read)

30-mers:

(no read)

Adult female, library 4:

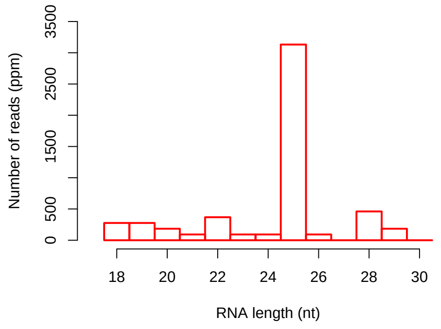

18-mers:

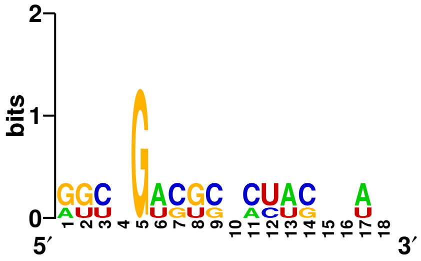

19-mers:

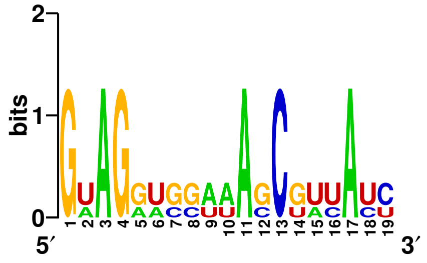

20-mers:

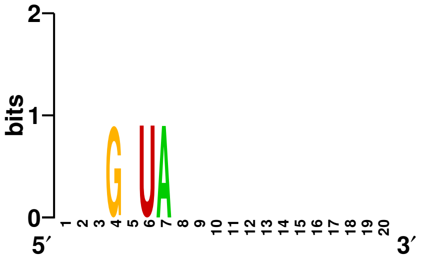

21-mers:

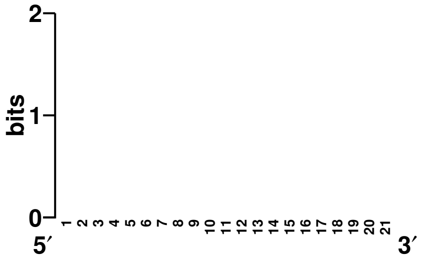

22-mers:

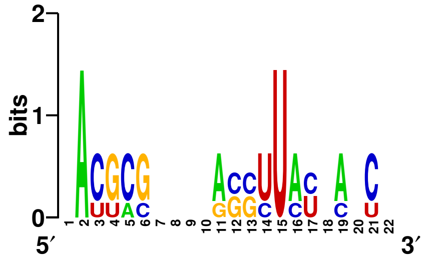

23-mers:

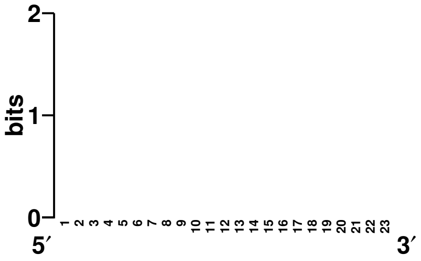

24-mers:

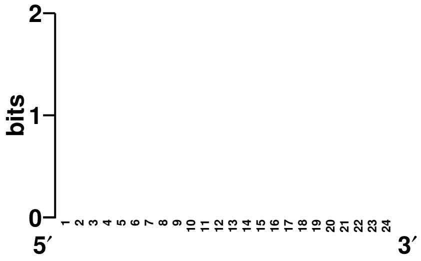

25-mers:

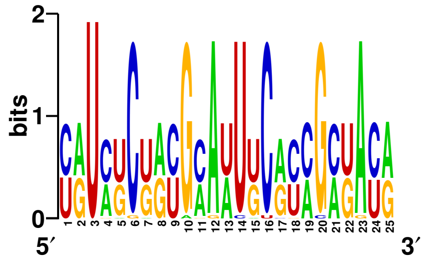

26-mers:

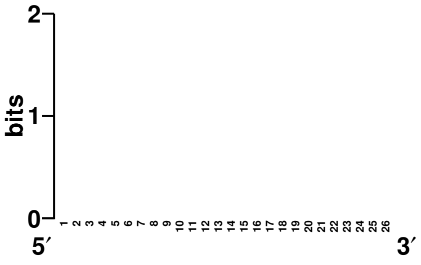

27-mers:

(no read)

28-mers:

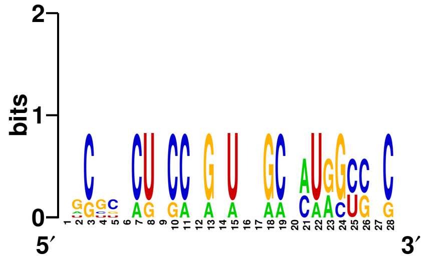

29-mers:

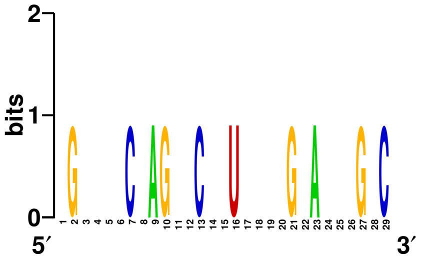

30-mers:

(no read)

Adult male, library 4:

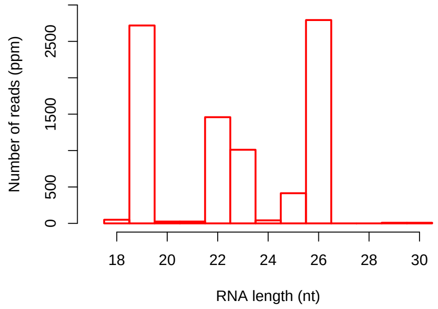

18-mers:

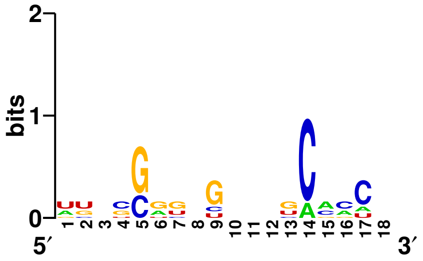

19-mers:

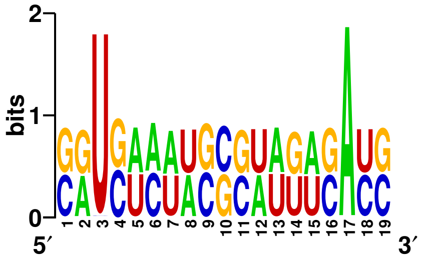

20-mers:

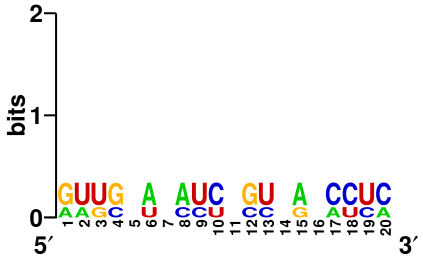

21-mers:

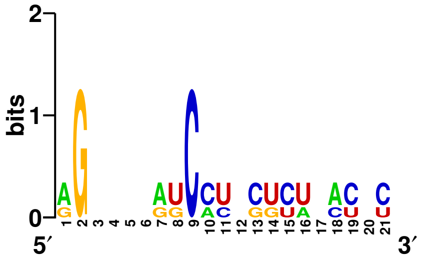

22-mers:

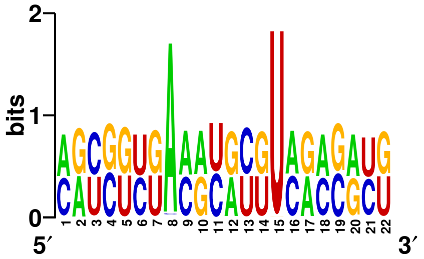

23-mers:

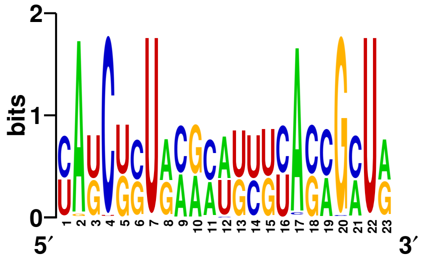

24-mers:

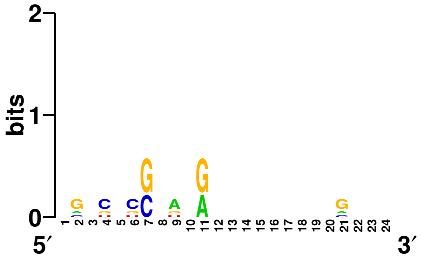

25-mers:

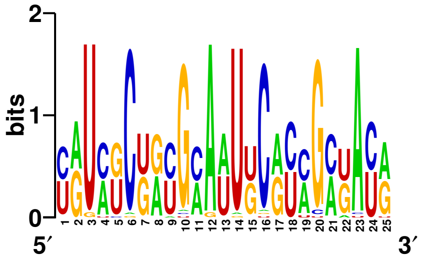

26-mers:

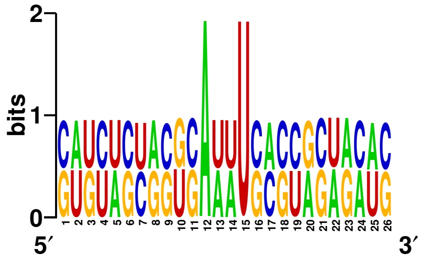

27-mers:

(no read)

28-mers:

(no read)

29-mers:

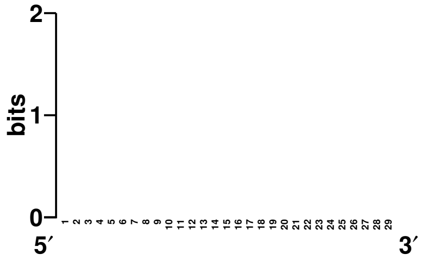

30-mers:

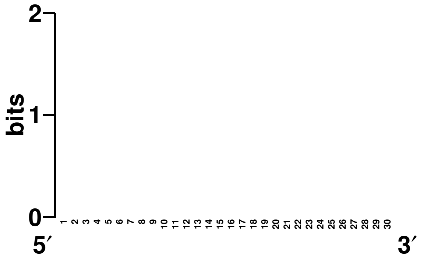

10 Extragenomic and extratranscriptomic reads matching the ATCV1 genome

10.1 Libraries #1 (total 5´ monophosphorylated small RNAs)

Embryo 8h, library 1:

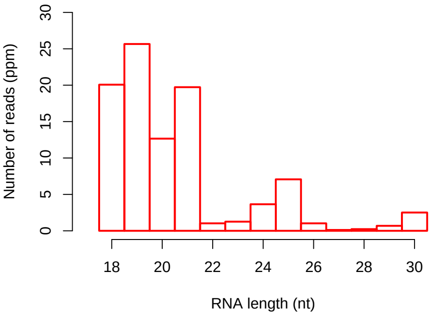

18-mers:

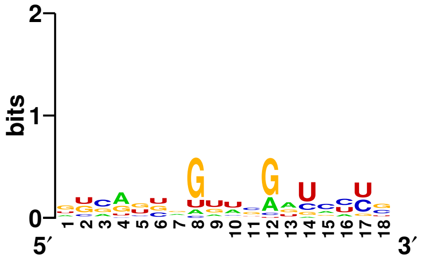

19-mers:

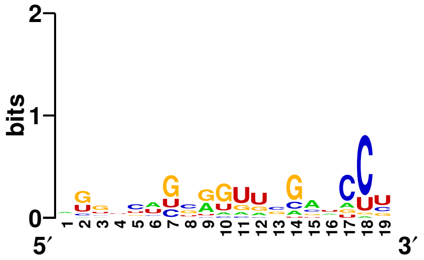

20-mers:

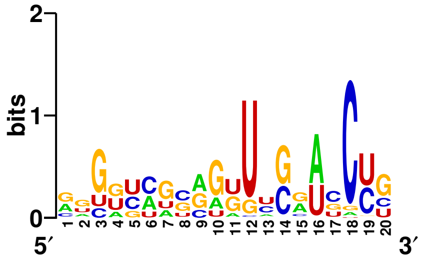

21-mers:

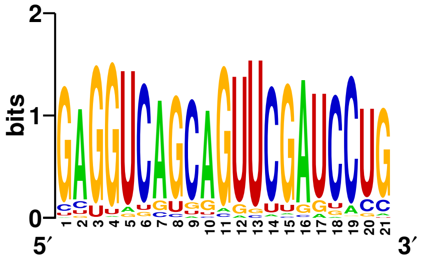

22-mers:

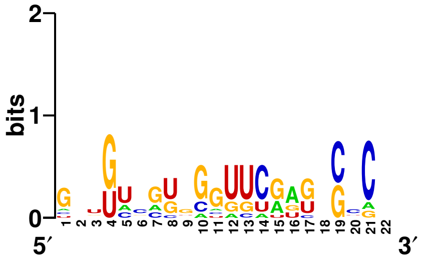

23-mers:

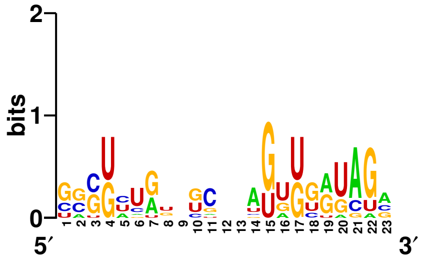

24-mers:

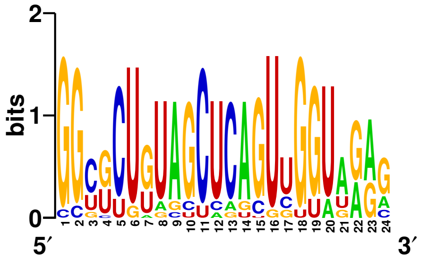

25-mers:

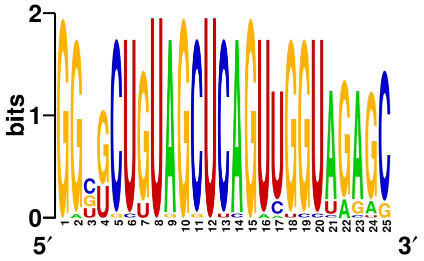

26-mers:

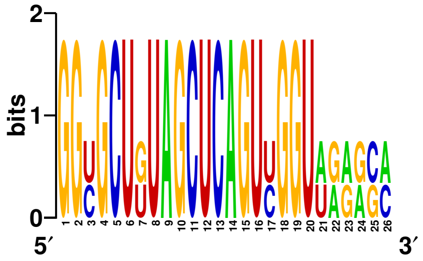

27-mers:

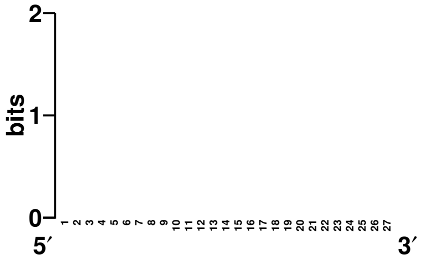

28-mers:

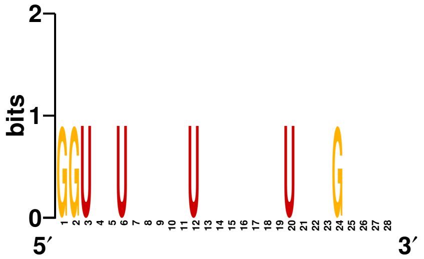

29-mers:

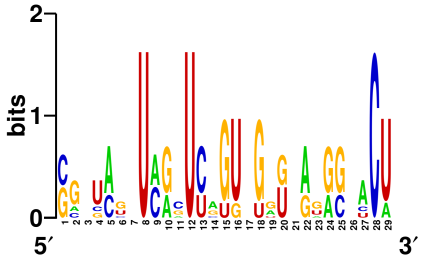

30-mers:

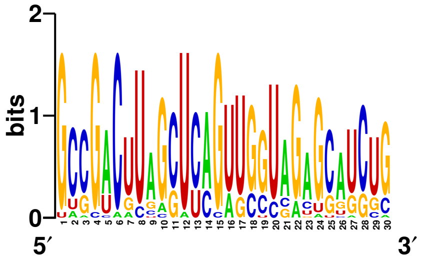

Embryo 15h, library 1:

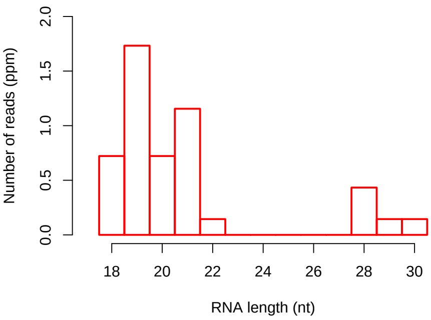

18-mers:

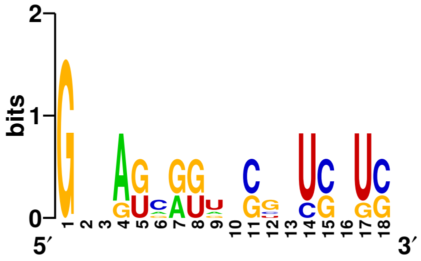

19-mers:

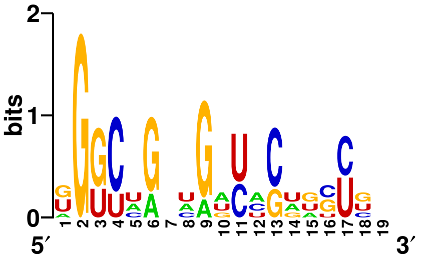

20-mers:

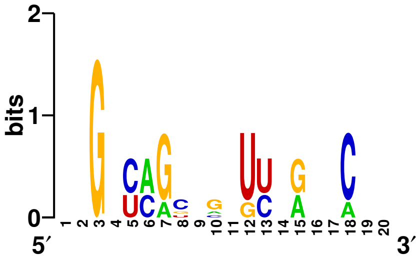

21-mers:

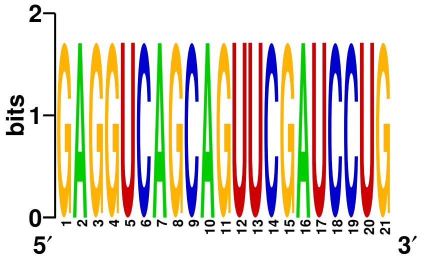

22-mers:

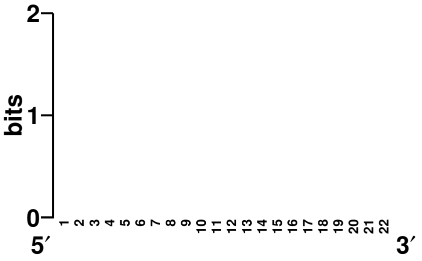

23-mers:

(no read)

24-mers:

(no read)

25-mers:

(no read)

26-mers:

(no read)

27-mers:

(no read)

28-mers:

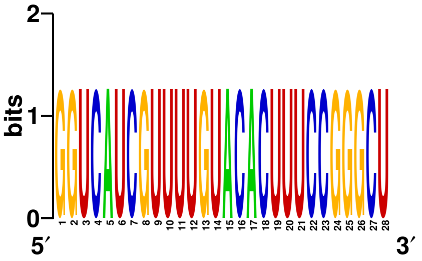

29-mers:

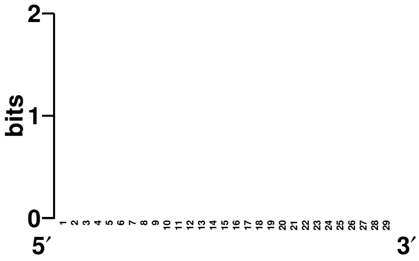

30-mers:

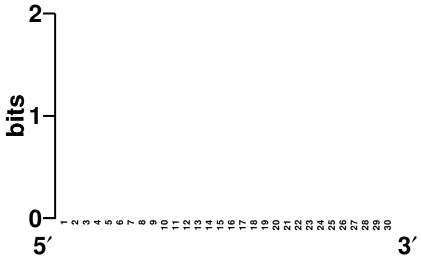

Embryo 36h, library 1:

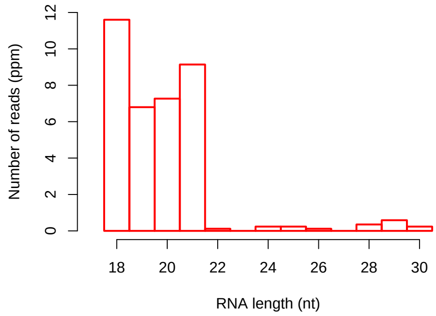

18-mers:

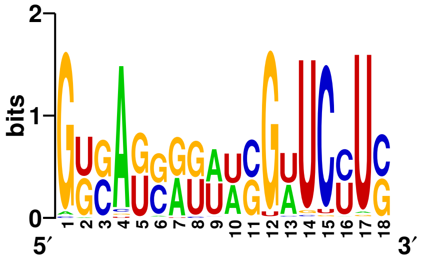

19-mers:

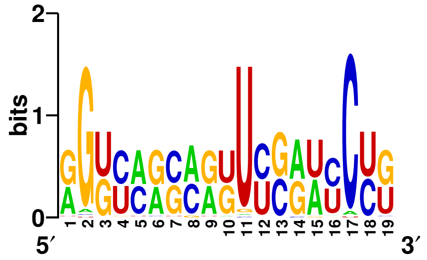

20-mers:

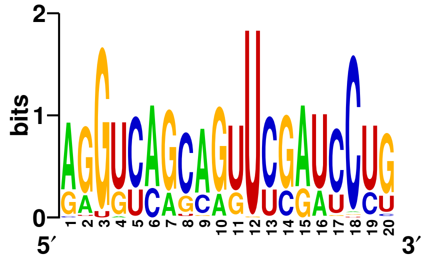

21-mers:

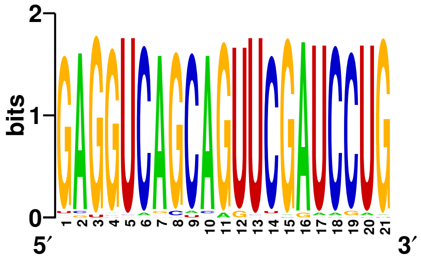

22-mers:

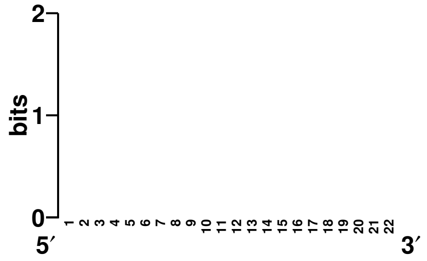

23-mers:

(no read)

24-mers:

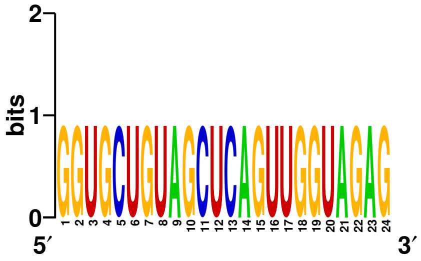

25-mers:

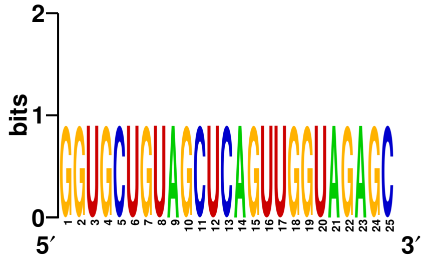

26-mers:

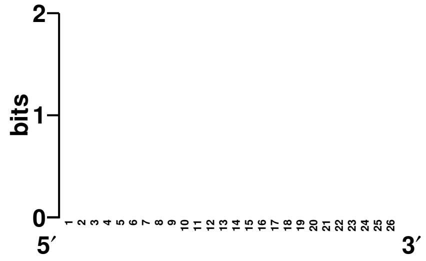

27-mers:

(no read)

28-mers:

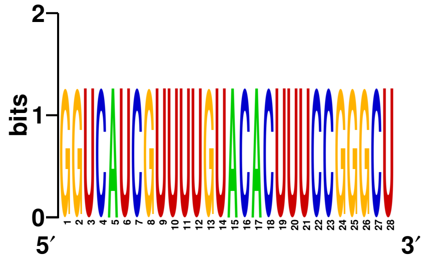

29-mers:

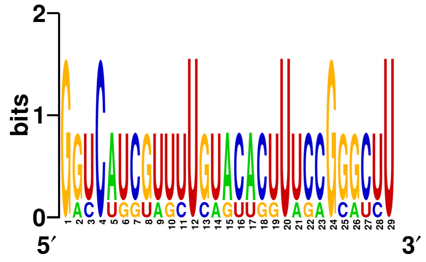

30-mers:

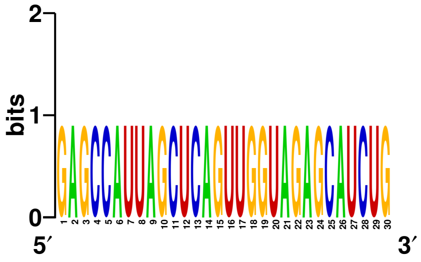

Embryo 60h, library 1:

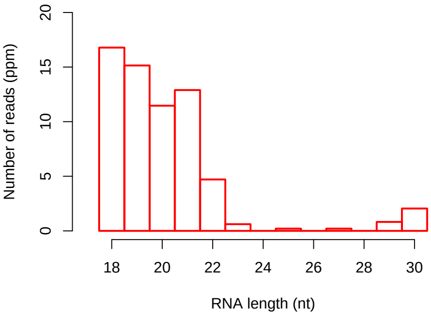

18-mers:

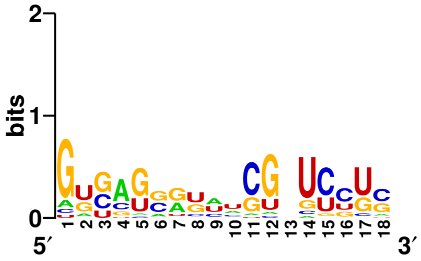

19-mers:

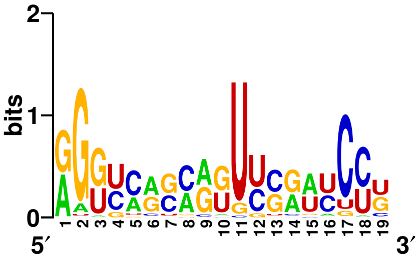

20-mers:

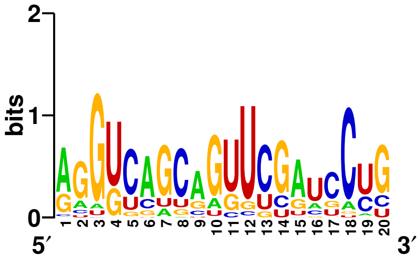

21-mers:

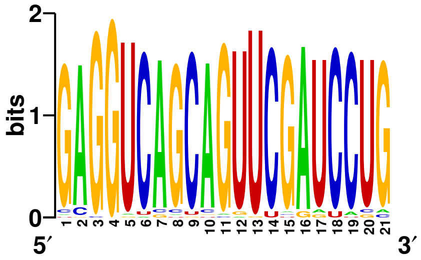

22-mers:

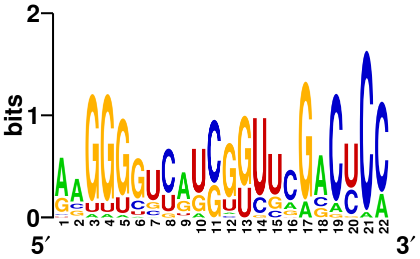

23-mers:

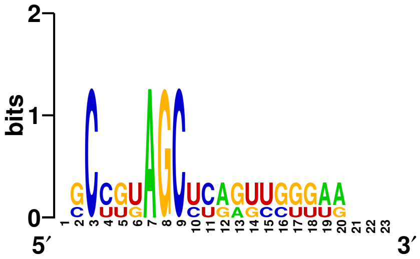

24-mers:

(no read)

25-mers:

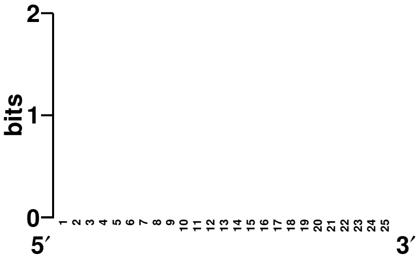

26-mers:

(no read)

27-mers:

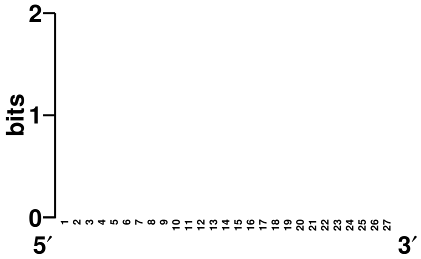

28-mers:

(no read)

29-mers:

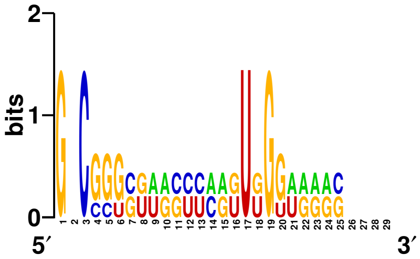

30-mers:

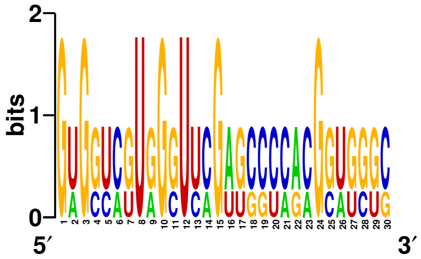

Adult female, library 1:

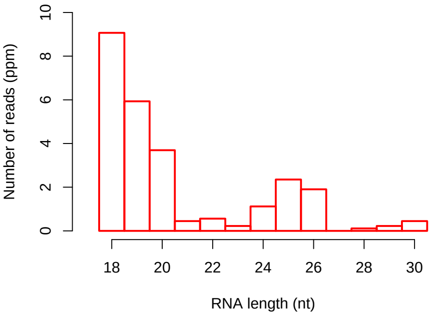

18-mers:

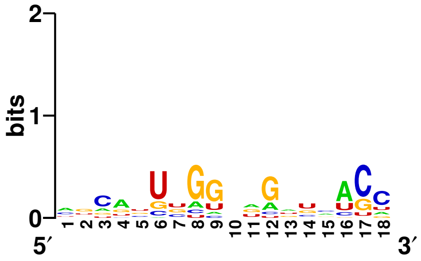

19-mers:

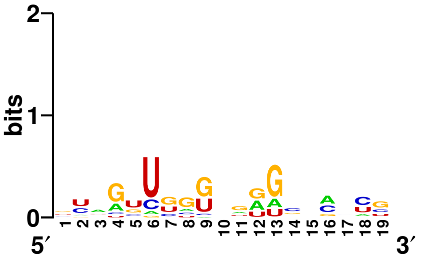

20-mers:

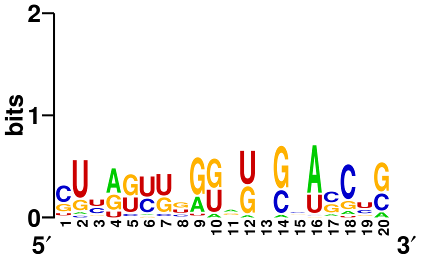

21-mers:

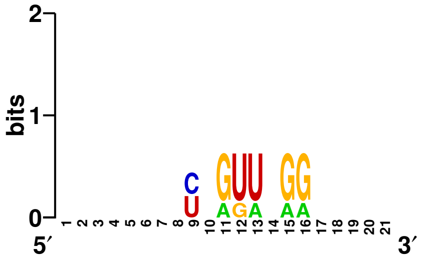

22-mers:

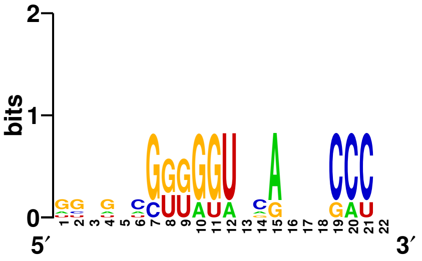

23-mers:

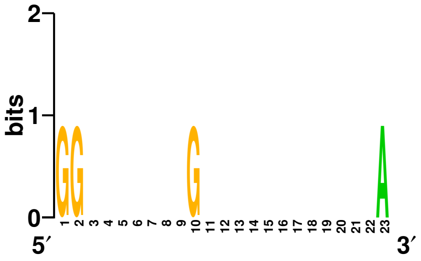

24-mers:

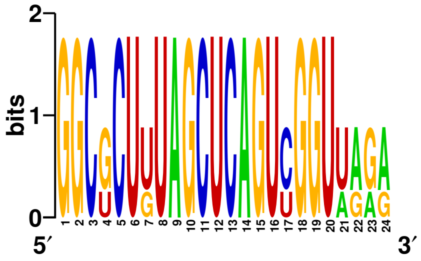

25-mers:

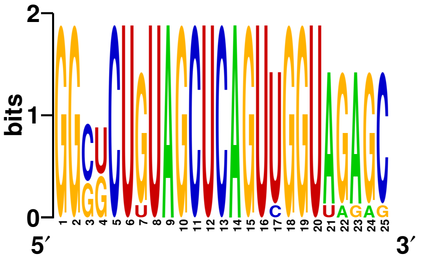

26-mers:

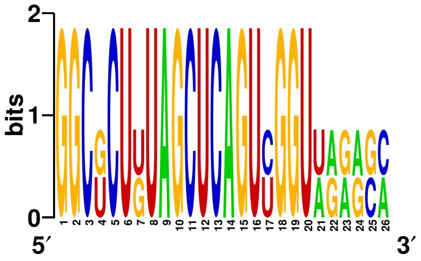

27-mers:

(no read)

28-mers:

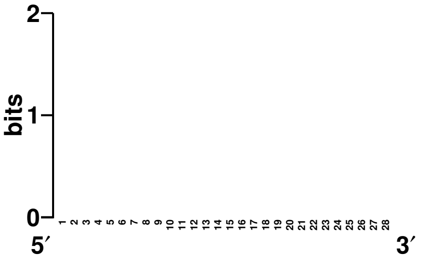

29-mers:

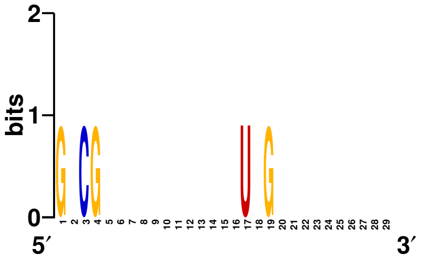

30-mers:

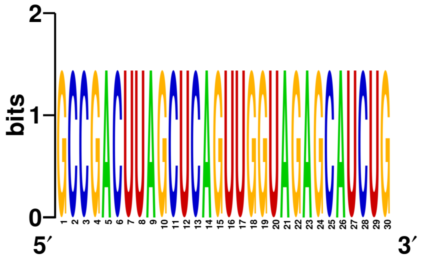

Adult male, library 1:

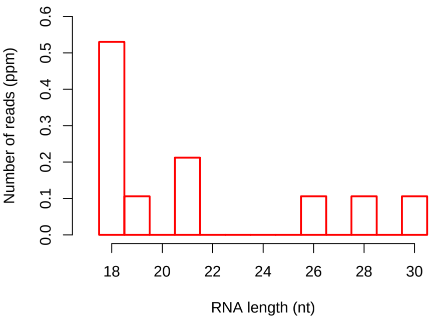

18-mers:

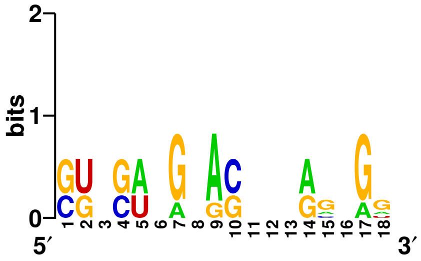

19-mers:

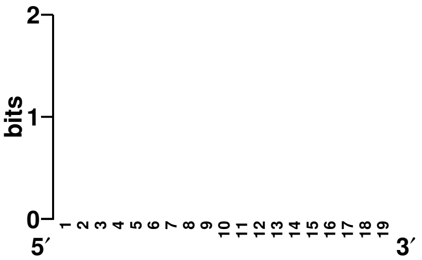

20-mers:

(no read)

21-mers:

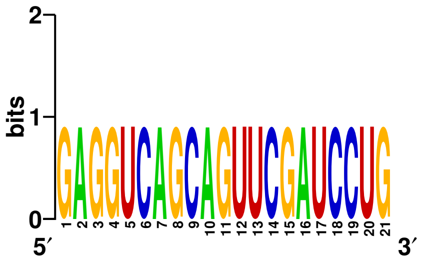

22-mers:

(no read)

23-mers:

(no read)

24-mers:

(no read)

25-mers:

(no read)

26-mers:

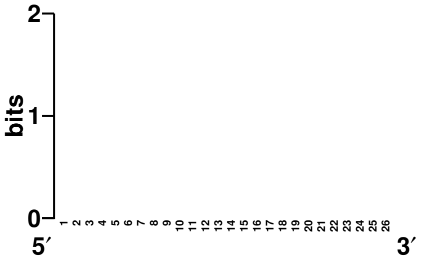

27-mers:

(no read)

28-mers:

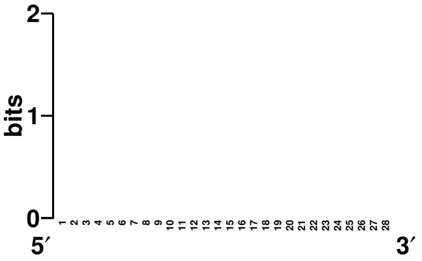

29-mers:

(no read)

30-mers:

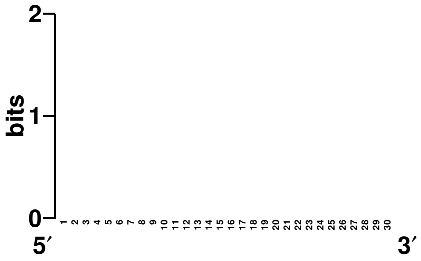

10.2 Libraries #2 (3´ modified, 5´ monophosphorylated small RNAs)

Embryo 8h, library 2:

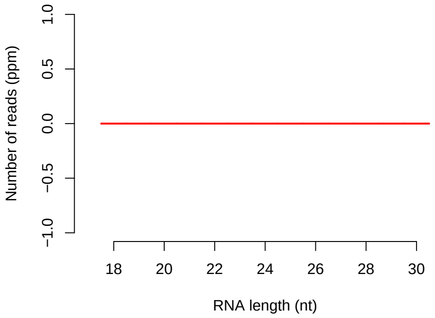

18-mers:

(no read)

19-mers:	20-mers:	21-mers:
(no read)	(no read)	(no read)
22-mers:	23-mers:	24-mers:
(no read)	(no read)	(no read)
25-mers:	26-mers:	27-mers:
(no read)	(no read)	(no read)
28-mers:	29-mers:	30-mers:
(no read)	(no read)	(no read)

Embryo 15h, library 2:

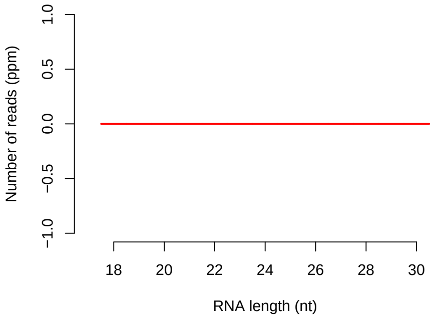

18-mers:

(no read)

19-mers:	20-mers:	21-mers:
(no read)	(no read)	(no read)
22-mers:	23-mers:	24-mers:
(no read)	(no read)	(no read)
25-mers:	26-mers:	27-mers:
(no read)	(no read)	(no read)
28-mers:	29-mers:	30-mers:
(no read)	(no read)	(no read)

Embryo 36h, library 2:

18-mers:

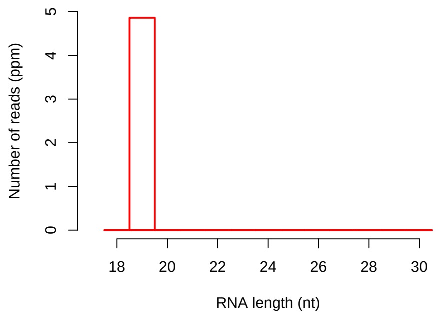

(no read)

19-mers:

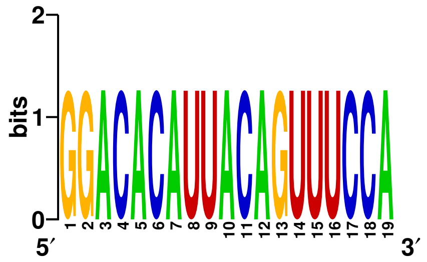

20-mers:

(no read)

21-mers:

(no read)

22-mers:

(no read)

23-mers:

(no read)

24-mers:

(no read)

25-mers:

(no read)

26-mers:

(no read)

27-mers:

(no read)

28-mers:

(no read)

29-mers:

(no read)

30-mers:

(no read)

Embryo 60h, library 2:

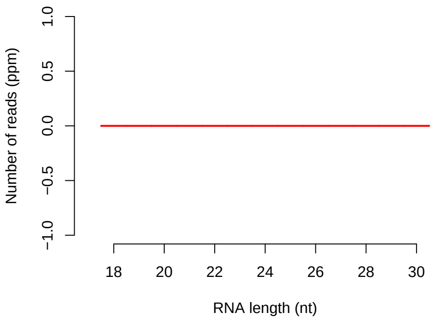

18-mers:
(no read)

19-mers:	20-mers:	21-mers:
(no read)	(no read)	(no read)
22-mers:	23-mers:	24-mers:
(no read)	(no read)	(no read)
25-mers:	26-mers:	27-mers:
(no read)	(no read)	(no read)
28-mers:	29-mers:	30-mers:
(no read)	(no read)	(no read)

Adult female, library 2:

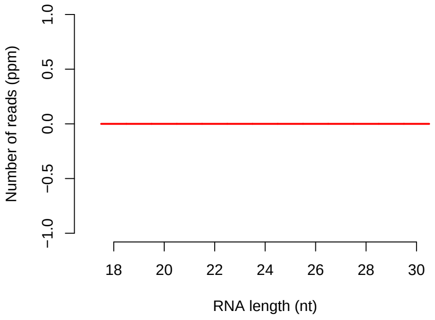

18-mers:		
		(no read)
19-mers:	20-mers:	21-mers:
(no read)	(no read)	(no read)
22-mers:	23-mers:	24-mers:
(no read)	(no read)	(no read)
25-mers:	26-mers:	27-mers:
(no read)	(no read)	(no read)
28-mers:	29-mers:	30-mers:
(no read)	(no read)	(no read)

Adult male, library 2:

18-mers:

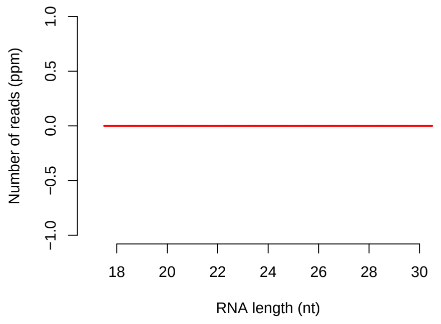

(no read)

19-mers:	20-mers:	21-mers:
(no read)	(no read)	(no read)
22-mers:	23-mers:	24-mers:
(no read)	(no read)	(no read)
25-mers:	26-mers:	27-mers:
(no read)	(no read)	(no read)
28-mers:	29-mers:	30-mers:
(no read)	(no read)	(no read)

10.3 Libraries #3 (total 5´ hydroxyl or polyphosphorylated small RNAs)

Embryo 8h, library 3:

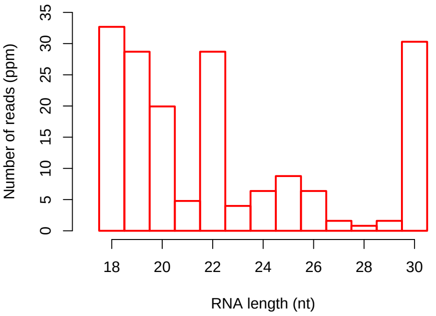

18-mers:

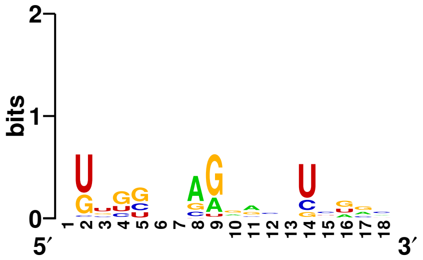

19-mers:

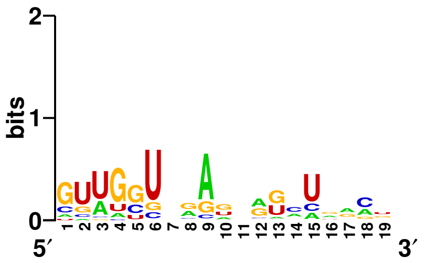

20-mers:

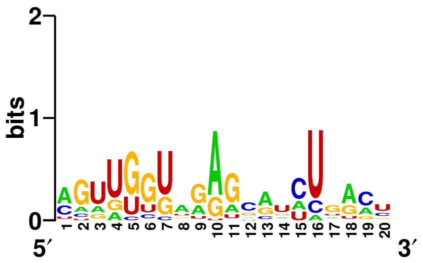

21-mers:

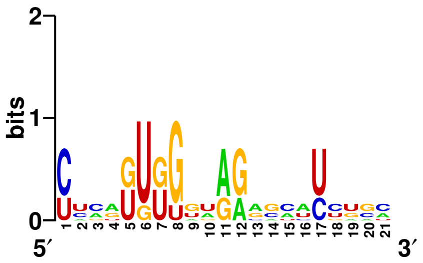

22-mers:

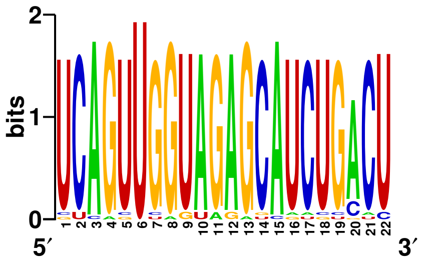

23-mers:

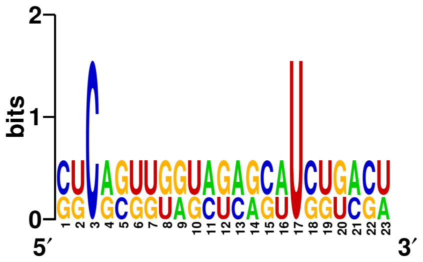

24-mers:

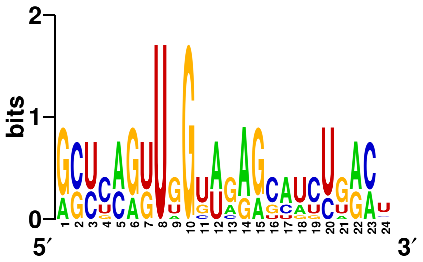

25-mers:

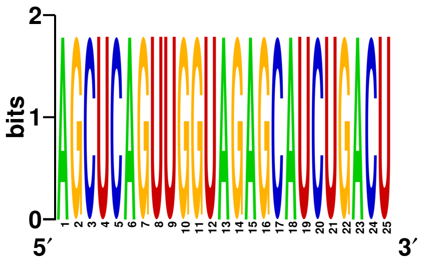

26-mers:

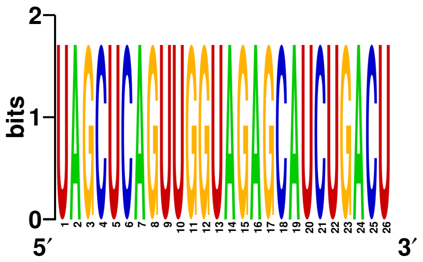

27-mers:

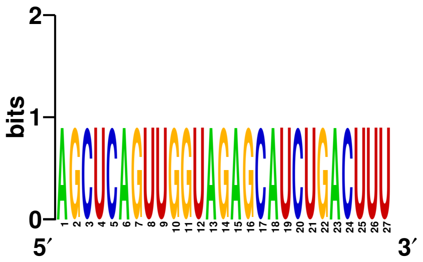

28-mers:

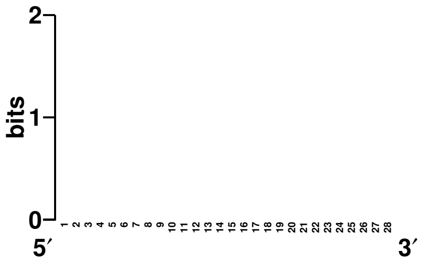

29-mers:

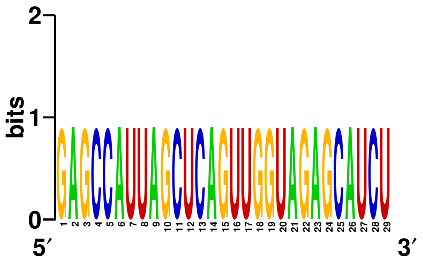

30-mers:

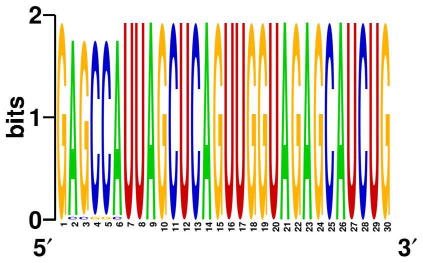

Embryo 15h, library 3:

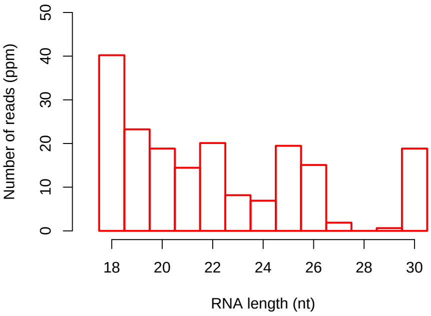

18-mers:

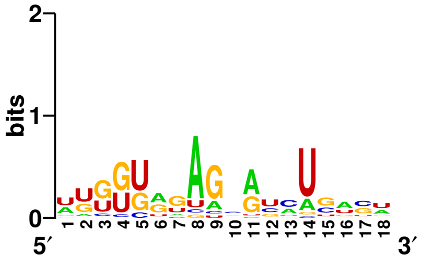

19-mers:

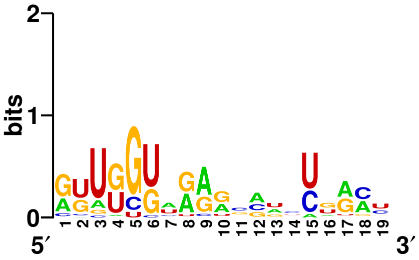

20-mers:

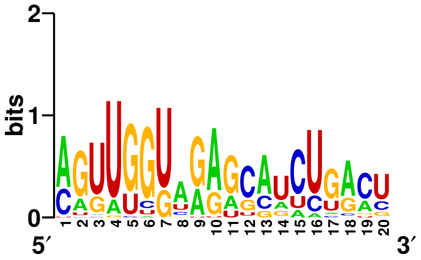

21-mers:

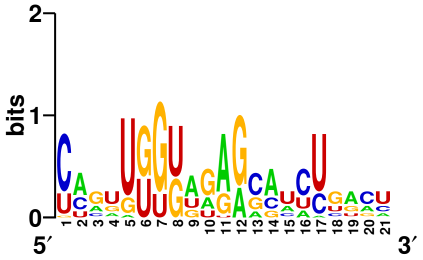

22-mers:

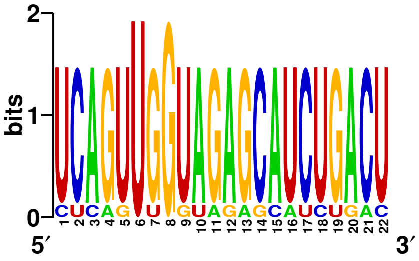

23-mers:

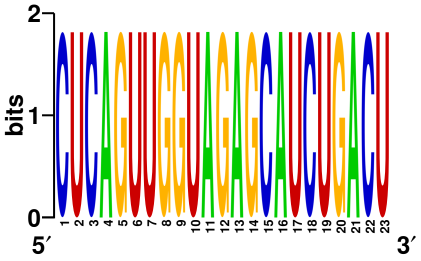

24-mers:

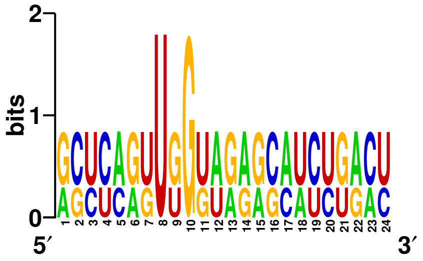

25-mers:

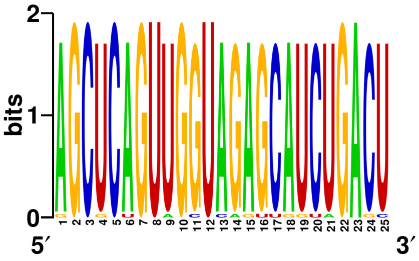

26-mers:

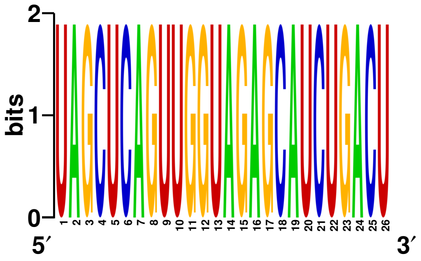

27-mers:

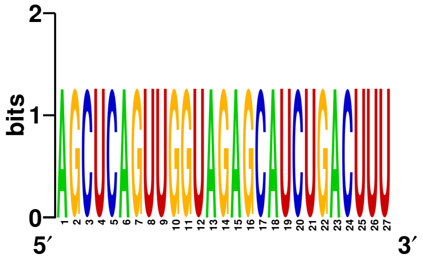

28-mers:

(no read)

29-mers:

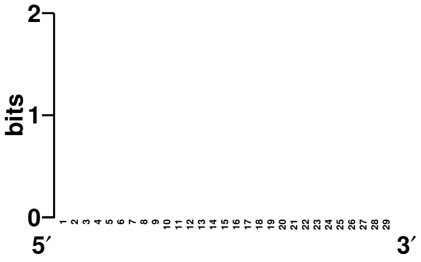

30-mers:

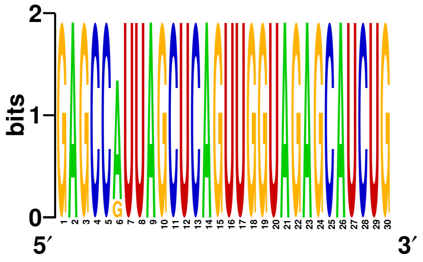

Embryo 36h, library 3:

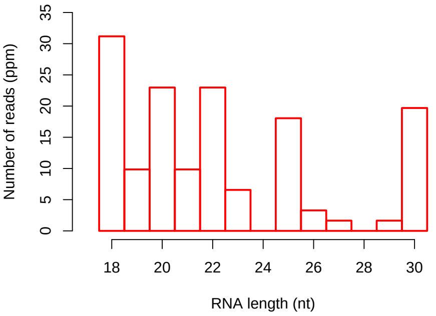

18-mers:

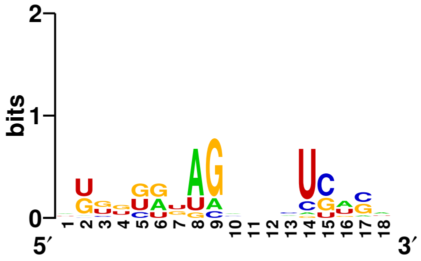

19-mers:

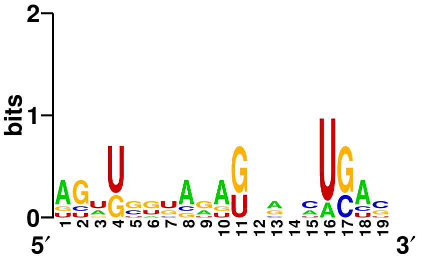

20-mers:

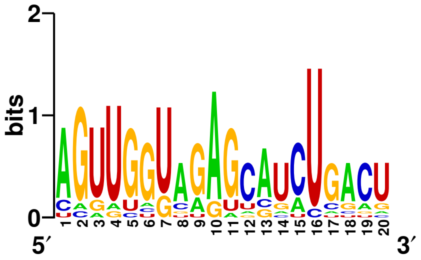

21-mers:

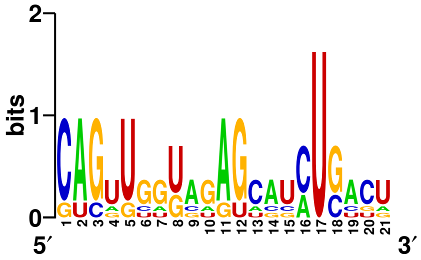

22-mers:

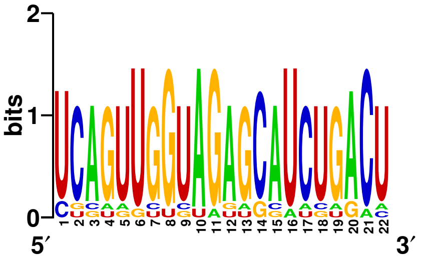

23-mers:

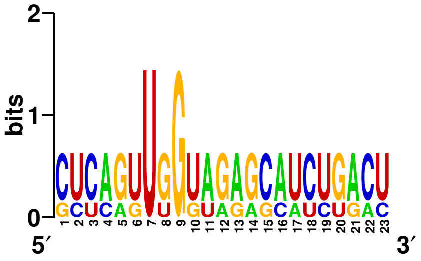

24-mers:

(no read)

25-mers:

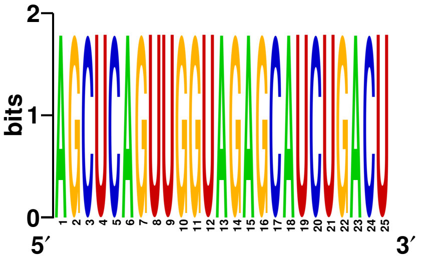

26-mers:

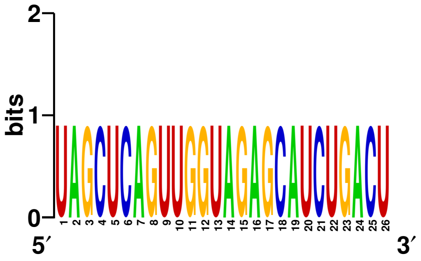

27-mers:

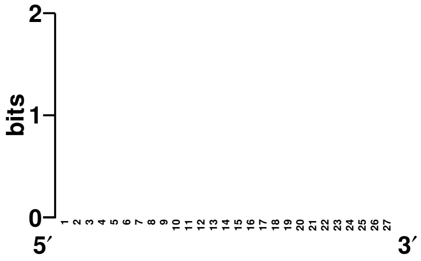

28-mers:

(no read)

29-mers:

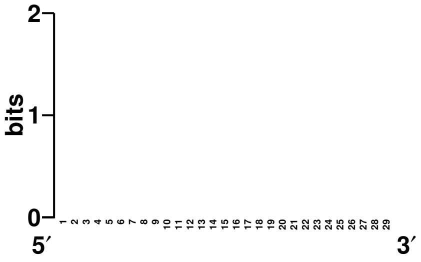

30-mers:

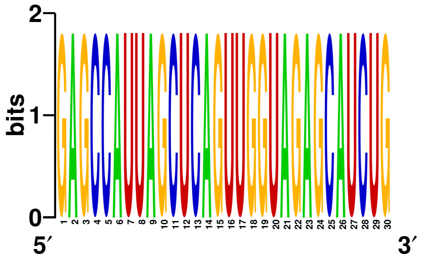

Embryo 60h, library 3:

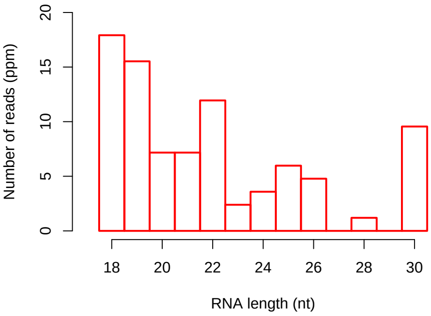

18-mers:

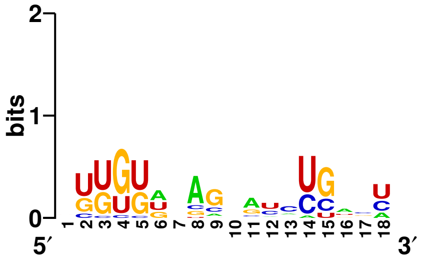

19-mers:

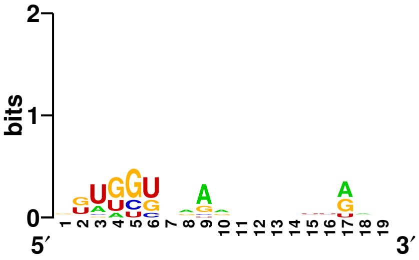

20-mers:

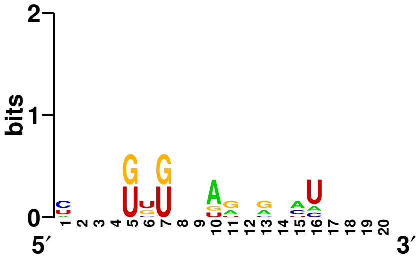

21-mers:

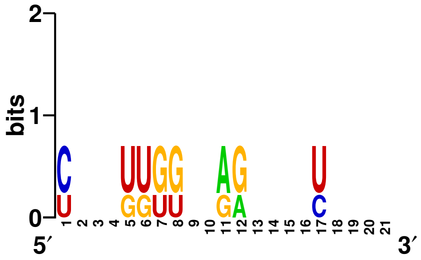

22-mers:

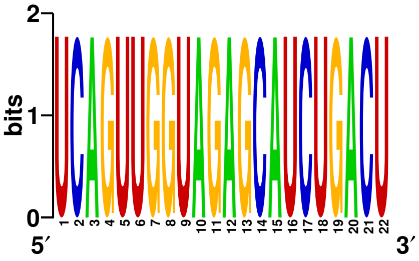

23-mers:

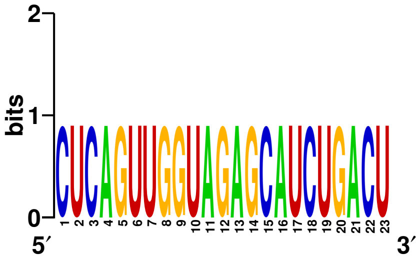

24-mers:

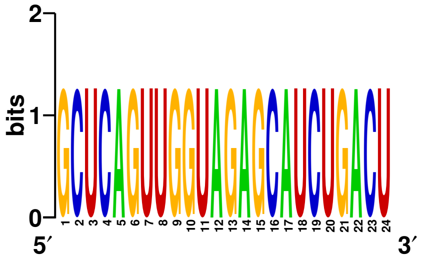

25-mers:

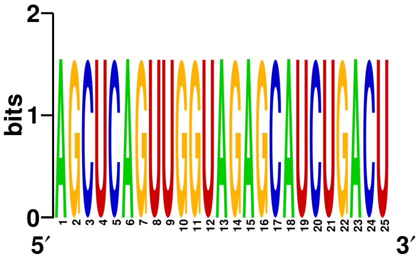

26-mers:

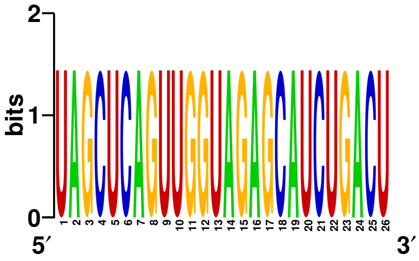

27-mers:

(no read)

28-mers:

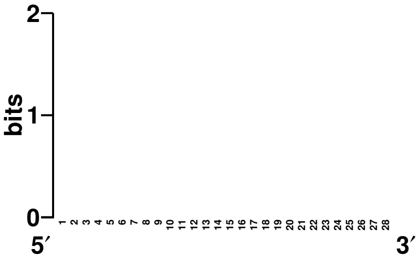

29-mers:

(no read)

30-mers:

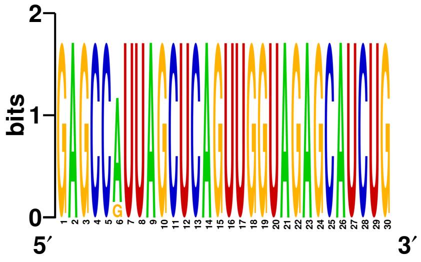

Adult female, library 3:

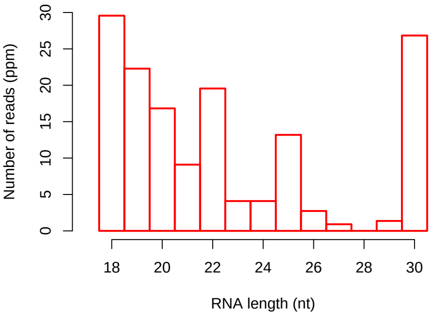

18-mers:

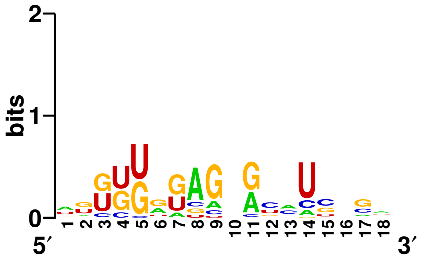

19-mers:

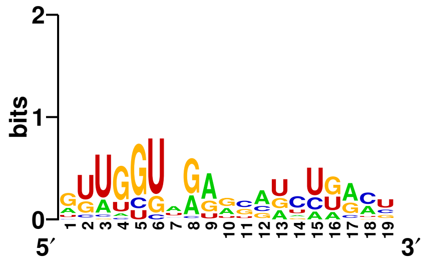

20-mers:

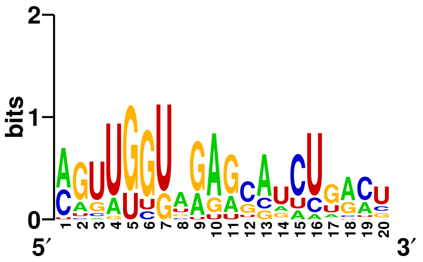

21-mers:

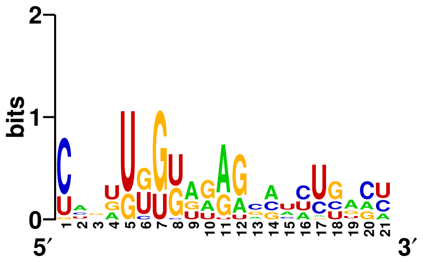

22-mers:

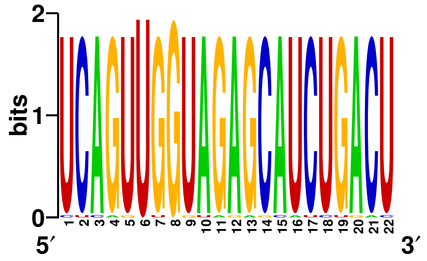

23-mers:

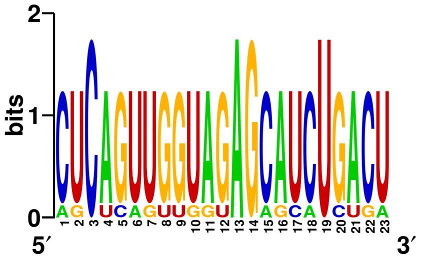

24-mers:

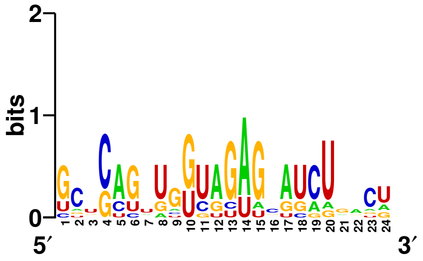

25-mers:

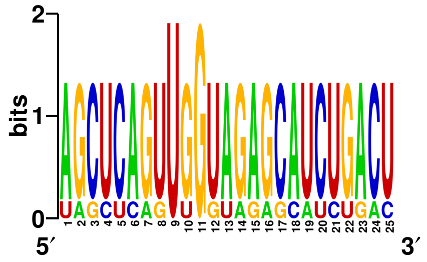

26-mers:

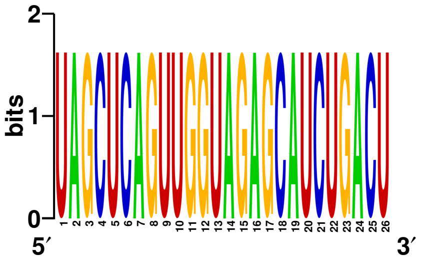

27-mers:

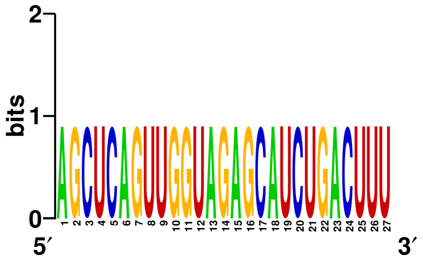

28-mers:

(no read)

29-mers:

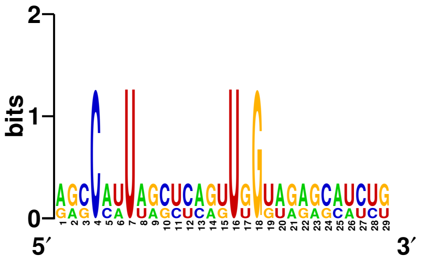

30-mers:

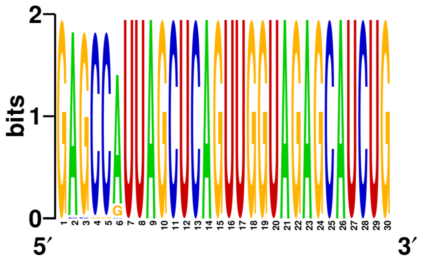

Adult male, library 3:

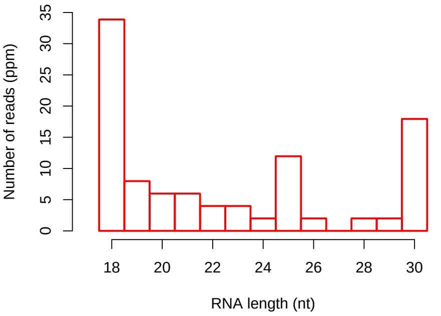

18-mers:

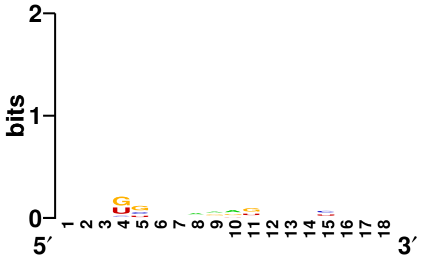

19-mers:

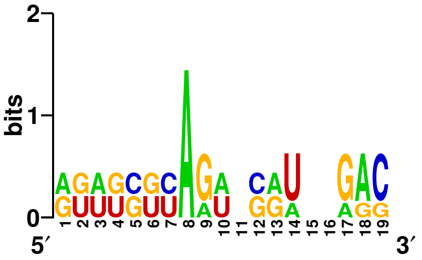

20-mers:

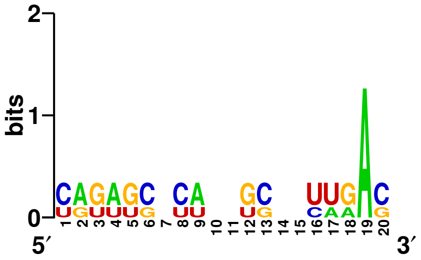

21-mers:

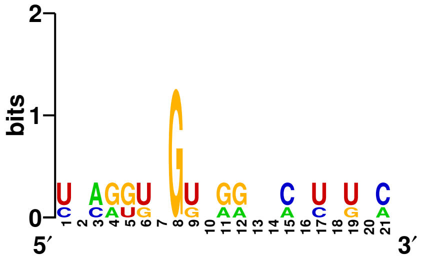

22-mers:

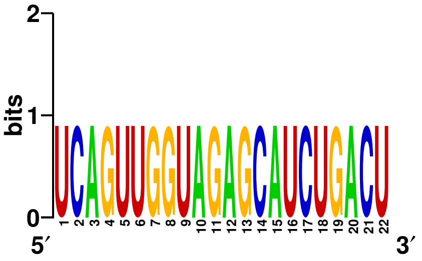

23-mers:

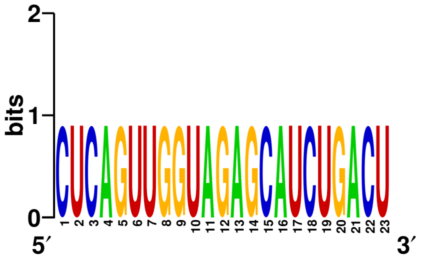

24-mers:

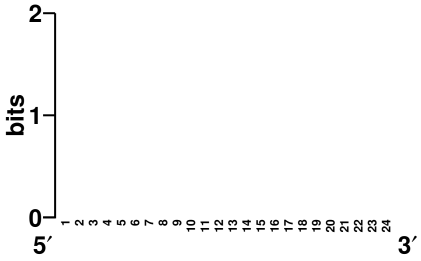

25-mers:

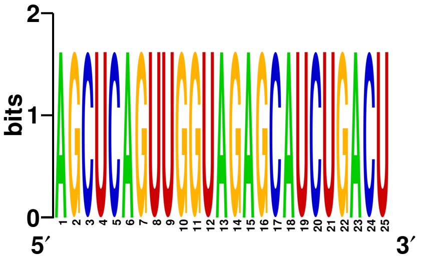

26-mers:

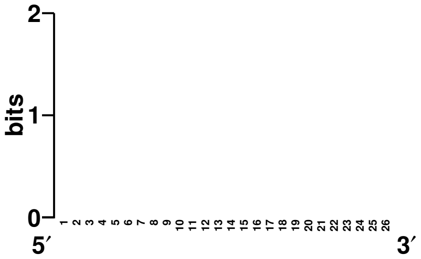

27-mers:

(no read)

28-mers:

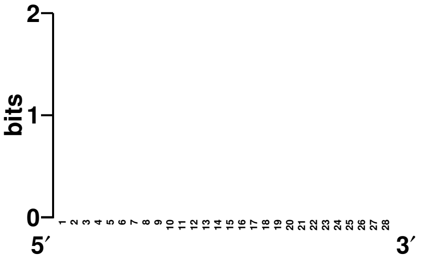

29-mers:

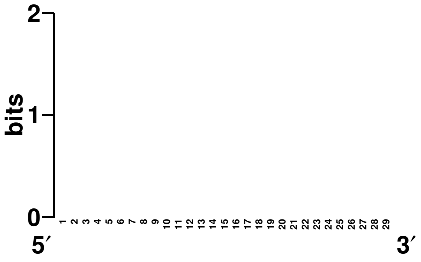

30-mers:

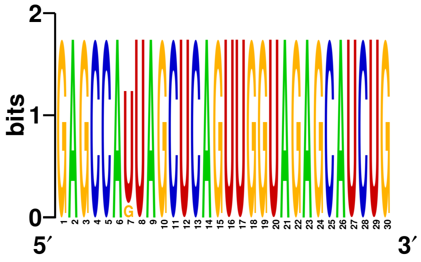

10.4 Libraries #4 (3´ modified, 5´ hydroxyl or polyphosphorylated small RNAs)

Embryo 8h, library 4:

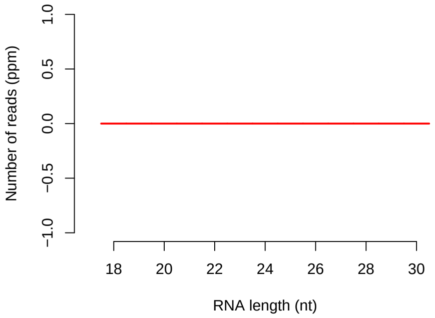

18-mers:

(no read)

19-mers: (no read)

20-mers: (no read)

21-mers: (no read)

22-mers: (no read)

23-mers: (no read)

24-mers: (no read)

25-mers: (no read)

26-mers: (no read)

27-mers: (no read)

28-mers: (no read)

29-mers: (no read)

30-mers: (no read)

Embryo 15h, library 4:

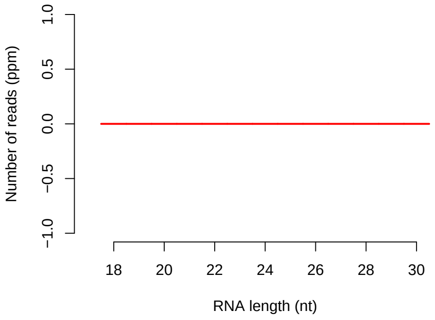

18-mers:

(no read)

19-mers:

(no read)

20-mers:

(no read)

21-mers:

(no read)

22-mers:

(no read)

23-mers:

(no read)

24-mers:

(no read)

25-mers:

(no read)

26-mers:

(no read)

27-mers:

(no read)

28-mers:

(no read)

29-mers:

(no read)

30-mers:

(no read)

Embryo 36h, library 4:

18-mers:

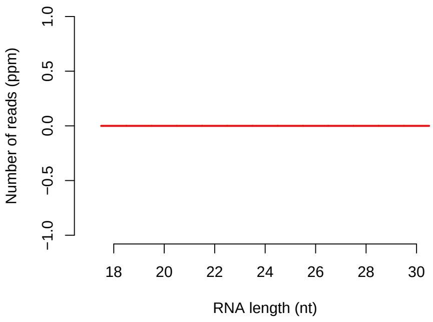

(no read)

19-mers:	20-mers:	21-mers:
(no read)	(no read)	(no read)
22-mers:	23-mers:	24-mers:
(no read)	(no read)	(no read)
25-mers:	26-mers:	27-mers:
(no read)	(no read)	(no read)
28-mers:	29-mers:	30-mers:
(no read)	(no read)	(no read)

Embryo 60h, library 4:

18-mers:

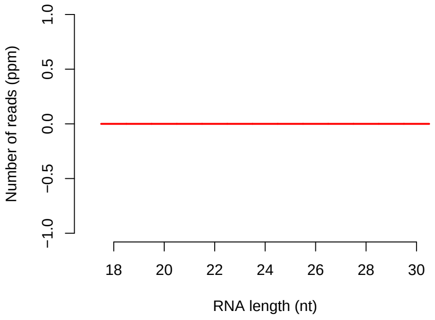

(no read)

19-mers:	20-mers:	21-mers:
(no read)	(no read)	(no read)
22-mers:	23-mers:	24-mers:
(no read)	(no read)	(no read)
25-mers:	26-mers:	27-mers:
(no read)	(no read)	(no read)
28-mers:	29-mers:	30-mers:
(no read)	(no read)	(no read)

Adult female, library 4:

18-mers:

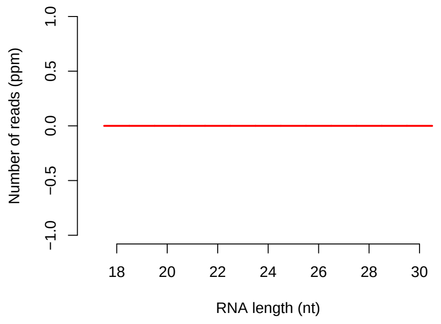

(no read)

19-mers:	20-mers:	21-mers:
(no read)	(no read)	(no read)
22-mers:	23-mers:	24-mers:
(no read)	(no read)	(no read)
25-mers:	26-mers:	27-mers:
(no read)	(no read)	(no read)
28-mers:	29-mers:	30-mers:
(no read)	(no read)	(no read)

Adult male, library 4:

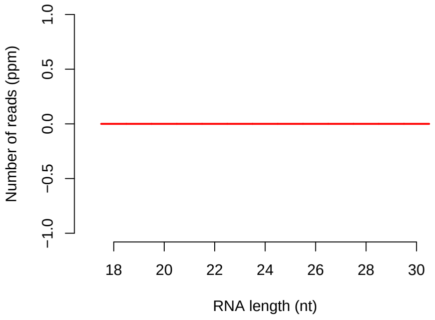

18-mers:

(no read)

19-mers:	20-mers:	21-mers:
(no read)	(no read)	(no read)
22-mers:	23-mers:	24-mers:
(no read)	(no read)	(no read)
25-mers:	26-mers:	27-mers:
(no read)	(no read)	(no read)
28-mers:	29-mers:	30-mers:
(no read)	(no read)	(no read)

11 Extragenomic and extratranscriptomic reads matching extragenomic contig #18690

11.1 Libraries #1 (total 5´ monophosphorylated small RNAs)

Embryo 8h, library 1:

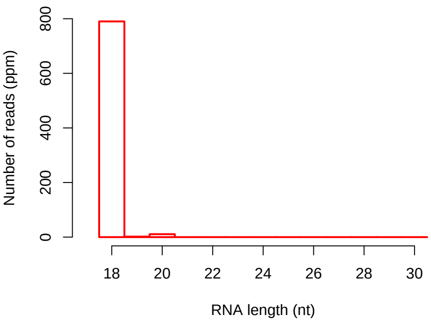

18-mers:

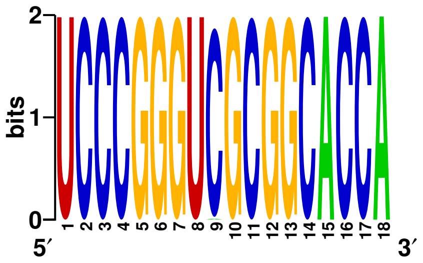

19-mers:

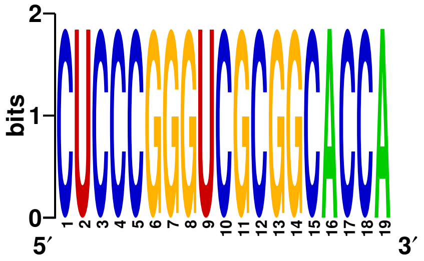

20-mers:

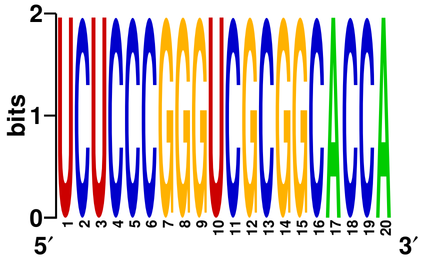

21-mers:

(no read)

22-mers:

(no read)

23-mers:

(no read)

24-mers:

(no read)

25-mers:

(no read)

26-mers:

(no read)

27-mers:

(no read)

28-mers:

(no read)

29-mers:

(no read)

30-mers:

(no read)

Embryo 15h, library 1:

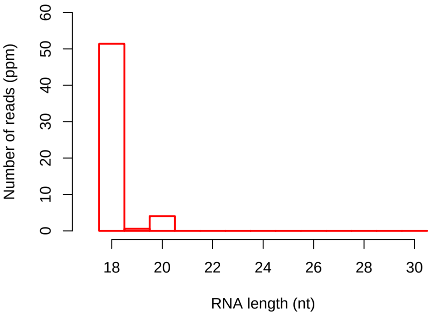

18-mers:

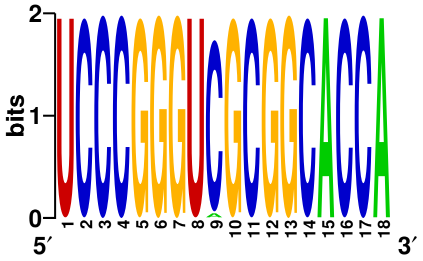

19-mers:

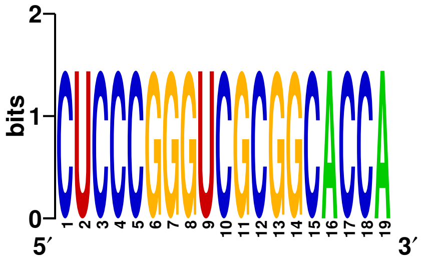

20-mers:

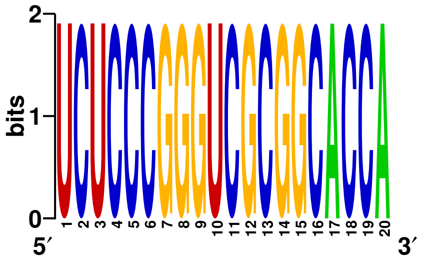

21-mers:

(no read)

22-mers:

(no read)

23-mers:

(no read)

24-mers:

(no read)

25-mers:

(no read)

26-mers:

(no read)

27-mers:

(no read)

28-mers:

(no read)

29-mers:

(no read)

30-mers:

(no read)

Embryo 36h, library 1:

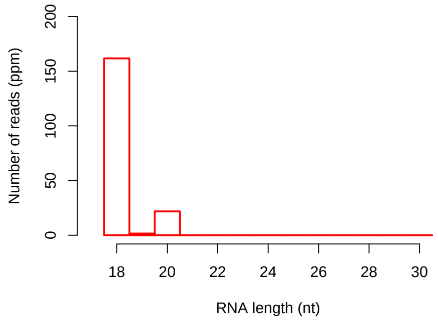

18-mers:

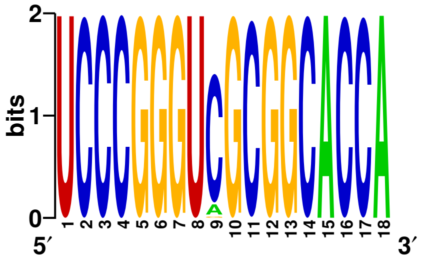

19-mers:

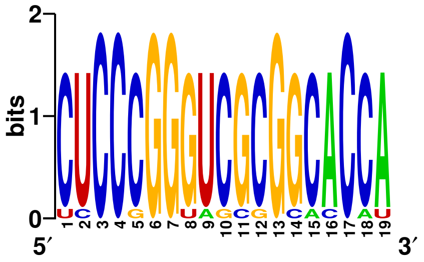

20-mers:

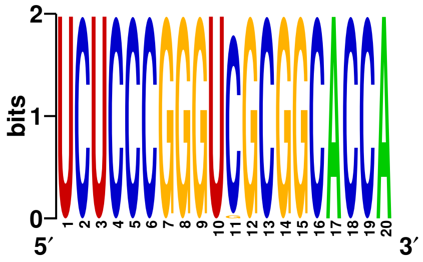

21-mers:

(no read)

22-mers:

(no read)

23-mers:

(no read)

24-mers:

(no read)

25-mers:

(no read)

26-mers:

(no read)

27-mers:

(no read)

28-mers:

(no read)

29-mers:

(no read)

30-mers:

(no read)

Embryo 60h, library 1:

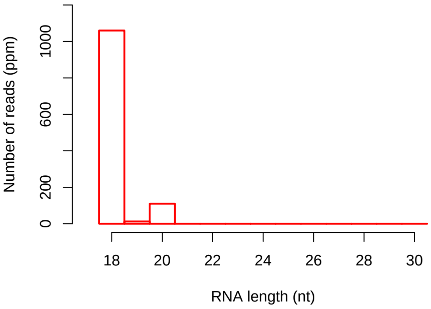

18-mers:

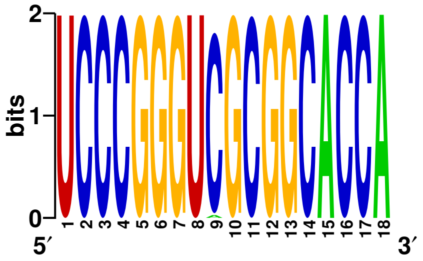

19-mers:

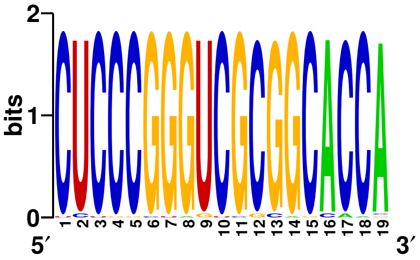

20-mers:

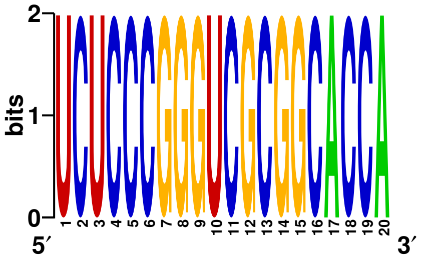

21-mers:

(no read)

22-mers:

(no read)

23-mers:

(no read)

24-mers:

(no read)

25-mers:

(no read)

26-mers:

(no read)

27-mers:

(no read)

28-mers:

(no read)

29-mers:

(no read)

30-mers:

(no read)

Adult female, library 1:

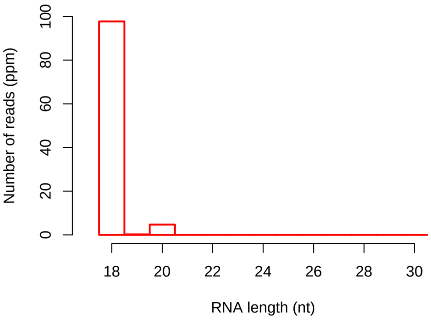

18-mers:

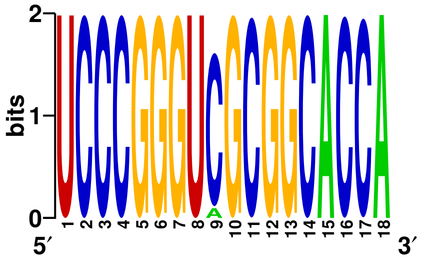

19-mers:

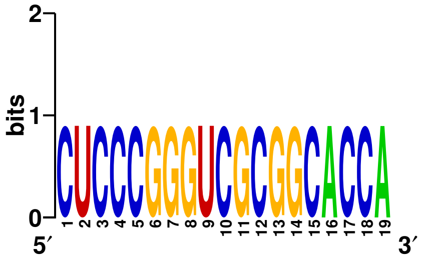

20-mers:

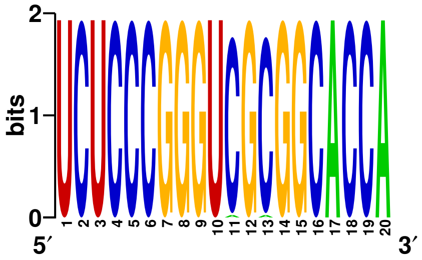

21-mers:

(no read)

22-mers:

(no read)

23-mers:

(no read)

24-mers:

(no read)

25-mers:

(no read)

26-mers:

(no read)

27-mers:

(no read)

28-mers:

(no read)

29-mers:

(no read)

30-mers:

(no read)

Adult male, library 1:

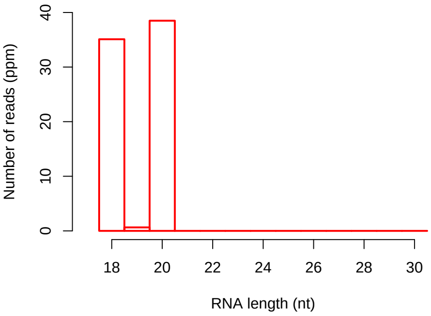

18-mers:

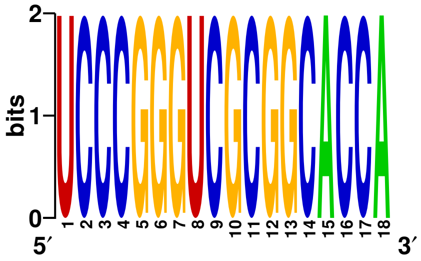

19-mers:

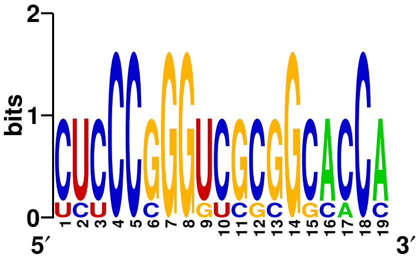

20-mers:

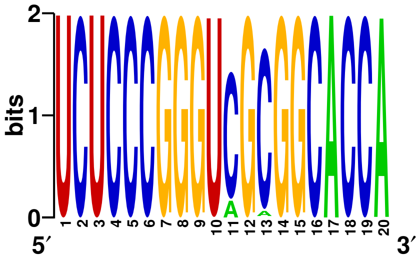

21-mers:
(no read)

22-mers:
(no read)

23-mers:
(no read)

24-mers:
(no read)

25-mers:
(no read)

26-mers:
(no read)

27-mers:
(no read)

28-mers:
(no read)

29-mers:
(no read)

30-mers:
(no read)

11.2 Libraries #2 (3´ modified, 5´ monophosphorylated small RNAs)

Embryo 8h, library 2:

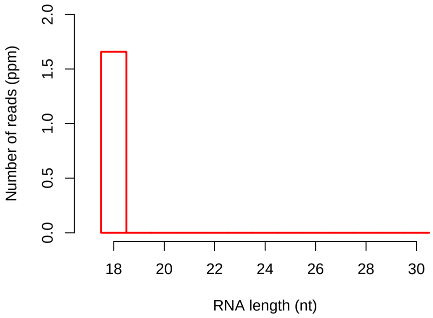

18-mers:

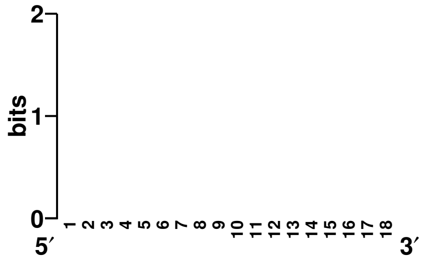

19-mers:	20-mers:	21-mers:
(no read)	(no read)	(no read)
22-mers:	23-mers:	24-mers:
(no read)	(no read)	(no read)
25-mers:	26-mers:	27-mers:
(no read)	(no read)	(no read)
28-mers:	29-mers:	30-mers:
(no read)	(no read)	(no read)

Embryo 15h, library 2:

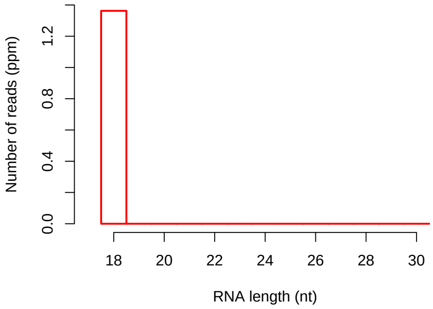

18-mers:

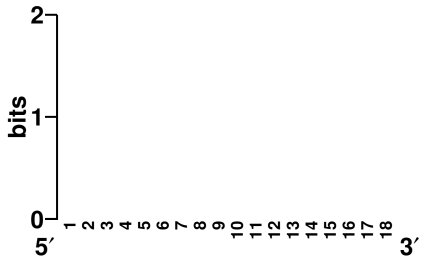

19-mers:	20-mers:	21-mers:
(no read)	(no read)	(no read)
22-mers:	23-mers:	24-mers:
(no read)	(no read)	(no read)
25-mers:	26-mers:	27-mers:
(no read)	(no read)	(no read)
28-mers:	29-mers:	30-mers:
(no read)	(no read)	(no read)

Embryo 36h, library 2:

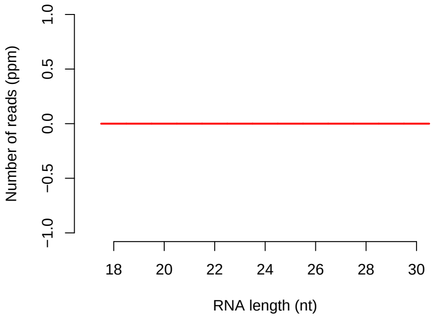

18-mers:

(no read)

19-mers:	20-mers:	21-mers:
(no read)	(no read)	(no read)
22-mers:	23-mers:	24-mers:
(no read)	(no read)	(no read)
25-mers:	26-mers:	27-mers:
(no read)	(no read)	(no read)
28-mers:	29-mers:	30-mers:
(no read)	(no read)	(no read)

Embryo 60h, library 2:

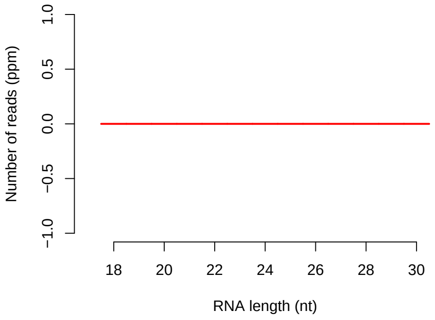

18-mers:
(no read)

19-mers:
(no read)

20-mers:
(no read)

21-mers:
(no read)

22-mers:
(no read)

23-mers:
(no read)

24-mers:
(no read)

25-mers:
(no read)

26-mers:
(no read)

27-mers:
(no read)

28-mers:
(no read)

29-mers:
(no read)

30-mers:
(no read)

Adult female, library 2:

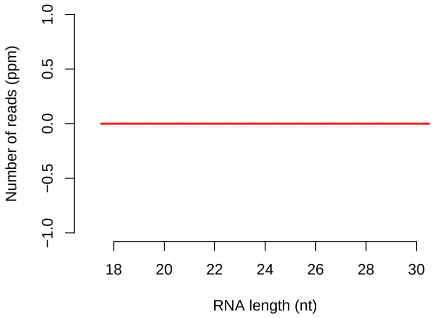

18-mers:

(no read)

19-mers:

(no read)

20-mers:

(no read)

21-mers:

(no read)

22-mers:

(no read)

23-mers:

(no read)

24-mers:

(no read)

25-mers:

(no read)

26-mers:

(no read)

27-mers:

(no read)

28-mers:

(no read)

29-mers:

(no read)

30-mers:

(no read)

Adult male, library 2:

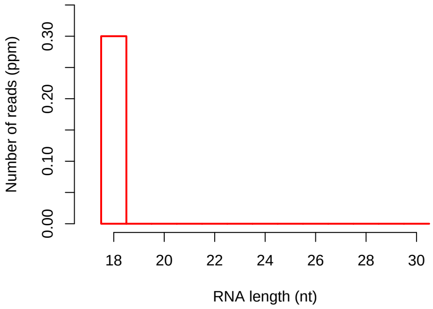

18-mers:

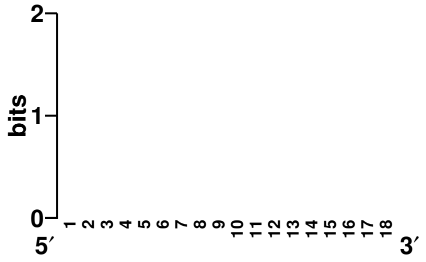

19-mers:	20-mers:	21-mers:
(no read)	(no read)	(no read)
22-mers:	23-mers:	24-mers:
(no read)	(no read)	(no read)
25-mers:	26-mers:	27-mers:
(no read)	(no read)	(no read)
28-mers:	29-mers:	30-mers:
(no read)	(no read)	(no read)

11.3 Libraries #3 (total 5´ hydroxyl or polyphosphorylated small RNAs)

Embryo 8h, library 3:

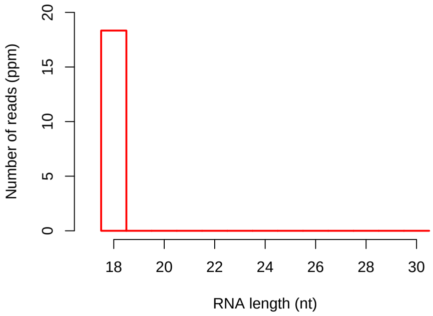

18-mers:

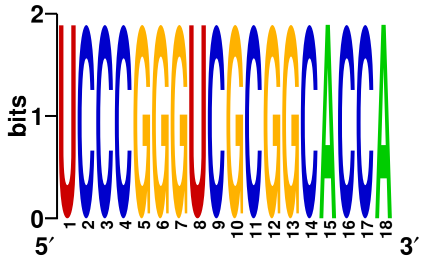

19-mers:	20-mers:	21-mers:
(no read)	(no read)	(no read)
22-mers:	23-mers:	24-mers:
(no read)	(no read)	(no read)
25-mers:	26-mers:	27-mers:
(no read)	(no read)	(no read)
28-mers:	29-mers:	30-mers:
(no read)	(no read)	(no read)

Embryo 15h, library 3:

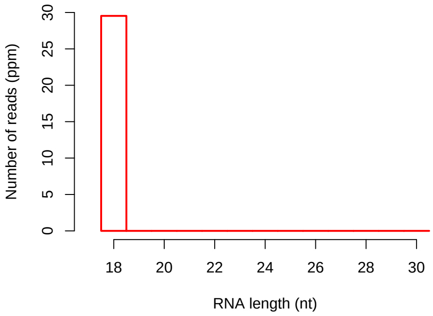

18-mers:

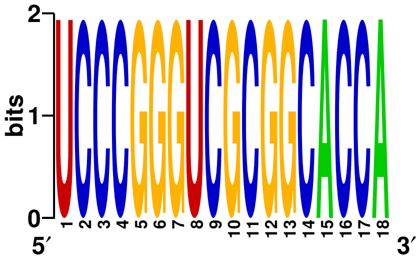

19-mers:	20-mers:	21-mers:
(no read)	(no read)	(no read)
22-mers:	23-mers:	24-mers:
(no read)	(no read)	(no read)
25-mers:	26-mers:	27-mers:
(no read)	(no read)	(no read)
28-mers:	29-mers:	30-mers:
(no read)	(no read)	(no read)

Embryo 36h, library 3:

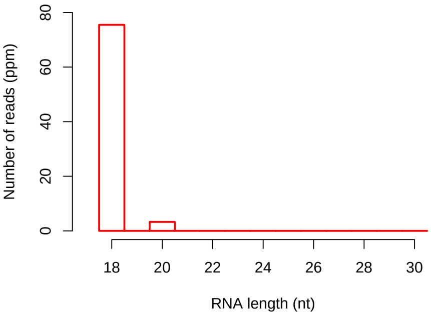

18-mers:

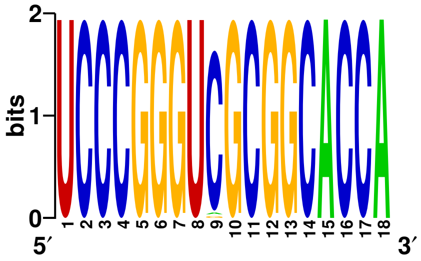

19-mers:

(no read)

20-mers:

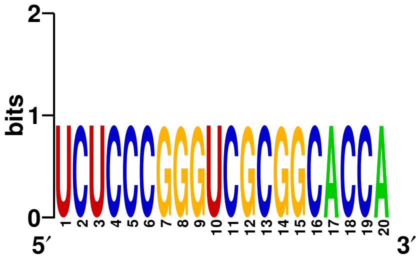

21-mers:

(no read)

22-mers:

(no read)

23-mers:

(no read)

24-mers:

(no read)

25-mers:

(no read)

26-mers:

(no read)

27-mers:

(no read)

28-mers:

(no read)

29-mers:

(no read)

30-mers:

(no read)

Embryo 60h, library 3:

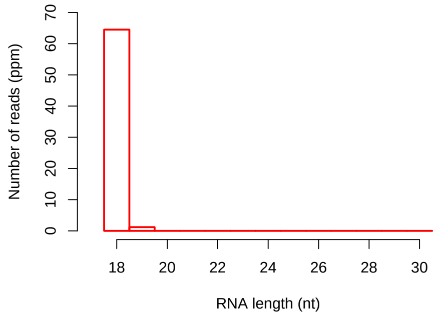

18-mers:

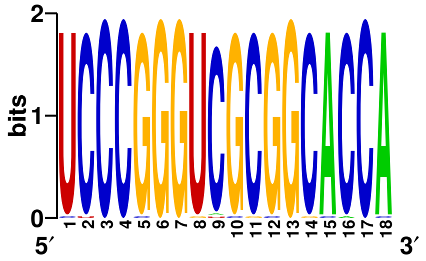

19-mers:

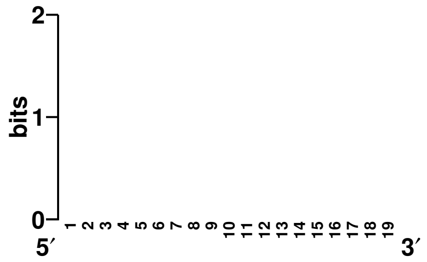

20-mers:

(no read)

21-mers:

(no read)

22-mers:

(no read)

23-mers:

(no read)

24-mers:

(no read)

25-mers:

(no read)

26-mers:

(no read)

27-mers:

(no read)

28-mers:

(no read)

29-mers:

(no read)

30-mers:

(no read)

Adult female, library 3:

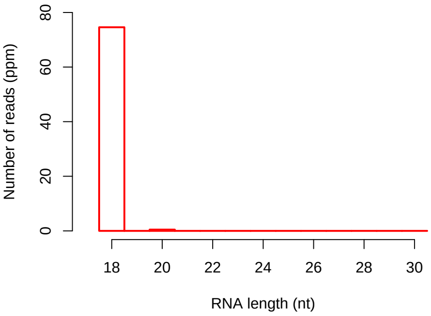

18-mers:

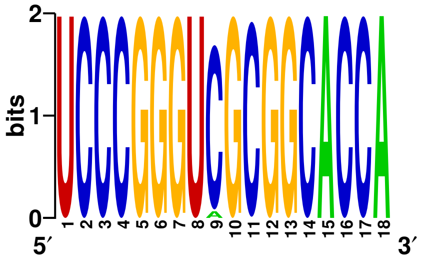

19-mers:

(no read)

20-mers:

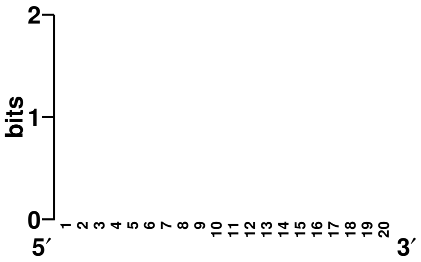

21-mers:

(no read)

22-mers:

(no read)

23-mers:

(no read)

24-mers:

(no read)

25-mers:

(no read)

26-mers:

(no read)

27-mers:

(no read)

28-mers:

(no read)

29-mers:

(no read)

30-mers:

(no read)

Adult male, library 3:

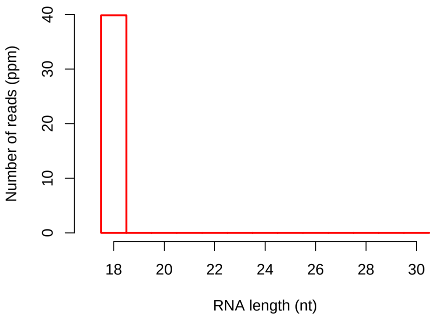

18-mers:

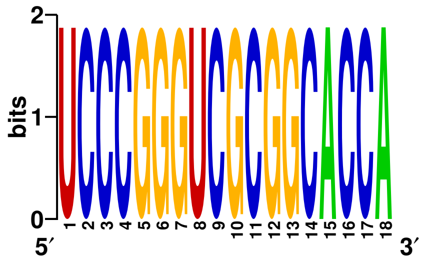

19-mers:	20-mers:	21-mers:
(no read)	(no read)	(no read)
22-mers:	23-mers:	24-mers:
(no read)	(no read)	(no read)
25-mers:	26-mers:	27-mers:
(no read)	(no read)	(no read)
28-mers:	29-mers:	30-mers:
(no read)	(no read)	(no read)

11.4 Libraries #4 (3´ modified, 5´ hydroxyl or polyphosphorylated small RNAs)

Embryo 8h, library 4:

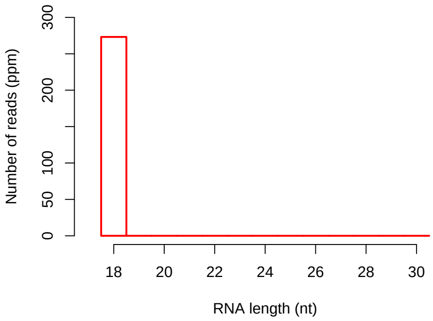

18-mers:

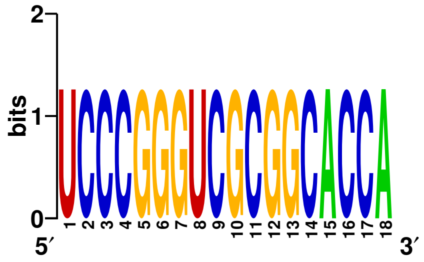

19-mers:	20-mers:	21-mers:
(no read)	(no read)	(no read)
22-mers:	23-mers:	24-mers:
(no read)	(no read)	(no read)
25-mers:	26-mers:	27-mers:
(no read)	(no read)	(no read)
28-mers:	29-mers:	30-mers:
(no read)	(no read)	(no read)

Embryo 15h, library 4:

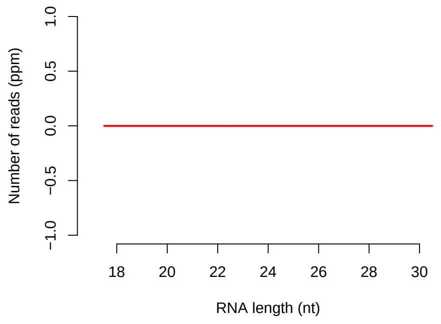

18-mers:

(no read)

19-mers:	20-mers:	21-mers:
(no read)	(no read)	(no read)
22-mers:	23-mers:	24-mers:
(no read)	(no read)	(no read)
25-mers:	26-mers:	27-mers:
(no read)	(no read)	(no read)
28-mers:	29-mers:	30-mers:
(no read)	(no read)	(no read)

Embryo 36h, library 4:

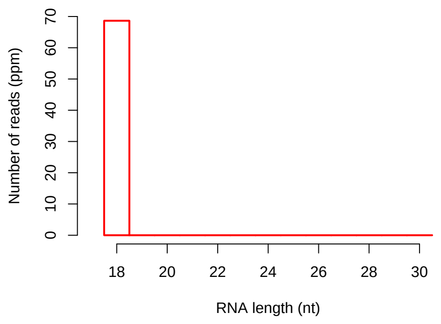

18-mers:

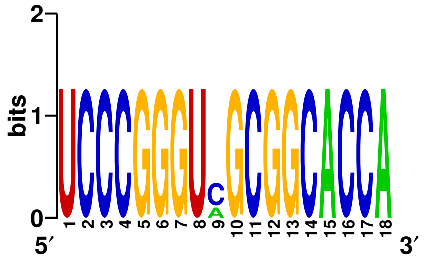

19-mers:	20-mers:	21-mers:
(no read)	(no read)	(no read)
22-mers:	23-mers:	24-mers:
(no read)	(no read)	(no read)
25-mers:	26-mers:	27-mers:
(no read)	(no read)	(no read)
28-mers:	29-mers:	30-mers:
(no read)	(no read)	(no read)

Embryo 60h, library 4:

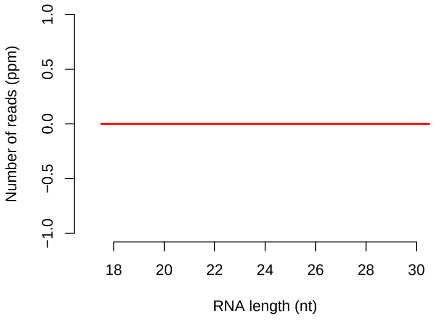

18-mers:

(no read)

19-mers:	20-mers:	21-mers:
(no read)	(no read)	(no read)
22-mers:	23-mers:	24-mers:
(no read)	(no read)	(no read)
25-mers:	26-mers:	27-mers:
(no read)	(no read)	(no read)
28-mers:	29-mers:	30-mers:
(no read)	(no read)	(no read)

Adult female, library 4:

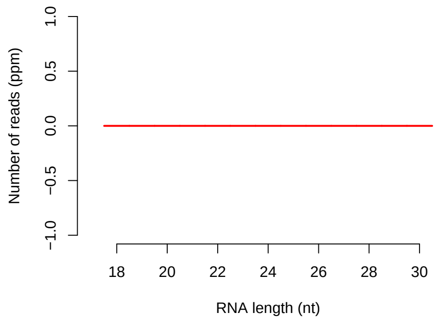

		18-mers:
		(no read)
19-mers:	20-mers:	21-mers:
(no read)	(no read)	(no read)
22-mers:	23-mers:	24-mers:
(no read)	(no read)	(no read)
25-mers:	26-mers:	27-mers:
(no read)	(no read)	(no read)
28-mers:	29-mers:	30-mers:
(no read)	(no read)	(no read)

Adult male, library 4:

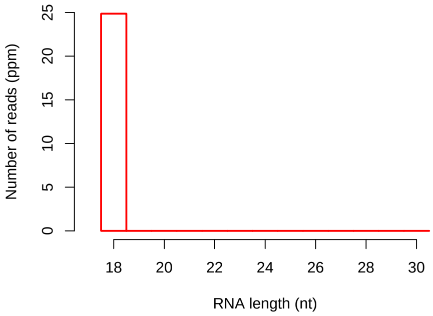

18-mers:

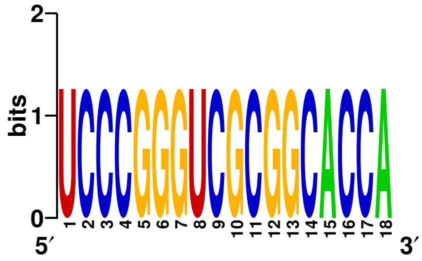

19-mers:	20-mers:	21-mers:
(no read)	(no read)	(no read)
22-mers:	23-mers:	24-mers:
(no read)	(no read)	(no read)
25-mers:	26-mers:	27-mers:
(no read)	(no read)	(no read)
28-mers:	29-mers:	30-mers:
(no read)	(no read)	(no read)

12 Extragenomic and extratranscriptomic reads matching extragenomic contig #7601

12.1 Libraries #1 (total 5´ monophosphorylated small RNAs)

Embryo 8h, library 1:

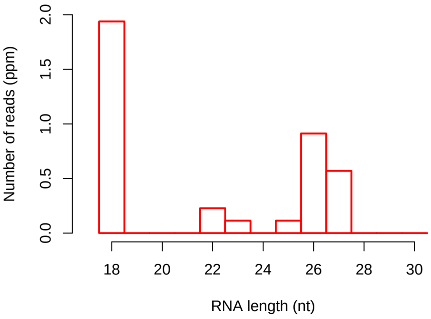

18-mers:

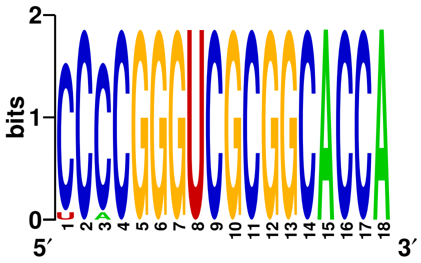

19-mers:
(no read)

20-mers:
(no read)

21-mers:
(no read)

22-mers:

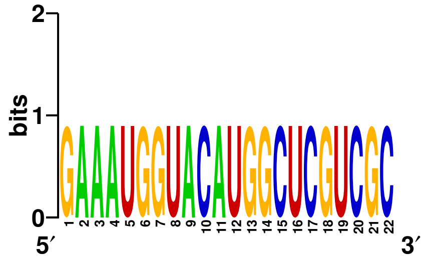

23-mers:

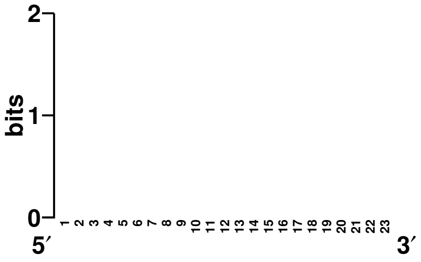

24-mers:
(no read)

25-mers:

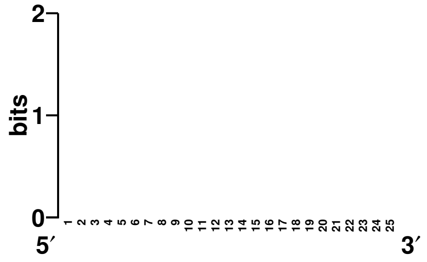

26-mers:

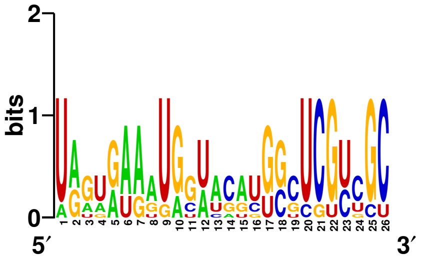

27-mers:

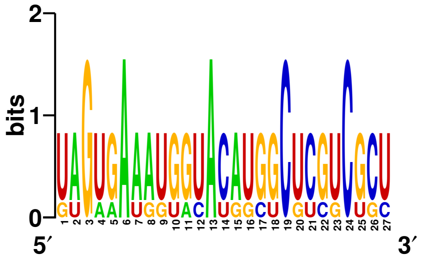

28-mers:
(no read)

29-mers:
(no read)

30-mers:
(no read)

Embryo 15h, library 1:

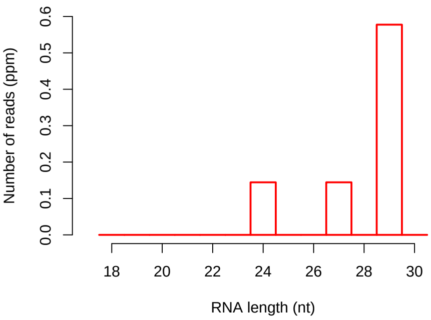

18-mers:

(no read)

19-mers:

(no read)

20-mers:

(no read)

21-mers:

(no read)

22-mers:

(no read)

23-mers:

(no read)

24-mers:

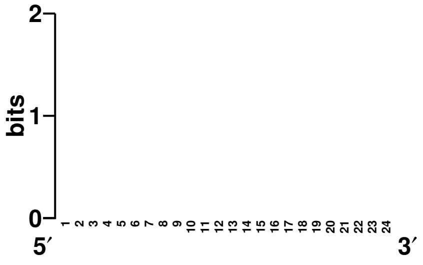

25-mers:

(no read)

26-mers:

(no read)

27-mers:

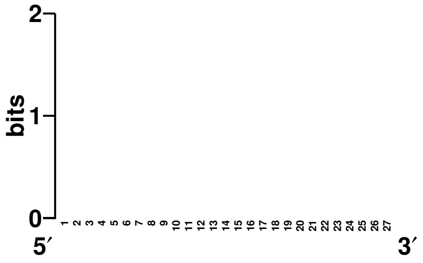

28-mers:

(no read)

29-mers:

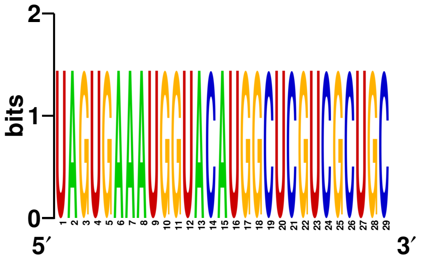

30-mers:

(no read)

Embryo 36h, library 1:

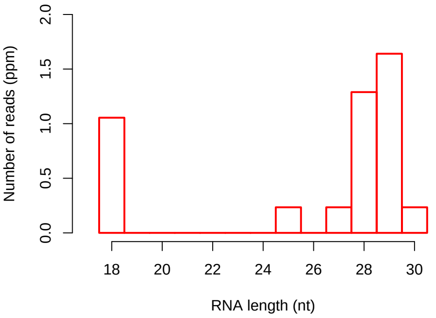

18-mers:

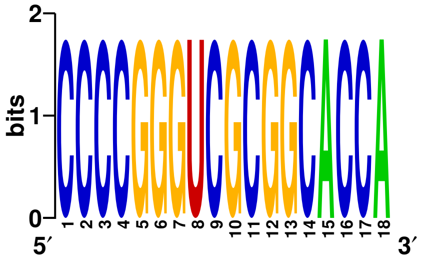

19-mers:
(no read)

20-mers:
(no read)

21-mers:
(no read)

22-mers:
(no read)

23-mers:
(no read)

24-mers:
(no read)

25-mers:

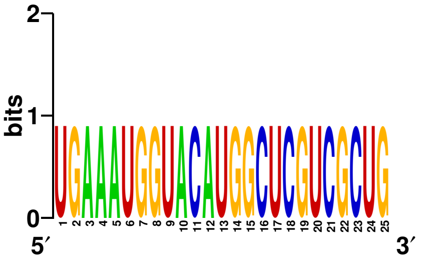

26-mers:
(no read)

27-mers:

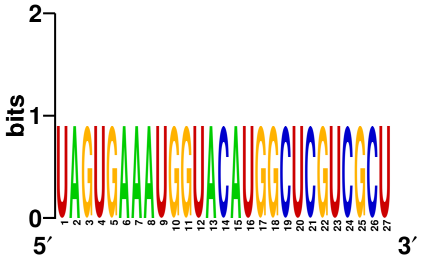

28-mers:

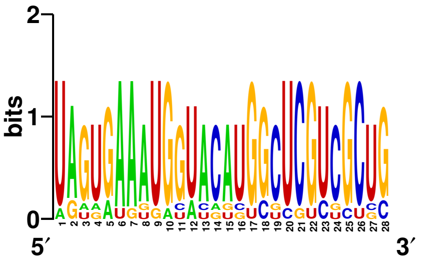

29-mers:

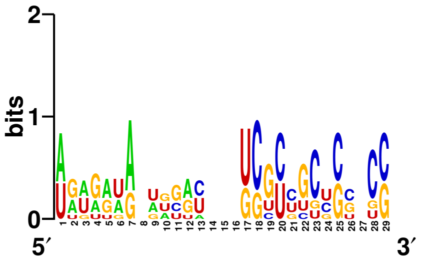

30-mers:

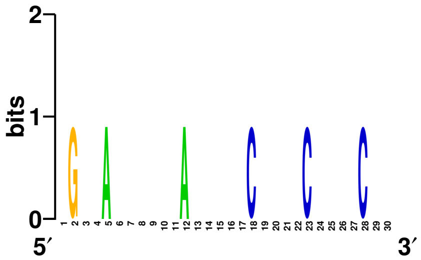

Embryo 60h, library 1:

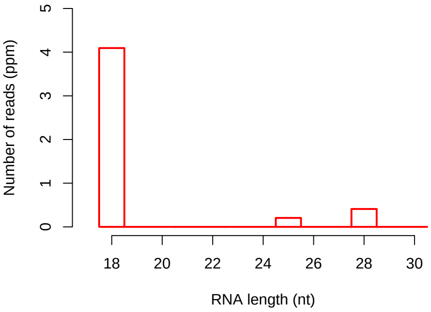

18-mers:

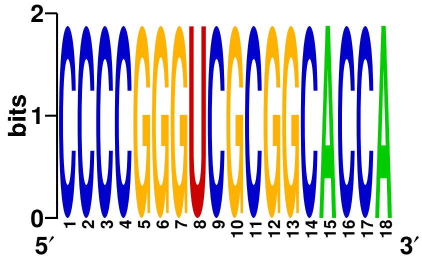

19-mers:
(no read)

20-mers:
(no read)

21-mers:
(no read)

22-mers:
(no read)

23-mers:
(no read)

24-mers:
(no read)

25-mers:

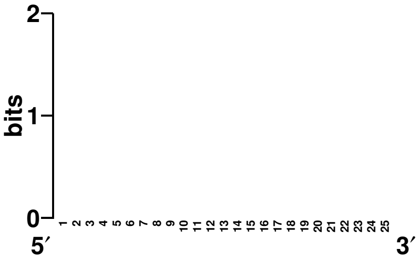

26-mers:
(no read)

27-mers:
(no read)

28-mers:

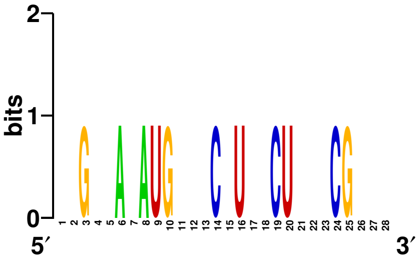

29-mers:
(no read)

30-mers:
(no read)

Adult female, library 1:

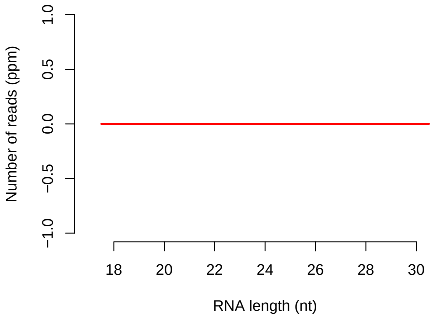

18-mers:
(no read)

19-mers:
(no read)

20-mers:
(no read)

21-mers:
(no read)

22-mers:
(no read)

23-mers:
(no read)

24-mers:
(no read)

25-mers:
(no read)

26-mers:
(no read)

27-mers:
(no read)

28-mers:
(no read)

29-mers:
(no read)

30-mers:
(no read)

Adult male, library 1:

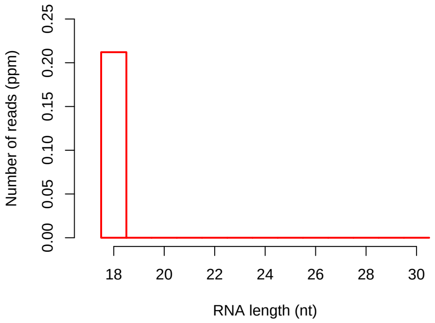

18-mers:

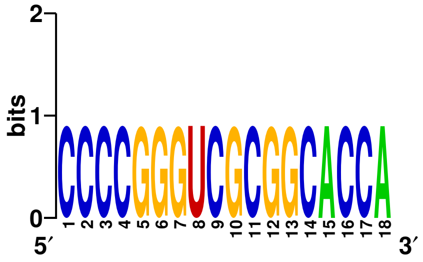

19-mers:	20-mers:	21-mers:
(no read)	(no read)	(no read)
22-mers:	23-mers:	24-mers:
(no read)	(no read)	(no read)
25-mers:	26-mers:	27-mers:
(no read)	(no read)	(no read)
28-mers:	29-mers:	30-mers:
(no read)	(no read)	(no read)

12.2 Libraries #2 (3´ modified, 5´ monophosphorylated small RNAs)

Embryo 8h, library 2:

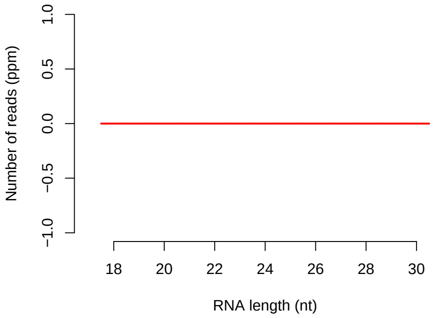

18-mers:

(no read)

19-mers:	20-mers:	21-mers:
(no read)	(no read)	(no read)
22-mers:	23-mers:	24-mers:
(no read)	(no read)	(no read)
25-mers:	26-mers:	27-mers:
(no read)	(no read)	(no read)
28-mers:	29-mers:	30-mers:
(no read)	(no read)	(no read)

Embryo 15h, library 2:

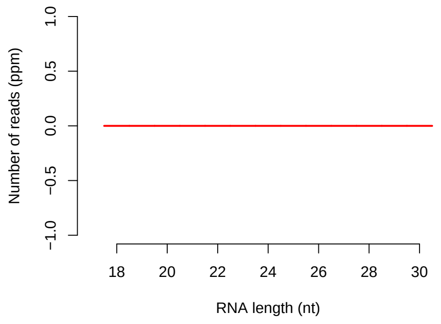

18-mers:

(no read)

19-mers:

(no read)

20-mers:

(no read)

21-mers:

(no read)

22-mers:

(no read)

23-mers:

(no read)

24-mers:

(no read)

25-mers:

(no read)

26-mers:

(no read)

27-mers:

(no read)

28-mers:

(no read)

29-mers:

(no read)

30-mers:

(no read)

Embryo 36h, library 2:

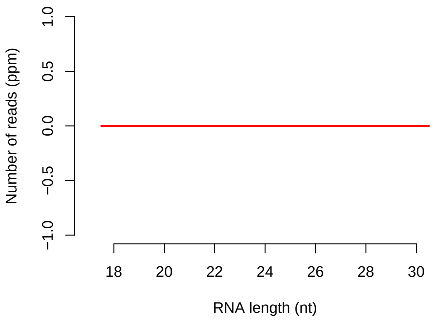

18-mers:
(no read)

19-mers:
(no read)

20-mers:
(no read)

21-mers:
(no read)

22-mers:
(no read)

23-mers:
(no read)

24-mers:
(no read)

25-mers:
(no read)

26-mers:
(no read)

27-mers:
(no read)

28-mers:
(no read)

29-mers:
(no read)

30-mers:
(no read)

Embryo 60h, library 2:

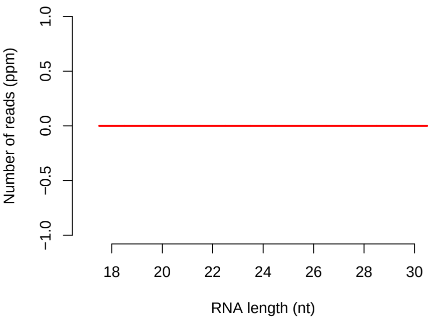

18-mers:

(no read)

19-mers:	20-mers:	21-mers:
(no read)	(no read)	(no read)
22-mers:	23-mers:	24-mers:
(no read)	(no read)	(no read)
25-mers:	26-mers:	27-mers:
(no read)	(no read)	(no read)
28-mers:	29-mers:	30-mers:
(no read)	(no read)	(no read)

Adult female, library 2:

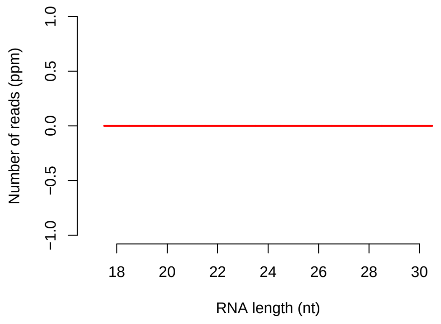

18-mers:

(no read)

19-mers:

(no read)

20-mers:

(no read)

21-mers:

(no read)

22-mers:

(no read)

23-mers:

(no read)

24-mers:

(no read)

25-mers:

(no read)

26-mers:

(no read)

27-mers:

(no read)

28-mers:

(no read)

29-mers:

(no read)

30-mers:

(no read)

Adult male, library 2:

18-mers:

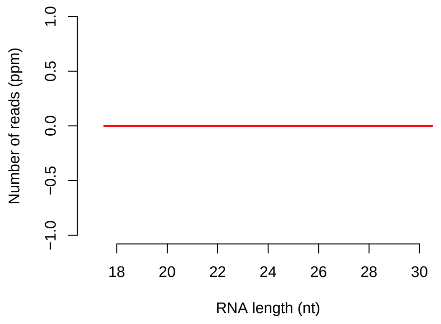

(no read)

19-mers:	20-mers:	21-mers:
(no read)	(no read)	(no read)
22-mers:	23-mers:	24-mers:
(no read)	(no read)	(no read)
25-mers:	26-mers:	27-mers:
(no read)	(no read)	(no read)
28-mers:	29-mers:	30-mers:
(no read)	(no read)	(no read)

12.3 Libraries #3 (total 5´ hydroxyl or polyphosphorylated small RNAs)

Embryo 8h, library 3:

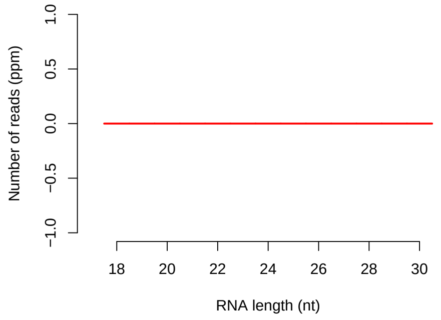

18-mers:

(no read)

19-mers:	20-mers:	21-mers:
(no read)	(no read)	(no read)
22-mers:	23-mers:	24-mers:
(no read)	(no read)	(no read)
25-mers:	26-mers:	27-mers:
(no read)	(no read)	(no read)
28-mers:	29-mers:	30-mers:
(no read)	(no read)	(no read)

Embryo 15h, library 3:

18-mers:

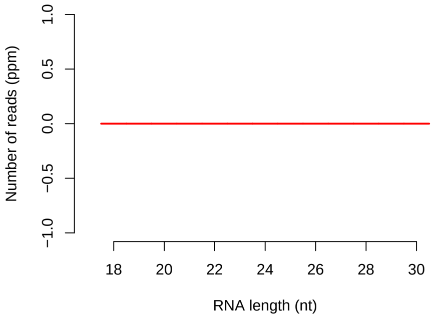

(no read)

19-mers:	20-mers:	21-mers:
(no read)	(no read)	(no read)
22-mers:	23-mers:	24-mers:
(no read)	(no read)	(no read)
25-mers:	26-mers:	27-mers:
(no read)	(no read)	(no read)
28-mers:	29-mers:	30-mers:
(no read)	(no read)	(no read)

Embryo 36h, library 3:

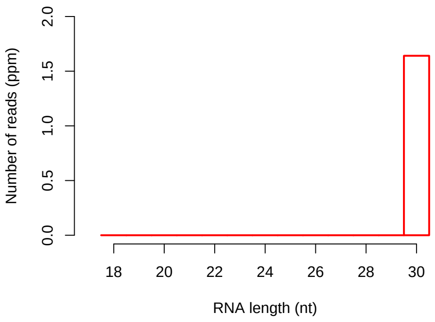

18-mers:

(no read)

19-mers:
(no read)

20-mers:
(no read)

21-mers:
(no read)

22-mers:
(no read)

23-mers:
(no read)

24-mers:
(no read)

25-mers:
(no read)

26-mers:
(no read)

27-mers:
(no read)

28-mers:
(no read)

29-mers:
(no read)

30-mers:

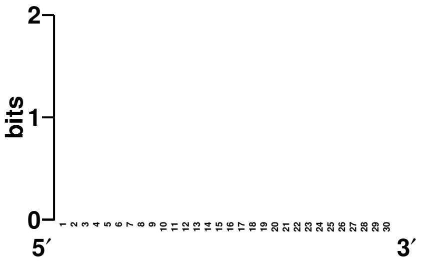

Embryo 60h, library 3:

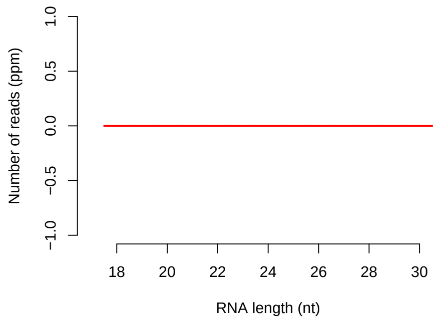

18-mers:

(no read)

19-mers:

(no read)

20-mers:

(no read)

21-mers:

(no read)

22-mers:

(no read)

23-mers:

(no read)

24-mers:

(no read)

25-mers:

(no read)

26-mers:

(no read)

27-mers:

(no read)

28-mers:

(no read)

29-mers:

(no read)

30-mers:

(no read)

Adult female, library 3:

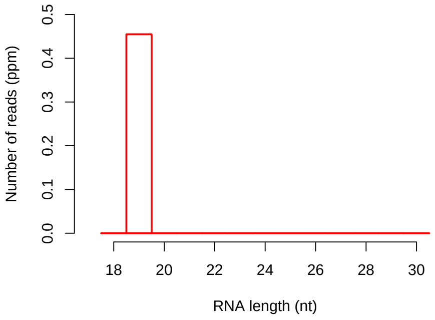

18-mers:

(no read)

19-mers:

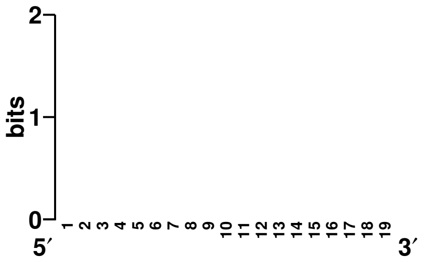

20-mers:

(no read)

21-mers:

(no read)

22-mers:

(no read)

23-mers:

(no read)

24-mers:

(no read)

25-mers:

(no read)

26-mers:

(no read)

27-mers:

(no read)

28-mers:

(no read)

29-mers:

(no read)

30-mers:

(no read)

Adult male, library 3:

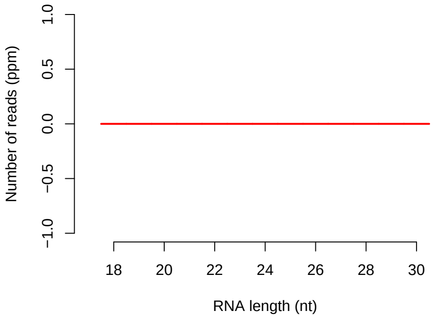

18-mers:
(no read)

19-mers:
(no read)

20-mers:
(no read)

21-mers:
(no read)

22-mers:
(no read)

23-mers:
(no read)

24-mers:
(no read)

25-mers:
(no read)

26-mers:
(no read)

27-mers:
(no read)

28-mers:
(no read)

29-mers:
(no read)

30-mers:
(no read)

12.4 Libraries #4 (3´ modified, 5´ hydroxyl or polyphosphorylated small RNAs)

Embryo 8h, library 4:

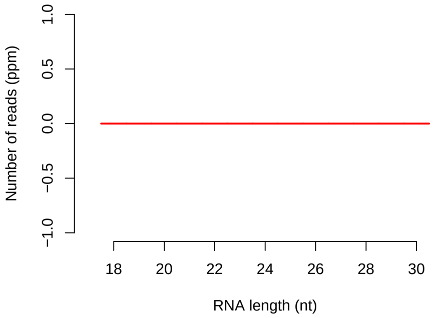

18-mers:

(no read)

19-mers:	20-mers:	21-mers:
(no read)	(no read)	(no read)
22-mers:	23-mers:	24-mers:
(no read)	(no read)	(no read)
25-mers:	26-mers:	27-mers:
(no read)	(no read)	(no read)
28-mers:	29-mers:	30-mers:
(no read)	(no read)	(no read)

Embryo 15h, library 4:

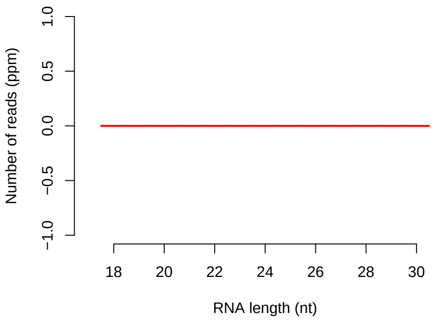

18-mers:

(no read)

19-mers:

(no read)

20-mers:

(no read)

21-mers:

(no read)

22-mers:

(no read)

23-mers:

(no read)

24-mers:

(no read)

25-mers:

(no read)

26-mers:

(no read)

27-mers:

(no read)

28-mers:

(no read)

29-mers:

(no read)

30-mers:

(no read)

Embryo 36h, library 4:

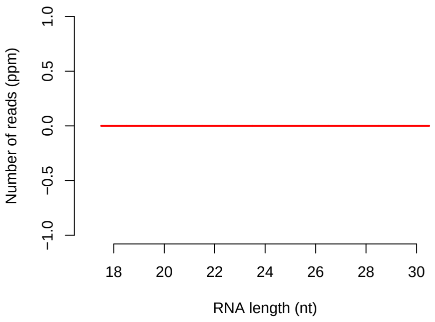

18-mers:
(no read)

19-mers:	20-mers:	21-mers:
(no read)	(no read)	(no read)
22-mers:	23-mers:	24-mers:
(no read)	(no read)	(no read)
25-mers:	26-mers:	27-mers:
(no read)	(no read)	(no read)
28-mers:	29-mers:	30-mers:
(no read)	(no read)	(no read)

Embryo 60h, library 4:

18-mers:

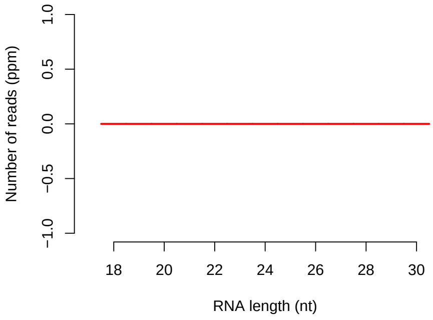

(no read)

19-mers:	20-mers:	21-mers:
(no read)	(no read)	(no read)
22-mers:	23-mers:	24-mers:
(no read)	(no read)	(no read)
25-mers:	26-mers:	27-mers:
(no read)	(no read)	(no read)
28-mers:	29-mers:	30-mers:
(no read)	(no read)	(no read)

Adult female, library 4:

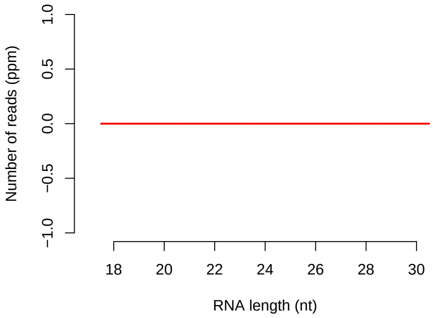

18-mers:

(no read)

19-mers:	20-mers:	21-mers:
(no read)	(no read)	(no read)
22-mers:	23-mers:	24-mers:
(no read)	(no read)	(no read)
25-mers:	26-mers:	27-mers:
(no read)	(no read)	(no read)
28-mers:	29-mers:	30-mers:
(no read)	(no read)	(no read)

Adult male, library 4:

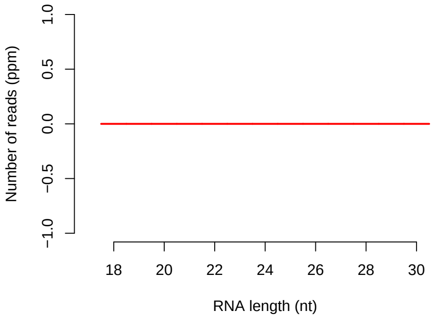

18-mers:

(no read)

19-mers:

(no read)

20-mers:

(no read)

21-mers:

(no read)

22-mers:

(no read)

23-mers:

(no read)

24-mers:

(no read)

25-mers:

(no read)

26-mers:

(no read)

27-mers:

(no read)

28-mers:

(no read)

29-mers:

(no read)

30-mers:

(no read)

13 Extragenomic and extratranscriptomic reads matching extragenomic contig #38312

13.1 Libraries #1 (total 5´ monophosphorylated small RNAs)

Embryo 8h, library 1:

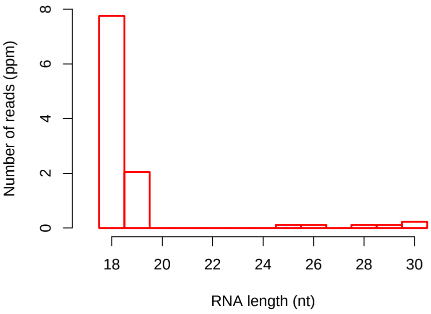

18-mers:

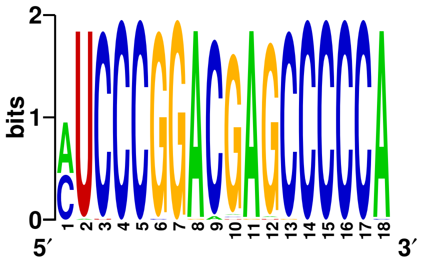

19-mers:

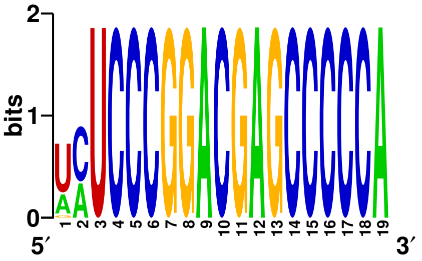

20-mers:

(no read)

21-mers:

(no read)

22-mers:

(no read)

23-mers:

(no read)

24-mers:

(no read)

25-mers:

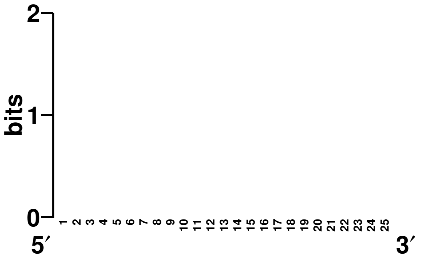

26-mers:

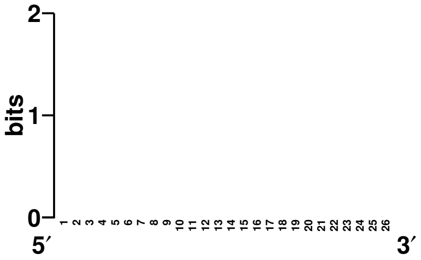

27-mers:

(no read)

28-mers:

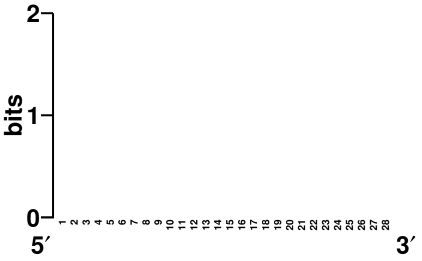

29-mers:

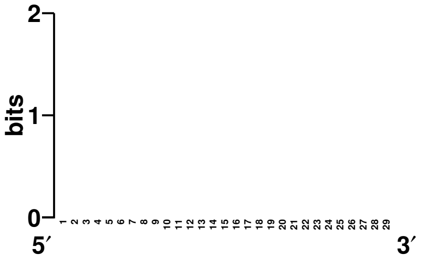

30-mers:

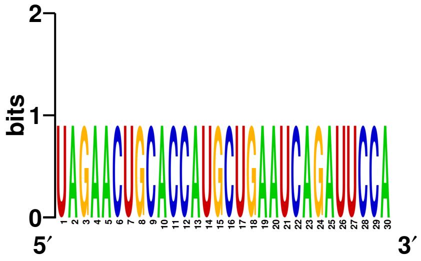

Embryo 15h, library 1:

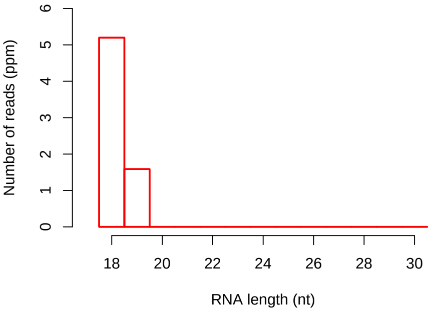

18-mers:

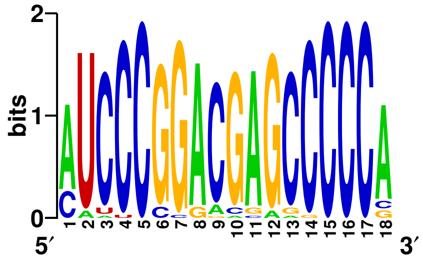

19-mers:

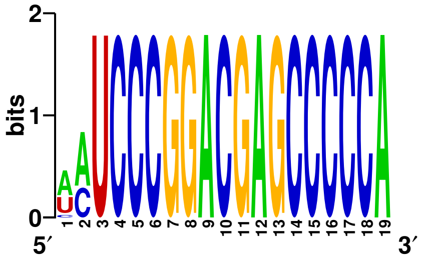

20-mers:

(no read)

21-mers:

(no read)

22-mers:

(no read)

23-mers:

(no read)

24-mers:

(no read)

25-mers:

(no read)

26-mers:

(no read)

27-mers:

(no read)

28-mers:

(no read)

29-mers:

(no read)

30-mers:

(no read)

Embryo 36h, library 1:

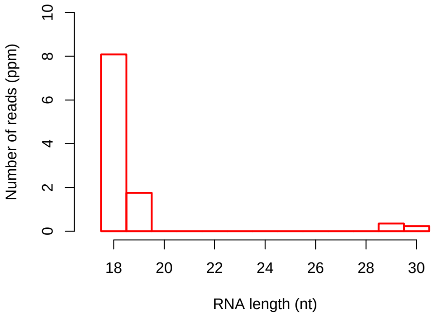

18-mers:

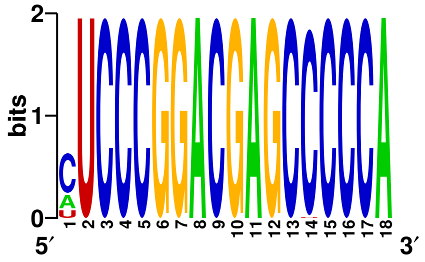

19-mers:

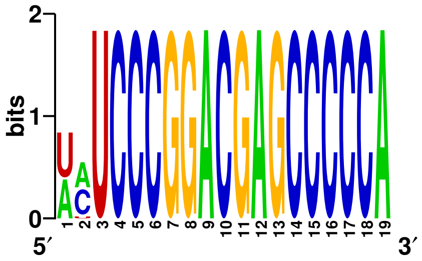

20-mers:

(no read)

21-mers:

(no read)

22-mers:

(no read)

23-mers:

(no read)

24-mers:

(no read)

25-mers:

(no read)

26-mers:

(no read)

27-mers:

(no read)

28-mers:

(no read)

29-mers:

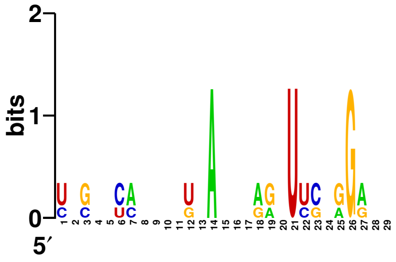

30-mers:

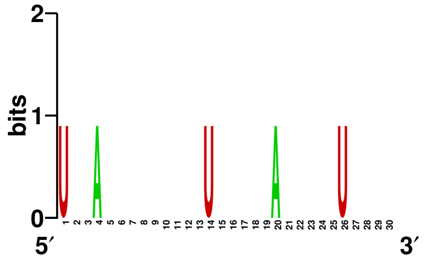

Embryo 60h, library 1:

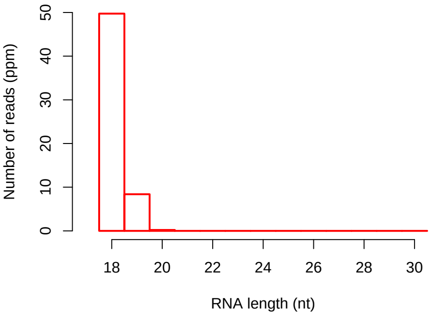

18-mers:

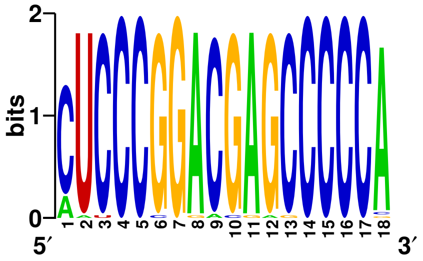

19-mers:

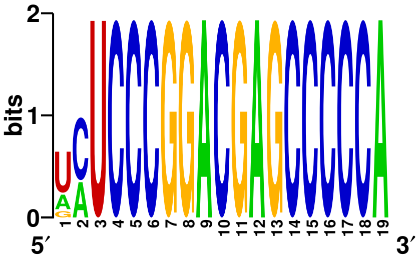

20-mers:

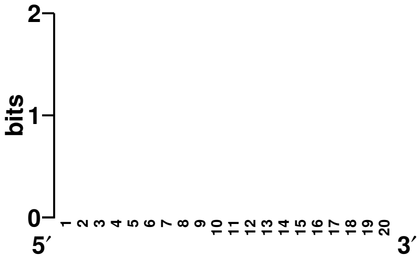

21-mers:

(no read)

22-mers:

(no read)

23-mers:

(no read)

24-mers:

(no read)

25-mers:

(no read)

26-mers:

(no read)

27-mers:

(no read)

28-mers:

(no read)

29-mers:

(no read)

30-mers:

(no read)

Adult female, library 1:

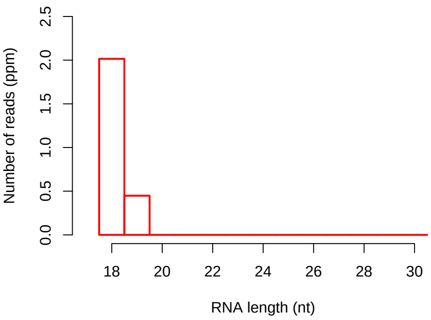

18-mers:

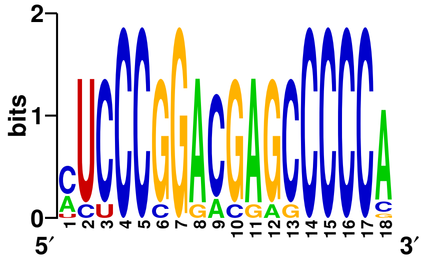

19-mers:

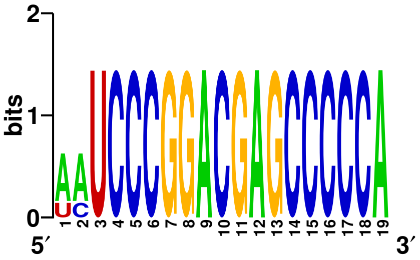

20-mers:

(no read)

21-mers:

(no read)

22-mers:

(no read)

23-mers:

(no read)

24-mers:

(no read)

25-mers:

(no read)

26-mers:

(no read)

27-mers:

(no read)

28-mers:

(no read)

29-mers:

(no read)

30-mers:

(no read)

Adult male, library 1:

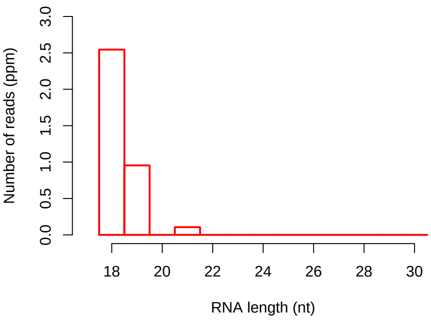

18-mers:

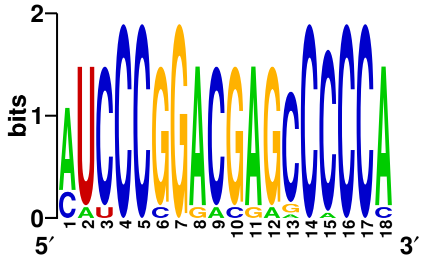

19-mers:

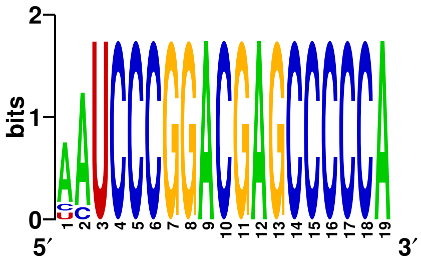

20-mers:

(no read)

21-mers:

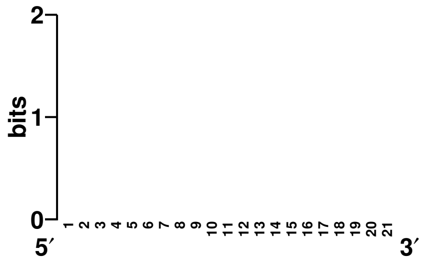

22-mers:
(no read)

23-mers:
(no read)

24-mers:
(no read)

25-mers:
(no read)

26-mers:
(no read)

27-mers:
(no read)

28-mers:
(no read)

29-mers:
(no read)

30-mers:
(no read)

13.2 Libraries #2 (3´ modified, 5´ monophosphorylated small RNAs)

Embryo 8h, library 2:

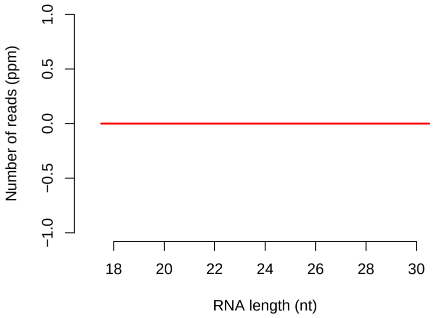

18-mers:

(no read)

19-mers:	20-mers:	21-mers:
(no read)	(no read)	(no read)
22-mers:	23-mers:	24-mers:
(no read)	(no read)	(no read)
25-mers:	26-mers:	27-mers:
(no read)	(no read)	(no read)
28-mers:	29-mers:	30-mers:
(no read)	(no read)	(no read)

Embryo 15h, library 2:

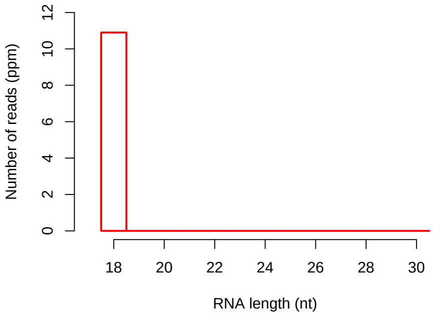

18-mers:

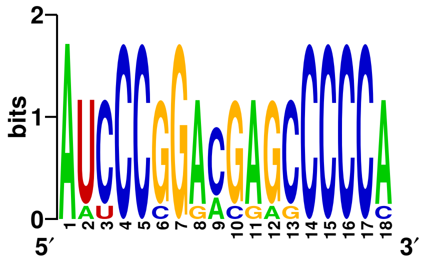

19-mers:
(no read)

20-mers:
(no read)

21-mers:
(no read)

22-mers:
(no read)

23-mers:
(no read)

24-mers:
(no read)

25-mers:
(no read)

26-mers:
(no read)

27-mers:
(no read)

28-mers:
(no read)

29-mers:
(no read)

30-mers:
(no read)

Embryo 36h, library 2:

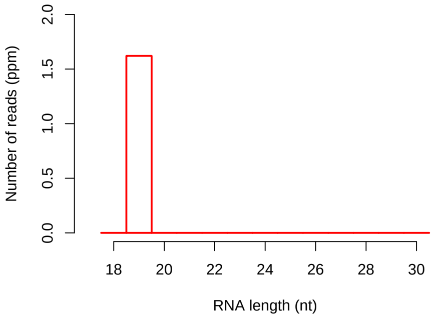

18-mers:

(no read)

19-mers:

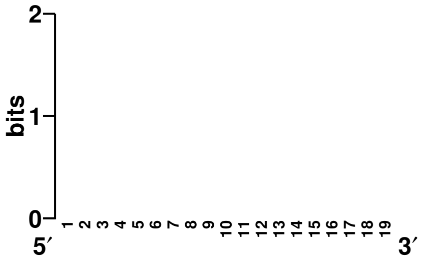

20-mers:

(no read)

21-mers:

(no read)

22-mers:

(no read)

23-mers:

(no read)

24-mers:

(no read)

25-mers:

(no read)

26-mers:

(no read)

27-mers:

(no read)

28-mers:

(no read)

29-mers:

(no read)

30-mers:

(no read)

Embryo 60h, library 2:

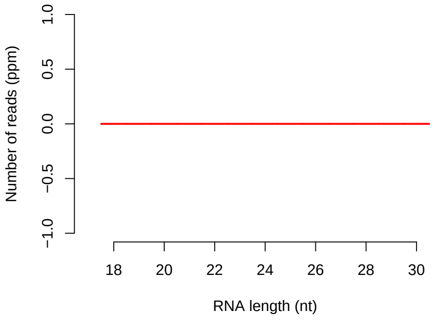

18-mers:
(no read)

19-mers:
(no read)

20-mers:
(no read)

21-mers:
(no read)

22-mers:
(no read)

23-mers:
(no read)

24-mers:
(no read)

25-mers:
(no read)

26-mers:
(no read)

27-mers:
(no read)

28-mers:
(no read)

29-mers:
(no read)

30-mers:
(no read)

Adult female, library 2:

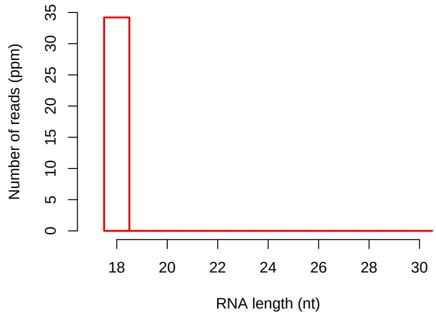

18-mers:

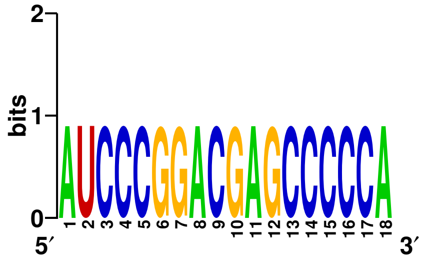

19-mers:	20-mers:	21-mers:
(no read)	(no read)	(no read)
22-mers:	23-mers:	24-mers:
(no read)	(no read)	(no read)
25-mers:	26-mers:	27-mers:
(no read)	(no read)	(no read)
28-mers:	29-mers:	30-mers:
(no read)	(no read)	(no read)

Adult male, library 2:

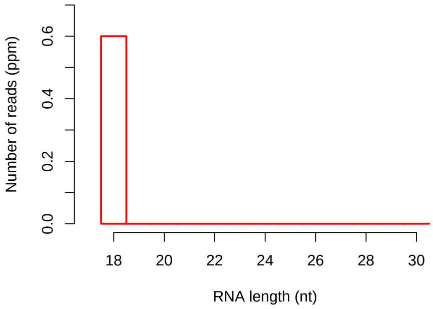

18-mers:

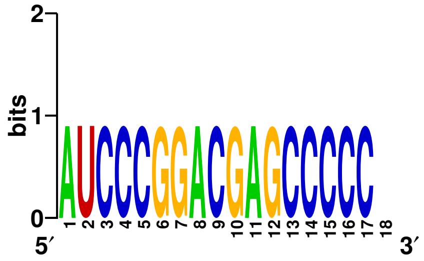

19-mers:	20-mers:	21-mers:
(no read)	(no read)	(no read)
22-mers:	23-mers:	24-mers:
(no read)	(no read)	(no read)
25-mers:	26-mers:	27-mers:
(no read)	(no read)	(no read)
28-mers:	29-mers:	30-mers:
(no read)	(no read)	(no read)

13.3 Libraries #3 (total 5´ hydroxyl or polyphosphorylated small RNAs)

Embryo 8h, library 3:

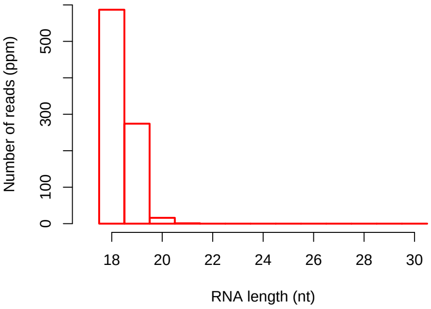

18-mers:

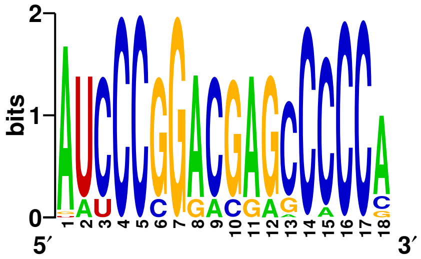

19-mers:

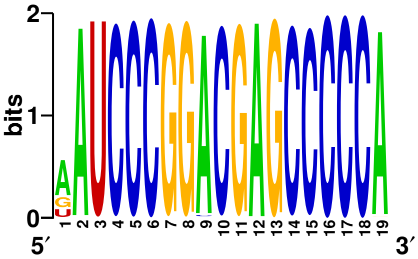

20-mers:

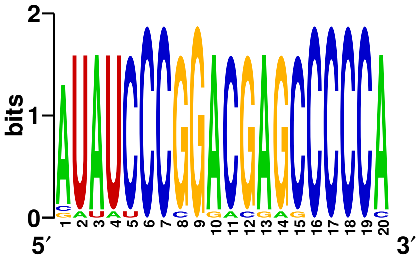

21-mers:

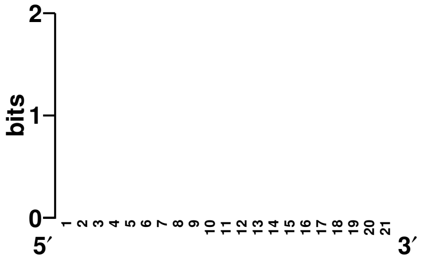

22-mers:
(no read)

23-mers:
(no read)

24-mers:
(no read)

25-mers:
(no read)

26-mers:
(no read)

27-mers:
(no read)

28-mers:
(no read)

29-mers:
(no read)

30-mers:
(no read)

Embryo 15h, library 3:

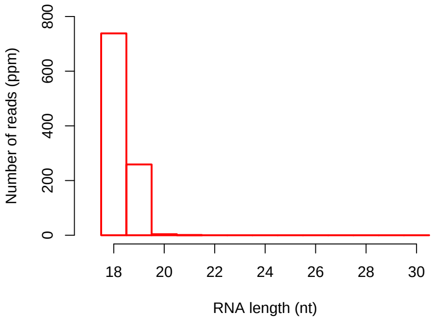

18-mers:

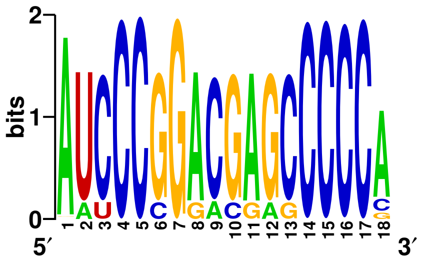

19-mers:

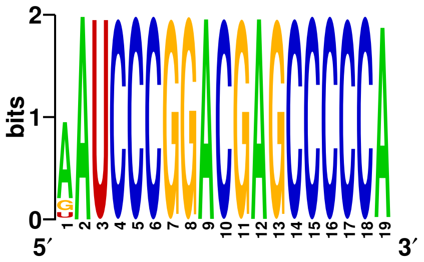

20-mers:

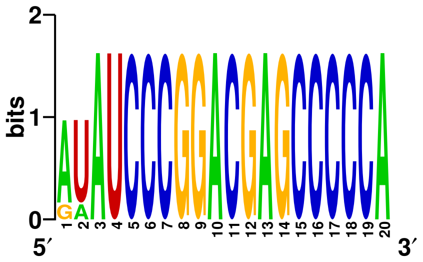

21-mers:

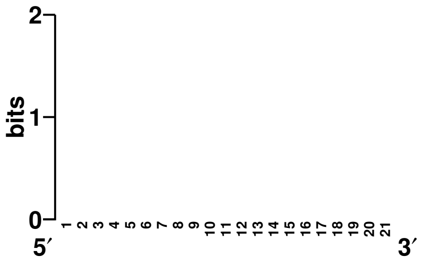

22-mers:
(no read)

23-mers:
(no read)

24-mers:
(no read)

25-mers:
(no read)

26-mers:
(no read)

27-mers:
(no read)

28-mers:
(no read)

29-mers:
(no read)

30-mers:
(no read)

Embryo 36h, library 3:

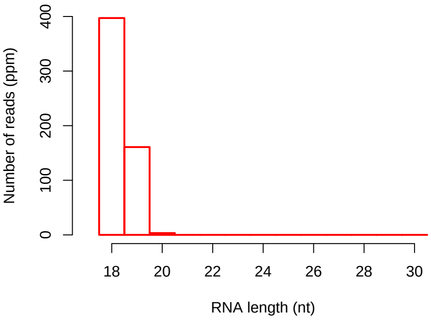

18-mers:

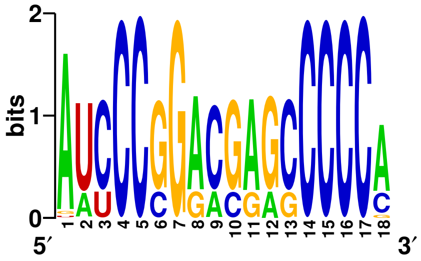

19-mers:

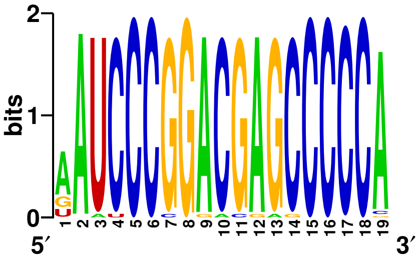

20-mers:

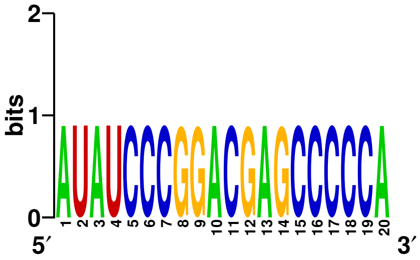

21-mers:

(no read)

22-mers:

(no read)

23-mers:

(no read)

24-mers:

(no read)

25-mers:

(no read)

26-mers:

(no read)

27-mers:

(no read)

28-mers:

(no read)

29-mers:

(no read)

30-mers:

(no read)

Embryo 60h, library 3:

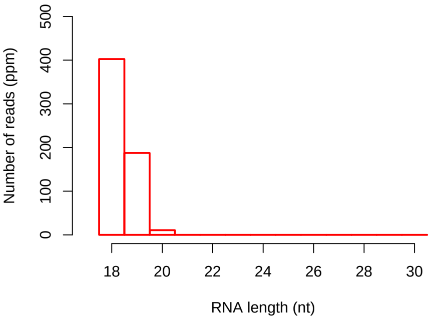

18-mers:

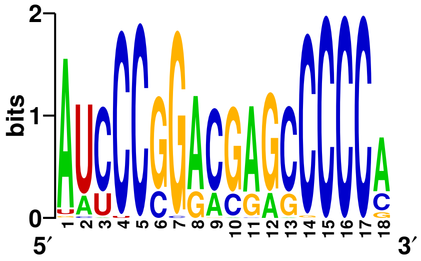

19-mers:

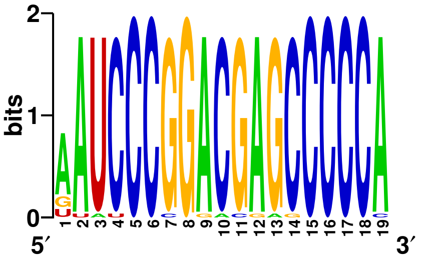

20-mers:

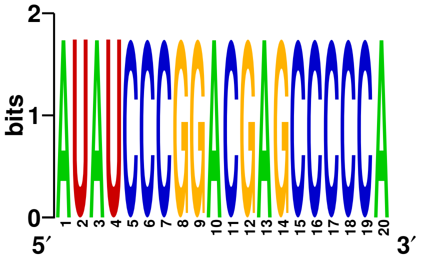

21-mers:

(no read)

22-mers:

(no read)

23-mers:

(no read)

24-mers:

(no read)

25-mers:

(no read)

26-mers:

(no read)

27-mers:

(no read)

28-mers:

(no read)

29-mers:

(no read)

30-mers:

(no read)

Adult female, library 3:

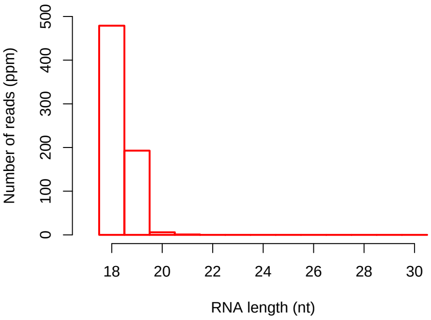

18-mers:

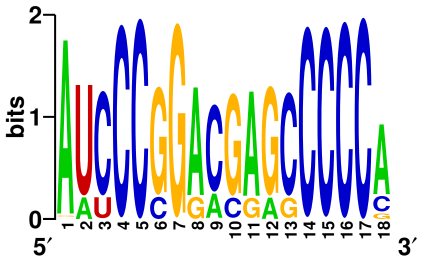

19-mers:

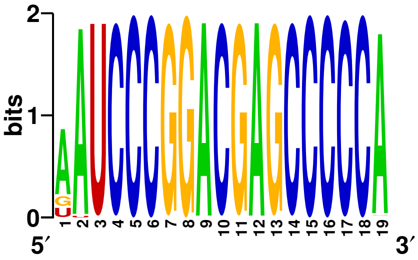

20-mers:

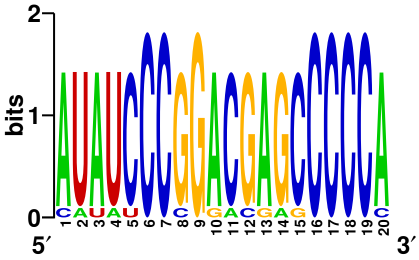

21-mers:

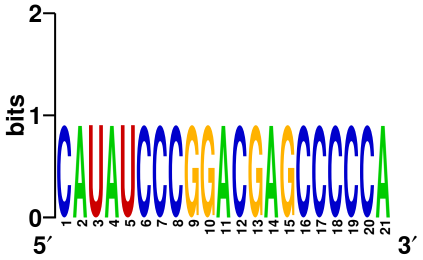

22-mers:
(no read)

23-mers:
(no read)

24-mers:
(no read)

25-mers:
(no read)

26-mers:
(no read)

27-mers:
(no read)

28-mers:
(no read)

29-mers:
(no read)

30-mers:
(no read)

Adult male, library 3:

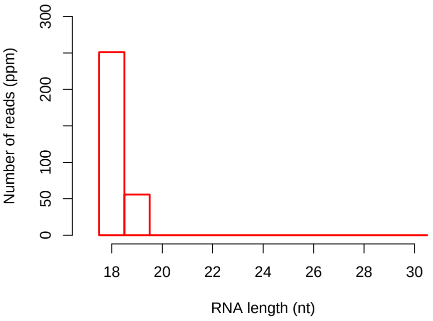

18-mers:

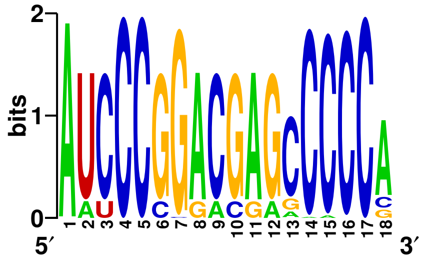

19-mers:

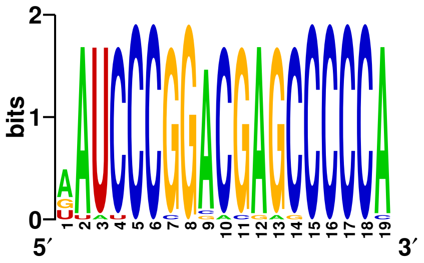

20-mers:

(no read)

21-mers:

(no read)

22-mers:

(no read)

23-mers:

(no read)

24-mers:

(no read)

25-mers:

(no read)

26-mers:

(no read)

27-mers:

(no read)

28-mers:

(no read)

29-mers:

(no read)

30-mers:

(no read)

13.4 Libraries #4 (3´ modified, 5´ hydroxyl or polyphosphorylated small RNAs)

Embryo 8h, library 4:

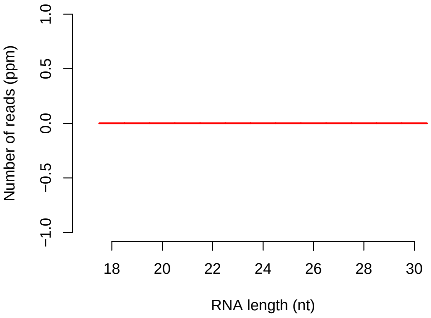

18-mers:
(no read)

19-mers:	20-mers:	21-mers:
(no read)	(no read)	(no read)
22-mers:	23-mers:	24-mers:
(no read)	(no read)	(no read)
25-mers:	26-mers:	27-mers:
(no read)	(no read)	(no read)
28-mers:	29-mers:	30-mers:
(no read)	(no read)	(no read)

Embryo 15h, library 4:

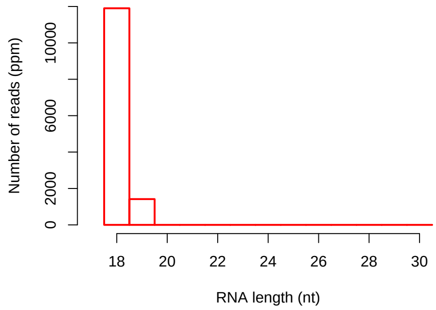

18-mers:

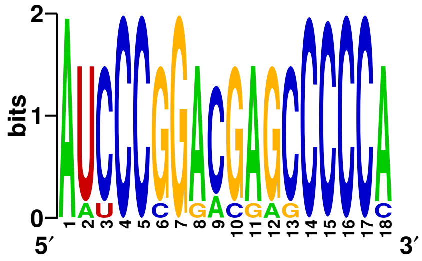

19-mers:

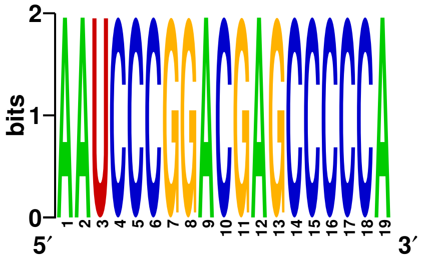

20-mers:

(no read)

21-mers:

(no read)

22-mers:

(no read)

23-mers:

(no read)

24-mers:

(no read)

25-mers:

(no read)

26-mers:

(no read)

27-mers:

(no read)

28-mers:

(no read)

29-mers:

(no read)

30-mers:

(no read)

Embryo 36h, library 4:

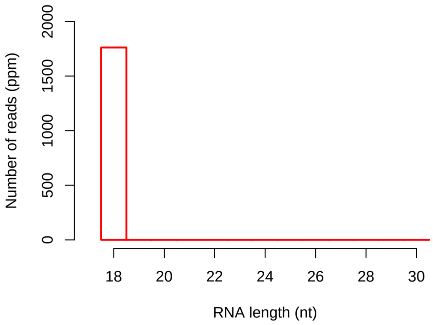

18-mers:

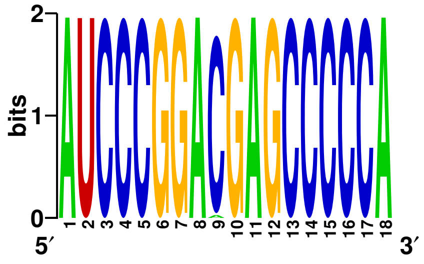

19-mers:	20-mers:	21-mers:
(no read)	(no read)	(no read)
22-mers:	23-mers:	24-mers:
(no read)	(no read)	(no read)
25-mers:	26-mers:	27-mers:
(no read)	(no read)	(no read)
28-mers:	29-mers:	30-mers:
(no read)	(no read)	(no read)

Embryo 60h, library 4:

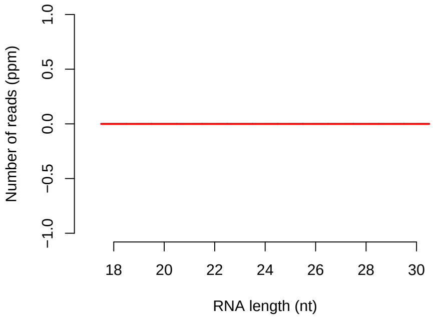

		18-mers:
		(no read)
19-mers:	20-mers:	21-mers:
(no read)	(no read)	(no read)
22-mers:	23-mers:	24-mers:
(no read)	(no read)	(no read)
25-mers:	26-mers:	27-mers:
(no read)	(no read)	(no read)
28-mers:	29-mers:	30-mers:
(no read)	(no read)	(no read)

Adult female, library 4:

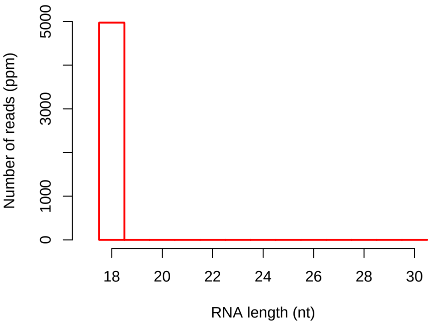

18-mers:

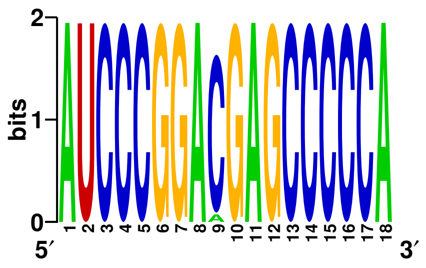

19-mers:	20-mers:	21-mers:
(no read)	(no read)	(no read)
22-mers:	23-mers:	24-mers:
(no read)	(no read)	(no read)
25-mers:	26-mers:	27-mers:
(no read)	(no read)	(no read)
28-mers:	29-mers:	30-mers:
(no read)	(no read)	(no read)

Adult male, library 4:

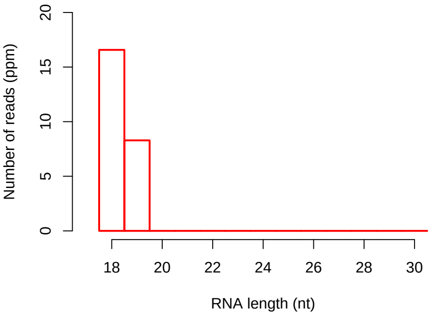

18-mers:

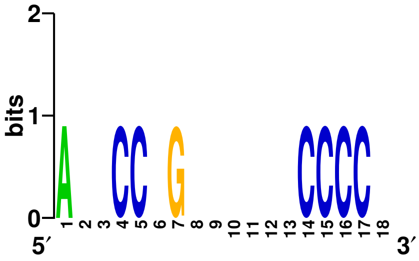

19-mers:

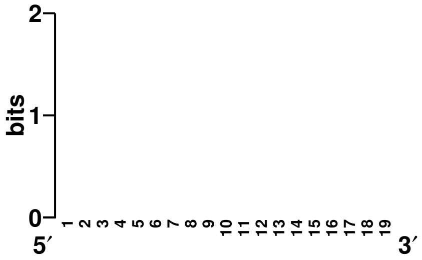

20-mers:

(no read)

21-mers:

(no read)

22-mers:

(no read)

23-mers:

(no read)

24-mers:

(no read)

25-mers:

(no read)

26-mers:

(no read)

27-mers:

(no read)

28-mers:

(no read)

29-mers:

(no read)

30-mers:

(no read)

14 Extragenomic and extratranscriptomic reads matching extragenomic contig #3365

14.1 Libraries #1 (total 5´ monophosphorylated small RNAs)

Embryo 8h, library 1:

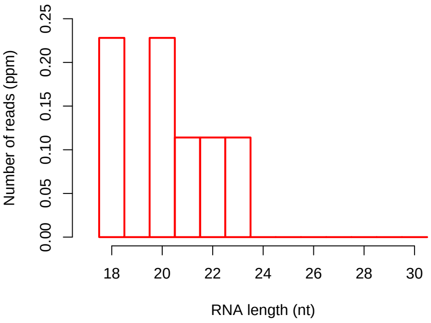

18-mers:

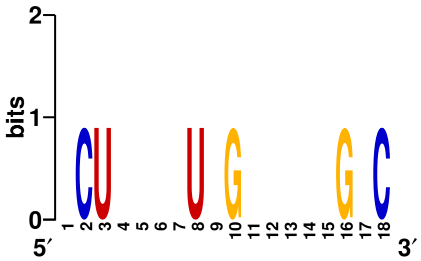

19-mers:

(no read)

20-mers:

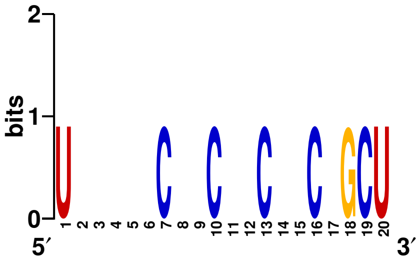

21-mers:

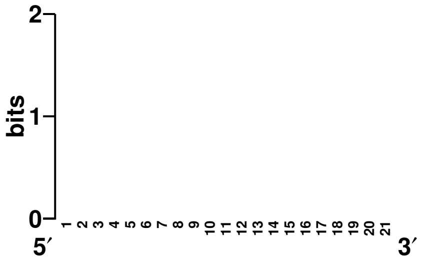

22-mers:

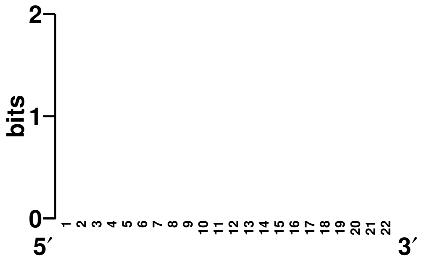

23-mers:

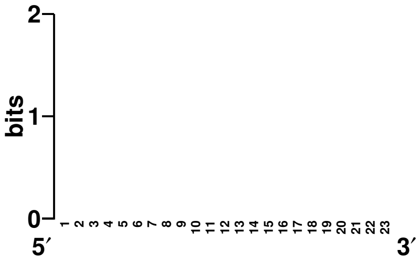

24-mers:

(no read)

25-mers:

(no read)

26-mers:

(no read)

27-mers:

(no read)

28-mers:

(no read)

29-mers:

(no read)

30-mers:

(no read)

Embryo 15h, library 1:

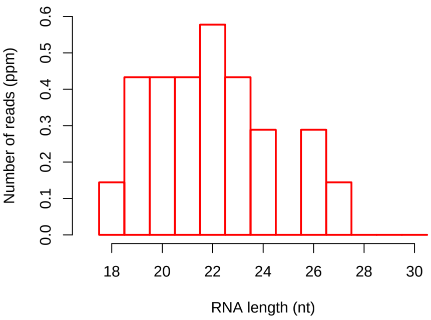

18-mers:

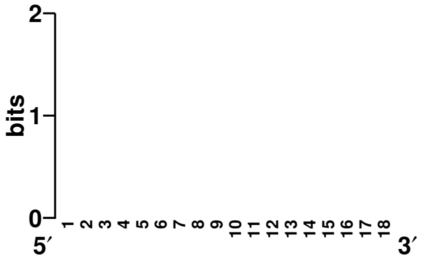

19-mers:

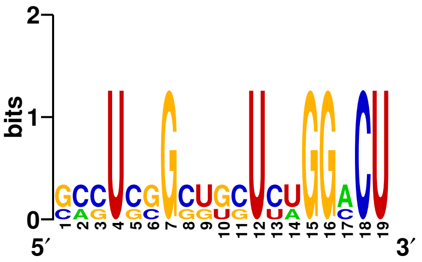

20-mers:

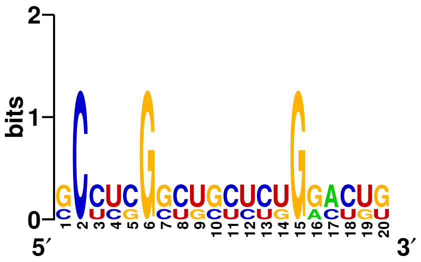

21-mers:

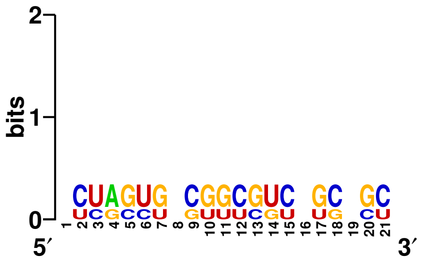

22-mers:

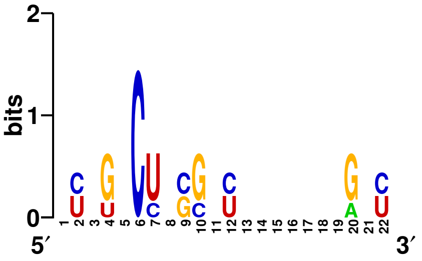

23-mers:

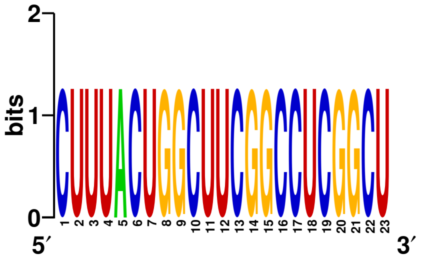

24-mers:

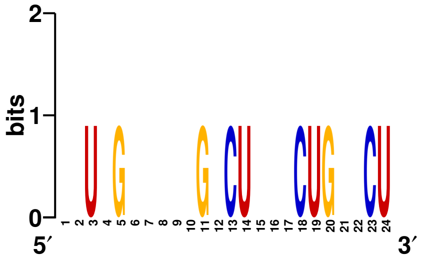

25-mers:

(no read)

26-mers:

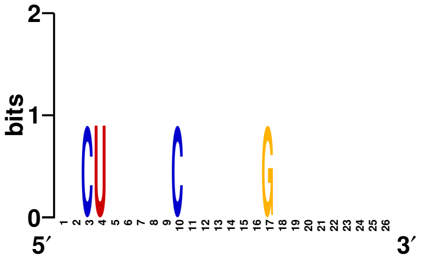

27-mers:

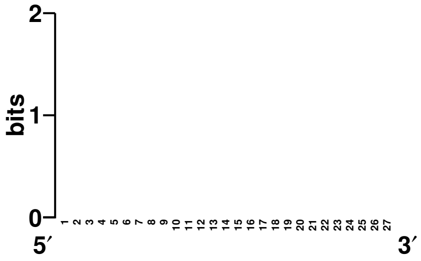

28-mers:

(no read)

29-mers:

(no read)

30-mers:

(no read)

Embryo 36h, library 1:

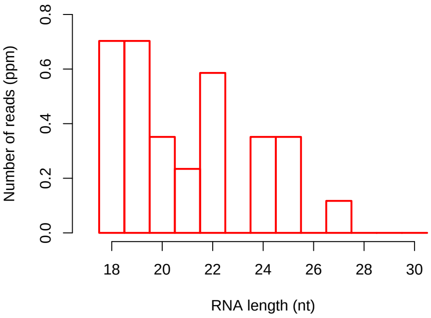

18-mers:

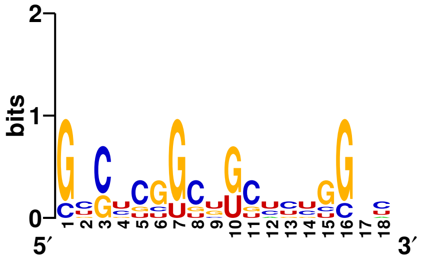

19-mers:

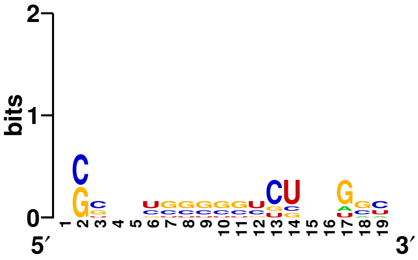

20-mers:

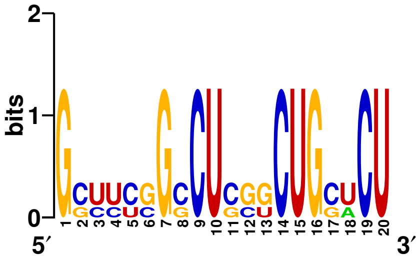

21-mers:

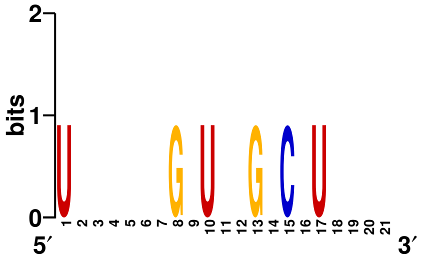

22-mers:

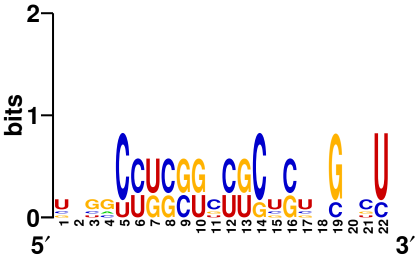

23-mers:

(no read)

24-mers:

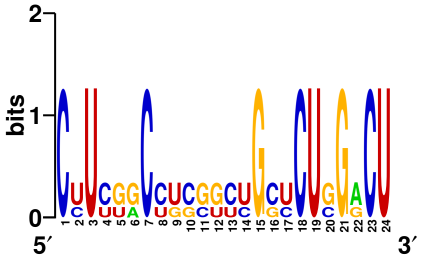

25-mers:

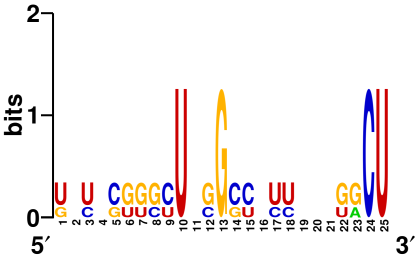

26-mers:

(no read)

27-mers:

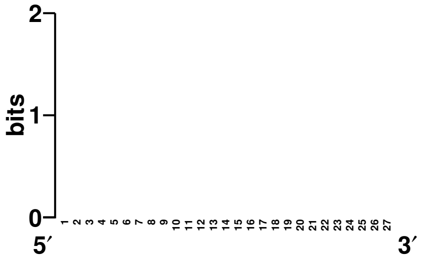

28-mers:

(no read)

29-mers:

(no read)

30-mers:

(no read)

Embryo 60h, library 1:

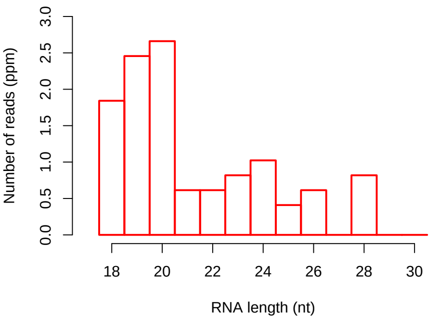

18-mers:

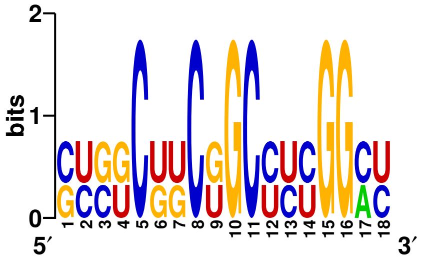

19-mers:

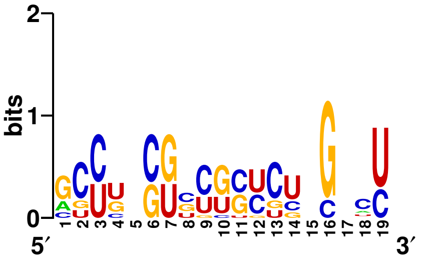

20-mers:

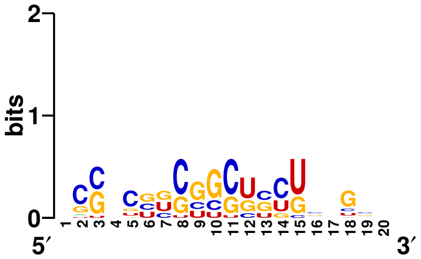

21-mers:

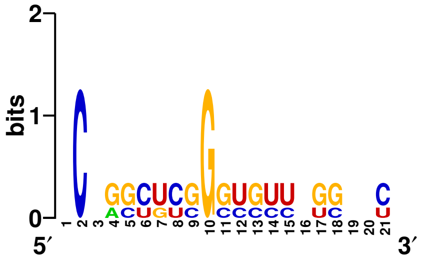

22-mers:

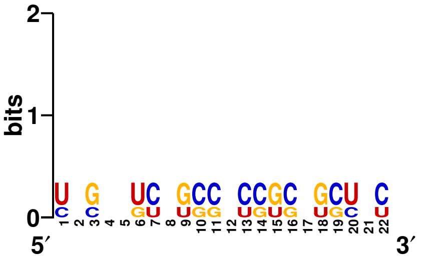

23-mers:

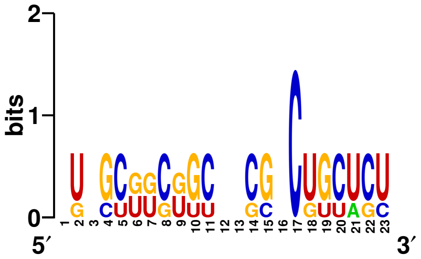

24-mers:

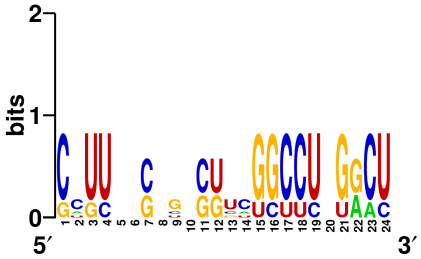

25-mers:

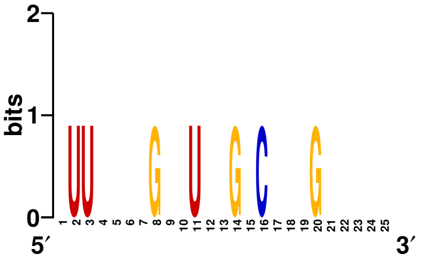

26-mers:

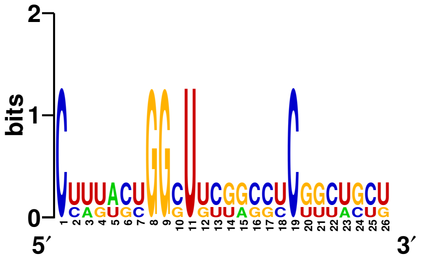

27-mers:

(no read)

28-mers:

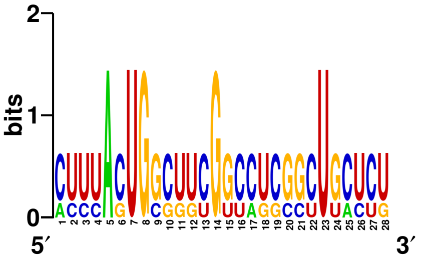

29-mers:

(no read)

30-mers:

(no read)

Adult female, library 1:

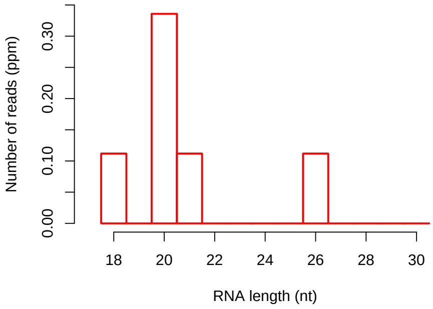

18-mers:

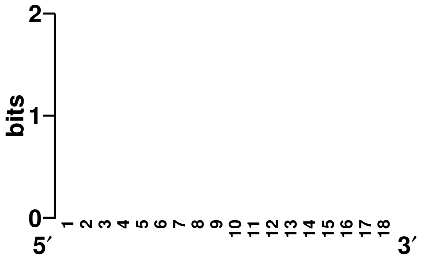

19-mers:

(no read)

20-mers:

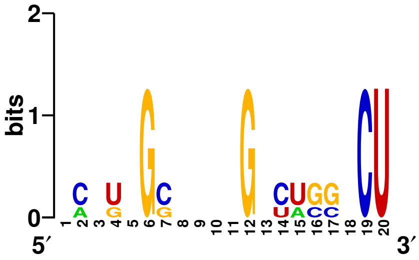

21-mers:

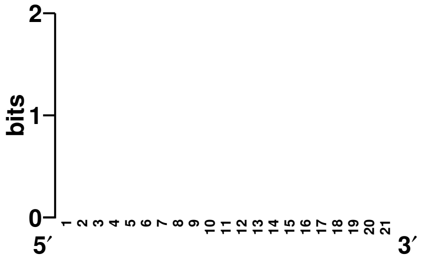

22-mers:

(no read)

23-mers:

(no read)

24-mers:

(no read)

25-mers:

(no read)

26-mers:

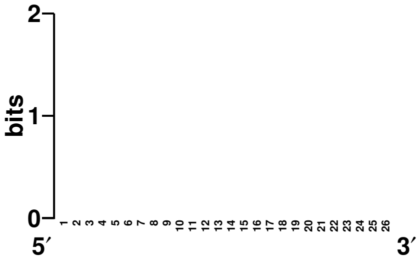

27-mers:

(no read)

28-mers:

(no read)

29-mers:

(no read)

30-mers:

(no read)

Adult male, library 1:

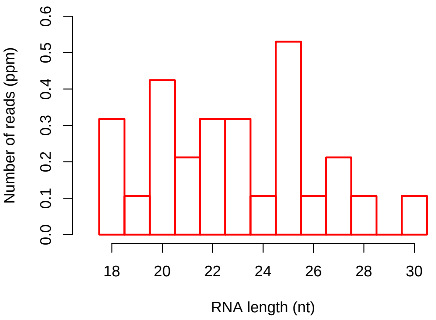

18-mers:

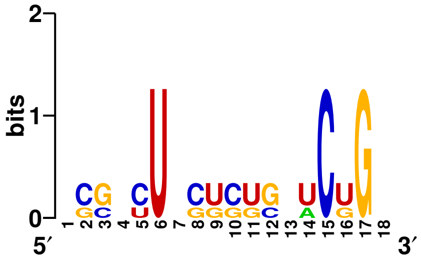

19-mers:

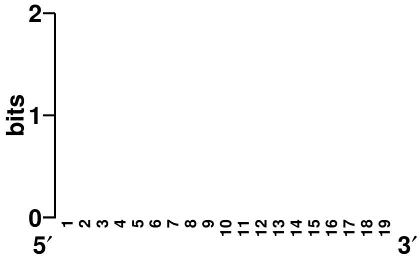

20-mers:

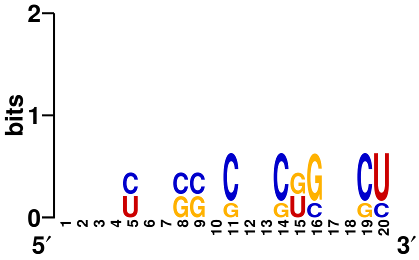

21-mers:

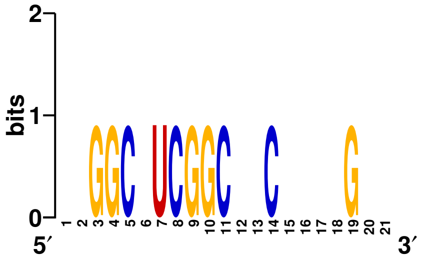

22-mers:

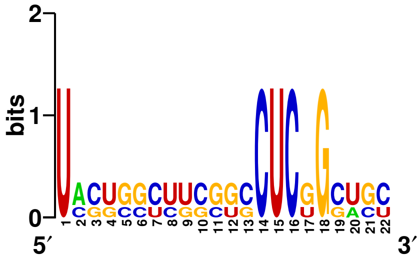

23-mers:

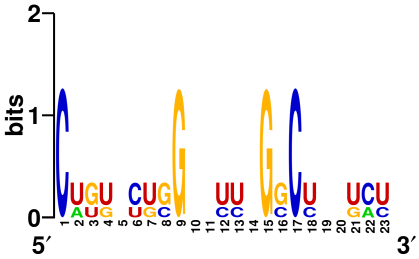

24-mers:

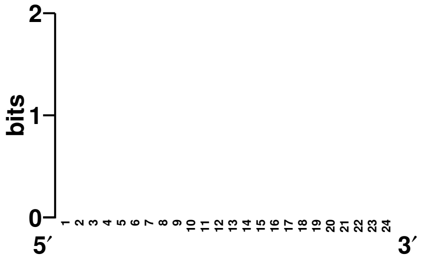

25-mers:

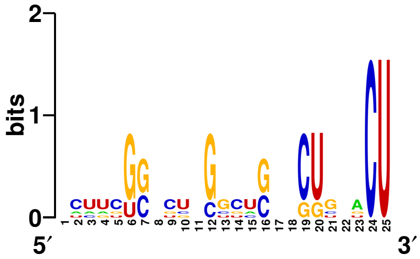

26-mers:

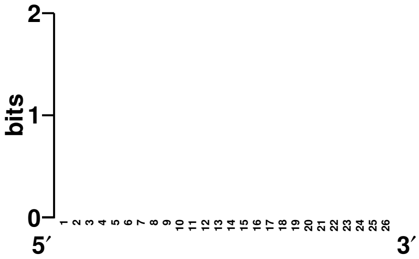

27-mers:

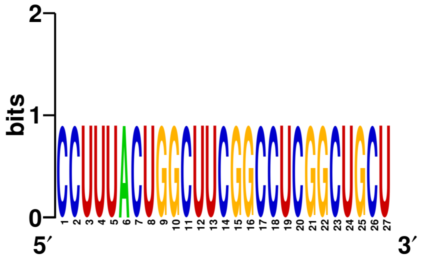

28-mers:

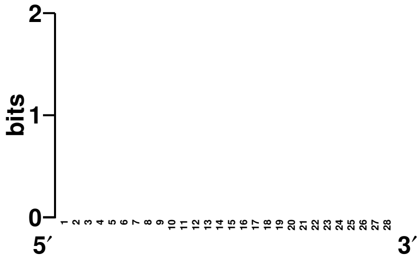

29-mers:

(no read)

30-mers:

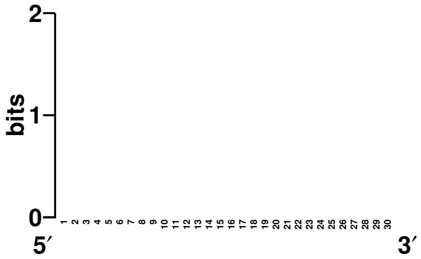

14.2 Libraries #2 (3´ modified, 5´ monophosphorylated small RNAs)

Embryo 8h, library 2:

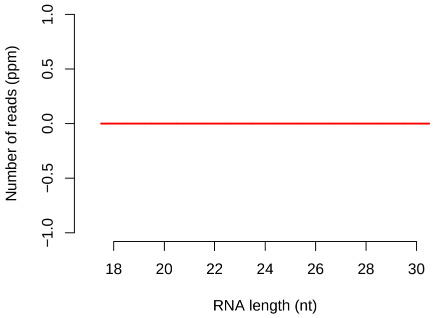

18-mers:

(no read)

19-mers:	20-mers:	21-mers:
(no read)	(no read)	(no read)
22-mers:	23-mers:	24-mers:
(no read)	(no read)	(no read)
25-mers:	26-mers:	27-mers:
(no read)	(no read)	(no read)
28-mers:	29-mers:	30-mers:
(no read)	(no read)	(no read)

Embryo 15h, library 2:

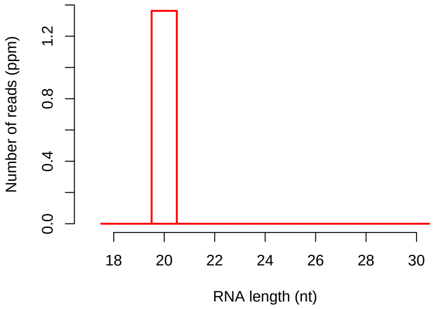

18-mers:

(no read)

19-mers:

(no read)

20-mers:

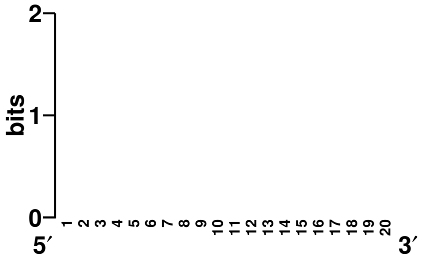

21-mers:

(no read)

22-mers:

(no read)

23-mers:

(no read)

24-mers:

(no read)

25-mers:

(no read)

26-mers:

(no read)

27-mers:

(no read)

28-mers:

(no read)

29-mers:

(no read)

30-mers:

(no read)

Embryo 36h, library 2:

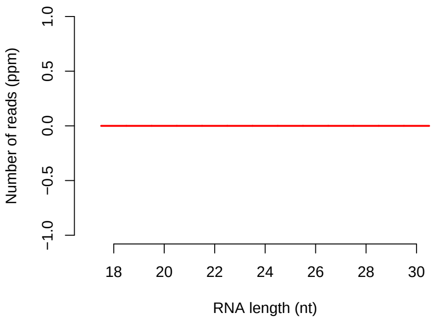

18-mers:
(no read)

19-mers:
(no read)

20-mers:
(no read)

21-mers:
(no read)

22-mers:
(no read)

23-mers:
(no read)

24-mers:
(no read)

25-mers:
(no read)

26-mers:
(no read)

27-mers:
(no read)

28-mers:
(no read)

29-mers:
(no read)

30-mers:
(no read)

Embryo 60h, library 2:

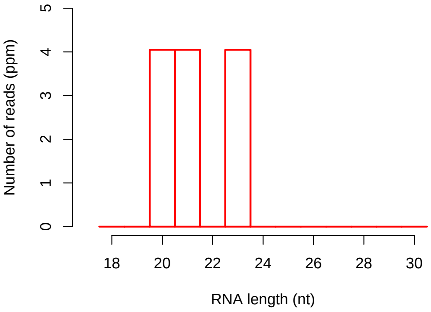

18-mers:

(no read)

19-mers:

(no read)

20-mers:

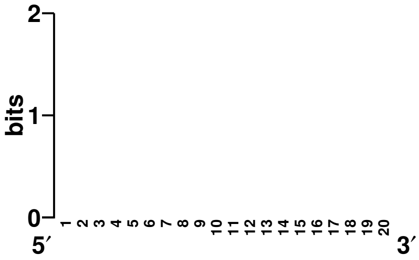

21-mers:

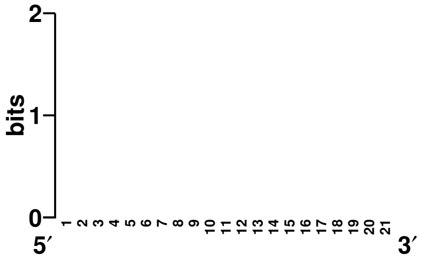

22-mers:

(no read)

23-mers:

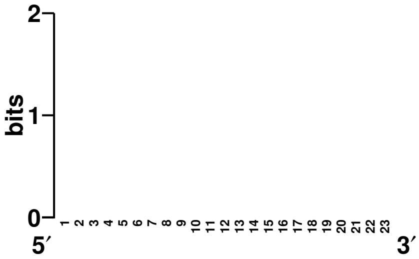

24-mers:

(no read)

25-mers:

(no read)

26-mers:

(no read)

27-mers:

(no read)

28-mers:

(no read)

29-mers:

(no read)

30-mers:

(no read)

Adult female, library 2:

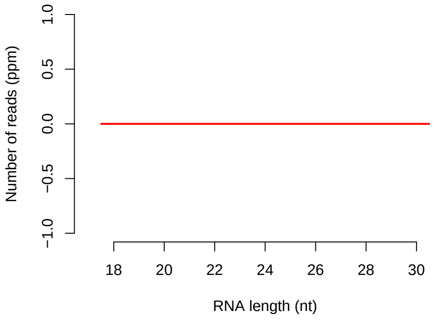

18-mers:
(no read)

19-mers:	20-mers:	21-mers:
(no read)	(no read)	(no read)
22-mers:	23-mers:	24-mers:
(no read)	(no read)	(no read)
25-mers:	26-mers:	27-mers:
(no read)	(no read)	(no read)
28-mers:	29-mers:	30-mers:
(no read)	(no read)	(no read)

Adult male, library 2:

18-mers:

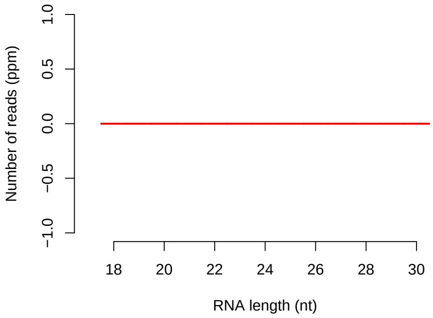

(no read)

19-mers:	20-mers:	21-mers:
(no read)	(no read)	(no read)
22-mers:	23-mers:	24-mers:
(no read)	(no read)	(no read)
25-mers:	26-mers:	27-mers:
(no read)	(no read)	(no read)
28-mers:	29-mers:	30-mers:
(no read)	(no read)	(no read)

14.3 Libraries #3 (total 5´ hydroxyl or polyphosphorylated small RNAs)

Embryo 8h, library 3:

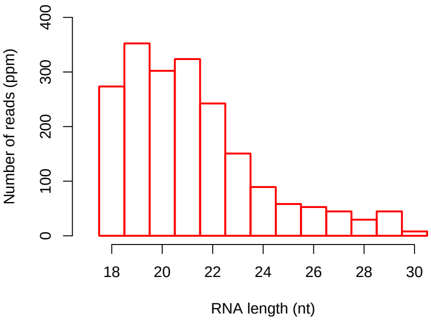

18-mers:

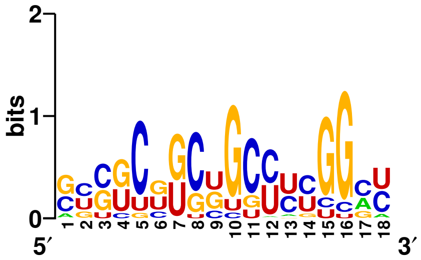

19-mers:

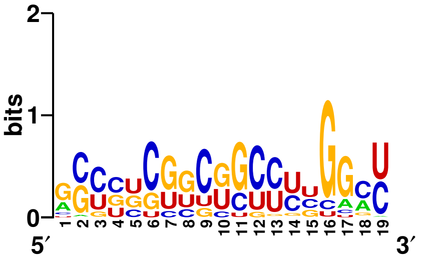

20-mers:

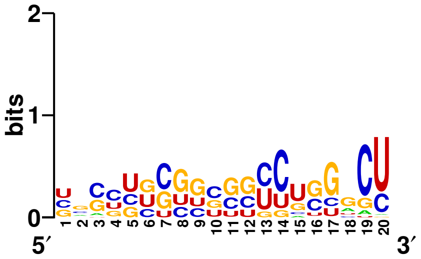

21-mers:

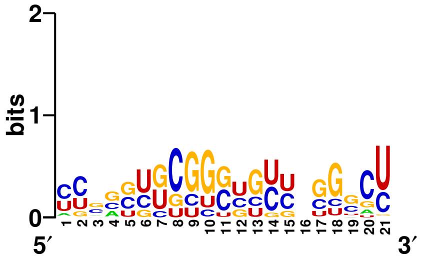

22-mers:

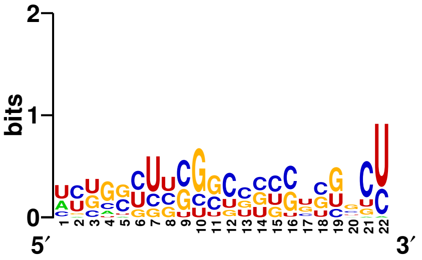

23-mers:

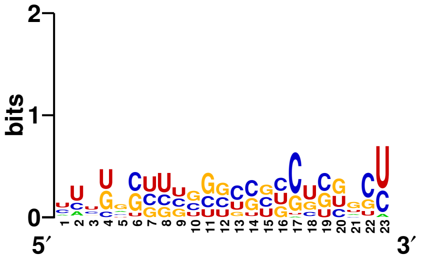

24-mers:

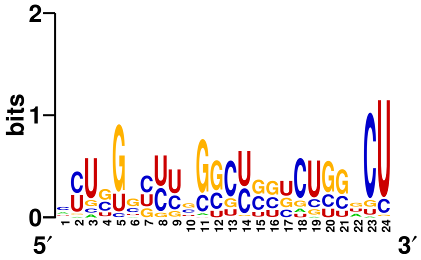

25-mers:

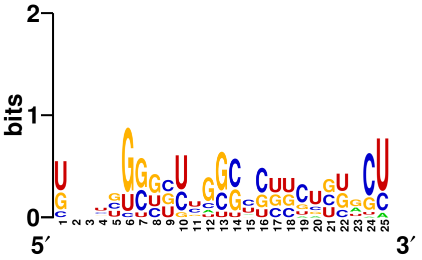

26-mers:

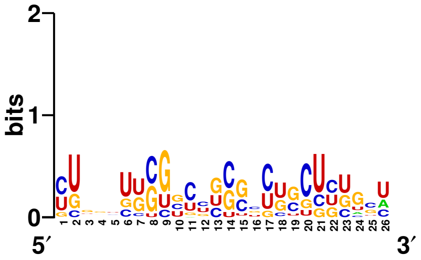

27-mers:

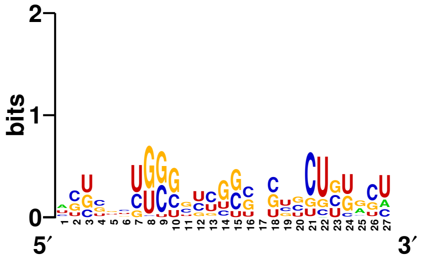

28-mers:

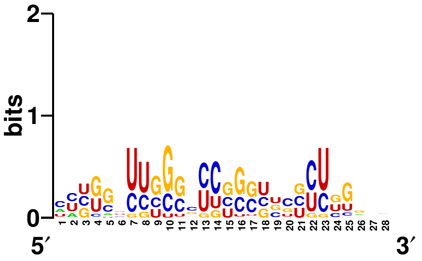

29-mers:

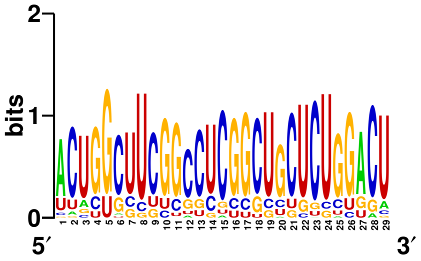

30-mers:

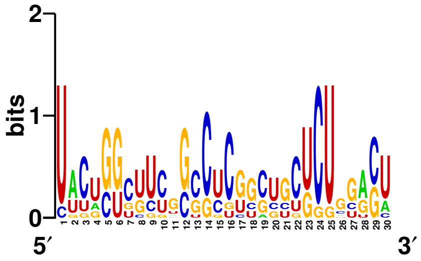

Embryo 15h, library 3:

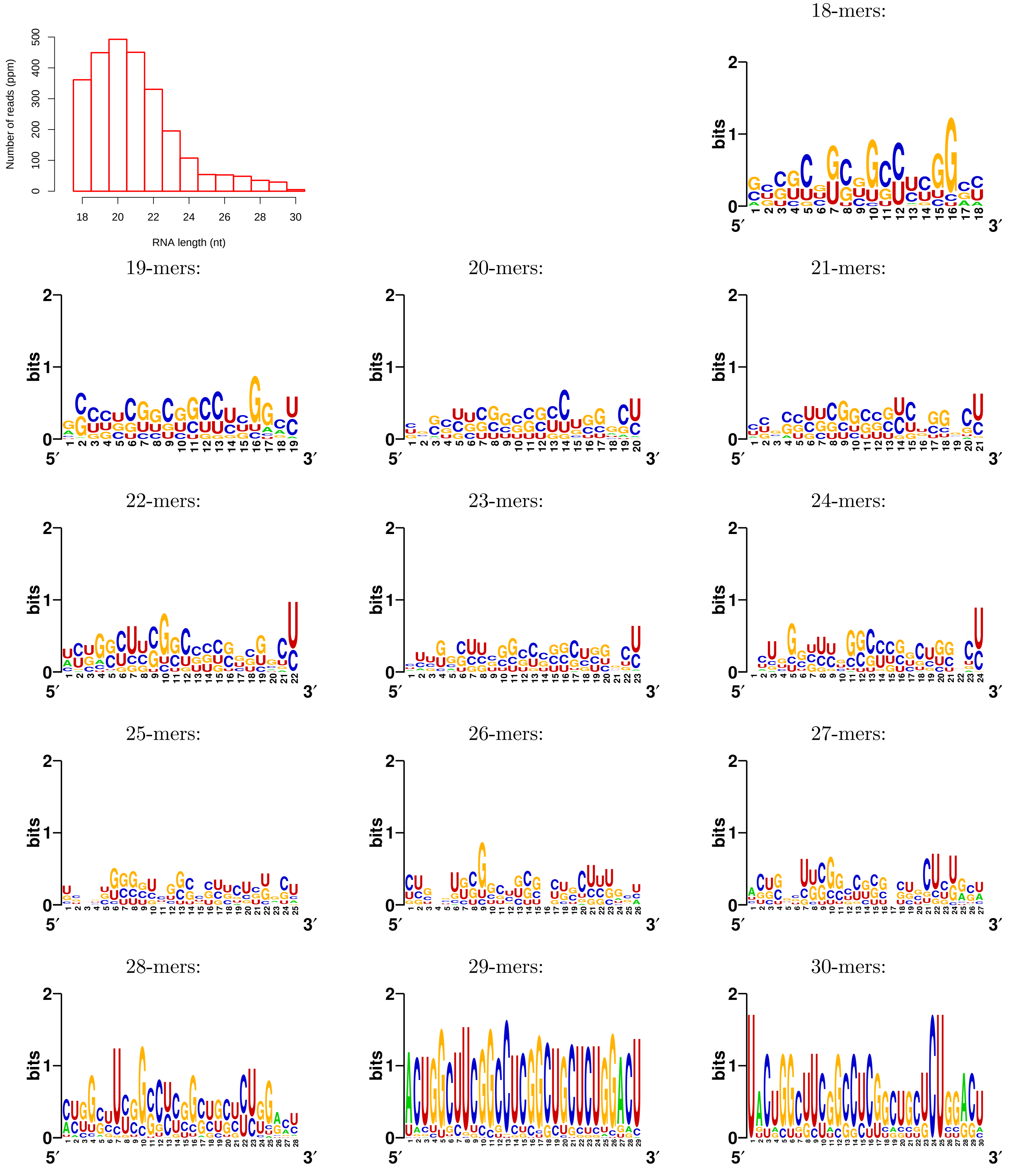

Embryo 36h, library 3:

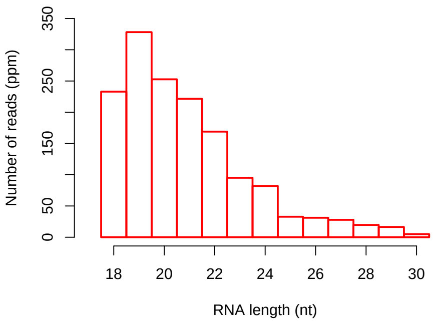

18-mers:

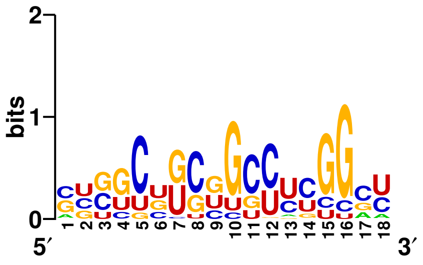

19-mers:

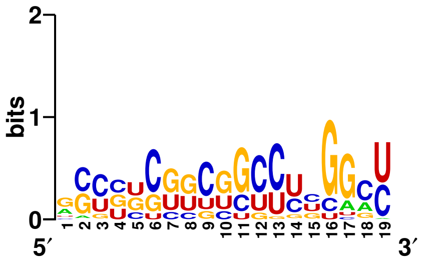

20-mers:

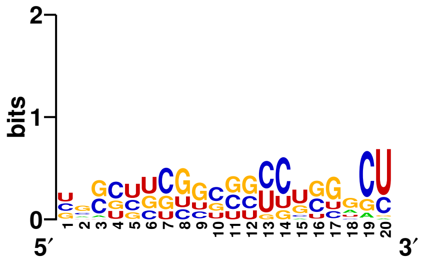

21-mers:

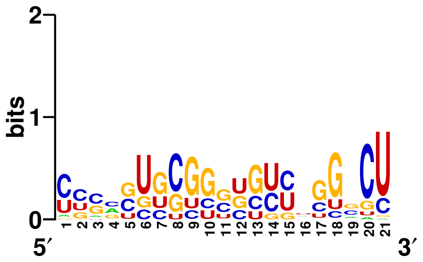

22-mers:

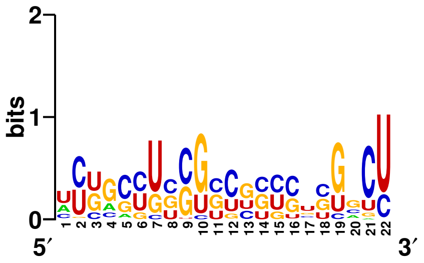

23-mers:

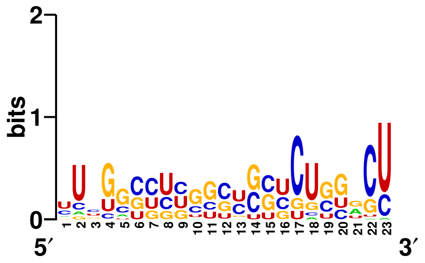

24-mers:

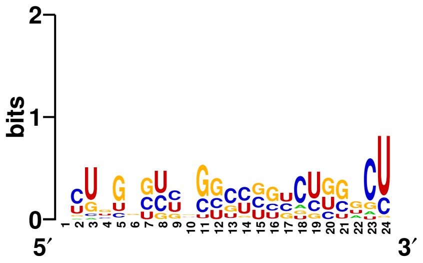

25-mers:

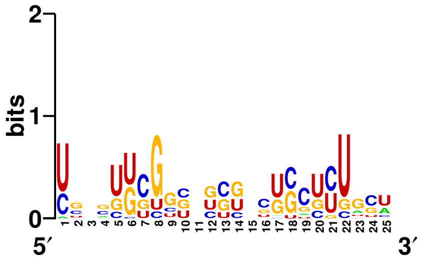

26-mers:

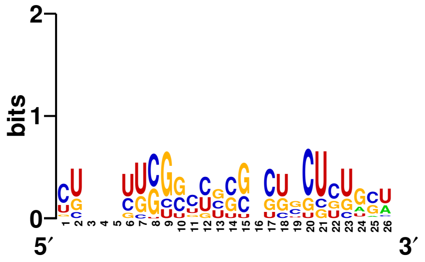

27-mers:

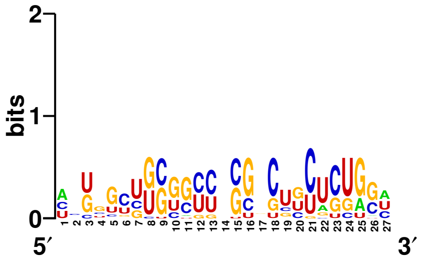

28-mers:

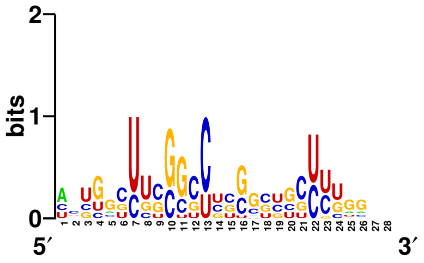

29-mers:

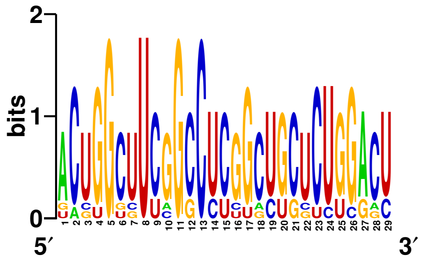

30-mers:

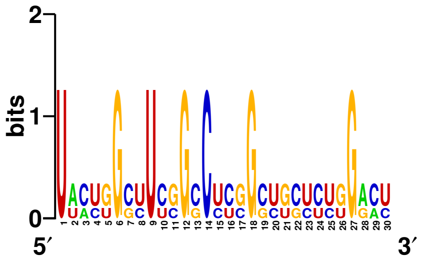

Embryo 60h, library 3:

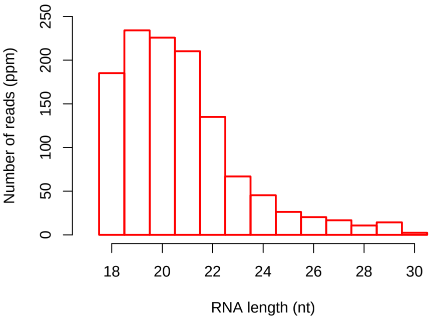

18-mers:

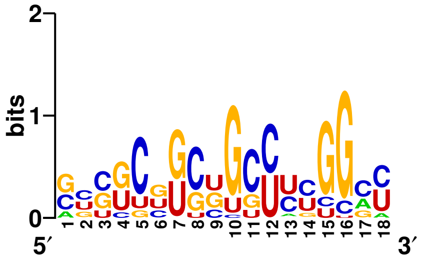

19-mers:

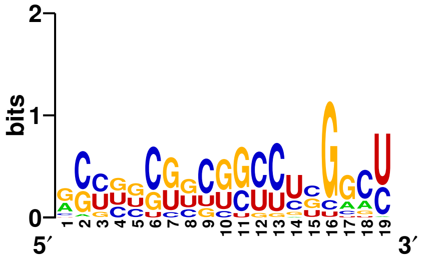

20-mers:

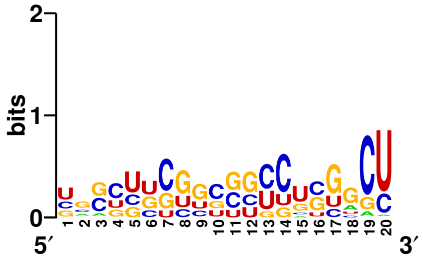

21-mers:

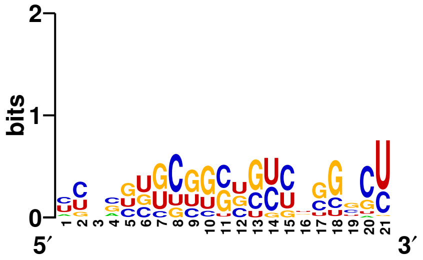

22-mers:

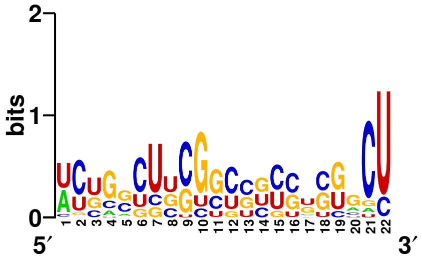

23-mers:

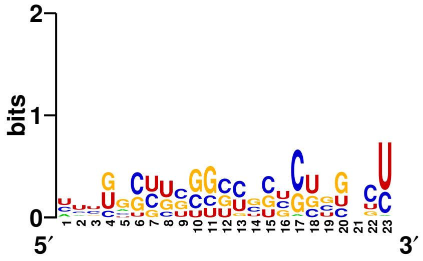

24-mers:

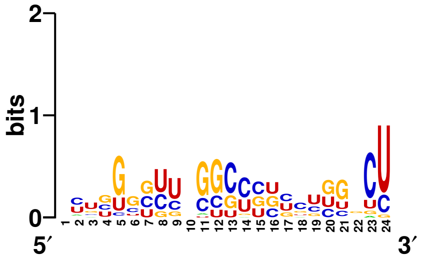

25-mers:

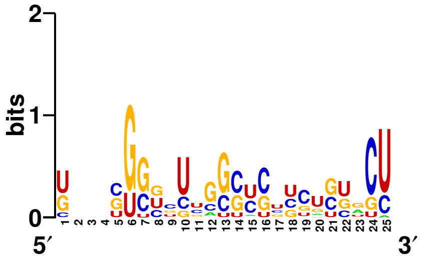

26-mers:

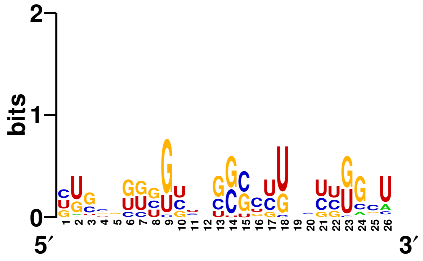

27-mers:

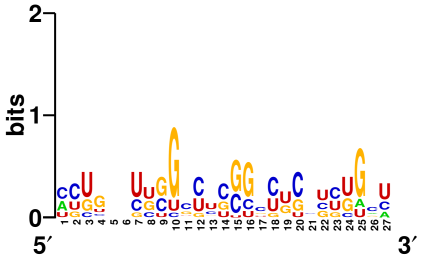

28-mers:

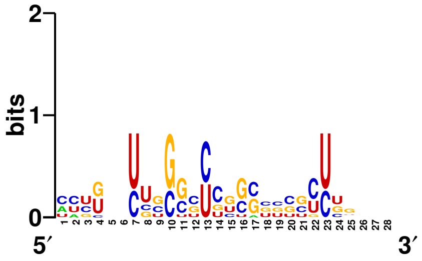

29-mers:

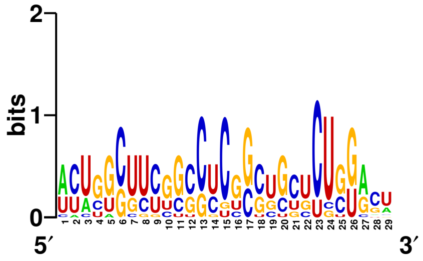

30-mers:

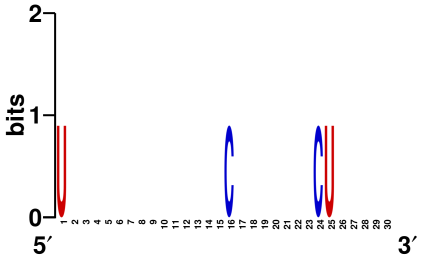

Adult female, library 3:

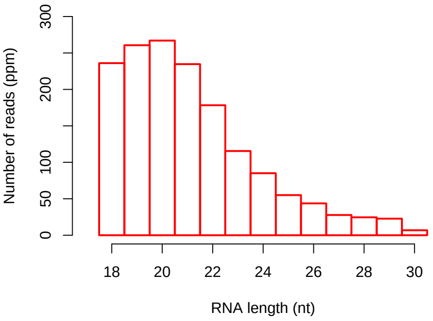

18-mers:

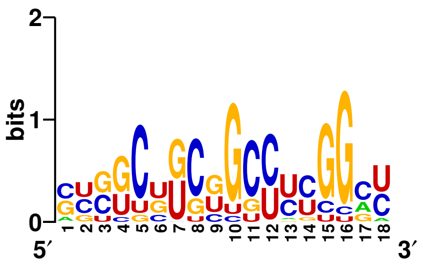

19-mers:

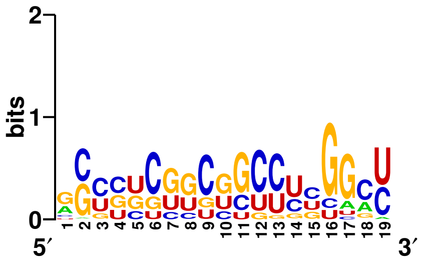

20-mers:

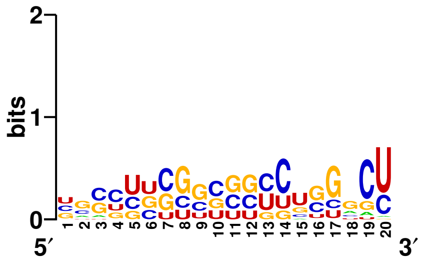

21-mers:

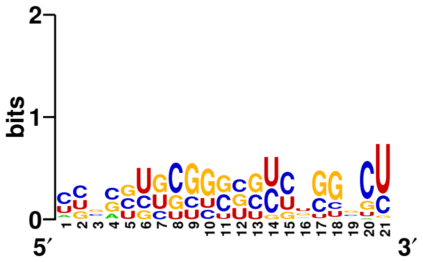

22-mers:

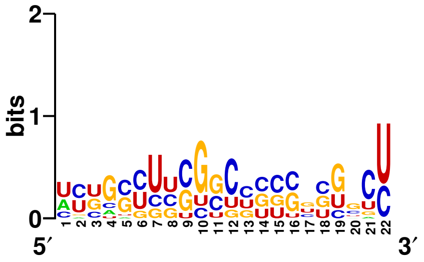

23-mers:

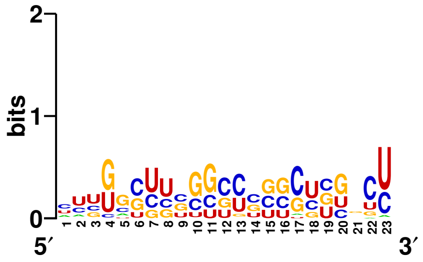

24-mers:

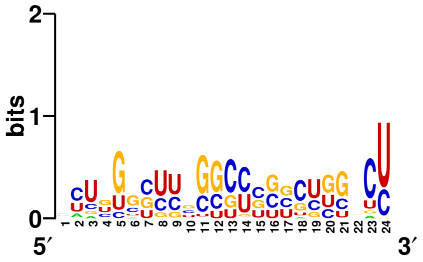

25-mers:

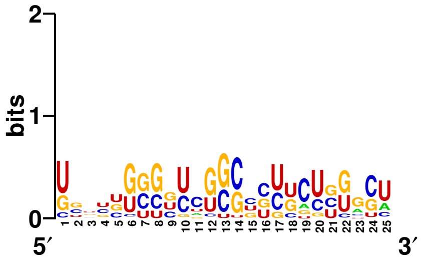

26-mers:

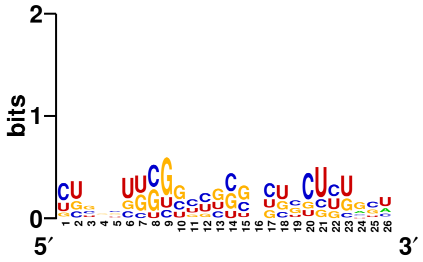

27-mers:

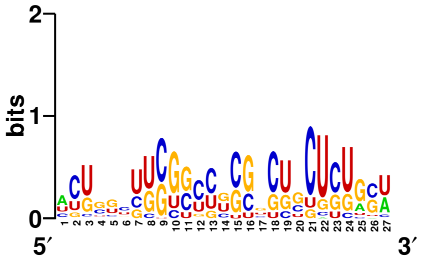

28-mers:

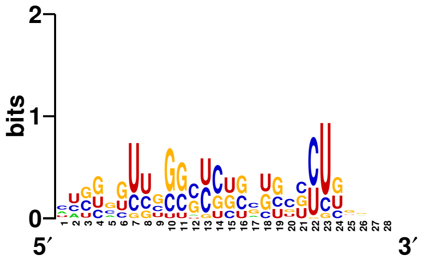

29-mers:

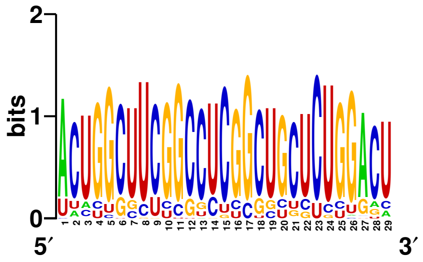

30-mers:

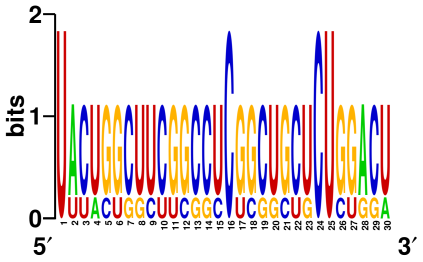

Adult male, library 3:

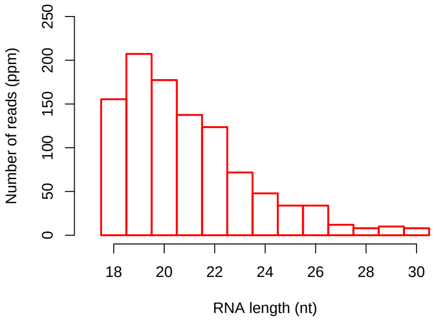

18-mers:

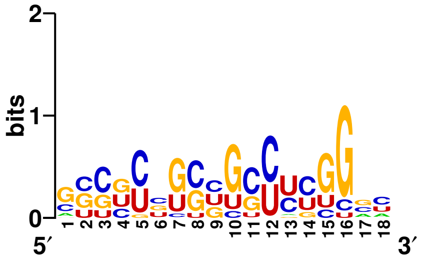

19-mers:

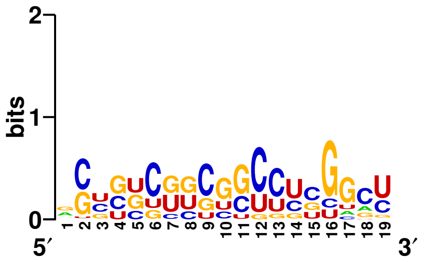

20-mers:

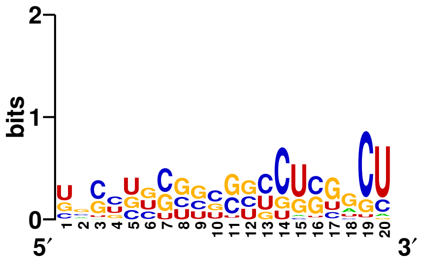

21-mers:

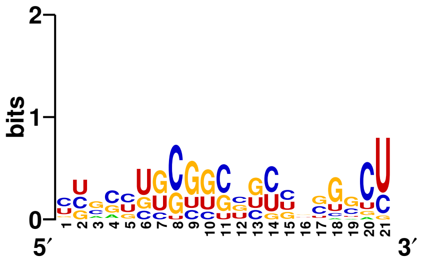

22-mers:

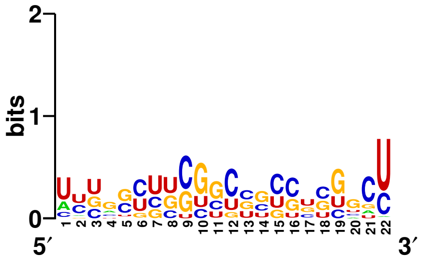

23-mers:

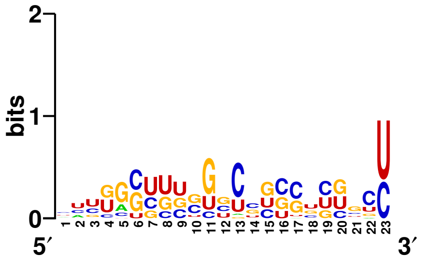

24-mers:

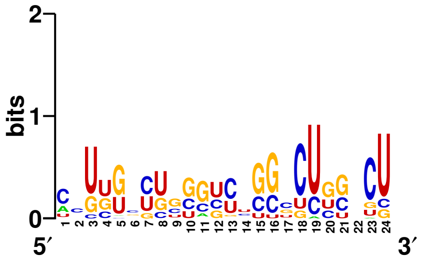

25-mers:

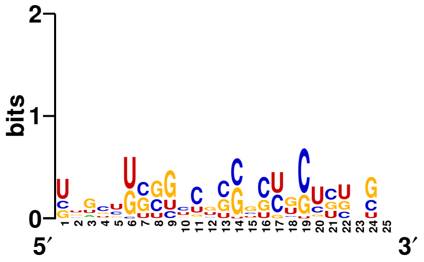

26-mers:

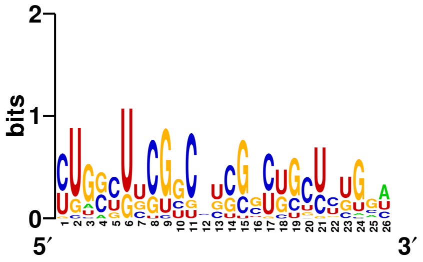

27-mers:

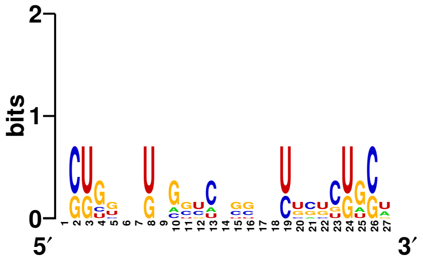

28-mers:

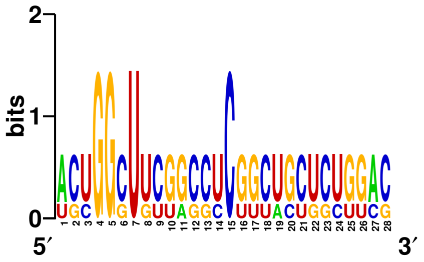

29-mers:

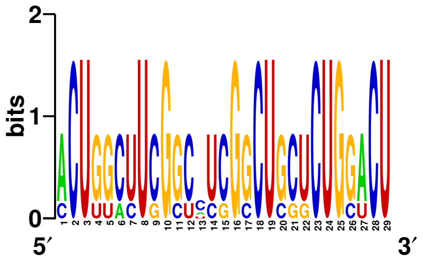

30-mers:

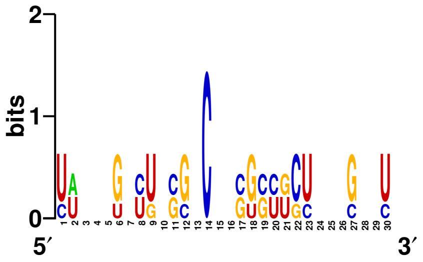

14.4 Libraries #4 (3´ modified, 5´ hydroxyl or polyphosphorylated small RNAs)

Embryo 8h, library 4:

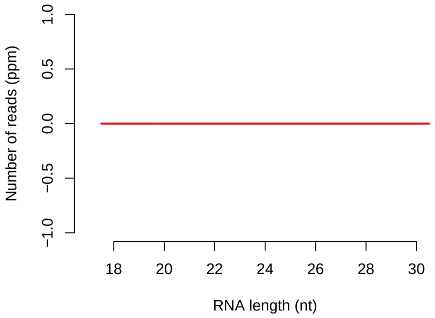

18-mers:
(no read)

19-mers:
(no read)

20-mers:
(no read)

21-mers:
(no read)

22-mers:
(no read)

23-mers:
(no read)

24-mers:
(no read)

25-mers:
(no read)

26-mers:
(no read)

27-mers:
(no read)

28-mers:
(no read)

29-mers:
(no read)

30-mers:
(no read)

Embryo 15h, library 4:

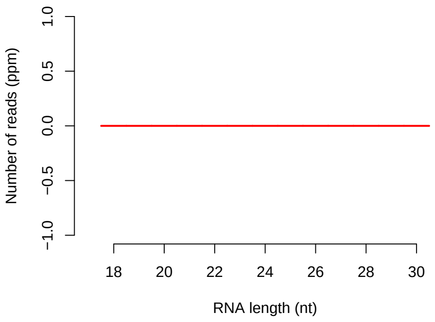

18-mers:

(no read)

19-mers:	20-mers:	21-mers:
(no read)	(no read)	(no read)
22-mers:	23-mers:	24-mers:
(no read)	(no read)	(no read)
25-mers:	26-mers:	27-mers:
(no read)	(no read)	(no read)
28-mers:	29-mers:	30-mers:
(no read)	(no read)	(no read)

Embryo 36h, library 4:

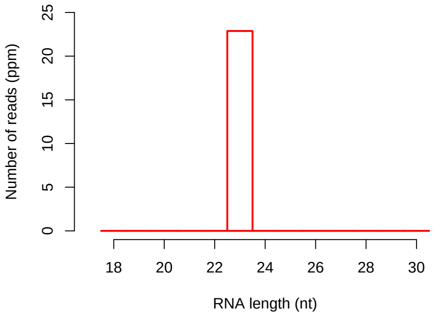

18-mers:
(no read)

19-mers:
(no read)

20-mers:
(no read)

21-mers:
(no read)

22-mers:
(no read)

23-mers:

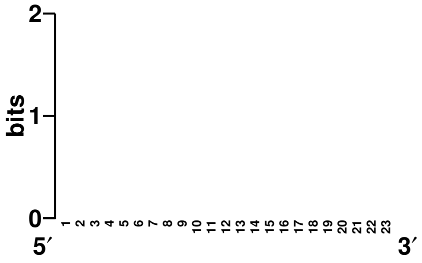

24-mers:
(no read)

25-mers:
(no read)

26-mers:
(no read)

27-mers:
(no read)

28-mers:
(no read)

29-mers:
(no read)

30-mers:
(no read)

Embryo 60h, library 4:

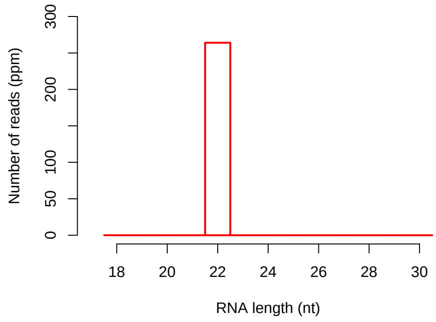

18-mers:

(no read)

19-mers:

(no read)

20-mers:

(no read)

21-mers:

(no read)

22-mers:

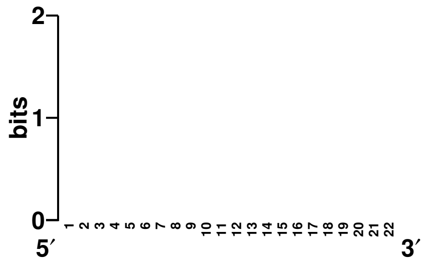

23-mers:

(no read)

24-mers:

(no read)

25-mers:

(no read)

26-mers:

(no read)

27-mers:

(no read)

28-mers:

(no read)

29-mers:

(no read)

30-mers:

(no read)

Adult female, library 4:

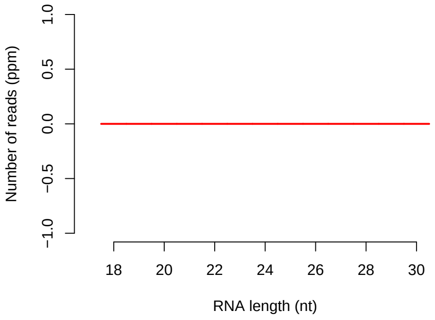

18-mers:
(no read)

19-mers:
(no read)

20-mers:
(no read)

21-mers:
(no read)

22-mers:
(no read)

23-mers:
(no read)

24-mers:
(no read)

25-mers:
(no read)

26-mers:
(no read)

27-mers:
(no read)

28-mers:
(no read)

29-mers:
(no read)

30-mers:
(no read)

Adult male, library 4:

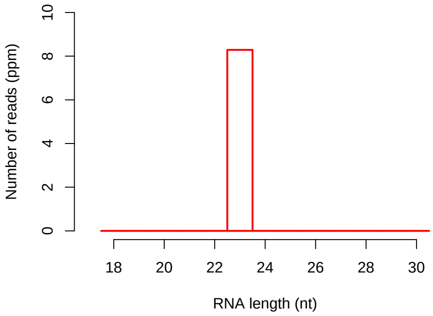

18-mers:

(no read)

19-mers:

(no read)

20-mers:

(no read)

21-mers:

(no read)

22-mers:

(no read)

23-mers:

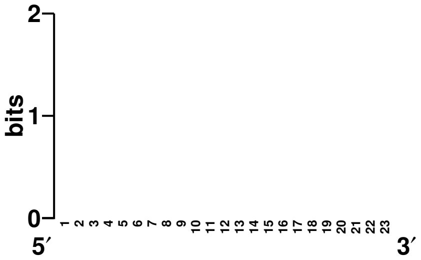

24-mers:

(no read)

25-mers:

(no read)

26-mers:

(no read)

27-mers:

(no read)

28-mers:

(no read)

29-mers:

(no read)

30-mers:

(no read)

15 Extragenomic and extratranscriptomic reads matching extragenomic contig #10883

15.1 Libraries #1 (total 5´ monophosphorylated small RNAs)

Embryo 8h, library 1:

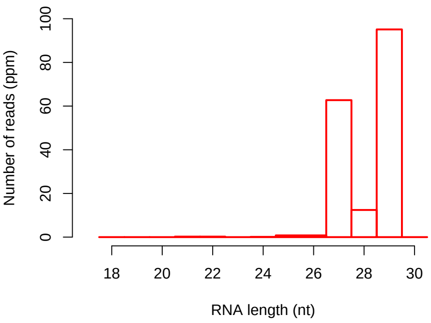

18-mers:

(no read)

19-mers:

(no read)

20-mers:

(no read)

21-mers:

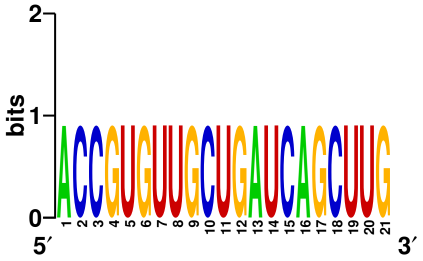

22-mers:

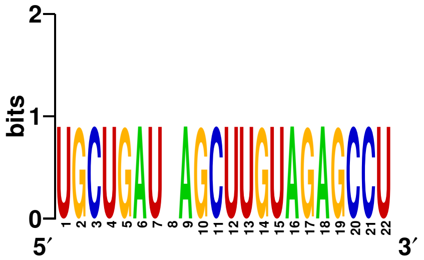

23-mers:

(no read)

24-mers:

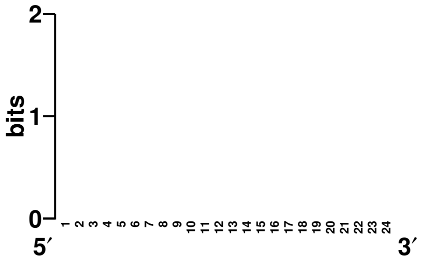

25-mers:

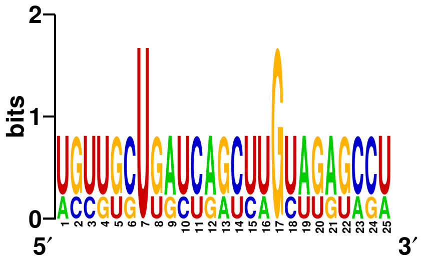

26-mers:

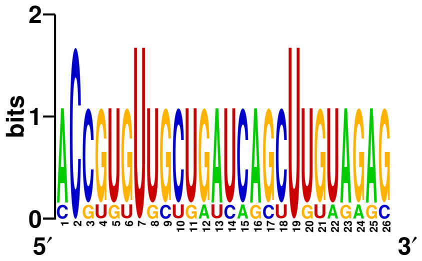

27-mers:

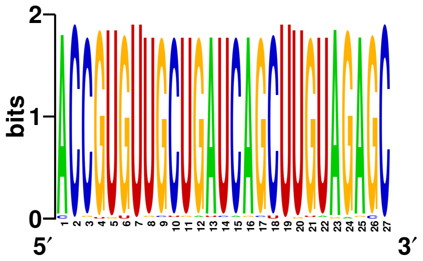

28-mers:

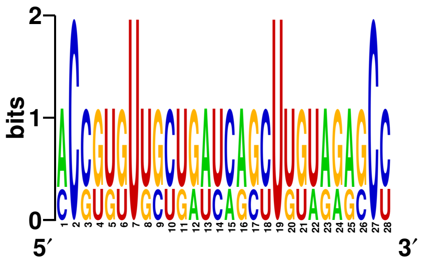

29-mers:

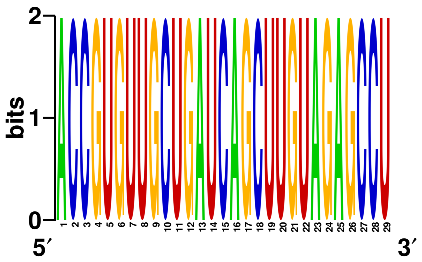

30-mers:

(no read)

Embryo 15h, library 1:

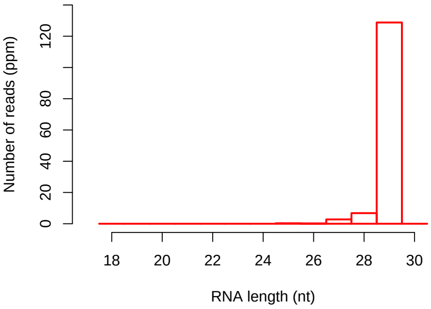

18-mers:

(no read)

19-mers:

(no read)

20-mers:

(no read)

21-mers:

(no read)

22-mers:

(no read)

23-mers:

(no read)

24-mers:

(no read)

25-mers:

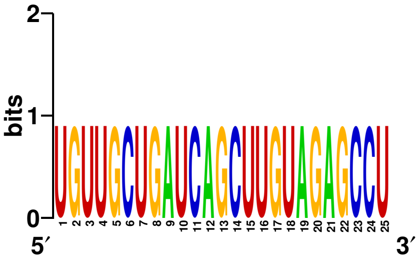

26-mers:

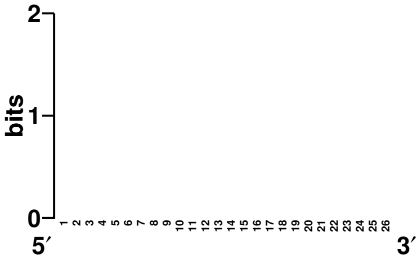

27-mers:

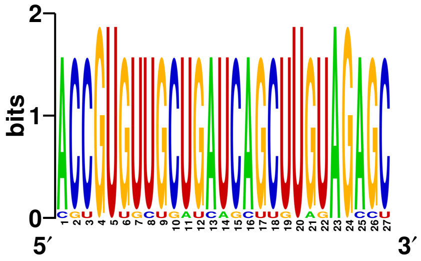

28-mers:

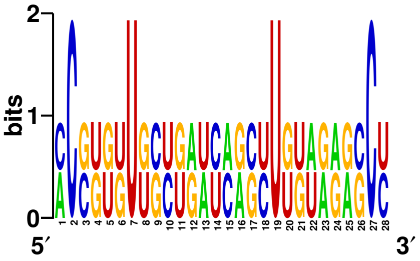

29-mers:

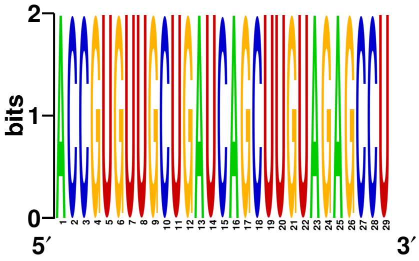

30-mers:

(no read)

Embryo 36h, library 1:

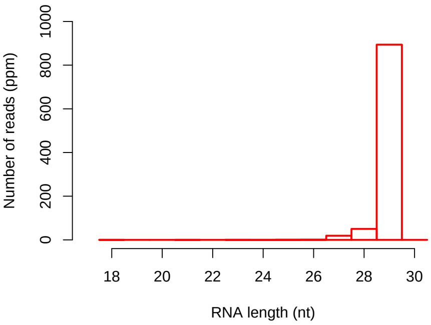

18-mers:

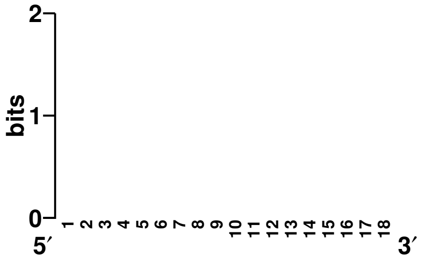

19-mers:

(no read)

20-mers:

(no read)

21-mers:

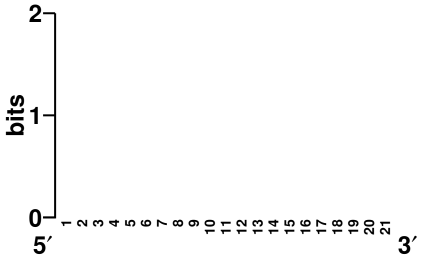

22-mers:

(no read)

23-mers:

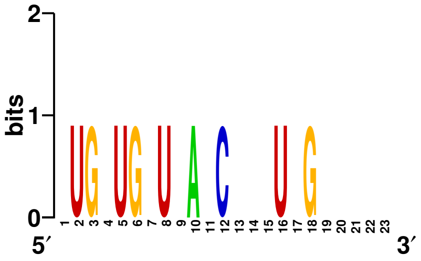

24-mers:

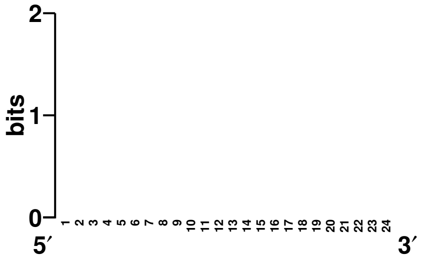

25-mers:

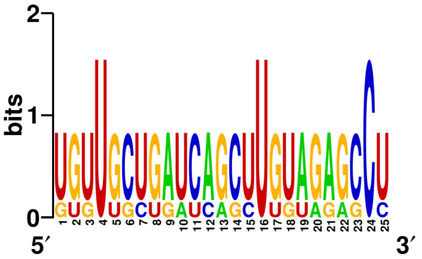

26-mers:

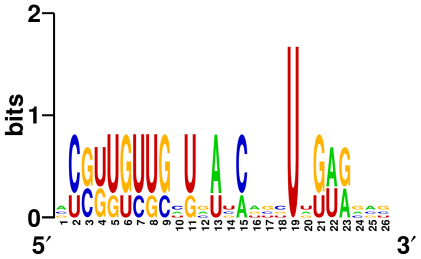

27-mers:

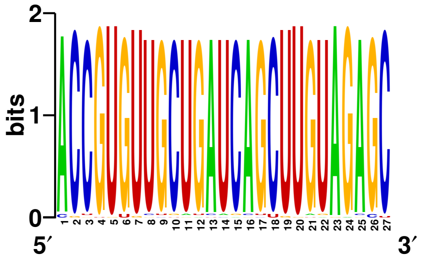

28-mers:

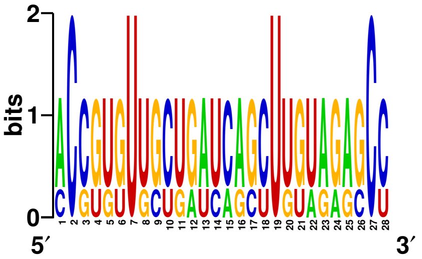

29-mers:

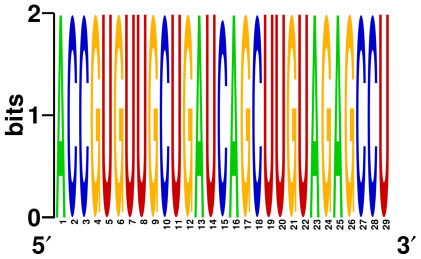

30-mers:

(no read)

Embryo 60h, library 1:

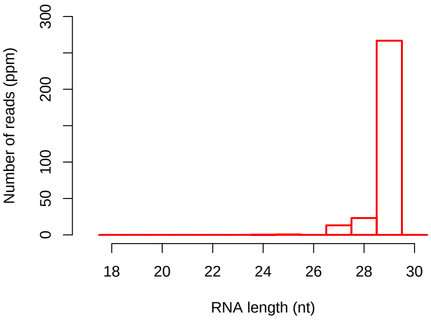

18-mers:

(no read)

19-mers:

(no read)

20-mers:

(no read)

21-mers:

(no read)

22-mers:

(no read)

23-mers:

(no read)

24-mers:

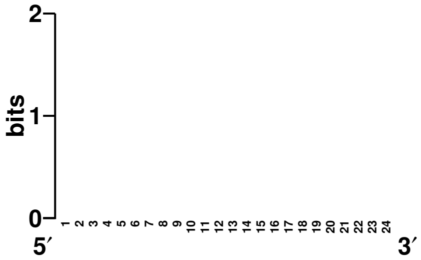

25-mers:

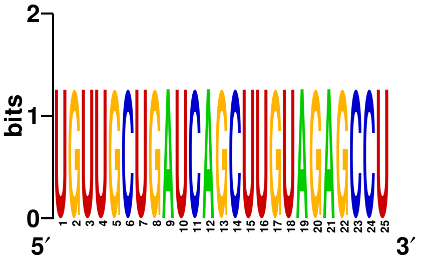

26-mers:

(no read)

27-mers:

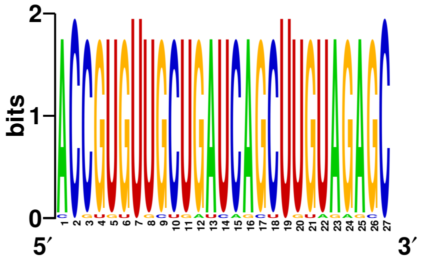

28-mers:

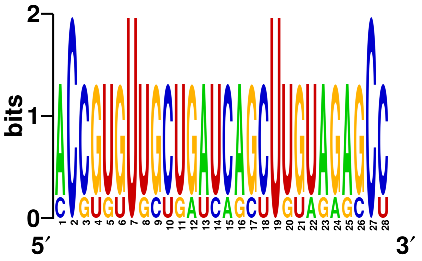

29-mers:

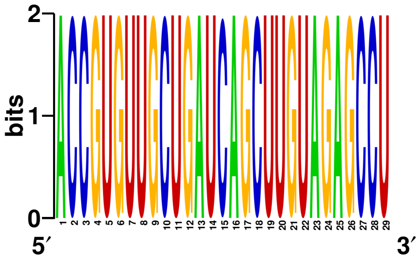

30-mers:

(no read)

Adult female, library 1:

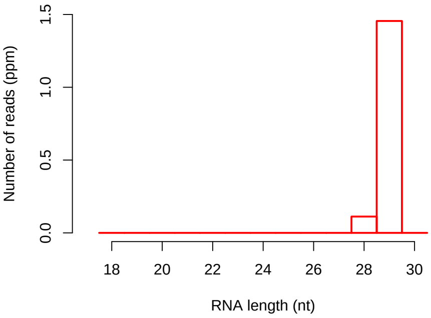

18-mers:

(no read)

19-mers:

(no read)

20-mers:

(no read)

21-mers:

(no read)

22-mers:

(no read)

23-mers:

(no read)

24-mers:

(no read)

25-mers:

(no read)

26-mers:

(no read)

27-mers:

(no read)

28-mers:

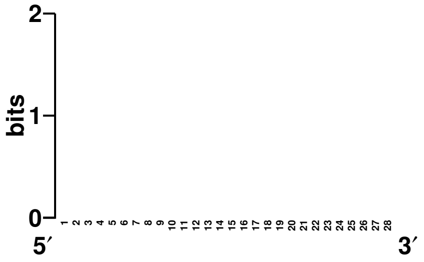

29-mers:

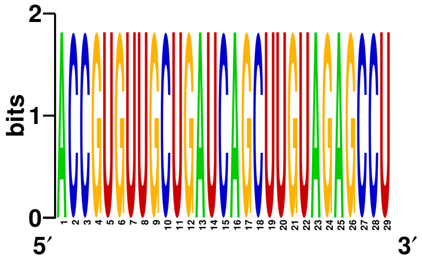

30-mers:

(no read)

Adult male, library 1:

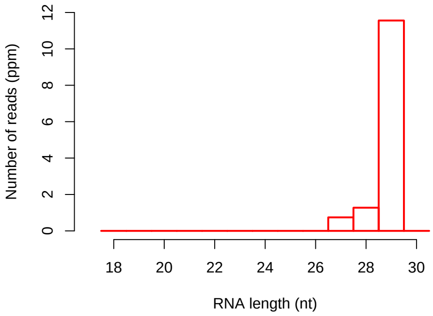

18-mers:

(no read)

19-mers:

(no read)

20-mers:

(no read)

21-mers:

(no read)

22-mers:

(no read)

23-mers:

(no read)

24-mers:

(no read)

25-mers:

(no read)

26-mers:

(no read)

27-mers:

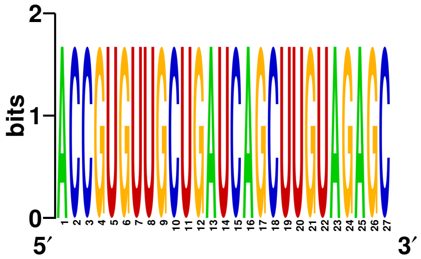

28-mers:

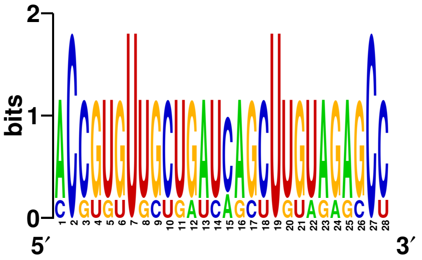

29-mers:

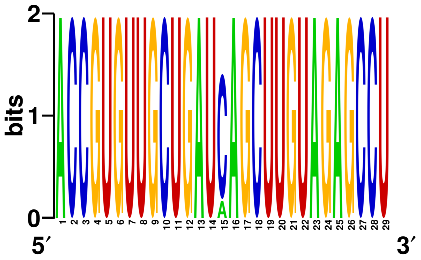

30-mers:

(no read)

15.2 Libraries #2 (3´ modified, 5´ monophosphorylated small RNAs)

Embryo 8h, library 2:

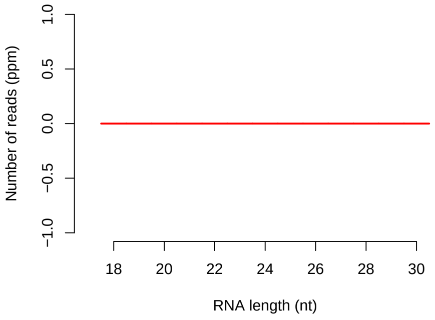		18-mers: (no read)
19-mers:	20-mers:	21-mers:
(no read)	(no read)	(no read)
22-mers:	23-mers:	24-mers:
(no read)	(no read)	(no read)
25-mers:	26-mers:	27-mers:
(no read)	(no read)	(no read)
28-mers:	29-mers:	30-mers:
(no read)	(no read)	(no read)

Embryo 15h, library 2:

18-mers:

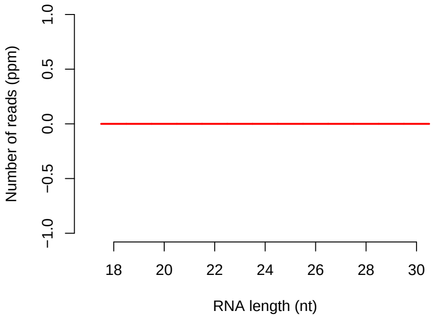

(no read)

19-mers:	20-mers:	21-mers:
(no read)	(no read)	(no read)
22-mers:	23-mers:	24-mers:
(no read)	(no read)	(no read)
25-mers:	26-mers:	27-mers:
(no read)	(no read)	(no read)
28-mers:	29-mers:	30-mers:
(no read)	(no read)	(no read)

Embryo 36h, library 2:

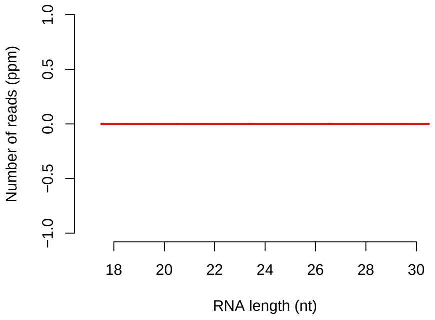

		18-mers:
		(no read)
19-mers:	20-mers:	21-mers:
(no read)	(no read)	(no read)
22-mers:	23-mers:	24-mers:
(no read)	(no read)	(no read)
25-mers:	26-mers:	27-mers:
(no read)	(no read)	(no read)
28-mers:	29-mers:	30-mers:
(no read)	(no read)	(no read)

Embryo 60h, library 2:

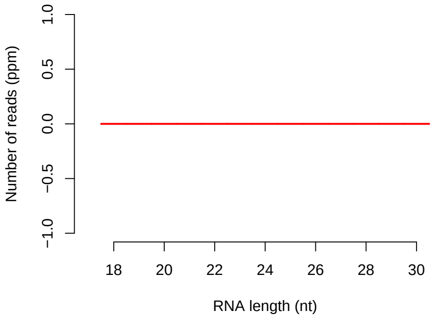

18-mers:
(no read)

19-mers:
(no read)

20-mers:
(no read)

21-mers:
(no read)

22-mers:
(no read)

23-mers:
(no read)

24-mers:
(no read)

25-mers:
(no read)

26-mers:
(no read)

27-mers:
(no read)

28-mers:
(no read)

29-mers:
(no read)

30-mers:
(no read)

Adult female, library 2:

18-mers:

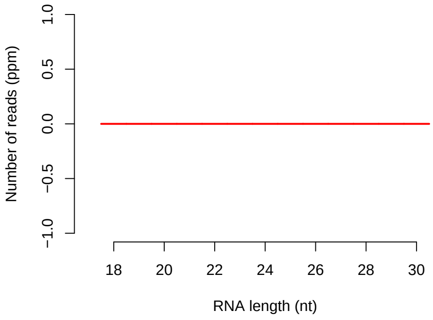

(no read)

19-mers:	20-mers:	21-mers:
(no read)	(no read)	(no read)
22-mers:	23-mers:	24-mers:
(no read)	(no read)	(no read)
25-mers:	26-mers:	27-mers:
(no read)	(no read)	(no read)
28-mers:	29-mers:	30-mers:
(no read)	(no read)	(no read)

Adult male, library 2:

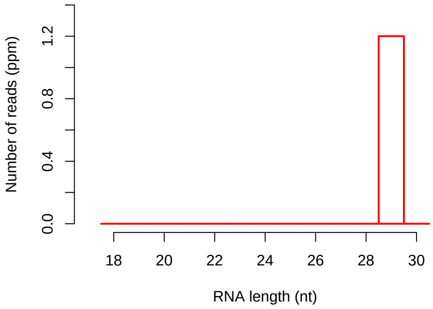

18-mers:

(no read)

19-mers:

(no read)

20-mers:

(no read)

21-mers:

(no read)

22-mers:

(no read)

23-mers:

(no read)

24-mers:

(no read)

25-mers:

(no read)

26-mers:

(no read)

27-mers:

(no read)

28-mers:

(no read)

29-mers:

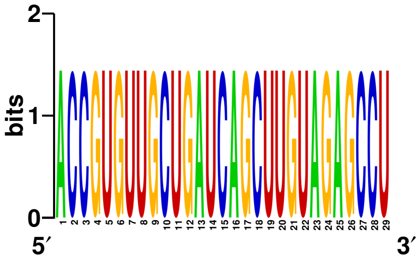

30-mers:

(no read)

15.3 Libraries #3 (total 5´ hydroxyl or polyphosphorylated small RNAs)

Embryo 8h, library 3:

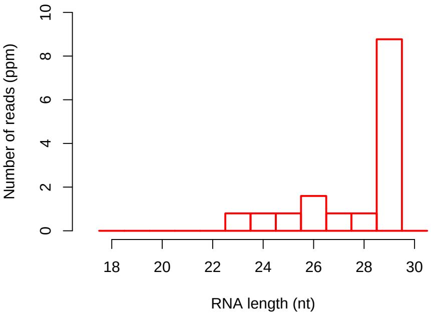

18-mers:

(no read)

19-mers:

(no read)

20-mers:

(no read)

21-mers:

(no read)

22-mers:

(no read)

23-mers:

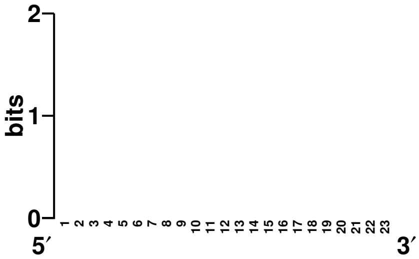

24-mers:

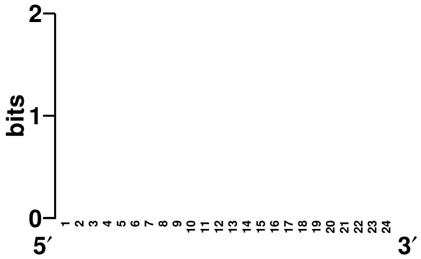

25-mers:

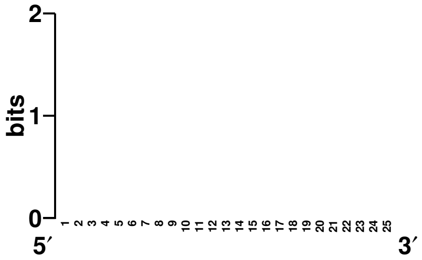

26-mers:

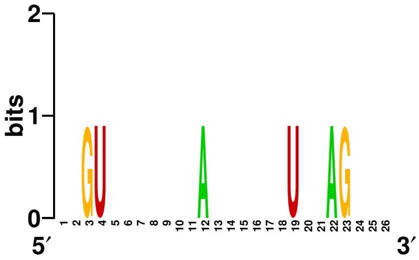

27-mers:

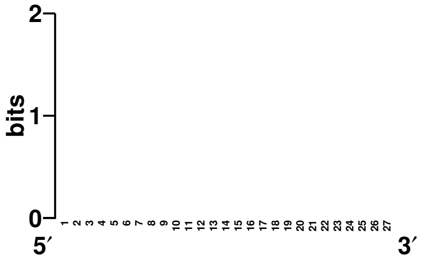

28-mers:

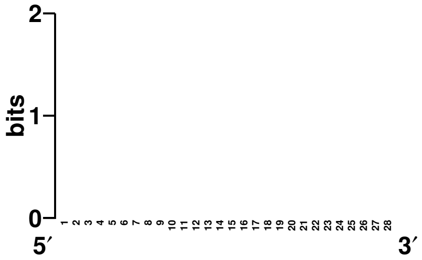

29-mers:

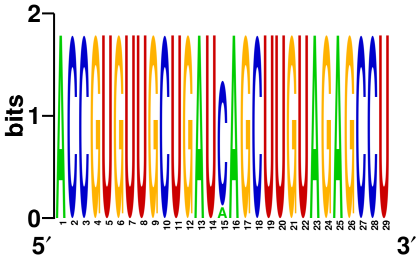

30-mers:

(no read)

Embryo 15h, library 3:

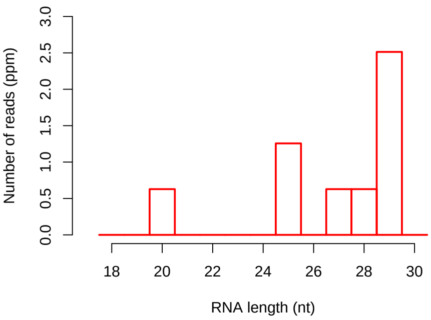

18-mers:

(no read)

19-mers:

(no read)

20-mers:

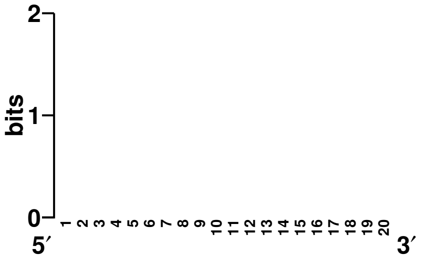

21-mers:

(no read)

22-mers:

(no read)

23-mers:

(no read)

24-mers:

(no read)

25-mers:

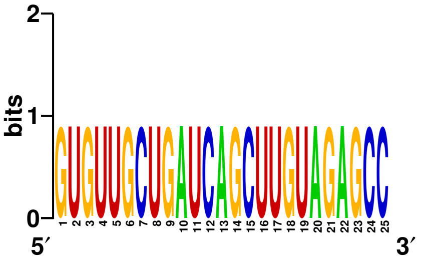

26-mers:

(no read)

27-mers:

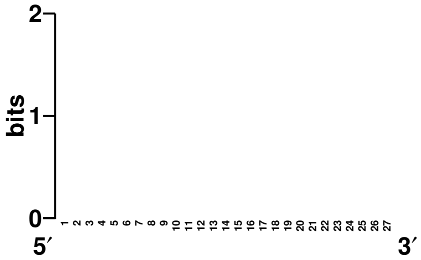

28-mers:

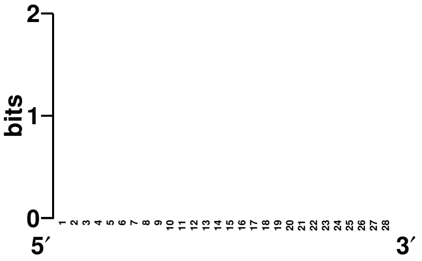

29-mers:

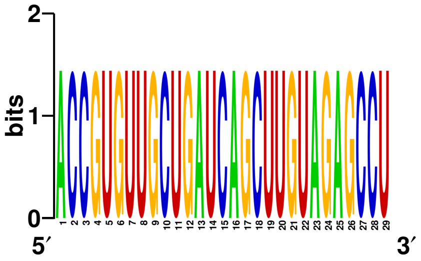

30-mers:

(no read)

Embryo 36h, library 3:

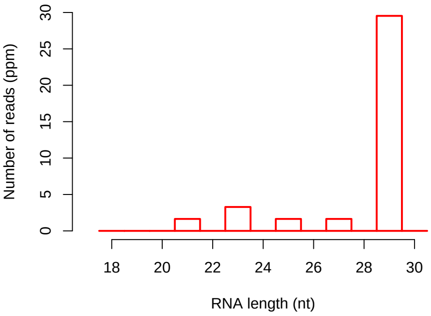

18-mers:

(no read)

19-mers:

(no read)

20-mers:

(no read)

21-mers:

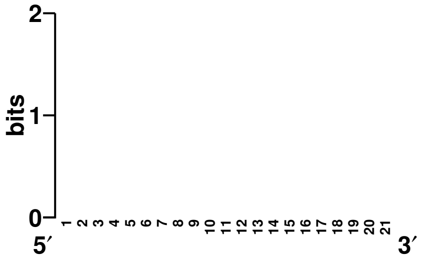

22-mers:

(no read)

23-mers:

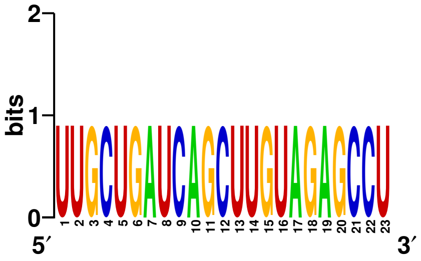

24-mers:

(no read)

25-mers:

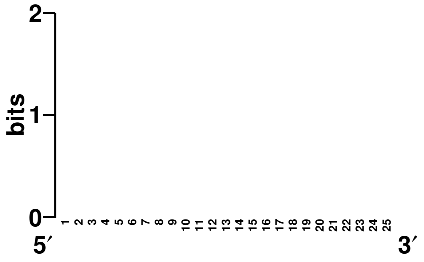

26-mers:

(no read)

27-mers:

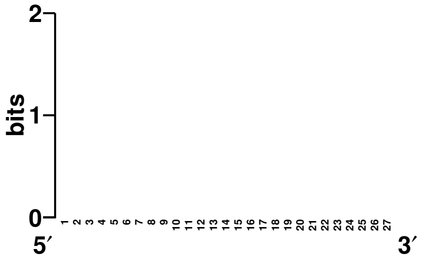

28-mers:

(no read)

29-mers:

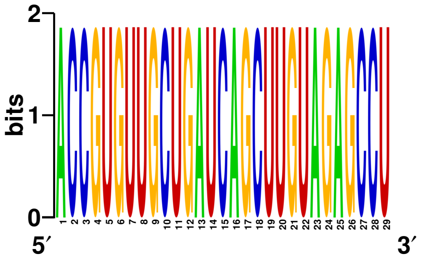

30-mers:

(no read)

Embryo 60h, library 3:

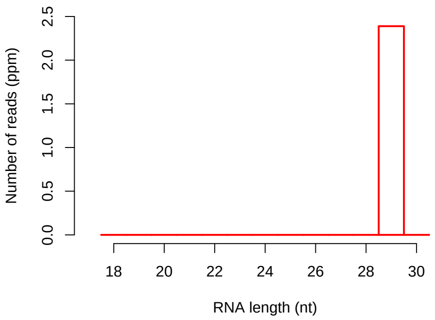

18-mers:

(no read)

19-mers:	20-mers:	21-mers:
(no read)	(no read)	(no read)
22-mers:	23-mers:	24-mers:
(no read)	(no read)	(no read)
25-mers:	26-mers:	27-mers:
(no read)	(no read)	(no read)
28-mers:	29-mers:	30-mers:
(no read)	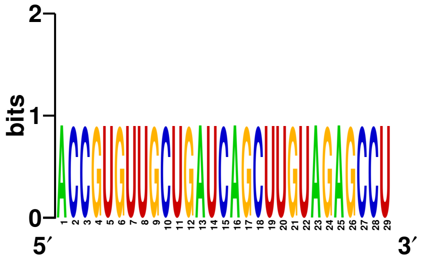	(no read)

Adult female, library 3:

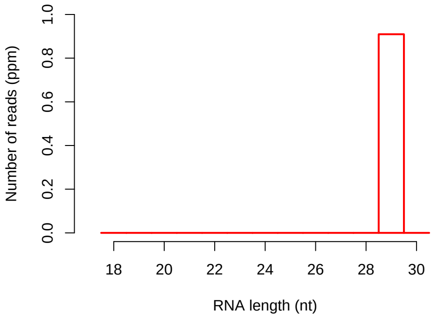

18-mers:

(no read)

19-mers:

(no read)

20-mers:

(no read)

21-mers:

(no read)

22-mers:

(no read)

23-mers:

(no read)

24-mers:

(no read)

25-mers:

(no read)

26-mers:

(no read)

27-mers:

(no read)

28-mers:

(no read)

29-mers:

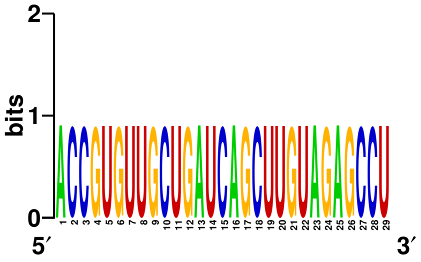

30-mers:

(no read)

Adult male, library 3:

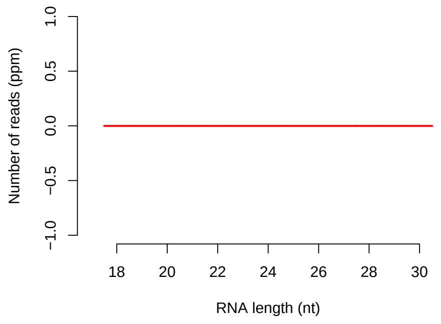

18-mers:
(no read)

19-mers:
(no read)

20-mers:
(no read)

21-mers:
(no read)

22-mers:
(no read)

23-mers:
(no read)

24-mers:
(no read)

25-mers:
(no read)

26-mers:
(no read)

27-mers:
(no read)

28-mers:
(no read)

29-mers:
(no read)

30-mers:
(no read)

15.4 Libraries #4 (3´ modified, 5´ hydroxyl or polyphosphorylated small RNAs)

Embryo 8h, library 4:

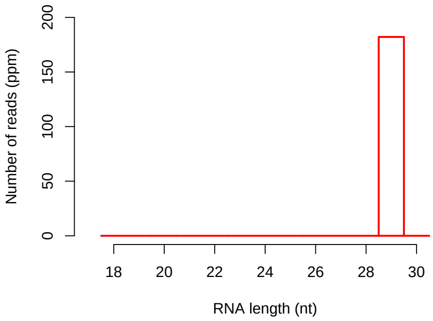

18-mers:
(no read)

19-mers:	20-mers:	21-mers:
(no read)	(no read)	(no read)
22-mers:	23-mers:	24-mers:
(no read)	(no read)	(no read)
25-mers:	26-mers:	27-mers:
(no read)	(no read)	(no read)
28-mers:	29-mers:	30-mers:
(no read)	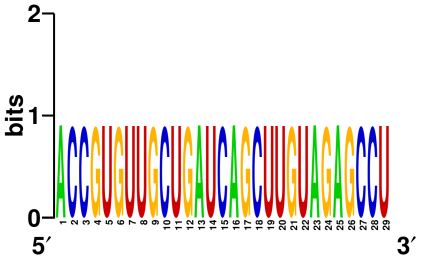	(no read)

Embryo 15h, library 4:

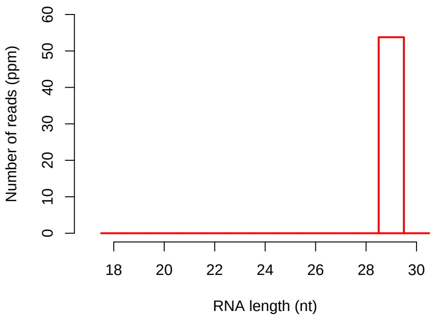

18-mers:
(no read)

19-mers:
(no read)

20-mers:
(no read)

21-mers:
(no read)

22-mers:
(no read)

23-mers:
(no read)

24-mers:
(no read)

25-mers:
(no read)

26-mers:
(no read)

27-mers:
(no read)

28-mers:
(no read)

29-mers:

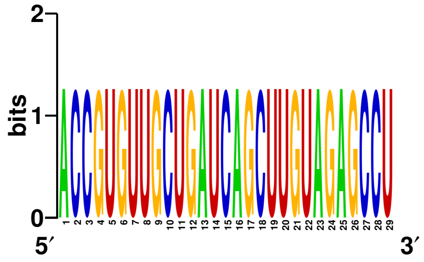

30-mers:
(no read)

Embryo 36h, library 4:

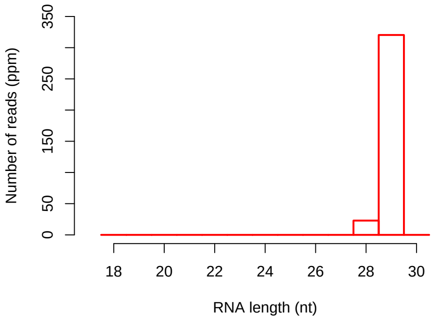

18-mers:

(no read)

19-mers:

(no read)

20-mers:

(no read)

21-mers:

(no read)

22-mers:

(no read)

23-mers:

(no read)

24-mers:

(no read)

25-mers:

(no read)

26-mers:

(no read)

27-mers:

(no read)

28-mers:

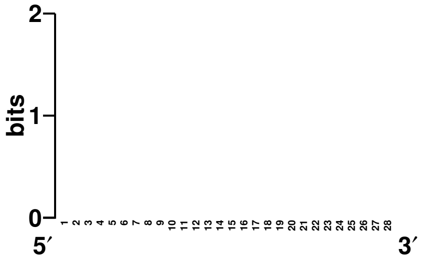

29-mers:

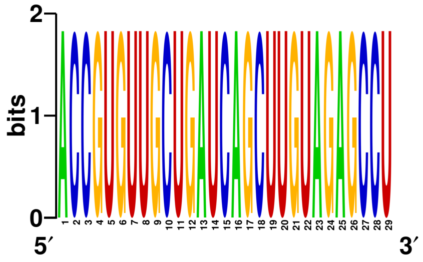

30-mers:

(no read)

Embryo 60h, library 4:

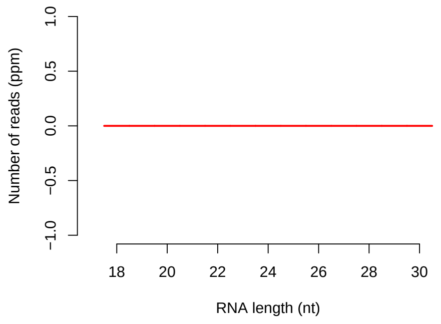

18-mers:
(no read)

19-mers:
(no read)

20-mers:
(no read)

21-mers:
(no read)

22-mers:
(no read)

23-mers:
(no read)

24-mers:
(no read)

25-mers:
(no read)

26-mers:
(no read)

27-mers:
(no read)

28-mers:
(no read)

29-mers:
(no read)

30-mers:
(no read)

Adult female, library 4:

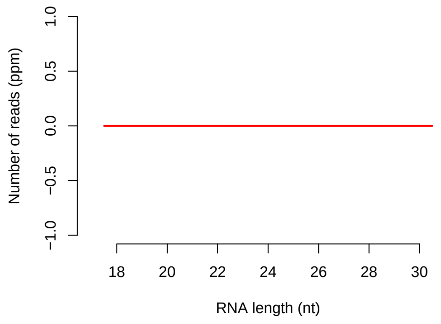

18-mers:

(no read)

19-mers:

(no read)

20-mers:

(no read)

21-mers:

(no read)

22-mers:

(no read)

23-mers:

(no read)

24-mers:

(no read)

25-mers:

(no read)

26-mers:

(no read)

27-mers:

(no read)

28-mers:

(no read)

29-mers:

(no read)

30-mers:

(no read)

Adult male, library 4:

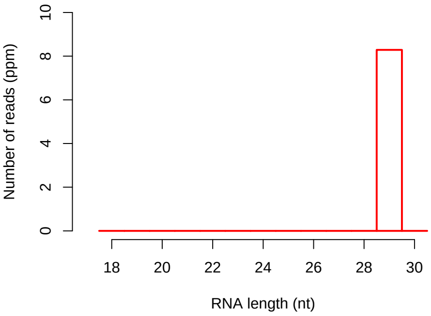

18-mers:
(no read)

19-mers:
(no read)

20-mers:
(no read)

21-mers:
(no read)

22-mers:
(no read)

23-mers:
(no read)

24-mers:
(no read)

25-mers:
(no read)

26-mers:
(no read)

27-mers:
(no read)

28-mers:
(no read)

29-mers:

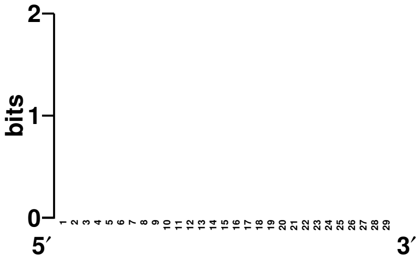

30-mers:
(no read)

References

[Gu et al., 2009] Gu, W., Shirayama, M., Conte, D. J., Vasale, J., Batista, P. J., Claycomb, J. M., Moresco, J. J., Youngman, E. M., Keys, J., Stoltz, M. J., Chen, C. C., Chaves, D. A., Duan, S., Kasschau, K. D., Fahlgren, N., Yates, J. R., Mitani, S., Carrington, J. C., and Mello, C. C. (2009). Distinct argonaute-mediated 22G-RNA pathways direct genome surveillance in the *C. elegans* germline. *Mol Cell*, 36(2):231–244.
