# Supplementary material for: Functional lability of RNA-dependent RNA polymerases in animals
Source: PLoS Genet. 2019 Feb 19;15(2):e1007915. doi: 10.1371/journal.pgen.1007915 (PMC6396948; doi:10.1371/journal.pgen.1007915)
Supplement: S1 Table — Branchiostoma lanceolatum orthologs for B. floridae or B. belcheri pre-miRNA hairpins (as described in miRBase v.22) were screened for their predicted secondary structure and the abundance of the small RNAs they generate. Only those hairpins that comply with these rules are shown in this table. First column: name of orthologous pre-miRNA, and genomic coordinates in B. lanceolatum. Second column: sequences of the major forms of the 5′ arm and 3′ arm miRNAs, if expressed at ≥10 ppm in at least one developmental stage (miRNAs that do not meet that criterion are flagged “low abundance”). Third column: abundance of the 5′ arm and 3′ arm miRNAs in Libraries #1 along development. Embryonic stages contain mixed sexes; adult stages are shown in blue and pink for males and females, respectively. Trimming (up to 3 nt) and templated extension of miRNA 3′ ends were considered when measuring read counts. (PDF) [file pgen.1007915.s008.pdf]

Pre-miRNA	miRNA sequences	Abundance profile in development
bfl-mir-71 ortholog at Sc0000005 bp 2992213-2992115 (- strand)	5´arm: `UGAAAGACAUGGGUAGUGAGAU`<br><br>3´arm: `CCCAUUUCCCUGUCUUUCAAC`	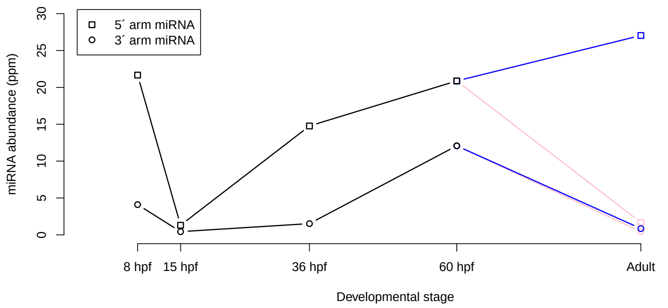
bfl-mir-4856b ortholog at Sc0000022 bp 2102339-2102425 (+ strand)	5´arm: `UCGCAUUGACGUCAGCGCCGUU`<br><br>3´arm: `(low abundance)`	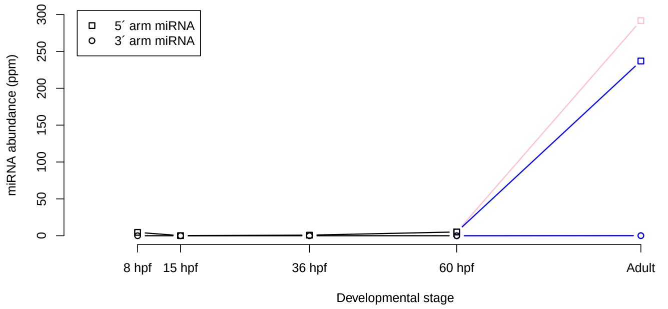
bfl-mir-4876 ortholog at Sc0000005 bp 2219860-2219951 (+ strand)	5´arm: `(low abundance)`<br><br>3´arm: `CUUACGUGCCACGUGAGGACUCU`	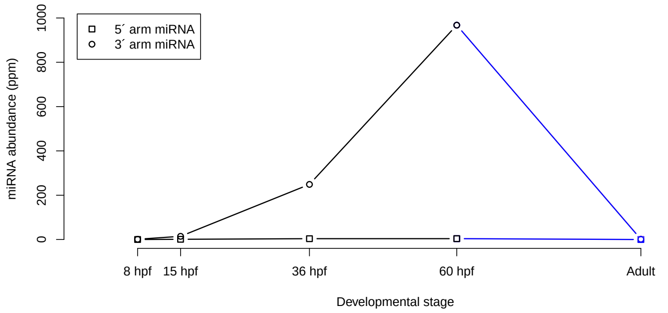
bfl-mir-10c ortholog at Sc0000000 bp 2027699-2027621 (- strand)	5´arm: `UACCCUGUAGAUCCGGACUUGUGA`<br><br>3´arm: `(low abundance)`	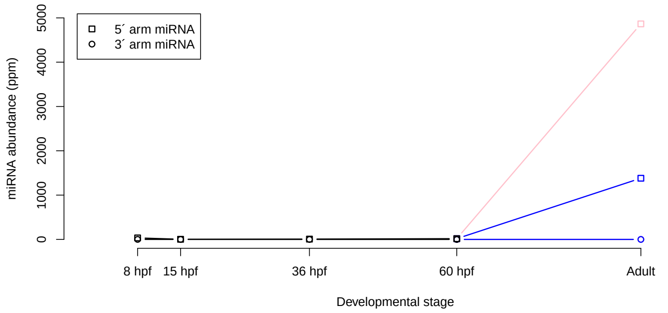

(continued on next page)

Pre-miRNA	miRNA sequences	Abundance profile in development
bfl-mir-4869 ortholog at Sc0000022 bp 2099003-2099080 (+ strand)	5´arm: `ACGAUGUUGACUUCCGCUCCUCU`<br>3´arm: `GGGACCUGUAGUCAACACGAGA`	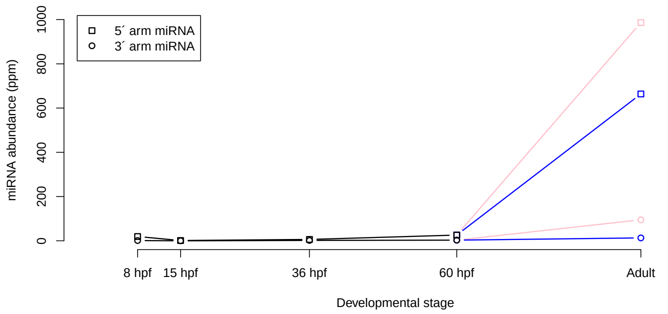
bbe-mir-125a ortholog at Sc0000265 bp 98448-98548 (+ strand)	5´arm: `UCCCUGAGACCCUAACUUGUGA`<br>3´arm: `ACAGGUUAGGAUCUUGGGAGCU`	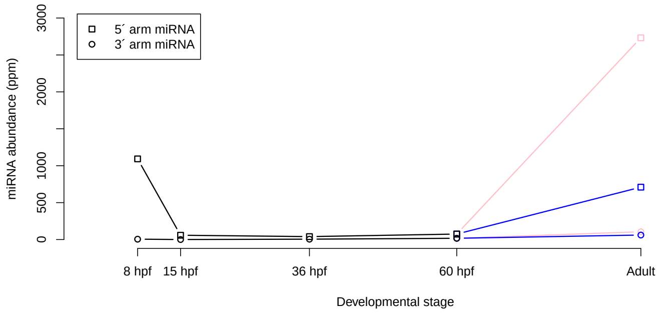
bbe-mir-4874 ortholog at Sc0000043 bp 1147175-1147086 (- strand)	5´arm: `AGUUUGUAGCAUCCAGCUGAGCU`<br>3´arm: `CUGGCUGGUUGGUGCAAACAGG`	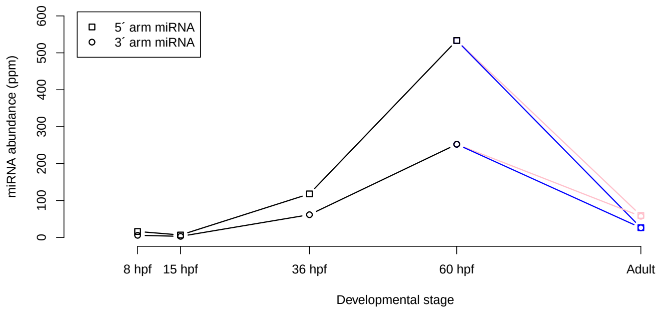
bbe-mir-281 ortholog at Sc0000000 bp 9083742-9083658 (- strand)	5´arm: `AGGAGAGCCGUUCUGUGACUGU`<br>3´arm: `(low abundance)`	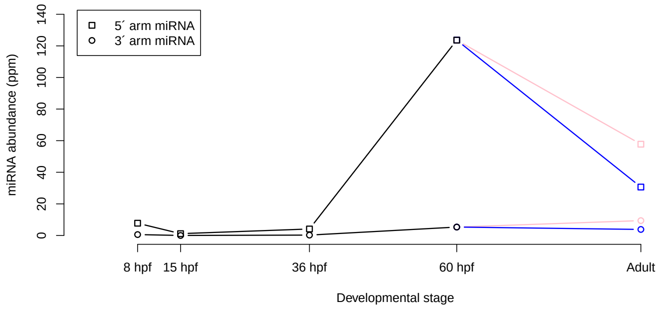

(continued on next page)

Pre-miRNA	miRNA sequences	Abundance profile in development
bbe-mir-92a-2 ortholog at Sc0000007 bp 4735603-4735697 (+ strand)	5´arm: AGGCCAGGAUUGGUGGCAAUGCC<br>3´arm: UAUUGCACUUGUCCCGGCCUUU	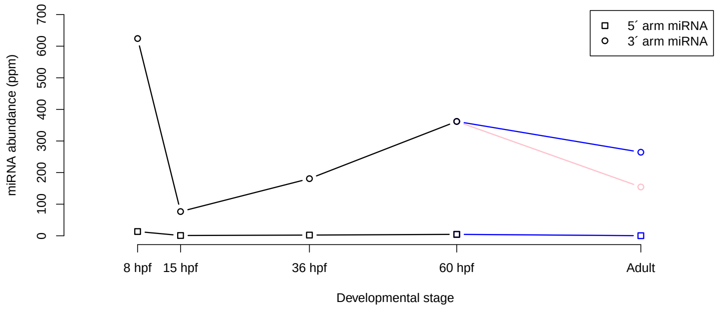
bbe-mir-2056 ortholog at Sc0000086 bp 322130-322049 (- strand)	5´arm: CAGGUAUGUCUGCGGUGAGGCU<br>3´arm: UUUCACUGUAGAUCUACCUGCG	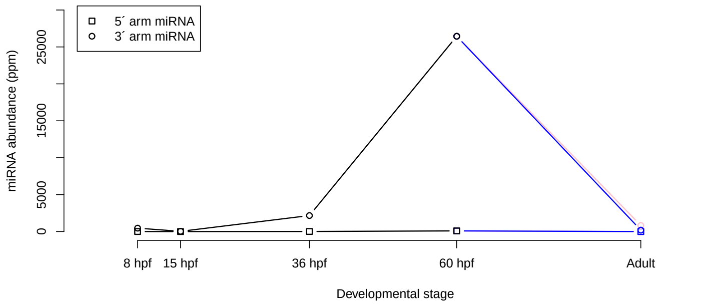
bbe-mir-4880 ortholog at Sc0000057 bp 1180856-1180942 (+ strand)	5´arm: UUUGCUAUUCGAUGACCAGUGG<br>3´arm: (low abundance)	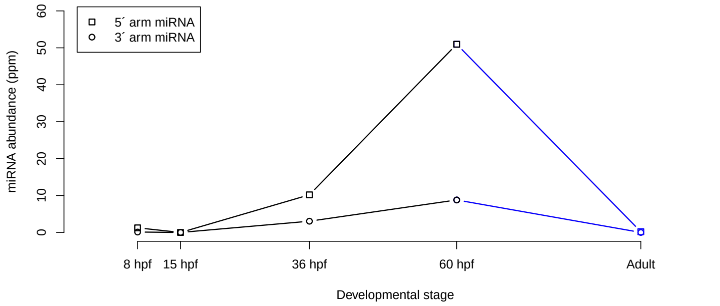
bbe-mir-31 ortholog at Sc0000399 bp 174399-174498 (+ strand)	5´arm: UGGCAAGAUGUUGGCAUAGCUG<br>3´arm: (low abundance)	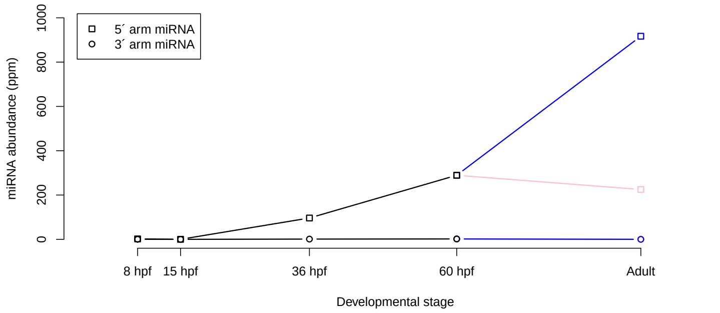

(continued on next page)

Pre-miRNA	miRNA sequences	Abundance profile in development
bfl-mir-200b ortholog at Sc0000010 bp 3877902-3877999 (+ strand)	5´arm:<br>(low abundance)<br><br>3´arm:<br>UAAUACUGUCUGGUAAUGAUGUU	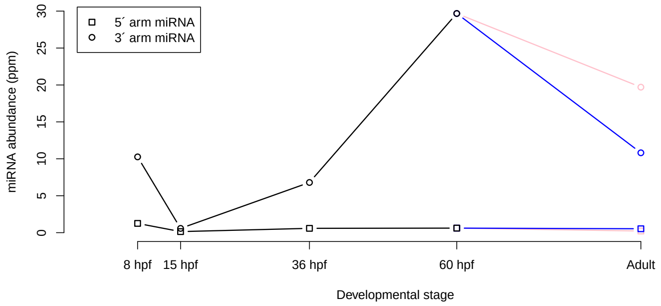
bfl-mir-2071 ortholog at Sc0000288 bp 188813-188893 (+ strand)	5´arm:<br>AUGCGGUGCGGUGGUAGCAACCG<br><br>3´arm:<br>AUUGUUACACCGCGCCGCAAAAG	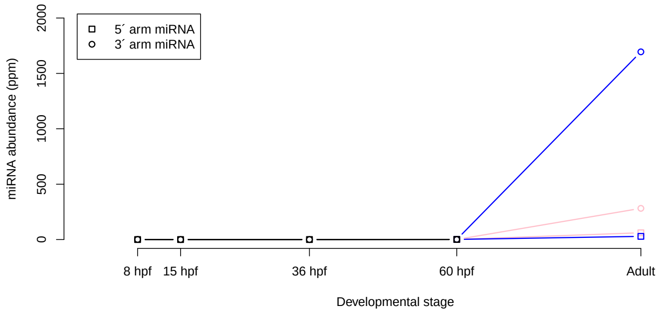
bfl-mir-4865 ortholog at Sc0000063 bp 315717-315798 (+ strand)	5´arm:<br>(low abundance)<br><br>3´arm:<br>UGUAGAGAGAGUGACAGGUUGU	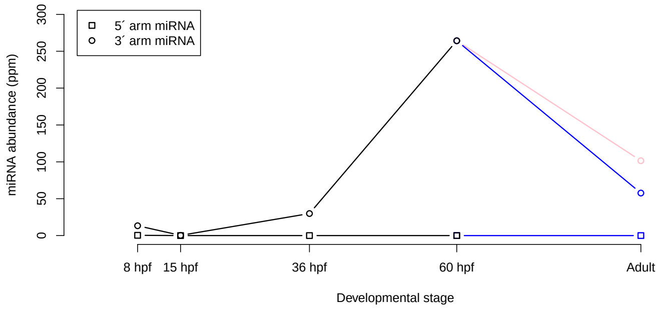
bbe-mir-200c ortholog at Sc0000010 bp 3875272-3875359 (+ strand)	5´arm:<br>(low abundance)<br><br>3´arm:<br>UAACACUGUCUGGUAAUGAUG	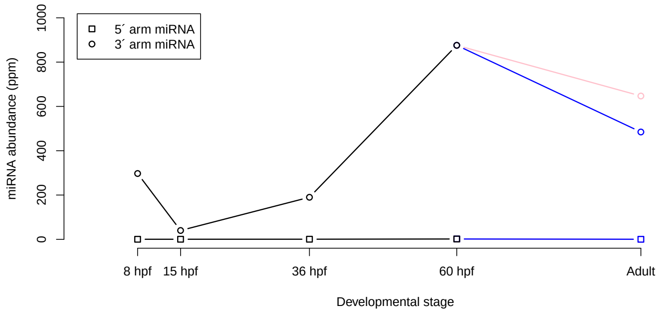

(continued on next page)

Pre-miRNA	miRNA sequences	Abundance profile in development
bfl-mir-29a ortholog at Sc0000043 bp 1849481-1849575 (+ strand)	5´arm: `GCUGAUUUCAGUUGGUGCUAGA`<br><br>3´arm: `(low abundance)`	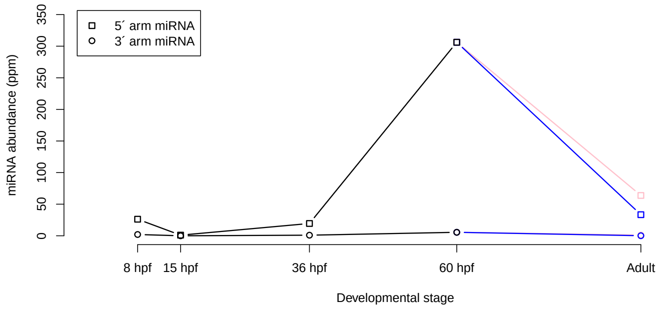
bbe-mir-2058 ortholog at Sc0000110 bp 921627-921549 (- strand)	5´arm: `UGAGAAGUAAGACUACCAUCCCGU`<br><br>3´arm: `(low abundance)`	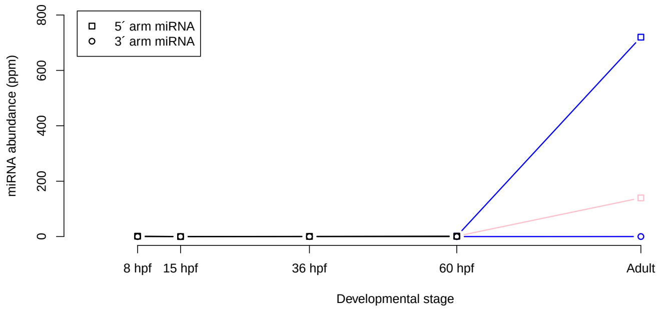
bfl-let-7a-2 ortholog at Sc0000265 bp 98758-98852 (+ strand)	5´arm: `UGAGGUAGUAGGUUGUAUAGUU`<br><br>3´arm: `CUGUGCAACCUGCUAGCUCUCC`	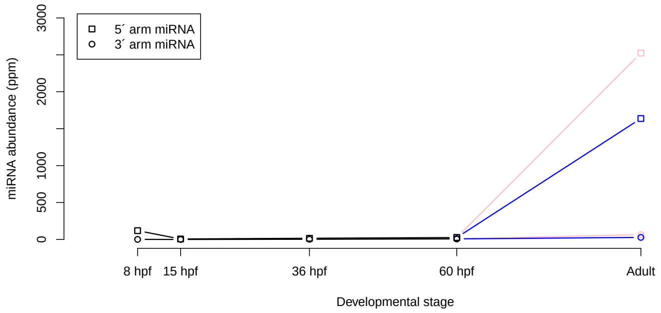
bfl-mir-200c ortholog at Sc0000010 bp 3875267-3875358 (+ strand)	5´arm: `(low abundance)`<br><br>3´arm: `UAACACUGUCUGGUAAUGAUG`	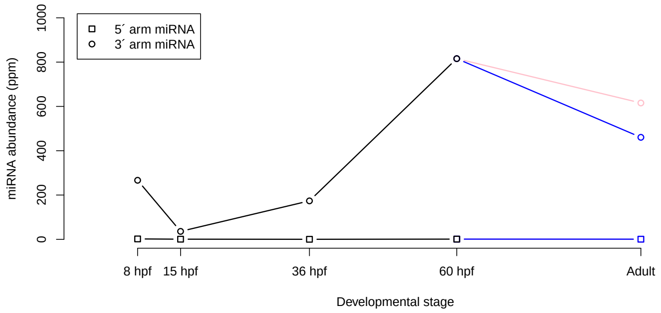

(continued on next page)

Pre-miRNA	miRNA sequences	Abundance profile in development
bfl-mir-4860 ortholog at Sc0000063 bp 316331-316404 (+ strand)	5´arm: UGCCUGUCAACGUCUCUGUACA<br>3´arm: UGUAGAGAUUGUGUGACGGGUAGU	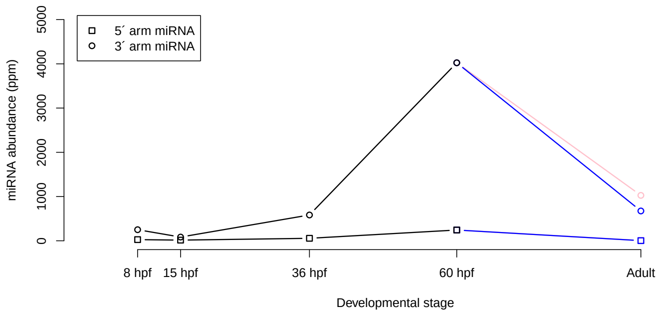
bfl-mir-4864 ortholog at Sc0000184 bp 79034-79113 (+ strand)	5´arm: AGGGAGAUCGUCUCGGGCAUACA<br>3´arm: UAGCCAGACCUGAUCUCCCUGC	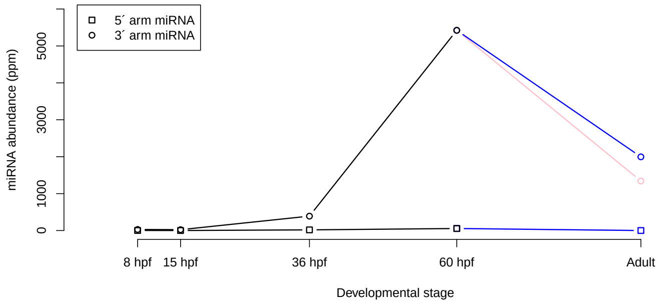
bfl-mir-4861 ortholog at Sc0000005 bp 3994270-3994362 (+ strand)	5´arm: AGCCAAUGCGGCAUGUAAAAGGC<br>3´arm: UUUACGUGCCACAUUGUCUCCU	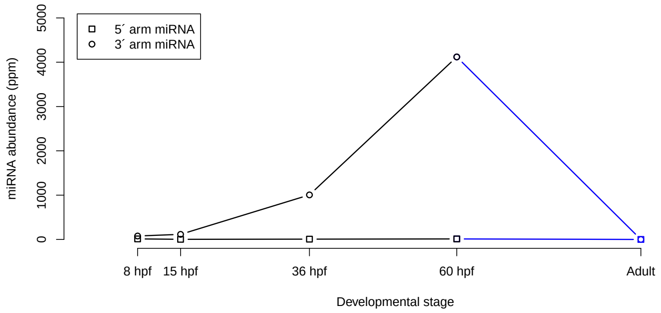
bfl-mir-2062 ortholog at Sc0000099 bp 755622-755703 (+ strand)	5´arm: UGCAACAAUAUUUCAUCAGUGG<br>3´arm: ACUGGUGAAAUGUAGUUGCGUA	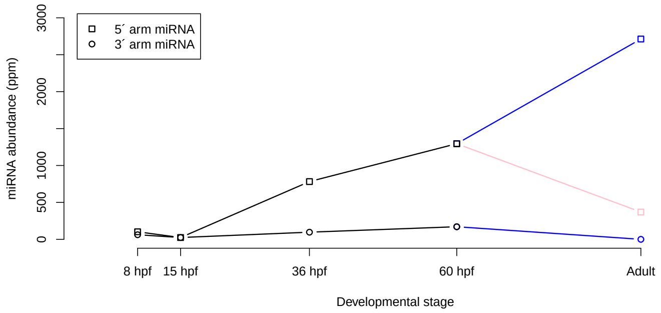

(continued on next page)

<table>
<tr><th>Pre-miRNA</th><th>miRNA sequences</th><th>Abundance profile in development</th></tr>
<tr><td>bbe-mir-4859 ortholog at Sc0000221 bp 303532-303447 (- strand)</td><td>5´arm:
AGCAGCGAGCAUUACGGUCAUU

3´arm:
UGACAGUAAUGCCCGCUGACUU</td><td>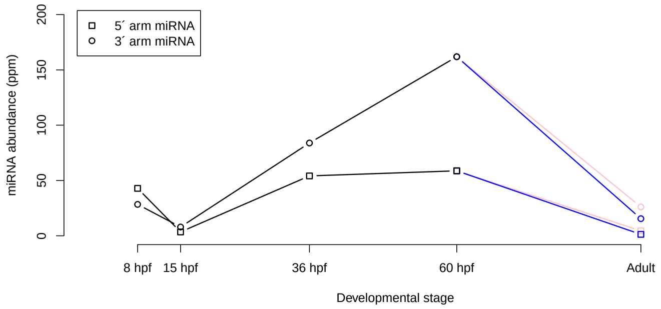
</td></tr>
<tr><td>bfl-mir-4871 ortholog at Sc0000001 bp 4527403-4527475 (+ strand)</td><td>5´arm:
UCUGAAGUACCUGUUGCCAAAGG

3´arm:
UUUGGCACUGGUACUUUGGAGU</td><td>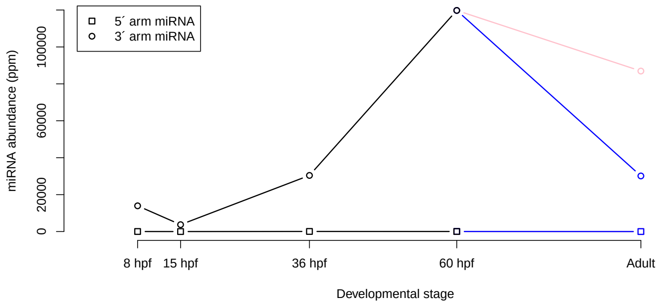
</td></tr>
<tr><td>bbe-mir-100 ortholog at Sc0000265 bp 96682-96781 (+ strand)</td><td>5´arm:
AACCCGUAGAUCCGAACUUGUGU

3´arm:
CAAGCUCGUGUCUAUGGGUCU</td><td>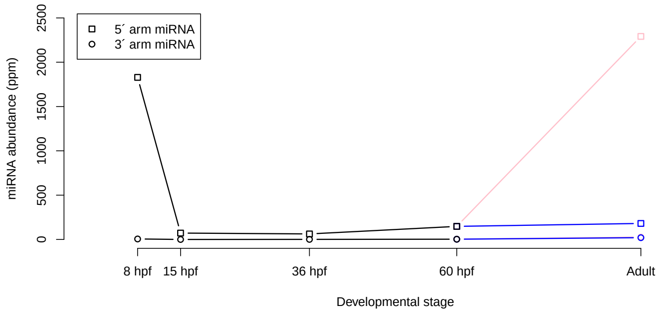
</td></tr>
<tr><td>bbe-mir-22 ortholog at Sc0000015 bp 676722-676797 (+ strand)</td><td>5´arm:
AGCUCUUCACUCGGUAGCUCUG

3´arm:
AAGCUGCCAGAUGAAGAGCUGU</td><td>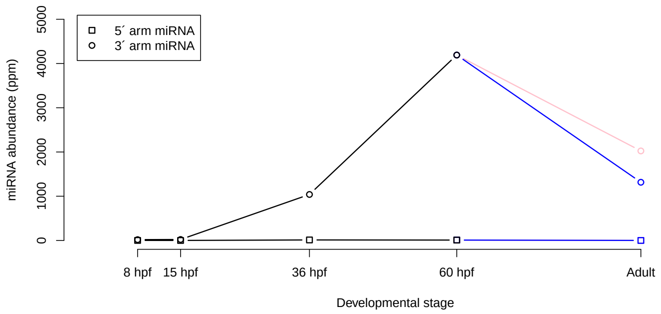
</td></tr>
<tr><td colspan="3">(continued on next page)</td></tr>
</table>

<table>
<tr><th>Pre-miRNA</th><th>miRNA sequences</th><th>Abundance profile in development</th></tr>
<tr><td>bfl-mir-190 ortholog at Sc0000166 bp 638463-638558 (+ strand)</td><td>5´arm:
UGAUAUGUUUGAUAUUUGGUUG

3´arm:
(low abundance)</td><td>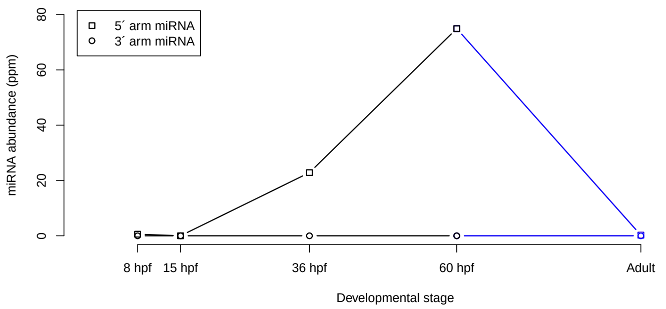
</td></tr>
<tr><td>bbe-mir-2057 ortholog at Sc0000110 bp 922600-922519 (- strand)</td><td>5´arm:
UGAGAAGUUAGCCAACCAUCCGG

3´arm:
(low abundance)</td><td>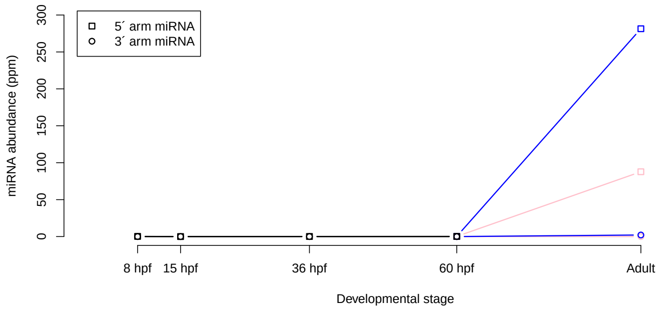
</td></tr>
<tr><td>bbe-mir-2061 ortholog at Sc0000005 bp 2547710-2547629 (- strand)</td><td>5´arm:
(low abundance)

3´arm:
UUGCAUAGGUACAUUGGUCAGU</td><td>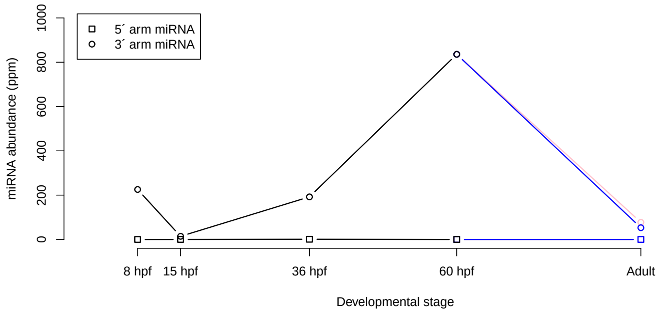
</td></tr>
<tr><td>bfl-mir-124 ortholog at Sc0000076 bp 963692-963792 (+ strand)</td><td>5´arm:
AGUGUUCACGGCGGUCCUUAAU

3´arm:
(low abundance)</td><td>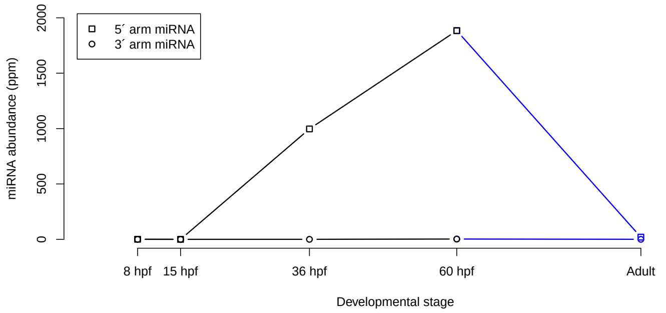
</td></tr>
</table>

(continued on next page)

Pre-miRNA	miRNA sequences	Abundance profile in development
bfl-mir-4873 ortholog at Sc0000015 bp 1919187-1919110 (- strand)	5´arm: UGUUCCCACCUUCUGAUGUUGUU<br>3´arm: (low abundance)	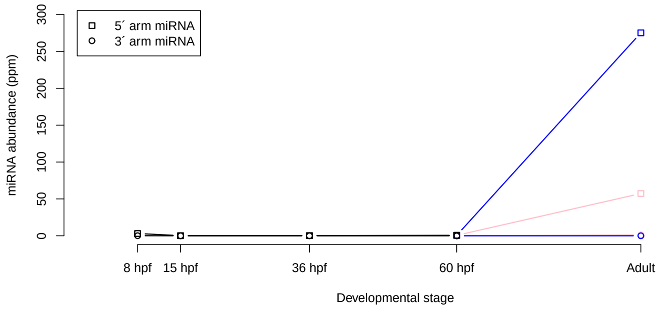
bfl-mir-4891 ortholog at Sc0000002 bp 2469330-2469421 (+ strand)	5´arm: (low abundance)<br>3´arm: CGUACCAGGACGCUCGUCUGCC	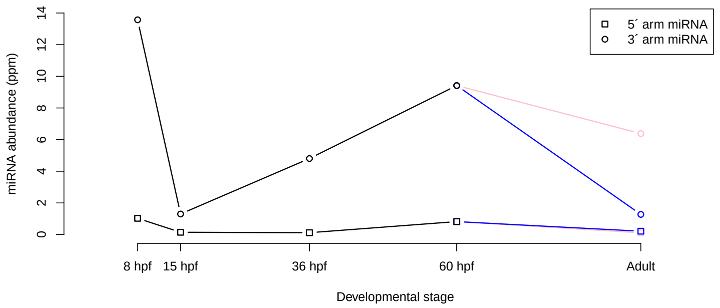
bfl-mir-2070 ortholog at Sc0000079 bp 1008448-1008530 (+ strand)	5´arm: UUUCCACAGCCUCUACACAUGU<br>3´arm: AUGUGCAUAAGCUGUGGGAGCA	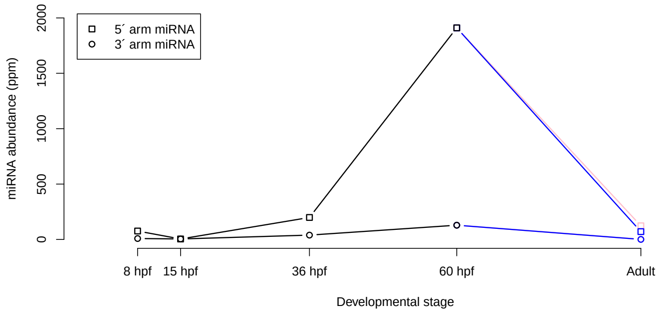
bfl-mir-2076 ortholog at Sc0000004 bp 4642959-4643036 (+ strand)	5´arm: AAUUGCACUAGAGUGAUUUGUU<br>3´arm: (low abundance)	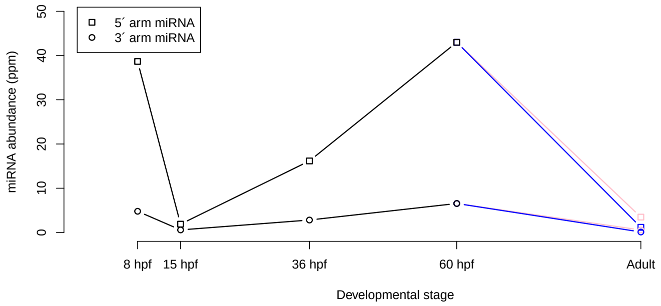

(continued on next page)

Pre-miRNA	miRNA sequences	Abundance profile in development
bbe-mir-133 ortholog at Sc0000092 bp 690262-690162 (- strand)	5´arm:<br>`AAAGCUGGUAAAUUGGAACCA`<br><br>3´arm:<br>`UUGGUCCCCUUCAACCAGCUGU`	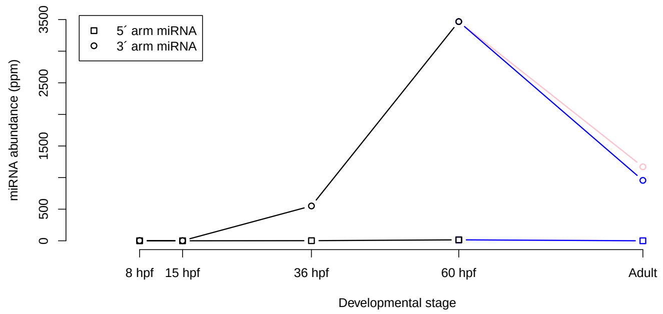
bfl-mir-92b ortholog at Sc0000007 bp 2890460-2890560 (+ strand)	5´arm:<br>`AGGUCUGGACAGUUGCAAUCUU`<br><br>3´arm:<br>`CAUUGCACUCGUCCCGGCCUGA`	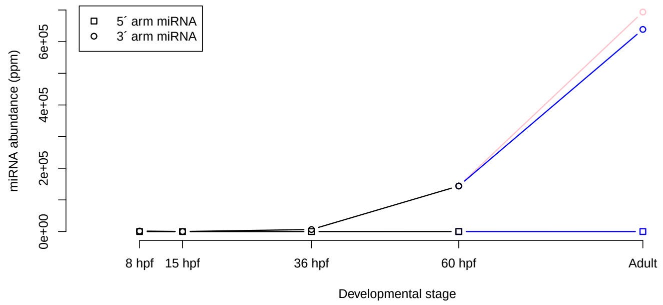
bfl-mir-137 ortholog at xpSc0039671 bp 309878-309778 (- strand)	5´arm:<br>`(low abundance)`<br><br>3´arm:<br>`UAUUGCUUGAGAAUACACGUGA`	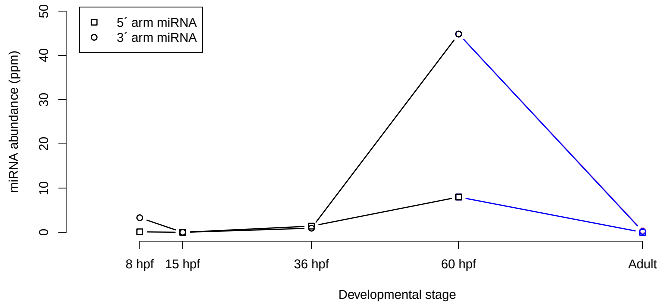
bfl-mir-4868b ortholog at Sc0000017 bp 1346380-1346293 (- strand)	5´arm:<br>`CUCAUCACACCGGAAGCUGUUA`<br><br>3´arm:<br>`UCAGCUCCAGCUGUGAUGAGUG`	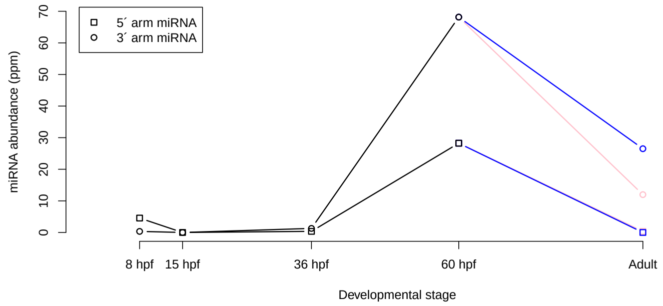

(continued on next page)

<table>
<tr><th>Pre-miRNA</th><th>miRNA sequences</th><th>Abundance profile in development</th></tr>
<tr><td>bfl-mir-242 ortholog at Sc0000023 bp 500019-499937 (- strand)</td><td>5´arm:
UUGCGUAGGCGUUGUGCACACU

3´arm:
(low abundance)</td><td>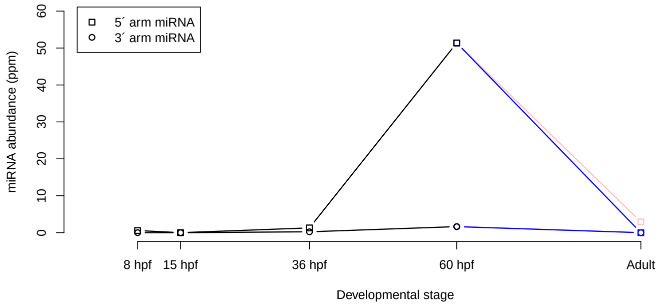
</td></tr>
<tr><td>bfl-mir-92d ortholog at Sc0000062 bp 529331-529413 (+ strand)</td><td>5´arm:
(low abundance)

3´arm:
UAUUGCACUUAUCCUGGCCUGU</td><td>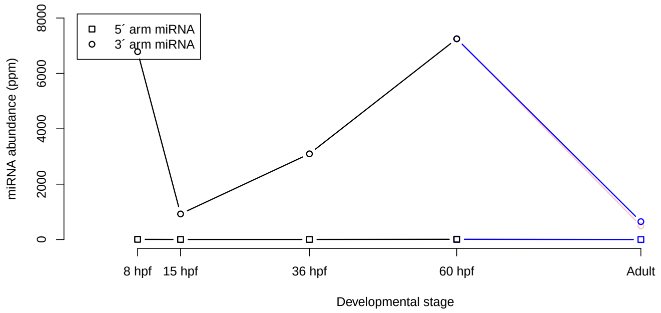
</td></tr>
<tr><td>bfl-mir-4880 ortholog at Sc0000057 bp 1180852-1180940 (+ strand)</td><td>5´arm:
UUUGCUAUUCGAUGACCAGUGG

3´arm:
(low abundance)</td><td>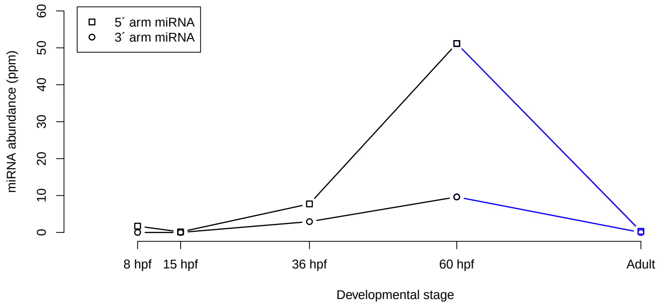
</td></tr>
<tr><td>bbe-mir-92a-1 ortholog at Sc0000007 bp 4735908-4735986 (+ strand)</td><td>5´arm:
AGGUCGUGAUAGGCGGCAAUGUU

3´arm:
UAUUGCACUUGUCCCGGCCUUU</td><td>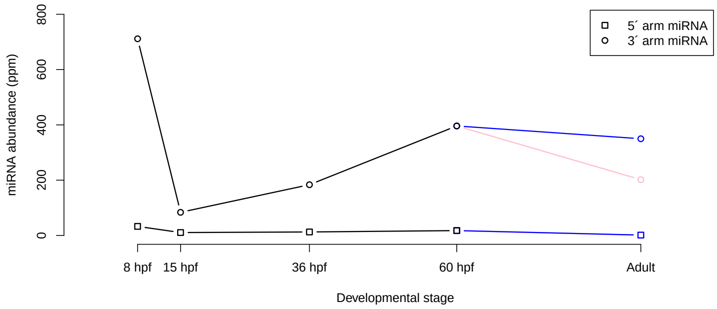
</td></tr>
</table>

(continued on next page)

Pre-miRNA	miRNA sequences	Abundance profile in development
bfl-mir-2058 ortholog at Sc0000110 bp 921624-921546 (- strand)	5´arm: `UGAGAAGUAAGACUACCAUCCCGU`<br>3´arm: `(low abundance)`	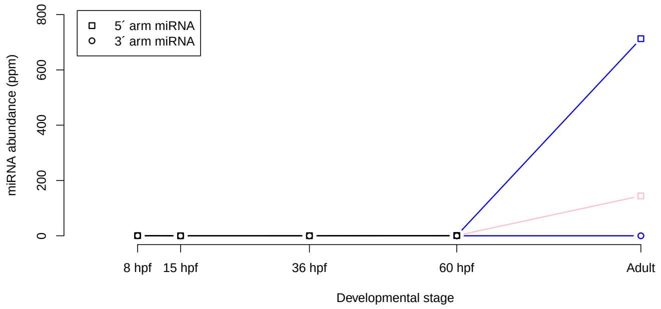
bfl-mir-10b ortholog at Sc0000000 bp 1938354-1938254 (- strand)	5´arm: `AACCCUGUGGAUCCGAUCUUGUG`<br>3´arm: `(low abundance)`	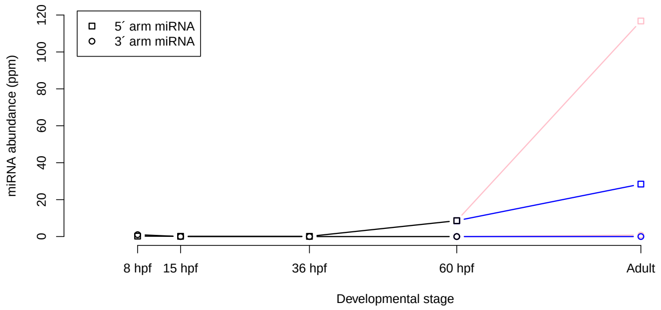
bfl-mir-4856a ortholog at Sc0000022 bp 2103002-2103085 (+ strand)	5´arm: `ACGCAGUGACGUCAGCGCCUCU`<br>3´arm: `(low abundance)`	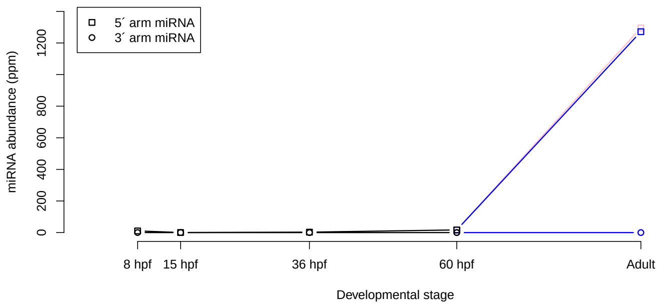
bfl-mir-183 ortholog at Sc0000043 bp 398562-398462 (- strand)	5´arm: `UAUGGCACUGGUAGAAUUCACUGA`<br>3´arm: `(low abundance)`	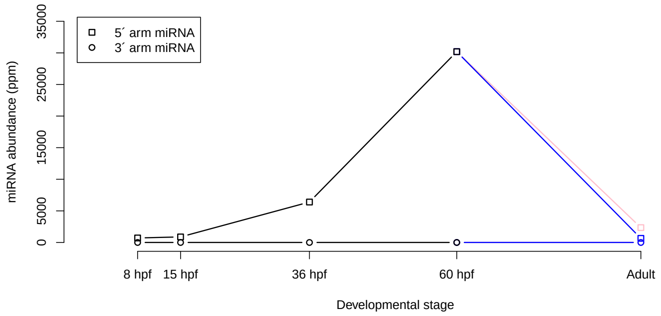

(continued on next page)

<table>
<tr><th>Pre-miRNA</th><th>miRNA sequences</th><th>Abundance profile in development</th></tr>
<tr><td>bbe-mir-2067 ortholog at Sc0000082 bp 843448-843367 (- strand)</td><td>5´arm:
(low abundance)

3´arm:
AAGCAGCAGUAUGCAAUGGUGA</td><td>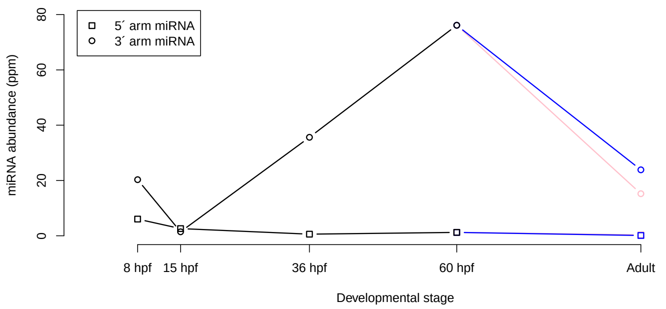</td></tr>
<tr><td>bfl-mir-184 ortholog at Sc0000017 bp 1306111-1306211 (+ strand)</td><td>5´arm:
CUUAUCACUUCUUCCGCCGAGC

3´arm:
UGGACGGAGAACUGAUAAGGGCC</td><td>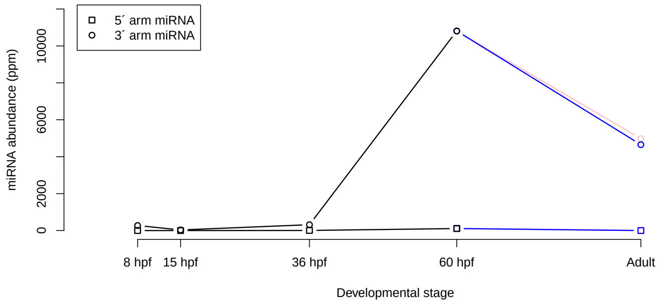</td></tr>
<tr><td>bbe-mir-34a ortholog at Sc0000034 bp 210967-210880 (- strand)</td><td>5´arm:
CGUUCCCUGUGUGCUGCUG

3´arm:
AGCCACUGUACACACUCCCGUA</td><td>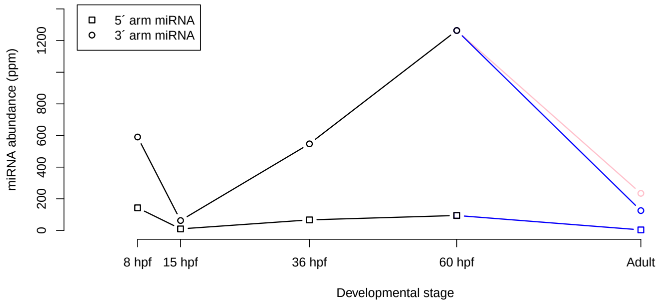</td></tr>
<tr><td>bfl-mir-4904 ortholog at Sc0000028 bp 432156-432080 (- strand)</td><td>5´arm:
UCCCGGAGUCGUUCUAUACGCCG

3´arm:
(low abundance)</td><td>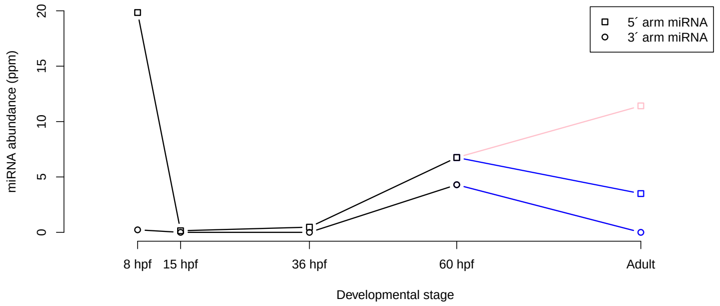</td></tr>
</table>

(continued on next page)

Pre-miRNA	miRNA sequences	Abundance profile in development
bfl-mir-4870 ortholog at Sc0000009 bp 4682265-4682351 (+ strand)	5´arm: `GAUGUUUGUACUGUCUGUCUGUU` 3´arm: `(low abundance)`	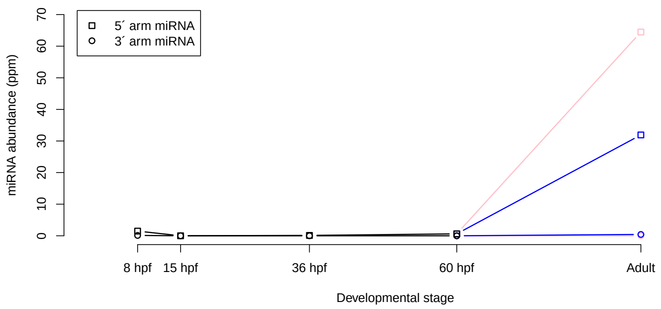
bfl-mir-4866 ortholog at Sc0000043 bp 1850204-1850295 (+ strand)	5´arm: `UCACACUUGUACUUCUUAGCAU` 3´arm: `(low abundance)`	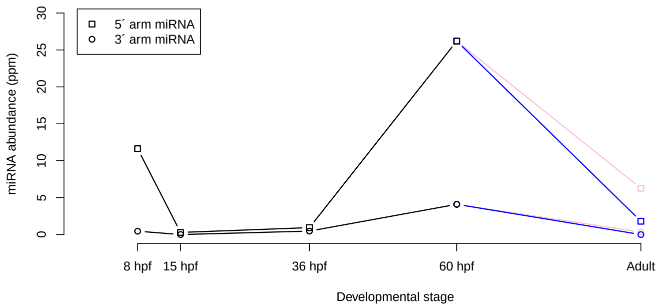
bbe-mir-9 ortholog at Sc0000019 bp 2253983-2254064 (+ strand)	5´arm: `UCUUUGGUUAUCUAGCUGUAUGA` 3´arm: `(low abundance)`	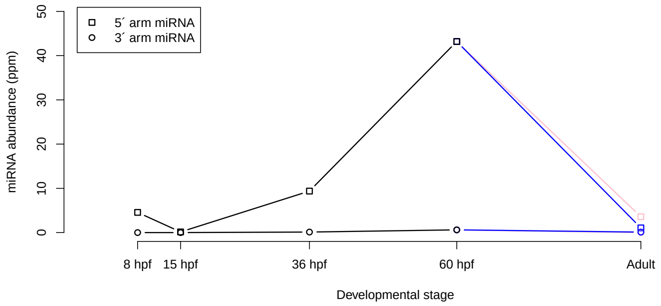
bfl-mir-217 ortholog at Sc0000230 bp 138098-138165 (+ strand)	5´arm: `UACUGCAUCAGGAACUGAUUGG` 3´arm: `AAUCUGUUCCUCAUGCAUGGCU`	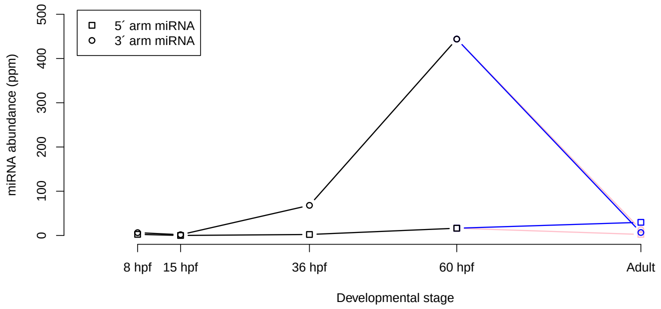
